# Correction: Upadyshev et al. Mass Spectrometric Identification of Metabolites after Magnetic-Pulse Treatment of Infected *Pyrus communis* L. Microplants. *Int. J. Mol. Sci.* 2023, *24*, 16776

**DOI:** 10.3390/ijms26083832

**Published:** 2025-04-18

**Authors:** Mikhail Upadyshev, Bojidarka Ivanova, Svetlana Motyleva

**Affiliations:** 1 Laboratory of Virology, Russian State Agrarian University—Moscow Timiryazev Agricultural Academy, Timiryazevskaya Str. 49, 127422 Moscow, Russia; upad8@mail.ru; 2 Independent Researcher, 44139 Dortmund, Germany; bojidarka.ivanova@yahoo.com; 3 Federal State Budgetary Scientific Institution “Federal Scientific Center of Legumes and Groat Crops”, Molodezhnaya Str. 10, 302502 Oryol, Russia

Following a request from the university (Dortmund University), a previous affiliation of the Guest Editor (Bojidarka Ivanova) in the published publication [1] has been updated. The authors state that the scientific conclusions are unaffected. This correction was approved by the Academic Editor. The original publication has also been updated.
