# Supplementary material for: Mass Spectrometric Identification of Metabolites after Magnetic-Pulse Treatment of Infected Pyrus communis L. Microplants
Source: Int J Mol Sci. 2023 Nov 26;24(23):16776. doi: 10.3390/ijms242316776 (PMC10705910; doi:10.3390/ijms242316776)
Supplement: Supplementary file 1 [file ijms-2671639-supplementary-update.pdf › ijms-2671639-supplementary-update.pdf]

SUPPORTING INFORMATION

TITLE: Mass spectrometric identification of metabolites after magnetic pulse treatment of infected *Pyrus communis L.* microplants

(Chromatographic and mass spectrometric data)

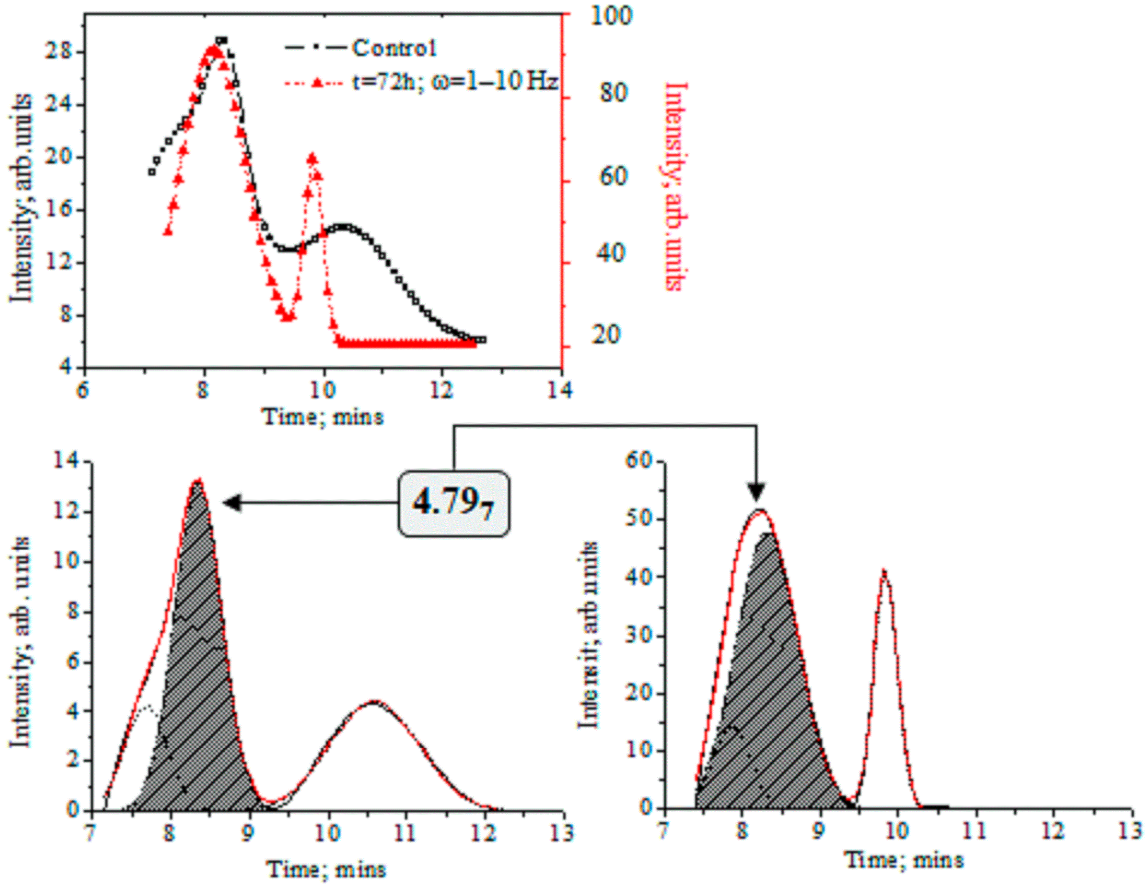

χ^2/DoF	0.02741	
r^2	0.99819	
xc1	7.67856	±0.02463
w1	0.53861	±0.03259
A1	2.87919	±0.28005
xc2	**8.34719**	±0.00902
w2	0.59955	±0.01208
**A2**	**9.92793**	**±0.28079**
xc3	10.5915	±0.00919
w3	1.12944	±0.01849
A3	6.25712	±0.08836

χ^2/DoF	0.76632	
r^2	0.99793	
xc1	7.86023	±0.02989
w1	0.47852	±0.09156
A1	8.71864	±6.34179
xc2	**8.31934**	±0.05715
w2	0.7906	±0.01208
**A2**	**47.626**	±0.05112
xc3	9.83585	±0.00252
w3	0.29501	±0.00505
A3	15.4965	±0.23002

Figure S1.Curve-fitted chromatographic data on Figure 1, using Gauss-fitting function; area ratio of peak at RT=8.33±0.0197 mins; chemometrics.

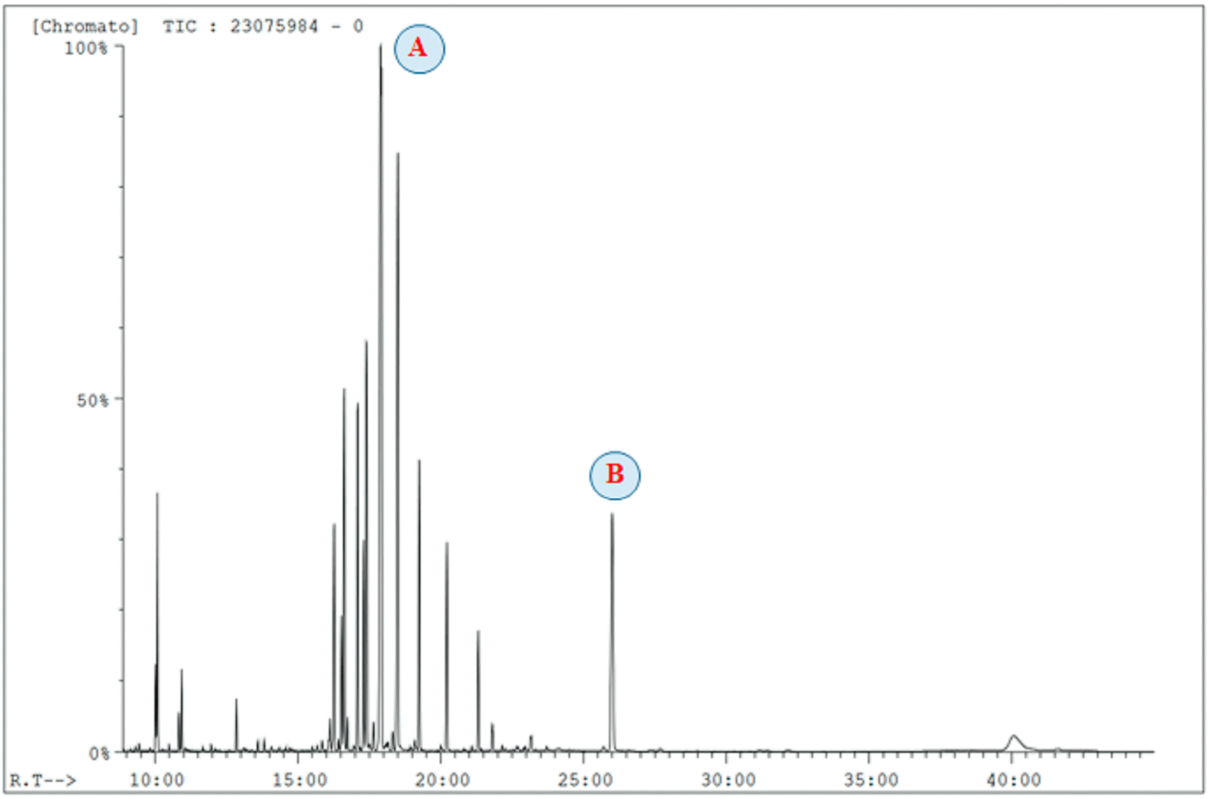

Figure S2. Chromatographic data on silylated carbohydrates; carbohydrates (A) (RT=18.3 mins) and (B) (RT=25.59 mins) are highlighted.

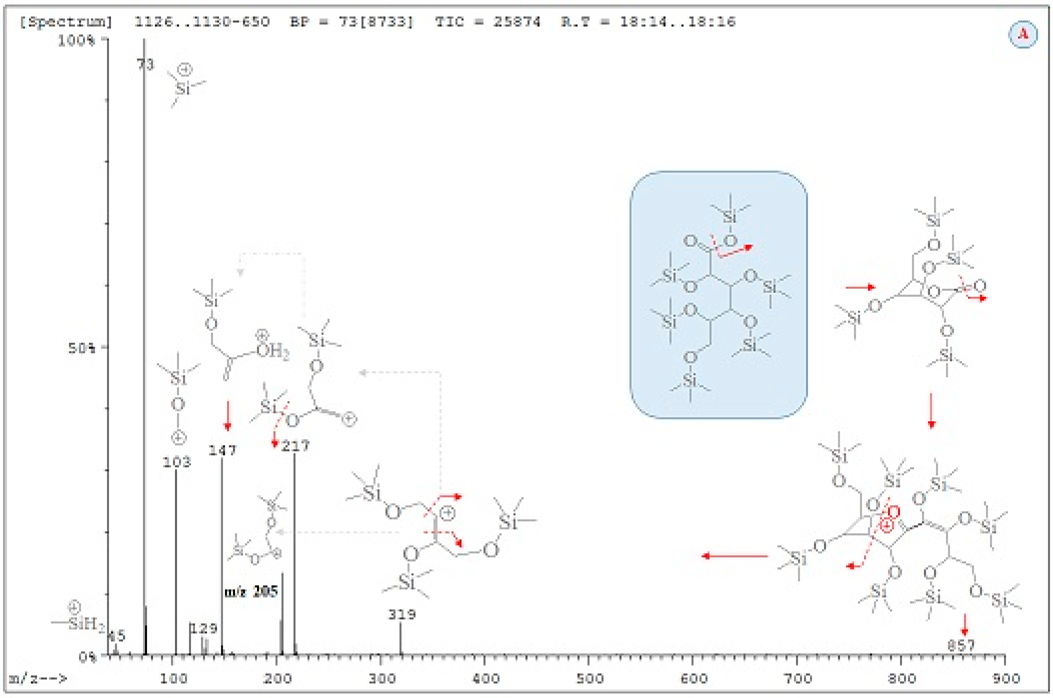

Figure S3. Mass spectrum of carbohydrate (A) at RT=18.3 mins assigned to silylated D-gluconic acid, using NIST database searching algorithms (see next **Figure S4**) at a statistical significantce of $|r|=0.9488_5$ and $|r|=0.9393_7$ using EI data on standard D-gluconic acid, 2,3,4,5,6-pentakis-O-(trimethylsilyl)-, trimethylsilyl ester [https://webbook.nist.gov/cgi/cbook.cgi?ID=C34290523&Units=SI&Mask=3FFF]; chemical diagrams of the analyte and ist fragmentation ions.

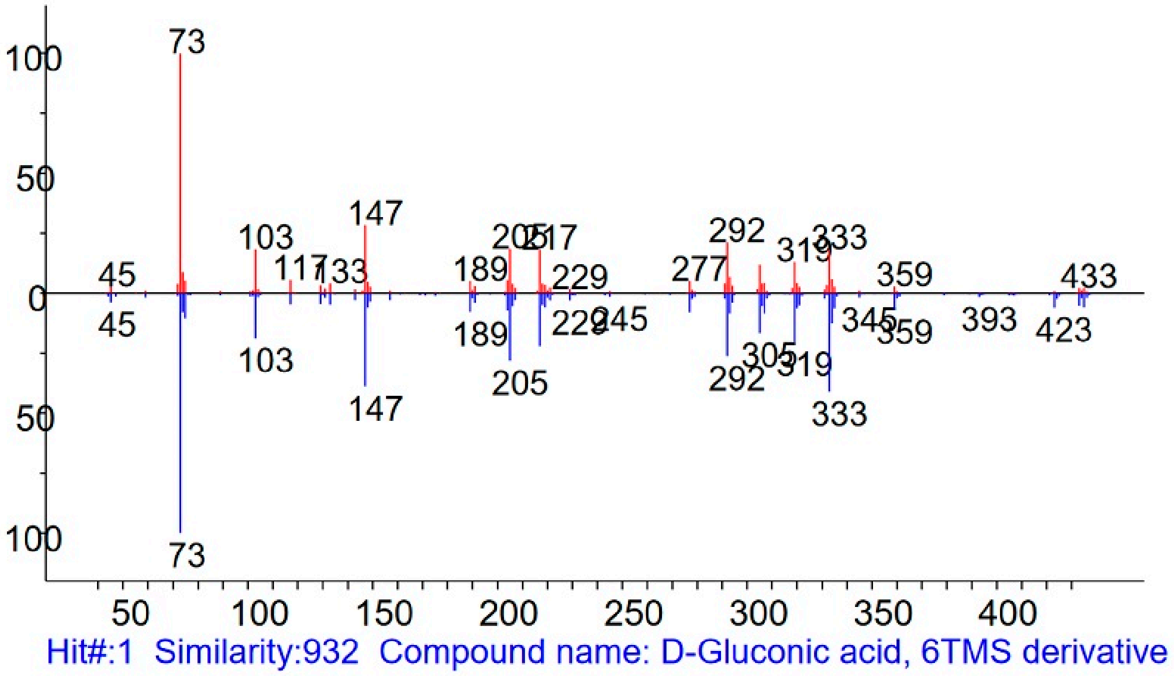

Figure S4. NIST database searching algorithms applied to electron impact mass spectrum of silylated D-gluconic acid.

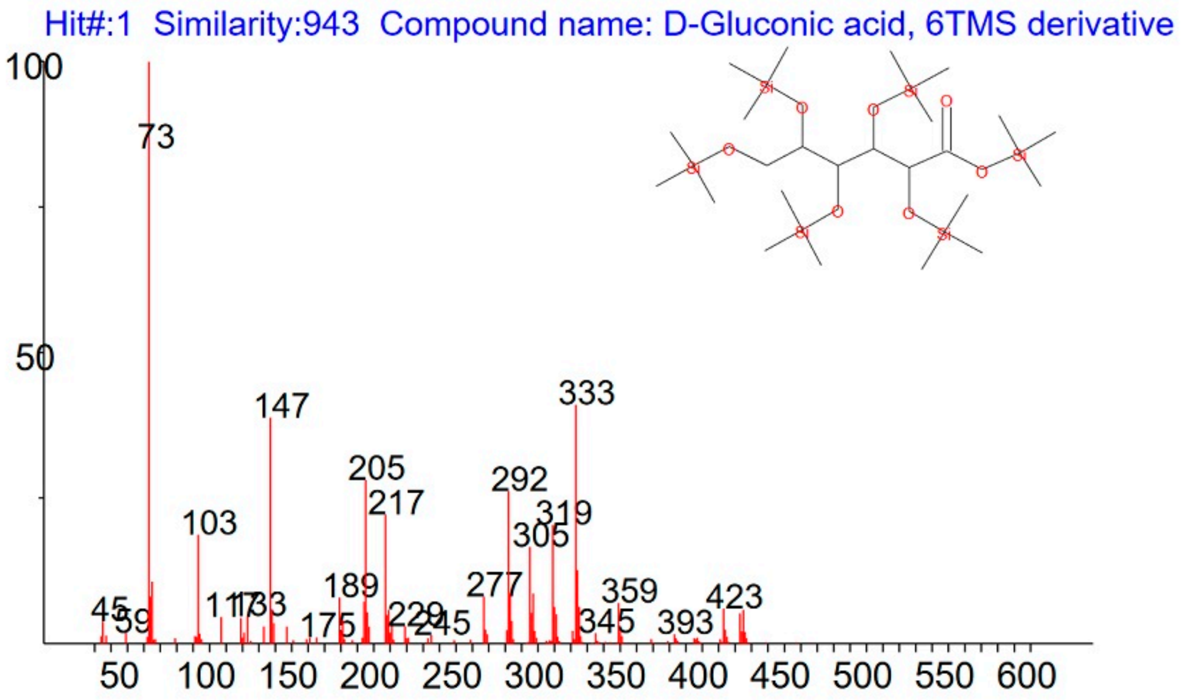

Figure S5. NIST standard mass spectrum of silylated D-gluconic acid; chemical diagrams of the carbohydrate monomer.

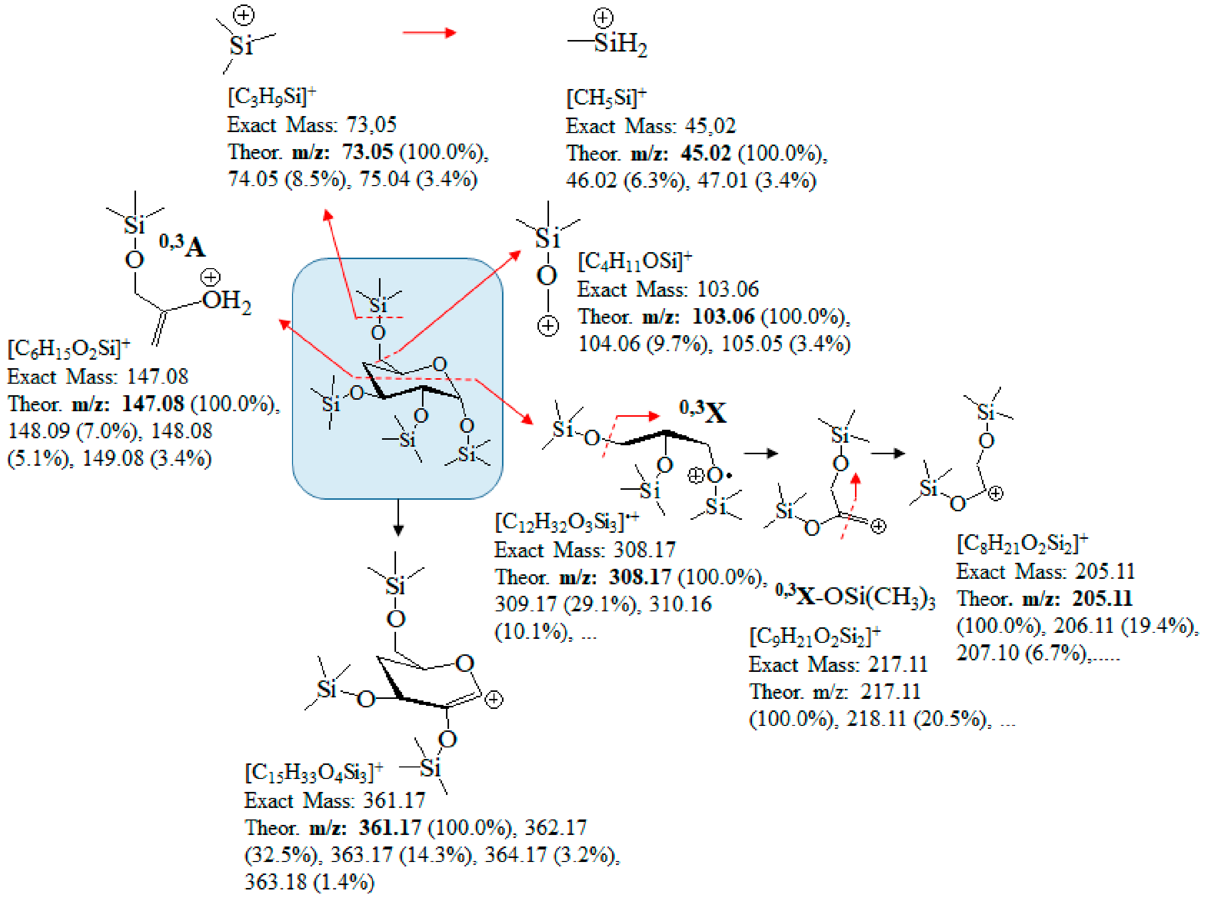

Figure S6. Mass spectrometric fragmentation scheme of silylated 4-deosoxy-glucose; chemical diagrams of species; theoretical exact masses of ions; theoretical m/z data on fragmentation ions and intensity ratio (%) of peaks of their isotope shapes, respectively.

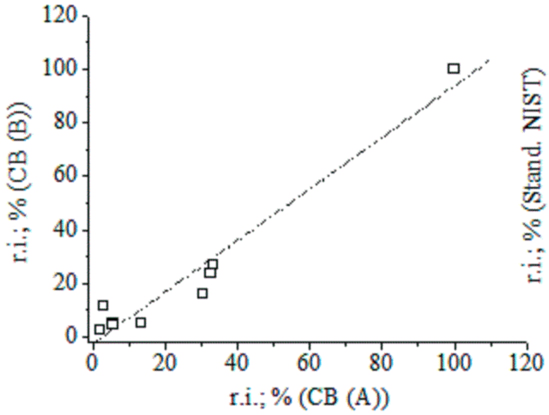

	Value	Error	
A	4,14714	5,06568	
B	0,91242	0,12394	
|r|	sd(yEr±)	N	*p*
0.9488_5	10.46088	8	$3.22.10^{-4}$

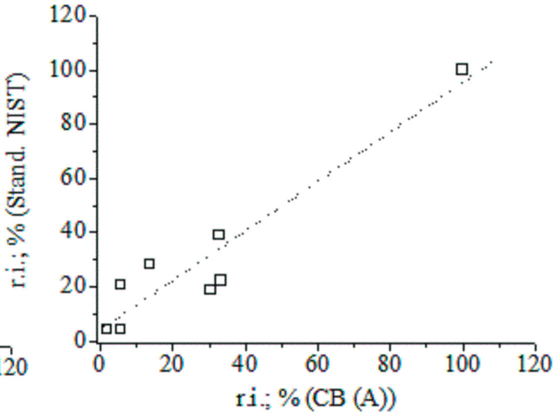

	Value	Error	
A	−2.5531	3.17874	
B	0.96434	0.08246	
|r|	sd(yEr±)	N	*p*
0.9753_5	7.22366	9	<0.0001

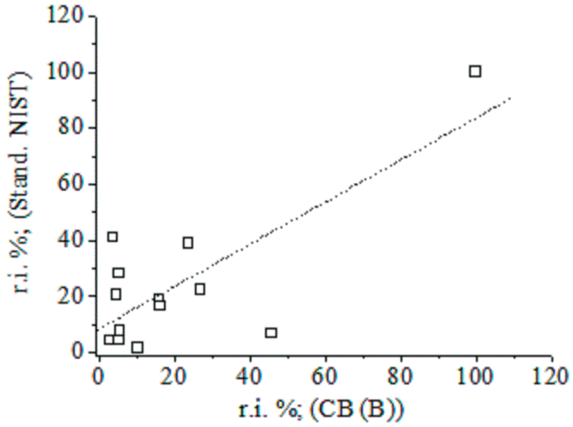

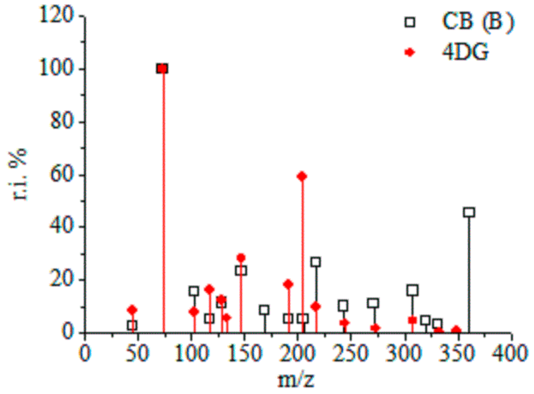

	Value	Error	
A	8.5518	6.15437	
B	0.7514	0.18688	
|r|	sd(yEr±)	N	*p*
0.7714_3	17.43812	13	0.00201

Figure S7. Linear relationships between datasets of relative intensities of fragmentation peaks of carbohydrates (A) and (B) as well as between data on latter analytes and NIST standard ones of silylated D-gluconic acid; chemometrics.

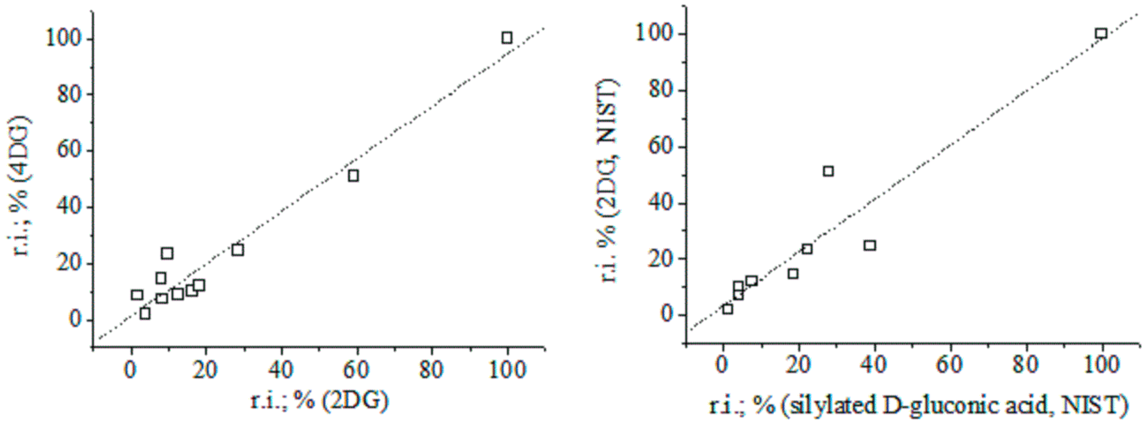

	Value	Error	
A	1.22399	2.68614	
B	0,93436	0,07198	
|r|	sd(yEr±)	N	*p*
0.9743_2	6.77658	11	<0.0001

	Value	Error	
A	3.42579	4.55112	
B	0.94915	0.11861	
|r|	sd(yEr±)	N	*p*
0.9494_5	10.33821	9	<0.0001

Figure S8. Linear relationships between datasets of relative intensities of fragmentation peaks of silylated D-gluconic acid (NIST standard), silylated α-2-deoxy-D-glucose (NIST standard) [https://webbook.nist.gov/cgi/inchi?ID=U79295&Mask=200#], and silylated 4-deoxy-D-glucose; chemometrics.

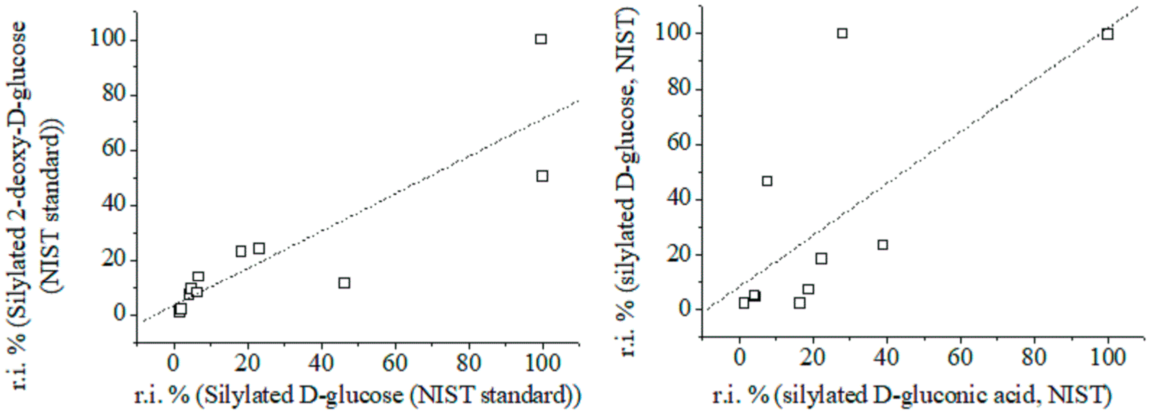

	Value	Error	
A	3.27754	5.6889	
B	0.67895	0.12413	
|r|	sd(yEr±)	N	*p*
0.87678	14.76989	11	$3.955.10^{-4}$

	Value	Error	
A	7.92653	12.2722	
B	0.94168	0.33372	
|r|	sd(yEr±)	N	*p*
0.70628	29.2133	10	0.02243

Figure S9. Linear relationships between datasets of relative intensities of fragmentation peaks of silylated α-2-deoxy-D-glucose (NIST standard) [https://webbook.nist.gov/cgi/inchi?ID=U79295&Mask=200#], silylated D-glucose (NIST standard) [https://webbook.nist.gov/cgi/inchi?ID=C6736976&Mask=200], and silylated D-gluconic acid (NIST standard); chemometrics.

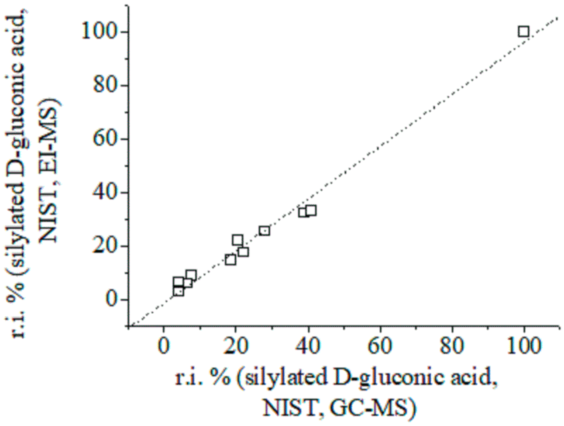

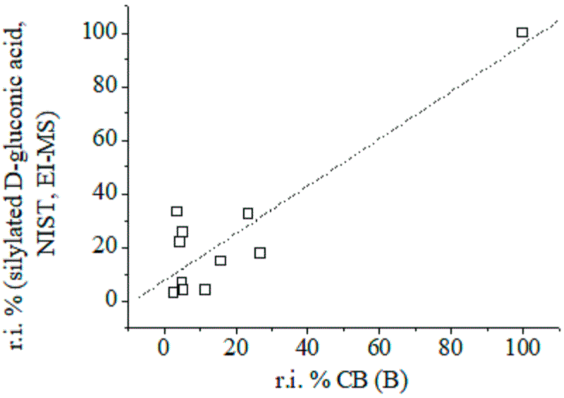

	Value	Error	
A	–1.4679	1.49853	
B	0.97595	0.04007	
|r|	sd(yEr±)	N	*p*
0.9925	3.49751	11	<0.0001

	Value	Error	
A	7.41839	4.56469	
B	0.88274	0.13945	
|r|	sd(yEr±)	N	*p*
0.9036_6	12.46166	11	$1.36.10^{-4}$

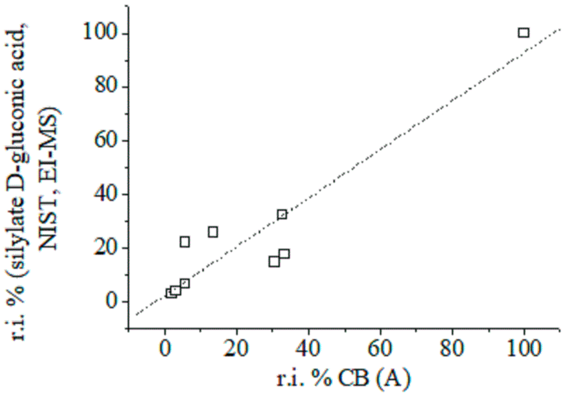

	Value	Error	
A	2.21418	4.81568	
B	0.90547	0.12493	
|r|	sd(yEr±)	N	*p*
0.9393_7	10.94359	9	$1.703.10^{-4}$

Figure S10. Linear relationships between datasets of relative intensities of GC-MS and electron impact MS fragmentation peaks of NIST standard samples of silylated D-gluconic acid [https://webbook.nist.gov/cgi/cbook.cgi?ID=C34290523&Units=SI&Mask=3FFF] (see **Figure S12**), as well as data on CB (A) and CB (B); chemometrics.

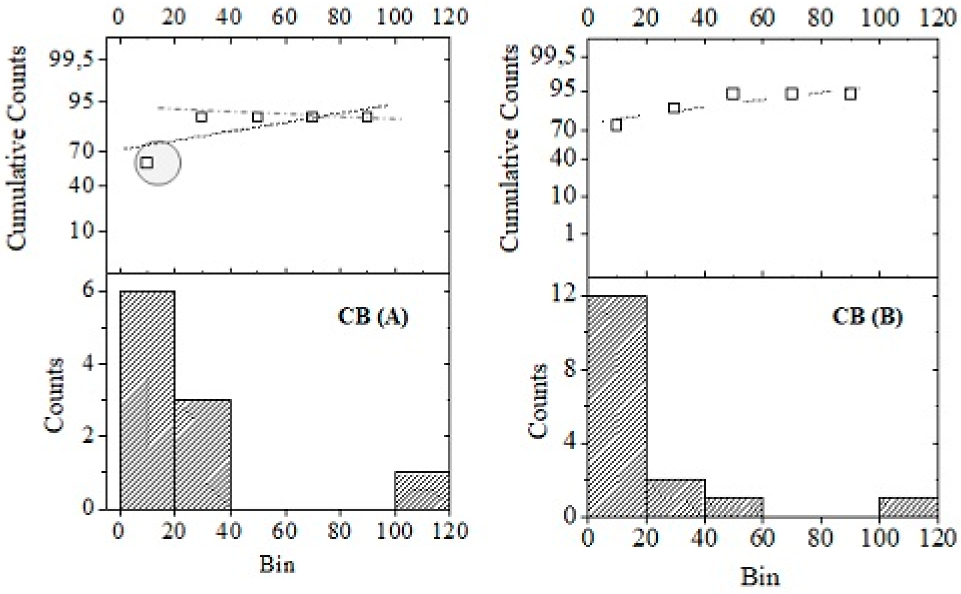

Figure S11. Histograms and probability plots of mass spectrometric data on CBs (A) and (B).

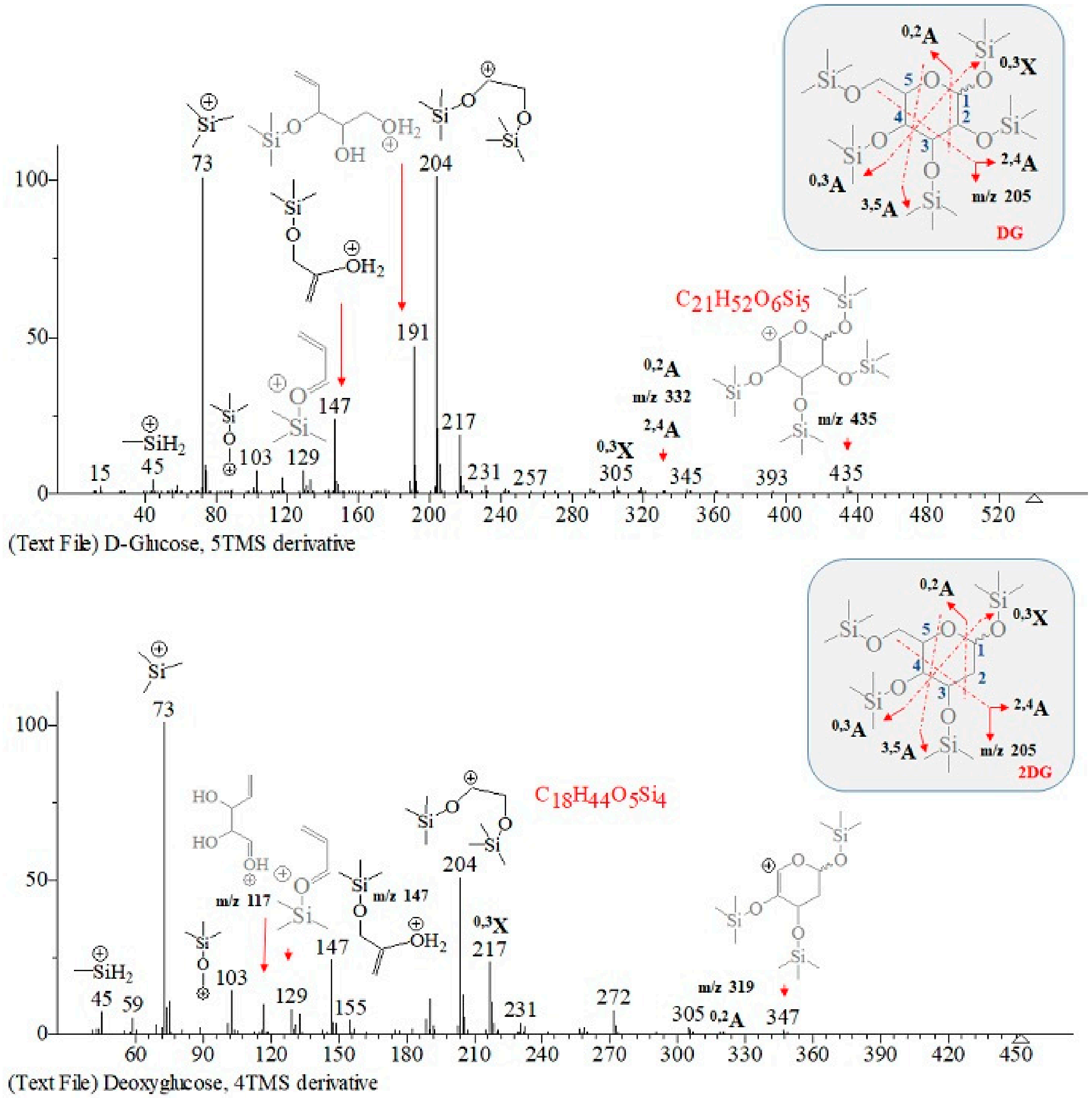

Figure S12. EI-spectrum of silylated D-glucose (NIST standard) [https://webbook.nist.gov/cgi/inchi?ID=C6736976&Mask=200] and silylated α-2-deoxy-D-glucose (NIST standard) [https://webbook.nist.gov/cgi/inchi?ID=U79295&Mask=200#]; chemical diagrams of fragmentation ions.

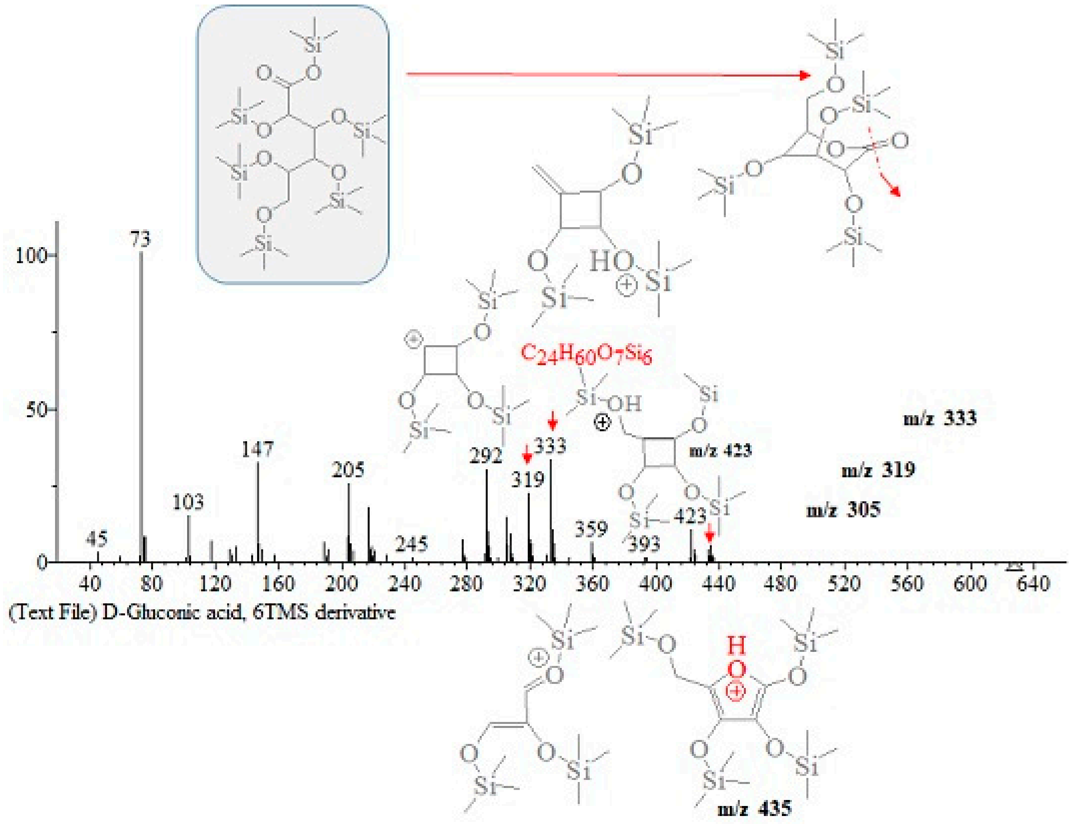

Figure S13. NIST electron impact mass spectrum of standard silylated D-gluconic acid [https://webbook.nist.gov/cgi/cbook.cgi?ID=C34290523&Units=SI&Mask=3FFF]; chemical diagram of the analyte and its fragmentation ions.

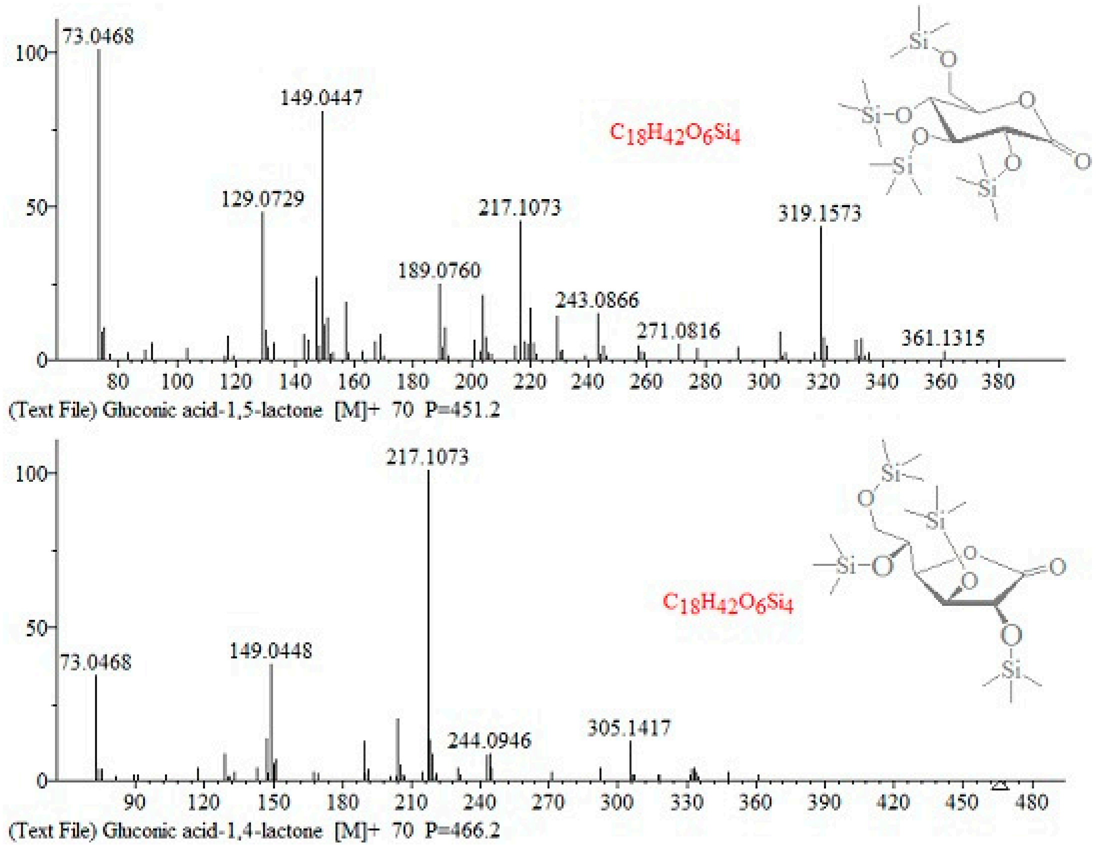

Figure S14. GC-EI-Orbitrap MS/MS spectra of silylated D-glucono-1,5- and 1-4-lacton according to database [72] (main text;) chemical diagrams in analytes.

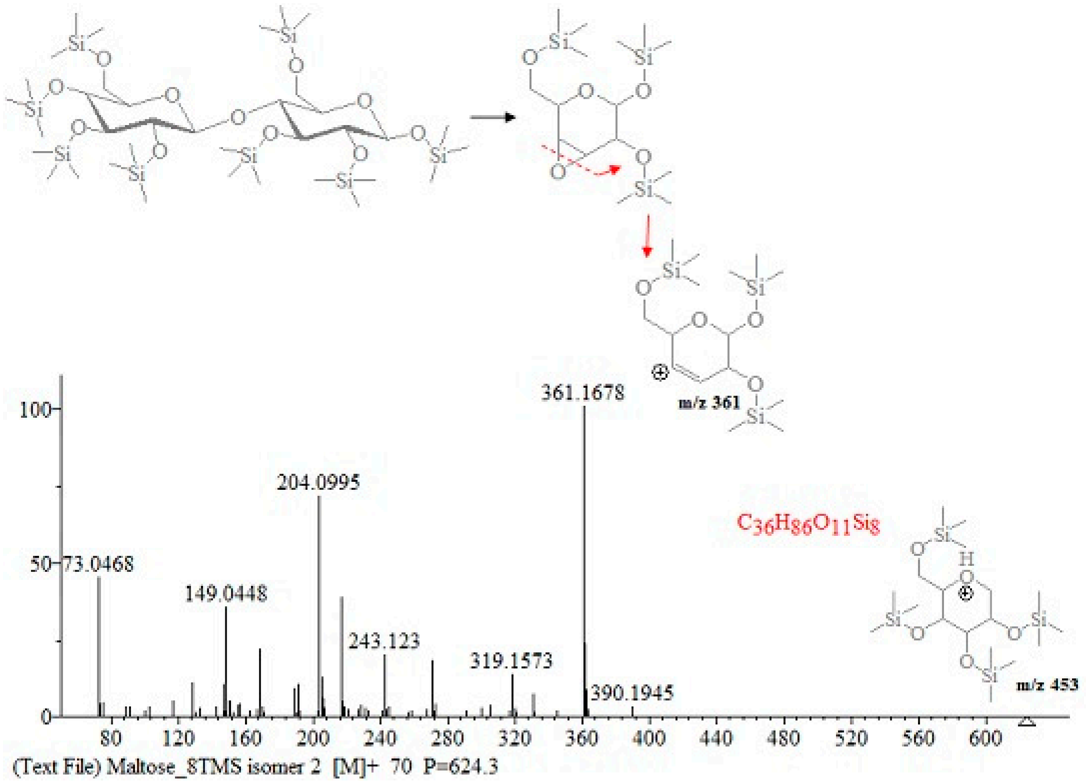

Figure S15. GC-EI-Orbitrap MS/MS spectra of silylated maltose according to database [72] (main text;) chemical diagrams in analyte and ist fragmentation ions at m/z 361 and 453.

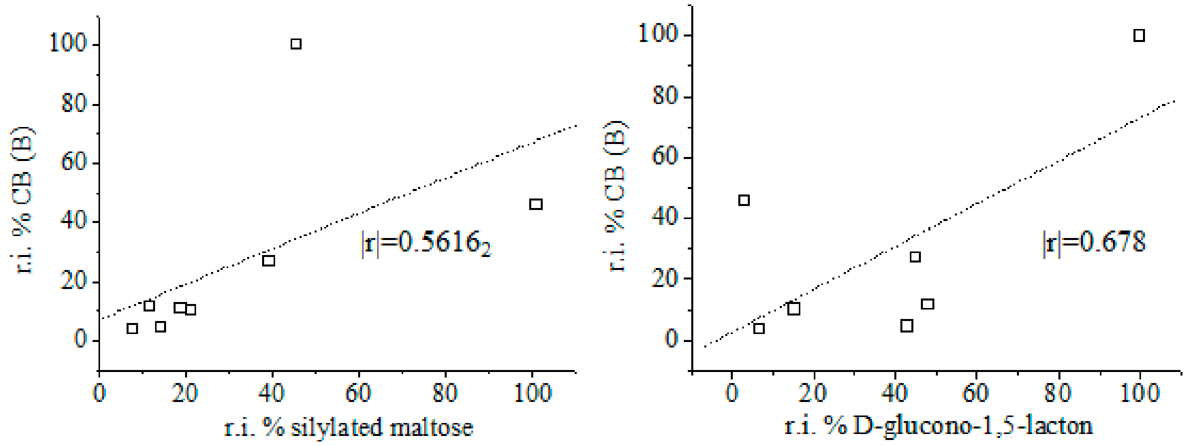

Figure S16. Correlative analysis between relative intensity data [%] of fragmentation ions of silylated maltose, respectively, D-glucono-1,5-lacton and CB (B) using our GC-MS results and those GC-EI-Orbitrap MS/MS charecteritic peaks of database [72]; chemometrics, (see **Figures S14** and **S15**, as well.)

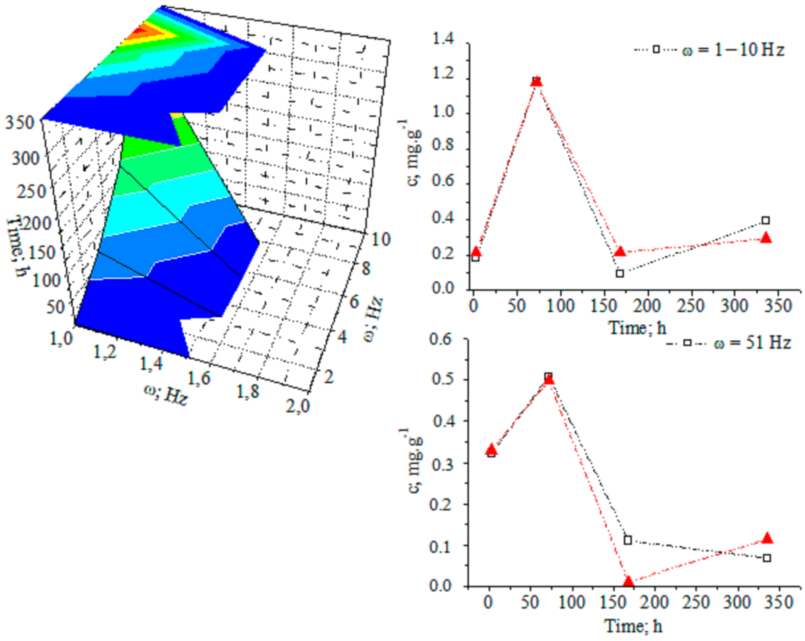

χ^2/DoF	r^2	
0.0245	0.96653	
	Value	Error
x_c	337.942	0.41311
w	18.6999	0.02368
A	2.86348	0.60006

χ^2/DoF	r^2	
0.01221	0.90182	
	Value	Error
x_c	355.087	0.77741
w	16.94963	0.0941
A	0.76851	0.33508

Figure S17. 3D and 2D plots of temporal distribution for measurand concentration of chlorogenic acid with respect to time [h] and magnetic pulse frequency (ω [Hz];) experimental curve and fitted relationship of SineSqr function; chemometrics.

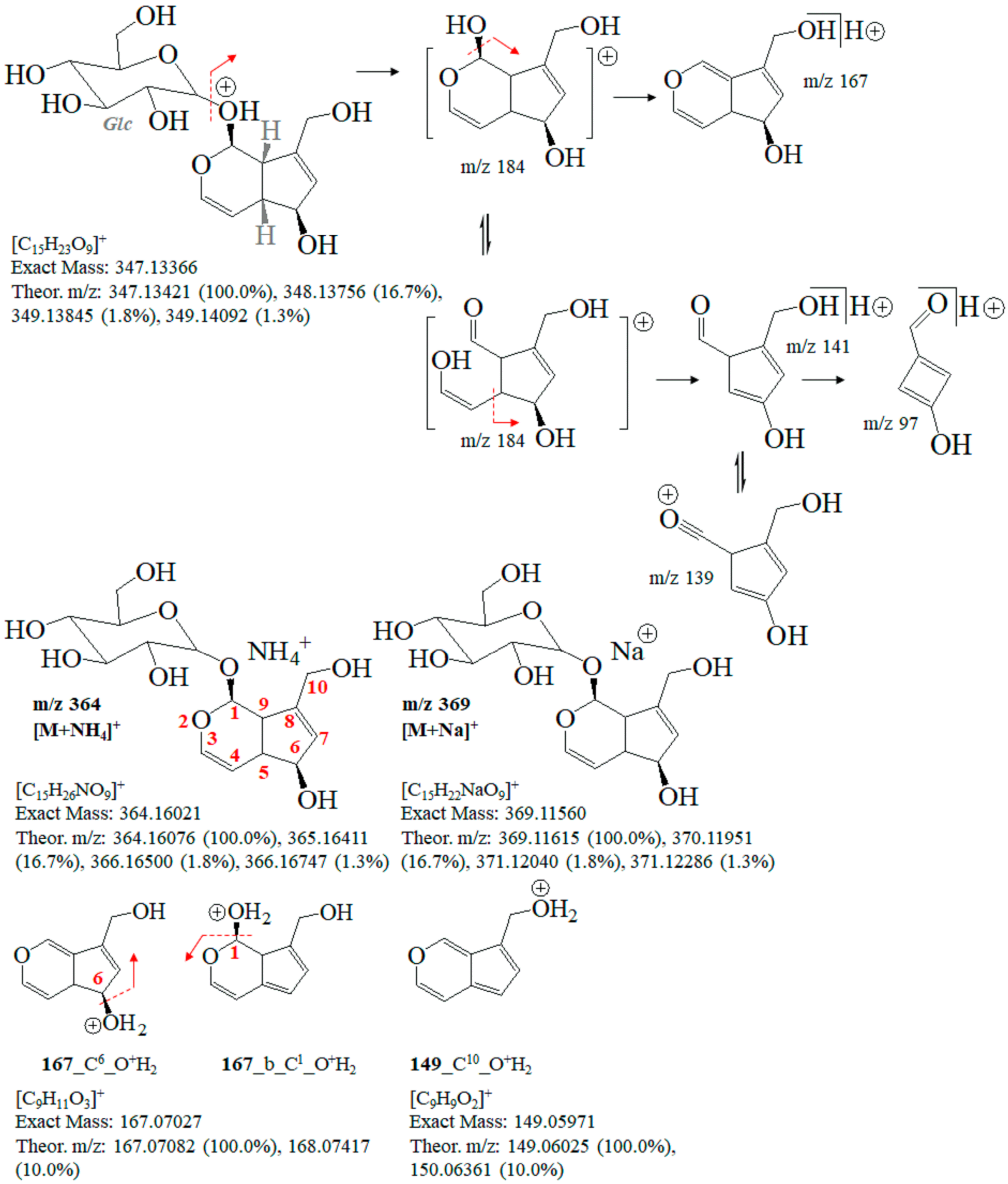

Figure S18. Chemical diagrams of adducts ([M+NH4]+ and [M+Na]+) and fragmentation product ions in aucubin; theoretical exact mass of protonated [M+H]+ cation (the proton position is obtained *via* MM2 computations,) as well as its theoretical m/z data in addition to intensity ratio of the isotope shape of the peak.

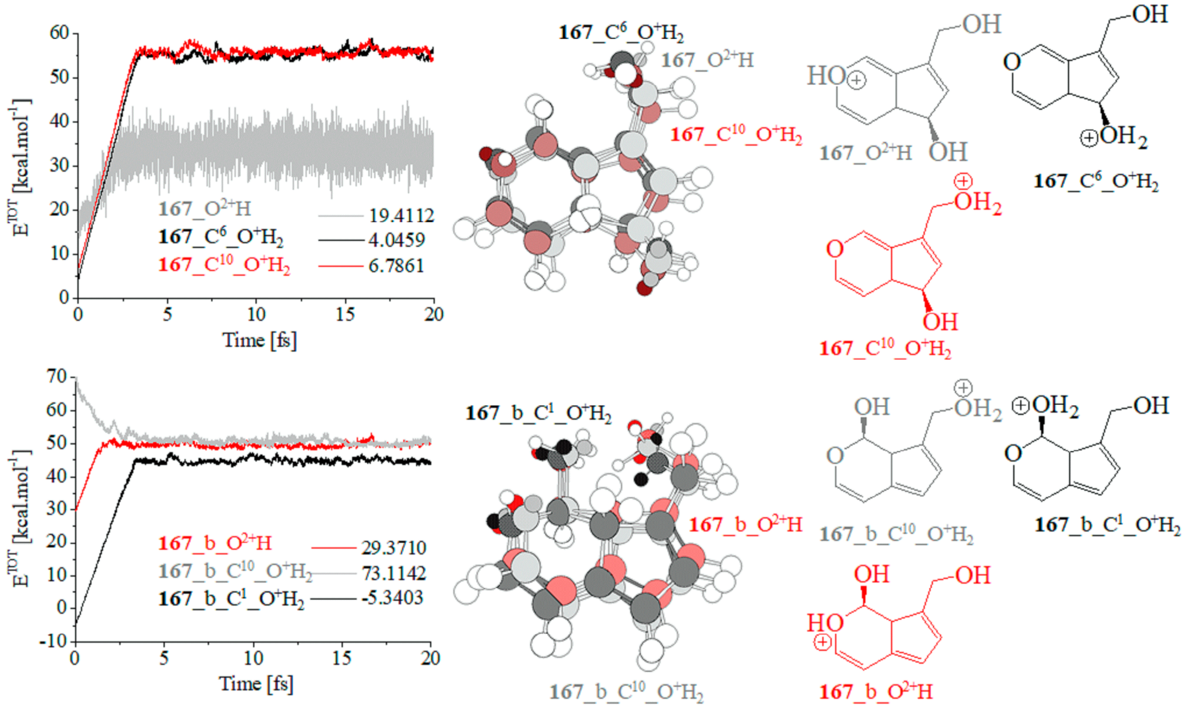

Figure S19. MM2 molecular dynamics of species in aucubin at m/z 167, depending on proton accepting position: Total energy (E^{TOT}, kcal/mol) *versus* time [fs]; 3D MD molecular geometry of the different protonated forms of the cation; 2D chemical diagrams of species.

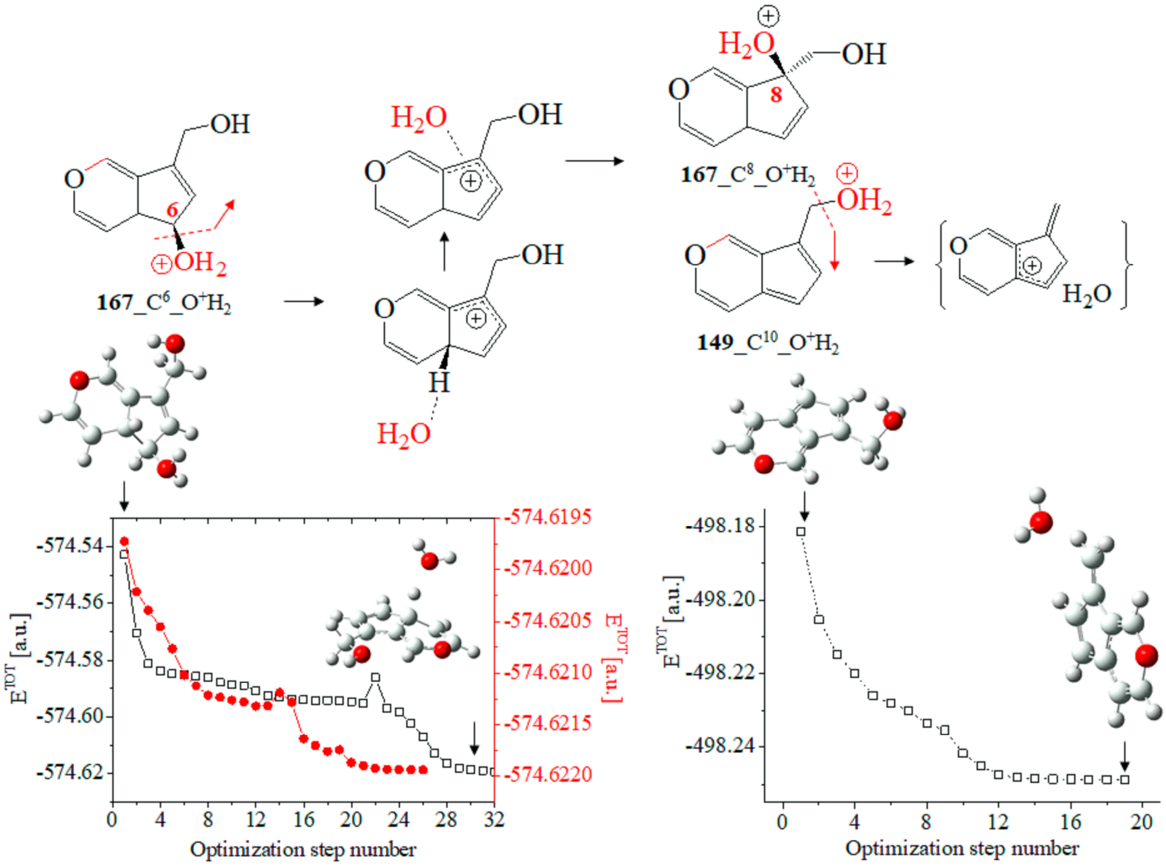

Figure S20. The M062X/SDD optimization in MS 167_C^6_O$^+$H$_2$ and 149_C^{10}_O$^+$H$_2$ cations in aucubin at m/z 167 ad 149: Total energy, E^{TOT} [a.u.] *versus* optimization step number; chemical diagrams of initial ion, its intermediate species and the final state; 3D molecular geometry of species; BO-MD data on fragmentation ions 121$_{_b}$ and 131$_{_b}$ at the same theoretical level: Total energy E^{TOT} [a.u.] *versus* time in trajectory [fs].

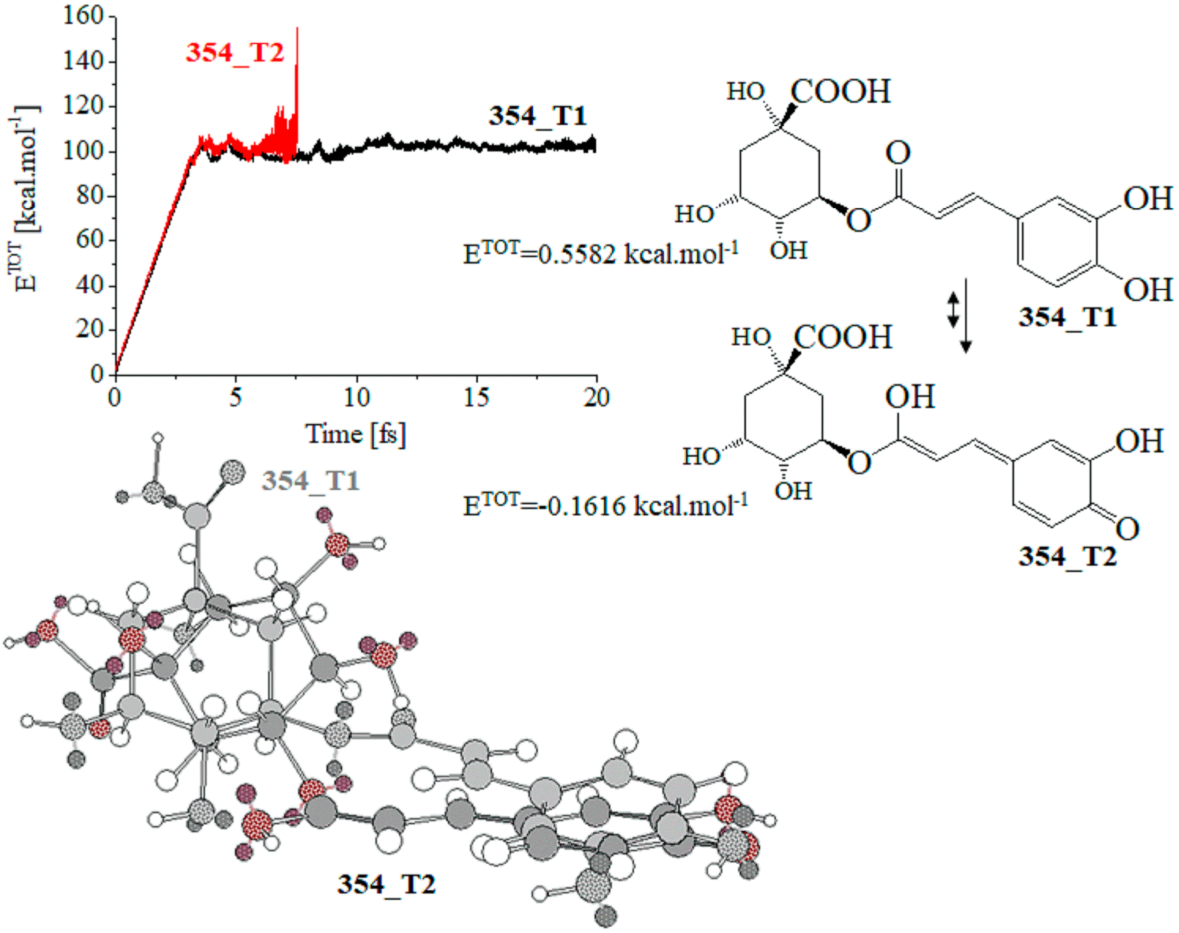

Figure S21. MM2 molecular dynamics of neutral chlorogenic acid depending on its tautomeric forms (T1 and T2): Total energy (E^{TOT}, [kcal/mol]) *versus* time [fs]; 3D MD structures of species.

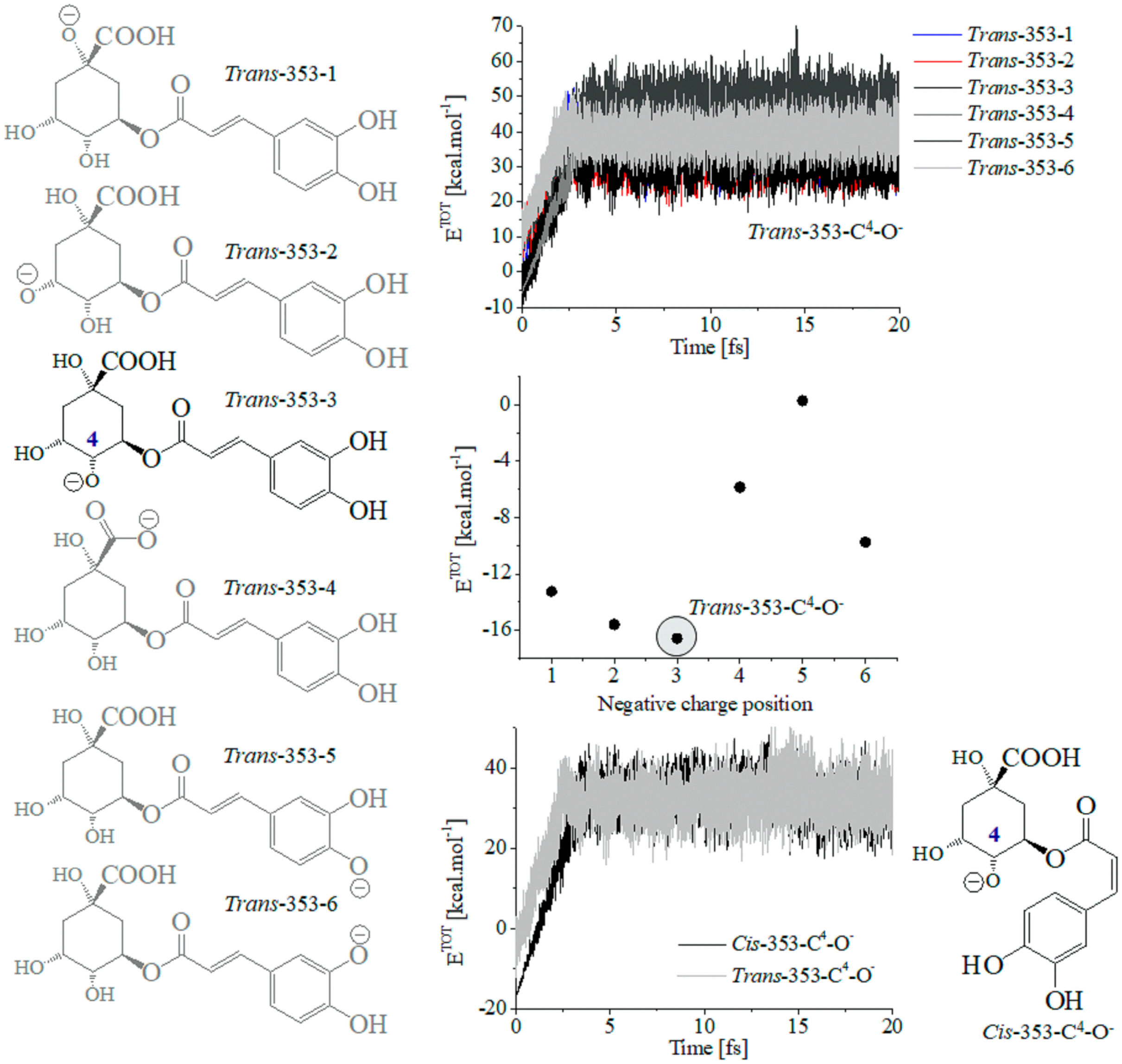

Figure S22. MM2 molecular dynamics of $[M-H]^-$ ion of chlorogenic acid at m/z 353 depending on position of negative charge at the ionic species: Total energy (E^{TOT}, [kcal/mol]) *versus* time [fs]; chemical diagrams of species.

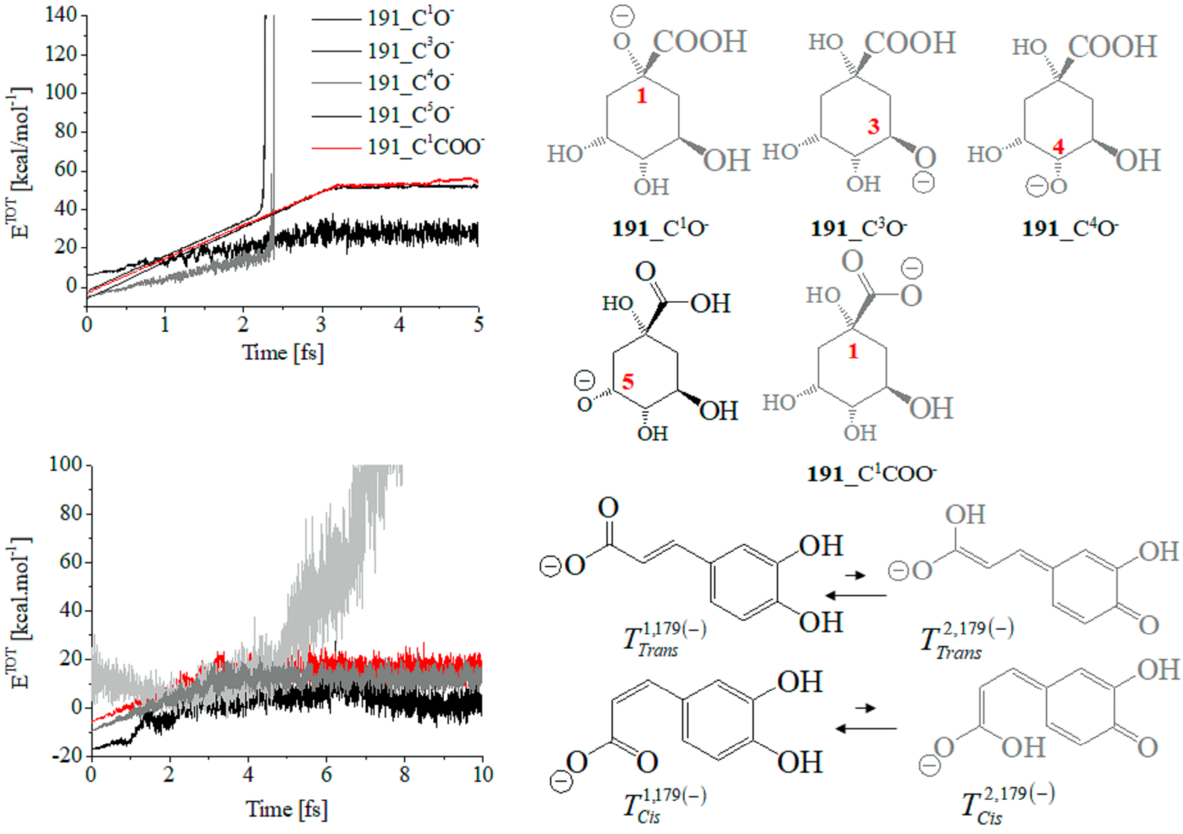

Figure S23. MM2 molecular dynamics of chlorogenic acid fragmentation anions at m/z 191 and 179 depending on position of negative charge at ionic species and their tautomers T1 and T2: Total energy (E^{TOT}, [kcal/mol]) *versus* time [fs]; chemical diagrams of species.

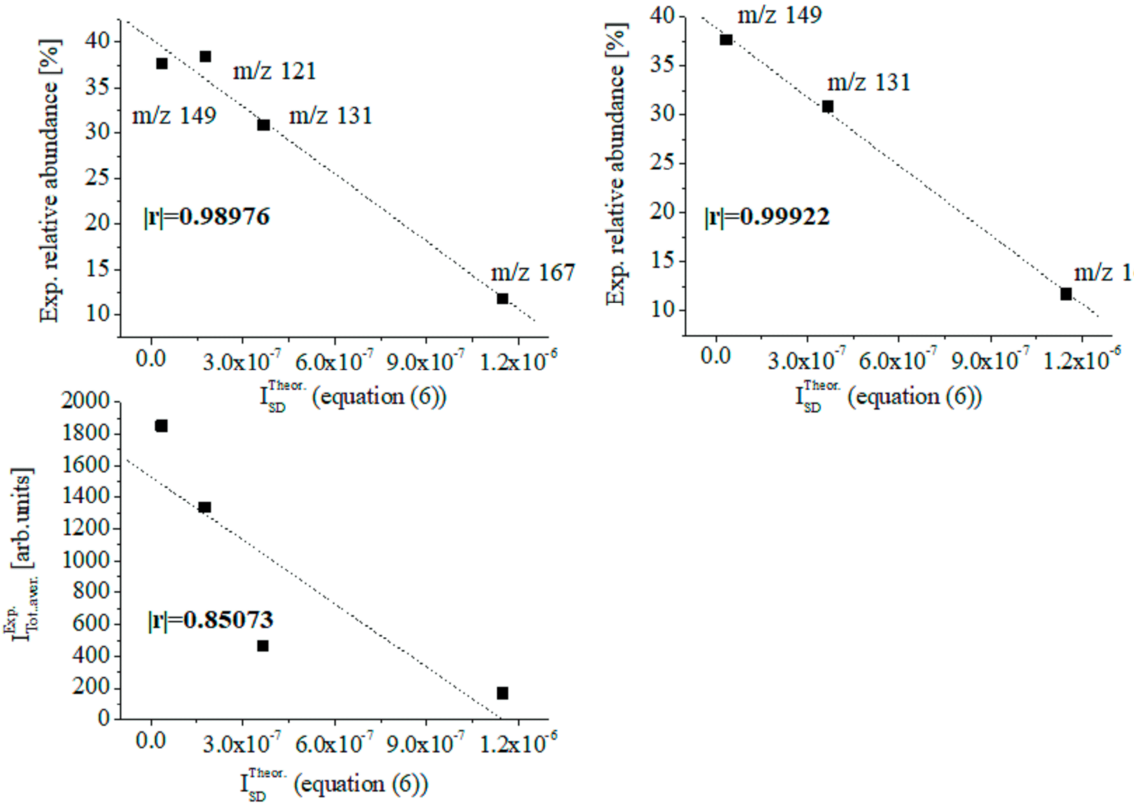

Figure S24. Functional relations between theoretical stochastic dynamic mass spectrometric intensity data on (I^{theor}_{SD}) of equation (6) in MS ions in aucubin at m/z 167, 149, 131, and 121 (consider data on **Tables S5 and S6**) and experimental total average intensities [%] or [arb.units] with respect to experimental ionization conditions; chemometrics; (see data on reference [S1], as well.)

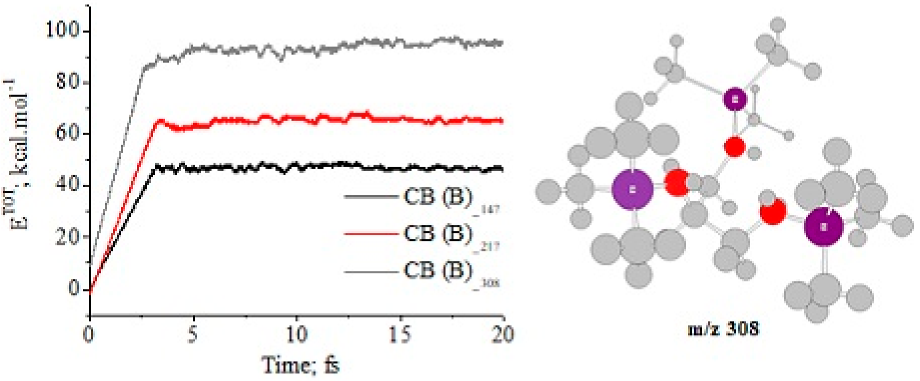

Figure S25. MM2 molecular dynamics of fragmentation ions of carbohydrate (B) at m/z 147, 217, and 308 depicted in **Figure 5**: Total energy (E^{TOT}, [kcal/mol]) *versus* time [fs]; 3D MM2 molecular structure of the latter cation.

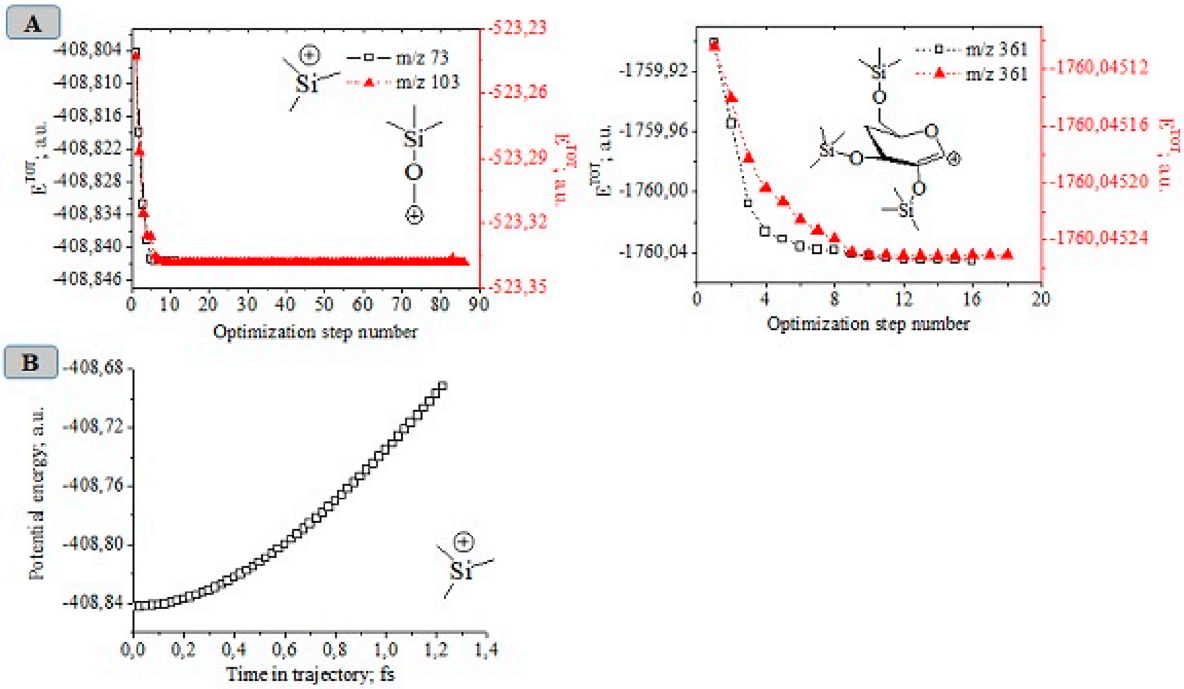

Figure S26. Theoretical M062X/SDD data on fragmentation ions of silylated carbohydrates: Total energy, E^{TOT} [a.u.] with respect to optimization step numbers (A); M062X/SDD BOMD molecular dynamics: Potential energy [a.u.] *versus* time in trajectory [fs] (B); chemical diagrams of species.

Table S1

ANOVA data on datasets of intensity values of fragmentation ions, m/z parameters of carbohydrate (A), and results from spectrum of silylated D-gluconic acid (**Figures S3** and **S5**)

Data on m/z values of carbohydrates (A) and silylated D-gluconic acid					
Dataset	N	Mean	sd(yEr±)	se(yEr±)	
Data1_CBA	10	221.2	237.09623	74.97641	
Data1_4DG	14	199.28571	101.66266	27.17049	
H_0: The means of all selected datasets are equal					
H_1: The means of one or more selected datasets are different					
Source	DoF	Sum of square	Mean square	F value	P value
Model	1	2801.37619	2801.37619	0.09625	0.75929
Error	22	640290.457	29104.1117		
At the 0.05 level, the population means are not significantly different					
Means Comparison using Bonferroni Test					
Dataset	Mean	Difference between means	Simultaneous Confidence Intervals		Significant at 0,05 Level
Data1_CBA	221.2		Lower Limit	Upper Limit	
Data1_4DG	199.2857	21.91429	-124.57334	168.40191	No
Intensity data on carbohydrates (A) and silylated D-gluconic acid					
Dataset	N	Mean	sd(yEr±)	se(yEr±)	
Data1_CBA	10	22,69459	30,22574	9,55822	
Data1_4DG	14	22,49264	25,71879	6,87364	
H_0: The means of all selected datasets are equal					
H_1: The means of one or more selected datasets are different					
Source	DoF	Sum of square	Mean square	F value	P value
Model	1	0.237915612	0.237915612	$3.1116.10^{-4}$	0.98609
Error	22	16821.2900	764.604092		
At the 0.05 level, the population means are not significantly different					
Means Comparison using Bonferroni Test					
Dataset	Mean	Difference between means	Simultaneous Confidence Intervals		Significant at 0,05 Level
Data1_CBA	22.69459		Lower Limit	Upper Limit	
Data1_4DG	22.49264	0.20195	-23.5414	23.94531	No

Table S2

ANOVA data on analysis of datasets of variables intensity of fragmentation ions and m/z values of carbohydrate (B) and results from standard spectrum of silylated D-gluconic acid (measurands of **Figures 5** and **S5**)

Intensity data on carbohydrates (B) and silylated D-gluconic acid					
Dataset	N	Mean	sd(yEr±)	se(yEr±)	
Data1_CBB	16	18.47876	24.44037	6.11009	
Data1_NIST	14	22.49264	25.71879	6.87364	
H_0: The means of all selected datasets are equal					
H_1: The means of one or more selected datasets are different					
Source	DoF	Sum of square	Mean square	F value	P value
Model	1	120.296797	120.296797	0.19183	0.66476
Error	28	17558.9050	627.103750		
At the 0.05 level, the population means are not significantly different					
Means Comparison using Bonferroni Test					
Dataset	Mean	Difference between means	Simultaneous Confidence Intervals		Significant at 0,05 Level
Data1_CBB	18.47876		Lower Limit	Upper Limit	
Data1_NIST	22.49264	-4.01387	-22.78639	14.75865	No
Intensity data on carbohydrates (B) and 4DG					
Dataset	N	Mean	sd(yEr±)	se(yEr±)	
Data1_CBB	16	18.47876	24.44037	6.11009	
Data1_4DG	15	18.52754	27.02123	6.97685	
H_0: The means of all selected datasets are equal					
H_1: The means of one or more selected datasets are different					
Source	DoF	Sum of square	Mean square	F value	P value
Model	1	0.01842298	0.0184229786	$2.78524.10^{-5}$	0.99583
Error	29	19182.0282	661.449248		
At the 0.05 level, the population means are not significantly different					
Means Comparison using Bonferroni Test					
Dataset	Mean	Difference between means	Simultaneous Confidence Intervals		Significant at 0,05 Level
Data1_CBB	18.47876		Lower Limit	Upper Limit	
Data1_4DG	18.52754	-0.04878	-18.9533	18.85573	No

Table S3

Shapiro-Wilk normality test data on measurands of carbohydrates (A), (B), and 4DG, respectively

Dataset	N	W	*p*	Decision
CB (A)	10	0.72109	0.00187	Not normal at 0.05 level
CB (B)	16	0.62737	$1.22079.10^{-5}$	Not normal at 0.05 level
4DG	15	0.65963	$5.16588.10^{-5}$	Not normal at 0.05 level
2DG	11	0.69228	$5.99848.10^{-4}$	Not normal at 0.05 level

Table S4

ANOVA data on analysis of datasets of variables intensity of fragmentation ions of silylated 4-deoxy-D-glucose and silylated α-2-deoxy-D-glucose (NIST standard) [https://webbook.nist.gov/cgi/inchi?ID=U79295&Mask=200#]

<table>
<tr><td>Dataset</td><td>N</td><td>Mean</td><td>sd(yEr±)</td><td>se(yEr±)</td><td></td></tr>
<tr><td>Data1_2DG</td><td>11</td><td>23.85794</td><td>28.5489</td><td>8.60782</td><td></td></tr>
<tr><td>Data1_4DG</td><td>15</td><td>18.52754</td><td>27.02123</td><td>6.97685</td><td></td></tr>
<tr><td colspan="6">H_0: The means of all selected datasets are equal</td></tr>
<tr><td colspan="6">H_1: The means of one or more selected datasets are different</td></tr>
<tr><td>Source</td><td>DoF</td><td>Sum of square</td><td>Mean square</td><td>F value</td><td>P value</td></tr>
<tr><td>Model</td><td>1</td><td>180.313794</td><td>180.313794</td><td>0.23554</td><td>0.63184</td></tr>
<tr><td>Error</td><td>24</td><td>18372.4508</td><td>765.518781</td><td></td><td></td></tr>
<tr><td colspan="6">At the 0.05 level, the population means are not significantly different</td></tr>
<tr><td colspan="6">Means Comparison using Bonferroni Test</td></tr>
<tr><td>Dataset</td><td>Mean</td><td rowspan="2">Difference between means</td><td colspan="2">Simultaneous Confidence Intervals</td><td rowspan="2">Significant at 0,05 Level</td></tr>
<tr><td>Data1_2DG</td><td>23.85794</td><td>Lower Limit</td><td>Upper Limit</td></tr>
<tr><td>Data1_4DG</td><td>18.52754</td><td>5.33039</td><td>-17.33749</td><td>27.99828</td><td>No</td></tr>
</table>

Table S5

Thermochemistry (M062X/SDD) of ions of aucubin and chlorogenic acid at GS and TS in gas-phase**;** results from Si-containing fragmentation ions involve PBE0 functional and Douglas-Kroll-Hess approach, as well as; Z-matrixes are given in **Table S6**.

	Aucubin					
	m/z 167		m/z 121		m/z 131	
	167_{a}	167_{a}	121_{b}	121_{b}	131_{b}	131_{b}
	$167_{C8\ O+H2}$	$167_{C6\ O+H2}$	121_{b}	121_{b}	131_{b}	131_{b}
	GS	TS	GS	TS	GS	TS
**E_{ZPVE}**	119.32626	117.36959	65.48905	64.26299	86.75057	85.20305
**E_{corr}**	0.200759	0.198693	0.110840	0.108832	0.144920	0.142857
**H_{corr}**	0.201703	0.199638	0.111784	0.109776	0.145865	0.143802
**G_{corr}**	0.154414	0.148746	0.073387	0.071598	0.106998	0.104034
**ε_0**	0.190159	0.187040	0.104363	0.102410	0.138246	0.135780
**E**	-574.410022	-574.42103	-419.499674	-419.479249	-421.743181	-421.714697
**H**	-574.409078	-574.42009	-419.498729	-419.478304	-421.742237	-421.713753
**G**	-574.456367	-574.47097	-419.537127	-419.516483	-421.781103	-421.75352
	Aucubin		Silylated 4-desoxy-glucose			
	m/z 149		m/z 73* $(SiH(CH_3)_2(C^+H_2))$		m/z 103	
	GS	TS	GS	TS	GS	TS
**E_{ZPVE}**	100.65561	100.40799	78.04071	74.42081	90.77273	90.00465
**E_{corr}**	0.171242	0.170039	0.131025	0.124524	0.155014	0.153733
**H_{corr}**	0.172186	0.170983	0.131969	0.125469	0.155958	0.154677
**G_{corr}**	0.122563	0.123837	0.093807	0.089484	0.109753	0.107768
**ε_0**	0.160405	0.160010	0.124366	0.118597	0.144656	0.143432
**E**	-498.077595	-498.07261	-404.111591	-404.024638	-523.186576	-523.184482
**H**	-498.076650	-498.07167	-404.110647	-404.023694	-523.185632	-523.183538
**G**	-498.126274	-498.11882	-404.148809	-404.059678	-523.231837	-523.230447
	Silylated 4-desoxy-glucose					
	m/z 361		m/z 73 $(Si^+(CH_3)_3)$		m/z 147	
	GS	TS	TS*	TS	GS	TS
**E_{ZPVE}**	290.61667	290.53478	77.82701	70.58159	130.63768	130.61702
**E_{corr}**	0.496600	0.495586	0.130787	0.119229	0.221545	0.220629
**H_{corr}**	0.497544	0.496530	0.131731	0.120173	0.222489	0.221573
**G_{corr}**	0.392974	0.396334	0.091193	0.081941	0.168085	0.169486
**ε_0**	0.463127	0.462997	0.124025	0.112479	0.208184	0.208151
**E**	-1759.54865	-1759.5489	-404.144788	-408.723408	-676.918893	-676.915473
**H**	-1759.54770	-1759.5479	-404.143843	-408.722464	-676.917948	-676.914529
**G**	-1759.65227	-1759.6481	-404.184382	-408.760697	-676.972352	-676.966616
	Silylated 4-desoxy-glucose					
	m/z 205		m/z 45*		m/z 45	
	GS	TS	GS	TS	TS	-
**E_{ZPVE}**	177.15737	176.15495	36.51720	36.15780	32.84378	-
**E_{corr}**	0.302224	0.300450	0.062419	0.061121	0.055782	-
**H_{corr}**	0.303168	0.301395	0.063363	0.062065	0.056726	-
**G_{corr}**	0.234056	0.232895	0.032191	0.033403	0.028243	-
**ε_0**	0.282318	0.280721	0.058194	0.05762	0.052340	-
**E**	-1046.13142	-1046.1176	-326.453462	-326.439950	-330.134613	-
**H**	-1046.13048	-1046.1167	-326.452518	-326.439006	-330.133669	-
**G**	-1046.19959	-1046.1852	-326.483690	-326.467668	-330.162152	-

E_{ZPVE} – zero-point vibrational energy [kcal/mol]; ε_0 – zero-point correction [Hartree/partice]; E_{corr} – thermal correction to energy [Hartree/partice]; H_{corr} – thermal correction to enthalpy [Hartree/partice]; G_{corr} – thermal correction to free energy [Hartree/partice]; E – sum of electronic and thermal energies [Hartree/partice]; H – sum of electronic and thermal enthalpies [Hartree/partice]; G – sum of electronic and thermal free energies [Hartree.(partice)$^{-1}$]

*Sto-3g (basis set)

Table S6

Z-matrixes of mass spectrometric species in ground and transition states

Aucubin							
167 a				167 a			
167_C^8_O$^+$H$_2$				167_C^6_O$^+$H$_2$			
GS	*x*	*y*	*z*	TS	*x*	*y*	*z*
O	2.351351	-1.555009	0.133941	O	-0.0329	-2.54008	0.35608
C	3.070332	-0.411864	-0.297278	C	1.21706	-2.45595	-0.32791
C	0.986289	-1.448539	0.208675	C	-0.83973	-1.47992	0.44817
C	0.382602	-0.244552	0.28317	C	-0.38158	-0.22936	0.06386
C	-1.037103	0.076001	0.011638	C	-1.10321	0.92424	-0.2891
C	-1.880283	-0.882933	-0.832495	C	-2.5648	1.16421	-0.10641
C	1.192083	1.044467	0.366569	C	1.07811	-0.00832	-0.24564
C	0.223531	2.049536	-0.235371	C	1.06626	1.28869	-0.98299
C	2.545082	0.820498	-0.280372	C	1.74287	-1.28021	-0.70077
O	-3.256729	-0.559482	-0.479336	O	-3.10734	0.0642	0.64051
C	-1.011225	1.52535	-0.395878	C	-0.19534	1.84764	-0.92586
O	-1.921525	-0.011408	1.382175	O	2.86601	1.84792	1.25912
H	4.052268	-0.691852	-0.647733	H	1.61167	-3.44511	-0.49747
H	0.501103	-2.41642	0.160443	H	-1.84528	-1.69673	0.78353
H	-1.676609	-0.690897	-1.888929	H	-3.03281	1.25505	-1.09914
H	-1.666381	-1.928139	-0.590365	H	-2.71435	2.12299	0.40955
H	1.370633	1.342885	1.412709	H	1.60645	0.32548	0.67707
H	0.499027	3.0734	-0.461225	H	1.95802	1.76378	-1.374
H	3.120842	1.654296	-0.66245	H	2.67462	-1.26405	-1.25224
H	-3.945374	-0.934731	-1.057213	H	-4.05836	0.15716	0.83617
H	-1.892384	2.028415	-0.774309	H	-0.47324	2.82264	-1.30319
H	-2.879548	-0.149607	1.09574	H	2.73398	2.80744	1.36744
H	-1.696204	0.552057	2.147149	H	3.61527	1.54885	1.80646
121 b				121 b			
C	2.29125	-0.538142	-0.24938	C	2.34878	-0.54599	-0.22793
C	1.874422	0.850944	-0.147572	C	1.94906	0.88312	-0.17763
C	1.234211	-1.369961	0.044879	C	1.32609	-1.41213	0.1007
C	0.543006	0.87211	0.253013	C	0.53765	0.93864	0.32599
C	0.07471	-0.532316	0.525305	C	0.12652	-0.52873	0.52959
C	-0.57243	1.678633	0.005913	C	-0.52066	1.68225	0.01018
O	-1.690432	0.961868	-0.082868	O	-1.63864	0.96545	-0.0786
C	-1.349609	-0.534356	-0.005927	C	-1.2978	-0.53077	-0.00166
O	-2.173629	-1.336964	-0.302148	O	-2.12183	-1.33338	-0.29788
H	3.2799	-0.856668	-0.547999	H	3.33175	-0.85309	-0.54374
H	2.480292	1.704653	-0.429924	H	2.53233	1.70853	-0.4258
H	1.248805	-2.45273	0.056133	H	1.30064	-2.44954	0.06045
H	-0.015047	-0.701932	1.614655	H	0.03672	-0.69836	1.61894
H	-0.654816	2.745968	-0.170116	H	-0.60302	2.74954	-0.16587
131 b				131 b			
O	2.343022	-0.721234	0.000061	O	-2.31199	-0.72601	0.01979
C	2.410686	0.656541	-0.000151	C	-2.3559	0.70311	-0.04563
C	1.187568	-1.378003	0.000378	C	-1.10984	-1.43051	0.06834
C	1.26544	1.394567	-0.000082	C	-1.31725	1.48688	0.0917
C	-0.034368	-0.703147	0.00011	C	0.05876	-0.6947	-0.00009
C	-0.01142	0.739705	-0.000107	C	0.03571	0.74822	0.00013
C	-1.277048	-1.41162	-0.000419	C	1.30142	-1.40316	0.00042
C	-1.237387	1.440932	0.000049	C	1.26174	1.44938	-0.00005
C	-2.456542	-0.696244	-0.000048	C	2.48091	-0.68779	0.00005
C	-2.432575	0.732208	0.000245	C	2.45695	0.74065	-0.00024
H	3.429522	1.011555	-0.00024	H	-3.40497	1.0199	0.00012
H	1.308793	-2.454997	0.000097	H	-1.28443	-2.44737	0.00002
H	1.332731	2.475447	-0.000079	H	-1.30853	2.48527	0.00017
H	-1.279981	-2.496754	-0.000342	H	1.30435	-2.4883	0.00035
H	-1.238455	2.52514	0.000068	H	1.26282	2.53359	-0.00005
H	-3.409362	-1.211653	0.000041	H	3.43373	-1.20319	-0.00003
H	-3.373544	1.271492	0.000121	H	3.39791	1.27995	-0.00012
149				149			
O	-1.92927	-1.78447	-0.13077	O	-1.87578	-1.7852	-0.11637
C	-2.86575	-0.87753	0.26771	C	-2.80268	-0.8577	0.25583
C	-0.64048	-1.436	-0.39295	C	-0.56825	-1.45586	-0.35899
C	-0.25505	-0.1313	-0.2564	C	-0.23475	-0.09314	-0.21329
C	1.02366	0.56905	-0.46254	C	1.07451	0.57362	-0.45608
C	2.21238	0.05728	-0.87203	C	2.26323	0.06184	-0.86557
C	-1.22267	0.85533	0.16598	C	-1.17183	0.8599	0.17244
C	-0.57317	2.13578	0.23078	C	-0.52231	2.14035	0.23723
C	-2.55425	0.45181	0.42916	C	-2.5034	0.45637	0.43562

O	4.07209	-0.99775	0.78091	O	4.12294	-0.99319	0.78736
C	0.7468	1.96854	-0.13765	C	0.79765	1.97311	-0.13119
H	-3.83049	-1.33708	0.42571	H	-3.77961	-1.33249	0.43216
H	-0.03934	-2.28197	-0.69571	H	0.01155	-2.27744	-0.68926
H	2.35669	-0.99476	-1.09351	H	2.40754	-0.9902	-1.08705
H	3.08034	0.69604	-0.99479	H	3.1312	0.7006	-0.98833
H	-1.04907	3.06152	0.51897	H	-0.99822	3.06608	0.52543
H	-3.3208	1.14555	0.74736	H	-3.26995	1.15011	0.75382
H	3.94843	-1.15171	1.73433	H	3.99928	-1.14714	1.74078
H	4.99001	-1.1893	0.51579	H	5.04086	-1.18474	0.52225
H	1.49292	2.7517	-0.19163	H	1.54377	2.75626	-0.18518
Silylated 4-desoxy-glucose							
m/z 103 (TS)				m/z 103 (GS)			
Si	0,55476	-0,00663	0,00425	Si	0,53093	0,08426	-6,51E-04
O	-1,34124	0,01954	-0,03205	O	-1,24638	-0,64365	0,0042
C	0,97552	1,81029	-0,04173	C	0,17869	1,92048	-0,01901
C	0,88076	-0,98988	-1,54835	C	1,15528	-0,67425	-1,58362
C	0,80772	-0,88842	1,62999	C	1,14975	-0,64237	1,59949
C	-2,58384	0,06739	-0,0234	C	-2,43419	-0,255	8,96E-04
H	0,57449	2,33932	0,82817	H	-0,37143	2,24777	0,87016
H	0,59425	2,28849	-0,9508	H	-0,37094	2,23076	-0,91448
H	2,06129	1,95217	-0,03073	H	1,121	2,48161	-0,02395
H	0,42939	-1,98437	-1,49676	H	1,0564	-1,76382	-1,56303
H	1,95943	-1,1275	-1,68762	H	2,21772	-0,44453	-1,72409
H	0,50104	-0,47799	-2,4367	H	0,62076	-0,29452	-2,45954
H	0,37045	-0,33036	2,46332	H	0,62018	-0,23612	2,46647
H	1,87723	-1,00116	1,84063	H	2,21465	-0,42018	1,73319
H	0,3713	-1,89018	1,61486	H	1,03989	-1,73107	1,60513
H	-3,16016	-0,86051	0,0114	H	-3,22622	-1,00559	0,00724
H	-3,09633	1,03225	-0,05783	H	-2,68108	0,812	-0,00809
Silylated 4-desoxy-glucose							
m/z 361 (TS)				m/z 361 (GS)			
C	-1,14895	-1,08658	-1,18211	C	-1,15278	-1,07716	-1,20781
C	-1,15405	0,5076	-1,06334	C	-1,16246	0,50634	-1,07529
C	0,31108	-1,62327	-1,28235	C	0,29808	-1,60468	-1,31721
O	-1,67988	-1,45094	0,08585	O	-1,66896	-1,45913	0,04885
C	0,03381	1,01461	-1,46909	C	0,03151	1,02099	-1,49021
O	-2,25448	1,07713	-0,64096	O	-2,25678	1,0835	-0,66208
C	1,29257	-0,73559	-0,51313	C	1,29027	-0,72922	-0,53424
Si	-2,49305	2,61502	0,28717	Si	-2,49535	2,6214	0,26605
C	2,74395	-1,14061	-0,51283	C	2,74164	-1,13423	-0,53394
O	1,22645	0,59641	-1,34005	O	1,22414	0,60279	-1,36116
O	3,42611	-0,22238	0,32915	O	3,42381	-0,21601	0,30804
Si	5,15925	0,00888	0,45284	Si	5,15695	0,01525	0,43173
C	-3,98187	3,37375	-0,5435	C	-3,98417	3,38012	-0,56462
C	-0,86342	3,51589	0,02391	C	-0,86572	3,52227	0,0028
C	-2,7573	2,02461	2,04381	C	-2,7596	2,03098	2,02269
Si	-3,26415	-2,13473	0,44573	Si	-3,26645	-2,12836	0,42462
C	5,32018	1,34949	1,75032	C	5,31787	1,35586	1,7292
C	5,76903	0,54002	-1,24957	C	5,76672	0,54639	-1,27068
C	-3,21865	-2,24469	2,31315	C	-3,22095	-2,23832	2,29203
C	-3,3126	-3,80451	-0,42085	C	-3,3149	-3,79814	-0,44196
C	-4,58205	-0,96787	-0,22055	C	-4,58435	-0,9615	-0,24167
C	5,90806	-1,63837	0,97676	C	5,90576	-1,63199	0,95565
H	-1,76539	-1,39757	-2,03831	H	-1,7677	-1,3912	-2,05943
H	0,28853	-2,61362	-0,86081	H	0,28622	-2,60724	-0,88193
H	0,63081	-1,68234	-2,3377	H	0,62851	-1,67597	-2,35882
H	0,94492	-0,47515	0,49247	H	0,94261	-0,46878	0,47135
H	3,12611	-1,13372	-1,54587	H	3,1238	-1,12734	-1,56698
H	2,80854	-2,17156	-0,13038	H	2,80624	-2,16519	-0,1515
H	-4,30192	4,28265	-0,02239	H	-4,30422	4,28902	-0,04351
H	-4,82343	2,67311	-0,53972	H	-4,82573	2,67948	-0,56083
H	-3,76518	3,63902	-1,58271	H	-3,76748	3,6454	-1,60383
H	-0,89698	4,50871	0,48918	H	-0,89928	4,51508	0,46806
H	-0,65115	3,67495	-1,04006	H	-0,65345	3,68132	-1,06117
H	-0,01711	2,99184	0,48748	H	-0,01941	2,99821	0,46636
H	-2,82292	2,86962	2,73872	H	-2,82522	2,87599	2,7176
H	-1,92864	1,38615	2,37027	H	-1,93094	1,39252	2,34915
H	-3,68637	1,45169	2,13104	H	-3,68868	1,45807	2,10993
H	6,36984	1,59419	1,94471	H	6,36754	1,60056	1,92359
H	4,86822	1,02786	2,69424	H	4,86592	1,03423	2,67313
H	4,81405	2,26446	1,42587	H	4,81175	2,27083	1,40476

H	6,82993	0,81355	-1,21589	H	6,82763	0,81993	-1,237
H	5,21112	1,41382	-1,60406	H	5,20882	1,42019	-1,62518
H	5,66541	-0,25829	-1,99372	H	5,6631	-0,25192	-2,01484
H	-4,14655	-2,67037	2,70987	H	-4,14885	-2,664	2,68875
H	-3,0859	-1,25306	2,7587	H	-3,0882	-1,24668	2,73758
H	-2,38928	-2,87762	2,64437	H	-2,39158	-2,87125	2,62325
H	-4,25155	-4,32827	-0,20864	H	-4,25385	-4,32189	-0,22975
H	-2,49209	-4,44519	-0,08053	H	-2,49439	-4,43882	-0,10164
H	-3,24007	-3,70334	-1,51045	H	-3,24238	-3,69696	-1,53156
H	-5,57037	-1,43976	-0,17096	H	-5,57267	-1,43339	-0,19208
H	-4,39165	-0,69453	-1,26464	H	-4,39395	-0,68816	-1,28576
H	-4,61787	-0,04072	0,36084	H	-4,62017	-0,03435	0,33972
H	6,99076	-1,54849	1,12208	H	6,98846	-1,54212	1,10096
H	5,7468	-2,41655	0,22103	H	5,74449	-2,41018	0,19991
H	5,47403	-1,98317	1,9216	H	5,47173	-1,9768	1,90048
Silylated 4-desoxy-glucose							
m/z 73 (TS)				m/z 147 (GS)			
Si	$-2{,}00.10^{-5}$	-0,0074	-0,01489	C	3,61444	-0,72514	-0,335
C	-0,57774	1,7552	0,06173	C	2,52557	-0,05683	0,04553
C	-1,23647	-1,3834	-0,03894	O	2,38026	1,3373	0,11809
C	1,81179	-0,38069	-0,0046	C	1,2159	-0,6565	0,4998
H	0,17071	2,45131	-0,3295	O	0,16219	0,31507	0,10059
H	-0,76495	2,04023	1,10629	Si	-1,65334	-0,03368	-0,06466
H	-1,51412	1,9016	-0,47798	C	-1,73496	-1,15006	-1,56088
H	-0,82944	-2,28972	-0,50038	C	-2,28734	1,70785	-0,29731
H	-2,15764	-1,10357	-0,55676	C	-2,04345	-0,85373	1,56885
H	-1,52234	-1,65277	0,99151	H	3,60946	-1,80735	-0,38094
H	2,01679	-1,43923	-0,17263	H	4,5399	-0,2225	-0,59679
H	2,25146	-0,09479	0,96281	H	3,1808	1,86558	-0,05912
H	2,34667	0,20824	-0,75922	H	0,59194	1,21013	0,01232
				H	1,00541	-1,60732	0,01014
				H	1,16284	-0,75314	1,58704
				H	-2,77841	-1,35369	-1,82851
				H	-1,25928	-2,12001	-1,38179
				H	-1,2591	-0,68626	-2,43058
				H	-3,37769	1,70681	-0,4066
				H	-1,8782	2,17306	-1,20039
				H	-2,05267	2,34335	0,56298
				H	-1,77909	-0,21182	2,41534
				H	-1,52353	-1,8113	1,68224
				H	-3,11609	-1,06644	1,64443
Silylated 4-desoxy-glucose							
m/z 205 (GS)							
C	-0,9039	2,25809	0,01455	H	3,07095	0,91411	2,03371
C	0,54724	1,92307	-0,01961	H	3,64138	1,5568	-0,95438
O	-1,82622	1,40939	0,02006	H	2,94771	0,5437	-2,24066
O	0,72153	0,52685	-0,07684	H	4,35525	-0,00707	-1,33034
Si	2,27954	-0,32192	0,00264	H	-1,65788	-2,17039	1,66171
C	2,88523	-0,1342	1,77209	H	-1,92102	-0,61496	2,44951
C	3,40188	0,52664	-1,24353	H	-0,39076	-0,92704	1,59481
Si	-2,22632	-0,43998	-0,00583	H	-2,07157	-0,52221	-2,47859
C	-1,46954	-1,09338	1,57391	H	-1,7254	-2,09923	-1,76528
C	-1,55812	-1,02139	-1,65008	H	-0,48612	-0,82853	-1,72198
C	-4,08667	-0,29082	0,05621	H	-4,41699	0,21701	0,96764
C	1,82004	-2,07865	-0,45826	H	-4,55476	-1,28181	0,04151
H	-1,20062	3,31167	0,03833	H	-4,4703	0,26779	-0,80323
H	0,97948	2,45417	-0,88778	H	2,65817	-2,76011	-0,27439
H	0,99809	2,39398	0,875	H	1,55645	-2,15867	-1,51796
H	3,83003	-0,67049	1,91713	H	0,97018	-2,43552	0,13484
H	2,16313	-0,5427	2,48752				

FILE: UNTITLED-qq3.opj

DESCRIPTION: Tabulated form of quantum chemical data on optimized ion (first stage) at m/z 361 at M062X/SDD level of theory and using Douglas-Kroll-Hess approach in the ground state. There are used atomic coordinates as imput parameters of the molecular ions as listed in **Table S6.**

```
# Total Energy
# Total Energy (Hartree) vs Optimization Step Number
#            X              Y
      1.0000000000   -1759.9015223800
      2.0000000000   -1759.9549135600
```

3.0000000000	-1760.0075333000
4.0000000000	-1760.0263868500
5.0000000000	-1760.0310029200
6.0000000000	-1760.0361605700
7.0000000000	-1760.0379353500
8.0000000000	-1760.0385641300
9.0000000000	-1760.0410469400
10.0000000000	-1760.0426065200
11.0000000000	-1760.0440331900
12.0000000000	-1760.0443382100
13.0000000000	-1760.0446529900
14.0000000000	-1760.0448022000
15.0000000000	-1760.0449325200
16.0000000000	-1760.0451047800

DESCRIPTION: Tabulated form of quantum chemical data on optimized ion (second stage) at m/z 361 at M062X/SDD level of theory and using Douglas-Kroll-Hess approach in the ground state. There are used atomic coordinates as imput parameters of the molecular ions as listed in **Table S6.**

Total Energy
Total Energy (Hartree) vs Optimization Step Number

# X	Y
1.0000000000	-1760.0451047700
2.0000000000	-1760.0451406700
3.0000000000	-1760.0451827000
4.0000000000	-1760.0452039900
5.0000000000	-1760.0452136800
6.0000000000	-1760.0452258400
7.0000000000	-1760.0452338400
8.0000000000	-1760.0452395700
9.0000000000	-1760.0452488200
10.0000000000	-1760.0452506200
11.0000000000	-1760.0452510100
12.0000000000	-1760.0452512600
13.0000000000	-1760.0452514000
14.0000000000	-1760.0452514100
15.0000000000	-1760.0452512000
16.0000000000	-1760.0452510200
17.0000000000	-1760.0452508200
18.0000000000	-1760.0452505800

DESCRIPTION: Tabulated form of quantum chemical data on Born-Oppenheimer molecular dynamics of ion at m/z 73 of $[Si(CH_3)_3]^+$ at M062X/SDD level of theory. There is used Douglas-Kroll-Hess approach to ground state molecular optimization. The atomic coordinates of the ground state optimized geometry are used as imput parameters of computing Born-Oppenheimer molecular dynamics the ion as listed in **Table S6.**

Potential Energy
Potential Energy (Hartree) vs Time in Trajectory (femptosec)

# X	Y
0.0000000000	-408.8425885000
0.0250000000	-408.8425053000
0.0500000000	-408.8422573000
0.0750000000	-408.8418465000
0.1000000000	-408.8412750000
0.1250000000	-408.8405449000
0.1500000000	-408.8396582000
0.1750000000	-408.8386174000
0.2000000000	-408.8374249000
0.2250000000	-408.8360830000
0.2500000000	-408.8345946000
0.2750000000	-408.8329624000
0.3000000000	-408.8311889000
0.3250000000	-408.8292772000
0.3500000000	-408.8272298000
0.3750000000	-408.8250497000
0.4000000000	-408.8227394000
0.4250000000	-408.8203017000
0.4500000000	-408.8177392000
0.4750000000	-408.8150546000
0.5000000000	-408.8122507000
0.5250000000	-408.8093300000
0.5500000000	-408.8062954000
0.5750000000	-408.8031496000
0.6000000000	-408.7998957000
0.6250000000	-408.7965363000
0.6500000000	-408.7930746000
0.6750000000	-408.7895134000
0.7000000000	-408.7858556000
0.7250000000	-408.7821043000
0.7500000000	-408.7782623000
0.7750000000	-408.7743325000
0.8000000000	-408.7703179000
0.8250000000	-408.7662214000
0.8500000000	-408.7620458000
0.8750000000	-408.7577943000
0.9000000000	-408.7534699000
0.9250000000	-408.7490756000
0.9500000000	-408.7446146000
0.9750000000	-408.7400903000
1.0000000000	-408.7355058000
1.0250000000	-408.7308646000
1.0500000000	-408.7261699000
1.0750000000	-408.7214250000
1.1000000000	-408.7166331000
1.1250000000	-408.7117974000
1.1500000000	-408.7069209000
1.1750000000	-408.7020067000
1.2000000000	-408.6970576000
1.2250000000	-408.6920766000

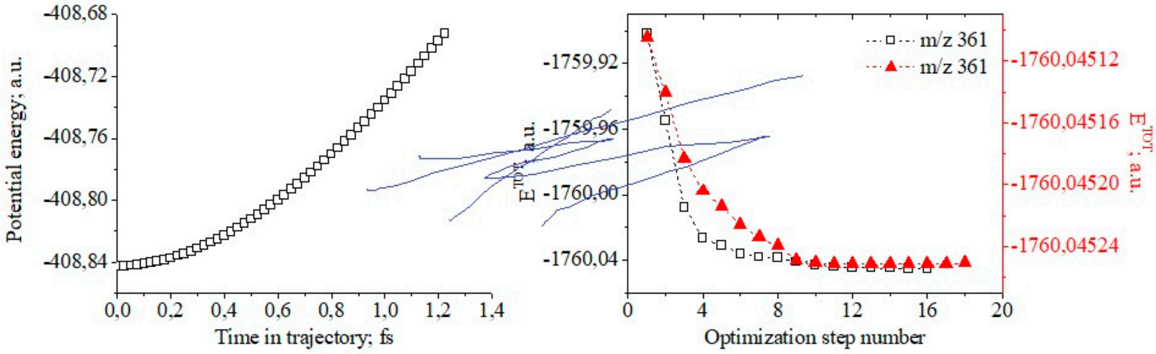

Illustration of tabulated data on file entitled UNTITLED-qq3.opj (see description to tabulated data shown above.)

FILE: UNTITLED-qq1.opj

DESCRIPTION: Tabulated form of molecular dynamics of ion at m/z 205 at MM2 level of theory (Computational detail: Step interval 2 fs; frame interval, 10 fs; targed temperature, 300K, heating/cooling rate 1 kcal/atom/ps). The atomic coordinates are used as imput parameters of computing as listed in **Table S6.**

Time (fs)/Total energy (kcal/mol)/Potential energy (kcal/mol)/Temperature

0.002	3.7121	3.7120	0.00	
0.004	3.8548	3.7502	0.95	heating
0.006	3.9023	3.7791	1.12	heating
0.008	3.9536	3.7948	1.44	heating
0.010	4.0382	3.8557	1.66	heating
0.012	4.0800	3.8586	2.01	heating
0.014	4.1561	3.8857	2.45	heating
0.016	4.2225	3.9278	2.67	heating
0.018	4.2818	3.9090	3.38	heating
0.020	4.3181	3.9236	3.58	heating
0.022	4.3602	3.9364	3.84	heating
0.024	4.4118	3.9668	4.03	heating
0.026	4.4525	4.0251	3.88	heating
0.028	4.5027	4.0110	4.46	heating
0.030	4.5438	3.9885	5.03	heating
0.032	4.5668	3.9839	5.28	heating
0.034	4.6099	3.9948	5.58	heating
0.036	4.6575	4.0404	5.60	heating
0.038	4.6872	4.0774	5.53	heating
0.040	4.7302	4.0988	5.72	heating
0.042	4.7496	4.0980	5.91	heating
0.044	4.7880	4.0746	6.47	heating
0.046	4.8319	4.1021	6.62	heating
0.048	4.8842	4.0987	7.12	heating
0.050	4.9193	4.0896	7.52	heating
0.052	4.9516	4.0908	7.80	heating
0.054	5.0045	4.0881	8.31	heating
0.056	5.0397	4.1586	7.99	heating
0.058	5.0870	4.1504	8.49	heating
0.060	5.1259	4.1351	8.98	heating
0.062	5.1735	4.1888	8.93	heating
0.064	5.2154	4.1485	9.67	heating
0.066	5.2499	4.1863	9.64	heating
0.068	5.3046	4.3043	9.07	heating
0.070	5.3330	4.2764	9.58	heating
0.072	5.3988	4.2465	10.45	heating
0.074	5.4227	4.1676	11.38	heating
0.076	5.4469	4.0887	12.32	heating
0.078	5.5021	4.2352	11.49	heating
0.080	5.5258	4.2642	11.44	heating
0.082	5.5829	4.2934	11.69	heating
0.084	5.6247	4.4803	10.38	heating
0.086	5.6495	4.4027	11.31	heating
0.088	5.7147	4.3533	12.34	heating
0.090	5.7681	4.4149	12.27	heating
0.092	5.8124	4.3202	13.53	heating
0.094	5.8758	4.4455	12.97	heating
0.096	5.8924	4.4851	12.76	heating
0.098	5.9426	4.4741	13.31	heating
0.100	5.9882	4.6830	11.83	heating
0.102	6.0040	4.5547	13.14	heating
0.104	6.0619	4.3863	15.19	heating
0.106	6.1130	4.4901	14.71	heating
0.108	6.1260	4.4913	14.82	heating
0.110	6.1860	4.7030	13.45	heating
0.112	6.2311	4.9533	11.59	heating
0.114	6.2518	4.8571	12.65	heating
0.116	6.3168	4.8993	12.85	heating
0.118	6.3332	4.7259	14.57	heating
0.120	6.3903	4.5040	17.10	heating
0.122	6.4413	4.7241	15.57	heating
0.124	6.4396	4.7575	15.25	heating
0.126	6.4918	4.8295	15.07	heating
0.128	6.5345	5.1000	13.01	heating
0.130	6.5529	5.0534	13.60	heating
0.132	6.6416	5.0806	14.15	heating
0.134	6.6546	4.9861	15.13	heating
0.136	6.7019	4.7388	17.80	heating
0.138	6.7637	5.0012	15.98	heating
0.140	6.7743	5.1989	14.28	heating
0.142	6.8066	5.2817	13.83	heating
0.144	6.8388	5.4833	12.29	heating
0.146	6.8556	5.3465	13.68	heating
0.148	6.9020	5.1604	15.79	heating
0.150	6.9373	5.1616	16.10	heating
0.152	6.9722	5.1381	16.63	heating
0.154	7.0174	5.2610	15.93	heating
0.156	7.0737	5.3429	15.69	heating
0.158	7.1299	5.3671	15.98	heating
0.160	7.1759	5.5966	14.32	heating
0.162	7.2048	5.6712	13.91	heating
0.164	7.3039	5.5793	15.64	heating
0.166	7.3687	5.4653	17.26	heating
0.168	7.4442	5.4372	18.20	heating
0.170	7.4674	5.5343	17.53	heating
0.172	7.5014	5.6486	16.80	heating
0.174	7.5549	5.8058	15.86	heating
0.176	7.5774	5.7064	16.96	heating
0.178	7.6096	5.4722	19.38	heating
0.180	7.6638	5.4525	20.05	heating
0.182	7.6801	5.4527	20.20	heating
0.184	7.7438	5.6891	18.63	heating
0.186	7.7548	5.8771	17.03	heating
0.188	7.7679	5.7454	18.34	heating
0.190	7.8428	5.9595	17.08	heating
0.192	7.8523	5.9982	16.81	heating
0.194	7.9002	5.7219	19.75	heating
0.196	7.9627	5.8604	19.06	heating
0.198	7.9720	5.6582	20.98	heating
0.200	8.0630	5.6237	22.12	heating
0.202	7.9240	5.9792	17.63	heating
0.204	7.9126	5.7858	19.28	heating
0.206	8.0058	5.9419	18.71	heating
0.208	8.0259	5.9708	18.63	heating
0.210	8.0264	5.5408	22.54	heating
0.212	8.1573	5.9471	20.04	heating
0.214	8.1537	6.0977	18.64	heating
0.216	8.2173	5.8534	21.43	heating
0.218	8.2680	6.1721	19.00	heating
0.220	8.2713	5.8374	22.07	heating
0.222	8.3751	5.7212	24.06	heating
0.224	8.4173	5.9594	22.29	heating

0.226	8.4073	5.5470	25.94	heating
0.228	8.5181	5.8682	24.03	heating
0.230	8.4837	6.0230	22.31	heating
0.232	8.5240	5.6003	26.51	heating
0.234	8.6078	5.8713	24.81	heating
0.236	8.6013	5.6981	26.32	heating
0.238	8.6890	5.3388	30.38	heating
0.240	8.7213	5.6687	27.68	heating
0.242	8.7656	5.6159	28.56	heating
0.244	8.8888	5.8582	27.48	heating
0.246	8.8955	6.0900	25.44	heating
0.248	8.8746	5.4755	30.82	heating
0.250	8.9784	5.4019	32.43	heating
0.252	8.9472	5.2674	33.37	heating
0.254	8.9853	4.8825	37.20	heating
0.256	9.0736	5.2191	34.95	heating
0.258	9.0553	5.4325	32.85	heating
0.260	9.1505	5.4785	33.30	heating
0.262	9.1923	5.7195	31.49	heating
0.264	9.1704	5.3907	34.27	heating
0.266	9.2279	5.2857	35.74	heating
0.268	9.2667	5.2743	36.20	heating
0.270	9.2953	4.9339	39.55	heating
0.272	9.3932	5.1651	38.34	heating
0.274	9.4088	5.4309	36.07	heating
0.276	9.4600	5.4553	36.31	heating
0.278	9.5095	5.5838	35.59	heating
0.280	9.5219	5.5233	36.26	heating
0.282	9.5521	5.3264	38.32	heating
0.284	9.5983	5.2741	39.21	heating
0.286	9.6587	5.3116	39.42	heating
0.288	9.6999	5.5259	37.85	heating
0.290	9.7186	5.8029	35.50	heating
0.292	9.7458	5.8495	35.33	heating
0.294	9.8096	5.7137	37.14	heating
0.296	9.8771	5.6055	38.73	heating
0.298	9.9043	5.4271	40.60	heating
0.300	9.9312	5.1038	43.77	heating
0.302	9.9980	5.2288	43.24	heating
0.304	10.0170	5.4771	41.16	heating
0.306	10.0722	5.5199	41.28	heating
0.308	10.1363	5.8556	38.81	heating
0.310	10.1438	5.7351	39.97	heating
0.312	10.2012	5.3586	43.91	heating
0.314	10.2670	5.3094	44.95	heating
0.316	10.2519	4.9516	48.06	heating
0.318	10.3794	5.2317	46.67	heating
0.320	10.3712	5.5623	43.60	heating
0.322	10.3845	5.0875	48.03	heating
0.324	10.4930	5.2507	47.53	heating
0.326	10.4791	5.3555	46.46	heating
0.328	10.5286	4.9212	50.84	heating
0.330	10.6193	5.2791	48.42	heating
0.332	10.6003	5.1162	49.73	heating
0.334	10.6658	5.0076	51.30	heating
0.336	10.7327	5.5200	47.26	heating
0.338	10.6690	5.0685	50.78	heating
0.340	10.8158	5.2473	50.49	heating
0.342	10.7748	5.4393	48.38	heating
0.344	10.7732	4.6787	55.26	heating
0.346	10.8776	4.9556	53.70	heating
0.348	10.8815	5.1816	51.68	heating
0.350	10.9405	4.8405	55.31	heating
0.352	11.0168	5.2558	52.24	heating
0.354	10.9669	4.7610	56.27	heating
0.356	11.0537	4.5744	58.75	heating
0.358	11.1272	5.1872	53.86	heating
0.360	11.0491	4.7354	57.25	heating
0.362	11.1888	5.1345	54.89	heating
0.364	11.1345	5.5015	51.08	heating
0.366	11.1473	4.8238	57.34	heating
0.368	11.2586	4.9528	57.18	heating
0.370	11.2043	4.7396	58.62	heating
0.372	11.2953	4.5083	61.54	heating
0.374	11.3561	5.2197	55.64	heating
0.376	11.3191	5.0483	56.86	heating
0.378	11.4224	5.2990	55.52	heating
0.380	11.4452	5.7771	51.39	heating
0.382	11.4277	5.1625	56.81	heating
0.384	11.5852	5.4136	55.96	heating
0.386	11.5487	5.6799	53.21	heating
0.388	11.6137	5.5190	55.26	heating
0.390	11.7087	6.0628	51.19	heating
0.392	11.6679	6.2475	49.15	heating
0.394	11.7458	6.2629	49.71	heating
0.396	11.8098	6.5919	47.31	heating
0.398	11.8036	6.1478	51.28	heating
0.400	11.9001	6.1242	52.37	heating
0.402	11.9353	6.3721	50.44	heating
0.404	11.9644	6.3634	50.78	heating
0.406	12.0374	6.8437	47.09	heating
0.408	12.0408	7.1943	43.94	heating
0.410	12.0932	7.2468	43.94	heating
0.412	12.1391	7.1732	45.03	heating
0.414	12.1747	6.7967	48.76	heating
0.416	12.2335	6.6608	50.53	heating
0.418	12.2700	6.5684	51.70	heating
0.420	12.3244	6.5548	52.31	heating
0.422	12.4049	6.9652	49.32	heating
0.424	12.4343	7.2524	46.98	heating
0.426	12.4925	7.2474	47.56	heating
0.428	12.4997	6.7906	51.77	heating
0.430	12.5696	6.2945	56.90	heating
0.432	12.6192	6.2595	57.66	heating
0.434	12.6184	6.1750	58.42	heating
0.436	12.6993	6.5389	55.86	heating
0.438	12.7466	7.2614	49.74	heating
0.440	12.7392	7.1080	51.06	heating
0.442	12.8081	7.0241	52.44	heating
0.444	12.8244	6.6989	55.54	heating
0.446	12.8519	6.2128	60.20	heating
0.448	12.9496	6.4401	59.02	heating
0.450	12.9029	6.1170	61.53	heating
0.452	13.0304	6.4281	59.86	heating
0.454	13.0556	7.4062	51.22	heating
0.456	13.0381	7.2147	52.80	heating
0.458	13.1383	7.0997	54.75	heating
0.460	13.1557	6.9410	56.35	heating
0.462	13.1808	6.0025	65.09	heating
0.464	13.2998	6.4321	62.27	heating
0.466	13.2810	6.6443	60.18	heating
0.468	13.3580	6.7470	59.94	heating
0.470	13.4608	7.5733	53.38	heating
0.472	13.3476	6.6481	60.75	heating
0.474	13.5503	6.1506	67.09	heating
0.476	13.5468	6.5671	63.29	heating
0.478	13.5221	5.9244	68.89	heating
0.480	13.6783	6.1832	67.96	heating
0.482	13.6588	6.4338	65.51	heating
0.484	13.6924	5.8453	71.15	heating
0.486	13.8422	6.7797	64.04	heating
0.488	13.7393	6.4768	65.85	heating
0.490	13.8329	5.8616	72.28	heating
0.492	13.9489	6.2564	69.75	heating
0.494	13.8206	5.3078	77.19	heating
0.496	14.0560	5.6173	76.51	heating
0.498	14.0002	6.5736	67.34	heating
0.500	13.9901	5.9243	73.13	heating
0.502	14.0705	6.0511	72.71	heating
0.504	14.0248	5.9518	73.20	heating
0.506	14.0799	5.4255	78.47	heating
0.508	14.1906	6.4659	70.04	heating
0.510	14.0812	6.1065	72.31	heating
0.512	14.2046	5.8219	76.01	heating
0.514	14.2567	6.3597	71.60	heating
0.516	14.1911	5.7705	76.35	heating
0.518	14.3945	6.1228	75.00	heating
0.520	14.3335	6.5287	70.77	heating
0.522	14.3481	5.9382	76.25	heating
0.524	14.4374	6.1101	75.50	heating
0.526	14.4467	6.0144	76.46	heating
0.528	14.5583	5.9333	78.20	heating
0.530	14.6026	6.7348	71.34	heating
0.532	14.5419	6.1255	76.31	heating
0.534	14.6514	5.8783	79.55	heating
0.536	14.7184	6.3309	76.05	heating
0.538	14.7198	6.2103	77.16	heating
0.540	14.8428	6.5693	75.02	heating
0.542	14.8266	6.5993	74.60	heating
0.544	14.8995	6.4000	77.07	heating
0.546	14.9638	6.3361	78.23	heating
0.548	14.9692	5.8024	83.12	heating
0.550	15.0792	5.9124	83.12	heating
0.552	15.1125	6.4338	78.69	heating
0.554	15.1389	6.5281	78.08	heating
0.556	15.1756	6.8866	75.16	heating
0.558	15.2027	7.0675	73.76	heating
0.560	15.2640	7.0967	74.05	heating
0.562	15.2799	6.8365	76.56	heating
0.564	15.2881	6.1396	82.95	heating
0.566	15.3445	6.3619	81.45	heating
0.568	15.3402	6.6796	78.53	heating
0.570	15.3555	6.8041	77.54	heating
0.572	15.4519	7.4698	72.37	heating
0.574	15.4163	7.1553	74.90	heating
0.576	15.4993	6.8811	78.14	heating

0.578	15.4927	6.6642	80.05	heating
0.580	15.5247	6.2896	83.74	heating
0.582	15.6548	7.0455	78.06	heating
0.584	15.5926	7.4294	74.02	heating
0.586	15.7006	7.3338	75.86	heating
0.588	15.7588	8.1210	69.25	heating
0.590	15.7310	7.8854	71.14	heating
0.592	15.8051	7.3001	77.12	heating
0.594	15.8975	7.4496	76.60	heating
0.596	15.8247	6.7261	82.50	heating
0.598	16.0121	7.5179	77.02	heating
0.600	15.9736	8.3659	68.98	heating
0.602	15.9653	8.2158	70.27	heating
0.604	16.1300	9.0306	64.37	heating
0.606	15.9920	8.5314	67.65	heating
0.608	16.1608	7.7705	76.08	heating
0.610	16.2125	8.6249	68.80	heating
0.612	16.1583	8.3310	70.97	heating
0.614	16.2824	8.4468	71.05	heating
0.616	16.3294	9.1857	64.77	heating
0.618	16.2773	8.4524	70.95	heating
0.620	16.4760	9.3269	64.82	heating
0.622	16.3642	9.1638	65.29	heating
0.624	16.4121	7.9500	76.73	heating
0.626	16.5987	8.5485	72.99	heating
0.628	16.4306	8.1474	75.11	heating
0.630	16.6070	8.0639	77.46	heating
0.632	16.6221	9.2714	66.65	heating
0.634	16.5532	8.7781	70.50	heating
0.636	16.6980	8.6607	72.88	heating
0.638	16.6946	8.5291	74.04	heating
0.640	16.6868	7.2583	85.49	heating
0.642	16.8902	8.0768	79.91	heating
0.644	16.7452	7.8316	80.82	heating
0.646	16.8308	6.9936	89.20	heating
0.648	16.9756	7.8428	82.81	heating
0.650	16.8655	7.3233	86.52	heating
0.652	17.0357	6.9546	91.41	heating
0.654	17.0237	7.3481	87.73	heating
0.656	17.0152	6.5444	94.94	heating
0.658	17.1577	6.7444	94.42	heating
0.660	17.1260	6.7442	94.13	heating
0.662	17.1699	6.1520	99.90	heating
0.664	17.3122	7.0489	93.06	heating
0.666	17.2091	6.5266	96.86	heating
0.668	17.3203	5.9303	103.27	heating
0.670	17.3844	6.2668	100.81	heating
0.672	17.3769	6.0266	102.91	heating
0.674	17.5145	6.4510	100.31	heating
0.676	17.4686	6.6653	97.96	heating
0.678	17.5476	6.4161	100.93	heating
0.680	17.6271	6.8731	97.51	heating
0.682	17.5813	6.4929	100.54	heating
0.684	17.6890	6.4429	101.97	heating
0.686	17.7301	7.2204	95.29	heating
0.688	17.7324	7.6046	91.83	heating
0.690	17.7969	8.0582	88.30	heating
0.692	17.7830	7.9410	89.24	heating
0.694	17.8627	7.8574	90.72	heating
0.696	17.9037	8.1609	88.34	heating
0.698	17.8949	7.6900	92.53	heating
0.700	18.0026	8.3312	87.69	heating
0.702	17.9878	9.2515	79.21	heating
0.704	17.9956	9.2914	78.92	heating
0.706	18.1344	9.6534	76.90	heating
0.708	18.0542	9.1119	81.08	heating
0.710	18.1933	9.1396	82.09	heating
0.712	18.1787	9.4321	79.31	heating
0.714	18.1825	8.9526	83.69	heating
0.716	18.3531	9.7492	78.01	heating
0.718	18.2956	10.5624	70.12	heating
0.720	18.3637	10.2505	73.56	heating
0.722	18.4358	10.9504	67.87	heating
0.724	18.4053	10.6013	70.76	heating
0.726	18.4059	9.7167	78.79	heating
0.728	18.5737	10.2412	75.55	heating
0.730	18.4342	9.9128	77.26	heating
0.732	18.6583	11.0466	69.02	heating
0.734	18.5612	11.8174	61.15	heating
0.736	18.5566	11.1194	67.43	heating
0.738	18.7466	11.4705	65.97	heating
0.740	18.6454	11.2254	67.28	heating
0.742	18.7772	10.1156	78.54	heating
0.744	18.8069	10.5128	75.20	heating
0.746	18.8151	10.2269	77.87	heating
0.748	18.8653	10.2906	77.75	heating
0.750	19.0168	11.3219	69.77	heating
0.752	18.8852	10.4237	76.72	heating
0.754	19.1096	10.5478	77.63	heating
0.756	19.0041	10.0168	81.49	heating
0.758	19.0203	8.6018	94.47	heating
0.760	19.1950	8.8363	93.92	heating
0.762	19.1289	9.4639	87.63	heating
0.764	19.2588	9.3946	89.44	heating
0.766	19.2322	9.7849	85.66	heating
0.768	19.2641	9.1166	92.01	heating
0.770	19.2950	8.3774	98.99	heating
0.772	19.4040	8.1353	102.17	heating
0.774	19.3372	7.0653	111.27	heating
0.776	19.5248	7.9862	104.62	heating
0.778	19.4607	8.8442	96.26	heating
0.780	19.4527	8.8668	95.98	heating
0.782	19.5729	9.0615	95.31	heating
0.784	19.5447	8.9238	96.30	heating
0.786	19.6877	8.5225	101.24	heating
0.788	19.6559	8.3802	102.24	heating
0.790	19.7051	8.4607	101.95	heating
0.792	19.7057	9.0416	96.69	heating
0.794	19.7990	9.7251	91.34	heating
0.796	19.8183	9.5733	92.89	heating
0.798	19.8659	9.6060	93.03	heating
0.800	19.9202	9.7904	91.85	heating
0.802	19.9160	9.6677	92.92	heating
0.804	20.0025	9.4683	95.51	heating
0.806	20.1025	10.0243	91.38	heating
0.808	20.1462	10.8134	84.62	heating
0.810	20.1472	10.8013	84.74	heating
0.812	20.1772	10.9828	83.37	heating
0.814	20.1891	10.7404	85.67	heating
0.816	20.2380	10.5809	87.56	heating
0.818	20.3567	10.7636	86.98	heating
0.820	20.2910	10.3419	90.21	heating
0.822	20.4711	11.1800	84.24	heating
0.824	20.3969	11.6391	79.41	heating
0.826	20.4263	11.0666	84.87	heating
0.828	20.5576	11.2741	84.17	heating
0.830	20.5094	11.4319	82.31	heating
0.832	20.6051	11.0066	87.03	heating
0.834	20.6340	11.6844	81.15	heating
0.836	20.6390	11.6245	81.74	heating
0.838	20.6810	11.5675	82.63	heating
0.840	20.8324	11.9569	80.48	heating
0.842	20.7256	10.6162	91.66	heating
0.844	20.9571	11.0310	90.00	heating
0.846	20.8616	11.0156	89.28	heating
0.848	20.8760	10.1571	97.19	heating
0.850	21.0654	10.5642	95.22	heating
0.852	20.9499	10.7302	92.66	heating
0.854	21.1197	10.6168	95.23	heating
0.856	21.0611	11.2262	89.17	heating
0.858	21.1245	10.4337	96.94	heating
0.860	21.1787	9.8573	102.65	heating
0.862	21.3174	10.2892	99.99	heating
0.864	21.2234	9.5169	106.14	heating
0.866	21.4659	10.7115	97.51	heating
0.868	21.3367	11.1627	92.25	heating
0.870	21.3764	10.1133	102.12	heating
0.872	21.5585	10.0014	104.79	heating
0.874	21.4329	9.4146	108.97	heating
0.876	21.6295	9.1226	113.40	heating
0.878	21.5884	9.9799	105.26	heating
0.880	21.5887	9.6590	108.17	heating
0.882	21.6733	10.0542	105.35	heating
0.884	21.7585	10.9527	97.98	heating
0.886	21.6273	9.6274	108.80	heating
0.888	21.8344	9.3232	113.44	heating
0.890	21.7586	8.9797	115.87	heating
0.892	21.7898	8.1433	123.74	heating
0.894	21.9085	8.5377	121.23	heating
0.896	21.9132	9.2912	114.45	heating
0.898	22.0275	9.4398	114.13	heating
0.900	22.0332	9.6656	112.14	heating
0.902	21.9956	8.5096	122.28	heating
0.904	22.0719	8.0514	127.13	heating
0.906	22.1577	8.8011	121.11	heating
0.908	22.0872	8.6729	121.63	heating
0.910	22.2321	9.2348	117.85	heating
0.912	22.2218	10.1504	109.45	heating
0.914	22.2720	10.3918	107.72	heating
0.916	22.3167	10.2204	109.68	heating
0.918	22.3580	10.0292	111.79	heating
0.920	22.4172	9.9153	113.36	heating
0.922	22.4594	10.4352	109.02	heating
0.924	22.4877	11.1526	102.78	heating
0.926	22.5412	11.9108	96.39	heating
0.928	22.6493	12.9517	87.93	heating

0.930	22.6626	13.2009	85.79	heating
0.932	22.6619	12.2370	94.52	heating
0.934	22.7684	12.1398	96.37	heating
0.936	22.7625	12.4415	93.58	heating
0.938	22.7571	12.0355	97.21	heating
0.940	22.8863	12.5541	93.68	heating
0.942	22.9032	13.1663	88.29	heating
0.944	22.9639	13.4637	86.14	heating
0.946	22.9876	13.8514	82.84	heating
0.948	22.9163	12.6588	93.01	heating
0.950	23.0177	11.9231	100.60	heating
0.952	23.0898	12.5177	95.86	heating
0.954	22.9966	11.9547	100.12	heating
0.956	23.2446	12.8659	94.11	heating
0.958	23.2014	13.8849	84.47	heating
0.960	23.2077	13.2017	90.73	heating
0.962	23.3588	12.8843	94.97	heating
0.964	23.3110	12.1024	101.63	heating
0.966	23.4527	11.4105	109.19	heating
0.968	23.5077	12.5760	99.12	heating
0.970	23.4360	12.1947	101.93	heating
0.972	23.5838	12.5287	100.24	heating
0.974	23.6844	13.5726	91.68	heating
0.976	23.5218	11.9364	105.05	heating
0.978	23.8340	12.1071	106.33	heating
0.980	23.6891	12.3051	103.22	heating
0.982	23.7628	11.6688	109.66	heating
0.984	23.8908	12.3273	104.85	heating
0.986	23.7714	12.0984	105.84	heating
0.988	23.9369	11.4995	112.77	heating
0.990	23.9314	12.4797	103.83	heating
0.992	23.8563	11.1282	115.41	heating
0.994	23.9511	10.6365	120.73	heating
0.996	24.1225	11.8521	111.26	heating
0.998	23.9146	10.8387	118.56	heating
1.000	24.2890	11.8232	113.03	heating
1.002	24.1033	12.3282	106.77	heating
1.004	24.1635	11.1839	117.69	heating
1.006	24.2985	11.1886	118.87	heating
1.008	24.2194	10.9415	120.39	heating
1.010	24.4333	10.6979	124.54	heating
1.012	24.4214	12.1756	111.03	heating
1.014	24.3633	11.2713	118.71	heating
1.016	24.4448	10.8909	122.90	heating
1.018	24.6064	12.1288	113.14	heating
1.020	24.4251	11.6666	115.68	heating
1.022	24.6848	12.2429	112.81	heating
1.024	24.5817	12.6774	107.94	heating
1.026	24.6748	12.6822	108.74	heating
1.028	24.7563	13.4465	102.55	heating
1.030	24.7675	14.2239	95.60	heating
1.032	24.8921	14.2651	96.36	heating
1.034	24.8850	14.4945	94.21	heating
1.036	24.8966	13.0089	107.79	heating
1.038	24.9443	12.2063	115.50	heating
1.040	25.0958	13.3427	106.57	heating
1.042	24.9924	14.1838	98.00	heating
1.044	25.1194	14.7951	93.61	heating
1.046	25.0815	14.6575	94.52	heating
1.048	25.1738	14.6986	94.98	heating
1.050	25.1462	14.2268	99.01	heating
1.052	25.1971	13.4990	106.07	heating
1.054	25.3101	13.3344	108.59	heating
1.056	25.2877	13.6814	105.24	heating
1.058	25.3584	14.3335	99.96	heating
1.060	25.3046	14.1543	101.10	heating
1.062	25.4011	13.5205	107.72	heating
1.064	25.5013	13.5308	108.54	heating
1.066	25.4332	12.6848	115.59	heating
1.068	25.5258	12.1018	121.72	heating
1.070	25.5772	12.9877	114.15	heating
1.072	25.5486	13.3689	110.44	heating
1.074	25.6683	13.9634	106.13	heating
1.076	25.6822	14.3230	103.00	heating
1.078	25.6745	13.1557	113.51	heating
1.080	25.7942	13.3564	112.78	heating
1.082	25.6950	12.4188	120.38	heating
1.084	25.7900	11.3217	131.19	heating
1.086	26.0031	12.7605	120.07	heating
1.088	25.7534	12.4082	121.00	heating
1.090	26.0499	12.7382	120.70	heating
1.092	25.9855	13.5575	112.69	heating
1.094	25.9483	12.4360	122.52	heating
1.096	26.0977	12.1542	126.43	heating
1.098	26.0247	11.7501	129.43	heating
1.100	26.1066	10.5546	141.01	heating
1.102	26.2338	12.2890	126.44	heating
1.104	26.1510	12.3395	125.23	heating
1.106	26.2402	11.9211	129.83	heating
1.108	26.4583	12.9653	122.34	heating
1.110	26.1639	11.0938	136.64	heating
1.112	26.5727	10.9632	141.53	heating
1.114	26.4201	11.7792	132.75	heating
1.116	26.4268	11.6824	133.69	heating
1.118	26.5917	12.9281	123.89	heating
1.120	26.4617	13.0260	121.82	heating
1.122	26.6059	12.3980	128.83	heating
1.124	26.6550	14.2906	112.11	heating
1.126	26.5610	13.8527	115.23	heating
1.128	26.6357	13.5965	118.23	heating
1.130	26.7657	14.9520	107.12	heating
1.132	26.5794	14.4021	110.41	heating
1.134	26.9400	15.4007	104.63	heating
1.136	26.7446	15.8553	98.73	heating
1.138	26.8092	15.2668	104.66	heating
1.140	26.9696	15.8150	101.14	heating
1.142	26.8624	15.1946	105.79	heating
1.144	27.0237	14.8105	110.74	heating
1.146	27.0832	16.7636	93.57	heating
1.148	27.0154	16.3331	96.86	heating
1.150	27.1035	15.6116	104.20	heating
1.152	27.1808	15.4457	106.40	heating
1.154	27.1571	14.8637	111.47	heating
1.156	27.3555	15.3239	109.09	heating
1.158	27.1877	14.7963	112.35	heating
1.160	27.3311	14.5849	115.57	heating
1.162	27.3582	15.2578	109.72	heating
1.164	27.3672	14.7070	114.79	heating
1.166	27.4748	14.2291	120.10	heating
1.168	27.4991	14.9632	113.66	heating
1.170	27.4473	14.4391	117.95	heating
1.172	27.5626	14.6502	117.08	heating
1.174	27.5775	15.0172	113.89	heating
1.176	27.6251	15.9352	105.99	heating
1.178	27.7375	16.9814	97.53	heating
1.180	27.5696	15.6649	107.94	heating
1.182	27.7854	14.6747	118.88	heating
1.184	27.6884	14.1722	122.55	heating
1.186	27.8353	13.7701	127.53	heating
1.188	27.8376	14.0292	125.20	heating
1.190	27.8459	14.2250	123.50	heating
1.192	27.9594	14.7155	120.08	heating
1.194	27.9505	15.0113	117.32	heating
1.196	27.9829	13.5868	130.53	heating
1.198	28.1327	13.5132	132.56	heating
1.200	28.1195	12.8514	138.44	heating
1.202	28.0918	11.1989	153.17	heating
1.204	28.3091	11.7619	150.04	heating
1.206	28.1955	12.3257	143.89	heating
1.208	28.3372	12.8488	140.44	heating
1.210	28.3302	13.1238	137.88	heating
1.212	28.2778	11.5016	152.11	heating
1.214	28.4916	11.7755	151.57	heating
1.216	28.3949	12.4059	144.98	heating
1.218	28.4165	11.6749	151.80	heating
1.220	28.6231	13.5846	136.36	heating
1.222	28.4602	14.4752	126.80	heating
1.224	28.6670	14.8816	124.99	heating
1.226	28.7649	16.1236	114.62	heating
1.228	28.6236	14.7333	125.95	heating
1.230	28.8403	14.4566	130.42	heating
1.232	28.8433	14.8063	127.28	heating
1.234	28.7989	13.6031	137.78	heating
1.236	29.0772	16.0501	118.12	heating
1.238	28.8913	17.2421	105.63	heating
1.240	28.9558	16.6328	111.73	heating
1.242	29.2280	17.9063	102.66	heating
1.244	28.9439	16.8808	109.38	heating
1.246	29.2972	16.5621	115.47	heating
1.248	29.1885	17.6054	105.03	heating
1.250	29.2075	16.9065	111.53	heating
1.252	29.3873	18.1081	102.27	heating
1.254	29.3544	18.6814	96.77	heating
1.256	29.3063	16.5665	115.51	heating
1.258	29.6340	17.4843	110.16	heating
1.260	29.4109	16.2566	119.27	heating
1.262	29.5227	14.6350	134.99	heating
1.264	29.6854	14.9803	133.33	heating
1.266	29.5028	14.2206	138.57	heating
1.268	29.8663	14.8996	135.71	heating
1.270	29.6180	15.7096	126.11	heating
1.272	29.7260	15.1147	132.48	heating
1.274	29.7656	15.7581	127.01	heating
1.276	29.7881	15.4188	130.29	heating
1.278	29.7935	13.9099	144.02	heating
1.280	29.9758	15.4923	131.32	heating

1.282	29.9238	16.0623	125.68	heating
1.284	29.9630	16.2259	124.56	heating
1.286	29.9983	15.6176	130.39	heating
1.288	30.0433	15.2953	133.72	heating
1.290	30.2979	16.1966	127.86	heating
1.292	30.0831	15.8031	129.48	heating
1.294	30.2576	15.5199	133.63	heating
1.296	30.1854	15.5500	132.70	heating
1.298	30.3539	15.4549	135.09	heating
1.300	30.3827	14.9835	139.63	heating
1.302	30.4038	14.6726	142.64	heating
1.304	30.4460	14.0573	148.60	heating
1.306	30.4506	13.9277	149.82	heating
1.308	30.4481	12.7360	160.60	heating
1.310	30.5984	13.4048	155.90	heating
1.312	30.7121	15.1571	141.04	heating
1.314	30.5891	15.0732	140.69	heating
1.316	30.7747	15.5988	137.60	heating
1.318	30.6004	15.1647	139.96	heating
1.320	30.8405	15.6263	137.95	heating
1.322	30.9072	17.3809	122.64	heating
1.324	30.7660	16.8862	125.85	heating
1.326	31.0067	17.9460	118.42	heating
1.328	30.9050	18.7111	110.56	heating
1.330	30.8515	16.3032	131.91	heating
1.332	31.1123	16.7439	130.28	heating
1.334	31.0227	17.3154	124.29	heating
1.336	31.0973	16.6132	131.33	heating
1.338	31.2504	18.3360	117.10	heating
1.340	31.0709	18.3044	115.76	heating
1.342	31.2190	18.4434	115.84	heating
1.344	31.3875	19.8997	104.16	heating
1.346	31.0999	17.5908	122.49	heating
1.348	31.5016	18.1297	121.24	heating
1.350	31.3191	18.8437	113.12	heating
1.352	31.2527	16.3264	135.34	heating
1.354	31.6658	17.5103	128.35	heating
1.356	31.3824	17.8056	123.10	heating
1.358	31.5803	16.4249	137.42	heating
1.360	31.6785	17.9233	124.72	heating
1.362	31.4585	16.0547	139.67	heating
1.364	31.6568	15.2251	148.99	heating
1.366	31.8304	17.2800	131.93	heating
1.368	31.4692	15.3294	146.34	heating
1.370	31.9609	17.0857	134.88	heating
1.372	31.7199	18.8437	116.75	heating
1.374	31.6661	16.2384	139.89	heating
1.376	32.0391	16.1609	143.97	heating
1.378	31.7946	15.3133	149.44	heating
1.380	32.0151	13.2555	170.10	heating
1.382	32.1540	15.6110	150.00	heating
1.384	31.9277	15.2140	151.55	heating
1.386	32.0793	15.0935	154.01	heating
1.388	32.2909	17.3775	135.22	heating
1.390	31.9320	14.9526	153.96	heating
1.392	32.4201	14.8965	158.89	heating
1.394	32.2090	15.8425	148.40	heating
1.396	32.2008	14.3405	161.94	heating
1.398	32.4140	14.5156	162.29	heating
1.400	32.3225	14.8170	158.72	heating
1.402	32.4355	13.5042	171.65	heating
1.404	32.5470	14.6787	162.01	heating
1.406	32.3795	13.6303	170.00	heating
1.408	32.4259	12.3976	181.60	heating
1.410	32.7094	14.0265	169.40	heating
1.412	32.4476	13.9908	167.35	heating
1.414	32.7529	14.5874	164.71	heating
1.416	32.6769	16.8485	143.52	heating
1.418	32.6817	17.8005	134.93	heating
1.420	32.6620	17.3106	139.19	heating
1.422	32.9314	18.4286	131.50	heating
1.424	32.7888	17.6529	137.24	heating
1.426	32.9176	17.7064	137.92	heating
1.428	32.9674	18.5312	130.90	heating
1.430	32.8539	18.2982	131.98	heating
1.432	33.1574	19.4782	124.03	heating
1.434	32.9735	20.1125	116.61	heating
1.436	33.0760	18.4876	132.28	heating
1.438	33.1524	19.4012	124.68	heating
1.440	33.1316	20.2490	116.81	heating
1.442	33.0106	18.2797	133.57	heating
1.444	33.4214	19.2167	128.80	heating
1.446	33.1685	18.6998	131.19	heating
1.448	33.3383	18.3149	136.22	heating
1.450	33.4638	19.9107	122.89	heating
1.452	33.1983	18.3273	134.84	heating
1.454	33.5722	17.7902	143.10	heating
1.456	33.5182	19.6538	125.71	heating
1.458	33.3772	17.8533	140.76	heating
1.460	33.6706	18.9758	133.24	heating
1.462	33.5552	20.1585	121.47	heating
1.464	33.4131	17.7461	142.05	heating
1.466	33.8427	19.5816	129.31	heating
1.468	33.5262	19.5628	126.61	heating
1.470	33.7016	17.9305	143.00	heating
1.472	33.9066	19.7367	128.48	heating
1.474	33.5545	17.7973	142.87	heating
1.476	33.8928	16.4189	158.44	heating
1.478	34.0502	18.8206	138.09	heating
1.480	33.6611	16.0316	159.85	heating
1.482	34.1538	15.8420	166.04	heating
1.484	34.0042	17.3562	150.95	heating
1.486	33.8644	14.9662	171.35	heating
1.488	34.2801	17.2370	154.53	heating
1.490	33.9966	18.3223	142.12	heating
1.492	34.0959	16.5377	159.20	heating
1.494	34.4343	19.6372	134.17	heating
1.496	33.9934	18.1026	144.08	heating
1.498	34.3039	16.2955	163.29	heating
1.500	34.4946	18.0614	149.00	heating
1.502	34.0744	15.8297	165.43	heating
1.504	34.6627	16.8418	161.59	heating
1.506	34.3873	19.0790	138.80	heating
1.508	34.3489	16.2904	163.74	heating
1.510	34.5480	15.9606	168.53	heating
1.512	34.5234	16.2038	166.11	heating
1.514	34.4614	14.1357	184.30	heating
1.516	34.8378	17.6020	156.28	heating
1.518	34.4530	17.5752	153.03	heating
1.520	34.6032	16.4809	164.32	heating
1.522	34.8678	17.9895	153.04	heating
1.524	34.4528	16.4564	163.18	heating
1.526	35.0564	16.4189	168.99	heating
1.528	34.7446	17.4885	156.46	heating
1.530	34.8643	16.5385	166.16	heating
1.532	34.8638	16.9127	162.77	heating
1.534	35.0135	18.6439	148.43	heating
1.536	34.9180	18.3677	150.06	heating
1.538	35.1292	20.3928	133.62	heating
1.540	34.9494	19.4768	140.29	heating
1.542	34.9815	17.8650	155.20	heating
1.544	35.2477	17.9581	156.77	heating
1.546	35.0241	17.6522	157.51	heating
1.548	35.3977	17.6986	160.48	heating
1.550	35.1888	18.4740	151.56	heating
1.552	35.3766	19.2681	146.06	heating
1.554	35.1926	18.5442	150.95	heating
1.556	35.5299	18.8053	151.64	heating
1.558	35.4157	18.1613	156.45	heating
1.560	35.5266	18.2243	156.88	heating
1.562	35.5833	19.0291	150.10	heating
1.564	35.4451	19.3634	145.81	heating
1.566	35.6541	19.4342	147.07	heating
1.568	35.7023	20.8644	134.54	heating
1.570	35.7318	19.8631	143.88	heating
1.572	35.7374	18.8347	153.26	heating
1.574	35.8208	18.4500	157.50	heating
1.576	35.6046	15.7378	180.14	heating
1.578	36.0417	16.1324	180.52	heating
1.580	35.8577	16.6170	174.46	heating
1.582	35.8876	15.8090	182.06	heating
1.584	36.1139	17.8133	165.93	heating
1.586	35.8457	17.5350	166.03	heating
1.588	36.0381	16.3884	178.17	heating
1.590	36.2522	18.7467	158.73	heating
1.592	35.9465	17.3628	168.50	heating
1.594	36.2854	17.7885	167.71	heating
1.596	36.1899	19.8115	148.51	heating
1.598	36.0035	18.3978	159.63	heating
1.600	36.5293	20.3278	146.90	heating
1.602	36.3043	20.9096	139.59	heating
1.604	36.3321	18.1655	164.72	heating
1.606	36.6276	20.0768	150.07	heating
1.608	36.2913	19.9374	148.28	heating
1.610	36.5091	19.3289	155.78	heating
1.612	36.7485	22.2184	131.75	heating
1.614	36.3657	20.0360	148.06	heating
1.616	36.8327	18.9107	162.50	heating
1.618	36.6883	19.6940	154.09	heating
1.620	36.5599	17.1501	175.99	heating
1.622	36.9178	18.0197	171.35	heating
1.624	36.7795	18.4233	166.44	heating
1.626	36.8027	16.2079	186.74	heating
1.628	37.1128	18.4614	169.12	heating
1.630	36.7049	17.3072	175.88	heating
1.632	36.9885	16.7593	183.42	heating

1.634	37.0865	18.7841	165.95	heating
1.636	36.8076	17.0670	178.99	heating
1.638	37.2526	16.8264	185.21	heating
1.640	37.1049	18.9831	164.31	heating
1.642	37.0735	18.3751	169.54	heating
1.644	37.2177	19.3450	162.05	heating
1.646	37.2264	19.5313	160.44	heating
1.648	37.0577	16.9659	182.18	heating
1.650	37.4671	18.5215	171.78	heating
1.652	37.1325	18.3101	170.67	heating
1.654	37.3450	17.6984	178.14	heating
1.656	37.4212	18.4143	172.34	heating
1.658	37.2929	17.3625	180.71	heating
1.660	37.5531	16.5788	190.18	heating
1.662	37.4647	18.0539	176.00	heating
1.664	37.5407	18.8971	169.04	heating
1.666	37.5192	20.1604	157.39	heating
1.668	37.6542	20.6663	154.03	heating
1.670	37.4727	19.8659	159.64	heating
1.672	37.8823	21.3357	150.03	heating
1.674	37.6129	21.4425	146.62	heating
1.676	37.7533	20.6540	155.04	heating
1.678	37.6674	19.6727	163.16	heating
1.680	37.8338	19.8831	162.76	heating
1.682	37.8457	20.1287	160.64	heating
1.684	37.8638	22.2469	141.60	heating
1.686	37.8635	22.6308	138.12	heating
1.688	37.8134	21.8570	144.68	heating
1.690	38.0478	20.4375	159.68	heating
1.692	37.8677	18.8354	172.57	heating
1.694	38.2003	18.9907	174.18	heating
1.696	38.0181	19.9295	164.01	heating
1.698	38.1358	20.2895	161.82	heating
1.700	38.0066	18.9788	172.53	heating
1.702	38.3026	18.9923	175.09	heating
1.704	38.2887	18.5428	179.04	heating
1.706	38.3314	18.7589	177.47	heating
1.708	38.3778	18.9658	176.01	heating
1.710	38.2878	18.3897	180.42	heating
1.712	38.4812	17.6354	189.01	heating
1.714	38.5004	18.6565	179.93	heating
1.716	38.5999	19.3816	174.26	heating
1.718	38.5795	20.0310	168.18	heating
1.720	38.5888	20.2148	166.60	heating
1.722	38.5104	18.7586	179.09	heating
1.724	38.7484	18.9362	179.64	heating
1.726	38.6945	19.4031	174.92	heating
1.728	38.8088	20.2759	168.04	heating
1.730	38.8514	21.5517	156.86	heating
1.732	38.7589	20.4375	166.12	heating
1.734	38.8492	18.8275	181.54	heating
1.736	39.0466	19.6889	175.52	heating
1.738	38.9463	19.9546	172.20	heating
1.740	39.1090	20.9740	164.43	heating
1.742	39.0803	22.1135	153.84	heating
1.744	39.0242	21.1854	161.75	heating
1.746	39.2641	22.2010	154.71	heating
1.748	39.2225	23.5871	141.77	heating
1.750	39.1913	21.8905	156.87	heating
1.752	39.3444	21.5593	161.26	heating
1.754	39.2416	20.4646	170.25	heating
1.756	39.2952	20.2521	172.67	heating
1.758	39.5006	22.3350	155.64	heating
1.760	39.2757	22.6536	150.72	heating
1.762	39.5619	22.8334	151.68	heating
1.764	39.4283	22.8275	150.52	heating
1.766	39.4925	21.6072	162.17	heating
1.768	39.5560	21.6448	162.40	heating
1.770	39.7375	23.4705	147.49	heating
1.772	39.5651	21.2364	166.19	heating
1.774	39.8240	20.6371	173.97	heating
1.776	39.8323	20.8660	171.97	heating
1.778	39.6524	19.8442	179.60	heating
1.780	39.9803	20.1762	179.57	heating
1.782	39.6843	19.5246	182.79	heating
1.784	40.0823	18.7159	193.73	heating
1.786	39.9354	20.3135	177.92	heating
1.788	39.9750	20.9001	172.95	heating
1.790	39.9239	20.1468	179.32	heating
1.792	40.2602	21.3727	171.26	heating
1.794	39.9501	18.6451	193.18	heating
1.796	40.1961	17.5056	205.74	heating
1.798	40.3240	19.1287	192.18	heating
1.800	40.0585	19.2484	188.69	heating
1.802	40.3241	19.7432	186.61	heating
1.804	40.2250	20.7268	176.79	heating
1.806	40.4215	19.0579	193.71	heating
1.808	40.4234	19.2552	191.94	heating
1.810	40.4349	19.7993	187.11	heating
1.812	40.2984	18.7982	194.95	heating
1.814	40.7708	21.8506	171.55	heating
1.816	40.3868	21.9377	167.28	heating
1.818	40.6485	21.7249	171.58	heating
1.820	40.7396	22.8441	162.26	heating
1.822	40.4820	21.8713	168.75	heating
1.824	40.6923	21.1381	177.30	heating
1.826	40.6603	22.2681	166.76	heating
1.828	40.7224	21.1207	177.73	heating
1.830	40.8150	22.2162	168.64	heating
1.832	40.8751	24.5551	147.98	heating
1.834	40.6064	23.4137	155.89	heating
1.836	41.0887	24.4597	150.78	heating
1.838	40.8514	24.5079	148.19	heating
1.840	40.9432	23.0654	162.10	heating
1.842	41.1920	25.5749	141.60	heating
1.844	40.9246	26.8310	127.79	heating
1.846	40.9438	25.1715	143.01	heating
1.848	41.2265	26.2579	135.72	heating
1.850	41.0191	23.5336	158.54	heating
1.852	41.2153	21.6477	177.42	heating
1.854	41.3196	23.3047	163.34	heating
1.856	40.9805	21.0208	180.98	heating
1.858	41.4696	21.6980	179.27	heating
1.860	41.3396	23.2761	163.78	heating
1.862	41.2589	21.7932	176.50	heating
1.864	41.6418	23.9768	160.17	heating
1.866	41.3084	24.0733	156.27	heating
1.868	41.3628	20.5846	188.40	heating
1.870	41.6242	21.0465	186.58	heating
1.872	41.4547	19.8801	195.62	heating
1.874	41.5798	18.6044	208.32	heating
1.876	41.7497	22.3092	176.27	heating
1.878	41.3661	21.3391	181.59	heating
1.880	41.6802	20.5639	191.46	heating
1.882	41.8009	22.0605	178.99	heating
1.884	41.4026	19.9942	194.11	heating
1.886	41.9237	20.9263	190.39	heating
1.888	41.5960	22.7124	171.22	heating
1.890	41.6790	21.9707	178.70	heating
1.892	41.8112	22.7147	173.15	heating
1.894	41.7919	22.4032	175.80	heating
1.896	41.9160	20.5398	193.82	heating
1.898	42.0441	22.6323	176.01	heating
1.900	41.7932	21.8858	180.50	heating
1.902	41.8606	20.4666	193.98	heating
1.904	42.1950	21.8101	184.83	heating
1.906	41.8836	21.5821	184.08	heating
1.908	42.3506	22.9304	176.09	heating
1.910	42.1508	25.0297	155.24	heating
1.912	42.0937	23.9283	164.71	heating
1.914	42.1324	22.7252	175.97	heating
1.916	42.3478	23.0407	175.06	heating
1.918	42.3128	21.6937	186.96	heating
1.920	42.4911	25.1606	157.14	heating
1.922	42.3004	26.0051	147.75	heating
1.924	42.2035	23.4488	170.05	heating
1.926	42.7276	24.1365	168.57	heating
1.928	42.2799	24.0525	165.27	heating
1.930	42.7199	23.2088	176.91	heating
1.932	42.6139	24.3909	165.23	heating
1.934	42.5621	23.8670	169.51	heating
1.936	42.5158	22.4518	181.92	heating
1.938	42.9329	24.9336	163.20	heating
1.940	42.6489	23.4136	174.41	heating
1.942	42.8944	23.9177	172.06	heating
1.944	42.8526	24.3082	168.14	heating
1.946	42.6049	21.1827	194.24	heating
1.948	43.2292	21.4340	197.62	heating
1.950	42.7561	21.7153	190.78	heating
1.952	43.1054	20.5511	204.50	heating
1.954	43.0852	21.6266	194.57	heating
1.956	43.0281	21.1170	198.67	heating
1.958	42.9805	19.4869	213.02	heating
1.960	43.4559	23.1209	184.38	heating
1.962	43.0392	22.6758	184.64	heating
1.964	43.4359	23.2062	183.43	heating
1.966	43.3494	23.9979	175.46	heating
1.968	43.0661	20.9879	200.19	heating
1.970	43.5986	21.4614	200.72	heating
1.972	43.3168	22.9891	184.31	heating
1.974	43.4418	21.2040	201.63	heating
1.976	43.5923	22.9549	187.12	heating
1.978	43.4335	22.9077	186.11	heating
1.980	43.3612	20.8359	204.24	heating
1.982	43.8732	24.3950	176.61	heating
1.984	43.4526	24.1997	174.57	heating

1.986	43.7888	23.9944	179.48	heating
1.988	43.7761	24.6058	173.82	heating
1.990	43.4351	21.1473	202.09	heating
1.992	43.9953	22.0117	199.33	heating
1.994	43.7863	24.4750	175.10	heating
1.996	43.7748	22.6696	191.36	heating
1.998	44.0728	24.2745	179.51	heating
2.000	43.7906	23.9205	180.16	heating
2.002	43.8559	21.5378	202.36	heating
2.004	44.2262	24.5561	178.35	heating
2.006	43.9408	24.2920	178.16	heating
2.008	44.1772	23.5735	186.82	heating
2.010	44.3425	25.7135	168.91	heating
2.012	43.9246	23.5967	184.32	heating
2.014	44.3970	23.8567	186.24	heating
2.016	44.2599	24.8513	175.98	heating
2.018	44.2056	22.8135	193.97	heating
2.020	44.5887	23.2710	193.29	heating
2.022	44.2447	23.5522	187.62	heating
2.024	44.5146	22.7027	197.77	heating
2.026	44.5163	23.7406	188.38	heating
2.028	44.5664	21.9850	204.75	heating
2.030	44.5901	20.0368	222.63	heating
2.032	44.8077	20.8865	216.90	heating
2.034	44.5874	19.9622	223.28	heating
2.036	44.8613	21.3451	213.23	heating
2.038	44.6919	21.1613	213.36	heating
2.040	44.8394	20.7677	218.26	heating
2.042	45.0688	21.2678	215.81	heating
2.044	44.7928	21.5566	210.69	heating
2.046	45.1214	22.0684	209.03	heating
2.048	44.7931	20.6423	218.98	heating
2.050	45.1941	18.9857	237.64	heating
2.052	44.9891	18.0505	244.26	heating
2.054	45.2957	19.7061	232.02	heating
2.056	45.1531	21.9025	210.82	heating
2.058	45.1761	24.0352	191.69	heating
2.060	45.0829	22.8649	201.45	heating
2.062	45.3532	23.1266	201.53	heating
2.064	45.3957	21.2663	218.79	heating
2.066	45.2525	20.5317	224.15	heating
2.068	45.5270	22.6741	207.21	heating
2.070	45.0830	21.0867	217.58	heating
2.072	45.6521	20.1523	231.21	heating
2.074	45.3542	20.1494	228.54	heating
2.076	45.5869	21.0207	222.75	heating
2.078	45.6122	23.2286	202.96	heating
2.080	45.4594	23.5269	198.87	heating
2.082	45.5461	21.3325	219.55	heating
2.084	45.8738	22.3228	213.54	heating
2.086	45.7965	20.6994	227.56	heating
2.088	45.7744	20.6360	227.93	heating
2.090	45.9465	23.4317	204.14	heating
2.092	45.5964	22.9203	205.61	heating
2.094	46.0357	22.9343	209.46	heating
2.096	45.8636	23.0490	206.86	heating
2.098	46.0087	22.3223	214.77	heating
2.100	46.1122	24.2032	198.65	heating
2.102	45.8719	24.0601	197.77	heating
2.104	45.9194	22.0892	216.07	heating
2.106	46.2432	23.3137	207.91	heating
2.108	46.0931	22.1142	217.42	heating
2.110	46.2018	22.0415	219.06	heating
2.112	46.2005	23.1729	208.79	heating
2.114	46.0691	21.9502	218.69	heating
2.116	46.3200	20.9748	229.81	heating
2.118	46.3097	20.8394	230.94	heating
2.120	46.4132	21.3881	226.91	heating
2.122	46.4597	23.0777	212.01	heating
2.124	46.3080	22.8174	212.99	heating
2.126	46.3598	21.1258	228.80	heating
2.128	46.5633	21.2025	229.95	heating
2.130	46.6115	22.0422	222.77	heating
2.132	46.7399	24.1358	204.96	heating
2.134	46.5847	24.5724	199.59	heating
2.136	46.6133	23.1323	212.91	heating
2.138	46.7469	22.3346	221.35	heating
2.140	46.7410	21.7368	226.72	heating
2.142	46.9041	23.0565	216.23	heating
2.144	46.8202	23.7066	209.57	heating
2.146	46.7819	22.6983	218.37	heating
2.148	46.9140	22.1501	224.54	heating
2.150	46.8072	21.1185	232.92	heating
2.152	47.2414	22.7356	222.20	heating
2.154	47.0133	24.3256	205.71	heating
2.156	47.0101	24.3161	205.77	heating
2.158	47.1707	25.1574	199.60	heating
2.160	47.0811	24.2741	206.79	heating
2.162	47.1443	22.2421	225.79	heating
2.164	47.3558	24.5877	206.44	heating
2.166	47.2127	23.7890	212.39	heating
2.168	47.2884	23.1481	218.88	heating
2.170	47.5177	24.8824	205.24	heating
2.172	47.0308	22.3625	223.67	heating
2.174	47.9005	24.6625	210.70	heating
2.176	47.3621	26.2528	191.40	heating
2.178	47.4111	23.6763	215.21	heating
2.180	47.7082	24.4502	210.88	heating
2.182	47.4445	23.8368	214.05	heating
2.184	47.5742	20.1559	248.61	heating
2.186	47.9043	23.0610	225.26	heating
2.188	47.7289	22.0131	233.17	heating
2.190	47.7579	21.7933	235.42	heating
2.192	47.9939	23.3295	223.64	heating
2.194	47.3887	19.0501	256.95	heating
2.196	48.4727	21.6123	243.55	heating
2.198	47.7336	22.9281	224.92	heating
2.200	47.8721	19.6371	256.01	heating
2.202	48.2136	22.5289	232.89	heating
2.204	47.8546	23.7052	218.97	heating
2.206	48.0187	21.3271	242.02	heating
2.208	48.4378	25.7647	205.58	heating
2.210	48.0662	23.5169	222.59	heating
2.212	48.2362	22.7150	231.40	heating
2.214	48.4510	25.1433	211.33	heating
2.216	47.8570	22.0509	233.99	heating
2.218	48.8924	25.2510	214.36	heating
2.220	48.2154	26.7977	194.20	heating
2.222	48.3340	23.0758	229.02	heating
2.224	48.7047	25.3572	211.70	heating
2.226	48.2926	26.5541	197.11	heating
2.228	48.4434	24.0024	221.61	heating
2.230	48.8357	26.6067	201.55	heating
2.232	48.3756	22.8044	231.86	heating
2.234	48.6938	22.6171	236.44	heating
2.236	48.8200	26.8930	198.82	heating
2.238	48.3135	24.5385	215.57	heating
2.240	49.1052	26.2844	206.92	heating
2.242	48.6174	26.9628	196.35	heating
2.244	48.6706	22.7709	234.84	heating
2.246	48.9933	24.8003	219.36	heating
2.248	48.6507	26.6146	199.80	heating
2.250	48.7453	25.5891	209.96	heating
2.252	49.1084	28.2345	189.27	heating
2.254	48.7997	26.7551	199.88	heating
2.256	49.0125	25.1308	216.54	heating
2.258	49.0653	26.4346	205.20	heating
2.260	48.8600	23.9942	225.46	heating
2.262	49.1621	22.6117	240.74	heating
2.264	49.2249	23.9533	229.14	heating
2.266	49.0577	22.3325	242.32	heating
2.268	49.3198	22.7597	240.82	heating
2.270	49.2489	24.1612	227.47	heating
2.272	49.2004	22.9838	237.71	heating
2.274	49.3382	22.0262	247.64	heating
2.276	49.4270	23.1620	238.15	heating
2.278	49.3353	22.5073	243.25	heating
2.280	49.4142	22.7093	242.14	heating
2.282	49.5418	23.7159	234.17	heating
2.284	49.3163	22.0466	247.26	heating
2.286	49.7472	22.9407	243.06	heating
2.288	49.5427	23.8929	232.57	heating
2.290	49.5994	22.6799	244.08	heating
2.292	49.8543	24.6464	228.56	heating
2.294	49.5585	24.9463	223.16	heating
2.296	49.6681	22.3223	247.95	heating
2.298	50.0007	24.9760	226.90	heating
2.300	49.7198	25.9217	215.78	heating
2.302	49.8704	26.7217	209.89	heating
2.304	50.1386	29.4778	187.33	heating
2.306	49.6839	25.7983	216.57	heating
2.308	50.1365	24.5977	231.56	heating
2.310	50.1186	26.5537	213.67	heating
2.312	49.9730	24.4399	231.51	heating
2.314	50.3685	26.9600	212.25	heating
2.316	50.0045	29.1085	189.47	heating
2.318	50.1015	26.7291	211.92	heating
2.320	50.5529	28.6568	198.53	heating
2.322	50.1993	28.6729	195.18	heating
2.324	50.3869	28.1307	201.80	heating
2.326	50.5445	30.7634	179.36	heating
2.328	50.1547	27.7050	203.55	heating
2.330	50.5200	27.3822	209.79	heating
2.332	50.7232	29.9481	188.37	heating
2.334	50.3859	26.3835	217.63	heating
2.336	50.9279	28.1741	206.31	heating

2.338	50.3821	28.4607	198.76	heating
2.340	50.5523	24.5130	236.10	heating
2.342	51.1366	26.7300	221.30	heating
2.344	50.5918	26.1467	221.65	heating
2.346	50.8931	24.8331	236.29	heating
2.348	51.0019	27.2319	215.53	heating
2.350	50.6516	23.9403	242.19	heating
2.352	51.0295	23.8435	246.50	heating
2.354	51.1970	26.9796	219.58	heating
2.356	50.8542	23.5836	247.27	heating
2.358	51.3044	24.3071	244.79	heating
2.360	50.9793	25.0633	234.98	heating
2.362	51.0348	21.7461	265.57	heating
2.364	51.6168	24.0929	249.56	heating
2.366	51.1173	24.2823	243.32	heating
2.368	51.3317	22.2122	264.03	heating
2.370	51.5773	25.3415	237.88	heating
2.372	51.1050	23.8920	246.74	heating
2.374	51.4823	24.1174	248.12	heating
2.376	51.6003	27.5203	218.34	heating
2.378	51.2977	24.3401	244.43	heating
2.380	51.6462	23.8289	252.22	heating
2.382	51.4697	25.3522	236.81	heating
2.384	51.4399	24.4918	244.34	heating
2.386	51.8398	26.8626	226.47	heating
2.388	51.5897	26.9601	223.32	heating
2.390	51.6857	25.1937	240.21	heating
2.392	51.9301	27.2170	224.08	heating
2.394	51.5942	27.0448	222.59	heating
2.396	51.8637	27.8804	217.46	heating
2.398	51.9680	31.1798	188.49	heating
2.400	51.8163	29.9789	198.00	heating
2.402	51.9728	28.8563	209.60	heating
2.404	51.9741	29.5833	203.02	heating
2.406	52.0305	29.6115	203.28	heating
2.408	52.1111	30.0957	199.62	heating
2.410	52.1244	28.7704	211.75	heating
2.412	52.0693	27.0811	226.57	heating
2.414	52.2372	26.3942	234.32	heating
2.416	52.0640	26.7442	229.58	heating
2.418	52.2535	28.6095	214.38	heating
2.420	52.1739	29.8966	201.99	heating
2.422	52.3826	30.4282	199.06	heating
2.424	52.1698	27.7241	221.65	heating
2.426	52.4199	26.9993	230.49	heating
2.428	52.6071	27.9516	223.55	heating
2.430	52.4042	27.6850	224.13	heating
2.432	52.7203	28.4902	219.70	heating
2.434	52.4703	28.1703	220.33	heating
2.436	52.6505	25.7830	243.61	heating
2.438	52.7663	27.5813	228.36	heating
2.440	52.6225	28.6355	217.49	heating
2.442	52.6108	27.5506	227.22	heating
2.444	52.9462	28.9379	217.69	heating
2.446	52.5067	25.4627	245.21	heating
2.448	53.0864	26.1385	244.34	heating
2.450	53.0247	29.8293	210.32	heating
2.452	52.6991	27.2956	230.34	heating
2.454	53.1987	27.5841	232.25	heating
2.456	52.9799	28.0443	226.09	heating
2.458	53.1005	26.0934	244.88	heating
2.460	53.3922	29.7206	214.63	heating
2.462	52.9016	28.5683	220.63	heating
2.464	53.1370	26.2928	243.40	heating
2.466	53.4551	29.1728	220.17	heating
2.468	52.8344	26.3235	240.38	heating
2.470	53.7367	29.9465	215.71	heating
2.472	53.4216	35.8062	159.72	heating
2.474	53.0623	31.8058	192.74	heating
2.476	53.7121	32.8810	188.88	heating
2.478	53.3297	32.7874	186.26	heating
2.480	53.3833	28.4151	226.39	heating
2.482	53.9727	33.2933	187.50	heating
2.484	53.1020	31.2413	198.21	heating
2.486	53.6183	28.4474	228.23	heating
2.488	53.9324	32.1110	197.86	heating
2.490	53.3466	27.4870	234.47	heating
2.492	54.2015	27.2959	243.96	heating
2.494	53.8374	31.4629	202.87	heating
2.496	53.4973	26.9811	240.43	heating
2.498	54.1929	28.8224	230.04	heating
2.500	53.8794	29.9481	216.99	heating
2.502	53.9516	26.4707	249.17	heating
2.504	54.5075	31.3284	210.17	heating
2.506	53.6499	28.9267	224.17	heating
2.508	54.0775	26.0267	254.34	heating
2.510	54.4633	28.6653	233.91	heating
2.512	53.9550	24.8851	263.58	heating
2.514	54.7687	26.7221	254.30	heating
2.516	54.1658	29.2506	225.91	heating
2.518	54.1691	25.2413	262.29	heating
2.520	54.5154	25.5708	262.45	heating
2.522	54.3100	25.2523	263.47	heating
2.524	54.5187	22.9790	285.97	heating
2.526	54.8313	26.9812	252.52	heating
2.528	54.2760	25.7085	259.03	heating
2.530	54.5586	25.1835	266.35	heating
2.532	54.6918	25.6649	263.19	heating
2.534	54.4326	22.2965	291.38	heating
2.536	55.0273	23.1130	289.37	heating
2.538	54.5119	23.3222	282.80	heating
2.540	54.7478	22.5683	291.78	heating
2.542	54.7741	24.8838	271.02	heating
2.544	54.7157	26.4847	255.97	heating
2.546	55.0991	27.8345	247.21	heating
2.548	55.0299	29.1836	234.35	heating
2.550	55.0003	27.0601	253.34	heating
2.552	55.0152	27.4787	249.68	heating
2.554	55.1122	27.1776	253.29	heating
2.556	55.0374	26.1924	261.54	heating
2.558	55.3320	27.5661	251.76	heating
2.560	55.0224	27.2352	251.95	heating
2.562	55.3031	27.7524	249.81	heating
2.564	55.0343	27.7575	247.32	heating
2.566	55.2046	28.1778	245.06	heating
2.568	55.4919	29.1783	238.59	heating
2.570	55.2059	27.3630	252.46	heating
2.572	55.4654	26.0743	266.49	heating
2.574	55.3054	27.1005	255.74	heating
2.576	55.3703	26.3976	262.70	heating
2.578	55.5040	26.3993	263.90	heating
2.580	55.5900	25.7914	270.19	heating
2.582	55.4903	22.5265	298.89	heating
2.584	55.8997	24.9102	280.99	heating
2.586	55.5045	25.3295	273.60	heating
2.588	55.5732	25.0634	276.64	heating
2.590	55.9946	27.6717	256.81	heating
2.592	55.5921	25.5050	272.80	heating
2.594	55.9958	23.4709	294.91	heating
2.596	55.7784	24.3571	284.90	heating
2.598	55.7579	24.5090	283.34	heating
2.600	56.0181	26.5096	267.56	heating
2.602	55.9844	28.0261	253.50	heating
2.604	56.0537	25.6445	275.72	heating
2.606	56.2115	26.3288	270.95	heating
2.608	56.0334	26.0621	271.75	heating
2.610	55.7822	23.4576	293.09	heating
2.612	56.3371	24.7402	286.49	heating
2.614	56.0809	24.6708	284.80	heating
2.616	56.3750	25.4678	280.24	heating
2.618	56.3865	29.5039	243.75	heating
2.620	56.2171	31.2292	226.57	heating
2.622	56.4252	31.7825	223.44	heating
2.624	56.6176	33.2956	211.46	heating
2.626	56.4332	28.5763	252.58	heating
2.628	56.6695	26.8286	270.57	heating
2.630	56.6982	29.4879	246.72	heating
2.632	56.2343	28.6614	250.01	heating
2.634	56.7962	30.1486	241.62	heating
2.636	56.7570	31.9756	224.70	heating
2.638	56.7323	28.8917	252.44	heating
2.640	57.0280	29.6205	248.51	heating
2.642	56.7764	31.0727	233.06	heating
2.644	56.6829	28.1094	259.08	heating
2.646	57.1154	29.5644	249.81	heating
2.648	56.9630	29.5594	248.47	heating
2.650	56.9983	26.8216	273.62	heating
2.652	57.3050	29.9995	247.58	heating
2.654	56.6885	28.5697	254.96	heating
2.656	56.9735	25.6821	283.72	heating
2.658	57.5525	27.9679	268.25	heating
2.660	57.0508	25.6702	284.53	heating
2.662	57.4340	26.5326	280.19	heating
2.664	57.3568	31.7305	232.36	heating
2.666	56.9409	28.7287	255.80	heating
2.668	57.3888	26.5509	279.61	heating
2.670	57.5117	27.1486	275.31	heating
2.672	57.2245	22.4162	315.61	heating
2.674	57.8545	25.7345	291.24	heating
2.676	57.2894	27.7981	267.40	heating
2.678	57.1355	23.3998	305.89	heating
2.680	58.2321	27.1177	282.12	heating
2.682	57.4291	26.4775	280.64	heating
2.684	57.8303	23.0879	315.01	heating
2.686	58.0867	27.7988	274.62	heating
2.688	57.3766	23.9973	302.66	heating

2.690	57.8268	20.8082	335.65	heating
2.692	58.2444	26.0630	291.79	heating
2.694	57.6868	23.2906	311.88	heating
2.696	58.5073	26.6825	288.56	heating
2.698	57.9647	31.0670	243.89	heating
2.700	57.5524	25.3907	291.61	heating
2.702	58.5717	27.6790	280.11	heating
2.704	58.1188	29.9244	255.64	heating
2.706	58.1162	25.1234	299.15	heating
2.708	58.7350	32.0235	242.20	heating
2.710	57.8039	31.0391	242.68	heating
2.712	57.9325	26.2074	287.66	heating
2.714	58.9034	32.9398	235.42	heating
2.716	57.8324	30.1945	250.60	heating
2.718	58.9993	30.1030	262.01	heating
2.720	58.5207	35.4961	208.77	heating
2.722	58.0160	30.6005	248.58	heating
2.724	58.7659	30.7208	254.29	heating
2.726	58.8426	33.9013	226.15	heating
2.728	58.2754	26.8029	285.37	heating
2.730	59.2720	31.3447	253.22	heating
2.732	58.4183	31.8927	240.51	heating
2.734	58.3789	26.0861	292.80	heating
2.736	59.2754	30.1446	264.13	heating
2.738	58.4981	29.6848	261.25	heating
2.740	59.3431	26.8983	294.18	heating
2.742	59.0541	31.3116	251.55	heating
2.744	58.4110	26.3103	291.06	heating
2.746	58.8648	23.2135	323.26	heating
2.748	59.5277	28.3132	283.03	heating
2.750	58.4482	22.8629	322.66	heating
2.752	59.6097	25.0936	312.96	heating
2.754	58.9880	28.0300	280.70	heating
2.756	58.6074	22.3513	328.74	heating
2.758	59.4221	23.9200	321.90	heating
2.760	59.0387	25.2456	306.41	heating
2.762	59.4410	22.0085	339.41	heating
2.764	59.5574	28.8502	278.43	heating
2.766	58.8845	27.5748	283.89	heating
2.768	59.0907	23.6193	321.62	heating
2.770	59.9029	29.9430	271.65	heating
2.772	58.9851	28.4713	276.67	heating
2.774	59.7076	26.1334	304.42	heating
2.776	59.4776	26.8341	295.98	heating
2.778	59.0993	23.6467	321.45	heating
2.780	59.6468	26.5410	300.17	heating
2.782	59.8679	32.9339	244.21	heating
2.784	59.4078	30.0951	265.78	heating
2.786	60.1149	33.2203	243.86	heating
2.788	59.5202	32.8900	241.46	heating
2.790	59.5492	26.6184	298.59	heating
2.792	60.2985	29.3077	281.00	heating
2.794	59.6575	30.0138	268.78	heating
2.796	60.2255	30.8646	266.22	heating
2.798	60.1300	34.6688	230.86	heating
2.800	59.5636	30.1209	266.96	heating
2.802	60.1091	28.9104	282.88	heating
2.804	60.4544	32.5770	252.77	heating
2.806	59.8398	28.1287	287.53	heating
2.808	60.5402	28.5075	290.44	heating
2.810	60.1160	29.9040	273.94	heating
2.812	60.0930	26.9776	300.26	heating
2.814	60.5342	30.0517	276.39	heating
2.816	60.3466	32.5432	252.10	heating
2.818	60.4682	28.5578	289.34	heating
2.820	60.7827	30.5679	273.96	heating
2.822	60.2081	26.4024	306.52	heating
2.824	60.3552	22.6957	341.46	heating
2.826	61.1411	28.0664	299.89	heating
2.828	60.2651	27.4823	297.25	heating
2.830	61.1205	28.1334	299.10	heating
2.832	60.6427	29.3903	283.37	heating
2.834	60.6153	26.0572	313.34	heating
2.836	60.8932	26.5956	310.98	heating
2.838	60.9974	30.2554	278.74	heating
2.840	60.8070	27.0302	306.26	heating
2.842	61.3025	30.0173	283.67	heating
2.844	60.9717	31.0157	271.62	heating
2.846	60.7474	28.4783	292.59	heating
2.848	61.6098	31.2630	275.16	heating
2.850	60.7362	29.1567	286.34	heating
2.852	61.6122	28.5327	299.94	heating
2.854	61.2867	31.8233	267.15	heating
2.856	61.1509	31.1624	271.91	heating
2.858	61.4048	32.9464	258.04	heating
2.860	61.6004	38.6151	208.41	heating
2.862	61.2637	34.9043	239.00	heating
2.864	61.9127	36.1803	233.32	heating
2.866	61.6348	37.4514	219.27	heating
2.868	61.2533	34.4447	243.08	heating
2.870	62.0323	36.3540	232.83	heating
2.872	61.4533	35.6969	233.54	heating
2.874	61.9795	32.6572	265.87	heating
2.876	62.0114	36.4396	231.86	heating
2.878	61.7276	36.6891	227.03	heating
2.880	61.6605	34.2213	248.80	heating
2.882	62.1019	36.0424	236.29	heating
2.884	61.8427	33.2011	259.70	heating
2.886	62.3872	33.9924	257.46	heating
2.888	62.1722	36.7615	230.40	heating
2.890	61.7044	33.2896	257.64	heating
2.892	62.2711	32.5442	269.54	heating
2.894	62.1418	33.0879	263.44	heating
2.896	61.9965	28.3511	305.07	heating
2.898	62.6045	30.8553	287.87	heating
2.900	62.1191	32.6650	267.06	heating
2.902	62.0245	30.3469	287.23	heating
2.904	62.6369	32.9591	269.09	heating
2.906	62.3907	31.0239	284.41	heating
2.908	62.6551	28.6328	308.49	heating
2.910	62.7106	33.0131	269.27	heating
2.912	62.2100	31.3319	279.98	heating
2.914	62.5509	30.0320	294.85	heating
2.916	62.8087	33.7804	263.20	heating
2.918	62.3263	31.5407	279.14	heating
2.920	63.0702	31.3904	287.24	heating
2.922	62.5887	31.3519	283.23	heating
2.924	62.3395	26.9309	321.06	heating
2.926	62.9251	28.5561	311.63	heating
2.928	62.8716	31.6393	283.19	heating
2.930	62.8054	31.7269	281.79	heating
2.932	63.1379	38.7141	221.45	heating
2.934	62.6735	38.8951	215.60	heating
2.936	62.8264	34.8721	253.47	heating
2.938	63.0731	33.6096	267.15	heating
2.940	62.7669	29.8016	298.90	heating
2.942	63.4580	31.5824	289.02	heating
2.944	63.0718	36.2588	243.12	heating
2.946	62.7718	33.9736	261.12	heating
2.948	63.2040	34.7212	258.26	heating
2.950	63.5696	38.6101	226.31	heating
2.952	62.9808	35.4513	249.61	heating
2.954	63.5559	36.6611	243.86	heating
2.956	63.1603	36.9288	237.85	heating
2.958	63.0859	33.5474	267.83	heating
2.960	63.4593	35.4613	253.86	heating
2.962	63.2631	38.9706	220.26	heating
2.964	63.5556	39.0901	221.83	heating
2.966	63.6397	41.0224	205.07	heating
2.968	63.1561	35.1682	253.77	heating
2.970	63.3313	28.1815	318.71	heating
2.972	63.9117	30.5117	302.84	heating
2.974	63.4996	31.7610	287.78	heating
2.976	63.7079	32.7351	280.84	heating
2.978	63.9125	36.7973	245.86	heating
2.980	63.4071	33.4136	271.96	heating
2.982	63.8741	31.9808	289.18	heating
2.984	64.1056	35.7487	257.12	heating
2.986	63.6983	31.6674	290.43	heating
2.988	64.4100	31.9261	294.54	heating
2.990	63.9234	32.2470	287.21	heating
2.992	63.7217	28.5237	319.15	heating
2.994	64.5134	33.7442	278.99	heating
2.996	63.9316	34.6202	265.77	heating
2.998	64.0652	31.2780	297.29	heating
3.000	64.5009	35.3715	264.12	heating
3.002	63.6342	31.2303	293.81	heating
3.004	64.2684	29.1067	318.82	heating
3.006	64.4641	34.9896	267.25	heating
3.008	63.9589	33.6474	274.84	heating
3.010	64.7219	34.9500	269.95	heating
3.012	64.3784	38.1241	238.05	heating
3.014	64.0699	32.1103	289.78	heating
3.016	64.7374	33.0953	286.90	heating
3.018	64.4681	33.6300	279.61	heating
3.020	64.3515	29.7587	313.66	heating
3.022	65.1317	36.6102	258.61	heating
3.024	64.1114	36.6803	248.72	heating
3.026	64.5333	33.4906	281.47	heating
3.028	64.8131	37.1961	250.41	heating
3.030	64.4744	35.9830	258.34	heating
3.032	65.0090	36.2359	260.89	heating
3.034	64.8017	40.1502	223.52	heating
3.036	64.3730	36.6639	251.24	heating
3.038	65.0859	38.0494	245.14	heating
3.040	64.8016	37.6779	245.93	heating

3.042	64.7345	31.8278	298.37	heating
3.044	65.3649	33.6894	287.21	heating
3.046	64.7495	33.9036	279.68	heating
3.048	64.9585	32.5641	293.73	heating
3.050	65.2090	35.5177	269.22	heating
3.052	65.3071	35.4789	270.46	heating
3.054	65.4514	36.2493	264.78	heating
3.056	65.2481	38.7448	240.31	heating
3.058	64.9552	35.4316	267.69	heating
3.060	65.4648	35.1412	274.95	heating
3.062	65.1569	34.4234	278.66	heating
3.064	65.2483	31.8446	302.88	heating
3.066	65.6186	33.6224	290.11	heating
3.068	65.3821	37.0375	257.00	heating
3.070	65.2720	37.2658	253.94	heating
3.072	65.3057	35.3257	271.83	heating
3.074	65.7442	33.2242	294.86	heating
3.076	65.5374	33.7373	288.34	heating
3.078	65.7685	37.2882	258.24	heating
3.080	65.3861	38.9362	239.83	heating
3.082	65.7639	38.7779	244.69	heating
3.084	65.4649	36.0154	267.02	heating
3.086	65.9839	34.9614	281.29	heating
3.088	65.5912	29.7620	324.87	heating
3.090	65.9889	32.0763	307.49	heating
3.092	65.7196	33.3306	293.68	heating
3.094	65.4519	29.3979	326.91	heating
3.096	66.5007	31.8951	313.77	heating
3.098	65.5664	33.5367	290.42	heating
3.100	66.1394	32.8492	301.85	heating
3.102	66.0084	36.3899	268.55	heating
3.104	65.7983	33.2809	294.84	heating
3.106	65.8382	28.9676	334.31	heating
3.108	66.5976	34.6196	289.95	heating
3.110	65.8992	33.4098	294.59	heating
3.112	66.5352	35.9837	277.01	heating
3.114	66.1174	35.9462	273.57	heating
3.116	65.8554	29.2182	332.19	heating
3.118	66.8571	31.3268	322.16	heating
3.120	66.1641	33.3484	297.54	heating
3.122	66.5067	30.1491	329.66	heating
3.124	66.7224	34.7598	289.81	heating
3.126	66.2678	33.6851	295.43	heating
3.128	66.3357	30.5996	324.02	heating
3.130	67.1013	36.6613	276.00	heating
3.132	66.3212	33.1464	300.80	heating
3.134	67.0484	31.8558	319.10	heating
3.136	66.6934	32.9091	306.33	heating
3.138	66.4039	28.5358	343.36	
3.140	67.0555	31.9237	318.55	
3.142	66.6800	37.4444	265.08	
3.144	66.6525	34.3484	292.91	
3.146	66.9940	35.8836	282.08	
3.148	66.5561	34.1940	293.43	
3.150	66.4713	31.1695	320.09	
3.152	67.2393	37.1917	272.45	
3.154	66.6100	37.5903	263.13	
3.156	67.0496	38.0538	262.91	
3.158	66.8873	41.6441	228.88	heating
3.160	66.6060	38.2084	257.48	heating
3.162	66.9509	35.5889	284.36	heating
3.164	66.9266	35.5064	284.89	heating
3.166	66.7449	30.8030	325.89	heating
3.168	67.2560	31.7852	321.62	heating
3.170	66.7294	32.2718	312.43	heating
3.172	66.8810	31.8871	317.29	heating
3.174	67.3166	35.9933	284.01	
3.176	67.0480	35.6349	284.83	
3.178	67.0144	31.4630	322.35	
3.180	67.1508	32.5593	313.65	
3.182	66.7364	31.0489	323.58	
3.184	66.9248	30.5336	329.96	
3.186	66.8972	30.9420	326.01	
3.188	66.8006	29.7430	336.01	
3.190	67.2528	31.8153	321.32	
3.192	66.7184	32.2581	312.46	
3.194	66.8667	29.9402	334.82	
3.196	66.9266	28.6207	347.33	
3.198	67.2373	28.9412	347.24	
3.200	66.8705	29.1459	342.05	
3.202	66.9804	30.2255	333.26	
3.204	66.9194	32.9979	307.57	
3.206	66.7799	33.9538	297.64	
3.208	66.8045	33.2367	304.36	
3.210	67.1404	33.0926	308.72	
3.212	66.7436	28.0528	350.82	
3.214	67.1378	29.6197	340.18	
3.216	66.9206	33.7873	300.42	cooling
3.218	66.6741	33.1410	304.05	cooling
3.220	67.1951	32.7618	312.21	cooling
3.222	66.5253	31.3151	319.26	cooling
3.224	66.9038	31.0085	325.47	cooling
3.226	66.7107	36.0639	277.88	cooling
3.228	66.5176	37.2307	265.55	cooling
3.230	66.3797	36.7405	268.74	
3.232	67.1952	40.9162	238.28	
3.234	66.1243	32.9199	301.07	
3.236	67.0978	32.3160	315.37	
3.238	66.6758	35.8491	279.51	
3.240	66.2604	31.6468	313.85	
3.242	67.0918	31.1532	325.86	
3.244	66.2681	29.6749	331.80	
3.246	66.8307	26.5795	364.96	
3.248	67.0274	35.5318	285.58	
3.250	66.3561	35.5895	278.97	
3.252	66.2235	30.0119	328.34	
3.254	67.4811	35.7913	287.34	
3.256	66.0302	29.9528	327.12	
3.258	67.2774	30.9708	329.20	
3.260	66.6586	34.1927	294.37	
3.262	66.1437	26.7453	357.23	
3.264	67.1014	27.2340	361.48	
3.266	66.5129	31.4609	317.82	cooling
3.268	66.5784	26.1008	367.02	cooling
3.270	67.1020	33.7484	302.42	cooling
3.272	66.3114	34.9195	284.64	cooling
3.274	65.9848	30.0049	326.23	cooling
3.276	67.3885	37.8506	267.82	cooling
3.278	65.8329	35.5385	274.68	cooling
3.280	66.9133	34.6558	292.48	cooling
3.282	66.3837	37.7823	259.33	
3.284	65.8689	31.2930	313.50	
3.286	66.5266	30.9041	322.99	
3.288	66.4479	35.7813	278.06	
3.290	66.2103	30.8908	320.25	
3.292	66.8114	34.7570	290.64	
3.294	66.1752	35.6110	277.13	
3.296	65.9612	31.3620	313.72	
3.298	66.8698	34.6882	291.80	
3.300	66.1083	34.2167	289.17	
3.302	66.6189	32.0368	313.56	
3.304	66.5946	36.9789	268.53	
3.306	66.1343	36.1102	272.23	
3.308	66.0936	31.4661	313.97	
3.310	66.7340	33.2074	303.99	
3.312	66.2411	30.0946	327.75	
3.314	66.8071	29.9166	334.49	
3.316	66.4090	33.1092	301.93	
3.318	65.9918	29.4360	331.46	
3.320	66.4135	27.5685	352.21	
3.322	66.6226	30.9678	323.29	
3.324	66.1680	29.6646	330.98	
3.326	66.6086	32.4356	309.85	cooling
3.328	66.1580	33.7214	294.11	cooling
3.330	65.8683	28.3871	339.85	
3.332	66.3951	28.1019	347.21	cooling
3.334	66.2564	29.2374	335.66	cooling
3.336	66.2167	26.3559	361.42	cooling
3.338	66.3356	29.6984	332.20	cooling
3.340	65.7694	27.6690	345.46	cooling
3.342	65.8253	23.7997	381.05	cooling
3.344	66.5930	30.9967	322.76	cooling
3.346	65.4616	30.6030	316.07	cooling
3.348	66.1485	31.2056	316.83	cooling
3.350	65.9476	37.3466	259.33	cooling
3.352	65.3053	35.4704	270.52	cooling
3.354	65.8875	35.6076	274.55	cooling
3.356	65.8994	39.2548	241.59	cooling
3.358	65.2766	31.0920	309.96	cooling
3.360	65.9297	31.7056	310.32	cooling
3.362	65.4471	33.9960	285.17	cooling
3.364	65.0261	30.5949	312.19	cooling
3.366	66.0519	36.7337	265.83	cooling
3.368	65.1108	38.3959	242.23	
3.370	65.6714	34.5534	282.15	
3.372	65.7339	38.3757	248.06	
3.374	65.1427	35.1873	271.61	
3.376	65.4022	31.5058	307.34	
3.378	66.0381	36.8646	264.52	
3.380	65.1100	32.3873	296.70	
3.382	65.8908	32.7030	300.92	
3.384	65.6144	35.6338	271.84	
3.386	65.0805	29.9470	318.56	
3.388	65.8958	31.4562	312.27	
3.390	65.3961	33.8388	286.13	
3.392	65.5635	29.5847	326.22	

3.394	65.8093	32.8901	298.48	
3.396	65.2001	32.7424	294.30	
3.398	65.3477	31.5501	306.45	
3.400	66.0403	37.7859	256.19	
3.402	65.1182	33.9915	282.23	
3.404	65.6884	31.1053	313.57	
3.406	65.5583	31.1476	312.01	
3.408	65.2146	27.4181	342.71	
3.410	65.7052	29.7870	325.68	
3.412	65.4183	33.2049	292.08	
3.414	65.5767	29.6503	325.75	
3.416	65.8661	31.9232	307.77	
3.418	65.4452	32.2513	300.97	
3.420	65.2206	28.7177	330.98	
3.422	65.9485	32.5605	302.73	
3.424	65.2452	32.3441	298.32	
3.426	65.7790	34.0658	287.55	
3.428	65.5491	37.0023	258.84	
3.430	65.2076	34.7468	276.19	
3.432	65.5183	32.9116	295.65	
3.434	65.5092	33.0792	294.05	
3.436	65.6901	29.2495	330.41	
3.438	65.8149	31.2119	313.75	
3.440	65.6137	33.3878	292.20	
3.442	65.1980	30.8560	311.38	
3.444	65.9963	33.4751	294.87	
3.446	65.2523	32.3811	298.05	
3.448	65.8726	31.5749	310.98	
3.450	65.5905	33.9954	286.48	
3.452	65.2144	31.6083	304.71	
3.454	65.5213	31.0909	312.19	
3.456	65.6243	34.9114	278.48	
3.458	65.7132	33.6483	290.74	
3.460	65.8106	34.0112	288.33	
3.462	65.7098	33.3263	293.63	
3.464	65.3665	30.5341	315.83	
3.466	65.7544	31.4997	310.59	
3.468	65.5452	32.9003	296.00	
3.470	65.6171	34.8072	279.36	
3.472	65.7326	38.0407	251.09	
3.474	65.6275	40.0365	232.04	
3.476	65.4402	36.5356	262.08	
3.478	65.6261	34.6639	280.74	
3.480	65.6970	32.1793	303.91	
3.482	65.6330	28.5021	336.67	
3.484	65.8450	29.9990	325.02	
3.486	65.5306	32.4962	299.53	
3.488	65.4934	33.1990	292.82	
3.490	65.8121	35.7756	272.35	
3.492	65.2077	32.6830	294.91	
3.494	65.7175	28.3300	339.00	
3.496	65.7358	30.0495	323.57	
3.498	65.4068	30.3826	317.57	
3.500	65.5924	32.0755	303.90	
3.502	65.8882	36.5888	265.66	
3.504	65.3986	33.0604	293.21	
3.506	65.8837	33.6528	292.24	
3.508	65.7200	32.8315	298.20	
3.510	65.4073	29.0653	329.52	
3.512	65.9260	29.8530	327.08	
3.514	65.1355	28.2691	334.27	
3.516	66.0438	28.9756	336.10	
3.518	65.8936	35.8594	272.32	
3.520	65.4831	36.7958	260.11	
3.522	65.4038	33.6360	288.04	
3.524	65.9084	33.4813	294.02	
3.526	65.3181	26.1864	354.81	
3.528	65.9625	27.3176	350.40	
3.530	65.8024	31.2694	313.12	
3.532	65.1755	28.6009	331.63	
3.534	65.9041	30.1051	324.59	
3.536	65.2151	29.9306	319.93	
3.538	65.9847	27.6763	347.35	
3.540	65.8825	30.5849	320.05	
3.542	65.3522	29.4920	325.15	
3.544	65.4235	27.4288	344.50	
3.546	65.8819	33.0349	297.83	cooling
3.548	65.2477	32.1599	300.01	cooling
3.550	65.8586	34.5701	283.70	cooling
3.552	65.5727	36.8623	260.32	
3.554	64.9926	30.9788	308.41	
3.556	65.7172	31.2101	312.88	
3.558	65.2705	31.1367	309.50	
3.560	65.5690	26.2548	356.47	
3.562	65.8818	29.3574	331.17	
3.564	65.2402	30.8953	311.41	cooling
3.566	65.2910	29.0736	328.39	cooling
3.568	65.6339	32.4502	300.88	cooling
3.570	65.1794	30.8230	311.51	cooling
3.572	65.3673	29.7371	323.06	cooling
3.574	65.4097	33.2257	291.82	cooling
3.576	64.8455	32.1216	296.71	cooling
3.578	65.1479	33.6536	285.56	cooling
3.580	65.1058	35.6178	267.37	
3.582	64.9921	32.0259	298.91	
3.584	65.6433	34.8300	279.39	
3.586	65.0179	35.9768	263.32	
3.588	64.9806	32.6447	293.19	
3.590	65.2845	33.6541	286.80	
3.592	64.9210	34.0506	279.91	
3.594	65.1839	35.8873	265.64	
3.596	65.3208	39.8448	230.99	
3.598	64.9428	38.1395	243.03	
3.600	65.0875	34.8777	273.92	
3.602	64.9929	33.4288	286.20	
3.604	65.3897	32.4972	298.24	
3.606	65.2957	32.0021	301.88	
3.608	65.3774	33.2361	291.43	
3.610	65.0795	32.5832	294.65	
3.612	65.2791	32.8991	293.59	
3.614	65.0973	32.9203	291.75	
3.616	65.0899	32.1174	298.97	
3.618	65.3469	32.1666	300.85	
3.620	65.2899	33.4739	288.48	
3.622	65.0346	33.3938	286.89	
3.624	65.0931	35.0364	272.53	
3.626	65.3690	35.9411	266.83	
3.628	64.9299	31.5138	302.99	
3.630	65.4693	30.3441	318.49	
3.632	65.0868	30.1406	316.86	
3.634	65.1573	30.5720	313.59	
3.636	65.0395	32.2543	297.27	
3.638	65.0015	31.6558	302.35	
3.640	65.4577	27.6611	342.71	
3.642	65.3224	28.3386	335.34	
3.644	65.0617	26.6263	348.50	
3.646	65.0294	28.1273	334.60	
3.648	65.4899	32.0972	302.78	
3.650	64.6326	28.8779	324.19	
3.652	65.7617	32.0440	305.72	
3.654	64.9243	31.6057	302.10	
3.656	65.1115	28.8877	328.45	
3.658	65.1216	29.0691	326.89	
3.660	64.8608	27.3893	339.76	
3.662	65.5823	26.0666	358.29	
3.664	65.3619	33.5568	288.38	
3.666	65.1369	32.9987	291.40	
3.668	65.0351	35.1021	271.41	
3.670	65.5667	39.5777	235.65	
3.672	64.5875	34.1895	275.62	
3.674	65.8405	36.5540	265.54	
3.676	64.8730	36.6733	255.69	
3.678	65.0778	33.5680	285.70	
3.680	65.1460	34.2216	280.40	
3.682	64.8916	34.3155	277.24	
3.684	65.4682	33.1607	292.94	
3.686	65.3474	38.0871	247.17	
3.688	65.1026	36.2063	262.01	
3.690	64.9479	34.4654	276.39	
3.692	65.4309	35.9957	266.89	
3.694	64.6643	32.2948	293.50	
3.696	65.5812	35.7846	270.17	
3.698	65.0768	37.9880	245.62	heating
3.700	65.1241	36.6814	257.89	heating
3.702	65.2810	36.5486	260.52	heating
3.704	64.8642	33.2932	286.26	heating
3.706	65.8696	30.8962	317.11	heating
3.708	65.4145	32.3352	299.94	heating
3.710	65.4729	33.5139	289.78	heating
3.712	65.2607	34.1052	282.49	heating
3.714	65.6443	35.6976	271.53	heating
3.716	65.2917	32.9693	293.07	heating
3.718	65.5931	31.9742	304.83	heating
3.720	65.6359	30.7236	316.56	heating
3.722	65.4563	30.6049	316.00	heating
3.724	65.5409	30.1513	320.88	heating
3.726	65.3683	29.3273	326.79	
3.728	66.1122	30.4968	322.93	
3.730	65.5948	31.2967	310.99	
3.732	65.9059	32.6952	301.13	
3.734	65.5261	31.3975	309.45	
3.736	65.7267	29.9898	324.03	
3.738	65.6194	29.2236	330.01	
3.740	65.6645	31.1442	313.00	
3.742	66.0378	33.7426	292.83	
3.744	65.6471	38.0468	250.26	

3.746	65.4775	37.3981	254.60	
3.748	65.4245	35.1943	274.10	
3.750	65.9975	35.6770	274.92	
3.752	65.5859	35.8889	269.27	
3.754	65.8485	38.4468	248.46	
3.756	65.5440	36.9172	259.56	heating
3.758	65.6285	33.9143	287.56	heating
3.760	65.8722	34.4487	284.92	heating
3.762	65.7405	36.0718	269.01	heating
3.764	66.2167	36.9600	265.27	heating
3.766	65.8916	38.9257	244.50	heating
3.768	65.7322	37.3667	257.19	heating
3.770	65.9536	36.9438	263.04	heating
3.772	66.2588	37.0856	264.52	heating
3.774	65.9833	33.7070	292.65	heating
3.776	66.4043	35.8096	277.41	heating
3.778	65.9481	33.5970	293.33	heating
3.780	66.1318	30.9443	319.05	heating
3.782	66.4132	33.4826	298.59	heating
3.784	66.0925	32.8215	301.67	heating
3.786	66.7760	31.9057	316.17	heating
3.788	66.6080	34.5889	290.32	heating
3.790	66.2375	32.1982	308.64	heating
3.792	66.5372	31.6249	316.55	heating
3.794	66.7823	32.2500	313.11	heating
3.796	66.4158	27.7716	350.39	heating
3.798	66.9211	30.1560	333.35	
3.800	66.7194	33.8067	298.42	
3.802	66.4483	32.5340	307.51	
3.804	66.7788	34.3391	294.14	
3.806	66.4644	33.1132	302.40	
3.808	66.8502	28.6707	346.18	
3.810	66.9152	29.8840	335.77	
3.812	66.4885	30.5259	326.08	
3.814	66.6307	29.6162	335.62	
3.816	66.9459	35.1279	288.50	
3.818	66.6526	38.4205	255.98	
3.820	66.6261	37.8381	261.03	
3.822	66.8660	35.9303	280.50	
3.824	66.4258	29.8138	331.97	
3.826	66.7003	28.4404	346.91	
3.828	66.5590	30.7898	324.33	
3.830	67.0242	34.9363	290.95	
3.832	66.7648	38.4122	257.08	
3.834	66.5371	38.7662	251.80	
3.836	66.6128	37.1485	267.16	
3.838	66.7466	36.0910	277.96	
3.840	66.6804	33.6469	299.52	
3.842	66.6810	32.9970	305.42	
3.844	66.7690	34.2183	295.14	
3.846	66.5755	36.5100	272.61	
3.848	66.5478	37.0200	267.73	
3.850	66.5387	36.2310	274.80	
3.852	66.8703	35.2944	286.30	
3.854	66.6667	33.5998	299.82	
3.856	66.8118	33.4356	302.63	
3.858	66.5772	34.3680	292.05	
3.860	66.6872	33.3244	302.51	
3.862	66.5812	32.0177	313.39	
3.864	66.8049	33.2392	304.34	
3.866	66.7099	32.9885	305.76	
3.868	66.8242	35.5722	283.37	
3.870	66.5094	33.9131	295.56	
3.872	66.4287	31.2371	319.09	
3.874	67.0602	32.3786	314.46	
3.876	66.3768	30.7375	323.15	
3.878	67.0634	34.4561	295.66	
3.880	66.5121	37.6637	261.57	
3.882	66.5638	35.9056	277.98	
3.884	66.5213	33.2270	301.88	
3.886	66.6351	31.7507	316.30	
3.888	66.8953	30.7262	327.95	
3.890	66.6896	36.2052	276.41	
3.892	66.4785	38.0236	258.00	
3.894	66.3429	37.7407	259.34	
3.896	67.0710	39.4161	250.75	
3.898	66.1367	36.0054	273.21	
3.900	67.0689	36.2084	279.82	
3.902	66.5228	35.9885	276.86	heating
3.904	66.4786	32.1194	311.54	heating
3.906	66.5070	30.5946	325.62	
3.908	66.8280	34.4450	293.62	
3.910	66.9714	35.7817	282.80	
3.912	66.7200	39.8633	243.51	
3.914	66.5589	39.1763	248.28	heating
3.916	66.4443	35.3632	281.82	heating
3.918	67.0061	34.0684	298.65	heating
3.920	66.6087	32.9500	305.19	heating

3.922	67.1235	32.9002	310.31	heating
3.924	66.8606	34.3320	294.94	heating
3.926	66.7928	33.5601	301.33	heating
3.928	66.6756	29.5077	337.01	heating
3.930	67.2645	29.4797	342.60	
3.932	67.1699	29.7057	339.69	
3.934	66.9810	30.3225	332.39	
3.936	66.8756	30.1061	333.39	
3.938	66.8043	29.1480	341.43	
3.940	66.8975	29.6016	338.17	
3.942	66.9731	31.1838	324.51	
3.944	67.1192	32.8115	311.07	
3.946	66.9392	32.3699	313.45	
3.948	66.6911	30.0588	332.15	
3.950	66.8709	28.8296	344.93	
3.952	67.0856	28.4915	349.94	
3.954	67.1508	30.0298	336.58	
3.956	66.8663	30.6685	328.21	cooling
3.958	66.6663	31.3176	320.51	cooling
3.960	66.7919	31.4622	320.34	cooling
3.962	66.4345	30.7504	323.55	cooling
3.964	67.0416	34.1509	298.22	cooling
3.966	66.6843	36.8200	270.78	cooling
3.968	66.7505	36.4595	274.65	cooling
3.970	66.4311	33.9226	294.76	
3.972	66.5785	32.7230	306.97	
3.974	66.7973	32.9715	306.70	
3.976	66.8458	35.1249	287.62	
3.978	66.6803	35.3060	284.48	
3.980	66.4297	35.2874	282.37	
3.982	66.5387	34.3541	291.82	
3.984	66.3496	34.6685	287.26	
3.986	66.8954	38.2799	259.46	
3.988	66.5393	38.4097	255.06	
3.990	66.7831	36.7564	272.26	
3.992	66.5159	36.4363	272.74	
3.994	66.5218	36.0738	276.08	
3.996	66.7267	36.0891	277.80	
3.998	66.9111	38.1859	260.45	
4.000	66.4480	36.4426	272.06	
4.002	66.6402	35.7391	280.19	
4.004	66.6014	35.6700	280.46	
4.006	66.4437	33.0196	303.06	
4.008	66.7202	32.8632	306.99	
4.010	66.5408	32.7805	306.11	
4.012	66.8626	34.1340	296.76	
4.014	66.7608	38.8785	252.81	
4.016	66.4139	39.4032	244.91	
4.018	66.6169	37.4949	264.05	heating
4.020	66.9675	35.5291	285.06	heating
4.022	66.5794	31.8378	315.01	heating
4.024	66.9130	30.4324	330.78	heating
4.026	66.6638	30.5066	327.84	
4.028	66.7706	30.1059	332.44	
4.030	66.7908	31.7045	318.13	
4.032	67.0068	34.0925	298.44	
4.034	66.9004	31.1071	324.54	
4.036	67.0122	31.8445	318.87	
4.038	66.7323	32.9926	305.92	
4.040	66.6270	32.3673	310.64	
4.042	67.0599	32.7185	311.38	
4.044	66.7188	31.4117	320.13	
4.046	66.9158	29.5536	338.77	
4.048	66.5525	28.1574	348.13	
4.050	66.8527	29.1283	342.05	
4.052	66.6265	29.2148	339.22	
4.054	67.0655	29.6862	338.92	
4.056	66.8407	29.1307	341.92	
4.058	66.7826	29.7461	335.82	cooling
4.060	66.8007	32.2515	313.26	cooling
4.062	66.5743	33.1302	303.24	cooling
4.064	66.7124	31.6768	317.67	cooling
4.066	66.6969	31.4916	319.21	cooling
4.068	66.7761	29.0454	342.11	cooling
4.070	66.5185	27.3246	355.38	cooling
4.072	66.6132	29.9250	332.66	cooling
4.074	66.4074	31.8851	313.02	cooling
4.076	66.6342	32.5633	308.93	cooling
4.078	66.6228	33.3497	301.69	cooling
4.080	66.3689	33.6776	296.42	cooling
4.082	66.3647	34.4948	288.97	cooling
4.084	66.1380	34.2374	289.25	cooling
4.086	66.0986	32.9412	300.64	cooling
4.088	66.4032	34.8991	285.65	cooling
4.090	66.2423	35.4240	279.43	cooling
4.092	66.3565	34.6092	287.86	
4.094	66.2327	35.4375	279.22	
4.096	66.0517	33.2898	297.06	

4.098	66.3499	31.5789	315.27	
4.100	66.4563	31.2752	318.99	
4.102	66.1411	30.9135	319.41	
4.104	66.3222	33.7292	295.53	
4.106	66.0650	34.3100	287.93	
4.108	66.0134	32.3092	305.60	
4.110	66.1757	32.3102	307.06	
4.112	66.1000	31.9386	309.75	
4.114	66.4722	32.7030	306.19	
4.116	66.0755	35.1460	280.44	
4.118	66.1098	35.0056	282.03	
4.120	66.1248	34.4606	287.10	
4.122	66.5096	35.5694	280.54	
4.124	65.8715	32.6105	301.58	
4.126	66.3072	32.8024	303.79	
4.128	66.0973	33.6330	294.36	
4.130	65.9259	33.3527	295.35	
4.132	66.1595	35.8551	274.77	
4.134	66.2163	40.3143	234.86	
4.136	66.4661	38.8514	250.39	
4.138	66.1969	37.9417	256.19	
4.140	66.1128	34.8627	283.35	
4.142	65.9207	30.5497	320.71	
4.144	66.5977	32.9220	305.34	
4.146	65.9395	33.9133	290.39	
4.148	66.2102	34.8887	284.00	
4.150	66.0705	35.1597	280.27	
4.152	65.9705	33.4185	295.15	
4.154	65.8488	29.9311	325.67	
4.156	66.3935	32.7131	305.38	
4.158	66.3241	33.1736	300.58	
4.160	66.1799	33.7179	294.34	
4.162	66.2781	34.3492	289.50	
4.164	65.7337	29.9622	324.35	
4.166	66.5603	29.6507	334.66	
4.168	65.8501	30.5665	319.92	
4.170	66.2769	30.4179	325.14	
4.172	66.0940	31.8151	310.81	
4.174	66.0059	30.8499	318.76	
4.176	65.7482	26.4399	356.41	
4.178	66.4554	29.6021	334.15	
4.180	66.3836	31.5304	316.02	
4.182	66.1601	31.1752	317.21	
4.184	66.2200	29.8861	329.44	
4.186	65.7810	25.9618	361.05	
4.188	66.2148	23.6910	385.57	cooling
4.190	66.0550	26.1718	361.63	cooling
4.192	66.0980	26.2001	361.76	cooling
4.194	66.0214	29.5357	330.82	cooling
4.196	65.9512	32.5772	302.61	cooling
4.198	65.4423	29.9389	321.91	cooling
4.200	66.4730	33.6313	297.78	cooling
4.202	65.8658	34.1533	287.54	cooling
4.204	65.8784	31.5932	310.87	cooling
4.206	65.7614	30.7403	317.54	cooling
4.208	65.5039	29.9879	322.03	cooling
4.210	65.6187	32.2511	302.55	cooling
4.212	65.7866	39.8451	235.22	cooling
4.214	65.4545	38.3742	245.54	cooling
4.216	65.6251	36.5709	263.44	
4.218	65.5953	35.0188	277.24	
4.220	65.0427	29.4859	322.40	
4.222	66.2688	33.7768	294.61	
4.224	65.4510	37.3521	254.78	
4.226	65.5570	34.2155	284.18	
4.228	65.7791	35.2413	276.89	
4.230	65.3430	33.3448	290.13	
4.232	65.5622	31.7266	306.79	
4.234	65.8579	36.4752	266.42	
4.236	65.4627	32.1394	302.15	
4.238	65.7441	29.8315	325.62	
4.240	65.7994	32.0249	306.24	
4.242	65.2250	28.1495	336.17	
4.244	66.1157	30.8873	319.42	
4.246	65.5452	32.6524	298.24	
4.248	65.5632	28.5511	335.59	
4.250	65.9537	30.1740	324.42	
4.252	65.3418	31.0561	310.87	
4.254	65.4880	29.6877	324.61	
4.256	65.8548	32.7996	299.72	
4.258	65.5574	30.1154	321.36	
4.260	65.6579	26.6619	353.58	
4.262	65.8977	28.4194	339.82	
4.264	65.5044	26.7953	350.98	cooling
4.266	65.7870	29.1383	332.30	cooling
4.268	65.5390	31.3913	309.62	cooling
4.270	65.2256	29.6504	322.57	cooling
4.272	65.7782	31.2669	312.92	cooling
4.274	65.0041	33.1249	289.05	cooling
4.276	65.4040	34.7044	278.36	cooling
4.278	65.1979	37.1459	254.35	cooling
4.280	65.4120	37.4887	253.18	
4.282	65.1204	34.0133	282.05	
4.284	65.5776	35.3392	274.18	
4.286	65.3573	34.5493	279.34	
4.288	65.2071	33.5929	286.65	
4.290	65.3205	33.0192	292.88	
4.292	65.1406	34.0285	282.10	
4.294	65.6166	35.8294	270.08	
4.296	65.1483	39.5389	232.20	
4.298	65.3569	38.8745	240.12	
4.300	64.9808	34.3838	277.43	
4.302	65.7346	34.3084	284.95	
4.304	64.9887	31.4815	303.82	
4.306	65.4362	33.1317	292.91	
4.308	65.2343	36.0178	264.91	
4.310	64.8932	33.5437	284.25	
4.312	65.2729	31.6590	304.78	
4.314	65.2194	34.5900	277.72	
4.316	65.2750	33.8102	285.30	
4.318	65.2576	37.0100	256.13	
4.320	65.1206	36.0998	263.14	heating
4.322	64.7630	27.2858	339.81	heating
4.324	65.8397	28.3946	339.52	
4.326	64.8589	29.1750	323.55	
4.328	65.3839	31.1864	310.07	
4.330	65.4723	36.5603	262.15	
4.332	64.7754	31.0131	306.13	
4.334	65.2001	24.5556	368.53	
4.336	65.4660	27.4704	344.51	
4.338	65.2358	25.9972	355.78	
4.340	65.4190	29.9794	321.34	
4.342	65.2778	34.4744	279.30	
4.344	64.7615	31.1008	305.21	
4.346	65.7100	32.1451	304.34	
4.348	64.9812	32.0934	298.20	
4.350	65.3368	31.0307	311.06	
4.352	65.4498	35.6797	269.93	
4.354	65.0566	35.5964	267.12	
4.356	65.0703	32.3798	296.41	
4.358	65.6165	36.9403	260.01	
4.360	65.1270	35.5109	268.53	
4.362	65.4570	36.0251	266.86	
4.364	65.2807	37.5101	251.80	
4.366	64.9639	34.1059	279.79	
4.368	65.6187	35.3652	274.31	
4.370	65.1966	38.0377	246.25	
4.372	65.2813	37.1620	254.96	heating
4.374	65.6359	40.1538	231.05	heating
4.376	65.2666	38.6162	241.64	heating
4.378	65.1140	33.0737	290.51	heating
4.380	65.9216	36.9194	262.97	heating
4.382	65.4346	37.7077	251.40	heating
4.384	65.5963	34.5913	281.13	heating
4.386	65.8220	35.3146	276.62	heating
4.388	65.5977	32.8914	296.55	heating
4.390	65.8328	30.2404	322.72	heating
4.392	65.9804	34.6400	284.17	heating
4.394	65.7121	34.6878	281.30	heating
4.396	66.0100	34.3338	287.21	heating
4.398	66.1281	35.8396	274.63	heating
4.400	65.5721	32.0030	304.38	heating
4.402	66.2402	32.0663	309.86	heating
4.404	66.2308	35.0075	283.11	heating
4.406	65.8546	31.9688	307.25	heating
4.408	66.1840	32.1487	308.60	heating
4.410	66.2627	35.5576	278.41	heating
4.412	66.1082	34.5583	286.07	heating
4.414	66.3267	35.4919	279.58	heating
4.416	66.2728	35.9799	274.67	heating
4.418	66.3376	33.0807	301.55	heating
4.420	66.5548	35.2316	284.01	heating
4.422	66.3468	37.0921	265.26	heating
4.424	66.2761	34.3993	289.03	heating
4.426	67.0602	37.7297	265.94	heating
4.428	66.2219	36.2913	271.39	heating
4.430	66.5448	32.6954	306.92	heating
4.432	66.9109	35.7018	282.98	heating
4.434	66.4672	34.9238	286.01	heating
4.436	66.7197	31.9445	315.31	heating
4.438	66.9573	34.8102	291.48	heating
4.440	66.7766	31.5710	319.21	heating
4.442	66.8122	32.3101	312.84	heating
4.444	67.1789	36.1118	281.69	heating
4.446	66.2897	31.2201	317.98	heating
4.448	67.6240	34.6403	299.07	heating

4.450	66.6055	36.5095	272.88	heating
4.452	66.9241	32.2558	314.34	heating
4.454	67.1711	33.1437	308.53	heating
4.456	66.8057	32.4073	311.89	
4.458	66.8761	27.0026	361.54	
4.460	67.5897	33.8624	305.81	
4.462	66.9247	31.7910	318.56	
4.464	66.9942	31.3491	323.20	
4.466	67.5436	35.4150	291.31	
4.468	66.2431	29.2937	335.03	
4.470	67.8128	31.3153	330.93	
4.472	66.6253	34.8318	288.28	
4.474	66.9939	32.9714	308.49	
4.476	67.0561	35.7609	283.76	
4.478	66.9311	38.1925	260.58	
4.480	66.6080	31.4947	318.38	
4.482	67.5108	35.2313	292.68	
4.484	66.8390	31.2872	322.35	
4.486	66.8456	29.0549	342.65	
4.488	67.4203	34.4889	298.59	
4.490	66.2838	32.2657	308.45	
4.492	67.6377	32.6013	317.68	
4.494	66.6569	35.5817	281.76	
4.496	66.9584	33.4543	303.79	
4.498	67.0556	35.8530	282.92	
4.500	67.1376	40.7531	239.23	
4.502	66.5123	36.6438	270.82	
4.504	67.2244	36.1721	281.56	
4.506	67.0430	34.0370	299.27	
4.508	66.8620	34.7152	291.48	
4.510	67.0163	38.0441	262.70	
4.512	66.6482	38.5377	254.88	
4.514	67.2397	39.2855	253.46	
4.516	66.7354	39.8421	243.84	
4.518	66.9762	38.2689	260.29	heating
4.520	67.0082	35.5705	285.05	heating
4.522	67.1142	36.9916	273.13	heating
4.524	66.9983	38.3769	259.51	heating
4.526	67.0778	36.9769	272.93	heating
4.528	67.6391	35.7241	289.38	heating
4.530	67.2291	36.1352	281.93	heating
4.532	67.3269	35.1270	291.96	heating
4.534	67.3661	34.3910	298.99	heating
4.536	67.5313	32.5489	317.19	heating
4.538	67.2156	31.6252	322.70	heating
4.540	67.7372	35.2143	294.89	heating
4.542	67.5610	34.0076	304.23	heating
4.544	67.7077	34.9152	297.33	heating
4.546	67.5736	37.9976	268.17	heating
4.548	67.6767	37.0163	278.00	heating
4.550	68.1150	34.3269	306.36	heating
4.552	67.6545	33.6887	307.97	heating
4.554	67.7018	32.2086	321.82	heating
4.556	67.9174	35.5397	293.57	
4.558	67.6436	35.9060	287.77	
4.560	67.5487	34.0880	303.39	
4.562	68.3835	36.0029	293.60	
4.564	67.7658	34.6137	300.60	
4.566	67.9947	36.3302	287.11	
4.568	67.6764	38.9780	260.21	
4.570	67.8709	37.3243	276.97	
4.572	68.1320	35.5109	295.78	heating
4.574	67.7734	34.9610	297.51	heating
4.576	67.9386	33.3568	313.56	
4.578	68.0083	35.5661	294.16	
4.580	67.6346	33.1003	313.13	
4.582	67.5719	29.2777	347.22	
4.584	68.5083	31.8236	332.63	
4.586	67.6407	31.1119	331.21	
4.588	68.1544	33.6691	312.68	
4.590	67.7888	35.6140	291.73	
4.592	67.7584	31.8120	325.93	
4.594	68.1105	33.2719	315.89	
4.596	67.7095	36.6066	282.01	
4.598	67.9414	34.8512	300.03	
4.600	67.8809	35.3063	295.36	
4.602	67.5972	30.0898	340.09	
4.604	67.5524	24.9907	385.91	
4.606	68.3892	30.1327	346.88	
4.608	67.7268	29.9143	342.85	
4.610	67.9387	31.0923	334.09	
4.612	67.8086	31.9966	324.71	
4.614	67.5320	29.1868	347.68	cooling
4.616	68.1383	29.5072	350.27	cooling
4.618	67.7253	31.5677	327.85	cooling
4.620	67.8532	31.2238	332.12	cooling
4.622	67.6233	31.8495	324.37	cooling
4.624	67.6632	32.1623	321.89	cooling
4.626	67.3714	31.5467	324.83	cooling
4.628	67.9545	35.3115	295.98	cooling
4.630	67.5723	33.2209	311.47	cooling
4.632	67.5551	32.3074	319.60	cooling
4.634	67.4925	30.9076	331.72	cooling
4.636	67.1908	28.5421	350.43	cooling
4.638	67.8263	29.4866	347.63	cooling
4.640	67.1143	32.9208	310.04	cooling
4.642	67.6505	36.5186	282.28	cooling
4.644	67.0338	36.4810	277.03	cooling
4.646	67.2682	36.3382	280.45	cooling
4.648	66.8037	33.8996	298.35	cooling
4.650	67.0605	33.5821	303.55	cooling
4.652	67.3417	33.0636	310.80	cooling
4.654	66.8543	32.8673	308.17	cooling
4.656	67.0720	31.6992	320.73	cooling
4.658	66.7718	34.2270	295.09	cooling
4.660	67.0846	35.8700	283.03	cooling
4.662	66.4258	33.5718	297.89	cooling
4.664	67.2671	33.9857	301.77	cooling
4.666	66.5401	30.1284	330.15	cooling
4.668	66.8536	30.2464	331.92	cooling
4.670	66.4283	31.9225	312.87	cooling
4.672	66.6357	33.9773	296.12	cooling
4.674	66.9561	33.9144	299.59	cooling
4.676	66.3184	32.4916	306.71	cooling
4.678	66.6700	29.4784	337.22	cooling
4.680	66.4977	32.1439	311.49	cooling
4.682	66.5810	31.6777	316.47	cooling
4.684	65.9738	26.7308	355.82	cooling
4.686	67.0904	30.3697	332.95	cooling
4.688	66.0006	28.2043	342.70	cooling
4.690	66.5026	29.8361	332.46	cooling
4.692	66.1758	34.2887	289.13	cooling
4.694	66.1212	34.9714	282.44	cooling
4.696	66.5403	35.0988	285.08	cooling
4.698	65.8689	33.2981	295.32	cooling
4.700	66.2919	28.9189	338.87	cooling
4.702	66.3326	33.7108	295.79	cooling
4.704	65.8436	34.1285	287.57	cooling
4.706	65.6708	33.1713	294.68	cooling
4.708	66.6252	40.0904	240.59	cooling
4.710	65.3510	35.4204	271.39	
4.712	66.1666	34.2487	289.40	
4.714	65.8787	36.6201	265.29	
4.716	65.5311	32.3384	300.96	
4.718	66.3061	33.6897	295.74	
4.720	65.7325	35.3294	275.67	
4.722	66.0191	31.3674	314.19	
4.724	66.3822	37.6021	260.95	
4.726	65.6313	36.0739	268.00	
4.728	65.7564	30.5632	319.10	
4.730	66.6919	35.8347	279.79	
4.732	65.3880	30.5907	315.51	
4.734	66.3409	31.9141	312.15	
4.736	65.9813	37.0397	262.42	
4.738	65.4257	33.6174	288.41	
4.740	66.5024	37.5479	262.53	
4.742	65.7704	39.2266	240.68	
4.744	66.1494	33.7281	293.97	
4.746	66.3285	38.0143	256.73	
4.748	65.6996	36.5645	264.17	
4.750	65.7668	30.1359	323.07	
4.752	66.5040	33.0326	303.49	
4.754	65.5523	29.9376	322.92	
4.756	66.3508	30.8488	321.90	
4.758	66.0190	34.2481	288.07	
4.760	65.4907	29.6781	324.72	
4.762	66.2811	31.2843	317.32	
4.764	66.0177	35.2816	278.69	
4.766	66.0677	31.8110	310.61	
4.768	66.2313	35.6697	277.11	
4.770	65.8525	36.6970	264.36	
4.772	65.6733	32.8529	297.59	
4.774	66.4105	35.5988	279.37	
4.776	65.6755	32.9821	296.44	
4.778	66.2431	31.4890	315.12	
4.780	66.0633	33.3259	296.84	
4.782	65.6672	31.6278	308.64	
4.784	65.9934	30.2561	324.04	
4.786	66.0129	32.6679	302.34	
4.788	65.9982	32.4393	304.28	
4.790	65.9435	34.4054	285.96	
4.792	66.0325	35.9859	272.44	
4.794	65.4824	33.1943	292.76	
4.796	66.3300	34.6298	287.43	
4.798	65.7407	34.4150	284.03	
4.800	66.1356	34.7143	284.90	

4.802	65.9624	34.2019	287.98	
4.804	65.8239	33.4312	293.71	
4.806	65.9439	30.8944	317.80	
4.808	65.7798	31.5339	310.51	
4.810	66.3417	33.2637	299.92	
4.812	65.6049	33.3126	292.80	
4.814	66.2864	34.4463	288.70	
4.816	65.5599	32.4725	300.01	
4.818	66.1963	30.2970	325.50	
4.820	65.8394	29.4485	329.96	
4.822	66.1516	30.7401	321.08	
4.824	65.8736	28.6774	337.26	
4.826	66.0897	31.9611	309.45	
4.828	65.8240	30.8352	317.25	
4.830	65.7212	28.6483	336.15	
4.832	66.3581	29.0758	338.04	
4.834	65.4114	25.8283	358.91	
4.836	66.3358	25.5920	369.43	
4.838	65.6250	27.3603	346.95	cooling
4.840	65.8704	28.5136	338.72	cooling
4.842	65.7161	31.9311	306.33	cooling
4.844	65.8767	33.8726	290.19	cooling
4.846	65.6686	27.5739	345.41	cooling
4.848	65.8788	28.5436	338.52	cooling
4.850	65.4004	26.8765	349.30	cooling
4.852	65.4536	26.9696	348.94	cooling
4.854	65.9489	32.8591	300.03	cooling
4.856	64.9060	31.7759	300.40	cooling
4.858	65.8528	33.4542	293.76	cooling
4.860	65.2299	35.4798	269.75	cooling
4.862	65.1606	32.1166	299.61	cooling
4.864	65.3924	32.6301	297.06	cooling
4.866	65.2501	32.7910	294.31	cooling
4.868	65.2268	28.0913	336.71	cooling
4.870	65.4160	33.3537	290.71	cooling
4.872	65.0156	36.2892	260.47	cooling
4.874	65.0399	36.4707	259.04	
4.876	65.5372	40.3010	228.82	
4.878	64.7692	35.3086	267.12	
4.880	65.4593	30.6251	315.85	
4.882	65.3082	30.7053	313.75	
4.884	64.9513	29.7316	319.34	
4.886	65.3754	33.8512	285.83	
4.888	65.3797	38.6348	242.50	
4.890	65.2778	35.3064	271.76	
4.892	65.3682	35.2926	272.70	
4.894	65.2277	35.5106	269.45	
4.896	64.9699	30.7516	310.26	
4.898	65.3755	29.2573	327.49	
4.900	65.1499	28.1179	335.77	
4.902	65.1985	27.9791	337.47	
4.904	65.3960	30.8012	313.68	
4.906	64.9752	31.3852	304.57	
4.908	65.2476	30.0692	318.97	
4.910	65.1385	29.7649	320.74	
4.912	65.4042	29.2368	327.93	
4.914	65.1970	29.0785	327.49	
4.916	65.3947	31.6367	306.09	
4.918	65.0197	30.4953	313.04	
4.920	65.1462	28.7816	329.72	
4.922	65.4676	29.8474	322.97	
4.924	65.1535	30.7952	311.53	
4.926	65.3982	30.7203	314.43	
4.928	65.2433	32.9254	293.03	
4.930	65.1068	31.5101	304.63	
4.932	65.1342	31.7166	303.00	
4.934	65.7198	35.2791	276.01	
4.936	64.9760	33.1246	288.80	
4.938	65.5799	33.9391	286.89	
4.940	65.1254	36.1194	263.00	
4.942	65.2404	35.4996	269.66	
4.944	65.4283	36.0137	266.71	
4.946	65.4214	37.9768	248.84	
4.948	65.1718	33.7380	285.01	
4.950	65.5144	34.7580	278.87	
4.952	65.0887	32.9008	291.85	
4.954	65.0298	30.7687	310.65	
4.956	65.8335	35.0952	278.71	
4.958	64.8377	33.6934	282.39	
4.960	65.8185	35.2280	277.37	
4.962	65.1480	37.9009	247.05	
4.964	65.1211	32.8052	293.01	
4.966	65.2834	30.4132	316.17	
4.968	65.5705	31.2256	311.41	
4.970	65.0645	25.5916	357.91	
4.972	65.7876	31.9986	306.37	
4.974	64.9837	33.3924	286.44	
4.976	65.0085	30.0461	317.01	
4.978	65.7154	31.4656	310.55	
4.980	64.6819	27.5300	336.86	
4.982	65.8283	26.1034	360.19	
4.984	65.1849	30.9786	310.15	
4.986	64.9720	28.7642	328.30	
4.988	65.1848	30.4823	314.65	
4.990	65.6838	35.7761	271.18	
4.992	64.9224	30.7272	310.05	
4.994	65.8411	35.8050	272.34	
4.996	64.9124	35.1122	270.20	
4.998	64.9468	29.3637	322.64	
5.000	65.6265	32.5782	299.65	
5.002	64.7856	33.2093	286.31	
5.004	65.7330	33.2840	294.22	
5.006	65.3743	40.4733	225.78	
5.008	64.9144	37.5667	247.97	
5.010	65.1184	34.1522	280.78	
5.012	65.7320	36.2033	267.74	
5.014	64.8342	30.5122	311.20	
5.016	65.7050	32.4647	301.39	
5.018	65.0746	34.2750	279.26	
5.020	65.0498	32.7343	293.01	
5.022	65.5551	36.9449	259.41	
5.024	65.0252	36.8358	255.60	
5.026	65.4778	31.6061	307.12	
5.028	65.4354	32.2953	300.49	
5.030	64.9579	28.0997	334.20	
5.032	65.1404	25.0888	363.15	
5.034	65.6697	30.8920	315.33	
5.036	64.9949	30.9310	308.86	
5.038	65.4562	31.7611	305.52	
5.040	65.2811	32.7435	295.02	
5.042	64.9918	28.7708	328.42	
5.044	65.4727	29.5367	325.84	
5.046	65.1979	30.4980	314.63	
5.048	65.1452	29.2748	325.24	
5.050	65.4510	32.4667	299.07	
5.052	65.0745	34.0965	280.88	
5.054	65.1609	32.8845	292.65	
5.056	65.4014	35.1830	273.99	
5.058	65.1993	33.2949	289.28	
5.060	65.1356	29.2853	325.06	
5.062	65.5309	30.7070	315.75	
5.064	64.9677	29.4999	321.59	
5.066	65.2980	28.4385	334.21	
5.068	65.1741	29.7734	320.98	
5.070	65.3959	30.8832	312.93	
5.072	65.1594	29.9794	318.98	
5.074	65.2918	31.1501	309.57	
5.076	65.1270	29.2069	325.69	
5.078	65.3349	28.8965	330.39	
5.080	65.4685	29.2034	328.82	
5.082	65.1138	29.1583	326.01	
5.084	65.6182	28.7616	334.18	
5.086	65.0626	29.0035	326.95	cooling
5.088	65.2071	28.5736	332.16	cooling
5.090	64.8833	27.5014	338.95	cooling
5.092	65.3300	29.1950	327.64	cooling
5.094	64.8223	30.7106	309.30	cooling
5.096	65.3004	34.9306	275.37	cooling
5.098	64.8192	34.6108	273.90	cooling
5.100	65.0134	34.0561	280.69	cooling
5.102	65.1637	36.1114	263.42	
5.104	64.9526	36.7973	255.29	
5.106	65.2267	33.7736	285.19	
5.108	65.0462	35.7152	265.95	
5.110	65.0779	37.1435	253.29	
5.112	64.8139	37.1384	250.94	
5.114	65.3043	37.3333	253.62	
5.116	64.5756	32.2255	293.32	
5.118	65.4192	31.7818	305.00	
5.120	64.9298	32.6124	293.03	
5.122	64.9327	31.1678	306.15	
5.124	64.9573	30.6498	311.07	
5.126	65.1682	32.1968	298.96	
5.128	65.1797	30.4997	314.45	
5.130	65.2464	32.8147	294.06	
5.132	64.8948	29.9157	317.16	
5.134	64.8668	26.4669	348.18	
5.136	65.3429	28.2264	336.54	
5.138	64.5704	26.8670	341.86	
5.140	65.3980	28.0562	338.58	
5.142	65.1032	31.3548	306.00	
5.144	64.9724	30.0044	317.06	
5.146	64.9458	28.7563	328.14	
5.148	65.3377	30.0477	319.98	
5.150	64.9127	27.4360	339.81	
5.152	65.3888	30.7816	313.79	

5.154	64.9348	33.1178	288.49	
5.156	64.8314	32.2506	295.41	
5.158	64.9555	33.1541	288.35	
5.160	65.0621	34.6330	275.90	
5.162	65.2111	33.4911	287.61	
5.164	65.2276	35.8249	266.60	
5.166	64.8746	34.9177	271.62	
5.168	64.9693	33.8990	281.72	
5.170	65.2238	35.3817	270.58	
5.172	64.8485	33.5275	283.99	
5.174	65.3040	34.1138	282.81	
5.176	64.9733	36.1714	261.15	
5.178	64.8855	35.3983	267.36	
5.180	64.8533	33.8515	281.10	
5.182	65.2311	32.5426	296.39	
5.184	65.2726	32.3765	298.27	
5.186	65.2946	35.1316	273.49	
5.188	64.9318	35.6577	265.43	
5.190	65.0563	35.4535	268.41	
5.192	65.0527	35.9325	264.04	
5.194	65.2505	35.8363	266.70	
5.196	65.0630	34.7529	274.83	heating
5.198	65.2394	34.4132	279.51	heating
5.200	65.0073	33.5416	285.30	heating
5.202	65.3109	35.1874	273.13	heating
5.204	65.2217	33.9899	283.18	heating
5.206	65.4831	32.2753	301.10	heating
5.208	65.6640	31.1687	312.77	heating
5.210	65.3844	31.9750	302.93	heating
5.212	65.5163	32.3084	301.10	heating
5.214	65.1524	31.3230	306.74	heating
5.216	65.9618	34.2740	287.32	
5.218	65.2033	35.8954	265.74	
5.220	65.7151	37.3922	256.81	heating
5.222	65.3344	37.4717	252.63	heating
5.224	65.5665	36.3339	265.06	heating
5.226	65.4520	33.6530	288.33	heating
5.228	66.0630	36.0161	272.44	heating
5.230	65.7565	34.8761	280.00	heating
5.232	65.8120	34.5728	283.25	heating
5.234	65.8194	33.3677	294.24	heating
5.236	65.5638	30.2349	320.33	heating
5.238	66.1521	29.4771	332.54	heating
5.240	65.6934	31.2547	312.26	heating
5.242	66.1885	32.6850	303.78	heating
5.244	65.8830	34.0964	288.21	heating
5.246	66.0900	33.6714	293.94	heating
5.248	65.8468	31.0111	315.86	heating
5.250	66.4777	33.4812	299.18	
5.252	65.9466	32.0949	306.94	
5.254	66.0588	30.5039	322.38	
5.256	65.9925	29.8061	328.11	
5.258	65.9620	30.7644	319.14	
5.260	66.1546	31.8356	311.18	
5.262	65.9537	32.0410	307.49	
5.264	66.1488	28.8726	337.99	
5.266	65.9928	28.8896	336.42	
5.268	66.1375	30.0418	327.29	
5.270	65.7256	28.6469	336.20	
5.272	66.3685	30.1903	328.03	
5.274	65.9159	30.9930	316.65	
5.276	66.1795	32.4561	305.78	
5.278	65.9577	32.1581	306.47	
5.280	65.8886	30.2232	323.38	
5.282	65.8963	27.1956	350.90	
5.284	66.1349	28.1926	344.03	
5.286	66.1357	28.5859	340.47	
5.288	65.8264	30.0344	324.53	cooling
5.290	65.9575	31.1351	315.74	cooling
5.292	65.6711	30.2744	320.95	cooling
5.294	66.0483	31.2479	315.54	cooling
5.296	65.7753	33.0464	296.76	cooling
5.298	65.9458	34.0449	289.25	cooling
5.300	65.6537	33.7932	288.88	cooling
5.302	65.6430	33.2775	293.46	
5.304	65.7007	32.3751	302.17	
5.306	65.9271	33.6180	292.95	
5.308	66.1593	36.1407	272.18	
5.310	65.5805	35.7046	270.89	
5.312	65.7343	31.8362	307.36	
5.314	65.5945	29.8825	323.81	
5.316	65.8424	30.7646	318.06	
5.318	65.7000	31.2608	312.26	
5.320	65.9799	33.6145	293.46	
5.322	65.5388	32.5089	299.49	
5.324	65.7037	32.3299	302.60	
5.326	65.8195	33.5299	292.77	
5.328	65.6095	31.0762	313.12	
5.330	65.8971	29.2164	332.59	
5.332	65.8156	32.1211	305.51	
5.334	65.6592	32.2610	302.83	
5.336	65.6894	32.9367	296.97	
5.338	65.7671	33.9937	288.09	
5.340	65.8015	32.7382	299.79	
5.342	66.1090	34.5854	285.83	
5.344	65.5520	32.6766	298.09	
5.346	65.6434	30.3331	320.16	
5.348	66.0233	33.6875	293.19	
5.350	65.5752	32.3005	301.71	
5.352	65.8418	30.2516	322.70	
5.354	65.9143	32.6986	301.17	
5.356	65.5758	31.5693	308.34	
5.358	65.7771	31.5779	310.09	
5.360	65.7103	32.8010	298.39	
5.362	65.5911	29.6995	325.43	
5.364	66.1240	33.0382	299.99	
5.366	65.7018	33.2137	294.57	
5.368	65.6665	29.9315	324.01	
5.370	66.2198	33.1550	299.80	
5.372	65.4630	31.1247	311.35	
5.374	66.0116	28.9324	336.20	
5.376	65.8709	31.2102	314.27	
5.378	65.6589	31.0505	313.80	
5.380	65.8832	32.2899	304.60	
5.382	65.6900	33.4438	292.38	
5.384	65.5191	28.8806	332.21	
5.386	66.0294	30.7162	320.19	
5.388	65.8253	32.3076	303.91	
5.390	65.7606	32.7255	299.53	
5.392	66.0446	35.6419	275.67	
5.394	65.3788	32.7935	295.46	
5.396	66.0023	31.9891	308.40	
5.398	65.7280	33.2401	294.57	
5.400	65.7492	28.8404	334.66	
5.402	65.8934	28.8977	335.45	
5.404	65.8115	31.2812	313.09	
5.406	65.6029	29.4048	328.21	
5.408	66.2395	31.8881	311.47	
5.410	65.7046	29.2125	330.88	cooling
5.412	65.8125	27.9351	343.44	cooling
5.414	65.9555	31.0863	316.16	cooling
5.416	65.2887	30.1805	318.33	cooling
5.418	65.7693	31.6193	309.64	cooling
5.420	65.5064	35.0570	276.09	cooling
5.422	65.8908	35.8755	272.15	cooling
5.424	65.5499	36.9400	259.41	
5.426	65.4498	36.2275	264.96	
5.428	65.3742	34.5718	279.29	
5.430	65.8257	35.0567	278.99	
5.432	65.2237	30.4075	315.68	
5.434	65.7625	32.7706	299.14	
5.436	65.5034	36.8560	259.75	
5.438	65.2398	37.0535	255.57	
5.440	65.5066	36.4859	263.14	
5.442	65.3337	34.6186	278.50	
5.444	66.0636	36.5359	267.73	
5.446	65.4814	38.8424	241.54	
5.448	65.2819	34.8294	276.12	
5.450	65.5233	34.1090	284.84	
5.452	65.7265	37.4815	256.10	
5.454	65.2240	36.6562	259.03	
5.456	65.9712	37.8939	254.58	heating
5.458	65.1526	33.6330	285.79	heating
5.460	65.6581	31.4962	309.75	heating
5.462	65.5468	32.5453	299.23	heating
5.464	65.7136	29.7206	326.35	heating
5.466	65.9885	28.3789	341.01	
5.468	65.6981	31.8888	306.55	
5.470	65.4737	30.8480	313.96	
5.472	65.8934	33.8723	290.34	
5.474	65.6062	32.4324	300.79	
5.476	65.5148	28.3109	337.33	
5.478	66.1793	31.0866	318.19	
5.480	65.2307	27.8230	339.18	
5.482	65.8404	28.1792	341.48	
5.484	65.5737	31.8795	305.51	
5.486	65.4635	28.6850	333.48	
5.488	66.0119	30.7565	319.67	
5.490	65.6214	34.3287	283.74	
5.492	65.4426	31.6792	306.14	
5.494	65.9660	34.0546	289.35	
5.496	65.2192	30.9619	310.62	
5.498	65.7539	29.4713	328.98	
5.500	65.8912	35.3734	276.71	
5.502	65.4104	35.5176	271.04	
5.504	65.5579	35.8104	269.73	

5.506	65.5675	36.6052	262.61	
5.508	65.4081	31.7437	305.24	
5.510	66.0680	35.3519	278.51	
5.512	65.4104	37.0205	257.42	
5.514	65.4623	35.7024	269.84	
5.516	65.9265	37.6423	256.46	
5.518	65.1474	35.4282	269.47	
5.520	65.9619	35.8644	272.90	
5.522	65.4467	36.5259	262.23	
5.524	65.8210	32.3211	303.75	heating
5.526	65.7311	34.2104	285.80	
5.528	65.7283	36.3316	266.54	heating
5.530	65.5462	32.1303	302.99	heating
5.532	66.2750	33.9393	293.19	heating
5.534	65.5306	30.4989	317.64	heating
5.536	66.1186	33.1263	299.15	
5.538	65.9865	35.6919	274.69	
5.540	65.3112	30.0305	319.90	
5.542	66.2884	30.9337	320.57	
5.544	65.6966	33.0905	295.64	
5.546	66.1643	30.5380	323.03	
5.548	65.9805	34.1717	288.41	
5.550	65.6803	32.9039	297.19	
5.552	65.8220	30.6895	318.55	
5.554	66.3188	34.5342	288.20	
5.556	65.4246	29.8924	322.18	
5.558	66.4321	32.4037	308.54	
5.560	65.7320	33.0904	295.97	
5.562	65.4846	28.7295	333.26	
5.564	66.1730	31.7559	312.06	
5.566	65.7368	33.2355	294.69	
5.568	66.1169	32.8051	302.04	
5.570	65.9944	37.6130	257.34	
5.572	65.5073	34.6182	280.08	
5.574	66.0534	33.9170	291.38	
5.576	65.9801	35.4564	276.76	
5.578	65.5161	29.9634	322.36	
5.580	66.5184	30.9131	322.84	
5.582	65.6880	33.8613	288.58	
5.584	65.7538	33.6020	291.53	
5.586	66.0556	36.8281	265.01	
5.588	65.9922	33.8272	291.64	
5.590	65.8831	32.3080	304.43	
5.592	66.2484	37.3645	261.89	
5.594	65.4503	36.0104	266.94	
5.596	66.1985	37.2178	262.77	
5.598	65.4757	36.6204	261.64	
5.600	66.0552	35.0249	281.36	heating
5.602	66.4664	37.4215	263.35	heating
5.604	65.8702	37.4572	257.63	heating
5.606	65.9085	32.2331	305.34	heating
5.608	66.2125	32.3141	307.36	heating
5.610	66.4891	30.1615	329.39	heating
5.612	66.1691	30.6546	322.01	
5.614	66.5025	34.3397	291.63	
5.616	65.5945	34.2467	284.24	
5.618	66.5796	38.0020	259.12	
5.620	65.5894	37.3645	255.92	heating
5.622	66.6946	36.1502	276.95	heating
5.624	66.3694	35.6640	278.41	heating
5.626	66.4976	35.5004	281.06	heating
5.628	66.0818	32.1462	307.70	heating
5.630	66.7004	33.7829	298.47	heating
5.632	66.6202	32.0771	313.21	heating
5.634	66.7766	35.5606	283.04	heating
5.636	66.7085	34.3109	293.75	heating
5.638	66.2715	32.0936	309.90	heating
5.640	67.2562	34.9582	292.85	heating
5.642	65.9823	32.4531	304.01	
5.644	67.3422	31.6239	323.86	
5.646	66.5109	32.5760	307.69	
5.648	66.9367	34.8945	290.53	
5.650	66.6380	37.7801	261.66	
5.652	66.8153	37.6047	264.86	
5.654	66.6934	29.4485	337.70	heating
5.656	67.2320	31.8888	320.46	
5.658	66.6500	30.1025	331.38	
5.660	66.6575	29.3072	338.66	
5.662	67.0464	31.5359	321.98	
5.664	66.1562	29.9657	328.14	
5.666	67.5138	33.8613	305.13	
5.668	66.5360	36.2842	274.30	
5.670	66.8246	33.4011	303.06	
5.672	66.8021	35.0733	287.69	
5.674	66.6500	33.2193	303.12	
5.676	66.7804	29.5650	337.44	
5.678	67.1449	36.3872	278.88	
5.680	66.4592	34.3819	290.85	

5.682	66.9855	34.7383	292.39
5.684	66.7794	35.1434	286.85
5.686	66.3087	29.5422	333.37
5.688	67.3732	30.9813	329.97
5.690	66.5623	30.6522	325.60
5.692	66.7007	28.4752	346.60
5.694	67.0186	34.5213	294.66
5.696	66.5854	35.3544	283.18
5.698	66.9127	33.3168	304.62
5.700	66.7976	34.1354	296.15
5.702	66.6800	29.3585	338.40
5.704	67.1177	29.7765	338.58
5.706	66.8668	33.2812	304.52
5.708	66.3110	28.9956	338.34
5.710	67.1704	31.1794	326.34
5.712	66.7891	32.7749	308.41
5.714	66.5966	33.5653	299.50
5.716	67.1637	38.6769	258.29
5.718	66.2176	36.0705	273.35
5.720	66.9295	32.8241	309.24
5.722	66.5445	34.9304	286.65
5.724	66.9853	35.8628	282.19
5.726	66.7597	33.5996	300.67
5.728	66.8132	32.9669	306.89
5.730	66.3046	28.9769	338.46
5.732	67.0651	32.8265	310.45
5.734	66.9430	34.3957	295.11
5.736	66.4791	34.0989	293.60
5.738	67.1050	35.2966	288.41
5.740	66.4631	36.7172	269.71
5.742	67.0708	35.9320	282.34
5.744	66.3258	34.2837	290.53
5.746	67.3268	35.5991	287.68
5.748	66.5645	33.5859	299.02
5.750	67.0755	35.4070	287.14
5.752	66.3760	33.1640	301.14
5.754	66.8802	33.1726	305.63
5.756	67.0343	31.4059	323.05
5.758	66.5815	30.8973	323.55
5.760	66.9307	30.1435	333.56
5.762	66.6422	35.6099	281.38
5.764	67.0710	38.8963	255.47
5.766	66.0958	35.1693	280.41
5.768	67.4107	37.7256	269.16
5.770	66.3618	37.6647	260.20
5.772	66.9608	38.0183	262.43
5.774	66.4362	35.9379	276.53
5.776	66.3976	29.9004	330.93
5.778	67.0116	28.1203	352.63
5.780	66.9388	35.4764	285.28
5.782	66.6018	33.7636	297.75
5.784	66.7796	35.5935	282.77
5.786	66.9332	38.0506	261.88
5.788	66.2065	34.1514	290.65
5.790	67.4399	37.6326	270.27
5.792	66.2058	36.3857	270.38
5.794	66.8143	34.4551	293.41
5.796	66.7882	38.0612	260.47
5.798	66.5282	35.3224	282.95
5.800	66.9331	32.9036	308.55
5.802	67.1531	38.3868	260.83
5.804	66.3939	30.7513	323.18
5.806	66.9384	27.5041	357.56
5.808	66.9615	31.0017	326.05
5.810	66.3781	30.4806	325.49
5.812	67.3400	35.3995	289.61
5.814	66.3312	35.9697	275.29
5.816	66.6534	32.9000	306.05
5.818	67.0709	37.7782	265.60
5.820	66.4387	36.0011	275.98
5.822	66.6739	29.1470	340.26
5.824	66.9013	31.5014	320.98
5.826	66.8199	31.1991	322.98
5.828	66.8731	32.2220	314.19
5.830	66.9785	34.9522	290.39
5.832	66.3813	30.9240	321.50
5.834	67.2348	32.4225	315.65
5.836	66.7033	32.1092	313.67
5.838	66.5512	29.5053	335.90
5.840	66.9903	31.7712	319.34
5.842	66.6315	36.0255	277.51
5.844	66.8972	36.4536	276.04
5.846	66.5473	36.0662	276.38
5.848	67.2933	36.4103	280.02
5.850	66.4694	33.2422	301.28
5.852	67.0501	34.6365	293.90
5.854	66.5012	34.2913	292.05
5.856	66.7326	33.4954	301.37

5.858	66.9220	33.9624	298.85	
5.860	66.5716	35.8323	278.72	
5.862	66.8550	34.2348	295.77	
5.864	66.7488	37.8727	261.82	
5.866	66.8776	38.6188	256.23	
5.868	66.3916	34.2564	291.37	
5.870	67.5228	36.5570	280.77	
5.872	66.2435	33.1561	300.01	
5.874	67.0718	34.2107	297.96	
5.876	66.6242	37.6829	262.41	
5.878	66.4222	34.3295	290.99	
5.880	66.7620	31.4264	320.39	
5.882	67.0486	37.8348	264.89	
5.884	66.7269	33.5774	300.57	
5.886	66.8198	33.9607	297.94	
5.888	66.8615	33.4234	303.19	
5.890	66.2957	28.9701	338.44	
5.892	67.8160	36.9436	279.92	
5.894	66.0038	35.3099	278.31	
5.896	66.9105	32.0779	315.83	
5.898	66.7375	36.6966	272.39	
5.900	66.4140	34.9849	284.97	
5.902	66.8419	31.9973	315.94	
5.904	67.0931	38.0769	263.09	
5.906	66.6585	33.1908	303.46	
5.908	67.0188	36.4031	277.60	
5.910	66.9885	38.7325	256.20	
5.912	66.1230	30.0049	327.49	
5.914	67.7682	35.0800	296.39	
5.916	66.1509	37.2931	261.66	
5.918	66.7393	33.0527	305.44	
5.920	66.8205	35.6269	282.84	
5.922	66.4351	32.8811	304.24	
5.924	66.7646	27.0781	359.84	
5.926	67.2929	32.3923	316.45	
5.928	66.6078	28.0748	349.38	
5.930	66.9819	30.2069	333.44	
5.932	67.1827	36.2422	280.54	
5.934	66.1143	30.4958	322.96	
5.936	67.5019	30.9749	331.20	
5.938	66.5280	31.9717	313.33	
5.940	66.7012	27.5370	355.11	
5.942	66.8983	28.4800	348.35	
5.944	66.7032	29.1185	340.79	
5.946	67.0637	28.7087	347.77	
5.948	67.1865	36.5838	277.48	
5.950	66.6667	33.8757	297.32	
5.952	66.6364	28.6628	344.31	
5.954	67.1026	29.7419	338.75	
5.956	66.3962	29.3942	335.50	cooling
5.958	67.0384	31.9941	317.75	cooling
5.960	66.5508	34.1528	293.76	cooling
5.962	66.4058	32.8940	303.86	cooling
5.964	66.7051	34.0506	296.08	cooling
5.966	66.5188	35.9520	277.15	cooling
5.968	66.4680	30.3622	327.38	
5.970	66.4783	28.7101	342.45	
5.972	66.8372	32.5543	310.85	cooling
5.974	66.2122	34.1000	291.17	cooling
5.976	66.8031	37.8399	262.61	cooling
5.978	66.2940	38.0337	256.24	
5.980	66.5236	35.6096	280.30	
5.982	66.6482	36.2877	275.28	
5.984	66.4229	37.3194	263.89	
5.986	66.4212	31.9577	312.49	
5.988	66.7375	34.8809	288.85	
5.990	66.6248	36.1892	275.96	
5.992	66.1434	32.6739	303.47	
5.994	67.2271	37.8127	266.70	
5.996	65.8799	35.6603	274.01	
5.998	66.7449	34.1769	295.30	
6.000	66.4842	35.0514	285.01	
6.002	66.0572	30.2572	324.60	
6.004	66.7814	30.6507	327.60	
6.006	66.4068	34.5099	289.21	
6.008	66.2877	28.2827	344.60	
6.010	66.7437	33.0346	305.65	
6.012	66.5440	37.1096	266.89	
6.014	65.9403	31.8808	308.82	
6.016	67.2681	35.6475	286.71	
6.018	65.7213	30.4446	319.86	
6.020	66.6543	29.2229	339.40	
6.022	66.6578	35.9272	278.64	
6.024	65.7529	30.4055	320.50	
6.026	66.7890	28.5768	346.48	
6.028	66.5835	35.6093	280.85	
6.030	66.0891	30.7591	320.34	
6.032	66.7422	34.2178	294.90	
6.034	66.3415	35.1171	283.12	
6.036	66.1157	27.0980	353.78	
6.038	67.2284	33.6420	304.53	
6.040	65.8087	32.3828	303.08	
6.042	66.4866	29.2540	337.59	
6.044	66.9281	37.2313	269.27	
6.046	65.9017	33.5079	293.72	
6.048	66.7459	31.1379	322.86	
6.050	66.9113	38.5291	257.35	
6.052	66.0325	32.3806	305.13	
6.054	66.7765	33.9616	297.54	
6.056	66.5771	36.8864	269.21	
6.058	66.1532	30.3909	324.26	
6.060	67.2038	36.5125	278.28	
6.062	66.1405	38.5002	250.62	
6.064	66.3364	32.0774	310.63	
6.066	66.9161	36.0662	279.72	
6.068	66.1694	33.8738	292.83	
6.070	66.5195	28.6959	342.95	
6.072	66.8906	32.1740	314.78	
6.074	65.9351	27.7397	346.32	
6.076	66.7143	29.0851	341.19	
6.078	66.6568	35.6676	280.98	
6.080	65.9219	32.0449	307.17	
6.082	66.7613	30.3480	330.16	
6.084	66.4413	31.3314	318.35	
6.086	66.5031	27.7643	351.25	
6.088	66.4277	27.8625	349.68	
6.090	66.5001	27.7210	351.62	
6.092	66.1507	23.6192	385.64	cooling
6.094	66.7172	27.3434	357.01	cooling
6.096	66.3211	31.2599	317.91	cooling
6.098	66.1896	30.3037	325.38	cooling
6.100	66.4292	31.9559	312.57	cooling
6.102	66.1412	33.6330	294.76	cooling
6.104	66.1033	30.1634	325.87	cooling
6.106	66.4138	34.3303	290.91	cooling
6.108	66.1420	36.6283	267.60	cooling
6.110	65.8009	32.8799	298.50	cooling
6.112	66.3023	34.1275	291.73	cooling
6.114	65.6697	32.5765	300.06	cooling
6.116	66.0324	31.0207	317.46	cooling
6.118	66.3663	35.1559	282.99	cooling
6.120	65.4879	32.8592	295.85	cooling
6.122	65.8185	30.3190	321.88	cooling
6.124	66.0139	35.6657	275.17	cooling
6.126	65.5050	34.6113	280.12	
6.128	66.0349	35.9457	272.82	
6.130	65.9328	40.4357	231.19	
6.132	65.3798	33.6095	288.07	
6.134	66.0569	35.3221	278.68	
6.136	65.7358	36.6990	263.28	
6.138	65.3813	28.3395	335.86	
6.140	66.5461	32.6586	307.26	
6.142	65.2030	32.1948	299.29	
6.144	65.7433	28.6256	336.55	
6.146	66.1342	35.0687	281.68	
6.148	65.1493	32.5210	295.85	
6.150	65.8869	29.5938	329.07	
6.152	66.2340	37.8463	257.40	
6.154	65.1926	30.5575	314.04	
6.156	65.9331	30.4509	321.72	
6.158	66.0145	34.9502	281.67	
6.160	65.1894	26.0258	355.10	
6.162	66.6731	31.4273	319.58	
6.164	65.1809	32.1919	299.12	
6.166	65.6244	23.8568	378.71	
6.168	66.3026	30.2984	326.46	
6.170	65.1364	29.4791	323.31	
6.172	65.7635	25.8438	361.96	
6.174	66.4476	37.3287	264.02	cooling
6.176	64.9497	32.0659	298.16	
6.178	65.8225	30.2408	322.62	
6.180	66.0382	36.8554	264.60	
6.182	64.8888	30.1775	314.73	
6.184	66.4806	32.6782	306.49	
6.186	65.2064	34.4558	278.82	
6.188	65.5327	27.9257	340.99	
6.190	66.1085	35.9399	273.54	
6.192	65.4835	39.0191	239.96	
6.194	65.4605	31.7903	305.29	
6.196	66.4212	38.1589	256.26	
6.198	65.1766	35.0278	273.36	
6.200	65.7869	35.3856	275.65	
6.202	66.0144	42.1736	216.17	
6.204	64.9493	34.8611	272.81	
6.206	66.0772	32.1890	307.27	
6.208	65.6375	36.0152	268.59	

6.210	65.4084	32.7135	296.45	
6.212	65.8015	34.0084	288.27	
6.214	65.6251	34.2648	284.35	
6.216	65.5473	29.7884	324.23	
6.218	66.1705	35.2142	280.68	
6.220	65.4573	35.7568	269.30	
6.222	65.6817	32.3955	301.81	
6.224	65.6805	32.3376	302.32	
6.226	65.4401	31.2035	310.43	
6.228	65.8068	33.0067	297.40	
6.230	65.6646	35.5130	273.39	
6.232	65.4282	33.2712	291.57	
6.234	65.6268	32.2496	302.64	
6.236	65.7520	32.2876	303.43	
6.238	65.5624	30.2065	320.58	
6.240	65.6018	28.2036	339.10	
6.242	65.7015	27.6610	344.92	
6.244	65.7045	28.9094	333.63	
6.246	65.5086	31.0181	312.73	
6.248	65.7538	35.5179	274.15	
6.250	65.6482	35.3030	275.14	
6.252	65.3712	29.9465	321.20	
6.254	65.9651	31.7823	309.94	
6.256	65.4550	31.0529	311.93	
6.258	65.8209	30.5173	320.10	
6.260	65.8197	32.7330	300.00	
6.262	65.0199	28.0238	335.45	
6.264	66.2234	30.4013	324.80	
6.266	65.6623	36.5209	264.23	
6.268	65.3619	32.5253	297.73	
6.270	65.7972	34.6472	282.44	
6.272	65.6842	36.0148	269.02	
6.274	65.2752	32.3339	298.68	
6.276	66.2020	40.2723	235.11	
6.278	65.1383	38.7389	239.37	
6.280	65.7137	35.8509	270.77	
6.282	65.9857	40.4725	231.33	
6.284	64.8048	34.3681	275.97	
6.286	66.5073	35.5229	280.94	heating
6.288	65.6599	39.2000	239.92	heating
6.290	65.5635	31.3331	310.37	heating
6.292	66.1631	35.3315	279.55	heating
6.294	65.7772	37.8501	253.22	heating
6.296	65.5322	34.1382	284.65	heating
6.298	66.6611	41.9192	224.34	heating
6.300	65.3318	37.4073	253.20	heating
6.302	66.4289	36.3136	273.06	heating
6.304	66.0915	41.1786	225.89	heating
6.306	65.5778	37.0644	258.54	heating
6.308	66.5387	36.2767	274.39	heating
6.310	66.1816	38.4023	251.88	heating
6.312	66.2360	33.2900	298.73	heating
6.314	66.4602	36.3550	272.97	heating
6.316	66.3141	35.7629	277.01	heating
6.318	65.8778	29.9432	325.82	heating
6.320	67.0845	35.4286	287.03	heating
6.322	65.9746	35.3211	277.94	heating
6.324	66.8608	36.9241	271.44	heating
6.326	66.4057	40.2566	237.10	heating
6.328	66.1671	36.4766	269.21	heating
6.330	66.8540	33.9723	298.14	heating
6.332	66.8658	36.6966	273.55	heating
6.334	66.6178	34.1710	294.20	heating
6.336	66.5272	33.0978	303.11	heating
6.338	66.8038	32.3813	312.11	heating
6.340	66.4969	29.0802	339.26	heating
6.342	67.2372	31.2103	326.66	heating
6.344	66.7591	32.7748	308.14	heating
6.346	66.9214	31.3732	322.32	heating
6.348	66.8267	32.8741	307.85	heating
6.350	67.0334	36.1840	279.72	heating
6.352	67.1920	35.0810	291.16	heating
6.354	67.2870	35.0165	292.60	heating
6.356	67.0084	31.3817	323.03	heating
6.358	66.9048	28.0752	352.07	heating
6.360	67.4338	31.3857	326.85	
6.362	66.9413	31.1000	324.98	
6.364	67.0908	29.6815	339.20	
6.366	67.5150	32.8977	313.88	
6.368	66.9121	32.3661	313.23	
6.370	67.2069	31.4405	324.30	
6.372	67.0909	32.3675	314.84	
6.374	67.1790	30.4748	332.80	
6.376	67.2249	32.6851	313.18	
6.378	67.3165	38.1322	264.62	
6.380	66.9700	37.6414	265.93	
6.382	67.4147	40.3650	245.26	
6.384	66.8931	37.7491	264.25	
6.386	66.9579	32.9869	308.02	
6.388	67.6567	34.8307	297.64	
6.390	66.7897	34.7561	290.45	
6.392	67.2248	33.6067	304.82	
6.394	67.2590	39.6544	250.29	
6.396	67.2012	42.3198	225.60	
6.398	67.0240	42.5109	222.26	
6.400	67.3840	43.6902	214.83	heating
6.402	66.9724	37.7886	264.61	heating
6.404	67.4294	38.1198	265.75	heating
6.406	67.0344	39.3346	251.16	heating
6.408	67.1798	38.1393	263.31	heating
6.410	67.6101	39.2591	257.06	heating
6.412	67.1658	38.5627	259.35	heating
6.414	67.4792	37.1865	274.67	heating
6.416	67.5178	39.5639	253.46	heating
6.418	67.2401	36.2374	281.11	heating
6.420	67.5460	34.4126	300.43	heating
6.422	67.9009	37.0164	280.03	heating
6.424	67.4295	36.5145	280.31	heating
6.426	68.0264	39.3780	259.76	heating
6.428	67.4307	38.7908	259.68	heating
6.430	67.6347	32.5560	318.06	heating
6.432	67.9512	31.8047	327.75	heating
6.434	67.9943	33.0758	316.61	heating
6.436	67.9909	31.8653	327.56	heating
6.438	68.0449	32.5342	321.98	heating
6.440	67.7253	29.4137	347.38	heating
6.442	68.0800	30.3624	341.99	heating
6.444	68.2365	33.7230	312.94	
6.446	67.9387	32.8692	317.98	
6.448	68.1442	30.2531	343.56	
6.450	67.8307	30.6894	336.77	
6.452	68.0828	32.0111	327.07	
6.454	68.0730	32.9462	318.50	
6.456	68.2838	34.2376	308.70	
6.458	67.7538	31.1550	331.85	
6.460	68.1392	30.8405	338.19	
6.462	67.9473	31.9347	326.53	
6.464	67.7232	30.6739	335.93	
6.466	68.0099	30.6551	338.70	
6.468	68.3230	33.9739	311.45	
6.470	68.0993	35.7113	293.67	
6.472	67.9777	38.6902	265.55	
6.474	68.1393	38.8376	265.68	
6.476	67.9260	33.3665	313.36	
6.478	68.3628	32.7424	322.98	
6.480	68.0314	34.9132	300.29	
6.482	67.9788	33.6983	310.83	
6.484	68.0204	34.9710	299.66	
6.486	67.8010	35.1215	296.31	
6.488	68.1519	33.8776	310.77	
6.490	68.5657	39.0537	267.59	
6.492	67.7687	37.9717	270.17	
6.494	67.9593	35.4692	294.59	
6.496	68.2587	37.4494	279.35	
6.498	67.9691	35.5550	293.90	
6.500	68.2545	38.3446	271.20	
6.502	68.1523	41.1019	245.27	
6.504	67.8167	36.1024	287.56	
6.506	68.2637	36.7684	285.57	
6.508	67.6901	36.6446	281.49	
6.510	68.1589	35.6039	295.18	
6.512	68.3813	40.1657	255.83	
6.514	67.6542	37.0551	277.45	
6.516	68.0628	34.6623	302.85	
6.518	68.4366	40.5959	252.44	
6.520	67.5607	36.5349	281.32	
6.522	68.2173	37.4297	279.16	
6.524	68.2381	40.6792	249.88	heating
6.526	67.6602	35.4922	291.67	heating
6.528	68.5731	39.9478	259.55	heating
6.530	67.8797	43.8724	217.68	heating
6.532	68.1585	38.6136	267.89	heating
6.534	68.5275	38.8139	269.42	heating
6.536	67.8952	34.3893	303.80	heating
6.538	68.3536	30.4992	343.23	heating
6.540	68.8131	38.5774	274.15	heating
6.542	67.8540	37.6165	274.17	heating
6.544	68.4799	37.7952	278.22	heating
6.546	68.9174	42.7071	237.65	heating
6.548	68.0552	36.3733	287.26	heating
6.550	69.0309	37.3050	287.66	heating
6.552	68.3505	38.3268	272.23	heating
6.554	68.3854	31.1019	338.05	heating
6.556	68.9950	34.4082	313.60	heating
6.558	68.5569	38.5374	272.19	heating
6.560	68.7690	35.3840	302.71	heating

6.562	69.2726	39.4023	270.84	heating
6.564	68.3429	33.9947	311.44	heating
6.566	68.7412	28.4068	365.72	heating
6.568	69.4378	31.8185	341.10	heating
6.570	68.6985	28.5041	364.45	
6.572	69.2175	29.2468	362.42	
6.574	68.7455	32.9138	324.89	
6.576	68.7177	32.5451	327.98	
6.578	69.2372	35.9111	302.17	
6.580	68.7777	35.1695	304.73	
6.582	68.8750	29.4434	357.53	
6.584	69.2030	29.6176	358.93	
6.586	68.7311	30.2577	348.84	
6.588	68.7954	30.7169	345.26	
6.590	69.0787	33.2120	325.21	
6.592	69.0555	33.8120	319.56	
6.594	68.8827	33.6510	319.45	cooling
6.596	68.7611	35.8542	298.37	cooling
6.598	68.7121	37.0744	286.86	cooling
6.600	68.7743	35.6481	300.36	
6.602	68.9168	35.6159	301.94	
6.604	69.0623	39.0399	272.22	
6.606	68.8442	40.9492	252.93	
6.608	68.8337	43.7383	227.54	
6.610	68.7306	43.7662	226.36	
6.612	68.7795	40.6856	254.73	
6.614	69.4803	44.9573	222.35	
6.616	68.3358	41.2657	245.45	
6.618	68.8581	37.4362	284.91	
6.620	69.2135	43.3874	234.17	
6.622	68.3320	40.7658	249.95	heating
6.624	69.3244	41.2807	254.28	heating
6.626	69.1095	45.8168	211.20	heating
6.628	68.7063	36.2229	294.53	heating
6.630	69.3340	37.7403	286.46	heating
6.632	68.9907	40.0084	262.79	heating
6.634	68.6173	33.0846	322.18	heating
6.636	70.1648	39.8766	274.63	heating
6.638	68.2937	36.4556	288.68	heating
6.640	69.4154	33.6542	324.25	heating
6.642	69.7783	42.2476	249.62	heating
6.644	68.5195	34.4365	309.04	heating
6.646	69.6893	30.5749	354.66	heating
6.648	69.8351	38.0719	288.00	heating
6.650	69.0570	29.8476	355.52	heating
6.652	70.1766	35.0111	318.85	heating
6.654	69.3743	37.8399	285.93	
6.656	69.0552	27.4817	376.95	
6.658	70.4346	35.7732	314.28	
6.660	68.9655	37.2556	287.52	
6.662	69.5992	30.7055	352.65	
6.664	70.1386	38.5050	286.83	
6.666	68.7861	33.9867	315.53	
6.668	69.6117	31.6998	343.75	
6.670	70.0499	40.3175	269.59	
6.672	68.6909	32.5614	327.59	
6.674	70.2763	33.9247	329.61	
6.676	69.3832	38.3245	281.61	
6.678	69.1123	31.2447	343.35	
6.680	69.8379	36.3080	304.02	
6.682	69.4421	38.0139	284.96	
6.684	69.3339	30.4972	352.14	
6.686	70.1349	37.7487	293.65	
6.688	68.8161	36.0414	297.17	
6.690	69.4963	33.5996	325.48	
6.692	69.7702	36.7363	299.52	
6.694	69.0655	33.2193	325.02	
6.696	70.3037	35.9378	311.60	
6.698	69.3902	41.2072	255.54	
6.700	69.3670	36.7115	296.09	
6.702	69.4224	35.2563	309.79	
6.704	69.8882	35.7030	309.96	
6.706	69.3103	34.8750	312.23	
6.708	69.8890	37.7300	291.59	
6.710	69.0496	35.3752	305.33	
6.712	69.6968	35.2072	312.72	
6.714	69.2213	32.9930	328.49	
6.716	69.8785	35.5918	310.88	
6.718	69.5664	36.8277	296.85	
6.720	69.1005	33.3205	324.42	
6.722	69.6801	32.8411	334.02	
6.724	69.0431	31.6506	339.04	
6.726	69.6603	31.5193	345.83	
6.728	69.7242	39.6599	272.60	cooling
6.730	69.2020	38.2384	280.75	
6.732	69.2024	33.0151	328.12	
6.734	69.8399	35.9192	307.56	
6.736	68.9663	31.9985	335.19	
6.738	69.9697	35.0873	316.28	
6.740	69.2022	38.4100	279.20	
6.742	68.9054	31.8620	335.88	
6.744	69.9710	37.6901	292.70	
6.746	68.8931	38.9424	271.57	
6.748	69.1832	33.7444	321.33	
6.750	70.0975	40.4294	269.01	
6.752	68.6743	35.5352	300.48	
6.754	69.5349	32.7539	333.50	
6.756	69.8178	41.8091	253.96	
6.758	68.8432	36.5934	292.41	
6.760	69.8064	38.1295	287.22	
6.762	69.2326	41.5603	250.91	
6.764	68.8536	34.3277	313.05	
6.766	69.8634	38.7900	281.75	
6.768	68.7400	37.1130	286.77	
6.770	69.1630	30.6073	349.59	
6.772	69.9578	39.4514	276.61	
6.774	68.6085	38.3695	274.18	
6.776	69.4692	35.5416	307.63	
6.778	69.6271	39.0423	277.32	
6.780	68.8266	30.4630	347.85	
6.782	69.8277	33.9574	325.24	
6.784	69.3403	39.1741	273.52	
6.786	68.9619	31.1056	343.25	
6.788	69.8560	33.6417	328.36	
6.790	68.6996	30.7717	343.90	
6.792	69.4800	27.2571	382.84	
6.794	69.7117	34.0079	323.73	
6.796	68.6492	29.4924	355.04	cooling
6.798	69.5835	27.2047	384.25	cooling
6.800	69.5463	30.7276	351.97	cooling
6.802	68.9019	26.5305	384.19	cooling
6.804	69.5382	31.3758	346.02	cooling
6.806	68.9010	33.0324	325.23	cooling
6.808	68.7891	27.0311	378.63	cooling
6.810	69.4639	32.1972	337.90	cooling
6.812	68.6090	31.6398	335.21	cooling
6.814	69.1130	31.1848	343.90	cooling
6.816	68.9493	34.9747	308.05	cooling
6.818	68.4062	30.6323	342.50	cooling
6.820	69.5385	33.6487	325.42	cooling
6.822	68.8855	36.7347	291.52	cooling
6.824	68.6486	32.5719	327.11	cooling
6.826	69.1299	36.1798	298.76	cooling
6.828	68.3853	35.3901	299.17	cooling
6.830	68.6844	34.1155	313.44	cooling
6.832	69.1672	39.2337	271.41	cooling
6.834	68.3402	34.9641	302.63	cooling
6.836	69.0177	35.4094	304.73	cooling
6.838	68.5374	39.1135	266.79	cooling
6.840	68.5641	36.5798	290.01	cooling
6.842	68.9372	38.7413	273.79	cooling
6.844	68.3881	38.3236	272.60	cooling
6.846	68.5566	33.8541	314.65	cooling
6.848	68.8456	40.6216	255.91	cooling
6.850	68.0993	40.1452	253.46	cooling
6.852	68.3133	38.6812	268.68	
6.854	68.8475	40.8234	254.10	
6.856	67.9810	36.8876	281.93	
6.858	69.1063	41.1413	253.56	
6.860	68.0345	42.1365	234.82	
6.862	68.3589	37.9409	275.80	
6.864	68.5698	39.5954	262.71	
6.866	68.3168	38.8256	267.40	
6.868	68.5030	34.9718	304.03	
6.870	68.5056	37.3335	282.64	
6.872	68.0337	32.1874	325.02	
6.874	68.5852	33.8025	315.38	
6.876	68.3712	35.6716	296.49	
6.878	68.0594	33.0821	317.14	
6.880	68.9613	36.1436	297.56	
6.882	67.8857	32.2416	323.19	
6.884	68.5196	30.3193	346.37	
6.886	68.3596	32.6083	324.16	
6.888	68.2918	30.2249	345.16	
6.890	68.5663	32.9803	322.66	
6.892	68.2156	34.6753	304.11	
6.894	68.3726	31.2875	336.26	
6.896	68.7046	35.5662	300.47	
6.898	67.9731	32.9563	317.50	
6.900	68.3921	30.3833	344.63	
6.902	68.9050	35.7663	300.47	
6.904	67.8724	33.7916	309.02	
6.906	68.5779	34.1400	312.25	
6.908	68.3657	36.2776	290.95	
6.910	67.8288	31.4864	329.52	
6.912	68.9169	37.4159	285.62	

6.914	68.0771	38.3764	269.30	
6.916	68.3275	30.7132	341.05	
6.918	68.5785	32.7405	324.95	
6.920	67.9507	31.2915	332.39	
6.922	68.7062	32.2017	330.99	
6.924	68.7461	39.9838	260.79	cooling
6.926	67.8070	34.0278	306.28	
6.928	68.6208	34.2375	311.76	
6.930	68.3497	37.5257	279.49	
6.932	67.9209	33.7331	309.99	
6.934	68.7944	38.2923	276.57	
6.936	67.9101	37.6099	274.74	
6.938	68.3456	32.0071	329.49	
6.940	68.3449	34.2736	308.93	
6.942	68.0140	32.4223	322.71	
6.944	68.4784	32.0373	330.42	
6.946	68.6098	36.3631	292.39	
6.948	67.9787	31.7275	328.70	
6.950	68.5607	33.1237	321.31	
6.952	68.3590	36.4910	288.95	
6.954	68.3250	36.6821	286.91	
6.956	68.5478	37.6236	280.39	
6.958	68.0457	34.9681	299.92	
6.960	68.6369	33.9965	314.09	
6.962	68.2277	32.0082	328.41	
6.964	68.3265	29.0475	356.15	
6.966	68.4387	29.9171	349.28	
6.968	68.4702	32.8223	323.23	cooling
6.970	68.3457	36.4118	289.55	cooling
6.972	68.2218	38.9395	265.51	cooling
6.974	68.1790	35.9540	292.19	
6.976	68.6060	37.2539	284.27	
6.978	68.2536	38.7312	267.68	
6.980	67.9782	38.9777	262.95	
6.982	68.6645	42.9346	233.30	
6.984	67.9858	39.2704	260.37	
6.986	68.2507	35.9899	292.51	
6.988	68.3893	38.3160	272.68	
6.990	68.1298	35.6997	294.05	
6.992	68.3796	35.7462	295.89	
6.994	68.1853	36.8227	284.37	
6.996	68.1258	31.6678	330.57	
6.998	68.9838	35.7168	301.64	
7.000	68.0364	37.8407	273.79	
7.002	68.0733	34.9260	300.55	
7.004	68.7326	37.2951	285.05	
7.006	68.2347	34.3628	307.12	
7.008	68.2888	31.1018	337.18	
7.010	68.5994	35.7364	297.97	
7.012	68.0175	32.9463	318.00	
7.014	68.3660	31.6246	333.14	
7.016	68.0853	32.1975	325.40	
7.018	68.2335	28.9872	355.85	
7.020	68.9894	33.6279	320.63	
7.022	68.0748	36.1105	289.82	
7.024	68.1347	31.9878	327.75	
7.026	68.7068	35.7354	298.96	
7.028	68.4280	36.6825	287.84	
7.030	67.9521	32.8079	318.66	
7.032	68.7104	36.1829	294.93	
7.034	67.8123	33.2901	313.02	
7.036	68.2247	32.6270	322.77	
7.038	68.3140	37.6426	278.10	
7.040	68.2275	33.8285	311.90	
7.042	68.6782	33.0048	323.46	
7.044	68.3271	37.2720	281.58	
7.046	67.9331	34.0316	307.39	
7.048	68.7370	37.2012	285.94	
7.050	68.0865	36.9652	282.18	
7.052	68.0311	33.8264	310.14	
7.054	68.6375	35.1467	303.67	
7.056	68.0551	35.7807	292.64	
7.058	68.2919	36.0333	292.49	
7.060	68.1136	37.7557	275.26	
7.062	68.6662	35.6096	299.73	
7.064	68.1864	32.8609	320.30	
7.066	68.6172	35.9639	296.07	
7.068	67.7717	34.0930	305.37	
7.070	68.5234	33.0061	322.04	
7.072	67.9223	31.0237	334.57	
7.074	68.2811	31.9002	329.87	
7.076	68.3575	33.0886	319.79	
7.078	68.3945	36.4178	289.94	
7.080	68.0882	32.2972	324.52	
7.082	68.1620	31.5972	331.54	
7.084	68.9471	35.5302	303.00	
7.086	67.8112	35.9702	288.71	
7.088	68.7375	37.5910	282.41	
7.090	67.8752	37.5115	275.31	
7.092	68.3168	34.9419	302.61	
7.094	68.0702	36.5509	285.79	
7.096	68.3785	38.5649	270.32	
7.098	68.0269	33.3088	314.79	
7.100	68.7135	36.5209	291.90	
7.102	68.1858	37.2755	280.27	
7.104	68.2360	39.1602	263.63	
7.106	68.8536	41.7845	245.44	
7.108	67.6938	36.2815	284.82	
7.110	68.6526	34.1265	313.05	
7.112	67.9536	35.6964	292.48	
7.114	68.1663	32.8393	320.32	
7.116	68.1289	32.8460	319.92	
7.118	68.2384	35.2868	298.78	
7.120	68.0532	32.4939	322.42	
7.122	68.7203	37.2129	285.68	
7.124	67.9522	34.6444	302.01	
7.126	68.1465	31.5391	331.92	
7.128	68.5651	33.6192	316.86	
7.130	67.8011	31.5060	329.09	
7.132	68.4604	33.9489	312.92	
7.134	68.1790	36.4247	287.92	
7.136	68.0509	31.4027	332.29	
7.138	68.4604	31.7371	332.98	
7.140	67.9545	33.0083	316.86	
7.142	68.2975	32.6878	322.88	
7.144	68.3793	35.1430	301.36	
7.146	68.0822	32.3265	324.20	
7.148	68.2509	31.9671	328.99	
7.150	68.6077	38.0343	277.21	
7.152	67.9527	35.7166	292.29	
7.154	68.3321	36.7494	286.36	
7.156	68.3352	38.3597	271.79	
7.158	67.7901	34.9286	297.96	
7.160	68.5850	35.7977	297.29	
7.162	67.9708	36.7450	283.13	
7.164	68.4169	35.6387	297.20	
7.166	68.1237	37.6749	276.08	
7.168	68.1061	36.7321	284.47	
7.170	68.0610	34.0066	308.78	
7.172	68.4802	36.9190	286.17	
7.174	67.9302	34.6898	301.40	
7.176	68.3099	36.7305	286.33	
7.178	68.1392	38.3535	270.07	
7.180	67.8421	34.5611	301.76	
7.182	68.5969	33.5558	317.72	
7.184	68.0108	32.9733	317.69	
7.186	68.3965	33.7719	313.95	
7.188	68.0908	35.5602	294.96	
7.190	68.0660	33.3307	314.95	
7.192	68.2764	31.8680	330.12	
7.194	68.3921	34.5001	307.30	
7.196	68.2751	36.2978	289.94	
7.198	68.3159	38.9217	266.52	
7.200	67.9923	37.5413	276.10	
7.202	68.1580	34.8700	301.83	
7.204	68.4765	35.9765	294.68	
7.206	68.4794	38.3336	273.34	
7.208	68.4571	40.9329	249.57	
7.210	68.0941	40.1786	253.11	
7.212	68.1483	35.3936	296.99	
7.214	68.4514	35.1884	301.60	
7.216	68.3376	34.9628	302.61	
7.218	68.3770	36.6172	287.97	
7.220	68.2011	36.9824	283.06	
7.222	67.9834	34.2708	305.68	
7.224	68.6116	36.8354	288.12	
7.226	68.2291	36.5905	286.87	
7.228	68.4583	33.2483	319.25	
7.230	68.4322	36.0649	293.48	
7.232	67.9505	34.0849	307.06	
7.234	68.1704	32.2997	325.24	
7.236	68.7146	37.3280	284.59	
7.238	67.9989	32.7175	319.90	
7.240	68.6384	35.8157	297.61	
7.242	68.1409	37.4981	277.84	
7.244	67.7997	31.9041	325.47	
7.246	69.2276	37.6793	286.05	
7.248	67.6468	36.0382	286.60	
7.250	68.6760	34.8153	307.02	
7.252	68.6754	43.9338	224.34	
7.254	67.6455	40.3487	247.50	
7.256	68.4640	39.1196	266.07	
7.258	68.4667	41.6341	243.30	
7.260	67.9862	32.2677	323.86	heating
7.262	69.0384	38.7716	274.43	heating
7.264	67.9809	41.4669	240.41	heating

7.266	68.1357	33.9055	310.37	heating
7.268	69.3863	40.6851	260.24	heating
7.270	67.9861	39.8854	254.79	heating
7.272	68.8695	35.9288	298.68	heating
7.274	69.1138	40.7342	257.32	heating
7.276	68.1110	31.8735	328.57	heating
7.278	69.0290	30.7770	346.84	heating
7.280	69.0960	37.9056	282.81	heating
7.282	68.3406	33.5556	315.40	heating
7.284	69.4305	35.9790	303.31	heating
7.286	68.4509	35.3578	300.06	
7.288	69.0137	30.2208	351.74	
7.290	69.2932	34.6086	314.49	
7.292	68.6874	34.6957	308.21	
7.294	68.9170	30.4488	348.80	
7.296	69.2310	33.4958	324.02	
7.298	68.6370	30.4273	346.45	
7.300	69.0945	32.9565	327.67	
7.302	69.0053	36.8372	291.67	
7.304	68.3484	31.4381	334.67	
7.306	69.2802	30.3329	353.14	
7.308	68.8017	31.4727	338.47	
7.310	69.1229	30.8469	347.05	
7.312	68.9921	35.1796	306.58	
7.314	68.7305	37.1127	286.68	
7.316	68.7594	35.1306	304.92	
7.318	69.1131	37.2011	289.35	
7.320	68.8593	37.4483	284.81	
7.322	69.0303	38.7827	274.26	
7.324	68.6393	36.9330	287.49	
7.326	68.7903	35.3552	303.16	
7.328	69.1757	38.5693	277.51	
7.330	68.9772	40.0969	261.86	
7.332	68.9192	37.3784	285.98	
7.334	68.8162	36.3672	294.22	
7.336	68.8481	36.8531	290.10	
7.338	68.9163	37.8460	281.72	
7.340	69.0135	35.1328	307.20	
7.342	68.8429	31.7705	336.14	
7.344	69.2249	34.3845	315.90	
7.346	68.5898	35.1653	303.07	
7.348	69.0276	34.7396	310.89	
7.350	68.8705	35.0827	306.36	
7.352	69.0702	34.8804	310.00	
7.354	69.0903	37.0058	290.92	
7.356	68.7436	37.5202	283.11	
7.358	68.7472	36.0647	296.34	
7.360	69.1785	40.0319	264.28	
7.362	68.7925	37.5822	282.99	
7.364	69.0395	34.9448	309.14	
7.366	69.1402	38.0133	282.23	
7.368	68.5164	36.2160	292.87	
7.370	69.1123	36.8099	292.89	
7.372	68.8738	39.0481	270.43	
7.374	69.0446	38.9505	272.87	
7.376	69.3628	43.3317	236.03	
7.378	68.5742	39.6168	262.56	
7.380	68.5858	32.0660	331.13	
7.382	69.5232	37.5270	290.11	
7.384	68.7537	36.5039	292.41	
7.386	69.1706	35.3654	306.52	
7.388	68.9338	36.9178	290.29	
7.390	68.6161	32.4424	327.99	
7.392	69.2314	32.6463	331.72	
7.394	68.7377	33.1268	322.89	
7.396	68.7993	28.2658	367.52	
7.398	69.4103	34.0938	320.22	
7.400	68.6319	33.8552	315.33	
7.402	68.6448	31.4612	337.15	
7.404	69.5680	38.3169	283.36	
7.406	68.3062	33.4149	316.36	
7.408	69.2709	30.9048	347.87	
7.410	68.8825	34.8660	308.43	
7.412	68.7842	34.2512	313.12	
7.414	69.1939	36.5558	295.93	
7.416	68.7856	37.1197	287.12	
7.418	68.7446	31.8393	334.63	
7.420	69.4050	37.2966	291.13	
7.422	68.8005	36.4175	293.62	
7.424	68.7253	31.6727	335.96	
7.426	69.3478	33.9894	320.60	
7.428	68.4278	31.7917	332.19	
7.430	69.4289	32.0354	339.05	
7.432	69.0338	35.8136	301.21	
7.434	68.9441	35.1877	306.07	
7.436	68.9771	35.2059	306.21	
7.438	68.9932	36.1913	297.42	
7.440	68.7097	32.2609	330.49	
7.442	69.2413	36.4440	297.38	
7.444	68.8378	36.4204	293.93	
7.446	68.7605	34.4397	311.19	
7.448	69.1927	40.3480	261.54	
7.450	68.5822	42.2670	238.60	
7.452	69.3105	40.1732	264.19	
7.454	69.0868	39.2900	270.17	
7.456	68.9149	37.1663	287.87	
7.458	68.9377	38.2473	278.27	
7.460	69.0058	40.1193	261.92	
7.462	68.5865	35.8693	296.65	
7.464	69.2427	38.9338	274.81	
7.466	69.1050	43.1302	235.52	
7.468	68.7925	40.6213	255.43	
7.470	68.9210	38.5903	275.01	
7.472	68.7545	36.9085	288.75	
7.474	69.3332	35.9852	302.37	
7.476	69.3428	36.3050	299.56	
7.478	68.9726	36.4966	294.47	
7.480	69.0309	37.3512	287.24	
7.482	68.7488	34.1623	313.60	
7.484	68.9643	29.6968	356.04	
7.486	69.0320	29.7139	356.50	
7.488	69.1643	33.0175	327.75	
7.490	68.9089	32.6335	328.91	
7.492	68.6301	28.8080	361.07	
7.494	69.2637	31.1003	346.03	
7.496	69.0319	35.6489	302.69	
7.498	69.0381	32.7895	328.67	
7.500	68.9832	30.1510	352.10	
7.502	68.9689	29.5250	357.64	
7.504	68.6883	30.4919	346.33	cooling
7.506	69.1903	35.1861	308.32	cooling
7.508	68.6638	33.9556	314.70	cooling
7.510	69.1800	36.1346	299.63	cooling
7.512	68.6924	38.6062	272.80	cooling
7.514	68.4057	33.5146	316.36	
7.516	68.9914	33.2257	324.29	cooling
7.518	68.7013	37.7047	281.05	cooling
7.520	68.6012	36.0467	295.18	
7.522	68.8378	38.6088	274.09	
7.524	68.4539	36.6237	288.61	
7.526	68.5038	35.7269	297.19	
7.528	69.2368	41.2569	253.70	
7.530	68.0661	34.6969	302.56	
7.532	69.1941	35.2377	307.89	
7.534	68.5335	39.2463	265.55	
7.536	68.4624	34.3716	309.11	
7.538	68.9530	33.9665	317.23	
7.540	68.5537	36.0432	294.78	
7.542	68.5453	31.0535	339.94	
7.544	68.9713	36.0474	298.53	
7.546	68.5235	35.3595	300.70	
7.548	68.3261	31.5567	333.39	
7.550	69.2936	37.5208	288.09	
7.552	67.9366	34.6619	301.71	
7.554	69.1061	35.4216	305.42	
7.556	68.6391	40.3167	256.80	
7.558	68.3984	35.1310	301.64	
7.560	68.7584	33.6164	318.64	
7.562	68.7579	38.7964	271.67	
7.564	68.2271	34.9038	302.15	
7.566	68.8651	36.8152	290.60	
7.568	68.5663	36.7197	288.76	
7.570	68.5142	34.8513	305.23	
7.572	69.0448	38.0728	280.83	
7.574	67.8890	32.7041	319.03	
7.576	68.9985	29.7734	355.66	
7.578	68.7002	35.4505	301.48	
7.580	68.4159	34.8958	303.93	
7.582	68.5586	34.3699	309.99	
7.584	68.8901	38.3016	277.35	
7.586	68.1515	33.4341	314.79	
7.588	68.8269	33.2969	322.16	
7.590	68.7484	33.1365	322.90	
7.592	68.5524	32.0472	331.00	
7.594	68.8029	35.7012	300.14	
7.596	68.2008	35.3790	297.60	
7.598	68.9107	31.2265	341.69	
7.600	68.9332	36.8278	291.10	
7.602	68.4948	37.3700	282.21	
7.604	68.3618	33.5719	315.44	
7.606	68.8896	35.2836	304.71	
7.608	68.1952	33.5374	314.25	
7.610	68.8103	36.0539	297.01	
7.612	68.7430	39.4113	265.95	
7.614	68.4544	37.2104	283.29	
7.616	68.5440	36.5081	290.47	

7.618	68.7244	36.7255	290.14
7.620	68.4563	30.5061	344.10
7.622	68.9423	32.9206	326.61
7.624	68.5014	37.9370	277.13
7.626	68.2707	34.1452	309.42
7.628	68.7876	35.2250	304.32
7.630	68.5010	35.7914	296.58
7.632	68.4182	30.7627	341.43
7.634	69.0017	34.9383	308.86
7.636	68.1440	32.7279	321.12
7.638	68.4228	30.8853	340.36
7.640	68.9324	36.5933	293.22
7.642	68.0985	35.9938	291.10
7.644	68.9204	37.9739	280.60
7.646	68.3860	41.6777	242.17
7.648	68.2568	35.1606	300.09
7.650	68.5597	31.8514	332.84
7.652	68.8003	33.7430	317.87
7.654	68.3165	30.0187	347.25
7.656	69.2217	38.1479	281.75
7.658	68.1898	38.7297	267.12
7.660	68.2749	33.4091	316.13
7.662	69.2650	37.7930	285.36
7.664	68.1522	35.9223	292.23
7.666	69.0137	35.7426	301.67
7.668	68.4445	38.9395	267.53
7.670	68.2893	34.8037	303.62
7.672	68.8340	37.3831	285.17
7.674	68.6868	41.9179	242.72
7.676	68.3662	37.5253	279.64
7.678	68.9949	39.5048	267.39
7.680	68.2255	36.1134	291.17
7.682	68.3796	31.2359	336.79
7.684	69.3030	37.3502	289.72
7.686	68.3546	36.8083	286.04
7.688	68.6982	37.5431	282.49
7.690	68.7579	42.9618	233.90
7.692	68.1097	35.7783	293.15
7.694	68.9952	32.8142	328.06
7.696	68.8660	36.7236	291.44
7.698	68.3915	31.2331	336.92
7.700	69.1432	33.3276	324.74
7.702	68.5749	35.8119	297.07
7.704	68.5117	32.8501	323.35
7.706	69.3417	37.9702	284.45
7.708	68.3158	34.7632	304.23
7.710	68.6440	29.6691	353.39
7.712	68.9073	35.9828	298.53
7.714	68.3065	34.7033	304.69
7.716	68.8596	33.1295	323.97
7.718	68.9009	38.7453	273.43
7.720	68.5842	38.8675	269.45
7.722	68.9893	40.6841	256.65
7.724	68.6786	40.5545	255.01
7.726	68.2968	34.2029	309.13
7.728	69.2219	36.8274	293.73
7.730	68.6516	37.6777	280.85
7.732	68.6254	34.7924	306.77
7.734	68.7291	37.3128	284.86
7.736	68.4398	36.1963	292.36
7.738	68.7395	34.3711	311.62
7.740	68.7242	36.0876	295.92
7.742	68.4943	31.6492	334.08
7.744	68.9251	29.1766	360.41
7.746	68.7534	30.4113	347.65
7.748	68.4080	30.1705	346.71
7.750	68.8074	32.1205	332.65
7.752	68.6228	32.1282	330.90
7.754	68.6498	30.8646	342.60
7.756	68.6778	32.9509	323.94
7.758	68.5444	35.1964	302.37
7.760	68.4096	34.5275	307.21
7.762	68.7485	37.8699	279.98
7.764	68.7956	41.6428	246.20
7.766	68.6122	39.9070	260.27
7.768	68.5995	37.3182	283.63
7.770	68.5260	37.7622	278.94
7.772	68.5564	36.7756	288.16
7.774	68.6443	37.0838	286.16
7.776	68.5937	37.7542	279.63
7.778	68.2986	35.7499	295.12
7.780	68.9485	39.4480	267.49
7.782	68.3508	39.3222	263.21
7.784	68.3216	33.3615	316.99
7.786	69.1990	34.9167	310.84
7.788	68.4285	35.4959	298.61
7.790	68.6607	32.5422	327.49
7.792	68.7183	35.0945	304.87

7.794	68.4144	31.6443	333.40	
7.796	68.6658	31.0100	341.43	
7.798	68.8135	33.8521	317.00	
7.800	68.0115	26.6746	374.81	
7.802	69.1604	30.1128	354.05	
7.804	68.3207	35.4157	298.35	
7.806	68.3452	33.3603	317.21	
7.808	69.0225	35.6500	302.59	
7.810	68.3568	36.3057	290.61	
7.812	68.5534	33.7489	315.58	
7.814	68.8261	41.2028	250.46	
7.816	68.4437	38.6991	269.70	
7.818	68.3548	32.9482	321.04	
7.820	69.0635	37.8848	282.70	
7.822	67.8643	33.7646	309.19	
7.824	69.1109	35.8806	301.30	
7.826	68.4250	40.0432	257.34	
7.828	68.1132	36.0128	291.06	
7.830	69.1338	38.9783	273.42	
7.832	68.4164	41.4138	244.84	
7.834	68.3879	35.1413	301.45	
7.836	68.7764	40.6630	254.91	
7.838	68.4735	40.6850	251.96	
7.840	68.2857	33.8258	312.45	
7.842	69.0298	36.9063	291.27	
7.844	67.8990	33.3038	313.68	
7.846	68.9766	34.4798	312.79	
7.848	68.5575	37.8552	278.38	
7.850	68.0563	33.6796	311.70	
7.852	68.9794	34.8349	309.59	
7.854	68.3766	37.3420	281.39	
7.856	68.3342	32.7697	322.47	
7.858	68.4522	33.0960	320.58	
7.860	68.5934	32.7664	324.85	
7.862	68.3628	31.4168	334.99	
7.864	68.7429	35.8147	298.57	
7.866	68.1525	35.4962	296.10	
7.868	69.0312	39.2412	270.11	
7.870	68.4859	41.1500	247.86	
7.872	68.1576	35.7703	293.66	
7.874	68.7727	32.4626	329.23	
7.876	68.4928	33.9623	313.09	
7.878	68.4704	34.1779	310.94	
7.880	68.4434	36.0936	293.32	
7.882	68.7058	36.7728	289.54	
7.884	68.5785	34.5140	308.87	
7.886	68.4160	34.1015	311.13	
7.888	68.6431	34.7571	307.25	
7.890	68.6479	32.9596	323.59	
7.892	68.4901	31.4219	336.10	
7.894	68.5948	34.8460	306.01	
7.896	68.4737	36.2610	292.08	
7.898	68.6333	37.5993	281.39	
7.900	68.6443	39.3046	266.03	
7.902	68.0364	32.9057	318.53	
7.904	69.0314	33.2201	324.71	
7.906	68.5944	36.8189	288.11	
7.908	68.1946	35.1281	299.82	
7.910	69.1523	39.4841	269.01	
7.912	68.5763	40.8683	251.23	
7.914	68.5315	35.2974	301.34	
7.916	68.8483	38.7186	273.19	
7.918	68.5678	37.5470	281.27	
7.920	68.4930	32.9147	322.59	
7.922	68.9698	38.8440	273.16	
7.924	68.2017	36.8007	284.72	
7.926	69.2130	39.9146	265.65	
7.928	68.7221	45.0027	215.07	
7.930	68.0124	39.9938	254.05	
7.932	69.4044	40.5495	261.63	heating
7.934	68.5526	39.5549	262.93	heating
7.936	68.5853	31.6140	335.22	heating
7.938	69.0471	35.0885	307.91	heating
7.940	68.8232	35.0189	306.51	heating
7.942	68.6270	31.3791	337.73	
7.944	69.3741	38.6872	278.24	
7.946	68.3016	33.4602	315.91	
7.948	69.2436	31.3747	343.36	
7.950	68.9767	33.4225	322.37	
7.952	68.0859	27.5325	367.70	
7.954	69.6886	32.3713	338.36	
7.956	68.5720	37.0963	285.39	
7.958	68.6767	32.7150	326.07	
7.960	69.0165	37.9342	281.83	
7.962	68.7738	38.1041	278.09	
7.964	68.6462	33.5469	318.25	
7.966	69.4897	40.7738	260.37	
7.968	68.1056	34.9674	300.47	

7.970	69.2143	34.3552	316.07	
7.972	69.0379	39.9815	263.46	
7.974	68.1438	35.5526	295.51	
7.976	69.5608	37.0497	294.78	
7.978	68.6650	39.0383	268.63	
7.980	68.7461	31.4096	338.54	
7.982	69.1369	38.0324	282.03	
7.984	68.9472	43.3463	232.13	
7.986	68.5981	38.9190	269.10	
7.988	69.6491	47.1879	203.66	
7.990	68.2676	43.0485	228.66	heating
7.992	69.1460	37.4228	287.64	heating
7.994	69.3020	37.8484	285.19	heating
7.996	68.4158	31.7228	332.70	heating
7.998	69.6327	33.3001	329.43	heating
8.000	68.9126	37.6155	283.78	
8.002	68.8162	32.9114	325.55	heating
8.004	69.1710	35.2975	307.14	
8.006	69.1542	39.0846	272.65	
8.008	68.8481	36.4581	293.69	
8.010	69.4653	41.6081	252.59	
8.012	68.8223	39.2569	268.07	heating
8.014	69.0543	35.0779	308.07	heating
8.016	69.4069	37.4253	289.98	heating
8.018	68.8534	36.9211	289.54	heating
8.020	69.5630	36.1185	303.25	heating
8.022	69.1727	35.3868	306.34	heating
8.024	69.2471	32.7220	331.18	
8.026	69.3363	32.8521	330.81	
8.028	69.3713	35.7559	304.80	
8.030	69.1233	36.6531	294.41	
8.032	69.2841	39.6298	268.88	
8.034	69.5748	41.6667	253.05	
8.036	69.0435	38.7605	274.58	
8.038	69.3213	36.7127	295.67	
8.040	69.3254	38.4215	280.21	
8.042	69.5315	37.7285	288.36	
8.044	69.0383	34.9998	308.63	
8.046	69.5471	37.4032	291.45	
8.048	69.1140	38.1864	280.43	
8.050	69.5359	41.3573	255.50	
8.052	68.9550	37.5937	284.36	heating
8.054	69.0585	31.4821	340.71	heating
8.056	70.1628	35.1061	317.86	
8.058	68.9301	36.2874	295.98	
8.060	69.3784	35.5128	307.06	
8.062	69.4001	41.2462	255.28	
8.064	69.4668	41.0000	258.11	
8.066	69.2119	37.4044	288.40	heating
8.068	69.6968	37.1451	295.15	heating
8.070	68.9243	28.7098	364.63	heating
8.072	69.9515	34.1730	324.41	
8.074	69.0458	38.2822	278.94	
8.076	68.9005	33.8837	317.50	
8.078	70.4556	38.3568	291.04	
8.080	68.8512	40.1585	260.16	
8.082	69.5450	35.6057	307.73	
8.084	69.4667	38.8423	277.68	
8.086	69.4203	35.6664	306.05	
8.088	69.3374	32.9815	329.64	
8.090	70.1412	41.0079	264.16	
8.092	68.6916	32.0177	332.53	
8.094	70.0290	33.1195	334.66	
8.096	69.2653	35.5600	305.61	
8.098	68.9335	29.5019	357.53	
8.100	70.3717	32.2668	345.50	
8.102	68.9997	36.7180	292.70	
8.104	69.6328	34.9014	314.91	
8.106	69.3435	39.6168	269.54	
8.108	69.8034	38.8015	281.10	
8.110	69.1162	33.3009	324.74	
8.112	70.2338	40.4532	270.03	
8.114	68.7993	34.6853	309.32	
8.116	69.7933	32.4801	338.32	
8.118	69.4701	32.6856	333.53	
8.120	68.8509	29.2102	359.43	
8.122	70.3091	33.2459	336.06	
8.124	68.9507	38.4828	276.26	
8.126	69.5032	36.5218	299.05	
8.128	69.2963	39.3625	271.41	
8.130	69.6533	39.5488	272.96	
8.132	69.1955	36.8939	292.88	
8.134	69.8649	41.8449	254.06	
8.136	68.9829	38.3215	278.01	
8.138	69.6868	39.1423	276.95	
8.140	69.3264	39.3983	271.36	
8.142	68.9590	35.0895	307.10	
8.144	70.0635	33.7609	329.16	
8.146	69.0106	34.7435	310.71	
8.148	69.6104	36.0912	303.92	
8.150	69.2513	39.7207	267.76	
8.152	69.7953	42.7200	245.50	
8.154	69.1207	41.7339	248.32	
8.156	69.7391	44.6490	227.50	
8.158	69.3770	40.3526	263.17	
8.160	69.5336	37.7457	288.23	heating
8.162	69.4152	35.9814	303.15	heating
8.164	69.2069	34.3079	316.43	heating
8.166	70.0727	33.6560	330.20	
8.168	69.1606	33.5432	322.95	
8.170	69.9881	33.7939	328.18	
8.172	69.3347	31.4555	343.46	
8.174	69.8335	30.7729	354.17	
8.176	69.2108	30.5025	350.97	
8.178	69.7662	35.0346	314.92	
8.180	69.8156	35.3378	312.61	
8.182	69.4540	37.0476	293.83	
8.184	69.4974	35.7148	306.31	
8.186	69.2568	35.3494	307.44	
8.188	69.9601	34.3437	322.94	
8.190	69.0237	32.0930	334.86	
8.192	70.1484	35.2381	316.54	
8.194	69.2652	33.3640	325.52	
8.196	69.9506	34.6211	320.34	
8.198	69.2016	35.1168	309.05	
8.200	69.5387	34.8366	314.65	
8.202	69.8958	35.0271	316.16	
8.204	69.3069	36.9974	292.95	
8.206	69.5178	34.9469	313.46	
8.208	69.5191	38.3713	282.42	
8.210	69.9113	40.2735	268.73	
8.212	68.9515	38.1018	279.72	
8.214	70.5110	42.6224	252.87	
8.216	69.1661	37.0721	291.00	
8.218	69.9532	33.4463	331.01	
8.220	69.4365	33.8523	322.65	
8.222	69.3912	33.5701	324.80	
8.224	70.0288	33.1321	334.55	
8.226	69.4074	36.4106	299.19	
8.228	69.5943	33.4293	327.91	
8.230	69.4278	34.9550	312.57	
8.232	69.9636	36.4067	304.27	
8.234	68.9545	32.1194	333.99	
8.236	70.3315	35.8604	312.55	
8.238	69.0289	33.8677	318.81	
8.240	69.8445	32.9715	334.33	
8.242	69.6250	36.2412	302.70	cooling
8.244	69.3432	37.3968	289.66	
8.246	69.7349	34.9806	315.12	
8.248	69.4949	35.8583	304.99	
8.250	69.5723	29.6559	361.93	
8.252	69.4731	30.5012	353.36	cooling
8.254	69.8569	33.9356	325.70	cooling
8.256	68.7952	30.9756	342.92	cooling
8.258	70.1433	36.2185	307.60	cooling
8.260	68.9867	36.7653	292.16	cooling
8.262	69.4614	36.0704	302.76	cooling
8.264	69.3513	36.9497	293.79	cooling
8.266	69.0101	36.7801	292.23	cooling
8.268	69.3461	34.6785	314.34	cooling
8.270	69.2444	37.6017	286.91	cooling
8.272	69.1890	34.5859	313.75	cooling
8.274	69.0959	35.6873	302.92	cooling
8.276	69.5811	41.1667	257.64	cooling
8.278	68.3583	36.0912	292.57	
8.280	69.6956	36.4024	301.87	
8.282	69.0027	36.9855	290.31	
8.284	69.0508	36.3501	296.50	
8.286	69.3311	40.4380	261.98	
8.288	68.7490	41.8054	244.30	
8.290	69.1309	38.3244	279.33	
8.292	69.1062	41.5208	250.12	
8.294	69.2564	39.9771	265.48	
8.296	68.8995	35.3479	304.22	
8.298	69.5142	37.8736	286.89	
8.300	68.4804	35.5979	298.15	
8.302	69.5323	37.1693	293.44	
8.304	69.2907	39.9363	266.16	
8.306	68.6694	36.7375	289.53	
8.308	69.3669	36.3490	299.38	
8.310	68.9625	38.3818	277.28	
8.312	68.8662	30.9489	343.80	
8.314	68.7814	28.3716	366.40	
8.316	69.6292	32.2783	338.67	
8.318	68.9372	31.7054	337.59	
8.320	69.4539	35.1261	311.26	

8.322	68.4454	29.7843	350.55	
8.324	69.3036	26.5279	387.85	
8.326	69.5776	30.4399	354.87	
8.328	68.5337	32.5067	326.66	cooling
8.330	69.0365	32.6163	330.23	cooling
8.332	68.9396	37.3079	286.81	cooling
8.334	68.7200	34.5279	310.02	cooling
8.336	68.4424	30.4153	344.80	cooling
8.338	69.4009	30.9083	349.02	cooling
8.340	68.4461	27.5725	370.61	cooling
8.342	69.1893	33.1001	327.23	cooling
8.344	68.1484	33.2331	316.58	cooling
8.346	68.7830	31.0279	342.33	cooling
8.348	69.1027	34.1058	317.32	cooling
8.350	68.2882	38.3842	271.14	cooling
8.352	68.6788	37.9712	278.43	cooling
8.354	68.3405	39.2279	263.97	cooling
8.356	68.4495	37.4936	280.68	cooling
8.358	68.0883	37.9873	272.93	cooling
8.360	69.0353	42.4834	240.75	cooling
8.362	67.8769	38.5126	266.25	
8.364	68.4439	35.5103	298.61	
8.366	68.0586	34.5230	304.07	
8.368	68.6020	34.6557	307.80	
8.370	68.4592	33.5328	316.68	
8.372	68.4175	35.8099	295.66	
8.374	68.3135	35.8097	294.72	
8.376	68.3414	39.1583	264.61	
8.378	68.4686	39.5923	261.83	
8.380	67.9049	37.1614	278.76	
8.382	68.9790	38.7312	274.26	
8.384	68.1797	38.4287	269.76	
8.386	68.3822	34.6566	305.79	
8.388	68.0314	30.3952	341.25	
8.390	68.7267	32.9916	324.02	
8.392	68.4689	34.4963	308.03	
8.394	68.4318	37.8412	277.37	
8.396	68.2946	34.8088	303.62	
8.398	68.2609	32.6203	323.16	
8.400	68.8398	36.4258	293.90	
8.402	67.7912	35.3860	293.82	
8.404	68.7638	34.5924	309.84	
8.406	68.4748	39.4052	263.58	
8.408	68.2854	40.3836	252.99	
8.410	68.2130	39.6810	258.70	
8.412	68.4071	40.0978	256.69	
8.414	68.0858	33.5507	313.13	
8.416	68.6011	37.2970	283.84	
8.418	68.4271	38.7631	268.97	
8.420	67.9479	32.9987	316.89	
8.422	68.9871	37.6100	284.50	
8.424	67.7761	39.6955	254.61	
8.426	68.6140	36.3526	292.52	
8.428	68.4107	34.9636	303.27	
8.430	68.0636	31.5535	331.04	
8.432	68.4848	31.4996	335.35	
8.434	68.6426	38.4813	273.48	
8.436	68.0024	33.3054	314.60	
8.438	68.4216	34.4271	308.23	
8.440	68.7778	37.7119	281.68	
8.442	67.7691	31.4125	329.65	
8.444	68.9002	32.9358	326.09	
8.446	67.7399	32.4179	320.27	
8.448	68.5859	31.0621	340.23	
8.450	68.6631	35.6523	299.31	
8.452	67.9255	35.0953	297.68	
8.454	68.5865	32.4787	327.39	
8.456	68.4922	37.9312	277.10	
8.458	68.1103	34.8121	301.92	
8.460	68.2745	34.9132	302.49	
8.462	68.7930	36.8629	289.51	
8.464	67.8043	31.3314	330.70	
8.466	69.0663	38.6904	275.42	
8.468	67.8292	41.7006	236.91	
8.470	68.4442	38.0649	275.45	
8.472	68.6342	39.3285	265.72	
8.474	68.0665	38.4635	268.41	
8.476	68.6586	35.7518	298.37	
8.478	68.3614	38.4604	271.12	
8.480	68.2839	34.9977	301.81	
8.482	68.3800	36.5319	288.77	
8.484	68.8453	40.1107	260.54	
8.486	67.9895	37.0799	280.26	
8.488	68.8517	39.1132	269.64	
8.490	67.8998	37.3874	276.66	
8.492	68.5132	34.8430	305.29	heating
8.494	68.4650	33.9673	312.80	
8.496	68.3864	33.4060	317.17	
8.498	68.7527	37.0234	287.69	
8.500	68.2389	40.0114	255.94	
8.502	68.1661	35.5796	295.47	
8.504	68.4751	35.8846	295.50	
8.506	68.4349	35.3460	300.02	
8.508	68.4686	35.6528	297.55	
8.510	68.6245	38.2691	275.24	
8.512	67.8704	35.0627	297.47	
8.514	68.7529	38.3153	275.98	
8.516	68.2670	37.8714	275.60	
8.518	68.5037	33.4263	318.05	heating
8.520	68.8743	36.7174	291.57	
8.522	67.9821	36.2970	287.29	
8.524	68.4098	34.4799	307.65	
8.526	68.5149	38.7539	269.85	
8.528	68.2680	38.2871	271.84	
8.530	68.6603	41.1085	249.82	heating
8.532	68.4552	40.2803	255.47	heating
8.534	68.0154	31.0729	334.96	heating
8.536	69.0441	32.8110	328.53	heating
8.538	68.1219	31.9827	327.68	
8.540	68.7458	30.8044	344.02	
8.542	69.0541	36.2886	297.09	
8.544	67.9741	33.7377	310.43	
8.546	68.8518	33.6773	318.93	
8.548	68.4667	37.5077	280.71	
8.550	68.4447	32.8264	322.96	
8.552	68.7743	35.0751	305.56	
8.554	68.4779	36.2974	291.79	
8.556	68.2517	30.8190	339.41	
8.558	69.0794	36.3206	297.03	
8.560	68.0051	33.2447	315.18	
8.562	68.5954	31.1287	339.72	
8.564	69.1447	36.6841	294.33	
8.566	67.7813	31.9965	324.47	
8.568	68.9054	32.2052	332.77	
8.570	68.3709	36.8374	285.92	
8.572	68.5835	34.6383	307.79	
8.574	68.7888	39.3496	266.93	
8.576	68.4444	40.9380	249.40	
8.578	68.3307	34.6900	305.02	
8.580	69.0643	40.6116	257.99	
8.582	67.9349	36.9827	280.65	
8.584	68.6881	34.6253	308.85	
8.586	69.3916	39.8882	267.51	
8.588	67.9643	36.7125	283.36	
8.590	69.0912	39.4806	268.48	
8.592	68.2851	40.5526	251.45	
8.594	68.5873	34.4137	309.86	
8.596	68.7097	36.3626	293.30	
8.598	68.5125	38.0141	276.53	
8.600	68.5797	34.9727	304.72	
8.602	68.9264	42.3078	241.35	
8.604	68.1462	41.7952	238.93	
8.606	68.7141	39.8236	261.95	heating
8.608	69.2151	37.4210	288.28	heating
8.610	68.1855	30.3329	343.22	heating
8.612	69.1588	31.5523	340.98	heating
8.614	68.4775	34.5890	307.27	
8.616	68.9560	35.7128	301.42	
8.618	68.5598	37.4889	281.72	
8.620	68.8096	38.7668	272.40	
8.622	68.8235	36.0493	297.17	
8.624	68.6553	36.2625	293.71	
8.626	68.3841	33.1538	319.44	
8.628	68.9402	35.4415	303.74	
8.630	69.1514	35.8588	301.87	
8.632	68.5403	35.3725	300.74	
8.634	69.0542	38.6351	275.81	
8.636	68.3124	36.7438	286.24	
8.638	69.0831	35.3405	305.95	
8.640	68.3027	33.5004	315.56	
8.642	68.8893	33.7802	318.34	
8.644	68.8221	36.7584	290.73	
8.646	68.4208	37.3163	282.03	
8.648	68.5444	36.4672	290.85	
8.650	68.7496	38.6320	273.08	
8.652	68.7742	33.7793	317.30	
8.654	68.8479	34.9869	307.02	
8.656	68.8787	35.9129	298.91	
8.658	68.3027	30.9995	338.23	
8.660	69.3814	34.2911	318.17	
8.662	68.2095	32.0914	327.49	
8.664	69.0778	32.5482	331.22	
8.666	68.8283	36.3634	294.36	
8.668	68.2457	34.4027	306.86	
8.670	68.8684	34.1281	315.00	
8.672	68.4733	35.8608	295.70	

8.674	68.7670	32.1498	332.01	
8.676	68.8468	35.9951	297.87	
8.678	68.7902	36.7939	290.11	
8.680	68.3651	30.9885	338.90	
8.682	69.3201	34.0000	320.25	
8.684	68.2307	29.6651	349.68	
8.686	68.9314	29.7261	355.48	
8.688	68.9415	34.9480	308.22	
8.690	68.0251	33.0853	316.80	
8.692	68.9968	36.5133	294.53	
8.694	68.3678	41.7717	241.15	
8.696	68.6064	36.5327	290.82	
8.698	68.8116	35.3219	303.66	
8.700	68.4798	30.9527	340.26	
8.702	68.4934	24.0580	402.90	
8.704	69.1297	30.6044	349.31	cooling
8.706	68.1622	29.8664	347.23	cooling
8.708	68.8011	32.7964	326.46	cooling
8.710	68.8711	37.8315	281.44	cooling
8.712	67.9123	32.1083	324.64	cooling
8.714	68.9249	32.0835	334.05	cooling
8.716	68.0207	32.2194	324.62	cooling
8.718	68.4998	31.9114	331.75	cooling
8.720	68.4789	37.8423	277.79	cooling
8.722	68.1250	38.9966	264.11	cooling
8.724	68.4237	38.3883	272.33	cooling
8.726	68.3092	40.1297	255.51	cooling
8.728	67.9937	33.7689	310.32	
8.730	68.4489	33.6981	315.09	
8.732	68.3911	35.1359	301.53	
8.734	67.9230	34.5209	302.86	
8.736	68.6788	39.6083	263.59	
8.738	67.9655	40.6514	247.66	
8.740	68.4709	41.9186	240.75	
8.742	68.1060	42.8308	229.17	
8.744	68.1769	39.2398	262.38	
8.746	68.5271	38.1585	275.36	
8.748	68.0506	39.1389	262.15	
8.750	68.1754	39.3795	261.10	
8.752	68.4078	42.8611	231.64	
8.754	68.1440	39.4216	260.43	heating
8.756	68.2476	35.2423	299.26	heating
8.758	68.4411	34.1770	310.68	heating
8.760	68.2711	32.0563	328.37	heating
8.762	68.6050	32.8434	324.26	
8.764	67.9943	31.4920	330.97	
8.766	68.7157	32.9191	324.57	
8.768	68.4316	37.1648	283.50	
8.770	68.2625	36.2226	290.51	
8.772	68.4193	35.1265	301.87	
8.774	68.4770	33.7758	314.64	
8.776	68.0006	26.6289	375.12	
8.778	68.8340	29.5939	355.80	
8.780	68.1978	28.8434	356.83	
8.782	68.3880	30.4727	343.78	
8.784	68.7564	36.9234	288.63	
8.786	67.7320	33.0760	314.23	
8.788	69.0148	32.6882	329.38	
8.790	68.2682	34.5835	305.42	
8.792	68.2721	29.9280	347.67	
8.794	68.5114	31.1157	339.07	
8.796	68.2120	33.5049	314.69	cooling
8.798	68.0467	31.3191	333.01	cooling
8.800	68.7092	37.8488	279.82	cooling
8.802	68.2208	35.7464	294.45	cooling
8.804	68.0442	31.0579	335.36	
8.806	68.5983	33.2826	320.21	cooling
8.808	67.5214	29.1408	348.00	cooling
8.810	68.6844	29.7170	353.32	cooling
8.812	67.9792	34.9668	299.33	cooling
8.814	67.9019	36.0029	289.23	cooling
8.816	68.0860	38.5004	268.26	cooling
8.818	67.7269	38.1993	267.73	cooling
8.820	67.6968	30.2436	339.59	
8.822	68.0383	30.7050	338.51	cooling
8.824	68.0517	31.6022	330.49	cooling
8.826	67.5733	33.2973	310.79	cooling
8.828	68.0020	38.1647	270.54	cooling
8.830	67.1206	35.1579	289.81	cooling
8.832	68.1439	34.9535	300.94	
8.834	67.6919	35.8082	289.09	
8.836	67.4887	33.5049	308.14	
8.838	67.7923	34.0423	306.02	
8.840	67.4948	35.8332	287.08	
8.842	67.7242	34.5420	300.87	
8.844	67.6246	37.4606	273.50	
8.846	68.1161	38.7280	266.47	
8.848	67.5121	37.0808	275.92	

8.850	67.8820	36.0250	288.85	
8.852	67.1967	30.9682	328.49	
8.854	68.1208	31.8253	329.10	
8.856	67.8011	32.2485	322.36	
8.858	67.7497	34.6864	299.79	
8.860	67.7738	34.2508	303.96	
8.862	67.5516	33.3416	310.19	
8.864	67.9744	33.2290	315.04	
8.866	67.3465	32.3781	317.06	
8.868	68.3329	34.3520	308.11	
8.870	67.4549	33.6521	306.50	
8.872	67.7022	32.1708	322.17	
8.874	67.5334	32.4610	318.01	
8.876	67.8747	33.8481	308.52	
8.878	67.7278	31.2277	330.95	
8.880	67.8272	33.3994	312.16	
8.882	67.6324	31.9462	323.57	
8.884	67.7381	33.2314	312.88	
8.886	67.6813	34.6703	299.32	cooling
8.888	67.3488	33.1876	309.74	
8.890	68.3647	35.6020	297.06	
8.892	67.3783	35.4442	289.55	
8.894	67.7527	34.0808	305.31	
8.896	67.5924	36.5861	281.14	
8.898	67.7831	37.9766	270.26	
8.900	67.6319	35.3972	292.28	
8.902	67.9790	36.0866	289.17	
8.904	67.4699	30.0567	339.23	
8.906	67.8012	31.1294	332.51	
8.908	67.7608	35.2482	294.80	
8.910	67.3280	33.7113	304.81	
8.912	68.2777	34.3367	307.75	
8.914	67.3724	36.7166	277.96	
8.916	67.8998	36.2727	286.77	
8.918	67.5984	38.0366	268.04	
8.920	67.8013	37.7937	272.08	
8.922	67.4548	32.5396	316.58	
8.924	68.1404	33.1581	317.19	
8.926	67.6042	28.8002	351.84	
8.928	67.7579	29.3120	348.59	
8.930	67.8802	33.6522	310.35	
8.932	67.3139	32.0293	319.93	
8.934	68.0961	30.6387	339.63	
8.936	67.5841	33.1508	312.21	
8.938	67.7708	33.0186	315.10	
8.940	67.7017	36.1311	286.25	
8.942	67.7908	39.4044	257.38	
8.944	67.3062	35.2180	290.95	
8.946	68.3174	39.8433	258.18	
8.948	67.6780	38.6370	263.32	
8.950	67.5103	34.1673	302.33	
8.952	68.0833	36.9238	282.53	
8.954	67.0901	33.1561	307.68	
8.956	67.8595	28.9429	352.86	
8.958	67.8765	33.7678	309.27	
8.960	67.4700	33.1866	310.85	
8.962	67.7630	34.0744	305.46	
8.964	67.7849	38.0007	270.06	
8.966	67.2472	32.3480	316.44	
8.968	68.0883	34.1301	307.90	
8.970	67.6959	35.9878	287.50	
8.972	67.4320	31.8528	322.60	
8.974	68.0423	35.6036	294.13	
8.976	67.1092	33.3104	306.46	
8.978	67.5976	28.1368	357.80	
8.980	67.8304	31.8061	326.64	
8.982	67.3880	31.2743	327.45	
8.984	67.7149	30.6670	335.92	
8.986	67.7221	34.7717	298.77	
8.988	67.3574	33.0522	311.05	
8.990	67.8586	35.2871	295.33	
8.992	67.9963	38.9200	263.64	
8.994	67.4337	34.7935	295.95	
8.996	67.9136	35.3960	294.84	
8.998	67.4777	34.9578	294.86	
9.000	67.5965	31.2792	329.29	
9.002	67.9501	34.0263	307.59	
9.004	67.6101	37.7499	270.75	
9.006	67.6197	36.7115	280.25	
9.008	67.6158	37.5786	272.35	
9.010	67.4559	35.5380	289.41	
9.012	67.6736	33.9543	305.74	
9.014	67.9071	34.4156	303.67	
9.016	67.6569	35.1302	294.92	
9.018	67.6475	34.5532	300.07	
9.020	67.5562	34.3597	301.00	
9.022	67.9188	34.9639	298.81	
9.024	67.4713	30.6316	334.03	

9.026	67.9970	30.6518	338.61	
9.028	67.6192	30.6021	335.64	
9.030	67.4643	29.1667	347.25	
9.032	67.7276	31.4352	329.07	
9.034	67.3670	33.2957	308.93	
9.036	67.8477	33.4649	311.75	
9.038	67.7370	36.5248	283.01	
9.040	67.4166	32.5646	316.01	
9.042	67.5400	31.8907	323.24	
9.044	68.0579	36.4027	287.02	
9.046	67.0372	31.0446	326.35	
9.048	68.3130	33.9141	311.90	
9.050	67.4622	36.4772	280.95	
9.052	67.4734	33.8485	304.88	
9.054	67.8694	36.4660	284.74	
9.056	67.2063	35.2317	289.92	
9.058	67.8161	32.3057	321.98	
9.060	68.0149	39.5567	258.03	
9.062	67.3004	36.1353	282.58	
9.064	67.6381	35.5140	291.27	
9.066	68.1461	41.6894	239.89	
9.068	66.9741	35.1047	288.96	
9.070	68.3725	36.3638	290.23	
9.072	67.4119	37.7194	269.23	
9.074	67.5466	32.0541	321.82	
9.076	67.8698	32.8453	317.57	
9.078	67.2559	30.5955	332.40	
9.080	67.7235	26.9438	369.75	
9.082	68.1202	35.2718	297.84	
9.084	67.3694	33.7091	305.20	
9.086	67.5854	33.0194	313.42	
9.088	68.1845	38.9038	265.49	
9.090	67.0707	32.9398	309.47	
9.092	68.2475	32.2937	326.00	
9.094	67.6244	33.7435	307.20	
9.096	67.4394	29.8346	340.97	
9.098	67.8156	32.5724	319.56	
9.100	67.3796	33.8021	304.45	
9.102	67.6578	29.0309	350.24	
9.104	68.1051	33.0710	317.66	
9.106	67.3660	30.1627	337.33	
9.108	67.4469	27.0814	366.00	cooling
9.110	67.9661	32.7045	319.72	cooling
9.112	66.9287	31.7458	319.01	cooling
9.114	67.8508	33.3736	312.61	cooling
9.116	67.5998	37.0056	277.40	cooling
9.118	67.2656	35.4184	288.76	cooling
9.120	67.4585	36.5612	280.15	cooling
9.122	67.2637	36.3845	279.99	
9.124	67.4410	30.5141	334.82	
9.126	67.8558	33.5317	311.22	
9.128	67.2373	33.5395	305.54	
9.130	67.0897	29.0598	344.82	
9.132	67.7469	34.3387	302.92	cooling
9.134	66.9301	36.2016	278.62	cooling
9.136	67.4092	36.5263	280.02	
9.138	67.6738	39.2917	257.34	
9.140	67.1267	39.5246	250.27	
9.142	67.4445	40.8094	241.50	
9.144	67.1144	40.5631	240.74	
9.146	67.4945	38.1225	266.32	
9.148	67.2138	37.1140	272.92	
9.150	67.5998	39.5433	254.39	
9.152	67.0463	36.8699	273.61	
9.154	67.1634	33.7641	302.84	
9.156	67.3039	34.5846	296.67	
9.158	67.1196	34.4236	296.46	
9.160	67.5646	33.2100	311.50	
9.162	67.3506	37.6680	269.14	
9.164	67.1422	37.7213	266.76	
9.166	67.1228	36.3812	278.74	
9.168	67.6494	36.6963	280.66	
9.170	66.7383	30.0442	332.71	
9.172	67.8836	32.1264	324.22	
9.174	67.1420	33.5517	304.57	
9.176	66.9271	29.2921	341.24	
9.178	67.5313	32.1241	321.04	
9.180	67.0697	32.5452	313.04	
9.182	67.5199	25.7954	378.32	
9.184	67.6713	31.7695	325.53	
9.186	66.9683	29.5644	339.15	
9.188	67.1604	28.4672	350.84	
9.190	68.0035	36.5000	285.65	
9.192	66.3425	30.1169	328.46	
9.194	68.1797	34.2525	307.62	
9.196	66.9693	36.2437	278.59	
9.198	66.7567	26.7989	362.30	
9.200	67.6998	30.0768	341.13	
9.202	66.9691	32.5772	311.84	
9.204	67.3937	26.5222	370.59	
9.206	67.8651	35.3808	294.54	cooling
9.208	66.8453	33.4161	303.11	cooling
9.210	67.2430	32.9026	311.37	cooling
9.212	67.7731	39.1366	259.65	cooling
9.214	66.1192	29.9189	328.23	
9.216	68.0666	33.9773	309.09	cooling
9.218	66.6817	37.7192	262.61	cooling
9.220	66.7916	30.1851	331.92	
9.222	67.3608	33.5701	306.39	
9.224	66.8290	36.1704	277.99	
9.226	67.0372	30.5809	330.55	
9.228	67.5451	36.9615	277.31	
9.230	66.6673	33.9431	296.72	
9.232	66.9814	33.8623	300.30	
9.234	67.4595	39.8792	250.07	
9.236	66.1709	33.6625	294.76	
9.238	67.7285	35.6493	290.87	
9.240	66.7435	38.1936	258.87	
9.242	67.0127	33.3663	305.08	
9.244	67.1795	33.3669	306.58	
9.246	66.9063	34.0603	297.82	
9.248	67.1558	32.4643	314.55	
9.250	67.1870	35.0902	291.03	
9.252	66.8300	30.7329	327.30	
9.254	67.1054	32.1677	316.79	
9.256	67.1967	36.1752	281.28	
9.258	66.6120	34.2759	293.20	
9.260	67.3409	36.3476	281.02	
9.262	66.7918	37.0457	269.71	
9.264	67.2657	36.9756	274.64	
9.266	67.0045	36.7650	274.19	
9.268	66.8327	35.0892	287.82	
9.270	67.2338	34.7670	294.38	
9.272	66.7861	32.6915	309.14	
9.274	67.1454	28.7431	348.20	
9.276	67.0158	31.8263	319.07	
9.278	66.9738	33.3097	305.24	
9.280	66.8735	34.7033	291.69	
9.282	67.2497	37.4249	270.43	
9.284	66.6942	34.0542	295.95	
9.286	67.4932	37.7924	269.30	
9.288	66.7534	35.6652	281.88	
9.290	66.9226	33.3226	304.66	
9.292	67.4000	36.6721	278.61	
9.294	66.3324	32.9546	302.64	
9.296	67.5743	32.7048	316.17	
9.298	66.9116	36.4158	276.51	
9.300	66.8959	34.2485	296.02	
9.302	66.9813	34.0726	298.39	
9.304	67.0525	34.3140	296.84	
9.306	66.6663	27.7555	352.81	
9.308	67.5663	34.5331	299.52	
9.310	66.6516	34.8331	288.50	
9.312	66.7359	31.5738	318.82	
9.314	67.5441	36.7324	279.37	
9.316	66.2021	32.1088	309.13	
9.318	67.6169	29.9024	341.96	
9.320	66.9652	34.7308	292.27	
9.322	66.7478	31.1398	322.86	
9.324	67.1935	33.9812	301.14	
9.326	67.0041	36.1899	279.40	
9.328	66.6473	27.3377	356.43	
9.330	67.6976	33.7348	307.95	
9.332	66.5798	30.4704	327.41	
9.334	66.7544	25.3316	375.59	
9.336	67.6641	33.1201	313.22	
9.338	66.1598	32.0492	309.29	
9.340	67.7881	33.6596	309.45	
9.342	66.8252	36.6443	273.65	
9.344	66.7674	30.0562	332.87	
9.346	67.2155	30.5379	332.56	
9.348	67.0724	32.7887	310.86	
9.350	66.8125	25.7440	372.37	
9.352	67.6496	34.4811	300.74	cooling
9.354	66.7371	35.5606	282.68	cooling
9.356	66.6642	32.5089	309.69	cooling
9.358	67.4204	36.5068	280.30	cooling
9.360	66.0696	30.9494	318.44	
9.362	67.4820	30.1341	338.64	
9.364	66.7187	33.2212	303.73	cooling
9.366	66.6923	32.1907	312.83	cooling
9.368	66.8928	37.0932	270.20	cooling
9.370	66.5797	40.7672	234.05	
9.372	66.4211	34.3889	290.44	
9.374	66.9521	36.7738	273.63	
9.376	66.6238	36.0050	277.62	

9.378	66.4803	34.3544	291.29	
9.380	67.0682	37.0432	272.24	
9.382	66.0763	34.7113	284.39	
9.384	67.2063	36.4769	278.63	
9.386	66.5229	36.4755	272.44	
9.388	66.8136	34.1148	296.48	
9.390	66.6559	33.4867	300.75	
9.392	66.5082	33.6330	298.08	
9.394	66.6277	31.7519	316.22	
9.396	66.5708	33.8844	296.37	
9.398	66.8061	34.8504	289.75	
9.400	66.5010	34.0662	294.09	
9.402	66.7765	32.2796	312.79	
9.404	66.2858	29.6257	332.40	
9.406	67.0180	30.8205	328.21	
9.408	66.5207	28.4085	345.57	
9.410	67.0525	30.1787	334.34	
9.412	66.5520	31.2785	319.83	
9.414	66.5081	31.4324	318.04	
9.416	66.7836	32.5750	310.18	
9.418	66.2198	30.9949	319.39	
9.420	67.0486	30.3902	332.39	
9.422	66.6079	33.7445	297.98	
9.424	66.5899	32.4682	309.39	
9.426	66.5589	34.5839	289.92	
9.428	67.0600	39.7843	247.31	
9.430	66.3655	34.7956	286.25	
9.432	67.1859	35.8806	283.85	
9.434	66.4984	35.4823	281.23	
9.436	66.5222	34.0975	294.00	
9.438	66.9092	37.0336	270.89	
9.440	66.1087	34.4974	286.62	
9.442	67.1264	33.4070	305.74	
9.444	66.8754	39.9409	244.22	
9.446	66.3467	36.2369	273.01	
9.448	66.6910	35.9120	279.08	
9.450	66.9858	39.0262	253.51	
9.452	66.2597	30.3284	325.79	
9.454	67.4427	34.7543	296.39	
9.456	66.3668	35.2735	281.93	
9.458	66.5514	32.9547	304.63	
9.460	67.1394	38.4634	260.01	
9.462	65.8845	33.8665	290.31	
9.464	67.1986	31.8173	320.81	
9.466	66.8313	38.6465	255.56	
9.468	66.2861	32.4341	306.94	
9.470	66.7745	31.3490	321.21	
9.472	67.0402	35.9323	282.06	
9.474	66.0350	26.8531	355.27	
9.476	67.4414	29.7987	341.31	
9.478	66.3773	29.6948	332.61	
9.480	66.4562	26.4994	362.29	
9.482	67.1655	32.8893	310.79	
9.484	66.0203	32.2044	306.61	
9.486	67.1093	31.3482	324.25	
9.488	66.8088	38.0100	261.12	
9.490	66.5783	31.8193	315.16	
9.492	66.7210	28.5154	346.42	
9.494	67.1261	33.4499	305.35	
9.496	66.1404	28.7577	338.95	
9.498	67.4317	32.6699	315.19	
9.500	66.4997	33.6922	297.47	
9.502	66.5727	31.8807	314.56	
9.504	67.0295	33.9990	299.49	
9.506	66.2047	30.8564	320.51	
9.508	66.9442	26.7931	364.06	
9.510	66.7813	32.1478	314.03	
9.512	66.9205	33.6438	301.73	
9.514	66.5975	35.5423	281.58	
9.516	67.0149	41.3157	233.02	
9.518	66.3346	38.7768	249.87	
9.520	67.0504	38.9269	255.00	
9.522	66.5477	34.4388	291.14	
9.524	66.6846	31.6022	318.10	
9.526	67.0700	33.0509	308.46	
9.528	66.2346	32.5317	305.59	
9.530	67.0290	33.4231	304.71	
9.532	66.6593	36.4163	274.22	
9.534	67.0137	37.1087	271.15	
9.536	66.3925	34.6352	287.95	
9.538	66.7550	34.1030	296.06	
9.540	66.4943	33.4589	299.54	
9.542	66.6545	36.5946	272.56	
9.544	66.8340	37.3391	267.43	
9.546	66.6064	37.5291	263.65	
9.548	66.8194	35.9663	279.75	
9.550	66.2488	33.4411	297.47	
9.552	66.9215	32.0578	316.11	
9.554	66.4864	29.6563	333.94	
9.556	67.0563	32.0493	317.41	
9.558	66.4345	33.0626	302.59	
9.560	66.6298	33.0871	304.14	
9.562	66.6556	33.7840	298.05	
9.564	66.4809	32.4503	308.56	
9.566	66.9358	28.3608	349.77	
9.568	66.8768	30.0872	333.58	
9.570	66.6029	29.6678	334.90	
9.572	66.4672	30.3248	327.71	
9.574	67.0354	33.7475	301.83	
9.576	66.4163	31.2141	319.18	
9.578	67.1057	33.6471	303.37	
9.580	66.2832	31.9386	311.41	
9.582	66.5866	28.2562	347.55	
9.584	66.8932	30.9580	325.83	
9.586	66.3086	29.7313	331.65	
9.588	66.9511	27.4246	358.39	
9.590	66.6786	31.9774	314.64	cooling
9.592	66.3895	33.1481	301.41	cooling
9.594	66.3612	34.8513	285.70	cooling
9.596	66.3918	35.0573	284.11	cooling
9.598	66.0674	29.9930	327.09	
9.600	66.7179	33.1696	304.19	cooling
9.602	66.0908	33.2292	297.96	
9.604	66.1527	32.0642	309.08	
9.606	66.5519	35.8009	278.82	
9.608	66.0085	35.3855	277.66	
9.610	66.5991	32.7507	306.91	
9.612	66.3125	33.8507	294.34	
9.614	66.3458	33.2903	299.72	
9.616	66.3043	34.6213	287.27	
9.618	66.3880	34.8727	285.75	
9.620	66.1482	30.9412	319.23	
9.622	66.7787	35.0403	287.78	
9.624	66.0508	34.5618	285.52	
9.626	66.3813	34.4910	289.15	
9.628	66.4575	36.2394	273.99	
9.630	66.0662	35.4955	277.19	
9.632	66.7186	36.4193	274.73	
9.634	66.1485	37.3759	260.89	
9.636	66.6041	35.8823	278.56	
9.638	66.1710	35.9756	273.79	
9.640	66.5081	34.9732	285.93	
9.642	66.1112	31.7816	311.27	
9.644	66.6709	34.0948	295.37	
9.646	66.2069	34.5051	287.45	
9.648	66.5141	36.8171	269.27	
9.650	66.4071	36.2529	273.41	
9.652	66.2430	34.9008	284.18	
9.654	66.6752	32.9785	305.53	
9.656	66.2068	30.6656	322.26	
9.658	66.7127	29.9768	333.09	
9.660	66.1655	32.4048	306.11	
9.662	66.5841	34.1274	294.29	
9.664	66.0698	32.8383	301.32	
9.666	66.6506	33.5610	300.03	
9.668	66.4458	31.2097	319.49	
9.670	66.7314	30.0430	332.66	
9.672	66.3050	26.2155	363.50	
9.674	66.3124	26.5163	360.84	
9.676	66.5901	28.6207	344.27	
9.678	66.1610	28.3877	342.50	
9.680	66.7935	30.5274	328.83	
9.682	66.1147	31.3937	314.82	
9.684	66.4535	29.1241	338.47	
9.686	66.0117	27.0631	353.15	cooling
9.688	66.4051	27.8671	349.43	cooling
9.690	66.4452	29.3405	336.43	cooling
9.692	66.5361	34.5972	289.59	cooling
9.694	65.9925	32.5951	302.82	cooling
9.696	66.0416	32.3876	305.15	cooling
9.698	66.4415	34.3186	291.26	cooling
9.700	65.6993	30.8725	315.78	cooling
9.702	66.4328	32.5677	307.06	cooling
9.704	65.7487	34.2933	285.21	cooling
9.706	65.9200	35.5460	275.41	cooling
9.708	65.7424	36.8131	262.31	cooling
9.710	65.7274	36.1297	268.37	
9.712	66.3777	36.9243	267.06	
9.714	65.8806	40.6758	228.54	
9.716	65.8255	39.0212	243.04	
9.718	65.7342	38.8139	244.09	
9.720	66.1679	40.7099	230.83	
9.722	65.5807	38.7958	242.86	
9.724	66.1588	38.7658	248.38	
9.726	65.7236	35.9403	270.05	heating
9.728	66.1032	35.9356	273.53	heating

9.730	65.8985	34.8198	281.79	heating
9.732	65.9803	32.8536	300.37	heating
9.734	66.7407	34.1781	295.25	heating
9.736	65.9994	36.8960	263.88	heating
9.738	66.3842	36.7849	268.38	heating
9.740	66.1590	37.3108	261.57	heating
9.742	66.5099	35.4150	281.94	heating
9.744	66.2266	31.8757	311.46	heating
9.746	66.7559	33.0422	305.69	heating
9.748	66.2527	29.8648	329.93	heating
9.750	66.7831	32.7320	308.75	heating
9.752	66.3742	33.9926	293.61	heating
9.754	66.3828	30.7628	322.97	heating
9.756	67.1718	30.3355	334.00	
9.758	66.4084	29.4114	335.46	
9.760	66.8466	29.9969	334.12	
9.762	66.5373	32.3125	310.32	
9.764	66.5146	29.9881	331.19	
9.766	66.7365	31.8333	316.47	
9.768	66.5303	34.3622	291.67	
9.770	66.5337	31.9578	313.50	
9.772	66.7980	34.0364	297.05	
9.774	66.1344	31.7303	311.95	
9.776	66.6044	29.8856	332.94	
9.778	66.9520	32.3547	313.70	
9.780	66.7024	33.1305	304.40	
9.782	66.6688	36.0319	277.79	
9.784	66.5743	36.5263	272.45	
9.786	66.4501	32.3083	309.57	
9.788	66.9643	33.7731	300.95	
9.790	66.3548	33.3554	299.21	
9.792	66.7470	33.1644	304.50	
9.794	66.9079	37.0179	271.02	
9.796	66.1769	37.8022	257.28	
9.798	66.7153	38.6313	254.64	
9.800	66.7583	38.6923	254.48	
9.802	66.9560	34.7195	292.29	
9.804	66.6222	36.0410	277.28	
9.806	66.5064	35.6384	279.88	
9.808	66.6099	34.8237	288.21	
9.810	67.1845	39.2348	253.42	
9.812	66.1527	35.3973	278.86	
9.814	67.1188	36.4932	277.69	heating
9.816	66.6554	34.9133	287.81	heating
9.818	66.4480	30.6449	324.63	heating
9.820	66.9664	32.3251	314.10	
9.822	66.5157	33.1415	302.61	
9.824	67.1786	33.4139	306.15	
9.826	66.7763	39.5231	247.11	
9.828	66.5952	38.8971	251.14	
9.830	66.7687	37.5418	265.01	heating
9.832	67.1116	37.5609	267.94	heating
9.834	66.4511	31.0743	320.77	heating
9.836	67.5190	34.2441	301.71	heating
9.838	66.7222	34.8509	288.98	heating
9.840	66.9095	34.0154	298.26	heating
9.842	67.2322	37.7897	266.96	heating
9.844	66.8408	35.8494	281.00	heating
9.846	67.3263	32.8665	312.45	heating
9.848	67.2694	35.5785	287.35	heating
9.850	66.9437	32.1975	315.05	heating
9.852	67.2548	31.7943	321.53	heating
9.854	67.2471	33.4416	306.52	
9.856	67.0698	33.3870	305.41	
9.858	67.6324	37.5433	272.82	
9.860	67.0517	39.1099	253.35	
9.862	67.2318	37.5819	268.84	heating
9.864	67.4397	39.0110	257.77	heating
9.866	67.2022	37.3945	270.27	heating
9.868	67.4339	36.1632	283.54	heating
9.870	67.6600	39.3010	257.13	heating
9.872	67.2269	37.5113	269.44	heating
9.874	67.5775	36.6719	280.23	heating
9.876	67.4766	38.1227	266.16	heating
9.878	67.4769	36.7218	278.86	heating
9.880	67.7962	37.8571	271.46	heating
9.882	67.6239	39.1183	258.46	heating
9.884	67.4795	36.9666	276.67	heating
9.886	67.8971	41.2578	241.54	heating
9.888	67.6161	41.8057	234.03	heating
9.890	67.6957	39.5520	255.18	heating
9.892	68.1516	40.9454	246.68	heating
9.894	67.5731	37.7561	270.35	heating
9.896	68.0520	34.4008	305.12	heating
9.898	67.8343	33.8082	308.52	heating
9.900	67.8407	31.4901	329.60	heating
9.902	68.1228	32.1017	326.61	heating
9.904	68.1675	32.6926	321.66	heating
9.906	67.8400	30.0664	342.50	heating
9.908	68.3342	33.4122	316.64	heating
9.910	68.0570	34.9660	300.04	heating
9.912	68.1708	33.0359	318.57	heating
9.914	68.4361	33.5707	316.13	heating
9.916	68.2016	34.0953	309.25	heating
9.918	68.3888	33.1973	319.09	
9.920	68.3905	34.9370	303.33	
9.922	68.0322	32.5336	321.87	
9.924	68.4640	31.6218	334.05	
9.926	68.5414	31.9268	331.99	
9.928	68.0365	29.3308	350.95	
9.930	68.5451	31.6344	334.68	
9.932	68.2078	34.3949	306.59	
9.934	68.4250	33.5946	315.81	
9.936	68.2644	34.5875	305.35	
9.938	68.6394	36.2530	293.65	
9.940	68.1223	33.5977	313.04	
9.942	68.5858	35.4364	300.57	
9.944	68.0407	33.9987	308.66	
9.946	68.4460	32.3301	327.47	
9.948	68.6811	33.9288	315.10	
9.950	67.9730	32.4267	322.30	
9.952	68.4811	29.9066	349.76	
9.954	68.4027	33.9112	312.74	
9.956	68.2042	32.5139	323.61	
9.958	68.2735	33.7402	313.12	
9.960	68.8620	36.5858	292.65	
9.962	68.0254	33.2002	315.76	
9.964	68.7540	35.9377	297.55	
9.966	67.9252	34.6959	301.29	
9.968	68.5818	32.0204	331.51	
9.970	68.6067	32.8345	324.35	
9.972	68.0503	33.5275	313.02	
9.974	68.5941	34.9587	304.98	
9.976	68.5034	41.9305	240.94	
9.978	68.1335	40.2741	252.61	
9.980	68.2071	37.7592	276.08	
9.982	68.8802	39.6086	265.41	
9.984	68.0666	37.0755	281.00	
9.986	68.6088	38.3784	274.10	
9.988	68.1080	37.5569	277.01	
9.990	68.4437	34.4869	307.89	
9.992	68.5805	34.9553	304.88	
9.994	68.3195	36.8436	285.40	
9.996	68.4712	36.4517	290.33	
9.998	68.4130	38.1462	274.43	
10.000	68.2394	36.2386	290.16	
10.002	68.0597	33.4133	314.14	
10.004	68.7768	36.6318	291.46	
10.006	68.2573	37.8293	275.90	
10.008	68.2743	37.2138	281.63	
10.010	68.3102	36.9796	284.08	
10.012	68.3235	34.6976	304.89	
10.014	68.5243	32.7803	324.10	
10.016	68.3699	33.2672	318.28	
10.018	68.3545	30.6442	341.92	
10.020	68.3512	28.7000	359.52	
10.022	68.3943	29.9962	348.16	
10.024	68.0526	30.2284	342.96	
10.026	68.6830	33.8217	316.09	
10.028	68.5859	36.5680	290.31	
10.030	68.0621	34.1572	307.42	
10.032	68.5849	34.0155	313.45	
10.034	68.1703	32.1758	326.37	
10.036	68.5701	28.1986	366.05	
10.038	68.3003	28.7450	358.65	
10.040	68.2806	28.9831	356.32	cooling
10.042	68.0970	28.0933	362.72	cooling
10.044	68.3593	32.3639	326.38	cooling
10.046	67.6617	29.8569	342.78	cooling
10.048	68.2645	30.7827	339.85	cooling
10.050	68.5267	35.7280	297.39	cooling
10.052	67.5417	35.2328	292.95	cooling
10.054	68.1376	36.3648	288.09	cooling
10.056	67.8555	39.9021	253.46	cooling
10.058	67.9877	38.7795	264.83	cooling
10.060	67.8504	39.3426	258.48	cooling
10.062	67.9655	39.5268	257.86	
10.064	67.6454	34.4844	300.68	
10.066	68.1545	36.7032	285.17	
10.068	67.5490	35.8672	287.26	
10.070	67.9185	36.3344	286.38	
10.072	68.5433	41.5216	245.01	
10.074	67.3905	40.5744	243.15	
10.076	68.0810	37.4815	277.45	
10.078	67.8457	38.5083	266.01	
10.080	67.9143	35.1451	297.12	

10.082	67.8869	34.3246	304.31	
10.084	68.1879	36.6525	285.94	
10.086	67.6821	33.1475	313.13	
10.088	68.2512	37.9754	274.52	
10.090	67.5849	38.2086	266.36	
10.092	67.9568	36.4924	285.29	
10.094	68.2533	36.9767	283.59	
10.096	67.4637	33.3134	309.65	
10.098	68.0852	31.1488	334.91	
10.100	67.7911	33.7643	308.53	
10.102	67.8884	33.7665	309.39	
10.104	67.7687	33.3834	311.78	
10.106	68.2089	35.1409	299.83	
10.108	67.5946	31.6621	325.80	
10.110	67.9939	33.4884	312.87	
10.112	67.7698	35.7172	290.63	
10.114	67.7847	34.8782	298.37	
10.116	67.9749	35.5417	294.08	
10.118	67.6695	36.4970	282.65	
10.120	68.0498	36.7429	283.86	
10.122	67.8488	37.7908	272.54	
10.124	67.8289	36.3688	285.25	
10.126	67.8496	34.5872	301.60	
10.128	68.0472	32.4589	322.68	
10.130	67.8005	31.1313	332.48	
10.132	67.9414	32.3540	322.68	
10.134	67.8363	35.0163	297.58	
10.136	67.9171	35.7356	291.79	
10.138	67.6178	34.4823	300.44	
10.140	68.1779	37.7689	275.72	
10.142	67.7547	37.8051	271.56	
10.144	67.9605	38.0217	271.46	
10.146	67.8380	38.0695	269.91	
10.148	67.6363	35.0956	295.05	
10.150	68.0830	32.8305	319.64	
10.152	67.8504	34.4450	302.89	
10.154	67.9576	34.2946	305.23	
10.156	67.8016	33.3393	312.47	
10.158	68.0425	33.0032	317.71	
10.160	67.5716	30.3028	337.92	
10.162	68.2180	34.3822	306.79	
10.164	67.8607	36.4881	284.46	
10.166	67.7112	34.9501	297.05	
10.168	67.9920	34.7864	301.08	
10.170	67.4919	32.6984	315.48	
10.172	68.1007	31.2836	333.83	
10.174	67.8980	33.5709	311.25	
10.176	67.8589	34.3284	304.03	
10.178	67.7194	34.6587	299.77	
10.180	67.9861	36.9107	281.76	
10.182	67.5669	34.0925	303.52	
10.184	67.9214	35.1205	297.41	
10.186	67.9402	35.9140	290.39	
10.188	67.5238	32.9751	313.26	
10.190	68.0610	34.5984	303.41	
10.192	67.4716	36.7782	278.30	
10.194	68.1109	36.9922	282.16	
10.196	67.8275	37.7581	272.64	
10.198	67.8272	37.6661	273.48	
10.200	67.7714	37.5768	273.78	
10.202	68.0617	41.9548	236.72	
10.204	67.5557	39.8479	251.23	
10.206	67.8283	41.1105	242.25	
10.208	68.4113	44.6966	215.02	heating
10.210	67.6718	42.3803	229.32	heating
10.212	68.3324	40.7380	250.20	heating
10.214	67.6517	37.8541	270.18	heating
10.216	68.3215	33.9067	312.04	heating
10.218	68.1418	33.0408	318.27	heating
10.220	68.0791	34.5353	304.15	heating
10.222	68.1378	35.2873	297.86	heating
10.224	68.2608	39.9826	256.40	heating
10.226	67.8287	37.5644	274.41	heating
10.228	68.1541	36.8017	284.28	heating
10.230	68.8520	38.2227	277.72	heating
10.232	67.8885	35.0379	297.86	heating
10.234	68.6555	34.4315	310.31	heating
10.236	68.1386	34.6020	304.08	heating
10.238	68.7289	32.9764	324.17	heating
10.240	68.4303	32.1106	329.32	heating
10.242	68.7380	31.9292	333.75	heating
10.244	68.4277	26.5979	379.28	
10.246	68.9950	30.6598	347.59	
10.248	68.3470	30.8753	339.76	
10.250	68.5589	30.8915	341.54	
10.252	69.1919	34.2650	316.69	
10.254	68.3571	35.1710	300.90	
10.256	68.9337	35.9034	299.49	
10.258	68.3614	36.0191	293.25	
10.260	68.7459	33.5572	319.06	
10.262	68.3958	31.1873	337.38	
10.264	68.8840	31.0662	342.90	
10.266	68.4693	29.8919	349.79	
10.268	68.8930	35.7488	300.52	
10.270	68.4618	38.0498	275.75	
10.272	68.3506	35.6190	296.78	
10.274	69.1440	36.4748	296.22	
10.276	68.3495	38.0151	275.05	
10.278	68.8309	37.7946	281.41	
10.280	68.4165	39.5410	261.82	
10.282	68.6634	39.2157	267.01	
10.284	68.4429	38.0542	275.54	
10.286	68.9970	40.8910	254.84	
10.288	68.4396	38.1844	274.33	
10.290	68.7504	38.2044	276.97	
10.292	68.6500	38.3503	274.73	
10.294	68.3619	36.1288	292.26	
10.296	69.0570	36.5297	294.93	
10.298	68.6810	39.8334	261.57	
10.300	68.6606	36.7613	289.24	
10.302	68.5550	35.7718	297.25	
10.304	68.8371	36.0486	297.30	
10.306	68.3332	31.0405	338.14	
10.308	69.1476	32.4881	332.40	
10.310	68.3690	29.8919	348.88	
10.312	68.7330	30.1432	349.90	
10.314	68.8574	35.3643	303.69	
10.316	68.2043	33.6749	313.08	
10.318	68.8982	31.7384	336.93	
10.320	68.6809	34.2446	312.24	
10.322	68.4979	31.8258	332.51	
10.324	68.5949	32.3767	328.40	
10.326	68.7251	34.8644	307.02	
10.328	68.2081	30.2155	344.48	
10.330	69.1321	31.8942	337.64	
10.332	68.4495	30.7071	342.22	
10.334	68.5935	29.6154	353.42	cooling
10.336	68.8660	35.1848	305.39	cooling
10.338	68.1542	34.8825	301.68	cooling
10.340	68.6752	34.2225	312.39	cooling
10.342	68.5051	39.1497	266.17	cooling
10.344	68.2550	38.1680	272.80	
10.346	68.4528	37.6121	279.64	
10.348	68.3814	38.0933	274.63	
10.350	67.9504	33.5455	311.95	
10.352	68.7891	35.6523	300.46	
10.354	68.3351	34.5306	306.51	
10.356	68.2945	32.4824	324.71	
10.358	68.6515	37.5346	282.14	
10.360	68.0083	36.5454	285.28	
10.362	68.4501	32.9604	321.79	
10.364	68.3406	35.3037	299.55	
10.366	68.4250	35.7421	296.34	
10.368	68.4805	36.7883	287.36	
10.370	68.3509	38.3991	271.58	
10.372	67.9586	34.9402	299.38	
10.374	68.6918	35.7152	299.00	
10.376	68.4667	33.5546	316.55	
10.378	68.2698	31.2290	335.86	
10.380	68.6461	33.4385	319.23	
10.382	68.1030	32.6460	321.49	
10.384	68.5380	31.0885	339.56	
10.386	68.3066	33.9744	311.30	
10.388	68.5762	35.5716	299.26	
10.390	68.5591	35.3579	301.04	
10.392	68.4393	34.8839	304.25	
10.394	68.0083	31.2250	333.52	
10.396	68.7832	35.3021	303.58	
10.398	68.4862	37.1278	284.33	
10.400	68.3093	36.5852	287.65	
10.402	68.6054	37.5258	281.80	
10.404	68.0953	36.1124	289.99	
10.406	68.6150	34.3177	310.98	
10.408	67.9846	32.1743	324.70	
10.410	68.7209	33.1297	322.71	
10.412	68.4280	35.2092	301.20	
10.414	68.4049	37.3778	281.33	
10.416	68.1440	36.6381	285.67	
10.418	68.4626	37.9520	276.64	
10.420	68.3701	34.9108	303.38	
10.422	68.2505	33.0915	318.79	
10.424	68.5378	34.2832	310.59	
10.426	68.1552	38.1135	272.39	
10.428	68.5854	42.5201	236.34	
10.430	67.9342	41.7770	237.17	
10.432	68.8492	41.8366	244.93	

10.434	68.5606	41.4177	246.11	
10.436	68.4280	37.5420	280.05	
10.438	68.3388	34.1913	309.62	
10.440	68.4695	34.9129	304.26	
10.442	68.6521	35.1310	303.94	
10.444	68.5691	38.3411	274.08	
10.446	68.5561	38.3757	273.65	
10.448	68.2922	37.3020	280.99	
10.450	68.5412	37.5870	280.67	
10.452	67.9108	32.9549	316.95	
10.454	68.7967	33.9123	316.30	
10.456	68.5010	36.5658	289.56	
10.458	68.2741	37.5378	278.69	
10.460	68.4305	39.4537	262.74	
10.462	68.0397	37.9335	272.98	
10.464	68.6701	35.0676	304.68	heating
10.466	68.4016	33.5135	316.34	
10.468	68.3041	27.5202	369.79	
10.470	68.5593	28.9125	359.48	
10.472	68.6166	35.1868	303.11	
10.474	67.9839	32.4761	321.95	
10.476	68.7842	33.5225	319.72	
10.478	68.5579	31.2506	338.27	
10.480	68.4093	28.8647	358.56	
10.482	68.7492	33.6169	318.55	
10.484	67.9565	33.8247	309.48	
10.486	68.8500	34.7880	308.85	
10.488	68.3609	37.2854	281.77	
10.490	68.4397	34.8208	304.83	
10.492	68.6398	36.3714	292.58	
10.494	68.5556	39.0388	267.63	
10.496	67.9739	30.9710	335.51	
10.498	68.8953	30.7322	346.03	
10.500	68.4727	29.9489	349.30	
10.502	68.3389	30.7429	340.89	
10.504	68.8443	38.7420	272.94	
10.506	67.8395	37.6873	273.40	
10.508	68.6919	36.3364	293.37	
10.510	68.3454	36.9442	284.72	
10.512	68.4264	32.8574	322.51	
10.514	68.7957	35.9498	297.82	
10.516	68.4554	41.3139	246.10	
10.518	68.0613	37.5936	276.25	
10.520	68.9268	40.1799	260.65	
10.522	68.2808	37.0428	283.24	
10.524	68.4172	33.9559	312.47	
10.526	68.8168	36.8350	289.98	
10.528	67.8531	31.9866	325.21	
10.530	68.6461	29.9270	351.07	
10.532	68.3305	32.6294	323.71	
10.534	68.6841	31.0638	341.11	
10.536	68.8278	35.5154	302.05	
10.538	68.4320	38.5267	271.16	
10.540	68.1252	33.2655	316.08	
10.542	68.9670	34.9316	308.60	
10.544	68.2185	32.2626	326.02	
10.546	68.7331	34.3286	311.95	
10.548	68.6708	37.9318	278.72	
10.550	68.0515	34.3366	305.70	
10.552	68.6759	33.4843	319.09	
10.554	68.2017	32.6853	322.03	
10.556	68.9191	31.1385	342.56	
10.558	68.7473	35.8399	298.38	
10.560	68.3502	37.5677	279.11	
10.562	68.3132	36.9068	284.77	
10.564	68.6949	39.3403	266.16	
10.566	68.2378	37.1336	282.03	
10.568	68.7628	39.6794	263.70	
10.570	68.4633	38.9707	267.41	
10.572	68.3023	36.7033	286.51	
10.574	68.6088	39.2546	266.16	
10.576	68.0731	39.5509	258.61	
10.578	69.0108	41.3468	250.83	
10.580	68.4986	41.5287	244.54	
10.582	68.4042	36.4506	289.73	
10.584	68.5394	35.1436	302.80	
10.586	68.3798	34.4703	307.46	
10.588	68.2565	32.7147	322.26	
10.590	68.8762	39.7560	264.04	
10.592	68.2551	39.1074	264.29	
10.594	68.5295	37.9142	277.59	
10.596	68.5654	38.6612	271.15	
10.598	67.7947	31.1751	332.04	heating
10.600	69.2914	32.1977	336.33	
10.602	68.3119	33.1336	318.97	
10.604	68.6152	31.4548	336.94	
10.606	68.7678	37.1528	286.66	
10.608	68.1072	36.0987	290.23	
10.610	68.5122	32.9808	322.17	
10.612	68.7831	37.3310	285.18	
10.614	68.1769	31.0746	336.41	
10.616	68.6597	30.3459	347.40	
10.618	68.5415	34.5744	307.98	
10.620	67.9358	31.5797	329.65	
10.622	69.2643	37.4582	288.39	
10.624	68.2751	39.6273	259.75	
10.626	68.4645	34.7887	305.34	
10.628	68.8985	39.1018	270.17	
10.630	67.9765	36.6735	283.83	
10.632	68.4708	33.5254	316.85	
10.634	68.8991	40.4531	257.92	
10.636	68.2293	36.5820	286.95	
10.638	68.8692	37.7155	282.48	
10.640	68.4463	41.0342	248.55	
10.642	68.0136	35.3763	295.93	
10.644	69.1044	37.3083	288.30	
10.646	68.0965	33.4388	314.25	
10.648	68.4813	29.6074	352.47	
10.650	68.9487	39.1071	270.58	
10.652	68.0780	41.2980	242.82	
10.654	68.4772	39.6561	261.32	
10.656	68.8302	42.9224	234.91	
10.658	68.4583	37.6886	278.99	heating
10.660	68.9158	36.8009	291.19	heating
10.662	68.6790	40.4023	256.39	heating
10.664	68.3426	37.4560	280.05	heating
10.666	69.2108	38.7000	276.65	heating
10.668	68.5579	35.6859	298.05	heating
10.670	68.7574	31.7325	335.71	heating
10.672	69.1525	34.8550	310.98	heating
10.674	68.4711	33.8660	313.77	heating
10.676	68.8001	31.4988	338.22	
10.678	68.9646	33.8262	318.61	
10.680	69.1326	35.6149	303.91	
10.682	69.0256	38.2099	279.41	
10.684	68.8414	40.8920	253.42	
10.686	68.6639	37.6240	281.44	
10.688	68.9389	35.0480	307.29	
10.690	68.8610	33.2437	322.95	
10.692	68.8845	33.4745	321.07	
10.694	69.0619	35.4496	304.77	
10.696	68.7062	35.0695	304.99	
10.698	68.8632	33.0542	324.69	
10.700	68.6514	30.3095	347.65	
10.702	69.3545	32.7417	331.97	
10.704	68.6850	33.2442	321.35	
10.706	68.8246	35.1429	305.40	
10.708	68.9086	39.0512	270.72	
10.710	68.4797	38.9110	268.10	
10.712	69.1132	38.9470	273.52	
10.714	68.7311	40.1775	258.90	
10.716	68.9369	36.0616	298.09	
10.718	68.8984	36.8918	290.21	
10.720	68.8970	37.5594	284.14	
10.722	68.3899	32.3194	327.06	
10.724	69.5756	39.0790	276.52	
10.726	68.5800	41.0370	249.74	
10.728	68.7969	37.7839	281.20	
10.730	69.1260	40.3609	260.82	
10.732	68.2419	37.0831	282.52	
10.734	68.9404	35.0976	306.86	
10.736	69.1408	42.5254	241.33	
10.738	68.7331	39.4785	265.26	heating
10.740	69.2231	42.5754	241.62	heating
10.742	69.1285	44.9667	219.08	heating
10.744	68.4007	34.0236	311.70	heating
10.746	70.0217	37.2286	297.34	heating
10.748	68.7164	36.4115	292.91	heating
10.750	69.2133	31.1663	344.98	heating
10.752	69.7119	37.7844	289.49	heating
10.754	68.6268	36.8904	287.76	heating
10.756	69.2235	34.1820	317.73	heating
10.758	69.9926	41.5763	257.65	heating
10.760	69.0179	31.8688	336.84	heating
10.762	70.0064	31.9172	345.36	heating
10.764	69.7042	37.7151	290.05	
10.766	68.8051	29.4376	356.95	
10.768	70.4685	34.1404	329.39	
10.770	69.1269	37.7887	284.15	
10.772	69.5007	32.4683	335.78	
10.774	70.0361	38.4601	286.30	
10.776	69.0031	36.1748	297.66	
10.778	69.1681	30.6832	348.95	
10.780	70.2932	37.9492	293.27	
10.782	69.1841	31.9627	337.49	
10.784	69.9088	32.0061	343.67	

10.786	69.5414	37.0633	294.48	
10.788	68.8654	30.5936	347.02	
10.790	70.0935	33.6102	330.80	
10.792	69.4036	38.8468	277.06	
10.794	69.3989	32.5424	334.18	
10.796	70.0979	36.5750	303.96	
10.798	69.2342	36.9060	293.12	
10.800	69.1206	33.0442	327.11	
10.802	70.2470	40.2565	271.93	
10.804	69.3232	37.6790	286.92	
10.806	69.8115	34.7919	317.53	
10.808	69.6208	39.3101	274.83	
10.810	69.2382	36.9942	292.36	
10.812	69.6608	35.5713	309.09	
10.814	69.5480	37.0987	294.22	
10.816	69.3559	33.0307	329.37	
10.818	69.9674	34.4039	322.46	
10.820	69.5719	37.7857	288.21	
10.822	69.2098	36.7440	294.37	
10.824	69.7645	37.9884	288.12	
10.826	69.8039	38.6387	282.58	
10.828	69.4529	35.6580	306.42	
10.830	69.6286	36.4412	300.92	
10.832	69.5614	38.9358	277.69	
10.834	69.3634	35.7627	304.66	
10.836	69.5227	34.2678	319.66	
10.838	69.8953	37.9921	289.27	
10.840	69.4551	35.6130	306.85	
10.842	69.8881	38.6599	283.15	
10.844	69.3099	38.6771	277.75	
10.846	69.2837	33.5827	323.71	
10.848	70.3426	38.0587	292.72	
10.850	69.0147	36.3533	296.15	
10.852	69.6970	34.5309	318.86	
10.854	69.7958	41.5479	256.13	
10.856	69.0199	38.1569	279.84	
10.858	69.4945	35.9195	304.43	
10.860	70.1217	43.2220	243.90	
10.862	69.0930	35.6425	303.30	
10.864	70.0387	38.0318	290.21	
10.866	69.3545	39.4813	270.87	
10.868	68.9134	29.7033	355.52	
10.870	70.3913	33.5330	334.20	
10.872	68.8105	32.7506	326.96	
10.874	69.7983	28.5031	374.43	
10.876	70.0041	39.2270	279.06	
10.878	68.8492	36.0038	297.81	
10.880	69.2748	30.8912	348.03	
10.882	70.2909	39.4337	279.79	
10.884	68.7483	32.6575	327.24	
10.886	70.1482	36.5532	304.61	
10.888	69.3826	41.4800	253.00	
10.890	68.8556	30.6527	346.39	
10.892	70.1513	31.8688	347.11	
10.894	69.3029	33.1708	327.62	
10.896	69.6877	25.0766	404.50	
10.898	70.2076	34.7441	321.55	cooling
10.900	68.8394	32.2217	332.02	cooling
10.902	69.0915	27.0464	381.23	cooling
10.904	70.3176	38.3783	289.60	cooling
10.906	68.5733	35.8781	296.45	cooling
10.908	69.9124	37.5094	293.80	cooling
10.910	69.1378	42.6945	239.76	cooling
10.912	68.6219	35.0470	304.43	cooling
10.914	69.3620	35.1717	310.01	
10.916	69.3033	39.9275	266.35	
10.918	68.9790	31.9946	335.34	
10.920	69.8030	35.9028	307.38	
10.922	68.8391	34.9968	306.85	
10.924	68.8333	28.6759	364.11	
10.926	69.7428	33.9540	324.50	cooling
10.928	68.5112	32.8054	323.75	cooling
10.930	69.3126	32.0869	337.53	cooling
10.932	69.1975	39.3667	270.48	cooling
10.934	68.4924	36.8637	286.78	cooling
10.936	68.9044	36.5234	293.60	cooling
10.938	69.0867	39.9128	264.52	cooling
10.940	68.6919	34.7983	307.32	
10.942	69.3026	36.0320	301.67	
10.944	68.8411	38.4023	275.99	
10.946	68.5988	33.8209	315.34	
10.948	69.0687	34.3796	314.53	
10.950	68.9741	36.7852	291.86	
10.952	68.7393	33.1799	322.42	
10.954	69.3291	35.8424	303.63	
10.956	68.6273	36.3186	292.95	
10.958	68.6489	34.3319	311.16	
10.960	69.1900	38.9610	274.09	
10.962	68.8939	40.9634	253.25	
10.964	68.9218	37.1659	287.94	
10.966	69.2061	39.8523	266.15	
10.968	68.6110	36.9530	287.05	
10.970	68.7306	33.9610	315.26	
10.972	69.2387	38.6217	277.61	
10.974	68.3460	36.5136	288.63	
10.976	69.3927	36.8890	294.72	
10.978	68.8933	41.8353	245.34	
10.980	68.5353	37.5849	280.63	
10.982	68.8874	34.6992	309.99	
10.984	69.2378	35.0384	310.09	
10.986	68.6143	26.2960	383.71	
10.988	69.4730	30.8667	350.05	
10.990	68.7420	33.9888	315.11	
10.992	68.4255	30.1809	346.77	
10.994	69.2365	33.4865	324.15	
10.996	68.3773	31.6654	332.87	
10.998	69.3234	27.4330	379.83	
11.000	69.1816	35.5008	305.39	cooling
11.002	68.3908	34.0718	311.18	cooling
11.004	68.5408	30.5241	344.70	cooling
11.006	69.5653	36.2048	302.48	cooling
11.008	68.0869	29.2884	351.79	cooling
11.010	69.3757	32.1700	337.35	cooling
11.012	68.4969	34.0958	311.92	cooling
11.014	68.1778	25.9246	383.12	cooling
11.016	68.7319	26.2846	384.88	cooling
11.018	68.4590	31.2409	337.46	cooling
11.020	68.5406	29.1898	356.80	cooling
11.022	68.8035	37.8873	280.32	cooling
11.024	67.9852	38.5004	267.34	cooling
11.026	67.9353	34.5663	302.56	cooling
11.028	68.9993	38.3344	278.04	cooling
11.030	67.5117	32.3162	319.12	cooling
11.032	68.8033	32.1698	332.16	cooling
11.034	68.0773	37.8193	274.35	cooling
11.036	67.7316	35.5662	291.65	cooling
11.038	68.0349	36.0244	290.24	cooling
11.040	68.0912	37.9859	272.97	cooling
11.042	68.0422	30.3786	341.50	cooling
11.044	68.4716	34.3306	309.56	cooling
11.046	67.7683	34.5680	301.03	cooling
11.048	67.4666	30.6047	334.23	cooling
11.050	68.3794	34.9622	303.00	cooling
11.052	67.4884	36.4097	281.79	cooling
11.054	68.2784	38.9109	266.28	cooling
11.056	67.7060	43.0928	223.17	cooling
11.058	67.3850	38.2825	263.88	
11.060	67.5008	31.9364	322.47	
11.062	67.7780	30.7040	336.16	
11.064	67.8666	29.7695	345.43	
11.066	68.1028	34.9612	300.50	
11.068	67.6049	36.2495	284.30	
11.070	67.5186	34.6223	298.28	
11.072	67.8348	34.6538	300.86	
11.074	67.9703	35.2290	296.87	
11.076	68.0306	36.2014	288.60	
11.078	67.6049	35.1172	294.57	
11.080	67.7036	34.7993	298.35	
11.082	67.4704	34.0911	302.65	
11.084	67.7503	35.0284	296.69	
11.086	68.0337	38.7995	265.07	
11.088	67.6935	36.1177	286.30	
11.090	67.8953	34.2472	305.09	
11.092	67.6694	34.1079	304.31	
11.094	67.3544	29.2387	345.60	
11.096	68.2818	31.3768	334.62	
11.098	67.7207	33.4873	310.40	
11.100	67.6166	29.9216	341.79	
11.102	67.9615	33.8344	309.44	
11.104	67.3515	32.2398	318.36	
11.106	67.4999	27.6896	360.97	
11.108	68.3325	33.9992	311.30	cooling
11.110	67.0903	30.3709	332.94	cooling
11.112	67.9854	30.5796	339.16	cooling
11.114	67.5213	35.3705	291.52	cooling
11.116	66.8855	29.7511	336.70	cooling
11.118	67.9145	31.6198	329.09	cooling
11.120	67.4235	35.6451	288.14	cooling
11.122	67.2108	29.1277	345.31	cooling
11.124	67.7125	32.6108	318.27	cooling
11.126	66.7585	29.8299	334.84	cooling
11.128	66.9455	25.7426	373.59	cooling
11.130	67.9773	36.1782	288.33	cooling
11.132	66.4404	33.9437	294.65	cooling
11.134	67.5553	34.0369	303.92	cooling
11.136	67.0183	40.8994	236.82	cooling

11.138	66.3194	36.7439	268.17	cooling
11.140	67.3958	39.8437	249.82	cooling
11.142	67.0566	43.1454	216.81	
11.144	66.8186	32.5185	311.00	
11.146	67.5219	36.6128	280.26	
11.148	66.6070	36.1848	275.84	
11.150	66.6636	31.1605	321.91	
11.152	67.8151	37.9960	270.37	
11.154	66.2093	33.2329	299.00	
11.156	67.5354	31.2502	329.00	
11.158	67.0361	38.5251	258.51	
11.160	66.3602	34.1163	292.36	
11.162	66.9450	32.9983	307.80	
11.164	67.3949	38.1303	265.35	
11.166	66.7786	33.1455	304.96	
11.168	67.6478	39.6555	253.81	
11.170	66.7034	39.7930	244.00	
11.172	66.7219	32.7016	308.47	
11.174	67.4802	33.3456	309.50	
11.176	66.5125	28.8124	341.83	
11.178	67.4489	26.8638	367.99	
11.180	67.0570	32.6495	311.98	
11.182	66.6254	30.9924	323.09	
11.184	66.8583	31.5954	319.73	
11.186	67.5155	37.1084	275.71	
11.188	66.6904	32.3985	310.93	
11.190	67.3281	31.6960	323.08	
11.192	66.8327	31.9447	316.33	
11.194	66.8387	30.5789	328.77	
11.196	67.1076	32.0465	317.90	
11.198	66.9537	34.1921	297.05	
11.200	67.2441	32.7129	313.10	
11.202	67.0299	34.1579	298.06	
11.204	66.9942	34.9999	290.10	
11.206	66.6547	32.1003	313.31	
11.208	67.1821	30.3281	334.16	
11.210	67.1411	29.9232	337.46	
11.212	67.0380	29.7279	338.30	
11.214	67.2168	33.7524	303.43	
11.216	66.9150	35.2656	286.97	
11.218	66.6145	29.1651	339.56	
11.220	67.4347	30.5959	334.02	
11.222	66.8935	31.7164	318.96	cooling
11.224	67.0548	31.7332	320.27	cooling
11.226	67.1512	38.1430	263.02	cooling
11.228	66.3756	36.4523	271.32	
11.230	66.8747	34.6239	292.42	
11.232	67.4541	41.2891	237.24	
11.234	66.5850	37.8043	260.96	
11.236	67.2966	38.7778	258.58	
11.238	66.7242	38.9237	252.07	
11.240	66.2415	29.2726	335.20	
11.242	67.3990	32.0184	320.80	
11.244	66.7917	36.2179	277.22	
11.246	66.8886	31.7603	318.51	
11.248	67.2340	38.7847	257.95	
11.250	66.4247	38.1653	256.23	
11.252	66.5303	33.1188	302.95	
11.254	67.7463	41.4550	238.39	
11.256	66.2008	36.1191	272.76	
11.258	67.4598	34.2422	301.19	
11.260	66.8781	37.7570	264.05	
11.262	66.1769	30.9442	319.46	
11.264	67.2314	32.7442	312.70	
11.266	66.7607	35.3096	285.17	
11.268	66.8236	25.7313	372.59	
11.270	67.3358	31.7648	322.53	
11.272	66.4589	31.2052	319.65	
11.274	66.3425	24.6026	378.46	
11.276	67.8391	33.1659	314.39	
11.278	66.0931	30.8558	319.50	
11.280	67.5956	33.1263	312.54	
11.282	66.8387	38.7914	254.31	
11.284	66.0777	30.2859	324.53	
11.286	67.0348	30.0613	335.24	
11.288	67.1040	36.3652	278.71	
11.290	66.8440	28.7870	345.07	
11.292	67.4057	34.1263	301.75	
11.294	66.5394	33.0284	303.85	
11.296	66.3950	25.3429	372.23	
11.298	67.8939	32.6139	319.89	
11.300	66.1968	31.7729	312.13	
11.302	67.4766	33.0386	312.25	
11.304	66.8272	39.8156	244.92	
11.306	66.2154	36.2933	271.31	
11.308	66.8979	37.5664	265.95	
11.310	67.1724	43.0478	218.74	
11.312	66.7807	34.4559	293.09	

11.314	67.2599	36.6166	277.85	
11.316	66.5207	37.2108	265.76	
11.318	66.5102	34.8654	286.93	
11.320	67.4908	39.6778	252.18	
11.322	66.4258	37.2232	264.78	
11.324	67.3003	33.5732	305.81	
11.326	66.8727	35.5668	283.86	
11.328	66.5998	32.3928	310.16	
11.330	66.6465	30.4474	328.22	
11.332	67.3185	35.8288	285.52	
11.334	66.9166	35.1598	287.94	
11.336	67.0344	37.8348	264.76	
11.338	66.7164	38.3486	257.21	
11.340	66.5154	35.3746	282.36	
11.342	67.1962	35.0560	291.42	
11.344	66.7171	34.5343	291.81	
11.346	67.0990	34.4405	296.12	
11.348	66.7688	34.0963	296.25	
11.350	66.8964	33.3812	303.89	
11.352	66.5718	29.5617	335.58	
11.354	67.0483	29.8544	337.24	
11.356	67.1833	31.8405	320.46	
11.358	66.6331	30.2425	329.96	
11.360	67.0502	32.8223	310.35	
11.362	66.7473	37.9022	261.54	
11.364	66.8387	38.3313	258.48	
11.366	67.1316	39.2089	253.18	
11.368	66.9382	38.0804	261.66	
11.370	66.8002	33.7260	299.89	
11.372	67.0779	37.0440	272.32	
11.374	66.6406	36.8905	269.75	
11.376	66.6930	33.7169	299.00	
11.378	67.4266	37.2409	273.70	
11.380	66.4298	33.5648	297.99	heating
11.382	67.1687	32.5068	314.28	
11.384	66.9604	36.6371	274.95	
11.386	66.5484	31.9345	313.85	
11.388	67.1984	31.6674	322.17	
11.390	67.1105	36.7860	274.96	
11.392	66.7062	33.9137	297.33	
11.394	67.3799	39.5383	252.44	
11.396	66.6446	37.5424	263.87	
11.398	66.6751	29.6289	335.90	heating
11.400	67.6516	33.9588	305.50	
11.402	66.4697	31.3171	318.73	
11.404	67.2535	27.9519	356.35	
11.406	67.2213	33.1141	309.26	
11.408	66.4539	29.8959	331.48	
11.410	67.1104	29.2308	343.46	
11.412	67.1543	33.9464	301.10	
11.414	66.5582	27.8509	350.96	
11.416	67.3790	32.9729	311.96	
11.418	66.7680	35.2685	285.61	
11.420	66.6167	29.0699	340.44	
11.422	67.6444	33.6086	308.61	
11.424	66.3367	31.9273	312.00	
11.426	67.3496	28.8448	349.13	
11.428	67.2514	35.6220	286.79	
11.430	66.4724	33.0902	302.68	
11.432	66.8459	31.5214	320.29	
11.434	67.3807	36.6463	278.67	
11.436	66.5379	31.0551	321.73	
11.438	67.4212	36.0171	284.75	
11.440	66.7794	38.4338	257.01	
11.442	66.5247	29.4467	336.19	
11.444	67.4417	30.4680	335.25	
11.446	66.4781	31.3068	318.90	
11.448	67.2362	30.7133	331.16	
11.450	67.3175	38.9605	257.12	
11.452	66.5455	36.8351	269.39	
11.454	66.7704	33.1058	305.24	
11.456	67.5703	39.0806	258.32	
11.458	66.5514	34.5266	290.37	
11.460	67.3471	36.0934	283.38	
11.462	66.9488	37.2397	269.38	
11.464	66.4903	31.1056	320.84	
11.466	67.3536	32.8920	312.47	
11.468	66.6248	35.0197	286.57	
11.470	67.1553	30.9432	328.34	
11.472	67.0036	30.1426	334.22	
11.474	66.8010	27.6025	355.42	
11.476	66.7453	26.3385	366.37	
11.478	67.3866	33.8149	304.40	
11.480	66.6736	34.7192	289.74	
11.482	67.0613	35.8578	282.93	
11.484	67.2135	37.6177	268.35	
11.486	66.5401	32.4239	309.34	
11.488	67.0970	29.0088	345.35	

11.490	66.7716	27.7494	353.82	
11.492	67.0064	23.2874	396.41	
11.494	66.9253	23.2375	396.12	cooling
11.496	66.9306	27.0215	361.86	cooling
11.498	66.6856	26.9886	359.94	cooling
11.500	66.8641	28.5850	347.08	cooling
11.502	66.6435	27.8441	351.80	cooling
11.504	66.6523	27.7546	352.69	cooling
11.506	67.0436	30.1691	334.35	cooling
11.508	66.4329	32.2883	309.59	cooling
11.510	66.4266	32.3545	308.94	cooling
11.512	66.3745	33.2258	300.56	cooling
11.514	66.6699	33.0826	304.54	cooling
11.516	66.3222	27.6656	350.50	cooling
11.518	66.6935	29.0760	341.08	cooling
11.520	66.0609	28.2214	343.10	cooling
11.522	66.0660	28.1773	343.54	cooling
11.524	66.4308	33.9882	294.16	cooling
11.526	65.7300	34.1601	286.25	cooling
11.528	66.6205	35.4375	282.74	cooling
11.530	65.9994	39.7619	237.90	cooling
11.532	65.7697	37.9550	252.20	cooling
11.534	65.8415	37.0282	261.25	cooling
11.536	66.3042	37.9850	256.77	cooling
11.538	65.7786	31.9157	307.04	cooling
11.540	66.3248	37.0905	265.07	cooling
11.542	65.6835	39.6070	236.44	
11.544	65.5386	35.4620	272.71	
11.546	66.3457	36.7826	268.05	
11.548	65.3168	33.7377	286.33	
11.550	66.3176	31.1602	318.78	
11.552	65.9777	36.5027	267.25	
11.554	65.4699	34.1626	283.87	
11.556	65.6273	31.5080	309.36	
11.558	66.4627	34.2826	291.78	
11.560	65.7151	27.1718	349.48	
11.562	66.3478	30.7479	322.79	
11.564	65.6718	32.1482	303.96	
11.566	65.4953	26.6650	352.08	
11.568	66.4839	29.7906	332.70	
11.570	65.3857	31.5132	307.13	
11.572	66.3026	30.4777	324.83	
11.574	66.0327	34.7698	283.47	
11.576	65.5536	30.7613	315.47	
11.578	65.5610	26.8367	351.12	
11.580	66.5867	32.5114	308.97	
11.582	65.5908	27.5617	344.82	
11.584	66.2566	30.9711	319.94	cooling
11.586	65.7089	34.8698	279.62	cooling
11.588	65.4356	31.3130	309.39	
11.590	66.3059	33.3829	298.52	
11.592	65.4960	35.6004	271.07	
11.594	66.1046	34.1164	290.04	
11.596	65.9962	39.9163	236.47	
11.598	65.6405	40.1970	230.70	
11.600	65.5147	37.7150	252.06	
11.602	66.3473	40.6208	233.27	
11.604	65.3524	34.0786	283.56	
11.606	66.1270	33.4087	296.66	
11.608	65.7044	34.9695	278.68	
11.610	65.5293	31.7490	306.29	
11.612	66.0158	31.8929	309.40	
11.614	65.6095	33.0311	295.39	
11.616	66.1134	33.5038	295.68	
11.618	65.8926	36.3877	267.53	
11.620	65.8435	33.9562	289.13	
11.622	65.6585	30.8471	315.64	
11.624	66.0694	33.2170	297.88	
11.626	65.6572	33.3163	293.24	
11.628	66.0039	34.8011	282.92	
11.630	65.8667	34.4062	285.26	
11.632	65.6013	31.6160	308.15	
11.634	65.8580	29.2453	331.97	
11.636	65.6377	28.1091	340.28	
11.638	66.2037	30.3761	324.85	
11.640	65.6110	29.7055	325.56	
11.642	66.0453	30.2343	324.70	
11.644	65.8694	33.7459	291.27	
11.646	65.5908	36.2152	266.35	
11.648	65.8477	38.1770	250.89	
11.650	65.7495	38.8357	244.03	
11.652	66.0065	36.1588	270.63	
11.654	65.8363	38.7263	245.81	
11.656	65.6509	38.9726	241.90	
11.658	65.6106	37.4467	255.37	heating
11.660	66.3663	40.8525	231.34	heating
11.662	65.6488	37.1091	258.77	heating
11.664	66.3943	36.8986	267.44	heating
11.666	66.2000	39.2413	244.44	heating
11.668	65.7348	35.4350	274.73	heating
11.670	66.2306	33.0446	300.90	heating
11.672	66.1979	35.0272	282.63	heating
11.674	66.5588	35.5293	281.35	heating
11.676	66.5365	42.6371	216.70	heating
11.678	66.0687	42.8425	210.60	heating
11.680	65.8863	36.6494	265.10	heating
11.682	67.0507	39.8038	247.05	heating
11.684	66.1697	35.8329	275.07	heating
11.686	66.8975	33.8060	300.05	heating
11.688	66.6454	35.3356	283.89	heating
11.690	66.1372	31.0287	318.33	heating
11.692	66.8639	31.4009	321.55	heating
11.694	66.5939	34.7988	288.29	heating
11.696	66.8464	28.9682	343.45	heating
11.698	67.0718	32.3671	314.67	heating
11.700	66.6368	33.6234	299.34	heating
11.702	66.2115	29.5390	332.51	heating
11.704	67.5718	36.2111	284.35	heating
11.706	66.4534	36.2352	273.99	heating
11.708	67.2705	35.9958	283.57	heating
11.710	67.0309	38.5342	258.38	heating
11.712	66.6053	35.3063	283.79	heating
11.714	67.0759	33.0647	308.38	heating
11.716	67.1137	33.4102	305.59	heating
11.718	67.2815	27.7465	358.47	heating
11.720	67.5495	33.1556	311.85	heating
11.722	67.2762	37.9275	266.11	heating
11.724	66.6561	32.4987	309.71	heating
11.726	68.0417	34.3091	305.86	heating
11.728	66.9911	33.1074	307.23	
11.730	67.9788	34.8456	300.42	
11.732	67.5757	38.9142	259.88	
11.734	67.1615	36.7547	275.70	heating
11.736	67.5262	35.5550	289.89	heating
11.738	67.7235	38.9140	261.22	heating
11.740	67.7926	33.3286	312.49	heating
11.742	67.8325	33.6047	310.35	heating
11.744	67.8516	34.1447	305.63	heating
11.746	67.2493	30.4570	333.60	
11.748	68.1477	33.1945	316.93	
11.750	67.3813	34.9149	294.38	
11.752	68.0414	35.8530	291.86	
11.754	67.8014	36.2567	286.02	
11.756	67.5154	36.0729	285.09	
11.758	67.5324	34.8837	296.03	
11.760	67.7575	35.9499	288.40	
11.762	67.8692	32.7242	318.67	
11.764	67.6103	31.8055	324.65	
11.766	68.0524	35.3927	296.13	
11.768	67.3338	34.3726	298.86	
11.770	67.7885	32.8177	317.09	
11.772	67.5327	32.2977	319.48	
11.774	68.0141	34.1742	306.83	
11.776	67.9207	34.6820	301.38	
11.778	67.6544	36.2823	284.46	
11.780	67.4371	33.1929	310.50	
11.782	67.6871	31.4871	328.23	
11.784	68.1819	30.1566	344.78	
11.786	67.3761	25.7493	377.44	
11.788	68.1959	29.7080	348.98	
11.790	67.4065	32.8768	313.09	
11.792	67.5010	31.5326	326.13	
11.794	67.8564	35.7918	290.73	
11.796	67.8367	42.0007	234.26	
11.798	67.9959	40.4093	250.13	
11.800	67.7327	40.4900	247.01	
11.802	67.4367	35.6479	288.23	
11.804	67.4408	32.2397	319.17	
11.806	68.5416	35.9444	295.56	
11.808	67.2361	30.9351	329.15	
11.810	68.2018	33.4941	314.70	
11.812	67.5758	38.6355	262.41	
11.814	67.4108	36.3082	282.01	
11.816	67.8186	34.9778	297.77	
11.818	67.5572	36.4381	282.16	
11.820	68.1393	34.9361	301.06	
11.822	67.8509	41.0226	243.26	
11.824	67.5167	39.7021	252.20	
11.826	67.1604	35.4374	287.64	
11.828	68.7185	41.2232	249.30	
11.830	67.1647	34.9691	291.92	
11.832	68.2458	33.1108	318.57	
11.834	67.6147	34.5032	300.23	
11.836	67.2285	29.3704	343.27	
11.838	67.8891	28.3119	358.85	
11.840	67.6235	32.7193	316.48	

11.842	68.1034	30.8479	337.80	
11.844	67.9319	37.9803	271.58	
11.846	67.6107	37.4468	273.50	
11.848	67.0727	30.8096	328.80	
11.850	68.7415	38.7529	271.91	
11.852	67.1084	37.2304	270.91	
11.854	68.1936	37.5781	277.59	
11.856	67.7234	40.3145	248.52	
11.858	67.2174	36.9336	274.59	
11.860	68.0753	36.8420	283.20	
11.862	67.6501	38.5553	263.81	
11.864	67.9565	32.8147	318.64	
11.866	67.8794	36.8471	281.38	
11.868	67.7338	38.9167	261.29	
11.870	67.2703	36.8623	275.71	
11.872	68.3969	43.7853	223.16	heating
11.874	67.3337	41.5088	234.16	heating
11.876	68.3543	40.2595	254.74	heating
11.878	68.0679	41.4469	241.38	heating
11.880	67.6054	39.5080	254.76	heating
11.882	68.1948	38.2639	271.39	heating
11.884	67.9156	37.1933	278.56	heating
11.886	68.2639	33.2360	317.60	heating
11.888	68.1440	35.6473	294.65	heating
11.890	68.3730	38.2228	273.38	heating
11.892	67.8713	33.9705	307.38	heating
11.894	68.4446	33.7452	314.62	heating
11.896	68.2399	33.0186	319.36	heating
11.898	68.7506	34.5681	309.94	heating
11.900	68.6905	34.7355	307.87	heating
11.902	68.1152	32.8783	319.50	heating
11.904	68.6815	32.5299	327.79	heating
11.906	68.5022	32.2939	328.31	heating
11.908	68.8995	31.9768	334.78	
11.910	68.3901	32.0497	329.50	
11.912	68.8880	34.4522	312.23	
11.914	68.4819	33.9158	313.42	
11.916	68.3385	31.5416	333.64	
11.918	68.6103	32.0742	331.28	
11.920	68.7481	34.5646	309.95	
11.922	68.8402	33.0318	324.68	
11.924	68.3315	32.5175	324.73	
11.926	68.5830	33.5712	317.46	
11.928	68.4080	33.8460	313.38	
11.930	68.9354	35.7434	300.96	
11.932	68.3113	34.2945	308.44	
11.934	68.7348	35.7407	299.16	
11.936	68.6583	39.9281	260.50	
11.938	68.1167	37.6962	275.83	
11.940	68.4452	34.0783	311.61	
11.942	68.7456	35.8912	297.90	
11.944	68.6428	33.7572	316.31	
11.946	68.5763	34.1640	312.02	
11.948	68.4659	34.0071	312.44	
11.950	68.1840	30.4031	342.57	
11.952	69.1079	34.8677	310.46	
11.954	68.2088	34.9612	301.46	
11.956	68.7923	34.3084	312.67	
11.958	68.7193	38.1000	277.63	
11.960	68.0646	34.2866	306.27	
11.962	68.4608	30.0550	348.23	
11.964	68.9275	34.0454	316.28	
11.966	68.4869	31.1175	338.83	
11.968	68.7637	33.8604	316.47	
11.970	68.4559	35.6125	297.80	
11.972	68.1642	32.4120	324.17	
11.974	69.2365	37.1628	290.82	
11.976	68.1200	36.9353	282.76	
11.978	68.8392	32.6181	328.42	
11.980	68.6527	35.6103	299.60	
11.982	68.3814	34.9267	303.34	
11.984	68.2499	31.3206	334.84	
11.986	69.0198	35.8285	300.95	
11.988	68.3989	34.8505	304.19	
11.990	68.8324	39.1520	269.12	
11.992	68.4306	40.7381	251.09	
11.994	68.1134	37.3702	278.75	
11.996	68.9570	39.9314	263.18	
11.998	68.1521	41.4126	242.45	
12.000	68.9169	39.5092	266.64	
12.002	68.5085	41.7620	242.51	
12.004	68.5346	41.7219	243.12	
12.006	68.1949	37.3811	279.39	
12.008	68.9573	38.2490	278.44	
12.010	68.4679	34.7511	305.72	
12.012	68.6059	35.0293	304.44	
12.014	68.6283	36.5272	291.07	
12.016	68.1929	35.8361	293.38	

12.018	68.9013	39.1454	269.80	
12.020	68.4968	43.7740	224.17	
12.022	68.8953	41.5925	247.56	heating
12.024	68.6910	40.6673	254.09	heating
12.026	68.8302	42.4147	239.51	heating
12.028	68.5639	39.8990	259.91	heating
12.030	69.0210	37.7671	283.38	heating
12.032	68.8232	33.2419	322.62	heating
12.034	68.9066	32.8002	327.38	heating
12.036	69.2192	35.2043	308.42	heating
12.038	68.6698	32.8926	324.40	heating
12.040	69.0498	30.7311	347.44	heating
12.042	69.1156	33.6403	321.66	
12.044	69.3422	36.6495	296.43	
12.046	69.1278	37.0541	290.82	
12.048	69.0836	38.8677	273.97	
12.050	69.0194	38.1949	279.49	
12.052	68.9506	37.0164	289.55	
12.054	69.2616	35.4341	306.72	
12.056	68.8362	33.0476	324.50	
12.058	69.3621	32.5599	333.69	
12.060	68.6782	28.8061	361.53	
12.062	68.9675	24.6067	402.23	
12.064	69.1336	25.8276	392.66	
12.066	69.3936	32.7702	332.07	
12.068	69.0267	31.8953	336.68	
12.070	69.0614	31.6321	339.38	
12.072	69.2120	32.8078	330.08	
12.074	68.6208	31.8980	332.97	cooling
12.076	69.3585	33.6618	323.67	cooling
12.078	68.5988	32.4629	327.65	cooling
12.080	69.2751	32.5223	333.24	cooling
12.082	68.8086	36.2249	295.44	cooling
12.084	68.6764	35.8175	297.94	cooling
12.086	68.6902	32.6664	326.63	cooling
12.088	68.9622	35.1837	306.28	cooling
12.090	68.5968	31.1448	339.58	cooling
12.092	68.7894	33.6146	318.94	cooling
12.094	68.9001	39.0724	270.45	cooling
12.096	68.1142	38.6822	266.86	cooling
12.098	69.1405	41.8310	247.62	cooling
12.100	68.0663	40.9501	245.87	
12.102	68.9354	39.3705	268.07	
12.104	68.6341	43.3782	229.00	
12.106	68.3063	40.5228	251.92	
12.108	68.2334	33.4278	315.59	
12.110	69.0929	38.3499	278.75	
12.112	68.3863	36.1327	292.45	
12.114	68.7699	37.2853	285.48	
12.116	68.5942	38.1547	276.00	
12.118	68.0426	34.5775	303.43	
12.120	69.1538	37.8525	283.81	
12.122	68.1133	37.5833	276.82	
12.124	68.8385	32.5293	329.22	
12.126	68.7488	33.8795	316.17	
12.128	68.4182	31.1297	338.10	
12.130	68.1833	26.2007	380.66	
12.132	69.1106	32.3574	333.25	
12.134	68.1813	30.7738	339.18	
12.136	68.8484	32.2941	331.44	
12.138	68.5008	34.6609	306.83	
12.140	68.3096	33.8628	312.33	
12.142	68.9498	39.0047	271.52	
12.144	68.2383	39.9700	256.31	
12.146	68.7012	35.4967	301.07	
12.148	68.8113	38.6650	273.34	
12.150	68.4428	38.0983	275.14	
12.152	68.3250	34.7303	304.61	
12.154	68.7299	35.3654	302.52	
12.156	68.2628	33.7135	313.26	
12.158	68.8337	36.5595	292.63	
12.160	68.4730	39.6999	260.89	
12.162	68.4105	39.2471	264.43	
12.164	68.5449	40.5367	253.95	
12.166	68.5116	40.7227	251.97	
12.168	68.4993	37.6188	280.00	
12.170	68.8751	39.6372	265.10	
12.172	68.5029	37.5412	280.73	
12.174	68.5741	35.5090	299.81	
12.176	68.5107	34.2061	311.05	
12.178	68.3346	32.4912	325.00	
12.180	68.9457	35.0620	307.23	
12.182	68.3601	33.7392	313.91	
12.184	68.6164	30.8155	342.75	
12.186	68.5377	32.3675	327.96	
12.188	68.5945	33.8101	315.40	
12.190	68.5596	33.7348	315.76	
12.192	68.6125	33.0230	322.69	

12.194	68.6066	32.7314	325.29	
12.196	68.7102	36.2573	294.25	
12.198	68.1141	33.1509	317.02	
12.200	68.4952	31.9284	331.56	
12.202	68.8449	33.9807	316.12	
12.204	68.3640	30.8029	340.57	
12.206	68.8899	30.5597	347.55	
12.208	68.2228	30.6436	340.74	
12.210	68.3723	29.1342	355.78	
12.212	68.6481	33.0218	323.03	cooling
12.214	68.3353	34.1523	309.94	cooling
12.216	68.5851	34.8402	305.97	cooling
12.218	68.5263	40.9236	250.28	cooling
12.220	67.9092	38.8914	263.11	
12.222	68.3893	37.9215	276.26	
12.224	68.5329	39.7929	260.59	
12.226	68.3500	35.9173	294.07	
12.228	68.7440	37.5276	283.04	
12.230	68.1929	38.1719	272.20	
12.232	68.0563	35.1127	298.70	
12.234	68.8763	40.2547	259.52	
12.236	68.1502	38.7111	266.93	
12.238	68.5542	34.6138	307.74	
12.240	68.6281	39.0590	268.11	
12.242	67.8507	37.1789	278.11	
12.244	68.2668	35.8139	294.26	
12.246	68.5711	39.7049	261.73	
12.248	68.4383	36.9497	285.51	
12.250	68.8299	40.9389	252.89	
12.252	68.3277	42.8366	231.13	
12.254	67.8493	36.9151	280.48	heating
12.256	69.0210	38.8136	273.89	heating
12.258	68.1914	35.2941	298.28	heating
12.260	68.8035	31.8601	334.97	heating
12.262	68.7488	35.4659	301.78	
12.264	68.1330	33.4625	314.36	
12.266	68.4912	31.1409	338.66	
12.268	68.6683	35.2176	303.30	
12.270	68.6852	34.5484	309.52	
12.272	68.7816	38.6526	273.18	
12.274	68.4704	39.6976	260.89	
12.276	67.9181	33.0048	316.56	
12.278	69.0147	34.8796	309.51	
12.280	68.2046	34.3376	307.08	
12.282	68.7716	32.6939	327.12	
12.284	68.5839	33.7441	315.90	
12.286	68.1603	30.9844	337.08	
12.288	68.4413	29.2415	355.43	
12.290	68.5150	32.4555	326.96	
12.292	68.7011	32.4096	329.06	
12.294	68.6494	36.0072	295.97	
12.296	68.5203	36.7715	287.87	
12.298	68.2352	34.6339	304.67	
12.300	68.7911	36.1249	296.19	
12.302	68.2372	33.2106	317.59	
12.304	68.8078	32.3331	330.72	
12.306	68.5649	32.0424	331.16	
12.308	68.5229	33.8491	314.39	
12.310	68.6506	37.9280	278.57	
12.312	68.4348	39.3791	263.45	
12.314	68.9388	40.8741	254.47	
12.316	68.5345	42.1518	239.22	
12.318	68.5152	40.7780	251.50	
12.320	68.4901	40.4426	254.31	
12.322	68.1608	37.6439	276.70	
12.324	68.6347	35.8184	297.55	
12.326	68.7459	38.0937	277.93	
12.328	68.5558	36.1654	293.69	
12.330	68.7015	38.3111	275.55	
12.332	68.4676	39.9660	258.43	
12.334	68.2412	35.7454	294.64	
12.336	69.0760	40.1491	262.28	
12.338	68.3344	39.3253	263.03	heating
12.340	68.7042	37.4234	283.63	heating
12.342	68.8501	41.4046	248.85	heating
12.344	67.9586	37.3493	277.54	heating
12.346	68.9882	36.6372	293.33	heating
12.348	68.8321	39.8621	262.68	heating
12.350	68.6200	33.2747	320.48	heating
12.352	69.1344	34.9860	309.63	heating
12.354	68.7434	36.7198	290.36	heating
12.356	68.3765	30.5843	342.67	heating
12.358	69.6392	36.2329	302.90	heating
12.360	68.5935	34.7852	306.54	
12.362	69.2649	34.8891	311.69	
12.364	69.2562	41.7216	249.66	
12.366	68.1884	37.8924	274.70	heating
12.368	69.4055	38.5126	280.11	heating
12.370	69.0946	42.7836	238.57	heating
12.372	69.0465	36.1772	298.03	heating
12.374	69.6972	39.8034	271.05	heating
12.376	69.0190	40.4564	258.98	heating
12.378	68.6862	31.8294	334.19	heating
12.380	70.0268	37.0601	298.91	heating
12.382	68.7780	34.6283	309.64	heating
12.384	69.8695	35.0791	315.45	heating
12.386	69.6795	44.6312	227.12	heating
12.388	68.7735	41.6538	245.90	heating
12.390	69.7676	42.5083	247.16	heating
12.392	69.7078	48.0330	196.53	heating
12.394	69.3703	39.9125	267.10	heating
12.396	70.1528	42.0791	254.55	heating
12.398	69.4545	41.1150	256.96	heating
12.400	69.3178	34.7898	313.07	heating
12.402	70.2383	38.2816	289.76	heating
12.404	69.1703	35.0771	309.13	heating
12.406	70.4171	35.7624	314.22	heating
12.408	69.9717	42.8699	245.74	heating
12.410	69.4138	38.2842	282.26	heating
12.412	69.8818	36.1937	305.46	heating
12.414	70.2497	40.0689	273.65	heating
12.416	69.6968	34.2137	321.73	heating
12.418	70.6265	38.0122	295.72	heating
12.420	69.9463	37.4430	294.71	heating
12.422	69.9370	33.0616	334.35	heating
12.424	70.3719	35.0959	319.85	heating
12.426	69.6747	32.7985	334.36	heating
12.428	70.9710	32.8053	346.05	heating
12.430	70.2713	36.7173	304.24	
12.432	70.0205	32.2762	342.23	
12.434	70.1360	30.3091	361.12	
12.436	70.5316	32.6237	343.72	
12.438	70.0141	31.5476	348.78	
12.440	70.6435	34.5417	327.34	
12.442	70.2159	33.6586	331.47	
12.444	70.3199	37.0567	301.60	
12.446	69.9490	39.1337	279.41	
12.448	70.0851	38.4621	286.73	
12.450	70.8027	37.1870	304.80	
12.452	70.1062	35.0340	318.00	
12.454	70.4138	35.0522	320.63	
12.456	69.9480	35.3769	313.46	
12.458	70.3894	35.3419	317.78	
12.460	70.0942	36.8654	301.29	
12.462	70.4430	38.7087	287.74	
12.464	70.2363	37.7000	295.01	
12.466	70.4074	41.7820	259.55	
12.468	69.6981	39.7608	271.45	
12.470	70.1914	37.9334	292.49	
12.472	70.7747	39.3511	284.92	
12.474	70.0396	38.0424	290.12	
12.476	70.6134	41.7930	261.32	
12.478	69.8446	41.6020	256.08	
12.480	69.9324	34.6099	320.27	
12.482	70.5282	37.8034	296.72	
12.484	70.0884	37.6380	294.23	
12.486	70.5786	35.4704	318.33	
12.488	70.4109	39.6983	278.48	
12.490	69.5096	35.2258	310.86	
12.492	70.5096	36.5192	308.20	
12.494	70.5818	41.2271	266.16	
12.496	70.0106	35.6772	311.31	
12.498	70.7325	39.9062	279.51	
12.500	69.8079	40.0323	269.98	
12.502	69.7254	32.9376	333.56	
12.504	70.8401	39.2980	286.00	
12.506	69.8293	37.5115	293.03	
12.508	70.6366	35.0190	322.95	
12.510	70.3819	40.6684	269.42	
12.512	69.4747	35.3282	309.61	
12.514	70.4860	34.6785	324.67	
12.516	70.5264	38.8783	286.96	
12.518	69.9171	30.3099	359.12	
12.520	70.8093	36.1881	313.92	
12.522	69.8910	38.6058	283.67	
12.524	69.6291	32.6882	334.95	
12.526	70.9858	39.7589	283.14	
12.528	69.6265	35.6960	307.65	
12.530	70.9945	32.8119	346.21	
12.532	70.3990	38.8387	286.16	
12.534	69.5603	32.4790	336.22	
12.536	70.5545	34.2407	329.26	
12.538	70.5321	42.0600	258.16	
12.540	70.0636	38.0538	290.24	
12.542	71.0134	46.8140	219.42	
12.544	69.8356	44.7495	227.46	

12.546	69.8573	36.7287	300.38	
12.548	70.7069	39.4113	283.76	
12.550	69.6149	35.0458	313.44	
12.552	71.1716	36.6616	312.91	
12.554	70.1137	41.4011	260.34	
12.556	69.9055	37.3047	295.60	
12.558	70.5816	41.0234	268.01	
12.560	70.2265	43.8253	239.38	
12.562	70.1490	37.6373	294.79	
12.564	70.7799	42.5496	255.97	
12.566	69.9067	40.0045	271.13	heating
12.568	70.2799	40.0828	273.80	heating
12.570	70.4145	43.2237	246.54	heating
12.572	70.1583	37.1422	299.36	heating
12.574	71.4182	37.0348	311.76	heating
12.576	70.2786	34.6709	322.86	heating
12.578	70.6425	30.2043	366.66	heating
12.580	70.9241	35.0436	325.33	
12.582	70.3315	34.6856	323.21	
12.584	70.7577	34.8848	325.26	
12.586	70.9192	41.0884	270.48	
12.588	70.3022	38.6159	287.30	
12.590	70.6944	38.9152	288.15	
12.592	70.1969	36.0767	309.37	
12.594	70.5030	30.3555	364.02	
12.596	71.2458	34.3809	334.26	
12.598	70.2295	34.0799	327.77	
12.600	70.7853	34.3985	329.92	
12.602	70.7933	39.9003	280.11	
12.604	70.1222	34.9174	319.21	
12.606	71.0105	36.5487	312.47	
12.608	70.6978	38.6846	290.27	
12.610	70.4474	31.8992	349.52	
12.612	70.9843	36.9598	308.51	
12.614	70.0457	37.9882	290.67	
12.616	70.4440	36.3214	309.39	
12.618	71.1795	44.2734	243.96	
12.620	69.9613	39.8940	272.62	
12.622	70.9506	38.4456	294.73	
12.624	70.5693	41.1029	267.18	
12.626	70.0591	33.6650	329.99	
12.628	71.1094	35.9885	318.45	
12.630	70.5016	37.7182	297.25	
12.632	70.4650	33.7392	333.00	
12.634	71.0139	40.7046	274.82	
12.636	69.8872	37.5154	293.52	
12.638	70.4947	33.0977	339.08	
12.640	71.0013	38.0761	298.54	
12.642	70.0275	34.4024	323.02	
12.644	71.1126	38.3628	296.95	
12.646	70.4121	42.4019	253.97	
12.648	70.1073	34.0738	326.72	
12.650	71.0946	35.4539	323.16	
12.652	70.2469	34.7588	321.78	
12.654	70.7233	30.6373	363.47	
12.656	70.9165	37.0451	307.12	
12.658	69.9101	32.2939	341.07	
12.660	70.6008	31.5408	354.16	
12.662	70.9252	38.7181	292.03	cooling
12.664	70.0543	34.0524	326.43	
12.666	71.1228	36.4548	314.34	cooling
12.668	69.8421	33.9466	325.47	cooling
12.670	70.3915	30.6099	360.71	cooling
12.672	70.7770	37.6043	300.78	cooling
12.674	69.9587	37.7181	292.33	cooling
12.676	70.6659	35.4810	319.03	cooling
12.678	70.3853	39.5953	279.18	cooling
12.680	69.8785	33.6483	328.50	cooling
12.682	70.3109	35.5483	315.20	cooling
12.684	70.4887	38.5144	289.92	cooling
12.686	69.5635	32.0852	339.82	cooling
12.688	71.0132	37.5170	303.71	cooling
12.690	69.3927	35.7334	305.19	cooling
12.692	70.4261	36.4326	308.22	cooling
12.694	70.0530	41.4275	259.55	cooling
12.696	69.5160	36.7655	296.95	
12.698	70.6096	36.0197	313.63	
12.700	69.8315	39.4383	275.58	
12.702	70.0577	36.3815	305.35	
12.704	70.1584	41.8172	256.97	
12.706	69.9205	40.4798	266.94	
12.708	69.6574	36.5850	299.87	
12.710	70.7467	40.8313	271.25	
12.712	69.3677	34.4352	316.74	
12.714	70.6415	36.8546	306.35	
12.716	69.5350	38.3283	282.96	
12.718	69.9179	36.3160	304.67	
12.720	70.6509	39.7846	279.87	
12.722	69.4853	38.6278	279.79	
12.724	70.3517	35.7239	313.98	
12.726	69.9015	37.8390	290.72	
12.728	69.7599	30.7839	353.40	
12.730	69.9101	32.5180	339.04	
12.732	70.4049	39.6835	278.56	
12.734	69.6561	35.9860	305.29	
12.736	70.7698	43.1071	250.82	
12.738	69.2352	39.8614	266.34	
12.740	70.0053	34.7604	319.57	
12.742	70.4181	35.7511	314.33	
12.744	69.2664	27.3740	379.84	
12.746	70.5415	28.2217	383.72	
12.748	69.9606	35.1381	315.74	
12.750	69.5504	29.3024	364.93	
12.752	70.3953	36.3247	308.92	cooling
12.754	69.7162	39.1620	277.04	cooling
12.756	69.9794	33.3836	331.82	
12.758	70.6607	40.5252	273.24	cooling
12.760	69.0084	32.8707	327.67	
12.762	70.1503	28.8934	374.08	
12.764	70.3180	36.7681	304.20	cooling
12.766	69.0202	33.1347	325.38	cooling
12.768	70.4533	39.3500	282.02	cooling
12.770	69.5393	43.8218	233.18	cooling
12.772	69.1745	32.2570	334.74	
12.774	70.2627	35.5305	314.92	
12.776	69.4773	35.9125	304.34	
12.778	69.8992	31.3284	349.73	
12.780	70.3507	41.9696	257.34	cooling
12.782	68.8869	39.2686	268.55	
12.784	69.8984	38.1094	288.24	
12.786	70.0816	45.5418	222.51	
12.788	68.7256	35.7138	299.32	
12.790	70.3333	37.9407	293.71	
12.792	69.4063	41.2671	255.14	
12.794	69.1567	34.5884	313.44	
12.796	70.1180	41.2770	261.51	
12.798	69.2555	41.8233	248.73	
12.800	69.8683	35.5546	311.13	
12.802	69.9956	40.3859	268.48	
12.804	69.0542	34.7405	311.13	
12.806	69.7608	32.3338	339.36	
12.808	69.9150	38.9462	280.80	
12.810	68.9631	34.3683	313.68	
12.812	70.3130	38.1223	291.88	
12.814	69.7711	42.1588	250.36	
12.816	69.2276	34.8286	311.90	
12.818	69.8989	34.0301	325.23	
12.820	69.5266	33.3415	328.10	
12.822	69.9330	29.8335	363.59	
12.824	69.9491	35.8575	309.11	
12.826	69.3024	35.6762	304.89	
12.828	69.7734	34.9268	315.96	
12.830	69.8380	40.5908	265.19	
12.832	69.1442	38.2609	280.02	
12.834	69.9720	37.5869	293.64	
12.836	69.9362	37.9473	290.05	
12.838	69.4782	34.4509	317.60	
12.840	69.6015	33.8775	323.91	
12.842	69.6825	35.0121	314.36	
12.844	69.9292	33.9247	326.46	
12.846	69.6907	34.9238	315.24	
12.848	69.9085	36.0841	306.69	
12.850	69.6310	34.7476	316.29	
12.852	69.8441	36.4908	302.42	
12.854	69.4059	36.7931	295.70	
12.856	69.7706	34.5725	319.15	
12.858	70.0747	35.5205	313.31	
12.860	69.6709	37.6734	290.13	
12.862	69.4332	34.8391	313.67	
12.864	69.7324	32.6614	336.13	
12.866	69.8991	31.6819	346.52	
12.868	69.3757	29.5941	360.70	
12.870	70.1283	34.2081	325.69	cooling
12.872	69.5016	37.0510	294.23	cooling
12.874	69.3148	34.4170	316.42	cooling
12.876	69.4926	35.6456	306.90	cooling
12.878	69.4707	34.8920	313.53	cooling
12.880	69.6149	33.1573	330.57	cooling
12.882	69.6954	38.6622	281.38	cooling
12.884	68.9066	35.0893	306.63	cooling
12.886	69.4944	34.3032	319.08	cooling
12.888	69.5196	37.9186	286.53	cooling
12.890	68.8320	35.8565	298.99	
12.892	69.6999	37.0939	295.64	
12.894	69.0662	37.5608	285.66	
12.896	69.0001	32.3502	332.31	

12.898	69.4027	36.2840	300.29	
12.900	69.3296	39.3576	271.76	
12.902	69.2894	36.8323	294.29	
12.904	69.7702	42.5339	246.96	
12.906	68.7365	39.5268	264.85	
12.908	69.3042	36.9146	293.68	
12.910	69.5920	41.1863	257.56	
12.912	68.7037	35.4708	301.33	
12.914	69.8549	35.9020	307.86	
12.916	69.2581	39.5064	269.76	
12.918	68.9053	34.3797	313.05	
12.920	69.6483	38.6247	281.30	
12.922	68.9807	38.5344	276.06	
12.924	69.2345	32.0268	337.37	
12.926	69.8629	37.4493	293.90	
12.928	68.7357	36.4006	293.19	
12.930	69.2541	34.6804	313.48	
12.932	69.5148	39.6866	270.46	
12.934	69.0194	35.5532	303.44	
12.936	69.6733	33.9014	324.35	
12.938	69.4081	36.6378	297.13	
12.940	68.9503	30.5837	347.88	
12.942	69.5051	32.0274	339.82	
12.944	69.3145	34.5047	315.63	
12.946	69.2094	32.0684	336.76	
12.948	69.8494	38.2560	286.46	
12.950	68.9385	38.9845	271.60	
12.952	69.0859	35.4102	305.34	
12.954	69.4317	35.6218	306.56	
12.956	68.9701	30.8319	345.80	
12.958	69.6346	30.7904	352.21	
12.960	69.3427	35.8106	304.04	
12.962	69.1076	37.1845	289.45	
12.964	69.3070	40.1396	264.46	
12.966	69.1397	40.0083	264.14	
12.968	69.1792	35.1757	308.31	
12.970	69.4525	34.3776	318.03	
12.972	69.1720	33.1779	326.36	
12.974	69.2349	33.5649	323.43	
12.976	69.1710	34.8337	311.34	
12.978	69.1238	34.9126	310.20	
12.980	69.5864	35.9921	304.60	
12.982	69.2989	36.3681	298.59	
12.984	69.5348	37.5500	290.01	
12.986	69.0932	35.6940	302.84	
12.988	69.2779	32.6134	332.44	
12.990	69.4480	33.3262	327.52	
12.992	69.3540	35.4419	307.49	
12.994	69.4110	35.5900	306.66	
12.996	69.3380	37.7432	286.47	
12.998	68.8472	33.5480	320.06	
13.000	69.2347	33.0718	327.89	
13.002	69.7351	36.3285	302.90	
13.004	68.8943	33.5164	320.78	
13.006	69.8265	37.3378	294.58	
13.008	68.8282	38.6382	273.74	
13.010	69.1083	34.3685	314.99	
13.012	69.3913	36.4618	298.58	
13.014	69.0865	35.2447	306.85	
13.016	69.4843	32.6961	333.56	
13.018	69.5415	38.5913	280.63	
13.020	68.7425	34.1539	313.62	
13.022	69.4898	35.6960	306.41	
13.024	69.7962	40.0815	269.43	
13.026	68.8216	33.8990	316.65	
13.028	70.0431	36.9533	300.03	
13.030	68.6849	35.5608	300.34	
13.032	69.0793	29.0094	363.32	
13.034	69.3844	32.2866	336.37	
13.036	69.0305	31.4006	341.20	
13.038	69.6852	32.3836	338.22	cooling
13.040	69.6517	43.6720	235.56	cooling
13.042	68.5336	37.5294	281.12	
13.044	69.5224	36.4154	300.19	
13.046	69.3885	38.7505	277.80	
13.048	68.6999	32.4136	329.01	
13.050	69.8835	36.9726	298.41	
13.052	68.5337	35.1159	303.00	
13.054	69.0225	30.4279	349.94	
13.056	69.1805	34.3962	315.39	
13.058	69.1806	33.7924	320.87	
13.060	69.7265	34.0950	323.08	
13.062	69.2897	39.5030	270.08	cooling
13.064	68.6821	34.4414	310.47	
13.066	69.4385	36.3929	299.63	
13.068	69.3030	40.2030	263.85	
13.070	68.7169	36.8416	289.02	
13.072	69.8246	39.6973	273.17	
13.074	68.6643	37.3228	284.18	
13.076	69.0608	33.7158	320.48	
13.078	69.0792	35.9355	300.52	
13.080	69.3086	35.9110	302.82	
13.082	69.6640	33.8888	324.38	
13.084	69.0935	33.7445	320.51	
13.086	69.1410	31.4630	341.63	
13.088	69.1664	32.4501	332.91	
13.090	69.2961	34.0356	319.71	
13.092	69.0106	34.2712	314.99	
13.094	69.4698	35.3245	309.60	
13.096	69.0727	34.7449	311.26	
13.098	69.1171	36.3794	296.84	
13.100	68.7570	35.2047	304.22	
13.102	69.3409	34.4676	316.20	
13.104	69.3624	32.2208	336.77	
13.106	69.0147	31.8202	337.25	cooling
13.108	69.3985	35.9575	303.21	cooling
13.110	68.6284	35.6072	299.41	cooling
13.112	69.1145	34.2786	315.86	cooling
13.114	68.9226	36.8104	291.17	cooling
13.116	68.9622	35.8798	299.96	cooling
13.118	69.0718	36.8240	292.40	
13.120	68.8311	39.5833	265.19	
13.122	68.4746	36.3659	291.13	
13.124	69.2126	38.9763	274.16	
13.126	69.0355	38.6027	275.94	
13.128	68.7922	37.3628	284.97	
13.130	69.2373	41.0463	255.61	
13.132	68.3787	37.3945	280.94	
13.134	68.9984	35.0178	308.11	
13.136	68.8950	38.2674	277.70	
13.138	68.6893	35.6670	299.42	
13.140	69.1730	35.8867	301.81	
13.142	68.8934	39.2823	268.49	
13.144	68.3804	34.6736	305.62	
13.146	69.3260	35.6302	305.53	
13.148	68.7214	31.8862	333.99	
13.150	68.8850	28.0764	370.02	
13.152	69.3482	34.4953	316.02	
13.154	68.4674	34.5733	307.32	
13.156	68.9143	33.3357	322.60	
13.158	68.8954	35.4839	302.95	
13.160	68.7842	30.8330	344.11	
13.162	69.3886	31.4191	344.27	
13.164	68.9602	34.2662	314.58	
13.166	68.4328	29.6110	352.00	
13.168	69.3277	31.8212	340.08	cooling
13.170	68.6514	30.8004	343.20	cooling
13.172	68.7279	27.7271	371.76	cooling
13.174	69.1899	34.5342	314.23	cooling
13.176	68.1732	33.0485	318.48	cooling
13.178	68.5237	29.7542	351.53	cooling
13.180	68.7707	33.6935	318.05	cooling
13.182	68.4238	30.6417	342.58	cooling
13.184	69.0212	34.4818	313.17	cooling
13.186	68.3653	37.9809	275.50	cooling
13.188	68.0056	33.5601	312.32	cooling
13.190	68.7953	36.7488	290.57	cooling
13.192	68.0340	36.5410	285.55	cooling
13.194	68.2229	33.4716	315.10	cooling
13.196	68.7736	39.3284	266.98	cooling
13.198	67.7769	37.3658	275.74	cooling
13.200	67.9779	34.8203	300.64	cooling
13.202	68.1919	37.6653	276.79	cooling
13.204	67.8194	33.1224	314.60	
13.206	68.5728	35.4887	299.98	
13.208	67.9482	37.5471	275.65	
13.210	67.8933	33.7795	309.31	
13.212	68.3533	35.9383	293.91	
13.214	67.5644	33.7343	306.74	
13.216	68.2863	32.7141	322.54	
13.218	68.3255	36.2484	290.85	
13.220	67.9514	34.7021	301.48	
13.222	67.9470	33.2575	314.54	
13.224	68.0416	33.6742	311.61	
13.226	67.9588	30.8390	336.57	
13.228	68.2926	32.8318	321.53	
13.230	67.8406	32.9635	316.24	
13.232	67.9266	32.1027	324.82	
13.234	67.9897	34.5991	302.76	cooling
13.236	67.6322	34.9360	296.46	cooling
13.238	68.0615	35.3823	296.31	
13.240	68.1079	37.2731	279.58	
13.242	67.9366	35.6942	292.35	
13.244	67.8719	34.6389	301.33	
13.246	67.7820	33.8375	307.78	
13.248	68.0122	33.6558	311.51	

13.250	67.9719	34.2858	305.44	
13.252	67.9558	34.8584	300.10	
13.254	67.9389	36.3395	286.52	
13.256	67.8530	37.4919	275.29	
13.258	67.7468	35.9389	288.41	
13.260	67.7569	32.8773	316.26	
13.262	68.1332	31.8860	328.66	
13.264	68.0840	33.1884	316.40	
13.266	67.8875	35.6594	292.22	
13.268	67.6666	34.4888	300.83	
13.270	68.0046	33.6092	311.87	
13.272	67.9856	33.8290	309.70	
13.274	67.8713	34.9235	298.74	
13.276	68.0969	36.5784	285.78	
13.278	67.6889	35.3612	293.12	
13.280	67.7854	33.4711	311.13	
13.282	67.7808	33.2771	312.85	
13.284	68.1597	32.4654	323.65	
13.286	68.1344	34.1659	308.00	
13.288	67.7626	34.7561	299.27	
13.290	67.6212	32.0253	322.75	
13.292	68.1238	34.1051	308.45	
13.294	67.7307	33.9616	306.19	
13.296	67.9074	35.3894	294.85	
13.298	68.2313	37.2774	280.66	
13.300	67.5556	34.1120	303.24	
13.302	67.9663	34.5368	303.11	
13.304	67.7974	36.2956	285.63	
13.306	68.1128	32.5048	322.86	
13.308	68.1517	32.3929	324.23	
13.310	67.7670	33.7258	308.66	
13.312	67.6673	33.0062	314.28	
13.314	68.2227	36.5566	287.12	
13.316	67.3146	32.5913	314.84	
13.318	68.2590	32.2708	326.31	
13.320	68.4298	35.9975	294.07	
13.322	67.6490	34.5428	300.18	
13.324	68.0253	36.1403	289.11	
13.326	67.8213	38.4200	266.59	
13.328	68.2069	34.8111	302.80	
13.330	68.2505	37.7921	276.17	
13.332	67.8151	40.2732	249.73	
13.334	67.6549	36.7944	279.82	
13.336	68.2016	38.3402	270.76	
13.338	67.3111	34.5355	297.18	
13.340	68.2775	35.4673	297.49	
13.342	68.4756	41.0119	249.02	
13.344	67.5881	39.4007	255.58	
13.346	68.0105	39.8512	255.32	
13.348	67.8660	41.9226	235.23	
13.350	68.2682	38.8526	266.72	heating
13.352	68.1398	40.7041	248.76	heating
13.354	67.9483	39.3316	259.47	heating
13.356	67.8365	36.3421	285.56	heating
13.358	68.3499	39.5724	260.93	heating
13.360	67.5806	37.1015	276.36	heating
13.362	68.4855	37.2212	283.48	heating
13.364	68.5801	37.4635	282.14	heating
13.366	67.8339	33.5048	311.27	heating
13.368	68.4818	35.5709	298.41	heating
13.370	68.1354	38.4854	268.84	heating
13.372	68.5620	35.8868	296.27	heating
13.374	68.5201	35.5626	298.83	heating
13.376	68.4047	34.1633	310.47	heating
13.378	68.3847	31.4887	334.54	heating
13.380	68.6295	32.6207	326.50	heating
13.382	68.0467	27.7888	365.02	
13.384	68.7072	27.5753	372.95	
13.386	68.9473	31.0972	343.19	
13.388	68.2477	32.0077	328.59	
13.390	68.5062	32.5211	326.28	
13.392	68.1709	31.1220	335.93	
13.394	68.7127	27.5314	373.40	
13.396	68.5069	25.9833	385.57	
13.398	68.5918	28.1032	367.12	
13.400	68.3501	30.5920	342.36	cooling
13.402	68.4248	34.2802	309.59	cooling
13.404	68.0042	31.3045	332.76	cooling
13.406	68.3643	30.7286	341.25	cooling
13.408	68.5977	32.2734	329.36	cooling
13.410	68.1044	34.2390	307.06	cooling
13.412	68.1298	35.0578	299.87	cooling
13.414	67.8239	34.6479	300.81	cooling
13.416	68.5431	37.3707	282.64	cooling
13.418	67.9495	38.1521	270.18	cooling
13.420	68.1509	38.4072	269.69	cooling
13.422	67.9731	37.4267	276.97	
13.424	67.9867	36.4523	285.93	

13.426	67.8440	34.9432	298.32	
13.428	68.0696	36.4218	286.96	
13.430	68.2149	36.2416	289.91	
13.432	68.0824	38.4939	268.28	
13.434	67.9756	41.5808	239.33	
13.436	67.8189	39.9063	253.09	
13.438	68.4072	39.7896	259.48	
13.440	67.7870	37.4013	275.51	
13.442	68.0387	35.4696	295.31	
13.444	68.2338	37.0574	282.68	
13.446	67.7684	36.3258	285.09	
13.448	67.9373	37.5219	275.78	
13.450	68.0476	40.8094	246.97	
13.452	68.0824	39.0503	263.24	
13.454	68.0797	39.1572	262.24	
13.456	67.8890	38.4089	267.30	heating
13.458	68.0934	35.3987	296.45	heating
13.460	68.4198	38.0478	275.39	heating
13.462	67.9335	39.6273	256.66	heating
13.464	68.2110	40.1663	254.29	heating
13.466	68.5209	41.9378	241.03	heating
13.468	68.0289	38.4518	268.18	heating
13.470	68.3124	37.9240	275.54	heating
13.472	68.3374	38.4188	271.28	heating
13.474	68.4396	36.8073	286.81	heating
13.476	68.4683	38.1933	274.51	heating
13.478	68.3596	37.9883	275.38	heating
13.480	68.6971	37.6404	281.60	heating
13.482	68.6970	39.3442	266.15	heating
13.484	68.3062	36.8724	285.01	heating
13.486	68.7962	35.3929	302.87	heating
13.488	68.9831	35.5331	303.30	heating
13.490	68.7217	37.3260	284.67	heating
13.492	68.7801	40.7736	253.94	heating
13.494	68.6586	39.5436	263.99	heating
13.496	68.8153	36.2311	295.45	heating
13.498	68.9081	35.4521	303.35	heating
13.500	68.7400	33.7599	317.17	heating
13.502	69.1266	33.8847	319.54	heating
13.504	68.8489	35.1860	305.23	heating
13.506	68.9210	36.7984	291.26	heating
13.508	68.9035	36.9593	289.64	heating
13.510	69.2192	36.5549	296.17	heating
13.512	68.9092	36.4436	294.37	heating
13.514	68.9806	36.8306	291.51	heating
13.516	68.9316	35.8316	300.12	heating
13.518	69.1845	36.2270	298.83	heating
13.520	68.9775	35.5542	303.05	heating
13.522	69.4033	38.1035	283.80	heating
13.524	69.3680	39.9797	266.47	heating
13.526	69.0921	39.6282	267.15	heating
13.528	69.3807	40.3784	262.97	heating
13.530	69.1205	38.1273	281.02	heating
13.532	69.6087	34.2009	321.05	heating
13.534	69.4948	34.0551	321.34	heating
13.536	69.4429	35.6003	306.86	heating
13.538	69.4676	35.8345	304.96	heating
13.540	69.6930	37.3310	293.43	heating
13.542	69.3779	34.4842	316.39	heating
13.544	69.8855	36.3894	303.71	
13.546	69.7935	38.6665	282.23	
13.548	69.2634	34.8310	312.20	
13.550	69.7279	34.2781	321.43	
13.552	69.2277	32.9972	328.51	
13.554	69.8986	31.9159	344.39	
13.556	69.7620	35.7596	308.30	
13.558	69.4447	34.5121	316.74	
13.560	69.5309	31.6059	343.87	
13.562	69.8483	33.1628	332.63	
13.564	69.1347	28.6175	367.38	
13.566	69.8129	30.8413	353.36	
13.568	69.8047	34.9807	315.75	
13.570	69.2771	32.8247	330.52	
13.572	69.6182	32.6441	335.25	
13.574	69.4618	34.5892	316.19	
13.576	69.6783	33.8439	324.92	
13.578	69.7508	37.9769	288.10	cooling
13.580	69.4501	36.5821	298.02	
13.582	69.4935	34.8987	313.68	
13.584	69.9303	38.5486	284.54	
13.586	68.9948	34.4499	313.22	
13.588	69.8985	35.1341	315.21	
13.590	69.7190	38.0444	287.20	
13.592	69.3344	37.0111	293.08	
13.594	69.6770	39.5610	273.07	
13.596	69.3871	40.3254	263.51	
13.598	69.5716	35.6098	307.94	
13.600	69.6972	38.3508	284.22	

13.602	69.6407	40.0624	268.19	
13.604	69.5482	37.8357	287.54	
13.606	69.8825	40.2933	268.29	
13.608	69.0335	36.3599	296.26	
13.610	69.9835	35.1862	315.51	
13.612	69.6656	38.3478	283.96	
13.614	69.1968	36.0213	300.81	
13.616	69.4735	36.0148	303.37	
13.618	69.4648	38.1985	283.50	
13.620	69.3390	36.2033	300.45	
13.622	69.7044	39.6102	272.87	
13.624	69.8165	40.1750	268.76	
13.626	69.4516	36.1482	301.97	
13.628	69.7324	37.3802	293.34	
13.630	69.3997	37.9844	284.85	
13.632	69.6859	35.3133	311.66	
13.634	69.9216	37.0283	298.25	
13.636	69.3921	38.4697	280.38	
13.638	69.4558	39.0064	276.09	
13.640	69.7314	43.1737	240.80	
13.642	69.2768	41.1900	254.67	
13.644	69.6466	37.6089	290.49	
13.646	69.8722	36.0379	306.78	
13.648	69.7371	34.3829	320.56	
13.650	69.6652	35.2206	312.31	
13.652	69.5576	36.5752	299.06	
13.654	69.5139	34.2566	319.68	
13.656	69.9777	33.8217	327.83	
13.658	69.5964	35.3429	310.58	
13.660	69.5068	35.1250	311.75	
13.662	69.6380	35.7851	306.95	
13.664	69.4828	34.4850	317.33	
13.666	69.4906	30.2315	355.97	
13.668	70.0207	33.2975	332.97	
13.670	69.7659	34.8035	317.01	
13.672	69.4371	33.8490	322.68	
13.674	69.7709	34.5296	319.54	
13.676	69.2838	30.5842	350.89	
13.678	70.0066	28.4668	376.65	
13.680	69.5633	33.1596	330.08	cooling
13.682	69.4088	34.5813	315.79	cooling
13.684	69.4389	33.8719	322.49	cooling
13.686	69.6718	36.1842	303.64	cooling
13.688	69.1028	33.5969	321.94	cooling
13.690	69.7523	37.2854	294.38	cooling
13.692	69.4010	37.7822	286.69	cooling
13.694	69.0310	33.2467	324.46	cooling
13.696	69.4493	34.2091	319.53	cooling
13.698	68.8798	34.2667	313.84	cooling
13.700	69.3489	32.5442	333.71	cooling
13.702	69.2884	34.4692	315.71	cooling
13.704	68.9507	34.1210	315.81	cooling
13.706	69.0622	33.7853	319.86	cooling
13.708	69.2343	37.0517	291.80	cooling
13.710	68.4946	32.5057	326.32	cooling
13.712	69.2033	32.8860	329.29	cooling
13.714	69.0946	36.3225	297.15	cooling
13.716	68.6238	32.6369	326.30	cooling
13.718	69.0647	34.8947	309.82	cooling
13.720	68.5139	35.5611	298.79	cooling
13.722	68.6393	31.6228	335.63	cooling
13.724	69.1099	35.8705	301.39	cooling
13.726	68.4677	34.5580	307.46	cooling
13.728	68.6115	32.1277	330.80	cooling
13.730	68.7451	35.0628	305.40	cooling
13.732	68.0565	31.4897	331.56	cooling
13.734	68.7319	30.9396	342.67	cooling
13.736	68.5380	35.3389	301.02	cooling
13.738	68.1283	33.3319	315.50	cooling
13.740	68.3922	36.1557	292.29	cooling
13.742	68.2675	39.9560	256.70	cooling
13.744	67.7354	34.4871	301.47	cooling
13.746	68.6018	36.0456	295.19	cooling
13.748	68.0993	35.0576	299.59	cooling
13.750	68.0748	32.9176	318.78	cooling
13.752	68.2921	36.7820	285.71	cooling
13.754	67.5833	34.9666	295.74	
13.756	68.3710	34.2608	309.28	
13.758	68.1389	37.9584	273.65	
13.760	67.8991	36.2074	287.35	
13.762	68.1299	38.0913	272.36	
13.764	68.0341	39.5136	258.60	
13.766	67.4930	33.5546	307.72	
13.768	68.6354	36.5657	290.78	
13.770	68.0049	38.4054	268.38	
13.772	68.1572	38.3562	270.21	
13.774	68.0568	39.7875	256.32	
13.776	67.7482	35.6730	290.83	
13.778	68.3724	32.8868	321.75	
13.780	68.1225	35.8501	292.62	
13.782	67.9659	36.3223	286.92	
13.784	67.9998	38.3731	268.63	
13.786	67.8545	38.5587	265.63	
13.788	67.7336	35.9567	288.13	
13.790	68.4133	37.3085	282.03	
13.792	67.9860	34.4379	304.19	
13.794	68.1859	33.1291	317.87	
13.796	67.9149	33.4411	312.58	
13.798	67.8338	30.5713	337.86	
13.800	68.0890	30.8200	337.92	
13.802	68.2395	35.4444	297.36	
13.804	68.1508	36.4093	287.80	
13.806	68.0997	38.4685	268.67	
13.808	67.7793	35.2060	295.35	
13.810	67.9085	31.9178	326.33	
13.812	68.4907	32.4017	327.22	
13.814	68.0543	30.4966	340.54	
13.816	68.3278	34.8455	303.59	
13.818	67.8779	39.3280	258.87	
13.820	67.9985	39.8529	255.20	
13.822	68.0798	39.5641	258.56	
13.824	68.0231	36.5270	285.58	
13.826	68.3575	35.5372	297.59	
13.828	68.1928	37.9649	274.08	
13.830	67.7683	35.1119	296.10	
13.832	68.0972	35.0382	299.75	
13.834	68.4002	38.6533	269.72	
13.836	68.1576	39.0800	263.65	
13.838	68.3737	41.3712	244.84	
13.840	67.7496	37.9842	269.89	
13.842	68.0497	34.3187	305.84	heating
13.844	68.2908	37.0205	283.53	
13.846	68.0135	37.6569	275.25	
13.848	68.6063	40.1899	257.66	heating
13.850	68.1868	42.0062	237.38	heating
13.852	68.0637	38.4986	268.07	heating
13.854	68.3856	39.5353	261.59	heating
13.856	68.4615	40.5136	253.41	heating
13.858	68.2362	36.4009	288.66	heating
13.860	68.6780	37.4610	283.05	heating
13.862	68.3485	37.4053	280.57	heating
13.864	68.4533	36.9476	285.67	heating
13.866	68.9305	41.2838	250.68	heating
13.868	68.3824	38.4069	271.79	heating
13.870	69.0375	33.2516	324.48	heating
13.872	68.9226	33.9207	317.37	heating
13.874	68.7798	32.4889	329.05	heating
13.876	68.9805	34.9711	308.37	heating
13.878	68.8259	37.9796	279.69	heating
13.880	68.6899	34.3839	311.06	heating
13.882	69.3067	37.5179	288.23	heating
13.884	68.8298	37.1192	287.52	heating
13.886	68.8908	34.4068	312.67	heating
13.888	69.1560	34.0506	318.31	heating
13.890	68.8543	30.1213	351.20	heating
13.892	69.3433	28.9626	366.14	
13.894	69.2458	34.8420	311.94	
13.896	69.1404	37.2876	288.81	
13.898	68.8690	37.9780	280.09	
13.900	69.2701	39.4842	270.07	
13.902	68.7414	34.8345	307.44	
13.904	69.6339	35.5078	309.43	
13.906	69.0287	36.1731	297.91	
13.908	68.9541	35.8121	300.50	
13.910	69.1940	37.6656	285.87	
13.912	68.9213	37.8503	281.73	
13.914	69.2564	36.2339	299.42	
13.916	69.2691	38.8734	275.60	
13.918	69.1592	39.0508	273.00	
13.920	68.9443	38.6741	274.46	
13.922	69.2686	40.5346	260.54	
13.924	68.9996	38.7333	274.43	
13.926	69.5490	38.3785	282.63	heating
13.928	69.2754	39.4749	270.21	heating
13.930	69.1205	39.5688	267.95	heating
13.932	69.2147	37.7205	285.56	heating
13.934	69.3585	36.0981	301.58	heating
13.936	69.5235	33.0424	330.78	heating
13.938	69.5650	34.2981	319.77	heating
13.940	69.5970	38.3723	283.12	
13.942	69.0280	36.0378	299.13	
13.944	69.4625	34.0850	320.77	
13.946	69.5254	33.3836	327.70	
13.948	69.6070	31.9426	341.51	
13.950	69.8725	37.3172	295.18	
13.952	69.2292	38.2215	281.15	

13.954	69.0995	32.1999	334.57	
13.956	69.5937	29.9719	359.26	
13.958	69.3375	27.1273	382.73	
13.960	69.6624	27.8210	379.38	
13.962	69.5223	33.4955	326.66	
13.964	68.9993	32.5815	330.21	
13.966	69.1261	30.1334	353.55	
13.968	69.7799	34.6093	318.90	cooling
13.970	69.2548	35.1174	309.53	cooling
13.972	69.7131	37.3370	293.56	cooling
13.974	69.1139	37.7304	284.56	cooling
13.976	68.9104	31.7346	337.08	
13.978	69.4211	31.3483	345.21	cooling
13.980	69.2415	34.3155	316.68	cooling
13.982	69.3694	33.5414	324.86	cooling
13.984	69.3646	38.5174	279.70	cooling
13.986	68.8631	37.8740	280.98	cooling
13.988	68.6127	33.8709	315.01	cooling
13.990	69.7970	38.9344	279.84	cooling
13.992	68.6039	37.4118	282.82	
13.994	69.3160	37.4934	288.54	
13.996	69.1010	42.8154	238.34	
13.998	68.6845	41.5083	246.41	
14.000	69.1135	41.8775	246.95	
14.002	69.2635	46.4133	207.19	
14.004	68.9664	37.6159	284.26	
14.006	69.4055	38.7379	278.07	
14.008	68.8483	37.3286	285.79	
14.010	68.4849	31.5474	334.92	
14.012	70.1195	39.9405	273.64	
14.014	68.1867	38.7044	267.32	
14.016	69.6580	37.6652	290.08	
14.018	69.1564	40.4448	260.33	
14.020	68.7030	33.7288	317.12	
14.022	69.1839	31.9615	337.50	
14.024	69.4147	37.9959	284.88	
14.026	68.8101	30.1407	350.62	
14.028	69.5355	35.1350	311.91	
14.030	68.9384	37.4511	285.50	
14.032	68.5080	32.4387	327.05	
14.034	69.7787	38.2625	285.76	
14.036	67.9908	34.1585	306.76	
14.038	69.5177	30.1886	356.60	
14.040	69.2848	35.5221	306.13	
14.042	68.6243	34.2168	311.98	
14.044	69.1096	35.4461	305.23	
14.046	69.2581	40.2898	262.66	
14.048	68.6992	31.8086	334.49	
14.050	69.4135	34.4853	316.70	
14.052	68.9778	36.4275	295.14	
14.054	68.7157	32.8810	324.92	
14.056	69.5220	38.3625	282.53	
14.058	68.3149	36.9041	284.81	
14.060	69.6048	36.8116	297.34	
14.062	69.3978	42.3467	245.28	
14.064	68.6520	39.3085	266.06	
14.066	69.0594	36.5761	294.53	
14.068	69.1737	37.9061	283.51	
14.070	68.8852	33.5890	320.04	
14.072	69.2505	35.1539	309.16	
14.074	69.1196	37.1708	289.68	
14.076	68.8356	36.4308	293.82	
14.078	69.1218	38.4612	278.00	
14.080	68.7119	35.9992	296.61	
14.082	69.3878	32.5945	333.61	
14.084	69.3190	34.3169	317.37	
14.086	68.8225	34.5761	310.52	
14.088	68.8446	34.4323	312.02	
14.090	69.0870	35.9862	300.13	
14.092	69.0145	35.8754	300.48	
14.094	69.0887	35.1658	307.58	
14.096	69.1375	34.7733	311.59	
14.098	68.9691	35.0403	307.64	
14.100	68.7148	34.2172	312.79	
14.102	69.0778	34.9043	309.86	
14.104	69.2339	34.1946	317.71	
14.106	69.1589	31.1602	344.54	
14.108	69.1683	35.0703	309.17	
14.110	68.7198	35.2740	303.26	
14.112	68.8595	31.6160	337.69	
14.114	69.4065	34.3150	318.18	
14.116	68.7160	32.7251	326.33	
14.118	69.4529	36.2701	300.87	
14.120	69.0687	40.0839	262.81	
14.122	68.5282	33.9794	313.26	
14.124	69.3135	34.3508	317.01	
14.126	69.0313	37.0383	290.09	
14.128	69.1481	33.3430	324.65	

14.130	69.4658	41.2943	255.44	
14.132	68.6403	40.9740	250.85	
14.134	68.6578	33.4284	319.43	
14.136	69.7901	38.3164	285.38	
14.138	68.3728	34.8891	303.60	
14.140	69.4610	36.2691	300.96	
14.142	69.0333	41.1878	252.48	
14.144	68.5204	35.2635	301.55	
14.146	69.3658	37.8822	285.47	
14.148	68.9911	42.6999	238.39	
14.150	69.0741	35.9169	300.64	
14.152	69.3779	38.4678	280.27	
14.154	68.6649	34.2587	311.97	
14.156	68.7555	29.1870	358.77	
14.158	69.9422	36.2263	305.71	
14.160	68.2471	31.2707	335.27	
14.162	69.6195	31.7222	343.62	
14.164	68.9814	35.4808	303.75	
14.166	68.7921	29.4128	357.06	
14.168	69.3500	32.2981	335.95	
14.170	69.0397	36.5218	294.84	
14.172	69.0731	28.8121	365.05	
14.174	69.6400	33.8973	324.08	
14.176	68.6040	33.1979	321.03	
14.178	68.8316	32.0365	333.63	cooling
14.180	69.5383	38.7836	278.86	cooling
14.182	68.1096	31.4868	332.06	cooling
14.184	69.4080	31.9020	340.07	cooling
14.186	68.8238	37.0774	287.85	cooling
14.188	68.5622	35.8307	296.78	cooling
14.190	68.9403	40.0731	261.74	cooling
14.192	68.4592	40.4975	253.53	
14.194	68.8927	35.9368	298.82	
14.196	69.1115	40.0249	263.73	
14.198	68.4045	37.4268	280.88	
14.200	68.8056	37.4047	284.72	
14.202	68.9171	40.9876	253.24	
14.204	68.3271	37.2447	281.83	
14.206	69.2649	40.6102	259.82	
14.208	68.6608	42.9870	232.79	
14.210	68.6861	40.8713	252.20	
14.212	68.8186	42.0238	242.95	
14.214	68.4913	38.1300	275.29	
14.216	69.1067	35.2341	307.13	heating
14.218	68.8948	37.3332	286.17	
14.220	68.5780	34.7870	306.39	
14.222	68.9720	37.6123	284.34	
14.224	68.7859	38.4105	275.42	
14.226	68.5440	33.5160	317.60	heating
14.228	69.2609	35.5855	305.34	
14.230	68.6024	34.0236	313.53	
14.232	68.9213	34.3368	313.58	
14.234	68.7099	35.5589	300.59	
14.236	68.6925	33.7576	316.76	
14.238	69.4221	34.9522	312.54	
14.240	68.4344	33.3976	317.68	
14.242	69.0826	33.0261	326.93	
14.244	68.8060	37.2964	285.70	
14.246	68.7667	35.0061	306.11	
14.248	68.7695	32.6149	327.82	
14.250	69.0818	33.1704	325.61	
14.252	68.6656	30.3615	347.31	
14.254	69.1955	36.3030	298.24	
14.256	68.4436	37.0743	284.43	
14.258	68.9090	37.0161	289.18	
14.260	69.4140	41.2960	254.95	
14.262	68.1952	36.8645	284.08	
14.264	69.3628	35.7628	304.66	
14.266	68.6635	38.8871	269.99	
14.268	68.8254	34.8975	307.63	
14.270	68.8908	35.8975	299.15	
14.272	69.0803	36.8832	291.94	
14.274	68.7425	32.3193	330.25	
14.276	69.4084	38.8395	277.17	
14.278	68.2857	35.0080	301.73	
14.280	69.0402	34.0232	317.50	
14.282	69.3148	38.9313	275.49	
14.284	68.3073	36.4720	288.66	
14.286	69.5302	38.4658	281.67	
14.288	68.6035	40.6869	253.12	
14.290	68.8067	35.8952	298.41	
14.292	68.9885	36.8412	291.48	
14.294	68.9801	36.0194	298.86	
14.296	68.7296	30.9317	342.72	
14.298	69.3348	36.3389	299.18	
14.300	68.4121	32.8307	322.62	
14.302	69.0329	32.5989	330.35	
14.304	69.0467	36.7217	293.10	

14.306	68.3958	34.1798	310.24	
14.308	69.5571	36.2297	302.18	
14.310	68.5109	35.8271	296.35	
14.312	68.8461	33.5812	319.75	
14.314	68.9661	38.0504	280.32	
14.316	68.8279	38.3884	276.00	
14.318	68.7631	35.7589	299.25	
14.320	68.9739	38.2973	278.15	
14.322	68.6485	35.9214	296.74	
14.324	68.8796	36.0679	297.51	
14.326	68.8907	37.9045	280.96	
14.328	68.5114	34.6953	306.62	
14.330	69.1878	35.0332	309.68	
14.332	68.7769	35.3026	303.52	
14.334	68.8028	35.2302	304.41	
14.336	68.8820	36.7747	291.12	
14.338	68.8626	37.5871	283.58	
14.340	68.8779	36.6414	292.29	
14.342	68.8167	37.4189	284.69	
14.344	69.0355	36.5485	294.56	
14.346	68.8539	35.8313	299.42	
14.348	68.7935	33.8339	316.98	
14.350	68.7013	32.7885	325.63	
14.352	69.1400	34.5544	313.59	
14.354	68.8606	33.3015	322.42	
14.356	68.9686	37.3519	286.67	
14.358	68.6895	38.3286	275.29	
14.360	68.6374	35.6052	299.51	
14.362	69.1391	36.5115	295.84	
14.364	68.1775	32.7051	321.63	
14.366	69.1912	32.2164	335.26	
14.368	68.7573	35.5336	301.24	
14.370	68.5712	33.4116	318.80	
14.372	68.8914	35.4594	303.13	
14.374	69.0294	38.7770	274.30	
14.376	68.7266	32.3747	329.61	
14.378	69.1350	35.3195	306.61	
14.380	68.5774	33.5432	317.66	
14.382	68.6230	28.5805	363.07	
14.384	69.3882	31.1916	346.33	
14.386	68.0556	28.9704	354.39	
14.388	69.4912	31.6885	342.76	
14.390	68.7040	38.2504	276.13	cooling
14.392	68.5248	34.7637	306.12	
14.394	68.7840	34.9295	306.96	
14.396	69.1849	38.5884	277.42	
14.398	68.5073	29.5134	353.56	
14.400	69.3530	34.8218	313.10	cooling
14.402	68.3840	35.9871	293.75	cooling
14.404	68.5486	33.0877	321.53	
14.406	69.3955	37.8287	286.22	cooling
14.408	67.6909	32.8381	316.02	
14.410	69.3187	32.1131	337.35	
14.412	68.6003	37.7450	279.77	cooling
14.414	68.4899	31.8789	331.96	
14.416	68.5801	31.1064	339.78	cooling
14.418	68.9374	36.5809	293.38	cooling
14.420	68.2687	31.4373	333.96	cooling
14.422	69.0528	37.0476	290.20	cooling
14.424	68.0876	35.4709	295.74	cooling
14.426	68.1390	31.8497	329.04	cooling
14.428	69.0660	37.1857	289.06	cooling
14.430	67.5758	34.0923	303.60	cooling
14.432	68.7022	30.3921	347.36	cooling
14.434	68.3304	34.6206	305.65	cooling
14.436	68.0893	33.1370	316.92	cooling
14.438	68.1086	33.4823	313.96	cooling
14.440	68.3226	37.1809	282.37	cooling
14.442	67.7607	31.4801	328.96	cooling
14.444	68.4564	34.5284	307.63	cooling
14.446	67.6912	33.3385	311.48	cooling
14.448	67.7359	30.9028	333.97	cooling
14.450	68.2991	35.1230	300.81	cooling
14.452	67.3360	34.0008	302.25	cooling
14.454	67.9757	32.0989	325.30	cooling
14.456	68.0404	36.1781	288.90	cooling
14.458	67.8536	36.4599	284.65	cooling
14.460	67.7151	34.4898	301.26	cooling
14.462	67.9326	36.5416	284.63	
14.464	67.7020	34.3559	302.35	
14.466	67.8832	35.0040	298.12	
14.468	67.9504	38.3947	267.99	
14.470	67.5764	39.7191	252.59	
14.472	68.0339	41.2872	242.52	
14.474	67.4860	39.8349	250.72	
14.476	67.9542	37.7167	274.17	
14.478	67.6831	35.0401	295.98	
14.480	68.1067	37.3140	279.20	
14.482	67.6355	38.7710	261.72	
14.484	67.6997	40.5125	246.51	
14.486	68.0819	43.6892	221.17	
14.488	67.4837	41.8581	232.35	
14.490	68.2322	41.2769	244.41	heating
14.492	67.6945	39.5670	255.04	heating
14.494	67.8645	32.9644	316.44	heating
14.496	68.0663	33.1179	316.88	heating
14.498	67.9542	34.5006	303.33	heating
14.500	67.9478	32.0397	325.58	heating
14.502	68.7085	40.5867	254.98	heating
14.504	67.7902	39.1929	259.30	heating
14.506	67.9801	33.4480	313.11	heating
14.508	68.8348	34.7965	308.63	heating
14.510	67.7667	28.3715	357.20	heating
14.512	68.7697	26.9371	379.30	
14.514	68.3625	33.3112	317.81	
14.516	67.9778	31.6131	329.72	
14.518	68.4764	34.9722	303.79	
14.520	68.4128	38.6886	269.51	
14.522	67.8208	28.2218	359.05	
14.524	68.9763	31.8189	336.91	
14.526	67.8425	29.9931	343.19	
14.528	68.0564	24.0987	398.57	
14.530	68.9728	30.7892	346.22	
14.532	67.4873	29.7935	341.78	
14.534	68.8301	29.7026	354.77	cooling
14.536	68.3309	36.9781	284.28	cooling
14.538	67.8031	32.0967	323.76	cooling
14.540	68.3379	32.6997	323.14	cooling
14.542	68.2681	36.5608	287.49	cooling
14.544	67.4995	28.2102	356.24	cooling
14.546	68.7823	35.6284	300.61	cooling
14.548	67.8727	39.4603	257.62	cooling
14.550	67.8370	35.6426	291.91	cooling
14.552	68.4553	39.2026	265.24	
14.554	67.4261	35.6185	288.40	
14.556	68.3519	32.8943	321.50	
14.558	68.3083	40.0453	256.26	
14.560	67.6036	36.8791	278.58	
14.562	68.1736	37.8683	274.78	
14.564	68.1509	42.8823	229.11	
14.566	67.3861	36.5896	279.24	
14.568	68.7529	40.0024	260.69	
14.570	67.8825	40.7986	245.57	
14.572	67.8821	36.0304	288.80	
14.574	68.3529	38.6703	269.14	
14.576	67.5512	35.2445	292.93	
14.578	68.3380	33.8277	312.91	
14.580	68.0515	38.3675	269.15	
14.582	67.6341	34.2587	302.62	
14.584	68.1558	36.1398	290.29	
14.586	67.8583	39.2636	259.27	
14.588	67.5401	34.2155	302.16	
14.590	68.3733	35.2797	300.06	
14.592	67.9811	35.0986	298.15	
14.594	68.0010	34.0048	308.25	
14.596	68.0892	37.4807	277.53	
14.598	67.7354	35.9219	288.46	
14.600	68.2205	35.0052	301.17	
14.602	68.1446	36.2536	289.16	
14.604	67.8117	36.1121	287.43	
14.606	68.1526	38.2814	270.85	
14.608	67.5766	37.5031	272.68	
14.610	68.0255	36.0728	289.72	
14.612	68.0374	34.8721	300.71	
14.614	68.2705	33.6387	314.01	
14.616	68.2123	36.1458	290.75	
14.618	67.7934	37.8641	271.37	
14.620	67.8180	36.1213	287.40	
14.622	68.2306	38.7596	267.22	
14.624	68.2282	36.7977	284.99	heating
14.626	68.1740	36.2504	289.46	heating
14.628	68.2357	36.2425	290.09	heating
14.630	67.7683	33.4388	311.27	heating
14.632	68.6685	37.5038	282.57	
14.634	68.0149	37.9742	272.38	heating
14.636	68.8778	36.5761	292.88	heating
14.638	68.5930	38.0797	276.67	heating
14.640	68.0912	34.2779	306.59	heating
14.642	68.2570	29.8648	348.11	heating
14.644	68.8209	32.1810	332.22	
14.646	68.3897	27.3550	372.07	
14.648	68.6217	30.9124	341.92	
14.650	68.4102	35.3276	299.96	
14.652	67.8883	33.0534	315.85	
14.654	68.8256	35.6318	300.97	
14.656	67.8271	31.6547	327.98	

14.658	68.8155	26.7429	381.48	
14.660	68.6577	31.6250	335.78	
14.662	68.1306	32.3642	324.30	
14.664	68.2225	31.2892	334.88	
14.666	68.8284	35.5842	301.43	
14.668	68.2280	29.3252	352.74	
14.670	68.7411	29.5646	355.22	
14.672	68.4647	31.0018	339.68	cooling
14.674	67.9320	27.2792	368.61	cooling
14.676	68.7835	31.0758	341.90	cooling
14.678	68.0405	34.1288	307.48	cooling
14.680	68.6842	35.8575	297.64	cooling
14.682	68.6260	42.9573	232.74	cooling
14.684	67.7894	41.0365	242.57	cooling
14.686	68.0863	37.0309	281.58	
14.688	68.2824	37.8766	275.69	
14.690	68.1079	34.1982	307.46	
14.692	68.6639	37.6360	281.33	
14.694	68.2512	41.3407	244.00	
14.696	67.8801	36.3082	286.27	
14.698	68.6228	38.3093	274.86	
14.700	67.9355	37.3791	277.06	
14.702	68.5348	36.9425	286.45	
14.704	68.4457	41.5021	244.30	
14.706	67.7210	38.0222	269.28	
14.708	68.2882	37.9845	274.77	
14.710	68.0537	40.1344	253.15	
14.712	68.0691	37.8525	273.98	
14.714	68.5877	41.2489	247.89	
14.716	68.0863	39.6474	257.86	heating
14.718	68.1795	33.9383	310.47	heating
14.720	68.4362	35.0645	302.59	heating
14.722	68.4167	34.5603	306.98	heating
14.724	68.4103	34.5403	307.10	
14.726	68.6744	37.0036	287.16	
14.728	67.9470	34.6472	301.93	
14.730	68.5783	34.6533	307.60	
14.732	67.9943	33.9449	308.73	
14.734	68.4538	33.0851	320.69	
14.736	68.5815	33.1524	321.24	
14.738	68.3403	30.8183	340.22	
14.740	68.6196	30.1261	349.03	
14.742	68.2939	31.9472	329.56	
14.744	68.4174	30.6954	342.03	
14.746	68.5180	33.6144	316.48	
14.748	68.2983	33.4748	315.75	
14.750	68.3216	34.8241	303.73	
14.752	68.4994	38.8614	268.73	
14.754	67.9075	37.3050	277.48	
14.756	68.7276	38.8758	270.67	
14.758	68.2414	36.1096	291.34	
14.760	68.4868	30.9616	340.25	
14.762	68.5634	31.9844	331.67	
14.764	68.1785	33.2144	317.02	
14.766	68.5570	34.6324	307.60	
14.768	68.5331	38.8039	269.56	
14.770	68.3103	36.7207	286.43	
14.772	68.6078	36.9127	287.38	
14.774	68.5761	38.9452	268.67	
14.776	68.1753	36.8618	283.92	
14.778	68.7830	39.7304	263.42	
14.780	68.3031	37.0804	283.10	
14.782	68.6652	34.2500	312.05	
14.784	68.8389	39.5189	265.85	
14.786	68.1387	37.4479	278.28	
14.788	68.5153	35.4964	299.39	
14.790	68.5619	36.8704	287.35	
14.792	67.8863	30.4292	339.63	
14.794	69.0847	33.5618	322.09	
14.796	68.2608	36.6254	286.84	
14.798	68.2087	33.9990	310.18	
14.800	68.8089	38.8773	271.39	
14.802	68.0478	36.7797	283.51	
14.804	68.8220	34.2760	313.23	
14.806	68.7439	38.3387	275.69	
14.808	68.0575	29.9389	345.63	
14.810	68.7513	28.3729	366.12	
14.812	68.4041	30.0089	348.14	
14.814	67.7800	24.8330	389.41	
14.816	69.3121	34.9210	311.83	
14.818	68.1014	39.7330	257.22	
14.820	68.3440	35.6539	296.41	
14.822	68.5577	38.4228	273.24	
14.824	68.1143	35.3698	296.90	
14.826	68.4276	31.3914	335.81	
14.828	68.9218	37.9754	280.60	
14.830	67.8640	33.4616	311.93	
14.832	68.7423	35.1294	304.77	

14.834	68.3141	38.4649	270.65	
14.836	67.6921	32.0195	323.45	
14.838	69.1204	34.8023	311.17	
14.840	68.0965	35.2142	298.15	
14.842	68.4753	31.5646	334.67	
14.844	68.4844	36.3691	291.19	
14.846	68.2627	36.6869	286.30	
14.848	68.3787	35.9108	294.39	
14.850	68.8457	41.4029	248.83	
14.852	67.9169	37.1307	279.14	
14.854	68.4383	33.8820	313.33	
14.856	68.0945	32.9794	318.39	
14.858	68.1234	31.3607	333.33	
14.860	68.7350	32.9166	324.77	
14.862	68.4997	35.9075	295.52	
14.864	68.2967	33.5451	315.10	
14.866	68.3392	35.5240	297.54	
14.868	68.4834	36.3958	290.94	
14.870	68.1575	34.8333	302.16	
14.872	68.8433	37.1386	287.47	
14.874	68.0396	34.8719	300.74	
14.876	68.4495	34.2679	309.93	
14.878	68.0483	34.3496	305.55	
14.880	68.3514	35.7325	295.76	
14.882	68.4986	35.6797	297.57	
14.884	68.5625	36.3143	292.40	
14.886	68.3827	35.1623	301.21	
14.888	68.1448	34.9250	301.21	
14.890	68.5296	36.9978	285.90	
14.892	68.1587	37.1751	280.93	
14.894	68.5280	34.8100	305.73	
14.896	68.4341	35.8312	295.62	
14.898	68.2411	35.8317	293.86	
14.900	68.1745	35.2700	298.35	
14.902	68.4126	35.9640	294.22	
14.904	68.3750	32.6262	324.14	
14.906	68.9065	35.0091	307.35	
14.908	68.5984	39.4406	264.38	
14.910	68.0545	37.6804	275.41	
14.912	68.8549	39.9816	261.80	
14.914	68.2591	40.0855	255.45	
14.916	68.2744	34.3779	307.34	
14.918	68.9491	38.2177	278.65	
14.920	68.0539	36.7043	284.25	
14.922	68.1325	31.8853	328.66	
14.924	68.9323	37.7939	282.34	
14.926	67.9792	33.4636	312.96	
14.928	68.7232	31.5660	336.91	
14.930	68.6963	38.7942	271.13	
14.932	67.6909	32.5556	318.58	
14.934	68.9438	35.9682	298.99	
14.936	68.2418	41.3078	244.21	
14.938	68.0756	36.1054	289.88	
14.940	69.1287	41.1886	253.34	
14.942	67.8401	36.9163	280.39	
14.944	68.4478	29.7169	351.18	
14.946	68.9109	37.1198	288.25	
14.948	68.0171	33.5855	312.20	
14.950	68.6559	33.1470	321.96	
14.952	68.8034	41.8792	244.13	
14.954	67.7369	35.6166	291.24	
14.956	68.9311	37.9680	280.75	
14.958	68.1244	39.1720	262.52	
14.960	68.0416	33.1851	316.05	
14.962	69.0694	39.1933	270.89	
14.964	67.8031	39.4828	256.78	
14.966	68.4960	34.7663	305.83	
14.968	68.7339	40.4851	256.14	
14.970	68.1246	35.9954	291.32	heating
14.972	68.6875	35.4796	301.10	heating
14.974	68.8162	39.8566	262.58	heating
14.976	67.9236	32.5682	320.57	heating
14.978	69.2555	37.7474	285.69	
14.980	68.0332	37.6460	275.52	heating
14.982	68.7566	35.5686	300.92	heating
14.984	69.1440	42.0043	246.08	heating
14.986	68.3248	42.0503	238.23	heating
14.988	69.0420	40.0610	262.77	heating
14.990	68.7888	41.4902	247.52	heating
14.992	68.8008	34.8135	308.17	heating
14.994	68.9617	34.7813	309.92	heating
14.996	69.1216	35.9861	300.44	heating
14.998	68.3167	32.4450	325.25	heating
15.000	69.7235	38.5128	282.99	heating
15.002	68.4274	37.6314	279.23	heating
15.004	69.3506	35.5835	306.17	heating
15.006	69.2118	35.3503	307.03	heating
15.008	68.8810	30.3727	349.16	heating

15.010	69.4729	30.6534	351.98	
15.012	68.9743	34.5596	312.04	
15.014	69.1032	30.4662	350.33	
15.016	69.3435	34.1938	318.71	
15.018	69.0929	33.8803	319.28	
15.020	68.8438	32.7853	326.95	
15.022	69.6199	36.8519	297.11	
15.024	68.5260	31.6041	334.78	
15.026	69.6877	33.5572	327.60	
15.028	68.8374	34.0819	315.13	
15.030	69.1054	30.8074	347.25	
15.032	69.5653	35.0665	312.81	
15.034	68.6246	34.0032	313.92	
15.036	69.2346	30.8974	347.61	
15.038	69.3854	35.6930	305.49	
15.040	68.8399	31.3087	340.30	
15.042	69.3146	32.4811	333.97	
15.044	69.3088	36.3947	298.44	cooling
15.046	68.6694	30.2849	348.04	
15.048	69.5781	35.7415	306.80	cooling
15.050	68.7024	38.0719	277.73	cooling
15.052	68.9944	35.6392	302.44	
15.054	69.5535	41.8693	251.02	
15.056	68.2961	37.3134	280.92	
15.058	69.3677	36.1613	301.09	
15.060	69.1707	41.5680	250.28	
15.062	68.3657	34.5869	306.28	
15.064	69.5214	36.8502	296.23	
15.066	68.9398	40.7529	255.57	
15.068	68.9474	36.0034	298.71	
15.070	69.5239	42.3006	246.84	
15.072	68.7149	40.7073	253.95	
15.074	69.0691	36.1682	298.32	
15.076	69.5788	40.9955	259.17	
15.078	68.3517	32.9870	320.66	
15.080	69.5120	32.2508	337.85	
15.082	69.0732	36.8411	292.25	
15.084	68.4674	33.7159	315.10	
15.086	69.5454	37.2615	292.72	
15.088	68.7317	37.2727	285.24	
15.090	69.2059	31.8432	338.77	
15.092	69.3598	37.9957	284.38	
15.094	68.7204	34.7314	308.18	
15.096	69.0086	30.7115	347.25	
15.098	69.5165	35.5483	308.00	
15.100	68.3323	31.0358	338.17	
15.102	69.6970	35.9331	306.14	
15.104	68.6497	38.4536	273.79	
15.106	68.7612	33.3423	321.15	
15.108	69.3994	33.4569	325.90	
15.110	68.7270	29.9653	351.46	
15.112	69.4254	27.8634	376.85	
15.114	69.0992	35.7778	302.13	
15.116	68.7748	35.5507	301.25	
15.118	68.9368	35.7749	300.68	
15.120	69.4608	38.5464	280.31	
15.122	68.5496	34.9656	304.51	
15.124	69.5235	36.3472	300.81	
15.126	68.4942	33.4622	317.64	
15.128	69.0432	31.1970	343.16	
15.130	69.0676	34.2561	315.64	
15.132	69.2452	38.9314	274.86	
15.134	69.2824	42.4211	243.56	
15.136	68.8297	42.9480	234.67	
15.138	68.9785	38.0642	280.30	
15.140	69.3229	39.1635	273.46	
15.142	69.0543	35.4568	304.63	
15.144	68.9526	35.4900	303.41	
15.146	69.3062	40.2083	263.83	
15.148	68.4316	37.3357	281.95	
15.150	69.3679	39.3640	272.05	
15.152	68.6114	39.2522	266.20	
15.154	69.1872	35.4187	306.18	
15.156	69.3075	37.5598	287.86	
15.158	68.4758	33.3061	318.89	
15.160	69.0608	29.5578	358.18	
15.162	69.3897	36.2112	300.83	
15.164	68.7871	34.9626	306.69	
15.166	69.2644	38.1613	282.02	
15.168	69.1365	40.1026	263.25	
15.170	68.6112	33.2041	321.04	
15.172	69.5872	36.5125	299.89	
15.174	68.6791	33.4742	319.21	
15.176	69.1328	25.7849	393.04	
15.178	69.5815	34.1506	321.26	
15.180	68.4980	35.9923	294.73	
15.182	69.1663	36.3033	297.97	
15.184	69.3960	42.6594	242.42	
15.186	68.3755	35.7315	295.99	
15.188	69.5154	36.8647	296.05	
15.190	69.0765	40.1878	261.94	
15.192	68.6416	32.6802	326.07	
15.194	69.6226	37.2876	293.19	
15.196	68.8412	37.9994	279.65	
15.198	69.0596	32.7295	329.41	
15.200	69.4801	38.3475	282.28	
15.202	68.4035	34.9174	303.62	
15.204	69.3063	34.0639	319.55	
15.206	69.2433	39.3656	270.91	
15.208	68.4752	32.9342	322.25	
15.210	69.3998	35.0988	311.01	
15.212	69.1122	41.1592	253.45	
15.214	68.4940	36.1512	293.26	
15.216	69.3295	36.9899	293.23	
15.218	68.8415	35.8519	299.12	
15.220	68.9839	32.0542	334.85	
15.222	69.3007	35.9877	302.05	
15.224	68.5753	33.2167	320.60	
15.226	69.1446	29.2099	362.09	
15.228	69.1435	31.9495	337.24	
15.230	68.6964	27.4525	373.96	
15.232	69.1742	26.5252	386.70	
15.234	69.1626	31.3374	342.97	cooling
15.236	68.5674	30.6358	343.93	cooling
15.238	69.3259	35.5946	305.85	cooling
15.240	68.7426	34.9697	306.22	cooling
15.242	68.8107	32.3705	330.41	cooling
15.244	69.2302	35.7744	303.35	cooling
15.246	68.4521	33.6144	315.88	cooling
15.248	68.9927	33.8782	318.39	cooling
15.250	68.5742	37.4070	282.60	cooling
15.252	68.7176	39.3396	266.37	cooling
15.254	68.6744	40.6237	254.34	cooling
15.256	68.5016	38.4665	272.33	
15.258	68.5885	34.3079	310.83	
15.260	68.8422	35.9105	298.60	
15.262	68.4133	35.3397	299.88	
15.264	68.5047	34.6527	306.94	
15.266	68.6875	34.8636	306.69	
15.268	68.8170	36.2191	295.57	
15.270	68.9005	40.1042	261.10	
15.272	68.3522	39.3024	263.40	
15.274	68.7277	37.3956	284.09	
15.276	68.6135	36.1955	293.94	
15.278	68.5480	31.9722	331.64	
15.280	68.7394	32.4995	328.59	
15.282	68.5689	35.7511	297.56	
15.284	68.4929	38.7935	269.29	
15.286	68.6280	42.4904	236.99	
15.288	68.4641	38.3644	272.92	
15.290	68.7911	35.3772	302.97	
15.292	68.8554	35.7433	300.23	
15.294	68.3857	33.7641	313.92	
15.296	68.7960	34.2393	313.33	
15.298	68.3881	33.6812	314.69	
15.300	68.9522	35.6889	301.60	
15.302	68.8752	41.3332	249.73	
15.304	68.4033	38.5275	270.89	
15.306	68.5695	34.4120	309.71	
15.308	68.7853	35.8814	298.34	
15.310	68.2380	32.3369	325.52	
15.312	68.9085	34.6037	311.05	
15.314	68.6916	37.0636	286.78	
15.316	68.4571	34.9232	304.06	
15.318	68.9198	38.2941	277.69	
15.320	68.3126	36.4992	288.46	
15.322	68.7644	31.1878	340.71	
15.324	68.8895	34.5785	311.10	
15.326	68.2880	34.1835	309.23	
15.328	68.5728	32.8611	323.80	
15.330	68.6916	34.6605	308.57	
15.332	68.2170	31.1331	336.25	
15.334	69.0068	34.5767	312.18	
15.336	68.5895	36.6442	289.65	
15.338	68.5370	33.5434	317.29	
15.340	68.8653	36.7057	291.60	
15.342	68.3417	35.7487	295.53	
15.344	68.6215	32.6805	325.88	
15.346	68.7733	35.6249	300.56	
15.348	68.3329	35.6688	296.17	
15.350	68.7440	38.0007	278.75	
15.352	68.4648	38.8257	268.74	
15.354	68.3100	33.4924	315.70	
15.356	68.8096	33.9473	316.10	
15.358	68.8019	38.1581	277.85	
15.360	68.4267	35.7622	296.17	

15.362	68.6972	35.5308	300.72	
15.364	68.4617	34.6828	306.28	
15.366	68.5734	34.3581	310.23	
15.368	68.8306	38.3133	276.70	
15.370	68.3150	37.4649	279.72	
15.372	68.8171	37.6260	282.81	
15.374	68.3469	38.2556	272.84	
15.376	68.4606	35.4585	299.24	
15.378	68.5481	33.9291	313.90	
15.380	68.6818	34.1924	312.72	
15.382	68.7892	37.0725	287.58	
15.384	68.5873	40.1391	257.94	
15.386	68.4474	39.5862	261.69	
15.388	68.6272	40.8615	251.76	
15.390	68.7236	40.7728	253.43	
15.392	68.6733	38.0412	277.75	
15.394	68.6798	37.7930	280.06	
15.396	68.2949	36.8164	285.42	
15.398	68.6898	37.8891	279.27	heating
15.400	68.7302	38.1700	277.09	heating
15.402	68.8084	34.0729	314.95	heating
15.404	68.9956	34.7757	310.28	heating
15.406	68.6764	38.4682	273.90	
15.408	68.3901	35.3545	299.54	
15.410	68.7604	34.5237	310.43	
15.412	68.5919	31.6859	334.63	
15.414	69.1464	32.2405	334.63	
15.416	69.0148	36.0052	299.30	
15.418	68.2843	32.4683	324.75	
15.420	68.8599	31.2554	340.97	
15.422	68.9329	32.2563	332.55	
15.424	68.6924	28.3489	365.80	
15.426	69.0067	30.8467	346.00	
15.428	68.6027	30.9737	341.19	
15.430	68.6700	29.7804	352.62	
15.432	69.0056	34.9092	309.16	
15.434	68.5253	33.5183	317.41	
15.436	69.2129	32.7230	330.86	
15.438	69.0378	37.6634	284.48	cooling
15.440	68.2959	34.9515	302.34	
15.442	68.7374	33.3220	321.12	
15.444	68.9260	34.7970	309.45	
15.446	68.6766	32.5709	327.38	
15.448	69.1957	37.7077	285.51	cooling
15.450	68.4723	37.1030	284.43	
15.452	68.5353	32.5335	326.43	
15.454	69.0023	36.8523	291.51	
15.456	68.5544	36.2355	293.04	
15.458	69.0382	32.6328	330.09	
15.460	68.9976	33.2687	323.96	
15.462	68.5543	31.0819	339.77	
15.464	68.9473	33.6832	319.75	cooling
15.466	68.8679	36.3692	294.67	cooling
15.468	68.5360	32.4943	326.80	cooling
15.470	69.1622	34.4255	314.96	cooling
15.472	68.4293	34.6967	305.86	cooling
15.474	68.5596	33.6401	316.62	cooling
15.476	68.4543	33.6201	315.85	cooling
15.478	68.2832	31.6058	332.56	cooling
15.480	68.8007	32.9437	325.12	cooling
15.482	68.4071	36.1233	292.72	cooling
15.484	68.1950	33.8932	311.02	cooling
15.486	68.2833	35.3954	298.20	cooling
15.488	68.1388	36.2015	289.58	cooling
15.490	68.1243	34.9006	301.24	cooling
15.492	68.3943	36.3879	290.21	cooling
15.494	67.7391	33.4346	311.04	
15.496	68.2739	34.8668	302.91	
15.498	68.0591	36.7766	283.64	
15.500	67.8293	32.6418	319.05	
15.502	68.4724	34.1692	311.03	
15.504	68.0722	37.3600	278.47	
15.506	68.2416	37.8932	275.17	
15.508	68.0010	38.1210	270.93	
15.510	67.7757	32.6521	318.47	
15.512	68.2029	32.4520	324.16	
15.514	68.4100	36.3403	290.78	
15.516	67.9993	35.5586	294.15	
15.518	68.2733	38.6802	268.32	
15.520	67.7692	37.6143	273.42	
15.522	68.1738	35.7888	293.64	
15.524	68.3325	37.6589	278.12	
15.526	67.8007	34.8815	298.48	
15.528	68.3345	35.9776	293.39	
15.530	68.0149	38.3331	269.13	
15.532	67.6457	33.5470	309.18	
15.534	68.4441	36.0401	293.81	
15.536	68.1503	36.4790	287.17	

15.538	68.2326	35.5501	296.34	
15.540	68.4044	38.8355	268.11	
15.542	67.3883	32.3899	317.34	
15.544	68.4855	31.0522	339.41	
15.546	68.1424	34.9336	301.11	
15.548	67.9764	29.8883	345.35	
15.550	68.5399	34.2302	311.09	
15.552	67.8766	36.4820	284.66	
15.554	67.6545	31.6950	326.05	
15.556	68.9120	37.4189	285.55	
15.558	67.6238	30.6273	335.45	
15.560	68.5246	28.6069	361.94	
15.562	68.4652	37.3451	282.17	
15.564	67.3271	32.8774	312.36	
15.566	68.2692	32.7273	322.26	
15.568	68.3083	38.0243	274.59	
15.570	68.0977	29.9388	345.99	
15.572	68.8041	36.5412	292.53	
15.574	67.7727	38.0877	269.16	
15.576	67.6398	29.7327	343.71	
15.578	69.2149	38.4494	278.95	
15.580	67.2954	36.3219	280.84	
15.582	68.5362	33.9352	313.73	
15.584	68.4388	41.9433	240.24	
15.586	67.3158	35.6186	287.40	
15.588	68.2985	35.8896	293.86	
15.590	68.1054	40.9453	246.27	
15.592	67.8178	35.6891	291.32	
15.594	68.8178	44.5518	220.02	
15.596	67.4896	43.9346	213.58	
15.598	67.8074	33.7419	308.88	
15.600	68.7088	37.2882	284.90	
15.602	67.3490	34.3032	299.63	
15.604	68.5751	34.3263	310.54	
15.606	68.2670	42.3859	234.67	
15.608	67.6695	36.1575	285.72	heating
15.610	68.3787	37.8344	276.95	heating
15.612	68.5413	41.0271	249.48	heating
15.614	67.9179	30.5723	338.62	heating
15.616	69.0489	33.8617	319.05	heating
15.618	67.7327	32.7624	317.08	
15.620	68.3687	32.1356	328.53	
15.622	68.6215	40.0830	258.76	
15.624	67.6651	38.8106	261.63	
15.626	68.8274	38.1178	278.45	
15.628	68.4150	41.0012	248.57	heating
15.630	68.1510	32.6146	322.21	heating
15.632	68.4870	32.3898	327.30	heating
15.634	68.8200	35.5301	301.84	
15.636	67.9809	31.0805	334.58	
15.638	69.0423	35.9343	300.19	
15.640	68.1032	37.9841	273.09	
15.642	68.6591	38.1930	276.24	
15.644	68.2874	37.9042	275.49	
15.646	68.2586	32.6277	323.07	
15.648	68.7858	27.2092	376.98	
15.650	68.5888	29.3230	356.03	
15.652	68.6095	30.6398	344.28	
15.654	68.2707	34.2777	308.22	
15.656	68.5053	34.9286	304.45	
15.658	67.9081	34.0888	306.64	
15.660	69.1035	37.4333	287.16	
15.662	68.0948	33.5273	313.43	
15.664	68.7415	32.7685	326.17	
15.666	67.9606	31.0304	334.85	
15.668	68.7087	32.4241	329.00	
15.670	68.5689	34.9663	304.68	
15.672	68.5340	39.5409	262.88	
15.674	68.6703	41.3708	247.53	
15.676	68.2367	41.4085	243.25	
15.678	68.2053	35.2551	298.76	
15.680	68.1086	32.7064	321.00	
15.682	68.9836	33.9508	317.65	
15.684	68.3571	36.9246	285.00	
15.686	68.7133	43.1350	231.92	
15.688	67.7827	41.8586	235.06	
15.690	68.8543	42.1003	242.58	
15.692	68.4163	41.7126	242.13	
15.694	68.2949	38.1225	273.58	heating
15.696	68.7104	37.0397	287.16	heating
15.698	68.2574	37.6442	277.57	heating
15.700	68.1963	34.3352	307.02	heating
15.702	68.6725	37.3180	284.30	heating
15.704	69.1477	36.7989	293.31	heating
15.706	68.7971	34.3034	312.76	heating
15.708	69.1161	38.4003	278.50	heating
15.710	68.1554	34.0991	308.79	heating
15.712	69.2942	33.5197	324.37	heating

15.714	68.8528	35.1648	305.45	
15.716	68.6270	31.6734	335.06	
15.718	69.1729	32.1463	335.73	
15.720	68.4703	34.0716	311.90	
15.722	68.3950	31.6053	333.58	
15.724	69.0426	36.0416	299.23	
15.726	68.9756	33.3332	323.17	
15.728	68.8334	28.0761	369.55	
15.730	69.1619	32.7619	330.04	
15.732	68.2028	29.9207	347.11	
15.734	68.9042	28.9975	361.84	
15.736	69.0467	32.8218	328.46	
15.738	68.6201	29.1244	358.11	
15.740	69.4284	34.7319	314.60	cooling
15.742	68.5519	38.3061	274.24	cooling
15.744	68.3298	31.0170	338.32	
15.746	69.2550	34.6961	313.35	cooling
15.748	68.2775	33.8982	311.72	cooling
15.750	68.8470	30.1644	350.74	cooling
15.752	68.9708	36.9357	290.47	cooling
15.754	67.8943	32.8796	317.48	cooling
15.756	68.5106	30.5913	343.82	cooling
15.758	68.8959	36.0826	297.52	cooling
15.760	67.7725	31.9587	324.73	cooling
15.762	68.9052	37.0401	288.93	cooling
15.764	68.1140	40.1017	253.99	cooling
15.766	67.9363	32.4934	321.37	cooling
15.768	68.4320	34.5210	307.48	cooling
15.770	68.0325	38.2593	269.96	cooling
15.772	68.3323	36.4608	288.98	
15.774	68.5696	43.7274	225.25	
15.776	67.6292	40.1308	249.33	
15.778	68.2602	37.3900	279.90	
15.780	68.7281	41.2800	248.88	
15.782	67.4858	34.8808	295.63	
15.784	68.6181	33.1824	321.30	
15.786	68.0321	35.3279	296.53	
15.788	68.1405	33.4896	314.19	
15.790	68.1187	35.1557	298.88	
15.792	68.2131	35.2645	298.75	
15.794	67.9823	31.6820	329.14	
15.796	68.3105	33.2477	317.92	
15.798	67.8104	31.3561	330.54	
15.800	68.1486	30.1434	344.60	
15.802	68.1972	30.3360	343.29	
15.804	67.9341	30.6400	338.15	
15.806	68.2052	31.2136	335.41	
15.808	68.1497	33.8040	311.42	
15.810	68.3403	35.2249	300.26	
15.812	67.9164	34.3890	304.00	
15.814	68.2980	32.5680	323.97	
15.816	67.7916	28.7763	353.76	
15.818	68.3294	29.8253	349.12	cooling
15.820	68.0740	34.9464	300.37	cooling
15.822	67.9653	37.5788	275.52	cooling
15.824	67.6730	35.0562	295.74	cooling
15.826	68.2445	37.3819	279.84	cooling
15.828	67.7122	35.9637	287.87	
15.830	68.0315	35.3379	296.44	
15.832	68.0152	36.6269	284.60	
15.834	67.4591	32.8382	313.91	
15.836	67.8926	32.0941	324.59	
15.838	68.0649	34.8718	300.97	
15.840	67.8187	30.8499	335.20	
15.842	68.1600	34.6880	303.50	
15.844	67.7856	38.1009	269.16	
15.846	67.5699	33.4888	309.02	
15.848	68.2408	33.9662	310.77	
15.850	67.5649	31.6844	325.33	
15.852	68.0918	28.7649	356.58	
15.854	68.2043	33.8861	311.17	cooling
15.856	67.4199	31.3198	327.33	cooling
15.858	67.7305	30.0764	341.42	cooling
15.860	68.4110	36.1019	292.95	cooling
15.862	67.2538	33.1121	309.57	cooling
15.864	68.0159	33.9965	308.46	cooling
15.866	67.4049	35.6310	288.10	cooling
15.868	67.5852	32.5625	317.56	cooling
15.870	67.7524	35.2836	294.40	cooling
15.872	67.5664	37.6428	271.32	cooling
15.874	67.5892	32.3601	319.43	
15.876	68.0154	38.5680	267.00	
15.878	67.2190	38.1470	263.60	
15.880	67.2853	34.4260	297.94	
15.882	68.0668	35.8624	292.00	
15.884	67.0808	31.6899	320.89	
15.886	68.1258	30.7959	338.48	
15.888	67.5738	35.7098	288.92	

15.890	67.6632	34.0221	305.03	
15.892	67.5943	35.3293	292.55	
15.894	67.9391	40.8511	245.61	
15.896	67.1832	37.2337	271.56	
15.898	68.0478	37.6755	275.39	
15.900	67.3702	35.0517	293.04	
15.902	67.2663	29.6356	341.20	
15.904	67.7830	29.9161	343.35	
15.906	67.3350	31.4560	325.32	
15.908	67.7296	33.9562	306.23	
15.910	67.7322	40.1251	250.32	
15.912	67.5377	37.7675	269.93	
15.914	67.3773	31.8402	322.22	
15.916	67.7818	33.4837	310.99	
15.918	67.4474	34.4125	299.53	
15.920	67.8138	37.3308	276.39	
15.922	67.5664	37.7627	270.23	
15.924	67.2542	33.3266	307.63	
15.926	67.8167	34.4812	302.26	
15.928	67.3306	35.4920	288.69	
15.930	67.4451	32.4419	317.38	
15.932	67.7468	33.2930	312.40	
15.934	67.5385	33.6515	307.26	
15.936	67.4416	32.3331	318.33	
15.938	67.6271	33.6895	307.72	
15.940	67.3452	31.2199	327.55	
15.942	67.6468	30.8709	333.45	
15.944	67.7636	35.7043	290.69	
15.946	67.2105	33.6059	304.70	
15.948	67.5674	31.8448	323.90	
15.950	67.4595	31.0677	329.97	
15.952	67.4100	26.8222	368.01	
15.954	68.0026	29.8744	345.71	
15.956	67.4395	34.3756	299.80	cooling
15.958	67.3431	34.5676	297.18	cooling
15.960	67.6472	37.2060	276.01	
15.962	67.3452	34.9334	293.88	
15.964	67.4615	30.8300	332.14	
15.966	67.8136	36.6584	282.49	
15.968	67.1096	38.1043	263.00	
15.970	67.1817	36.1760	281.13	
15.972	67.6319	37.9406	269.22	
15.974	67.1658	35.6512	285.75	
15.976	68.0476	37.5690	276.35	
15.978	67.3293	39.7478	250.09	
15.980	67.0897	32.7542	311.33	
15.982	67.5764	32.4055	318.90	
15.984	67.9284	37.6995	274.09	
15.986	67.3985	32.8033	313.68	
15.988	67.8669	34.6600	301.09	
15.990	67.3345	34.2773	299.73	
15.992	67.3087	32.8673	312.29	
15.994	67.8689	38.7502	264.02	
15.996	66.9998	38.6284	257.25	
15.998	67.8967	36.3227	286.29	
16.000	67.5395	41.2599	238.28	
16.002	67.0909	37.6294	267.13	
16.004	67.3295	34.2279	300.14	
16.006	68.2542	40.1653	254.69	
16.008	67.0172	37.7407	265.45	
16.010	67.7240	38.5698	264.34	
16.012	67.3167	39.4785	252.41	heating
16.014	67.3985	36.7141	278.22	heating
16.016	67.7891	36.9884	279.27	heating
16.018	67.2200	34.4803	296.86	heating
16.020	67.8674	30.2989	340.64	heating
16.022	67.8875	35.7792	291.13	heating
16.024	67.5519	35.8616	287.34	heating
16.026	67.6285	32.0852	322.28	heating
16.028	68.4126	33.6927	314.81	
16.030	67.4394	30.9265	331.07	
16.032	68.0531	32.7511	320.09	
16.034	67.7740	33.3552	312.08	
16.036	67.6559	28.8152	352.17	
16.038	68.0250	29.3988	350.23	
16.040	67.5914	31.4965	327.28	
16.042	67.9818	30.5210	339.66	
16.044	67.7298	31.8532	325.30	
16.046	67.6047	29.9719	341.22	
16.048	67.6705	28.7219	353.15	
16.050	68.3279	34.3493	308.09	
16.052	67.6589	35.0740	295.45	
16.054	67.8114	36.0850	287.67	
16.056	67.9615	38.1746	270.08	
16.058	67.6156	36.4287	282.78	
16.060	67.9647	35.6561	292.95	
16.062	67.5845	36.2703	283.93	
16.064	67.8863	35.2265	296.13	

16.066	67.6759	35.8060	288.97	
16.068	67.8165	36.6958	282.18	
16.070	67.5244	34.0328	303.67	
16.072	68.1159	34.9773	300.47	
16.074	67.7734	35.4744	292.86	
16.076	67.6678	34.0797	304.55	
16.078	67.9835	34.4133	304.39	
16.080	67.9158	36.1892	287.67	
16.082	67.9307	35.1712	297.04	
16.084	67.7566	34.0287	305.82	
16.086	67.9776	32.1726	324.65	
16.088	67.6669	30.5441	336.60	
16.090	68.0676	35.8716	291.93	
16.092	67.5871	36.1417	285.12	
16.094	67.7397	34.3334	302.90	
16.096	68.1018	37.9981	272.95	
16.098	67.3456	37.6683	269.09	
16.100	68.0003	35.3005	296.49	
16.102	67.9340	35.8038	291.33	
16.104	67.5229	29.9161	340.99	
16.106	67.8809	27.7257	364.09	
16.108	68.0614	29.8716	346.27	
16.110	67.7268	28.4723	355.93	
16.112	68.2191	36.4101	288.42	
16.114	67.6012	38.6375	262.62	
16.116	67.7235	33.6213	309.21	
16.118	68.1181	34.7044	302.97	
16.120	67.3673	31.7641	322.82	
16.122	68.0466	30.9554	336.31	
16.124	68.0213	34.9302	300.04	
16.126	67.3587	30.8813	330.75	
16.128	67.8490	30.6849	336.97	
16.130	68.1695	38.0852	272.78	
16.132	67.4972	36.1345	284.37	
16.134	68.0754	35.9320	291.45	
16.136	67.6077	33.9979	304.74	
16.138	67.6191	29.0199	349.98	
16.140	68.0467	34.0247	308.48	
16.142	67.6088	39.4444	255.37	
16.144	67.8000	38.3652	266.89	
16.146	68.1503	43.8109	220.69	
16.148	67.5574	40.9384	241.36	
16.150	67.7113	37.1113	277.46	
16.152	68.3163	40.3357	253.70	
16.154	67.3464	36.4739	279.93	
16.156	68.0473	36.2305	288.49	
16.158	67.6027	37.3584	274.23	
16.160	67.7294	36.3148	284.84	
16.162	67.9362	40.8062	245.99	
16.164	67.6608	42.9319	224.22	
16.166	67.7325	35.8192	289.36	heating
16.168	68.0476	33.8363	310.20	heating
16.170	67.8342	31.3645	330.68	heating
16.172	67.8861	30.7946	336.31	
16.174	68.2313	33.7741	312.43	
16.176	67.4042	31.3884	326.56	
16.178	68.1379	32.3103	324.85	
16.180	68.1629	36.2778	289.11	
16.182	67.9003	33.6351	310.69	
16.184	68.0211	31.4303	331.77	
16.186	67.7560	29.1305	350.22	
16.188	67.9626	25.3374	386.49	
16.190	68.1197	28.8123	356.41	
16.192	67.8634	32.1978	323.39	
16.194	67.8509	33.9485	307.40	
16.196	68.0992	35.6909	293.85	
16.198	67.5324	32.8012	314.91	
16.200	67.9046	30.6993	337.35	
16.202	68.2385	33.0191	319.34	
16.204	67.9742	34.7455	301.29	
16.206	67.7959	33.2546	313.19	
16.208	68.0816	34.5750	303.81	
16.210	68.1849	35.1554	299.48	
16.212	67.9335	33.1300	315.57	
16.214	68.0494	34.4281	304.85	
16.216	67.7601	32.8810	316.25	
16.218	67.8687	31.5090	329.68	
16.220	67.9676	34.1951	306.22	
16.222	67.5615	34.4837	299.92	
16.224	68.1674	35.2509	298.46	
16.226	67.7846	37.5752	273.91	
16.228	67.6718	37.5339	273.26	
16.230	67.8029	38.0449	269.82	
16.232	68.2296	42.0492	237.38	
16.234	67.4912	38.1712	265.85	
16.236	68.0041	38.7725	265.05	
16.238	67.7915	38.8883	262.07	
16.240	67.6146	34.5457	299.84	
16.242	67.9360	34.4995	303.17	
16.244	67.6491	34.9273	296.69	
16.246	68.1557	31.3254	333.94	
16.248	68.0136	34.6554	302.46	
16.250	67.7343	35.8950	288.69	
16.252	67.8579	35.2454	295.70	
16.254	68.5563	39.6539	262.06	
16.256	67.5071	34.2557	301.50	
16.258	67.9967	32.8603	318.59	
16.260	68.1013	35.4132	296.39	
16.262	67.5671	30.9036	332.43	
16.264	68.0816	30.9137	337.01	
16.266	67.8290	33.2330	313.69	
16.268	68.0258	29.6888	347.61	
16.270	68.2330	34.2524	308.11	
16.272	67.6957	34.9423	296.98	
16.274	67.8319	29.0042	352.06	
16.276	68.6268	33.0559	322.53	
16.278	67.5663	31.5457	326.60	
16.280	68.0617	31.0446	335.64	
16.282	68.4273	38.2177	273.91	
16.284	67.5204	34.8229	296.47	
16.286	68.0644	33.5489	312.96	
16.288	68.0010	36.5403	285.26	
16.290	67.8657	32.7013	318.84	
16.292	68.2910	37.7162	277.23	
16.294	67.5665	39.8088	251.68	
16.296	67.9061	36.5656	284.17	
16.298	68.4915	42.2672	237.78	
16.300	67.4451	39.8628	250.09	
16.302	67.9088	34.8673	299.59	
16.304	68.4683	37.4927	280.86	
16.306	67.7012	32.3488	320.55	
16.308	68.1802	33.4350	315.04	
16.310	68.0542	37.7681	274.61	
16.312	67.8818	34.8435	299.56	
16.314	68.4199	38.7460	269.06	
16.316	67.6025	39.9889	250.38	
16.318	68.0506	36.6291	284.90	
16.320	68.1777	39.4547	260.44	
16.322	67.7406	38.1538	268.27	
16.324	67.7930	34.7654	299.47	
16.326	68.4791	37.4621	281.24	
16.328	67.7348	34.2952	303.20	
16.330	68.1812	34.6352	304.17	
16.332	67.8344	34.4679	302.54	
16.334	67.8546	31.8117	326.81	
16.336	68.1773	33.6781	312.81	
16.338	67.7755	35.4630	292.98	
16.340	68.0618	32.1792	325.35	
16.342	67.9508	32.9768	317.11	
16.344	67.8570	32.5988	319.69	
16.346	67.8506	32.8357	317.49	
16.348	68.4021	35.8839	294.85	
16.350	67.6822	34.3832	301.93	
16.352	68.1657	34.7306	303.16	
16.354	67.6536	32.6408	317.47	
16.356	68.1543	31.8085	329.55	
16.358	67.9194	30.8384	336.22	
16.360	67.8025	29.1877	350.13	
16.362	68.0738	28.6427	357.53	
16.364	67.8451	33.4443	311.92	
16.366	67.9257	36.9509	280.85	
16.368	67.8777	40.2221	250.76	
16.370	68.3991	39.2303	264.48	
16.372	67.8069	35.4456	293.42	
16.374	68.3949	36.8091	286.39	
16.376	67.6227	35.8522	288.07	
16.378	68.3954	37.7562	277.81	
16.380	67.6988	36.1042	286.47	
16.382	67.9964	34.6864	302.03	
16.384	68.3016	36.7999	285.63	
16.386	67.6826	37.6763	272.07	
16.388	67.9173	35.5015	293.92	
16.390	67.9641	37.3598	277.49	
16.392	68.0372	32.7728	319.75	
16.394	67.9999	34.0671	307.67	
16.396	68.2860	40.4538	252.36	
16.398	67.2479	35.9852	283.46	
16.400	68.5672	37.0892	285.42	
16.402	67.3233	33.6051	305.73	
16.404	68.0323	29.0724	353.26	
16.406	68.4104	35.2266	300.88	
16.408	67.5640	35.4044	291.60	
16.410	67.8346	31.8934	325.88	
16.412	68.3193	38.1063	273.95	
16.414	67.8572	34.2107	305.08	
16.416	68.1083	34.3814	305.81	

16.418	68.1117	35.6701	294.15	
16.420	67.3316	27.4702	361.43	
16.422	68.7066	31.5643	336.77	
16.424	67.5721	34.4311	300.49	
16.426	67.8793	30.4345	339.52	
16.428	68.4948	37.5783	280.32	
16.430	67.5573	40.1588	248.43	
16.432	67.7058	36.0124	287.37	
16.434	68.4287	40.3357	254.72	
16.436	67.4724	32.8729	313.72	
16.438	68.0945	30.6307	339.69	
16.440	68.1902	34.6597	304.03	
16.442	67.3274	28.9316	348.14	
16.444	68.5054	33.9240	313.55	
16.446	67.6314	39.5329	254.77	
16.448	67.9069	35.4671	294.14	
16.450	68.3674	37.9656	275.66	
16.452	67.6196	36.4014	283.06	
16.454	67.8562	32.2689	322.68	
16.456	68.2841	36.8179	285.31	
16.458	67.6136	34.8028	297.50	
16.460	67.9932	35.8015	291.89	
16.462	68.1912	41.5298	241.74	
16.464	67.6226	39.2782	257.00	
16.466	68.0980	37.9628	273.24	
16.468	67.7600	35.8033	289.76	
16.470	68.0081	30.9702	335.83	
16.472	68.3817	32.7146	323.40	
16.474	67.7184	34.9981	296.68	
16.476	67.9036	35.7493	291.55	
16.478	68.0058	38.8318	264.52	
16.480	67.8259	38.0833	269.68	
16.482	67.8057	35.5232	292.71	
16.484	68.0414	34.1177	307.59	
16.486	67.8219	30.9946	333.92	
16.488	67.9060	29.6448	346.92	
16.490	67.7735	29.7650	344.63	
16.492	68.1708	32.9510	319.34	
16.494	68.2309	34.9166	302.07	
16.496	67.8198	35.4826	293.21	
16.498	67.9271	33.2215	314.68	
16.500	67.6703	27.6955	362.46	
16.502	68.2525	27.3076	371.25	
16.504	67.6286	27.5939	363.00	
16.506	68.0825	27.6262	366.82	
16.508	67.8915	30.8608	335.76	cooling
16.510	67.6624	32.1854	321.68	cooling
16.512	67.6240	31.7564	325.22	cooling
16.514	68.2716	36.8793	284.64	cooling
16.516	67.8809	32.7296	318.72	cooling
16.518	67.7803	29.8571	343.85	cooling
16.520	67.8575	29.9262	343.93	cooling
16.522	67.3723	29.1440	346.62	cooling
16.524	68.1834	36.9217	283.45	cooling
16.526	67.1057	39.9848	245.91	cooling
16.528	67.7452	36.2183	285.86	cooling
16.530	67.6010	36.1744	284.95	cooling
16.532	67.2747	34.1240	300.58	cooling
16.534	67.2548	31.9796	319.84	cooling
16.536	68.0449	38.9233	264.05	cooling
16.538	67.1675	33.5579	304.74	
16.540	67.5952	34.0043	304.57	
16.542	67.7016	40.1160	250.12	
16.544	66.9255	36.8358	272.83	
16.546	68.0522	39.0961	262.55	
16.548	66.9093	37.9775	262.33	
16.550	67.5393	31.2749	328.81	
16.552	67.7471	34.7628	299.07	
16.554	67.0508	35.2314	288.51	
16.556	67.2164	31.6814	322.20	
16.558	68.0424	38.6859	266.18	
16.560	67.0535	32.4930	313.37	
16.562	67.4938	31.5455	325.95	
16.564	67.8780	39.5054	257.26	
16.566	66.8597	36.7769	272.76	
16.568	67.9437	39.4059	258.76	
16.570	67.1430	40.4556	241.98	
16.572	67.3880	33.6749	305.68	
16.574	68.0155	37.5105	276.59	
16.576	67.0215	36.0070	281.21	
16.578	67.2024	30.9547	328.66	
16.580	67.9037	36.9299	280.84	
16.582	67.0123	33.5424	303.48	
16.584	67.5776	31.7960	324.44	
16.586	67.5409	35.5221	290.32	
16.588	66.9455	30.1774	333.38	
16.590	67.6880	33.1860	312.83	
16.592	67.3097	36.8400	276.27	

16.594	67.2950	32.5221	315.29
16.596	67.9662	35.7243	292.34
16.598	67.0672	33.9121	300.62
16.600	67.3097	28.6168	350.83
16.602	67.6470	31.0006	332.28
16.604	67.1462	29.5415	340.97
16.606	67.5511	29.8404	341.93
16.608	67.6292	35.0657	295.26
16.610	67.2138	33.5721	305.03
16.612	67.5414	36.5688	280.83
16.614	67.4349	37.7486	269.17
16.616	67.1881	31.8361	320.54
16.618	68.0051	32.3135	323.62
16.620	67.1265	33.6846	303.22
16.622	67.6219	35.2059	293.92
16.624	67.2925	37.1072	273.69
16.626	67.4262	37.3939	272.31
16.628	67.3874	35.8206	286.22
16.630	67.6069	37.2637	275.13
16.632	67.5329	37.2923	274.20
16.634	67.2763	37.7019	268.16
16.636	67.4535	35.8170	286.85
16.638	67.5392	35.4363	291.08
16.640	67.7413	35.6463	291.01
16.642	67.3442	35.0993	292.37
16.644	67.6173	35.0216	295.55
16.646	67.1999	32.1589	317.72
16.648	67.6309	31.8109	324.78
16.650	67.2030	30.5533	332.31
16.652	67.6331	32.0558	322.58
16.654	67.7069	37.3418	275.32
16.656	67.0548	37.0247	272.29
16.658	67.3444	33.0599	310.86
16.660	67.6911	34.5590	300.41
16.662	67.4331	32.5351	316.42
16.664	67.4082	33.0085	311.91
16.666	67.5783	35.2464	293.16
16.668	67.0624	30.9227	327.68
16.670	67.7321	32.9966	314.95
16.672	67.2602	34.3686	298.23
16.674	67.4111	29.5030	343.72
16.676	67.8029	32.2690	322.19
16.678	67.1564	33.5870	304.38
16.680	67.3490	32.6092	314.99
16.682	67.8355	38.4199	266.72
16.684	67.2467	37.5308	269.44
16.686	67.5316	38.2922	265.12
16.688	67.6398	40.7830	243.51
16.690	66.9662	34.0979	298.02
16.692	67.6077	32.4607	318.68
16.694	67.2734	33.5142	306.10
16.696	67.4710	30.2198	337.76
16.698	67.6863	32.8677	315.71
16.700	67.3080	35.5143	288.28
16.702	67.2479	34.0638	300.89
16.704	67.8582	37.3220	276.88
16.706	67.2389	34.6773	295.24
16.708	67.5367	33.7486	306.36
16.710	67.8338	38.2031	268.67
16.712	67.1467	37.8923	265.25
16.714	67.5751	38.6831	261.97
16.716	67.2696	39.5184	251.62
16.718	67.5042	35.7660	287.77
16.720	67.5659	33.9868	304.47
16.722	67.2921	33.2930	308.28
16.724	67.1630	30.5479	331.99
16.726	67.6037	35.1874	293.92
16.728	67.3482	39.0211	256.85
16.730	67.2222	38.2044	263.11
16.732	67.6907	37.6641	272.25
16.734	67.2028	35.4896	287.55
16.736	67.2728	32.6232	314.17
16.738	67.4455	32.6583	315.42
16.740	67.5644	32.5316	317.65
16.742	67.4023	32.3888	317.47
16.744	67.5471	35.8689	287.23
16.746	67.2347	35.3695	288.93
16.748	67.2698	33.2882	308.12
16.750	67.5646	33.6379	307.62
16.752	67.0825	28.8499	346.66
16.754	67.7229	25.2211	385.37
16.756	67.5339	28.3605	355.19
16.758	67.1455	29.0496	345.42
16.760	67.4743	31.8938	322.61
16.762	67.7047	37.1182	277.33
16.764	67.1262	32.4039	314.83
16.766	67.6374	33.7321	307.42
16.768	67.3315	35.2600	290.80

16.770	66.9048	29.5531	338.67	
16.772	67.5735	30.9857	331.75	
16.774	67.2290	33.0835	309.60	
16.776	67.5989	32.0193	322.60	
16.778	67.7409	39.6042	255.12	
16.780	67.0081	39.3908	250.41	
16.782	67.2750	33.4539	306.66	
16.784	67.9508	37.3536	277.43	
16.786	67.0695	33.7184	302.40	
16.788	67.5508	34.6864	297.99	
16.790	67.5290	40.3298	246.62	
16.792	66.6081	36.5459	272.58	
16.794	67.8267	40.6262	246.63	
16.796	67.1027	42.4644	223.40	
16.798	67.4702	36.6292	279.64	
16.800	67.7580	41.0606	242.07	
16.802	66.9933	40.0127	244.64	
16.804	67.2230	34.1472	299.90	heating
16.806	68.1865	40.0694	254.94	heating
16.808	67.1417	35.6156	285.85	heating
16.810	67.8925	37.0094	280.02	heating
16.812	67.9004	41.1045	242.96	heating
16.814	66.8718	30.7628	327.41	heating
16.816	68.3454	31.5580	333.56	heating
16.818	67.4854	35.3707	291.19	heating
16.820	67.9891	29.2321	351.42	heating
16.822	68.0746	32.6470	321.23	
16.824	67.6425	34.3786	301.61	
16.826	67.7254	30.8992	333.91	
16.828	68.5784	36.9838	286.47	
16.830	67.2677	30.2077	336.03	
16.832	68.1636	30.0170	345.88	
16.834	68.1939	36.8296	284.38	
16.836	67.1581	31.4608	323.67	
16.838	68.2771	31.9953	328.97	
16.840	67.6164	33.9419	305.33	
16.842	68.0631	28.5005	358.72	
16.844	68.1237	32.8092	320.20	
16.846	67.6185	34.0047	304.78	
16.848	67.8438	31.9360	325.58	
16.850	68.4211	38.5257	271.07	
16.852	67.3367	33.4268	307.47	
16.854	68.0987	33.8617	310.43	
16.856	68.1445	39.2151	262.31	
16.858	67.4115	36.9659	276.05	
16.860	68.1358	39.3142	261.33	
16.862	67.6128	41.0272	241.06	
16.864	68.0774	38.9740	263.88	
16.866	68.0035	40.4105	250.19	
16.868	67.6831	36.7449	280.52	
16.870	67.7628	30.9009	334.23	
16.872	67.9821	30.2943	341.72	
16.874	67.6146	29.6654	344.09	
16.876	68.1956	35.5776	295.75	
16.878	67.9367	39.4284	258.49	
16.880	67.7926	38.7182	263.62	
16.882	68.0015	39.0910	262.14	
16.884	67.6201	36.3846	283.22	
16.886	68.3554	36.1763	291.77	
16.888	67.8413	36.1436	287.41	
16.890	67.8074	32.4743	320.37	
16.892	68.0856	33.0353	317.81	
16.894	67.6004	31.3931	328.30	
16.896	67.9272	29.8827	344.96	
16.898	68.1156	32.7290	320.86	
16.900	67.6812	27.8073	361.54	
16.902	68.1633	27.8434	365.59	
16.904	67.7904	30.8563	334.89	
16.906	67.6622	30.4868	337.08	
16.908	68.5250	38.0128	276.66	
16.910	67.6010	37.4926	273.00	
16.912	67.9274	33.0672	316.08	
16.914	68.1539	36.0047	291.50	
16.916	67.3187	31.3618	326.03	
16.918	68.0183	30.9094	336.47	
16.920	67.9339	34.3697	304.33	
16.922	67.5499	29.0388	349.19	
16.924	68.3148	34.1081	310.16	
16.926	67.5392	37.1677	275.38	
16.928	67.4490	31.0725	329.83	
16.930	68.4875	36.9550	285.91	
16.932	67.3808	35.5190	288.90	
16.934	68.0616	33.5241	313.16	
16.936	68.1696	39.7619	257.58	
16.938	67.2135	34.9820	292.25	
16.940	68.2611	36.9793	283.64	
16.942	67.9208	41.2157	242.14	
16.944	67.6186	32.5225	318.22	
16.946	68.5117	37.9457	277.15	
16.948	67.5418	38.6819	261.68	
16.950	67.4914	30.7108	333.49	
16.952	68.7101	38.5176	273.76	
16.954	67.2176	36.7568	276.19	
16.956	68.2394	36.7533	285.49	
16.958	68.1472	42.5316	232.26	
16.960	67.2079	34.7072	294.69	
16.962	68.2742	34.6185	305.16	
16.964	67.7624	38.4434	265.84	
16.966	67.5717	29.9569	341.06	
16.968	68.3168	33.6333	314.48	
16.970	67.6990	35.3384	293.42	
16.972	67.6075	32.5899	317.51	
16.974	68.5406	39.3506	264.67	
16.976	67.0386	33.5847	303.33	
16.978	68.3956	32.8010	322.74	
16.980	67.9671	37.2097	278.88	
16.982	67.4433	32.7755	314.34	
16.984	68.2443	34.8364	302.91	
16.986	67.6713	36.4272	283.29	
16.988	67.8578	28.8038	354.11	
16.990	68.2349	32.4826	324.17	
16.992	67.7703	30.2216	340.46	
16.994	67.6865	26.2318	375.88	
16.996	68.4809	32.5673	325.63	
16.998	67.1571	31.1177	326.77	
17.000	68.5379	33.9232	313.86	
17.002	67.8350	36.7186	282.14	
17.004	67.7491	35.0478	296.51	
17.006	68.0542	36.1998	288.83	
17.008	67.5333	33.0964	312.24	
17.010	68.0208	29.2735	351.33	
17.012	68.0254	35.6672	293.40	
17.014	67.8268	34.8162	299.31	
17.016	67.8310	33.1564	314.40	
17.018	67.9546	34.0642	307.29	
17.020	67.4587	32.8813	313.52	
17.022	68.6746	38.8898	270.06	
17.024	67.6040	37.6001	272.05	
17.026	68.0331	37.8915	273.30	
17.028	67.9266	42.3025	232.34	
17.030	67.7338	41.6059	236.91	
17.032	68.1786	40.8362	247.92	
17.034	67.7163	40.0686	250.69	
17.036	68.0025	35.4447	295.21	
17.038	68.0871	36.8043	283.65	
17.040	67.5925	33.4146	309.90	
17.042	67.8062	31.2408	331.54	
17.044	68.6821	39.2419	266.94	
17.046	67.5911	37.5060	272.79	
17.048	67.9538	36.3888	286.21	
17.050	67.9891	39.2132	260.92	
17.052	67.6876	35.7650	289.45	
17.054	68.3835	36.6104	288.09	
17.056	67.5834	34.4342	300.57	
17.058	68.0546	29.8726	346.20	
17.060	68.3021	35.5409	297.05	
17.062	67.4561	33.2975	309.72	
17.064	67.9418	30.6371	338.25	
17.066	68.4598	34.8836	304.44	
17.068	67.9126	31.0736	334.02	
17.070	68.2057	33.2695	316.77	
17.072	67.7284	32.3855	320.46	
17.074	67.7024	26.2699	375.67	
17.076	68.6674	33.5535	318.38	
17.078	67.4287	36.2256	282.92	
17.080	68.0150	36.4718	286.01	
17.082	68.2000	42.2675	235.13	
17.084	67.2948	36.3810	280.30	
17.086	68.0417	33.0835	316.97	
17.088	68.1840	38.3378	270.62	
17.090	67.6729	33.4069	310.69	
17.092	68.0285	35.3303	296.48	
17.094	67.8830	37.2491	277.76	
17.096	67.6598	31.7619	325.49	
17.098	68.7250	38.0291	278.32	
17.100	67.2649	36.0705	282.84	
17.102	68.0381	32.1278	325.60	
17.104	68.2212	37.8181	275.67	
17.106	67.4237	37.1020	274.93	
17.108	68.0952	37.7117	275.49	
17.110	68.0586	43.4109	223.48	
17.112	67.8268	38.9756	261.60	
17.114	67.9759	38.7185	265.28	
17.116	67.8677	36.3749	285.55	heating
17.118	67.9865	33.6871	311.00	heating
17.120	68.6237	39.6409	262.79	

17.122	67.3887	35.8682	285.80	heating
17.124	68.2682	31.7247	331.35	heating
17.126	68.2337	34.5242	305.65	
17.128	67.9381	35.4415	294.65	
17.130	68.0827	36.5258	286.13	
17.132	68.0004	36.9486	281.55	
17.134	68.1966	35.0401	300.63	
17.136	68.1382	38.1117	272.26	
17.138	68.1082	36.4260	287.27	
17.140	68.0044	32.0924	325.62	
17.142	68.4363	35.3451	300.04	
17.144	67.7570	34.5128	301.43	
17.146	68.2948	32.7476	322.31	
17.148	68.0834	31.0220	336.04	
17.150	67.9898	31.6781	329.24	
17.152	68.1862	34.6460	304.11	
17.154	67.7677	33.1404	313.97	
17.156	68.1924	32.7299	321.54	
17.158	68.1021	37.3540	278.80	
17.160	68.2468	38.6328	268.51	
17.162	68.2289	37.8138	275.78	
17.164	67.8628	34.3902	303.50	
17.166	68.2102	33.7389	312.56	
17.168	68.4243	36.2166	292.03	
17.170	67.9105	32.4815	321.24	
17.172	68.2809	33.6973	313.57	
17.174	68.1536	37.0312	282.19	
17.176	67.9260	35.2820	295.99	
17.178	68.4968	38.2036	274.67	
17.180	67.8895	36.8512	281.43	
17.182	68.2503	34.1360	309.32	
17.184	68.4023	37.6880	278.49	
17.186	67.5465	34.7278	297.57	
17.188	68.3969	33.5076	316.35	
17.190	68.3016	37.9109	275.56	
17.192	67.8900	32.3040	322.66	
17.194	68.3074	33.9408	311.61	
17.196	68.1757	38.1891	271.89	
17.198	67.7151	33.4185	310.97	
17.200	68.7582	38.0977	278.00	
17.202	67.8061	34.8271	299.03	
17.204	68.2458	31.0019	337.70	
17.206	68.5584	35.4061	300.60	
17.208	67.4283	31.2882	327.69	
17.210	68.5984	30.0340	349.67	
17.212	68.0501	34.3803	305.29	
17.214	68.0388	30.4867	340.49	
17.216	68.2470	32.9264	320.26	
17.218	68.2494	33.2334	317.50	
17.220	67.6218	23.7848	397.48	
17.222	68.7389	27.4795	374.10	
17.224	67.7957	29.6432	345.93	cooling
17.226	68.3193	34.6892	304.93	cooling
17.228	68.2908	41.7654	240.51	cooling
17.230	67.4093	38.3540	263.45	cooling
17.232	68.6571	37.3995	283.42	
17.234	68.0422	37.0810	280.73	
17.236	68.0386	30.9248	336.52	
17.238	67.9662	30.1162	343.19	
17.240	68.2287	32.0672	327.88	
17.242	67.8878	31.0050	334.42	
17.244	68.4586	37.4939	280.76	cooling
17.246	67.8279	38.5903	265.10	
17.248	68.1840	38.4765	269.36	
17.250	68.0899	38.6352	267.07	
17.252	67.6203	35.3144	292.92	
17.254	68.2529	35.2071	299.63	
17.256	68.0941	40.0343	254.42	
17.258	67.9194	38.7970	264.06	
17.260	67.7725	36.2953	285.41	
17.262	68.3259	36.1298	291.93	
17.264	68.0625	37.0091	281.57	
17.266	68.1619	37.2966	279.86	
17.268	67.9065	35.5693	293.21	
17.270	68.0852	33.3630	314.83	
17.272	68.1775	34.9117	301.63	
17.274	67.9832	38.2038	270.01	
17.276	67.7794	36.3465	285.01	
17.278	68.3124	38.6789	268.69	
17.280	67.7814	37.5482	274.13	
17.282	67.9613	32.5606	320.98	
17.284	68.5417	31.2051	338.54	
17.286	67.7572	27.8397	361.94	
17.288	68.0669	26.8962	373.30	
17.290	68.0659	30.1280	343.99	
17.292	68.1492	29.9257	346.58	
17.294	68.0832	32.6832	320.98	
17.296	68.2338	37.7631	276.28	
17.298	67.6401	32.7752	316.13	
17.300	68.3549	32.3373	326.58	
17.302	67.7849	34.5926	300.96	
17.304	67.8439	34.4011	303.23	
17.306	68.5585	37.8574	278.37	
17.308	67.8185	38.3053	267.60	
17.310	67.9057	36.4560	285.16	
17.312	68.3187	38.9759	266.06	
17.314	68.1063	36.2610	288.75	
17.316	68.1653	34.3518	306.59	
17.318	68.1314	38.0336	272.90	
17.320	67.8700	35.9743	289.20	
17.322	68.1226	34.2667	306.98	
17.324	68.1308	38.8623	265.38	
17.326	67.7230	36.7811	280.55	
17.328	68.3947	35.1314	301.60	
17.330	68.0554	35.6004	294.27	
17.332	67.7727	35.1249	296.02	
17.334	68.2678	38.4653	270.22	
17.336	67.9204	38.0632	270.72	
17.338	68.1005	34.3792	305.76	
17.340	68.1084	36.2588	288.79	
17.342	67.9038	35.8991	290.19	
17.344	67.8933	32.6691	319.38	
17.346	68.3363	35.9165	293.95	
17.348	67.6209	32.9941	313.97	
17.350	68.1394	30.7101	339.38	
17.352	68.2859	33.8187	312.52	
17.354	67.6580	32.2228	321.30	
17.356	68.2245	32.3597	325.19	
17.358	67.8709	32.8117	317.89	
17.360	68.0243	28.4242	359.06	
17.362	68.2232	33.4107	315.65	
17.364	68.0099	36.1355	289.01	
17.366	67.6131	31.5090	327.36	
17.368	68.4294	34.2414	309.99	
17.370	67.4702	34.0416	303.10	
17.372	68.1869	36.4192	288.04	
17.374	68.0766	39.0827	262.89	
17.376	67.5969	35.2872	292.96	
17.378	68.0395	33.4393	313.73	
17.380	67.9159	34.8003	300.26	
17.382	67.9110	29.7102	346.37	
17.384	68.1183	32.5963	322.08	
17.386	68.0183	35.4397	295.40	
17.388	67.7502	32.7356	317.48	
17.390	68.4221	36.8299	286.45	
17.392	67.4709	35.9060	286.20	
17.394	68.2523	34.4917	306.11	
17.396	68.0173	32.5331	321.74	
17.398	67.7452	28.2806	357.83	
17.400	67.9597	29.6499	347.36	
17.402	68.0152	35.3160	296.49	
17.404	67.8200	34.2009	304.83	
17.406	67.9011	34.6608	301.39	
17.408	68.0087	35.8123	291.93	
17.410	67.8205	36.5413	283.61	
17.412	67.9348	36.5107	284.93	
17.414	67.5457	33.3100	310.42	
17.416	68.0427	31.4048	332.20	
17.418	67.8946	31.9553	325.87	
17.420	67.9946	34.0583	307.71	
17.422	67.7679	35.9756	288.27	
17.424	67.9478	37.0324	280.31	
17.426	67.9426	36.2148	287.68	
17.428	67.9021	33.7615	309.56	
17.430	68.0346	30.9230	336.50	
17.432	67.9310	30.5875	338.60	
17.434	67.6612	27.0846	367.91	
17.436	67.8359	25.7678	381.44	cooling
17.438	68.0173	29.0273	353.53	cooling
17.440	67.6040	32.9875	313.87	cooling
17.442	67.9744	38.5293	266.98	cooling
17.444	67.4682	38.3542	263.98	cooling
17.446	67.4841	32.0953	320.88	cooling
17.448	67.7748	32.4357	320.42	cooling
17.450	67.6819	34.3222	302.48	cooling
17.452	67.7578	34.9792	297.21	cooling
17.454	67.7987	39.3676	257.79	cooling
17.456	67.0280	35.5044	285.83	
17.458	67.8599	35.1191	296.87	
17.460	67.8382	37.6692	273.55	
17.462	67.2691	31.9205	320.51	
17.464	67.8670	32.1310	324.02	
17.466	67.3405	32.5210	315.71	
17.468	67.4274	30.7313	332.73	
17.470	67.9078	37.1378	279.00	
17.472	67.2525	36.5945	277.98	

17.474	67.7851	35.1412	295.99	
17.476	67.6732	38.2265	267.00	
17.478	66.9305	32.5964	311.31	
17.480	67.8157	32.9180	316.42	
17.482	67.8347	39.9623	252.72	
17.484	67.0983	32.0090	318.16	
17.486	67.8369	31.6437	328.17	
17.488	67.5105	33.4114	309.18	
17.490	67.0630	28.2943	351.52	
17.492	68.3525	36.3980	289.74	
17.494	66.8204	34.3564	294.36	
17.496	67.9397	31.3922	331.38	
17.498	67.8164	37.6259	273.74	
17.500	67.1342	34.7062	294.03	
17.502	67.7715	32.3194	321.45	
17.504	67.6602	35.1718	294.58	
17.506	67.3997	28.3925	353.68	
17.508	67.8860	33.2194	314.33	
17.510	67.7594	36.5173	283.28	
17.512	67.1381	32.4842	314.21	
17.514	68.3155	36.9956	283.98	
17.516	66.9024	33.8393	299.79	
17.518	68.0694	32.8835	319.04	
17.520	67.7049	36.7616	280.57	
17.522	67.2497	34.1989	299.68	
17.524	67.7671	34.4648	301.96	
17.526	67.6151	38.1664	267.02	
17.528	67.4312	33.7557	305.34	
17.530	67.6246	34.7955	297.67	
17.532	67.8315	36.8067	281.31	
17.534	67.3758	36.2691	282.05	
17.536	67.7592	37.0943	278.04	
17.538	67.3349	36.6316	278.39	
17.540	67.9523	37.2375	278.50	
17.542	67.4302	35.3097	291.24	
17.544	67.6155	34.6245	299.13	
17.546	67.4005	32.6070	315.48	
17.548	67.6615	33.5380	309.40	
17.550	67.7035	36.2027	285.62	
17.552	67.3331	35.0478	292.74	
17.554	67.8216	32.4158	321.03	
17.556	67.7368	35.3615	293.55	
17.558	67.3142	32.9604	311.49	
17.560	67.5606	30.7653	333.63	
17.562	67.9904	33.6128	311.71	
17.564	67.3101	30.9846	329.37	
17.566	67.8059	34.6805	300.35	
17.568	67.4017	37.7329	269.01	
17.570	67.3245	36.2151	282.07	
17.572	67.8728	38.6963	264.55	
17.574	67.1053	33.3590	305.98	
17.576	67.8922	29.5099	348.02	
17.578	67.8160	34.1178	305.55	
17.580	66.9592	29.5338	339.34	
17.582	67.7956	29.1591	350.32	
17.584	67.8043	35.4800	293.09	
17.586	67.2367	32.4116	315.76	
17.588	68.0419	40.0452	253.85	
17.590	67.3801	43.7732	214.05	
17.592	67.0654	35.0722	290.09	
17.594	68.1935	38.8062	266.46	
17.596	67.0078	35.8938	282.11	
17.598	67.9080	34.9914	298.46	
17.600	67.9258	43.3387	222.93	
17.602	66.7047	37.2245	267.30	
17.604	67.7510	35.5177	292.26	
17.606	67.9087	43.6088	220.33	
17.608	67.2057	36.1941	281.19	heating
17.610	68.0210	36.7065	283.93	heating
17.612	67.5773	37.2083	275.36	heating
17.614	67.2003	29.3496	343.20	heating
17.616	68.7280	37.7842	280.57	
17.618	67.0578	36.6105	276.07	heating
17.620	68.1746	33.5979	313.51	heating
17.622	68.3254	41.5489	242.79	
17.624	67.0767	32.8831	310.04	heating
17.626	68.1218	28.5855	358.48	heating
17.628	68.3261	34.9130	302.96	
17.630	67.6037	26.9092	368.98	
17.632	68.5600	30.6182	344.02	
17.634	67.6568	31.7440	325.63	
17.636	67.5354	26.3314	373.60	
17.638	68.8070	35.5458	301.58	
17.640	67.2553	34.5662	296.40	
17.642	68.1111	27.4965	368.26	
17.644	68.3416	35.4427	298.30	
17.646	67.6248	35.4929	291.34	
17.648	67.8544	32.4750	320.79	
17.650	68.3725	35.5746	297.38	
17.652	67.2969	27.7258	358.80	
17.654	68.6155	31.4600	336.89	
17.656	67.7838	33.6994	309.05	
17.658	67.5494	28.3275	355.63	
17.660	68.4727	33.1824	319.98	cooling
17.662	67.3345	34.2131	300.32	cooling
17.664	68.1095	30.9458	336.97	cooling
17.666	68.0124	38.1524	270.75	cooling
17.668	67.6544	38.5963	263.47	cooling
17.670	67.4336	35.0763	293.39	
17.672	68.2227	37.9661	274.34	
17.674	67.1843	33.6190	304.34	
17.676	68.3922	36.2436	291.50	
17.678	67.7622	39.5960	255.39	
17.680	67.4761	35.4491	290.39	
17.682	67.8215	33.6616	309.73	
17.684	67.9672	36.1513	288.48	
17.686	67.8455	33.0361	315.62	
17.688	67.9342	34.9469	299.10	
17.690	67.7659	33.7364	308.55	
17.692	67.5301	31.9617	322.50	
17.694	68.1040	34.4831	304.85	
17.696	67.3533	34.1116	301.41	
17.698	68.3237	35.1752	300.56	
17.700	67.6594	35.7254	289.55	
17.702	67.6354	33.2771	311.53	
17.704	67.4292	30.1397	338.11	
17.706	68.1355	32.8810	319.66	
17.708	67.5948	31.1720	330.25	
17.710	67.6618	30.3074	338.70	
17.712	67.8949	30.8664	335.74	
17.714	67.6268	33.0230	313.76	
17.716	67.9006	33.6958	310.14	
17.718	67.6016	34.1556	303.26	
17.720	68.2256	33.1046	318.45	
17.722	67.7363	31.3607	329.82	cooling
17.724	67.9576	34.2104	305.99	cooling
17.726	67.1245	29.6561	339.73	cooling
17.728	67.9059	30.3875	340.19	cooling
17.730	67.8168	35.5700	292.39	cooling
17.732	67.1621	33.1438	308.45	cooling
17.734	67.8696	33.9074	307.94	cooling
17.736	67.4095	37.8369	268.14	cooling
17.738	67.1485	34.3923	297.01	cooling
17.740	67.6050	34.8536	296.96	
17.742	67.3329	32.5529	315.36	
17.744	67.5317	31.2109	329.33	
17.746	67.7146	39.1907	258.63	cooling
17.748	66.7272	36.3587	275.36	
17.750	67.5794	35.5420	290.49	
17.752	67.7848	42.4424	229.78	
17.754	66.8349	37.7078	264.10	
17.756	67.7803	38.2787	267.49	
17.758	67.1559	37.3988	269.81	
17.760	67.0041	31.0104	326.36	
17.762	68.0059	38.3260	269.11	
17.764	66.8843	40.0849	242.99	
17.766	67.7012	38.2334	267.19	
17.768	67.6871	45.8097	198.37	
17.770	66.7975	38.5122	256.47	
17.772	67.3267	29.7235	340.95	heating
17.774	68.0017	33.5684	312.21	
17.776	66.8494	27.8538	353.58	
17.778	67.9137	31.5114	330.07	
17.780	67.2308	35.8898	284.17	
17.782	67.2046	33.8270	302.64	
17.784	67.8667	38.3003	268.08	
17.786	66.9887	36.9569	272.30	
17.788	67.6386	31.3864	328.70	
17.790	67.4576	33.7522	305.61	
17.792	67.1389	30.1585	335.31	
17.794	67.3095	29.7107	340.91	
17.796	67.8385	36.6522	282.77	
17.798	66.8858	35.2534	286.82	
17.800	67.8104	35.6622	291.49	
17.802	67.2411	34.0169	301.25	
17.804	67.3943	32.0993	320.02	
17.806	67.4791	32.6594	315.72	
17.808	67.3763	32.4730	316.47	
17.810	67.6254	31.9772	323.23	
17.812	67.4485	37.2351	273.95	
17.814	67.4795	37.8836	268.35	
17.816	67.2928	36.0307	283.46	
17.818	67.5732	35.2746	292.86	
17.820	67.2474	35.7171	285.89	
17.822	67.8451	36.0294	288.48	
17.824	67.2728	33.6800	304.59	

17.826	67.8652	38.6675	264.74	
17.828	67.0394	38.3145	260.45	
17.830	67.6774	35.6233	290.64	
17.832	67.4513	34.5235	298.56	
17.834	67.3163	34.2119	300.16	
17.836	67.5961	36.3222	283.57	
17.838	67.2593	36.9627	274.70	
17.840	67.2401	34.4257	297.53	
17.842	67.5580	39.9757	250.09	
17.844	67.6436	41.7232	235.02	
17.846	67.2085	36.0009	282.96	heating
17.848	67.8145	34.2648	304.20	heating
17.850	67.0693	30.9821	327.21	heating
17.852	68.0941	31.5039	331.77	
17.854	67.4755	33.1722	311.03	
17.856	67.4192	31.1452	328.90	
17.858	67.8481	34.7438	300.16	
17.860	67.5167	36.2343	283.64	
17.862	66.9939	26.7607	364.80	
17.864	68.2040	33.0358	318.88	
17.866	67.3915	34.5605	297.68	
17.868	67.3204	29.8864	339.42	
17.870	68.0741	35.1322	298.69	
17.872	66.8811	34.4840	293.75	
17.874	67.9993	35.7127	292.75	
17.876	67.5878	39.4811	254.85	
17.878	67.0704	29.5407	340.29	
17.880	68.0264	29.9538	345.21	
17.882	67.6685	35.3650	292.90	
17.884	66.8374	27.6358	355.45	
17.886	68.5233	36.7594	288.01	
17.888	67.1758	40.1836	244.74	
17.890	67.4845	35.2638	292.15	
17.892	68.1028	41.1130	244.72	
17.894	66.8694	36.4868	275.48	
17.896	67.8586	34.3492	303.83	
17.898	67.9006	40.6711	246.89	
17.900	66.9431	32.8123	309.47	
17.902	68.2597	37.9706	274.64	
17.904	67.6387	44.8197	206.90	
17.906	66.9510	34.3799	295.33	
17.908	68.3443	36.3093	290.47	
17.910	67.2579	37.5810	269.09	
17.912	67.6802	33.4649	310.24	heating
17.914	68.2100	41.7641	239.79	
17.916	67.1653	38.1805	262.81	heating
17.918	67.8358	35.8529	289.99	heating
17.920	68.3249	42.9708	229.89	heating
17.922	66.9308	33.1901	305.93	heating
17.924	68.6020	34.9091	305.50	heating
17.926	67.7988	39.4611	256.94	heating
17.928	67.5359	32.2971	319.52	heating
17.930	68.6615	36.3379	293.08	heating
17.932	67.6337	37.8324	270.21	heating
17.934	68.1929	35.3373	297.91	heating
17.936	68.4646	40.8827	250.09	heating
17.938	67.6347	31.7762	325.13	heating
17.940	68.3067	29.1127	355.38	heating
17.942	68.6391	37.0902	286.06	heating
17.944	67.7044	31.8932	324.71	heating
17.946	69.1730	34.3591	315.66	
17.948	67.9763	35.2165	297.04	
17.950	68.3532	31.3944	335.11	
17.952	68.4635	30.7490	341.96	
17.954	68.1881	27.6876	367.22	
17.956	68.3305	27.9450	366.18	
17.958	68.3614	34.3486	308.40	
17.960	68.1555	33.8586	310.98	
17.962	68.5981	38.1448	276.12	
17.964	68.1952	38.4297	269.89	
17.966	68.1493	33.9640	309.96	
17.968	68.7951	30.3919	348.21	
17.970	67.7842	24.5707	391.82	
17.972	68.6874	27.6370	372.21	
17.974	68.1207	32.1524	326.13	
17.976	68.3878	32.0533	329.45	cooling
17.978	68.2591	36.6377	286.72	cooling
17.980	67.9551	38.5303	266.80	cooling
17.982	68.3674	36.5131	288.83	
17.984	68.3793	38.0648	274.87	
17.986	67.7851	32.0844	323.70	
17.988	68.6314	36.6197	290.25	
17.990	68.2063	39.5368	259.95	
17.992	67.9584	36.2232	287.75	
17.994	68.6659	42.6519	235.87	
17.996	67.5778	38.1553	266.78	
17.998	68.3900	32.4317	326.04	
18.000	68.2693	34.4230	306.89	
18.002	67.7869	29.8127	344.32	
18.004	68.7276	33.5295	319.15	
18.006	68.0781	37.9679	273.01	
18.008	67.9425	34.7133	301.29	
18.010	68.8168	41.0079	252.15	
18.012	67.8042	38.7097	263.80	
18.014	68.1500	32.2455	325.55	
18.016	68.4058	36.0481	293.39	
18.018	67.6801	32.2497	321.25	
18.020	68.2687	31.0949	337.06	
18.022	68.2619	35.8026	294.31	
18.024	67.8735	32.1029	324.34	
18.026	68.6712	34.1125	313.35	
18.028	67.8825	30.7132	337.02	
18.030	67.8707	25.1531	387.33	
18.032	68.7502	32.9419	324.68	
18.034	67.7605	35.8539	289.30	
18.036	68.3529	35.3445	299.29	
18.038	68.0262	38.9671	263.48	
18.040	68.0069	36.3521	287.02	
18.042	68.1237	34.2816	306.85	
18.044	68.2829	35.5957	296.38	
18.046	67.6898	30.0853	340.97	
18.048	68.6026	33.0433	322.42	
18.050	67.9367	35.1299	297.46	
18.052	67.8958	32.7784	318.41	
18.054	68.5813	38.0395	276.93	
18.056	67.8647	37.4689	275.60	
18.058	68.1401	31.4691	332.50	
18.060	68.1390	32.7126	321.22	
18.062	67.9375	32.3985	322.24	
18.064	68.1829	35.3416	297.78	
18.066	68.1071	38.7409	266.27	
18.068	67.7465	35.4602	292.74	
18.070	68.4893	37.3374	282.46	
18.072	68.0041	37.3072	278.33	
18.074	67.9996	31.5210	330.76	
18.076	68.2191	28.7910	357.50	
18.078	68.2041	28.6407	358.73	
18.080	67.9472	29.0365	352.81	
18.082	68.2581	36.0625	291.92	cooling
18.084	67.9860	38.0071	271.82	
18.086	68.0132	37.8577	273.42	
18.088	67.8870	35.2108	296.28	
18.090	67.8097	31.0881	332.96	
18.092	68.4554	32.1955	328.77	
18.094	67.7546	34.0791	305.34	
18.096	68.0974	34.1121	308.15	
18.098	67.9180	34.0661	306.94	
18.100	68.1111	34.1627	307.82	
18.102	67.8841	35.6470	292.30	
18.104	68.1555	37.7497	275.69	
18.106	67.8515	35.1195	296.79	
18.108	68.0611	35.2452	297.55	
18.110	68.0022	35.9335	290.77	
18.112	68.3459	38.3105	272.33	
18.114	68.2993	39.4186	261.87	
18.116	67.7381	36.9892	278.80	
18.118	68.3157	35.3762	298.67	
18.120	67.9384	32.8646	318.02	
18.122	68.3670	33.9567	312.00	
18.124	67.9171	36.4000	285.77	
18.126	68.0715	36.0260	290.56	
18.128	68.2287	37.7246	276.59	
18.130	68.0167	38.7434	265.43	
18.132	68.0426	35.5966	294.19	
18.134	68.5266	38.6408	270.98	
18.136	68.0068	36.3017	287.47	
18.138	67.9611	34.1344	306.71	
18.140	68.4626	36.7864	287.21	
18.142	67.7193	32.7626	316.96	
18.144	68.3837	32.7580	323.02	
18.146	68.0595	35.2845	297.18	
18.148	67.8098	31.8159	326.36	
18.150	68.4172	33.0171	320.98	
18.152	67.8384	34.6494	300.93	
18.154	68.1230	33.3794	315.02	
18.156	68.3772	36.2709	291.11	
18.158	67.9790	36.7259	283.38	
18.160	68.1631	36.5958	286.23	
18.162	68.0590	37.1768	280.01	
18.164	68.0944	33.4199	314.40	
18.166	68.3300	32.7772	322.36	
18.168	68.2271	33.0845	318.64	
18.170	67.9393	30.6329	338.26	
18.172	68.1947	29.3678	352.05	
18.174	67.9235	27.9653	362.31	
18.176	68.3671	30.0942	347.03	

18.178	68.0741	31.6288	330.45	
18.180	68.1152	33.2852	315.81	cooling
18.182	68.1394	33.7066	312.21	cooling
18.184	67.9939	34.5411	303.32	cooling
18.186	68.3455	35.9566	293.68	cooling
18.188	67.8919	36.6706	283.09	
18.190	68.1219	34.7193	302.87	
18.192	67.9059	35.1535	296.97	
18.194	67.9278	33.1981	314.90	
18.196	67.8144	31.0331	333.50	
18.198	68.4971	36.0821	293.91	
18.200	67.4906	34.6681	297.61	
18.202	68.3212	36.7833	285.96	
18.204	67.7752	37.4480	274.98	
18.206	68.0729	36.9550	282.15	
18.208	68.5079	41.8138	242.04	
18.210	67.7426	41.4946	237.99	
18.212	68.2683	36.9028	284.40	
18.214	68.1244	39.1878	262.37	
18.216	68.0052	37.7211	274.59	
18.218	67.9389	37.5478	275.56	
18.220	68.2917	40.0433	256.13	
18.222	67.4677	33.4877	308.10	
18.224	68.3985	34.9047	303.69	
18.226	67.4877	34.6248	297.97	
18.228	68.1194	33.4635	314.23	
18.230	68.4175	38.0845	275.03	
18.232	67.5302	38.0134	267.63	
18.234	68.1105	34.5710	304.11	
18.236	68.3466	39.3194	263.19	
18.238	67.9328	34.8137	300.30	
18.240	67.9567	34.9292	299.47	
18.242	68.2202	35.2519	298.93	
18.244	67.6087	27.6388	362.41	
18.246	68.6749	32.8748	324.60	
18.248	67.3920	34.9862	293.83	
18.250	68.3141	34.0337	310.82	
18.252	68.5025	36.9015	286.53	
18.254	67.7089	35.1469	295.24	
18.256	68.0248	33.6575	311.61	
18.258	68.6093	40.5753	254.19	
18.260	68.1232	35.9117	292.07	
18.262	68.0995	36.8006	283.79	
18.264	68.1620	38.6429	267.65	
18.266	67.8232	32.7688	317.84	
18.268	68.8179	35.0786	305.92	
18.270	67.5677	34.4743	300.06	
18.272	68.3820	32.2028	328.04	
18.274	68.4257	35.3494	299.91	
18.276	68.0472	33.3650	314.47	
18.278	68.1926	33.6354	313.34	
18.280	68.4200	39.7542	259.92	
18.282	67.9794	36.5335	285.12	
18.284	68.1917	35.1274	299.80	
18.286	67.9863	35.3376	296.03	
18.288	67.9126	33.6211	310.93	
18.290	68.2158	34.1622	308.77	
18.292	67.8355	30.8353	335.49	
18.294	68.0888	29.0770	353.73	
18.296	68.2095	31.7087	330.96	
18.298	67.8637	32.0445	324.78	
18.300	68.3797	35.1421	301.37	
18.302	67.7519	34.8379	298.44	
18.304	68.2864	34.6111	305.34	
18.306	68.0929	34.3706	305.77	
18.308	68.1502	32.5082	323.17	
18.310	68.0966	33.6366	312.45	
18.312	67.8859	35.4239	294.34	
18.314	68.0780	32.6830	320.93	
18.316	68.1153	33.4785	314.06	
18.318	68.0296	33.3304	314.62	
18.320	67.8912	33.6121	310.81	
18.322	68.5089	36.2189	292.78	
18.324	67.5828	33.0525	313.09	
18.326	68.2664	34.8316	303.16	
18.328	68.0679	38.2161	270.67	
18.330	68.1470	36.3844	288.00	
18.332	68.0949	36.4207	287.19	
18.334	67.8795	37.3024	277.25	
18.336	68.0618	33.9695	309.12	
18.338	68.3427	37.0075	284.12	
18.340	67.7958	32.9924	315.57	
18.342	68.0246	33.3981	313.96	
18.344	68.5559	38.3890	273.53	
18.346	67.4307	34.0463	302.70	
18.348	68.5228	36.3886	291.37	
18.350	67.8084	37.1695	277.81	
18.352	68.4420	34.8603	304.49	

18.354	68.4236	38.8884	267.80
18.356	67.6977	39.3306	257.21
18.358	68.1985	34.4759	305.77
18.360	68.5388	38.0587	276.37
18.362	67.7766	34.3787	302.82
18.364	68.0144	34.4956	303.92
18.366	68.5685	38.1694	275.63
18.368	67.3701	31.3603	326.51
18.370	68.5878	32.7958	324.53
18.372	67.5112	32.7364	315.31
18.374	68.4236	32.3336	327.23
18.376	68.1827	37.0326	282.44
18.378	67.4484	35.8831	286.21
18.380	68.1449	33.2685	316.23
18.382	68.4825	38.7578	269.52
18.384	67.8338	34.7598	299.89
18.386	68.0310	33.5904	312.28
18.388	68.0413	32.8414	319.16
18.390	67.6977	27.6951	362.71
18.392	68.5551	33.8558	314.62
18.394	67.6667	35.7566	289.33
18.396	68.3056	32.3505	326.01
18.398	68.3165	35.2496	299.82
18.400	67.5280	31.2642	328.81
18.402	68.3392	30.6020	342.17
18.404	68.6051	37.7208	280.03
18.406	67.9602	35.5099	294.23
18.408	68.3871	37.3643	281.29
18.410	67.9810	37.8712	273.01
18.412	67.9873	32.9789	317.43
18.414	68.4845	36.8157	287.15
18.416	67.9013	37.6377	274.40
18.418	68.0429	32.5959	321.40
18.420	68.2323	34.5778	305.15
18.422	67.9401	33.3871	313.30
18.424	68.1270	34.8936	301.33
18.426	68.4472	41.5660	243.74
18.428	67.7465	37.7320	272.15
18.430	68.4394	37.9938	276.06
18.432	68.0791	39.3848	260.18
18.434	67.8176	36.5384	283.61
18.436	68.3961	38.3760	272.20
18.438	67.9398	38.0386	271.12
18.440	67.9784	35.2573	296.69
18.442	67.9075	35.8739	290.45
18.444	68.0810	36.6568	284.93
18.446	68.2577	36.3013	289.75
18.448	68.0077	35.3403	296.20
18.450	68.0423	32.9817	317.90
18.452	68.0774	32.7952	319.91
18.454	68.5427	35.8745	296.21
18.456	67.9917	39.1847	261.20
18.458	68.3184	39.7933	258.64
18.460	68.0093	36.2356	288.10
18.462	68.5549	36.9947	286.16
18.464	67.7098	32.1678	322.26
18.466	68.2199	31.6933	331.19
18.468	68.4523	32.3823	327.05
18.470	67.6692	31.4070	328.79
18.472	68.5862	36.4172	291.68
18.474	67.5213	36.1889	284.10
18.476	68.7389	33.4177	320.26
18.478	68.0074	36.1324	289.02
18.480	67.9760	34.9455	299.49
18.482	68.1002	32.5278	322.54
18.484	68.7913	38.6788	273.03
18.486	67.6282	31.3310	329.11
18.488	68.4235	34.8776	304.17
18.490	68.5442	38.6575	270.99
18.492	67.4086	31.1187	329.05
18.494	68.7577	33.3551	321.00
18.496	67.3590	32.9590	311.91
18.498	68.8843	29.0749	360.96
18.500	68.3592	38.1536	273.88
18.502	67.9530	41.0163	244.24
18.504	67.8460	36.1889	287.04
18.506	69.3539	44.2760	227.38
18.508	67.4028	33.9672	303.16
18.510	68.6016	35.2225	302.65
18.512	68.4300	37.2744	282.49
18.514	67.4170	30.3472	336.12
18.516	68.9584	36.5507	293.85
18.518	67.2412	38.2175	263.16
18.520	68.7366	34.9693	306.17
18.522	68.3585	41.3899	244.53
18.524	67.6490	38.0915	268.00
18.526	67.9606	32.5987	320.63
18.528	68.8464	40.2678	259.13

18.530	67.6584	34.8577	297.41	
18.532	68.5902	37.6365	280.66	
18.534	68.2582	38.6064	268.86	
18.536	67.5628	29.9616	340.94	
18.538	68.7624	32.3639	330.03	
18.540	67.6128	33.0337	313.53	
18.542	68.3686	29.4954	352.47	
18.544	68.2586	32.6369	322.99	
18.546	67.8517	29.8561	344.51	
18.548	68.1634	29.1831	353.44	
18.550	68.2032	36.2211	289.99	
18.552	68.0124	36.4081	286.56	
18.554	68.3573	36.4546	289.27	
18.556	68.1929	34.1418	308.75	
18.558	67.8068	29.3611	348.59	
18.560	68.3887	31.2821	336.45	
18.562	68.0256	32.8560	318.89	
18.564	68.0796	31.8654	328.36	
18.566	67.8775	32.2409	323.12	
18.568	68.3131	33.9553	311.53	
18.570	68.0761	33.8540	310.30	
18.572	67.9502	35.1086	297.78	
18.574	68.1419	35.9333	292.04	
18.576	68.0620	33.9478	309.32	
18.578	68.5195	32.8578	323.35	
18.580	67.9173	35.7162	291.97	
18.582	68.1567	35.6040	295.16	
18.584	68.1738	36.0853	290.95	
18.586	68.2329	37.4279	279.31	
18.588	67.5577	31.6436	325.64	
18.590	68.5103	34.7248	306.34	
18.592	68.0441	37.2934	278.82	
18.594	67.6460	34.7571	298.21	
18.596	68.2749	37.5246	278.82	
18.598	67.9472	37.4714	276.33	
18.600	68.5418	33.0614	321.71	
18.602	68.1304	36.1170	290.27	
18.604	67.7902	35.2970	294.62	
18.606	68.1191	34.5979	303.94	
18.608	68.5265	39.2559	265.40	
18.610	67.3489	33.3524	308.25	
18.612	68.5577	35.2158	302.32	
18.614	68.0878	36.4015	287.30	
18.616	67.7656	33.3930	311.66	
18.618	68.1129	36.2923	288.52	
18.620	67.8712	36.2598	286.63	
18.622	68.6560	31.5557	336.39	
18.624	68.3393	36.0344	292.91	
18.626	67.8740	34.6279	301.45	
18.628	67.9997	32.2752	323.92	
18.630	68.7619	36.5924	291.69	
18.632	67.1762	30.3796	333.64	
18.634	68.7408	31.3743	338.81	
18.636	67.9674	34.5541	302.96	
18.638	67.9712	32.0743	325.48	
18.640	67.9382	34.3272	304.76	
18.642	68.1257	36.0123	291.18	
18.644	68.4225	29.2235	355.42	
18.646	68.4836	34.6990	306.33	
18.648	67.9888	33.9309	308.81	
18.650	67.9169	33.9199	308.26	
18.652	68.7262	37.7328	281.02	
18.654	67.3057	32.4553	315.99	
18.656	68.8875	36.0668	297.59	
18.658	67.7030	37.1342	277.17	
18.660	68.0544	33.4283	313.96	
18.662	67.8688	34.2131	305.16	
18.664	68.1052	37.6367	276.26	
18.666	68.1188	34.9410	300.83	
18.668	68.4767	41.4361	245.18	
18.670	68.0924	37.9285	273.50	
18.672	68.0371	35.9802	290.66	
18.674	68.6139	38.2090	275.69	
18.676	67.4360	34.3067	300.39	
18.678	69.2611	39.3561	271.15	
18.680	67.6501	39.0455	259.36	
18.682	68.2652	36.3781	289.12	
18.684	68.0379	36.7654	283.55	
18.686	67.8662	36.4128	285.19	
18.688	67.9781	35.4072	295.33	
18.690	68.0767	38.6149	267.13	
18.692	68.2322	33.4624	315.26	
18.694	68.0755	33.8638	310.20	
18.696	68.1644	35.2778	298.19	
18.698	67.7940	33.2941	312.82	
18.700	69.0877	36.1222	298.90	
18.702	67.7076	31.5827	327.55	
18.704	68.3876	31.1290	337.83	
18.706	68.1912	33.4865	314.67	
18.708	67.7654	31.1355	332.13	
18.710	68.0963	31.2591	334.01	
18.712	67.8583	32.0722	324.48	
18.714	68.5468	29.2623	356.20	
18.716	68.3481	31.5763	333.42	
18.718	67.9262	31.5398	329.92	
18.720	68.1129	30.3102	342.76	
18.722	68.7203	32.5465	327.99	
18.724	68.1407	29.1861	353.21	cooling
18.726	68.1298	32.4531	323.49	cooling
18.728	67.9503	36.5525	284.69	cooling
18.730	67.5277	35.0666	294.33	cooling
18.732	67.8540	36.3067	286.04	cooling
18.734	67.2731	34.5513	296.69	cooling
18.736	68.4839	34.2788	310.14	
18.738	68.2278	37.4555	279.02	
18.740	67.6077	35.0904	294.84	
18.742	67.8090	33.7043	309.23	
18.744	68.1186	35.1125	299.27	
18.746	67.9141	30.2153	341.82	
18.748	68.1034	34.3008	306.49	
18.750	67.8707	36.7824	281.88	
18.752	67.5874	36.0866	285.62	
18.754	67.9169	38.9477	262.67	
18.756	67.2142	35.7425	285.36	
18.758	68.4127	35.0254	302.73	
18.760	68.1286	37.7479	275.47	
18.762	67.8577	35.9217	289.57	
18.764	67.7950	34.6186	300.82	
18.766	68.0427	36.1707	288.99	
18.768	67.8882	32.7169	318.90	
18.770	68.2680	36.3835	289.10	
18.772	68.0133	36.1977	288.48	
18.774	67.6225	35.0776	295.09	
18.776	68.0309	37.2238	279.33	
18.778	67.1106	33.0680	308.67	
18.780	68.4874	33.9288	313.35	
18.782	67.7552	35.4768	292.67	
18.784	67.9702	35.8387	291.34	
18.786	67.7185	36.2334	285.48	
18.788	67.6263	35.1918	294.09	
18.790	67.9163	33.4685	312.34	
18.792	68.0521	34.3327	305.74	
18.794	68.2668	32.0094	328.75	
18.796	67.8893	34.4963	302.78	
18.798	67.7771	35.3652	293.88	
18.800	67.4457	32.9232	313.02	
18.802	68.7551	36.4239	293.15	
18.804	67.4345	34.1195	302.07	
18.806	68.1295	33.4291	314.63	
18.808	67.6156	33.6237	308.21	
18.810	67.5794	31.7672	324.71	
18.812	67.6650	33.3745	310.92	
18.814	67.9708	36.7313	283.25	
18.816	68.2572	34.5260	305.85	
18.818	67.9798	37.7982	273.66	
18.820	67.2323	34.1198	300.24	
18.822	67.8137	33.6957	309.35	
18.824	68.5803	39.3537	265.00	
18.826	67.2732	35.6054	287.14	
18.828	68.2238	36.8076	284.85	
18.830	67.3559	35.9212	285.02	
18.832	67.6364	31.0711	331.54	
18.834	67.5041	33.6976	306.53	
18.836	67.8751	34.8294	299.63	
18.838	68.4787	32.5708	325.58	
18.840	68.3237	39.6057	260.39	
18.842	67.2304	35.4455	288.20	
18.844	68.2713	38.2841	271.90	
18.846	68.2645	41.3838	243.73	
18.848	67.2355	34.8401	293.73	
18.850	68.6271	38.2855	275.11	
18.852	67.1200	36.9070	273.95	
18.854	67.9001	33.7281	309.84	
18.856	67.5919	38.2246	266.28	
18.858	67.8781	36.3424	285.94	
18.860	68.4319	32.1349	329.11	
18.862	68.2176	36.4308	288.22	
18.864	67.6204	32.3536	319.77	
18.866	68.1879	35.5553	295.89	
18.868	67.9004	36.1434	287.95	
18.870	67.2829	31.1569	327.56	
18.872	68.7487	36.4272	293.06	
18.874	67.3964	36.0672	284.07	
18.876	67.9619	35.3279	295.90	
18.878	67.5676	36.9711	277.42	
18.880	67.7618	33.7847	308.07	

18.882	68.5137	33.2528	319.72	
18.884	67.9933	37.5620	275.92	
18.886	68.0047	34.3043	305.57	
18.888	68.0339	37.0262	281.15	
18.890	67.8992	36.5133	284.58	
18.892	67.3522	30.7097	332.24	
18.894	68.8015	36.6679	291.36	
18.896	67.4608	36.5237	280.51	
18.898	67.9200	36.2342	287.30	
18.900	67.8162	38.4371	266.38	
18.902	67.5392	36.4319	282.05	
18.904	68.2958	35.5782	296.65	
18.906	67.8083	38.1390	269.02	
18.908	68.2028	34.7598	303.23	
18.910	67.7325	36.6259	282.05	
18.912	68.0249	40.2327	252.00	
18.914	67.2959	36.8647	275.92	
18.916	68.8343	40.6396	255.65	heating
18.918	67.6398	37.8885	269.76	heating
18.920	68.0007	36.2011	288.33	heating
18.922	68.0391	37.0208	281.25	heating
18.924	67.6817	34.6240	299.74	heating
18.926	68.0623	32.3091	324.18	heating
18.928	68.0992	37.3477	278.83	heating
18.930	68.7666	36.5423	292.18	heating
18.932	68.0334	37.0680	280.77	heating
18.934	68.3764	38.6992	269.09	heating
18.936	67.6738	34.9094	297.08	heating
18.938	69.4083	36.1924	301.17	heating
18.940	68.0521	32.6254	321.22	heating
18.942	68.5975	32.6871	325.60	heating
18.944	68.1901	32.8287	320.63	
18.946	68.4748	33.1811	320.01	
18.948	68.1707	31.0870	336.24	
18.950	68.5441	34.4078	309.52	
18.952	69.0710	33.7458	320.30	
18.954	68.3855	32.3765	326.50	
18.956	68.4307	30.1985	346.66	
18.958	68.2708	32.4211	325.05	
18.960	69.1599	34.7032	312.42	
18.962	68.2261	34.7026	303.96	
18.964	68.7974	35.8219	298.99	
18.966	67.8577	31.0586	333.66	
18.968	69.0601	32.4180	332.24	
18.970	67.9052	32.0285	325.30	
18.972	68.5837	31.9002	332.61	
18.974	68.8126	32.4600	329.61	
18.976	68.4207	32.6929	323.95	
18.978	68.5853	29.5515	353.93	
18.980	68.5177	34.5095	308.36	cooling
18.982	68.5631	32.9929	322.52	cooling
18.984	68.2633	33.8657	311.89	cooling
18.986	69.1111	39.4055	269.34	cooling
18.988	67.7763	34.3350	303.22	
18.990	69.2539	39.4265	270.45	
18.992	67.7363	39.5030	256.00	
18.994	68.1523	34.3219	306.74	
18.996	68.7419	36.7354	290.21	
18.998	67.8884	35.6845	292.00	
19.000	68.7160	33.3866	320.34	
19.002	68.5376	40.6674	252.70	
19.004	67.8967	35.4648	294.06	
19.006	68.3611	35.2328	300.38	
19.008	69.2851	41.8939	248.36	
19.010	68.1939	34.8623	302.22	
19.012	69.3848	41.5395	252.48	
19.014	67.7797	40.8192	244.45	
19.016	68.1946	32.6864	321.96	
19.018	68.9119	37.9027	281.17	
19.020	67.7567	36.0591	287.41	
19.022	68.8166	32.3677	330.49	
19.024	68.4597	38.0759	275.49	
19.026	67.6337	31.6519	326.25	
19.028	68.2591	29.8012	348.70	
19.030	69.2302	34.5868	314.12	
19.032	68.2034	28.7770	357.48	
19.034	68.9479	34.7362	310.20	
19.036	67.8476	34.8490	299.20	
19.038	68.0818	29.3382	351.29	
19.040	68.5681	33.5869	317.18	
19.042	67.7798	34.3161	303.42	
19.044	68.8105	30.4075	348.21	
19.046	68.3562	34.7179	305.00	
19.048	67.9142	31.2090	332.81	
19.050	68.1346	28.5695	358.74	
19.052	69.1708	33.0724	327.31	cooling
19.054	68.1473	27.7642	366.16	cooling
19.056	68.6367	31.7978	334.02	cooling

19.058	67.8992	34.5504	302.38	cooling
19.060	67.9176	35.1274	297.31	cooling
19.062	68.1637	37.5139	277.91	cooling
19.064	67.6480	39.3421	256.65	cooling
19.066	68.5885	38.6097	271.82	
19.068	67.7787	39.1620	259.47	
19.070	68.1380	36.6348	285.64	
19.072	67.5119	33.2430	310.72	
19.074	68.9002	37.2155	287.29	
19.076	68.1811	35.6991	294.52	
19.078	68.3984	37.9068	276.47	
19.080	67.9883	36.8871	282.00	
19.082	68.0147	37.4014	277.57	
19.084	68.4634	36.5069	289.75	
19.086	67.6749	35.2697	293.82	
19.088	68.7753	35.8485	298.55	
19.090	67.5614	35.9350	286.76	
19.092	68.5768	37.5992	280.88	
19.094	67.3523	35.2188	291.36	
19.096	68.5181	35.2462	301.68	
19.098	68.1532	33.8729	310.83	
19.100	68.2035	33.8290	311.68	
19.102	68.0397	29.1859	352.29	
19.104	68.1341	32.7661	320.69	
19.106	68.3691	32.7994	322.52	
19.108	67.8124	34.9194	298.25	
19.110	68.6422	39.2908	266.13	
19.112	67.3658	34.8251	295.05	
19.114	68.8049	34.9198	307.24	
19.116	67.5827	34.8845	296.48	
19.118	68.0059	31.0795	334.82	
19.120	68.2568	32.8648	320.90	
19.122	68.1605	35.7357	294.00	
19.124	67.9496	31.0876	334.23	
19.126	68.5732	37.3090	283.48	
19.128	67.6870	33.5407	309.61	
19.130	68.1111	32.9994	318.36	
19.132	68.6553	39.9984	259.84	
19.134	67.2508	34.3685	298.15	
19.136	68.8380	37.6748	282.56	
19.138	67.6927	41.3567	238.79	
19.140	67.8852	35.0788	297.46	
19.142	68.3814	38.7897	268.31	
19.144	67.8884	36.2687	286.70	
19.146	68.2161	28.0926	363.81	
19.148	68.6768	35.7965	298.13	
19.150	67.2646	32.4345	315.81	
19.152	68.2740	33.8462	312.16	
19.154	68.4074	42.0734	238.77	
19.156	67.2984	37.6067	269.22	
19.158	68.7424	41.4333	247.62	
19.160	67.5908	41.5363	236.24	
19.162	68.0255	32.6526	320.73	
19.164	68.4600	38.3062	273.41	
19.166	68.0198	37.5235	276.51	
19.168	67.8907	29.6474	346.76	
19.170	68.9428	35.2834	305.20	
19.172	67.5081	29.3628	345.87	
19.174	68.4807	29.2777	355.46	
19.176	68.0702	32.3542	323.84	
19.178	67.7202	29.5526	346.07	
19.180	68.6877	35.0285	305.19	
19.182	67.5937	37.1637	275.91	
19.184	68.1580	34.2249	307.68	
19.186	68.3016	39.0986	264.79	
19.188	67.7567	36.2589	285.59	
19.190	67.8154	30.6283	337.18	
19.192	68.6437	31.7132	334.85	
19.194	68.0255	25.6923	383.84	
19.196	68.4332	29.1920	355.81	
19.198	67.8451	31.3821	330.61	
19.200	67.7950	27.9063	361.68	
19.202	68.5995	30.9206	341.64	cooling
19.204	67.3356	31.0615	328.90	cooling
19.206	68.1297	33.4621	314.34	cooling
19.208	67.7661	37.8461	271.29	cooling
19.210	67.5313	36.7371	279.22	cooling
19.212	67.4011	35.9139	285.50	cooling
19.214	68.1453	38.8662	265.48	
19.216	68.0451	35.7484	292.84	
19.218	67.9236	39.8897	254.19	
19.220	67.5320	37.8524	269.11	
19.222	67.4381	31.9569	321.71	
19.224	68.4122	33.5793	315.83	
19.226	66.8879	32.2009	314.51	
19.228	68.3361	38.6399	269.26	
19.230	67.1922	40.9273	238.15	
19.232	67.5784	38.8469	260.51	

19.234	67.3639	38.1376	265.00
19.236	67.8669	37.6705	273.80
19.238	68.5494	33.2639	319.94
19.240	67.7195	37.3269	275.57
19.242	67.6528	35.1060	295.11
19.244	67.6382	36.1157	285.82
19.246	68.3615	38.6004	269.85
19.248	66.7812	34.9417	288.69
19.250	68.6490	37.3351	283.93
19.252	66.8451	33.1828	305.22
19.254	67.9801	31.2594	332.95
19.256	67.1753	32.5422	314.02
19.258	68.1545	34.6840	303.48
19.260	68.3202	33.2008	318.43
19.262	67.7577	36.7807	280.87
19.264	67.5985	29.1041	349.03
19.266	67.7318	29.5419	346.27
19.268	68.3676	30.8362	340.30
19.270	67.0146	29.4526	340.58
19.272	68.7009	35.9916	296.58
19.274	66.6336	32.6428	308.20
19.276	68.2905	34.3623	307.63
19.278	67.1657	35.8854	283.62
19.280	67.6619	34.1372	303.97
19.282	67.8686	32.4514	321.13
19.284	67.4471	33.3545	309.12
19.286	67.5984	28.3498	355.87
19.288	67.7984	34.7795	299.39
19.290	67.7825	35.2319	295.14
19.292	67.2747	34.1823	300.05
19.294	68.4032	39.4476	262.55
19.296	66.7313	34.1430	295.48
19.298	68.4653	36.2185	292.39
19.300	67.2650	37.6097	268.89
19.302	67.5729	37.3532	274.01
19.304	67.7750	39.9897	251.93
19.306	67.6050	42.0138	232.04
19.308	67.9550	37.5078	276.07
19.310	67.7609	39.9919	251.79
19.312	67.6264	35.0273	295.58
19.314	67.4639	32.1165	320.50
19.316	68.1555	37.3672	279.16
19.318	67.1138	34.9907	291.26
19.320	68.2451	36.4919	287.91
19.322	67.4816	37.2691	273.94
19.324	67.3814	35.6078	288.10
19.326	67.6675	36.1787	285.51
19.328	67.7813	36.9910	279.18
19.330	67.9330	34.2887	305.06
19.332	67.5680	35.6731	289.20
19.334	67.6353	36.7732	279.83
19.336	67.5290	36.4293	281.99
19.338	67.5909	35.6573	289.55
19.340	67.3336	31.8555	321.68
19.342	67.8133	28.8605	353.19
19.344	67.6937	28.6996	353.56
19.346	67.4859	30.1361	338.66
19.348	67.5577	32.4800	318.05
19.350	67.7712	34.0421	305.83
19.352	67.9298	35.0797	297.86
19.354	67.5119	34.5986	298.43
19.356	67.7251	33.5985	309.43
19.358	67.6303	34.0405	304.56
19.360	67.4210	31.2301	328.15
19.362	67.5312	31.7358	324.56
19.364	67.9734	36.2031	288.07
19.366	67.5813	35.6922	289.14
19.368	67.7772	36.6254	282.46
19.370	67.4396	34.4741	298.90
19.372	67.8494	31.2868	331.52
19.374	68.1195	32.1979	325.71
19.376	67.3589	32.7451	313.85
19.378	67.8960	33.1240	315.28
19.380	67.5318	35.7492	288.18
19.382	67.6325	37.0550	277.25
19.384	67.3368	38.8953	257.88
19.386	68.2667	40.9861	247.36
19.388	67.6215	33.5338	309.08
19.390	68.0809	35.1900	298.23
19.392	67.5022	35.4878	290.28
19.394	67.7584	35.0885	296.22
19.396	68.1054	37.8317	274.50
19.398	66.9953	35.1624	288.63
19.400	68.2374	31.8283	330.13
19.402	67.5910	33.9634	304.91
19.404	67.5783	32.4400	318.60
19.406	67.2668	33.2086	308.81
19.408	68.4711	38.4097	272.57

19.410	67.2619	31.4183	325.00
19.412	68.2402	36.8122	284.96
19.414	67.3180	38.1180	264.76
19.416	67.4280	34.6959	296.79
19.418	67.7237	33.7412	308.12
19.420	67.0004	30.9267	327.08
19.422	68.6270	27.5928	372.06
19.424	67.7319	34.3086	303.05
19.426	67.7142	33.6310	309.04
19.428	67.1865	29.8162	338.84
19.430	68.7944	34.4359	311.53
19.432	66.9723	28.7449	346.61
19.434	68.3184	32.8279	321.80
19.436	67.4204	35.1797	292.33
19.438	67.4060	34.4002	299.27
19.440	67.3632	34.4769	298.18
19.442	67.9095	37.4148	276.50
19.444	68.5090	33.8276	314.46
19.446	67.8123	37.4671	275.14
19.448	67.8531	36.3865	285.31
19.450	67.2827	32.5053	315.33
19.452	68.7903	36.6565	291.36
19.454	67.0533	35.8931	282.53
19.456	68.1531	35.3721	297.23
19.458	67.6582	37.5408	273.08
19.460	67.5989	38.3170	265.50
19.462	67.3296	35.2722	290.67
19.464	68.3743	41.7705	241.22
19.466	67.9483	35.5182	294.05
19.468	67.7855	34.5560	301.30
19.470	68.1979	38.4042	270.14
19.472	67.0021	32.6394	311.57
19.474	68.2328	35.3766	297.91
19.476	67.2712	37.9514	265.85
19.478	67.8510	34.6241	301.27
19.480	68.1530	37.8562	274.71
19.482	67.1288	35.4912	286.86
19.484	67.7638	29.8080	344.15
19.486	68.0354	34.9258	300.21
19.488	67.8429	34.3147	304.01
19.490	67.4843	32.2535	319.44
19.492	68.2316	35.6315	295.59
19.494	67.3687	30.7255	332.25
19.496	68.0264	31.6903	329.46
19.498	67.5356	34.1472	302.74
19.500	67.5537	34.2426	302.04
19.502	68.2729	36.1237	291.50
19.504	67.1930	32.9386	310.59
19.506	68.0250	30.3706	341.42
19.508	67.5050	31.8052	323.70
19.510	67.8392	32.0210	324.77
19.512	67.6096	32.3613	319.60
19.514	68.0497	34.0615	308.18
19.516	67.7620	32.7206	317.73
19.518	67.8010	35.2072	295.53
19.520	67.6337	33.8389	306.42
19.522	67.7435	34.1858	304.27
19.524	68.0465	34.0134	308.58
19.526	67.6374	35.2661	293.52
19.528	68.0870	39.9854	254.80
19.530	67.3270	39.0937	255.99
19.532	67.7286	37.5821	273.34
19.534	67.9897	36.2750	287.56
19.536	68.0276	32.8705	318.77
19.538	68.1894	34.0655	309.41
19.540	67.6380	37.2465	275.56
19.542	67.7350	35.6228	291.17
19.544	67.8308	35.8996	289.53
19.546	67.7712	34.5668	301.07
19.548	67.7946	34.2659	304.01
19.550	67.9087	38.7040	264.80
19.552	67.4709	38.8685	259.34
19.554	67.5525	39.0027	258.86
19.556	67.8423	39.4564	257.38
19.558	68.0582	36.0320	290.39
19.560	68.1870	37.7222	276.23
19.562	67.6690	36.2566	284.82
19.564	67.5260	32.3651	318.81
19.566	68.1605	32.9786	319.00
19.568	67.6536	30.7371	334.73
19.570	68.2178	33.2227	317.31
19.572	67.6338	36.3298	283.84
19.574	67.6325	36.9023	278.64
19.576	67.6597	38.7371	262.25
19.578	67.6031	39.8934	251.25
19.580	67.8632	36.1984	287.11
19.582	67.8980	37.6172	274.56
19.584	68.0243	37.2462	279.07

19.586	67.4387	34.8211	295.75	heating
19.588	68.3627	37.8317	276.83	
19.590	67.5159	35.2709	292.37	heating
19.592	68.5583	35.6423	298.45	heating
19.594	68.0469	35.0797	298.92	heating
19.596	67.7651	32.5894	318.94	
19.598	68.1087	32.8500	319.70	
19.600	67.6858	35.8582	288.59	
19.602	68.0304	35.4501	295.41	
19.604	67.8923	40.1145	251.86	
19.606	68.2787	39.7866	258.34	
19.608	67.6863	36.0573	286.79	heating
19.610	68.4655	37.4632	281.10	heating
19.612	67.4961	31.6320	325.19	heating
19.614	68.7059	31.6892	335.64	
19.616	68.4622	32.5579	325.55	
19.618	67.9772	32.3523	323.02	
19.620	68.4255	34.5734	306.94	
19.622	67.7683	35.4814	292.75	
19.624	68.0474	32.6881	320.61	
19.626	68.1234	34.8613	301.59	
19.628	68.3285	33.5873	315.00	
19.630	68.3049	32.5064	324.59	
19.632	68.2754	36.6286	286.95	
19.634	67.7356	33.5840	309.66	
19.636	68.4533	34.2214	310.39	
19.638	68.6250	35.4590	300.72	
19.640	67.9822	32.5364	321.39	
19.642	68.5765	34.5119	308.87	
19.644	67.7826	34.3331	303.29	
19.646	68.0421	33.3149	314.88	
19.648	68.1972	35.3153	298.15	
19.650	68.1550	35.4720	296.34	
19.652	68.2689	36.1608	291.13	
19.654	67.8673	37.0347	279.56	
19.656	67.9327	34.1070	306.70	
19.658	68.1683	33.5164	314.19	
19.660	68.5706	33.3099	319.71	
19.662	68.0992	31.6483	330.51	
19.664	68.4290	34.5966	306.76	
19.666	67.9092	35.3420	295.29	
19.668	68.2677	37.1874	281.81	
19.670	68.1755	35.3208	297.90	
19.672	68.2408	36.3654	289.02	
19.674	68.4304	36.8619	286.24	
19.676	67.8050	35.2899	294.82	
19.678	68.3623	36.3163	290.57	
19.680	67.6587	35.6236	290.47	
19.682	68.5393	36.5688	289.88	
19.684	68.2029	36.8828	283.98	
19.686	68.0300	34.8318	301.01	
19.688	67.9527	32.6517	320.08	
19.690	68.1009	34.3462	306.06	
19.692	68.1452	34.3178	306.72	
19.694	68.2645	39.9212	256.99	
19.696	68.1496	41.3234	243.24	
19.698	67.9536	38.8784	263.63	
19.700	68.4366	41.5932	243.39	
19.702	67.4475	38.1757	265.41	
19.704	68.3263	36.6917	286.84	
19.706	68.3677	37.6557	278.47	
19.708	68.0144	36.4226	286.45	heating
19.710	68.1742	36.8992	283.58	heating
19.712	68.0494	36.7578	283.73	heating
19.714	68.2493	31.9019	329.57	heating
19.716	68.4326	33.1885	319.56	
19.718	68.0068	32.4052	322.80	
19.720	68.3062	34.6680	305.00	
19.722	68.4272	38.5491	270.91	
19.724	67.7854	36.2481	285.95	
19.726	68.4098	34.8491	304.30	
19.728	68.5041	35.1348	302.56	
19.730	68.5245	32.8973	323.04	
19.732	68.4538	34.0605	311.85	
19.734	68.2071	34.4646	305.95	
19.736	68.3243	31.8962	330.30	
19.738	68.3656	31.3976	335.19	
19.740	68.0868	29.6062	348.91	
19.742	68.4702	31.1541	338.35	
19.744	68.3247	35.1632	300.68	
19.746	68.0952	35.9230	291.71	
19.748	68.1696	36.2663	289.27	
19.750	68.5155	36.9809	285.93	
19.752	68.5907	34.1705	312.09	
19.754	68.6030	37.1785	284.93	
19.756	68.3035	38.9293	266.34	
19.758	68.1836	37.7790	275.68	
19.760	68.5803	35.9367	295.98	
19.762	68.1148	34.7033	302.95	
19.764	68.4926	36.5099	289.99	
19.766	67.9782	38.5835	266.53	
19.768	68.2376	39.6723	259.01	
19.770	67.8508	38.2063	268.79	
19.772	68.5676	39.3820	264.63	
19.774	68.1739	38.7208	267.06	
19.776	68.5213	40.4554	254.48	
19.778	68.2619	39.1219	264.22	heating
19.780	68.2804	36.4256	288.83	heating
19.782	68.4300	34.4456	308.14	heating
19.784	68.6437	36.7463	289.22	heating
19.786	68.8177	38.9080	271.20	heating
19.788	68.3561	38.7737	268.23	heating
19.790	68.5407	35.2900	301.49	heating
19.792	68.6459	33.4927	318.74	heating
19.794	68.8526	33.2250	323.04	heating
19.796	68.7620	35.7285	299.52	
19.798	68.7462	38.3285	275.80	
19.800	68.5919	35.9095	296.34	
19.802	68.6100	35.5611	299.66	
19.804	68.5908	34.2671	311.22	
19.806	68.8304	32.6671	327.90	
19.808	69.1933	36.1166	299.91	
19.810	68.4786	38.0114	276.25	
19.812	68.4825	36.3353	291.48	
19.814	68.8753	36.1839	296.42	
19.816	68.6970	33.7374	316.98	
19.818	68.7341	33.9208	315.66	
19.820	68.6912	36.6157	290.83	
19.822	68.6195	35.4872	300.42	
19.824	68.5597	35.1215	303.19	
19.826	68.4155	35.2825	300.42	
19.828	68.9180	34.1905	314.88	
19.830	68.9007	36.5498	293.33	
19.832	68.6197	36.3285	292.79	
19.834	68.3084	35.3971	298.41	
19.836	69.0466	37.1121	289.55	
19.838	68.5041	35.6759	297.66	
19.840	68.8193	36.8891	289.52	
19.842	68.4095	36.9009	285.69	
19.844	68.6752	36.7372	289.59	
19.846	68.5710	35.2326	302.28	
19.848	68.4452	34.9867	303.37	
19.850	68.7982	34.6781	309.37	
19.852	68.5226	33.8588	314.30	
19.854	69.0478	32.9420	327.38	
19.856	68.4069	33.8841	313.02	
19.858	68.8310	36.2612	295.32	
19.860	68.5318	39.1674	266.25	
19.862	69.0917	41.5751	249.50	
19.864	68.4759	37.1046	284.45	
19.866	68.9230	37.8689	281.57	
19.868	68.6132	36.6280	290.01	
19.870	68.6049	35.3822	301.24	
19.872	68.8602	35.9456	298.44	
19.874	68.3658	36.3741	290.07	
19.876	69.1164	36.6641	294.25	
19.878	68.6619	37.7142	280.61	
19.880	68.7662	35.8677	298.30	
19.882	68.3474	33.2136	318.56	
19.884	69.0903	34.8287	310.66	
19.886	68.6687	34.0756	313.66	
19.888	68.8099	36.3780	294.06	
19.890	68.5397	36.9756	286.20	
19.892	68.5590	36.0318	294.93	
19.894	68.5965	33.4035	319.10	
19.896	68.5320	32.1350	330.02	
19.898	68.7831	31.2659	340.17	
19.900	68.6713	32.5818	327.23	
19.902	68.6980	34.0626	314.04	
19.904	68.2879	31.7782	331.04	
19.906	68.7657	32.6130	327.80	
19.908	68.7859	34.4767	311.09	
19.910	68.7218	33.4554	319.77	
19.912	68.8798	34.8312	308.72	
19.914	68.3822	34.6467	305.88	
19.916	68.5563	33.2659	319.98	
19.918	68.9180	35.1260	306.40	
19.920	68.5735	35.1081	303.44	
19.922	68.9029	38.9792	271.32	
19.924	68.5004	41.3958	245.76	
19.926	68.4366	37.7343	278.38	
19.928	68.5851	36.5075	290.85	
19.930	68.6820	37.0512	286.80	
19.932	68.8428	38.0047	279.61	
19.934	68.8847	41.5133	248.18	
19.936	68.2075	39.4996	260.30	

19.938	68.6874	39.4029	265.53	
19.940	68.8520	39.6281	264.98	
19.942	68.6047	35.8848	296.68	
19.944	68.9617	34.5989	311.57	
19.946	68.3310	34.5558	306.25	
19.948	68.5546	35.1116	303.23	
19.950	68.5023	38.8384	268.97	
19.952	68.6900	39.7018	262.84	
19.954	69.0132	40.8964	254.94	
19.956	68.8362	41.9603	243.69	
19.958	68.4481	34.2006	310.53	heating
19.960	68.8587	31.8294	335.75	heating
19.962	69.0103	31.8831	336.64	
19.964	68.5611	30.6437	343.80	
19.966	69.1516	33.6461	321.93	
19.968	68.3244	33.0485	319.85	
19.970	68.7862	34.8410	307.79	
19.972	68.4787	37.3872	281.91	
19.974	68.7702	36.7854	290.01	
19.976	68.9327	36.3948	295.03	
19.978	68.8310	38.7905	272.38	
19.980	68.7091	36.4375	292.61	
19.982	68.6557	34.3900	310.69	
19.984	68.5804	32.3125	328.85	
19.986	68.6711	34.4076	310.67	
19.988	68.9169	37.4972	284.89	
19.990	68.5213	35.8693	296.06	
19.992	68.5583	35.4447	300.25	
19.994	68.5936	34.8229	306.20	
19.996	68.7031	33.6574	317.76	
19.998	68.9017	34.8564	308.69	
20.000	68.5285	37.6501	279.98	

DESCRIPTION: Tabulated form of quantum chemical data on optimized ion at m/z 103 at M062X/SDD level of theory and using Douglas-Kroll-Hess approach in the ground state. There are used atomic coordinates as imput parameters of the molecular ions as listed in **Table S6.**

Total Energy
Total Energy (Hartree) vs Optimization Step Number
X Y

1.0000000000	-523.2429975390
2.0000000000	-523.2871538560
3.0000000000	-523.3157098590
4.0000000000	-523.3259480850
5.0000000000	-523.3260969200
6.0000000000	-523.3354537740
7.0000000000	-523.3372614590
8.0000000000	-523.3379155120
9.0000000000	-523.3380737190
10.0000000000	-523.3381655030
11.0000000000	-523.3382223120
12.0000000000	-523.3382207240
13.0000000000	-523.3382186060
14.0000000000	-523.3382178250
15.0000000000	-523.3382177770
16.0000000000	-523.3382189820
17.0000000000	-523.3382195070
18.0000000000	-523.3382200790
19.0000000000	-523.3382201560
20.0000000000	-523.3382204100
21.0000000000	-523.3382208170
22.0000000000	-523.3382224610
23.0000000000	-523.3382274040
24.0000000000	-523.3382200940
25.0000000000	-523.3382264280
26.0000000000	-523.3382348140
27.0000000000	-523.3382355050
28.0000000000	-523.3382235840
29.0000000000	-523.3382306280
30.0000000000	-523.3382362370
31.0000000000	-523.3382277860
32.0000000000	-523.3382303270
33.0000000000	-523.3382302740
34.0000000000	-523.3382297300
35.0000000000	-523.3382299520
36.0000000000	-523.3382402280
37.0000000000	-523.3382347370
38.0000000000	-523.3382348130
39.0000000000	-523.3382310760
40.0000000000	-523.3382358360
41.0000000000	-523.3382320870
42.0000000000	-523.3382364150
43.0000000000	-523.3382294650
44.0000000000	-523.3382298540
45.0000000000	-523.3382300090
46.0000000000	-523.3382358280
47.0000000000	-523.3382194140
48.0000000000	-523.3382279660
49.0000000000	-523.3382325650
50.0000000000	-523.3382219140
51.0000000000	-523.3382167820
52.0000000000	-523.3382222950
53.0000000000	-523.3382304790
54.0000000000	-523.3381808550
55.0000000000	-523.3382183830
56.0000000000	-523.3382048500
57.0000000000	-523.3382282220
58.0000000000	-523.3382427140
59.0000000000	-523.3382408320
60.0000000000	-523.3382116960
61.0000000000	-523.3382250560
62.0000000000	-523.3382174320
63.0000000000	-523.3382177150
64.0000000000	-523.3382285340
65.0000000000	-523.3382175180
66.0000000000	-523.3382178120
67.0000000000	-523.3382300340
68.0000000000	-523.3382211160
69.0000000000	-523.3382285160
70.0000000000	-523.3382338530
71.0000000000	-523.3382332410
72.0000000000	-523.3382219910
73.0000000000	-523.3382314150
74.0000000000	-523.3382196210
75.0000000000	-523.3382313330
76.0000000000	-523.3382294040
77.0000000000	-523.3382328590
78.0000000000	-523.3382223790
79.0000000000	-523.3382237020
80.0000000000	-523.3382202100
81.0000000000	-523.3382252980
82.0000000000	-523.3382436690
83.0000000000	-523.3362392420
84.0000000000	-523.3382436580
85.0000000000	-523.3382470750
86.0000000000	-523.3382145780

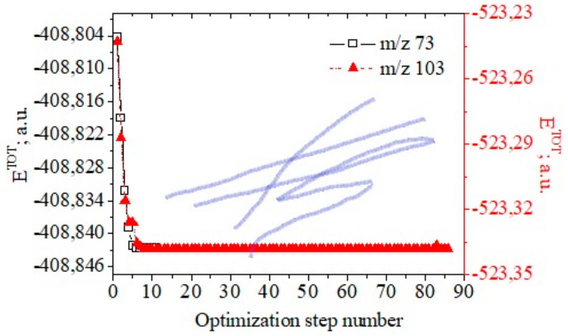

Illustration of tabulated data on file entitled UNTITLED-qq1.opj (see description to tabulated data shown above.)

FILE: UNTITLED4.opj

DESCRIPTION: Tabulated form of quantum chemical data on Born-Oppenheimer molecular dynamics of ions at m/z 121 and 131 of aucubin at M062X/SDD level of theory. The atomic coordinates of the ground state optimized geometry are used as imput parameters of computing Born-Oppenheimer molecular dynamics the ion as listed in **Table S6.**

Potential Energy
Potential Energy (Hartree) vs Time in Trajectory (femptosec)

X	Y	X	Y
	m/z 121		m/z 131
	Structure $121_{\_b}$		Structure $131_{\_b}$
0	-419,61051	0	-421,8881
0,025	-419,61041	0,025	-421,88802
0,05	-419,61009	0,05	-421,88777
0,075	-419,60957	0,075	-421,88736
0,1	-419,60884	0,1	-421,88679
0,125	-419,60791	0,125	-421,88606
0,15	-419,60678	0,15	-421,88518
0,175	-419,60546	0,175	-421,88413
0,2	-419,60394	0,2	-421,88294
0,225	-419,60222	0,225	-421,88159
0,25	-419,60032	0,25	-421,8801
0,275	-419,59823	0,275	-421,87845
0,3	-419,59596	0,3	-421,87667
0,325	-419,59351	0,325	-421,87474
0,35	-419,59088	0,35	-421,87267
0,375	-419,58808	0,375	-421,87047
0,4	-419,5851	0,4	-421,86814
0,425	-419,58196	0,425	-421,86568
0,45	-419,57864	0,45	-421,86309
0,475	-419,57517	0,475	-421,86038
0,5	-419,57153	0,5	-421,85755
0,525	-419,56774	0,525	-421,85461
0,55	-419,56379	0,55	-421,85155
0,575	-419,55969	0,575	-421,84839
0,6	-419,55545	0,6	-421,84512
0,625	-419,55106	0,625	-421,84176
0,65	-419,54654	0,65	-421,8383
0,675	-419,54188	0,675	-421,83474
0,7	-419,53708	0,7	-421,8311
0,725	-419,53216		
0,75	-419,52712		
0,775	-419,52195		
0,8	-419,51667		
0,825	-419,51128		
0,85	-419,50577		
0,875	-419,50016		

0,9	-419,49445
0,925	-419,48865
0,95	-419,48275
0,975	-419,47676
1	-419,47068
1,025	-419,46453
1,05	-419,45831

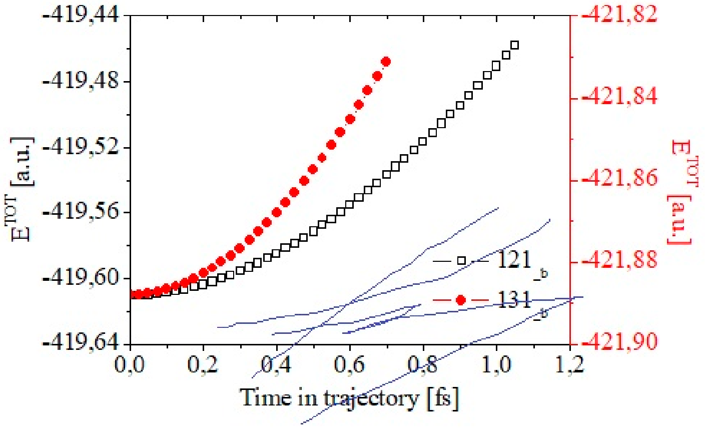

(Consider detail on figure caption of **Figure S20**.)

FILE: UNTITLED-GCMS.opj

DESCRIPTION: Tabulated form of experimental mass spectrometric relative intensity parameters (%) of analytes looking at mass spectrometric parent and product ions at m/z 45, 73, 103, 117, 129, 147, 149, 189, 191, 205, 217, 244, 273, 292, 305, 308, 319, 331, 333, 359, 423, 429, 359, 361, 437, 449, and 451 of carbohydrates CB (A) (CBA, column 8), CB (B) (CBB, column 15), Sucrose, 8TMS derivative (Sucrose8TMS, column 10), glucoronic acid, 6TMS derivative (Glucoronicacid6TMS, column 2) D-lactose, (isomer 1), 8TMS derivative (Maltose8TMS, column 6), D-Gluconic acid, 6TMS derivative (GluconicacidNIST, column 22), D-Glucose, 5TMS derivative (DglucoseSiNIST, column 24), α-2-deoxy-D-glucose (2DG, 2DGNIST, column 28), Gluconolactone (3R,4S,5R,6R)-, 4TMS derivative (Gluconicacid15lac, column 4), 4-deoxy-D-glucose (4DG, DGlucref, column 20),

There are listed theoretical intensity data on equation (6) calculated for any of the ions (ITheor, column 11), as well.

TMS - denotes trimethylsilyl.

There are used mass spectrometric experimental data of analytes downloaded from NIST database as JCAMP-DX format free of charge. The links are provided below.

1	2	3	4	5	6	7	8	9	10	11	12	13	14	15	16	17	18	19	20	21	22	23	24	25	26	27	28
	Glucoronicacid6TMS		Gluconicacid15lac		Maltose8TMS	CBA	CBA		Sucrose8TMS	Itheor			CBB	CBB					DGlucref		GluconicacidNIST		DGlucoseSiNIST				2DGNIST
						45	2,006	--	--	1,11E-07	45	2,006	45	2,7168	1,11E-07	45	4,26	45	8,3974	45	2,8	45	4,27	45	6,14	45	6,97
73,0468	642,97	73,0468	100	73,0468	45,5374	73	100	73	60,67	4,72E-12	73	100	73	100	4,72E-12	73	100	73	100	73	100	73	99,75	73	100	73	100
--	--	--	--	--	--	--	--	--	--	--	--	--	--	--	--	--	--	--	--	--	--	--	--	--	--	--	--
--	--	--	--	--	--	103	30,5021	103	13,99	1,91E-07	103	30,5021	103	15,8377	1,91E-07	103	18,77	103	8,0578	103	14,7	103	6,85	103	2,01	103	13,69
--	--	--	--	--	--	117	5,6491	117	2,86	--	117	5,6491	117	5,1248	--	117	4,206	117	16,3626	117	6,5	117	4,75	117	1,4	117	9,35
129,073	180,78	129,0729	47,945	129,0366	11,6699	129	3,0564	129	9,91	--	129	3,0564	129	11,5155	--	--	--	129	12,5343	129	3,9	129	6,47	129	2,56	129	7,86
--	--	--	--	--	--	--	--	--	--	--	--	--	--	--	--	--	--	--	--	--	--	--	--	--	--	--	--
--	--	--	--	--	--	--	--	--	--	--	--	--	--	--	--	133	4,785	133	5,6498	--	--	--	--	--	--	--	--
147,0655	268,8	--	--	--	--	147	32,7556	147	15,86	3,22E-08	147	32,7556	147	23,5868	3,22E-08	147	38,96	147	28,4339	147	32,2	147	23,24	147	24,27	147	23,89
149,0447	706	149,0447	80,9177	149,0448	35,8742	--	--	--	--	--	--	--	--	--	--	--	--	--	--	--	--	--	--	--	--	--	--
--	--	--	--	--	--	--	--	169	9,07	--	--	--	169	8,3974	--	--	--	--	--	--	--	--	--	--	--	--	--
--	--	--	--	--	--	--	--	--	--	--	--	--	--	--	--	189	7,71	--	--	189	8,9	--	--	--	--	--	--
--	--	--	--	--	--	--	--	191	5,26	--	--	--	191	5,465	--	--	--	191	18,2458	191	4	191	46,29	191	9,95	191	11,32
--	--	--	--	204,0995	71,9339	--	--	--	--	--	--	--	--	--	1,33E-07	--	--	204	59,1832	--	--	204	100	204	34,39	204	50,19
--	--	--	--	--	--	205	13,584	205	3,69	1,33E-07	205	13,584	205	5,31	1,33E-07	205	28,06	--	--	205	25,5	--	--	--	--	--	--
217,1073	409,04	217,1073	45,01	217,1047	39,1468	217	33,28	217	30,04	--	217	33,28	217	26,89	--	217	22,28	217	9,7863	217	17,5	217	18,27	217	32,94	217	22,8
--	--	243,0866	15,31	243,123	21,1787	--	--	243	6,73	--	--	--	243	10,1263	--	245	1,327	244	3,7947	--	--	243	1,73	--	--	243	0,99

--	--	--	--	271,118	18,7707	--	--	271	13,7	--	--	--	271	10,9907	--	--	--	273	1,669	--	--	--	--	--	--	272	7,61
--	--	--	--	--	--	--	--	--	--	--	--	--	--	--	--	--	--	--	--	277	7	--	--	--	--	--	--
292,134	689,82	--	--	--	--	--	--	--	--	--	--	--	--	--	--	--	--	--	--	292	29,7	--	--	--	--	--	--
305,1417	409,28	305,1417	8,92	--	--	--	--	--	--	--	--	--	--	--	--	--	--	--	--	305	14,3	305	2,08	305	10,62	305	1,98
--	--	--	--	--	--	--	--	--	--	--	--	--	308	15,992	--	305	16,51	308	4,6005	--	--	--	--	--	--	--	--
319,1573	218,22	319,1573	42,994	319,1573	14,2673	319	5,6497	319	6,26	--	319	5,6497	320	4,446	--	319	20,4995	--	--	319	21,9	--	--	319	0,57	--	--
333,1365	999	333,1365	6,85	331,1573	7,719	--	--	331	2,96	--	--	--	331	3,5812	--	333	40,8756	333	0,531	333	33,1	--	--	333	1,32	--	--
--	--	--	--	--	--	--	--	--	--	--	--	--	--	--	--	--	--	349	0,66686	--	--	--	--	--	--	--	--
--	--	--	--	--	--	--	--	--	--	--	--	--	--	--	1,24 E-08	359	6,6538	--	--	359	6	--	--	359	0,99	--	--
--	--	361,3056	3,056	361,1678	100,954	--	--	361	100	1,24 E-08	--	--	361	45,68	1,24 E-08	--	--	--	--	--	--	--	--	--	--	--	--
--	--					--	--	--	--	--	--	--	--	--	--	--	--	--	--	423	10	--	--	--	--	--	--
--	--					--	--	--	--	--	--	--	--	--	--	--	--	--	--	435	5,5	--	--	--	--	--	--
--	--					--	--	437	12,6	--	--	--	--	--	--	--	--	--	--	--	--	--	--	449	2,37	--	--
--	--					857	0,463	451	10,35	--	857	0,463	--	--	--	--	--	--	--	--	--	--	--	--	--	--	--
						--	--				--	--	--	--		--	--	--	--			--	--	--	--	--	--
						--	--				--	--	--	--		--	--	--	--			--	--	--	--	--	--

Sucrose, 8TMS derivative [https://webbook.nist.gov/cgi/inchi?ID=C19159252&Mask=200];
D-Gluconic acid, 6TMS derivative [https://webbook.nist.gov/cgi/cbook.cgi?ID=C34290523&Units=SI&Mask=3FFF]
D-Lactose, (isomer 1), 8TMS derivative [https://webbook.nist.gov/cgi/cbook.cgi?ID=U380082]
D-Gluconic acid, 6TMS derivative [https://webbook.nist.gov/cgi/inchi?ID=C34290523]
D-Glucose, 5TMS derivative [https://webbook.nist.gov/cgi/inchi?ID=C6736976&Mask=200]
2-deoxy-D-glucose [https://webbook.nist.gov/cgi/inchi?ID=U79295&Mask=200#]
Gluconolactone (3R,4S,5R,6R)-, 4TMS derivative [https://webbook.nist.gov/cgi/inchi?ID=C55515292]

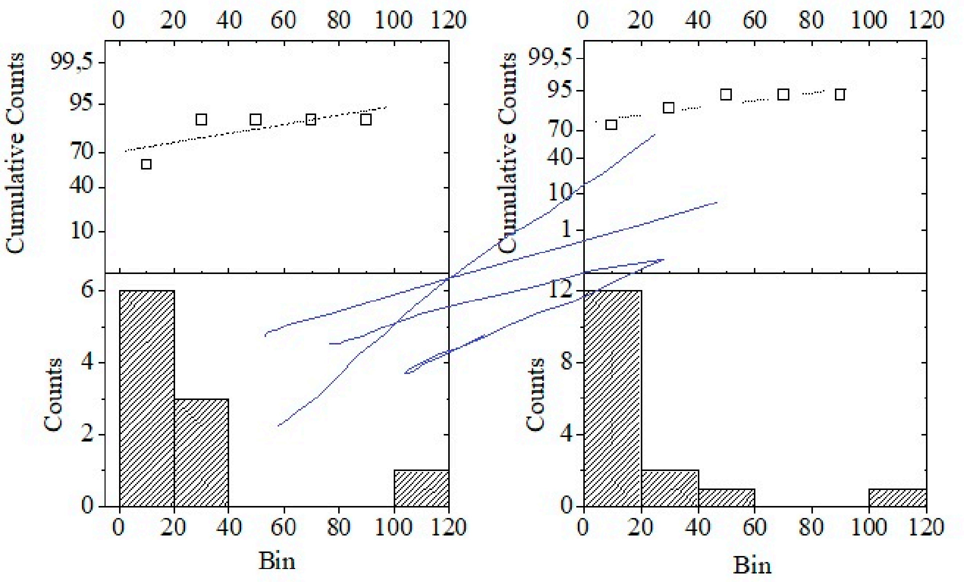

(Consider, figure caption of **Figure S11**.)

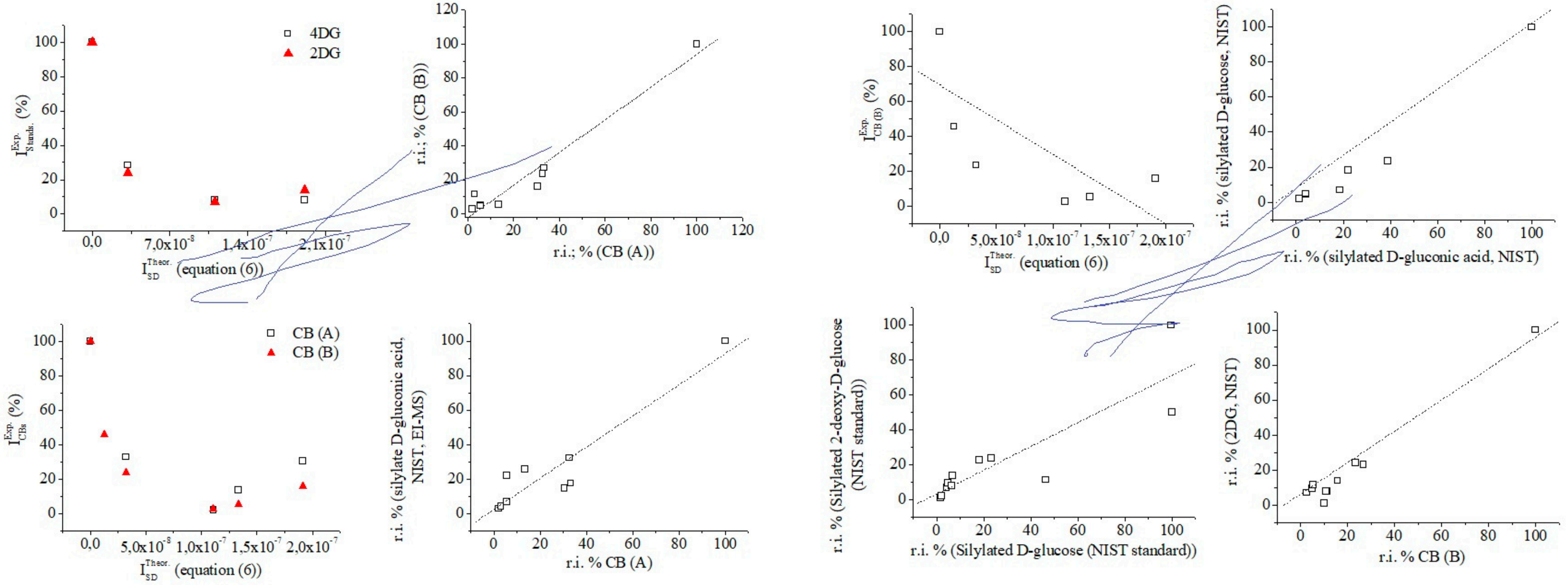

Data on experimental and theoretical mass spectrometric relative intensities (%) and theoretical mass spectrometric intensity ($I^{Theor}{}_{SD}$) according to equation (6) of carbohydrates given in tabulated for relating to file entitled UNTITLED-GCMS.opj (see above)

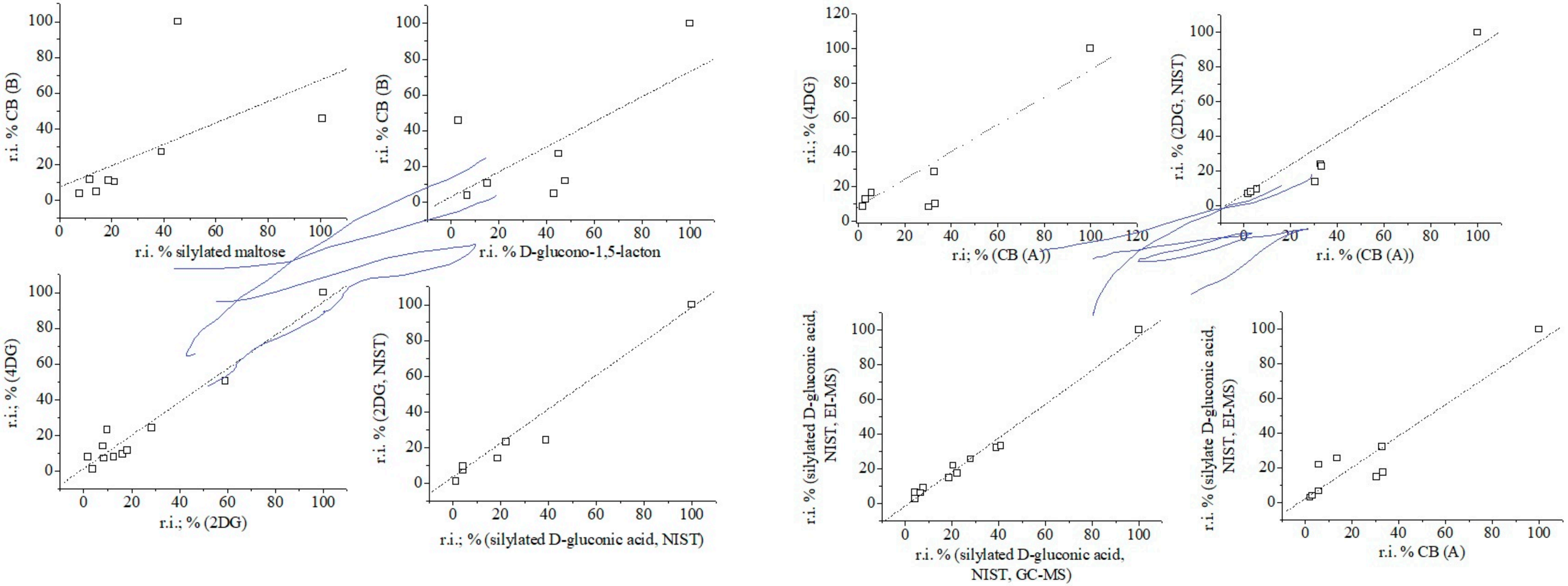

Data on experimental mass spectrometric relative intensities (%)of carbohydrates given in tabulated for relating to file entitled UNTITLED-GCMS.opj (see above)

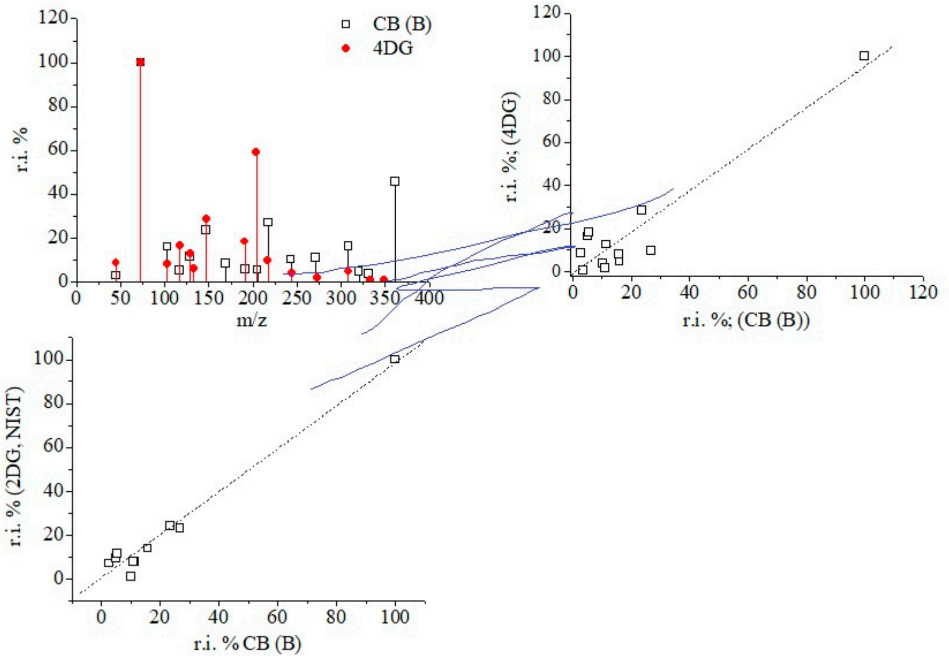

Data on experimental mass spectrometric relative intensities (%)of carbohydrates given in tabulated for relating to file entitled UNTITLED-GCMS.opj (see above)

FILE: UNTITLED-chromatogr2.opj

DESCRIPTION: Chromatographic data on control sample (column ‘control’) and sample treated with magnetic poulse energy for t=72h and ω=1-10 Hz (column sample-MPT); RT denotes retention time [mins]; Intensity [arb.units]]

Control		Sample-MPT	
RT	Intensity	RT	Intensity
7,14	18,89404	7,39	47,73338
7,23424	19,7807	7,47661	53,8724
7,32847	20,57333	7,56322	60,41863

7,42271	21,25753	7,64983	67,10131
7,51695	21,83448	7,73644	73,58953
7,61119	22,33347	7,82305	79,51697
7,70542	22,82487	7,90966	84,51492
7,79966	23,42154	7,99627	88,24978
7,8939	24,25033	8,08288	90,45974
7,98814	25,38259	8,16949	90,98517
8,08237	26,74284	8,2561	89,78758
8,17661	28,05376	8,34271	86,95437
8,27085	28,88481	8,42932	82,68823
8,36508	28,81841	8,51593	77,28322
8,45932	27,65565	8,60254	71,09124
8,55356	25,53154	8,68915	64,4844
8,6478	22,85941	8,77576	57,81868
8,74203	20,14251	8,86237	51,40378
8,83627	17,77651	8,94898	45,48241
8,93051	15,95038	9,03559	40,22038
9,02475	14,6666	9,1222	35,70764
9,11898	13,82595	9,20881	31,97418
9,21322	13,30946	9,29542	29,05393
9,30746	13,02249	9,38203	27,21875
9,40169	12,90324	9,46864	27,53228
9,49593	12,91333	9,55525	32,22611
9,59017	13,02579	9,64186	43,02766
9,68441	13,21692	9,72847	56,92662
9,77864	13,4626	9,81508	65,14694
9,87288	13,73748	9,90169	60,89185
9,96712	14,01556	9,98831	47,32146
10,06136	14,2714	10,07492	33,62268

10,15559	14,48141	10,16153	25,33977
10,24983	14,62509	10,24814	21,98657
10,34407	14,6861	10,33475	21,03555
10,43831	14,65303	10,42136	20,83868
10,53254	14,51991	10,50797	20,80557
10,62678	14,28623	10,59458	20,79919
10,72102	13,95678	10,68119	20,79707
10,81525	13,54099	10,7678	20,79609
10,90949	13,05209	10,85441	20,79561
11,00373	12,5061	10,94102	20,79538
11,09797	11,92061	11,02763	20,79528
11,1922	11,3137	11,11424	20,79523
11,28644	10,70279	11,20085	20,79521
11,38068	10,10382	11,28746	20,7952
11,47492	9,53046	11,37407	20,7952
11,56915	8,99372	11,46068	20,79519
11,66339	8,50169	11,54729	20,79519
11,75763	8,05955	11,6339	20,79519
11,85186	7,66976	11,72051	20,79519
11,9461	7,3324	11,80712	20,79519
12,04034	7,0456	11,89373	20,79519
12,13458	6,806	11,98034	20,79519
12,22881	6,6092	12,06695	20,79519
12,32305	6,45026	12,15356	20,79519
12,41729	6,32397	12,24017	20,79519
12,51153	6,22525	12,32678	20,79519
12,60576	6,14929	12,41339	20,79519
12,7	6,09178	12,5	20,79519

DESCRIPTION: Fitting data on chromatogram of control sample with Gauss-function

Independent variable	Gauss fit	Peak 1	Peak 2	Peak 3	Peak 4	Peak 5
7,14	18,89404	18,85501	5,90764	5,94389	5,90903	5,90718
7,23424	19,7807	19,73905	5,90913	5,94389	5,91016	5,90718
7,32847	20,57333	20,52447	5,91459	5,94389	5,91192	5,90718
7,42271	21,25753	21,18827	5,9323	5,94389	5,91461	5,90718
7,51695	21,83448	21,71033	5,98313	5,94389	5,91866	5,90718
7,61119	22,33347	22,07439	6,11204	5,94389	5,92469	5,90718
7,70542	22,82487	22,26887	6,40015	5,94389	5,93349	5,90718
7,79966	23,42154	22,28753	6,96547	5,94389	5,94619	5,90718
7,8939	24,25033	22,12976	7,93401	5,94389	5,96421	5,90718
7,98814	25,38259	21,80065	9,37018	5,94389	5,9894	5,90718
8,08237	26,74284	21,31067	11,18572	5,94389	6,0241	5,90718
8,17661	28,05376	20,67517	13,08511	5,94389	6,07113	5,90718
8,27085	28,88481	19,91355	14,61499	5,94389	6,13392	5,90718
8,36508	28,81841	19,04828	15,33135	5,94389	6,21642	5,90718
8,45932	27,65565	18,1038	15,00636	5,94389	6,32314	5,90718
8,55356	25,53154	17,10541	13,74482	5,94389	6,45895	5,90718
8,6478	22,85941	16,07811	11,92991	5,94389	6,62903	5,90718
8,74203	20,14251	15,04567	10,036	5,94389	6,83849	5,90718
8,83627	17,77651	14,02968	8,4323	5,94389	7,09217	5,90718
8,93051	15,95038	13,04896	7,28491	5,94389	7,39415	5,90718
9,02475	14,6666	12,1191	6,57779	5,94389	7,74736	5,90718
9,11898	13,82595	11,25216	6,19839	5,94389	8,15305	5,90718
9,21322	13,30946	10,45674	6,01999	5,94389	8,61038	5,90718
9,30746	13,02249	9,73801	5,94617	5,94389	9,11595	5,90718

9,40169	12,90324	9,09813	5,9192	5,94389	9,66356	5,90718
9,49593	12,91333	8,53653	5,91049	5,94389	10,24396	5,90718
9,59017	13,02579	8,05045	5,90799	5,94389	10,84499	5,90718
9,68441	13,21692	7,63544	5,90736	5,94389	11,45177	5,90718
9,77864	13,4626	7,2858	5,90721	5,94389	12,04724	5,90718
9,87288	13,73748	6,99505	5,90718	5,94389	12,61288	5,90718
9,96712	14,01556	6,75639	5,90718	5,94389	13,12963	5,90718
10,06136	14,2714	6,56296	5,90718	5,94389	13,57891	5,90718
10,15559	14,48141	6,40813	5,90718	5,94389	13,94374	5,90718
10,24983	14,62509	6,28574	5,90718	5,94389	14,20981	5,90718
10,34407	14,6861	6,19018	5,90718	5,94389	14,36638	5,90718
10,43831	14,65303	6,11646	5,90718	5,94389	14,40704	5,90718
10,53254	14,51991	6,06028	5,90718	5,94389	14,33009	5,90718
10,62678	14,28623	6,01798	5,90718	5,94389	14,13872	5,90718
10,72102	13,95678	5,9865	5,90718	5,94389	13,84075	5,90718
10,81525	13,54099	5,96335	5,90718	5,94389	13,4481	5,90718
10,90949	13,05209	5,94653	5,90718	5,94389	12,97603	5,90718
11,00373	12,5061	5,93445	5,90718	5,94389	12,44211	5,90718
11,09797	11,92061	5,92588	5,90718	5,94389	11,8652	5,90718
11,1922	11,3137	5,91986	5,90718	5,94389	11,2643	5,90718
11,28644	10,70279	5,91569	5,90718	5,94389	10,65757	5,90718
11,38068	10,10382	5,91283	5,90718	5,94389	10,06146	5,90718
11,47492	9,53046	5,91089	5,90718	5,94389	9,49004	5,90718
11,56915	8,99372	5,90959	5,90718	5,94389	8,9546	5,90718
11,66339	8,50169	5,90873	5,90718	5,94389	8,46343	5,90718
11,75763	8,05955	5,90816	5,90718	5,94389	8,02186	5,90718
11,85186	7,66976	5,9078	5,90718	5,94389	7,63243	5,90718
11,9461	7,3324	5,90756	5,90718	5,94389	7,2953	5,90718
12,04034	7,0456	5,90742	5,90718	5,94389	7,00865	5,90718

12,13458	6,806	5,90732	5,90718	5,94389	6,76914	5,90718
12,22881	6,6092	5,90727	5,90718	5,94389	6,5724	5,90718
12,32305	6,45026	5,90723	5,90718	5,94389	6,41349	5,90718
12,41729	6,32397	5,90721	5,90718	5,94389	6,28723	5,90718
12,51153	6,22525	5,9072	5,90718	5,94389	6,18851	5,90718
12,60576	6,14929	5,90719	5,90718	5,94389	6,11257	5,90718
12,7	6,09178	5,90718	5,90718	5,94389	6,05506	5,90718

DESCRIPTION: Fitting data on chromatogram of sample treated with magnetic poulse energy for t=72h and ω=1-10 Hz, using Gauss-fitting function

Independent variable	Gauss fit	Peak 1	Peak 2
7,39	47,73338	47,73338	20,79519
7,47661	53,8724	53,8724	20,79519
7,56322	60,41863	60,41863	20,79519
7,64983	67,10131	67,10131	20,79519
7,73644	73,58953	73,58953	20,79519
7,82305	79,51697	79,51697	20,79519
7,90966	84,51492	84,51492	20,79519
7,99627	88,24978	88,24978	20,79519
8,08288	90,45974	90,45974	20,79519
8,16949	90,98517	90,98517	20,79519
8,2561	89,78758	89,78758	20,79519
8,34271	86,95437	86,95437	20,79519
8,42932	82,68823	82,68823	20,79519
8,51593	77,28322	77,28322	20,79519
8,60254	71,09124	71,09124	20,79519
8,68915	64,4844	64,4844	20,79519
8,77576	57,81868	57,81868	20,79519

8,86237	51,40378	51,40378	20,79519
8,94898	45,48241	45,48241	20,7952
9,03559	40,22038	40,22031	20,79526
9,1222	35,70764	35,70659	20,79624
9,20881	31,97418	31,96217	20,8072
9,29542	29,05393	28,9538	20,89533
9,38203	27,21875	26,61032	21,40363
9,46864	27,53228	24,83876	23,48872
9,55525	32,22611	23,53824	29,48306
9,64186	43,02766	22,61056	41,2123
9,72847	56,92662	21,96728	55,75454
9,81508	65,14694	21,53347	64,40867
9,90169	60,89185	21,24886	60,43819
9,98831	47,32146	21,06716	47,04949
10,07492	33,62268	20,95426	33,46362
10,16153	25,33977	20,88595	25,24902
10,24814	21,98657	20,84571	21,93605
10,33475	21,03555	20,82263	21,00811
10,42136	20,83868	20,80973	20,82415
10,50797	20,80557	20,80271	20,79806
10,59458	20,79919	20,79898	20,7954
10,68119	20,79707	20,79706	20,7952
10,7678	20,79609	20,79609	20,79519
10,85441	20,79561	20,79561	20,79519
10,94102	20,79538	20,79538	20,79519
11,02763	20,79528	20,79528	20,79519
11,11424	20,79523	20,79523	20,79519
11,20085	20,79521	20,79521	20,79519
11,28746	20,7952	20,7952	20,79519

11,37407	20,7952	20,7952	20,79519
11,46068	20,79519	20,79519	20,79519
11,54729	20,79519	20,79519	20,79519
11,6339	20,79519	20,79519	20,79519
11,72051	20,79519	20,79519	20,79519
11,80712	20,79519	20,79519	20,79519
11,89373	20,79519	20,79519	20,79519
11,98034	20,79519	20,79519	20,79519
12,06695	20,79519	20,79519	20,79519
12,15356	20,79519	20,79519	20,79519
12,24017	20,79519	20,79519	20,79519
12,32678	20,79519	20,79519	20,79519
12,41339	20,79519	20,79519	20,79519
12,5	20,79519	20,79519	20,79519

FILE: UNTITLED3.opj

DESCRIPTION: Tabulated form of molecular dynamics of ion at m/z 179 of clorogenic acid at MM2 level of theory (Computational detail: Step interval 2 fs; frame interval, 10 fs; targed temperature, 300K, heating/cooling rate 1 kcal/atom/ps). There is accounted for cis/trans isomers of two tautomeric forms of the acid depicted in **Figure S23**.

Time (fs)/Total energy (kcal/mol)/Potential energy (kcal/mol)/Temperature

T1_179_cis/T1_179_trans/T2_179_trans/T2_179_cis

0,004	-17,2828	-17,3434	0,85	heating	0,004	-5,5613	-5,6563	1,33	heating	0,004	46,3805	11,7591	483,96	heating	0,004	-9,4103	-9,4655	0,77	heating
0,006	-17,2429	-17,3228	1,12	heating	0,006	-5,4921	-5,6228	1,83	heating	0,006	46,521	3,4594	601,94	cooling	0,006	-9,363	-9,4178	0,77	heating
0,008	-17,2031	-17,2916	1,24	heating	0,008	-5,4571	-5,6142	2,2	heating	0,008	46,2592	8,611	526,27	cooling	0,008	-9,3274	-9,3993	1,01	heating
0,01	-17,1674	-17,2932	1,76	heating	0,01	-5,4308	-5,5666	1,9	heating	0,01	45,6456	10,8614	486,23	cooling	0,01	-9,293	-9,3774	1,18	heating
0,012	-17,1115	-17,2502	1,94	heating	0,012	-5,4095	-5,6015	2,68	heating	0,012	45,4063	5,5104	557,69	cooling	0,012	-9,2675	-9,3817	1,6	heating
0,014	-17,0964	-17,2797	2,56	heating	0,014	-5,3918	-5,6092	3,04	heating	0,014	45,53	3,0163	594,28	cooling	0,014	-9,2227	-9,3695	2,05	heating
0,016	-17,0359	-17,2062	2,38	heating	0,016	-5,3595	-5,5793	3,07	heating	0,016	45,5573	8,4268	519,03	cooling	0,016	-9,1821	-9,3388	2,19	heating
0,018	-17,0154	-17,2	2,58	heating	0,018	-5,3165	-5,5609	3,42	heating	0,018	45,2826	15,8735	411,09	cooling	0,018	-9,1269	-9,3558	3,2	heating
0,02	-16,9455	-17,1944	3,48	heating	0,02	-5,27	-5,5382	3,75	heating	0,02	45,0029	20,1428	347,51	cooling	0,02	-9,0852	-9,2925	2,9	heating
0,022	-16,8998	-17,0986	2,78	heating	0,022	-5,2139	-5,4926	3,9	heating	0,022	44,9469	22,4834	314,01	cooling	0,022	-9,0569	-9,3152	3,61	heating
0,024	-16,8585	-17,1235	3,7	heating	0,024	-5,1516	-5,4592	4,3	heating	0,024	44,952	24,7704	282,11	cooling	0,024	-9,0132	-9,2916	3,89	heating
0,026	-16,8256	-17,1001	3,84	heating	0,026	-5,1183	-5,4048	4	heating	0,026	44,9555	24,9688	279,38	cooling	0,026	-8,9753	-9,2925	4,43	heating
0,028	-16,7991	-17,0638	3,7	heating	0,028	-5,0792	-5,3925	4,38	heating	0,028	44,8256	20,4061	341,35	cooling	0,028	-8,9416	-9,2327	4,07	heating
0,03	-16,7862	-17,1068	4,48	heating	0,03	-5,0497	-5,3983	4,87	heating	0,03	44,9421	15,56	410,72	cooling	0,03	-8,9277	-9,2442	4,42	heating

0,032	-16,7505	-17,0694	4,46	heating	0,032	-5,0427	-5,3939	4,91	heating	0,032	45,1637	15,708	411,75	cooling	0,032	-8,8773	-9,1477	3,78	heating
0,034	-16,7419	-17,009	3,73	heating	0,034	-5,0336	-5,4571	5,92	heating	0,034	45,0511	16,5661	398,18	cooling	0,034	-8,8596	-9,2147	4,96	heating
0,036	-16,7113	-17,0686	4,99	heating	0,036	-4,9979	-5,4411	6,2	heating	0,036	44,762	12,2189	454,9	cooling	0,036	-8,7904	-9,1863	5,53	heating
0,038	-16,6775	-16,9519	3,84	heating	0,038	-4,9727	-5,4138	6,17	heating	0,038	44,5976	5,8141	542,14	cooling	0,038	-8,7763	-9,1942	5,84	heating
0,04	-16,6548	-17,0106	4,97	heating	0,04	-4,9245	-5,3721	6,26	heating	0,04	44,6145	4,4521	561,41	cooling	0,04	-8,7211	-9,1638	6,19	heating
0,042	-16,6169	-16,9931	5,26	heating	0,042	-4,882	-5,3085	5,96	heating	0,042	44,5627	9,2329	493,86	cooling	0,042	-8,6887	-9,0876	5,58	heating
0,044	-16,5859	-16,9407	4,96	heating	0,044	-4,8321	-5,2789	6,25	heating	0,044	44,4229	15,8943	398,79	cooling	0,044	-8,6731	-9,0667	5,5	heating
0,046	-16,5656	-16,9668	5,61	heating	0,046	-4,7851	-5,2961	7,14	heating	0,046	44,2917	19,8127	342,18	cooling	0,046	-8,6539	-9,0932	6,14	heating
0,048	-16,5404	-16,9739	6,06	heating	0,048	-4,7709	-5,3062	7,48	heating	0,048	43,9449	18,3778	357,39	cooling	0,048	-8,606	-9,1412	7,48	heating
0,05	-16,5225	-16,9163	5,51	heating	0,05	-4,7726	-5,3236	7,7	heating	0,05	44,1948	18,0216	365,86	cooling	0,05	-8,5765	-9,1043	7,38	heating
0,052	-16,5265	-17,0117	6,78	heating	0,052	-4,7874	-5,3266	7,54	heating	0,052	44,4017	21,6481	318,06	cooling	0,052	-8,5461	-9,146	8,39	heating
0,054	-16,4945	-16,9228	5,99	heating	0,054	-4,7843	-5,3781	8,3	heating	0,054	44,0746	19,7949	339,39	cooling	0,054	-8,5279	-8,9833	6,37	heating
0,056	-16,5058	-16,9549	6,28	heating	0,056	-4,7586	-5,3675	8,51	heating	0,056	44,1173	13,2846	431	cooling	0,056	-8,5249	-9,0893	7,89	heating
0,058	-16,4776	-17,0258	7,66	heating	0,058	-4,7216	-5,3954	9,42	heating	0,058	44,3867	11,72	456,63	cooling	0,058	-8,4877	-8,9994	7,15	heating
0,06	-16,4587	-16,9775	7,25	heating	0,06	-4,6786	-5,3829	9,84	heating	0,06	44,3715	13,2715	434,73	cooling	0,06	-8,4863	-9,044	7,8	heating
0,062	-16,4345	-17,024	8,24	heating	0,062	-4,6214	-5,3233	9,81	heating	0,062	44,1316	9,6958	481,36	cooling	0,062	-8,4483	-8,9547	7,08	heating
0,064	-16,4213	-17,0009	8,1	heating	0,064	-4,5912	-5,3003	9,91	heating	0,064	44,2063	3,001	575,99	cooling	0,064	-8,4297	-9,0182	8,23	heating
0,066	-16,3862	-16,9501	7,88	heating	0,066	-4,5779	-5,2836	9,87	heating	0,066	44,367	1,2068	603,32	cooling	0,066	-8,3546	-8,9682	8,58	heating
0,068	-16,3722	-16,9974	8,74	heating	0,068	-4,574	-5,2556	9,53	heating	0,068	44,3435	6,2412	532,61	cooling	0,068	-8,3614	-9,077	10	heating
0,07	-16,3281	-17,0583	10,21	heating	0,07	-4,5875	-5,3068	10,06	heating	0,07	44,2168	13,5376	428,85	cooling	0,07	-8,2797	-8,942	9,26	heating
0,072	-16,3044	-16,9886	9,56	heating	0,072	-4,5794	-5,3039	10,13	heating	0,072	44,1797	19,4817	345,24	cooling	0,072	-8,2934	-8,9184	8,74	heating
0,074	-16,2846	-17,0513	10,72	heating	0,074	-4,6033	-5,3141	9,94	heating	0,074	44,1818	22,7188	300,02	cooling	0,074	-8,229	-8,9504	10,08	heating
0,076	-16,2185	-16,9106	9,67	heating	0,076	-4,5605	-5,3085	10,46	heating	0,076	44,4207	26,2584	253,88	cooling	0,076	-8,2004	-8,8946	9,7	heating
0,078	-16,2208	-17,026	11,26	heating	0,078	-4,5263	-5,2308	9,85	heating	0,078	44,6622	29,7933	207,85	cooling	0,078	-8,1618	-8,9011	10,33	heating
0,08	-16,1619	-16,9492	11,01	heating	0,08	-4,4889	-5,1914	9,82	heating	0,08	44,5485	28,4417	225,15	cooling	0,08	-8,1483	-8,821	9,4	heating
0,082	-16,1345	-16,9607	11,55	heating	0,082	-4,4257	-5,1785	10,52	heating	0,082	44,4762	23,5655	292,3	cooling	0,082	-8,1139	-8,9046	11,05	heating
0,084	-16,0939	-16,9356	11,77	heating	0,084	-4,3627	-5,0873	10,13	heating	0,084	44,4237	20,5686	333,46	cooling	0,084	-8,0714	-8,9278	11,97	heating
0,086	-16,0682	-16,8986	11,61	heating	0,086	-4,3355	-5,1481	11,36	heating	0,086	44,2499	17,589	372,68	cooling	0,086	-8,0123	-9,011	13,96	heating
0,088	-16,0173	-16,8021	10,97	heating	0,088	-4,2846	-5,074	11,03	heating	0,088	44,0982	11,5045	455,61	cooling	0,088	-7,9874	-8,7125	10,14	heating
0,09	-16,0151	-16,8805	12,1	heating	0,09	-4,2771	-5,0621	10,97	heating	0,09	44,1059	4,9749	546,99	cooling	0,09	-7,9913	-8,774	10,94	heating
0,092	-15,9128	-16,6821	10,75	heating	0,092	-4,2372	-5,0774	11,74	heating	0,092	43,938	1,3209	595,72	cooling	0,092	-7,9239	-8,6434	10,06	heating
0,094	-15,9209	-16,7565	11,68	heating	0,094	-4,2054	-5,0247	11,45	heating	0,094	44,0861	3,901	561,73	cooling	0,094	-7,9417	-8,8309	12,43	heating
0,096	-15,8414	-16,7412	12,58	heating	0,096	-4,1809	-5,0542	12,21	heating	0,096	44,0544	10,3577	471,03	cooling	0,096	-7,8826	-8,8014	12,84	heating
0,098	-15,8387	-16,6054	10,72	heating	0,098	-4,1457	-5,0782	13,03	heating	0,098	43,9213	14,215	415,25	cooling	0,098	-7,903	-8,8467	13,19	heating
0,1	-15,7933	-16,7856	13,87	heating	0,1	-4,0783	-4,9292	11,89	heating	0,1	44,0908	17,6733	369,28	cooling	0,1	-7,8662	-8,7588	12,48	heating
0,102	-15,7504	-16,7245	13,62	heating	0,102	-4,0635	-4,9884	12,93	heating	0,102	44,3769	22,9919	298,93	cooling	0,102	-7,8739	-8,8735	13,97	heating
0,104	-15,7398	-16,7286	13,82	heating	0,104	-4,0051	-4,959	13,33	heating	0,104	44,2117	22,1816	307,95	cooling	0,104	-7,7919	-8,7605	13,54	heating
0,106	-15,7453	-16,7896	14,6	heating	0,106	-3,9973	-4,956	13,4	heating	0,106	44,3409	15,4445	403,93	cooling	0,106	-7,8164	-8,7578	13,16	heating
0,108	-15,6689	-16,5532	12,36	heating	0,108	-3,952	-5,0848	15,84	heating	0,108	44,5049	8,9522	496,97	cooling	0,108	-7,7655	-8,8008	14,47	heating
0,11	-15,6855	-16,5471	12,04	heating	0,11	-3,9245	-4,9691	14,6	heating	0,11	44,4646	5,7423	541,28	cooling	0,11	-7,7717	-8,7853	14,17	heating
0,112	-15,6425	-16,7992	16,17	heating	0,112	-3,8942	-4,9451	14,69	heating	0,112	44,4385	7,4982	516,37	cooling	0,112	-7,7384	-8,812	15,01	heating
0,114	-15,5945	-16,5746	13,7	heating	0,114	-3,8547	-4,9035	14,66	heating	0,114	44,1659	10,1761	475,13	cooling	0,114	-7,735	-8,6763	13,16	heating
0,116	-15,6096	-16,8457	17,28	heating	0,116	-3,8206	-4,7969	13,65	heating	0,116	43,8193	8,7848	489,73	cooling	0,116	-7,695	-8,6762	13,72	heating
0,118	-15,5428	-16,5988	14,76	heating	0,118	-3,823	-4,915	15,27	heating	0,118	43,9392	8,4197	496,51	cooling	0,118	-7,6961	-8,6356	13,13	heating
0,12	-15,5643	-16,5035	13,13	heating	0,12	-3,7812	-5,0529	17,78	heating	0,12	43,9633	11,9702	447,22	cooling	0,12	-7,6905	-8,8756	16,56	heating
0,122	-15,5267	-16,729	16,81	heating	0,122	-3,7449	-4,9724	17,16	heating	0,122	43,645	15,106	398,93	cooling	0,122	-7,6538	-8,7395	15,18	heating
0,124	-15,5019	-16,7182	17	heating	0,124	-3,7461	-5,0208	17,82	heating	0,124	43,5186	18,3298	352,1	cooling	0,124	-7,6362	-8,7701	15,85	heating
0,126	-15,4973	-16,6009	15,43	heating	0,126	-3,6957	-4,7536	14,79	heating	0,126	43,2002	21,0721	309,32	cooling	0,126	-7,5823	-8,5273	13,21	heating
0,128	-15,4951	-16,8937	19,55	heating	0,128	-3,6928	-4,6932	13,98	heating	0,128	43,1843	23,386	276,75	cooling	0,128	-7,5932	-8,5808	13,81	heating
0,13	-15,402	-16,4913	15,23	heating	0,13	-3,658	-4,7922	15,85	heating	0,13	43,1592	26,1917	237,18	cooling	0,13	-7,5247	-8,526	14	heating
0,132	-15,4848	-16,7492	17,67	heating	0,132	-3,6262	-4,7604	15,85	heating	0,132	42,9345	23,6207	269,98	cooling	0,132	-7,5347	-8,6827	16,05	heating
0,134	-15,3532	-16,852	20,95	heating	0,134	-3,5748	-4,7068	15,82	heating	0,134	42,9409	17,6312	353,79	cooling	0,134	-7,4536	-8,5055	14,7	heating
0,136	-15,3726	-16,6086	17,28	heating	0,136	-3,5638	-4,6865	15,69	heating	0,136	43,2856	14,6257	400,62	cooling	0,136	-7,509	-8,5388	14,4	heating
0,138	-15,3236	-16,8534	21,38	heating	0,138	-3,5372	-4,4976	13,42	heating	0,138	42,9037	10,2198	456,87	cooling	0,138	-7,4137	-8,3821	13,54	heating
0,14	-15,2516	-16,6499	19,55	heating	0,14	-3,5441	-4,6492	15,45	heating	0,14	43,4902	10,9079	455,45	cooling	0,14	-7,4571	-8,4293	13,59	heating
0,142	-15,2153	-16,4526	17,29	heating	0,142	-3,489	-4,5947	15,46	heating	0,142	43,4256	13,3228	420,79	cooling	0,142	-7,3975	-8,66	17,65	heating

0,144	-15,2139	-16,8278	22,56	heating	0,144	-3,5001	-4,4981	13,95	heating	0,144	43,0923	9,7745	465,73	cooling	0,144	-7,3698	-8,6499	17,89	heating
0,146	-15,1307	-16,648	21,21	heating	0,146	-3,4679	-4,5582	15,24	heating	0,146	43,3587	6,5252	514,88	cooling	0,146	-7,3138	-8,6007	17,99	heating
0,148	-15,157	-16,5017	18,8	heating	0,148	-3,4289	-4,4624	14,45	heating	0,148	43,143	4,4411	540,99	cooling	0,148	-7,3117	-8,4334	15,68	heating
0,15	-15,1197	-16,7617	22,95	heating	0,15	-3,3971	-4,5531	16,16	heating	0,15	43,2379	2,099	575,06	cooling	0,15	-7,2723	-8,4149	15,97	heating
0,152	-15,0521	-16,2459	16,69	heating	0,152	-3,3593	-4,6491	18,03	heating	0,152	43,666	9,1897	481,93	cooling	0,152	-7,2626	-8,3328	14,96	heating
0,154	-15,131	-16,7604	22,78	heating	0,154	-3,298	-4,5652	17,71	heating	0,154	43,6072	19,8915	331,51	cooling	0,154	-7,2282	-8,5908	19,05	heating
0,156	-15,0116	-16,8016	25,02	heating	0,156	-3,2739	-4,5525	17,87	heating	0,156	43,2801	23,9069	270,81	cooling	0,156	-7,1868	-8,3299	15,98	heating
0,158	-15,0362	-16,5038	20,52	heating	0,158	-3,211	-4,5068	18,11	heating	0,158	43,6072	22,815	290,64	cooling	0,158	-7,2239	-8,5727	18,85	heating
0,16	-15,0278	-16,6134	22,17	heating	0,16	-3,1856	-4,32	15,86	heating	0,16	43,0127	13,0943	418,21	cooling	0,16	-7,1445	-8,4072	17,65	heating
0,162	-14,9714	-16,5118	21,53	heating	0,162	-3,2104	-4,5293	18,44	heating	0,162	43,5188	6,1338	522,59	cooling	0,162	-7,155	-8,4245	17,75	heating
0,164	-14,9361	-16,2481	18,34	heating	0,164	-3,1796	-4,4476	17,72	heating	0,164	43,5794	9,4239	477,44	cooling	0,164	-7,0944	-8,2715	16,45	heating
0,166	-14,9952	-16,8823	26,38	heating	0,166	-3,1922	-4,5184	18,54	heating	0,166	43,1584	9,4357	471,39	cooling	0,166	-7,0511	-8,3745	18,5	heating
0,168	-14,8561	-16,4181	21,83	heating	0,168	-3,1356	-4,5692	20,04	heating	0,168	43,2358	6,1026	519,07	cooling	0,168	-6,9493	-8,1261	16,45	heating
0,17	-14,9702	-16,4902	21,25	heating	0,17	-3,1093	-4,5289	19,84	heating	0,17	43,782	7,883	501,81	cooling	0,17	-7,0085	-8,3584	18,87	heating
0,172	-14,8919	-16,7719	26,28	heating	0,172	-3,0725	-4,5454	20,59	heating	0,172	43,4319	8,3997	489,7	cooling	0,172	-6,8835	-8,0715	16,61	heating
0,174	-14,8984	-16,3928	20,89	heating	0,174	-3,037	-4,5762	21,52	heating	0,174	43,8717	12,5959	437,19	cooling	0,174	-6,9356	-8,0464	15,53	heating
0,176	-14,9403	-16,7623	25,47	heating	0,176	-2,9931	-4,3455	18,9	heating	0,176	43,6592	17,7729	361,85	cooling	0,176	-6,8681	-8,3156	20,23	heating
0,178	-14,8938	-16,8241	26,98	heating	0,178	-3,0023	-4,4874	20,76	heating	0,178	43,3783	16,7938	371,61	cooling	0,178	-6,8568	-8,2916	20,06	heating
0,18	-14,8793	-16,3808	20,99	heating	0,18	-2,9287	-4,4727	21,58	heating	0,18	43,7385	16,6534	378,61	cooling	0,18	-6,7871	-8,2758	20,81	heating
0,182	-14,9511	-16,7305	24,87	heating	0,182	-2,9715	-4,4842	21,14	heating	0,182	43,6416	18,1219	356,73	cooling	0,182	-6,7905	-8,0705	17,89	heating
0,184	-14,8331	-16,6094	24,83	heating	0,184	-2,9974	-4,5804	22,13	heating	0,184	43,5304	16,0913	383,56	cooling	0,184	-6,7435	-7,9116	16,33	heating
0,186	-14,8943	-16,3582	20,46	heating	0,186	-3,0121	-4,3505	18,71	heating	0,186	43,7512	17,7979	362,79	cooling	0,186	-6,7584	-8,0136	17,55	heating
0,188	-14,8986	-17,0139	29,57	heating	0,188	-3,0455	-4,3352	18,03	heating	0,188	43,501	17,7222	360,35	cooling	0,188	-6,6831	-8,4371	24,52	heating
0,19	-14,8082	-16,4297	22,67	heating	0,19	-3,0209	-4,4209	19,57	heating	0,19	43,2629	12,4287	431,02	cooling	0,19	-6,6553	-8,1087	20,32	heating
0,192	-14,8662	-16,432	21,89	heating	0,192	-2,9763	-4,3486	19,18	heating	0,192	43,6205	9,8315	472,32	cooling	0,192	-6,6665	-8,3741	23,87	heating
0,194	-14,823	-16,5611	24,3	heating	0,194	-2,9616	-4,4129	20,29	heating	0,194	43,2669	7,9425	493,78	cooling	0,194	-6,5637	-8,0121	20,25	heating
0,196	-14,7908	-16,2605	20,54	heating	0,196	-2,9013	-4,4293	21,36	heating	0,196	43,3929	6,3972	517,15	cooling	0,196	-6,5967	-8,0414	20,19	heating
0,198	-14,8061	-16,5936	24,99	heating	0,198	-2,8798	-4,2002	18,46	heating	0,198	43,4496	9,8144	470,17	cooling	0,198	-6,5238	-7,8619	18,7	heating
0,2	-14,7457	-16,9131	30,3	heating	0,2	-2,8319	-4,3468	21,17	heating	0,2	43,3844	14,3912	405,28	cooling	0,2	-6,5269	-7,9101	19,33	heating
0,202	-14,7872	-16,2637	20,64	heating	0,202	-3,18	-4,1558	13,64	heating	0,202	43,38	19,2123	337,83	cooling	0,202	-6,5582	-7,6962	15,91	heating
0,204	-14,8485	-16,6617	25,35	heating	0,204	-3,2015	-4,2432	14,56	heating	0,204	43,3841	22,939	285,79	cooling	0,204	-6,621	-8,2667	23	heating
0,206	-14,6626	-16,3646	23,79	heating	0,206	-3,1658	-4,4112	17,41	heating	0,206	42,9861	20,2411	317,94	cooling	0,206	-6,4724	-7,9378	20,48	heating
0,208	-14,7415	-16,4032	23,23	heating	0,208	-3,159	-4,2871	15,77	heating	0,208	43,1359	16,8047	368,07	cooling	0,208	-6,5699	-7,8806	18,32	heating
0,21	-14,6417	-16,9219	31,87	heating	0,21	-3,1362	-4,2873	16,09	heating	0,21	43,1668	15,6364	384,83	cooling	0,21	-6,4416	-7,8609	19,84	heating
0,212	-14,5907	-16,5421	27,28	heating	0,212	-3,1002	-4,4105	18,32	heating	0,212	43,1135	13,0585	420,12	cooling	0,212	-6,4942	-7,7046	16,92	heating
0,214	-14,5616	-16,4132	25,88	heating	0,214	-3,0788	-4,3706	18,06	heating	0,214	43,4456	11,8929	441,06	cooling	0,214	-6,4006	-7,9002	20,96	heating
0,216	-14,5481	-16,6063	28,77	heating	0,216	-3,0933	-4,6137	21,25	heating	0,216	43,6273	12,0824	440,95	cooling	0,216	-6,4044	-7,812	19,68	heating
0,218	-14,4121	-16,2084	25,11	heating	0,218	-3,0252	-4,4345	19,7	heating	0,218	43,465	8,3491	490,87	cooling	0,218	-6,3357	-7,6668	18,61	heating
0,22	-14,4847	-16,5794	29,28	heating	0,22	-2,9979	-4,0885	15,25	heating	0,22	43,7367	7,5076	506,43	cooling	0,22	-6,3506	-7,6909	18,74	heating
0,222	-14,3664	-16,7464	33,27	heating	0,222	-2,9405	-4,2327	18,06	heating	0,222	43,6491	10,0284	469,97	cooling	0,222	-6,2365	-7,9574	24,06	heating
0,224	-14,3572	-16,1903	25,63	heating	0,224	-2,8376	-4,1051	17,72	heating	0,224	43,4074	11,7985	441,85	cooling	0,224	-6,2279	-7,4726	17,4	heating
0,226	-14,3424	-16,5726	31,17	heating	0,226	-2,8219	-4,208	19,38	heating	0,226	43,218	13,8371	410,7	cooling	0,226	-6,2829	-7,972	23,61	heating
0,228	-14,2424	-16,2	27,36	heating	0,228	-2,7466	-4,2924	21,61	heating	0,228	42,8202	16,0306	374,48	cooling	0,228	-6,1896	-7,5204	18,6	heating
0,23	-14,2691	-16,3405	28,96	heating	0,23	-2,6867	-4,0431	18,96	heating	0,23	42,5286	17,1661	354,53	cooling	0,23	-6,2681	-7,896	22,76	heating
0,232	-14,1837	-16,5879	33,61	heating	0,232	-2,6919	-3,9894	18,14	heating	0,232	42,2604	17,5395	345,56	cooling	0,232	-6,2078	-7,8211	22,55	heating
0,234	-14,1361	-16,2709	29,84	heating	0,234	-2,6611	-4,1607	20,96	heating	0,234	41,8567	14,7532	378,87	cooling	0,234	-6,2285	-7,94	23,93	heating
0,236	-14,1283	-15,9773	25,85	heating	0,236	-2,6348	-3,9082	17,8	heating	0,236	41,9399	13,2703	400,76	cooling	0,236	-6,0869	-7,6041	21,21	heating
0,238	-14,1193	-16,2584	29,9	heating	0,238	-2,6921	-4,3243	22,82	heating	0,238	42,1707	15,8523	367,89	cooling	0,238	-6,1559	-8,0547	26,54	heating
0,24	-14,0148	-15,7725	24,57	heating	0,24	-2,6184	-4,3426	24,1	heating	0,24	42,1224	17,3818	345,84	cooling	0,24	-5,9935	-7,4671	20,6	heating
0,242	-14,1138	-16,4009	31,97	heating	0,242	-2,6131	-4,2438	22,79	heating	0,242	42,1539	15,1299	377,76	cooling	0,242	-6,0836	-7,5627	20,68	heating
0,244	-14,0054	-16,2716	31,68	heating	0,244	-2,5844	-4,3069	24,08	heating	0,244	42,2012	12,6424	413,19	cooling	0,244	-5,8943	-7,3257	20,01	heating
0,246	-14,0806	-16,006	26,91	heating	0,246	-2,5268	-3,9729	20,21	heating	0,246	42,0865	10,6066	440,04	cooling	0,246	-6,0039	-7,1948	16,65	heating
0,248	-14,0455	-16,1408	29,29	heating	0,248	-2,5139	-3,8535	18,73	heating	0,248	42,0011	9,9974	447,36	cooling	0,248	-5,9202	-7,4831	21,85	heating
0,25	-14,0241	-15,9922	27,51	heating	0,25	-2,5197	-4,1417	22,67	heating	0,25	41,9451	9,5714	452,54	cooling	0,25	-5,9639	-7,4818	21,22	heating
0,252	-14,0028	-16,1317	29,76	heating	0,252	-2,4379	-3,8586	19,86	heating	0,252	41,7164	6,7922	488,19	cooling	0,252	-5,9303	-7,5398	22,5	heating
0,254	-13,9902	-16,5036	35,13	heating	0,254	-2,5045	-3,9166	19,74	heating	0,254	42,0568	6,9118	491,27	cooling	0,254	-5,9624	-7,612	23,06	heating

0,256	-13,9245	-16,1286	30,81	heating	0,256	-2,433	-4,0907	23,17	heating	0,256	42,3087	11,7491	427,18	cooling	0,256	-5,8304	-7,5452	23,97	heating
0,258	-13,9997	-16,0866	29,17	heating	0,258	-2,4056	-3,8107	19,64	heating	0,258	42,2538	13,7741	398,1	cooling	0,258	-5,837	-7,0091	16,38	heating
0,26	-13,8953	-16,0636	30,31	heating	0,26	-2,3877	-4,1407	24,5	heating	0,26	42,3432	11,9196	425,28	cooling	0,26	-5,8213	-7,4407	22,64	heating
0,262	-13,8815	-15,7753	26,47	heating	0,262	-2,3277	-3,9382	22,51	heating	0,262	42,616	10,3067	451,64	cooling	0,262	-5,6944	-6,8523	16,19	heating
0,264	-13,8698	-16,301	33,98	heating	0,264	-2,3105	-3,7634	20,31	heating	0,264	42,5887	7,7503	486,99	cooling	0,264	-5,7565	-7,4148	23,18	heating
0,266	-13,7589	-15,9548	30,7	heating	0,266	-2,2909	-4,0318	24,34	heating	0,266	42,8029	8,7921	475,42	cooling	0,266	-5,6285	-7,2679	22,92	heating
0,268	-13,7867	-15,9726	30,56	heating	0,268	-2,2205	-4,0073	24,98	heating	0,268	42,5947	9,5746	461,57	cooling	0,268	-5,7006	-7,4972	25,11	heating
0,27	-13,7345	-15,9857	31,47	heating	0,27	-2,216	-4,0143	25,14	heating	0,27	42,4001	8,2316	477,62	cooling	0,27	-5,575	-7,4248	25,86	heating
0,272	-13,7101	-15,7878	29,04	heating	0,272	-2,2237	-4,4172	30,66	heating	0,272	42,558	10,2528	451,58	cooling	0,272	-5,6855	-7,827	29,93	heating
0,274	-13,7064	-15,9098	30,8	heating	0,274	-2,1593	-4,1177	27,38	heating	0,274	42,1909	12,5079	414,93	cooling	0,274	-5,5279	-7,0919	21,86	heating
0,276	-13,6628	-16,212	35,63	heating	0,276	-2,2495	-4,3396	29,22	heating	0,276	41,9481	10,5917	438,32	cooling	0,276	-5,6381	-7,3095	23,36	heating
0,278	-13,6247	-15,8468	31,06	heating	0,278	-2,1481	-4,2189	28,95	heating	0,278	41,8501	9,0901	457,94	cooling	0,278	-5,4463	-7,1257	23,48	heating
0,28	-13,6723	-16,2237	35,67	heating	0,28	-2,1636	-3,9061	24,36	heating	0,28	41,7593	10,1817	441,41	cooling	0,28	-5,5697	-7,2876	24,01	heating
0,282	-13,5186	-15,9118	33,45	heating	0,282	-2,1231	-4,1365	28,14	heating	0,282	41,7524	14,916	375,13	cooling	0,282	-5,413	-7,6649	31,48	heating
0,284	-13,5658	-15,9085	32,75	heating	0,284	-2,0894	-4,2872	30,72	heating	0,284	41,6513	20,1071	301,16	cooling	0,284	-5,4588	-7,3518	26,46	heating
0,286	-13,4778	-16,234	38,53	heating	0,286	-2,0158	-4,1988	30,52	heating	0,286	41,1934	18,3708	319,03	cooling	0,286	-5,4038	-7,2579	25,92	heating
0,288	-13,453	-16,0137	35,79	heating	0,288	-2,0241	-4,5692	35,58	heating	0,288	41,0777	14,4361	372,41	cooling	0,288	-5,4291	-7,1495	24,05	heating
0,29	-13,4307	-15,9885	35,75	heating	0,29	-1,8971	-4,1901	32,05	heating	0,29	40,9549	13,5965	382,43	cooling	0,29	-5,3041	-7,0766	24,78	heating
0,292	-13,4063	-16,0047	36,32	heating	0,292	-1,9472	-3,9076	27,4	heating	0,292	40,652	13,1805	384,01	cooling	0,292	-5,403	-6,6715	17,73	heating
0,294	-13,2966	-15,6043	32,26	heating	0,294	-1,9132	-3,9439	28,39	heating	0,294	40,5862	13,1138	384,02	cooling	0,294	-5,4048	-7,2204	25,38	heating
0,296	-13,3301	-15,9353	36,42	heating	0,296	-1,899	-3,5664	23,31	heating	0,296	40,4696	14,296	365,87	cooling	0,296	-5,2854	-6,8338	21,64	heating
0,298	-13,2224	-15,9782	38,52	heating	0,298	-1,9483	-4,0645	29,58	heating	0,298	40,2359	13,634	371,86	cooling	0,298	-5,3128	-7,5248	30,92	heating
0,3	-13,2136	-15,5826	33,12	heating	0,3	-1,8914	-4,3308	34,1	heating	0,3	40,2749	13,0266	380,89	cooling	0,3	-5,1855	-7,0317	25,81	heating
0,302	-13,1865	-15,9543	38,69	heating	0,302	-1,9055	-4,252	32,8	heating	0,302	40,1565	12,3138	389,2	cooling	0,302	-5,2234	-6,8487	22,72	heating
0,304	-13,1023	-15,7051	36,38	heating	0,304	-1,9078	-4,1349	31,13	heating	0,304	40,0578	11,3769	400,92	cooling	0,304	-5,0963	-6,497	19,58	heating
0,306	-13,1623	-16,0836	40,84	heating	0,306	-1,8849	-3,8495	27,46	heating	0,306	40,1902	12,7175	384,03	cooling	0,306	-5,1779	-7,119	27,13	heating
0,308	-13,093	-16,2374	43,95	heating	0,308	-1,8586	-3,4759	22,61	heating	0,308	40,1011	13,2411	375,46	cooling	0,308	-5,0352	-6,958	26,88	heating
0,31	-13,0573	-15,7249	37,29	heating	0,31	-1,8566	-3,9801	29,68	heating	0,31	39,9218	8,3123	441,85	cooling	0,31	-5,1428	-7,5496	33,64	heating
0,312	-13,0975	-15,5522	34,31	heating	0,312	-1,7177	-3,6902	27,57	heating	0,312	40,0113	4,0362	502,88	cooling	0,312	-4,9714	-7,2228	31,47	heating
0,314	-13,0714	-15,7345	37,23	heating	0,314	-1,7497	-3,7978	28,63	heating	0,314	40,2331	6,5168	471,3	cooling	0,314	-5,145	-7,2362	29,23	heating
0,316	-13,0143	-15,4308	33,78	heating	0,316	-1,6647	-3,8612	30,7	heating	0,316	40,3249	12,5667	388,02	cooling	0,316	-5,0007	-7,2006	30,75	heating
0,318	-13,1002	-16,1646	42,84	heating	0,318	-1,645	-3,5967	27,28	heating	0,318	40,5067	16,383	337,21	cooling	0,318	-5,0444	-6,792	24,43	heating
0,32	-12,9759	-15,8394	40,03	heating	0,32	-1,6278	-3,6813	28,71	heating	0,32	40,5777	14,7139	361,54	cooling	0,32	-4,9779	-6,8252	25,82	heating
0,322	-13,0402	-15,5343	34,86	heating	0,322	-1,5879	-3,7044	29,59	heating	0,322	40,7996	10,2039	427,68	cooling	0,322	-4,9901	-6,9592	27,53	heating
0,324	-12,97	-15,5411	35,94	heating	0,324	-1,5355	-3,5415	28,04	heating	0,324	41,027	9,5866	439,49	cooling	0,324	-4,8468	-7,3264	34,66	heating
0,326	-12,895	-15,1016	30,85	heating	0,326	-1,5353	-3,7586	31,08	heating	0,326	40,791	11,0227	416,12	cooling	0,326	-4,8837	-7,2432	32,98	heating
0,328	-12,9162	-15,2099	32,06	heating	0,328	-1,4426	-3,6446	30,78	heating	0,328	40,3784	10,7804	413,74	cooling	0,328	-4,8383	-7,3679	35,36	heating
0,33	-12,9203	-15,685	38,65	heating	0,33	-1,4607	-3,5121	28,67	heating	0,33	40,2014	11,4265	402,23	cooling	0,33	-4,7516	-6,4489	23,73	heating
0,332	-12,8643	-15,2442	33,27	heating	0,332	-1,4277	-3,9499	35,26	heating	0,332	39,9637	14,7045	353,09	cooling	0,332	-4,7831	-7,1151	32,6	heating
0,334	-12,9729	-15,4199	34,2	heating	0,334	-1,3768	-3,7486	33,15	heating	0,334	39,8834	17,8404	308,13	cooling	0,334	-4,6633	-6,7592	29,3	heating
0,336	-12,8888	-15,5946	37,82	heating	0,336	-1,3962	-3,7376	32,73	heating	0,336	39,7201	15,2812	341,62	cooling	0,336	-4,7234	-6,9715	31,43	heating
0,338	-12,8854	-15,4429	35,75	heating	0,338	-1,3947	-3,3161	26,86	heating	0,338	39,8197	10,7106	406,9	cooling	0,338	-4,5723	-6,5424	27,54	heating
0,34	-12,8896	-16,1642	45,77	heating	0,34	-1,3889	-2,9712	22,12	heating	0,34	40,1068	10,1421	418,86	cooling	0,34	-4,6781	-6,7334	28,73	heating
0,342	-12,8135	-15,715	40,56	heating	0,342	-1,3895	-3,1619	24,78	heating	0,342	39,8095	11,0162	402,49	cooling	0,342	-4,5635	-6,4639	26,57	heating
0,344	-12,8322	-15,4234	36,22	heating	0,344	-1,3235	-3,6008	31,83	heating	0,344	40,0264	13,7934	366,7	cooling	0,344	-4,6609	-7,1683	35,05	heating
0,346	-12,8479	-15,9503	43,37	heating	0,346	-1,2625	-3,4026	29,92	heating	0,346	39,7417	15,475	339,21	cooling	0,346	-4,4591	-6,9352	34,61	heating
0,348	-12,7379	-16,0427	46,2	heating	0,348	-1,2272	-3,4423	30,96	heating	0,348	39,5249	12,184	382,19	cooling	0,348	-4,6437	-7,2011	35,75	heating
0,35	-12,769	-16,2308	48,39	heating	0,35	-1,111	-2,9391	25,55	heating	0,35	39,626	11,2749	396,31	cooling	0,35	-4,4358	-7,1689	38,2	heating
0,352	-12,6782	-16,4705	53,01	heating	0,352	-1,1466	-2,9261	24,87	heating	0,352	39,4331	13,8812	357,18	cooling	0,352	-4,4971	-6,7776	31,88	heating
0,354	-12,6315	-15,4105	38,85	heating	0,354	-1,0257	-2,9782	27,29	heating	0,354	38,7138	11,8938	374,9	cooling	0,354	-4,4157	-6,7942	33,25	heating
0,356	-12,7303	-15,7823	42,66	heating	0,356	-1,0592	-2,9654	26,65	heating	0,356	38,7218	10,4363	395,39	cooling	0,356	-4,4345	-6,5585	29,69	heating
0,358	-12,5948	-15,9108	46,35	heating	0,358	-1,012	-3,1123	29,36	heating	0,358	38,4786	9,1544	409,91	cooling	0,358	-4,2788	-6,7971	35,2	heating
0,36	-12,6023	-15,6758	42,96	heating	0,36	-1,0195	-3,1046	29,15	heating	0,36	38,5709	6,3874	449,88	cooling	0,36	-4,3057	-6,8822	36,02	heating
0,362	-12,6204	-16,1164	48,87	heating	0,362	-0,9867	-2,9709	27,74	heating	0,362	39,12	5,6513	467,84	cooling	0,362	-4,2558	-7,306	42,64	heating
0,364	-12,5375	-15,8313	46,04	heating	0,364	-1,0113	-2,8821	26,15	heating	0,364	39,4268	5,2256	478,08	cooling	0,364	-4,1782	-6,7994	36,64	heating
0,366	-12,5512	-15,2777	38,11	heating	0,366	-0,9876	-2,8173	25,58	heating	0,366	40,0664	5,417	484,35	cooling	0,366	-4,2267	-7,3988	44,34	heating

0,368	-12,6311	-15,8272	44,68	heating	0,368	-1,0221	-2,8497	25,55	heating	0,368	40,6882	9,5528	435,23	cooling	0,368	-4,0722	-6,7388	37,27	heating
0,37	-12,4465	-15,135	37,58	heating	0,37	-0,9978	-2,9385	27,13	heating	0,37	41,0138	12,5869	397,37	cooling	0,37	-4,2001	-7,1393	41,09	heating
0,372	-12,5951	-15,2073	36,51	heating	0,372	-0,9983	-2,6327	22,85	heating	0,372	41,2412	13,1418	392,79	cooling	0,372	-3,9648	-6,5285	35,84	heating
0,374	-12,4693	-15,5306	42,79	heating	0,374	-0,9963	-2,7639	24,71	heating	0,374	41,7391	15,7773	362,91	cooling	0,374	-4,1184	-6,8418	38,07	heating
0,376	-12,4487	-14,777	32,55	heating	0,376	-0,9161	-2,7062	25,02	heating	0,376	41,5591	17,817	331,88	cooling	0,376	-3,9767	-6,7139	38,26	heating
0,378	-12,4857	-15,2309	38,37	heating	0,378	-0,901	-2,8905	27,81	heating	0,378	41,6785	18,939	317,87	cooling	0,378	-4,0996	-7,2648	44,24	heating
0,38	-12,3985	-15,6064	44,84	heating	0,38	-0,7714	-2,6441	26,18	heating	0,38	41,2626	15,8023	355,9	cooling	0,38	-3,9191	-6,9579	42,48	heating
0,382	-12,3457	-15,2366	40,41	heating	0,382	-0,7756	-2,5346	24,59	heating	0,382	41,0519	10,5788	425,97	cooling	0,382	-4,0648	-7,1518	43,15	heating
0,384	-12,4269	-15,6607	45,2	heating	0,384	-0,7041	-2,5881	26,33	heating	0,384	41,1057	10,4525	428,49	cooling	0,384	-3,8068	-6,4966	37,6	heating
0,386	-12,3079	-15,2007	40,44	heating	0,386	-0,738	-2,9316	30,66	heating	0,386	40,8205	13,0779	387,8	cooling	0,386	-3,952	-6,276	32,49	heating
0,388	-12,4227	-14,7365	32,34	heating	0,388	-0,6661	-2,8679	30,78	heating	0,388	40,5237	11,8781	400,42	cooling	0,388	-3,8204	-6,4503	36,76	heating
0,39	-12,5165	-15,9281	47,69	heating	0,39	-0,7315	-2,9832	31,48	heating	0,39	40,274	9,1129	435,59	cooling	0,39	-3,888	-6,5425	37,11	heating
0,392	-12,3658	-15,4553	43,19	heating	0,392	-0,677	-2,7455	28,92	heating	0,392	40,1819	7,0909	462,56	cooling	0,392	-3,7552	-7,2896	49,41	heating
0,394	-12,5222	-15,5714	42,62	heating	0,394	-0,717	-2,7987	29,1	heating	0,394	40,1238	7,7125	453,06	cooling	0,394	-3,758	-6,998	45,29	heating
0,396	-12,4664	-15,7066	45,29	heating	0,396	-0,6549	-2,9099	31,52	heating	0,396	40,1385	9,8294	423,67	cooling	0,396	-3,74	-6,8514	43,49	heating
0,398	-12,4439	-15,2474	39,19	heating	0,398	-0,6116	-2,837	31,11	heating	0,398	39,6913	9,2063	426,14	cooling	0,398	-3,666	-6,0438	33,24	heating
0,4	-12,467	-15,7761	46,26	heating	0,4	-0,5785	-2,9045	32,51	heating	0,4	39,8312	10,3111	412,65	cooling	0,4	-3,6755	-6,3968	38,04	heating
0,402	-12,419	-16,1694	52,43	heating	0,402	-0,5426	-2,6736	29,79	heating	0,402	39,8241	15,3523	342,08	cooling	0,402	-3,6891	-6,2111	35,25	heating
0,404	-12,2791	-15,0462	38,68	heating	0,404	-0,5083	-2,5736	28,87	heating	0,404	39,6633	17,2283	313,61	cooling	0,404	-3,7552	-7,2428	48,75	heating
0,406	-12,3708	-15,5631	44,62	heating	0,406	-0,4654	-2,4581	27,86	heating	0,406	39,6333	12,9025	373,66	cooling	0,406	-3,5035	-6,6932	44,59	heating
0,408	-12,1751	-15,1506	41,59	heating	0,408	-0,4371	-2,5648	29,74	heating	0,408	40,145	9,5667	427,44	cooling	0,408	-3,7017	-7,3887	51,54	heating
0,41	-12,2793	-15,0318	38,48	heating	0,41	-0,4041	-2,5543	30,06	heating	0,41	40,2722	7,7627	454,44	cooling	0,41	-3,527	-6,7217	44,66	heating
0,412	-12,205	-15,7287	49,26	heating	0,412	-0,4112	-2,8094	33,52	heating	0,412	40,7141	8,6136	448,72	cooling	0,412	-3,6755	-6,6498	41,58	heating
0,414	-12,1034	-15,1984	43,26	heating	0,414	-0,3631	-2,3489	27,76	heating	0,414	40,7755	9,7679	433,44	cooling	0,414	-3,5153	-6,4028	40,36	heating
0,416	-12,1505	-15,2627	43,5	heating	0,416	-0,412	-2,6202	30,87	heating	0,416	40,6539	7,2793	466,53	cooling	0,416	-3,6607	-6,8334	44,35	heating
0,418	-12,093	-15,6768	50,1	heating	0,418	-0,3159	-2,8834	35,89	heating	0,418	40,9135	5,6752	492,58	cooling	0,418	-3,4556	-6,9466	48,8	heating
0,42	-11,9186	-14,8732	41,3	heating	0,42	-0,3054	-3,0032	37,71	heating	0,42	41,0791	8,0025	462,36	cooling	0,42	-3,5574	-7,2622	51,79	heating
0,422	-12,0417	-15,0986	42,73	heating	0,422	-0,2181	-2,6628	34,17	heating	0,422	41,1982	10,3389	431,37	cooling	0,422	-3,4005	-6,7683	47,08	heating
0,424	-11,909	-15,401	48,81	heating	0,424	-0,2052	-2,248	28,56	heating	0,424	41,2832	12,7717	398,55	cooling	0,424	-3,5288	-6,4814	41,27	heating
0,426	-11,8651	-14,442	36,02	heating	0,426	-0,1487	-2,0598	26,72	heating	0,426	41,3159	15,8175	356,43	cooling	0,426	-3,3536	-6,8023	48,21	heating
0,428	-11,9105	-15,015	43,4	heating	0,428	-0,1814	-2,5934	33,72	heating	0,428	41,0501	16,645	341,15	cooling	0,428	-3,4477	-6,4693	42,24	heating
0,43	-11,8212	-14,557	38,24	heating	0,43	-0,0808	-2,7985	37,99	heating	0,43	41,0541	18,4997	315,28	cooling	0,43	-3,4237	-6,8889	48,44	heating
0,432	-11,9044	-14,6952	39,01	heating	0,432	-0,1269	-2,7309	36,4	heating	0,432	40,7439	20,2674	286,23	cooling	0,432	-3,3455	-6,2432	40,51	heating
0,434	-11,8739	-15,5628	51,57	heating	0,434	-0,0625	-2,6712	36,47	heating	0,434	40,0306	16,9594	322,5	cooling	0,434	-3,3324	-6,5763	45,35	heating
0,436	-11,7989	-15,3825	50,09	heating	0,436	-0,0906	-2,4155	32,5	heating	0,436	40,0077	16,0248	335,25	cooling	0,436	-3,3201	-6,5338	44,92	heating
0,438	-11,8566	-14,8917	42,43	heating	0,438	-0,0653	-2,6319	35,88	heating	0,438	39,3082	16,8189	314,37	cooling	0,438	-3,2908	-6,8884	50,29	heating
0,44	-11,8439	-15,3973	49,67	heating	0,44	-0,0313	-2,6196	36,18	heating	0,44	38,7369	13,2984	355,59	cooling	0,44	-3,1683	-6,3036	43,83	heating
0,442	-11,6661	-14,6719	42,02	heating	0,442	0,0229	-2,9032	40,9	heating	0,442	38,8474	10,4961	396,31	cooling	0,442	-3,2562	-6,9722	51,94	heating
0,444	-11,8577	-15,4498	50,21	heating	0,444	0,0635	-3,2031	45,66	heating	0,444	38,8116	6,9277	445,69	cooling	0,444	-3,1057	-6,1349	42,34	heating
0,446	-11,6663	-15,3949	52,12	heating	0,446	0,1233	-3,2613	47,31	heating	0,446	38,9876	2,5162	509,82	cooling	0,446	-3,2813	-6,5077	45,1	heating
0,448	-11,7685	-14,9339	44,25	heating	0,448	0,1712	-2,6972	40,1	heating	0,448	39,2114	7,9239	437,35	cooling	0,448	-3,0663	-6,1031	42,45	heating
0,45	-11,7921	-15,4693	51,4	heating	0,45	0,1584	-2,6408	39,13	heating	0,45	38,776	10,3748	397,01	cooling	0,45	-3,1996	-6,1543	41,3	heating
0,452	-11,8574	-15,4404	50,08	heating	0,452	0,2111	-2,5512	38,61	heating	0,452	38,6526	10,1311	398,69	cooling	0,452	-3,0377	-6,6263	50,16	heating
0,454	-11,8249	-15,0242	44,72	heating	0,454	0,1817	-2,978	44,17	heating	0,454	39,3342	14,9611	340,7	cooling	0,454	-3,1027	-7,1125	56,05	heating
0,456	-11,9448	-15,4726	49,31	heating	0,456	0,3027	-2,7529	42,71	heating	0,456	38,4588	11,291	379,77	cooling	0,456	-2,9646	-6,7892	53,46	heating
0,458	-11,8353	-15,0444	44,86	heating	0,458	0,2557	-2,5123	38,69	heating	0,458	39,3113	10,7165	399,71	cooling	0,458	-3,1075	-6,6603	49,66	heating
0,46	-11,9391	-15,0336	43,26	heating	0,46	0,3097	-2,4248	38,22	heating	0,46	38,5536	13,2377	353,88	cooling	0,46	-2,8853	-6,2209	46,63	heating
0,462	-11,8141	-15,4011	50,14	heating	0,462	0,2709	-2,7417	42,11	heating	0,462	37,8783	8,2131	414,68	cooling	0,462	-3,0411	-6,1542	43,52	heating
0,464	-11,7404	-14,7018	41,4	heating	0,464	0,3708	-2,6965	42,88	heating	0,464	38,1727	7,4575	429,35	cooling	0,464	-2,9541	-6,9853	56,35	heating
0,466	-11,8069	-15,321	49,12	heating	0,466	0,3875	-2,4459	39,61	heating	0,466	38,3775	11,6006	374,3	cooling	0,466	-2,903	-6,2382	46,62	heating
0,468	-11,6342	-15,0402	47,61	heating	0,468	0,4966	-2,1456	36,93	heating	0,468	38,2287	7,1312	434,7	cooling	0,468	-2,955	-6,5619	50,42	heating
0,47	-11,6449	-14,7557	43,48	heating	0,47	0,4696	-2,2771	38,39	heating	0,47	38,7085	11,3252	382,78	cooling	0,47	-2,9177	-6,3007	47,29	heating
0,472	-11,6511	-14,6383	41,76	heating	0,472	0,5438	-2,6399	44,5	heating	0,472	38,4011	13,1576	352,87	cooling	0,472	-2,9084	-6,3946	48,73	heating
0,474	-11,6361	-14,861	45,08	heating	0,474	0,5967	-2,6046	44,75	heating	0,474	38,4642	11,369	378,75	cooling	0,474	-2,8401	-6,5134	51,35	heating
0,476	-11,5985	-14,9621	47,02	heating	0,476	0,6396	-2,8771	49,16	heating	0,476	39,2606	15,3276	334,55	cooling	0,476	-2,8405	-6,9593	57,57	heating
0,478	-11,6496	-16,0071	60,91	heating	0,478	0,7076	-2,7443	48,25	heating	0,478	38,3416	12,0798	367,1	cooling	0,478	-2,7223	-5,9937	45,73	heating

0,48	-11,4585	-14,9782	49,2	heating	0,48	0,7	-2,9268	50,7	heating	0,48	39,3258	7,5408	444,31	cooling	0,48	-2,8695	-6,4484	50,03	heating
0,482	-11,5171	-14,8194	46,16	heating	0,482	0,7499	-2,6646	47,73	heating	0,482	38,833	11,0979	387,7	cooling	0,482	-2,6092	-5,5611	41,26	heating
0,484	-11,3443	-14,5305	44,54	heating	0,484	0,7102	-2,6023	46,3	heating	0,484	38,4807	11,0392	383,59	cooling	0,484	-2,8421	-5,8803	42,47	heating
0,486	-11,3635	-14,6511	45,96	heating	0,486	0,7409	-2,6926	48	heating	0,486	38,3913	10,7233	386,76	cooling	0,486	-2,6705	-6,5808	54,66	heating
0,488	-11,2406	-15,0385	53,09	heating	0,488	0,7412	-3,2779	56,18	heating	0,488	39,0148	14,1899	347,01	cooling	0,488	-2,7304	-6,6935	55,4	heating
0,49	-11,1728	-14,9896	53,35	heating	0,49	0,8278	-3,0149	53,71	heating	0,49	38,1063	8,3539	415,89	cooling	0,49	-2,6545	-6,7455	57,19	heating
0,492	-11,1005	-14,6204	49,2	heating	0,492	0,8005	-3,1397	55,08	heating	0,492	38,7321	9,2351	412,32	cooling	0,492	-2,6816	-6,5031	53,42	heating
0,494	-11,1128	-14,6573	49,55	heating	0,494	0,9168	-2,7782	51,65	heating	0,494	38,1114	7,7826	423,95	cooling	0,494	-2,5273	-5,7462	44,99	heating
0,496	-10,9278	-14,2739	46,77	heating	0,496	0,8411	-2,9582	53,11	heating	0,496	38,0538	2,0861	502,77	cooling	0,496	-2,7022	-6,1522	48,23	heating
0,498	-11,0046	-14,1947	44,59	heating	0,498	0,9341	-3,1759	57,45	heating	0,498	38,4858	3,0195	495,77	cooling	0,498	-2,5636	-6,7421	58,41	heating
0,5	-10,9108	-14,6458	52,21	heating	0,5	0,932	-3,0575	55,77	heating	0,5	38,4289	8,9498	412,07	cooling	0,5	-2,5407	-6,3768	53,62	heating
0,502	-10,8546	-14,1019	45,39	heating	0,502	0,959	-3,2532	58,88	heating	0,502	38,7701	11,7434	377,79	cooling	0,502	-2,516	-7,09	63,94	heating
0,504	-10,8471	-14,4168	49,9	heating	0,504	0,9402	-3,5161	62,29	heating	0,504	38,3893	15,6523	317,83	cooling	0,504	-2,4048	-5,8704	48,44	heating
0,506	-10,8211	-13,855	42,41	heating	0,506	1,0092	-3,3605	61,08	heating	0,506	38,1716	13,7007	342,07	cooling	0,506	-2,5462	-5,7683	45,04	heating
0,508	-10,8499	-14,0951	45,36	heating	0,508	0,9928	-3,0063	55,9	heating	0,508	37,9892	9,3029	400,99	cooling	0,508	-2,4461	-5,7442	46,1	heating
0,51	-10,8298	-14,7672	55,04	heating	0,51	1,0467	-2,8142	53,97	heating	0,51	38,4356	8,701	415,65	cooling	0,51	-2,4719	-6,2394	52,66	heating
0,512	-10,76	-15,2222	62,37	heating	0,512	1,0473	-2,6082	51,1	heating	0,512	37,9351	6,51	439,28	cooling	0,512	-2,4298	-6,4925	56,79	heating
0,514	-10,818	-14,717	54,5	heating	0,514	1,0796	-3,1938	59,74	heating	0,514	38,3065	7,3714	432,43	cooling	0,514	-2,4062	-6,9035	62,86	heating
0,516	-10,8238	-14,7553	54,96	heating	0,516	1,2024	-2,6054	53,23	heating	0,516	38,0746	10,4965	385,5	cooling	0,516	-2,204	-5,4101	44,82	heating
0,518	-10,7117	-14,0354	46,46	heating	0,518	1,1792	-2,52	51,71	heating	0,518	37,8341	13,1443	345,13	cooling	0,518	-2,422	-5,7747	46,87	heating
0,52	-10,9016	-15,2257	60,44	heating	0,52	1,2741	-2,417	51,6	heating	0,52	37,5906	13,1732	341,32	cooling	0,52	-2,1901	-5,4421	45,46	heating
0,522	-10,7248	-15,3077	64,06	heating	0,522	1,2947	-2,4715	52,65	heating	0,522	37,7771	14,359	327,35	cooling	0,522	-2,3738	-5,7348	46,98	heating
0,524	-10,8213	-14,758	55,03	heating	0,524	1,3103	-2,4112	52,02	heating	0,524	37,7503	15,1281	316,23	cooling	0,524	-2,2253	-6,2073	55,66	heating
0,526	-10,7706	-14,5173	52,37	heating	0,526	1,255	-2,5522	53,22	heating	0,526	37,7809	15,3619	313,38	cooling	0,526	-2,2369	-5,8459	50,45	heating
0,528	-10,8069	-14,0698	45,61	heating	0,528	1,2767	-2,3372	50,52	heating	0,528	38,0349	18,1996	277,27	cooling	0,528	-2,161	-5,877	51,94	heating
0,53	-10,686	-13,958	45,74	heating	0,53	1,1207	-3,132	59,45	heating	0,53	37,7095	16,6954	293,75	cooling	0,53	-2,2645	-6,2162	55,24	heating
0,532	-10,808	-14,8242	56,14	heating	0,532	1,2466	-3,1169	60,99	heating	0,532	37,616	11,7203	361,98	cooling	0,532	-2,1822	-6,1272	55,15	heating
0,534	-10,6394	-14,5355	54,46	heating	0,534	1,1721	-2,9609	57,77	heating	0,534	38,2124	12,2262	363,25	cooling	0,534	-2,2496	-6,1311	54,26	heating
0,536	-10,8159	-14,5139	51,69	heating	0,536	1,2902	-3,1336	61,84	heating	0,536	37,8134	9,6687	393,42	cooling	0,536	-2,1188	-6,499	61,23	heating
0,538	-10,6314	-14,4183	52,94	heating	0,538	1,3049	-2,9224	59,09	heating	0,538	37,8459	5,7167	449,12	cooling	0,538	-2,1104	-5,6892	50,03	heating
0,54	-10,7065	-13,3254	36,61	heating	0,54	1,3756	-2,9349	60,26	heating	0,54	37,3639	4,8282	454,8	cooling	0,54	-2,172	-6,1332	55,37	heating
0,542	-10,7493	-14,2057	48,31	heating	0,542	1,4234	-2,657	57,04	heating	0,542	36,4813	0,9464	496,73	cooling	0,542	-2,0265	-5,2519	45,09	heating
0,544	-10,6194	-14,2851	51,24	heating	0,544	1,5236	-2,4316	55,29	heating	0,544	36,329	1,1701	491,47	cooling	0,544	-2,1304	-5,5934	48,41	heating
0,546	-10,6435	-14,3651	52,02	heating	0,546	1,4529	-2,7414	58,63	heating	0,546	36,1397	7,9767	393,68	cooling	0,546	-2,0398	-5,9005	53,97	heating
0,548	-10,6415	-14,356	51,92	heating	0,548	1,4842	-3,1795	65,19	heating	0,548	35,5529	8,3625	380,08	cooling	0,548	-2,0044	-6,3484	60,72	heating
0,55	-10,5845	-13,8481	45,62	heating	0,55	1,5044	-2,5816	57,12	heating	0,55	35,9634	12,1357	333,08	cooling	0,55	-1,8577	-5,8313	55,54	heating
0,552	-10,5362	-13,5216	41,73	heating	0,552	1,4788	-2,7325	58,87	heating	0,552	35,9892	16,4183	273,57	cooling	0,552	-1,9572	-5,8686	54,68	heating
0,554	-10,6159	-15,0226	61,6	heating	0,554	1,6239	-2,2171	53,69	heating	0,554	35,9805	15,1255	291,52	cooling	0,554	-1,7708	-4,8876	43,57	heating
0,556	-10,4189	-14,0777	51,14	heating	0,556	1,5918	-2,6094	58,73	heating	0,556	37,0612	17,9725	266,83	cooling	0,556	-1,9597	-5,3817	47,83	heating
0,558	-10,5943	-14,3842	52,98	heating	0,558	1,7361	-2,4617	58,68	heating	0,558	36,6486	15,748	292,16	cooling	0,558	-1,7786	-5,8053	56,29	heating
0,56	-10,3584	-13,8299	48,53	heating	0,56	1,7187	-2,1702	54,36	heating	0,56	37,007	6,3573	428,44	cooling	0,56	-1,8232	-5,6245	53,14	heating
0,562	-10,5174	-13,5841	42,87	heating	0,562	1,7519	-2,1265	54,21	heating	0,562	37,2494	6,7417	426,45	cooling	0,562	-1,7279	-5,6299	54,54	heating
0,564	-10,4246	-14,4626	56,45	heating	0,564	1,6418	-2,8182	62,34	heating	0,564	36,7048	5,4409	437,02	cooling	0,564	-1,8183	-5,4055	50,14	heating
0,566	-10,3936	-14,7798	61,31	heating	0,566	1,7542	-2,4049	58,14	heating	0,566	36,6746	2,9929	470,82	cooling	0,566	-1,7295	-5,2091	48,64	heating
0,568	-10,3421	-14,2979	55,3	heating	0,568	1,6237	-2,2593	54,28	heating	0,568	37,4501	8,4813	404,94	cooling	0,568	-1,7533	-5,3692	50,55	heating
0,57	-10,4655	-15,0315	63,83	heating	0,57	1,731	-1,9081	50,87	heating	0,57	36,6373	7,0979	412,92	cooling	0,57	-1,6245	-5,9089	59,89	heating
0,572	-10,2548	-14,529	59,75	heating	0,572	1,6322	-1,9378	49,9	heating	0,572	37,5845	6,8752	429,27	cooling	0,572	-1,6288	-5,7789	58,01	heating
0,574	-10,4567	-14,436	55,62	heating	0,574	1,7961	-2,3005	57,27	heating	0,574	37,6837	11,4171	367,17	cooling	0,574	-1,6594	-6,4526	67	heating
0,576	-10,4142	-15,3399	68,85	heating	0,576	1,9163	-1,7916	51,83	heating	0,576	37,5661	9,0951	397,98	cooling	0,576	-1,5275	-5,477	55,21	heating
0,578	-10,3136	-14,3138	55,92	heating	0,578	2,0553	-1,3268	47,28	heating	0,578	38,4513	11,0557	382,95	cooling	0,578	-1,6385	-5,1289	48,79	heating
0,58	-10,4073	-14,957	63,6	heating	0,58	2,0749	-0,9669	42,52	heating	0,58	38,6184	16,8668	304,06	cooling	0,58	-1,545	-4,8711	46,49	heating
0,582	-10,2913	-14,5402	59,39	heating	0,582	2,1004	-1,25	46,83	heating	0,582	38,5384	16,0741	314,02	cooling	0,582	-1,5632	-5,4511	54,35	heating
0,584	-10,3088	-14,04	52,16	heating	0,584	2,0854	-1,2644	46,83	heating	0,584	38,7738	16,7864	307,35	cooling	0,584	-1,4854	-5,7951	60,24	heating
0,586	-10,2867	-14,3504	56,81	heating	0,586	2,0438	-1,6458	51,57	heating	0,586	38,3416	13,0599	353,4	cooling	0,586	-1,5696	-6,2324	65,18	heating
0,588	-10,2626	-14,9254	65,18	heating	0,588	1,9872	-1,5504	49,45	heating	0,588	38,1965	6,2055	447,19	cooling	0,588	-1,4257	-5,0432	50,57	heating
0,59	-10,2336	-13,9628	52,13	heating	0,59	1,8672	-2,1539	56,21	heating	0,59	38,6735	5,8375	459	cooling	0,59	-1,6014	-5,2658	51,22	heating

0,592	-10,4076	-14,9838	63,97	heating	0,592	1,9144	-1,6771	50,2	heating	0,592	37,9322	3,4942	481,39	cooling	0,592	-1,3735	-5,4064	56,37	heating
0,594	-10,2202	-14,0923	54,13	heating	0,594	1,809	-1,8573	51,25	heating	0,594	38,219	2,9571	492,91	cooling	0,594	-1,3802	-5,3194	55,06	heating
0,596	-10,5507	-14,6798	57,72	heating	0,596	1,9653	-1,6954	51,17	heating	0,596	37,8801	7,9944	417,76	cooling	0,596	-1,294	-5,5562	59,58	heating
0,598	-10,4221	-15,1705	66,38	heating	0,598	1,8651	-2,2894	58,07	heating	0,598	37,2317	9,3127	390,27	cooling	0,598	-1,3346	-4,9473	50,5	heating
0,6	-10,458	-14,339	54,25	heating	0,6	2,0258	-2,3936	61,78	heating	0,6	37,2791	11,7345	357,08	cooling	0,6	-1,3419	-4,7183	47,2	heating
0,602	-10,4496	-14,1456	51,66	heating	0,602	1,9953	-2,0175	56,09	heating	0,602	37,2088	15,8509	298,55	cooling	0,602	-1,3631	-5,1402	52,8	heating
0,604	-10,5042	-14,636	57,76	heating	0,604	2,1147	-1,4221	49,44	heating	0,604	36,9493	14,1804	318,28	cooling	0,604	-1,1976	-5,2893	57,2	heating
0,606	-10,2598	-14,1476	54,34	heating	0,606	2,0879	-1,5183	50,41	heating	0,606	37,1199	13,8433	325,37	cooling	0,606	-1,2735	-5,3975	57,65	heating
0,608	-10,4414	-15,0289	64,13	heating	0,608	2,2579	-1,4704	52,12	heating	0,608	36,6403	13,009	330,33	cooling	0,608	-1,1895	-5,8001	64,45	heating
0,61	-10,1381	-14,9468	67,22	heating	0,61	2,2385	-1,4865	52,07	heating	0,61	36,2779	9,1352	379,41	cooling	0,61	-1,1359	-4,559	47,85	heating
0,612	-10,1876	-13,7015	49,12	heating	0,612	2,3442	-1,5449	54,36	heating	0,612	36,5207	8,3247	394,14	cooling	0,612	-1,2515	-4,8573	50,4	heating
0,614	-10,1487	-14,5118	60,99	heating	0,614	2,3179	-1,3299	50,99	heating	0,614	36,6127	8,1378	398,04	cooling	0,614	-1,1174	-4,4882	47,12	heating
0,616	-10,1078	-13,9318	53,46	heating	0,616	2,2679	-1,9691	59,23	heating	0,616	36,9664	7,0335	418,42	cooling	0,616	-1,1232	-4,8824	52,55	heating
0,618	-10,1605	-14,1579	55,88	heating	0,618	2,3041	-1,542	53,76	heating	0,618	37,3843	8,9226	397,85	cooling	0,618	-1,0247	-5,7984	66,73	heating
0,62	-10,1704	-14,7049	63,39	heating	0,62	2,1672	-1,5635	52,15	heating	0,62	37,482	9,3279	393,55	cooling	0,62	-0,9937	-5,8354	67,68	heating
0,622	-10,0085	-14,2145	58,79	heating	0,622	2,1718	-1,3623	49,4	heating	0,622	37,724	7,9605	416,05	cooling	0,622	-0,9397	-4,8055	54,04	heating
0,624	-10,127	-13,8052	51,42	heating	0,624	2,0816	-1,532	50,51	heating	0,624	37,9114	7,945	418,89	cooling	0,624	-1,0855	-4,915	53,53	heating
0,626	-10,0876	-14,4978	61,65	heating	0,626	2,1352	-1,339	48,56	heating	0,626	38,0367	9,7973	394,74	cooling	0,626	-0,9056	-4,2674	46,99	heating
0,628	-9,882	-13,384	48,95	heating	0,628	2,1219	-1,5175	50,87	heating	0,628	38,0976	11,756	368,22	cooling	0,628	-1,0645	-4,9598	54,45	heating
0,63	-10,1054	-14,7918	65,51	heating	0,63	2,3651	-0,7456	43,48	heating	0,63	37,9002	16,6172	297,5	cooling	0,63	-0,8093	-5,5612	66,42	heating
0,632	-9,7568	-14,4806	66,03	heating	0,632	2,3302	-1,4384	52,68	heating	0,632	37,4729	18,9571	258,82	cooling	0,632	-0,9092	-4,9304	56,21	heating
0,634	-9,8629	-13,6024	52,27	heating	0,634	2,6136	-1,0657	51,43	heating	0,634	37,1269	19,1198	251,71	cooling	0,634	-0,9178	-4,9238	56	heating
0,636	-9,7483	-13,6922	55,13	heating	0,636	2,5854	-0,3738	41,37	heating	0,636	37,2136	20,4297	234,61	cooling	0,636	-0,8498	-4,4014	49,65	heating
0,638	-9,7719	-13,6146	53,72	heating	0,638	2,7302	0,0174	37,92	heating	0,638	36,5742	17,7101	263,69	cooling	0,638	-0,8513	-4,6552	53,17	heating
0,64	-9,6855	-13,4026	51,96	heating	0,64	2,7278	-0,0315	38,57	heating	0,64	36,7089	12,2501	341,9	cooling	0,64	-0,8778	-5,3343	62,3	heating
0,642	-9,8574	-14,9106	70,64	heating	0,642	2,7872	-0,548	46,62	heating	0,642	36,4473	8,6908	387,99	cooling	0,642	-0,7878	-5,4644	65,37	heating
0,644	-9,5356	-14,0581	63,22	heating	0,644	2,7347	-1,1909	54,87	heating	0,644	36,2248	4,3879	445,03	cooling	0,644	-0,7689	-4,7757	56,01	heating
0,646	-9,7995	-14,2333	61,98	heating	0,646	2,8749	-1,1658	56,48	heating	0,646	36,1461	2,6811	467,79	cooling	0,646	-0,8146	-4,5986	52,89	heating
0,648	-9,6195	-14,8757	73,47	heating	0,648	2,7903	-1,1328	54,84	heating	0,648	36,2428	6,0245	422,41	cooling	0,648	-0,719	-3,5098	39,01	heating
0,65	-9,6326	-13,9586	60,47	heating	0,65	2,8311	-1,6305	62,37	heating	0,65	35,7483	6,1471	413,78	cooling	0,65	-0,8291	-4,3751	49,57	heating
0,652	-9,7118	-14,7598	70,56	heating	0,652	2,9045	-0,7601	51,23	heating	0,652	35,6625	9,7842	361,74	cooling	0,652	-0,595	-4,6329	56,44	heating
0,654	-9,4862	-14,5616	70,95	heating	0,654	2,8424	-1,0228	54,03	heating	0,654	35,3719	10,9181	341,83	cooling	0,654	-0,726	-5,1212	61,44	heating
0,656	-9,4872	-14,1822	65,63	heating	0,656	2,8578	-0,9298	52,95	heating	0,656	35,2258	6,3893	403,09	cooling	0,656	-0,606	-5,04	61,98	heating
0,658	-9,535	-14,7099	72,34	heating	0,658	2,7359	-1,7121	62,18	heating	0,658	35,9723	4,5843	438,76	cooling	0,658	-0,6611	-4,6148	55,27	heating
0,66	-9,4922	-14,9043	75,65	heating	0,66	2,7742	-1,9679	66,29	heating	0,66	35,8922	3,8599	447,76	cooling	0,66	-0,6494	-4,6009	55,24	heating
0,662	-9,3832	-14,0033	64,58	heating	0,662	2,7593	-1,8585	64,55	heating	0,662	36,8229	4,1257	457,06	cooling	0,662	-0,6209	-4,8864	59,63	heating
0,664	-9,5409	-15,3866	81,71	heating	0,664	2,8571	-0,9761	53,58	heating	0,664	36,9115	10,7061	366,31	cooling	0,664	-0,5315	-4,7754	59,32	heating
0,666	-9,2425	-13,8935	65,01	heating	0,666	2,812	-1,3402	58,04	heating	0,666	36,7807	12,9729	332,8	cooling	0,666	-0,6433	-5,3903	66,36	heating
0,668	-9,4833	-14,097	64,49	heating	0,668	3,008	-1,0205	56,31	heating	0,668	36,8157	14,5327	311,48	cooling	0,668	-0,5287	-5,3504	67,4	heating
0,67	-9,2513	-13,9131	65,17	heating	0,67	3,0209	-0,7261	52,38	heating	0,67	37,1544	17,7504	271,24	cooling	0,67	-0,4891	-4,3918	54,55	heating
0,672	-9,3411	-13,6401	60,09	heating	0,672	3,2064	-0,3202	49,3	heating	0,672	36,4946	13,6907	318,76	cooling	0,672	-0,5397	-4,5656	56,28	heating
0,674	-9,1834	-13,8711	65,53	heating	0,674	3,1263	-0,4138	49,49	heating	0,674	37,0602	11,8939	351,79	cooling	0,674	-0,3897	-3,6167	45,11	heating
0,676	-9,155	-14,2819	71,67	heating	0,676	3,1776	-0,9388	57,54	heating	0,676	36,9115	10,1433	374,18	cooling	0,676	-0,4645	-4,2743	53,26	heating
0,678	-9,0339	-12,9307	54,47	heating	0,678	3,1127	-1,0146	57,69	heating	0,678	37,371	8,7425	400,18	cooling	0,678	-0,3342	-4,8857	63,62	heating
0,68	-9,2454	-13,5912	60,75	heating	0,68	3,1549	-0,7465	54,54	heating	0,68	37,957	12,3568	357,85	cooling	0,68	-0,3197	-4,7414	61,81	heating
0,682	-9,0229	-13,6007	63,99	heating	0,682	2,9642	-0,7409	51,79	heating	0,682	37,9918	14,6136	326,79	cooling	0,682	-0,3728	-4,8219	62,19	heating
0,684	-9,1007	-13,3167	58,93	heating	0,684	3,0009	-1,2757	59,78	heating	0,684	38,1904	12,8255	354,56	cooling	0,684	-0,2701	-4,8993	64,71	heating
0,686	-9,0681	-14,3065	73,23	heating	0,686	2,9935	-1,0262	56,19	heating	0,686	38,0316	14,2802	332,01	cooling	0,686	-0,2512	-4,1593	54,63	heating
0,688	-8,9049	-13,2818	61,18	heating	0,688	2,989	-1,1279	57,55	heating	0,688	37,8329	13,0691	346,16	cooling	0,688	-0,3435	-4,9534	64,44	heating
0,69	-8,9264	-13,6124	65,5	heating	0,69	3,0603	-0,6784	52,26	heating	0,69	37,7121	10,7913	376,31	cooling	0,69	-0,152	-4,1543	55,95	heating
0,692	-8,9417	-13,946	69,95	heating	0,692	3,114	-0,963	56,99	heating	0,692	37,5933	6,9007	429,04	cooling	0,692	-0,2546	-3,5362	45,87	heating
0,694	-8,8779	-13,9157	70,42	heating	0,694	3,258	-0,9443	58,74	heating	0,694	36,8573	3,0904	472,01	cooling	0,694	-0,3073	-4,5617	59,47	heating
0,696	-8,9038	-13,6984	67,02	heating	0,696	3,3819	-0,647	56,32	heating	0,696	36,9186	6,3389	427,46	cooling	0,696	-0,1418	-4,202	56,76	heating
0,698	-8,8221	-14,413	78,15	heating	0,698	3,5486	0,1684	47,25	heating	0,698	35,8248	10,2154	357,98	cooling	0,698	-0,2147	-4,79	63,96	heating
0,7	-8,8873	-13,4436	63,69	heating	0,7	3,4353	-0,453	54,35	heating	0,7	35,592	11,9685	330,22	cooling	0,7	-0,2218	-5,3626	71,86	heating
0,702	-9,0598	-14,7252	79,19	heating	0,702	3,5495	-0,7673	60,34	heating	0,702	35,3133	12,1258	324,13	cooling	0,702	-0,1019	-4,469	61,04	heating

0,704	-8,858	-14,0481	72,55	heating	0,704	3,4415	-1,1297	63,9	heating	0,704	35,5312	10,5981	348,53	cooling	0,704	-0,21	-4,478	59,66	heating
0,706	-9,0221	-14,0167	69,82	heating	0,706	3,6352	-1,008	64,91	heating	0,706	35,6961	7,3832	395,77	cooling	0,706	-0,0083	-4,1989	58,58	heating
0,708	-8,9409	-14,0278	71,11	heating	0,708	3,5312	-0,8979	61,91	heating	0,708	35,621	4,9061	429,35	cooling	0,708	-0,0708	-2,9026	39,58	heating
0,71	-8,8625	-13,4202	63,71	heating	0,71	3,5549	-1,2048	66,53	heating	0,71	35,6165	3,3236	451,41	cooling	0,71	-0,1206	-4,3857	59,62	heating
0,712	-8,8028	-13,3826	64,02	heating	0,712	3,4467	-1,4559	68,53	heating	0,712	35,586	5,6718	418,16	cooling	0,712	0,0396	-4,5177	63,7	heating
0,714	-8,9122	-14,204	73,97	heating	0,714	3,4786	-1,3927	68,09	heating	0,714	35,403	8,8491	371,18	cooling	0,714	-0,0619	-4,8984	67,61	heating
0,716	-8,6643	-13,7792	71,5	heating	0,716	3,3305	-1,521	67,82	heating	0,716	35,1301	11,1399	335,35	cooling	0,716	-0,0547	-5,4285	75,12	heating
0,718	-8,8449	-13,9827	71,82	heating	0,718	3,3445	-1,7692	71,48	heating	0,718	35,0179	11,4921	328,86	cooling	0,718	0,0923	-4,0055	57,28	heating
0,72	-8,5258	-13,3371	67,26	heating	0,72	3,3501	-1,4251	66,75	heating	0,72	34,6869	9,3799	353,75	cooling	0,72	-0,0111	-3,7393	52,11	heating
0,722	-8,7604	-12,7555	55,85	heating	0,722	3,2726	-1,5144	66,92	heating	0,722	35,1355	11,5594	329,56	cooling	0,722	0,0309	-4,8189	67,79	heating
0,724	-8,6364	-13,8958	73,52	heating	0,724	3,3264	-0,8427	58,28	heating	0,724	35,137	12,1138	321,83	cooling	0,724	0,2318	-4,0241	59,49	heating
0,726	-8,6745	-13,8404	72,21	heating	0,726	3,2417	-1,4327	65,34	heating	0,726	35,8467	12,1287	331,54	cooling	0,726	0,0196	-4,7518	66,7	heating
0,728	-8,6594	-14,1919	77,34	heating	0,728	3,4399	-1,6063	70,54	heating	0,728	36,4995	14,601	306,11	cooling	0,728	0,2059	-4,5799	66,9	heating
0,73	-8,6202	-13,7927	72,3	heating	0,73	3,4039	-1,5769	69,62	heating	0,73	36,9367	15,741	296,28	cooling	0,73	0,1685	-3,443	50,48	heating
0,732	-8,4894	-13,1917	65,73	heating	0,732	3,5053	-1,1205	64,66	heating	0,732	37,522	15,6769	305,36	cooling	0,732	0,1684	-4,5864	66,47	heating
0,734	-8,5601	-13,3892	67,5	heating	0,734	3,4523	-1,2828	66,19	heating	0,734	37,6041	12,9149	345,12	cooling	0,734	0,2408	-4,5477	66,94	heating
0,736	-8,4977	-14,2574	80,51	heating	0,736	3,5906	-0,9986	64,15	heating	0,736	37,7531	7,0864	428,67	cooling	0,736	0,1971	-4,3428	63,46	heating
0,738	-8,308	-13,4272	71,56	heating	0,738	3,556	-1,3071	67,98	heating	0,738	38,211	5,704	454,4	cooling	0,738	0,2532	-5,0896	74,68	heating
0,74	-8,4759	-14,5922	85,5	heating	0,74	3,8076	-0,7338	63,48	heating	0,74	38,1292	6,7207	439,04	cooling	0,74	0,4434	-4,2015	64,93	heating
0,742	-8,144	-13,3089	72,2	heating	0,742	3,7767	-0,9297	65,79	heating	0,742	37,9031	5,8348	448,27	cooling	0,742	0,2859	-3,7	55,72	heating
0,744	-8,4489	-13,8977	76,17	heating	0,744	3,8478	-1,5778	75,84	heating	0,744	37,9585	5,5121	453,55	cooling	0,744	0,3832	-4,2763	65,13	heating
0,746	-8,1585	-14,1681	84,01	heating	0,746	3,8617	-1,6074	76,45	heating	0,746	37,8112	5,4334	452,59	cooling	0,746	0,5647	-3,4169	55,66	heating
0,748	-8,3165	-14,1406	81,41	heating	0,748	3,822	-1,7398	77,75	heating	0,748	37,7726	7,9242	417,24	cooling	0,748	0,3186	-4,6173	69	heating
0,75	-8,2175	-14,247	84,28	heating	0,75	3,8204	-1,2082	70,29	heating	0,75	37,4559	11,2376	366,49	cooling	0,75	0,5265	-5,1681	79,6	heating
0,752	-8,1717	-14,2754	85,32	heating	0,752	3,704	-1,278	69,64	heating	0,752	37,2112	12,7837	341,46	cooling	0,752	0,5297	-4,1458	65,36	heating
0,754	-8,0171	-13,1999	72,45	heating	0,754	3,7026	-1,3091	70,06	heating	0,754	37,2228	14,9785	310,94	cooling	0,754	0,4445	-4,363	67,2	heating
0,756	-8,2245	-14,4649	87,23	heating	0,756	3,6967	-1,2206	68,74	heating	0,756	37,3135	15,605	303,45	cooling	0,756	0,4521	-4,3862	67,63	heating
0,758	-7,9204	-14,1398	86,94	heating	0,758	3,7453	-0,9364	65,44	heating	0,758	37,2982	11,9531	354,29	cooling	0,758	0,5106	-4,1705	65,44	heating
0,76	-8,0637	-13,6723	78,4	heating	0,76	3,8337	-1,6668	76,89	heating	0,76	37,8845	10,7456	379,36	cooling	0,76	0,434	-5,3875	81,38	heating
0,762	-7,9898	-14,1246	85,76	heating	0,762	4,0479	-1,0441	71,18	heating	0,762	37,3561	9,1949	393,65	cooling	0,762	0,6269	-4,9843	78,44	heating
0,764	-7,8615	-12,3	62,04	heating	0,764	4,0208	-1,3982	75,75	heating	0,764	37,8432	10,0472	388,55	cooling	0,764	0,5695	-4,3738	69,1	heating
0,766	-8,0527	-13,5661	77,07	heating	0,766	4,262	-0,7564	70,15	heating	0,766	37,9487	12,307	358,43	cooling	0,766	0,677	-4,7312	75,6	heating
0,768	-7,9563	-14,0503	85,19	heating	0,768	4,1896	-0,7065	68,44	heating	0,768	37,9366	10,3118	386,15	cooling	0,768	0,7121	-3,758	62,48	heating
0,77	-7,9948	-13,6256	78,71	heating	0,77	4,1849	-1,3087	76,79	heating	0,77	37,7281	5,7429	447,11	cooling	0,77	0,6042	-4,2336	67,63	heating
0,772	-8,0806	-13,5664	76,68	heating	0,772	4,2802	-1,0451	74,44	heating	0,772	38,0244	6,9177	434,83	cooling	0,772	0,8282	-4,47	74,06	heating
0,774	-7,9294	-13,5367	78,38	heating	0,774	4,2687	-1,0584	74,46	heating	0,774	37,1079	6,2081	431,93	cooling	0,774	0,807	-4,2192	70,26	heating
0,776	-8,0383	-12,7287	65,56	heating	0,776	4,1976	-1,9483	85,91	heating	0,776	36,581	5,9483	428,2	cooling	0,776	0,7205	-4,7555	76,55	heating
0,778	-8,1563	-15,2501	99,16	heating	0,778	4,2193	-2,0283	87,33	heating	0,778	36,3531	10,5601	360,55	cooling	0,778	0,825	-4,9569	80,82	heating
0,78	-7,8428	-14,2868	90,08	heating	0,78	4,1351	-1,8377	83,49	heating	0,78	35,7088	11,721	335,31	cooling	0,78	0,9044	-4,0477	69,22	heating
0,782	-8,1285	-15,0894	97,3	heating	0,782	4,2719	-1,1043	75,15	heating	0,782	35,8962	11,4413	341,84	cooling	0,782	0,7265	-4,4484	72,34	heating
0,784	-7,9621	-15,4477	104,64	heating	0,784	4,2385	0,264	55,56	heating	0,784	36,0197	11,9067	337,06	cooling	0,784	0,8931	-3,6993	64,2	heating
0,786	-7,8919	-13,9619	84,85	heating	0,786	4,1808	-0,9826	72,18	heating	0,786	36,9375	10,0355	376,05	cooling	0,786	0,8819	-3,3852	59,65	heating
0,788	-7,9702	-14,4971	91,24	heating	0,788	4,3349	-1,1797	77,09	heating	0,788	37,2342	5,8586	438,58	cooling	0,788	0,9438	-4,1334	70,97	heating
0,79	-7,9518	-14,9974	98,49	heating	0,79	4,2975	-1,2839	78,02	heating	0,79	37,2327	8,3019	404,41	cooling	0,79	0,9779	-4,2323	72,83	heating
0,792	-7,854	-13,8642	84,01	heating	0,792	4,3335	-1,2969	78,7	heating	0,792	36,8266	7,9682	403,4	cooling	0,792	1,0388	-4,6509	79,53	heating
0,794	-8,1376	-15,0401	96,49	heating	0,794	4,5082	-0,5218	70,31	heating	0,794	37,0078	9,0513	390,79	cooling	0,794	1,0835	-4,9753	84,69	heating
0,796	-7,7461	-13,9485	86,7	heating	0,796	4,4929	-0,3624	67,87	heating	0,796	36,9564	13,3108	330,53	cooling	0,796	1,0904	-4,901	83,75	heating
0,798	-8,1601	-13,0828	68,81	heating	0,798	4,5249	-1,1491	79,31	heating	0,798	36,016	13,3399	316,98	cooling	0,798	1,0792	-4,9372	84,1	heating
0,8	-8,0229	-14,7051	93,41	heating	0,8	4,6355	-0,3099	69,13	heating	0,8	37,0503	12,5605	342,33	cooling	0,8	1,1614	-4,9925	86,02	heating
0,802	-8,0375	-13,4166	75,19	heating	0,802	4,4959	-0,836	74,53	heating	0,802	36,0278	14,1616	305,66	cooling	0,802	1,1362	-3,9172	70,64	heating
0,804	-8,2335	-14,0077	80,71	heating	0,804	4,6653	-0,7805	76,12	heating	0,804	35,7158	10,4097	353,74	cooling	0,804	1,0532	-4,0815	71,78	heating
0,806	-8,1545	-14,031	82,14	heating	0,806	4,6366	0,1372	62,9	heating	0,806	35,7739	8,4705	381,66	cooling	0,806	1,255	-4,0524	74,19	heating
0,808	-7,9629	-13,0681	71,36	heating	0,808	4,6945	-0,1125	67,19	heating	0,808	37,4872	14,3941	322,81	cooling	0,808	1,2099	-4,5616	80,68	heating
0,81	-8,1494	-14,2185	84,84	heating	0,81	4,7071	0,2302	62,58	heating	0,81	36,4312	10,3881	364,04	cooling	0,81	1,2	-4,7449	83,1	heating
0,812	-7,9459	-14,5483	92,29	heating	0,812	4,7059	0,2192	62,72	heating	0,812	36,7809	14,4894	311,6	cooling	0,812	1,2344	-4,8352	84,84	heating
0,814	-7,8228	-13,0227	72,69	heating	0,814	4,7057	-0,2613	69,43	heating	0,814	36,0363	14,5501	300,35	cooling	0,814	1,354	-4,1041	76,3	heating

0,816	-7,9595	-14,6243	93,16	heating	0,816	4,9797	0,7164	59,59	heating	0,816	35,8461	8,03	388,83	cooling	0,816	1,2323	-4,18	75,66	heating
0,818	-7,4811	-13,2576	80,75	heating	0,818	4,7755	0,5135	59,58	heating	0,818	35,643	3,3031	452,06	cooling	0,818	1,2988	-3,9606	73,52	heating
0,82	-7,8605	-13,9336	84,89	heating	0,82	4,891	-0,6629	77,64	heating	0,82	36,3955	5,5526	431,14	cooling	0,82	1,3165	-3,8451	72,15	heating
0,822	-7,4698	-14,8664	103,39	heating	0,822	4,9626	-0,0594	70,2	heating	0,822	37,0309	2,1654	487,37	cooling	0,822	1,3443	-4,1984	77,48	heating
0,824	-7,6298	-14,1869	91,66	heating	0,824	4,8511	-0,5112	74,96	heating	0,824	36,249	10,2155	363,91	cooling	0,824	1,2908	-4,7692	84,71	heating
0,826	-7,573	-14,3391	94,58	heating	0,826	4,9736	0,211	66,57	heating	0,826	35,8055	13,4403	312,63	cooling	0,826	1,4273	-4,5885	84,09	heating
0,828	-7,6356	-14,9476	102,21	heating	0,828	4,9649	0,824	57,88	heating	0,828	35,7174	11,0118	345,35	cooling	0,828	1,364	-4,2379	78,31	heating
0,83	-7,3597	-13,6755	88,29	heating	0,83	4,9598	-0,5521	77,05	heating	0,83	37,0376	13,2754	332,16	cooling	0,83	1,4245	-3,9722	75,44	heating
0,832	-7,7458	-15,2158	104,42	heating	0,832	5,03	-0,7382	80,63	heating	0,832	35,5877	8,4566	379,25	cooling	0,832	1,4654	-3,7905	73,47	heating
0,834	-7,3673	-15,2753	110,54	heating	0,834	5,0754	-0,1281	72,74	heating	0,834	37,484	6,7508	429,61	cooling	0,834	1,5555	-4,5719	85,65	heating
0,836	-7,5273	-14,7138	100,46	heating	0,836	4,9675	-0,8577	81,43	heating	0,836	36,8644	10,4219	369,63	cooling	0,836	1,5909	-3,6904	73,83	heating
0,838	-7,4981	-15,7261	115,02	heating	0,838	5,2072	0,2502	69,29	heating	0,838	36,8635	10,6597	366,29	cooling	0,838	1,4963	-3,649	71,92	heating
0,84	-7,2156	-13,9501	94,14	heating	0,84	4,8651	0,1567	65,82	heating	0,84	36,7358	10,2507	370,22	cooling	0,84	1,6779	-4,1285	81,17	heating
0,842	-7,3025	-13,7689	90,39	heating	0,842	5,0115	0,1069	68,56	heating	0,842	38,5479	17,8558	289,24	cooling	0,842	1,7326	-4,6576	89,33	heating
0,844	-7,1542	-14,2145	98,69	heating	0,844	4,8403	0,8825	55,32	heating	0,844	37,3295	12,394	348,56	cooling	0,844	1,6204	-5,2633	96,22	heating
0,846	-7,1292	-13,9141	94,84	heating	0,846	4,7051	0,0498	65,07	heating	0,846	37,4447	12,9716	342,1	cooling	0,846	1,6867	-5,2344	96,75	heating
0,848	-7,0462	-13,6465	92,26	heating	0,848	4,7857	-0,0509	67,61	heating	0,848	36,9199	11,4933	355,43	cooling	0,848	1,8666	-3,2981	72,2	heating
0,85	-7,0508	-14,3249	101,68	heating	0,85	4,8824	0,8356	56,57	heating	0,85	36,5667	5,0405	440,69	cooling	0,85	1,5949	-3,6958	73,96	heating
0,852	-6,8452	-12,7517	82,56	heating	0,852	4,7971	0,1043	65,6	heating	0,852	36,6945	3,5442	463,39	cooling	0,852	1,7825	-3,8901	79,29	heating
0,854	-7,0427	-14,2278	100,44	heating	0,854	5,0203	0,4877	63,36	heating	0,854	37,0215	9,9417	378,54	cooling	0,854	1,7767	-3,7328	77,01	heating
0,856	-6,5942	-13,4767	96,21	heating	0,856	5,0961	1,0579	56,45	heating	0,856	37,9754	8,8065	407,74	cooling	0,856	1,7848	-4,9421	94,03	heating
0,858	-6,8487	-13,5176	93,22	heating	0,858	5,1675	0,1816	69,7	heating	0,858	37,1982	15,9458	297,08	cooling	0,858	1,8181	-4,2736	85,15	heating
0,86	-6,7966	-14,1103	102,23	heating	0,86	5,4439	0,979	62,41	heating	0,86	36,8659	16,2518	288,16	cooling	0,86	1,875	-3,8001	79,33	heating
0,862	-6,7839	-14,1744	103,31	heating	0,862	5,2797	-0,1344	75,68	heating	0,862	36,5059	9,8339	372,84	cooling	0,862	1,8964	-3,7984	79,6	heating
0,864	-6,7914	-14,159	102,99	heating	0,864	5,4676	-0,7997	87,61	heating	0,864	37,3576	10,5408	374,86	cooling	0,864	1,9691	-2,9618	68,93	heating
0,866	-7,0022	-15,062	112,67	heating	0,866	5,3917	0,1842	72,79	heating	0,866	35,9789	11,243	345,77	cooling	0,866	1,8842	-3,2222	71,38	heating
0,868	-6,9069	-14,2694	102,92	heating	0,868	5,4053	0,9821	61,83	heating	0,868	36,8999	8,7022	394,16	cooling	0,868	2,0565	-4,9173	97,48	heating
0,87	-7,0809	-14,4361	102,82	heating	0,87	5,4393	0,7581	65,44	heating	0,87	36,4498	10,561	361,89	cooling	0,87	2,1143	-3,8227	82,99	heating
0,872	-7,0308	-14,8728	109,62	heating	0,872	5,5537	1,0008	63,64	heating	0,872	36,1513	9,0925	378,24	cooling	0,872	1,882	-4,2962	86,36	heating
0,874	-7,0637	-14,0557	97,74	heating	0,874	5,4785	-0,3544	81,53	heating	0,874	36,0999	6,6893	411,12	cooling	0,874	2,2463	-3,1792	75,84	heating
0,876	-7,0892	-15,1068	112,07	heating	0,876	5,677	0,3791	74,06	heating	0,876	36,9174	11,8734	350,08	cooling	0,876	2,1267	-2,8096	69	heating
0,878	-6,9663	-14,7124	108,28	heating	0,878	5,5538	0,7341	67,37	heating	0,878	35,9534	12,3014	330,62	cooling	0,878	2,0345	-4,0747	85,4	heating
0,88	-7,061	-14,8133	108,37	heating	0,88	5,5894	0,0994	76,74	heating	0,88	36,188	13,2498	320,64	cooling	0,88	2,1638	-4,0733	87,19	heating
0,882	-7,0658	-14,5752	104,97	heating	0,882	5,8126	0,6858	71,67	heating	0,882	35,9142	12,5459	326,65	cooling	0,882	2,3147	-3,5442	81,9	heating
0,884	-6,9311	-14,4731	105,43	heating	0,884	5,7425	0,6258	71,52	heating	0,884	35,4623	7,304	393,61	cooling	0,884	2,1138	-4,5967	93,8	heating
0,886	-7,0167	-14,0043	97,68	heating	0,886	5,8069	0,6535	72,04	heating	0,886	35,7953	5,659	421,26	cooling	0,886	2,39	-3,6414	84,31	heating
0,888	-7,0611	-15,1655	113,29	heating	0,888	5,7798	1,0734	65,79	heating	0,888	35,1986	7,1876	391,55	cooling	0,888	2,1895	-3,8682	84,68	heating
0,89	-6,9319	-14,8114	110,14	heating	0,89	5,7313	0,6498	71,03	heating	0,89	35,4232	7,4437	391,11	cooling	0,89	2,3875	-4,7333	99,54	heating
0,892	-7,013	-15,8292	123,24	heating	0,892	5,7759	-0,1021	82,17	heating	0,892	35,4441	10,9962	341,74	cooling	0,892	2,1969	-4,005	86,69	heating
0,894	-6,9688	-15,5045	119,32	heating	0,894	5,7866	0,5198	73,62	heating	0,894	35,299	11,5407	332,11	cooling	0,894	2,2486	-4,5231	94,66	heating
0,896	-6,9972	-14,8191	109,34	heating	0,896	5,7027	-0,3572	84,71	heating	0,896	34,9537	8,0939	375,46	cooling	0,896	2,3764	-3,336	79,85	heating
0,898	-6,8251	-14,561	108,14	heating	0,898	5,7898	0,1771	78,46	heating	0,898	35,122	8,4531	372,79	cooling	0,898	2,2971	-2,6145	68,66	heating
0,9	-6,8015	-14,4152	106,43	heating	0,9	5,7227	0,9014	67,39	heating	0,9	34,615	7,9435	372,83	cooling	0,9	2,3186	-3,0243	74,69	heating
0,902	-6,6378	-13,9967	102,87	heating	0,902	5,7256	1,1364	64,15	heating	0,902	34,294	8,1875	364,93	cooling	0,902	2,5175	-3,8847	89,49	heating
0,904	-6,6861	-14,4154	108,04	heating	0,904	5,881	1,1073	66,73	heating	0,904	34,2822	10,5646	331,54	cooling	0,904	2,4286	-4,1208	91,55	heating
0,906	-6,351	-14,0603	107,76	heating	0,906	5,8111	0,0752	80,18	heating	0,906	33,643	9,8151	333,08	cooling	0,906	2,3907	-5,0416	103,89	heating
0,908	-6,5476	-14,0324	104,63	heating	0,908	5,9347	-0,1914	85,63	heating	0,908	33,9437	8,8534	350,73	cooling	0,908	2,7737	-3,1913	83,38	heating
0,91	-6,2715	-14,1279	109,82	heating	0,91	6,0503	1,2409	67,23	heating	0,91	33,8326	8,3279	356,52	cooling	0,91	2,5466	-3,313	81,91	heating
0,912	-6,3246	-13,5742	101,34	heating	0,912	6,0018	1,6956	60,19	heating	0,912	34,5228	8,4318	364,71	cooling	0,912	2,658	-2,4523	71,43	heating
0,914	-6,2747	-13,8411	105,77	heating	0,914	6,0285	0,9555	70,91	heating	0,914	34,9431	9,7949	351,53	cooling	0,914	2,505	-2,426	68,93	heating
0,916	-6,3164	-13,634	102,29	heating	0,916	6,0733	0,8322	73,26	heating	0,916	35,3681	9,367	363,46	cooling	0,916	2,7379	-3,2306	83,43	heating
0,918	-6,231	-13,8557	106,58	heating	0,918	6,0957	0,2274	82,03	heating	0,918	35,6755	6,7021	405,01	cooling	0,918	2,527	-4,4078	96,94	heating
0,92	-6,2433	-14,2286	111,62	heating	0,92	6,0734	1,1179	69,27	heating	0,92	36,2611	9,1743	378,63	cooling	0,92	2,6007	-4,5611	100,11	heating
0,922	-6,1651	-14,3694	114,68	heating	0,922	6,0361	1,3001	66,2	heating	0,922	36,1282	12,6408	328,32	cooling	0,922	2,5802	-4,1179	93,63	heating
0,924	-6,0618	-14,1259	112,72	heating	0,924	6,0433	0,6014	76,07	heating	0,924	35,9328	13,4031	314,93	cooling	0,924	2,8225	-2,9612	80,85	heating
0,926	-6,0622	-14,797	122,1	heating	0,926	6,2314	0,5822	78,97	heating	0,926	36,0172	15,011	293,63	cooling	0,926	2,5227	-3,2372	80,51	heating

0,928	-5,8797	-13,8103	110,86	heating	0,928	6,1111	0,574	77,4	heating	0,928	35,3193	13,3522	307,07	cooling	0,928	2,7756	-4,1106	96,26	heating
0,93	-6,0837	-14,4304	116,68	heating	0,93	6,225	0,4662	80,5	heating	0,93	34,9129	8,9306	363,19	cooling	0,93	2,7539	-3,855	92,38	heating
0,932	-5,7472	-13,9714	114,96	heating	0,932	6,2598	0,9191	74,66	heating	0,932	34,4953	6,3804	393	cooling	0,932	2,6815	-4,5	100,39	heating
0,934	-5,9255	-13,9242	111,81	heating	0,934	6,2142	0,3879	81,44	heating	0,934	34,2873	6,2265	392,25	cooling	0,934	2,8157	-3,1136	82,88	heating
0,936	-5,8272	-13,8119	111,61	heating	0,936	6,3869	0,3824	83,93	heating	0,936	34,2361	6,7662	383,99	cooling	0,936	2,8114	-3,7589	91,84	heating
0,938	-5,9014	-13,7333	109,48	heating	0,938	6,4002	0,8502	77,58	heating	0,938	34,1569	6,7861	382,6	cooling	0,938	2,8946	-3,591	90,66	heating
0,94	-5,8242	-13,3368	105,01	heating	0,94	6,3552	-0,2498	92,33	heating	0,94	34,1729	6,1992	391,03	cooling	0,94	2,7579	-3,5556	88,25	heating
0,942	-6,0513	-14,4437	117,31	heating	0,942	6,4184	0,0083	89,6	heating	0,942	34,2371	8,1753	364,31	cooling	0,942	2,9284	-3,4048	88,53	heating
0,944	-5,8774	-13,8487	111,43	heating	0,944	6,4651	0,4036	84,73	heating	0,944	34,2636	11,5458	317,56	cooling	0,944	2,9104	-4,3241	101,13	heating
0,946	-6,0476	-13,6303	105,99	heating	0,946	6,3854	-0,4296	95,26	heating	0,946	34,3403	13,596	289,97	cooling	0,946	3,0305	-3,1955	87,03	heating
0,948	-5,9941	-13,7183	107,97	heating	0,948	6,594	-0,4812	98,9	heating	0,948	34,2997	12,1979	308,95	cooling	0,948	2,8337	-3,6875	91,16	heating
0,95	-5,9941	-13,0619	98,8	heating	0,95	6,4661	-0,6553	99,55	heating	0,95	34,0536	7,8484	366,31	cooling	0,95	3,2837	-2,2068	76,75	heating
0,952	-6,0343	-14,1028	112,79	heating	0,952	6,6197	-0,4871	99,34	heating	0,952	34,0411	5,0587	405,13	cooling	0,952	2,8751	-2,8704	80,31	heating
0,954	-5,9727	-14,4309	118,23	heating	0,954	6,5539	0,6336	82,76	heating	0,954	33,4649	3,4603	419,42	cooling	0,954	3,0804	-4,0394	99,52	heating
0,956	-6,0179	-14,107	113,07	heating	0,956	6,4349	-0,3115	94,3	heating	0,956	33,5739	3,3938	421,87	cooling	0,956	3,1177	-3,0402	86,08	heating
0,958	-6,0161	-13,8582	109,62	heating	0,958	6,5446	-0,9418	104,65	heating	0,958	33,7745	6,6039	379,8	cooling	0,958	3,1558	-3,1563	88,23	heating
0,96	-5,9095	-13,7972	110,26	heating	0,96	6,5196	-0,3498	96,02	heating	0,96	33,6928	9,298	341	cooling	0,96	3,0427	-3,3936	89,97	heating
0,962	-5,962	-13,2587	102	heating	0,962	6,467	-0,1547	92,56	heating	0,962	33,5046	7,6792	361	cooling	0,962	3,2137	-3,6498	95,94	heating
0,964	-6,0186	-14,4626	118,04	heating	0,964	6,4703	0,7987	79,28	heating	0,964	33,2963	3,68	413,99	cooling	0,964	3,0758	-4,4964	105,85	heating
0,966	-5,7148	-13,8129	113,2	heating	0,966	6,4584	1,0121	76,13	heating	0,966	33,4534	3,5329	418,24	cooling	0,966	3,1752	-3,8988	98,88	heating
0,968	-5,7917	-14,1102	116,28	heating	0,968	6,3927	-0,2484	92,83	heating	0,968	33,2537	8,48	346,3	cooling	0,968	3,2095	-1,2869	62,85	heating
0,97	-5,6894	-13,9729	115,79	heating	0,97	6,7611	0,549	86,84	heating	0,97	32,9664	11,7114	297,11	cooling	0,97	3,1625	-2,9947	86,07	heating
0,972	-5,5421	-13,3376	108,97	heating	0,972	6,5885	-0,0795	93,21	heating	0,972	32,8752	13,5113	270,68	cooling	0,972	3,3874	-2,5547	83,06	heating
0,974	-5,3993	-13,171	108,64	heating	0,974	6,8356	-0,4809	102,27	heating	0,974	33,5897	17,9286	218,92	cooling	0,974	3,0794	-4,1355	100,85	heating
0,976	-5,3699	-13,7159	116,66	heating	0,976	6,8443	0,1276	93,89	heating	0,976	33,0032	13,7692	268,86	cooling	0,976	3,4739	-4,2314	107,71	heating
0,978	-5,2048	-13,1677	111,31	heating	0,978	6,9658	-0,5197	104,64	heating	0,978	34,047	8,8509	352,2	cooling	0,978	3,4366	-3,4834	96,73	heating
0,98	-5,2312	-13,4344	114,67	heating	0,98	7,1168	-0,524	106,81	heating	0,98	34,2926	7,6164	372,89	cooling	0,98	3,3793	-3,3914	94,64	heating
0,982	-5,0839	-13,3268	115,22	heating	0,982	7,1385	-0,0244	100,13	heating	0,982	34,4911	5,1807	409,72	cooling	0,982	3,3965	-3,8272	100,98	heating
0,984	-5,2841	-13,0166	108,09	heating	0,984	7,0888	-0,7084	108,99	heating	0,984	34,7439	4,8073	418,47	cooling	0,984	3,769	-1,8975	79,21	heating
0,986	-5,2682	-13,9006	120,67	heating	0,986	7,1316	-0,0552	100,46	heating	0,986	35,5948	10,1027	356,34	cooling	0,986	3,2013	-3,7199	96,75	heating
0,988	-5,3194	-13,8767	119,62	heating	0,988	6,9564	-0,2353	100,53	heating	0,988	34,9423	7,4605	384,15	cooling	0,988	3,6574	-3,7404	103,41	heating
0,99	-5,3093	-13,3188	111,96	heating	0,99	6,883	-2,2348	127,45	heating	0,99	35,1638	9,275	361,89	cooling	0,99	3,393	-3,0897	90,62	heating
0,992	-5,4729	-13,1957	107,95	heating	0,992	7,2353	-1,5763	123,17	heating	0,992	34,7516	10,625	337,25	cooling	0,992	3,552	-3,7283	101,77	heating
0,994	-5,244	-13,0538	109,17	heating	0,994	7,0535	-1,2894	116,62	heating	0,994	34,4398	6,8429	385,76	cooling	0,994	3,3969	-1,523	68,77	heating
0,996	-5,4618	-13,6325	114,21	heating	0,996	7,2798	0,2101	98,82	heating	0,996	34,9354	6,1517	402,35	cooling	0,996	3,3472	-2,2281	77,93	heating
0,998	-5,4499	-14,7944	130,62	heating	0,998	7,2386	1,0909	85,94	heating	0,998	35,2012	8,4945	373,32	cooling	0,998	3,3686	-3,8103	100,35	heating
1	-5,2995	-13,8994	120,21	heating	1	7,2595	-0,5218	108,77	heating	1	35,7694	5,9884	416,29	cooling	1	3,5713	-3,6203	100,53	heating
1,002	-5,2178	-13,6544	117,93	heating	1,002	7,4334	-1,2044	120,74	heating	1,002	35,3331	8,1748	379,63	cooling	1,002	3,471	-3,3308	95,08	heating
1,004	-5,0789	-13,1706	113,11	heating	1,004	7,5224	-0,2361	108,45	heating	1,004	34,8238	9,2258	357,82	cooling	1,004	3,8164	-3,8204	106,75	heating
1,006	-5,0073	-12,7349	108,02	heating	1,006	7,5055	-0,6823	114,45	heating	1,006	34,3068	7,9706	368,14	cooling	1,006	3,9029	-1,4943	75,45	heating
1,008	-4,772	-12,9807	114,74	heating	1,008	7,5859	0,4534	99,7	heating	1,008	34,7268	9,4869	352,82	cooling	1,008	3,4917	-3,628	99,52	heating
1,01	-4,7477	-13,6996	125,13	heating	1,01	7,4017	0,4921	96,59	heating	1,01	33,6715	6,5014	379,8	cooling	1,01	4,1455	-1,6465	80,96	heating
1,012	-4,6624	-13,659	125,76	heating	1,012	7,4556	-0,0731	105,24	heating	1,012	33,8818	4,9836	403,95	cooling	1,012	3,7312	-1,5448	73,75	heating
1,014	-4,7681	-14,0093	129,18	heating	1,014	7,6639	1,6494	84,07	heating	1,014	33,2842	6,151	379,28	cooling	1,014	3,7942	-2,9962	94,92	heating
1,016	-4,4628	-12,6032	113,79	heating	1,016	7,3115	0,3149	97,8	heating	1,016	32,9593	8,018	348,64	cooling	1,016	3,7124	-2,8449	91,66	heating
1,018	-4,7205	-12,2706	105,54	heating	1,018	7,5919	0,2591	102,5	heating	1,018	32,8974	10,0818	318,93	cooling	1,018	3,9028	-2,5182	89,76	heating
1,02	-4,487	-12,1335	106,89	heating	1,02	7,5298	1,0566	90,49	heating	1,02	33,9407	14,589	270,51	cooling	1,02	3,6418	-4,3366	111,53	heating
1,022	-4,4993	-12,1138	106,44	heating	1,022	7,4278	0,2817	99,89	heating	1,022	34,5045	13,5353	293,12	cooling	1,022	4,1447	-2,0817	87,04	heating
1,024	-4,2874	-12,3452	112,64	heating	1,024	7,6477	0,5149	99,71	heating	1,024	35,0352	14,5811	285,92	cooling	1,024	3,6612	-2,8639	91,21	heating
1,026	-4,2057	-12,2324	112,2	heating	1,026	7,6096	1,1184	90,74	heating	1,026	35,8119	17,3761	257,71	cooling	1,026	4,1973	-2,9131	99,39	heating
1,028	-3,942	-11,6482	107,72	heating	1,028	7,6358	0,1349	104,85	heating	1,028	35,8336	16,246	273,81	cooling	1,028	3,9298	-0,5831	63,08	heating
1,03	-3,9151	-11,8988	111,6	heating	1,03	7,7389	1,8519	82,29	heating	1,03	36,2241	13,3821	319,3	cooling	1,03	3,8336	-2,6237	90,26	heating
1,032	-3,7656	-11,2422	104,51	heating	1,032	7,504	0,961	91,46	heating	1,032	35,7536	9,9893	360,15	cooling	1,032	4,0528	-2,1895	87,26	heating
1,034	-3,7812	-10,5777	95	heating	1,034	7,6605	-0,7662	117,79	heating	1,034	35,8933	7,0866	402,68	cooling	1,034	3,9	-1,9436	81,68	heating
1,036	-3,8011	-11,4032	106,27	heating	1,036	7,8237	0,1214	107,67	heating	1,036	35,0194	6,0281	405,25	cooling	1,036	3,8729	-2,8839	94,45	heating
1,038	-3,7663	-11,7989	112,28	heating	1,038	7,7334	-0,5415	115,67	heating	1,038	34,2005	7,5925	371,94	cooling	1,038	4,1389	-2,3317	90,45	heating

1,04	-3,9451	-13,0465	127,22	heating	1,04	8,0147	0,544	104,43	heating	1,04	33,5585	5,9579	385,82	cooling	1,04	4,0498	-1,1801	73,11	heating
1,042	-3,7364	-12,2911	119,58	heating	1,042	7,8864	0,6229	101,53	heating	1,042	33,8428	5,9488	389,92	cooling	1,042	3,8182	-3,9415	108,47	heating
1,044	-3,7923	-11,558	108,55	heating	1,044	7,8714	-0,9399	123,17	heating	1,044	33,8949	6,3274	385,35	cooling	1,044	4,5326	-0,9117	76,1	heating
1,046	-3,6331	-11,5672	110,91	heating	1,046	8,2462	0,3452	110,45	heating	1,046	33,4503	4,9149	398,88	cooling	1,046	3,9491	-2,7407	93,51	heating
1,048	-3,4579	-12,5228	126,71	heating	1,048	8,024	1,248	94,72	heating	1,048	34,2471	6,4241	388,92	cooling	1,048	4,4179	-2,6861	99,3	heating
1,05	-3,1279	-12,1749	126,46	heating	1,05	8,1087	0,3128	108,97	heating	1,05	33,915	8,5613	354,41	cooling	1,05	4,1698	-1,4314	78,3	heating
1,052	-3,0736	-12,5472	132,43	heating	1,052	8,1625	1,7657	89,42	heating	1,052	34,1833	7,4826	373,24	cooling	1,052	4,3634	-1,7215	85,06	heating
1,054	-2,7976	-11,4637	121,14	heating	1,054	7,9005	0,4603	104	heating	1,054	34,1149	7,2124	376,06	cooling	1,054	4,2425	-2,2195	90,33	heating
1,056	-2,8391	-11,7087	123,98	heating	1,056	8,0185	0,4661	105,57	heating	1,056	35,0644	11,7514	325,88	cooling	1,056	4,4109	-1,2259	78,79	heating
1,058	-2,7601	-12,0449	129,79	heating	1,058	8,056	2,0385	84,12	heating	1,058	34,1546	8,938	352,49	cooling	1,058	4,0645	-3,4092	104,47	heating
1,06	-2,8244	-11,7569	124,86	heating	1,06	7,9487	0,4755	104,46	heating	1,06	34,2803	9,1684	351,03	cooling	1,06	4,558	-2,2815	95,61	heating
1,062	-2,9593	-11,8533	124,33	heating	1,062	8,1385	0,666	104,45	heating	1,062	34,1556	8,7377	355,3	cooling	1,062	4,1204	-0,8301	69,2	heating
1,064	-3,0693	-12,1986	127,61	heating	1,064	8,077	1,4917	92,05	heating	1,064	34,4021	7,3395	378,3	cooling	1,064	4,2902	-2,4085	93,64	heating
1,066	-3,1565	-12,1416	125,6	heating	1,066	7,9744	0,0852	110,28	heating	1,066	34,7791	8,9447	361,13	cooling	1,066	4,5827	-0,3739	69,29	heating
1,068	-3,1861	-12,3275	127,78	heating	1,068	8,3813	0,9256	104,22	heating	1,068	35,2595	14,5868	288,97	cooling	1,068	4,3469	-2,2758	92,57	heating
1,07	-2,9955	-12,5474	133,52	heating	1,07	8,0993	1,0069	99,14	heating	1,07	35,7391	14,275	300,04	cooling	1,07	4,6849	-2,3662	98,56	heating
1,072	-2,9842	-12,3541	130,98	heating	1,072	8,4241	0,7921	106,68	heating	1,072	35,5681	15,9989	273,55	cooling	1,072	4,8029	-1,9374	94,22	heating
1,074	-2,8146	-12,3866	133,8	heating	1,074	8,3298	2,2816	84,55	heating	1,074	35,5243	14,1253	299,13	cooling	1,074	4,6727	-1,7197	89,36	heating
1,076	-2,5312	-10,8841	116,76	heating	1,076	8,1481	0,9573	100,52	heating	1,076	35,2312	8,2438	377,24	cooling	1,076	4,6212	-2,357	97,54	heating
1,078	-2,3705	-10,3278	111,23	heating	1,078	8,2013	0,7018	104,83	heating	1,078	35,7725	7,153	400,06	cooling	1,078	4,9846	1,0922	54,41	heating
1,08	-2,4742	-10,8328	116,84	heating	1,08	8,2889	2,209	84,99	heating	1,08	34,6142	4,5449	420,32	cooling	1,08	4,5349	-1,3016	81,59	heating
1,082	-2,4158	-11,4971	126,94	heating	1,082	8,0456	1,8576	86,5	heating	1,082	34,6024	3,2187	438,7	cooling	1,082	4,999	-1,0682	84,81	heating
1,084	-2,5339	-11,6647	127,64	heating	1,084	8,1409	1,9968	85,88	heating	1,084	33,7504	3,4443	423,63	cooling	1,084	4,5728	-2,4984	98,85	heating
1,086	-2,6819	-11,5897	124,52	heating	1,086	8,0395	1,5179	91,16	heating	1,086	33,1274	2,0529	434,38	cooling	1,086	4,9717	-2,979	111,14	heating
1,088	-2,8656	-10,4093	105,45	heating	1,088	8,051	0,0614	111,68	heating	1,088	32,894	3,0923	416,58	cooling	1,088	4,7445	-1,2979	84,46	heating
1,09	-3,1222	-10,9234	109,05	heating	1,09	8,3978	2,0378	88,9	heating	1,09	32,8587	8,2529	343,95	cooling	1,09	4,8798	-0,3931	73,71	heating
1,092	-2,9875	-11,1766	114,47	heating	1,092	8,2177	1,9785	87,21	heating	1,092	32,6583	9,2263	327,54	cooling	1,092	4,6642	-1,4783	85,86	heating
1,094	-3,1789	-11,2697	113,1	heating	1,094	8,7047	2,1277	91,94	heating	1,094	32,787	8,5406	338,93	cooling	1,094	5,0635	-1,5867	92,96	heating
1,096	-3,0828	-11,5661	118,58	heating	1,096	8,5775	2,4945	85,03	heating	1,096	32,6945	3,4292	409,09	cooling	1,096	4,5771	-2,0702	92,92	heating
1,098	-3,0406	-12,0549	126,01	heating	1,098	8,7254	1,9118	95,24	heating	1,098	32,9131	-0,7262	470,23	cooling	1,098	4,9508	-2,2951	101,29	heating
1,1	-2,6704	-11,4444	122,65	heating	1,1	8,7977	1,7365	98,7	heating	1,1	33,4804	3,1974	423,31	cooling	1,1	4,8267	-0,4962	74,41	heating
1,102	-2,732	-11,9582	128,97	heating	1,102	8,9594	2,098	95,91	heating	1,102	33,3568	8,4421	348,27	cooling	1,102	4,8522	-1,7217	91,89	heating
1,104	-2,3098	-11,2788	125,37	heating	1,104	8,8284	1,193	106,73	heating	1,104	33,7781	13,4226	284,54	cooling	1,104	5,0188	-0,0065	70,25	heating
1,106	-2,3988	-11,6384	129,16	heating	1,106	9,0572	2,3792	93,35	heating	1,106	33,7621	13,8478	278,37	cooling	1,106	4,9854	-0,7426	80,07	heating
1,108	-2,2296	-12,0072	136,68	heating	1,108	8,8689	2,5561	88,24	heating	1,108	33,791	10,2166	329,54	cooling	1,108	5,0608	-0,9989	84,71	heating
1,11	-2,1924	-11,5271	130,48	heating	1,11	8,8549	1,6694	100,44	heating	1,11	34,0207	9,3553	344,79	cooling	1,11	4,9485	-2,1022	98,56	heating
1,112	-2,1154	-11,2619	127,85	heating	1,112	8,9416	2,2346	93,75	heating	1,112	33,614	9,1528	341,93	cooling	1,112	5,2313	-0,8471	84,97	heating
1,114	-2,1406	-11,5885	132,07	heating	1,114	8,8795	1,9349	97,08	heating	1,114	33,6109	8,9606	344,58	cooling	1,114	4,9374	-2,011	97,13	heating
1,116	-2,1729	-11,3446	128,21	heating	1,116	9,0781	3,0117	84,8	heating	1,116	33,4673	11,3528	309,13	cooling	1,116	5,2139	-0,5532	80,61	heating
1,118	-2,1265	-10,9895	123,89	heating	1,118	8,851	2,3313	91,14	heating	1,118	33,1431	10,9427	310,33	cooling	1,118	4,7264	-2,4712	100,61	heating
1,12	-1,9843	-10,7179	122,08	heating	1,12	9,0556	1,752	102,09	heating	1,12	33,0711	8,9553	337,1	cooling	1,12	5,4071	-1,4042	95,21	heating
1,122	-1,7402	-9,4742	108,11	heating	1,122	9,0758	1,698	103,13	heating	1,122	33,0561	7,9368	351,13	cooling	1,122	4,9743	-0,7713	80,31	heating
1,124	-1,5908	-10,3133	121,93	heating	1,124	9,2002	3,0537	85,92	heating	1,124	33,1849	7,7663	355,31	cooling	1,124	5,3977	-0,2471	78,91	heating
1,126	-0,9643	-9,6309	121,15	heating	1,126	9,1825	3,1246	84,68	heating	1,126	33,4196	9,4855	334,56	cooling	1,126	5,1351	-0,8526	83,7	heating
1,128	-0,546	-8,8351	115,87	heating	1,128	9,3307	3,5269	81,13	heating	1,128	33,4908	8,2528	352,79	cooling	1,128	5,457	-1,5779	98,34	heating
1,13	-0,1033	-7,9349	109,47	heating	1,13	9,1431	2,2209	96,76	heating	1,13	33,7259	6,1584	385,35	cooling	1,13	5,143	-2,0733	100,87	heating
1,132	0,1621	-8,217	117,13	heating	1,132	9,3348	3,0345	88,07	heating	1,132	33,7483	5,5982	393,5	cooling	1,132	5,5229	-1,7302	101,39	heating
1,134	0,2936	-8,0452	116,56	heating	1,134	9,1769	3,6998	76,56	heating	1,134	33,4564	5,033	397,32	cooling	1,134	5,3532	-1,1878	91,43	heating
1,136	0,0933	-8,7929	124,22	heating	1,136	9,2582	3,4497	81,19	heating	1,136	33,4839	6,7763	373,33	cooling	1,136	5,4945	-1,8909	103,24	heating
1,138	-0,0332	-7,8883	109,8	heating	1,138	9,304	3,67	78,75	heating	1,138	32,9342	6,9494	363,23	cooling	1,138	5,548	-0,5065	84,63	heating
1,14	-0,4626	-8,8364	117,05	heating	1,14	9,2761	3,3592	82,71	heating	1,14	32,7577	6,8052	362,78	cooling	1,14	5,5237	-2,1074	106,67	heating
1,142	-0,6133	-10,124	132,95	heating	1,142	9,2553	3,9876	73,63	heating	1,142	32,7134	9,8749	319,25	cooling	1,142	5,7369	-0,879	92,48	heating
1,144	-0,826	-10,6074	136,73	heating	1,144	9,196	3,6704	77,24	heating	1,144	32,4294	10,4243	307,6	cooling	1,144	5,4528	-2,288	108,21	heating
1,146	-0,8994	-10,7261	137,36	heating	1,146	9,2808	3,0179	87,55	heating	1,146	32,5979	7,9925	343,95	cooling	1,146	5,8927	-1,2538	99,9	heating
1,148	-1,0222	-10,4367	131,6	heating	1,148	9,4111	2,4721	97	heating	1,148	32,8575	6,658	366,23	cooling	1,148	5,6462	-1,8891	105,33	heating
1,15	-0,7991	-10,0798	129,73	heating	1,15	9,374	3,1936	86,39	heating	1,15	33,0097	6,4772	370,89	cooling	1,15	5,7331	-1,1851	96,71	heating

1,152	-0,719	-10,5726	137,74	heating	1,152	9,3399	2,4403	96,45	heating	1,152	33,3277	9,7515	329,56	cooling	1,152	5,3954	-3,0646	118,26	heating
1,154	-0,3247	-10,7447	145,66	heating	1,154	9,5197	2,7081	95,22	heating	1,154	33,3667	13,4362	278,6	cooling	1,154	5,8616	-1,7746	106,74	heating
1,156	-0,2494	-10,1998	139,09	heating	1,156	9,4832	1,6093	110,07	heating	1,156	33,4231	14,1127	269,93	cooling	1,156	5,3745	-3,0213	117,36	heating
1,158	-0,012	-10,725	149,75	heating	1,158	9,7006	2,579	99,55	heating	1,158	33,34	11,6542	303,14	cooling	1,158	5,8755	-1,6594	105,33	heating
1,16	0,1354	-9,6019	136,11	heating	1,16	9,667	2,8548	95,22	heating	1,16	32,8213	6,7243	364,8	cooling	1,16	5,511	-0,5814	85,16	heating
1,162	0,2229	-9,0888	130,16	heating	1,162	9,7765	2,0951	107,37	heating	1,162	32,3857	3,9438	397,58	cooling	1,162	5,6839	-1,7586	104,04	heating
1,164	0,2875	-8,318	120,29	heating	1,164	9,6483	2,0482	106,24	heating	1,164	31,7339	4,1623	385,41	cooling	1,164	5,5909	-1,796	103,26	heating
1,166	0,4188	-8,3488	122,56	heating	1,166	9,7095	2,9884	93,95	heating	1,166	31,4453	6,4513	349,38	cooling	1,166	5,8055	-2,2647	112,81	heating
1,168	0,5605	-8,3043	123,92	heating	1,168	9,6701	3,5742	85,21	heating	1,168	31,4282	10,6964	289,8	cooling	1,168	5,703	-2,7557	118,24	heating
1,17	0,7001	-8,7405	131,97	heating	1,17	9,8752	3,9539	82,77	heating	1,17	31,4801	11,1525	284,15	cooling	1,17	5,922	-2,4771	117,41	heating
1,172	0,9972	-7,6543	120,93	heating	1,172	9,5695	2,5361	98,32	heating	1,172	31,9256	6,4559	356,03	cooling	1,172	5,8184	-0,1541	83,49	heating
1,174	1,0369	-8,4423	132,51	heating	1,174	9,8127	1,7367	112,89	heating	1,174	32,8299	3,3241	412,45	cooling	1,174	5,8873	-1,1755	98,73	heating
1,176	1,4634	-7,893	130,79	heating	1,176	9,5495	2,4686	98,98	heating	1,176	33,4774	3,3308	421,4	cooling	1,176	6,0026	0,2847	79,93	heating
1,178	1,365	-7,9637	130,4	heating	1,178	9,5543	2,0461	104,95	heating	1,178	34,0447	6,3687	386,87	cooling	1,178	5,7365	-2,9296	121,14	heating
1,18	1,7308	-7,2574	125,64	heating	1,18	9,5348	2,5818	97,19	heating	1,18	34,2772	8,441	361,15	cooling	1,18	6,1639	-2,7714	124,9	heating
1,182	1,5898	-7,2142	123,07	heating	1,182	9,6255	2,0269	106,22	heating	1,182	34,1938	7,1502	378,03	cooling	1,182	5,9195	-2,9367	123,8	heating
1,184	1,8021	-7,7443	133,44	heating	1,184	9,6332	2,14	104,74	heating	1,184	34,2383	6,4324	388,69	cooling	1,184	6,0285	-1,8158	109,65	heating
1,186	1,7207	-9,1042	151,32	heating	1,186	9,6045	2,3315	101,67	heating	1,186	33,929	6,7477	379,95	cooling	1,186	5,8735	-1,7521	106,59	heating
1,188	2,1827	-9,0402	156,88	heating	1,188	9,6199	1,8274	108,93	heating	1,188	33,6513	6,5752	378,48	cooling	1,188	6,1646	-0,5854	94,36	heating
1,19	2,1766	-8,8434	154,04	heating	1,19	9,6895	0,8331	123,8	heating	1,19	33,3108	6,3576	376,77	cooling	1,19	5,7584	-2,6991	118,22	heating
1,192	2,6883	-8,0719	150,41	heating	1,192	9,9292	1,9435	111,63	heating	1,192	32,9548	5,1463	388,72	cooling	1,192	6,3618	-1,9943	116,81	heating
1,194	3,0269	-7,1572	142,36	heating	1,194	9,714	1,5333	114,35	heating	1,194	32,6958	3,2289	411,9	cooling	1,194	5,9177	-2,2236	113,8	heating
1,196	3,5376	-7,4721	153,9	heating	1,196	10,1109	2,6724	103,98	heating	1,196	32,655	3,0588	413,71	cooling	1,196	6,3011	-2,873	128,24	heating
1,198	3,8279	-7,4727	157,96	heating	1,198	9,8058	2,0887	107,87	heating	1,198	32,6065	3,7548	403,3	cooling	1,198	6,0824	-1,5394	106,54	heating
1,2	4,0003	-8,7936	178,84	heating	1,2	10,1003	2,5329	105,78	heating	1,2	32,7107	5,6056	378,89	cooling	1,2	6,2905	-2,3687	121,04	heating
1,202	4,4044	-8,5277	180,77	heating	1,202	9,9246	2,9918	96,91	heating	1,202	32,7009	7,8681	347,13	cooling	1,202	6,1775	-2,1221	116,02	heating
1,204	4,4106	-8,5155	180,69	heating	1,204	10,1069	2,4809	106,6	heating	1,204	32,6838	9,4233	325,15	cooling	1,204	6,3441	-2,361	121,68	heating
1,206	4,4525	-7,0887	161,33	heating	1,206	9,907	1,2036	121,66	heating	1,206	32,6492	9,6038	322,14	cooling	1,206	6,2361	-2,169	117,49	heating
1,208	4,2123	-7,231	159,96	heating	1,208	10,112	1,4224	121,47	heating	1,208	32,5727	8,0781	342,4	cooling	1,208	6,5316	-2,1881	121,89	heating
1,21	4,2754	-6,9805	157,34	heating	1,21	9,9739	2,1517	109,34	heating	1,21	32,5574	5,9384	372,09	cooling	1,21	6,4046	-1,1236	105,23	heating
1,212	3,8922	-8,5595	174,06	heating	1,212	10,1494	3,1658	97,62	heating	1,212	32,5943	5,3275	381,15	cooling	1,212	6,2205	-4,207	145,76	heating
1,214	4,0387	-7,702	164,12	heating	1,214	10,0967	3,13	97,38	heating	1,214	32,6055	6,0506	371,2	cooling	1,214	6,755	-1,6598	117,63	heating
1,216	3,8109	-8,1819	167,64	heating	1,216	10,1869	1,7613	117,78	heating	1,216	32,6541	8,414	338,84	cooling	1,216	6,2647	-1,4843	108,32	heating
1,218	4,0705	-7,8586	166,75	heating	1,218	10,2773	2,6692	106,35	heating	1,218	32,6743	10,6287	308,16	cooling	1,218	6,6931	0,7167	83,54	heating
1,22	3,8966	-8,6362	175,19	heating	1,22	10,0886	1,8646	114,96	heating	1,22	32,7646	12	290,26	cooling	1,22	6,3057	0,4943	81,23	heating
1,222	4,3008	-8,1935	174,65	heating	1,222	10,3895	2,57	109,3	heating	1,222	33,1226	13,3506	276,38	cooling	1,222	6,6799	-0,8367	105,07	heating
1,224	4,3779	-7,3247	163,59	heating	1,224	10,2172	1,4165	123,02	heating	1,224	33,155	11,2165	306,67	cooling	1,224	6,3178	-3,6077	138,74	heating
1,226	4,5747	-6,9543	161,16	heating	1,226	10,4192	2,1934	114,98	heating	1,226	33,4267	7,2586	365,79	cooling	1,226	6,8813	-2,126	125,91	heating
1,228	4,4325	-7,4258	165,76	heating	1,228	10,3193	2,4707	109,71	heating	1,228	33,7906	6,7379	378,16	cooling	1,228	6,2822	-3,2708	133,54	heating
1,23	4,5926	-8,4755	182,67	heating	1,23	10,482	2,8482	106,71	heating	1,23	34,01	8,7679	352,85	cooling	1,23	6,8774	-1,6411	119,08	heating
1,232	4,374	-8,9611	186,41	heating	1,232	10,3686	1,6722	121,56	heating	1,232	34,1716	11,2385	320,57	cooling	1,232	6,3333	-1,233	105,77	heating
1,234	4,3822	-9,2359	190,36	heating	1,234	10,6847	2,9577	108,01	heating	1,234	34,1875	13,1953	293,44	cooling	1,234	6,6708	-2,3015	125,42	heating
1,236	4,4026	-7,7435	169,78	heating	1,236	10,522	2,63	110,32	heating	1,236	33,9685	13,029	292,7	cooling	1,236	6,6557	-0,8651	105,13	heating
1,238	4,5706	-8,2711	179,51	heating	1,238	10,7945	2,7742	112,11	heating	1,238	33,8298	12,9511	291,85	cooling	1,238	6,5765	-2,1124	121,46	heating
1,24	4,865	-7,2334	169,12	heating	1,24	10,537	2,0868	118,12	heating	1,24	33,3302	9,9546	326,76	cooling	1,24	6,7984	-1,7596	119,63	heating
1,242	5,1782	-8,495	191,13	heating	1,242	10,7379	1,4045	130,47	heating	1,242	32,9106	4,3794	398,82	cooling	1,242	6,9545	-1,3118	115,55	heating
1,244	5,9366	-8,2648	198,51	heating	1,244	10,7841	3,3872	103,4	heating	1,244	32,8785	3,008	417,55	cooling	1,244	6,854	-1,5996	118,17	heating
1,246	6,4039	-7,4324	193,41	heating	1,246	10,6771	3,7326	97,07	heating	1,246	32,6628	5,2408	383,32	cooling	1,246	6,8824	-4,1581	154,33	heating
1,248	6,8269	-5,0786	166,42	heating	1,248	10,6129	3,5731	98,41	heating	1,248	32,643	7,48	351,74	cooling	1,248	7,3023	-1,4715	122,65	heating
1,25	6,7512	-4,6408	159,24	heating	1,25	10,7422	2,972	108,62	heating	1,25	32,8029	8,212	343,74	cooling	1,25	6,7731	-4,252	154,11	heating
1,252	6,7997	-4,7521	161,48	heating	1,252	10,5313	2,8808	106,94	heating	1,252	33,0723	5,9019	379,8	cooling	1,252	7,374	-0,8529	115	heating
1,254	6,1766	-7,5691	192,14	heating	1,254	10,668	2,6663	111,85	heating	1,254	33,7076	3,4078	423,55	cooling	1,254	6,8478	-0,5721	103,72	heating
1,256	6,3841	-7,648	196,15	heating	1,256	10,6862	3,6816	97,91	heating	1,256	34,2521	6,0644	394,02	cooling	1,256	7,1593	-0,7594	110,69	heating
1,258	6,0307	-6,63	176,98	heating	1,258	10,7156	1,9788	122,13	heating	1,258	34,3199	9,4591	347,52	cooling	1,258	6,9527	-1,1884	113,8	heating
1,26	6,4084	-6,5667	181,37	heating	1,26	10,8897	3,4153	104,48	heating	1,26	33,9312	9,9902	334,66	cooling	1,26	7,1545	-0,7949	111,12	heating
1,262	6,5356	-6,1702	177,61	heating	1,262	10,6907	3,3089	103,19	heating	1,262	33,4465	8,8752	343,47	cooling	1,262	6,9689	-2,2581	128,98	heating

1,264	7,1858	-6,2255	187,47	heating	1,264	10,9657	3,4279	105,37	heating	1,264	32,3704	5,1708	380,21	cooling	1,264	7,3218	-0,1867	104,96	heating
1,266	7,273	-6,2237	188,66	heating	1,266	10,8754	2,7244	113,94	heating	1,266	31,6801	2,6647	405,59	cooling	1,266	6,8967	-0,7585	107,01	heating
1,268	7,6569	-6,0591	191,73	heating	1,268	10,9542	1,9291	126,16	heating	1,268	30,9135	3,8974	377,65	cooling	1,268	7,4072	-1,1295	119,33	heating
1,27	7,4976	-6,5133	195,85	heating	1,27	10,8664	1,5006	130,92	heating	1,27	30,3322	6,9653	326,64	cooling	1,27	7,1906	1,0357	86,04	heating
1,272	7,5194	-8,281	220,87	heating	1,272	11,0825	2,7733	116,15	heating	1,272	30,1	9,3618	289,89	cooling	1,272	7,082	-1,6057	121,44	heating
1,274	7,3486	-7,603	209	heating	1,274	10,6108	1,096	133	heating	1,274	30,1073	10,3191	276,61	cooling	1,274	7,396	-0,7349	113,66	heating
1,276	7,1393	-8,7153	221,62	heating	1,276	11,1155	1,6229	132,69	heating	1,276	30,2506	7,0908	323,74	cooling	1,276	7,3049	-1,7454	126,51	heating
1,278	7,3502	-8,3228	219,09	heating	1,278	10,7462	2,3872	116,85	heating	1,278	30,3737	3,1335	380,78	cooling	1,278	7,1737	-2,1225	129,95	heating
1,28	7,1236	-8,3191	215,87	heating	1,28	10,903	1,941	125,28	heating	1,28	30,8605	1,8699	405,25	cooling	1,28	7,3597	-1,8901	129,3	heating
1,282	7,3595	-7,0245	201,07	heating	1,282	10,8745	2,9332	111,01	heating	1,282	31,043	3,3718	386,8	cooling	1,282	7,379	-0,0817	104,29	heating
1,284	7,1923	-6,369	189,57	heating	1,284	10,9928	1,7244	129,56	heating	1,284	31,5492	7,7042	333,32	cooling	1,284	7,25	-2,3602	134,34	heating
1,286	7,483	-5,5257	181,84	heating	1,286	11,0278	1,5158	132,96	heating	1,286	31,5528	10,5145	294,08	cooling	1,286	7,5927	-0,567	114,06	heating
1,288	7,0807	-7,3898	202,28	heating	1,288	11,1032	2,422	121,35	heating	1,288	31,7133	10,0816	302,38	cooling	1,288	7,2499	-2,9238	142,21	heating
1,29	7,607	-6,2246	193,34	heating	1,29	11,084	2,3635	121,9	heating	1,29	31,715	7,8528	333,56	cooling	1,29	7,6853	-2,5392	142,92	heating
1,292	7,1503	-6,1491	185,9	heating	1,292	11,2377	2,2222	126,02	heating	1,292	31,6635	7,559	336,95	cooling	1,292	7,2864	-1,6662	125,14	heating
1,294	7,5028	-5,5996	183,15	heating	1,294	11,2766	4,3367	97,01	heating	1,294	31,5909	7,6122	335,19	cooling	1,294	7,6408	-1,2897	124,84	heating
1,296	7,2029	-5,9498	183,86	heating	1,296	10,9829	2,6563	116,39	heating	1,296	31,4346	7,6254	332,82	cooling	1,296	7,4258	-1,397	123,33	heating
1,298	7,5942	-6,9794	203,72	heating	1,298	11,5262	4,2932	101,11	heating	1,298	31,6036	8,335	325,26	cooling	1,298	7,6664	-1,3188	125,6	heating
1,3	7,5896	-7,3462	208,78	heating	1,3	10,9652	3,0901	110,08	heating	1,3	31,3418	8,3688	321,13	cooling	1,3	7,3133	-2,2594	133,81	heating
1,302	7,6764	-7,1272	206,93	heating	1,302	11,2154	2,0678	127,87	heating	1,302	31,5753	9,4852	308,79	cooling	1,302	7,9739	-1,4691	132	heating
1,304	7,6911	-7,4582	211,77	heating	1,304	11,0892	2,7388	116,73	heating	1,304	31,2826	8,7656	314,75	cooling	1,304	7,4678	-0,5055	111,46	heating
1,306	7,7746	-8,4224	226,41	heating	1,306	11,2232	3,4296	108,94	heating	1,306	31,351	7,1577	338,19	cooling	1,306	7,7532	-1,373	127,57	heating
1,308	7,8177	-7,1701	209,51	heating	1,308	10,929	2,1729	122,4	heating	1,308	31,3458	6,2868	350,29	cooling	1,308	7,7128	-0,5097	114,94	heating
1,31	7,6663	-7,3138	209,4	heating	1,31	11,3375	3,5431	108,95	heating	1,31	31,2104	5,6236	357,66	cooling	1,31	7,845	-2,2263	140,78	heating
1,312	8,1441	-5,4236	189,66	heating	1,312	10,966	2,7325	115,09	heating	1,312	31,1068	2,9801	393,17	cooling	1,312	7,848	-1,3247	128,22	heating
1,314	7,7952	-6,7388	203,16	heating	1,314	11,3307	3,2647	112,75	heating	1,314	30,757	2,5774	393,91	cooling	1,314	7,815	-1,6235	131,94	heating
1,316	8,0589	-6,7752	207,36	heating	1,316	11,1684	4,6717	90,81	heating	1,316	30,5016	3,4296	378,43	cooling	1,316	8,0981	0,0054	113,12	heating
1,318	7,9374	-7,3532	213,74	heating	1,318	11,1663	2,6922	118,46	heating	1,318	30,137	5,7472	340,93	cooling	1,318	7,7824	-1,2712	126,56	heating
1,32	8,4086	-5,5431	195,02	heating	1,32	11,4526	3,8148	106,77	heating	1,32	30,2327	8,92	297,92	cooling	1,32	8,0721	-0,8098	124,15	heating
1,322	8,0818	-6,2357	200,14	heating	1,322	11,1967	3,8973	102,03	heating	1,322	29,63	7,2063	313,45	cooling	1,322	7,6877	-3,3233	153,92	heating
1,324	8,5285	-4,7432	185,52	heating	1,324	11,4098	3,5402	110,01	heating	1,324	29,9464	5,8578	336,72	cooling	1,324	8,2756	-2,017	143,88	heating
1,326	8,4078	-4,5971	181,79	heating	1,326	11,4486	3,4389	111,96	heating	1,326	29,676	3,3155	368,48	cooling	1,326	7,6252	-2,3346	139,22	heating
1,328	8,6468	-4,4298	182,79	heating	1,328	11,4458	4,5431	96,49	heating	1,328	29,7722	0,9491	402,9	cooling	1,328	8,2191	0,3713	109,7	heating
1,33	8,6114	-4,1681	178,64	heating	1,33	11,2372	2,5551	121,36	heating	1,33	29,926	1,2097	401,41	cooling	1,33	7,6485	0,0696	105,94	heating
1,332	8,6559	-5,4715	197,48	heating	1,332	11,6705	4,8171	95,8	heating	1,332	29,93	2,915	377,63	cooling	1,332	8,0528	-0,3338	117,23	heating
1,334	8,7052	-5,172	193,98	heating	1,334	11,1196	2,8775	115,21	heating	1,334	30,0844	3,3878	373,18	cooling	1,334	7,7398	-2,057	136,95	heating
1,336	8,4785	-4,5516	182,14	heating	1,336	11,4615	3,5365	110,78	heating	1,336	29,9286	6,6356	325,6	cooling	1,336	8,2422	-2,8074	154,46	heating
1,338	8,417	-3,7841	170,55	heating	1,338	11,2672	4,2979	97,42	heating	1,338	29,7382	6,2053	328,96	cooling	1,338	7,9074	-2,1962	141,23	heating
1,34	8,5142	-4,3404	179,69	heating	1,34	11,3886	4,1347	101,4	heating	1,34	29,577	3,641	362,55	cooling	1,34	8,0002	-1,9977	139,76	heating
1,342	8,706	-2,4845	156,43	heating	1,342	11,4126	4,4194	97,76	heating	1,342	29,9646	3,4465	370,68	cooling	1,342	8,0467	-1,3498	131,35	heating
1,344	8,6425	-3,9494	176,02	heating	1,344	11,4981	4,9978	90,86	heating	1,344	29,3135	0,5772	401,69	cooling	1,344	8,0576	-2,5567	148,37	heating
1,346	9,0459	-2,5152	161,61	heating	1,346	11,3858	4,1059	101,76	heating	1,346	29,7299	1,3138	397,22	cooling	1,346	8,11	-1,2581	130,95	heating
1,348	8,722	-4,0198	178,11	heating	1,348	11,6242	4,7232	96,47	heating	1,348	29,5644	4,9649	343,87	cooling	1,348	8,025	-1,6813	135,68	heating
1,35	8,8294	-3,9194	178,21	heating	1,35	11,4822	4,879	92,3	heating	1,35	29,591	6,8349	318,1	cooling	1,35	8,3556	0,7477	106,35	heating
1,352	8,8502	-2,5808	159,79	heating	1,352	11,7731	3,0962	121,29	heating	1,352	29,8472	9,2552	287,84	cooling	1,352	7,9554	-0,6365	120,1	heating
1,354	8,8522	-1,8504	149,61	heating	1,354	11,8058	4,851	97,22	heating	1,354	30,0592	11,628	257,64	cooling	1,354	8,3418	0,6048	108,15	heating
1,356	8,8707	-2,669	161,31	heating	1,356	11,6215	3,6424	111,54	heating	1,356	30,1627	8,676	300,35	cooling	1,356	8,0366	-1,5808	134,44	heating
1,358	9,0153	-2,8726	166,18	heating	1,358	11,8883	4,204	107,42	heating	1,358	30,2273	7,0108	324,53	cooling	1,358	8,5053	-1,1812	135,4	heating
1,36	8,7158	-4,5247	185,08	heating	1,36	11,8875	3,1736	121,81	heating	1,36	30,1542	4,1354	363,7	cooling	1,36	8,0849	-1,7524	137,51	heating
1,362	8,9995	-4,3602	186,75	heating	1,362	11,8358	2,7486	127,03	heating	1,362	30,0951	1,5327	399,26	cooling	1,362	8,5287	-0,7651	129,91	heating
1,364	8,9026	-2,642	161,38	heating	1,364	11,939	2,6157	130,33	heating	1,364	30,3509	3,4981	375,36	cooling	1,364	8,2468	-0,6073	123,77	heating
1,366	9,1074	-4,2907	187,29	heating	1,366	12,0682	4,0263	112,41	heating	1,366	29,8113	5,1799	344,31	cooling	1,366	8,372	-0,072	118,03	heating
1,368	9,2804	-3,2542	175,21	heating	1,368	11,7456	2,1036	134,78	heating	1,368	29,8563	5,6305	338,64	cooling	1,368	8,1738	-0,7536	124,79	heating
1,37	9,3344	-4,1742	188,83	heating	1,37	11,948	3,1254	123,33	heating	1,37	29,4878	6,7313	318,1	cooling	1,37	8,6519	-0,469	127,5	heating
1,372	9,4605	-5,1853	204,73	heating	1,372	11,5983	2,5276	126,8	heating	1,372	29,1845	6,4408	317,92	cooling	1,372	8,234	-0,0372	115,62	heating
1,374	9,7406	-5,6228	214,76	heating	1,374	11,9493	2,8631	127,01	heating	1,374	28,8491	5,0138	333,18	cooling	1,374	8,2698	-0,7543	126,14	heating

1,376	9,5783	-5,7685	214,53	heating	1,376	11,67	3,5726	113,19	heating	1,376	28,7045	4,6215	336,64	cooling	1,376	8,3993	0,8829	105,07	heating
1,378	9,7533	-6,2468	223,66	heating	1,378	11,9164	2,659	129,41	heating	1,378	28,4565	4,4795	335,16	cooling	1,378	8,2596	-1,5199	136,7	heating
1,38	9,8929	-3,6714	189,61	heating	1,38	11,9008	1,9929	138,5	heating	1,38	28,4774	6,5833	306,05	cooling	1,38	8,3726	-1,1822	133,56	heating
1,382	9,5596	-4,7205	199,61	heating	1,382	12,2323	2,8458	131,21	heating	1,382	28,4404	8,1503	283,63	cooling	1,382	8,4075	-1,3328	136,15	heating
1,384	9,9686	-4,1725	197,67	heating	1,384	11,9327	2,459	132,43	heating	1,384	28,499	8,4818	279,81	cooling	1,384	8,661	-0,2143	124,06	heating
1,386	9,7502	-3,8595	190,24	heating	1,386	12,4802	2,6643	137,21	heating	1,386	28,759	9,7942	265,1	cooling	1,386	8,5041	-0,2114	121,83	heating
1,388	10,1632	-3,5628	191,87	heating	1,388	12,1755	3,477	121,59	heating	1,388	28,9061	12,2424	232,93	cooling	1,388	8,6483	1,3549	101,95	heating
1,39	9,8854	-2,5081	173,24	heating	1,39	12,204	1,9759	142,97	heating	1,39	28,9961	12,9785	223,9	cooling	1,39	8,4606	-1,399	137,82	heating
1,392	10,3826	-0,8148	156,52	heating	1,392	12,3264	3,2622	126,7	heating	1,392	29,0046	11,2808	247,75	cooling	1,392	8,9363	-0,1818	127,46	heating
1,394	10,0487	-1,2241	157,58	heating	1,394	12,0988	1,7703	144,38	heating	1,394	28,9569	6,1399	318,95	cooling	1,394	8,2931	-1,8265	141,46	heating
1,396	10,7207	0,1795	147,35	heating	1,396	12,1336	1,5629	147,76	heating	1,396	28,8658	0,8967	390,97	cooling	1,396	8,8043	-1,3903	142,5	heating
1,398	10,053	-0,8213	152,01	heating	1,398	11,9843	1,7792	142,65	heating	1,398	29,0589	0,0992	404,81	cooling	1,398	8,4521	0,2571	114,56	heating
1,4	10,5545	-1,9384	174,63	heating	1,4	12,1175	2,164	139,14	heating	1,4	29,0418	1,2126	389,01	cooling	1,4	8,3605	0,1476	114,8	heating
1,402	10,0151	-1,6497	163,06	heating	1,402	11,8954	1,2332	149,04	heating	1,402	29,405	3,2	366,31	cooling	1,402	8,426	0,3724	112,58	heating
1,404	9,9714	-1,8585	165,36	heating	1,404	12,132	2,9356	128,55	heating	1,404	29,8857	5,7587	337,26	cooling	1,404	8,8634	0,4818	117,16	heating
1,406	9,8662	-0,9608	151,35	heating	1,406	11,7802	1,5326	143,25	heating	1,406	30,4388	6,3315	336,98	cooling	1,406	8,4876	-1,6181	141,26	heating
1,408	9,7885	-2,1805	167,31	heating	1,408	12,5299	3,5688	125,26	heating	1,408	31,1129	4,5505	371,3	cooling	1,408	8,8295	-1,1471	139,46	heating
1,41	9,7014	-3,8019	188,76	heating	1,41	11,9172	3,0779	123,56	heating	1,41	31,8703	4,1611	387,33	cooling	1,41	8,6911	0,5986	113,12	heating
1,412	9,5731	-4,9113	202,47	heating	1,412	12,3806	2,1107	143,56	heating	1,412	32,1485	4,4302	387,46	cooling	1,412	8,6834	-1,6053	143,82	heating
1,414	9,8644	-3,7868	190,82	heating	1,414	12,2216	3,1793	126,4	heating	1,414	32,4116	7,0988	353,83	cooling	1,414	8,8985	1,1504	108,31	heating
1,416	9,5917	-4,7226	200,09	heating	1,416	12,2477	3,2504	125,77	heating	1,416	32,3694	11,013	298,53	cooling	1,416	8,6433	-0,3827	126,17	heating
1,418	9,8943	-3,2159	183,26	heating	1,418	12,1338	3,1883	125,04	heating	1,418	31,8284	11,5388	283,62	cooling	1,418	9,0942	-0,1941	129,84	heating
1,42	9,3964	-5,5102	208,37	heating	1,42	12,4857	4,2372	115,3	heating	1,42	31,3559	10,3793	293,22	cooling	1,42	8,8628	0,4653	117,38	heating
1,422	9,8994	-4,4292	200,29	heating	1,422	12,0616	3,2421	123,28	heating	1,422	30,5989	9,3784	296,63	cooling	1,422	8,6983	0,4632	115,11	heating
1,424	9,1274	-5,6706	206,85	heating	1,424	12,2051	2,5742	134,62	heating	1,424	29,903	7,855	308,2	cooling	1,424	8,8724	-0,5199	131,29	heating
1,426	9,3403	-5,6845	210,02	heating	1,426	12,3484	4,7419	106,33	heating	1,426	29,2416	8,0768	295,85	cooling	1,426	9,1522	1,8989	101,39	heating
1,428	8,984	-6,2864	213,46	heating	1,428	12,0938	1,7376	144,76	heating	1,428	28,6302	8,1888	285,74	cooling	1,428	8,6549	-0,7771	131,85	heating
1,43	9,0697	-6,8391	222,38	heating	1,43	12,5326	3,4752	126,61	heating	1,43	28,1407	6,6085	300,99	cooling	1,43	9,4063	1,5648	109,61	heating
1,432	8,983	-7,5471	231,07	heating	1,432	12,0696	2,52	133,49	heating	1,432	28,2647	7,3172	292,81	cooling	1,432	8,8411	1,8068	98,33	heating
1,434	9,3224	-6,7689	224,93	heating	1,434	12,5492	1,9114	148,7	heating	1,434	28,2442	9,1742	266,57	cooling	1,434	9,0029	0,084	124,67	heating
1,436	9,2886	-7,6629	236,96	heating	1,436	12,3764	2,1718	142,65	heating	1,436	28,4256	10,6126	249	cooling	1,436	9,0934	1,1186	111,48	heating
1,438	9,6394	-6,998	232,57	heating	1,438	12,4341	2,6152	137,25	heating	1,438	28,5704	10,5374	252,07	cooling	1,438	9,2231	-0,4709	135,51	heating
1,44	9,8448	-5,56	215,34	heating	1,44	12,3306	0,4956	165,44	heating	1,44	28,4881	7,3496	295,48	cooling	1,44	9,0691	-1,4765	147,41	heating
1,442	10,1671	-7,6452	248,99	heating	1,442	12,8601	3,0152	137,62	heating	1,442	28,5086	2,2165	367,52	cooling	1,442	9,271	1,0931	114,31	heating
1,444	10,5067	-6,0684	231,7	heating	1,444	12,0693	0,4686	162,16	heating	1,444	28,4928	-1,2306	415,49	cooling	1,444	8,9955	1,9937	97,88	heating
1,446	10,2052	-7,2906	244,57	heating	1,446	12,731	1,2653	160,27	heating	1,446	28,5609	-0,8192	410,69	cooling	1,446	9,4255	2,0367	103,29	heating
1,448	10,6126	-5,5739	226,26	heating	1,448	12,5116	3,8321	121,33	heating	1,448	28,4405	2,7977	358,45	cooling	1,448	9,1674	3,4686	79,66	heating
1,45	10,545	-4,0823	204,47	heating	1,45	12,4537	2,442	139,95	heating	1,45	28,488	6,7447	303,94	cooling	1,45	9,1099	-0,6656	136,65	heating
1,452	10,1754	-3,6458	193,2	heating	1,452	12,7506	3,7895	125,26	heating	1,452	28,3566	6,202	309,69	cooling	1,452	9,535	0,2394	129,94	heating
1,454	10,2986	-3,9449	199,1	heating	1,454	12,8393	3,5071	130,45	heating	1,454	28,6306	2,9692	358,71	cooling	1,454	9,0854	-0,6922	136,68	heating
1,456	10,368	-1,4974	165,86	heating	1,456	12,7821	1,7316	154,47	heating	1,456	28,9302	1,0671	389,48	cooling	1,456	9,2356	-0,2039	131,95	heating
1,458	10,1012	-4,5111	204,26	heating	1,458	13,095	3,7664	130,4	heating	1,458	29,2694	2,465	374,69	cooling	1,458	9,4782	2,3017	100,32	heating
1,46	10,4027	-3,9231	200,25	heating	1,46	12,9123	5,1885	107,97	heating	1,46	29,6492	5,3044	340,3	cooling	1,46	9,383	3,2884	85,19	heating
1,462	10,1802	-5,2369	215,51	heating	1,462	13,0273	2,1532	152	heating	1,462	29,9481	9,2701	289,05	cooling	1,462	9,2245	1,7044	105,12	heating
1,464	10,5513	-6,1724	233,77	heating	1,464	13,2436	5,0353	114,74	heating	1,464	30,0781	11,1312	264,85	cooling	1,464	9,7496	3,6002	85,96	heating
1,466	10,5935	-3,7045	199,86	heating	1,466	12,6213	1,7944	151,34	heating	1,466	30,1525	11,7618	257,07	cooling	1,466	9,2252	0,9763	115,31	heating
1,468	10,3895	-4,1961	203,89	heating	1,468	13,2191	2,5547	149,07	heating	1,468	30,1992	11,5429	260,79	cooling	1,468	9,7079	1,229	118,52	heating
1,47	10,3475	-6,4089	234,23	heating	1,47	12,8274	3,2	134,58	heating	1,47	29,8563	8,7864	294,53	cooling	1,47	9,3681	2,6819	93,46	heating
1,472	10,6494	-5,804	229,99	heating	1,472	12,677	1,2751	159,38	heating	1,472	29,926	7,1269	318,7	cooling	1,472	9,6562	0,8848	122,61	heating
1,474	10,1373	-8,4583	259,94	heating	1,474	12,9171	1,1036	165,13	heating	1,474	29,6554	4,5979	350,27	cooling	1,474	9,417	1,1314	115,82	heating
1,476	10,7536	-6,8592	246,2	heating	1,476	13,1026	3,0342	140,74	heating	1,476	29,6204	1,2316	396,83	cooling	1,476	9,3448	0,978	116,96	heating
1,478	10,3856	-3,9897	200,95	heating	1,478	12,7024	0,0877	176,34	heating	1,478	29,6698	2,4624	380,32	cooling	1,478	9,4306	0,7873	120,82	heating
1,48	10,3367	-5,7893	225,42	heating	1,48	13,4622	2,6724	150,83	heating	1,48	29,2653	7,2446	307,82	cooling	1,48	9,7945	2,0509	108,24	heating
1,482	10,7476	-3,2473	195,63	heating	1,482	12,9014	2,3874	146,97	heating	1,482	29,0527	10,5948	258,01	cooling	1,482	9,335	2,3079	98,23	heating
1,484	10,5165	-2,9366	188,05	heating	1,484	13,385	0,8614	175,06	heating	1,484	28,8331	11,8864	236,89	cooling	1,484	9,5144	0,8163	121,59	heating
1,486	10,6722	-4,5455	212,72	heating	1,486	13,2673	3,2583	139,91	heating	1,486	28,499	6,6692	305,15	cooling	1,486	9,8128	3,0919	93,95	heating

1,488	10,9482	-1,7704	177,79	heating	1,488	13,3022	1,4749	165,33	heating	1,488	28,3644	0,8205	385,02	cooling	1,488	9,3249	0,4237	124,43	heating
1,49	10,636	-1,909	175,36	heating	1,49	13,2812	1,7574	161,09	heating	1,49	28,3678	0,4568	390,15	cooling	1,49	9,767	1,03	122,13	heating
1,492	10,6459	-4,2049	207,59	heating	1,492	13,2809	3,8836	131,36	heating	1,492	27,5338	-0,7317	395,11	cooling	1,492	9,6203	1,6245	111,77	heating
1,494	10,9187	-1,283	170,56	heating	1,494	12,8559	2,5186	144,5	heating	1,494	27,5343	1,0062	370,82	cooling	1,494	9,7967	1,7284	112,78	heating
1,496	10,2837	-3,4596	192,11	heating	1,496	13,3791	1,7088	163,13	heating	1,496	26,945	0,9955	362,74	cooling	1,496	9,5063	1,0571	118,11	heating
1,498	10,7968	-2,2531	182,42	heating	1,498	13,0244	3,1113	138,57	heating	1,498	26,5758	-1,7156	395,47	cooling	1,498	9,9071	1,1806	121,98	heating
1,5	10,4955	-0,3505	151,61	heating	1,5	12,9578	0,5896	172,89	heating	1,5	26,7402	1,3631	354,73	cooling	1,5	9,6458	-1,8669	160,93	heating
1,502	10,4306	-3,5713	195,73	heating	1,502	13,6944	4,8889	123,09	heating	1,502	26,2299	4,5066	303,66	cooling	1,502	10,0581	-0,2692	144,36	heating
1,504	10,7493	-4,2451	209,6	heating	1,504	13,1256	3,9309	128,53	heating	1,504	26,2734	3,6999	315,54	cooling	1,504	9,5924	0,6882	124,47	heating
1,506	10,8571	-3,2326	196,95	heating	1,506	13,5348	3,3131	142,88	heating	1,506	26,2118	5,3719	291,31	cooling	1,506	10,0757	1,5189	119,61	heating
1,508	11,0316	-4,9214	223	heating	1,508	13,4356	4,6116	123,35	heating	1,508	26,0417	2,5343	328,6	cooling	1,508	9,8059	3,1207	93,45	heating
1,51	11,655	-1,7507	187,39	heating	1,51	13,5426	4,3456	128,56	heating	1,51	26,5767	1,304	353,27	cooling	1,51	9,6163	0,7447	124,01	heating
1,512	11,2144	-2,1192	186,38	heating	1,512	13,3244	3,429	138,32	heating	1,512	27,5235	6,6393	291,93	cooling	1,512	9,9612	0,1597	137,01	heating
1,514	11,4807	-4,7729	227,2	heating	1,514	13,7516	5,7963	111,2	heating	1,514	27,5582	7,828	275,8	cooling	1,514	10,0828	0,3505	136,04	heating
1,516	11,9697	-2,3908	200,74	heating	1,516	13,2101	2,457	150,31	heating	1,516	28,9071	12,5481	228,68	cooling	1,516	9,7568	0,0486	135,71	heating
1,518	11,4952	-4,7361	226,89	heating	1,518	13,9506	3,1363	151,17	heating	1,518	29,2255	15,3342	194,18	cooling	1,518	9,9624	0,3077	134,96	heating
1,52	11,752	-5,0145	234,37	heating	1,52	13,3215	4,2183	127,25	heating	1,52	29,596	10,7673	263,2	cooling	1,52	10,0615	2,5407	105,13	heating
1,522	11,5616	-3,1127	205,13	heating	1,522	13,6833	3,3101	145	heating	1,522	30,1522	9,5843	287,51	cooling	1,522	9,6692	-0,1472	137,22	heating
1,524	11,3063	-4,6901	223,61	heating	1,524	13,5827	5,7965	108,84	heating	1,524	29,6494	9,1505	286,55	cooling	1,524	10,0919	1,4086	121,38	heating
1,526	11,4227	-4,1763	218,05	heating	1,526	13,1746	4,3655	123,14	heating	1,526	29,3072	5,7791	328,89	cooling	1,526	9,8944	0,484	131,54	heating
1,528	11,2555	-2,2339	188,56	heating	1,528	13,3768	2,9455	145,81	heating	1,528	28,9616	6,4931	314,08	cooling	1,528	10,1867	0,6914	132,73	heating
1,53	11,1	-5,7489	235,52	heating	1,53	13,4877	2,558	152,78	heating	1,53	28,2891	3,6065	345,03	cooling	1,53	9,9787	2,1392	109,58	heating
1,532	11,5799	-3,9388	216,93	heating	1,532	13,302	2,3925	152,5	heating	1,532	28,2257	2,1014	365,18	cooling	1,532	9,9911	1,7693	114,93	heating
1,534	11,0688	-4,9737	224,25	heating	1,534	13,4841	3,2021	143,73	heating	1,534	28,2805	5,1418	323,45	cooling	1,534	9,9495	-0,2979	143,24	heating
1,536	11,39	-6,1737	245,51	heating	1,536	14,0629	7,1877	96,11	heating	1,536	28,1397	5,6298	314,65	cooling	1,536	10,1154	0,6881	131,78	heating
1,538	11,6204	-3,3728	209,58	heating	1,538	13,635	3,787	137,66	heating	1,538	29,0089	7,3245	303,12	cooling	1,538	9,8931	-0,1743	140,73	heating
1,54	11,3466	-2,6395	195,5	heating	1,54	14,3817	5,7358	120,86	heating	1,54	29,2078	5,7969	327,25	cooling	1,54	10,6097	1,3515	129,41	heating
1,542	11,4506	-2,0802	189,14	heating	1,542	13,8015	4,8797	124,71	heating	1,542	29,9052	2,9533	376,75	cooling	1,542	10,1456	2,4124	108,1	heating
1,544	11,5989	0,605	153,68	heating	1,544	14,3798	5,2398	127,76	heating	1,544	30,496	4,7455	359,95	cooling	1,544	10,0838	-0,2066	143,84	heating
1,546	11,0595	-2,128	184,34	heating	1,546	13,9186	6,3417	105,91	heating	1,546	30,0288	2,9788	378,12	cooling	1,546	10,6046	1,7336	124	heating
1,548	11,3951	-2,7961	198,37	heating	1,548	13,8596	5,6995	114,07	heating	1,548	30,2639	2,3626	390,02	cooling	1,548	10,4151	0,8836	133,24	heating
1,55	11,3046	-0,4455	164,25	heating	1,55	13,678	3,1227	147,55	heating	1,55	30,0488	5,1521	348,02	cooling	1,55	10,3518	0,1954	141,97	heating
1,552	11,1882	-2,0291	184,76	heating	1,552	13,903	4,3548	133,47	heating	1,552	29,295	3,9107	354,83	cooling	1,552	10,6136	1,5248	127,05	heating
1,554	11,3384	-0,1795	161	heating	1,554	13,3633	3,1158	143,25	heating	1,554	29,0179	7,5081	300,68	cooling	1,554	10,453	0,7911	135,06	heating
1,556	11,0873	-0,7976	166,13	heating	1,556	14,061	4,7342	130,38	heating	1,556	28,2844	11,6745	232,18	cooling	1,556	10,4521	-1,3356	164,78	heating
1,558	11,2451	-2,6366	194,05	heating	1,558	13,8587	7,1401	93,92	heating	1,558	27,7691	9,1605	260,12	cooling	1,558	10,5887	1,3505	129,14	heating
1,56	11,5997	-2,2904	194,16	heating	1,56	13,6445	3,5013	141,79	heating	1,56	28,2586	9,4183	263,36	cooling	1,56	10,2976	-0,0503	144,65	heating
1,562	11,2889	-3,4915	206,61	heating	1,562	14,066	4,8443	128,91	heating	1,562	28,0582	6,802	297,13	cooling	1,562	10,7553	2,874	110,17	heating
1,564	11,651	-6,1639	249,03	heating	1,564	13,6618	3,0265	148,67	heating	1,564	28,6093	3,5674	350,05	cooling	1,564	10,2062	4,0752	85,7	heating
1,566	12,0408	-3,9702	223,81	heating	1,566	13,5366	2,9886	147,45	heating	1,566	29,01	6,85	309,76	cooling	1,566	10,4491	1,2261	128,92	heating
1,568	11,8792	-5,1101	237,48	heating	1,568	13,5049	5,4543	112,54	heating	1,568	28,7157	9,083	274,44	cooling	1,568	10,6079	0,4604	141,85	heating
1,57	12,3204	-4,2075	231,04	heating	1,57	13,5126	6,8445	93,21	heating	1,57	28,9888	9,6719	270,02	cooling	1,57	10,3231	-1,2384	161,61	heating
1,572	12,3301	-3,6812	223,81	heating	1,572	13,6513	3,5245	141,56	heating	1,572	28,8304	6,1184	317,48	cooling	1,572	10,4016	-2,531	180,78	heating
1,574	12,4236	-5,9889	257,38	heating	1,574	14,1429	5,5056	120,74	heating	1,574	28,6177	-1,8413	425,77	cooling	1,574	11,1022	1,6463	132,18	heating
1,576	12,5394	-6,3435	263,95	heating	1,576	13,9252	2,9859	152,91	heating	1,576	28,5883	-3,6148	450,15	cooling	1,576	10,4127	1,7501	121,09	heating
1,578	12,58	-5,7982	256,9	heating	1,578	14,7088	5,9942	121,82	heating	1,578	28,0351	-1,659	415,08	cooling	1,578	10,7871	1,5391	129,27	heating
1,58	12,2725	-7,3064	273,68	heating	1,58	14,2486	7,7089	91,41	heating	1,58	28,1261	2,7948	354,09	cooling	1,58	10,9223	3,7785	99,86	heating
1,582	12,6988	-4,673	242,83	heating	1,582	14,1358	5,6833	118,15	heating	1,582	28,347	7,8474	286,55	cooling	1,582	10,7242	-0,3512	154,82	heating
1,584	12,1341	-3,6546	220,7	heating	1,584	14,0012	5,1532	123,68	heating	1,584	28,1984	4,7962	327,13	cooling	1,584	10,9104	-0,8671	164,63	heating
1,586	12,4208	-4,8284	241,12	heating	1,586	13,6975	4,5047	128,5	heating	1,586	28,7952	3,4594	354,16	cooling	1,586	10,7721	-1,0679	165,51	heating
1,588	12,307	-4,3781	233,23	heating	1,588	13,3659	3,4383	138,77	heating	1,588	28,7147	5,364	326,41	cooling	1,588	10,8801	-2,0564	180,83	heating
1,59	12,3429	-6,6817	265,94	heating	1,59	13,9148	6,2867	106,63	heating	1,59	28,508	3,2484	353,09	cooling	1,59	10,9629	1,1475	137,2	heating
1,592	12,7637	-5,5345	255,78	heating	1,592	13,4028	5,9106	104,73	heating	1,592	29,0108	2,5336	370,11	cooling	1,592	10,6488	1,4475	128,62	heating
1,594	13,0728	-2,6765	220,15	heating	1,594	13,9052	3,8673	140,32	heating	1,594	29,05	3,1736	361,71	cooling	1,594	10,8498	-1,1732	168,06	heating
1,596	12,9398	-2,6172	217,46	heating	1,596	14,0356	6,4066	106,64	heating	1,596	28,7536	3,3498	355,11	cooling	1,596	11,2171	1,2314	139,59	heating
1,598	13,1189	-3,764	236	heating	1,598	13,9405	3,9433	139,75	heating	1,598	28,1409	5,6587	314,27	cooling	1,598	10,6201	-0,2231	151,57	heating

1,6	13,0051	-3,5752	231,77	heating	1,6	14,0169	5,1861	123,44	heating	1,6	27,2104	5,1749	308,02	cooling	1,6	11,2811	-0,0708	158,68	heating
1,602	12,521	-5,9857	258,7	heating	1,602	13,875	5,9606	110,63	heating	1,602	26,9744	4,9479	307,9	cooling	1,602	11,1374	1,056	140,92	heating
1,604	12,5672	-3,2721	221,41	heating	1,604	13,9961	5,2558	122,18	heating	1,604	26,8087	7,1148	275,29	cooling	1,604	10,9301	-1,7228	176,87	heating
1,606	11,6838	-4,0125	219,41	heating	1,606	14,2236	4,9001	130,33	heating	1,606	26,5053	7,198	269,89	cooling	1,606	11,3194	-0,3687	163,38	heating
1,608	11,8466	-4,922	234,4	heating	1,608	14,1777	5,2071	125,4	heating	1,608	26,7136	8,3284	257	cooling	1,608	11,3547	1,3029	140,51	heating
1,61	11,5198	-5,531	238,34	heating	1,61	14,1903	2,572	162,41	heating	1,61	26,4821	8,6484	249,29	cooling	1,61	11,1291	-0,6207	164,24	heating
1,612	11,7147	-6,3093	251,95	heating	1,612	14,8907	6,3277	119,7	heating	1,612	26,49	6,95	273,14	cooling	1,612	11,3616	0,4742	152,19	heating
1,614	11,754	-6,7316	258,4	heating	1,614	14,1082	5,3671	122,19	heating	1,614	26,563	6,4691	280,88	cooling	1,614	11,2928	0,1295	156,05	heating
1,616	12,2386	-4,7609	237,63	heating	1,616	14,6534	4,2583	145,31	heating	1,616	26,3399	3,8896	313,82	cooling	1,616	11,4336	-1,4497	180,09	heating
1,618	12,0492	-5,9847	252,09	heating	1,618	14,3571	5,0739	129,77	heating	1,618	26,3514	0,6066	359,87	cooling	1,618	11,4856	0,6336	151,7	heating
1,62	12,6703	-5,7451	257,42	heating	1,62	14,1737	2,8689	158,02	heating	1,62	26,6354	1,6244	349,62	cooling	1,62	11,1289	-0,6361	164,46	heating
1,622	12,2106	-7,1834	271,1	heating	1,622	14,0636	3,3968	149,11	heating	1,622	26,9125	3,5055	327,2	cooling	1,622	11,6638	0,8969	150,5	heating
1,624	12,3081	-9,9272	310,82	heating	1,624	14,41	5,4391	125,4	heating	1,624	27,5355	4,9562	315,63	cooling	1,624	11,2963	2,7953	118,83	heating
1,626	12,4364	-7,8747	283,92	heating	1,626	14,069	3,3015	150,51	heating	1,626	28,1264	4,8263	325,7	cooling	1,626	11,255	0,6281	148,55	heating
1,628	12,114	-8,7875	292,17	heating	1,628	14,7664	4,4198	144,63	heating	1,628	28,6043	3,4734	351,29	cooling	1,628	11,5015	-0,3469	165,62	heating
1,63	12,05	-8,4424	286,45	heating	1,63	14,7407	6,6914	112,52	heating	1,63	29,0215	5,1926	333,09	cooling	1,63	11,3967	-0,974	172,93	heating
1,632	11,8625	-9,0411	292,2	heating	1,632	15,0616	4,6341	145,76	heating	1,632	28,8742	7,2617	302,11	cooling	1,632	11,331	-1,3145	176,77	heating
1,634	11,8826	-10,529	313,28	heating	1,634	15,1681	7,445	107,96	heating	1,634	29,0516	7,2193	305,18	cooling	1,634	11,6543	2,0794	133,84	heating
1,636	12,0159	-11,0008	321,74	heating	1,636	14,5325	5,0072	133,15	heating	1,636	29,4986	8,0455	299,88	cooling	1,636	11,2707	1,3257	139,02	heating
1,638	12,0986	-8,1884	283,58	heating	1,638	14,9781	4,7969	142,32	heating	1,638	30,0411	10,096	278,8	cooling	1,638	11,3746	-0,2147	162	heating
1,64	11,9674	-9,6562	302,27	heating	1,64	14,6194	6,7828	109,54	heating	1,64	30,5439	11,5615	265,35	cooling	1,64	11,4776	0,5948	152,13	heating
1,642	12,5401	-6,231	262,39	heating	1,642	14,1461	6,4106	108,13	heating	1,642	31,2515	11,2775	279,21	cooling	1,642	11,5249	-0,3866	166,51	heating
1,644	11,9998	-5,8297	249,23	heating	1,644	14,0848	4,5235	133,65	heating	1,644	31,9779	9,1734	318,77	cooling	1,644	11,7198	0,6499	154,74	heating
1,646	12,2965	-4,9	240,38	heating	1,646	14,6017	7,0951	104,93	heating	1,646	32,9622	10,1788	318,48	cooling	1,646	11,3907	0,8891	146,8	heating
1,648	12,0689	-4,0583	225,43	heating	1,648	14,0416	2,7748	157,49	heating	1,648	33,3482	13,6856	274,85	cooling	1,648	11,6136	0,3108	158	heating
1,65	11,7782	-5,9959	248,46	heating	1,65	15,0462	4,3866	149,01	heating	1,65	33,2074	15,941	241,36	cooling	1,65	11,6733	1,1448	147,17	heating
1,652	11,4974	-7,2612	262,22	heating	1,652	14,7639	4,9695	136,91	heating	1,652	32,7391	15,5168	240,74	cooling	1,652	11,4606	0,7636	149,53	heating
1,654	11,5561	-5,7245	241,56	heating	1,654	15,095	3,466	162,56	heating	1,654	31,9693	11,3837	287,76		1,654	11,5517	-0,6828	171,02	heating
1,656	10,9765	-6,6127	245,87	heating	1,656	15,1547	6,1975	125,21	heating	1,656	31,4231	7,0729	340,38		1,656	11,7873	0,8008	153,58	heating
1,658	11,2103	-5,9931	240,48	heating	1,658	15,0728	6,289	122,79	heating	1,658	30,7599	6,652	336,99		1,658	11,4593	0,5891	151,95	heating
1,66	10,9778	-5,0469	224	heating	1,66	14,9865	3,4528	161,22	heating	1,66	30,0066	8,5651	299,72	cooling	1,66	11,7206	1,7742	139,04	heating
1,662	11,2971	-7,1574	257,97	heating	1,662	15,119	4,4135	149,65	heating	1,662	29,4644	10,4952	265,16		1,662	11,672	1,7263	139,03	heating
1,664	11,4136	-4,1432	217,46	heating	1,664	14,6523	4,4942	142	heating	1,664	29,2341	10,2432	265,46		1,664	11,5718	-1,2893	179,78	heating
1,666	11,0762	-5,394	230,23	heating	1,666	14,9619	3,7996	156,03	heating	1,666	29,0263	5,0388	335,31		1,666	11,8715	-0,5839	174,11	heating
1,668	11,4274	-4,9653	229,15	heating	1,668	14,8781	7,1636	107,84	heating	1,668	29,4087	1,5219	389,82		1,668	11,7484	0,2033	161,38	heating
1,67	11,4151	-4,1638	217,77	heating	1,67	14,472	3,1958	157,62	heating	1,67	29,5603	3,5179	364,03	cooling	1,67	11,8547	0,4393	159,57	heating
1,672	10,9769	-5,0216	223,63	heating	1,672	15,2166	5,4385	136,68	heating	1,672	29,4533	7,4017	308,25	cooling	1,672	11,8274	2,3381	132,65	heating
1,674	10,9386	-6,4017	242,39	heating	1,674	15,0104	6,8794	113,66	heating	1,674	29,3377	9,1214	282,59	cooling	1,674	11,8487	2,2454	134,24	heating
1,676	11,1033	-4,569	219,07	heating	1,676	14,8684	6,0217	123,66	heating	1,676	29,2845	7,981	297,79	cooling	1,676	12,0428	0,8162	156,93	heating
1,678	10,3724	-8,4441	263,03	heating	1,678	15,1503	6,0241	127,57	heating	1,678	28,9242	4,1731	345,98	cooling	1,678	12,1107	1,1292	153,5	heating
1,68	10,8861	-6,3933	241,54	heating	1,68	15,1809	6,5612	120,49	heating	1,68	28,53	3,6603	347,64	cooling	1,68	11,7301	-1,3495	182,83	heating
1,682	10,3112	-6,4892	234,84	heating	1,682	15,165	3,2445	166,63	heating	1,682	27,977	6,5365	299,71	cooling	1,682	12,2077	-0,8001	181,83	heating
1,684	10,9331	-7,602	259,09	heating	1,684	15,6299	6,179	132,11	heating	1,684	27,6197	8,8591	262,25	cooling	1,684	12,0911	0,7953	157,9	heating
1,686	11,1188	-5,3301	229,93	heating	1,686	15,0965	4,5982	146,75	heating	1,686	27,8636	10,2045	246,85	cooling	1,686	11,9572	0,0579	166,33	heating
1,688	11,6322	-4,9992	232,48	heating	1,688	15,5548	4,2986	157,35	heating	1,688	27,9733	6,9202	294,29		1,688	12,1616	1,1393	154,08	heating
1,69	11,9302	-6,4104	256,37	heating	1,69	15,2463	6,5604	121,42	heating	1,69	28,3917	1,2116	379,94		1,69	12,0422	0,5081	161,23	heating
1,692	12,5544	-5,2486	248,86	heating	1,692	15,2193	4,2529	153,29	heating	1,692	28,8648	2,0639	374,64	cooling	1,692	12,0296	-0,926	181,1	heating
1,694	12,3387	-7,2063	273,21	heating	1,694	15,3646	3,5249	165,5	heating	1,694	28,8353	5,9258	320,24	cooling	1,694	12,2293	0,5854	162,77	heating
1,696	13,1329	-6,6993	277,22	heating	1,696	15,4592	3,7428	163,78	heating	1,696	28,8528	7,4731	298,86	cooling	1,696	12,048	1,1631	152,16	heating
1,698	12,9975	-2,9572	223,02	heating	1,698	15,1612	3,1984	167,22	heating	1,698	29,3732	9,2214	281,69	cooling	1,698	12,1345	0,0548	168,86	heating
1,7	12,7802	-5,3003	252,74	heating	1,7	15,8041	5,8132	139,66	heating	1,7	28,9325	5,9832	320,8	cooling	1,7	12,2657	1,5706	149,5	heating
1,702	13,3365	-4,5496	250,02	heating	1,702	15,1406	6,9762	114,13	heating	1,702	28,9904	3,972	349,72	cooling	1,702	12,0724	-0,8509	180,65	heating
1,704	12,9835	-7,708	289,24	heating	1,704	15,3566	3,8906	160,28	heating	1,704	28,7106	5,9509	318,15	cooling	1,704	12,3715	-0,799	184,1	heating
1,706	13,0588	-10,4977	329,28	heating	1,706	15,5199	6,2552	129,51	heating	1,706	28,3022	4,6145	331,12	cooling	1,706	12,0598	0,2106	165,63	heating
1,708	13,0996	-10,3292	327,5	heating	1,708	15,2341	4,9054	144,38	heating	1,708	28,4465	4,0607	340,88	cooling	1,708	12,1085	0,5547	161,51	heating
1,71	13,2868	-7,7658	294,28	heating	1,71	15,099	4,981	141,43	heating	1,71	28,3557	5,992	312,61	cooling	1,71	12,3741	2,0332	144,55	heating

1,712	13,1561	-8,632	304,56	heating	1,712	14,9328	5,3467	134	heating	1,712	27,9213	3,9297	335,37	cooling	1,712	12,3067	2,7192	134,02	heating
1,714	13,7014	-4,6539	256,58	heating	1,714	14,8561	4,6131	143,18	heating	1,714	28,0974	5,9819	309,14	cooling	1,714	12,1433	0,3104	165,41	heating
1,716	13,2402	-8,8918	309,37	heating	1,716	14,9087	5,5651	130,61	heating	1,716	27,6458	6,1148	300,97	cooling	1,716	12,7013	1,3732	158,35	heating
1,718	14,0026	-10,1484	337,6	heating	1,718	15,0215	7,8985	99,57	heating	1,718	27,4441	1,557	361,86	cooling	1,718	12,4173	1,6581	150,4	heating
1,72	13,7529	-8,7659	314,78	heating	1,72	14,8403	5,5571	129,77	heating	1,72	27,8328	1,2915	371,01	cooling	1,72	12,6528	1,4888	156,06	heating
1,722	13,9553	-7,8085	304,23	heating	1,722	15,6887	5,6873	139,81	heating	1,722	26,9966	-0,4255	383,32	cooling	1,722	12,552	3,2492	130,04	heating
1,724	14,17	-4,6284	262,77	heating	1,724	15,3434	4,4861	151,77	heating	1,724	27,1141	-0,807	390,3	cooling	1,724	12,4379	1,4065	154,2	heating
1,726	14,0572	-3,7013	248,24	heating	1,726	16,2902	4,7753	160,96	heating	1,726	26,8465	4,1954	316,63	cooling	1,726	12,5176	0,8199	163,52	heating
1,728	13,7725	-6,7047	286,24	heating	1,728	15,9774	6,2062	136,59	heating	1,728	26,3981	3,0407	326,5	cooling	1,728	12,5594	1,5551	153,82	heating
1,73	14,3045	-6,8264	295,38	heating	1,73	16,1406	5,1791	153,23	heating	1,73	26,6558	1,2203	355,55	cooling	1,73	12,308	0,9943	158,15	heating
1,732	13,682	-6,4319	281,16	heating	1,732	15,8008	5,2357	147,69	heating	1,732	27,1922	5,4559	303,84	cooling	1,732	12,5014	1,4746	154,14	heating
1,734	13,8125	-8,9483	318,16	heating	1,734	16,0369	6,2236	137,18	heating	1,734	26,7606	6,4295	284,2	cooling	1,734	12,482	2,7181	136,49	heating
1,736	14,0697	-5,3796	271,87	heating	1,736	15,4281	4,8087	148,44	heating	1,736	27,1781	9,3292	249,5	cooling	1,736	12,4743	1,4652	153,89	heating
1,738	13,8738	-8,0146	305,97	heating	1,738	15,7143	4,7472	153,3	heating	1,738	26,7841	8,9138	249,8	cooling	1,738	12,8163	2,8075	139,91	heating
1,74	13,975	-7,8861	305,59	heating	1,74	15,5578	4,2453	158,13	heating	1,74	26,7366	2,0576	344,98	cooling	1,74	12,4275	1,0556	158,96	heating
1,742	14,0445	-6,6611	289,43	heating	1,742	15,8139	2,6522	183,98	heating	1,742	27,2873	0,2423	378,05	cooling	1,742	12,8244	-0,3498	184,16	heating
1,744	13,9806	-6,9404	292,44	heating	1,744	16,1422	5,1059	154,27	heating	1,744	27,213	3,0451	337,83	cooling	1,744	12,801	0,7705	168,17	heating
1,746	14,1848	-6,468	288,7	heating	1,746	15,7171	3,5424	170,18	heating	1,746	27,4463	7,1888	283,17	cooling	1,746	12,8316	1,2395	162,04	heating
1,748	14,0417	-2,4005	229,84	heating	1,748	16,4188	5,2668	155,89	heating	1,748	27,6087	12,4283	212,2	cooling	1,748	12,6143	0,4708	169,75	heating
1,75	13,6264	-5,0174	260,61	heating	1,75	15,8253	7,3716	118,17	heating	1,75	27,549	11,3698	226,16	cooling	1,75	13,1002	2,4225	149,26	heating
1,752	14,2903	-3,6023	250,11	heating	1,752	15,8607	7,3521	118,94	heating	1,752	27,9144	8,5346	270,9	cooling	1,752	12,6734	2,0105	149,05	heating
1,754	13,4676	-5,3416	262,93	heating	1,754	15,3521	5,264	141,02	heating	1,754	28,0422	7,843	282,35	cooling	1,754	13,1352	2,7977	144,5	heating
1,756	13,7907	-8,8398	316,34	heating	1,756	15,5328	4,1233	159,49	heating	1,756	27,6337	5,7065	306,51	cooling	1,756	12,7773	3,9969	122,74	heating
1,758	13,6918	-7,2314	292,48	heating	1,758	14,95	0,7707	198,21	heating	1,758	27,5008	5,0125	314,35	cooling	1,758	12,7634	2,0064	150,37	heating
1,76	13,433	-8,0757	300,66	heating	1,76	15,7415	4,5546	156,38	heating	1,76	27,0762	4,0303	322,15	cooling	1,76	12,8731	1,528	158,59	heating
1,762	13,1296	-9,6234	318,05	heating	1,762	15,1236	5,486	134,72	heating	1,762	26,8262	2,6271	338,27	cooling	1,762	12,9361	0,9926	166,95	heating
1,764	13,8654	-7,9189	304,51	heating	1,764	15,7366	5,0915	148,8	heating	1,764	26,6525	5,4422	296,49	cooling	1,764	12,974	0,5186	174,11	heating
1,766	12,9979	-9,8491	319,37	heating	1,766	16,0712	6,7678	130,05	heating	1,766	25,9794	7,9556	251,95	cooling	1,766	12,9515	1,2496	163,58	heating
1,768	13,6244	-11,375	349,45	heating	1,768	16,1155	4,7722	158,56	heating	1,768	25,7131	7,5819	253,45		1,768	13,053	2,5882	146,28	heating
1,77	13,5323	-8,8073	312,27	heating	1,77	16,5348	5,0704	160,25	heating	1,77	25,6372	4,7619	291,8		1,77	12,8624	1,0411	165,24	heating
1,772	13,8126	-10,0859	334,07	heating	1,772	16,1972	5,6894	146,88	heating	1,772	25,8946	-0,5862	370,16		1,772	13,3097	3,3389	139,38	heating
1,774	14,0399	-6,1597	282,36	heating	1,774	16,0832	4,2679	165,16	heating	1,774	26,5571	-3,3955	418,69		1,774	12,7266	1,0789	162,82	heating
1,776	13,8162	-5,1933	265,73	heating	1,776	16,0875	5,357	150	heating	1,776	27,1166	-1,3554	398	cooling	1,776	13,4303	0,8403	175,99	heating
1,778	14,2518	-5,204	271,96	heating	1,778	15,9326	7,3362	120,16	heating	1,778	27,1974	2,2029	349,39	cooling	1,778	13,1818	0,5193	177	heating
1,78	14,3345	-4,6906	265,94	heating	1,78	15,651	3,9988	162,88	heating	1,78	27,5493	7,9668	273,74	cooling	1,78	13,289	0,5472	178,11	heating
1,782	14,2232	-3,2351	244,04	heating	1,782	16,3135	7,814	118,81	heating	1,782	27,2451	8,6353	260,14	cooling	1,782	13,0691	0,089	181,44	heating
1,784	14,0048	-4,2128	254,65	heating	1,784	15,6716	6,8927	122,72	heating	1,784	27,1614	3,6168	329,12	cooling	1,784	13,4954	1,8972	162,12	heating
1,786	14,7406	-0,314	210,44	heating	1,786	16,0428	6,6696	131,02	heating	1,786	27,1002	2,4298	344,86	cooling	1,786	13,1066	0,7325	172,97	heating
1,788	13,74	-3,0777	235,09	heating	1,788	16,0446	7,7691	115,68	heating	1,788	26,4601	3,4767	321,27	cooling	1,788	13,4891	1,7459	164,15	heating
1,79	14,655	-3,1017	248,21	heating	1,79	15,784	4,972	151,13	heating	1,79	26,0458	5,25	290,69	cooling	1,79	13,281	3,5762	135,66	heating
1,792	14,1229	-4,582	261,47	heating	1,792	16,268	4,5474	163,84	heating	1,792	25,8481	5,9991	277,46	cooling	1,792	13,2699	1,8138	160,14	heating
1,794	14,6474	-6,6277	297,39	heating	1,794	16,4108	8,648	108,51	heating	1,794	25,8173	1,9204	334,04	cooling	1,794	13,4002	1,3285	168,74	heating
1,796	14,3462	-6,2966	288,56	heating	1,796	16,2353	7,2372	125,78	heating	1,796	26,3916	-1,641	391,85	cooling	1,796	13,4065	0,1587	185,18	heating
1,798	14,8906	-4,9885	277,88	heating	1,798	16,8806	8,1194	122,47	heating	1,798	26,9058	-0,2554	379,67	cooling	1,798	13,5203	0,1135	187,41	heating
1,8	14,6104	-5,8848	286,49	heating	1,8	16,9639	8,8951	112,79	heating	1,8	27,2225	3,3309	333,97	cooling	1,8	13,4793	0,7613	177,78	heating
1,802	14,9212	-5,3751	283,71	heating	1,802	17,0433	5,0487	167,67	heating	1,802	27,7197	8,9404	262,51	cooling	1,802	13,5445	3,2149	144,39	heating
1,804	14,6498	-5,4007	280,28	heating	1,804	17,2528	7,8497	131,44	heating	1,804	27,6072	9,9752	246,47	cooling	1,804	13,2906	1,859	159,8	heating
1,806	14,9551	-6,325	297,46	heating	1,806	16,6728	6,7682	138,45	heating	1,806	27,7804	5,658	309,24	cooling	1,806	13,7133	4,491	128,91	heating
1,808	14,7925	-3,7229	258,82	heating	1,808	16,6983	6,1079	148,04	heating	1,808	27,7064	2,5775	351,26	cooling	1,808	13,3117	2,8935	145,63	heating
1,81	14,2839	-6,0268	283,91	heating	1,81	16,9751	9,0932	110,18	heating	1,81	27,4409	0,4975	376,63	cooling	1,81	13,8089	2,4968	158,13	heating
1,812	14,8505	-4,4805	270,22	heating	1,812	16,3627	8,1063	115,41	heating	1,812	27,188	0,2227	376,93	cooling	1,812	13,7357	2,5457	156,42	heating
1,814	14,4765	-5,788	283,27	heating	1,814	17,1105	6,342	150,53	heating	1,814	27,3364	1,6187	359,49	cooling	1,814	13,8075	2,6075	156,56	heating
1,816	14,5266	-4,6042	267,42	heating	1,816	17,1959	8,9768	114,89	heating	1,816	27,4533	4,1868	325,23	cooling	1,816	13,65	2,2596	159,22	heating
1,818	14,3423	-4,6139	264,98	heating	1,818	17,0256	6,0792	153,01	heating	1,818	27,9608	7,0784	291,91	cooling	1,818	14,0299	4,3263	135,64	heating
1,82	14,8532	-3,0157	249,78	heating	1,82	17,3027	7,6779	134,54	heating	1,82	27,8467	7,5552	283,65	cooling	1,82	13,604	2,4683	155,66	heating
1,822	14,4964	-6,6725	295,91	heating	1,822	16,9749	9,1197	109,8	heating	1,822	28,0428	5,9349	309,03	cooling	1,822	14,0635	2,1918	165,95	heating

1,824	15,0416	-4,4987	273,14	heating	1,824	16,4541	4,8694	161,94	heating	1,824	28,2637	5,5265	317,83	cooling	1,824	13,657	3,9381	135,86	heating
1,826	14,6755	-6,0522	289,74	heating	1,826	16,54	6,2663	143,61	heating	1,826	28,4317	2,432	363,44	cooling	1,826	13,5433	2,4453	155,13	heating
1,828	15,2817	-4,6003	277,92	heating	1,828	15,9843	6,7746	128,74	heating	1,828	28,5707	0,3725	394,17	cooling	1,828	13,7837	3,8767	138,48	heating
1,83	14,93	-3,0225	250,95	heating	1,83	15,6029	4,801	150,99	heating	1,83	28,2819	2,7079	357,49	cooling	1,83	13,8744	3,1881	149,38	heating
1,832	15,1753	-4,7255	278,18	heating	1,832	16,1318	7,0635	126,76	heating	1,832	27,7996	5,2946	314,59	cooling	1,832	13,8613	3,1522	149,7	heating
1,834	15,2941	-5,3994	289,26	heating	1,834	15,6566	5,8779	136,69	heating	1,834	27,3351	6,8504	286,34	cooling	1,834	14,0897	4,7724	130,24	heating
1,836	15,2782	-6,5811	305,56	heating	1,836	16,5573	4,6165	166,91	heating	1,836	26,9768	7,2771	275,37	cooling	1,836	14,1025	5,5948	118,93	heating
1,838	15,0689	-7,9705	322,06	heating	1,838	16,647	7,6361	125,96	heating	1,838	26,3328	5,4145	292,41	cooling	1,838	14,0281	2,5183	160,89	heating
1,84	15,6261	-6,9741	315,92	heating	1,84	16,879	5,6001	157,66	heating	1,84	26,2926	5,7397	287,3	cooling	1,84	14,377	5,0815	129,94	heating
1,842	15,1979	-5,4322	288,38	heating	1,842	17,3386	6,5971	150,15	heating	1,842	25,7876	6,8321	264,97	cooling	1,842	13,776	2,7568	154,03	heating
1,844	15,3556	-8,1537	328,63	heating	1,844	17,4761	9,5249	111,15	heating	1,844	25,7081	6,9399	262,35	cooling	1,844	14,5089	3,8996	148,3	heating
1,846	16,04	-4,28	284,04	heating	1,846	17,0499	7,0886	139,24	heating	1,846	25,7103	8,184	244,99	cooling	1,846	13,8397	4,5456	129,92	heating
1,848	15,9609	-6,5802	315,09	heating	1,848	17,2411	7,0103	143,01	heating	1,848	25,5811	7,4803	253,02	cooling	1,848	14,014	3,5706	145,98	heating
1,85	16,3445	-4,3467	289,23	heating	1,85	16,6008	5,7434	151,77	heating	1,85	25,8064	8,155	246,74	cooling	1,85	13,9415	4,2588	135,35	heating
1,852	16,4087	-2,4803	264,04	heating	1,852	16,3192	3,627	177,42	heating	1,852	25,9637	10,6482	214,09		1,852	14,2996	5,7047	120,14	heating
1,854	16,7351	-1,6485	256,98	heating	1,854	16,6909	7,1793	132,96	heating	1,854	26,1458	11,3323	207,07		1,854	13,8428	3,3096	147,24	heating
1,856	16,6436	-2,9991	274,58	heating	1,856	16,0984	7,3938	121,68	heating	1,856	26,4401	11,3945	210,32		1,856	14,5443	3,3984	155,8	heating
1,858	16,6399	-1,3648	251,68	heating	1,858	16,7455	5,7707	153,41	heating	1,858	26,9627	10,9905	223,27		1,858	14,0034	3,1694	151,44	heating
1,86	16,0949	-4,162	283,16	heating	1,86	16,8533	6,806	140,45	heating	1,86	27,1963	7,7926	271,24		1,86	14,1708	2,6256	161,38	heating
1,862	16,5802	-1,7091	255,66	heating	1,862	17,232	5,717	160,96	heating	1,862	27,9094	8,0145	278,1		1,862	14,1866	5,583	120,26	heating
1,864	15,4905	-2,5586	252,3	heating	1,864	17,3322	6,0728	157,39	heating	1,864	27,5471	7,0253	286,86		1,864	14,1383	3,5331	148,25	heating
1,866	16,0162	-4,4968	286,74	heating	1,866	17,8154	8,7436	126,81	heating	1,866	27,8302	4,5744	325,08		1,866	14,3332	4,7435	134,05	heating
1,868	15,7018	-4,6567	284,58	heating	1,868	17,6013	7,0555	147,42	heating	1,868	27,8448	3,599	338,92		1,868	14,0233	3,6828	144,54	heating
1,87	15,7552	-7,8724	330,28	heating	1,87	18,1273	8,1897	138,91	heating	1,87	27,8354	2,7004	351,35		1,87	14,4822	3,3388	155,77	heating
1,872	15,6811	-8,2614	334,68	heating	1,872	17,6022	8,5133	127,05	heating	1,872	27,9001	3,1179	346,42		1,872	14,3667	2,6713	163,48	heating
1,874	16,3598	-6,6415	321,53	heating	1,874	17,559	5,6505	166,46	heating	1,874	28,1909	4,3408	333,39		1,874	14,5736	5,2972	129,67	heating
1,876	15,6832	-7,3544	322,03	heating	1,876	17,8109	6,3629	160,03	heating	1,876	28,3145	2,6639	358,56		1,876	14,225	4,1188	141,27	heating
1,878	16,1297	-7,245	326,74	heating	1,878	17,5006	6,8287	149,18	heating	1,878	28,7524	1,0185	387,68		1,878	15,0055	6,4711	119,3	heating
1,88	15,966	-4,6807	288,61	heating	1,88	17,7946	7,1215	149,19	heating	1,88	28,8913	1,5982	381,52		1,88	14,1927	2,8729	158,23	heating
1,882	16,0993	-7,0399	323,45	heating	1,882	17,8736	8,163	135,74	heating	1,882	28,8262	2,3651	369,89		1,882	14,7635	2,5133	171,24	heating
1,884	16,0053	-3,2947	269,79	heating	1,884	17,902	6,6289	157,58	heating	1,884	29,0552	5,6292	327,46	cooling	1,884	14,362	2,7304	162,59	heating
1,886	15,3612	-4,6949	280,35	heating	1,886	17,8311	5,0293	178,95	heating	1,886	28,6767	5,1232	329,24	cooling	1,886	14,6779	2,8433	165,43	heating
1,888	15,9942	-4,3083	283,8	heating	1,888	18,2188	7,5766	148,76	heating	1,888	28,8962	3,1846	359,41	cooling	1,888	14,5794	4,6181	139,25	heating
1,89	15,6306	-4,0366	274,92	heating	1,89	17,7683	6,7414	154,14	heating	1,89	28,8	4,3099	342,33	cooling	1,89	14,7085	3,9379	150,56	heating
1,892	15,4348	-5,1612	287,9	heating	1,892	18,1031	6,8148	157,79	heating	1,892	28,2793	5,8938	312,91	cooling	1,892	14,4567	1,4839	181,34	heating
1,894	15,2938	-8,0779	326,7	heating	1,894	17,8982	6,3313	161,69	heating	1,894	27,7309	6,8502	291,88	cooling	1,894	14,6367	1,7461	180,19	heating
1,896	16,1001	-5,9706	308,51	heating	1,896	18,1655	6,4794	163,35	heating	1,896	27,5349	8,2121	270,1	cooling	1,896	14,4257	3,1017	158,29	heating
1,898	15,1177	-9,4323	343,17	heating	1,898	18,1216	7,4241	149,53	heating	1,898	27,1246	4,4347	317,17	cooling	1,898	14,5626	2,4156	169,8	heating
1,9	16,3405	-5,542	305,89		1,9	18,1526	7,5064	148,82	heating	1,9	27,6046	4,328	325,37	cooling	1,9	14,5586	6,1964	116,89	heating
1,902	15,5501	-3,9476	272,55		1,902	18,4699	7,2787	156,44	heating	1,902	27,4841	6,383	294,96	cooling	1,902	14,0478	3,4211	148,54	heating
1,904	16,0424	-6,2033	310,96		1,904	18,4963	7,8919	148,23	heating	1,904	27,2351	6,3506	291,93	cooling	1,904	14,7103	3,4702	157,12	heating
1,906	15,8644	-4,9887	291,5		1,906	18,3345	7,8277	146,87	heating	1,906	27,3129	7,3039	279,7	cooling	1,906	14,49	0,657	193,36	heating
1,908	16,0293	-6,3988	313,51		1,908	18,2422	7,0949	155,82	heating	1,908	26,7784	4,8495	306,53	cooling	1,908	14,8026	0,3546	201,96	heating
1,91	15,8627	-8,3879	338,99		1,91	18,353	7,5074	151,61	heating	1,91	26,736	2,5388	338,24	cooling	1,91	14,9695	2,5695	173,33	heating
1,912	16,1592	-5,3519	300,69		1,912	18,1764	6,5923	161,93	heating	1,912	26,3101	3,7218	315,75	cooling	1,912	15,3398	6,9551	117,21	heating
1,914	15,7863	-4,2395	279,93		1,914	18,3395	8,4629	138,06	heating	1,914	25,7271	3,1989	314,91	cooling	1,914	14,7793	4,9555	137,32	heating
1,916	16,263	-4,9293	296,24		1,916	18,2578	8,0623	142,52	heating	1,916	25,6169	1,9638	330,63	cooling	1,916	15,3767	5,5245	137,72	heating
1,918	16,2086	-1,6922	250,23		1,918	18,3586	7,4311	152,75	heating	1,918	25,905	3,0517	319,46	cooling	1,918	14,6792	2,5982	168,87	heating
1,92	15,4727	-8,7101	338,04		1,92	18,3109	6,8917	159,62	heating	1,92	25,8428	-1,4093	380,94	cooling	1,92	15,1087	2,6807	173,73	heating
1,922	16,7338	-8,1478	347,81		1,922	18,3202	6,7766	161,36	heating	1,922	26,6842	-0,5948	381,32	cooling	1,922	14,6432	6,4488	114,55	heating
1,924	16,0788	-8,8308	348,2		1,924	18,4057	6,5316	165,98	heating	1,924	26,797	1,1732	358,18	cooling	1,924	14,7554	5,0498	135,67	heating
1,926	16,2645	-8,9901	353,02		1,926	18,2833	6,8005	160,51	heating	1,926	26,9557	1,695	353,11	cooling	1,926	14,6813	4,0401	148,75	heating
1,928	16,2027	-6,3775	315,64		1,928	18,3091	6,7398	161,72	heating	1,928	27,4586	5,4726	307,33	cooling	1,928	14,8525	3,6903	156,03	heating
1,93	16,3726	-4,1234	286,5		1,93	18,432	7,1776	157,32	heating	1,93	27,1564	6,7157	285,73	cooling	1,93	14,8386	3,2319	162,25	heating
1,932	15,7619	-8,5415	339,73		1,932	18,4936	6,756	164,07	heating	1,932	27,5535	5,1278	313,48	cooling	1,932	15,3559	2,6099	178,17	heating
1,934	16,5919	-4,4326	293,89		1,934	18,8221	5,2389	189,87	heating	1,934	27,7099	8,1557	273,34	cooling	1,934	15,0504	4,0953	153,14	heating

1,936	15,438	-7,3168	318,08	1,936	19,0863	4,5136	203,71	heating	1,936	27,4656	8,2362	268,8	cooling	1,936	14,8445	0,9647	194,02	heating
1,938	16,1277	-7,5252	330,63	1,938	19,0001	3,7468	213,22	heating	1,938	27,534	6,5101	293,88	cooling	1,938	15,6095	4,4045	156,63	heating
1,94	15,62	-4,4699	280,83	1,94	18,764	6,1624	176,15	heating	1,94	28,0497	9,2583	262,68	cooling	1,94	14,8714	2,7631	169,26	heating
1,942	15,5988	-7,2985	320,07	1,942	18,5153	6,9716	161,36	heating	1,942	27,6521	7,6474	279,64	cooling	1,942	15,3037	3,6425	163,01	heating
1,944	15,9568	-4,6612	288,21	1,944	18,1519	5,3506	178,94	heating	1,944	27,9189	6,5888	298,16	cooling	1,944	15,0156	3,6153	159,36	heating
1,946	15,6657	-2,6762	256,39	1,946	18,2329	4,99	185,12	heating	1,946	27,4773	7,1651	283,93	cooling	1,946	15,5142	3,1896	172,28	heating
1,948	15,7451	-4,0221	276,32	1,948	17,8585	4,6454	184,7	heating	1,948	26,8056	5,4166	298,99	cooling	1,948	15,1425	2,0459	183,07	heating
1,95	16,4076	-2,7299	267,51	1,95	18,2495	4,5488	191,52	heating	1,95	26,8005	4,862	306,67	cooling	1,95	15,731	5,0528	149,27	heating
1,952	15,7853	-2,5335	256,07	1,952	18,2233	5,7992	173,67	heating	1,952	26,559	5,4152	295,56		1,952	15,08	2,2141	179,85	heating
1,954	15,7486	-7,6772	327,46	1,954	18,3735	5,4138	181,16	heating	1,954	26,5355	4,2198	311,94		1,954	15,7632	2,5589	184,58	heating
1,956	16,701	-2,5579	269,21	1,956	18,8952	5,7128	184,27	heating	1,956	26,5087	8,4934	251,83		1,956	15,103	3,3133	164,8	heating
1,958	15,8552	-6,624	314,23	1,958	19,1102	8,0488	154,62	heating	1,958	26,0146	9,6842	228,27		1,958	15,5441	1,1774	200,82	heating
1,96	16,3243	-6,1543	314,22	1,96	18,9884	7,3387	162,84	heating	1,96	25,9235	7,9435	251,33		1,96	15,3885	2,7941	176,05	heating
1,962	15,9606	-6,0997	308,37	1,962	19,0237	7,0755	167,02	heating	1,962	26,1754	6,2235	278,9		1,962	15,0883	2,0996	181,56	heating
1,964	16,1196	-8,4504	343,45	1,964	18,9132	6,9261	167,56	heating	1,964	26,0582	2,4637	329,82		1,964	15,2908	3,4258	165,86	heating
1,966	16,1495	-8,2065	340,46	1,966	18,3486	4,533	193,12	heating	1,966	26,3117	1,0463	353,17		1,966	15,5741	4,8172	150,37	heating
1,968	16,2032	-4,109	283,93	1,968	18,6173	3,4926	211,42	heating	1,968	26,3039	3,0144	325,55		1,968	15,2097	4,6887	147,07	heating
1,97	15,9226	-7,333	325,08	1,97	18,5803	3,0969	216,44	heating	1,97	25,9511	2,1123	333,23		1,97	15,2596	1,4568	192,94	heating
1,972	16,808	-4,1608	293,11	1,972	18,8211	3,1568	218,96	heating	1,972	26,3488	2,2108	337,41		1,972	15,9513	4,4716	160,47	heating
1,974	15,7687	-6,0258	304,65	1,974	18,8263	6,0909	178,02	heating	1,974	26,2407	2,0703	337,87		1,974	15,3952	0,6804	205,69	heating
1,976	16,4233	-8,8823	353,73	1,976	18,9434	6,9074	168,25	heating	1,976	26,6076	0,0554	371,16		1,976	15,827	2,4225	187,38	heating
1,978	16,4338	-4,6707	295,01	1,978	19,0962	5,1408	195,08	heating	1,978	26,6659	1,2872	354,76	cooling	1,978	15,3591	2,0834	185,57	heating
1,98	16,1149	-5,3226	299,66	1,98	19,1895	4,6895	202,69	heating	1,98	26,7172	1,115	357,88	cooling	1,98	15,9186	2,6755	185,12	heating
1,982	16,2233	-4,2911	286,76	1,982	19,0234	3,9205	211,12	heating	1,982	27,0377	1,0191	363,7	cooling	1,982	15,5993	3,7186	166,07	heating
1,984	16,79	-2,0523	263,39	1,984	19,1851	4,579	204,17	heating	1,984	27,9112	3,6271	339,45	cooling	1,984	15,8453	4,7749	154,75	heating
1,986	16,047	-4,8711	292,4	1,986	19,0786	6,1227	181,1	heating	1,986	28,0494	3,6361	341,26	cooling	1,986	15,5142	0,7762	206,02	heating
1,988	16,5106	-6,6847	324,24	1,988	18,8245	6,1261	177,5	heating	1,988	28,5041	2,2419	367,11	cooling	1,988	16,1436	1,3234	207,16	heating
1,99	16,4426	-3,3877	277,2	1,99	19,4107	7,5009	166,48	heating	1,99	28,6135	2,8184	360,58	cooling	1,99	15,4252	0,8941	203,12	heating
1,992	16,3365	-6,4223	318,13	1,992	19,4646	9,1263	144,51	heating	1,992	28,3346	1,4499	375,81	cooling	1,992	16,1193	1,9998	197,37	heating
1,994	16,4978	-2,7154	268,57	1,994	19,5682	8,1692	159,34	heating	1,994	28,5478	2,4065	365,42	cooling	1,994	15,82	4,3407	160,46	heating
1,996	15,8641	-3,796	274,82	1,996	19,8321	7,9797	165,68	heating	1,996	28,2589	4,5684	331,16	cooling	1,996	15,7055	1,4184	199,71	heating
1,998	16,4109	-5,9208	312,16	1,998	19,9093	7,8464	168,62	heating	1,998	28,5076	8,3049	282,4	cooling	1,998	15,952	3,0298	180,63	heating
2	16,3222	-6,4115	317,78	2	19,7333	6,8446	180,16	heating	2	28,0284	10,9493	238,74	cooling	2	16,1024	3,5357	175,66	heating
2,002	16,2367	-6,2797	314,75	2,002	19,7745	7,2612	174,92	heating	2,002	28,0657	9,4844	259,74	cooling	2,002	15,718	2,1658	189,44	heating
2,004	16,0915	-7,291	326,85	2,004	19,884	7,0585	179,28	heating	2,004	28,2339	6,4926	303,91	cooling	2,004	15,9625	1,0671	208,22	heating
2,006	16,7099	-3,1554	277,69	2,006	19,54	5,1003	201,85	heating	2,006	29,0046	6,6175	312,94	cooling	2,006	16,2314	3,7785	174,07	heating
2,008	15,5492	-6,5603	309,06	2,008	19,6455	6,3809	185,42	heating	2,008	28,9989	6,1196	319,82	cooling	2,008	16,1471	2,0561	196,97	heating
2,01	16,4546	-6,8696	326,04	2,01	19,4236	6,4073	181,95	heating	2,01	29,5437	9,2978	283,01	cooling	2,01	16,4158	6,5888	137,37	heating
2,012	15,8465	-6,0002	305,38	2,012	19,8515	5,6605	198,37	heating	2,012	29,2419	8,5288	289,54	cooling	2,012	15,72	4,0733	162,8	heating
2,014	15,9793	-7,842	332,99	2,014	19,8023	5,357	201,92	heating	2,014	29,2192	3,1624	364,24	cooling	2,014	16,5347	3,5386	181,67	heating
2,016	15,8368	-4,9928	291,17	2,016	20,0561	5,963	197	heating	2,016	29,3693	0,6929	400,85	cooling	2,016	15,987	3,6244	172,81	heating
2,018	15,9851	-3,019	265,65	2,018	20,2578	5,8198	201,82	heating	2,018	28,6219	-0,4408	406,25	cooling	2,018	16,1514	4,3907	164,4	heating
2,02	15,591	-4,0886	275,09	2,02	20,1951	6,8687	186,28	heating	2,02	28,3606	2,0254	368,13	cooling	2,02	15,8567	2,9491	180,43	heating
2,022	15,8132	-3,1812	265,51	2,022	19,9365	6,794	183,71	heating	2,022	27,6811	4,8073	319,74	cooling	2,022	16,4055	4,4417	167,24	heating
2,024	15,7157	-2,7379	257,95	2,024	20,0041	7,2006	178,97	heating	2,024	27,6477	5,8578	304,59	cooling	2,024	15,6459	2,9715	177,17	heating
2,026	16,0916	-5,6236	303,55	2,026	20,097	7,8998	170,5	heating	2,026	27,7809	6,4515	298,15	cooling	2,026	16,663	4,4306	170,99	heating
2,028	16,454	-1,5593	251,8	2,028	19,6961	7,18	174,96	heating	2,028	28,1341	8,9316	268,42	cooling	2,028	16,0639	4,1177	166,99	heating
2,03	15,9843	-3,6518	274,48	2,03	20,0872	6,964	183,44	heating	2,03	28,1905	8,2984	278,06	cooling	2,03	16,3855	0,7206	218,97	heating
2,032	17,2387	-1,072	255,96	2,032	19,7943	6,6772	183,36	heating	2,032	28,4392	10,4815	251,02	cooling	2,032	16,5511	2,7674	192,68	heating
2,034	16,8859	-1,2517	253,54	2,034	20,0267	8,1029	166,68	heating	2,034	28,4009	10,8959	244,69	cooling	2,034	16,7704	2,3964	200,93	heating
2,036	17,195	-3,0053	282,37	2,036	19,942	9,2066	150,07	heating	2,036	28,7057	11,4306	241,48	cooling	2,036	16,5308	3,224	186,01	heating
2,038	17,1877	-5,7333	320,4	2,038	20,727	10,3919	144,47	heating	2,038	29,2517	14,1807	210,67	cooling	2,038	16,4773	2,7682	191,63	heating
2,04	17,775	-4,3174	308,82	2,04	20,3821	8,199	170,3	heating	2,04	28,9464	14,7995	197,75		2,04	16,784	3,4326	186,63	heating
2,042	17,078	-6,8804	334,9	2,042	20,6009	8,101	174,73	heating	2,042	29,0605	11,6807	242,94		2,042	16,6922	1,9616	205,91	heating
2,044	17,8878	-2,931	291,02	2,044	20,4817	7,8203	176,99	heating	2,044	29,1063	7,7698	298,25		2,044	16,7982	5,0486	164,24	heating
2,046	17,2522	-2,8288	280,7	2,046	20,6311	8,2282	173,37	heating	2,046	29,3277	3,8014	356,82		2,046	16,1838	0,6752	216,79	heating

2,048	17,8523	-2,9702	291,07		2,048	20,4591	8,3509	169,26	heating	2,048	29,4678	2,0168	383,72		2,048	17,3034	2,2552	210,35	heating
2,05	17,4829	-2,2622	276,01		2,05	20,4495	8,9204	161,16	heating	2,05	29,997	8,3805	302,17		2,05	16,3157	1,4517	207,78	heating
2,052	17,5448	-4,066	302,09		2,052	20,6351	8,0826	175,47	heating	2,052	29,7869	10,4878	269,77		2,052	16,9484	2,9522	195,65	heating
2,054	17,6652	-3,1945	291,59		2,054	20,3139	6,6655	190,78	heating	2,054	29,7768	9,9667	276,92		2,054	16,5587	3,4508	183,23	heating
2,056	17,6314	-2,0085	274,54		2,056	20,4546	6,9205	189,19	heating	2,056	29,854	5,3553	342,46		2,056	16,9528	4,1062	179,58	heating
2,058	17,6024	-1,8253	271,57		2,058	20,3537	6,607	192,16	heating	2,058	29,7518	0,663	406,62		2,058	16,3269	3,5206	179,01	heating
2,06	17,9691	-1,5733	273,17		2,06	20,6467	8,5317	169,35	heating	2,06	30,2635	2,6798	385,58		2,06	17,1455	6,7812	144,88	heating
2,062	17,6625	-0,4325	252,94		2,062	20,0574	8,1103	167	heating	2,062	29,6296	5,4716	337,69	cooling	2,062	16,4781	5,2669	156,72	heating
2,064	17,4881	-4,2041	303,22	heating	2,064	20,3544	6,9742	187,04	heating	2,064	29,5377	4,2734	353,16	cooling	2,064	16,8207	2,4897	200,33	heating
2,066	18,1282	-3,0378	295,87	heating	2,066	20,3394	5,8319	202,79	heating	2,066	29,5233	3,0475	370,09	cooling	2,066	16,7741	2,7339	196,26	heating
2,068	17,6954	-8,1355	361,08	heating	2,068	20,4298	7,0593	186,9	heating	2,068	29,1844	0,637	399,05	cooling	2,068	16,9952	0,0554	236,79	heating
2,07	17,8391	-8,3378	365,91		2,07	20,3685	6,5992	192,47	heating	2,07	29,1629	-0,7719	418,44	cooling	2,07	16,9566	1,6997	213,27	heating
2,072	17,5812	-6,1848	332,21		2,072	20,9562	9,4001	161,54	heating	2,072	29,0039	3,5243	356,17	cooling	2,072	16,5377	0,0223	230,86	heating
2,074	17,6882	-2,8472	287,05		2,074	20,4756	8,3635	169,31	heating	2,074	28,6895	3,7319	348,87	cooling	2,074	17,3398	2,3583	209,42	heating
2,076	17,4567	-2,0155	272,19		2,076	20,3545	6,8801	188,35	heating	2,076	28,4685	5,6946	318,35	cooling	2,076	17,2246	3,4286	192,85	heating
2,078	17,596	0,4682	239,42		2,078	20,6664	8,6901	167,41	heating	2,078	28,5309	8,232	283,75	cooling	2,078	17,1407	6,7101	145,8	heating
2,08	17,1319	-3,0869	282,63		2,08	20,4668	8,7567	163,69	heating	2,08	27,7045	4,9695	317,8	cooling	2,08	16,6955	3,4304	185,43	heating
2,082	17,8813	-2,8169	289,33		2,082	20,4221	7,6083	179,12	heating	2,082	28,4441	4,8855	329,31	cooling	2,082	17,4772	3,8623	190,32	heating
2,084	17,3019	-2,7207	279,89		2,084	20,523	8,3293	170,45	heating	2,084	27,6148	4,239	326,76	cooling	2,084	16,65	-0,9883	246,56	heating
2,086	17,6283	-3,5	295,34		2,086	20,643	8,0468	176,08	heating	2,086	28,0391	4,0591	335,2	cooling	2,086	17,3811	1,6812	219,46	heating
2,088	17,4104	-1,5586	265,16		2,088	20,7641	7,0382	191,87	heating	2,088	28,018	8,0528	279,09	cooling	2,088	16,8489	2,2531	204,03	heating
2,09	17,3043	-0,8828	254,23		2,09	20,7469	7,7389	181,83	heating	2,09	27,7773	7,988	276,63	cooling	2,09	17,2178	2,5054	205,66	heating
2,092	16,8308	-1,9275	262,21	heating	2,092	20,7174	5,9988	205,74	heating	2,092	27,8026	5,6177	310,11	cooling	2,092	16,7106	3,7421	181,28	heating
2,094	17,0675	-2,7034	276,37	heating	2,094	20,9497	8,6257	172,27	heating	2,094	28,0007	6,2509	304,03	cooling	2,094	17,0385	4,8499	170,38	heating
2,096	16,2969	-4,138	285,65	heating	2,096	20,4346	8,5178	166,58	heating	2,096	27,41	-0,4617	389,6	cooling	2,096	16,7132	3,0602	190,85	heating
2,098	16,2066	-6,421	316,3	heating	2,098	20,487	6,7682	191,77	heating	2,098	27,4053	-1,5813	405,19	cooling	2,098	17,0367	2,6741	200,77	heating
2,1	16,4577	-2,3091	262,33	heating	2,1	20,7517	5,4188	214,33	heating	2,1	27,181	1,6581	356,77	cooling	2,1	16,6893	2,8877	192,93	heating
2,102	16,2314	-2,5761	262,9	heating	2,102	20,5727	4,6315	222,83	heating	2,102	26,4963	1,3307	351,78	cooling	2,102	17,4356	2,7817	204,84	heating
2,104	16,5526	-1,2646	249,06	heating	2,104	20,954	5,2695	219,25	heating	2,104	27,3678	6,2402	295,33	cooling	2,104	17,2815	6,3395	152,95	heating
2,106	16,5612	-2,0694	260,43	heating	2,106	21,2469	7,8366	187,46	heating	2,106	26,6894	6,3912	283,74	cooling	2,106	16,9814	1,6446	214,39	heating
2,108	17,2735	-1,897	267,98	heating	2,108	21,0952	6,9364	197,92	heating	2,108	27,7106	4,893	318,96	cooling	2,108	17,7733	2,3538	215,54	heating
2,11	17,3421	-2,5603	278,21	heating	2,11	21,2854	7,2989	195,51	heating	2,11	27,8729	9,5027	256,79	cooling	2,11	17,6026	1,3651	226,98	heating
2,112	17,7372	-0,6879	257,56	heating	2,112	21,2912	8,2123	182,82	heating	2,112	27,8226	10,1164	247,51	cooling	2,112	17,4333	3,0451	201,13	heating
2,114	17,3783	-2,5611	278,72	heating	2,114	21,4448	6,1023	214,47	heating	2,114	28,0157	9,1756	263,36	cooling	2,114	17,3772	3,4389	194,84	heating
2,116	18,089	-1,4574	273,23	heating	2,116	21,4129	7,0507	200,76	heating	2,116	28,6205	11,9262	233,36	cooling	2,116	17,8359	4,9503	180,12	heating
2,118	17,1878	-4,1276	297,96	heating	2,118	21,2868	6,385	208,3	heating	2,118	28,0678	7,8132	283,13	cooling	2,118	17,2795	0,8391	229,81	heating
2,12	17,4896	-5,0178	314,62	heating	2,12	21,3326	7,1578	198,14	heating	2,12	28,3912	8,619	276,39	cooling	2,12	17,8087	4,1241	191,29	heating
2,122	17,001	-3,3858	284,98	heating	2,122	21,4368	8,7145	177,84	heating	2,122	27,8519	9,519	256,27	cooling	2,122	17,0824	1,5372	217,3	heating
2,124	16,8719	-3,41	283,51	heating	2,124	20,9039	6,7049	198,48	heating	2,124	27,4888	5,7243	304,24		2,124	17,9255	0,9173	237,75	heating
2,126	16,765	-2,0711	263,3	heating	2,126	21,538	5,3592	226,16	heating	2,126	27,8502	6,5874	297,22		2,126	17,284	2,9495	200,38	heating
2,128	17,0439	-1,4956	259,16	heating	2,128	21,3185	7,0233	199,82	heating	2,128	27,437	5,888	301,22		2,128	17,744	4,9976	178,18	heating
2,13	16,9573	-2,105	266,46	heating	2,13	21,1991	6,2922	208,38	heating	2,13	27,949	2,417	356,9		2,13	17,4832	6,0924	159,23	heating
2,132	17,2725	-0,3185	245,9	heating	2,132	21,6184	8,0198	190,09	heating	2,132	27,925	3,5592	340,6	cooling	2,132	17,6684	5,2889	173,05	heating
2,134	17,4745	3,2472	198,88	heating	2,134	21,5426	7,0744	202,24	heating	2,134	27,9442	3,405	343,02	cooling	2,134	17,2067	2,2789	208,67	heating
2,136	17,5945	0,9134	233,18	heating	2,136	21,6489	5,2085	229,81	heating	2,136	27,792	1,0062	374,43	cooling	2,136	18,2053	3,3392	207,81	heating
2,138	17,9273	1,9373	223,52	heating	2,138	21,7543	7,0002	206,24	heating	2,138	28,4826	2,64	361,24	cooling	2,138	17,4999	7,3413	142	heating
2,14	17,3111	-2,3462	274,78	heating	2,14	22,002	7,0015	209,69	heating	2,14	27,7045	-1,0371	401,76	cooling	2,14	17,3701	5,1086	171,4	heating
2,142	17,8466	-3,8348	303,07	heating	2,142	21,5901	5,3869	226,5	heating	2,142	28,1609	1,0792	378,56	cooling	2,142	18,0506	7,3189	150,01	heating
2,144	17,6293	-4,2845	306,32	heating	2,144	21,9209	6,1622	220,28	heating	2,144	27,7964	5,8874	306,26	cooling	2,144	17,7352	2,6712	210,57	heating
2,146	17,543	-4,2274	304,32	heating	2,146	21,5201	5,531	223,5	heating	2,146	27,6699	6,2518	299,39	cooling	2,146	18,0816	2,671	215,42	heating
2,148	17,4732	-4,5763	308,22	heating	2,148	22,3851	7,9462	201,83	heating	2,148	27,7636	3,8566	334,18	cooling	2,148	17,8089	3,3102	202,67	heating
2,15	18,0169	-2,3852	285,19	heating	2,15	21,4944	7,2935	198,51	heating	2,15	27,7639	0,7784	377,22	cooling	2,15	18,4124	5,6044	179,04	heating
2,152	17,7537	-5,9253	331	heating	2,152	21,8545	6,2202	218,54	heating	2,152	27,8824	-3,5674	439,62	cooling	2,152	17,8236	4,5739	185,21	heating
2,154	18,5227	-4,6174	323,46	heating	2,154	21,8146	7,9739	193,47	heating	2,154	28,0487	0,2491	388,6	cooling	2,154	18,2848	8,1048	142,3	heating
2,156	18,2891	-3,6283	306,37	heating	2,156	21,6521	8,3402	186,08	heating	2,156	27,9388	5,2245	317,51	cooling	2,156	17,4686	3,1497	200,16	heating
2,158	19,0011	-1,5796	287,69	heating	2,158	21,5696	7,311	199,31	heating	2,158	27,6881	4,8944	318,62	cooling	2,158	18,5898	3,0326	217,47	heating

2,16	19,1445	3,0815	224,54	heating	2,16	21,7583	7,051	205,59	heating	2,16	28,4604	5,8709	315,77	cooling	2,16	17,4074	0,5659	235,42	heating
2,162	19,0492	1,2125	249,33	heating	2,162	21,9719	6,7005	213,47	heating	2,162	27,6662	-0,9967	400,66	cooling	2,162	18,2757	0,3087	251,15	heating
2,164	19,1499	-1,79	292,71	heating	2,164	21,867	8,5368	186,34	heating	2,164	28,1307	-3,0259	435,52	cooling	2,164	17,9905	2,7858	212,54	heating
2,166	19,1828	-3,2878	314,11	heating	2,166	22,3105	12,3401	139,37	heating	2,166	28,0944	1,2354	375,45	cooling	2,166	18,0907	3,4955	204,02	heating
2,168	18,833	-4,25	322,67	heating	2,168	21,9065	9,5928	172,13	heating	2,168	28,0519	4,7397	325,87	cooling	2,168	17,7415	1,8349	222,35	heating
2,17	19,2779	-2,8644	309,52	heating	2,17	22,5837	10,35	171,01	heating	2,17	28,3592	6,8608	300,52	cooling	2,17	18,6463	3,1957	215,98	heating
2,172	18,8677	0,135	261,86	heating	2,172	21,8891	8,1703	191,77	heating	2,172	28,6321	9,1603	272,19	cooling	2,172	18,16	5,7566	173,38	heating
2,174	18,7207	-2,755	300,2	heating	2,174	22,4224	8,6386	192,68	heating	2,174	28,6039	5,4419	323,77	cooling	2,174	18,0089	4,2501	192,33	heating
2,176	19,2247	0,3217	264,24	heating	2,176	21,9548	8,0621	194,2	heating	2,176	28,7199	6,9974	303,65	cooling	2,176	18,5886	6,9028	163,35	heating
2,178	19,0189	0,3738	260,63	heating	2,178	22,0158	8,1495	193,83	heating	2,178	28,2741	9,4689	262,87	cooling	2,178	18,2562	2,8809	214,92	heating
2,18	19,1403	1,1191	251,91	heating	2,18	22,1809	8,6109	189,69	heating	2,18	27,913	7,6449	283,32	cooling	2,18	18,5966	4,5259	196,69	heating
2,182	19,1846	3,4302	220,22	heating	2,182	22,417	10,0616	172,71	heating	2,182	28,4189	9,7432	261,06	cooling	2,182	18,0759	3,4114	204,99	heating
2,184	19,3968	4,6846	205,65	heating	2,184	22,3618	8,5557	192,99	heating	2,184	27,4706	7,5034	279,11	cooling	2,184	18,9201	4,777	197,7	heating
2,186	19,286	2,8689	229,49	heating	2,186	22,452	7,7109	206,06	heating	2,186	27,5257	6,2668	297,17	cooling	2,186	18,1576	3,4346	205,81	heating
2,188	19,3998	3,8519	217,34	heating	2,188	22,6391	8,4057	198,96	heating	2,188	27,4201	8,1906	268,8	cooling	2,188	18,6318	7,8325	150,96	heating
2,19	18,5712	-0,4027	265,23	heating	2,19	22,2457	7,2467	209,66	heating	2,19	27,0537	6,5611	286,46	cooling	2,19	17,9489	6,2443	163,61	heating
2,192	19,1444	-1,2482	285,06	heating	2,192	22,5322	9,3291	184,56	heating	2,192	27,5266	5,4791	308,19	cooling	2,192	18,7585	8,2584	146,78	heating
2,194	18,4338	-1,0245	272	heating	2,194	22,1556	6,1595	223,6	heating	2,194	27,7602	7,4096	284,47	cooling	2,194	17,5175	4,7805	178,04	heating
2,196	18,5273	-2,4754	293,59	heating	2,196	22,5003	7,9469	203,43	heating	2,196	27,7094	3,7813	334,48	cooling	2,196	18,2088	3,5841	204,43	heating
2,198	18,345	-2,7711	295,17	heating	2,198	22,1422	7,8241	200,15	heating	2,198	27,8711	4,6844	324,12	cooling	2,198	18,0354	4,5752	188,15	heating
2,2	18,4877	-3,4455	306,59	heating	2,2	22,4187	8,5934	193,26	heating	2,2	27,4675	5,1385	312,13	cooling	2,2	18,0367	2,6912	214,51	heating
2,202	18,2462	-4,4352	317,05	heating	2,202	22,5745	7,4756	211,06	heating	2,202	27,2462	3,7987	327,76	cooling	2,202	18,1676	2,2609	222,35	heating
2,204	18,7712	-2,4354	296,44	heating	2,204	22,3122	7,0033	214	heating	2,204	27,176	5,638	301,07	cooling	2,204	18,9617	3,2933	219,02	heating
2,206	18,3095	-2,8165	295,31	heating	2,206	22,5813	7,5152	210,6	heating	2,206	26,2976	4,1528	309,55	cooling	2,206	18,5985	4,5458	196,44	heating
2,208	18,3597	-4,3626	317,62	heating	2,208	22,7472	9,3711	186,98	heating	2,208	26,2465	2,9044	326,29	cooling	2,208	18,6849	4,3544	200,32	heating
2,21	18,5088	-0,3782	264,01	heating	2,21	22,5189	6,9949	217	heating	2,21	26,5275	5,0878	299,7	cooling	2,21	19,0645	7,3854	163,26	heating
2,212	18,0459	-0,3074	256,55	heating	2,212	22,6189	5,8186	234,84	heating	2,212	26,4163	3,427	321,36	cooling	2,212	18,9857	2,2519	233,91	heating
2,214	18,1237	0,8623	241,29	heating	2,214	22,654	6,5643	224,91	heating	2,214	27,4959	2,9423	343,22	cooling	2,214	19,2428	5,9772	185,43	heating
2,216	17,5915	0,4624	239,44	heating	2,216	22,8352	6,8511	223,44	heating	2,216	27,6852	3,491	338,2	cooling	2,216	18,4755	5,5	181,38	heating
2,218	17,7973	-1,3312	267,39	heating	2,218	22,3781	7,5318	207,53	heating	2,218	28,3203	3,608	345,44	cooling	2,218	19,06	6,321	178,07	heating
2,22	17,6717	-2,8071	286,26	heating	2,22	22,4343	5,592	235,43	heating	2,22	28,5526	8,3227	282,78	cooling	2,22	18,585	4,0411	203,3	heating
2,222	17,9141	-0,5971	258,76	heating	2,222	22,563	5,7407	235,15	heating	2,222	28,6086	10,1394	258,17	cooling	2,222	18,6702	5,1333	189,23	heating
2,224	17,5231	-2,2878	276,93	heating	2,224	23,0312	7,8886	211,67	heating	2,224	28,6789	8,7061	279,19	cooling	2,224	18,7591	4,6918	196,64	heating
2,226	18,6503	0,462	254,24	heating	2,226	22,5338	8,2943	199,05	heating	2,226	28,9985	10,2836	261,61	cooling	2,226	19,4448	9,5352	138,52	heating
2,228	18,3525	0,954	243,21	heating	2,228	23,2368	8,9046	200,34	heating	2,228	28,5283	8,9488	273,69	cooling	2,228	18,4441	7,0534	159,23	heating
2,23	19,0239	0,9749	252,3	heating	2,23	22,9966	8,4766	202,97	heating	2,23	28,4209	7,8377	287,72	cooling	2,23	19,4227	7,3472	168,8	heating
2,232	19,1753	2,1384	238,15	heating	2,232	23,0498	6,6331	229,48	heating	2,232	28,4454	7,8833	287,43	cooling	2,232	19,0745	8,3173	150,37	heating
2,234	19,4801	2,3819	239,01	heating	2,234	23,1876	8,3233	207,78	heating	2,234	28,1136	5,2281	319,91		2,234	19,2234	6,3868	179,44	heating
2,236	19,4704	2,4709	237,63	heating	2,236	23,1556	7,6427	216,85	heating	2,236	28,7977	7,1076	303,19	cooling	2,236	18,9583	8,3528	148,25	heating
2,238	19,6846	3,0715	232,23	heating	2,238	23,3731	9,0264	200,55	heating	2,238	28,1398	8,269	277,76		2,238	19,2739	6,275	181,7	heating
2,24	19,309	0,0315	269,47	heating	2,24	22,8496	11,1112	164,09	heating	2,24	28,4806	7,3056	295,99		2,24	18,9911	5,0987	194,2	heating
2,242	19,2184	-3,2963	314,72	heating	2,242	23,1564	11,5856	161,74	heating	2,242	28,311	6,8302	300,27		2,242	19,1742	5,5709	190,15	heating
2,244	19,176	-2,6041	304,45	heating	2,244	22,822	8,8217	195,7	heating	2,244	28,3962	4,905	328,37		2,244	19,1739	7,4599	163,74	heating
2,246	18,9783	-4,4783	327,89	heating	2,246	23,2023	8,0109	212,35	heating	2,246	28,2457	1,6954	371,13		2,246	19,3924	4,8617	203,12	heating
2,248	19,2219	-1,7597	293,29	heating	2,248	22,809	7,2989	216,81	heating	2,248	28,405	2,4525	362,78	cooling	2,248	19,1568	7,8567	157,96	heating
2,25	18,6464	-1,5079	281,73	heating	2,25	23,6105	11,6766	166,82	heating	2,25	27,7149	-0,1986	390,19	cooling	2,25	18,7041	4,4045	199,89	heating
2,252	19,083	-0,3438	271,56	heating	2,252	22,8464	12,0356	151,12	heating	2,252	27,7175	-0,0398	388,01	cooling	2,252	19,6109	6,6365	181,36	heating
2,254	19,3781	1,7801	245,99	heating	2,254	23,2626	10,8142	174,01	heating	2,254	27,6315	2,0734	357,26	cooling	2,254	18,5989	3,7719	207,26	heating
2,256	19,1663	1,274	250,11	heating	2,256	23,2437	10,5753	177,09	heating	2,256	27,3054	1,9281	354,74	cooling	2,256	18,889	3,0634	221,22	heating
2,258	19,2597	-2,1441	299,19	heating	2,258	23,5173	10,0125	188,78	heating	2,258	27,8394	5,4227	313,35	cooling	2,258	18,6077	4,5649	196,3	heating
2,26	20,0267	1,0186	265,7	heating	2,26	23,5373	10,2596	185,6	heating	2,26	27,086	4,2484	319,24	cooling	2,26	19,0765	6,3772	177,52	heating
2,262	19,7761	1,3092	258,14	heating	2,262	23,4548	10,1516	185,96	heating	2,262	27,4133	-0,3568	388,18	cooling	2,262	18,3735	4,1758	198,46	heating
2,264	20,2291	4,4187	221,01	heating	2,264	23,4815	9,8551	190,48	heating	2,264	27,2301	-2,6319	417,43	cooling	2,264	19,4926	5,6349	193,71	heating
2,266	20,1369	6,4026	191,98	heating	2,266	23,2841	10,0772	184,61	heating	2,266	27,1752	-3,2973	425,96	cooling	2,266	18,701	5,7579	180,93	heating
2,268	20,2925	3,1582	239,51	heating	2,268	23,5945	12,8226	150,58	heating	2,268	27,2392	-3,2808	426,62	cooling	2,268	19,3289	7,0147	172,13	heating
2,27	20,3958	2,0784	256,05	heating	2,27	23,2111	10,2126	181,7	heating	2,27	27,6837	1,6061	364,53	cooling	2,27	18,788	8,1526	148,67	heating

2,272	20,1986	0,2687	278,59	heating	2,272	23,6229	12,1942	159,76	heating	2,272	27,5135	2,5332	349,19	cooling	2,272	19,4594	4,0466	215,45	heating
2,274	20,0464	-2,8755	320,41	heating	2,274	23,1555	11,6503	160,83	heating	2,274	28,0379	4,9917	322,15	cooling	2,274	19,2148	5,4913	191,83	heating
2,276	20,1923	-2,3493	315,1	heating	2,276	23,7973	12,3265	160,34	heating	2,276	28,2473	5,641	316	cooling	2,276	18,9681	5,4998	188,27	heating
2,278	19,5748	-1,2587	291,22	heating	2,278	23,4658	10,226	185,07	heating	2,278	28,2863	3,7068	343,58	cooling	2,278	19,4823	7,8059	163,22	heating
2,28	19,8927	-0,6024	286,49	heating	2,28	23,8741	10,1251	192,19	heating	2,28	28,8234	6,2822	315,09	cooling	2,28	19,541	6,8137	177,91	heating
2,282	19,7574	3,3391	229,5	heating	2,282	23,8955	11,3734	175,04	heating	2,282	28,5048	8,0983	285,25	cooling	2,282	19,3326	7,4928	165,5	heating
2,284	19,3338	0,2745	266,42	heating	2,284	24,1	15,3313	122,57	heating	2,284	28,5721	6,1981	312,76	cooling	2,284	19,1504	6,0458	183,18	heating
2,286	19,8415	0,2942	273,24	heating	2,286	23,6373	14,7114	124,77	heating	2,286	28,6905	8,854	277,28	cooling	2,286	20,1605	10,7936	130,93	heating
2,288	20,1277	1,3402	262,62	heating	2,288	23,681	11,6861	167,67	heating	2,288	28,5733	11,5471	238	cooling	2,288	19,005	6,7315	171,57	heating
2,29	19,9586	0,4706	272,41	heating	2,29	23,8861	10,4084	188,4	heating	2,29	28,8452	11,4069	243,76	cooling	2,29	19,8519	7,9923	165,78	heating
2,292	20,2651	0,7582	272,68	heating	2,292	23,8379	9,2347	204,13	heating	2,292	29,623	14,4997	211,4	cooling	2,292	18,9355	6,4794	174,12	heating
2,294	20,4076	1,4389	265,15	heating	2,294	23,8329	12,1457	163,37	heating	2,294	29,4577	11,2218	254,91	cooling	2,294	19,6484	7,4511	170,5	heating
2,296	20,3045	-1,3886	303,24	heating	2,296	23,6846	10,895	178,78	heating	2,296	30,0453	9,8139	282,8	cooling	2,296	18,5197	5,952	175,68	heating
2,298	20,4126	0,7822	274,4	heating	2,298	23,8951	10,8344	182,57	heating	2,298	30,2653	10,7538	272,74	cooling	2,298	19,3803	4,6752	205,56	heating
2,3	19,689	-0,7886	286,25	heating	2,3	23,9686	10,724	185,14	heating	2,3	29,9186	7,8178	308,94	cooling	2,3	19,0755	3,4236	218,79	heating
2,302	20,0653	-2,5584	316,25	heating	2,302	24,0835	12,1858	166,31	heating	2,302	30,2451	5,7778	342,02	cooling	2,302	19,5149	6,8344	177,25	heating
2,304	19,7127	-0,8313	287,18	heating	2,304	24,4513	9,3316	211,35	heating	2,304	29,8117	4,2969	356,66	cooling	2,304	18,9114	9,1789	136,05	heating
2,306	19,5186	-1,7316	297,05	heating	2,306	24,461	10,0538	201,39	heating	2,306	29,6041	0,4221	407,92	cooling	2,306	19,6308	7,9439	163,37	heating
2,308	19,5369	-1,7055	296,94	heating	2,308	24,2272	9,3589	207,84	heating	2,308	29,5779	4,876	345,29	cooling	2,308	19,2686	5,6392	190,52	heating
2,31	19,6527	-0,7759	285,56	heating	2,31	24,179	10,9865	184,41	heating	2,31	28,8985	6,7088	310,18	cooling	2,31	19,3526	1,981	242,83	heating
2,312	19,1776	-1,7256	292,2	heating	2,312	24,0545	11,1052	181,01	heating	2,312	28,8748	3,7709	350,92	cooling	2,312	19,9691	5,9293	196,26	heating
2,314	19,9473	1,1272	263,08	heating	2,314	23,6404	9,4554	198,29	heating	2,314	29,5959	5,0379	343,29	cooling	2,314	19,8079	5,1607	204,75	heating
2,316	19,5314	1,9698	245,49	heating	2,316	23,5935	10,1477	187,95	heating	2,316	29,0556	-0,2724	409,96	cooling	2,316	19,6987	6,7934	180,4	heating
2,318	19,4488	-2,2496	303,31	heating	2,318	23,8855	11,7221	170,03	heating	2,318	29,8505	0,2199	414,19	cooling	2,318	19,4648	4,95	202,89	heating
2,32	19,7411	-1,4253	295,87	heating	2,32	23,5616	10,704	179,73	heating	2,32	29,987	7,1306	319,5	cooling	2,32	20,4181	7,2996	183,38	heating
2,322	19,7108	-2,4144	309,28	heating	2,322	24,2651	10,2887	195,37	heating	2,322	29,4508	7,4414	307,66	cooling	2,322	19,8378	5,0544	206,65	heating
2,324	19,7727	-2,1087	305,87	heating	2,324	24,1189	9,7167	201,32	heating	2,324	29,9523	9,2798	288,97	cooling	2,324	20,0112	7,2268	178,71	heating
2,326	19,5868	0,2586	270,18	heating	2,326	25,3134	11,2868	196,07	heating	2,326	29,7123	11,8153	250,17	cooling	2,326	19,3818	5,246	197,6	heating
2,328	19,7622	0,456	269,87	heating	2,328	24,8247	13,666	155,98	heating	2,328	29,4188	5,6563	332,16	cooling	2,328	20,0058	8,0797	166,71	heating
2,33	19,8827	-0,354	282,88	heating	2,33	24,9534	10,4047	203,37	heating	2,33	29,5219	8,9362	287,76	cooling	2,33	19,0322	7,966	154,69	heating
2,332	19,9281	2,6788	241,12	heating	2,332	24,5612	7,9909	231,63	heating	2,332	28,8356	9,6701	267,91	cooling	2,332	19,6697	7,6035	168,67	heating
2,334	19,2972	0,294	265,64	heating	2,334	24,7193	8,5619	225,86	heating	2,334	28,6877	4,107	343,6	cooling	2,334	19,6038	6,7023	180,34	heating
2,336	20,3193	2,158	253,87	heating	2,336	24,1994	9,179	209,96	heating	2,336	29,6062	5,3639	338,87	cooling	2,336	19,8674	5,2567	204,24	heating
2,338	19,7161	3,017	233,43	heating	2,338	24,2951	10,491	192,96	heating	2,338	28,9592	3,6529	353,75	cooling	2,338	19,8315	5,8823	194,99	heating
2,34	20,027	2,8393	240,26	heating	2,34	24,0446	9,8139	198,92	heating	2,34	29,553	2,9048	372,5	cooling	2,34	20,5256	5,2756	213,17	heating
2,342	20,2321	3,5183	233,64	heating	2,342	23,9734	8,3095	218,96	heating	2,342	29,5994	9,7799	277,05	cooling	2,342	20,3482	6,667	191,24	heating
2,344	20,3995	1,5431	263,58	heating	2,344	24,8248	12,6238	170,55	heating	2,344	29,0531	6,9122	309,5	cooling	2,344	20,2695	5,4672	206,91	heating
2,346	20,2823	-1,8681	309,63	heating	2,346	24,1631	9,1063	210,47	heating	2,346	29,7651	4,3738	354,93	cooling	2,346	20,7351	8,6657	168,71	heating
2,348	20,774	0,3769	285,12	heating	2,348	25,0748	8,9058	226,02	heating	2,348	29,8953	5,7889	336,97	cooling	2,348	20,4282	5,5538	207,92	heating
2,35	20,5119	1,728	262,57	heating	2,35	24,5209	9,5164	209,74	heating	2,35	29,9348	2,7737	379,67	cooling	2,35	20,1745	5,7238	202	heating
2,352	20,3974	1,1194	269,48	heating	2,352	25,0152	11,505	188,85	heating	2,352	30,089	4,5621	356,83	cooling	2,352	19,6453	2,061	245,8	heating
2,354	20,506	1,9638	259,19	heating	2,354	24,644	13,5868	154,56	heating	2,354	29,5012	4,7666	345,75	cooling	2,354	20,4986	4,7573	220,04	heating
2,356	20,4294	-0,9192	298,42	heating	2,356	24,7557	13,8674	152,2	heating	2,356	29,2514	1,0101	394,77	cooling	2,356	19,9276	4,6451	213,63	heating
2,358	20,9023	1,2989	274,03	heating	2,358	24,6008	10,6579	194,9	heating	2,358	29,7568	3,0881	372,79	cooling	2,358	20,4242	7,4613	181,2	heating
2,36	20,4294	0,9131	272,81	heating	2,36	24,9747	12,2702	177,59	heating	2,36	29,1215	3,8879	352,73	cooling	2,36	20,0211	3,8919	225,46	heating
2,362	21,0567	1,4327	274,31	heating	2,362	24,2746	13,186	155	heating	2,362	29,4204	5,7617	330,71	cooling	2,362	20,8914	3,5061	243,02	heating
2,364	21,2645	0,2439	293,84	heating	2,364	24,2713	12,2214	168,44	heating	2,364	29,2084	7,8992	297,87	cooling	2,364	20,3448	4,6165	219,86	heating
2,366	21,159	0,5425	288,19	heating	2,366	24,3163	12,1236	170,44	heating	2,366	28,8613	3,9441	348,31	cooling	2,366	20,8732	5,4536	215,54	heating
2,368	21,0214	-1,3303	312,44	heating	2,368	24,4934	10,015	202,39	heating	2,368	28,9867	3,526	355,9	cooling	2,368	20,4968	4,8129	219,24	heating
2,37	21,5044	1,1117	285,06	heating	2,37	24,9421	14,2432	149,56	heating	2,37	28,7624	5,2106	329,22	cooling	2,37	20,6576	4,6589	223,64	heating
2,372	21,0856	-0,6802	304,25	heating	2,372	24,7607	14,1532	148,28	heating	2,372	28,6735	4,574	336,88	cooling	2,372	20,2114	6,5054	191,59	heating
2,374	20,9537	0,1404	290,94	heating	2,374	24,9695	12,0875	180,07	heating	2,374	28,4784	7,9334	287,19	cooling	2,374	20,8114	6,4646	200,55	heating
2,376	20,6634	0,2345	285,56	heating	2,376	25,0831	10,179	208,34	heating	2,376	28,0888	9,5208	259,55	cooling	2,376	20,3563	5,8647	202,57	heating
2,378	20,6817	-0,7582	299,7	heating	2,378	25,1569	9,949	212,58	heating	2,378	27,8957	8,0233	277,79	cooling	2,378	20,4383	1,1403	269,76	heating
2,38	20,6268	0,1141	286,74	heating	2,38	25,1558	9,6848	216,26	heating	2,38	28,3004	10,59	247,56	cooling	2,38	21,1211	5,6074	216,86	heating
2,382	20,5394	1,5175	265,9	heating	2,382	25,2951	10,2041	210,95	heating	2,382	27,6172	9,5054	253,18	cooling	2,382	20,9899	6,0246	209,19	heating

2,384	20,6996	2,7394	251,06	heating	2,384	24,6831	6,2728	257,35	heating	2,384	28,0294	7,3544	289,01	cooling	2,384	20,6907	7,9752	177,74	heating
2,386	20,8092	2,7556	252,36	heating	2,386	25,5157	9,0004	230,86	heating	2,386	27,955	7,643	283,93	cooling	2,386	20,5636	5,3298	212,94	heating
2,388	20,82	1,8337	265,4	heating	2,388	24,6071	8,5571	224,35	heating	2,388	27,6115	5,5397	308,53	cooling	2,388	21,1699	5,5204	218,76	heating
2,39	21,1566	-0,8387	307,46	heating	2,39	25,3322	8,2119	239,32	heating	2,39	27,4838	5,5525	306,57	cooling	2,39	20,7287	2,3212	257,31	heating
2,392	21,1196	1,0705	280,26	heating	2,392	24,8196	8,9119	222,37	heating	2,392	27,6119	7,3305	283,5	cooling	2,392	21,1575	5,816	214,45	heating
2,394	20,6837	-0,8263	300,68	heating	2,394	25,1529	9,2157	222,78	heating	2,394	27,4667	3,5672	334,08	cooling	2,394	20,6419	4,0725	231,61	heating
2,396	21,1189	0,4695	288,65	heating	2,396	24,9895	11,4924	188,67	heating	2,396	27,8889	2,6574	352,7	cooling	2,396	21,5804	5,456	225,39	heating
2,398	21,0446	-1,525	315,49	heating	2,398	25,3036	12,2485	182,49	heating	2,398	28,0257	2,9149	351,01	cooling	2,398	20,7099	5,7511	209,1	heating
2,4	20,8841	-3,2122	336,83	heating	2,4	25,145	7,7221	243,55	heating	2,4	28,1759	0,9304	380,85	cooling	2,4	21,4838	6,8986	203,88	heating
2,402	20,6638	-3,8016	341,99	heating	2,402	25,3936	6,3146	266,7	heating	2,402	28,7706	2,4379	368,09	cooling	2,402	20,7486	5,3485	215,27	heating
2,404	21,049	-0,7473	304,68	heating	2,404	25,2755	8,2069	238,59	heating	2,404	28,4983	0,7944	387,26	cooling	2,404	21,0419	4,4847	231,44	heating
2,406	20,8615	-0,662	300,87	heating	2,406	25,2585	8,7227	231,15	heating	2,406	29,0073	-1,0352	419,95	cooling	2,406	20,3715	5,2702	211,09	heating
2,408	21,3363	2,741	259,93	heating	2,408	25,6135	13,0276	175,93	heating	2,408	28,9204	0,9644	390,78	cooling	2,408	21,2192	6,3692	207,58	heating
2,41	21,2931	2,858	257,69	heating	2,41	24,7877	8,6523	225,55	heating	2,41	28,9224	3,1641	360,06	cooling	2,41	20,4184	5,4281	209,54	heating
2,412	22,1683	2,4514	275,61	heating	2,412	25,4694	9,8378	218,51	heating	2,412	29,1178	5,337	332,42	cooling	2,412	20,6056	2,4596	253,65	heating
2,414	21,9824	1,7035	283,47	heating	2,414	25,021	8,8883	225,51	heating	2,414	29,6551	8,7048	292,85	cooling	2,414	20,8542	5,6141	213,03	heating
2,416	22,2415	1,0156	296,71	heating	2,416	25,118	7,0228	252,95	heating	2,416	29,3711	5,6413	331,71	cooling	2,416	20,9705	5,5258	215,89	heating
2,418	22,1145	0,6604	299,9	heating	2,418	25,2015	6,3926	262,92	heating	2,418	29,6218	5,8067	332,9	cooling	2,418	20,901	7,597	185,97	heating
2,42	22,26	1,7376	286,87	heating	2,42	25,5947	9,9057	219,31	heating	2,42	29,2693	6,3042	321,02	cooling	2,42	20,7808	5,9946	206,69	heating
2,422	21,7926	1,0386	290,11	heating	2,422	25,4177	8,4037	237,83	heating	2,422	29,2319	5,69	329,08	cooling	2,422	21,5697	6,0922	216,35	heating
2,424	22,17	1,3519	291,01	heating	2,424	25,8663	11,631	198,99	heating	2,424	29,7075	7,4023	311,79	cooling	2,424	20,9917	3,8732	239,29	heating
2,426	21,9224	3,7761	253,66	heating	2,426	25,2583	8,7544	230,7	heating	2,426	29,2683	4,6748	343,78	cooling	2,426	21,406	8,2374	184,08	heating
2,428	21,4552	2,0501	271,25	heating	2,428	25,8886	6,5185	270,77	heating	2,428	30,0497	5,1917	347,48	cooling	2,428	20,5452	5,7111	207,36	heating
2,43	21,9608	3,2399	261,69	heating	2,43	25,8901	10,1451	220,09	heating	2,43	29,6917	8,8214	291,74	cooling	2,43	21,6401	6,2108	215,68	heating
2,432	22,0801	1,1712	292,28	heating	2,432	25,4584	7,8178	246,59	heating	2,432	29,4494	7,3837	308,45	cooling	2,432	20,437	3,8807	231,43	heating
2,434	22,0879	-1,0271	323,11	heating	2,434	25,6191	8,001	246,28	heating	2,434	29,4798	6,9527	314,9	cooling	2,434	21,5169	4,7807	233,95	heating
2,436	22,2684	-0,4764	317,94	heating	2,436	25,8583	9,4453	229,43	heating	2,436	29,6465	8,035	302,1	cooling	2,436	20,8679	4,9271	222,83	heating
2,438	22,916	2,963	278,91	heating	2,438	25,1267	8,3899	233,96	heating	2,438	29,1522	1,9948	379,62	cooling	2,438	21,1594	4,3217	235,37	heating
2,44	22,9147	1,6976	296,58	heating	2,44	25,8269	10,9182	208,4	heating	2,44	29,5859	6,1572	327,5	cooling	2,44	20,5113	4,2005	228	heating
2,442	22,9536	3,8287	267,34	heating	2,442	25,3142	14,5563	150,38	heating	2,442	28,7426	6,8995	305,33	cooling	2,442	21,5378	7,5282	195,83	heating
2,444	22,3554	0,6005	304,1	heating	2,444	25,4132	9,215	226,43	heating	2,444	28,7462	3,6679	350,56	cooling	2,444	20,8302	7,4918	186,45	heating
2,446	23,126	-2,149	353,31	heating	2,446	25,7346	10,9107	207,22	heating	2,446	29,1505	5,4164	331,77	cooling	2,446	20,9133	4,017	236,18	heating
2,448	22,8403	-2,9284	360,21	heating	2,448	25,666	9,1196	231,29	heating	2,448	27,9902	1,5926	369	cooling	2,448	21,4705	6,3887	210,82	heating
2,45	22,7049	-3,9954	373,23		2,45	26,0993	7,9828	253,24	heating	2,45	28,4255	-0,489	404,18	cooling	2,45	21,268	4,021	241,09	heating
2,452	22,8852	-3,2767	365,7		2,452	26,074	9,8638	226,59	heating	2,452	28,0926	3,5837	342,6	cooling	2,452	21,4845	7,6174	193,84	heating
2,454	23,2579	2,2183	294,1		2,454	25,9641	10,4984	216,19	heating	2,454	27,7457	1,5093	366,75	cooling	2,454	20,9295	6,3198	204,22	heating
2,456	22,6245	3,4724	267,72		2,456	26,018	10,817	212,49	heating	2,456	28,0555	2,7614	353,57	cooling	2,456	21,8838	4,5914	241,72	heating
2,458	23,1431	3,459	275,15		2,458	26,3024	12,8052	188,67	heating	2,458	28,2133	6,8471	298,67	cooling	2,458	21,4111	2,1987	268,56	heating
2,46	23,0658	4,2674	262,77		2,46	25,3141	6,6437	260,98	heating	2,46	27,817	2,3879	355,46	cooling	2,46	21,5997	5,9806	218,33	heating
2,462	22,9133	1,2056	303,44		2,462	26,5264	7,8072	261,67	heating	2,462	28,1435	6,9198	296,67	cooling	2,462	20,8704	4,2174	232,79	heating
2,464	22,9371	2,3809	287,35		2,464	25,4291	9,1308	227,83	heating	2,464	27,3888	8,0087	270,91	cooling	2,464	22,0208	8,7031	186,16	heating
2,466	23,1894	2,4863	289,4		2,466	25,9745	8,1854	248,66	heating	2,466	27,4744	7,0402	285,64	cooling	2,466	20,6957	5,8936	206,91	heating
2,468	22,9315	1,1655	304,26		2,468	25,6423	11,1955	201,94	heating	2,468	28,0039	11,5632	229,82	cooling	2,468	21,7141	5,4257	227,69	heating
2,47	22,8571	2,2681	287,8		2,47	25,8796	9,9409	222,8	heating	2,47	27,0369	9,0513	251,41	cooling	2,47	21,266	5,6276	218,6	heating
2,472	22,8841	4,6866	254,37		2,472	25,7892	8,3306	244,04	heating	2,472	27,7919	7,0875	289,42	cooling	2,472	21,2124	1,6383	273,62	heating
2,474	23,0786	3,4485	274,4		2,474	26,2871	11,3447	208,87	heating	2,474	27,6446	9,0923	259,33	cooling	2,474	21,3201	1,6303	275,23	heating
2,476	22,7679	2,6818	280,77		2,476	25,6814	9,9761	219,54	heating	2,476	27,3448	4,28	322,41	cooling	2,476	21,7474	4,4946	241,17	heating
2,478	22,156	-2,1165	339,29		2,478	26,1135	7,6901	257,53	heating	2,478	27,8837	4,5141	326,67	cooling	2,478	21,4121	5,9583	216,02	heating
2,48	22,9336	-2,578	356,62		2,48	26,2282	9,6056	232,36	heating	2,48	28,0115	7,1698	291,34	cooling	2,48	21,3934	5,5767	221,09	heating
2,482	22,4243	-1,0139	327,63		2,482	25,8176	5,7373	280,69	heating	2,482	28,0227	3,0063	349,69	cooling	2,482	21,5701	6,0366	217,14	heating
2,484	22,2313	-0,3949	316,28		2,484	26,7606	10,5187	227,04	heating	2,484	28,4799	6,4825	307,49	cooling	2,484	21,598	1,8914	275,47	heating
2,486	21,8874	-1,1882	322,56		2,486	25,9268	10,6749	213,2	heating	2,486	28,1596	7,0337	295,31	cooling	2,486	22,0087	7,5459	202,17	heating
2,488	22,5861	0,5313	308,29		2,488	26,545	11,0253	216,94	heating	2,488	28,296	5,4806	318,93	cooling	2,488	21,1493	5,6647	216,45	heating
2,49	21,7906	-1,0813	319,71		2,49	26,005	12,7515	185,26	heating	2,49	28,8269	9,2065	274,26	cooling	2,49	22,3066	8,7076	190,09	heating
2,492	22,3503	-0,0381	312,96		2,492	26,0913	11,9484	197,7	heating	2,492	27,8818	5,6089	311,34	cooling	2,492	21,5751	6,8177	206,29	heating
2,494	22,0072	1,1243	291,91		2,494	25,7299	6,9716	262,21	heating	2,494	28,6598	3,8442	346,88	cooling	2,494	21,6604	5,6492	223,81	heating

2,496	22,0591	1,5626	286,51		2,496	26,7571	8,6592	252,98	heating	2,496	28,437	8,1344	283,8	cooling	2,496	21,5668	3,6854	249,96	heating
2,498	21,6686	2,6363	266,04		2,498	25,8829	7,0281	263,56	heating	2,498	28,0161	6,7483	297,29	cooling	2,498	22,0647	6,5422	216,98	heating
2,5	22,0874	4,7798	241,94		2,5	26,7429	10,3383	229,31	heating	2,5	28,3505	8,3702	279,29	cooling	2,5	21,3117	3,6943	246,27	heating
2,502	21,8574	4,654	240,48		2,502	26,0236	11,9363	196,92	heating	2,502	28,0575	7,0172	294,11	cooling	2,502	22,1062	6,303	220,9	heating
2,504	21,3154	4,0356	241,55		2,504	26,1998	8,5757	246,36	heating	2,504	28,3708	-0,4017	402,2	cooling	2,504	21,5382	6,1618	214,94	heating
2,506	21,4907	5,0332	230,05		2,506	26,1755	10,1483	224,04	heating	2,506	28,6734	1,2346	383,55	cooling	2,506	21,8645	4,3484	244,85	heating
2,508	21,6318	3,1263	258,68	heating	2,508	26,0605	8,5587	244,65	heating	2,508	28,2849	1,1045	379,94	cooling	2,508	21,8226	5,7213	225,07	heating
2,51	21,9242	3,5254	257,19	heating	2,51	26,1719	8,4342	247,95	heating	2,51	28,3426	-0,4423	402,37	cooling	2,51	21,9531	2,4544	272,56	heating
2,512	21,4282	0,7091	289,62	heating	2,512	26,1099	9,4223	233,27	heating	2,512	28,847	5,2447	329,92	cooling	2,512	21,9447	4,387	245,43	heating
2,514	22,5884	0,788	304,74	heating	2,514	26,0145	9,4917	230,96	heating	2,514	27,77	4,6533	323,14	cooling	2,514	21,6114	3,7458	249,73	heating
2,516	22,5163	1,1478	298,7	heating	2,516	26,4854	7,4756	265,73	heating	2,516	28,1887	7,2543	292,63	cooling	2,516	21,863	3,5678	255,74	heating
2,518	22,4188	2,8682	273,29	heating	2,518	27,1146	11,9207	212,39	heating	2,518	27,6699	11,2291	229,82	cooling	2,518	22,2977	2,2713	279,94	heating
2,52	22,1508	0,4753	302,99	heating	2,52	26,5632	9,0268	245,13	heating	2,52	27,2538	6,9974	283,15	cooling	2,52	21,9825	6,1394	221,46	heating
2,522	23,1105	2,8213	283,61	heating	2,522	27,3633	12,4361	208,66	heating	2,522	27,7836	6,2818	300,56	cooling	2,522	21,7632	3,0852	261,09	heating
2,524	22,6555	2,1114	287,18	heating	2,524	26,5525	12,0067	203,33	heating	2,524	27,7137	6,6843	293,96	cooling	2,524	22,6043	7,1041	216,67	heating
2,526	22,5487	2,5872	279,03	heating	2,526	26,7152	10,4458	227,42	heating	2,526	27,9137	2,2769	358,36	cooling	2,526	21,6517	2,9419	261,54	heating
2,528	22,5761	1,8903	289,16	heating	2,528	26,1967	8,9955	240,45	heating	2,528	28,2549	5,0139	324,87	cooling	2,528	22,2166	3,2628	264,95	heating
2,53	22,4524	-1,058	328,64	heating	2,53	26,4477	8,6683	248,53	heating	2,53	27,7388	5,4336	311,79	cooling	2,53	22,1673	5,2009	237,16	heating
2,532	22,5633	-2,4637	349,84	heating	2,532	25,9447	7,3333	260,16	heating	2,532	27,9634	3,9874	335,15	cooling	2,532	22,4903	6,1033	229,07	heating
2,534	22,9423	-0,759	331,31		2,534	26,1095	10,7863	214,2	heating	2,534	28,4978	9,4106	266,81	cooling	2,534	22,008	4,3608	246,68	heating
2,536	23,0762	0,043	321,97		2,536	25,4702	10,3859	210,86	heating	2,536	27,6375	6,84	290,72	cooling	2,536	22,5885	6,3184	227,43	heating
2,538	22,9693	-0,4045	326,73		2,538	26,2165	8,8163	243,23	heating	2,538	28,0927	5,3382	318,07	cooling	2,538	21,7727	4,6476	239,38	heating
2,54	22,9386	0,4611	314,2		2,54	26,2136	9,3977	235,06	heating	2,54	27,9963	6,1374	305,55	cooling	2,54	22,4027	5,9276	230,3	heating
2,542	23,3511	-1,2639	344,08		2,542	26,5971	7,1927	271,25	heating	2,542	27,7747	3,2067	343,43	cooling	2,542	22,03	8,327	191,55	heating
2,544	23,3013	0,7007	315,92		2,544	27,1069	10,9784	225,45	heating	2,544	28,4887	6,7421	303,99	cooling	2,544	22,6418	5,0201	246,32	heating
2,546	23,128	1,0016	309,29		2,546	26,8988	9,9851	236,43	heating	2,546	28,3316	11,163	239,99	cooling	2,546	22,2826	6,5285	220,22	heating
2,548	23,3069	1,7827	300,88		2,548	26,9824	9,2554	247,8	heating	2,548	28,3619	7,5412	291,04	cooling	2,548	21,9956	4,147	249,5	heating
2,55	23,7939	2,6035	296,21		2,55	26,9903	8,0019	265,43	heating	2,55	28,4863	7,3288	295,75	cooling	2,55	22,4704	5,5257	236,86	heating
2,552	23,1596	3,6919	272,13		2,552	27,001	8,616	257	heating	2,552	27,9675	5,5554	313,29	cooling	2,552	22,6954	4,5529	253,61	heating
2,554	23,3039	3,7905	272,77		2,554	26,469	6,1336	284,26	heating	2,554	28,2477	3,7845	341,96	cooling	2,554	22,2508	4,8619	243,07	heating
2,556	23,8875	7,0438	235,45		2,556	26,7207	11,0215	219,45	heating	2,556	28,5755	6,7976	304,42	cooling	2,556	22,2822	4,5305	248,14	heating
2,558	23,6349	6,5254	239,17		2,558	25,8353	9,2022	232,51	heating	2,558	28,0774	1,6395	369,56	cooling	2,558	22,6919	9,9537	178,06	heating
2,56	23,6287	6,2224	243,31		2,56	26,5342	10,7643	220,44	heating	2,56	28,4883	-1,1183	413,86	cooling	2,56	21,9717	7,303	205,05	heating
2,562	23,1947	3,1963	279,55	heating	2,562	26,223	11,7306	202,58	heating	2,562	28,7211	2,1678	371,18	cooling	2,562	22,5488	8,4158	197,56	heating
2,564	23,3742	-0,194	329,45	heating	2,564	26,7037	9,2094	244,55	heating	2,564	28,5371	3,9087	344,27	cooling	2,564	22,1887	6,0292	225,89	heating
2,566	23,0183	-0,4214	327,65		2,566	26,8302	9,0586	248,42	heating	2,566	29,1692	7,3126	305,52	cooling	2,566	22,6852	3,0086	275,05	heating
2,568	23,3085	2,8722	285,67		2,568	27,0451	11,4881	217,46	heating	2,568	29,0303	8,6796	284,47	cooling	2,568	22,2768	3,7945	258,36	heating
2,57	22,9995	3,4971	272,61		2,57	26,5576	10,8144	220,07	heating	2,57	29,0428	5,1456	334,05	cooling	2,57	22,5957	5,6934	236,27	heating
2,572	23,1814	2,4854	289,3		2,572	27,1466	11,1707	223,32	heating	2,572	29,2285	7,9832	296,98	cooling	2,572	22,2018	6,5556	218,71	heating
2,574	22,7385	-1,6438	340,83		2,574	26,5839	12,4709	197,28	heating	2,574	28,7629	8,8486	278,37	cooling	2,574	22,8258	8,6883	197,62	heating
2,576	23,4382	-4,7151	393,54		2,576	26,609	9,4338	240,08	heating	2,576	28,7935	6,3904	313,16	cooling	2,576	22,4394	9,4553	181,5	heating
2,578	23,2774	-2,5293	360,74		2,578	26,5282	12,8118	191,74	heating	2,578	28,9105	5,9108	321,5	cooling	2,578	22,9503	6,8716	224,76	heating
2,58	23,0847	-1,176	339,13		2,58	26,082	10,4143	219,01	heating	2,58	28,5857	1,9888	371,78	cooling	2,58	22,3229	6,6383	219,25	heating
2,582	23,5474	2,4871	294,39		2,582	26,226	9,8559	228,83	heating	2,582	28,5439	1,5463	377,39	cooling	2,582	22,3893	4,724	246,93	heating
2,584	23,7209	3,3956	284,12		2,584	26,5916	10,1153	230,31	heating	2,584	28,6058	5,381	324,65	cooling	2,584	22,7987	8,9162	194,06	heating
2,586	23,0705	2,05	293,84		2,586	26,7198	10,5833	225,56	heating	2,586	27,9488	3,1485	346,67	cooling	2,586	22,8019	8,8917	194,44	heating
2,588	23,1034	-0,0827	324,11		2,588	26,9417	9,0645	249,9	heating	2,588	28,5433	3,2803	353,14	cooling	2,588	22,5823	7,0444	217,2	heating
2,59	23,5476	1,2978	311,02		2,59	27,3281	12,0647	213,36	heating	2,59	28,0913	3,1485	348,66	cooling	2,59	22,4231	3,9196	258,65	heating
2,592	23,3751	1,8156	301,37		2,592	26,9	9,0129	250,04	heating	2,592	28,1182	0,5712	385,07	cooling	2,592	22,9392	5,9808	237,05	heating
2,594	23,4003	6,9012	230,63		2,594	27,7562	11,4613	227,78	heating	2,594	28,3314	4,8544	328,17	cooling	2,594	22,4148	6,2944	225,34	heating
2,596	23,2248	6,3102	236,44		2,596	26,92	11,5849	214,36	heating	2,596	28,0526	8,1497	278,21	cooling	2,596	23,105	10,5896	174,95	heating
2,598	23,637	5,1502	258,42		2,598	27,0577	9,4515	246,11	heating	2,598	28,1109	5,3284	318,47	cooling	2,598	22,4268	9,9999	173,71	heating
2,6	23,0265	3,3334	275,28		2,6	26,2828	9,8183	230,15	heating	2,6	28,4782	6,4508	307,91	cooling	2,6	23,1778	9,4612	191,74	heating
2,602	23,3056	4,6057	261,4		2,602	26,282	9,1778	239,09	heating	2,602	28,2527	4,9098	326,3	cooling	2,602	22,4811	7,5738	208,38	heating
2,604	23,1812	4,8647	256,04		2,604	25,8627	7,7199	253,61	heating	2,604	28,4117	4,1333	339,38	cooling	2,604	23,1093	6,7038	229,32	heating
2,606	23,0577	4,4846	259,63		2,606	27,0651	10,6609	229,31	heating	2,606	28,7688	6,2723	314,47	cooling	2,606	22,4256	6,4238	223,68	heating

2,608	22,4016	1,8593	287,15	heating	2,608	26,5936	10,3438	227,15	heating	2,608	28,0258	0,9342	378,7	cooling	2,608	22,8349	7,6454	212,33	heating
2,61	23,313	2,7033	288,09	heating	2,61	27,7268	9,4244	255,84	heating	2,61	28,7442	-0,6123	410,36	cooling	2,61	22,4187	7,8522	203,62	heating
2,612	22,7331	3,2826	271,89	heating	2,612	27,7421	11,8898	221,59	heating	2,612	28,1057	0,7015	383,07	cooling	2,612	23,0292	7,771	213,29	heating
2,614	22,5439	2,3871	281,76	heating	2,614	27,7415	9,419	256,12	heating	2,614	28,0622	1,0043	378,23	cooling	2,614	22,5216	9,049	188,33	heating
2,616	22,9426	4,2866	260,78	heating	2,616	27,9829	10,3641	246,29	heating	2,616	28,0939	4,3475	331,94	cooling	2,616	22,6244	6,8835	220,03	heating
2,618	23,4097	2,5942	290,97	heating	2,618	27,3791	8,8391	259,16	heating	2,618	27,8838	6,8776	293,64	cooling	2,618	23,207	10,2279	181,43	heating
2,62	22,9351	2,2384	289,31	heating	2,62	27,0687	8,9957	252,63	heating	2,62	27,7765	6,2294	301,2	cooling	2,62	22,9094	8,7061	198,54	heating
2,622	22,9631	3,0006	279,05	heating	2,622	26,1902	8,5861	246,08	heating	2,622	27,6354	9,128	258,71	cooling	2,622	23,2527	9,5125	192,07	heating
2,624	23,3342	4,2948	266,14	heating	2,624	26,1676	9,4086	234,27	heating	2,624	27,2151	8,2611	264,95	cooling	2,624	22,7614	6,9984	220,34	heating
2,626	23,242	3,3964	277,41	heating	2,626	25,482	6,1763	269,86	heating	2,626	27,2669	7,8428	271,52	cooling	2,626	23,3087	9,0893	198,77	heating
2,628	22,746	5,8068	236,78	heating	2,628	26,6004	8,5278	252,63	heating	2,628	27,5108	9,5082	251,65	cooling	2,628	22,6366	8,8846	192,23	heating
2,63	22,3646	1,7948	287,54	heating	2,63	26,0954	6,831	269,29	heating	2,63	26,9609	6,1118	291,44	cooling	2,63	22,9252	10,2821	176,73	heating
2,632	23,0719	-0,0166	322,74	heating	2,632	27,7724	9,3003	258,21	heating	2,632	27,5861	6,9518	288,44	cooling	2,632	22,2937	5,6962	232,01	heating
2,634	22,2609	-3,9881	366,92	heating	2,634	27,3706	9,7588	246,19	heating	2,634	26,9343	6,1291	290,83	cooling	2,634	23,1198	7,4215	219,44	heating
2,636	22,931	-2,4049	354,16	heating	2,636	28,2115	10,6813	245,05	heating	2,636	27,0852	3,1894	334,03	cooling	2,636	22,4504	7,0006	215,97	heating
2,638	23,235	-0,3523	329,72		2,638	28,0889	11,3532	233,94	heating	2,638	27,1767	3,9307	324,94	cooling	2,638	23,3077	8,7482	203,52	heating
2,64	23,9989	0,8582	323,47		2,64	28,3683	10,5585	248,96	heating	2,64	26,9395	4,6159	312,05	cooling	2,64	22,8734	10,4379	173,83	heating
2,642	23,7855	-1,2919	350,54		2,642	28,0224	9,7798	255,01	heating	2,642	26,7638	2,412	340,4	cooling	2,642	22,8423	6,7925	224,35	heating
2,644	25,3536	1,8571	328,45		2,644	27,6549	9,7264	250,61	heating	2,644	26,6964	4,5445	309,65	cooling	2,644	23,487	6,8109	233,11	heating
2,646	25,033	3,0652	307,08		2,646	27,4678	11,3617	225,14	heating	2,646	26,3994	2,2569	337,48	cooling	2,646	23,7719	8,7676	209,74	heating
2,648	25,2413	3,7313	300,68		2,648	27,2689	10,579	233,3	heating	2,648	26,6858	1,6801	349,54	cooling	2,648	23,1816	8,6125	203,65	heating
2,65	25,2338	4,4377	290,7		2,65	27,6192	13,1687	202	heating	2,65	26,9636	4,942	307,83	cooling	2,65	23,4502	8,6059	207,5	heating
2,652	25,5967	1,176	341,36		2,652	27,1417	10,2191	236,55	heating	2,652	26,407	3,8298	315,6	cooling	2,652	23,9051	12,3215	161,92	heating
2,654	25,0179	2,4124	315,99		2,654	28,0129	13,307	205,57	heating	2,654	27,0291	3,831	324,28	cooling	2,654	23,3566	7,7742	217,82	heating
2,656	24,4003	3,9934	285,26		2,656	27,4733	11,2915	226,2	heating	2,656	26,6898	0,5802	364,97	cooling	2,656	23,9334	11,1955	178,06	heating
2,658	24,6358	5,4631	268,01		2,658	28,1052	13,7657	200,45	heating	2,658	27,1383	-2,0958	408,65	cooling	2,658	23,0306	8,7905	199,06	heating
2,66	24,7267	4,7299	279,53		2,66	27,633	11,6151	223,91	heating	2,66	27,439	1,9627	356,12	cooling	2,66	23,7327	7,7963	222,77	heating
2,662	23,9158	4,5838	270,23		2,662	28,1437	11,2699	235,87	heating	2,662	27,1009	5,6725	299,54	cooling	2,662	23,3835	9,7861	190,07	heating
2,664	23,7416	-1,3604	350,89		2,664	27,9537	9,8691	252,8	heating	2,664	27,2523	5,7223	300,96	cooling	2,664	23,3751	10,8286	175,38	heating
2,666	24,8313	-0,0914	348,38		2,666	27,9233	11,5345	229,09	heating	2,666	27,3362	7,7418	273,9	cooling	2,666	23,2387	7,542	219,42	heating
2,668	24,1242	-2,3756	370,43		2,668	27,5027	11,0564	229,89	heating	2,668	27,2476	5,4129	305,22	cooling	2,668	23,9614	11,001	181,17	heating
2,67	24,4285	1,7263	317,34		2,67	27,8055	13,2847	202,98	heating	2,67	27,3993	7,5138	277,97	cooling	2,67	23,1864	7,796	215,14	heating
2,672	24,3249	4,4427	277,92		2,672	27,4302	12,2352	212,4	heating	2,672	27,3773	12,7068	205,07	cooling	2,672	24,1917	8,7524	215,82	heating
2,674	24,4885	4,502	279,38		2,674	27,6195	10,0866	245,08	heating	2,674	26,8786	12,9048	195,33	cooling	2,674	23,8496	10,6765	184,14	heating
2,676	23,8	1,2228	315,6		2,676	27,831	12,2149	218,29	heating	2,676	27,3883	13,1588	198,91	cooling	2,676	23,7254	6,1481	245,7	heating
2,678	24,3223	3,1781	295,57		2,678	28,0357	8,9091	267,36	heating	2,678	26,9309	10,7755	225,83		2,678	24,3841	8,7545	218,48	heating
2,68	23,6164	0,0464	329,47		2,68	28,2718	12,0641	226,56	heating	2,68	28,3383	6,4944	305,34		2,68	24,0872	10,6475	187,87	heating
2,682	23,7043	-0,3525	336,28		2,682	28,4088	12,9153	216,58	heating	2,682	29,8346	5,1791	344,65		2,682	23,6345	9,2083	201,66	heating
2,684	23,3876	1,3605	307,91		2,684	28,3816	12,9497	215,71	heating	2,684	29,0457	4,3642	345,01		2,684	23,8263	9,7365	196,95	heating
2,686	23,8952	-0,5536	341,76		2,686	29,0682	12,4699	232,02	heating	2,686	29,3558	1,5919	388,1		2,686	23,7319	9,6116	197,38	heating
2,688	23,9113	1,9123	307,51		2,688	28,2918	12,8554	215,78	heating	2,688	29,9286	2,8007	379,21	cooling	2,688	24,0416	5,3834	260,81	heating
2,69	23,4897	0,8957	315,83		2,69	28,3373	9,8883	257,89	heating	2,69	30,5769	2,8323	387,83	cooling	2,69	24,7157	11,2838	187,76	heating
2,692	24,3137	1,0186	325,63		2,692	28,19	14,3862	192,96	heating	2,692	30,966	4,2374	373,63	cooling	2,692	24,0411	9,4952	203,33	heating
2,694	24,7996	0,9463	333,43		2,694	27,5298	11,5934	222,77	heating	2,694	31,3163	7,0109	339,75	cooling	2,694	24,7606	10,1288	204,53	heating
2,696	24,0647	1,9706	308,84		2,696	27,9062	11,359	231,3	heating	2,696	30,8994	4,3769	370,74	cooling	2,696	24,5652	12,7	165,86	heating
2,698	23,9436	-3,1835	379,2		2,698	28,0766	11,1549	236,54	heating	2,698	31,395	2,2437	407,49	cooling	2,698	24,2769	11,2344	182,32	heating
2,7	25,0142	-0,869	361,81	cooling	2,7	28,4839	8,0843	285,16	heating	2,7	31,1763	3,2225	390,75	cooling	2,7	24,2013	9,2782	208,6	heating
2,702	24,2044	-5,5578	416,03	cooling	2,702	28,4605	9,3577	267,03	heating	2,702	30,9708	4,9767	363,36	cooling	2,702	24,2547	10,2415	195,88	heating
2,704	24,1553	-2,066	366,54	cooling	2,704	28,884	11,937	236,89	heating	2,704	30,9586	6,3834	343,52	cooling	2,704	23,5601	7,6975	221,74	heating
2,706	23,7723	0,343	327,51	cooling	2,706	28,5782	9,9947	259,77	heating	2,706	31,0471	6,8085	338,82	cooling	2,706	24,4092	11,3999	181,85	heating
2,708	24,0032	2,8323	295,94	cooling	2,708	29,9551	12,4642	244,5	heating	2,708	31,3076	5,048	367,07	cooling	2,708	23,6175	12,9392	149,27	heating
2,71	23,2839	2,9503	284,23	cooling	2,71	28,6426	11,673	237,21	heating	2,71	31,4738	6,099	354,7	cooling	2,71	23,9157	7,1041	235	heating
2,712	23,5037	5,9482	245,4	cooling	2,712	29,5424	10,1047	271,71	heating	2,712	32,0161	10,8293	296,16	cooling	2,712	24,5782	8,2064	228,85	heating
2,714	23,24	2,4252	290,96	cooling	2,714	28,7576	13,6519	211,15	heating	2,714	31,7937	11,2196	287,6	cooling	2,714	24,5401	6,4451	252,94	heating
2,716	23,6829	2,7773	292,23	cooling	2,716	28,5747	11,3594	240,64	heating	2,716	32,1768	8,5248	330,62	cooling	2,716	24,6916	7,6185	238,66	heating
2,718	22,999	1,0531	306,77	cooling	2,718	28,2359	11,8384	229,21	heating	2,718	32,4333	9,2483	324,09	cooling	2,718	24,9898	10,5647	201,64	heating

2,72	24,0824	-0,2999	340,83	cooling	2,72	28,5996	13,8212	206,58	heating	2,72	32,4661	12,4565	279,7	cooling	2,72	24,6809	9,7309	208,98	heating
2,722	23,902	3,7314	281,95	cooling	2,722	28,195	11,6541	231,22	heating	2,722	32,7382	17,5371	212,49	cooling	2,722	24,8047	8,1903	232,24	heating
2,724	23,5336	3,1584	284,82	cooling	2,724	28,4363	12,1907	227,09	heating	2,724	32,9219	16,2028	233,71	cooling	2,724	24,6714	11,9698	177,55	heating
2,726	24,058	4,1299	278,57	cooling	2,726	28,5245	14,5486	195,36	heating	2,726	33,1987	8,5837	344,08	cooling	2,726	24,0761	7,6534	229,56	heating
2,728	24,3219	2,5239	304,7	cooling	2,728	28,3158	11,3374	237,33	heating	2,728	33,6376	5,7998	389,13	cooling	2,728	24,7192	7,6536	238,55	heating
2,73	23,4299	1,0192	313,27	cooling	2,73	29,5667	13,8881	219,16	heating	2,73	33,4989	8,1824	353,89	cooling	2,73	24,3057	7,286	237,91	heating
2,732	23,4197	-0,9469	340,61	cooling	2,732	28,4754	12,0519	229,58	heating	2,732	33,5773	10,0727	328,56	cooling	2,732	24,7968	6,1389	260,81	heating
2,734	23,9139	2,7534	295,79	cooling	2,734	29,5216	13,1534	228,8	heating	2,734	33,6559	7,6095	364,09	cooling	2,734	24,7252	8,6525	224,67	heating
2,736	23,3271	9,00E-04	326,07	cooling	2,736	28,3807	15,3548	182,08	heating	2,736	33,6209	-1,9135	496,72	cooling	2,736	24,7054	9,4115	213,79	heating
2,738	23,4844	3,4405	280,18	cooling	2,738	28,6621	15,5025	183,95	heating	2,738	33,9108	-3,9198	528,82	cooling	2,738	24,4889	6,6093	249,93	heating
2,74	23,4036	2,1153	297,58	cooling	2,74	27,6517	14,1681	188,48	heating	2,74	33,4577	2,7495	429,25	cooling	2,74	25,3595	8,6551	233,5	heating
2,742	24,7907	3,9941	290,71	cooling	2,742	28,4747	15,8418	176,59	heating	2,742	32,8024	6,5778	366,58	cooling	2,742	24,3178	9,5497	206,44	heating
2,744	24,3361	3,1872	295,63		2,744	27,601	12,0777	216,99	heating	2,744	32,611	8,6304	335,21	cooling	2,744	24,6492	8,7418	222,36	heating
2,746	25,2344	3,9268	297,85		2,746	28,5711	14,0685	202,72	heating	2,746	32,4882	6,9569	356,89	cooling	2,746	24,9437	11,5384	187,39	heating
2,748	25,2913	1,5356	332,07		2,748	28,1061	14,9605	183,76	heating	2,748	32,5424	2,5984	418,57	cooling	2,748	24,9348	8,785	225,75	heating
2,75	25,8592	1,7997	336,32		2,75	28,5548	13,7643	206,75	heating	2,75	33,0421	5,1527	389,85	cooling	2,75	24,9288	7,4451	244,4	heating
2,752	25,1891	-0,1467	354,16	cooling	2,752	28,9528	16,563	173,19	heating	2,752	32,4198	7,3555	350,36	cooling	2,752	25,2228	7,0615	253,87	heating
2,754	26,5294	1,3407	352,1	cooling	2,754	28,4427	14,8154	190,49	heating	2,754	32,3656	7,9255	341,64	cooling	2,754	25,4157	6,4309	265,38	heating
2,756	25,82	2,9451	319,76	cooling	2,756	28,9536	14,7922	197,95	heating	2,756	32,2814	9,2994	321,25	cooling	2,756	25,1818	5,1614	279,86	heating
2,758	25,953	3,0078	320,74	cooling	2,758	28,223	12,3394	222,03	heating	2,758	31,9189	7,0299	347,91	cooling	2,758	25,3512	9,1811	226,03	heating
2,76	26,1061	5,3202	290,56	cooling	2,76	28,4577	11,1642	241,74	heating	2,76	31,838	4,7427	378,75	cooling	2,76	24,7166	7,7184	237,61	heating
2,762	26,2966	2,7431	329,24	cooling	2,762	27,6448	7,9239	275,67	heating	2,762	32,1954	5,4825	373,41	cooling	2,762	25,3483	9,4301	222,51	heating
2,764	26,0236	2,8808	323,5	cooling	2,764	28,4234	10,0516	256,81	heating	2,764	33,1021	4,8054	395,55	cooling	2,764	24,554	8,2993	227,22	heating
2,766	25,9075	3,2791	316,31	cooling	2,766	27,8185	8,7434	266,64	heating	2,766	35,008	8,7561	366,96	cooling	2,766	25,2119	8,1092	239,07	heating
2,768	26,4694	5,2551	296,54	cooling	2,768	28,8051	13,1416	218,95	heating	2,768	35,7455	12,3784	326,64	cooling	2,768	24,9111	8,184	233,82	heating
2,77	26,0743	4,4204	302,69	cooling	2,77	28,3566	12,7374	218,33	heating	2,77	35,7617	9,7136	364,11	cooling	2,77	25,1622	6,5211	260,57	heating
2,772	25,5201	5,9694	273,29	cooling	2,772	29,2588	12,8808	228,94	heating	2,772	36,4885	12,3046	338,05	cooling	2,772	25,2424	3,8692	298,77	heating
2,774	24,8861	1,6746	324,46	cooling	2,774	29,0676	12,335	233,9	heating	2,774	36,1421	15,0499	294,84	cooling	2,774	26,0495	4,7888	297,19	heating
2,776	25,7823	5,0487	289,82	cooling	2,776	29,3399	11,5044	249,31	heating	2,776	36,079	10,454	358,2	cooling	2,776	25,4303	6,0594	270,78	heating
2,778	24,2423	2,058	310,1	cooling	2,778	29,5538	12,3584	240,37	heating	2,778	36,5289	9,8878	372,4	cooling	2,778	25,5473	6,658	264,04	heating
2,78	24,456	1,6728	318,48	cooling	2,78	29,587	11,0396	259,27	heating	2,78	36,8771	14,088	318,56	cooling	2,78	25,7494	8,7094	238,2	heating
2,782	23,9766	-0,0675	336,1	cooling	2,782	29,6212	10,3354	269,59	heating	2,782	36,4594	13,7897	316,89	cooling	2,782	25,3692	7,5029	249,75	heating
2,784	24,0401	-0,8044	347,29	cooling	2,784	29,582	10,3849	268,35	heating	2,784	37,1313	13,629	328,53	cooling	2,784	25,2115	8,3422	235,81	heating
2,786	23,1822	-2,0446	352,63	cooling	2,786	29,5337	14,1953	214,41	heating	2,786	35,8465	5,8583	419,19	cooling	2,786	25,0674	7,6918	242,89	heating
2,788	24,1176	3,3645	290,1	cooling	2,788	29,0142	14,2331	206,62	heating	2,788	36,0261	2,5547	467,88	cooling	2,788	25,4694	7,2148	255,17	heating
2,79	23,254	4,0752	268,09	cooling	2,79	29,2276	17,0046	170,86	heating	2,79	35,5657	8,24	381,97	cooling	2,79	25,0068	4,7207	283,57	heating
2,792	23,4453	3,7035	275,96	cooling	2,792	28,8258	11,7954	238,06	heating	2,792	35,282	10,55	345,72	cooling	2,792	25,7021	6,8621	263,36	heating
2,794	23,2959	3,1533	281,56	cooling	2,794	29,2344	11,7152	244,89	heating	2,794	35,0764	7,5351	384,99	cooling	2,794	25,1904	5,986	268,45	heating
2,796	23,7997	-0,8717	344,87	cooling	2,796	28,9078	10,1457	262,27	heating	2,796	35,6967	7,3322	396,49	cooling	2,796	25,8865	6,3435	273,18	heating
2,798	23,4726	-0,1162	329,74	cooling	2,798	29,6675	13,2201	229,91	heating	2,798	35,1366	1,3171	472,75	cooling	2,798	25,5548	7,1363	257,46	heating
2,8	23,4504	1,5965	305,49	cooling	2,8	29,7372	15,0299	205,59	heating	2,8	35,3358	7,1171	394,45	cooling	2,8	26,0718	8,6766	243,16	heating
2,802	23,8295	3,8663	279,06	cooling	2,802	30,1004	17,6041	174,68	heating	2,802	34,5355	12,9895	301,18	cooling	2,802	25,5815	9,3689	226,63	heating
2,804	23,689	2,5211	295,9	cooling	2,804	30,0233	15,1501	207,91	heating	2,804	33,5639	7,0182	371,07	cooling	2,804	25,7546	9,7535	223,67	heating
2,806	23,4343	1,5813	305,47	cooling	2,806	30,4334	15,6212	207,05	heating	2,806	34,8891	7,1	388,45	cooling	2,806	25,9097	6,8863	265,92	heating
2,808	23,4541	-1,2358	345,13	cooling	2,808	29,5073	14,4692	210,21	heating	2,808	33,5175	5,8785	386,35	cooling	2,808	26,1653	4,3811	304,51	heating
2,81	24,1778	2,8172	298,59	cooling	2,81	29,6609	14,4675	212,38	heating	2,81	34,9237	9,0048	362,31	cooling	2,81	25,9666	5,6956	283,36	heating
2,812	23,277	4,2598	265,83	cooling	2,812	29,337	15,2519	196,89	heating	2,812	34,3098	18,7813	217,07	cooling	2,812	25,7008	5,2432	285,97	heating
2,814	23,5451	8,0087	217,18		2,814	29,3231	11,9254	243,19	heating	2,814	33,8508	14,8137	266,11	cooling	2,814	26,4612	8,4359	251,97	heating
2,816	23,1927	6,165	238,02		2,816	29,1144	12,0283	238,84	heating	2,816	33,669	6,2457	383,34	cooling	2,816	25,9927	8,2564	247,93	heating
2,818	23,5248	3,3778	281,63		2,818	29,3984	12,8699	231,04	heating	2,818	34,6488	11,2146	327,57	cooling	2,818	26,2382	9,0447	240,34	heating
2,82	22,8264	0,1305	317,25		2,82	29,685	16,8039	180,06	heating	2,82	33,5685	6,4832	378,61	cooling	2,82	26,0689	8,6329	243,73	heating
2,822	23,6368	1,6145	307,84		2,822	30,0262	18,8301	156,5	heating	2,822	33,939	11,4382	314,53	cooling	2,822	26,2758	8,9235	242,56	heating
2,824	23,3058	3,3077	279,54		2,824	30,161	19,4565	149,63	heating	2,824	33,7497	13,7934	278,96	cooling	2,824	25,5435	5,2982	283	heating
2,826	23,572	4,5408	266,03		2,826	30,442	14,093	228,54	heating	2,826	34,0031	5,7928	394,34	cooling	2,826	26,1616	6,2093	278,9	heating
2,828	23,4877	4,0081	272,3		2,828	30,5072	13,3055	240,45	heating	2,828	35,816	6,6864	407,19	cooling	2,828	25,7201	5,3012	285,43	heating
2,83	24,5692	3,0135	301,32		2,83	29,7736	11,4413	256,26	heating	2,83	35,1535	9,2248	362,44	cooling	2,83	26,0044	5,6011	285,21	heating

2,832	24,1306	1,5306	315,91		2,832	30,08	14,373	219,56	heating	2,832	36,1275	6,4618	414,68	cooling	2,832	26,0737	7,6282	257,84	heating
2,834	24,3697	-0,9822	354,38		2,834	29,7513	15,35	201,31	heating	2,834	35,8017	10,0728	359,65	cooling	2,834	26,2443	8,4646	248,53	heating
2,836	24,7973	1,812	321,3		2,836	29,7755	14,3335	215,86	heating	2,836	35,6451	7,4048	394,76	cooling	2,836	26,0499	8,6042	243,87	heating
2,838	24,2968	0,5208	332,35		2,838	29,4491	12,3405	239,15	heating	2,838	35,5565	2,7918	458	cooling	2,838	26,022	7,5663	257,98	heating
2,84	24,0514	1,1196	320,55		2,84	30,1371	13,4107	233,81	heating	2,84	37,4129	13,4929	334,37	cooling	2,84	26,6177	6,8019	276,99	heating
2,842	23,7181	0,5804	323,43		2,842	29,6296	12,6823	236,9	heating	2,842	35,8219	7,9518	389,58	cooling	2,842	26,6945	4,562	309,38	heating
2,844	24,113	3,0718	294,12		2,844	30,3547	12,473	249,96	heating	2,844	36,5407	7,4887	406,1	cooling	2,844	26,4509	5,2795	295,95	heating
2,846	23,3968	3,0763	284,05		2,846	30,0557	11,2488	262,89	heating	2,846	36,0949	8,9131	379,96	cooling	2,846	26,3185	2,8757	327,7	heating
2,848	23,7327	5,2492	258,37		2,848	30,715	8,9723	303,93	heating	2,848	35,808	7,0739	401,66	cooling	2,848	26,9966	4,3645	316,36	heating
2,85	23,4572	1,8706	301,75		2,85	30,1855	10,8649	270,07	heating	2,85	36,4073	11,1819	352,61	cooling	2,85	26,4608	4,0964	312,62	heating
2,852	24,4741	2,7512	303,65		2,852	30,1223	11,7034	257,47	heating	2,852	35,908	12,8477	322,35	cooling	2,852	26,5506	7,019	273,02	heating
2,854	23,9436	1,7736	309,9		2,854	29,6784	12,0808	245,99	heating	2,854	36,2178	1,611	483,75	cooling	2,854	26,7156	9,8286	236,06	heating
2,856	25,1004	3,236	305,63		2,856	29,7194	11,8031	250,44	heating	2,856	36,1884	4,7483	439,49	cooling	2,856	26,5295	10,9908	217,21	heating
2,858	24,9254	4,5427	284,92		2,858	29,2808	12,9553	228,21	heating	2,858	35,3872	8,0585	382,02	cooling	2,858	26,1606	7,9464	254,61	heating
2,86	25,0678	4,1952	291,77		2,86	29,3021	10,1223	268,11	heating	2,86	34,4252	5,816	399,91	cooling	2,86	26,6049	6,721	277,95	heating
2,862	24,6257	1,2476	326,79		2,862	29,7385	12,5396	240,41	heating	2,862	35,3177	12,315	321,54	cooling	2,862	26,5357	7,2802	269,16	heating
2,864	25,2974	2,0452	325,03		2,864	29,3557	10,3146	266,17	heating	2,864	34,0907	9,0241	350,39	cooling	2,864	26,4743	6,386	280,8	heating
2,866	24,1751	2,2062	307,09		2,866	30,0325	11,234	262,78	heating	2,866	35,0665	6,1186	404,65	cooling	2,866	26,8331	8,1631	260,98	heating
2,868	24,3239	1,582	317,9		2,868	30,0584	11,738	256,09	heating	2,868	34,8179	10,9639	333,44	cooling	2,868	27,113	7,162	278,89	heating
2,87	24,5282	4,8228	275,45		2,87	30,2944	10,2965	279,54	heating	2,87	34,547	12,3109	310,83	cooling	2,87	26,9538	8,3888	259,51	heating
2,872	24,4923	2,0786	313,31		2,872	30,0482	10,3795	274,94	heating	2,872	34,9371	13,7273	296,48	cooling	2,872	27,0221	9,3667	246,8	heating
2,874	24,7295	3,3052	299,48		2,874	30,5825	14,4056	226,13	heating	2,874	35,4545	18,6113	235,44	cooling	2,874	27,6138	11,3264	227,67	heating
2,876	24,9716	4,9862	279,37		2,876	29,6195	11,7297	250,07	heating	2,876	34,6424	9,2464	355	cooling	2,876	27,4393	9,0544	256,99	heating
2,878	25,3402	5,4442	278,12		2,878	30,2175	10,6548	273,46	heating	2,878	34,9127	6,9919	390,29	cooling	2,878	26,8984	9,6696	240,83	heating
2,88	25,1799	4,472	289,47		2,88	29,4615	12,378	238,8	heating	2,88	34,4678	7,9726	370,36	cooling	2,88	26,8448	8,3258	258,87	heating
2,882	25,2102	6,4909	261,67		2,882	29,6828	9,5526	281,39	heating	2,882	34,1353	5,9051	394,62	cooling	2,882	26,9986	8,0798	264,46	heating
2,884	24,7341	2,6344	308,92		2,884	29,3905	13,1194	227,45	heating	2,884	35,486	8,9869	370,42	cooling	2,884	26,592	8,0453	259,26	heating
2,886	25,0663	4,3183	290,03		2,886	29,427	12,5125	236,44	heating	2,886	35,4909	6,6921	402,56	cooling	2,886	26,6678	6,3052	284,64	heating
2,888	23,6371	2,8449	290,64		2,888	29,3207	8,4473	291,78	heating	2,888	36,1083	-1,407	524,41	cooling	2,888	27,008	6,9255	280,72	heating
2,89	23,8979	2,8004	294,91		2,89	30,3423	9,2008	295,53	heating	2,89	36,305	3,0398	465	cooling	2,89	26,8647	7,0877	276,45	heating
2,892	23,2532	3,0597	282,27		2,892	30,2395	12,5419	247,39	heating	2,892	35,795	6,2793	412,59	cooling	2,892	26,5826	7,7338	263,48	heating
2,894	23,0996	2,78	284,04		2,894	30,731	11,5128	268,64	heating	2,894	35,7942	4,6631	435,17	cooling	2,894	27,4759	8,95	258,97	heating
2,896	22,7665	1,6472	295,22		2,896	31,0917	16,9794	197,27	heating	2,896	37,1748	10,165	377,56	cooling	2,896	27,0383	11,5258	216,84	heating
2,898	23,7461	2,6721	294,58		2,898	30,3566	12,9268	243,64	heating	2,898	36,7177	5,9298	430,37	cooling	2,898	27,1215	9,5668	245,39	heating
2,9	23,1115	0,5723	315,06		2,9	31,0611	10,5753	286,36	heating	2,9	37,0903	11,1037	363,26	cooling	2,9	27,4835	10,1986	241,62	heating
2,902	23,8218	1,8248	307,49		2,902	30,8273	12,5737	255,16	heating	2,902	36,5361	16,117	285,43	cooling	2,902	27,6556	6,8484	290,85	heating
2,904	23,8382	5,8865	250,94		2,904	30,218	11,6813	259,12	heating	2,904	35,3566	9,726	358,28	cooling	2,904	27,1363	4,6891	313,78	heating
2,906	24,1806	5,6944	258,41		2,906	30,2566	14,01	227,1	heating	2,906	36,5509	9,9374	372,02	cooling	2,906	27,5245	5,9355	301,78	heating
2,908	23,984	8,3649	218,33		2,908	30,5022	16,8893	190,29	heating	2,908	36,2603	13,5604	317,31	cooling	2,908	28,2611	9,0148	269,03	heating
2,91	23,5993	4,642	265		2,91	29,8707	10,5585	269,96	heating	2,91	36,5808	12,4818	336,87	cooling	2,91	27,9074	8,5725	270,27	heating
2,912	23,8561	0,9427	320,29	heating	2,912	31,3197	10,3364	293,31	heating	2,912	36,2715	17,9415	256,23	cooling	2,912	27,9519	11,4347	230,89	heating
2,914	23,5505	-0,4652	335,7		2,914	31,1998	12,9393	255,26	heating	2,914	35,4647	15,8194	274,61	cooling	2,914	27,7778	10,6608	239,27	heating
2,916	23,0349	1,0308	307,58		2,916	31,6702	10,1727	300,5	heating	2,916	35,0401	8,7187	367,93	cooling	2,916	28,5794	9,8899	261,25	heating
2,918	23,0945	0,6964	313,09		2,918	31,365	15,6237	220,04	heating	2,918	35,6125	14,0749	301,06	cooling	2,918	27,7451	9,8395	250,29	heating
2,92	23,6191	5,1349	258,38		2,92	30,678	12,0281	260,7	heating	2,92	34,3187	11,4008	320,36	cooling	2,92	28,1055	8,0156	280,83	heating
2,922	22,6657	0,3286	312,24		2,922	30,5838	7,1688	327,31	heating	2,922	34,5149	10,2524	339,15	cooling	2,922	28,0147	8,5475	272,12	heating
2,924	23,881	1,5116	312,69		2,924	30,6196	8,8371	304,49	heating	2,924	34,5528	11,1415	327,25	cooling	2,924	27,852	9,1078	262,02	heating
2,926	23,6962	1,1685	314,9		2,926	29,6623	8,9961	288,88	heating	2,926	34,2426	5,6049	400,31	cooling	2,926	27,3735	7,5058	277,72	heating
2,928	24,3228	-0,2566	343,58		2,928	30,1286	8,4477	303,07	heating	2,928	35,6926	9,1815	370,59	cooling	2,928	28,4289	8,1573	283,37	heating
2,93	24,5755	2,9848	301,81		2,93	30,2966	11,6825	260,2	heating	2,93	35,1499	14,4126	289,88	cooling	2,93	27,8549	10,426	243,63	heating
2,932	25,2281	6,64	259,83		2,932	30,03	6,4064	330,22	heating	2,932	35,0534	10,8206	338,74	cooling	2,932	27,6149	7,7896	277,13	heating
2,934	24,8078	4,4429	284,67		2,934	31,6882	8,7628	320,46	heating	2,934	34,4188	8,9445	356,09	cooling	2,934	28,3936	11,0904	241,87	heating
2,936	25,2042	4,8973	283,86		2,936	31,573	11,3848	282,2	heating	2,936	33,2847	3,2561	419,76	cooling	2,936	28,133	8,2278	278,25	heating
2,938	24,4176	2,3643	308,27		2,938	32,1306	11,4334	289,32	heating	2,938	32,2892	-1,0121	465,5	cooling	2,938	27,9032	5,8838	307,8	heating
2,94	24,8795	-0,1155	349,39		2,94	32,0264	14,0857	250,78	heating	2,94	32,6152	7,7371	347,76	cooling	2,94	28,009	7,4746	287,04	heating
2,942	24,5216	3,9929	286,96		2,942	32,0141	11,7981	282,59	heating	2,942	31,2682	5,6438	358,19	cooling	2,942	28,7078	8,8836	277,11	heating

2,944	24,0727	0,9854	322,73	2,944	31,3847	7,8565	328,89	heating	2,944	32,2327	4,1619	392,39	cooling	2,944	28,3052	6,1704	309,41	heating
2,946	24,9436	2,3315	316,08	2,946	31,6277	11,0175	288,1	heating	2,946	32,8764	5,0335	389,2	cooling	2,946	28,517	9,1541	270,66	heating
2,948	24,9196	3,0729	305,38	2,948	30,5724	12,783	248,67	heating	2,948	33,0767	0,8147	450,97	cooling	2,948	28,0846	7,4558	288,36	heating
2,95	25,0827	3,8515	296,78	2,95	31,02	14,4198	232,05	heating	2,95	34,5125	8,963	357,14	cooling	2,95	28,9366	10,028	264,31	heating
2,952	25,662	4,8263	291,25	2,952	30,5867	16,2056	201,03	heating	2,952	34,1332	17,0928	238,2	cooling	2,952	27,954	12,7264	212,86	heating
2,954	26,4104	7,7354	261,05	2,954	30,7874	10,6518	281,47	heating	2,954	34,7309	13,2122	300,8	cooling	2,954	28,1167	9,0605	266,38	heating
2,956	25,7358	2,3183	327,34	2,956	31,6866	11,3184	284,72	heating	2,956	35,0177	10,9406	336,56	cooling	2,956	28,3738	9,2736	266,99	heating
2,958	26,371	4,2276	309,53	2,958	31,3417	11,5152	277,14	heating	2,958	34,8429	6,8913	390,72	cooling	2,958	27,6585	7,8641	276,7	heating
2,96	24,9328	2,4187	314,71	2,96	31,6578	12,8844	262,42	heating	2,96	35,0499	5,0334	419,59	cooling	2,96	27,4509	5,6319	305	heating
2,962	24,7547	2,6668	308,76	2,962	31,5691	13,796	248,44	heating	2,962	35,8554	13,2322	316,24	cooling	2,962	28,3113	7,7242	287,78	heating
2,964	23,6009	4,3986	268,42	2,964	31,5823	12,7469	263,29	heating	2,964	34,9589	11,2018	332,09	cooling	2,964	27,6512	9,8275	249,15	heating
2,966	23,7626	3,9631	276,77	2,966	31,1097	10,6544	285,93	heating	2,966	35,4692	9,0409	369,43	cooling	2,966	27,6963	8,669	265,97	heating
2,968	23,0701	0,0367	321,97	2,968	31,5477	14,4994	238,31	heating	2,968	35,159	9,7275	355,49	cooling	2,968	28,8213	15,5435	185,61	heating
2,97	23,2362	-1,016	339,01	2,97	30,5836	12,8209	248,3	heating	2,97	34,8413	6,4855	396,37	cooling	2,97	28,2219	10,9854	240,94	heating
2,972	22,8322	-2,6147	355,71	2,972	31,3718	15,4821	222,11	heating	2,972	35,2471	6,5552	401,07	cooling	2,972	28,4334	7,6616	290,36	heating
2,974	23,5045	-1,3766	347,8	2,974	30,7805	15,5757	212,54	heating	2,974	35,1586	4,5481	427,89	cooling	2,974	28,4536	8,0604	285,07	heating
2,976	23,0283	2,4953	287,02	2,976	31,5505	11,8143	275,88	heating	2,976	35,4991	1,2606	478,6	cooling	2,976	29,1007	9,5694	273,02	heating
2,978	22,9135	1,5512	298,61	2,978	31,9729	11,7027	283,35	heating	2,978	35,6009	9,8916	359,38	cooling	2,978	28,5728	11,1711	243,25	heating
2,98	23,6374	4,3477	269,64	2,98	32,3215	10,8851	299,65	heating	2,98	34,5281	13,2078	298,03	cooling	2,98	28,4907	13,5758	208,49	heating
2,982	23,3432	2,7461	287,92	2,982	32,8615	12,1203	289,93	heating	2,982	34,4819	10,0953	340,89	cooling	2,982	28,4781	8,7123	276,3	heating
2,984	23,2383	3,0432	282,3	2,984	33,4882	16,3786	239,17	heating	2,984	34,9659	11,478	328,33	cooling	2,984	29,5856	9,115	286,15	heating
2,986	23,269	2,1413	295,33	2,986	33,0695	16,5725	230,6	heating	2,986	34,2737	6,8641	383,15	cooling	2,986	28,7092	11,0214	247,25	heating
2,988	23,62	2,1934	299,51	2,988	33,2143	12,4904	289,69	heating	2,988	35,0251	9,8991	351,22	cooling	2,988	29,0863	9,2038	277,93	heating
2,99	23,1381	-1,2186	340,47	2,99	33,1624	13,0827	280,68	heating	2,99	34,8395	18,4944	228,48	cooling	2,99	29,0122	12,9583	224,41	heating
2,992	23,5057	1,7752	303,76	2,992	32,2297	9,159	322,49	heating	2,992	34,3889	15,7605	260,4	cooling	2,992	27,8916	11,5786	228,03	heating
2,994	23,0828	0,7971	311,52	2,994	32,5454	12,0507	286,49	heating	2,994	34,8522	11,0401	332,86	cooling	2,994	28,2488	13,0498	212,46	heating
2,996	23,6001	3,6828	278,41	2,996	31,8142	10,9596	291,52	heating	2,996	35,2621	8,4889	374,25	cooling	2,996	28,2664	13,0194	213,13	heating
2,998	22,9058	4,0146	264,07	2,998	31,8688	9,3001	315,48	heating	2,998	34,9919	2,779	450,29	cooling	2,998	27,5802	10,9657	232,25	heating
3	23,5911	3,0564	287,04	3	31,8628	10,6118	297,06	heating	3	35,2642	9,4492	360,86	cooling	3	27,2138	7,2598	278,93	heating
3,002	23,492	1,5214	307,12	3,002	32,317	13,5456	262,4	heating	3,002	34,3846	11,3114	322,53	cooling	3,002	28,4548	12,432	223,97	heating
3,004	23,3965	-1,0894	342,28	3,004	32,0744	12,1242	278,87	heating	3,004	34,5921	6,5349	392,2	cooling	3,004	28,0577	7,5983	285,99	heating
3,006	23,3209	-1,6906	349,62	3,006	32,7511	14,8148	250,72	heating	3,006	35,3814	8,0596	381,92	cooling	3,006	29,0512	9,2828	276,33	heating
3,008	24,1442	2,993	295,66	3,008	32,2852	12,0232	283,23	heating	3,008	34,5719	6,603	390,96	cooling	3,008	29,0048	11,0894	250,43	heating
3,01	23,4849	6,3632	239,33	3,01	33,2568	10,4853	318,31	heating	3,01	35,0846	9,1454	362,59	cooling	3,01	29,9487	11,3735	259,65	heating
3,012	23,8263	6,2194	246,12	3,012	33,047	12,2722	290,4	heating	3,012	34,1709	8,3954	360,3	cooling	3,012	29,2453	11,2423	251,65	heating
3,014	24,0397	6,0592	251,34	3,014	33,073	11,248	305,08	heating	3,014	33,7994	1,1139	456,9	cooling	3,014	28,9597	10,8013	253,83	heating
3,016	24,3739	0,9328	327,67	3,016	33,1812	12,6766	286,62	heating	3,016	34,2184	3,5039	429,34	cooling	3,016	28,9094	8,8877	279,87	heating
3,018	24,1893	2,0818	309,03	3,018	33,0787	13,3902	275,22	heating	3,018	34,0148	10,7724	324,89	cooling	3,018	29,0734	10,8901	254,18	heating
3,02	23,8421	1,1137	317,71	3,02	32,5789	11,358	296,64	heating	3,02	33,7165	10,505	324,46	cooling	3,02	28,2831	11,4271	235,62	heating
3,022	24,4008	2,203	310,29	3,022	33,0958	11,6726	299,47	heating	3,022	33,8188	14,3341	272,37	cooling	3,022	28,9601	9,5509	271,31	heating
3,024	23,9102	1,6628	310,99	3,024	33,0684	14,1152	264,94	heating	3,024	32,8398	9,9198	320,39	cooling	3,024	29,1649	12,3539	234,99	heating
3,026	23,6088	2,2467	298,61	3,026	32,8962	11,1446	304,05	heating	3,026	33,0014	6,1478	375,37	cooling	3,026	28,6233	8,83	276,68	heating
3,028	23,3411	-0,3837	331,64	3,028	33,1617	13,3961	276,29	heating	3,028	33,53	12,7838	290	cooling	3,028	29,5262	11,9146	246,18	heating
3,03	23,7296	2,0029	303,71	3,03	32,8747	11,4857	298,99	heating	3,03	32,662	12,5862	280,63	cooling	3,03	29,6799	11,7114	251,17	heating
3,032	22,4818	-0,7261	324,41	3,032	33,1553	12,5053	288,66	heating	3,032	33,1168	7,9252	352,14	cooling	3,032	29,2378	10,8753	256,68	heating
3,034	23,6217	2,7182	292,2	3,034	32,8417	14,2256	260,23	heating	3,034	32,9286	7,8993	349,87	cooling	3,034	29,1044	11,0584	252,26	heating
3,036	23,0455	4,4777	259,55	3,036	33,0444	15,6058	243,76	heating	3,036	32,3665	6,1786	366,07	cooling	3,036	29,7926	15,7501	196,29	heating
3,038	23,215	2,6832	287	3,038	32,9198	15,0948	249,17	heating	3,038	32,2625	6,9599	353,69	cooling	3,038	29,4028	12,71	233,34	heating
3,04	22,9369	0,9121	307,87	3,04	33,0929	15,4462	246,67	heating	3,04	32,3343	9,6636	316,9	cooling	3,04	29,6224	14,9168	205,56	heating
3,042	23,5222	-0,2945	332,92	3,042	32,773	11,2042	301,5	heating	3,042	31,6887	2,8247	403,48	cooling	3,042	29,1052	12,5134	231,93	heating
3,044	23,1272	-0,0067	323,38	3,044	33,7593	12,6467	295,12	heating	3,044	32,119	3,1949	404,32	cooling	3,044	30,2906	12,8491	243,81	heating
3,046	23,3348	4,6452	261,25	3,046	33,3554	15,3753	251,33	heating	3,046	31,5484	5,2386	367,77	cooling	3,046	29,3574	13,0767	227,58	heating
3,048	23,2476	8,0828	211,98	3,048	33,3772	13,1472	282,79	heating	3,048	31,3354	4,9204	369,24	cooling	3,048	29,4235	10,4892	264,67	heating
3,05	23,5063	4,77	261,91	3,05	33,6412	12,9813	288,79	heating	3,05	31,8482	6,0715	360,32	cooling	3,05	29,7148	11,0589	260,78	heating
3,052	23,1624	3,2939	277,73	3,052	33,5452	11,3021	310,93	heating	3,052	31,4679	2,6195	403,26	cooling	3,052	29,7404	10,8088	264,64	heating
3,054	22,7794	-3,3106	364,7	3,054	33,4312	11,588	305,34	heating	3,054	32,0611	0,1594	445,94	cooling	3,054	29,2828	10,3654	264,44	heating

3,056	23,7896	-2,7268	370,66		3,056	33,7751	15,0143	262,25	heating	3,056	31,704	8,0799	330,23	cooling	3,056	30,3084	12,3218	251,43	heating
3,058	23,6065	0,2516	326,47		3,058	33,763	17,6453	225,3	heating	3,058	30,7524	9,0229	303,75	cooling	3,058	29,806	14,0596	220,11	heating
3,06	23,6595	5,7785	249,95		3,06	33,402	13,7854	274,21	heating	3,06	30,8118	6,767	336,11	cooling	3,06	29,7781	12,4149	242,71	heating
3,062	23,648	6,2336	243,43		3,062	33,7601	12,1813	301,64	heating	3,062	31,6293	9,4607	309,89	cooling	3,062	30,6319	18,5198	169,31	heating
3,064	24,1719	6,919	241,17		3,064	33,5036	7,3817	365,15	heating	3,064	30,9474	3,639	381,73	cooling	3,064	30,3468	13,9617	229,04	heating
3,066	23,282	0,7105	315,52		3,066	34,0989	9,4258	344,89	heating	3,066	31,6239	6,5058	351,11	cooling	3,066	30,5631	12,2046	256,62	heating
3,068	23,8908	1,1659	317,66		3,068	33,6913	13,4612	282,79	heating	3,068	31,1552	12,0951	266,43	cooling	3,068	29,9457	10,0785	277,71	heating
3,07	23,0567	-0,6282	331,08		3,07	34,0266	15,6919	256,29	heating	3,07	30,6789	9,118	301,39	cooling	3,07	30,7392	9,6856	294,3	heating
3,072	22,9681	-2,2411	352,39		3,072	33,839	15,2318	260,1	heating	3,072	31,6609	9,4697	310,2	cooling	3,072	30,0988	9,5825	286,79	heating
3,074	22,2991	-2,0393	340,21		3,074	33,9328	14,6467	269,59	heating	3,074	31,1054	9,4797	302,3	cooling	3,074	30,0734	13,9721	225,07	heating
3,076	22,5432	-0,3669	320,25		3,076	33,6295	10,9077	317,62	heating	3,076	31,4652	5,3722	364,74	cooling	3,076	29,4654	13,7819	219,23	heating
3,078	22,1077	0,9093	296,32		3,078	34,5486	12,5801	307,09	heating	3,078	31,2963	11,0968	282,36	cooling	3,078	30,5846	17,2383	186,56	heating
3,08	22,3122	3,0418	269,37		3,08	33,9378	12,906	293,99	heating	3,08	30,7415	8,445	311,67	cooling	3,08	29,3201	16,476	179,54	heating
3,082	22,2422	2,9714	269,38		3,082	34,0811	11,3811	317,31	heating	3,082	31,1978	3,0831	393	cooling	3,082	30,1866	13,5904	231,99	heating
3,084	22,9925	0,8041	310,16		3,084	34,2008	11,5037	317,27	heating	3,084	32,6074	6,9198	359,07	cooling	3,084	30,3881	14,4161	223,26	heating
3,086	22,8067	2,5345	283,38		3,086	33,939	9,1043	347,15	heating	3,086	31,8121	5,2542	371,24	cooling	3,086	30,1723	10,0531	281,24	heating
3,088	22,8987	2,0776	291,05		3,088	34,1004	10,4036	331,25	heating	3,088	32,5284	9,3102	324,56	cooling	3,088	30,2709	9,9778	283,67	heating
3,09	23,7342	6,9778	234,23		3,09	33,9961	14,813	268,15	heating	3,09	31,9258	12,0607	277,68	cooling	3,09	30,7988	10,6543	281,59	heating
3,092	23,3428	6,0687	241,47		3,092	33,956	15,285	260,99	heating	3,092	31,6741	3,1698	398,45	cooling	3,092	30,4316	10,7847	274,63	heating
3,094	23,0115	5,7866	240,78		3,094	33,602	11,698	306,18	heating	3,094	32,4379	2,0047	425,41	cooling	3,094	30,1419	10,2498	278,06	heating
3,096	22,876	3,1977	275,07		3,096	34,0727	11,5477	314,87	heating	3,096	31,5829	5,5315	364,16	cooling	3,096	31,1868	16,7098	202,37	heating
3,098	23,0456	2,8096	282,87		3,098	33,5715	6,838	373,69	heating	3,098	31,385	5,0323	368,37	cooling	3,098	30,5235	12,828	247,36	heating
3,1	22,4852	0,5884	306,08		3,1	34,6909	10,9359	332,06		3,1	31,2024	10,4291	290,38	cooling	3,1	31,0779	14,9923	224,85	heating
3,102	22,8426	4,0195	263,12		3,102	33,9729	13,7854	282,19		3,102	31,1953	8,6848	314,66	cooling	3,102	30,0837	12,3247	248,24	heating
3,104	22,2969	0,8218	300,19	heating	3,104	34,4122	13,5814	291,18		3,104	32,3441	5,5078	375,13	cooling	3,104	31,4791	12,6156	263,68	heating
3,106	23,1224	0,6485	314,15		3,106	34,3666	13,41	292,94		3,106	34,3591	10,5423	332,92	cooling	3,106	30,4097	14,3336	224,72	heating
3,108	22,4467	0,024	313,44		3,108	34,363	13,5336	291,16		3,108	34,6018	11,5582	322,11	cooling	3,108	30,4402	14,1487	227,73	heating
3,11	23,472	1,2166	311,1		3,11	34,4083	12,1677	310,89		3,11	35,9816	13,7515	310,74	cooling	3,11	30,0204	9,3094	289,51	heating
3,112	23,3617	5,0495	255,98		3,112	34,6924	17,3406	242,55		3,112	35,898	13,2078	317,17	cooling	3,112	31,0738	11,9293	267,61	heating
3,114	23,4808	6,7336	234,1		3,114	33,8753	14,6661	268,52		3,114	36,1035	4,3533	443,82	cooling	3,114	29,7834	12,5415	241,02	heating
3,116	23,0856	4,9337	253,74	heating	3,116	34,0188	12,1234	306,06		3,116	36,6805	2,5806	476,67	cooling	3,116	30,5884	14,1461	229,84	heating
3,118	23,947	4,5575	271,04	heating	3,118	34,3962	14,7881	274,09		3,118	36,9725	8,2962	400,85	cooling	3,118	30,5733	18,3088	171,44	heating
3,12	22,8318	2,3538	286,25	heating	3,12	33,8061	12,4345	298,74		3,12	36,7552	11,1187	358,36	cooling	3,12	29,9389	11,6188	256,09	heating
3,122	22,8733	-0,6774	329,2	heating	3,122	34,5862	17,8172	234,41		3,122	36,7307	13,0468	331,07	cooling	3,122	30,7743	11,5347	268,94	heating
3,124	23,2032	4,0893	267,18	heating	3,124	34,1669	19,28	208,1	heating	3,124	36,3585	9,2235	379,31	cooling	3,124	30,8403	9,6838	295,74	heating
3,126	22,8115	2,0141	290,72	heating	3,126	34,2337	16,7885	243,86	heating	3,126	36,5459	7,6218	404,32	cooling	3,126	30,9302	7,8374	322,8	heating
3,128	22,7121	2,0214	289,23	heating	3,128	34,474	16,0751	257,19	heating	3,128	36,7615	11,9569	346,73	cooling	3,128	30,4865	6,5503	334,59	heating
3,13	22,5078	-0,7015	324,43	heating	3,13	34,1648	15,0797	266,78	heating	3,13	36,007	10,1646	361,24	cooling	3,13	31,5007	11,5481	278,91	heating
3,132	23,4032	-0,6164	335,76	heating	3,132	34,7837	11,5723	324,46	heating	3,132	36,5458	5,0704	439,98	cooling	3,132	30,6445	6,8574	332,51	heating
3,134	23,3532	1,3132	308,09		3,134	34,7356	16,9729	248,3	heating	3,134	36,5372	5,8949	428,33	cooling	3,134	31,3734	13,0333	256,37	heating
3,136	23,9642	8,1973	220,4		3,136	34,0629	13,5076	287,33	heating	3,136	36,641	10,7725	361,6	cooling	3,136	29,965	8,9589	293,63	heating
3,138	23,5545	5,4731	252,75	heating	3,138	34,4686	12,235	310,79	heating	3,138	36,1655	14,1477	307,78	cooling	3,138	31,6692	9,1927	314,19	heating
3,14	24,614	7,9071	233,54	heating	3,14	34,7838	15,7812	265,63	heating	3,14	36,467	13,5576	320,24	cooling	3,14	30,8574	11,6942	267,87	heating
3,142	22,9775	3,0314	278,82	heating	3,142	33,9466	12,5961	298,45	heating	3,142	36,1918	7,5357	400,57	cooling	3,142	30,9144	12,0166	264,16	heating
3,144	23,6807	1,9382	303,93	heating	3,144	35,0571	16,6506	257,3	heating	3,144	36,3602	5,9711	424,79	cooling	3,144	31,3187	11,0507	283,32	heating
3,146	23,0403	3,632	271,3	heating	3,146	34,7383	17,8179	236,52	heating	3,146	36,0292	7,1412	403,81	cooling	3,146	31,5567	12,7942	262,27	heating
3,148	22,8372	2,9228	278,37	heating	3,148	34,164	13,1497	293,75	heating	3,148	35,8266	8,41	383,24	cooling	3,148	30,6334	9,3502	297,51	heating
3,15	22,481	1,6148	291,68	heating	3,15	34,7449	12,8448	306,13	heating	3,15	35,5881	6,5007	406,6	cooling	3,15	31,2769	10,6026	289	heating
3,152	23,2948	3,0232	283,37	heating	3,152	34,5668	15,5409	265,95	heating	3,152	35,1922	0,4635	485,46	cooling	3,152	31,1823	14,8462	228,35	heating
3,154	22,5039	-1,5287	335,94	heating	3,154	35,1557	14,5115	288,57	heating	3,154	35,7785	0,4124	494,37	cooling	3,154	30,5802	11,5759	265,65	heating
3,156	23,2379	-1,0576	339,62	heating	3,156	35,0332	19,9518	210,82	heating	3,156	35,1694	6,6209	399,06	cooling	3,156	31,3263	18,5687	178,33	heating
3,158	23,5073	3,1756	284,21	heating	3,158	35,3057	17,8737	243,67	heating	3,158	35,0561	11,0714	335,27	cooling	3,158	30,7807	15,4131	214,82	heating
3,16	23,6792	-2,4267	364,92	heating	3,16	35,3385	15,2144	281,31	heating	3,16	34,689	9,7692	348,34	cooling	3,16	31,1905	15,5167	219,1	heating
3,162	24,1982	0,068	337,3		3,162	35,5664	16,3458	268,67	heating	3,162	35,1995	6,7822	397,23	cooling	3,162	30,5852	13,1385	243,88	heating
3,164	23,4571	-2,5317	363,28		3,164	34,8312	14,146	289,15	heating	3,164	34,9391	2,8271	448,88	cooling	3,164	31,1578	11,2241	278,64	heating
3,166	24,1261	-2,8429	376,99		3,166	35,5934	13,2799	311,91	heating	3,166	35,4706	6,3967	406,41	cooling	3,166	31,762	6,4268	354,15	heating

3,168	23,6078	-0,1886	332,64	3,168	35,0472	14,5573	286,42	heating	3,168	35,0887	9,5403	357,13	cooling	3,168	31,4424	10,4926	292,85	heating
3,17	23,0072	4,1458	263,65	3,17	34,2828	12,6744	302,05	heating	3,17	34,9467	9,1696	360,33	cooling	3,17	30,9277	6,3706	343,27	heating
3,172	22,5878	2,2049	284,92	3,172	34,7056	14,3529	284,5	heating	3,172	34,4363	6,0964	396,15	cooling	3,172	32,2608	10,1722	308,77	heating
3,174	23,1267	5,6849	243,81	3,174	34,4102	15,7946	260,22	heating	3,174	34,3311	7,1858	379,45	cooling	3,174	31,351	9,7862	301,44	heating
3,176	21,7272	-2,6996	341,45	3,176	34,5596	14,7557	276,83	heating	3,176	33,9843	9,9515	335,94	cooling	3,176	31,1602	8,8425	311,97	heating
3,178	22,813	-2,0361	347,35	3,178	34,5681	14,5923	279,23	heating	3,178	33,7548	9,1644	343,74	cooling	3,178	31,2379	11,2071	280	heating
3,18	22,2483	-0,043	311,6	3,18	35,634	14,3346	297,73	heating	3,18	34,7821	8,1324	372,52	cooling	3,18	31,1901	7,6655	328,84	heating
3,182	22,5101	1,1326	298,83	3,182	35,2871	12,5889	317,29	heating	3,182	35,5205	7,8494	386,8	cooling	3,182	30,7167	5,4223	353,58	heating
3,184	22,6315	6,5222	225,19	3,184	36,0651	14,4517	302,12	heating	3,184	36,3527	8,7753	385,49	cooling	3,184	31,3556	6,5249	347,1	heating
3,186	22,8154	9,2032	190,28	3,186	36,0741	17,3844	261,25	heating	3,186	36,8217	13,6962	323,26	cooling	3,186	30,9413	5,9802	348,92	heating
3,188	22,6765	4,6869	251,47	3,188	36,5168	18,089	257,59	heating	3,188	37,3798	18,6593	261,68	cooling	3,188	31,5428	7,1232	341,35	heating
3,19	23,2148	3,289	278,53	3,19	35,9486	18,8797	238,6	heating	3,19	37,4478	15,3176	309,35	cooling	3,19	30,7172	11,9752	261,99	heating
3,192	22,7379	-0,1905	320,51	3,192	35,4747	17,9353	245,18	heating	3,192	38,1024	11,4806	372,13	cooling	3,192	31,4195	8,6085	318,86	heating
3,194	23,3445	-4,3389	386,97	3,194	35,2657	16,5701	261,34	heating	3,194	37,643	6,235	439,04	cooling	3,194	31,4146	13,5303	250	heating
3,196	23,5264	3,1732	284,51	3,196	35,0067	17,6605	242,47	heating	3,196	38,4	10,9534	383,66	cooling	3,196	30,6406	10,4841	281,76	heating
3,198	22,9771	1,4802	300,5	3,198	34,9984	19,5851	215,46	heating	3,198	38,4165	17,68	289,87	cooling	3,198	31,7922	11,2713	286,85	heating
3,2	23,9866	3,7258	283,22	3,2	34,9666	18,0585	236,35	heating	3,2	38,1806	15,1715	321,63	cooling	3,2	32,2226	12,2633	279	heating
3,202	23,8597	1,4249	313,61	3,202	35,8276	20,4835	214,49	heating	3,202	38,4324	9,394	405,91	cooling	3,202	31,6238	10,0635	301,38	heating
3,204	23,8992	-1,005	348,12	3,204	35,3694	18,9752	229,17	heating	3,204	38,2894	8,8273	411,84	cooling	3,204	31,4656	7,2794	338,09	heating
3,206	24,5798	-1,6091	366,08	3,206	36,4867	20,2704	226,68	heating	3,206	38,4524	10,7022	387,91	cooling	3,206	32,5185	10,4529	308,44	heating
3,208	24,8043	2,0633	317,89	3,208	37,0575	23,5011	189,5	heating	3,208	38,1379	12,7349	355,1	cooling	3,208	30,9939	4,1591	375,11	heating
3,21	24,4437	-0,546	349,32	3,21	36,9624	21,0324	222,68	heating	3,21	38,1157	9,1703	404,61	cooling	3,21	32,2816	7,3155	348,99	heating
3,212	25,1155	4,1899	292,51	3,212	36,9787	17,944	266,08	heating	3,212	38,6102	7,4152	436,06	cooling	3,212	31,1647	9,4818	303,1	
3,214	24,4362	3,6108	291,11	3,214	36,6775	18,7586	250,48	heating	3,214	38,9365	12,3166	372,11	cooling	3,214	32,1054	6,0757	363,86	
3,216	24,5867	2,8458	303,91	3,216	35,6104	15,2374	284,78	heating	3,216	38,6425	12,3564	367,44	cooling	3,216	31,3117	10,1401	295,95	
3,218	24,1637	6,4271	247,93	3,218	35,4126	15,4946	278,42	heating	3,218	38,7883	13,5882	352,26	cooling	3,218	31,5051	7,7363	332,25	
3,22	24,2667	7,337	236,65	3,22	35,2275	18,8422	229,04	heating	3,22	38,7212	12,8466	361,69	cooling	3,22	31,5043	5,2446	367,07	
3,222	24,0272	7,3501	233,12	3,222	35,0947	17,6363	244,04	heating	3,222	38,3	8,5047	416,49	cooling	3,222	32,2054	8,0846	337,17	
3,224	24,0015	4,6928	269,91	3,224	35,3745	20,1935	212,21	heating	3,224	38,4521	9,9992	397,73	cooling	3,224	31,5122	7,5396	335,1	
3,226	23,8632	1,8209	308,12	3,226	35,1156	19,2928	221,18	heating	3,226	37,7951	13,1035	345,15	cooling	3,226	32,3582	7,4965	347,53	
3,228	24,6934	1,2137	328,21	3,228	35,4105	16,9273	258,37	heating	3,228	37,6185	8,408	408,32	cooling	3,228	32,1902	14,6901	244,62	
3,23	24,0474	3,6856	284,63	3,23	35,8457	17,7448	253,02	heating	3,23	37,9534	4,0799	473,5	cooling	3,23	30,8414	6,2959	343,11	
3,232	24,2271	2,5962	302,37	3,232	35,7591	18,3782	242,96	heating	3,232	37,8524	3,1423	485,2	cooling	3,232	32,8822	10,2833	315,9	
3,234	24,5915	7,4507	239,6	3,234	36,2265	17,5073	261,67	heating	3,234	38,0276	8,2656	416,03	cooling	3,234	31,4451	9,2982	309,58	
3,236	23,9765	2,4336	301,14	3,236	36,1721	20,8794	213,77	heating	3,236	37,884	14,2065	330,98	cooling	3,236	32,0368	8,9909	322,15	
3,238	23,9344	1,5069	313,5	3,238	35,6808	18,8878	234,74	heating	3,238	37,6308	10,145	384,21	cooling	3,238	31,1505	13,7141	243,74	
3,24	23,7391	0,234	328,57	3,24	36,0119	19,8838	225,45	heating	3,24	37,5828	1,6751	501,94	cooling	3,24	31,7044	14,748	237,02	
3,242	23,8723	0,367	328,57	3,242	35,8276	21,7821	196,34	heating	3,242	37,7859	-0,4282	534,18	cooling	3,242	30,6821	7,4112	325,29	
3,244	23,49	-0,3428	333,15	3,244	35,5561	19,1852	228,84	heating	3,244	37,4014	3,7474	470,43	cooling	3,244	32,6551	12,7017	278,92	
3,246	23,8062	1,9485	305,54	3,246	35,9918	19,661	228,28	heating	3,246	37,34	12,2993	350,03	cooling	3,246	31,0849	7,8553	324,71	
3,248	23,4273	-1,4103	347,19	3,248	35,8928	19,9663	222,63	heating	3,248	36,5156	12,3095	338,37	cooling	3,248	32,6833	8,2275	341,86	
3,25	24,4014	1,953	313,8	3,25	35,8676	18,6595	240,54	heating	3,25	36,9118	7,1521	416	cooling	3,25	31,6979	13,2684	257,62	
3,252	23,4611	2,983	286,25	3,252	36,1571	19,2349	236,55	heating	3,252	37,1608	10,2653	375,96	cooling	3,252	31,7517	8,6747	322,58	
3,254	24,5086	2,2435	311,23	3,254	36,0808	17,6852	257,14	heating	3,254	36,8071	14,1848	316,23	cooling	3,254	32,3572	12,1858	281,97	
3,256	24,4357	4,3915	280,19	3,256	36,4327	17,1116	270,08	heating	3,256	36,9082	15,3792	300,94	cooling	3,256	31,538	11,3298	282,48	
3,258	24,4042	2,742	302,81	3,258	36,394	21,2395	211,84	heating	3,258	37,1297	16,1965	292,61	cooling	3,258	32,0357	9,0476	321,34	
3,26	24,233	2,1078	309,28	3,26	36,0526	20,0824	223,24	heating	3,26	36,6182	11,1757	355,65	cooling	3,26	32,3559	9,4516	320,17	
3,262	24,6118	1,932	317,03	3,262	36,2126	18,5598	246,76	heating	3,262	36,4925	8,1634	396	cooling	3,262	31,8901	11,4383	285,89	
3,264	23,8466	1,461	312,92	3,264	35,5508	15,8817	274,94	heating	3,264	36,7163	12,4757	338,85	cooling	3,264	31,2552	5,2096	364,08	
3,266	23,9041	-0,0923	335,43	3,266	35,5914	14,662	292,56	heating	3,266	36,191	11,3388	347,4	cooling	3,266	33,0682	13,6176	271,89	
3,268	23,9162	2,8307	294,74	3,268	35,7764	18,3187	244,03	heating	3,268	36,8604	7,0974	416,04	cooling	3,268	31,3286	8,46	319,67	
3,27	23,869	1,6362	310,78	3,27	35,0878	19,3178	220,44	heating	3,27	36,3616	2,5512	472,62	cooling	3,27	32,4158	11,7703	288,59	
3,272	23,9824	5,4654	258,84	3,272	35,1532	17,3044	249,5	heating	3,272	37,1034	3,7961	465,59	cooling	3,272	31,4749	13,2767	254,38	
3,274	23,5267	1,9677	301,36	3,274	35,8057	18,3182	244,45	heating	3,274	36,3061	8,8198	384,22	cooling	3,274	31,9913	11,0854	292,23	
3,276	24,2576	3,6837	287,59	3,276	35,7601	16,9184	263,38	heating	3,276	35,7754	6,5131	409,04	cooling	3,276	31,318	8,6436	316,95	
3,278	23,4926	1,357	309,42	3,278	36,2572	18,2639	251,52	heating	3,278	35,7743	1,5192	478,84	cooling	3,278	31,79	10,765	293,9	

3,28	23,545	0,5003	322,13		3,28	36,5126	20,7275	220,65	heating	3,28	36,6998	5,386	437,72	cooling	3,28	31,522	8,9388	315,68	
3,282	23,3553	1,0969	311,14		3,282	36,7896	19,4536	242,33	heating	3,282	36,267	7,1257	407,35	cooling	3,282	32,3577	12,2516	281,05	
3,284	23,6171	5,4571	253,85		3,284	36,9345	20,3172	232,29	heating	3,284	36,1681	14,1242	308,14	cooling	3,284	31,2709	12,406	263,7	
3,286	22,632	4,593	252,16		3,286	36,3027	18,3079	251,54	heating	3,286	35,8066	15,5546	283,09	cooling	3,286	31,9321	5,3037	372,23	
3,288	23,4164	6,4845	236,68		3,288	36,4586	16,8342	274,32	heating	3,288	35,4591	7,1836	395,25	cooling	3,288	32,2044	9,114	322,77	
3,29	22,4495	2,2064	282,97		3,29	36,3729	20,7067	218,99	heating	3,29	36,451	7,6991	401,91	cooling	3,29	31,4822	8,0066	328,16	
3,292	23,0121	-0,8689	333,82		3,292	35,7907	21,7847	195,78	heating	3,292	34,4339	8,8911	357,05	cooling	3,292	32,3574	13,8753	258,35	
3,294	22,5509	0,6856	305,64		3,294	36,174	21,0903	210,85	heating	3,294	34,6808	13,2374	299,75	cooling	3,294	32,431	14,7431	247,25	
3,296	23,225	2,5203	289,42		3,296	36,4918	20,5383	223,01	heating	3,296	33,992	19,7524	199,05	cooling	3,296	32,1892	14,9581	240,87	
3,298	22,692	5,2735	243,48		3,298	36,5036	16,6628	277,35	heating	3,298	34,1234	17,2777	235,48	cooling	3,298	31,7022	9,9294	304,35	
3,3	22,8267	4,4999	256,18		3,3	37,0389	18,2116	263,18	heating	3,3	34,8005	8,5269	367,27	cooling	3,3	32,7523	12,3683	284,94	
3,302	22,6077	0,6993	306,25		3,302	36,7637	19,9915	234,45	heating	3,302	37,0191	9,8411	379,91	cooling	3,302	31,6182	6,4428	351,91	
3,304	23,4463	-1,8116	353,07		3,304	36,3754	17,4902	263,99	heating	3,304	36,6338	6,7644	417,53	cooling	3,304	32,412	12,4894	278,49	
3,306	22,7896	-0,4507	324,86		3,306	36,4405	17,7515	261,25	heating	3,306	36,7381	12,0571	345	cooling	3,306	31,1891	14,295	236,16	
3,308	23,0314	-2,7413	360,26		3,308	36,1963	16,7925	271,24	heating	3,308	36,4939	12,9129	329,63	cooling	3,308	32,0901	13,3049	262,59	
3,31	23,5646	1,5331	307,97		3,31	36,1023	16,9379	267,89	heating	3,31	36,6751	5,9481	429,52	cooling	3,31	31,3577	12,9603	257,17	
3,312	23,1467	0,9722	309,97		3,312	35,9851	18,756	240,84	heating	3,312	38,1	8,58	412,65	cooling	3,312	31,5481	9,1634	312,91	
3,314	23,575	2,3076	297,29		3,314	35,7191	18,9722	234,1	heating	3,314	37,2973	12,9578	340,23	cooling	3,314	31,2828	8,9272	312,5	
3,316	23,7283	2,3345	299,05		3,316	36,2304	19,0514	240,14	heating	3,316	37,8687	13,8384	335,91	cooling	3,316	31,8567	14,1918	246,93	
3,318	24,0662	2,1175	306,81		3,318	36,0755	18,6	244,28	heating	3,318	37,4566	13,133	340,01	cooling	3,318	31,0742	14,7798	227,77	
3,32	23,5872	0,5019	322,7		3,32	36,4993	19,1412	242,64	heating	3,32	37,3801	10,1071	381,24	cooling	3,32	31,8967	10,5175	298,85	heating
3,322	24,3449	4,4911	277,53		3,322	36,4801	20,4913	223,5	heating	3,322	37,1745	7,1314	419,96	cooling	3,322	32,1955	15,2674	236,63	heating
3,324	23,4694	0,2486	324,59		3,324	36,6056	21,3146	213,74	heating	3,324	38,3034	14,8359	328,04	cooling	3,324	31,3292	6,4236	348,14	heating
3,326	24,2928	-0,8068	350,86		3,326	36,4719	21,5283	208,89	heating	3,326	37,2863	11,6595	358,22	cooling	3,326	32,8235	10,0732	318,02	heating
3,328	23,5059	0,0062	328,49		3,328	36,6384	19,9766	232,91	heating	3,328	37,4487	6,281	435,68	cooling	3,328	31,7385	7,9366	332,72	
3,33	24,2947	0,6603	330,37		3,33	36,8964	17,6912	268,46	heating	3,33	37,908	5,6163	451,39	cooling	3,33	31,9921	7,6501	340,26	
3,332	23,7868	4,738	266,27		3,332	37,0717	20,2813	234,7	heating	3,332	37,891	9,067	402,92	cooling	3,332	31,6882	8,6073	322,64	
3,334	23,7447	4,6998	266,22		3,334	36,9819	21,0774	222,32	heating	3,334	37,9298	13,9216	335,6	cooling	3,334	32,1374	13,6002	259,12	
3,336	23,6624	1,5294	309,39		3,336	36,8567	21,5948	213,34	heating	3,336	37,384	14,8208	315,4	cooling	3,336	31,16	8,351	318,84	
3,338	24,7386	2,0186	317,59		3,338	36,7845	19,0361	248,1	heating	3,338	37,3299	8,8924	397,51	cooling	3,338	32,4199	14,4336	251,42	
3,34	23,8901	1,4962	313,03		3,34	36,4826	14,6231	305,56	heating	3,34	37,2792	7,91	410,54	cooling	3,34	30,7662	10,0673	289,34	
3,342	24,0814	-1,3124	354,97		3,342	37,5145	18,009	272,66	heating	3,342	37,2144	10,5143	373,23	cooling	3,342	31,8164	9,0083	318,82	
3,344	24,4291	2,7948	302,41		3,344	36,8537	21,4025	215,99	heating	3,344	37,2363	12,8284	341,19	cooling	3,344	31,1242	11,8835	268,96	
3,346	23,5017	0,252	325		3,346	36,8636	19,1128	248,13	heating	3,346	37,9774	15,2665	317,47	cooling	3,346	31,3226	7,1133	338,41	
3,348	23,9892	2,9863	293,59		3,348	37,1974	19,5527	246,65	heating	3,348	37,6986	8,7374	404,83	cooling	3,348	31,519	12,2823	268,9	
3,35	23,3592	3,0576	283,79		3,35	36,6155	15,2495	298,66	heating	3,35	38,5761	9,3685	408,28	cooling	3,35	31,4198	15,2809	225,6	
3,352	23,7623	1,9251	305,25		3,352	37,0258	14,4418	315,69	heating	3,352	38,5669	15,0068	329,34	cooling	3,352	31,4229	14,1164	241,92	
3,354	23,0981	0,3728	317,67		3,354	37,0068	19,6083	243,2	heating	3,354	37,8078	14,9496	319,52	cooling	3,354	32,4701	13,5686	264,22	heating
3,356	23,5069	1,917	301,8		3,356	36,7331	16,8662	277,71	heating	3,356	37,4822	10,3854	378,77	cooling	3,356	31,8844	15,9852	222,25	heating
3,358	22,708	-2,7056	355,24		3,358	37,0691	17,3365	275,83	heating	3,358	37,577	8,7128	403,48	cooling	3,358	32,0467	9,8208	310,68	heating
3,36	23,5967	-1,5294	351,23		3,36	36,7657	17,9259	263,35	heating	3,36	37,8195	3,9599	473,31	cooling	3,36	33,1135	18,5375	203,75	heating
3,362	22,7967	-2,9121	359,37	cooling	3,362	36,6724	14,1732	314,5	heating	3,362	37,9642	7,1557	430,66	cooling	3,362	31,592	10,6524	292,7	heating
3,364	24,0633	-2,2632	368	cooling	3,364	37,3478	16,1679	296,06	heating	3,364	38,2347	11,6904	371,05	cooling	3,364	32,3071	11,0419	297,26	heating
3,366	23,6756	1,0576	316,17	cooling	3,366	36,9391	18,2795	260,83	heating	3,366	37,8851	8,5806	409,63	cooling	3,366	31,7608	12,0945	274,9	heating
3,368	23,5624	-0,8858	341,75	cooling	3,368	37,5072	18,6863	263,09	heating	3,368	39,2246	5,9437	465,22	cooling	3,368	31,9123	10,5896	298,06	heating
3,37	24,11	0,9897	323,19	cooling	3,37	37,6856	22,0612	218,41	heating	3,37	38,6515	3,0387	497,81	cooling	3,37	31,5793	9,5078	308,53	heating
3,372	24,1567	3,3908	290,28	cooling	3,372	36,9964	18,195	262,82	heating	3,372	39,2025	9,4876	415,37	cooling	3,372	32,0734	14,3499	247,75	heating
3,374	23,6583	4,8227	263,29	cooling	3,374	37,5514	18,3394	268,56	heating	3,374	38,6976	14,7101	335,31	cooling	3,374	31,0409	9,9748	294,47	heating
3,376	23,6255	4,8975	261,79	cooling	3,376	37,7127	23,2631	201,98	heating	3,376	38,5027	11,6553	375,29	cooling	3,376	32,3329	14,3944	250,75	heating
3,378	23,9183	7,5702	228,52	cooling	3,378	37,4066	22,3267	210,79	heating	3,378	38,454	5,7716	456,85	cooling	3,378	30,9184	14,0676	235,55	heating
3,38	23,0557	-0,0384	322,82		3,38	37,5827	21,2254	228,65	heating	3,38	38,3075	6,1451	449,58	cooling	3,38	32,3103	7,8121	342,45	heating
3,382	23,4903	2,0911	299,13		3,382	37,122	15,858	297,24	heating	3,382	38,2492	6,4499	444,51	cooling	3,382	31,9709	10,0036	307,07	heating
3,384	22,5138	-0,79	325,75		3,384	37,1646	11,342	360,96	heating	3,384	37,4026	11,4868	362,26	cooling	3,384	31,8389	6,7864	350,2	heating
3,386	23,1797	1,0522	309,31		3,386	37,5421	15,7855	304,12	heating	3,386	36,7586	9,5501	380,33	cooling	3,386	32,2449	10,0894	309,7	heating
3,388	22,3573	3,1896	267,94		3,388	36,7359	17,489	269,04	heating	3,388	36,2893	5,2295	434,17	cooling	3,388	33,0467	13,6825	270,68	heating
3,39	22,692	4,7242	251,16		3,39	37,2437	17,2637	279,29	heating	3,39	36,9086	6,5133	424,88	cooling	3,39	32,233	14,1252	253,12	heating

3,392	22,6172	2,7986	277,03		3,392	36,8169	17,6279	268,23	heating	3,392	35,6926	6,0767	413,99	cooling	3,392	33,0344	11,5667	300,09	heating
3,394	23,0285	2,6827	284,4		3,394	36,9618	15,5277	299,62	heating	3,394	36,1931	10,1783	363,65	cooling	3,394	33,3487	15,1313	254,65	heating
3,396	22,2315	-3,2149	355,7		3,396	37,0375	15,8715	295,87	heating	3,396	35,8351	11,3963	341,62	cooling	3,396	32,7	7,7453	348,83	heating
3,398	23,5099	-2,446	362,82		3,398	37,3275	16,8995	285,55	heating	3,398	36,149	9,1657	377,19	cooling	3,398	33,4157	12,9789	285,68	heating
3,4	22,7413	-1,0234	332,2		3,4	37,1287	16,4738	288,72	heating	3,4	35,9825	9,4936	370,28	cooling	3,4	32,2375	11,0417	296,29	heating
3,402	22,7784	-3,3346	365,02		3,402	37,5599	17,5176	280,16	heating	3,402	36,2436	14,6676	301,6	cooling	3,402	33,5042	13,347	281,77	heating
3,404	23,2653	1,5117	304,08	cooling	3,404	37,2953	17,1154	282,09	heating	3,404	36,4147	16,7604	274,74	cooling	3,404	32,2422	15,1159	239,4	heating
3,406	23,4074	0,8502	315,32	cooling	3,406	37,2874	16,0019	297,54	heating	3,406	35,8833	15,1401	289,96	cooling	3,406	32,8601	14,0857	262,44	heating
3,408	23,0607	1,4938	301,47	cooling	3,408	37,5057	17,971	273,07	heating	3,408	35,3891	8,775	372,03	cooling	3,408	32,1469	9,8942	311,06	heating
3,41	23,1982	1,6062	301,82	cooling	3,41	37,1972	17,1337	280,46	heating	3,41	35,5084	9,4279	364,57	cooling	3,41	33,2243	13,8371	271	heating
3,412	23,3504	2,1823	295,9	cooling	3,412	37,5973	18,5414	266,37	heating	3,412	35,3865	12,7942	315,81	cooling	3,412	31,738	11,3782	284,6	heating
3,414	23,6314	1,409	310,64		3,414	37,1839	18,4692	261,6	heating	3,414	34,7989	11,7155	322,67	cooling	3,414	33,6378	12,9145	289,68	heating
3,416	23,3806	7,1543	226,82		3,416	37,7092	17,6323	280,64	heating	3,416	35,0089	9,1821	361,02	cooling	3,416	32,224	14,9123	241,99	heating
3,418	22,9972	2,7374	283,2		3,418	37,657	17,279	284,85	heating	3,418	34,5681	5,0805	412,19	cooling	3,418	32,6948	9,4608	324,78	heating
3,42	23,8123	4,4162	271,13		3,42	37,8767	17,1633	289,54	heating	3,42	34,77	4,1859	427,52	cooling	3,42	33,3027	14,1527	267,69	heating
3,422	22,7015	1,1802	300,84		3,422	37,5857	17,1602	285,52	heating	3,422	34,8559	11,1681	331,12	cooling	3,422	33,3508	13,5335	277,02	heating
3,424	22,7965	1,3527	299,75		3,424	37,8664	18,0618	276,84	heating	3,424	34,993	13,8968	294,89	cooling	3,424	33,2324	11,9453	297,56	heating
3,426	22,6431	2,8196	277,1		3,426	37,6837	17,4253	283,18	heating	3,426	35,2107	6,029	407,92	cooling	3,426	33,404	11,6653	303,87	heating
3,428	22,7514	3,4334	270,04		3,428	38,0043	18,2744	275,79	heating	3,428	35,6653	2,5672	462,66	cooling	3,428	33,5885	14,3299	269,21	heating
3,43	22,0417	0,1872	305,49		3,43	37,7427	18,909	263,27	heating	3,43	35,4929	4,7025	430,4	cooling	3,43	32,513	11,1583	298,51	heating
3,432	23,1854	2,2304	292,92		3,432	37,6057	15,8066	304,72	heating	3,432	35,1748	9,0847	364,7	cooling	3,432	33,1627	19,0317	197,53	heating
3,434	22,3493	-0,4554	318,77		3,434	37,9397	16,1137	305,1	heating	3,434	35,202	11	338,31	cooling	3,434	31,3062	14,3014	237,7	heating
3,436	22,9869	-1,4685	341,85		3,436	37,6433	14,4098	324,77	heating	3,436	34,6938	7,62	378,45	cooling	3,436	32,7716	16,4756	227,79	heating
3,438	22,6361	1,2474	298,98		3,438	37,8799	13,6994	338,01	heating	3,438	35,1934	5,9273	409,1	cooling	3,438	31,2731	14,8034	230,22	heating
3,44	23,1058	0,0257	322,63		3,44	37,8256	14,1407	331,08	heating	3,44	35,0243	12,0551	321,07	cooling	3,44	32,7411	11,4771	297,24	heating
3,442	22,7928	1,7745	293,8		3,442	37,9595	14,9877	321,11	heating	3,442	35,1006	18,2531	235,5	cooling	3,442	32,4181	11,8855	287,02	heating
3,444	22,6048	-0,0501	316,68		3,444	37,4968	13,5184	335,18	heating	3,444	34,551	15,5718	265,3	cooling	3,444	33,286	13,2753	279,72	heating
3,446	23,0792	0,7388	312,29		3,446	38,0682	15,2041	319,61	heating	3,446	35,278	12,7766	314,54	cooling	3,446	32,9292	10,1529	318,38	heating
3,448	23,2527	1,954	297,73		3,448	37,556	14,7936	318,18	heating	3,448	35,1278	10,9838	337,5	cooling	3,448	34,2013	13,4143	290,57	heating
3,45	22,6096	3,9242	261,19		3,45	37,7434	15,3813	312,59	heating	3,45	35,1419	14,2228	292,42	cooling	3,45	32,7585	14,3385	257,48	heating
3,452	22,5814	0,2201	312,58		3,452	37,4414	17,2874	281,72	heating	3,452	34,7799	12,4647	311,93	cooling	3,452	32,7309	9,7158	321,72	heating
3,454	23,3827	1,8745	300,65		3,454	37,6717	15,9958	303	heating	3,454	35,0636	9,3645	359,23	cooling	3,454	33,4319	16,6641	234,39	heating
3,456	22,503	-4,3	374,67		3,456	37,6969	15,0793	316,16	heating	3,456	35,4275	8,9523	370,08	cooling	3,456	32,2318	12,4503	276,52	heating
3,458	23,3589	-0,2691	330,28		3,458	37,8061	14,4187	326,92	heating	3,458	34,8372	9,9974	347,22	cooling	3,458	33,0938	14,6428	257,92	heating
3,46	22,9801	0,81	309,91		3,46	37,699	14,4142	325,49	heating	3,46	35,2575	13,6326	302,28	cooling	3,46	32,6595	12,8381	277,07	heating
3,462	23,731	2,0325	303,31		3,462	37,7918	15,3369	313,89		3,462	34,5622	11,0448	328,74	cooling	3,462	33,51	11,7146	304,67	heating
3,464	23,1004	2,4628	288,48		3,464	37,4496	17,2591	282,23		3,464	34,6259	-0,1119	485,58	cooling	3,464	33,2233	8,9967	338,65	heating
3,466	23,7338	3,6021	281,41		3,466	37,2237	13,4011	333		3,466	34,9536	-4,304	548,76	cooling	3,466	34,7403	17,528	240,6	heating
3,468	23,1439	1,1198	307,86		3,468	37,3647	11,9012	355,94		3,468	34,9621	1,2516	471,22	cooling	3,468	33,771	13,3504	285,45	heating
3,47	23,4188	2,5687	291,45		3,47	37,1178	9,8441	381,25		3,47	34,9434	8,9816	362,91	cooling	3,47	35,0014	19,6279	214,9	heating
3,472	22,9076	2,8993	279,69		3,472	37,0087	10,5835	369,39		3,472	34,8194	12,4655	312,47	cooling	3,472	33,0357	18,0901	208,92	heating
3,474	23,9011	2,4663	299,63		3,474	36,9773	13,5423	327,59		3,474	34,5805	5,2116	410,53	cooling	3,474	33,6	15,7025	250,18	heating
3,476	23,5249	6,0417	244,39		3,476	37,1605	17,5077	274,72		3,476	35,1197	0,3657	485,81	cooling	3,476	32,4909	15,5414	236,93	heating
3,478	23,174	1,7732	299,15		3,478	37,0265	16,8831	281,57		3,478	35,2203	2,8547	452,42	cooling	3,478	32,0731	13,1889	263,97	heating
3,48	24,0868	1,4504	316,42		3,48	37,2454	16,4924	290,1		3,48	34,9063	6,9299	391,07	cooling	3,48	32,4016	13,7479	260,75	heating
3,482	23,7438	-1,1705	348,27		3,482	37,0486	15,2426	304,81		3,482	34,6068	6,6773	390,41	cooling	3,482	33,1389	17,3617	220,54	heating
3,484	23,5456	0,9308	316,12		3,484	37,2072	14,1334	322,54		3,484	34,2797	3,7626	426,58	cooling	3,484	32,2052	15,9377	227,4	heating
3,486	23,6237	1,5352	308,76		3,486	37,0939	14,244	319,41		3,486	34,1211	5,7117	397,12	cooling	3,486	32,9947	11,9552	294,1	heating
3,488	24,6167	7,4802	239,54		3,488	37,3988	13,9827	327,32		3,488	33,6923	11,0349	316,72	cooling	3,488	33,5947	15,9227	247,03	heating
3,49	23,6444	3,2859	284,58		3,49	37,3411	14,3155	321,86		3,49	33,1194	9,9204	324,29	cooling	3,49	33,1764	7,4718	359,31	heating
3,492	24,1445	3,2441	292,16		3,492	37,4481	13,7415	331,38		3,492	32,6761	6,5517	365,18	cooling	3,492	33,8333	13,8955	278,7	heating
3,494	23,6441	-0,9205	343,38		3,494	37,1854	16,0113	295,98		3,494	32,46	2,9281	412,81	cooling	3,494	33,3104	11,7583	301,27	heating
3,496	24,3063	-1,4241	359,67		3,496	37,1386	16,2537	291,94		3,496	32,3688	0,8889	440,04	cooling	3,496	33,7273	11,5689	309,74	heating
3,498	23,2811	-1,6096	347,93		3,498	36,9782	16,4259	287,29		3,498	32,4859	6,3505	365,33	cooling	3,498	33,3582	12,9415	285,39	heating
3,5	24,2234	3,4288	290,68		3,5	37,1335	14,1737	320,94		3,5	32,0087	10,5618	299,8	cooling	3,5	33,531	17,3152	226,67	heating
3,502	23,5776	3,7125	277,68		3,502	37,225	13,3655	333,52		3,502	32,0118	7,2441	346,22	cooling	3,502	33,5432	12,0536	300,39	heating

3,504	24,216	3,5852	288,39		3,504	37,4146	14,5529	319,57		3,504	32,2965	4,4106	389,8	cooling	3,504	34,0378	18,0211	223,89	heating
3,506	23,7787	1,822	306,92		3,506	37,3561	18,382	265,23		3,506	31,6269	4,111	384,63	cooling	3,506	32,6983	12,8457	277,51	heating
3,508	25,1391	1,5711	329,45		3,508	37,1368	17,4056	275,81		3,508	30,9744	6,3256	344,55	cooling	3,508	34,2893	11,7029	315,73	heating
3,51	24,6998	5,736	265,09		3,51	37,7376	16,2906	299,8		3,51	30,4829	8,9239	301,36	cooling	3,51	33,7365	14,518	268,65	heating
3,512	24,6823	5,8054	263,87		3,512	37,3801	14,1749	324,37		3,512	30,4117	8,2006	310,48	cooling	3,512	33,2014	10,289	320,28	heating
3,514	25,2905	8,1798	239,18		3,514	37,6173	13,7968	332,98		3,514	30,7886	6,7755	335,67	cooling	3,514	33,9109	15,5418	256,77	heating
3,516	25,1137	5,6317	272,33		3,516	37,1501	15,1437	307,62		3,516	31,2501	5,7022	357,12	cooling	3,516	33,9541	18,6577	213,82	heating
3,518	24,7066	5,6259	266,72		3,518	37,357	15,3524	307,59		3,518	31,5663	5,6274	362,59	cooling	3,518	32,9121	14,8274	252,8	heating
3,52	24,4419	3,1965	296,98		3,52	36,7273	13,5227	324,37		3,52	31,871	10,6172	297,1	cooling	3,52	33,9419	14,7368	268,46	heating
3,522	25,2731	6,8168	257,99		3,522	37,4218	14,8986	314,84		3,522	31,5316	12,2231	269,9	cooling	3,522	33,6423	15,7248	250,46	heating
3,524	24,1331	2,5345	301,92		3,524	36,6399	13,4112	324,7		3,524	31,5044	7,6167	333,91	cooling	3,524	33,97	8,9357	349,94	heating
3,526	24,9681	5,6045	270,67		3,526	37,3119	11,9506	354,51		3,526	31,7233	5,0865	372,34	cooling	3,526	34,2579	15,9522	255,89	heating
3,528	24,3405	2,2608	308,64		3,528	37,0412	14,488	315,26	cooling	3,528	31,1952	3,8312	382,51	cooling	3,528	33,5421	8,4689	350,49	heating
3,53	25,3105	1,7379	329,51		3,53	37,1911	15,1147	308,6	cooling	3,53	31,1941	4,0563	379,35	cooling	3,53	34,4975	7,8585	372,37	heating
3,532	24,0377	-1,6843	359,56		3,532	37,2856	13,9582	326,08	cooling	3,532	32,2159	6,8772	354,2	cooling	3,532	33,956	10,111	333,32	heating
3,534	24,8204	-0,7406	357,3		3,534	37,2707	13,3514	334,36	cooling	3,534	33,7223	5,4645	395	cooling	3,534	34,1403	9,167	349,09	heating
3,536	24,1218	-2,1334	367,01		3,536	36,8575	10,7283	365,25	cooling	3,536	36,4656	4,918	440,99	cooling	3,536	34,4048	12,4484	306,92	heating
3,538	24,1868	1,6025	315,69		3,538	36,6692	11,613	350,25	cooling	3,538	37,7994	5,8739	446,27	cooling	3,538	33,8667	15,6702	254,36	heating
3,54	23,407	3,8666	273,15		3,54	36,3852	15,1161	297,31	cooling	3,54	38,3994	7,3231	434,4	cooling	3,54	33,2369	10,0363	324,31	heating
3,542	24,1641	3,6325	287		3,542	35,4706	12,4603	321,65	cooling	3,542	38,9059	13,2463	358,68	cooling	3,542	35,2846	15,9247	270,62	heating
3,544	23,4039	2,7776	288,33		3,544	36,3518	13,8584	314,42	cooling	3,544	39,0453	13,8377	352,37	cooling	3,544	33,3783	15,0911	255,63	heating
3,546	23,2578	-2,0636	353,96		3,546	35,6179	12,6355	321,26	cooling	3,546	39,1064	3,4234	498,8	cooling	3,546	34,4299	11,34	322,76	heating
3,548	23,945	-0,4476	340,97		3,548	36,2852	11,8864	341,06	cooling	3,548	40,6143	5,9431	484,65	cooling	3,548	34,6619	17,9557	233,53	heating
3,55	23,6046	1,1676	313,64		3,55	36,2079	14,1914	307,76	cooling	3,55	40,8112	13,0712	387,76	cooling	3,55	34,32	11,6035	317,54	heating
3,552	23,511	3,8637	274,64		3,552	36,6327	15,1846	299,81	cooling	3,552	40,8421	15,2402	357,88	cooling	3,552	34,075	13,3662	289,48	heating
3,554	23,5031	4,7639	261,95		3,554	36,3863	12,2507	337,38	cooling	3,554	41,1407	15,8869	353,01	cooling	3,554	34,5164	16,1475	256,77	heating
3,556	24,0425	8,3175	219,81		3,556	36,7863	13,3236	327,97	cooling	3,556	40,4977	10,6883	416,69	cooling	3,556	34,1295	11,4806	316,6	heating
3,558	23,3933	4,7573	260,5		3,558	36,2946	12,6687	330,26	cooling	3,558	41,5505	9,4892	448,17	cooling	3,558	34,4495	12,8539	301,87	heating
3,56	23,6556	5,2125	257,81		3,56	36,672	14,7016	307,11	cooling	3,56	41,8161	18,9505	319,63	cooling	3,56	33,7785	16,653	239,39	heating
3,562	23,3434	0,97	312,75		3,562	36,5225	16,9074	274,19	cooling	3,562	41,7945	18,9524	319,3	cooling	3,562	33,8753	9,8695	335,57	heating
3,564	23,7994	0,2919	328,6		3,564	36,3152	15,1591	295,73	cooling	3,564	41,9631	11,0837	431,65	cooling	3,564	34,9643	15,7522	268,56	heating
3,566	23,1835	0,3543	319,12		3,566	36,8728	15,8948	293,24	cooling	3,566	43,2582	10,1479	462,83	cooling	3,566	33,3193	12,6328	289,17	heating
3,568	23,7128	1,0189	317,23		3,568	36,2641	13,974	311,58	cooling	3,568	43,1352	7,7138	495,14	cooling	3,568	35,5819	13,6261	306,91	heating
3,57	23,5643	0,6217	320,7		3,57	36,7858	13,3686	327,34	cooling	3,57	44,0989	17,9218	365,92	cooling	3,57	34,8248	18,559	227,37	heating
3,572	23,5688	0,8432	317,67		3,572	36,5905	14,9714	302,2	cooling	3,572	44,5144	19,4466	350,41	cooling	3,572	34,6074	14,7619	277,41	heating
3,574	23,3209	-0,3091	330,31		3,574	36,4085	17,124	269,57	cooling	3,574	44,8202	11,5206	465,48	cooling	3,574	34,4587	13,0254	299,61	heating
3,576	24,3091	-0,3937	345,31		3,576	35,8841	14,6106	297,37	cooling	3,576	45,7126	13,0519	456,55	cooling	3,576	35,861	15,349	286,73	heating
3,578	23,5485	1,0438	314,58		3,578	36,4425	16,0415	285,17	cooling	3,578	44,0599	12,5665	440,23	cooling	3,578	33,8719	8,2752	357,8	heating
3,58	23,4074	-0,2632	330,88		3,58	36,1131	13,7223	312,99	cooling	3,58	45,4702	11,2536	478,3	cooling	3,58	35,0244	12,5626	313,98	heating
3,582	23,6643	2,2026	300		3,582	36,468	15,3412	295,32	cooling	3,582	44,5761	9,8932	484,81	cooling	3,582	33,9993	15,4675	259,05	heating
3,584	23,8383	2,0585	304,45		3,584	36,1811	16,1807	279,58	cooling	3,584	44,6225	3,6516	572,71	cooling	3,584	34,6606	11,9233	317,83	heating
3,586	23,0609	1,5468	300,73		3,586	36,2273	14,8261	299,16		3,586	44,7558	6,4229	535,84	cooling	3,586	34,2822	15,0361	269,03	heating
3,588	23,3693	0,9972	312,73		3,588	36,4219	14,1612	311,17		3,588	45,0546	16,783	395,2	cooling	3,588	34,1559	9,9422	338,47	heating
3,59	23,5681	2,1682	299,14		3,59	36,2842	15,406	291,85		3,59	44,3802	14,8005	413,48	cooling	3,59	35,4527	9,3076	365,47	heating
3,592	23,3488	0,2822	322,44		3,592	36,3438	14,8326	300,69		3,592	44,4166	14,0822	424,03	cooling	3,592	34,5005	10,3331	337,82	
3,594	23,1395	0,2467	320,01		3,594	36,4231	14,5884	305,22		3,594	44,0823	7,1467	516,3	cooling	3,594	34,5948	11,3012	325,61	
3,596	23,5601	-2,148	359,36		3,596	36,1114	13,5185	315,82		3,596	44,3406	2,3465	587,02	cooling	3,596	34,6927	10,1265	343,4	
3,598	24,3308	-1,1337	355,96		3,598	36,1298	12,3147	332,9		3,598	44,9893	11,8125	463,76	cooling	3,598	34,9287	12,1486	318,43	
3,6	23,6129	0,8383	318,36	cooling	3,6	36,4304	16,2343	282,31		3,6	44,1188	20,7383	326,83	cooling	3,6	33,4875	5,7329	387,97	
3,602	23,9612	2,7503	296,5	cooling	3,602	36,1759	15,9472	282,77		3,602	44,7439	19,2835	355,9	cooling	3,602	35,4503	11,1496	339,69	
3,604	23,7616	0,6364	323,26	cooling	3,604	36,1671	14,9477	296,62		3,604	44,2686	13,2772	433,21	cooling	3,604	33,905	8,7484	351,65	
3,606	24,253	1,1382	323,11	cooling	3,606	36,3311	12,3913	334,64		3,606	44,4646	6,4917	530,81	cooling	3,606	34,3379	7,604	373,7	
3,608	23,6127	2,0763	301,05	cooling	3,608	36,0433	11,4411	343,9		3,608	44,5151	10,6931	472,78	cooling	3,608	34,171	8,3562	360,85	
3,61	24,7196	3,1814	301,07	cooling	3,61	35,8914	10,3859	356,53		3,61	44,7553	20,3442	341,23	cooling	3,61	35,0823	7,2528	389,01	cooling
3,612	24,0036	5,8722	253,45	cooling	3,612	35,7729	12,9546	318,97	cooling	3,612	43,8574	16,6579	380,21	cooling	3,612	34,4068	6,6785	387,6	cooling
3,614	24,1285	6,6216	244,72		3,614	35,9079	14,3558	301,27	cooling	3,614	44,4806	12,512	446,87	cooling	3,614	34,238	11,116	323,21	cooling

3,616	24,0575	5,5954	258,07	3,616	35,953	13,564	312,96	cooling	3,616	44,0841	10,7564	465,87	cooling	3,616	34,6163	14,2511	284,67	cooling
3,618	24,9348	2,7618	309,95	3,618	36,2749	13,5241	318,02	cooling	3,618	44,0946	14,1315	418,84	cooling	3,618	34,5094	15,0715	271,71	cooling
3,62	24,0418	0,4465	329,83	3,62	36,6285	13,0302	329,87	cooling	3,62	43,4745	18,6201	347,43	cooling	3,62	34,0747	16,0915	251,38	cooling
3,622	24,242	-1,6348	361,72	3,622	36,6033	13,1229	328,22	cooling	3,622	42,8082	13,7084	406,77	cooling	3,622	33,7995	10,7745	321,86	cooling
3,624	24,6928	3,4156	297,42	3,624	36,8657	15,5826	297,51	cooling	3,624	43,3524	4,4016	544,47	cooling	3,624	34,6566	12,0533	315,96	cooling
3,626	24,2667	4,3543	278,35	3,626	36,4112	17,5748	263,3	cooling	3,626	42,4794	10,8027	442,79	cooling	3,626	33,3194	10,2849	321,99	cooling
3,628	23,8674	4,0679	276,77	3,628	36,5728	16,054	286,82	cooling	3,628	41,457	15,7905	358,78	cooling	3,628	33,7207	12,1015	302,2	cooling
3,63	23,7983	2,7152	294,71	3,63	35,8933	14,7943	294,93		3,63	40,8579	15,8176	350,03	cooling	3,63	33,5333	9,3505	338,04	cooling
3,632	24,0461	2,9245	295,25	3,632	35,9198	13,9315	307,36		3,632	41,7827	15,881	362,07	cooling	3,632	33,6863	9,4523	338,76	cooling
3,634	22,9363	0,9114	307,88	3,634	35,5602	15,0714	286,4		3,634	40,4906	6,2775	478,25	cooling	3,634	33,3294	8,9721	340,48	cooling
3,636	23,5047	3,858	274,63	3,636	36,0015	15,9897	279,74		3,636	42,019	3,2938	541,32	cooling	3,636	34,263	13,925	284,29	cooling
3,638	22,5074	0,3271	310,05	3,638	35,8062	13,1015	317,38		3,638	41,5918	12,3257	409,1	cooling	3,638	33,813	13,9077	278,25	cooling
3,64	23,2278	-0,6946	334,4	3,64	36,4319	12,9952	327,61		3,64	41,3087	17,4548	333,44	cooling	3,64	33,7022	13,0155	289,17	cooling
3,642	22,1096	-1,2117	326	3,642	36,3885	16,7094	275,08		3,642	40,7627	15,4159	354,31	cooling	3,642	34,2144	12,7156	300,52	cooling
3,644	22,7976	-0,1909	321,35	3,644	35,9776	17,5527	257,55		3,644	42,0263	16,7503	353,32	cooling	3,644	34,4161	8,8207	357,79	cooling
3,646	22,0043	0,6186	298,94	3,646	36,3824	18,2519	253,44		3,646	41,772	7,9497	472,79	cooling	3,646	33,9519	8,2856	358,78	cooling
3,648	22,4911	3,1519	270,33	3,648	35,5686	14,7425	291,12		3,648	40,5385	11,2956	408,77	cooling	3,648	33,5452	8,2972	352,93	cooling
3,65	22,2633	1,8868	284,83	3,65	35,8245	10,2833	357,03		3,65	39,9078	9,7579	421,45	cooling	3,65	34,1801	11,9747	310,4	cooling
3,652	23,3069	2,3415	293,07	3,652	35,0276	12,3326	317,24		3,652	40,1673	5,9123	478,83	cooling	3,652	33,7444	12,8054	292,7	cooling
3,654	22,4088	1,3537	294,32	3,654	34,8604	14,3808	286,28		3,654	42,1611	12,3679	416,47	cooling	3,654	33,5189	17,506	223,84	cooling
3,656	22,3852	-2,5621	348,73	3,656	33,9986	13,7649	282,84		3,656	40,0594	10,5408	412,63	cooling	3,656	33,4152	13,8158	273,97	cooling
3,658	23,2129	1,6722	301,11	3,658	35,1818	17,149	252,07		3,658	41,6596	11,3144	424,18	cooling	3,658	33,9447	13,7497	282,3	cooling
3,66	22,5192	1,6112	292,26	3,66	34,3241	13,5403	290,53		3,66	40,9924	13,6027	382,87	cooling	3,66	33,3459	11,8924	299,89	
3,662	22,8645	2,6003	283,26	3,662	35,5393	11,3799	337,71		3,662	41,1132	5,8454	492,99	cooling	3,662	33,6584	13,7496	278,3	
3,664	22,7112	2,7241	279,39	3,664	35,3496	13,5083	305,31		3,664	41,4526	-0,2194	582,51	cooling	3,664	33,8597	13,5446	283,98	
3,666	23,1773	2,4768	289,36	3,666	35,5134	13,2243	311,57		3,666	43,4469	10,5511	459,83	cooling	3,666	33,5254	13,4093	281,19	
3,668	22,7047	-0,2167	320,41	3,668	35,6075	15,1799	285,55		3,668	43,9529	15,4546	398,36	cooling	3,668	33,3752	11,4237	306,85	
3,67	23,6445	4,0036	274,55	3,67	35,3679	15,928	271,74		3,67	43,2909	23,6806	274,12	cooling	3,67	34,1806	14,3932	276,6	
3,672	22,7751	-2,4629	352,79	3,672	35,1676	10,4202	345,93		3,672	42,653	16,0921	371,28	cooling	3,672	33,2216	13,6995	272,89	
3,674	23,9178	-1,1044	349,77	3,674	35,0034	10,1615	347,25		3,674	42,2333	5,9693	506,92	cooling	3,674	32,8548	11,0249	305,15	
3,676	22,9189	1,5126	299,23	3,676	34,5125	14,632	277,9		3,676	43,6099	15,2697	396,15	cooling	3,676	33,4266	13,1184	283,88	
3,678	23,4641	2,4628	293,57	3,678	33,4086	12,9418	286,1		3,678	41,2827	18,4289	319,46	cooling	3,678	33,7741	11,0497	317,65	
3,68	23,1564	3,4623	275,29	3,68	34,491	14,6025	278,01		3,68	42,4055	17,7015	345,33	cooling	3,68	33,1805	10,6445	315,02	
3,682	23,0734	2,675	285,14	3,682	33,8401	11,2483	315,8		3,682	42,0275	15,0737	376,77	cooling	3,682	33,4252	9,0699	340,45	
3,684	23,2826	2,4756	290,85	3,684	34,6168	10,6668	334,79		3,684	42,2107	10,6906	440,61	cooling	3,684	33,8156	9,006	346,8	
3,686	23,7833	7,24	231,25	3,686	34,3059	15,1848	267,28		3,686	42,3959	10,6658	443,54	cooling	3,686	33,8775	7,9915	361,85	
3,688	22,8177	8,281	203,2	3,688	34,5248	16,6671	249,62		3,688	43,9723	17,911	364,3	cooling	3,688	33,2889	11,6371	302,66	
3,69	23,0121	3,5678	271,8	3,69	34,3801	14,4235	278,96		3,69	43,9563	8,8545	490,67	cooling	3,69	33,6969	8,383	353,85	
3,692	23,6482	5,6849	251,1	3,692	34,9739	16,1688	262,87		3,692	44,3204	3,9664	564,09	cooling	3,692	33,8806	9,4191	341,94	cooling
3,694	22,8876	0,2678	316,19	3,694	34,5709	16,0469	258,94		3,694	44,4308	5,3981	545,62	cooling	3,694	33,0256	7,4524	357,48	cooling
3,696	23,4449	3,0146	285,59	3,696	34,8468	15,5949	269,11		3,696	43,6429	6,9888	512,37	cooling	3,696	33,486	10,5031	321,27	cooling
3,698	22,9593	2,2278	289,8	3,698	34,4096	18,6388	220,45		3,698	44,0603	12,3767	442,89	cooling	3,698	33,7104	12,4656	296,97	cooling
3,7	23,0674	1,4255	302,52	3,7	33,8716	15,1119	262,23		3,7	42,2854	8,1999	476,47	cooling	3,7	33,2221	11,1787	308,13	cooling
3,702	22,7441	1,4722	297,35	3,702	34,2874	14,2171	280,55	heating	3,702	43,1283	4,3973	541,4	cooling	3,702	32,8093	9,4007	327,22	cooling
3,704	22,6956	2,7837	278,34	3,704	34,2254	13,8987	284,14	heating	3,704	41,5461	10,2825	437,02	cooling	3,704	33,9205	13,5022	285,42	cooling
3,706	22,5722	-1,5335	336,96	3,706	34,3053	14,546	276,21	heating	3,706	40,0787	12,626	383,75	cooling	3,706	32,9296	12,7473	282,12	cooling
3,708	22,9592	1,8052	295,7	3,708	34,2349	15,8178	257,44	heating	3,708	39,3026	9,5145	416,39	cooling	3,708	33,1567	12,0576	294,93	cooling
3,71	22,0704	0,0033	308,47	3,71	34,4469	17,817	232,46	heating	3,71	40,587	16,9626	330,23	cooling	3,71	32,9688	11,5857	298,9	cooling
3,712	22,9804	-0,093	322,53	3,712	34,2041	13,3903	290,95	heating	3,712	39,0983	11,8923	380,3	cooling	3,712	34,3075	11,6609	316,57	cooling
3,714	23,1984	3,7957	271,22	3,714	35,0982	14,4072	289,23	heating	3,714	39,8657	12,16	387,28	cooling	3,714	33,1536	14,2751	263,89	cooling
3,716	22,8652	0,854	307,68	3,716	34,7622	13,6478	295,15	heating	3,716	39,9656	15,1593	346,76	cooling	3,716	33,1421	12,1269	293,76	
3,718	23,4279	3,4214	279,66	3,718	35,1267	13,5597	301,47	heating	3,718	38,9981	10,967	391,83	cooling	3,718	33,6437	11,5614	308,68	
3,72	23,568	6,5802	237,46	3,72	34,733	16,9688	248,32	heating	3,72	39,3966	10,6053	402,46	cooling	3,72	33,8412	11,6195	310,63	
3,722	22,5335	4,3292	254,47	3,722	34,3203	14,0934	282,74	heating	3,722	39,5469	16,4032	323,51	cooling	3,722	33,1002	12,9727	281,35	
3,724	23,042	1,5481	300,45	3,724	34,4325	11,0714	326,55	heating	3,724	40,6705	11,5817	406,62	cooling	3,724	33,5965	12,0068	301,79	
3,726	22,5848	1,264	298,03	3,726	34,989	12,8696	309,2	heating	3,726	39,4923	15,3758	337,11	cooling	3,726	33,765	13,439	284,13	

3,728	22,804	-3,8909	373,16		3,728	34,1693	15,483	261,21	heating	3,728	39,5036	15,5521	334,81	cooling	3,728	32,6803	8,0279	344,6	
3,73	22,4568	1,3814	294,6		3,73	34,537	18,3451	226,34	heating	3,73	39,4933	6,89	455,75	cooling	3,73	33,6824	11,2761	313,21	
3,732	22,0789	1,3085	290,34		3,732	34,4171	22,0025	173,54	heating	3,732	42,021	12,9373	406,55	cooling	3,732	33,1372	11,4954	302,52	
3,734	22,2681	0,1801	308,76		3,734	34,3952	15,0498	270,42	heating	3,734	40,8923	10,8738	419,61	cooling	3,734	33,0955	11,7215	298,78	
3,736	22,5603	1,1031	299,94		3,736	34,9554	15,1913	276,27	heating	3,736	43,3017	11,0157	451,31	cooling	3,736	32,39	12,4125	279,25	
3,738	22,4731	2,0735	285,16		3,738	35,3001	15,9642	270,29	heating	3,738	43,5023	21,9677	301,02	cooling	3,738	33,3907	13,5218	277,74	
3,74	22,6269	2,2233	285,21		3,74	35,8662	17,5034	256,69	heating	3,74	42,7325	20,7406	307,41	cooling	3,74	32,7538	10,8845	305,7	
3,742	23,0538	5,9341	239,31		3,742	35,6331	20,2788	214,63	heating	3,742	42,7236	8,1031	483,94	cooling	3,742	33,0188	12,0014	293,79	
3,744	22,0587	2,6351	271,51		3,744	35,3008	19,8664	215,75	heating	3,744	44,9347	11,4502	468,06	cooling	3,744	32,8302	10,4699	312,56	
3,746	23,1725	3,1585	279,77		3,746	35,122	13,8652	297,14	heating	3,746	44,8788	6,3482	538,6	cooling	3,746	33,9449	12,083	305,6	
3,748	22,2375	1,7392	286,54		3,748	35,7724	15,112	288,8	heating	3,748	44,0315	14,1483	417,72	cooling	3,748	32,9843	16,3641	232,33	
3,75	22,8636	1,1758	303,16		3,75	34,6013	12,2487	312,46	heating	3,75	44,0324	19,7601	339,29	cooling	3,75	32,8374	14,5606	255,48	
3,752	22,0576	2,1859	277,78		3,752	35,558	15,8168	275,95	heating	3,752	43,1791	11,6634	440,54	cooling	3,752	33,6817	14,8157	263,72	
3,754	22,8814	6,249	232,5		3,754	34,9354	20,5167	201,55	heating	3,754	44,325	11,6649	456,54	cooling	3,754	33,3466	12,307	294,1	
3,756	21,8819	4,3691	244,8	heating	3,756	34,7942	15,4829	269,94	heating	3,756	43,3371	15,3576	391,11	cooling	3,756	33,1592	11,3439	304,95	
3,758	22,7013	2,4921	282,49	heating	3,758	35,3338	15,4366	278,13	heating	3,758	45,0625	12,374	456,94	cooling	3,758	33,5277	12,5073	293,83	
3,76	22,6851	2,2417	285,77	heating	3,76	36,2744	16,5637	275,53	heating	3,76	44,1194	15,8851	394,67	cooling	3,76	33,6456	13,4704	282,02	
3,762	22,8106	1,6197	296,22	heating	3,762	36,1814	17,3978	262,57	heating	3,762	43,9079	11,9225	447,11	cooling	3,762	32,8082	6,8152	363,34	
3,764	22,6675	3,5441	267,32	heating	3,764	36,6633	21,0761	217,89	heating	3,764	43,9505	4,8931	545,96	cooling	3,764	34,1043	8,7139	354,92	
3,766	22,5269	2,9429	273,75	heating	3,766	36,2388	21,088	211,79	heating	3,766	46,2678	19,5263	373,81	cooling	3,766	33,7143	7,694	363,72	
3,768	22,7707	1,0407	303,75	heating	3,768	36,4058	15,2852	295,23	heating	3,768	44,4399	20,9362	328,55	cooling	3,768	33,7899	9,599	338,15	
3,77	22,0936	-2,4577	343,19	heating	3,77	35,8746	17,5625	255,98	heating	3,77	44,4779	20,3175	337,73	cooling	3,77	33,2179	10,4395	318,41	
3,772	22,551	-0,3002	319,43	heating	3,772	34,9542	15,1504	276,83	heating	3,772	44,8951	18,8029	364,73	cooling	3,772	34,0557	9,1948	347,52	
3,774	21,8341	-3,1969	349,9	heating	3,774	35,3806	18,2424	239,57	heating	3,774	44,2402	12,7217	440,58	cooling	3,774	33,8385	7,9634	361,69	
3,776	22,3986	1,1023	297,69		3,776	34,6853	19,4978	212,3	heating	3,776	44,8123	14,6147	422,12	cooling	3,776	33,4554	8,9883	342,01	cooling
3,778	21,2004	-0,2168	299,38		3,778	34,5354	17,5382	237,59	heating	3,778	44,6177	23,6269	293,42	cooling	3,778	33,8798	10,083	332,64	cooling
3,78	22,2636	-1,0099	325,33		3,78	34,9134	17,6841	240,84	heating	3,78	45,3695	13,4252	446,53	cooling	3,78	34,628	13,2376	299,01	cooling
3,782	21,9007	-1,6339	328,98		3,782	35,4099	17,7574	246,76	heating	3,782	44,4548	11,4717	461,06	cooling	3,782	33,5164	15,205	255,97	cooling
3,784	22,2525	-0,0684	312,01		3,784	34,8929	15,3946	272,56	heating	3,784	44,5261	13,6294	431,89	cooling	3,784	33,2495	12,3584	292,03	cooling
3,786	22,3941	-0,5751	321,08		3,786	35,9528	18,5125	243,79	heating	3,786	43,5847	9,9923	469,57	cooling	3,786	34,372	14,5637	276,89	cooling
3,788	23,3511	3,6363	275,58		3,788	35,2382	17,8922	242,47	heating	3,788	45,4193	19,9873	355,5	cooling	3,788	33,4373	9,8286	330,01	
3,79	22,6007	3,0693	273,02		3,79	36,1844	16,3644	277,05	heating	3,79	42,5735	15,7526	374,92	cooling	3,79	33,3714	10,8994	314,12	
3,792	22,9237	1,2249	303,32		3,792	35,6803	17,5141	253,94	heating	3,792	42,9284	9,3537	469,32	cooling	3,792	33,3397	12,4155	292,49	
3,794	23,2981	2,9996	283,74		3,794	36,5186	15,4354	294,71	heating	3,794	42,6745	11,8925	430,29	cooling	3,794	33,4456	12,6562	290,6	
3,796	22,9516	-1,4006	340,41		3,796	36,0315	16,733	269,77	heating	3,796	42,11	12,8586	408,89	cooling	3,796	32,7712	9,0019	332,26	
3,798	23,0148	1,5421	300,16		3,798	35,9598	15,6422	284,01	heating	3,798	42,0739	11,45	428,08	cooling	3,798	33,507	11,4211	308,73	
3,8	22,873	3,1838	275,23		3,8	35,7139	12,8784	319,21	heating	3,8	43,9278	18,0185	362,17	cooling	3,8	33,5865	9,0292	343,27	
3,802	23,3346	3,7172	274,22		3,802	36,4765	13,1603	325,93	heating	3,802	42,8081	3,9401	543,32	cooling	3,802	33,8139	14,5227	269,66	cooling
3,804	23,2392	0,3772	319,58		3,804	35,9551	14,5599	299,07	heating	3,804	42,9232	8,333	483,52	cooling	3,804	33,0084	15,8308	240,12	
3,806	23,7934	3,2616	287		3,806	35,9638	15,051	292,33	heating	3,806	42,574	17,8923	345,01	cooling	3,806	34,0516	15,2756	262,46	
3,808	23,9812	-0,7867	346,22		3,808	36,4556	17,7443	261,56	heating	3,808	41,4802	11,4849	419,29	cooling	3,808	33,771	13,8497	278,47	
3,81	24,5934	2,6214	307,14		3,81	35,489	12,48	321,63	heating	3,81	43,1116	9,84	465,09	cooling	3,81	33,0183	9,4476	329,48	
3,812	23,7373	1,803	306,61		3,812	36,2699	12,7647	328,57	heating	3,812	39,9446	1,5413	536,82	cooling	3,812	33,538	7,641	362	
3,814	24,1225	3,3073	290,96		3,814	35,3393	12,9828	312,51	heating	3,814	42,6079	1,4149	575,82	cooling	3,814	34,3681	10,5701	332,66	
3,816	23,7956	5,1823	260,19		3,816	36,4251	15,0372	298,97	heating	3,816	41,5305	15,6508	361,76	cooling	3,816	33,0965	11,4226	302,97	
3,818	23,2533	5,597	246,81		3,818	35,1018	15,9161	268,19	heating	3,818	40,2745	13,5752	373,22	cooling	3,818	33,309	12,5961	289,54	
3,82	22,9104	3,2248	275,18		3,82	35,8874	17,3699	258,85	heating	3,82	39,888	6,6235	464,99	cooling	3,82	33,8323	16,9603	235,85	
3,822	23,1493	2,6625	286,37		3,822	34,9287	15,1887	275,94	heating	3,822	42,854	19,5494	325,76	cooling	3,822	33,6283	13,6256	279,61	
3,824	22,5288	2,5171	279,73		3,824	36,0203	18,5906	243,64	heating	3,824	39,7746	6,3251	467,57	cooling	3,824	33,2522	16,3732	235,94	
3,826	22,3952	0,6319	304,22		3,826	35,0011	19,7269	213,51	heating	3,826	38,3276	12,7527	357,5	cooling	3,826	33,4182	16,1594	241,25	
3,828	22,7921	3,7035	266,83		3,828	35,9186	19,3827	231,15	heating	3,828	37,4632	18,3183	267,62	cooling	3,828	33,2779	13,48	276,75	
3,83	22,8127	1,4568	298,52		3,83	35,4196	18,5538	235,76	heating	3,83	37,56	10,3409	380,48	cooling	3,83	32,9846	11,0641	306,42	
3,832	22,6382	2,2622	284,83		3,832	35,0926	12,3552	317,84	heating	3,832	38,216	9,7625	397,74	cooling	3,832	33,1242	10,3643	318,15	
3,834	22,8576	2,4339	285,49		3,834	36,0115	13,4584	315,26	heating	3,834	42,0856	22,2507	277,26	cooling	3,834	33,5024	9,0572	341,71	
3,836	23,0645	5,0083	252,4		3,836	35,8134	16,0969	275,61	heating	3,836	48,4205	11,7521	512,57	cooling	3,836	33,3027	14,2036	266,98	
3,838	22,7088	0,3913	311,97	heating	3,838	35,9557	18,3154	246,59	heating	3,838	44,9579	27,0291	250,62	cooling	3,838	32,744	14,3921	256,53	

3,84	23,0214	2,5397	286,3		3,84	35,5209	17,4403	252,74	heating	3,84	43,9796	22,9597	293,83	cooling	3,84	34,2016	14,9298	269,39	
3,842	22,5481	0,4695	308,62		3,842	36,5816	18,6348	250,87	heating	3,842	43,8783	5,7751	532,63	cooling	3,842	33,9581	17,6763	227,59	
3,844	22,5564	2,0668	286,41		3,844	35,5645	11,6985	333,61	heating	3,844	45,6746	13,6103	448,21	cooling	3,844	33,6126	15,6144	251,59	
3,846	21,8115	2,4862	270,14		3,846	37,0982	15,3959	303,37	heating	3,846	39,9015	9,9406	418,81	cooling	3,846	34,4765	18,1	228,92	
3,848	22,5374	4,2415	255,75	heating	3,848	36,1729	13,9208	311,05	heating	3,848	44,7155	9,7634	488,58	cooling	3,848	35,047	18,881	225,98	heating
3,85	22,4312	3,1354	269,73	heating	3,85	37,5989	15,7505	305,41	heating	3,85	42,4652	19,098	326,64	cooling	3,85	33,8789	14,761	267,24	heating
3,852	23,1873	4,0256	267,85	heating	3,852	36,9348	17,6303	269,85	heating	3,852	42,2575	12,8233	411,45	cooling	3,852	34,2937	15,2008	266,89	heating
3,854	22,8801	1,0863	304,65	heating	3,854	37,4877	16,8131	289	heating	3,854	41,7963	2,3418	551,52	cooling	3,854	34,0691	15,8712	254,38	heating
3,856	24,2687	1,0683	324,31	heating	3,856	37,4763	14,6814	318,64	heating	3,856	44,7503	18,1922	371,24	cooling	3,856	33,8772	13,9001	279,25	heating
3,858	24,0545	3,2236	291,19	heating	3,858	37,8796	16,682	296,31	heating	3,858	38,9735	11,2396	387,68	cooling	3,858	33,4819	18,4601	209,98	heating
3,86	24,477	1,7372	317,87	heating	3,86	37,6544	15,7244	306,55	heating	3,86	41,7567	9,8765	445,64	cooling	3,86	33,1314	17,1246	223,75	heating
3,862	25,1561	3,9627	296,25		3,862	37,7134	15,108	315,99	heating	3,862	40,5156	10,4796	419,86	cooling	3,862	33,4664	14,7801	261,21	heating
3,864	25,3929	3,0929	311,72		3,864	37,3273	16,9795	284,43	heating	3,864	40,491	7,3987	462,58	cooling	3,864	33,0371	14,4681	259,57	heating
3,866	24,8063	1,214	329,79		3,866	36,3283	11,6863	344,46	heating	3,866	39,9922	9,0266	432,85	cooling	3,866	33,6586	13,1355	286,88	heating
3,868	24,9682	0,8919	336,55		3,868	37,7438	17,8588	277,96	heating	3,868	43,4576	25,0957	256,67	cooling	3,868	34,154	13,909	283	heating
3,87	24,795	1,905	319,97		3,87	37,0052	19,7464	241,25	heating	3,87	42,0891	8,1252	474,77	cooling	3,87	33,8979	15,6686	254,82	heating
3,872	24,4424	-3,8474	395,45		3,872	37,7779	20,4745	241,88	heating	3,872	41,3202	10,5865	429,61	cooling	3,872	33,5291	11,6559	305,76	heating
3,874	24,7459	0,0854	344,72		3,874	37,9635	17,8277	281,47	heating	3,874	40,612	14,5575	364,2	cooling	3,874	35,3486	12,7364	316,08	heating
3,876	23,9598	-1,7088	358,81		3,876	39,141	17,2907	305,43	heating	3,876	40,1295	13,0387	378,69	cooling	3,876	34,1818	11,5904	315,79	heating
3,878	24,5605	0,1116	341,76		3,878	39,1464	13,6332	356,64	heating	3,878	39,5831	14,5095	350,49	cooling	3,878	34,2446	9,5752	344,84	heating
3,88	23,9591	1,4885	314,11		3,88	40,1719	20,291	277,91	heating	3,88	40,9628	16,9902	335,1	cooling	3,88	34,9023	13,9128	293,4	heating
3,882	24,3904	1,6345	318,09		3,882	39,1842	18,1209	294,43	heating	3,882	46,4156	1,8738	622,63	cooling	3,882	35,1743	15,056	281,22	heating
3,884	24,2478	-0,1962	341,69		3,884	39,1281	16,4625	316,83	heating	3,884	43,1175	13,5602	413,17	cooling	3,884	34,5007	14,863	274,51	heating
3,886	24,6837	3,878	290,83		3,886	38,0559	14,7871	325,26	heating	3,886	42,2274	17,1218	350,94	cooling	3,886	34,8984	18,7049	226,36	heating
3,888	23,8929	5,1801	261,58		3,888	37,9148	10,7432	379,82	heating	3,888	41,9985	10,3277	442,71	cooling	3,888	34,9704	17,5751	243,16	heating
3,89	24,9426	7,9381	237,7		3,89	37,7136	13,1693	343,09		3,89	42,135	13,9404	394,12	cooling	3,89	34,8392	16,1623	261,07	heating
3,892	24,151	7,4279	233,76		3,892	37,3399	15,3285	307,69		3,892	40,801	15,534	353,2	cooling	3,892	34,4042	16,6911	247,6	heating
3,894	24,5933	3,6323	293		3,894	37,3612	15,7515	302,07		3,894	47,7014	10,3593	521,99	cooling	3,894	34,2593	11,4282	319,14	heating
3,896	24,9457	7,4681	244,31		3,896	37,9891	16,0471	306,72		3,896	43,977	24,1455	277,21	cooling	3,896	34,8057	11,9789	319,08	heating
3,898	25,1741	7,0404	253,48		3,898	37,433	16,3577	294,6		3,898	43,1801	18,0456	351,34	cooling	3,898	33,6993	13,3119	284,99	heating
3,9	24,9479	6,8554	252,91		3,9	37,3706	12,2844	350,67		3,9	42,9682	3,368	553,55	cooling	3,9	34,6034	15,4645	267,53	heating
3,902	25,2744	4,6946	287,68		3,902	38,3169	18,5322	276,56		3,902	46,4951	16,8416	414,51	cooling	3,902	34,4392	17,6553	234,61	heating
3,904	25,4829	2,9765	314,61		3,904	37,7339	16,5428	296,22		3,904	39,5594	6,7053	459,25	cooling	3,904	33,7972	15,4848	255,98	heating
3,906	25,6512	0,9665	345,06		3,906	37,5654	17,7647	276,78		3,906	40,9314	13,7921	379,37	cooling	3,906	33,7841	11,5312	311,06	heating
3,908	25,6546	4,1754	300,25		3,908	37,3171	16,543	290,39		3,908	39,4721	17,1379	312,2	cooling	3,908	35,3536	13,9408	299,32	heating
3,91	25,1697	0,7248	341,7		3,91	37,9521	15,8625	308,78		3,91	40,0852	9,2283	431,33	cooling	3,91	34,5306	11,9597	315,51	heating
3,912	26,23	5,995	282,86		3,912	37,9134	14,5684	326,33		3,912	39,9303	0,6719	548,77	cooling	3,912	34,822	12,6618	309,77	heating
3,914	25,0341	5,5435	272,45		3,914	38,5738	18,1872	284,97		3,914	44,3863	14,1747	422,31	cooling	3,914	35,45	15,2657	282,15	heating
3,916	25,4585	3,4614	307,49		3,916	38,3653	15,0111	326,46		3,916	42,1235	7,9975	477,03	cooling	3,916	35,4307	12,2698	323,76	heating
3,918	24,6647	0,8966	332,24		3,918	38,6864	14,8534	333,15		3,918	41,1674	16,0653	350,89	cooling	3,918	34,9794	13,5457	299,61	heating
3,92	24,6585	0,0912	343,41		3,92	37,4376	14,3167	323,2		3,92	40,5593	12,275	395,37	cooling	3,92	34,5356	14,7678	276,32	heating
3,922	23,6392	0,8633	318,37		3,922	37,6009	14,1114	328,35		3,922	40,7368	6,5904	477,32	cooling	3,922	35,3154	15,3627	278,91	heating
3,924	24,3437	6,5389	248,88		3,924	37,4224	17,1334	283,61		3,924	40,0789	10,2916	416,38	cooling	3,924	35,0557	16,318	261,93	heating
3,926	23,1363	3,7124	271,52		3,926	37,2108	13,5844	330,26		3,926	40,3568	18,5612	304,67	cooling	3,926	34,8893	16,9768	250,39	heating
3,928	24,0554	1,1633	320		3,928	37,3771	12,0861	353,53		3,928	43,165	11,4505	443,32	cooling	3,928	35,004	14,3664	288,48	heating
3,93	23,3234	-0,2311	329,26		3,93	38,3561	13,4395	348,3		3,93	41,3719	11,9013	411,95	cooling	3,93	35,2848	16,3046	265,31	heating
3,932	23,9702	-3,5801	385,11		3,932	37,8998	15,4454	313,88		3,932	42,0595	9,7331	451,88	cooling	3,932	34,4086	14,7448	274,87	heating
3,934	23,9227	-0,1783	336,9		3,934	38,549	14,7215	333,07		3,934	41,9613	8,6821	465,19	cooling	3,934	35,1999	15,1269	280,59	heating
3,936	23,7579	-1,5276	353,45		3,936	39,1481	16,6559	314,41		3,936	42,8151	15,1452	386,78	cooling	3,936	35,3743	15,2467	281,35	heating
3,938	24,0703	-0,101	337,88		3,938	39,0486	10,4743	399,43		3,938	40,9988	14,6339	368,54	cooling	3,938	34,745	12,2538	314,39	heating
3,94	24,4064	3,9454	286,01		3,94	38,6828	13,4077	353,31	cooling	3,94	44,0752	7,9559	504,89	cooling	3,94	35,5394	13,5793	306,97	heating
3,942	23,6916	3,7814	278,32		3,942	37,5706	13,4436	337,26	cooling	3,942	42,4375	9,5269	460,04	cooling	3,942	36,2182	17,5497	260,96	heating
3,944	23,4396	-0,1932	330,35		3,944	37,8973	14,3139	329,66	cooling	3,944	41,8766	13,6603	394,42	cooling	3,944	35,7444	17,7822	251,09	heating
3,946	24,0378	4,7085	270,2		3,946	37,5789	15,4812	308,89	cooling	3,946	40,0907	11,3576	401,65	cooling	3,946	35,1457	17,0227	253,33	heating
3,948	23,066	2,0802	293,35		3,948	37,5911	14,5851	321,59	cooling	3,948	41,3892	15,8701	356,72	cooling	3,948	35,9009	14,5288	298,75	heating
3,95	24,5718	8,8133	220,28		3,95	37,4277	11,7489	358,95	cooling	3,95	38,7649	10,7231	391,98	cooling	3,95	35,9367	7,7603	393,86	heating

3,952	23,7175	9,2672	201,99		3,952	38,0333	15,3447	317,15	cooling	3,952	40,7891	9,57	436,4	cooling	3,952	35,7999	11,6578	337,47	heating
3,954	25,1571	7,8355	242,13		3,954	36,5912	16,16	285,6	cooling	3,954	40,7488	12,5553	394,1	cooling	3,954	35,4359	13,9468	300,39	heating
3,956	25,0994	8,1821	236,48		3,956	37,6405	19,0361	260,06	cooling	3,956	41,1903	16,0566	351,33	cooling	3,956	35,98	16,824	267,77	heating
3,958	25,8733	9,4217	229,97		3,958	36,796	20,4279	228,8	cooling	3,958	40,6173	13,3066	381,76	cooling	3,958	35,3853	17,0085	256,88	heating
3,96	25,7052	7,2553	257,9	heating	3,96	36,8527	13,6078	324,93	cooling	3,96	43,6508	18,0436	357,95	cooling	3,96	35,107	15,2219	277,97	heating
3,962	26,7684	11,2986	216,24	heating	3,962	36,7838	13,7375	322,15	cooling	3,962	43,5741	5,5472	531,56	cooling	3,962	35,5761	14,158	299,39	heating
3,964	25,8561	9,434	229,56	heating	3,964	37,0997	14,2078	320	cooling	3,964	43,0988	7,5839	496,45	cooling	3,964	35,0811	15,0527	279,97	heating
3,966	26,5735	7,2928	269,51	heating	3,966	36,9412	16,3636	287,64	cooling	3,966	43,3111	13,9539	410,37	cooling	3,966	34,9804	14,5886	285,05	heating
3,968	26,1977	7,2724	264,55	heating	3,968	37,838	19,2668	259,6	cooling	3,968	42,987	16,8401	365,49	cooling	3,968	35,5395	15,3566	282,13	heating
3,97	25,7793	-0,0182	360,61	heating	3,97	38,4767	17,4885	293,38		3,97	42,7363	14,7512	391,19	cooling	3,97	35,6386	15,9223	275,61	heating
3,972	26,6236	3,7457	319,8	heating	3,972	39,0812	10,4607	400,07		3,972	43,9256	13,9382	419,18	cooling	3,972	34,7815	10,6317	337,58	heating
3,974	26,0582	5,4513	288,05	heating	3,974	39,7305	14,6841	350,11	cooling	3,974	45,8549	4,0086	584,95	cooling	3,974	36,0843	12,6045	328,21	heating
3,976	26,3514	6,4962	277,55	heating	3,976	39,0603	13,9052	351,63	cooling	3,976	44,4455	15,4604	405,17	cooling	3,976	36,0073	11,0167	349,33	heating
3,978	26,4004	5,1612	296,89	heating	3,978	40,2289	17,2268	321,54	cooling	3,978	43,8853	24,1566	275,78	cooling	3,978	36,0123	11,6726	340,23	
3,98	26,8899	5,1117	304,43	heating	3,98	38,7481	16,3349	313,3	cooling	3,98	42,4695	13,841	400,18	cooling	3,98	35,6536	13,3544	311,71	
3,982	26,6795	0,4912	366,07	heating	3,982	38,9048	12,4394	369,95	cooling	3,982	46,0527	15,489	427,24	cooling	3,982	36,153	14,2492	306,18	
3,984	27,5606	8,303	269,19		3,984	38,1881	11,9338	367	cooling	3,984	42,8136	2,8499	558,63	cooling	3,984	36,0108	12,3253	331,09	
3,986	26,2983	4,182	309,15	heating	3,986	38,5252	16,5687	306,92	cooling	3,986	42,9622	6,8609	504,64	cooling	3,986	35,6243	13,356	311,28	
3,988	27,0391	3,9154	323,24	heating	3,988	37,6875	16,8103	291,83	cooling	3,988	42,3311	13,2734	406,18	cooling	3,988	35,5763	10,8039	346,28	
3,99	26,0411	4,5489	300,43		3,99	39,3738	20,8011	259,62	cooling	3,99	41,7394	12,5596	407,89	cooling	3,99	35,8865	10,7575	351,27	
3,992	25,7611	2,4617	325,69		3,992	38,7748	19,0138	276,23	cooling	3,992	41,4305	10,3079	435,05	cooling	3,992	35,528	11,0829	341,71	
3,994	25,7273	3,0026	317,66		3,994	39,3888	15,9974	326,98	cooling	3,994	43,7652	20,7486	321,74	cooling	3,994	35,4389	9,0883	368,34	
3,996	25,7391	4,2587	300,26		3,996	39,5139	18,5398	293,19	cooling	3,996	43,8401	12,6598	435,85	cooling	3,996	35,9852	11,1963	346,51	
3,998	25,2184	0,6037	344,08		3,998	39,2582	15,9569	325,72	cooling	3,998	42,5094	15,672	375,15	cooling	3,998	35,1482	10,8018	340,33	
4	26,3554	2,1772	337,98		4	38,94	18,0248	292,36	cooling	4	42,1035	11,8979	422,23	cooling	4	35,6606	13,4593	310,34	
4,002	25,7567	4,868	291,99		4,002	37,9597	17,9907	279,14	cooling	4,002	41,5438	1,7179	556,71	cooling	4,002	36,0441	14,2199	305,07	
4,004	25,6699	0,7303	348,62		4,004	38,2279	17,0704	295,75	cooling	4,004	42,2551	9,2357	461,56	cooling	4,004	36,0123	14,1108	306,15	
4,006	26,0624	5,2618	290,76		4,006	37,6202	14,5503	322,48	cooling	4,006	39,6521	17,9557	303,28	cooling	4,006	35,695	11,9971	331,26	
4,008	25,7136	1,2237	342,33		4,008	37,8649	17,6288	282,87	cooling	4,008	41,0818	16,3785	345,32	cooling	4,008	36,2859	14,3655	306,42	
4,01	25,8025	2,7789	321,84		4,01	37,4206	13,1734	338,94	cooling	4,01	40,308	15,1265	352	cooling	4,01	36,6576	13,4785	324,01	
4,012	25,6743	4,244	299,56		4,012	38,8973	16,515	312,87	cooling	4,012	40,4842	7,1005	466,65	cooling	4,012	36,2402	15,9982	282,95	cooling
4,014	25,9374	4,3417	301,88		4,014	37,5166	12,4136	350,9	cooling	4,014	40,8706	-0,4104	577,05	cooling	4,014	35,8184	13,1005	317,56	
4,016	26,4342	2,0208	341,26		4,016	38,0403	13,6767	340,57	cooling	4,016	42,9134	11,9312	433,09	cooling	4,016	36,5246	10,5704	362,8	
4,018	26,7124	6,7812	278,61		4,018	37,5898	17,1137	286,22	cooling	4,018	41,5116	17,783	331,69	cooling	4,018	36,506	10,1936	367,81	cooling
4,02	26,3974	2,9812	327,32		4,02	37,3324	18,3934	264,74	cooling	4,02	41,8086	20,8601	292,83	cooling	4,02	35,728	11,6124	337,1	cooling
4,022	27,7905	7,5177	283,38		4,022	36,8617	16,5621	283,76	cooling	4,022	41,532	19,1203	313,28	cooling	4,022	35,7802	14,9399	291,32	cooling
4,024	26,559	7,3964	267,86		4,024	37,5599	16,7502	290,89	cooling	4,024	40,5161	11,5557	404,82	cooling	4,024	35,614	16,4717	267,58	cooling
4,026	26,8075	6,3789	285,56		4,026	37,6001	14,6687	320,55	cooling	4,026	39,911	8,6421	437,09	cooling	4,026	35,2234	14,696	286,94	cooling
4,028	26,6065	7,5511	266,37		4,028	38,1048	15,8779	310,7	cooling	4,028	39,0504	10,9755	392,45	cooling	4,028	35,5858	11,0368	343,16	cooling
4,03	26,6039	4,5433	308,37		4,03	38,5306	22,2311	227,84	cooling	4,03	39,0655	11,175	389,87	cooling	4,03	35,5907	9,7648	361,01	cooling
4,032	25,8482	1,9173	334,52		4,032	38,533	18,2216	283,92		4,032	39,424	13,7641	358,69	cooling	4,032	35,4055	6,2396	407,7	cooling
4,034	26,6169	6,0063	288,1		4,034	38,8531	19,5762	269,46		4,034	39,5653	11,5604	391,47	cooling	4,034	35,7808	10,3209	355,89	cooling
4,036	25,9364	7,375	259,46		4,036	38,0394	15,0455	321,42		4,036	40,0366	9,5293	426,45	cooling	4,036	36,0718	12,6627	327,22	cooling
4,038	26,6348	6,202	285,62		4,038	38,8619	16,2955	315,45		4,038	40,878	14,8259	364,17	cooling	4,038	35,8612	13,1524	317,44	cooling
4,04	26,8299	7,7432	266,8		4,04	38,6337	17,3194	297,94		4,04	40,5565	10,6671	417,81	cooling	4,04	35,6226	11,6837	334,63	cooling
4,042	27,2899	0,9296	368,48		4,042	38,4675	19,172	269,72		4,042	41,4781	6,9713	482,35	cooling	4,042	36,0541	12,6216	327,55	cooling
4,044	27,7301	2,2124	356,7		4,044	38,755	18,1313	288,29		4,044	41,9383	11,4422	426,29	cooling	4,044	36,1351	13,0913	322,12	cooling
4,046	27,055	1,2732	360,39		4,046	39,7158	21,801	250,42		4,046	41,3627	16,2938	350,43	cooling	4,046	35,6691	14,624	294,18	cooling
4,048	27,6746	2,7408	348,54		4,048	38,9355	17,1783	304,13		4,048	41,5043	19,3089	310,26	cooling	4,048	35,1061	14,0061	294,95	cooling
4,05	27,505	2,7929	345,44		4,05	39,6231	19,082	287,13		4,05	41,3353	14,8914	369,65	cooling	4,05	35,9405	16,3667	273,61	cooling
4,052	27,369	3,3052	336,38		4,052	38,88	21,944	236,74		4,052	41,5952	5,1766	509,08	cooling	4,052	35,2522	15,5556	275,33	cooling
4,054	26,5701	-0,1873	374,03	cooling	4,054	38,347	18,9931	270,54		4,054	41,2407	4,1168	518,94	cooling	4,054	35,4031	13,2276	309,98	cooling
4,056	27,7045	5,6915	307,71	cooling	4,056	37,8735	18,4711	271,22		4,056	40,8838	12,3242	399,22	cooling	4,056	35,097	13,1729	306,47	cooling
4,058	25,9925	3,9666	307,89	cooling	4,058	37,7718	15,3592	313,29		4,058	40,3855	17,9771	313,24	cooling	4,058	34,9687	9,9921	349,14	cooling
4,06	26,8579	7,5019	270,57	cooling	4,06	37,5488	12,6147	348,54		4,06	40,6178	15,2981	353,93	cooling	4,06	34,544	9,0218	356,76	cooling
4,062	26,031	7,4012	260,42	cooling	4,062	37,899	13,3382	343,32		4,062	40,4015	4,012	508,67	cooling	4,062	35,079	11,5251	329,25	cooling

4,064	27,0311	4,4306	315,92		4,064	37,9175	13,9343	335,25		4,064	40,8882	4,2371	512,33	cooling	4,064	34,7997	12,4045	313,05	cooling
4,066	26,2732	2,713	329,34		4,066	38,7808	10,5426	394,73		4,066	40,3201	12,3989	390,3	cooling	4,066	34,7457	11,9628	318,47	cooling
4,068	26,7942	4,7292	308,44	cooling	4,068	39,0082	13,8562	351,59		4,068	40,0383	15,4684	343,45	cooling	4,068	35,0392	13,164	305,78	cooling
4,07	26,3483	5,947	285,18	cooling	4,07	38,5767	9,5258	406,09		4,07	41,2197	17,6373	329,65	cooling	4,07	34,9394	8,7546	366,03	cooling
4,072	26,6974	7,7033	265,51		4,072	39,4445	13,5347	362,18	cooling	4,072	41,6906	17,4622	338,68	cooling	4,072	35,1413	9,4342	359,35	cooling
4,074	25,9103	4,6964	296,54		4,074	38,4279	13,5848	347,27	cooling	4,074	42,291	14,6413	386,5	cooling	4,074	34,3496	9,2007	351,54	cooling
4,076	26,3492	-0,5483	375,99		4,076	37,7977	11,379	369,3	cooling	4,076	42,0873	18,2631	333,03	cooling	4,076	35,3104	9,5702	359,81	cooling
4,078	26,4215	1,8213	343,87		4,078	37,6101	12,5051	350,93	cooling	4,078	41,5678	19,7403	305,12	cooling	4,078	34,9495	10,518	341,52	cooling
4,08	25,7517	0,8343	348,31	cooling	4,08	37,5676	17,4123	281,74	cooling	4,08	40,8924	12,1593	401,65	cooling	4,08	34,984	11,9892	321,43	cooling
4,082	27,0565	7,2936	276,26	cooling	4,082	36,6503	17,5449	267,07	cooling	4,082	41,356	6,6986	484,46	cooling	4,082	34,4244	11,4238	321,51	cooling
4,084	26,8974	7,3444	273,32	cooling	4,084	37,6333	23,0709	203,56	cooling	4,084	40,8833	4,689	505,94	cooling	4,084	34,9897	10,4322	343,28	cooling
4,086	27,7185	6,8388	291,87		4,086	36,7447	19,4116	242,29	cooling	4,086	41,2149	12,0285	407,98	cooling	4,086	34,3053	7,8825	369,35	cooling
4,088	27,4523	5,0331	313,39		4,088	37,8857	15,547	312,26		4,088	41,3347	17,0337	339,69	cooling	4,088	34,8958	7,8504	378,06	cooling
4,09	28,6184	7,8269	290,63		4,09	37,7159	17,6168	280,96		4,09	41,8945	9,7957	448,69	cooling	4,09	34,4199	10,5393	333,82	cooling
4,092	27,9297	4,5556	326,74		4,092	38,2074	15,8166	312,99		4,092	43,8627	3,7975	560,05	cooling	4,092	33,996	8,9404	350,24	cooling
4,094	28,7784	10,8348	250,82		4,094	38,4147	19,0621	270,52		4,094	44,4777	3,8053	568,54	cooling	4,094	34,5027	12,8313	302,93	cooling
4,096	27,4329	8,0307	271,21		4,096	38,4624	20,5957	249,75		4,096	45,5395	14,0258	440,51	cooling	4,096	34,4109	13,3534	294,35	cooling
4,098	28,4675	8,9671	272,59		4,098	38,2928	16,5571	303,83		4,098	45,3945	20,6421	346	cooling	4,098	34,4229	13,1349	297,58	cooling
4,1	27,0918	7,5544	273,1		4,1	39,2344	15,6837	329,2		4,1	44,9717	13,5926	438,63	cooling	4,1	33,9648	9,4352	342,89	cooling
4,102	27,2375	3,265	335,1		4,102	38,2204	17,5621	288,77		4,102	46,0204	7,7682	534,71	cooling	4,102	34,8939	9,4383	355,83	cooling
4,104	27,2665	2,7969	342,05		4,104	38,0533	14,0602	335,39		4,104	45,5308	3,9323	581,49	cooling	4,104	34,5546	6,91	386,43	cooling
4,106	27,1306	2,8021	340,08		4,106	39,021	21,0969	250,55		4,106	47,4004	10,7957	511,68	cooling	4,106	35,2034	11,5845	330,16	cooling
4,108	26,7071	1,4829	352,6		4,108	38,0637	16,8191	296,97		4,108	47,9798	21,3448	372,32	cooling	4,108	34,2095	10,8814	326,09	cooling
4,11	27,2429	2,9797	339,16		4,11	38,5276	12,8833	358,47		4,11	47,8276	20,844	377,19	cooling	4,11	35,2902	11,4321	333,5	cooling
4,112	26,6604	4,6619	307,51	cooling	4,112	38,1995	13,365	347,15		4,112	47,4536	13,0932	480,31	cooling	4,112	34,3874	11,2309	323,69	cooling
4,114	26,2202	1,8003	341,35	cooling	4,114	38,4501	14,3285	337,19		4,114	49,0143	14,617	480,82	cooling	4,114	34,7067	11,8012	320,18	cooling
4,116	26,8118	7,9869	263,15	cooling	4,116	37,9514	14,7118	324,86		4,116	50,3936	13,3594	517,68	cooling	4,116	34,1059	10,5298	329,56	cooling
4,118	25,9062	3,4478	313,93		4,118	38,8842	19,6863	268,36		4,118	49,9535	19,8462	420,86	cooling	4,118	34,7126	12,2041	314,64	cooling
4,12	26,4201	6,0452	284,81	cooling	4,12	37,7253	11,5681	365,64		4,12	49,9153	15,1486	485,99	cooling	4,12	34,1175	13,523	287,88	cooling
4,122	25,4751	5,726	276,06		4,122	39,0851	11,2088	389,67	cooling	4,122	49,9078	11,8553	531,92	cooling	4,122	34,6273	15,1992	271,58	cooling
4,124	26,2411	6,8501	271,06		4,124	37,6114	12,1791	355,51	cooling	4,124	51,2029	21,5678	414,25	cooling	4,124	34,2548	16,5405	247,62	cooling
4,126	26,135	4,7442	299,01		4,126	38,3033	10,9834	381,89	cooling	4,126	49,8529	19,7778	420,4	cooling	4,126	33,7488	10,3796	326,67	cooling
4,128	26,249	7,5503	261,38		4,128	38,0615	14,5467	328,7	cooling	4,128	49,8882	11,0306	543,17	cooling	4,128	34,6388	11,1122	328,87	cooling
4,13	25,6407	3,531	309,06		4,13	38,0198	15,0822	320,63	cooling	4,13	49,5636	5,8201	611,47	cooling	4,13	34,5463	12,1103	313,62	cooling
4,132	26,6465	6,9977	274,66		4,132	38,2208	15,1529	322,45	cooling	4,132	49,9667	9,2778	568,77	cooling	4,132	34,6755	15,6455	266,01	cooling
4,134	25,3165	4,0482	297,3		4,134	38,6939	19,3161	270,87	cooling	4,134	49,2524	14,7235	482,66	cooling	4,134	33,8785	13,8655	279,75	cooling
4,136	25,8667	0,966	348,07		4,136	37,7727	17,7242	280,25	cooling	4,136	50,4541	22,5843	389,58	cooling	4,136	34,7295	13,085	302,56	cooling
4,138	25,9783	2,5869	326,98		4,138	37,8969	12,5997	353,62	cooling	4,138	51,0634	12,7059	536,18	cooling	4,138	33,8516	8,0197	361,09	cooling
4,14	26,1618	1,8616	339,68		4,14	39,007	15,9167	322,77	cooling	4,14	51,6016	6,2833	633,48	cooling	4,14	34,5253	13,6253	292,15	cooling
4,142	26,1179	2,2536	333,59		4,142	37,9387	10,7874	379,54	cooling	4,142	51,2203	12,0401	547,68	cooling	4,142	33,4208	13,0534	284,71	cooling
4,144	27,051	3,731	325,98		4,144	38,7227	14,8734	333,38	cooling	4,144	50,3438	17,2549	462,53	cooling	4,144	34,3967	13,654	289,95	cooling
4,146	26,8169	2,4301	340,89		4,146	37,5618	14,7427	318,98	cooling	4,146	51,8257	24,1291	387,16	cooling	4,146	33,7252	12,2183	300,63	cooling
4,148	27,4401	-0,9257	396,51	cooling	4,148	38,1074	13,8538	339,03	cooling	4,148	51,0679	25,6948	354,68	cooling	4,148	34,0221	10,3581	330,79	cooling
4,15	28,2181	3,209	349,59	cooling	4,15	37,2949	13,914	326,83	cooling	4,15	52,3525	22,487	417,48	cooling	4,15	33,9843	10,8964	322,73	cooling
4,152	28,4818	1,5127	376,99	cooling	4,152	37,4931	12,797	345,21	cooling	4,152	53,4745	17,6456	500,84	cooling	4,152	33,7522	9,9497	332,72	cooling
4,154	28,8509	6,9983	305,47	cooling	4,154	37,1496	8,3784	402,18	cooling	4,154	53,2446	19,4	473,1	cooling	4,154	33,9689	9,0097	348,89	cooling
4,156	28,3179	8,1587	281,8	cooling	4,156	37,7959	12,9856	346,81	cooling	4,156	51,9449	16,3171	498,02	cooling	4,156	34,354	9,7161	344,4	cooling
4,158	28,9286	8,5178	285,31	cooling	4,158	37,1385	14,2664	319,72	cooling	4,158	53,8781	22,3782	440,32	cooling	4,158	34,1077	14,198	278,31	cooling
4,16	28,8267	5,2827	329,11	cooling	4,16	38,082	14,5086	329,52	cooling	4,16	52,657	17,3948	492,91	cooling	4,16	33,1569	11,2035	306,88	cooling
4,162	28,6763	6,739	306,65	cooling	4,162	37,9553	15,9588	307,48	cooling	4,162	51,074	18,0641	461,43	cooling	4,162	34,5364	15,7132	263,12	cooling
4,164	28,0933	3,6415	341,8	cooling	4,164	37,4144	9,6485	388,13	cooling	4,164	51,6154	22,5507	406,28	cooling	4,164	33,5688	10,0402	328,9	cooling
4,166	29,1315	6,8441	311,54	cooling	4,166	38,3112	13,3763	348,55	cooling	4,166	50,5514	24,3736	365,93	cooling	4,166	34,137	12,9256	296,5	cooling
4,168	27,9682	5,25	317,57	cooling	4,168	38,1	16,415	303,12	cooling	4,168	50,7439	20,5428	422,17	cooling	4,168	33,225	11,4284	304,68	cooling
4,17	28,3695	3,2459	351,19	cooling	4,17	37,8595	17,4647	285,09	cooling	4,17	52,4134	20,3902	447,64	cooling	4,17	34,2485	11,6876	315,37	cooling
4,172	28,414	5,7701	316,53	cooling	4,172	37,7376	15,2564	314,25	cooling	4,172	55,2857	11,3271	614,48	cooling	4,172	33,602	10,2856	325,93	cooling
4,174	28,4364	5,5996	319,23	cooling	4,174	38,2652	15,798	314,06	cooling	4,174	54,0359	22,2914	443,74	cooling	4,174	34,1487	16,3078	249,39	cooling

4,176	27,6344	3,1552	342,18	cooling	4,176	37,9269	11,9593	362,99	cooling	4,176	53,6642	37,6151	224,34	cooling	4,176	33,4722	14,5069	265,11	cooling
4,178	28,4518	4,6508	332,7	cooling	4,178	38,2382	18,2642	279,21	cooling	4,178	51,4141	21,9331	412,1	cooling	4,178	34,0242	14,5032	272,87	cooling
4,18	27,4106	2,5975	346,85	cooling	4,18	37,4783	15,1579	312,01	cooling	4,18	55,3193	17,6449	526,63	cooling	4,18	33,1216	10,7611	312,57	
4,182	27,5588	0,1447	383,21	cooling	4,182	38,259	14,9471	325,87	cooling	4,182	50,7055	3,7887	655,83	cooling	4,182	33,7632	6,5246	380,75	
4,184	27,3816	5,8994	300,29	cooling	4,184	37,5153	15,3217	310,23	cooling	4,184	49,1537	10,7136	537,34	cooling	4,184	33,9142	10,131	332,45	cooling
4,186	27,1005	2,7846	339,9	cooling	4,186	37,709	15,0205	317,15	cooling	4,186	48,0371	19,9617	392,45	cooling	4,186	33,2183	10,1452	322,53	cooling
4,188	27,833	4,9334	320,1	cooling	4,188	37,6788	14,1812	328,46	cooling	4,188	46,6539	21,5328	351,16	cooling	4,188	33,7935	14,6585	267,48	cooling
4,19	27,707	6,6146	294,84	cooling	4,19	37,7496	12,6435	350,94	cooling	4,19	46,4204	13,1185	465,51	cooling	4,19	33,9304	13,6108	284,04	cooling
4,192	28,4663	4,9934	328,12	cooling	4,192	37,2091	10,962	366,9	cooling	4,192	50,136	16,6841	467,61	cooling	4,192	33,3687	13,1813	282,19	cooling
4,194	28,7941	4,0972	345,23	cooling	4,194	37,9077	13,9126	335,42	cooling	4,194	52,979	6,7266	646,54	cooling	4,194	32,9904	7,4664	356,79	cooling
4,196	28,8208	8,0935	289,74	cooling	4,196	37,0287	15,6359	299,04	cooling	4,196	50,7927	12,6956	532,54	cooling	4,196	33,9545	9,9976	334,88	cooling
4,198	28,0278	3,7291	339,66	cooling	4,198	37,1812	13,6412	329,05	cooling	4,198	51,2013	19,4181	444,28	cooling	4,198	33,081	4,9105	393,78	cooling
4,2	29,4604	6,4754	321,3	cooling	4,2	37,1233	17,2957	277,16	cooling	4,2	50,6688	11,1482	552,44	cooling	4,2	33,0729	11,6287	299,76	cooling
4,202	27,9592	2,9245	349,95	cooling	4,202	37,4638	16,7252	289,89	cooling	4,202	52,8911	15,5657	521,75	cooling	4,202	32,2133	12,2778	278,67	cooling
4,204	28,6369	1,6137	377,74	cooling	4,204	36,9231	17,0768	277,42	cooling	4,204	48,2393	14,5444	471,01	cooling	4,204	33,1698	13,8625	269,89	cooling
4,206	28,4583	5,2066	325,02	cooling	4,206	36,8666	13,9826	319,88	cooling	4,206	50,9088	20,4632	425,58	cooling	4,206	32,2883	10,9462	298,33	cooling
4,208	28,7934	5,4003	327	cooling	4,208	37,3007	15,104	310,28	cooling	4,208	49,8648	20,9332	404,42	cooling	4,208	32,9145	12,1936	289,65	cooling
4,21	28,2592	3,9133	340,32	cooling	4,21	37,5235	17,3574	281,89	cooling	4,21	49,5702	15,9081	470,55	cooling	4,21	32,6002	13,3355	269,29	cooling
4,212	28,8917	6,1019	318,57	cooling	4,212	37,1656	20,6118	231,4	cooling	4,212	49,0029	9,4928	552,29	cooling	4,212	32,925	15,5607	242,73	
4,214	28,1853	3,9022	339,44	cooling	4,214	36,6837	17,1872	272,53	cooling	4,214	52,0781	23,6339	397,61	cooling	4,214	32,2766	16,1192	225,86	
4,216	28,3309	5,7123	316,17	cooling	4,216	37,3424	16,1181	296,68	cooling	4,216	51,6185	21,179	425,5	cooling	4,216	33,1865	14,7698	257,44	
4,218	27,7399	8,6527	266,81	cooling	4,218	36,5316	13,9107	316,21	cooling	4,218	49,4654	20,6077	403,39	cooling	4,218	32,7014	16,1511	231,35	
4,22	27,906	3,6354	339,27	cooling	4,22	37,7894	19,3782	257,36	cooling	4,22	50,5084	12,345	533,47	cooling	4,22	32,6911	13,8284	263,67	
4,222	28,4332	7,9473	286,36	cooling	4,222	36,7953	20,9591	221,37	cooling	4,222	49,7631	8,7778	572,91	cooling	4,222	32,7455	15,8353	236,38	
4,224	27,3114	7,777	273,06	cooling	4,224	37,4905	19,2739	254,64		4,224	50,1238	15,8564	479,01	cooling	4,224	33,2304	14,0042	268,75	
4,226	28,227	10,8721	242,6	cooling	4,226	36,6463	16,1006	287,2		4,226	49,0283	21,623	383,09	cooling	4,226	32,6567	16,6786	223,35	
4,228	27,6221	8,7129	264,32	cooling	4,228	37,4971	15,91	301,75		4,228	52,0759	20,5997	439,99	cooling	4,228	32,4469	13,1031	270,4	heating
4,23	27,3832	5,4784	306,2	cooling	4,23	36,6664	16,517	281,66		4,23	49,7895	16,9589	458,92	cooling	4,23	33,5919	14,1906	271,2	heating
4,232	27,0462	3,1766	333,66	cooling	4,232	36,9116	19,3715	245,18		4,232	50,427	13,9855	509,4	cooling	4,232	33,3804	10,0024	326,79	heating
4,234	27,8467	6,5619	297,53	cooling	4,234	36,4496	19,7966	232,78		4,234	49,7466	12,5982	519,28	cooling	4,234	33,1772	10,881	311,67	heating
4,236	26,8729	2,9301	334,68	cooling	4,236	36,5801	15,9241	288,74		4,236	51,4054	19,8771	440,72	cooling	4,236	32,7448	11,103	302,52	
4,238	27,3854	5,1704	310,53	cooling	4,238	36,5296	14,0694	313,96		4,238	47,7424	7,8976	556,97	cooling	4,238	33,7415	14,804	264,72	
4,24	26,946	3,2625	331,06	cooling	4,24	36,4086	9,3286	378,54		4,24	50,1762	8,1688	587,2	cooling	4,24	32,8349	12,39	285,79	heating
4,242	27,59	1,852	359,78	cooling	4,242	37,5804	11,924	358,64		4,242	48,6959	13,9217	486,09	cooling	4,242	33,6937	14,0904	274,03	heating
4,244	27,1167	4,3953	317,61	cooling	4,244	36,6341	12,8275	332,78		4,244	47,8324	20,5842	380,89	cooling	4,244	32,7119	11,0899	302,24	heating
4,246	26,7665	2,2288	343	cooling	4,246	37,101	15,4253	302,99		4,246	46,7098	17,1361	413,4	cooling	4,246	34,2176	11,6596	315,33	heating
4,248	27,3038	2,7636	343,04	cooling	4,248	36,3437	12,5668	332,37		4,248	49,0882	14,9078	477,79	cooling	4,248	32,804	12,0471	290,15	
4,25	26,9269	2,1494	346,35	cooling	4,25	37,0802	12,1015	349,16		4,25	49,3407	-0,3334	694,37	cooling	4,25	33,6027	12,5253	294,63	
4,252	26,9913	3,4003	329,77	cooling	4,252	36,1819	11,2783	348,12		4,252	48,3187	5,9429	592,35	cooling	4,252	33,0679	13,6951	270,8	
4,254	27,5045	5,4403	308,43	cooling	4,254	37,0739	17,6317	271,77	cooling	4,254	49,0958	18,9938	420,78	cooling	4,254	33,6752	15,7079	251,16	heating
4,256	27,4631	9,1981	255,32	cooling	4,256	36,3531	19,0297	242,16		4,256	47,5077	21,7652	359,84	cooling	4,256	33,3364	16,1863	239,73	heating
4,258	26,7478	3,3376	327,24	cooling	4,258	36,3497	17,8754	258,24		4,258	48,2249	20,7115	384,6	cooling	4,258	34,1015	16,8099	241,71	heating
4,26	27,6278	6,4054	296,66	cooling	4,26	36,0174	16,544	272,21		4,26	45,5299	16,1306	410,96	cooling	4,26	33,4453	17,8504	217,99	heating
4,262	27,0171	2,7437	339,31	cooling	4,262	36,5737	17,2219	270,51		4,262	48,4359	16,6819	443,87	cooling	4,262	32,9707	12,4041	287,49	heating
4,264	27,1099	2,2731	347,18	cooling	4,264	36,6828	21,4543	212,87		4,264	45,0006	20,1918	346,79	cooling	4,264	34,6622	17,1338	245,02	heating
4,266	26,4851	2,5918	333,99	cooling	4,266	36,1767	20,5603	218,29		4,266	45,4261	20,8208	343,95	cooling	4,266	33,6805	13,2946	284,97	heating
4,268	27,3048	6,4668	291,28	cooling	4,268	36,7279	19,0686	246,85		4,268	45,3508	15,768	413,52	cooling	4,268	34,4776	15,3781	266,98	heating
4,27	27,3425	4,0219	325,99	cooling	4,27	36,7492	15,4729	297,41		4,27	49,3614	21,2134	393,47	cooling	4,27	33,4942	14,9152	259,71	heating
4,272	28,15	5,7819	312,67	cooling	4,272	36,8739	17,0733	276,78		4,272	47,1741	14,1772	461,25	cooling	4,272	34,7403	15,2333	272,68	heating
4,274	28,3092	3,897	341,25	cooling	4,274	36,5817	15,274	297,85		4,274	50,3242	22,0109	395,78	cooling	4,274	33,4962	11,0034	314,42	heating
4,276	29,6274	5,7836	333,3	cooling	4,276	37,7033	20,5199	240,2		4,276	50,4073	24,6029	360,71	cooling	4,276	34,513	15,1394	270,81	heating
4,278	29,1726	6,8205	312,45	cooling	4,278	37,0709	19,7854	241,63	heating	4,278	49,8006	17,3699	453,33	cooling	4,278	33,2412	12,5319	289,49	heating
4,28	29,3456	4,7995	343,12	cooling	4,28	37,2321	22,438	206,8	heating	4,28	49,7112	6,1637	608,73	cooling	4,28	34,0899	15,9722	253,26	heating
4,282	29,4889	2,9679	370,72	cooling	4,282	36,6816	20,1844	230,61	heating	4,282	51,2307	10,7945	565,24	cooling	4,282	32,9436	17,1704	220,49	heating
4,284	29,7427	0,9817	402,04	cooling	4,284	37,0641	18,902	253,88	heating	4,284	53,1317	14,8493	535,13	cooling	4,284	33,6205	15,317	255,86	heating
4,286	29,7325	5,29	341,67	cooling	4,286	36,6449	16,9847	274,82	heating	4,286	51,2605	25,185	364,5	cooling	4,286	33,366	17,6723	219,38	heating

4,288	30,086	8,028	308,34	cooling	4,288	36,9765	19,1264	249,52	heating	4,288	52,1071	19,7591	452,18	cooling	4,288	32,7404	13,6944	266,23	heating
4,29	30,3454	9,6088	289,87	cooling	4,29	36,9029	20,2998	232,09	heating	4,29	51,2305	10,4382	570,22	cooling	4,29	33,851	15,1445	261,49	heating
4,292	30,5404	3,8601	372,95	cooling	4,292	37,1191	20,2668	235,57	heating	4,292	52,7684	14,9146	529,14	cooling	4,292	34,1808	13,9866	282,28	heating
4,294	30,3851	2,2206	393,7	cooling	4,294	37,2147	19,9346	241,55	heating	4,294	49,7622	16,7317	461,72	cooling	4,294	34,0659	15,704	256,67	heating
4,296	30,6787	1,6171	406,24	cooling	4,296	37,9718	19,8916	252,73	heating	4,296	52,4497	22,1248	423,9	cooling	4,296	33,845	15,5023	256,4	heating
4,298	30,4257	7,6694	318,1	cooling	4,298	37,8012	21,7171	224,83	heating	4,298	50,2746	15,0427	492,49	cooling	4,298	34,9749	20,8518	197,42	heating
4,3	29,431	6,1173	325,89	cooling	4,3	37,4927	16,4	294,84	heating	4,3	50,8392	13,6287	520,15	cooling	4,3	34,2114	14,2634	278,84	heating
4,302	30,3432	8,8776	300,06	cooling	4,302	37,8339	15,967	305,67	heating	4,302	49,8485	17,6976	449,42	cooling	4,302	34,815	17,1511	246,91	heating
4,304	29,4948	6,6302	319,61	cooling	4,304	37,4858	13,9457	329,06	heating	4,304	50,2364	20,6875	413,05	cooling	4,304	33,7719	11,423	312,4	heating
4,306	29,1077	4,7799	340,07	cooling	4,306	37,28	18,8331	257,86	heating	4,306	49,5877	10,1893	550,73	cooling	4,306	35,1373	14,3427	290,68	heating
4,308	27,7156	3,8071	334,2	cooling	4,308	37,0421	20,5428	230,64	heating	4,308	50,3283	8,0513	590,97	cooling	4,308	33,7192	15,0947	260,34	heating
4,31	27,8862	4,399	328,32	cooling	4,31	37,1358	20,527	232,17	heating	4,31	51,9289	8,2808	610,14	cooling	4,31	34,1379	14,8719	269,31	heating
4,312	27,0133	1,4998	356,64	cooling	4,312	36,7112	15,8723	291,3	heating	4,312	51,7406	18,2631	467,97	cooling	4,312	34,149	15,4352	261,59	heating
4,314	28,2331	6,5619	302,93	cooling	4,314	37,148	19,1646	251,38	heating	4,314	52,3777	29,3998	321,2	cooling	4,314	34,0707	14,1851	277,97	heating
4,316	27,5019	4,2282	325,33	cooling	4,316	36,7351	17,7757	265,03	heating	4,316	51,8099	29,749	308,38	cooling	4,316	33,7144	11,493	310,62	heating
4,318	28,6117	2,3937	366,49	cooling	4,318	37,2979	17,8384	272,01	heating	4,318	52,7446	16,076	512,57	cooling	4,318	34,9299	13,4654	300,04	heating
4,32	27,9418	6,141	304,74	cooling	4,32	36,7601	16,9326	277,16	heating	4,32	50,8322	6,5187	619,44	cooling	4,32	34,1631	14,4414	275,68	heating
4,322	27,6879	4,1281	329,33	cooling	4,322	37,5227	15,5698	306,87	heating	4,322	51,1717	6,3566	626,45	cooling	4,322	34,4352	13,195	296,91	heating
4,324	28,2104	4,5515	330,72	cooling	4,324	37,2805	16,6025	289,05	heating	4,324	49,5324	15,5426	475,13	cooling	4,324	35,0157	19,1644	221,58	heating
4,326	28,2193	6,0299	310,18	cooling	4,326	37,3918	17,1419	283,06	heating	4,326	50,573	24,8434	359,66	cooling	4,326	34,7861	12,9297	305,52	heating
4,328	28,1741	5,3064	319,66	cooling	4,328	37,417	17,3713	280,21	heating	4,328	49,332	17,505	444,89	cooling	4,328	34,9003	16,0812	263,06	heating
4,33	28,4383	9,1169	270,09	cooling	4,33	38,0581	16,4138	302,56	heating	4,33	52,9097	15,9216	517,04	cooling	4,33	33,9851	13,7821	282,41	heating
4,332	28,1344	11,731	229,29	cooling	4,332	37,2309	15,5513	303,05	heating	4,332	53,3012	7,1646	644,92	cooling	4,332	35,283	15,4736	276,91	heating
4,334	27,4614	3,3506	337,03	cooling	4,334	37,1446	13,4109	331,76	heating	4,334	53,2496	15,1777	532,19	cooling	4,334	34,8378	14,186	288,68	heating
4,336	28,9888	5,875	323,1	cooling	4,336	37,5398	16,5347	293,62	heating	4,336	52,6995	19,9001	458,49	cooling	4,336	34,6092	16,367	255	heating
4,338	27,7414	5,1829	315,33	cooling	4,338	37,2241	14,9786	310,96	heating	4,338	54,0747	19,013	490,11	cooling	4,338	34,4769	13,6033	291,78	heating
4,34	28,1002	7,7597	284,33	cooling	4,34	37,4182	18,2434	268,03	heating	4,34	53,3725	6,9269	649,24	cooling	4,34	35,6073	18,6187	237,47	heating
4,342	27,2323	8,9069	256,16	cooling	4,342	37,0395	17,9656	266,63	heating	4,342	52,689	15,2941	522,72	cooling	4,342	34,1604	13,4043	290,14	heating
4,344	27,4658	4,9182	315,18	cooling	4,344	37,5559	16,9655	287,82	heating	4,344	53,5653	22,9186	428,4	cooling	4,344	35,2425	13,1786	308,42	heating
4,346	27,1937	2,354	347,22	cooling	4,346	36,884	14,6817	310,35	heating	4,346	51,7287	27,6106	337,13	cooling	4,346	34,5816	12,1802	313,14	heating
4,348	27,2176	6,8011	285,39	cooling	4,348	37,3281	18,1926	267,49	heating	4,348	51,6715	22,6181	406,12	cooling	4,348	34,8033	8,6772	365,21	heating
4,35	26,6861	6,021	288,87	cooling	4,35	36,8991	18,4687	257,63	heating	4,35	49,3204	7,979	577,89	cooling	4,35	34,5295	12,7517	304,42	heating
4,352	27,3961	8,7734	260,32	cooling	4,352	37,706	20,0635	246,62	heating	4,352	54,7489	10,7637	614,85	cooling	4,352	34,8286	13,1486	303,05	heating
4,354	26,4146	5,8788	287,06	cooling	4,354	36,8007	16,0175	290,52	heating	4,354	51,1413	23,1926	390,68	cooling	4,354	34,364	13,8452	286,82	heating
4,356	26,9697	0,6537	367,86	cooling	4,356	37,5961	12,7382	347,48	heating	4,356	51,5335	29,6676	305,65	cooling	4,356	34,2442	12,2891	306,9	heating
4,358	27,5706	5,5207	308,22	cooling	4,358	37,3971	13,1002	339,63	heating	4,358	50,1165	16,6298	468,09	cooling	4,358	34,6185	13,1921	299,51	heating
4,36	26,8309	5,9772	291,5	cooling	4,36	37,4006	13,3344	336,41	heating	4,36	53,9652	17,4921	509,84	cooling	4,36	35,3778	10,2684	350,99	heating
4,362	27,1532	9,1871	251,14	cooling	4,362	37,692	16,4308	297,2	heating	4,362	48,6324	7,3746	576,72	cooling	4,362	34,7277	15,6395	266,82	heating
4,364	27,1948	8,6351	259,44	cooling	4,364	37,856	15,7604	308,86	heating	4,364	50,4341	21,4169	405,62	cooling	4,364	34,2401	12,6124	302,32	heating
4,366	26,7105	3,7722	320,64	cooling	4,366	37,237	12,5889	344,54	heating	4,366	49,6078	23,673	362,53	cooling	4,366	35,9644	16,361	274,03	heating
4,368	26,6904	3,9428	317,98	cooling	4,368	37,2812	9,6331	386,48	heating	4,368	48,5817	18,982	413,76	cooling	4,368	35,1548	8,6615	370,34	heating
4,37	26,538	7,1479	271,04	cooling	4,37	37,8393	13,7067	337,34		4,37	47,2726	6,5471	569,28	cooling	4,37	35,4126	7,1039	395,71	heating
4,372	26,4581	3,8036	316,68	cooling	4,372	37,6435	13,2683	340,73		4,372	53,1338	19,3294	472,53	cooling	4,372	34,7433	7,7592	377,2	
4,374	26,9674	6,9585	279,69	cooling	4,374	38,1279	17,7855	284,36		4,374	56,0993	10,0532	643,66	cooling	4,374	35,8382	14,4865	298,46	
4,376	26,7193	4,1718	315,18		4,376	37,5985	16,4955	294,99		4,376	50,9635	21,9217	405,96	cooling	4,376	34,2638	15,8441	257,48	
4,378	28,6118	3,9553	344,66		4,378	37,9468	14,8287	323,16		4,378	51,6918	21,5415	421,46	cooling	4,378	35,1601	18,6493	230,8	
4,38	28,6868	7,306	298,87	cooling	4,38	37,4038	13,9715	327,55		4,38	49,8618	14,7347	491,03	cooling	4,38	34,4205	15,5633	263,6	
4,382	28,6262	6,972	302,69	cooling	4,382	37,4033	15,2756	309,31		4,382	48,7819	14,9981	472,25	cooling	4,382	35,1122	12,6402	314,13	
4,384	29,0184	9,151	277,72	cooling	4,384	37,0329	16,7919	282,94		4,384	47,1952	18,5106	400,97	cooling	4,384	34,1414	9,8164	340,03	
4,386	29,3357	11,3699	251,14		4,386	37,0542	19,3113	248,02		4,386	54,4514	14,6808	555,93	cooling	4,386	35,3306	8,5153	374,84	
4,388	28,19	5,5801	316,05		4,388	36,4413	16,9783	272,06		4,388	49,5395	21,5031	391,91	cooling	4,388	34,5728	11,533	322,06	
4,39	29,1158	6,3956	317,6		4,39	37,6644	15,2012	314		4,39	50,2245	21,6906	398,86	cooling	4,39	34,2117	9,2537	348,88	
4,392	28,4581	6,5096	306,81		4,392	37,438	16,6903	290,02		4,392	50,1544	10,7871	550,3	cooling	4,392	35,3327	14,3692	293,04	
4,394	28,5255	3,0358	356,31		4,394	37,855	15,9347	306,41		4,394	51,3364	16,863	481,89	cooling	4,394	35,1931	9,9747	352,52	
4,396	28,4598	6,1443	311,94		4,396	38,2209	18,9339	269,6		4,396	48,2475	20,0074	394,75	cooling	4,396	35,1268	11,6636	327,98	
4,398	28,413	6,2241	310,17		4,398	38,5392	17,8757	288,84		4,398	56,4921	17,6906	542,39	cooling	4,398	34,7363	10,5183	338,53	

4,4	28,8544	9,0146	277,33		4,4	38,0268	16,143	305,9		4,4	53,1712	19,2232	474,54	cooling	4,4	36,17	14,8524	297,99	
4,402	28,4751	12,2996	226,11		4,402	38,221	15,9162	311,79		4,402	52,7111	15,1177	525,5	cooling	4,402	34,8564	10,4282	341,47	
4,404	28,8184	9,6765	267,58		4,404	38,4074	18,3905	279,81		4,404	51,9592	6,3766	637,18	cooling	4,404	35,5614	14,2307	298,17	
4,406	29,0971	6,3786	317,57		4,406	37,6186	16,5719	294,2		4,406	54,3735	20,1093	478,96	cooling	4,406	34,3255	8,4051	362,33	
4,408	29,0573	6,4083	316,6		4,408	37,7632	17,6214	281,55		4,408	49,4418	16,1025	466,03	cooling	4,408	35,7305	10,312	355,31	
4,41	28,647	4,5429	336,94		4,41	37,1723	13,7447	327,48		4,41	52,0137	13,6159	536,75	cooling	4,41	33,8015	8,5779	352,59	cooling
4,412	29,9446	11,8811	252,5		4,412	38,3678	16,2231	309,55		4,412	52,0446	15,8198	506,37	cooling	4,412	34,8265	7,546	381,34	cooling
4,414	28,7748	9,8835	264,07		4,414	37,4713	17,5734	278,14		4,414	51,7205	16,7106	489,39	cooling	4,414	34,2721	9,4825	346,52	cooling
4,416	28,8074	5,9787	319,11		4,416	38,2963	17,4779	291,01		4,416	51,1786	13,2378	530,36	cooling	4,416	34,5279	12,5404	307,35	cooling
4,418	28,8435	7,6258	296,59		4,418	37,7266	15,9899	303,85		4,418	53,8346	25,0471	402,41	cooling	4,418	33,9263	14,5214	271,25	cooling
4,42	28,5596	8,1704	285,01		4,42	37,7411	15,1506	315,78		4,42	49,5197	16,5007	461,56	cooling	4,42	35,1928	16,0949	266,96	cooling
4,422	28,2011	7,9386	283,24		4,422	37,3278	12,9046	341,4		4,422	49,6218	9,1036	566,38	cooling	4,422	33,9836	16,2603	247,74	cooling
4,424	28,2252	10,226	251,6		4,424	37,9843	14,8443	323,46		4,424	48,1047	13,6972	480,97	cooling	4,424	33,9083	12,0275	305,86	cooling
4,426	27,9796	3,4246	343,24		4,426	37,3637	16,7422	288,26		4,426	46,6226	15,6215	433,35	cooling	4,426	34,8096	16,2098	260	
4,428	28,0352	1,6427	368,93		4,428	37,1146	14,461	316,66		4,428	44,7201	11,3283	466,77	cooling	4,428	34,5182	12,1077	313,27	
4,43	27,4618	4,5668	320,04		4,43	38,1505	16,6422	300,65		4,43	48,5152	24,3699	337,51	cooling	4,43	34,3166	14,8046	272,75	
4,432	27,3209	5,8599	299,99		4,432	37,2148	12,8365	340,77		4,432	45,8715	8,5918	521,12	cooling	4,432	33,3074	10,368	320,66	
4,434	27,9777	10,2328	248,05		4,434	37,5818	14,8562	317,67		4,434	46,4926	3,722	597,87	cooling	4,434	35,0616	12,9435	309,18	
4,436	27,1243	5,3773	303,99		4,436	37,0437	14,5455	314,49		4,436	45,9397	11,7291	478,21	cooling	4,436	34,1632	9,3439	346,94	
4,438	27,5605	-0,7896	396,29		4,438	37,8951	15,2303	316,82		4,438	45,5324	12,5408	461,17	cooling	4,438	34,8094	13,3127	300,49	
4,44	27,9504	0,8063	379,43		4,44	37,0672	11,7356	354,1		4,44	44,6959	10,3719	479,8	cooling	4,44	33,9826	10,9501	321,96	
4,442	27,2031	2,6666	342,98		4,442	37,4671	13,9717	328,43		4,442	49,6624	27,0108	316,64	cooling	4,442	35,2233	15,3824	277,35	
4,444	27,4048	3,6261	332,39	cooling	4,444	37,178	12,757	341,37		4,444	51,7401	9,0362	596,94	cooling	4,444	33,2732	11,7396	301,01	
4,446	27,7233	6,2613	300,01	cooling	4,446	37,5379	16,1126	299,49		4,446	49,4408	18,6537	430,36	cooling	4,446	34,4138	12,2882	309,28	
4,448	27,4752	2,8632	344,04	cooling	4,448	37,2382	16,6111	288,34		4,448	51,8017	30,6539	295,62	cooling	4,448	33,7103	10,5902	323,18	
4,45	27,0795	2,876	338,33	cooling	4,45	37,6576	13,6012	336,27		4,45	48,9329	18,0179	432,15	cooling	4,45	34,2334	10,8709	326,57	
4,452	26,7496	1,1587	357,72	cooling	4,452	38,0469	15,278	318,28		4,452	48,8015	16,2125	455,55	cooling	4,452	33,5143	11,7797	303,82	
4,454	26,7745	-2,58	410,33	cooling	4,454	37,2271	14,6055	316,22		4,454	48,1088	23,3682	345,84	cooling	4,454	34,3305	11,4067	320,44	
4,456	27,0952	0,2375	375,43	cooling	4,456	37,4204	14,9258	314,44		4,456	54,3424	9,4029	628,19	cooling	4,456	33,8296	12,575	297,11	
4,458	26,61	2,2767	340,14	cooling	4,458	37,4164	15,0162	313,12		4,458	50,0508	23,8041	366,89	cooling	4,458	33,4875	10,5471	320,67	
4,46	27,2664	6,2818	293,33	cooling	4,46	37,1154	16,3555	290,19		4,46	48,9544	24,2285	345,63	cooling	4,46	34,5155	15,045	272,17	
4,462	27,102	8,7571	256,43	cooling	4,462	36,8525	15,0287	305,06		4,462	48,4704	10,4125	531,99	cooling	4,462	34,5607	12,4902	308,51	
4,464	26,5356	4,0872	313,8	cooling	4,464	37,565	19,7926	248,43		4,464	51,3583	22,3128	406,01	cooling	4,464	33,9493	14,7862	267,87	
4,466	26,8935	-0,389	381,37	cooling	4,466	37,0088	15,6114	299,1		4,466	45,4978	16,773	401,53	cooling	4,466	33,2357	11,0091	310,7	
4,468	26,3756	-1,6659	391,98	cooling	4,468	37,1333	15,9431	296,21		4,468	51,7334	13,7752	530,6	cooling	4,468	34,8399	15,1517	275,21	
4,47	26,1542	-2,6853	403,13	cooling	4,47	37,1119	14,0713	322,07		4,47	49,7875	25,4777	339,81	cooling	4,47	33,764	8,5091	353,03	
4,472	25,9073	3,416	314,39	cooling	4,472	37,4587	12,9038	343,24		4,472	49,5617	19,8723	415,01	cooling	4,472	34,1892	11,3602	319,12	
4,474	25,8571	6,1649	275,27	cooling	4,474	37,3995	13,7368	330,77		4,474	49,0106	6,1184	599,57	cooling	4,474	33,2797	8,5633	345,5	
4,476	25,4301	3,6499	304,46	cooling	4,476	37,077	14,7474	312,13		4,476	53,3953	20,3031	462,58	cooling	4,476	34,5856	11,9363	316,6	
4,478	26,0896	2,3302	332,12	cooling	4,478	36,9368	14,4126	314,85		4,478	46,28	5,7227	566,93	cooling	4,478	33,0362	10,5033	314,98	cooling
4,48	26,0346	-0,2055	366,8	cooling	4,48	36,6527	14,9269	303,69		4,48	48,1561	10,3021	529,14	cooling	4,48	33,8657	10,1238	331,88	cooling
4,482	26,5645	1,983	343,61	cooling	4,482	36,2417	13,9097	312,17		4,482	45,9764	18,6238	382,35	cooling	4,482	33,5227	7,2973	366,59	cooling
4,484	26,0241	5,5167	286,66	cooling	4,484	36,7184	12,3048	341,27		4,484	45,4711	11,6074	473,36	cooling	4,484	33,9185	9,0835	347,16	cooling
4,486	25,9082	4,0004	306,24	cooling	4,486	36,5796	16,3892	282,23	cooling	4,486	44,5324	3,6651	571,26	cooling	4,486	32,9911	9,3801	330,05	cooling
4,488	26,4196	2,7555	330,79	cooling	4,488	36,7258	17,9029	263,12		4,488	50,1626	17,3728	458,35	cooling	4,488	34,0685	9,054	349,67	cooling
4,49	26,1381	2,4996	330,43	cooling	4,49	37,3744	21,9366	215,8		4,49	52,0761	-3,0122	770,05	cooling	4,49	33,5243	12,9389	287,75	cooling
4,492	26,3537	1,4918	347,53	cooling	4,492	37,4712	18,3935	266,68		4,492	49,4618	11,1375	535,72	cooling	4,492	32,8275	9,3489	328,2	cooling
4,494	26,5452	3,6986	319,36	cooling	4,494	37,2129	15,6958	300,78		4,494	48,476	16,7545	443,42	cooling	4,494	34,055	15,5123	259,2	cooling
4,496	26,665	5,6799	293,34	cooling	4,496	37,2746	14,057	324,55		4,496	47,9345	6,5221	578,88	cooling	4,496	33,7267	13,3508	284,82	cooling
4,498	25,8848	5,9111	279,2	cooling	4,498	37,3595	17,7551	274,04		4,498	48,8509	9,7746	546,23	cooling	4,498	33,4585	14,8996	259,43	cooling
4,5	26,3341	8,472	249,69	cooling	4,5	36,4006	17,7153	261,19		4,5	46,5327	12,8588	470,71	cooling	4,5	32,8308	13,0902	275,94	cooling
4,502	25,9747	8,2548	247,7	cooling	4,502	36,2028	19,2881	236,44		4,502	53,1357	6,0343	658,41	cooling	4,502	34,2037	17,2966	236,34	
4,504	26,2622	6,0306	282,81	cooling	4,504	35,8895	15,8306	280,39		4,504	48,992	22,1929	374,61	cooling	4,504	33,1805	11,1872	307,43	
4,506	26,2671	3,9096	312,53	cooling	4,506	36,5931	15,0434	301,23		4,506	48,391	25,2566	323,38	cooling	4,506	33,7577	13,5299	282,75	
4,508	26,66	2,5711	336,73	cooling	4,508	36,2014	15,4942	289,45		4,508	47,4629	15,126	452,02	cooling	4,508	32,5962	6,9536	358,45	
4,51	26,7804	3,3124	328,05	cooling	4,51	36,8123	16,7394	280,59		4,51	50,1769	24,8921	353,44	cooling	4,51	33,8482	9,8533	335,41	

4,512	26,4893	3,7835	317,39	cooling	4,512	36,6634	17,1181	273,21		4,512	45,292	18,8137	370,13	cooling	4,512	32,2449	11,1996	294,18	
4,514	26,9269	3,4786	327,77	cooling	4,514	36,6654	15,9821	289,12		4,514	51,321	14,7427	511,31	cooling	4,514	32,8737	10,5248	312,4	
4,516	26,7427	1,8939	347,35	cooling	4,516	36,6326	15,3676	297,25		4,516	48,851	24,029	346,98	cooling	4,516	32,5961	10,0791	314,75	
4,518	26,7062	2,6758	335,91	cooling	4,518	37,2579	15,2806	307,21		4,518	48,8686	20,0355	403,04	cooling	4,518	32,7469	10,6047	309,51	
4,52	25,9364	1,1831	346,02	cooling	4,52	36,738	16,3888	284,45		4,52	48,1981	9,3274	543,35	cooling	4,52	32,3584	10,6331	303,69	
4,522	26,9074	3,49	327,34	cooling	4,522	36,7087	15,3512	298,55		4,522	52,9124	25,6953	380,45	cooling	4,522	33,3841	10,0406	326,31	
4,524	26,2172	2,6114	329,97	cooling	4,524	37,2826	16,6921	287,82		4,524	48,6453	11,5134	519,05	cooling	4,524	32,705	9,9462	318,13	
4,526	26,7446	2,2324	342,64	cooling	4,526	37,5782	13,6741	334,14		4,526	49,0823	19,173	418,09	cooling	4,526	32,5639	4,8176	387,85	
4,528	26,5197	3,4664	322,25	cooling	4,528	37,7958	15,8684	306,51		4,528	47,8315	23,5516	339,4	cooling	4,528	33,7744	12,011	304,22	cooling
4,53	26,9155	5,2427	302,95	cooling	4,53	37,5818	17,6826	278,16		4,53	46,8815	10,4293	509,55	cooling	4,53	33,6328	9,5028	337,3	cooling
4,532	26,3513	3,595	318,1	cooling	4,532	37,69	19,2031	258,42		4,532	47,5582	5,8539	582,96	cooling	4,532	33,2534	12,5163	289,87	cooling
4,534	27,265	4,3062	320,93	cooling	4,534	36,8022	16,6443	281,78		4,534	48,7732	13,2725	496,25	cooling	4,534	32,725	10,8316	306,04	cooling
4,536	27,7357	2,7512	349,25	cooling	4,536	36,1165	16,2158	278,18		4,536	52,1923	4,5177	666,42	cooling	4,536	33,8195	14,9676	263,52	cooling
4,538	28,4838	3,8011	345,03	cooling	4,538	35,7746	12,9977	318,39		4,538	49,1511	17,7887	438,4	cooling	4,538	32,8633	12,0376	291,11	cooling
4,54	28,5846	8,1049	286,28	cooling	4,54	36,1987	17,2725	264,56		4,54	48,2905	16,3021	447,15	cooling	4,54	32,9436	15,706	240,96	
4,542	28,705	8,86	277,4	cooling	4,542	35,1266	18,975	225,78		4,542	47,3637	4,2927	602,07	cooling	4,542	32,1449	9,2368	320,22	
4,544	28,895	8,707	282,2	cooling	4,544	35,453	16,6324	263,08	heating	4,544	51,3084	21,4631	417,19	cooling	4,544	33,4232	11,3432	308,65	
4,546	28,356	5,1675	324,14	cooling	4,546	36,1463	17,0836	266,47	heating	4,546	45,3929	13,9558	439,44	cooling	4,546	31,8808	11,6625	282,62	
4,548	28,6703	1,7503	376,3	cooling	4,548	35,6987	12,8328	319,63	heating	4,548	48,6614	17,6093	434,06	cooling	4,548	32,728	10,6245	308,97	
4,55	28,803	0,7835	391,67	cooling	4,55	36,6818	14,5724	309,06	heating	4,55	46,881	25,4712	299,28	cooling	4,55	32,1096	8,075	335,97	
4,552	28,6272	3,1392	356,28	cooling	4,552	37,1889	17,5233	274,9	heating	4,552	46,0605	15,5508	426,48	cooling	4,552	32,5329	6,4858	364,1	
4,554	28,3368	3,8214	342,69	cooling	4,554	36,6335	15,3675	297,27	heating	4,554	45,6027	5,8315	555,94	cooling	4,554	31,8001	6,8818	348,32	cooling
4,556	29,4702	7,7648	303,41	cooling	4,556	36,5535	12,0967	341,87	heating	4,556	50,1471	23,3833	374,12	cooling	4,556	33,1972	7,7423	355,82	cooling
4,558	29,1524	4,9602	338,17	cooling	4,558	37,515	17,7952	275,65		4,558	47,6672	5,6171	587,8	cooling	4,558	32,031	8,5453	328,3	cooling
4,56	29,6428	4,4331	352,39	cooling	4,56	37,3815	12,7264	344,64		4,56	46,8279	14,6702	449,52	cooling	4,56	32,1335	6,6399	356,36	cooling
4,562	29,6881	3,8639	360,98	cooling	4,562	37,536	17,4666	280,54		4,562	45,7881	18,8223	376,94	cooling	4,562	32,5682	12,2588	283,9	cooling
4,564	29,8249	1,5765	394,87	cooling	4,564	36,9037	17,1217	276,52		4,564	45,0771	6,4331	540,19	cooling	4,564	32,7842	9,0273	332,09	cooling
4,566	29,6382	3,1161	370,74	cooling	4,566	37,2458	13,4326	332,87		4,566	46,0152	8,5955	523,07	cooling	4,566	32,2068	8,3152	333,97	cooling
4,568	29,3399	5,2902	336,18	cooling	4,568	37,5997	15,1326	314,06		4,568	46,5902	16,5758	419,56	cooling	4,568	31,9951	4,505	384,27	cooling
4,57	29,734	6,9325	318,73	cooling	4,57	36,306	14,5433	304,21		4,57	52,5792	4,6329	670,22	cooling	4,57	33,2732	9,2316	336,07	cooling
4,572	29,4807	7,0515	313,53	cooling	4,572	36,8478	13,8019	322,15		4,572	49,4923	17,3136	449,81	cooling	4,572	32,137	9,0304	323	cooling
4,574	28,9185	4,2597	344,69	cooling	4,574	36,7388	20,0199	233,7		4,574	48,9298	15,7819	463,36	cooling	4,574	32,5305	15,0425	244,46	cooling
4,576	29,0609	3,3562	359,31	cooling	4,576	35,3819	17,4249	251,01		4,576	48,5129	3,7911	625,14	cooling	4,576	31,645	10,9811	288,85	cooling
4,578	29,1733	5,3722	332,7	cooling	4,578	35,9671	13,5841	312,88		4,578	51,7069	17,6398	476,21	cooling	4,578	33,2554	12,554	289,37	cooling
4,58	28,6847	2,3052	368,75	cooling	4,58	36,816	18,0145	262,82		4,58	45,8628	8,0294	528,85	cooling	4,58	31,6217	13,2269	257,13	cooling
4,582	29,6624	4,1456	356,69	cooling	4,582	36,7388	11,6886	350,16		4,582	50,0646	3,4993	650,92	cooling	4,582	32,5378	13,6315	264,28	cooling
4,584	29,6729	3,528	365,47	cooling	4,584	37,6834	17,3643	284,03		4,584	46,7093	13,1583	468,99	cooling	4,584	31,6727	10,9338	289,9	cooling
4,586	30,0803	4,5114	357,42	cooling	4,586	37,6688	19,171	258,57		4,586	44,7722	8,0003	514,02	cooling	4,586	32,1677	10,88	297,57	cooling
4,588	30,3882	10,1609	282,75	cooling	4,588	36,7971	13,3056	328,38		4,588	43,6096	1,4898	588,77	cooling	4,588	31,3637	12,0033	270,63	
4,59	30,3412	12,1303	254,56	cooling	4,59	37,1462	11,6097	356,96		4,59	46,4256	19,0506	382,66	cooling	4,59	32,7442	13,4938	269,09	
4,592	29,6168	7,2387	312,81	cooling	4,592	36,4536	13,4407	321,69		4,592	42,5192	8,7408	472,17	cooling	4,592	31,9151	14,2773	246,55	
4,594	29,2216	7,3217	306,13	cooling	4,594	36,4794	8,7656	387,4		4,594	44,9552	13,6286	437,9	cooling	4,594	31,7298	10,8258	292,21	
4,596	28,4969	3,2161	353,39	cooling	4,596	36,2227	16,5557	274,92		4,596	43,9675	18,573	354,98	cooling	4,596	32,8653	16,6699	226,39	
4,598	28,5924	3,3551	352,78	cooling	4,598	35,2865	14,5577	289,76		4,598	44,7855	9,0025	500,19	cooling	4,598	32,4872	14,3327	253,77	
4,6	28,1474	5,6975	313,82	cooling	4,6	36,0671	11,8019	339,19		4,6	46,4965	7,4642	545,61	cooling	4,6	32,3479	16,605	220,06	
4,602	27,7733	1,8661	362,14	cooling	4,602	37,2151	17,7531	272,05		4,602	50,0268	21,0546	404,99	cooling	4,602	31,4697	11,2364	282,83	
4,604	28,8543	2,3934	369,88	cooling	4,604	36,9818	13,736	324,94		4,604	50,2014	9,2195	572,87	cooling	4,604	33,0741	13,2287	277,41	
4,606	28,1632	3,5664	343,83	cooling	4,606	37,3174	15,2525	308,43		4,606	49,1297	23,197	362,5	cooling	4,606	31,6655	10,8082	291,55	
4,608	28,3248	4,1269	338,25	cooling	4,608	36,6916	17,7561	264,69		4,608	48,0125	27,5163	286,51	cooling	4,608	31,9981	15,4539	231,26	
4,61	28,5191	7,9665	287,3	cooling	4,61	35,2171	11,9822	324,79		4,61	47,4007	18,1455	408,94	cooling	4,61	31,0873	11,6244	272,06	
4,612	28,0802	6,6542	299,5	cooling	4,612	35,8727	11,6173	339,05		4,612	48,1912	18,6861	412,44	cooling	4,612	32,5189	13,4821	266,11	heating
4,614	27,7441	0,92	374,96	cooling	4,614	34,4979	14,464	280,04		4,614	47,795	16,8246	432,92	cooling	4,614	30,8381	12,5856	255,14	heating
4,616	28,6394	5,1011	329,03	cooling	4,616	34,808	11,5656	324,89		4,616	50,4963	3,6596	654,71	cooling	4,616	32,0656	15,6898	228,91	heating
4,618	27,8332	3,2411	343,76	cooling	4,618	35,1869	18,7492	229,78		4,618	47,4526	13,2748	477,75	cooling	4,618	30,9751	13,7892	240,23	heating
4,62	28,0006	3,5834	341,32	cooling	4,62	35,0556	14,6517	285,22		4,62	46,4941	12,4869	475,37	cooling	4,62	31,3684	10,9656	285,2	heating
4,622	27,6198	5,3425	311,4	cooling	4,622	35,1967	8,9736	366,56		4,622	45,8614	5,6571	562	cooling	4,622	31,3284	13,9627	242,75	heating

4,624	27,2891	1,5116	360,33	cooling	4,624	36,6211	11,7287	347,96		4,624	47,4612	13,96	468,3	cooling	4,624	32,253	12,5391	275,57	heating
4,626	27,9414	2,4232	356,71	cooling	4,626	37,0035	12,2488	346,03		4,626	44,5206	6,385	533,08	cooling	4,626	31,9562	11,8014	281,73	heating
4,628	27,5116	6,8555	288,74	cooling	4,628	37,3536	14,1177	324,8		4,628	48,726	3,8953	626,67	cooling	4,628	31,8609	9,9411	306,41	heating
4,63	27,9087	4,3598	329,18	cooling	4,63	37,1032	18,3571	262,04		4,63	46,2347	13,8446	452,77	cooling	4,63	32,8405	14,878	251,09	heating
4,632	28,2045	8,5055	275,36	cooling	4,632	36,2997	10,3002	363,43		4,632	45,8959	11,7945	476,69	cooling	4,632	32,9796	13,9212	266,41	heating
4,634	27,5391	9,9637	245,68	cooling	4,634	37,522	10,8932	372,23		4,634	46,5119	7,9786	538,64	cooling	4,634	32,8466	19,0849	192,37	heating
4,636	27,1627	5,9603	296,38	cooling	4,636	35,8048	10,5403	353,16	cooling	4,636	48,9429	15,5127	467,3	cooling	4,636	31,9702	12,5041	272,11	heating
4,638	28,1803	9,71	258,19	cooling	4,638	36,5544	8,6214	390,46	cooling	4,638	47,1973	5,5419	582,28	cooling	4,638	33,6208	15,3242	255,76	heating
4,64	26,632	5,4944	295,47	cooling	4,64	36,4877	14,1231	312,62	cooling	4,64	50,4936	7,6864	598,38	cooling	4,64	32,0495	12,9455	267,05	heating
4,642	26,8414	3,0265	332,9	cooling	4,642	36,2121	14,5431	302,9	cooling	4,642	49,9786	13,8461	505,08	cooling	4,642	32,7032	15,4795	240,76	heating
4,644	26,4051	6,8812	272,91	cooling	4,644	35,812	10,7826	349,87	cooling	4,644	50,289	11,4042	543,55	cooling	4,644	31,9422	11,9165	279,93	heating
4,646	25,6976	5,366	284,21	cooling	4,646	37,2017	13,739	327,97	cooling	4,646	51,3705	11,0401	563,76	cooling	4,646	32,5943	8,2771	339,92	heating
4,648	25,6135	4,1357	300,23	cooling	4,648	36,1766	9,3441	375,08	cooling	4,648	53,597	16,409	519,83	cooling	4,648	31,5548	5,2971	367,04	heating
4,65	25,8645	7,8828	251,36	cooling	4,65	36,1796	9,7678	369,2	cooling	4,65	51,6784	6,0269	638,14	cooling	4,65	32,3619	10,5522	304,87	heating
4,652	25,5837	0,8127	346,26	cooling	4,652	36,0804	18,5043	245,69	cooling	4,652	51,6357	14,7251	515,95	cooling	4,652	31,4926	11,3351	281,77	heating
4,654	26,0572	3,1473	320,25	cooling	4,654	35,2937	15,1856	281,08	cooling	4,654	50,7371	18,5903	449,36	cooling	4,654	31,8793	13,1299	262,09	heating
4,656	25,663	6,1041	273,4	cooling	4,656	35,71	16,2732	271,7	cooling	4,656	50,0181	13,4656	510,95	cooling	4,656	31,4397	16,8999	203,25	heating
4,658	25,6083	2,7038	320,17	cooling	4,658	34,7354	15,224	272,74	cooling	4,658	50,6696	16,1541	482,48	cooling	4,658	32,4373	13,1522	269,58	heating
4,66	26,6254	4,8428	304,49	cooling	4,66	36,334	15,9515	284,92	cooling	4,66	51,8318	18,0295	472,51	cooling	4,66	31,9874	11,5755	285,33	heating
4,662	25,9437	6,4467	272,54	cooling	4,662	35,9001	19,3538	231,29	cooling	4,662	54,4341	16,7311	527,03	cooling	4,662	31,7909	5,7966	363,36	heating
4,664	26,8704	4,4454	313,47	cooling	4,664	35,8407	20,5971	213,08		4,664	52,5028	23,543	404,81	cooling	4,664	32,7814	9,1869	329,82	heating
4,666	26,9966	7,8792	267,23	cooling	4,666	35,4125	14,4994	292,33		4,666	51,8984	20,482	439,15	cooling	4,666	32,7384	8,5791	337,71	heating
4,668	26,6441	6,2457	285,14	cooling	4,668	37,0253	18,8015	254,74		4,668	52,4909	15,4412	517,9	cooling	4,668	32,5061	13,4746	266,03	heating
4,67	26,3064	1,7732	342,94	cooling	4,67	35,2732	15,3657	278,28		4,67	51,0774	11,5863	552,03	cooling	4,67	32,2159	10,9139	297,77	heating
4,672	27,1966	6,0992	294,91	cooling	4,672	36,3693	16,8937	272,24		4,672	51,72	17,0721	484,33	cooling	4,672	33,5733	14,0749	272,56	heating
4,674	26,215	4,9012	297,94	cooling	4,674	35,9884	20,3395	218,75		4,674	53,9519	17,5965	508,19	cooling	4,674	32,7971	12,2782	286,82	heating
4,676	26,5185	6,054	286,06	cooling	4,676	35,8508	15,7271	281,3		4,676	51,656	25,3041	368,36	cooling	4,676	33,064	17,3735	219,33	heating
4,678	26,266	7,9527	255,99	cooling	4,678	36,4554	16,7215	275,85		4,678	50,6404	26,2542	340,88	cooling	4,678	32,4826	15,2888	240,34	heating
4,68	25,9275	4,4436	300,31		4,68	36,8486	19,5827	241,35		4,68	51,2106	22,2408	404,96	cooling	4,68	33,4155	15,9642	243,94	heating
4,682	26,1594	3,3415	318,96		4,682	36,5334	14,3917	309,51		4,682	51,5282	16,6363	487,74	cooling	4,682	31,8989	12,2644	274,46	heating
4,684	25,9845	7,9071	252,69		4,684	36,1654	13,5117	316,66		4,684	49,2352	15,8694	466,41	cooling	4,684	33,1119	12,5653	287,21	heating
4,686	26,0169	6,2845	275,83		4,686	35,9259	15,4136	286,73		4,686	51,1374	13,446	526,87	cooling	4,686	32,1786	13,2839	264,12	heating
4,688	26,0319	7,133	264,18		4,688	35,2139	10,22	349,38		4,688	49,322	16,8079	454,5	cooling	4,688	32,0763	10,952	295,29	heating
4,69	25,6879	5,4557	282,82		4,69	35,9489	17,2334	261,61		4,69	48,783	21,2621	384,7	cooling	4,69	32,0486	14,7173	242,27	heating
4,692	25,6809	2,2834	327,06		4,692	34,3784	14,9246	271,94		4,692	47,3178	19,2831	391,88	cooling	4,692	33,1245	16,2362	236,07	heating
4,694	26,3959	4,1971	310,31		4,694	36,107	15,5187	287,79		4,694	51,1184	25,1052	363,63	cooling	4,694	32,1909	14,7235	244,17	heating
4,696	25,6467	3,1495	314,48		4,696	35,4021	18,6268	234,49		4,696	47,0312	21,6318	355,05	cooling	4,696	32,5199	12,8047	275,59	heating
4,698	26,2253	2,3682	333,49		4,698	35,4289	16,2345	268,31		4,698	48,225	13,8767	480,14	cooling	4,698	33,1524	15,5492	246,07	heating
4,7	26,3785	5,2521	295,32		4,7	35,6077	11,6654	334,68	heating	4,7	47,2754	11,154	504,92	cooling	4,7	32,9834	10,3834	315,91	heating
4,702	26,111	6,1849	278,54		4,702	36,986	18,7996	254,22		4,702	48,5609	13,7252	486,95	cooling	4,702	32,956	15,036	250,49	heating
4,704	26,142	5,7753	284,7		4,704	35,5854	16,9653	260,28	heating	4,704	46,6375	8,1155	538,48	cooling	4,704	31,6285	10,1796	299,83	heating
4,706	26,2425	5,3905	291,48		4,706	36,5872	18,5031	252,79	heating	4,706	52,1012	26,0369	364,34	cooling	4,706	33,1283	11,8567	297,35	heating
4,708	26,5612	2,0557	342,55		4,708	35,5022	18,7768	233,8	heating	4,708	50,3356	20,0211	423,75	cooling	4,708	31,5935	13,2031	257,07	heating
4,71	26,1161	2,0081	336,99		4,71	34,9	12,8072	308,83	heating	4,71	50,1057	19,6998	425,03	cooling	4,71	31,9099	15,6703	227,01	heating
4,712	26,1077	3,9696	309,46		4,712	35,1899	16,2214	265,15	heating	4,712	49,771	18,6464	435,08	cooling	4,712	31,5445	14,8126	233,89	heating
4,714	25,7442	2,5671	323,98		4,714	35,2402	17,8883	242,55	heating	4,714	51,3602	16,2406	490,92	cooling	4,714	32,73	16,8133	222,49	heating
4,716	26,0667	2,1091	334,89		4,716	35,4684	15,3229	281,6	heating	4,716	51,6839	6,9655	625,1	cooling	4,716	31,5474	11,3035	282,98	heating
4,718	25,5157	3,8621	302,68		4,718	35,6113	17,0735	259,13	heating	4,718	53,6145	19,0999	482,46	cooling	4,718	33,4352	14,1044	270,22	heating
4,72	25,8621	4,862	293,55		4,72	35,8655	17,2643	260,02	heating	4,72	55,5494	11,9225	609,84	cooling	4,72	32,4304	13,25	268,11	heating
4,722	25,8658	7,3628	258,64		4,722	36,3068	14,0407	311,25	heating	4,722	54,7384	16,9952	527,6	cooling	4,722	33,0865	10,6938	313,02	heating
4,724	25,4528	7,3352	253,26		4,724	36,2487	18,5264	247,73	heating	4,724	54,72	19,2744	495,48	cooling	4,724	33,3354	15,2764	252,44	heating
4,726	25,458	6,3771	266,72		4,726	35,254	14,1003	295,7	heating	4,726	54,1668	12,8289	577,84	cooling	4,726	33,8226	13,8539	279,13	heating
4,728	25,73	7,7442	251,42		4,728	36,15	13,8415	311,84	heating	4,728	57,4417	14,6563	598,08	cooling	4,728	33,7063	14,1925	272,77	heating
4,73	25,5519	8,906	232,68		4,73	35,3198	15,8965	271,51	heating	4,73	53,3931	17,9197	495,87	cooling	4,73	33,1247	11,9699	295,71	heating
4,732	25,0968	6,5813	258,82		4,732	34,5797	15,9104	260,97	heating	4,732	56,4098	15,6936	569,15	cooling	4,732	33,7529	12,0003	304,07	heating
4,734	25,6191	5,286	284,23		4,734	34,6671	15,3143	270,52	heating	4,734	54,5876	18,4513	505,13	cooling	4,734	33,4046	9,703	331,31	heating

4,736	25,2221	1,0456	337,95		4,736	35,5208	17,8482	247,04	heating	4,736	54,8097	17,39	523,07	cooling	4,736	33,1732	12,6966	286,23	heating
4,738	25,2954	-0,8844	365,95		4,738	34,5664	14,5407	279,93	heating	4,738	53,2656	11,7668	580,09	cooling	4,738	32,5513	8,0699	342,21	heating
4,74	25,1666	1,5338	330,35		4,74	35,8008	16,4017	271,17	heating	4,74	56,7277	22,1602	483,2	cooling	4,74	33,957	13,2385	289,61	heating
4,742	25,6947	6,7164	265,29		4,742	35,2438	16,9402	255,86	heating	4,742	57,3345	12,3019	629,49	cooling	4,742	32,4803	9,91	315,5	heating
4,744	25,3094	7,3913	250,47		4,744	36,0659	16,1787	277,99	heating	4,744	56,1066	18,912	519,93	cooling	4,744	33,4408	12,7542	289,17	heating
4,746	25,5287	7,7977	247,85		4,746	35,1712	15,7022	272,15	heating	4,746	55,6695	22,7313	460,43	cooling	4,746	33,0651	12,629	285,67	heating
4,748	25,819	6,0487	276,36		4,748	36,3085	18,779	245,04	heating	4,748	55,7315	21,3979	479,93	cooling	4,748	33,9721	12,7859	296,15	heating
4,75	26,0806	3,7582	312,03		4,75	35,0724	16,4865	259,8	heating	4,75	55,689	20,3184	494,43	cooling	4,75	33,6694	13,7157	278,92	heating
4,752	26,2204	3,9188	311,74		4,752	35,3548	16,4606	264,11	heating	4,752	53,2397	21,57	442,7	cooling	4,752	34,2802	13,6836	287,91	heating
4,754	26,054	3,7162	312,25		4,754	34,8752	15,3441	273,02	heating	4,754	53,6295	12,703	572,09	cooling	4,754	33,7302	11,2946	313,62	heating
4,756	26,2947	2,5006	332,61		4,756	36,0841	14,0671	307,77	heating	4,756	49,8391	15,7442	476,6	cooling	4,756	33,7473	11,0366	317,46	heating
4,758	26,0164	3,8116	310,39		4,758	36,2954	15,3064	293,4	heating	4,758	48,5342	14,751	472,24	cooling	4,758	33,2527	11,9657	297,56	heating
4,76	26,0621	5,126	292,65		4,76	36,7505	16,4062	284,38	heating	4,76	47,2982	7,7521	552,8	cooling	4,76	34,0235	13,1376	291,95	heating
4,762	26,1661	4,5022	302,83		4,762	37,8354	18,8493	265,4	heating	4,762	49,7235	14,0423	498,77	cooling	4,762	33,2004	16,9108	227,7	heating
4,764	26,5452	5,7721	290,38		4,764	36,9661	18,0551	264,35	heating	4,764	47,1031	15,3722	443,55	cooling	4,764	32,8165	11,8688	292,82	heating
4,766	26,3081	3,0709	324,82		4,766	36,8774	17,4753	271,21	heating	4,766	50,6726	13,1573	524,41	cooling	4,766	33,7194	17,24	230,36	heating
4,768	26,9902	3,0996	333,96		4,768	35,824	15,6533	281,96	heating	4,768	50,7566	13,8662	515,67	cooling	4,768	32,8832	13,3498	273,05	heating
4,77	27,2663	5,0414	310,67		4,77	36,587	18,4786	253,13	heating	4,77	53,4594	12,4403	573,39	cooling	4,77	33,0639	11,5666	300,5	heating
4,772	26,9016	5,845	294,34		4,772	34,9282	17,7578	240,02	heating	4,772	54,2393	8,1664	644,03	cooling	4,772	33,3452	10,9322	313,3	heating
4,774	27,5044	7,2284	283,43		4,774	35,8693	20,3758	216,58	heating	4,774	57,5309	23,6892	473,06	cooling	4,774	34,0188	10,7993	324,57	heating
4,776	27,2993	5,8259	300,17		4,776	34,8838	17,7632	239,32	heating	4,776	54,2896	27,4129	375,7	cooling	4,776	33,727	9,7298	335,45	heating
4,778	27,5902	0,3491	380,79		4,778	36,068	16,6552	271,36	heating	4,778	56,0139	24,4176	441,67	cooling	4,778	34,6269	15,8836	262	heating
4,78	27,6277	2,7183	348,2		4,78	35,7778	17,4479	256,22	heating	4,78	55,2553	24,0855	435,71	cooling	4,78	33,7574	12,5442	296,53	heating
4,782	27,662	3,5195	337,48		4,782	36,7282	18,3646	256,7	heating	4,782	54,9424	22,013	460,3	cooling	4,782	35,0222	14,5502	286,17	heating
4,784	27,4706	2,1421	354,05		4,784	36,9544	17,6491	269,86	heating	4,784	53,4331	10,2021	604,31	cooling	4,784	33,8143	14,6094	268,46	heating
4,786	27,9835	6,5612	299,45		4,786	37,0038	16,5542	285,85	heating	4,786	55,2117	19,2217	503,09	cooling	4,786	34,5257	11,6046	320,4	heating
4,788	27,0978	6,441	288,75		4,788	37,3731	18,096	269,46	heating	4,788	51,5466	13,8642	526,74	cooling	4,788	33,887	13,3109	287,62	heating
4,79	27,9056	4,9981	320,21		4,79	37,2786	21,3049	223,29	heating	4,79	51,9744	16,8274	491,3	cooling	4,79	33,3189	10,5818	317,83	heating
4,792	27,5709	6,6286	292,74		4,792	37,3106	25,1801	169,57	heating	4,792	52,1231	25,218	376,09	cooling	4,792	33,7032	11,1012	315,94	heating
4,794	27,1944	5,7665	299,53		4,794	36,2125	19,4366	234,5	heating	4,794	52,5356	24,041	398,31	cooling	4,794	34,6165	14,6792	278,69	heating
4,796	27,4008	7,4524	278,85		4,796	37,4655	19,2609	254,47	heating	4,796	53,906	16,1091	528,34	cooling	4,796	33,7189	14,8913	263,18	heating
4,798	27,1324	10,958	226,09		4,798	36,1503	15,1124	294,08	heating	4,798	56,8509	25,6703	435,86	cooling	4,798	34,1647	12,6312	301,01	heating
4,8	27,1191	5,332	304,55		4,8	36,5602	18,1031	258	heating	4,8	56,862	17,5088	550,1	cooling	4,8	34,9434	17,4984	243,86	heating
4,802	27,4256	4,6967	317,72		4,802	35,4124	17,723	247,27	heating	4,802	56,4332	19,6701	513,89	cooling	4,802	34,4933	13,7364	290,15	heating
4,804	27,4758	4,3459	323,32		4,804	35,5463	17,8665	247,14	heating	4,804	56,9461	25,6962	436,83	cooling	4,804	35,0914	16,9933	252,98	heating
4,806	27,0167	0,9871	363,86		4,806	34,6606	15,4618	268,37	heating	4,806	55,7708	17,4873	535,15	cooling	4,806	34,31	14,0923	282,61	heating
4,808	27,6858	0,9539	373,67		4,808	34,6836	16,364	256,08	heating	4,808	56,9646	15,8938	574,11	cooling	4,808	35,2313	13,9015	298,16	heating
4,81	27,2562	1,5749	358,99		4,81	34,5827	13,8623	289,64	heating	4,81	55,7973	19,3387	509,64	cooling	4,81	34,1543	13,6967	285,97	heating
4,812	27,8558	1,9084	362,71	cooling	4,812	34,8551	14,8116	280,18	heating	4,812	57,7098	13,7964	613,84	cooling	4,812	34,2932	16,3785	250,42	heating
4,814	27,5262	5,991	301,03	cooling	4,814	34,9402	14,869	280,57	heating	4,814	57,1154	19,5454	525,17	cooling	4,814	33,633	12,2939	298,29	heating
4,816	27,6079	7,1017	286,65	cooling	4,816	35,5242	14,6395	291,94	heating	4,816	56,7262	19,6275	518,59	cooling	4,816	34,5943	13,591	293,6	heating
4,818	27,4041	7,6024	276,8	cooling	4,818	36,2935	16,0567	282,88	heating	4,818	56,3469	9,9118	649,09	cooling	4,818	33,6469	11,7676	305,84	heating
4,82	27,6933	11,3644	228,25	cooling	4,82	36,0538	12,5367	328,73	heating	4,82	58,9622	17,4283	580,58	cooling	4,82	34,8719	13,2834	301,78	heating
4,822	26,8105	6,7033	281,07		4,822	36,8036	14,2713	314,97	heating	4,822	57,8084	9,4081	676,56	cooling	4,822	34,6222	14,9108	275,54	heating
4,824	26,8121	4,8243	307,36		4,824	36,7391	14,4582	311,45	heating	4,824	57,1561	17,1368	559,41	cooling	4,824	34,977	14,0489	292,54	heating
4,826	26,4081	5,6928	289,57		4,826	36,2532	14,5482	303,4	heating	4,826	57,6606	25,6972	446,8	cooling	4,826	35,3115	16,0974	268,58	heating
4,828	25,7223	4,003	303,6		4,828	35,82	11,1836	344,38	heating	4,828	57,5426	16,0401	580,14	cooling	4,828	35,7608	16,3669	271,1	heating
4,83	26,0292	3,3014	317,7		4,83	36,2823	14,8	300,29	heating	4,83	59,6837	8,6381	713,54	cooling	4,83	35,3121	16,7967	258,82	heating
4,832	25,433	2,0201	327,28		4,832	35,9784	15,8305	281,64	heating	4,832	61,7944	16,8744	627,91	cooling	4,832	35,12	12,0888	321,94	heating
4,834	25,829	-0,7993	372,22		4,834	36,1404	17,8704	255,39	heating	4,834	64,852	10,4619	760,29	cooling	4,834	35,449	13,1825	311,25	heating
4,836	25,524	2,5179	321,59	cooling	4,836	36,2332	13,5658	316,86	heating	4,836	62,8495	21,8146	573,61	cooling	4,836	34,8863	10,5613	340,03	heating
4,838	25,4823	5,1448	284,29	cooling	4,838	36,598	10,685	362,23	heating	4,838	62,5997	27,1558	495,45	cooling	4,838	34,6072	11,7695	319,24	heating
4,84	24,9531	4,539	285,36		4,84	36,1727	10,2699	362,08	heating	4,84	61,5077	16,9001	623,55	cooling	4,84	34,4424	11,1046	326,23	heating
4,842	26,1725	8,0097	253,89		4,842	36,0643	15,8248	282,92	heating	4,842	61,7168	21,6411	560,2	cooling	4,842	34,7512	12,5713	310,04	
4,844	25,8807	5,5438	284,28		4,844	35,6864	19,3663	228,13	heating	4,844	60,9696	27,3435	470,04	cooling	4,844	34,6339	13,426	296,45	
4,846	26,6149	4,3243	311,59		4,846	35,4295	16,6768	262,14	heating	4,846	66,104	11,8712	758,09	cooling	4,846	35,1906	16,4839	261,49	

4,848	26,4529	6,3834	280,54		4,848	34,7009	10,537	337,78	heating	4,848	63,4484	19,7235	611,21	cooling	4,848	34,822	12,7356	308,74	
4,85	26,1865	2,3248	333,55		4,85	35,3343	9,4115	362,36	heating	4,85	62,8925	20,0158	599,35	cooling	4,85	35,7353	11,8572	333,78	
4,852	26,6218	-0,2878	376,16		4,852	35,24	11,9301	325,84	heating	4,852	63,6675	13,8389	696,53	cooling	4,852	35,3556	12,1799	323,96	
4,854	26,492	3,9329	315,34		4,854	35,7526	14,274	300,24	heating	4,854	65,0854	26,2527	542,82	cooling	4,854	36,1163	10,8905	352,62	
4,856	26,7526	5,8285	292,49		4,856	36,0854	15,8487	282,88	heating	4,856	62,3534	27,5579	486,39	cooling	4,856	35,5627	13,1749	312,95	
4,858	26,9223	10,5686	228,6		4,858	37,0965	13,4921	329,95	heating	4,858	68,2075	20,8723	661,68	cooling	4,858	35,361	10,395	348,99	
4,86	26,7366	10,6661	224,64		4,86	36,9093	13,3933	328,72	heating	4,86	66,3861	31,5705	486,67	cooling	4,86	35,5912	10,5427	350,14	
4,862	26,3053	5,1448	295,79		4,862	36,5132	12,7534	332,13		4,862	65,9356	26,0267	557,87	cooling	4,862	36,2044	14,1569	308,19	
4,864	27,3784	5,7577	302,23		4,864	36,5008	16,8729	274,37		4,864	65,388	15,8938	691,86	cooling	4,864	35,2191	15,9983	268,68	
4,866	26,8669	6,8976	279,14		4,866	35,9606	16,9878	265,21		4,866	67,2344	20,4511	653,96	cooling	4,866	35,3254	12,7606	315,42	
4,868	26,9818	6,1175	291,65		4,868	35,3016	16,0195	269,54		4,868	61,3043	8,0607	744,27	cooling	4,868	35,9175	14,6189	297,72	
4,87	26,8691	8,2305	260,54		4,87	34,8049	12,7118	308,83		4,87	63,8622	17,678	645,59	cooling	4,87	35,557	10,0734	356,22	
4,872	26,576	4,7991	304,41		4,872	35,5382	13,9476	301,8		4,872	61,3668	28,0078	466,31	cooling	4,872	35,7871	13,9769	304,87	
4,874	26,4298	1,3552	350,51		4,874	35,3136	13,7847	300,94		4,874	62,295	25,436	515,23	cooling	4,874	35,0233	12,9474	308,59	
4,876	26,2563	1,2193	349,98		4,876	35,8766	15,8715	279,64		4,876	61,7045	24,4042	521,4	cooling	4,876	35,7841	13,8287	306,9	
4,878	26,4782	1,1953	353,42		4,878	35,9632	14,3148	302,61		4,878	65,1045	32,9405	449,6	cooling	4,878	34,8313	13,1896	302,52	
4,88	26,5922	2,9423	330,59		4,88	36,8503	14,3063	315,13		4,88	64,0955	18,3754	639,1	cooling	4,88	34,9604	14,5652	285,09	
4,882	26,3779	5,6605	289,6		4,882	36,145	15,1966	292,83		4,882	63,7415	27,2088	510,67	cooling	4,882	34,2713	11,3105	320,96	
4,884	26,367	3,8428	314,85		4,884	36,4886	17,1701	270,04		4,884	61,0554	23,9427	518,78	cooling	4,884	35,3147	14,8542	286,01	
4,886	27,5867	6,1364	299,84		4,886	36,1634	18,9747	240,27		4,886	60,6446	12,7536	669,44	cooling	4,886	34,1235	12,2275	306,07	
4,888	27,1294	7,8616	269,34		4,888	36,285	17,9098	256,86	heating	4,888	59,2106	12,1362	658,03	cooling	4,888	35,4944	11,7006	332,6	
4,89	27,4854	5,6096	305,79		4,89	36,0854	15,6316	285,91	heating	4,89	58,8162	17,113	582,95	cooling	4,89	34,9118	9,6826	352,67	
4,892	27,7175	5,4506	311,26		4,892	36,547	12,4077	337,43	heating	4,892	62,9143	11,4897	718,84	cooling	4,892	35,1766	5,0703	420,84	
4,894	27,5741	3,2852	339,52		4,894	36,6729	15,419	297,1	heating	4,894	61,1231	28,8406	451,26	cooling	4,894	35,3768	8,1948	379,96	cooling
4,896	27,2805	2,4136	347,6		4,896	36,6521	17,0704	273,72		4,896	60,3232	21,7864	538,69	cooling	4,896	35,6284	13,0872	315,09	cooling
4,898	27,7614	5,1881	315,54		4,898	37,1737	17,4371	275,89	heating	4,898	60,202	11,8116	676,43	cooling	4,898	34,9784	17,583	243,16	cooling
4,9	27,3939	3,0494	340,3		4,9	37,3362	12,9478	340,91	heating	4,9	62,5672	23,493	546,2	cooling	4,9	34,9369	17,2597	247,1	cooling
4,902	27,3245	1,5171	360,75		4,902	37,5723	15,6695	306,17		4,902	57,4494	19,3851	532,08	cooling	4,902	35,127	14,3765	290,06	
4,904	27,0215	1,8462	351,91	cooling	4,904	37,3165	19,1554	253,86		4,904	63,2487	24,7488	538,17	cooling	4,904	35,1895	7,5477	386,39	
4,906	27,0288	1,4085	358,13	cooling	4,906	38,1209	23,8339	199,71	heating	4,906	60,2713	34,7756	356,39	cooling	4,906	35,1752	11,5367	330,43	cooling
4,908	27,2265	4,2433	321,27	cooling	4,908	37,4371	21,2952	225,64	heating	4,908	61,2374	25,9528	493,23	cooling	4,908	34,4201	10,2217	338,26	cooling
4,91	26,6179	5,6274	293,42	cooling	4,91	37,8363	16,8822	292,91	heating	4,91	60,0055	17,1003	599,75	cooling	4,91	35,2507	14,9486	283,79	cooling
4,912	27,4786	7,2384	282,93	cooling	4,912	37,2472	11,7677	356,17	heating	4,912	63,9509	29,0623	487,69	cooling	4,912	34,3267	16,9853	242,41	cooling
4,914	27,1317	9,6645	244,17	cooling	4,914	37,753	13,4088	340,3	heating	4,914	58,1681	8,9397	688,14	cooling	4,914	34,6594	18,4034	227,24	
4,916	26,7303	7,7151	265,8	cooling	4,916	36,691	16,1721	286,83	heating	4,916	59,3031	17,0146	591,13	cooling	4,916	34,4213	16,5707	249,52	
4,918	26,838	5,3709	300,08		4,918	37,1422	18,2391	264,24	heating	4,918	55,6735	18,6261	517,87	cooling	4,918	34,749	15,8439	264,27	
4,92	27,5879	5,2072	312,85		4,92	36,2705	19,9417	228,25	heating	4,92	56,1106	12,9584	603,2	cooling	4,92	34,1601	12,4029	304,13	
4,922	26,8526	1,6098	352,86		4,922	36,2088	16,4842	275,72	heating	4,922	55,2658	10,1662	630,43	cooling	4,922	35,3183	14,1607	295,75	
4,924	27,5537	4,4795	322,54	cooling	4,924	36,2932	15,7325	287,41	heating	4,924	59,9282	20,2033	555,29	cooling	4,924	34,5235	13,1789	298,37	
4,926	27,2351	5,0811	309,68	cooling	4,926	36,9066	15,4707	299,64	heating	4,926	61,887	5,8088	783,89	cooling	4,926	34,8143	8,8328	363,18	
4,928	27,2179	2,8098	341,19	cooling	4,928	36,913	16,4392	286,19	heating	4,928	61,2435	27,6015	470,27	cooling	4,928	34,9445	10,6047	340,24	
4,93	27,0897	4,3503	317,86	cooling	4,93	36,9622	16,182	290,48	heating	4,93	61,0125	30,5256	426,16	cooling	4,93	35,1334	9,8992	352,74	
4,932	26,6817	5,8416	291,31	cooling	4,932	37,7547	17,7737	279,31	heating	4,932	61,7747	21,7546	559,42	cooling	4,932	34,8549	13,74	295,15	
4,934	26,8611	4,6111	311,02	cooling	4,934	37,0635	11,6957	354,6	heating	4,934	62,577	22,0109	567,05	cooling	4,934	34,5324	12,3344	310,3	
4,936	26,4728	7,6615	262,95	cooling	4,936	37,8593	13,5569	339,71	heating	4,936	61,1194	17,3074	612,43	cooling	4,936	35,0278	10,6756	340,41	
4,938	26,0094	2,4254	329,67	cooling	4,938	36,8186	13,971	319,38		4,938	66,7684	11,6885	769,94	cooling	4,938	35,0233	6,9309	392,69	
4,94	26,3645	-0,3074	372,83	cooling	4,94	37,4589	15,9764	300,29		4,94	63,8599	28,6703	491,9	cooling	4,94	34,7884	9,0553	359,71	cooling
4,942	26,4803	1,1908	353,51	cooling	4,942	36,369	15,3144	294,31		4,942	63,173	30,519	456,45	cooling	4,942	34,452	5,687	402,09	cooling
4,944	25,5398	-1,0608	371,84	cooling	4,944	37,1661	15,2425	306,46		4,944	62,8699	24,5136	536,16	cooling	4,944	35,253	8,7897	369,92	cooling
4,946	26,6979	3,2968	327,11	cooling	4,946	37,2471	14,4067	319,28		4,946	63,9212	26,3749	524,84	cooling	4,946	34,1672	7,3175	375,32	cooling
4,948	25,9668	4,5343	299,59	cooling	4,948	38,5458	20,173	256,82		4,948	59,5641	13,0693	649,93	cooling	4,948	34,9315	9,623	353,78	cooling
4,95	25,7942	1,4673	340,05	cooling	4,95	37,9766	20,9903	237,44		4,95	63,8326	11,4174	732,69	cooling	4,95	34,3449	11,5488	318,66	cooling
4,952	25,4649	3,6397	305,08	cooling	4,952	38,7936	18,4416	284,49	heating	4,952	61,2033	21,9573	548,6	cooling	4,952	34,6558	12,2176	313,65	cooling
4,954	25,214	6,62	259,92	cooling	4,954	38,3625	17,5994	290,24	heating	4,954	61,1246	26,2667	487,26	cooling	4,954	34,1178	11,6545	314	cooling
4,956	24,0487	4,2737	276,42	cooling	4,956	37,4771	15,0532	313,45	heating	4,956	59,6699	22,5689	518,62	cooling	4,956	34,6746	11,7151	320,94	cooling
4,958	24,2544	8,4587	220,8	cooling	4,958	37,5295	19,0921	257,73		4,958	63,715	33,6715	419,96	cooling	4,958	33,921	9,3153	343,95	cooling

4,96	23,7215	4,4289	269,68	cooling	4,96	36,9788	19,8052	240,06	heating	4,96	60,0909	16,5326	608,88	cooling	4,96	34,4621	8,7933	358,81	cooling
4,962	24,2974	2,8964	299,15		4,962	36,3968	17,2512	267,63	heating	4,962	57,9208	18,7229	547,93	cooling	4,962	34,5406	10,4052	337,38	cooling
4,964	24,5777	6,399	254,11		4,964	36,2503	13,0957	323,67	heating	4,964	57,1509	20,9114	506,58	cooling	4,964	35,0787	11,695	326,87	cooling
4,966	24,0581	5,9258	253,46		4,966	37,448	15,3663	308,67	heating	4,966	57,523	20,3953	518,99	cooling	4,966	34,4812	16,1505	256,24	cooling
4,968	24,9015	5,817	266,77		4,968	37,2021	12,8175	340,86	heating	4,968	57,3815	18,3142	546,1	cooling	4,968	33,989	12,3068	303,08	cooling
4,97	24,1765	7,7009	230,3		4,97	37,7396	15,8941	305,37		4,97	62,3734	31,6209	429,87	cooling	4,97	34,6951	10,7704	334,43	cooling
4,972	24,0496	1,9342	309,14		4,972	37,297	13,1458	337,6		4,972	66,6382	23,8582	598	cooling	4,972	34,6797	5,3603	409,84	cooling
4,974	24,2302	1,6282	315,94		4,974	38,5484	13,1425	355,14		4,974	63,5238	31,4178	448,79	cooling	4,974	34,5076	6,6626	389,23	cooling
4,976	24,2691	2,0437	310,68		4,976	38,0089	12,778	352,69		4,976	64,2434	35,4381	402,66	cooling	4,976	34,0772	7,5481	370,84	cooling
4,978	23,9266	-0,4276	340,44		4,978	38,4353	14,6395	332,63		4,978	63,6307	28,4548	491,71	cooling	4,978	34,6587	12,0807	315,61	cooling
4,98	25,2406	3,4419	304,71		4,98	38,1851	17,1669	293,8		4,98	64,4705	32,5469	446,24	cooling	4,98	33,8687	9,5199	340,36	cooling
4,982	25,0172	4,5139	286,61		4,982	38,3898	18,5941	276,71		4,982	59,7179	29,8754	417,15	cooling	4,982	34,2968	13,0357	297,2	cooling
4,984	25,359	4,6622	289,31		4,984	37,6973	15,9155	304,48		4,984	64,4686	24,1194	564,02	cooling	4,984	33,6912	8,8343	347,46	cooling
4,986	25,7524	8,8449	236,34		4,986	38,3792	14,5087	333,67		4,986	60,7925	24,0391	513,76	cooling	4,986	34,4666	9,8214	344,5	cooling
4,988	26,1528	10,4406	219,63		4,988	37,9659	16,5758	299		4,988	60,1101	18,9865	574,85	cooling	4,988	33,1976	8,5485	344,56	cooling
4,99	25,9546	5,2646	289,22		4,99	37,6894	17,7789	278,32		4,99	58,6627	6,4039	730,5	cooling	4,99	33,8614	10,0301	333,13	cooling
4,992	25,8859	5,9611	278,52		4,992	37,8819	22,967	208,49		4,992	62,8776	18,2975	623,16	cooling	4,992	33,3858	13,0108	284,81	cooling
4,994	25,5901	2,5837	321,6		4,994	37,5112	19,0157	258,54		4,994	58,2397	8,2191	699,21	cooling	4,994	33,4178	14,8213	259,95	cooling
4,996	26,081	3,8312	311,02		4,996	37,6478	16,8437	290,81		4,996	58,1663	14,6794	607,88	cooling	4,996	33,3882	16,6575	233,87	cooling
4,998	26,3753	6,5943	276,51		4,998	37,1615	12,9013	339,12		4,998	58,1843	21,9567	506,41	cooling	4,998	34,2365	15,2598	265,27	cooling
5	25,8941	2,5722	326,01		5	38,1916	17,2471	292,77		5	59,17	23,1358	503,7	cooling	5	33,7241	15,0596	260,9	cooling
5,002	27,5638	2,0783	356,25		5,002	37,8477	21,8483	223,65		5,002	59,6905	21,4819	534,1	cooling	5,002	33,6989	10,2282	328,09	cooling
5,004	27,1718	2,8957	339,34		5,004	38,0824	24,7095	186,93		5,004	64,7101	34,5935	420,99	cooling	5,004	34,6034	13,0253	301,63	cooling
5,006	27,2124	2,0936	351,12		5,006	37,6325	16,4997	295,4	heating	5,006	67,726	24,9745	597,6	cooling	5,006	34,4346	10,6835	332,01	cooling
5,008	27,0283	3,1586	333,66		5,008	38,9626	15,9967	321,03	heating	5,008	66,3608	30,3803	502,96	cooling	5,008	34,3516	15,696	260,78	cooling
5,01	27,5516	5,7058	305,37		5,01	37,9338	14,1379	332,63	heating	5,01	67,0326	30,9321	504,63	cooling	5,01	33,9164	14,081	277,27	cooling
5,012	26,4013	2,3509	336,19		5,012	38,8563	17,34	300,77		5,012	67,114	23,364	611,56	cooling	5,012	35,0118	16,998	251,81	cooling
5,014	26,9428	7,0336	278,3		5,014	37,9268	16,787	295,5		5,014	67,1302	23,9084	604,18	cooling	5,014	34,0227	12,1914	305,17	cooling
5,016	26,2062	6,7445	272,05		5,016	37,9157	12,6606	353,03		5,016	68,9158	32,7218	505,94	cooling	5,016	34,3243	12,2677	308,32	cooling
5,018	26,3822	5,4786	292,2		5,018	38,4038	15,1295	325,34		5,018	75,5765	27,4924	672,14	cooling	5,018	33,5285	8,4796	350,15	cooling
5,02	26,2181	4,0245	310,23		5,02	38,4006	15,213	324,13		5,02	72,9827	42,4586	426,68	cooling	5,02	34,2298	9,6736	343,26	cooling
5,022	26,4896	1,948	343,06		5,022	38,8583	17,6481	296,49		5,022	72,8209	47,1231	359,22	cooling	5,022	33,1337	9,7434	326,96	cooling
5,024	27,2076	-0,2299	383,54		5,024	38,6478	18,6884	279		5,024	71,9836	33,9579	531,54	cooling	5,024	33,9624	11,2981	316,81	cooling
5,026	26,7417	4,4678	311,36		5,026	38,907	17,823	294,72		5,026	72,8196	34,5585	534,83	cooling	5,026	33,2711	10,9255	312,36	cooling
5,028	26,7358	4,0904	316,55		5,028	38,4352	11,8886	371,08		5,028	68,0778	26,9093	575,47	cooling	5,028	33,6775	11,2803	313,08	cooling
5,03	27,1083	4,1401	321,06		5,03	38,2381	14,8523	326,9		5,03	72,8625	20,7817	728,01	cooling	5,03	33,1875	12,0765	295,1	cooling
5,032	27,472	6,3933	294,65		5,032	37,4378	14,0018	327,6		5,032	69,6824	26,745	600,2	cooling	5,032	33,969	13,1713	290,72	cooling
5,034	26,3233	3,8482	314,17		5,034	38,066	19,6422	257,54		5,034	68,6379	23,7999	626,77	cooling	5,034	33,3054	15,9361	242,8	cooling
5,036	27,3178	6,4496	291,71		5,036	37,0619	18,6766	257		5,036	67,1966	11,8267	773,99	cooling	5,036	33,2032	13,6672	273,08	cooling
5,038	26,7961	7,5241	269,39		5,038	37,5799	17,362	282,62		5,038	68,1562	19,3554	682,16	cooling	5,038	33,9044	17,4491	230,02	
5,04	26,606	7,8278	262,49		5,04	37,8103	16,7849	293,9		5,04	64,1572	21,9052	590,62	cooling	5,04	33,6011	11,191	313,26	
5,042	26,6628	9,0306	246,47		5,042	38,2322	17,3914	291,32		5,042	68,8046	23,1076	638,77	cooling	5,042	33,5359	9,8866	330,58	
5,044	27,2097	10,7728	229,76		5,044	38,3598	18,5856	276,41		5,044	67,1759	34,5499	456,06	cooling	5,044	33,4016	8,8394	343,34	
5,046	27,0732	4,6728	313,12		5,046	38,9147	19,9899	264,54		5,046	67,5961	34,1214	467,93	cooling	5,046	33,9378	10,2433	331,21	
5,048	27,0348	5,4664	301,49		5,048	38,7232	17,4336	297,6		5,048	67,8467	23,2174	623,85	cooling	5,048	33,4477	8,7539	345,18	cooling
5,05	27,0411	3,1749	333,61		5,05	38,7943	15,2389	329,27		5,05	69,9666	30,4374	552,56	cooling	5,05	33,6151	14,2876	270,17	cooling
5,052	26,8547	1,0246	361,07		5,052	39,0119	19,4368	273,63		5,052	67,1373	30,4452	512,9	cooling	5,052	32,7758	10,0789	317,27	
5,054	26,9719	2,2352	345,78		5,054	39,2956	18,565	289,78		5,054	72,4891	25,7165	653,81	cooling	5,054	33,7507	11,7048	308,17	cooling
5,056	26,5743	3,0288	329,13		5,056	39,3616	20,4286	264,66		5,056	74,94	41,102	473,01	cooling	5,056	32,5839	8,2213	340,55	cooling
5,058	27,1758	3,8716	325,76		5,058	39,2051	15,3821	333,01		5,058	76,3609	41,5616	486,44	cooling	5,058	33,4275	6,1737	380,97	cooling
5,06	26,9656	8,2857	261,12		5,06	40,0941	14,796	353,63		5,06	74,3912	29,9455	621,28	cooling	5,06	33,1921	11,9414	297,05	cooling
5,062	27,2893	8,3809	264,31		5,062	40,2747	14,4067	361,6		5,062	76,117	39,3794	513,54	cooling	5,062	32,9548	15,0098	250,84	cooling
5,064	27,6171	5,7834	305,2		5,064	39,7306	17,7336	307,49		5,064	69,5715	25,3692	617,88	cooling	5,064	33,0382	18,4856	203,42	cooling
5,066	28,2278	8,031	282,32		5,066	39,4399	17,7643	302,99		5,066	70,6061	26,5362	616,03	cooling	5,066	33,691	18,9858	205,56	
5,068	27,1549	3,5486	329,98		5,068	39,1257	17,9834	295,54		5,068	69,655	30,2009	551,51	cooling	5,068	32,5798	14,3174	255,28	
5,07	27,952	5,003	320,79		5,07	38,5511	15,8837	316,86		5,07	69,4593	23,4419	643,25	cooling	5,07	32,9075	9,8025	322,97	

5,072	27,6069	7,8846	275,69		5,072	38,289	18,0147	283,41		5,072	69,5741	18,9405	707,78	cooling	5,072	33,2554	13,6028	274,71	
5,074	26,9303	6,6308	283,76		5,074	38,2151	19,6079	260,1		5,074	73,1895	33,0472	561,13	cooling	5,074	32,9452	9,889	322,29	
5,076	27,2012	6,5735	288,34		5,076	38,4419	21,6989	234,04		5,076	72,4226	27,3916	629,47	cooling	5,076	33,0974	16,8263	227,45	
5,078	27,228	7,507	275,67		5,078	37,8596	23,0511	207		5,078	71,4012	41,3452	420,14	cooling	5,078	32,0717	14,8505	240,73	
5,08	27,2579	2,1308	351,24		5,08	38,3298	19,7125	260,24		5,08	70,2881	45,9042	340,85	cooling	5,08	33,2623	15,2006	252,48	
5,082	27,3673	6,4521	292,36		5,082	38,4033	19,1704	268,85		5,082	68,9134	32,7307	505,78	cooling	5,082	32,477	15,4759	237,65	
5,084	27,2061	8,7734	257,66		5,084	38,6422	18,3608	283,5	heating	5,084	68,0525	24,6047	607,34	cooling	5,084	32,636	13,5736	266,46	
5,086	26,7371	5,5493	296,17		5,086	38,6546	19,317	270,31	heating	5,086	69,2228	27,1801	587,7	cooling	5,086	32,6467	9,158	328,34	heating
5,088	27,2963	8,8331	258,09		5,088	39,4407	18,7699	288,95	heating	5,088	73,8403	13,7455	840,04	cooling	5,088	33,5219	12,0777	299,76	
5,09	26,3609	6,4594	278,19		5,09	38,3924	16,9609	299,58	heating	5,09	70,9988	23,695	661,24	cooling	5,09	32,3505	8,2787	336,49	
5,092	26,8742	4,0405	319,18		5,092	38,4857	12,4915	363,36	heating	5,092	71,3993	25,3331	643,94	cooling	5,092	33,9455	10,8606	322,69	
5,094	26,5237	6,548	279,23		5,094	39,3116	18,5795	289,8		5,094	77,212	22,0179	771,53	cooling	5,094	32,7691	13,4872	269,53	
5,096	26,2606	2,9378	326,02		5,096	39,1343	19,4104	275,71		5,096	81,6885	28,8381	738,77	cooling	5,096	32,8503	10,4663	312,9	
5,098	26,9226	-1,7215	400,4		5,098	39,3098	19,4493	277,62		5,098	80,8406	27,9405	739,47	cooling	5,098	33,2905	15,5694	247,71	
5,1	27,4096	1,9093	356,46		5,1	38,979	17,5197	299,97		5,1	88,4011	30,6566	807,18	cooling	5,1	33,2457	13,912	270,26	
5,102	26,8622	-0,7226	385,6		5,102	39,3533	12,9752	368,73		5,102	87,3913	48,1183	548,98	cooling	5,102	33,0411	14,3293	261,56	
5,104	27,3414	4,8967	313,74		5,104	38,9093	11,6202	381,46		5,104	87,4324	48,0425	550,61	cooling	5,104	33,1179	17,2068	222,41	heating
5,106	26,8297	9,0246	248,89		5,106	39,4432	18,4388	293,61		5,106	86,8574	33,2234	749,72	cooling	5,106	33,4976	19,7285	192,47	heating
5,108	25,9146	4,7097	296,41		5,108	38,1509	16,2585	306,02		5,108	89,3176	38,3778	712,06	cooling	5,108	33,5232	15,9115	246,19	heating
5,11	26,3933	5,5624	291,19		5,11	39,1591	16,8082	312,43		5,11	82,4552	25,4175	797,3	cooling	5,11	33,4437	17,3551	224,89	heating
5,112	25,2987	5,0585	282,93		5,112	37,736	17,0386	289,32		5,112	84,1423	26,2402	809,39	cooling	5,112	32,9701	10,7669	310,37	heating
5,114	25,3446	1,7006	330,51		5,114	38,3909	14,8328	329,31		5,114	82,7414	35,0217	667,05	cooling	5,114	34,288	15,5616	261,77	heating
5,116	25,1583	5,8434	269,99		5,116	38,2967	19,4294	263,74		5,116	82,7177	34,7209	670,92	cooling	5,116	32,929	14,454	258,25	heating
5,118	24,8266	4,3056	286,85		5,118	38,4614	21,6731	234,68		5,118	82,4269	29,8246	735,3	cooling	5,118	33,6508	14,3715	269,5	heating
5,12	25,0877	-1,476	371,32		5,12	38,701	19,6893	265,76		5,12	88,0654	50,7565	521,52	cooling	5,12	33,3063	15,7497	245,42	heating
5,122	26,16	1,7784	340,82		5,122	39,7352	21,5035	254,85		5,122	86,1734	40,2245	642,3	cooling	5,122	33,1443	11,9436	296,35	heating
5,124	25,5829	0,4756	350,96		5,124	39,1363	19,9691	267,93		5,124	85,456	41,3988	615,85	cooling	5,124	33,2808	11,1217	309,75	heating
5,126	26,2356	4,6115	302,27	cooling	5,126	39,3161	15,2115	336,95		5,126	85,2987	39,6873	637,58	cooling	5,126	33,8817	14,3968	272,37	heating
5,128	25,9405	9,4443	230,59		5,128	40,28	20,5896	275,24		5,128	85,2755	32,6996	734,93	cooling	5,128	32,7521	12,796	278,96	heating
5,13	25,7547	5,8069	278,84		5,13	38,9731	16,6281	312,35		5,13	85,0126	29,3872	777,56	cooling	5,13	33,5113	13,8059	275,45	heating
5,132	26,354	4,8262	300,93		5,132	39,868	14,9562	348,23		5,132	89,9705	45,7244	618,49	cooling	5,132	33,2736	15,0742	254,4	heating
5,134	25,5753	7,8212	248,18		5,134	39,2069	15,6633	329,11		5,134	93,6054	24,456	966,61	cooling	5,134	33,5752	9,2393	340,18	heating
5,136	25,3723	4,1537	296,6		5,136	39,767	14,8467	348,35		5,136	89,437	28,758	848,2	cooling	5,136	33,4317	13,9445	272,4	heating
5,138	25,7709	7,8238	250,87		5,138	39,4397	16,6257	318,91		5,138	89,7235	37,3858	731,6	cooling	5,138	32,8279	11,7368	294,82	heating
5,14	25,6772	8,5423	239,52		5,14	40,49	22,1055	256,99		5,14	88,1599	32,9258	772,09	cooling	5,14	33,6604	13,9101	276,08	heating
5,142	25,7897	4,8486	292,73		5,142	39,1832	12,4698	373,41		5,142	89,7976	46,095	610,9	cooling	5,142	33,3787	16,8078	231,64	heating
5,144	27,3387	8,8032	259,1		5,144	40,3687	10,4459	418,28		5,144	83,4253	42,8733	566,86	cooling	5,144	33,1806	16,994	226,26	heating
5,146	26,864	7,6773	268,2		5,146	39,3746	10,8718	398,43		5,146	88,9393	39,1909	695,41	cooling	5,146	33,2928	14,1651	267,38	heating
5,148	27,4405	6,4327	293,66		5,148	39,7966	10,4923	409,63	cooling	5,148	85,0616	39,4218	637,98	cooling	5,148	33,7354	15,5533	254,16	heating
5,15	27,626	8,4415	268,17		5,15	39,3792	17,4883	306	cooling	5,15	84,0824	29,9195	757,12	cooling	5,15	32,969	11,1411	305,12	heating
5,152	27,5606	4,9427	316,16		5,152	38,4123	16,653	304,16	cooling	5,152	82,9498	16,5848	927,68	cooling	5,152	34,2662	14,0786	282,19	heating
5,154	27,6659	1,1105	371,2		5,154	39,0526	13,338	359,45	cooling	5,154	86,7059	34,4693	730,19	cooling	5,154	33,21	13,9138	269,73	heating
5,156	27,9597	7,251	289,48		5,156	39,3543	15,2112	337,49	cooling	5,156	81,2883	28,3211	740,4	cooling	5,156	33,7483	10,6565	322,79	heating
5,158	27,6929	7,1505	287,15		5,158	38,9129	16,7804	309,38	cooling	5,158	82,0381	38,2507	612,08	cooling	5,158	33,8237	11,1756	316,59	heating
5,16	27,6907	9,1321	259,42		5,16	38,9065	15,8971	321,64	cooling	5,16	81,129	43,4897	526,14	cooling	5,16	34,2398	8,8181	355,36	heating
5,162	27,486	8,7579	261,79		5,162	40,0472	21,6714	256,87	cooling	5,162	79,9661	36,2297	611,37	cooling	5,162	33,5948	9,3945	338,29	heating
5,164	27,3583	4,3294	321,91		5,164	39,0311	16,2743	318,11	cooling	5,164	79,1716	27,9303	716,28	cooling	5,164	33,7483	12,3912	298,54	heating
5,166	28,4596	4,4871	335,1		5,166	40,0521	17,3513	317,32	cooling	5,166	82,3973	38,2541	617,06	cooling	5,166	33,9595	15,8395	253,29	heating
5,168	27,3722	5,065	311,82		5,168	39,5813	18,8936	289,18	cooling	5,168	80,493	20,8464	833,77	cooling	5,168	33,8107	14,3201	272,45	heating
5,17	27,299	1,23	364,41		5,17	39,9299	19,1391	290,62	cooling	5,17	77,275	24,8793	732,41	cooling	5,17	33,6013	17,4524	225,74	heating
5,172	27,4182	2,1858	352,71		5,172	39,2755	18,3978	291,84	cooling	5,172	77,3259	30,528	654,17	cooling	5,172	33,3605	15,2577	253,05	heating
5,174	27,1695	2,6177	343,2		5,174	39,6609	18,2848	298,81	cooling	5,174	77,5101	24,0727	746,98	cooling	5,174	34,3376	16,6395	247,39	heating
5,176	26,3139	1,6034	345,42		5,176	39,4286	11,6059	388,92	cooling	5,176	78,1975	21,4151	793,73	cooling	5,176	33,7442	13,0148	289,77	heating
5,178	26,9948	5,8496	295,58		5,178	39,7338	10,8066	404,36	cooling	5,178	82,5267	33,0024	692,28	cooling	5,178	33,9358	14,5503	270,98	heating
5,18	26,9405	6,5362	285,22		5,18	39,1376	12,4218	373,45	cooling	5,18	88,8874	24,4786	900,34	cooling	5,18	33,5944	12,424	295,93	heating
5,182	27,1347	5,5469	301,77		5,182	39,5025	14,3349	351,81	cooling	5,182	85,3611	44,364	573,08	cooling	5,182	34,5137	16,0391	258,25	heating

5,184	27,536	7,1196	285,39		5,184	39,3427	18,5964	290	cooling	5,184	84,4671	51,7163	457,81	cooling	5,184	33,6207	12,9304	289,22	heating
5,186	27,6642	5,1242	315,07		5,186	38,6836	13,5298	351,61	cooling	5,186	84,0387	32,4802	720,71	cooling	5,186	34,5087	15,3644	267,61	heating
5,188	28,5447	5,1482	327,05		5,188	39,6715	15,3781	339,59	cooling	5,188	83,2532	28,6463	763,32	cooling	5,188	33,9742	14,5191	271,95	heating
5,19	28,054	7,947	281,07		5,19	39,4624	15,5108	334,81	cooling	5,19	81,7874	35,4952	647,1	cooling	5,19	34,4008	13,6346	290,28	heating
5,192	28,6651	7,2225	299,74		5,192	39,2509	17,7056	301,17	cooling	5,192	88,965	27,8522	854,27	cooling	5,192	34,0765	11,9945	308,67	heating
5,194	28,3483	5,4713	319,79		5,194	38,9992	15,8608	323,44	cooling	5,194	84,7162	41,5927	602,8	cooling	5,194	34,5975	11,4128	324,09	heating
5,196	28,8425	6,3116	314,95		5,196	39,3047	14,8648	341,63	cooling	5,196	83,5417	37,0772	649,51	cooling	5,196	33,8207	11,3616	313,94	heating
5,198	27,6629	2,5859	350,54		5,198	39,1151	10,6016	398,58	cooling	5,198	84,6934	23,5108	855,24	cooling	5,198	33,9465	13,2818	288,86	heating
5,2	29,0447	4,7925	339,01		5,2	39,1801	17,8424	298,27	cooling	5,2	84,9632	30,6441	759,3	cooling	5,2	34,0438	16,2374	248,91	heating
5,202	28,1197	4,6635	327,88		5,202	38,7083	17,1691	301,09	cooling	5,202	85,4966	40,9337	622,92	cooling	5,202	34,1829	12,5257	302,74	heating
5,204	28,2609	4,0234	338,81		5,204	38,9394	15,6467	325,6	cooling	5,204	96,9623	28,4289	958	cooling	5,204	33,7074	13,961	276,02	heating
5,206	28,1072	4,7526	326,46	cooling	5,206	38,6569	14,5884	336,44	cooling	5,206	92,0299	40,2601	723,67	cooling	5,206	33,5768	10,5489	321,9	heating
5,208	28,9473	6,8	309,59	cooling	5,208	38,2874	12,274	363,63	cooling	5,208	91,9047	34,7419	799,05	cooling	5,208	34,4876	14,0041	286,33	heating
5,21	28,0713	3,4652	343,96	cooling	5,21	38,7297	17,7557	293,18	cooling	5,21	91,8147	20,1419	1001,88	cooling	5,21	34,1048	13,4729	288,4	heating
5,212	28,2996	2,2951	363,5	cooling	5,212	38,1923	20,3504	249,4	cooling	5,212	91,876	30,5845	856,76	cooling	5,212	34,1715	15,0069	267,89	heating
5,214	28,5399	3,8778	344,74	cooling	5,214	38,1258	19,1862	264,75	cooling	5,214	85,4122	31,4352	754,52	cooling	5,214	34,3612	15,3781	265,36	heating
5,216	28,2788	3,8747	341,13	cooling	5,216	38,8203	16,327	314,42	cooling	5,216	94,941	32,751	869,32	cooling	5,216	34,2769	16,7613	244,84	heating
5,218	27,9518	4,906	322,15	cooling	5,218	38,3818	15,4026	321,21	cooling	5,218	90,2008	46,8782	605,59	cooling	5,218	33,9112	14,6517	269,22	heating
5,22	27,8642	2,5813	353,42	cooling	5,22	38,8623	12,3004	371,3	cooling	5,22	90,0703	40,5827	691,76	cooling	5,22	34,5014	16,7025	248,8	heating
5,222	28,5464	0,3116	394,68	cooling	5,222	38,9676	19,2441	275,71	cooling	5,222	89,1248	23,6735	914,91	cooling	5,222	33,6968	14,4113	269,58	heating
5,224	27,9158	0,1453	388,19	cooling	5,224	38,8682	18,0134	291,52	cooling	5,224	92,8987	39,0234	753,1	cooling	5,224	34,0479	13,6342	285,35	heating
5,226	28,382	7,4066	293,2	cooling	5,226	38,3651	17,3615	293,6	cooling	5,226	83,3695	26,7185	791,9	cooling	5,226	33,8525	14,8065	266,23	heating
5,228	28,0187	8,6755	270,39	cooling	5,228	38,6492	16,2703	312,82	cooling	5,228	86,8109	40,7908	643,29	cooling	5,228	34,3645	10,7492	330,11	heating
5,23	28,4516	10,1114	256,37	cooling	5,23	38,1862	15,2203	321,03	cooling	5,23	83,5395	49,8117	471,46	cooling	5,23	33,9004	12,5968	297,79	heating
5,232	27,7681	9,852	250,44	cooling	5,232	38,4843	19,0474	271,7	cooling	5,232	83,3654	38,3816	628,81	cooling	5,232	34,1037	11,4186	317,1	heating
5,234	28,0358	7,3227	289,54	cooling	5,234	38,2748	20,7455	245,03	cooling	5,234	81,9173	23,8298	811,98	cooling	5,234	34,8409	10,9232	334,33	heating
5,236	27,8655	7,0029	291,63	cooling	5,236	37,9701	18,4847	272,38	cooling	5,236	89,6792	39,6029	699,99	cooling	5,236	35,7296	12,454	325,36	heating
5,238	27,2677	7,9863	269,52	cooling	5,238	38,6468	18,3534	283,67	cooling	5,238	96,6713	22,7597	1033,17	cooling	5,238	34,585	12,63	306,9	heating
5,24	27,2699	4,9881	311,47	cooling	5,24	37,5057	14,027	328,2	cooling	5,24	91,2098	46,6634	622,69	cooling	5,24	35,1381	11,6137	328,84	heating
5,242	28,5158	5,8113	317,38	cooling	5,242	39,3894	14,3041	350,66	cooling	5,242	90,5026	54,4107	504,51	cooling	5,242	35,2958	16,0571	268,93	heating
5,244	28,2669	6,6497	302,18	cooling	5,244	37,9876	18,8921	266,93	cooling	5,244	90,3052	43,0826	660,1	cooling	5,244	34,0442	10,0868	334,89	heating
5,246	28,1048	5,2256	319,82	cooling	5,246	38,4505	18,6089	277,36	cooling	5,246	89,2455	40,2806	684,46	cooling	5,246	34,4787	9,2732	352,33	heating
5,248	28,7317	9,455	269,46	cooling	5,248	37,9147	17,9809	278,64		5,248	88,8156	38,9437	697,14	cooling	5,248	34,123	10,4488	330,93	
5,25	28,3699	6,3778	307,42	cooling	5,25	38,7966	16,3643	313,57		5,25	101,5875	21,7065	1116,62	cooling	5,25	34,1613	8,1564	363,51	
5,252	28,0998	3,4326	344,81	cooling	5,252	37,9717	14,2847	331,11		5,252	94,1868	37,3263	794,83	cooling	5,252	33,945	11,1308	318,91	
5,254	28,5946	5,9596	316,4	cooling	5,254	38,5597	18,8383	275,68		5,254	93,5722	36,9761	791,13	cooling	5,254	34,4206	13,4082	293,72	
5,256	28,8663	8,6543	282,53	cooling	5,256	38,5846	18,9199	274,88		5,256	92,8531	20,8115	1007,03	cooling	5,256	33,5939	11,8685	303,69	
5,258	28,7193	7,3951	298,08	cooling	5,258	38,1906	15,5393	316,63		5,258	97,3034	41,3024	782,81	cooling	5,258	34,4647	15,5172	264,86	
5,26	28,4873	9,1901	269,75	cooling	5,26	38,7568	19,5457	268,54		5,26	85,2761	17,1213	952,7	cooling	5,26	34,0462	15,3686	261,09	
5,262	28,2032	4,428	332,34		5,262	37,2928	14,4218	319,7		5,262	87,4106	26,8031	847,2	cooling	5,262	35,1869	12,1961	321,38	
5,264	28,4229	4,5652	333,5	cooling	5,264	39,5994	16,0428	329,29		5,264	84,5786	36,5678	671,12	cooling	5,264	34,4237	16,3825	252,19	
5,266	28,0946	7,7554	284,31	cooling	5,266	37,3997	16,7793	288,24		5,266	84,7556	30,5639	757,52	cooling	5,266	34,3327	13,5282	290,82	
5,268	28,029	6,7426	297,55	cooling	5,268	38,2178	19,4885	261,81		5,268	83,6553	26,7419	795,57	cooling	5,268	35,3914	15,1396	283,09	
5,27	28,4764	6,1951	311,46		5,27	37,0631	19,3326	247,85		5,27	90,2196	51,0143	548,03	cooling	5,27	35,3836	14,552	291,2	
5,272	27,9006	4,9614	320,66		5,272	38,2578	21,1053	239,77		5,272	87,5793	28,4146	827,03	cooling	5,272	34,7339	11,2198	328,69	
5,274	27,7305	3,0031	345,65	cooling	5,274	36,8271	13,5614	325,22		5,274	84,7759	42,7942	586,84	cooling	5,274	34,8371	11,536	325,72	
5,276	28,3349	5,4025	320,56	cooling	5,276	38,4168	19,4074	265,72		5,276	82,7452	43,91	542,86	cooling	5,276	35,0166	15,7891	268,77	
5,278	27,8523	8,0276	277,12	cooling	5,278	37,6915	19,5395	253,74		5,278	82,2356	34,6297	665,46	cooling	5,278	33,8051	10,9497	319,48	
5,28	27,87	5,9178	306,86	cooling	5,28	37,7116	20,511	240,44		5,28	80,9543	34,35	651,46	cooling	5,28	34,574	13,3542	296,62	
5,282	27,9511	7,9745	279,24	cooling	5,282	38,1209	23,6359	202,48		5,282	86,6551	53,7507	459,95	cooling	5,282	33,475	10,4712	321,56	
5,284	27,577	4,3547	324,61		5,284	37,9604	18,9446	265,81		5,284	95,9554	37,7351	813,83	cooling	5,284	34,4719	9,9124	343,31	
5,286	27,3711	3,5963	332,34	cooling	5,286	39,6477	20,1603	272,4	heating	5,286	88,853	59,6493	408,22	cooling	5,286	33,8837	11,8348	308,21	
5,288	27,5681	5,537	307,96	cooling	5,288	37,9566	19,737	254,68	heating	5,288	87,6614	53,9667	471	cooling	5,288	34,6639	11,4644	324,29	
5,29	26,9142	4,464	313,82	cooling	5,29	38,821	19,9993	263,1	heating	5,29	87,4578	33,3355	756,55	cooling	5,29	34,2629	8,6591	357,9	
5,292	26,7259	2,8584	333,63	cooling	5,292	37,9653	16,2637	303,36	heating	5,292	87,1994	35,8454	717,85	cooling	5,292	34,9662	10,8879	336,58	
5,294	26,068	3,9747	308,83	cooling	5,294	38,5075	18,0634	285,78	heating	5,294	81,0507	29,834	715,93	cooling	5,294	34,8571	11,1192	331,82	

5,296	25,745	1,1634	343,61	cooling	5,296	37,3091	10,6149	373,14	heating	5,296	91,5128	30,2948	855,74	cooling	5,296	35,4818	11,3376	337,5	
5,298	25,6557	1,7019	334,84	cooling	5,298	39,1372	18,5552	287,71		5,298	86,2129	51,8559	480,26	cooling	5,298	34,585	12,8696	303,55	
5,3	26,1606	6,3399	277,06	cooling	5,3	37,6423	16,507	295,44		5,3	86,2541	44,9775	576,99	cooling	5,3	34,3162	12,1989	309,17	
5,302	26,6673	6,1818	286,36	cooling	5,302	37,8357	19,7892	252,26		5,302	86,4711	22,8549	889,26	cooling	5,302	34,9263	16,333	259,91	
5,304	26,7357	7,4768	269,21	cooling	5,304	37,3968	20,138	241,25	heating	5,304	89,1629	24,0429	910,28	cooling	5,304	34,1836	14,2013	279,32	
5,306	26,7545	6,8478	278,27	cooling	5,306	37,5394	16,6193	292,43	heating	5,306	80,2722	10,9619	968,86	cooling	5,306	34,3975	13,6297	290,3	
5,308	26,2113	3,5589	316,65		5,308	38,7628	17,3353	299,52	heating	5,308	84,6186	23,4341	855,27	cooling	5,308	34,2247	11,9226	311,75	
5,31	27,3265	6,498	291,15		5,31	38,1718	19,1281	266,2	heating	5,31	81,5799	34,2542	661,54	cooling	5,31	35,0362	15,4503	273,78	
5,312	26,0814	4,9004	296,08		5,312	38,6721	20,5329	253,56	heating	5,312	81,3243	36,0909	632,3	cooling	5,312	34,3977	14,846	273,3	
5,314	26,884	3,2652	330,16		5,314	38,2883	22,7262	217,54	heating	5,314	80,1199	30,9755	686,97	cooling	5,314	35,3151	16,3501	265,1	
5,316	26,5318	5,4721	294,38		5,316	38,4868	22,5655	222,56	heating	5,316	85,9896	42,2473	611,45	cooling	5,316	34,8476	13,382	300,06	
5,318	26,8726	2,3106	343,34		5,318	38,2139	14,1382	336,54	heating	5,318	81,8177	22,8741	823,94	cooling	5,318	34,8477	9,8831	348,97	
5,32	26,2912	-0,5929	375,8		5,32	39,7299	20,9143	263,01	heating	5,32	80,2093	31,606	679,4	cooling	5,32	35,0895	8,9386	365,55	
5,322	27,3464	3,436	334,23	cooling	5,322	38,0155	16,8226	296,25	heating	5,322	79,5792	35,3865	617,75	cooling	5,322	35,2749	7,2401	391,89	
5,324	26,65	3,834	318,93	cooling	5,324	38,749	21,1788	245,61	heating	5,324	80,3527	36,1128	618,41	cooling	5,324	34,8276	7,3302	384,37	
5,326	27,1041	4,1428	320,96	cooling	5,326	37,7186	21,1767	231,23	heating	5,326	79,2506	32,2593	656,87	cooling	5,326	34,5959	7,7694	375	cooling
5,328	26,7371	6,9394	276,74	cooling	5,328	37,4653	18,9109	259,36	heating	5,328	86,1301	45,3171	570,5	cooling	5,328	35,7277	12,9374	318,57	cooling
5,33	26,5604	3,0272	328,96	cooling	5,33	38,0197	17,3306	289,2	heating	5,33	88,8918	27,0582	864,34	cooling	5,33	35,3011	10,7795	342,78	cooling
5,332	27,1506	4,426	317,66	cooling	5,332	38,0037	20,4465	245,42	heating	5,332	83,1218	33,8366	688,93	cooling	5,332	35,1078	13,0481	308,36	cooling
5,334	26,505	6,1296	284,82	cooling	5,334	38,3724	15,3	322,52	heating	5,334	82,6666	36,4214	646,44	cooling	5,334	34,8336	10,2595	343,51	cooling
5,336	27,2132	3,5606	330,63	cooling	5,336	38,9883	18,6041	284,94	heating	5,336	82,3575	34,5921	667,69	cooling	5,336	35,732	11,4096	339,99	cooling
5,338	26,8001	3,9604	319,27	cooling	5,338	38,795	19,1082	275,19	heating	5,338	80,4637	29,7533	708,86	cooling	5,338	34,9787	10,9596	335,75	cooling
5,34	26,6151	2,882	331,75	cooling	5,34	38,654	14,6436	335,63	heating	5,34	88,2706	48,5086	555,81	cooling	5,34	34,7109	11,4582	325,04	cooling
5,342	25,9424	-1,8666	388,73	cooling	5,342	39,5997	19,7487	277,49	heating	5,342	100,5413	30,9573	972,68	cooling	5,342	34,7622	9,1359	358,22	cooling
5,344	27,7159	3,0644	344,59	cooling	5,344	38,7291	20,7665	251,09	heating	5,344	93,4892	43,1521	703,64	cooling	5,344	34,677	10,783	334	cooling
5,346	26,0925	2,9433	323,59	cooling	5,346	37,9398	19,4986	257,78	heating	5,346	94,5919	46,4295	673,24	cooling	5,346	33,8411	8,9939	347,33	cooling
5,348	26,8336	2,5469	339,49	cooling	5,348	37,4031	19,5502	249,56	heating	5,348	94,9876	33,719	856,44	cooling	5,348	34,5223	10,4414	336,61	cooling
5,35	26,7905	6,5749	282,59	cooling	5,35	37,2622	18,9419	256,09	heating	5,35	96,1127	44,1794	725,95	cooling	5,35	34,2699	12,6305	302,49	cooling
5,352	26,8015	6,1349	288,89	cooling	5,352	37,5938	14,9678	316,28	heating	5,352	86,3132	36,139	701,36	cooling	5,352	33,8892	9,7959	336,79	cooling
5,354	26,3131	6,9299	270,95	cooling	5,354	37,9772	20,6957	241,57	heating	5,354	94,1879	31,7546	872,73	cooling	5,354	34,3961	11,9003	314,46	cooling
5,356	26,7404	12,1653	203,74	cooling	5,356	37,8931	17,5081	284,95	heating	5,356	89,7849	40,5188	688,67	cooling	5,356	34,7305	11,1032	330,27	cooling
5,358	26,4756	9,0315	243,84	cooling	5,358	39,189	18,7285	286,01	heating	5,358	88,8565	42,1476	652,92	cooling	5,358	34,0401	8,6105	355,47	cooling
5,36	26,3926	7,0698	270,1		5,36	38,5187	16,9992	300,81	heating	5,36	86,6275	23,2733	885,6	cooling	5,36	34,2602	6,9787	381,36	cooling
5,362	25,9992	7,2991	261,4		5,362	38,975	16,2452	317,73	heating	5,362	92,9608	37,2637	778,56	cooling	5,362	34,8179	8,2992	370,69	cooling
5,364	25,6334	1,4672	337,81		5,364	39,0583	17,0846	307,16	heating	5,364	84,8961	15,9107	964,31	cooling	5,364	35,2926	5,0528	422,71	cooling
5,366	26,6302	4,5763	308,28		5,366	39,0842	19,4226	274,84	heating	5,366	83,7799	27,2788	789,8	cooling	5,366	34,6142	7,6693	376,65	cooling
5,368	25,2087	4,6736	287,05		5,368	38,1475	16,7855	298,61	heating	5,368	83,9259	44,2811	554,18	cooling	5,368	34,4209	5,566	403,35	cooling
5,37	26,0148	2,2093	332,76		5,37	39,2617	21,0441	254,65	heating	5,37	83,211	41,6521	580,93	cooling	5,37	35,0921	9,3169	360,3	cooling
5,372	25,8973	3,1364	318,16		5,372	38,2326	21,2107	237,94	heating	5,372	81,2336	21,3381	837,25	cooling	5,372	34,4419	12,065	312,8	cooling
5,374	25,4953	2,9296	315,43		5,374	38,4038	15,6549	318	heating	5,374	89,3052	36,3152	740,72	cooling	5,374	33,9427	11,1698	318,33	cooling
5,376	25,4909	2,1995	325,58		5,376	38,8034	17,9193	291,93	heating	5,376	97,0485	16,7082	1123,04	cooling	5,376	34,2113	9,7236	342,3	cooling
5,378	26,9451	7,9032	266,18		5,378	39,0547	21,0381	251,85	heating	5,378	88,4476	35,0005	747,11	cooling	5,378	34,1176	10,329	332,53	cooling
5,38	26,0367	3,3326	317,37		5,38	38,6208	22,6921	222,66	heating	5,38	88,5047	49,2757	548,36	cooling	5,38	33,3997	9,8786	328,79	cooling
5,382	26,37	0,6226	359,91		5,382	38,6351	19,8759	262,23	heating	5,382	87,5132	33,4715	755,42	cooling	5,382	34,3214	12,8879	299,61	cooling
5,384	26,6027	3,2374	326,61		5,384	39,3653	17,4378	306,51	heating	5,384	87,6166	31,1511	789,3	cooling	5,384	33,9561	14,7011	269,16	cooling
5,386	26,2049	0,5474	358,65	cooling	5,386	39,5764	15,2718	339,74	heating	5,386	87,6881	44,3069	606,4	cooling	5,386	34,2213	13,2609	292,99	cooling
5,388	26,3642	2,1161	338,95	cooling	5,388	39,3585	22,2406	239,28	heating	5,388	104,6873	37,7818	935,24	cooling	5,388	34,4667	15,8656	260,02	cooling
5,39	25,7483	3,7906	306,94	cooling	5,39	38,4589	19,7799	261,1	heating	5,39	96,873	57,8612	545,33	cooling	5,39	34,8621	12,5796	311,48	cooling
5,392	26,229	1,9018	340,06	cooling	5,392	39,2105	19,3149	278,11	heating	5,392	95,7616	60,7051	490,04	cooling	5,392	34,2572	9,7353	342,78	cooling
5,394	25,8894	4,0131	305,8	cooling	5,394	38,5495	18,5676	279,32	heating	5,394	93,8644	33,2601	847,16	cooling	5,394	34,2108	7,7206	370,29	cooling
5,396	25,4803	6,2357	269,01	cooling	5,396	38,8411	19,6034	268,91	heating	5,396	97,989	56,5268	579,58	cooling	5,396	34,5908	9,7792	346,83	cooling
5,398	25,514	3,4706	308,13	cooling	5,398	38,9859	19,9881	265,56	heating	5,398	83,507	30,8017	736,74	cooling	5,398	34,6445	10,3818	339,16	cooling
5,4	26,8732	7,3768	272,53	cooling	5,4	39,3045	22,9662	228,39	heating	5,4	81,3996	38,0826	605,51	cooling	5,4	33,7689	13,0311	289,88	cooling
5,402	25,7301	4,1931	301,05	cooling	5,402	38,6977	18,3057	285,05	heating	5,402	80,7758	45,475	493,45	cooling	5,402	33,619	10,0728	329,14	cooling
5,404	26,6087	4,7276	305,86	cooling	5,404	39,7852	15,3665	341,34	heating	5,404	80,618	36,6792	614,2	cooling	5,404	34,4143	10,6349	332,4	cooling
5,406	26,4024	7,6211	262,53		5,406	39,8068	16,4601	326,35	heating	5,406	80,8389	19,6726	855,01	cooling	5,406	34,1753	11,4131	318,18	cooling

5,408	26,1619	7,2577	264,25		5,408	39,6574	14,8038	347,42	heating	5,408	92,92	44,2182	680,78	cooling	5,408	34,433	12,1333	311,72	cooling
5,41	25,4852	4,7926	289,25		5,41	39,9386	17,169	318,28	heating	5,41	105,3066	26,5643	1100,7	cooling	5,41	34,3771	13,1542	296,66	cooling
5,412	26,0708	7,6002	258,19		5,412	39,9644	16,5064	327,91	heating	5,412	95,964	49,7737	645,67	cooling	5,412	34,4237	13,3884	294,04	cooling
5,414	25,5014	3,7694	303,78		5,414	39,6502	16,4314	324,57	heating	5,414	96,9442	55,7834	575,37	cooling	5,414	33,8073	12,6802	295,32	cooling
5,416	25,9074	6,5466	270,63		5,416	39,7432	15,1668	343,54	heating	5,416	95,6953	30,547	910,68	cooling	5,416	34,4246	13,1042	298,03	cooling
5,418	25,5841	7,7385	249,45		5,418	40,1561	16,1601	335,43	heating	5,418	93,6195	26,7982	934,06	cooling	5,418	33,6746	13,6711	279,62	cooling
5,42	26,6926	5,6462	294,2		5,42	40,2153	15,4966	345,53		5,42	94,4189	38,6579	779,46	cooling	5,42	33,6625	12,4543	296,46	cooling
5,422	27,3249	7,5481	276,45		5,422	39,9178	22,6904	240,81		5,422	117,6652	28,8141	1242,01	cooling	5,422	33,723	15,4041	256,07	cooling
5,424	27,0142	6,2283	290,56		5,424	39,2294	18,7115	286,81		5,424	105,7168	56,6315	686,14	cooling	5,424	34,3341	15,4632	263,79	cooling
5,426	27,9907	6,1116	305,84		5,426	39,6589	14,7231	348,56		5,426	104,4768	46,5165	810,2	cooling	5,426	33,6895	14,9436	262,04	cooling
5,428	28,2137	4,9048	325,82		5,428	39,4472	13,5179	362,45		5,428	105,7316	11,514	1317,02	cooling	5,428	34,1063	14,7503	270,57	cooling
5,43	27,6665	1,5952	364,44		5,43	39,3737	13,9433	355,48		5,43	120,3095	57,8398	873,23	cooling	5,43	34,7915	16,1282	260,89	cooling
5,432	27,8428	1,4705	368,65		5,432	39,5892	20,0925	272,53		5,432	130,3969	15,2051	1610,21	cooling	5,432	34,549	12,5036	308,16	cooling
5,434	27,823	4,1466	330,96		5,434	39,1675	21,6939	244,26		5,434	111,0497	52,3954	819,9	cooling	5,434	34,2704	12,6937	301,61	cooling
5,436	27,2723	0,7668	370,51		5,436	38,9725	17,4171	301,31		5,436	109,8335	57,1629	736,26	cooling	5,436	33,915	8,9598	348,84	
5,438	27,3219	3,7956	328,86		5,438	40,1731	17,5332	316,47		5,438	111,8556	27,0428	1185,56	cooling	5,438	34,5175	9,4034	351,06	cooling
5,44	26,9889	4,4872	314,54	cooling	5,44	39,5791	20,9725	260,09		5,44	111,2843	59,6006	722,46	cooling	5,44	33,6393	11,3002	312,27	cooling
5,442	27,4794	7,4843	279,5	cooling	5,442	39,6226	18,2321	299,01		5,442	90,2687	31,6297	819,69	cooling	5,442	33,6831	13,2477	285,66	cooling
5,444	27,3388	11,4631	221,92		5,444	39,6618	20,416	269,03		5,444	90,0845	36,661	746,78	cooling	5,444	33,4451	14,0356	271,32	cooling
5,446	27,6576	11,2576	229,25		5,446	39,4137	14,4252	349,3		5,446	87,8523	48,1261	555,31	cooling	5,446	33,4542	15,1609	255,71	cooling
5,448	28,0233	7,6723	284,48		5,448	39,1199	11,8792	380,78		5,448	88,1145	37,7196	704,45	cooling	5,448	32,8416	13,6233	268,64	
5,45	27,9954	6,397	301,91		5,45	39,8164	13,0361	374,35		5,45	87,8625	22,2069	917,77	cooling	5,45	33,7152	12,1579	301,34	
5,452	27,8271	5,7285	308,91		5,452	40,051	14,6914	354,49		5,452	100,86	59,4306	579,12	cooling	5,452	33,0752	10,6877	312,94	
5,454	28,6543	8,5064	281,64		5,454	39,8081	13,7964	363,61		5,454	105,2682	32,5599	1016,35	cooling	5,454	32,998	8,3823	344,09	
5,456	28,0058	8,0271	279,27		5,456	39,4627	14,9604	342,51		5,456	96,394	50,8361	636,83	cooling	5,456	33,8578	13,5954	283,24	
5,458	27,8813	3,1658	345,49		5,458	39,2157	11,7297	384,21		5,458	95,5151	50,6979	626,48	cooling	5,458	34,0648	15,337	261,79	
5,46	28,2728	5,8909	312,87		5,46	39,4541	11,7858	386,76	cooling	5,46	95,2016	26,522	960,04	cooling	5,46	33,7234	16,3531	242,81	
5,462	28,1974	9,3944	262,84		5,462	38,7409	14,8414	334,08	cooling	5,462	92,5193	23,9843	958,02	cooling	5,462	33,9173	14,8929	265,93	
5,464	27,4876	12,4708	209,91		5,464	39,0353	17,7591	297,41	cooling	5,464	98,4428	48,771	694,34	cooling	5,464	34,9036	14,7999	281,02	
5,466	27,4248	12,166	213,29		5,466	38,4733	18,2005	283,38	cooling	5,466	124,0381	31,5679	1292,6	cooling	5,466	34,3593	12,1402	310,59	
5,468	27,4707	9,2914	254,12		5,468	38,3671	15,2309	323,41	cooling	5,468	111,0582	64,1584	655,59	cooling	5,468	34,1396	16,4541	247,22	
5,47	27,5713	7,2173	284,52		5,47	38,2418	12,6195	358,16	cooling	5,47	108,1496	56,1505	726,87	cooling	5,47	33,6901	15,7383	250,94	
5,472	27,1826	8,8959	255,62		5,472	39,4406	12,7338	373,32	cooling	5,472	109,5396	27,5327	1146,34	cooling	5,472	34,0932	14,5176	273,64	
5,474	27,0391	6,0225	293,78	heating	5,474	38,9752	17,8632	295,11	cooling	5,474	122,6614	77,8806	625,97	cooling	5,474	33,398	15,1244	255,44	
5,476	27,4188	5,6363	304,49	heating	5,476	38,7485	16,8582	305,99	cooling	5,476	126,7427	30,9657	1338,82	cooling	5,476	33,4893	12,2669	296,66	
5,478	26,5062	2,6146	333,97	heating	5,478	39,2245	17,4861	303,87	cooling	5,478	109,4088	46,9602	872,94	cooling	5,478	33,5192	11,2788	310,89	
5,48	27,0192	1,4049	358,05		5,48	39,5803	16,7023	319,8	cooling	5,48	108,2168	45,1481	881,61	cooling	5,48	33,4241	14,196	268,78	
5,482	26,7832	2,6277	337,66		5,482	38,9002	16,7806	309,2	cooling	5,482	109,416	14,7932	1322,69	cooling	5,482	32,8236	14,8987	250,56	
5,484	26,47	1,0498	355,34		5,484	38,8213	16,2033	316,17	cooling	5,484	106,545	35,6711	990,71	cooling	5,484	33,7822	14,3365	271,82	
5,486	26,4184	0,9366	356,2		5,486	38,9574	18,0495	292,26	cooling	5,486	92,9783	40,7546	730,01	cooling	5,486	33,6325	16,3259	241,92	
5,488	26,8549	3,2606	329,81		5,488	37,8555	15,0316	319,04	cooling	5,488	104,8675	40,1102	905,21	cooling	5,488	32,969	9,6497	325,97	heating
5,49	25,8768	4,3388	301,07		5,49	37,9922	18,6308	270,64	cooling	5,49	99,3799	55,736	610,08	cooling	5,49	34,3769	14,0283	284,44	heating
5,492	26,1791	7,3988	262,52		5,492	37,4323	18,1689	269,27	cooling	5,492	98,2712	45,288	740,63	cooling	5,492	34,563	17,0969	244,15	heating
5,494	26,3175	9,3708	236,89		5,494	38,0619	15,3347	317,69	cooling	5,494	98,4809	26,0069	1013,08	cooling	5,494	33,871	16,0299	249,39	heating
5,496	26,8249	5,7968	293,94		5,496	37,4827	14,8019	317,04	cooling	5,496	106,8112	61,2125	637,4	cooling	5,496	34,4913	16,9802	244,78	heating
5,498	27,2721	7,8936	270,88		5,498	37,9933	13,7752	338,53	cooling	5,498	97,5531	35,5818	866,27	cooling	5,498	34,9323	18,252	233,17	heating
5,5	27,3213	7,9228	271,16		5,5	37,7542	14,4827	325,3	cooling	5,5	93,0395	42,5494	705,78	cooling	5,5	34,6029	12,619	307,3	heating
5,502	27,9746	6,3843	301,8		5,502	37,972	14,9042	322,45	cooling	5,502	91,0213	46,4076	623,63	cooling	5,502	34,5166	17,8519	232,95	heating
5,504	27,6481	4,1356	328,67		5,504	37,7676	11,8539	362,24	cooling	5,504	90,7167	29,9486	849,45	cooling	5,504	33,9451	15,2825	260,88	heating
5,506	27,3122	0,3316	377,15		5,506	39,1781	14,402	346,33	cooling	5,506	89,7036	19,4515	982,02	cooling	5,506	34,4328	10,5892	333,3	heating
5,508	27,5331	0,4269	378,9		5,508	38,2712	17,6468	288,3	cooling	5,508	100,6743	52,6279	671,62	cooling	5,508	34,2938	14,9211	270,8	heating
5,51	27,6916	5,7944	306,09		5,51	37,9329	13,7172	338,5	cooling	5,51	111,1007	32,7224	1095,61	cooling	5,51	33,3108	11,5383	304,35	heating
5,512	26,8263	5,7187	295,05		5,512	39,057	17,3172	303,89	cooling	5,512	103,5602	48,2274	773,47	cooling	5,512	34,2143	13,3212	292,05	heating
5,514	27,7398	7,5676	281,98		5,514	38,3579	14,2763	336,62	cooling	5,514	103,5132	45,3855	812,54	cooling	5,514	33,7348	17,5176	226,69	heating
5,516	27,5942	8,4354	267,81		5,516	38,7136	15,4455	325,25	cooling	5,516	104,6903	27,3857	1080,6	cooling	5,516	33,3808	15,7058	247,07	heating
5,518	27,8359	7,4446	285,04		5,518	38,0664	17,9237	281,56	cooling	5,518	104,344	41,075	884,41	cooling	5,518	34,6026	15,8324	262,38	heating

5,52	28,0074	10,7565	241,14		5,52	38,0364	16,2021	305,21	cooling	5,52	100,4631	49,4985	712,41	cooling	5,52	34,3002	19,2501	210,38	heating
5,522	28,5565	10,0036	259,34		5,522	37,0277	12,6475	340,8	cooling	5,522	111,772	32,8623	1103,04	cooling	5,522	33,6756	10,45	324,66	heating
5,524	28,1877	2,6842	356,5		5,524	37,5451	17,2701	283,41	cooling	5,524	104,1932	47,808	788,18	cooling	5,524	35,2652	15,3052	279,01	heating
5,526	28,5724	1,4009	379,82		5,526	36,6439	16,477	281,9	cooling	5,526	101,5368	42,5752	824,2	cooling	5,526	34,5204	14,3552	281,88	heating
5,528	28,2203	1,4249	374,56		5,528	37,1881	18,886	255,84	cooling	5,528	101,8719	23,4728	1095,9	cooling	5,528	33,904	11,8183	308,73	heating
5,53	28,87	1,8074	378,3		5,53	36,1528	18,0094	253,62	cooling	5,53	104,0732	43,1287	851,91	cooling	5,53	34,0911	14,1316	279	heating
5,532	28,5647	4,6863	333,78		5,532	36,9033	15,5817	298,04	cooling	5,532	95,658	42,4957	743,13	cooling	5,532	34,4612	15,1915	269,36	heating
5,534	27,976	1,2649	373,38	cooling	5,534	37,1344	21,5563	217,76		5,534	103,3561	42,8452	845,85	cooling	5,534	34,4268	14,8454	273,72	heating
5,536	28,8997	1,3627	384,93	cooling	5,536	36,6051	20,2165	229,09		5,536	100,7508	53,6826	657,94	cooling	5,536	34,283	18,5325	220,17	heating
5,538	29,0209	5,1203	334,09	cooling	5,538	38,004	17,7696	282,85		5,538	100,9645	40,8091	840,88	cooling	5,538	34,1224	13,304	291,01	heating
5,54	28,4784	4,9127	329,41	cooling	5,54	38,2415	16,6611	301,66		5,54	102,4499	29,9467	1013,49	cooling	5,54	35,1017	12,5769	314,86	heating
5,542	28,5269	5,0052	328,8	cooling	5,542	38,2382	16,1799	308,34		5,542	108,0937	58,4528	693,91	cooling	5,542	34,2383	16,645	245,93	heating
5,544	28,7927	7,6785	295,15	cooling	5,544	38,055	16,1262	306,53		5,544	100,0172	41,789	813,94	cooling	5,544	34,7096	15,5709	267,53	heating
5,546	27,3716	3,1385	338,74	cooling	5,546	39,4527	22,7186	233,92		5,546	100,1243	45,5045	763,5	cooling	5,546	35,0167	17,5679	243,91	heating
5,548	27,6897	4,6239	322,43	cooling	5,548	37,9425	16,9506	293,44		5,548	97,814	47,8046	699,06	cooling	5,548	34,2365	13,4073	291,16	heating
5,55	27,06	2,7545	339,75	cooling	5,55	39,3019	21,1162	254,21		5,55	98,0844	36,6095	859,33	cooling	5,55	34,4535	11,6158	319,24	heating
5,552	27,6994	-1,8187	412,62	cooling	5,552	37,8125	19,4541	256,62		5,552	98,0085	30,9536	937,33	cooling	5,552	35,2051	12,3075	320,07	heating
5,554	28,2555	1,064	380,1	cooling	5,554	38,3811	15,0355	326,34		5,554	105,1913	48,1046	797,99	cooling	5,554	34,4523	13,3523	294,95	heating
5,556	28,6255	2,8797	359,89	cooling	5,556	37,8008	16,0007	304,73		5,556	110,4265	35,8059	1043,09	cooling	5,556	34,4968	12,8287	302,89	heating
5,558	28,7539	3,1696	357,63	cooling	5,558	37,9544	16,9914	293,03		5,558	107,5265	56,2638	716,58	cooling	5,558	35,6746	18,1912	244,39	heating
5,56	28,9011	7,7606	295,51	cooling	5,56	38,0065	13,404	343,91		5,56	107,1837	60,8753	647,32	cooling	5,56	34,8996	12,6433	311,11	heating
5,562	28,5082	6,4492	308,35	cooling	5,562	38,2325	16,3588	305,76		5,562	108,0525	54,9353	742,5	cooling	5,562	34,8632	12,0929	318,29	heating
5,564	28,9823	4,7107	339,28	cooling	5,564	37,5541	12,9377	344,1		5,564	106,0764	50,5469	776,22	cooling	5,564	34,6519	13,2438	299,25	heating
5,566	28,7952	10,064	261,83	cooling	5,566	38,1913	10,2347	390,79		5,566	104,3442	48,0829	786,45	cooling	5,566	35,6077	12,9999	316,02	heating
5,568	27,5522	8,9187	260,47	cooling	5,568	38,4627	19,7904	261,01		5,568	108,5036	33,9598	1042,01	cooling	5,568	34,8793	14,0167	291,63	heating
5,57	28,4412	12,6409	220,86	cooling	5,57	36,9354	15,5787	298,53		5,57	101,1157	48,5948	734,16	cooling	5,57	34,8161	15,1222	275,29	heating
5,572	28,0166	11,9184	225,03	cooling	5,572	38,6667	18,1946	286,17		5,572	99,0028	49,1623	696,7	cooling	5,572	34,9198	13,7965	295,27	heating
5,574	27,8704	3,3528	342,72	cooling	5,574	38,0866	17,2852	290,77		5,574	100,0407	42,1342	809,45	cooling	5,574	35,3648	13,8497	300,75	heating
5,576	27,9132	0,4067	384,5	cooling	5,576	37,9795	14,4337	329,14		5,576	103,1111	48,1837	767,8	cooling	5,576	33,9983	12,2578	303,9	heating
5,578	27,7133	3,4312	339,43	cooling	5,578	37,7996	20,418	242,97		5,578	101,9789	46,3227	777,99	cooling	5,578	34,8854	12,3239	315,38	heating
5,58	27,0398	4,6911	312,4	cooling	5,58	38,001	25,6691	172,38		5,58	111,4074	46,2463	910,86	cooling	5,58	34,7239	15,7589	265,1	heating
5,582	27,5029	10,5319	237,23	cooling	5,582	36,9617	15,9176	294,17		5,582	110,1099	61,1969	683,73	cooling	5,582	33,9284	12,7507	296,03	heating
5,584	26,7982	6,9886	276,91	cooling	5,584	38,3546	20,4638	250,09		5,584	111,0558	63,1584	669,54	cooling	5,584	33,9902	13,8103	282,08	heating
5,586	27,1417	1,9364	352,33	cooling	5,586	36,4709	16,0208	285,86		5,586	109,5861	44,4031	911,16	cooling	5,586	34,4669	14,5775	278,02	heating
5,588	26,6323	4,1925	313,67	cooling	5,588	37,9122	14,8341	322,6		5,588	110,7764	43,825	935,88	cooling	5,588	34,2091	16,5479	246,88	heating
5,59	25,9387	2,7493	324,15	cooling	5,59	37,3975	22,8751	203		5,59	103,3285	45,3368	810,64	cooling	5,59	33,7216	14,5374	268,17	heating
5,592	25,8029	3,3511	313,84	cooling	5,592	37,0704	16,2415	291,16	heating	5,592	105,748	53,4973	730,39	cooling	5,592	34,8179	15,1651	274,72	heating
5,594	25,4933	3,7445	304,02	cooling	5,594	37,7968	15,4075	312,97	heating	5,594	101,5423	61,1431	564,72	cooling	5,594	35,0153	12,4868	314,92	heating
5,596	25,5563	-0,1305	359,06	cooling	5,596	37,7743	18,8728	264,22		5,596	100,8954	53,5602	661,68	cooling	5,596	34,8332	13,9001	292,61	heating
5,598	25,8104	0,5239	353,47	cooling	5,598	37,8907	14,2311	330,73	heating	5,598	99,4664	32,8517	931,17	cooling	5,598	34,5308	10,893	330,42	heating
5,6	25,3077	3,1025	310,4	cooling	5,6	38,7245	17,4527	297,35		5,6	105,2623	43,6278	861,56	cooling	5,6	36,2601	14,6484	302,1	heating
5,602	24,905	0,4701	341,56	cooling	5,602	37,6784	20,4265	241,16		5,602	102,5437	37,6209	907,52	cooling	5,602	35,3046	13,1508	309,68	heating
5,604	25,7616	4,7148	294,2	cooling	5,604	37,5482	11,5722	363,11	heating	5,604	101,6853	47,2937	760,31	cooling	5,604	35,1107	12,293	318,96	heating
5,606	24,766	4,5309	282,86	cooling	5,606	39,161	19,7796	270,92		5,606	101,3933	46,637	765,41	cooling	5,606	35,4481	13,4491	307,51	heating
5,608	24,6484	2,7438	306,19	cooling	5,608	37,6348	16,7535	291,89		5,608	100,6093	32,3275	954,48	cooling	5,608	35,2383	11,9311	325,8	heating
5,61	24,1965	4,4103	276,58	cooling	5,61	38,4189	14,8277	329,77		5,61	100,0327	26,3648	1029,77	cooling	5,61	34,7257	11,042	331,06	
5,612	23,696	3,7987	278,13	cooling	5,612	38,4741	18,7055	276,34		5,612	103,1411	49,3007	752,61	cooling	5,612	34,9044	15,7944	267,13	
5,614	23,4376	4,9932	257,83	cooling	5,614	38,4425	15,1135	326,1		5,614	103,9242	46,4775	803,02	cooling	5,614	34,656	16,9893	246,95	
5,616	23,5841	9,1171	202,23	cooling	5,616	38,3039	14,8628	327,67		5,616	100,1793	50,5766	693,37	cooling	5,616	34,1266	14,3106	277	heating
5,618	23,0757	7,6108	216,18		5,618	38,754	21,2403	244,81		5,618	99,0403	46,9795	727,73	cooling	5,618	34,7993	18,6342	225,96	heating
5,62	23,264	6,9762	227,68		5,62	37,5686	17,1034	286,07		5,62	99,7468	36,3256	886,54	cooling	5,62	34,537	13,2279	297,87	heating
5,622	22,3601	5,5275	235,29		5,622	38,715	20,0948	260,28		5,622	98,5874	36,0683	873,93	cooling	5,622	35,0698	13,6673	299,17	heating
5,624	22,7379	3,1207	274,22		5,624	37,5243	22,0271	216,63		5,624	105,6347	58,2869	661,85	cooling	5,624	34,3169	12,4835	305,2	heating
5,626	23,1282	2,8796	283,04		5,626	37,9858	14,352	330,37	heating	5,626	111,889	41,7077	981,03	cooling	5,626	35,263	12,6202	316,51	heating
5,628	23,4399	2,0174	299,46		5,628	37,9242	17,6442	283,48	heating	5,628	108,5929	50,2668	815,31	cooling	5,628	35,1589	11,4977	330,75	heating
5,63	23,9935	1,964	307,94		5,63	37,1545	15,3806	304,37	heating	5,63	106,2119	44,9821	855,9	cooling	5,63	34,9619	16,4374	258,94	

5,632	25,2883	4,6763	288,13		5,632	38,1126	16,6381	300,18	heating	5,632	107,9444	28,3607	1112,46	cooling	5,632	34,7946	14,6852	281,1	heating
5,634	25,3679	5,1908	282,05		5,634	38,5777	19,804	262,43		5,634	104,9581	27,357	1084,75	cooling	5,634	36,034	17,9368	252,97	heating
5,636	25,7733	2,4405	326,16		5,636	37,4211	15,8108	302,08	heating	5,636	109,6923	56,56	742,71	cooling	5,636	34,8279	15,1237	275,43	heating
5,638	26,3554	3,061	325,62		5,638	37,8476	12,978	347,64	heating	5,638	120,7752	46,0768	1044,17	cooling	5,638	34,684	10,0102	344,9	heating
5,64	26,488	2,9063	329,64		5,64	38,423	20,4722	250,93		5,64	115,9847	65,9714	699,11	cooling	5,64	35,4877	9,2052	367,39	heating
5,642	25,9003	3,6008	311,71		5,642	37,0329	16,1477	291,94		5,642	113,0248	55,523	803,79	cooling	5,642	35,1815	7,8614	381,9	
5,644	25,5055	1,967	329,03		5,644	37,7745	20,0878	247,23		5,644	116,459	36,4724	1118,09	cooling	5,644	35,006	8,7462	367,07	
5,646	26,0773	0,4567	358,14		5,646	36,8789	21,9296	208,97	heating	5,646	116,6122	43,1787	1026,49	cooling	5,646	35,4036	11,8389	329,4	
5,648	26,1452	0,8352	353,8		5,648	37,5245	15,5186	307,61	heating	5,648	114,4545	57,8601	791,1	cooling	5,648	35,3779	11,7166	330,75	
5,65	25,8202	4,7778	294,14		5,65	36,8838	19,0486	249,31	heating	5,65	126,9652	54,3628	1014,87	cooling	5,65	34,7401	6,7717	390,96	
5,652	25,8493	7,4001	257,89		5,652	37,3764	19,4002	251,28	heating	5,652	122,5471	79,0704	607,74	cooling	5,652	35,7287	11,9425	332,5	
5,654	26,4667	7,8918	259,65		5,654	36,6045	13,4051	324,29	heating	5,654	119,9506	72,2212	667,19	cooling	5,654	35,3428	9,8273	356,67	
5,656	26,1495	6,9903	267,82		5,656	38,1702	16,4455	303,68	heating	5,656	118,7626	50,9998	947,22	cooling	5,656	35,6927	12,1256	329,43	
5,658	26,7086	6,4512	283,17		5,658	36,7468	15,0638	303,1	heating	5,658	118,5791	59,1673	830,49	cooling	5,658	34,8307	11,2757	329,26	cooling
5,66	26,7206	5,364	298,53		5,66	38,4538	13,0515	355,09	heating	5,66	108,1487	49,8685	814,67	cooling	5,66	35,886	11,2339	344,6	cooling
5,662	26,9702	5,7144	297,12		5,662	37,6293	19,0831	259,25	heating	5,662	115,6154	45,9006	974,51	cooling	5,662	35,3801	8,596	374,4	cooling
5,664	26,4648	6,0614	285,21		5,664	37,1884	15,919	297,31	heating	5,664	113,0422	64,0349	685,05	cooling	5,664	35,3592	12,0589	325,7	cooling
5,666	26,9711	4,6595	311,88		5,666	38,43	18,0866	284,37	heating	5,666	113,0515	57,3461	778,68	cooling	5,666	34,8269	9,6352	352,14	cooling
5,668	26,9112	5,6883	296,67		5,668	38,2254	19,4166	262,92	heating	5,668	112,0475	37,8023	1037,84	cooling	5,668	36,0182	10,6395	354,76	cooling
5,67	26,5249	4,4235	308,94		5,67	38,1878	16,1114	308,6	heating	5,67	116,0073	52,8042	883,48	cooling	5,67	34,3878	9,4881	348,06	cooling
5,672	26,9659	4,0503	320,33		5,672	38,0219	17,0129	293,67	heating	5,672	105,4349	32,7403	1016,16	cooling	5,672	34,5468	7,4331	379,01	cooling
5,674	27,6956	4,9136	318,46		5,674	39,4095	20,9265	258,36	heating	5,674	107,5003	34,2344	1024,15	cooling	5,674	34,8538	10,7167	337,4	cooling
5,676	27,938	4,8967	322,08		5,676	38,0095	15,9887	307,82	heating	5,676	104,5121	43,7004	850,06	cooling	5,676	34,6447	11,6386	321,59	cooling
5,678	28,0643	3,3295	345,75		5,678	39,8729	20,2852	273,81	heating	5,678	104,2474	37,3369	935,31	cooling	5,678	34,1767	13,1638	293,73	cooling
5,68	29,3284	5,4706	333,5		5,68	38,1684	16,8864	297,49	heating	5,68	101,983	21,8023	1120,81	cooling	5,68	34,6616	12,4509	310,47	cooling
5,682	29,2655	3,724	357,03		5,682	39,6791	14,4816	352,22	heating	5,682	108,048	38,5143	971,98	cooling	5,682	34,5982	11,7799	318,97	cooling
5,684	29,6691	5,5168	337,61		5,684	38,4116	17,1164	297,68	heating	5,684	103,8727	22,9267	1131,5	cooling	5,684	33,9744	7,5748	369,03	cooling
5,686	29,3695	7,4748	306,06		5,686	38,8958	17,6677	296,74		5,686	102,3588	38,8992	887,07	cooling	5,686	35,0655	12,8427	310,64	cooling
5,688	29,7247	6,4643	325,15		5,688	38,1627	17,0083	295,71		5,688	100,9038	50,2501	708,06	cooling	5,688	34,7538	12,4267	312,1	cooling
5,69	29,4405	4,2525	352,09		5,69	38,4309	18,6006	277,2		5,69	101,093	43,5551	804,29	cooling	5,69	34,7652	14,5804	282,15	cooling
5,692	29,4138	4,5086	348,14	cooling	5,692	38,4592	17,6218	291,28	heating	5,692	99,9359	35,0833	906,54	cooling	5,692	33,8344	11,3232	314,67	cooling
5,694	29,1739	1,2665	390,1	cooling	5,694	38,9477	18,5216	285,53	heating	5,694	103,7257	45,9581	807,51	cooling	5,694	35,1555	13,0243	309,36	cooling
5,696	29,6263	0,1318	412,29	cooling	5,696	39,1074	22,2253	235,99	heating	5,696	106,511	38,408	951,98	cooling	5,696	34,697	11,9848	317,48	cooling
5,698	30,2023	3,9273	367,29	cooling	5,698	38,4454	18,1573	283,6	heating	5,698	105,1697	58,2443	655,95	cooling	5,698	34,5962	13,2899	297,83	cooling
5,7	30,2785	5,1205	351,67	cooling	5,7	40,0287	21,1946	263,27	heating	5,7	103,681	56,6742	657,08	cooling	5,7	34,4527	13,2878	295,85	cooling
5,702	31,1108	7,5632	329,16	cooling	5,702	38,7977	16,3772	313,4	heating	5,702	103,1976	38,1187	909,71	cooling	5,702	35,1962	13,3714	305,08	cooling
5,704	30,7402	5,9539	346,48	cooling	5,704	39,9549	19,667	283,59	heating	5,704	102,5671	28,2436	1038,93	cooling	5,704	34,0569	10,986	322,5	cooling
5,706	31,462	5,0282	369,5	cooling	5,706	38,6062	19,4493	267,79	heating	5,706	102,3142	29,7514	1014,32	cooling	5,706	34,2611	9,6785	343,63	cooling
5,708	31,1505	3,9384	380,38	cooling	5,708	38,9375	19,9311	265,68	heating	5,708	109,6221	27,5087	1147,82	cooling	5,708	34,4209	8,4375	363,21	cooling
5,71	31,2901	8,6185	316,92	cooling	5,71	37,323	15,5244	304,71	heating	5,71	106,6993	49,733	796,3	cooling	5,71	34,4767	7,0789	382,98	cooling
5,712	29,6558	5,9515	331,35	cooling	5,712	37,9722	17,0789	292,06	heating	5,712	106,2581	48,3808	809,04	cooling	5,712	33,9828	11,1647	318,96	cooling
5,714	29,5897	5,7603	333,1	cooling	5,714	36,984	15,5451	299,68	heating	5,714	106,089	33,272	1017,87	cooling	5,714	34,1877	10,4105	332,37	cooling
5,716	28,7472	3,5987	351,54	cooling	5,716	37,6694	20,1107	245,44	heating	5,716	108,3143	39,8416	957,15	cooling	5,716	34,095	10,2812	332,88	cooling
5,718	29,0207	3,5641	355,84	cooling	5,718	37,0242	18,8639	253,85	heating	5,718	100,9206	30,0854	990,17	cooling	5,718	33,4301	6,5685	375,48	cooling
5,72	29,3082	4,4935	346,87	cooling	5,72	37,909	14,5253	326,87	heating	5,72	107,3285	38,7356	958,83	cooling	5,72	34,2787	7,7659	370,61	cooling
5,722	29,8172	7,3472	314,1	cooling	5,722	37,506	14,1655	326,27	heating	5,722	103,7938	55,1542	679,91	cooling	5,722	34,5303	9,3199	352,4	cooling
5,724	29,3816	4,8107	343,46	cooling	5,724	37,266	11,9271	354,2	heating	5,724	104,8989	53,1235	723,74	cooling	5,724	34,1933	13,5229	288,94	cooling
5,726	29,3791	3,5211	361,46	cooling	5,726	38,6879	16,6949	307,43	heating	5,726	103,9142	41,9604	866,02	cooling	5,726	33,5644	11,3145	311,02	cooling
5,728	29,1081	1,7792	382,02	cooling	5,728	38,0509	15,927	309,26		5,728	107,4425	46,0219	858,57	cooling	5,728	35,04	13,4263	302,13	cooling
5,73	28,9083	-0,4607	410,54	cooling	5,73	37,9512	14,0224	334,49		5,73	100,2946	25,3831	1047,15	cooling	5,73	33,9937	11,6667	312,1	cooling
5,732	29,7415	6,134	330	cooling	5,732	37,5898	10,2807	381,74		5,732	104,1599	36,6645	943,49	cooling	5,732	34,3494	11,2767	322,52	cooling
5,734	29,1371	7,1234	307,72	cooling	5,734	39,2929	13,9755	353,9		5,734	101,1541	45,7928	773,87	cooling	5,734	34,2617	12,473	304,57	cooling
5,736	29,3958	6,345	322,22	cooling	5,736	38,1156	11,6746	369,61		5,736	100,9181	41,0122	837,4	cooling	5,736	34,7768	10,6491	337,27	cooling
5,738	29,3375	6,0921	324,94	cooling	5,738	39,4737	21,2901	254,18		5,738	98,3313	31,8058	929,93	cooling	5,738	33,8151	10,0743	331,86	cooling
5,74	29,7376	5,3261	341,24	cooling	5,74	38,458	20,74	247,67		5,74	105,7897	47,8837	809,44	cooling	5,74	34,7159	11,4523	325,19	cooling
5,742	30,3493	4,2986	364,15	cooling	5,742	39,5138	19,9648	273,27		5,742	108,9237	29,0977	1115,85	cooling	5,742	34,4538	12,759	303,26	cooling

5,744	31,1048	11,72	270,97	cooling	5,744	38,0063	16,8797	295,32		5,744	105,0735	49,7551	773,27	cooling	5,744	34,3261	10,8096	328,72	cooling
5,746	30,0385	7,5349	314,57	cooling	5,746	38,5179	16,1382	312,84		5,746	104,5217	54,0367	705,7	cooling	5,746	33,9394	12,9618	293,24	cooling
5,748	30,4868	9,141	298,38	cooling	5,748	38,1152	16,3766	303,87		5,748	104,8402	48,1663	792,22	cooling	5,748	34,3973	8,7423	358,62	cooling
5,75	29,6362	7,3089	312,1	cooling	5,75	37,1516	16,3567	290,68		5,75	104,3692	47,3809	796,61	cooling	5,75	34,6826	11,2727	327,24	cooling
5,752	29,0663	4,0507	349,68	cooling	5,752	36,6685	14,9349	303,8		5,752	106,7689	56,4741	703,05	cooling	5,752	33,5958	9,3504	338,91	cooling
5,754	29,474	6,5593	320,31	cooling	5,754	37,2405	13,5087	331,73		5,754	116,0649	39,5845	1069,08	cooling	5,754	34,5597	11,5793	321,23	cooling
5,756	29,1687	6,556	316,09	cooling	5,756	37,1022	17,1051	279,53		5,756	109,6035	52,16	802,97	cooling	5,756	34,257	10,0931	337,78	cooling
5,758	30,0328	3,378	372,6	cooling	5,758	36,3124	15,2799	294		5,758	110,1436	55,6984	761,06	cooling	5,758	34,0215	11,1454	319,77	cooling
5,76	31,2714	8,8103	313,97	cooling	5,76	37,7883	19,6527	253,51		5,76	107,7804	42,7243	909,39	cooling	5,76	33,4761	8,4699	349,55	cooling
5,762	31,4695	9,1546	311,93	cooling	5,762	37,1831	13,0811	336,91		5,762	108,3205	46,4374	865,03	cooling	5,762	35,07	12,78	311,58	cooling
5,764	32,319	6,6241	359,18	cooling	5,764	37,5677	14,0377	328,91		5,764	101,0818	38,2037	878,94	cooling	5,764	33,7438	10,6592	322,69	cooling
5,766	33,3269	12,1869	295,51	cooling	5,766	36,7985	11,7521	350,11		5,766	110,8345	32,4009	1096,39	cooling	5,766	34,4058	12,6116	304,65	cooling
5,768	32,2529	5,7448	370,54	cooling	5,768	38,2552	17,0617	296,25		5,768	105,0768	37,5015	944,6	cooling	5,768	34,0937	13,6508	285,76	cooling
5,77	33,2118	4,9829	394,6	cooling	5,77	37,5012	14,2175	325,47		5,77	106,025	37,0352	964,37	cooling	5,77	34,6903	11,8114	319,81	cooling
5,772	32,8393	7,0779	360,11	cooling	5,772	37,5493	15,3957	309,67		5,772	105,057	31,4418	1029,03	cooling	5,772	33,7644	12,6212	295,55	cooling
5,774	33,4941	8,2846	352,39	cooling	5,774	37,3526	11,0096	368,24		5,774	108,6075	47,1572	858,98	cooling	5,774	34,0754	13,0078	294,49	cooling
5,776	34,0028	10,2876	331,5	cooling	5,776	37,8781	11,0222	375,4		5,776	100,4248	31,5299	963,05	cooling	5,776	34,1051	13,5582	287,22	cooling
5,778	34,303	11,4013	320,13	cooling	5,778	37,7182	13,5761	337,47		5,778	105,8321	41,8628	894,2	cooling	5,778	33,766	10,9321	319,18	cooling
5,78	34,4465	1,3755	462,28	cooling	5,78	37,9794	15,4968	314,27	cooling	5,78	103,4184	48,6799	765,16	cooling	5,78	34,0441	12,7167	298,13	cooling
5,782	35,7966	8,6562	379,38	cooling	5,782	38,0951	18,1462	278,86	cooling	5,782	104,0193	45,8347	813,33	cooling	5,782	34,2253	9,4415	346,44	cooling
5,784	35,1231	11,1396	335,25	cooling	5,784	37,5218	14,7788	317,91		5,784	105,0578	45,0148	839,31	cooling	5,784	34,5825	14,6237	278,99	cooling
5,786	35,6446	12,4203	324,64	cooling	5,786	38,0848	17,4748	288,1		5,786	109,8064	56,6284	743,35	cooling	5,786	33,3648	10,9934	312,72	cooling
5,788	35,5411	13,1953	312,36	cooling	5,788	38,0796	16,5358	301,15		5,788	107,3709	42,5862	905,59	cooling	5,788	34,9048	12,6	311,79	cooling
5,79	35,075	7,7689	381,7	cooling	5,79	37,642	18,7885	263,54		5,79	110,0309	52,0038	811,13	cooling	5,79	34,0367	8,8952	351,44	cooling
5,792	35,2543	2,505	457,79	cooling	5,792	37,5719	15,5109	308,38		5,792	110,7088	55,0535	777,98	cooling	5,792	34,0239	9,7904	338,75	cooling
5,794	34,6421	8,1326	370,56	cooling	5,794	37,8649	16,5439	298,04		5,794	111,0963	51,8754	827,82	cooling	5,794	33,551	9,6068	334,71	cooling
5,796	33,7709	9,7663	335,55	cooling	5,796	37,6389	11,0226	372,06		5,796	110,518	50,532	838,51	cooling	5,796	34,9848	14,3855	287,95	cooling
5,798	33,5219	8,3371	352,05	cooling	5,798	37,3661	15,6332	303,79		5,798	112,9866	50,206	877,58	cooling	5,798	33,8206	14,383	271,71	cooling
5,8	34,3745	10,4978	333,76	cooling	5,8	37,0939	14,5022	315,8		5,8	114,3269	36,6552	1085,73	cooling	5,8	34,5841	15,9498	260,48	cooling
5,802	33,9172	5,8179	392,79	cooling	5,802	37,466	14,1213	326,32		5,802	113,0282	45,6789	941,44	cooling	5,802	34,2111	14,6198	273,86	cooling
5,804	34,9959	11,1864	332,82	cooling	5,804	36,7485	12,8978	333,4	cooling	5,804	110,8857	44,0464	934,31	cooling	5,804	34,7684	11,5267	324,88	cooling
5,806	34,4468	13,464	293,31	cooling	5,806	36,6947	13,0137	331,02	cooling	5,806	109,912	43,5404	927,78	cooling	5,806	33,7473	13,0342	289,54	cooling
5,808	35,2657	14,7593	286,65	cooling	5,808	36,3523	15,5033	291,44	cooling	5,808	109,0056	43,2226	919,55	cooling	5,808	33,6983	12,5965	294,97	cooling
5,81	34,9174	11,2494	330,84	cooling	5,81	36,5868	18,4253	253,87	cooling	5,81	106,9053	36,6316	982,32	cooling	5,81	33,8031	13,6751	281,36	cooling
5,812	35,4083	12,084	326,04	cooling	5,812	35,9053	16,7255	268,1		5,812	110,7931	42,7046	951,78	cooling	5,812	33,5337	12,0469	300,35	cooling
5,814	35,5206	5,9525	413,32	cooling	5,814	37,5967	15,7379	305,55		5,814	109,7629	52,0049	807,37	cooling	5,814	33,2814	13,4196	277,64	cooling
5,816	35,9174	13,6344	311,48	cooling	5,816	36,2075	16,6987	272,7		5,816	110,1511	47,6051	874,3	cooling	5,816	33,8737	11,3683	314,59	
5,818	35,1117	13,0737	308,06	cooling	5,818	36,4713	14,0423	313,52		5,818	109,8234	54,7532	769,8	cooling	5,818	34,2823	14,3356	278,83	cooling
5,82	34,9974	7,641	382,4	cooling	5,82	36,7054	20,5212	226,23		5,82	111,2593	60,979	702,84	cooling	5,82	33,6223	10,8534	318,28	
5,822	35,3847	2,3613	461,62	cooling	5,822	37,0588	18,3077	262,11		5,822	108,5307	52,5587	782,41	cooling	5,822	34,8915	14,5615	284,18	
5,824	34,813	-0,4919	493,51	cooling	5,824	36,4069	15,039	298,69		5,824	108,1879	64,5782	609,6	cooling	5,824	34,2472	12,6885	301,36	
5,826	35,5962	2,8748	457,4	cooling	5,826	37,5744	14,9702	315,97		5,826	106,1482	64,9176	576,34	cooling	5,826	34,3262	13,8142	286,73	
5,828	35,3055	11,2654	336,04	cooling	5,828	37,8603	14,4917	326,66		5,828	106,5068	60,3241	645,57	cooling	5,828	33,3509	12,3704	293,28	
5,83	34,7448	11,5285	324,53	cooling	5,83	37,7837	13,3608	341,4		5,83	104,2876	54,3677	697,81	cooling	5,83	34,2652	14,1153	281,66	
5,832	34,3053	4,7554	413,06	cooling	5,832	38,303	20,7193	245,79		5,832	107,6427	51,9962	777,86	cooling	5,832	32,9911	11,7078	297,51	
5,834	35,4875	7,9541	384,88	cooling	5,834	37,9393	17,735	282,43		5,834	109,5368	33,3256	1065,32	cooling	5,834	33,3745	13,2112	281,85	
5,836	34,579	2,8953	442,89	cooling	5,836	39,5406	17,7381	304,77		5,836	106,5919	30,2786	1066,75	cooling	5,836	33,118	13,9409	268,07	
5,838	34,2559	9,6819	343,51	cooling	5,838	38,3747	18,3378	280,09		5,838	107,5111	32,938	1042,42	cooling	5,838	34,1165	12,704	299,31	
5,84	33,3404	9,8503	328,36	cooling	5,84	38,9452	13,3966	357,13		5,84	107,899	35,4866	1012,22	cooling	5,84	33,7411	13,9827	276,19	
5,842	33,9162	8,586	354,08	cooling	5,842	38,1772	15,9092	311,27		5,842	108,1062	33,1376	1047,95	cooling	5,842	34,3921	12,9341	299,95	
5,844	34,4023	6,031	396,59	cooling	5,844	38,028	19,5856	257,8		5,844	111,713	46,8835	906,22	cooling	5,844	34,6028	16,4724	253,44	
5,846	34,4746	9,6876	346,49	cooling	5,846	36,6056	17,0729	273,04		5,846	119,3985	38,8388	1126,11	cooling	5,846	34,0199	14,9658	266,35	
5,848	34,9005	5,6598	408,74	cooling	5,848	38,4476	21,1479	241,83		5,848	116,9205	40,1952	1072,51	cooling	5,848	34,23	17,3083	236,54	
5,85	34,923	10,8055	337,13	cooling	5,85	36,6692	22,0438	204,44		5,85	117,1966	48,5743	959,24	cooling	5,85	34,2339	14,2891	278,8	
5,852	33,7761	7,4755	367,64	cooling	5,852	37,6417	17,3154	284,13		5,852	115,0353	42,4658	1014,41	cooling	5,852	33,9126	15,1153	262,76	
5,854	33,5346	3,1773	424,35	cooling	5,854	38,1884	24,5554	190,57		5,854	115,2695	45,5282	974,88	cooling	5,854	32,867	9,7653	322,93	

5,856	33,8641	5,0931	402,17	cooling	5,856	38,2822	21,6906	231,93	heating	5,856	114,1701	54,8982	828,53	cooling	5,856	34,0202	12,0701	306,83	
5,858	32,4923	5,6496	375,22	cooling	5,858	38,145	19,1963	264,87	heating	5,858	117,3518	37,0953	1121,87	cooling	5,858	33,4126	9,6851	331,67	
5,86	32,1968	8,5993	329,86	cooling	5,86	38,6884	21,5382	239,74	heating	5,86	115,9095	46,3655	972,12	cooling	5,86	33,7943	12,4179	298,81	
5,862	31,9359	9,4548	314,25	cooling	5,862	38,6222	18,8262	276,72	heating	5,862	115,4795	59,4905	782,64	cooling	5,862	33,441	13,1387	283,8	
5,864	31,9529	5,8633	364,69	cooling	5,864	38,1207	16,7541	298,67	heating	5,864	115,2798	55,7407	832,27	cooling	5,864	34,5646	14,1336	285,6	
5,866	32,7623	4,112	400,49	cooling	5,866	37,9824	20,579	243,27	heating	5,866	115,2849	67,6774	665,48	cooling	5,866	33,6703	12,1625	300,65	
5,868	33,4658	10,5846	319,85	cooling	5,868	36,6672	13,3164	326,41	heating	5,868	114,5779	75,8686	541,1	cooling	5,868	34,1599	9,9615	338,26	
5,87	34,2893	10,4461	333,29	cooling	5,87	37,971	14,3219	330,58	heating	5,87	120,3168	45,0166	1052,59	cooling	5,87	34,1053	10,1605	334,71	
5,872	33,5555	13,1011	285,92	cooling	5,872	36,5911	15,4532	295,48	heating	5,872	114,1231	46,8235	940,75	cooling	5,872	34,397	11,1647	324,75	
5,874	33,2785	7,7056	357,47	cooling	5,874	38,1068	14,3426	332,19	heating	5,874	113,0591	51,4296	861,49	cooling	5,874	33,8235	16,8797	236,85	
5,876	32,8157	2,6551	421,6	cooling	5,876	37,1395	18,9944	253,64	heating	5,876	112,2552	47,9365	899,08	cooling	5,876	34,0392	16,5356	244,67	
5,878	33,4279	4,6365	402,46	cooling	5,878	37,062	18,7718	255,67	heating	5,878	112,3975	57,6644	765,09	cooling	5,878	34,3837	19,0472	214,38	
5,88	32,0103	8,4334	329,57	cooling	5,88	36,7983	16,5804	282,62	heating	5,88	112,5032	61,4738	713,32	cooling	5,88	33,8721	14,6801	268,28	
5,882	32,471	10,9464	300,88	cooling	5,882	37,8627	17,1683	289,28	heating	5,882	128,7119	56,1958	1013,67	cooling	5,882	34,3724	15,4495	264,51	
5,884	32,4358	11,9067	286,97	cooling	5,884	36,6353	14,7501	305,92	heating	5,884	119,2225	61,6865	804,27	cooling	5,884	34,4245	12,9307	300,45	heating
5,886	33,1283	6,8567	367,24	cooling	5,886	37,2384	14,6651	315,54	heating	5,886	121,0998	63,2027	809,32	cooling	5,886	34,4786	17,0029	244,28	heating
5,888	34,041	7,7666	367,28	cooling	5,888	38,0324	19,9308	253,03	heating	5,888	120,9044	50,3595	986,11	cooling	5,888	33,3919	14,8906	258,62	heating
5,89	34,9633	12,4884	314,17	cooling	5,89	37,6238	15,8686	304,11	heating	5,89	120,2594	50,3469	977,27	cooling	5,89	34,5241	19,0079	216,89	heating
5,892	35,7502	12,4873	325,18	cooling	5,892	38,2254	18,7216	272,63	heating	5,892	117,0698	42,3704	1044,19	cooling	5,892	33,1791	16,402	234,52	heating
5,894	35,6935	12,9246	318,27	cooling	5,894	37,6294	16,5926	294,06	heating	5,894	130,9199	41,3253	1252,4	cooling	5,894	33,4437	15,2897	253,77	heating
5,896	35,7551	10,4464	353,78	cooling	5,896	39,1956	19,2107	279,36	heating	5,896	121,117	56,5447	902,63	cooling	5,896	32,8316	13,4746	270,58	heating
5,898	35,067	4,6739	424,85	cooling	5,898	38,0561	19,0906	265,11	heating	5,898	120,5924	56,7541	892,36	cooling	5,898	34,0698	14,2475	277,09	heating
5,9	35,6118	5,6532	418,78	cooling	5,9	39,1364	21,4571	247,13	heating	5,9	119,892	39,9979	1116,8	cooling	5,9	33,1865	12,8678	284,02	heating
5,902	33,9019	6,3863	384,63	cooling	5,902	38,5834	20,2525	256,24	heating	5,902	120,8555	42,6787	1092,8	cooling	5,902	34,0038	12,823	296,08	heating
5,904	34,3819	6,702	386,92	cooling	5,904	38,879	20,1647	261,6	heating	5,904	109,4531	20,1568	1248,23	cooling	5,904	33,6911	13,3611	284,18	heating
5,906	33,6667	5,1698	398,34	cooling	5,906	37,9408	16,6667	297,38	heating	5,906	119,0803	34,0668	1188,36	cooling	5,906	34,2206	12,6752	301,17	heating
5,908	34,0325	-0,3944	481,24	cooling	5,908	39,44	21,4502	251,47	heating	5,908	114,4404	61,0934	745,71	cooling	5,908	33,5799	15,5317	252,29	heating
5,91	34,113	0,3376	472,13	cooling	5,91	38,254	19,5481	261,48	heating	5,91	115,2954	63,9685	717,47	cooling	5,91	33,9018	12,9225	293,26	heating
5,912	34,9887	8,5919	368,99	cooling	5,912	38,1695	14,5255	330,51	heating	5,912	115,4673	51,1967	898,41	cooling	5,912	34,228	14,9485	269,5	heating
5,914	34,4161	10,631	332,48	cooling	5,914	38,857	16,6132	310,94	heating	5,914	122,3997	71,9067	705,82	cooling	5,914	33,6532	12,9294	289,69	heating
5,916	34,6749	8,668	363,54	cooling	5,916	38,5816	12,7771	360,71	heating	5,916	111,4431	40,7401	988,32	cooling	5,916	33,9631	15,6655	255,77	heating
5,918	34,3744	6,4012	391,02	cooling	5,918	38,5304	15,761	318,28	heating	5,918	117,7408	46,7306	992,62	cooling	5,918	33,8311	13,6006	282,79	heating
5,92	33,8417	4,1019	415,72	cooling	5,92	37,8992	17,8897	279,7	heating	5,92	114,6022	58,4241	785,29	cooling	5,92	34,0701	14,9507	267,26	heating
5,922	33,31	9,2977	335,66	cooling	5,922	38,7567	17,9571	290,75	heating	5,922	114,5686	46,7456	948,06	cooling	5,922	33,193	10,5441	316,6	heating
5,924	32,2458	10,403	305,33	cooling	5,924	38,4981	15,7284	318,29	heating	5,924	113,9313	30,1271	1171,46	cooling	5,924	34,7338	15,0242	275,51	heating
5,926	32,9804	8,2541	345,64	cooling	5,926	37,9046	18,6425	269,26	heating	5,926	120,7813	52,6836	951,9	cooling	5,926	33,2621	12,0844	296,03	heating
5,928	33,105	6,4917	372,02	cooling	5,928	37,3478	12,4375	348,21	heating	5,928	117,2947	37,5498	1114,72	cooling	5,928	33,9183	12,7603	295,76	heating
5,93	34,085	7,4992	371,63	cooling	5,93	38,8818	18,261	288,25	heating	5,93	114,752	54,2004	846,42	cooling	5,93	33,2861	13,6118	275,02	heating
5,932	34,5767	8,5095	364,38	cooling	5,932	37,5642	17,2565	283,87	heating	5,932	113,2187	51,6922	860,05	cooling	5,932	34,429	15,2187	268,53	heating
5,934	36,0454	12,3788	330,82	cooling	5,934	38,5351	18,7064	277,18	heating	5,934	112,3361	31,7958	1125,83	cooling	5,934	33,3104	15,9873	242,15	heating
5,936	35,1244	8,3606	374,12	cooling	5,936	38,9206	19,6664	269,15	heating	5,936	113,6576	26,6948	1215,61	cooling	5,936	34,0497	15,1615	264,03	heating
5,938	36,3622	8,5105	389,32	cooling	5,938	39,1527	16,8018	312,43	heating	5,938	113,331	32,0994	1135,5	cooling	5,938	33,5619	9,9315	330,32	heating
5,94	35,8695	4,1441	443,47	cooling	5,94	39,0923	17,4235	302,9	heating	5,94	118,455	36,7816	1141,67	cooling	5,94	34,1526	7,8574	367,57	heating
5,942	36,8997	3,2113	470,91	cooling	5,942	39,5639	20,2137	270,49	heating	5,942	113,4594	58,1076	773,74	cooling	5,942	34,0344	12,0479	307,34	heating
5,944	36,8695	11,0156	361,4	cooling	5,944	38,9564	16,2146	317,9	heating	5,944	112,7095	60,9769	723,15	cooling	5,944	34,3215	11,229	322,8	heating
5,946	36,7427	14,9514	304,61	cooling	5,946	39,3663	17,6437	303,65	heating	5,946	110,2582	49,6823	846,76	cooling	5,946	34,6282	16,0289	259,99	heating
5,948	36,4199	11,8561	343,37	cooling	5,948	38,6932	19,5249	267,95		5,948	112,8768	55,0325	808,58	cooling	5,948	33,3752	12,8165	287,38	heating
5,95	36,6287	8,1253	398,44	cooling	5,95	39,1983	11,9012	381,57	heating	5,95	106,5303	38,9222	945,06	cooling	5,95	34,5093	13,1643	298,37	heating
5,952	37,0764	3,3568	471,35	cooling	5,952	39,1645	16,8145	312,42		5,952	110,9476	47,1626	891,62	cooling	5,952	33,9626	10,6784	325,48	heating
5,954	37,9587	6,5559	438,96	cooling	5,954	38,5169	17,2097	297,84		5,954	108,3189	57,8208	705,89	cooling	5,954	34,1515	14,1844	279,11	heating
5,956	38,006	15,4801	314,88	cooling	5,956	39,5859	21,4432	253,61		5,956	108,6571	48,2208	844,81	cooling	5,956	33,1756	13,0391	281,48	heating
5,958	36,7578	13,8289	320,51	cooling	5,958	39,9707	23,0702	236,24		5,958	109,8371	38,7703	993,41	cooling	5,958	34,8486	18,5974	227,17	heating
5,96	37,5888	15,4109	310,01	cooling	5,96	39,4992	20,4428	266,38	heating	5,96	115,0194	51,153	892,76	cooling	5,96	33,7223	14,9238	262,77	heating
5,962	35,3901	9,517	361,67	cooling	5,962	40,2274	16,7023	328,85	heating	5,962	119,144	36,6357	1153,34	cooling	5,962	34,5506	13,9786	287,57	heating
5,964	34,8856	6,7601	393,15	cooling	5,964	41,2566	21,2036	280,31		5,964	117,4375	55,1744	870,35	cooling	5,964	33,9121	11,6722	310,88	heating
5,966	33,5819	7,4517	365,26	cooling	5,966	40,58	20,7521	277,16		5,966	116,9077	57,0461	836,78	cooling	5,966	34,6149	11,8922	317,63	heating

5,968	33,5927	7,1754	369,27	cooling	5,968	41,8352	24,6341	240,45	heating	5,968	115,9178	43,5427	1011,7	cooling	5,968	33,6606	13,4845	282,03	heating
5,97	32,7713	3,2752	412,31	cooling	5,97	40,8121	24,1386	233,07	heating	5,97	118,5106	48,6328	976,79	cooling	5,97	33,965	12,8988	294,47	heating
5,972	34,0035	8,3009	359,28	cooling	5,972	41,0334	18,2842	318	heating	5,972	114,3295	42,6977	1001,31	cooling	5,972	34,2532	12,5332	303,61	heating
5,974	33,3444	5,0871	395	cooling	5,974	39,9695	18,3944	301,59	heating	5,974	121,5145	43,761	1086,88	cooling	5,974	34,1217	9,5628	343,3	heating
5,976	34,1136	7,2108	376,06	cooling	5,976	40,2744	15,9798	339,6	heating	5,976	116,782	59,331	803,08	cooling	5,976	34,1539	12,3715	304,49	heating
5,978	33,0592	8,8561	338,32	cooling	5,978	39,5565	13,0257	370,86	heating	5,978	115,8507	54,4874	857,77	cooling	5,978	34,2949	11,2063	322,75	heating
5,98	33,491	8,5419	348,75	cooling	5,98	40,1295	15,1924	348,58		5,98	114,1091	35,1423	1103,84	cooling	5,98	34,9386	13,808	295,37	
5,982	33,1359	9,0937	336,07	cooling	5,982	39,3214	14,4905	347,1		5,982	121,2874	49,9528	997,15	cooling	5,982	34,1728	9,7573	341,29	
5,984	32,5457	7,4554	350,72	cooling	5,984	40,3719	14,1442	366,62		5,984	119,047	41,2265	1087,81	cooling	5,984	35,3706	15,6008	276,35	
5,986	32,7752	7,1553	358,13	cooling	5,986	39,7271	20,3753	270,51		5,986	119,412	60,2433	827,09	cooling	5,986	34,6213	14,6945	278,55	
5,988	32,6222	9,0267	329,83	cooling	5,988	39,1538	16,9677	310,13		5,988	119,9379	73,6987	646,36	cooling	5,988	34,6387	15,1411	272,55	
5,99	32,4146	8,8472	329,44	cooling	5,99	40,398	20,7188	275,09		5,99	120,8002	63,0255	807,61	cooling	5,99	33,6703	9,5941	336,55	
5,992	33,2926	8,4846	346,78	cooling	5,992	39,4884	20,1081	270,91		5,992	120,9367	45,8949	1048,97	cooling	5,992	34,8846	9,8567	349,85	
5,994	34,9632	8,583	368,76	cooling	5,994	39,3004	17,5642	303,84		5,994	126,5807	54,6395	1005,63	cooling	5,994	33,9571	9,8764	336,61	
5,996	35,0466	3,6788	438,48	cooling	5,996	39,1577	18,8767	283,5		5,996	129,2564	37,5092	1282,49	cooling	5,996	34,2974	14,6707	274,35	
5,998	36,2316	10,0152	366,47	cooling	5,998	39,7958	22,148	246,69		5,998	123,8274	51,136	1016,12	cooling	5,998	33,7802	15,9044	249,88	
6	35,2112	11,5116	331,29	cooling	6	39,0945	19,2668	277,16		6	122,2375	54,1498	951,77	cooling	6	34,6306	15,3432	269,61	
6,002	35,8096	10,7914	349,72	cooling	6,002	40,6039	23,1821	243,53		6,002	120,7602	34,4739	1206,16	cooling	6,002	33,9692	14,7078	269,25	
6,004	35,1442	9,7911	354,4	cooling	6,004	39,4392	16,893	315,16		6,004	120,5757	35,9711	1182,65	cooling	6,004	34,6493	10,9443	331,36	
6,006	35,5793	3,7647	444,72	cooling	6,006	41,2298	16,7499	342,19		6,006	120,1688	53,3132	934,54	cooling	6,006	34,7342	13,6321	294,98	
6,008	35,3559	3,2139	449,3	cooling	6,008	40,7422	21,937	262,87		6,008	129,1331	43,3826	1198,66	cooling	6,008	34,6	11,7924	318,82	
6,01	34,9979	10,0791	348,33	cooling	6,01	41,6222	21,6259	279,52		6,01	125,543	69,245	786,96	cooling	6,01	34,1229	13,2257	292,11	
6,012	33,755	11,2078	315,18	cooling	6,012	41,4427	21,0682	284,81		6,012	125,1071	80,658	621,33	cooling	6,012	34,4609	11,4803	321,23	
6,014	34,9668	11,7991	323,85	cooling	6,014	41,4705	19,5439	306,5		6,014	123,7325	53,9443	975,53	cooling	6,014	34,6326	11,3145	325,95	
6,016	34,2919	11,5057	318,52	cooling	6,016	41,315	15,6402	358,9		6,016	130,9298	63,1088	948,04	cooling	6,016	34,2809	9,2835	349,43	
6,018	34,5477	1,6868	459,35	cooling	6,018	41,9737	18,7015	325,31		6,018	122,3732	45,5997	1073,18	cooling	6,018	34,8883	13,5897	297,72	
6,02	35,684	5,5267	421,55	cooling	6,02	41,0832	20,8256	283,17		6,02	122,64	47,233	1054,08	cooling	6,02	34,7438	10,6585	336,68	
6,022	35,2526	6,7599	398,29	cooling	6,022	40,7381	16,1365	343,89		6,022	122,5503	62,3826	841,06	cooling	6,022	35,1059	13,8726	296,81	
6,024	34,8754	8,7217	365,59	cooling	6,024	42,0196	20,0719	306,8		6,024	121,9297	63,5794	815,65	cooling	6,024	34,1295	11,9635	309,85	
6,026	35,3464	11,0798	339,21	cooling	6,026	40,5138	14,646	361,59		6,026	120,9485	44,063	1074,74	cooling	6,026	35,6107	13,1819	313,52	
6,028	35,9094	8,4379	384,01	cooling	6,028	41,9254	17,5514	340,71		6,028	130,0229	55,087	1047,49	cooling	6,028	34,975	14,0603	292,36	
6,03	35,4258	6,0014	411,31	cooling	6,03	41,1248	18,4178	317,41		6,03	132,507	27,3667	1469,7	cooling	6,03	34,821	12,9196	306,15	
6,032	35,1129	12,5307	315,67	cooling	6,032	41,5346	16,4391	350,8		6,032	127,8921	45,8956	1146,19	cooling	6,032	34,4355	10,8059	330,31	
6,034	33,754	8,5642	352,12	cooling	6,034	40,6863	15,6542	349,91		6,034	128,0139	60,9916	936,87	cooling	6,034	35,1486	11,7019	327,75	
6,036	35,09	10,1637	348,43	cooling	6,036	40,981	17,5732	327,21		6,036	126,8779	60,8906	922,4	cooling	6,036	33,6758	10,8675	318,83	
6,038	33,4424	7,5138	362,44	cooling	6,038	40,2162	13,2569	376,85		6,038	125,9733	60,1378	920,28	cooling	6,038	34,6636	13,2081	299,92	
6,04	34,5568	3,0254	440,76	cooling	6,04	40,6322	15,966	344,8	cooling	6,04	129,1095	65,4366	890,05	cooling	6,04	34,3179	15,395	264,51	
6,042	34,4279	9,3165	351,02	cooling	6,042	39,9638	16,9949	321,07	cooling	6,042	140,2452	39,6247	1406,53	cooling	6,042	34,4157	11,4401	321,17	
6,044	34,2668	12,0014	311,24	cooling	6,044	40,2343	16,7616	328,11	cooling	6,044	132,9761	57,8707	1049,86	cooling	6,044	34,3059	13,4928	290,94	
6,046	33,9396	7,6681	367,24	cooling	6,046	39,564	21,0318	259,05	cooling	6,046	133,4396	71,2087	869,89	cooling	6,046	34,1701	10,0964	336,52	
6,048	36,7302	9,7223	377,53	cooling	6,048	38,4249	15,6857	317,86	cooling	6,048	133,4978	69,6371	892,68	cooling	6,048	34,723	11,8675	319,49	
6,05	36,0581	4,156	445,94	cooling	6,05	40,1125	17,8416	311,32	cooling	6,05	134,9741	78,8113	785,07	cooling	6,05	33,892	11,3293	315,39	
6,052	37,2277	3,9751	464,82	cooling	6,052	40,235	19,0594	296	cooling	6,052	127,6708	61,0644	931,06	cooling	6,052	34,8085	12,4938	311,93	
6,054	38,2133	15,1153	322,88	cooling	6,054	40,1997	19,2631	292,66	cooling	6,054	136,3201	47,1758	1246,11	cooling	6,054	34,3507	11,2571	322,81	
6,056	37,6882	11,2361	369,76	cooling	6,056	39,8403	17,3914	313,8	cooling	6,056	132,5301	63,7443	961,52	cooling	6,056	34,5144	14,3869	281,35	
6,058	37,6895	9,6423	392,06	cooling	6,058	40,2859	18,6165	302,9	cooling	6,058	132,8016	75,893	795,5	cooling	6,058	33,3683	10,9611	313,22	
6,06	36,9981	9,6865	381,78	cooling	6,06	39,3985	12,66	373,76	cooling	6,06	130,6639	59,7798	990,85	cooling	6,06	35,302	14,9578	284,38	
6,062	37,5637	6,7238	431,1	cooling	6,062	40,0796	19,8754	282,42	cooling	6,062	136,7292	63,4996	1023,64	cooling	6,062	33,7654	12,1237	302,52	
6,064	35,922	11,3264	343,81	cooling	6,064	39,1706	19,9228	269,05	cooling	6,064	127,8873	39,1596	1240,28	cooling	6,064	34,5702	13,0156	301,3	
6,066	35,2693	14,6762	287,86	cooling	6,066	40,0028	17,9036	308,91	cooling	6,066	129,5008	46,9824	1153,48	cooling	6,066	34,3335	17,0305	241,87	
6,068	34,2315	3,1746	434,13	cooling	6,068	39,183	16,4306	318,04	cooling	6,068	126,2978	57,4271	962,71	cooling	6,068	34,5794	13,7539	291,11	
6,07	36,9892	5,2149	444,16	cooling	6,07	39,3182	14,3459	349,08	cooling	6,07	125,9791	51,3532	1043,16	cooling	6,07	33,9532	11,91	308,13	
6,072	33,9779	-0,1266	476,73	cooling	6,072	39,3249	13,4297	361,98	cooling	6,072	125,8768	42,4625	1166,01	cooling	6,072	34,5435	11,9509	315,81	
6,074	35,7318	4,0931	442,26	cooling	6,074	40,3707	19,0787	297,63	cooling	6,074	134,794	66,6676	952,31	cooling	6,074	34,9573	10,2056	345,99	
6,076	34,374	15,9439	257,62	cooling	6,076	39,4352	15,76	330,94	cooling	6,076	137,8379	40,9587	1354,23	cooling	6,076	34,7314	11,9833	317,98	
6,078	33,3088	12,4491	291,59	cooling	6,078	40,7489	15,8674	347,81	cooling	6,078	138,0023	64,4001	1028,85	cooling	6,078	34,4095	16,6949	247,62	

6,08	32,3821	7,332	350,16	cooling	6,08	40,0171	18,7759	296,92	cooling	6,08	136,8596	73,6096	884,14	cooling	6,08	34,1693	10,089	336,61	
6,082	33,8053	11,5604	310,95	cooling	6,082	39,6183	12,5726	378,06	cooling	6,082	138,5257	57,5139	1132,42	cooling	6,082	35,6561	14,7181	292,68	
6,084	31,882	2,0663	416,78	cooling	6,084	40,1086	13,9402	365,8	cooling	6,084	139,7107	65,8582	1032,35	cooling	6,084	33,5936	7,6504	362,65	
6,086	31,5318	6,5545	349,14	cooling	6,086	39,9265	16,3163	330,04	cooling	6,086	140,9975	75,0469	921,89	cooling	6,086	35,2546	9,4252	361,06	
6,088	31,2472	14,4616	234,64	cooling	6,088	39,4516	16,8941	315,32	cooling	6,088	152,5952	40,6506	1564,82	cooling	6,088	34,2136	11,9283	311,52	
6,09	30,8769	5,1537	359,57	cooling	6,09	39,2756	16,8657	313,26	cooling	6,09	147,0026	74,7215	1010,38	cooling	6,09	34,2591	13,6279	288,39	
6,092	33,1554	11,5529	301,97	cooling	6,092	39,5199	16,2519	325,25	cooling	6,092	144,1706	84,2015	838,28	cooling	6,092	33,6774	11,8734	304,79	
6,094	31,4169	9,633	304,51	cooling	6,094	39,2847	12,5325	373,96	cooling	6,094	144,385	58,7643	1196,85	cooling	6,094	35,3817	16,6682	261,59	
6,096	34,6887	10,806	333,84	cooling	6,096	40,1957	20,5879	274,09	cooling	6,096	147,2511	76,9703	982,42	cooling	6,096	34,0437	10,5391	328,56	
6,098	33,443	15,3339	253,14	cooling	6,098	39,0546	18,3394	289,57	cooling	6,098	135,3434	61,7035	1029,38	cooling	6,098	34,4855	11,6244	319,56	
6,1	33,2969	9,8357	327,95	cooling	6,1	39,8498	16,689	323,75	cooling	6,1	148,1135	40,9626	1497,81	cooling	6,1	34,0574	11,0469	321,65	
6,102	32,8001	-2,2704	490,23	cooling	6,102	39,2313	18,1643	294,49	cooling	6,102	141,9114	68,1053	1031,7	cooling	6,102	34,3201	4,2391	420,49	
6,104	35,3857	5,7928	413,66	cooling	6,104	39,4966	17,5686	306,52	cooling	6,104	139,5598	69,9873	972,52	cooling	6,104	34,6678	7,5616	378,9	cooling
6,106	32,6458	0,4928	449,45	cooling	6,106	38,8854	17,5099	298,8	cooling	6,106	137,8046	53,2457	1182,01	cooling	6,106	34,0713	7,6317	369,59	cooling
6,108	34,0025	9,224	346,37	cooling	6,108	38,4909	16,9895	300,56	cooling	6,108	140,5002	78,407	867,97	cooling	6,108	35,0432	11,6158	327,48	cooling
6,11	32,7586	13,7059	266,33	cooling	6,11	38,2712	13,642	344,28	cooling	6,11	127,1473	64,1055	881,23	cooling	6,11	34,1264	13,0795	294,2	cooling
6,112	32,838	3,7111	407,15	cooling	6,112	38,698	13,8156	347,82	cooling	6,112	134,2885	57,4418	1074,2	cooling	6,112	34,1419	14,5906	273,3	cooling
6,114	34,336	5,2993	405,89	cooling	6,114	38,003	15,3285	316,96	cooling	6,114	127,1639	69,1976	810,28	cooling	6,114	34,0098	9,024	349,26	cooling
6,116	34,6275	7,9312	373,18	cooling	6,116	38,1288	14,6089	328,77	cooling	6,116	125,7665	62,0644	890,46	cooling	6,116	34,7785	13,8862	292,04	cooling
6,118	36,2872	8,0518	394,69	cooling	6,118	39,4226	21,1142	255,92	cooling	6,118	124,7486	47,5126	1079,64	cooling	6,118	33,6136	9,23	340,85	cooling
6,12	36,2317	13,7691	313,99	cooling	6,12	38,8105	18,2169	287,87	cooling	6,12	129,0719	67,0059	867,59	cooling	6,12	34,5957	12,7935	304,76	cooling
6,122	37,1287	14,1495	321,22	cooling	6,122	39,3157	20,3457	265,17	cooling	6,122	123,775	54,3401	970,6	cooling	6,122	33,7872	10,3831	327,15	cooling
6,124	37,4061	4,658	457,77	cooling	6,124	38,9304	20,9284	251,64	cooling	6,124	130,2617	48,9924	1136,02	cooling	6,124	33,9137	8,76	351,61	cooling
6,126	39,5938	13,4454	365,52	cooling	6,126	39,2912	18,2816	293,68	cooling	6,126	127,2119	61,7666	914,83	cooling	6,126	33,752	10,0694	331,05	cooling
6,128	37,332	6,963	424,51	cooling	6,128	38,6843	14,7982	333,89	cooling	6,128	126,1327	53,9003	1009,7	cooling	6,128	34,1308	12,9047	296,71	cooling
6,13	39,291	14,8995	340,96	cooling	6,13	38,6256	17,8221	290,8	cooling	6,13	125,266	35,6872	1252,18	cooling	6,13	33,5359	13,4202	281,19	cooling
6,132	37,7466	16,4125	298,22	cooling	6,132	37,9999	11,8202	365,95	cooling	6,132	129,2981	55,4127	1032,81	cooling	6,132	33,8197	14,048	276,38	cooling
6,134	37,8205	11,2449	371,49	cooling	6,134	38,8263	15,5028	326,03	cooling	6,134	124,609	50,5036	1035,88	cooling	6,134	33,7628	15,8242	250,75	cooling
6,136	37,4176	6,9808	425,46	cooling	6,136	37,7227	17,456	283,3	cooling	6,136	125,6912	65,1682	846,02	cooling	6,136	34,0167	13,2246	290,64	cooling
6,138	38,2349	13,4622	346,29	cooling	6,138	38,5089	17,9963	286,73	cooling	6,138	122,1629	70,2757	725,31	cooling	6,138	33,587	13,909	275,07	cooling
6,14	37,5901	11,0453	371,06	cooling	6,14	38,7805	19,4261	270,55	cooling	6,14	120,4613	47,3982	1021,31	cooling	6,14	33,8889	8,685	352,31	cooling
6,142	37,998	20,2484	248,11	cooling	6,142	38,2513	14,2746	335,16		6,142	120,9918	37,3826	1168,73	cooling	6,142	34,1424	9,7346	341,18	cooling
6,144	36,5638	16,9654	273,96	cooling	6,144	38,8551	12,6495	366,32	cooling	6,144	125,7399	59,8789	920,64	cooling	6,144	33,8349	6,3334	384,43	cooling
6,146	36,4761	5,6695	430,63	cooling	6,146	39,262	17,0127	311,01	cooling	6,146	125,978	46,5297	1110,57	cooling	6,146	33,8768	10,885	321,39	cooling
6,148	37,5985	9,8786	387,48	cooling	6,148	38,4308	18,7584	274,99	cooling	6,148	123,4634	66,7155	793,25	cooling	6,148	33,7041	11,8817	305,05	cooling
6,15	36,8143	9,3847	383,43	cooling	6,15	38,2438	17,844	285,16	cooling	6,15	122,3266	72,5547	695,74	cooling	6,15	33,7476	13,9735	276,41	cooling
6,152	37,7665	12,245	356,75	cooling	6,152	38,8279	19,6729	267,76	cooling	6,152	121,7728	52,6202	966,65	cooling	6,152	33,354	10,0759	325,39	cooling
6,154	37,3331	10,3106	377,73	cooling	6,154	39,1109	15,3594	332,01		6,154	121,6637	48,4548	1023,35	cooling	6,154	34,1332	9,5223	344,03	cooling
6,156	38,0044	6,4372	441,26	cooling	6,156	39,1108	22,118	237,53		6,156	127,9354	69,9544	810,49	cooling	6,156	33,0684	7,6992	354,62	cooling
6,158	37,9962	4,0921	473,93	cooling	6,158	38,5753	20,6842	250,09		6,158	129,0724	48,2428	1129,88	cooling	6,158	33,5047	11,3357	309,89	cooling
6,16	37,9539	12,3152	358,39	cooling	6,16	39,5229	18,3458	296,02		6,16	125,9201	59,3528	930,51	cooling	6,16	32,8233	12,3759	285,82	cooling
6,162	36,8739	6,4944	424,66	cooling	6,162	39,2761	16,7396	315,03		6,162	123,8529	53,7075	980,53	cooling	6,162	34,0427	12,8152	296,73	cooling
6,164	37,5273	9,2134	395,79	cooling	6,164	38,4977	14,6876	332,83		6,164	123,6706	34,1051	1251,99	cooling	6,164	32,9289	14,1909	261,93	cooling
6,166	35,5053	6,6295	403,64	cooling	6,166	38,8947	12,9037	363,32		6,166	123,3627	33,1791	1260,63	cooling	6,166	33,9465	12,1239	305,05	cooling
6,168	35,1221	7,9545	379,76	cooling	6,168	39,1352	17,932	296,39		6,168	123,4163	49,1667	1037,9	cooling	6,168	33,7342	14,0836	274,69	cooling
6,17	34,9482	8,303	372,46	cooling	6,17	37,9741	16,2558	303,59		6,17	127,7586	53,459	1038,6	cooling	6,17	33,8783	11,0731	318,78	cooling
6,172	35,1878	6,8933	395,52	cooling	6,172	38,8791	18,41	286,13		6,172	125,1255	66,2399	823,13	cooling	6,172	33,3723	10,2387	323,37	cooling
6,174	35,4368	6,8498	399,6	cooling	6,174	38,1963	17,0752	295,24		6,174	124,8403	53,8697	992,06	cooling	6,174	34,3691	10,4464	334,4	cooling
6,176	36,5721	9,6413	376,45	cooling	6,176	38,3175	11,375	376,62		6,176	124,6295	32,8293	1283,23	cooling	6,176	33,9888	12,5132	300,2	cooling
6,178	36,4351	7,3329	406,81	cooling	6,178	38,4123	17,7818	288,38		6,178	128,5508	42,163	1207,57	cooling	6,178	33,3687	10,0034	326,61	cooling
6,18	37,1924	7,19	419,39	cooling	6,18	38,3933	20,2357	253,82		6,18	123,9354	50,4301	1027,49	cooling	6,18	33,6762	14,9206	262,18	cooling
6,182	36,952	8,5033	397,67	cooling	6,182	37,9871	17,734	283,11		6,182	125,6292	69,9675	778,07	cooling	6,182	32,7164	11,2411	300,19	cooling
6,184	38,5828	6,7622	444,8	cooling	6,184	38,4708	16,2419	310,73		6,184	123,3709	74,6523	681,01	cooling	6,184	33,5848	12,814	290,35	cooling
6,186	37,9432	13,8866	336,28	cooling	6,186	38,2395	11,8188	369,32		6,186	123,7102	59,0482	903,88	cooling	6,186	32,7019	11,5481	295,7	cooling
6,188	36,9915	10,8642	365,22	cooling	6,188	39,2207	10,7346	398,19		6,188	122,0184	40,4423	1140,31	cooling	6,188	34,1297	12,1342	307,46	
6,19	37,0536	9,0906	390,88	cooling	6,19	39,0731	17,1249	306,8	cooling	6,19	126,9676	52,891	1035,48	cooling	6,19	33,0695	11,5303	301,09	

6,192	37,9388	12,0561	361,8	cooling	6,192	38,3669	11,629	373,76	cooling	6,192	122,7177	41,2732	1138,47	cooling	6,192	33,8238	13,7993	279,91	
6,194	37,1632	15,0414	309,23	cooling	6,194	39,5057	11,8875	386,06	cooling	6,194	123,1999	47,6028	1056,74	cooling	6,194	32,7601	10,8109	306,82	
6,196	38,8389	14,1962	344,47	cooling	6,196	38,4146	12,8538	357,3	cooling	6,196	121,3475	50,1997	994,54	cooling	6,196	34,2439	11,9305	311,91	
6,198	40,1759	13,9328	366,84	cooling	6,198	38,5529	11,3663	380,03	cooling	6,198	121,0321	44,7693	1066,04	cooling	6,198	32,8212	12,0645	290,15	
6,2	39,9584	5,9825	474,93	cooling	6,2	38,5164	15,7072	318,84	cooling	6,2	120,0508	43,7157	1067,05	cooling	6,2	33,8384	11,4599	312,82	
6,202	40,7688	5,9549	486,65	cooling	6,202	37,7423	16,3284	299,34	cooling	6,202	123,8294	69,8284	754,85	cooling	6,202	33,4231	16,0229	243,23	
6,204	40,9957	7,7112	465,27	cooling	6,204	37,4374	13,982	327,87	cooling	6,204	119,9155	55,1881	904,79	cooling	6,204	33,1874	12,3202	291,69	
6,206	41,9648	9,3443	455,99	cooling	6,206	38,5771	19,0264	273,29	cooling	6,206	119,7615	50,8097	963,84	cooling	6,206	33,2731	13,8765	271,14	
6,208	41,4204	15,1311	367,48	cooling	6,208	37,565	18,7329	263,24	cooling	6,208	118,3744	42,8182	1056,16	cooling	6,208	33,3631	12,8921	286,15	
6,21	42,4031	17,9694	341,55	cooling	6,21	38,0857	16,2878	304,7	cooling	6,21	119,1823	36,8352	1151,09	cooling	6,21	33,5843	15,0373	259,26	
6,212	41,8631	12,8942	404,94	cooling	6,212	38,4868	21,6204	235,77	cooling	6,212	120,5624	40,7764	1115,29	cooling	6,212	33,1568	12,2163	292,72	
6,214	43,1509	7,7326	495,1	cooling	6,214	37,6721	18,2198	271,91	cooling	6,214	121,3869	65,2156	785,19	cooling	6,214	34,1373	15,0511	266,8	
6,216	43,3447	6,4048	516,36	cooling	6,216	37,8276	19,1585	260,97	cooling	6,216	118,4071	76,3477	587,93	cooling	6,216	33,3276	9,5288	332,67	
6,218	46,0062	10,6716	493,92	cooling	6,218	37,6698	19,2809	257,05		6,218	119,6039	71,0179	679,16	cooling	6,218	34,4943	14,7452	276,06	
6,22	46,0199	20,6945	354,01	cooling	6,22	38,283	14,3879	334,02		6,22	118,7484	58,6551	840,02	cooling	6,22	32,6304	11,002	302,33	
6,222	46,6606	19,7196	376,59	cooling	6,222	37,9403	13,5417	341,06		6,222	118,677	46,774	1005,1	cooling	6,222	33,9573	13,1823	290,4	
6,224	47,6699	14,6977	460,9	cooling	6,224	37,7273	18,54	268,21		6,224	119,0336	36,3542	1155,74	cooling	6,224	32,5782	12,7845	276,69	
6,226	49,7764	11,9452	528,82	cooling	6,226	36,9915	15,5293	300,01		6,226	120,5521	49,3864	994,79	cooling	6,226	33,3485	13,888	272,03	
6,228	50,1031	16,2098	473,78	cooling	6,228	37,5102	17,5299	279,29		6,228	118,7083	68,6819	699,29	cooling	6,228	32,4962	13,5224	265,22	
6,23	51,1723	23,117	392,17	cooling	6,23	36,5146	16,5235	279,45		6,23	118,0683	67,4134	708,08	cooling	6,23	33,4254	15,0707	256,57	
6,232	50,9684	21,5272	411,54	cooling	6,232	37,7558	16,1797	301,6		6,232	119,1037	64,9581	756,87	cooling	6,232	32,4935	12,8293	274,88	
6,234	49,3631	6,014	605,96	cooling	6,234	38,2172	23,1185	211,06		6,234	118,4715	52,4719	922,58	cooling	6,234	33,4077	11,6823	303,69	
6,236	49,2199	7,3732	584,95	cooling	6,236	37,4883	19,559	250,63		6,236	118,9998	32,1658	1213,81	cooling	6,236	33,1548	14,2089	264,84	
6,238	49,0025	19,8229	407,89	cooling	6,238	38,1709	16,7313	299,69		6,238	119,0315	34,0094	1188,48	cooling	6,238	32,823	8,1894	344,34	
6,24	48,7235	27,5932	295,37	cooling	6,24	38,8756	17,7589	295,18		6,24	118,5161	37,9184	1126,64	cooling	6,24	33,6495	12,8969	290,09	
6,242	47,4542	21,9373	356,69	cooling	6,242	37,9739	15,7728	310,34		6,242	119,1698	45,4561	1030,41	cooling	6,242	32,8253	10,2549	315,5	
6,244	47,9474	11,2943	512,36	cooling	6,244	38,1377	15,2445	320,01		6,244	120,6292	65,6099	769,09	cooling	6,244	33,2107	13,2036	279,67	
6,246	48,0498	4,4093	610,03	cooling	6,246	38,3639	18,0603	283,81		6,246	118,2716	57,6094	847,97	cooling	6,246	32,7189	10,4105	311,84	
6,248	48,6937	9,2947	550,74	cooling	6,248	37,639	13,4133	338,64		6,248	120,7471	53,1306	945,18	cooling	6,248	33,8621	13,1562	289,44	
6,25	48,822	19,8667	404,75	cooling	6,25	38,0181	17,5498	286,12		6,25	118,6084	46,5641	1007,07	cooling	6,25	32,8529	6,395	369,84	
6,252	48,7841	17,2951	440,17	cooling	6,252	37,5916	16,6102	293,29		6,252	118,639	35,3346	1164,47	cooling	6,252	34,5803	12,7986	304,48	
6,254	48,12	10,0111	532,71	cooling	6,254	38,7258	13,8501	347,73		6,254	119,4522	46,7165	1016,74	cooling	6,254	33,1887	10,0918	322,86	
6,256	47,7013	4,4134	605,1	cooling	6,256	38,0839	16,8217	297,21		6,256	119,851	60,3219	832,13	cooling	6,256	34,3112	11,891	313,4	
6,258	47,9268	13,0209	487,93	cooling	6,258	38,2105	18,9805	268,81		6,258	118,3362	39,1299	1107,19	cooling	6,258	32,4714	11,4291	294,14	
6,26	47,5538	17,8361	415,41	cooling	6,26	38,1825	18,3829	276,77		6,26	120,4992	52,4899	950,67	cooling	6,26	32,8891	12,8569	280,02	
6,262	46,6334	17,7348	403,96	cooling	6,262	38,5971	18,7957	276,79		6,262	116,8697	55,5995	856,47	cooling	6,262	31,6682	13,3504	256,06	
6,264	46,0731	9,7485	507,76	cooling	6,264	37,5382	14,3564	324,05		6,264	119,8679	43,709	1064,59	cooling	6,264	32,0862	14,0062	252,73	
6,266	46,4265	7,7423	540,75	cooling	6,266	38,7121	13,9565	346,05		6,266	120,1669	59,6106	846,49	cooling	6,266	31,3033	12,4562	263,45	
6,268	45,7482	5,7304	559,39	cooling	6,268	38,0346	18,498	273,09		6,268	118,4332	49,1452	968,54	cooling	6,268	32,3446	12,9945	270,49	
6,27	45,394	9,5612	500,89	cooling	6,27	37,2535	14,4647	318,55		6,27	120,4649	26,8929	1308	cooling	6,27	32,1095	16,4042	219,54	
6,272	44,3693	8,5369	500,88	cooling	6,272	38,05	14,525	328,84		6,272	123,0517	49,3833	1029,77	cooling	6,272	32,3906	11,9038	286,37	heating
6,274	44,3235	9,1739	491,34	cooling	6,274	38,078	12,858	352,54		6,274	118,1063	50,838	940,31	cooling	6,274	33,5129	16,733	234,56	heating
6,276	43,4251	10,2896	463,19	cooling	6,276	38,2287	13,9213	339,78		6,276	121,1098	53,9077	939,39	cooling	6,276	32,8191	13,2197	273,97	heating
6,278	44,0027	12,2448	443,93	cooling	6,278	37,9875	17,6288	284,58		6,278	122,0223	74,7801	660,38	cooling	6,278	33,3374	17,6066	219,89	heating
6,28	44,1319	15,8055	395,96	cooling	6,28	38,2372	18,1664	280,56		6,28	119,9006	55,365	902,11	cooling	6,28	32,2445	15,3367	236,35	heating
6,282	44,6154	17,386	380,63	cooling	6,282	37,5285	13,2924	338,78		6,282	120,3837	48,9434	998,63	cooling	6,282	32,6335	15,9623	233,04	heating
6,284	44,2694	16,6125	386,6	cooling	6,284	38,2337	17,2029	293,98		6,284	121,9193	57,4924	900,59	cooling	6,284	31,4584	9,2241	310,8	heating
6,286	44,6747	12,4844	449,97	cooling	6,286	37,4407	18,9921	257,88		6,286	118,5815	39,2407	1109,07	cooling	6,286	32,7687	13,8899	263,9	heating
6,288	44,3831	11,5562	458,87	cooling	6,288	38,0661	20,604	244,1		6,288	120,3562	50,0706	982,49	cooling	6,288	31,7849	9,2244	315,36	heating
6,29	44,882	11,6695	464,26	cooling	6,29	36,8938	21,0128	221,99		6,29	119,1464	59,2945	836,64	cooling	6,29	33,1782	13,4595	275,64	heating
6,292	45,1757	17,9895	380,02	cooling	6,292	37,4651	16,6254	291,31		6,292	121,6691	48,3345	1025,11	cooling	6,292	32,0347	14,0095	251,97	heating
6,294	44,2623	13,2907	432,94	cooling	6,294	37,721	17,6675	280,32		6,294	121,976	58,8986	881,73	cooling	6,294	33,2966	14,7421	259,36	heating
6,296	43,8431	13,483	424,39	cooling	6,296	37,9094	18,9019	265,7		6,296	122,0423	60,3152	862,85	cooling	6,296	32,7325	16,8706	221,73	heating
6,298	43,2563	14,4758	402,31	cooling	6,298	37,9893	18,4308	273,4		6,298	125,9295	47,9343	1090,26	cooling	6,298	32,6928	16,9801	219,64	heating
6,3	42,9422	12,7036	422,69	cooling	6,3	38,4183	16,1966	310,63		6,3	126,3488	63,3062	881,24	cooling	6,3	32,1162	15,5442	231,65	heating
6,302	42,2031	7,7023	482,27	cooling	6,302	37,9368	16,2866	302,64		6,302	123,6602	52,3921	996,22	cooling	6,302	32,745	13,926	263,06	heating

6,304	42,3671	13,2417	407,13	cooling	6,304	37,4893	16,7736	289,57		6,304	125,7008	40,0004	1197,96	cooling	6,304	32,1772	14,3419	249,31	heating
6,306	42,0811	15,1484	376,48	cooling	6,306	38,3108	21,1761	239,52		6,306	126,1211	59,3062	933,97	cooling	6,306	32,6563	9,7054	320,82	heating
6,308	42,9894	14,6674	395,9	cooling	6,308	37,6003	15,1343	314,04		6,308	123,0297	64,468	818,61	cooling	6,308	33,9509	16,2418	247,55	heating
6,31	42,6543	9,8566	458,46	cooling	6,31	38,4541	16,7568	303,3		6,31	125,2384	63,6686	860,66	cooling	6,31	32,8531	13,7255	267,38	heating
6,312	43,5863	5,6758	529,93	cooling	6,312	37,6288	18,8682	262,25		6,312	126,9013	72,9371	754,34	cooling	6,312	33,7375	18,6763	210,53	heating
6,314	43,2577	8,1175	491,21	cooling	6,314	38,4909	20,4052	252,81		6,314	123,7611	47,0531	1072,26	cooling	6,314	33,0211	14,3943	260,38	heating
6,316	43,6356	14,0253	413,91	cooling	6,316	37,545	18,0811	272,08		6,316	125,593	40,4628	1189,99	cooling	6,316	33,6904	12,6444	294,19	heating
6,318	43,1642	15,2297	390,48	cooling	6,318	38,2147	19,1559	266,41	heating	6,318	126,3927	54,2925	1007,85	cooling	6,318	32,4416	9,1137	326,09	heating
6,32	43,7782	7,0198	513,83	cooling	6,32	37,723	18,6652	266,4	heating	6,32	124,5503	49,8582	1044,09	cooling	6,32	33,4616	15,7269	247,9	heating
6,322	43,5923	5,3288	534,87	cooling	6,322	38,4258	21,4375	237,47	heating	6,322	124,6555	50,663	1034,31	cooling	6,322	32,1187	13,4866	260,45	heating
6,324	43,6171	11,8184	444,5	cooling	6,324	37,1778	17,7542	271,51	heating	6,324	124,7261	60,6135	896,2	cooling	6,324	33,0872	14,7622	256,16	heating
6,326	43,5485	23,2121	284,27	cooling	6,326	38,0153	16,8325	296,1	heating	6,326	121,9567	46,2417	1058,38	cooling	6,326	31,9744	9,8307	309,54	heating
6,328	43,1979	23,0392	281,79	cooling	6,328	37,7926	19,1902	260,03	heating	6,328	123,395	45,9686	1082,31	cooling	6,328	33,5108	7,161	368,33	heating
6,33	42,9054	14,2942	399,94	cooling	6,33	37,4923	15,854	302,47	heating	6,33	120,7812	51,4427	969,25	cooling	6,33	32,8751	9,5065	326,66	heating
6,332	42,868	2,8611	559,24	cooling	6,332	38,4087	17,3427	294,47	heating	6,332	121,0801	48,0944	1020,23	cooling	6,332	33,0764	10,5038	315,53	heating
6,334	43,9908	7,572	509,08	cooling	6,334	38,3883	17,2693	295,21	heating	6,334	121,5089	46,2833	1051,54	cooling	6,334	33,3996	13,013	284,98	heating
6,336	42,6178	13,6856	404,43	cooling	6,336	38,4095	17,5151	292,07	heating	6,336	120,7424	45,0797	1057,65	cooling	6,336	33,9605	11,0274	320,57	heating
6,338	43,1099	17,9422	351,81	cooling	6,338	38,033	18,6295	271,23	heating	6,338	123,1026	46,5723	1069,78	cooling	6,338	33,2759	13,7803	272,52	heating
6,34	42,2959	10,7045	441,6	cooling	6,34	39,0534	21,9345	239,3	heating	6,34	130,7891	54,769	1062,65	cooling	6,34	33,3333	12,2228	295,09	heating
6,342	42,9928	6,8947	504,6	cooling	6,342	38,3921	15,1343	325,11	heating	6,342	127,7161	61,24	929,24	cooling	6,342	34,0642	17,0231	238,21	heating
6,344	42,4375	12,2787	421,58	cooling	6,344	38,6378	18,2781	284,6	heating	6,344	128,0043	65,3703	875,53	cooling	6,344	33,6625	12,5415	295,24	heating
6,346	43,0293	17,7125	353,89	cooling	6,346	37,727	18,8257	264,21	heating	6,346	128,4378	68,2979	840,67	cooling	6,346	34,5227	17,101	243,53	heating
6,348	42,6072	17,8993	345,38	cooling	6,348	38,7878	22,4861	227,87	heating	6,348	125,9094	53,3896	1013,72	cooling	6,348	33,821	12,7247	294,89	heating
6,35	42,8807	14,936	390,63	cooling	6,35	37,6842	16,9406	289,96	heating	6,35	121,6897	44,8452	1074,17	cooling	6,35	34,8158	15,3782	271,71	heating
6,352	42,5735	10,3014	451,12	cooling	6,352	38,1104	13,9636	337,54	heating	6,352	125,6699	50,5842	1049,59	cooling	6,352	33,3245	11,4599	305,64	heating
6,354	43,8095	11,4777	451,95	cooling	6,354	38,385	13,4804	348,13	heating	6,354	120,668	52,5669	951,95	cooling	6,354	34,2205	12,9543	297,27	heating
6,356	43,2547	19,6887	329,42	cooling	6,356	38,6534	16,5368	309,16	heating	6,356	119,5403	63,0625	789,48	cooling	6,356	33,1137	11,5111	301,97	heating
6,358	42,6846	16,7945	361,91	cooling	6,358	38,0865	16,6113	300,19	heating	6,358	118,9911	59,1437	836,58	cooling	6,358	33,6756	13,4311	282,99	heating
6,36	43,6003	18,5251	350,51	cooling	6,36	38,6651	17,9789	289,16	heating	6,36	119,1237	40,8357	1094,35	cooling	6,36	32,503	12,0609	285,75	heating
6,362	43,3031	16,0271	381,28	cooling	6,362	38,6188	17,0546	301,44	heating	6,362	116,2135	39,3446	1074,51	cooling	6,362	33,7477	13,7828	279,08	heating
6,364	43,0791	12,1704	432,06	cooling	6,364	38,2572	15,9552	311,75	heating	6,364	119,4158	46,7541	1015,7	cooling	6,364	32,7214	13,6767	266,22	heating
6,366	43,2457	9,6165	470,09	cooling	6,366	38,5564	20,2937	255,29	heating	6,366	119,9758	55,3899	902,81	cooling	6,366	33,1431	12,1204	293,87	heating
6,368	44,0405	14,6971	410,18	cooling	6,368	38,1788	16,0076	309,92	heating	6,368	118,9233	67,0542	725,05	cooling	6,368	33,3637	14,2624	267,01	heating
6,37	42,9477	15,1886	388,03	cooling	6,37	38,4253	17,2049	296,63	heating	6,37	118,7447	64,2308	762,02	cooling	6,37	33,8915	11,3199	315,52	heating
6,372	43,2097	18,693	342,71	cooling	6,372	37,6619	14,8159	319,35	heating	6,372	120,0043	62,5476	803,16	cooling	6,372	33,6315	16,3082	242,15	heating
6,374	42,1567	14,1409	391,62	cooling	6,374	38,754	16,4855	311,28		6,374	116,8837	72,4963	620,47	cooling	6,374	33,6793	14,7246	264,96	heating
6,376	43,0479	11,7282	437,8	cooling	6,376	38,1556	13,741	341,28		6,376	115,3602	64,4126	712,17	cooling	6,376	34,3587	17,0004	242,64	heating
6,378	42,2451	12,5769	414,72	cooling	6,378	38,1572	15,4065	318,02		6,378	115,0634	61,3452	750,9	cooling	6,378	33,1318	14,035	266,95	heating
6,38	43,1941	14,568	400,15	cooling	6,38	38,0689	13,7049	340,57		6,38	113,8156	60,6249	743,53	cooling	6,38	33,888	15,7675	253,3	heating
6,382	42,7393	14,1175	400,09	cooling	6,382	39,0404	16,4847	315,3		6,382	112,6583	49,2426	886,46	cooling	6,382	33,6925	12,3247	298,69	heating
6,384	43,4422	10,4435	461,27	cooling	6,384	37,9749	12,953	349,77		6,384	114,4057	49,6767	904,82	cooling	6,384	34,3227	14,2074	281,18	heating
6,386	43,753	7,9465	500,52	cooling	6,386	38,5822	12,6061	363,11		6,386	114,7325	62,0934	735,82	cooling	6,386	33,1778	11,9518	296,71	heating
6,388	45,1717	10,9792	477,96	cooling	6,388	38,7438	17,0828	302,79		6,388	115,9724	53,4709	873,68	cooling	6,388	34,7595	15,2463	272,77	heating
6,39	44,8484	18,1146	373,7	cooling	6,39	38,4095	16,0448	312,63		6,39	116,2549	59,0224	800,03	cooling	6,39	33,1991	16,4692	233,86	heating
6,392	44,8908	19,0129	361,73	cooling	6,392	38,3853	16,7159	302,91		6,392	117,5305	53,993	888,16	cooling	6,392	33,5109	17,0588	229,98	heating
6,394	45,3469	16,3041	405,98	cooling	6,394	38,6153	15,7468	319,67		6,394	116,5576	28,3357	1233,21	cooling	6,394	33,4121	17,8272	217,85	heating
6,396	45,0025	4,9666	559,64	cooling	6,396	38,6252	18,9521	275		6,396	118,532	28,1981	1262,73	cooling	6,396	34,4538	14,2252	282,77	heating
6,398	45,4843	4,2336	576,62	cooling	6,398	38,5061	20,338	253,96		6,398	116,7098	38,7634	1089,58	cooling	6,398	33,6686	12,2109	299,95	heating
6,4	45,2655	9,9079	494,25	cooling	6,4	39,1626	23,1619	223,67		6,4	119,526	46,7576	1017,19	cooling	6,4	33,9084	8,8371	350,46	heating
6,402	46,1821	20,0831	364,83	cooling	6,402	39,1505	17,7606	299		6,402	118,2507	61,1372	798,36	cooling	6,402	34,4102	10,8365	329,52	heating
6,404	45,3636	18,121	380,81	cooling	6,404	39,3254	21,6675	246,83		6,404	118,586	61,0162	804,74	cooling	6,404	34,1953	9,7314	341,97	heating
6,406	46,2036	14,5522	442,44	cooling	6,406	38,3976	18,6797	275,63		6,406	119,6521	46,8195	1018,09	cooling	6,406	34,3718	14,9539	271,43	heating
6,408	44,9496	5,5531	550,7	cooling	6,408	39,4758	21,3886	252,83		6,408	121,0896	52,5131	958,6	cooling	6,408	34,057	11,9796	308,61	heating
6,41	45,298	6,8857	536,95	cooling	6,41	38,4458	21,3675	238,73		6,41	119,2341	39,3588	1116,54	cooling	6,41	34,8216	16,1479	261,03	heating
6,412	44,0677	15,2918	402,24	cooling	6,412	38,4267	20,6405	248,63	heating	6,412	121,4712	46,1776	1052,49	cooling	6,412	32,7636	11,678	294,74	heating
6,414	44,1602	20,124	335,99	cooling	6,414	38,128	19,6397	258,44	heating	6,414	120,5508	62,4183	812,61	cooling	6,414	33,7398	14,3753	270,69	heating

6,416	43,6863	15,0171	400,75	cooling	6,416	38,1872	21,4352	234,17	heating	6,416	117,4449	61,8024	777,8	cooling	6,416	32,9741	12,6228	284,48	heating
6,418	44,948	10,3762	483,26	cooling	6,418	37,2322	19,118	253,21	heating	6,418	116,0719	62,5797	747,74	cooling	6,418	33,3231	12,8181	286,63	heating
6,42	45,2396	6,9125	535,76	cooling	6,42	38,0476	17,4108	288,47	heating	6,42	119,0508	78,543	566,24	cooling	6,42	32,704	12,1788	286,91	heating
6,422	46,6667	12,8165	473,18	cooling	6,422	37,4977	19,1907	255,91	heating	6,422	121,7726	41,7953	1117,96	cooling	6,422	34,3933	15,4344	265,02	heating
6,424	45,9409	20,6671	353,29	cooling	6,424	37,6583	20,0818	245,69	heating	6,424	117,6896	47,2458	984,7	cooling	6,424	33,4076	15,0614	256,45	heating
6,426	45,0192	14,3456	428,77	cooling	6,426	37,8567	22,0593	220,83	heating	6,426	118,6063	57,2606	857,52	cooling	6,426	34,0187	13,912	281,06	heating
6,428	45,9251	13,5612	452,4	cooling	6,428	38,5927	17,1617	299,57	heating	6,428	117,0721	40,1383	1075,42	cooling	6,428	34,3437	16,1869	253,81	heating
6,43	44,4673	10,4612	475,36	cooling	6,43	38,66	17,1565	300,59	heating	6,43	115,4278	41,7032	1030,56	cooling	6,43	34,8798	12,8106	308,5	heating
6,432	44,5195	12,6188	445,92	cooling	6,432	38,6072	15,449	323,72	heating	6,432	125,2902	83,1507	589,05	cooling	6,432	33,8565	14,469	271,01	heating
6,434	43,5088	15,1646	396,21	cooling	6,434	39,5346	20,2096	270,13	heating	6,434	134,9684	58,1974	1073,15	cooling	6,434	33,7477	10,175	329,51	heating
6,436	43,4301	13,5261	418,01	cooling	6,436	38,7281	17,8619	291,68	heating	6,436	126,8046	72,8915	753,63	cooling	6,436	34,3747	10,2044	337,87	heating
6,438	42,574	8,9338	470,24	cooling	6,438	38,5993	15,7123	319,93	heating	6,438	126,8668	71,5908	772,68	cooling	6,438	33,4268	8,017	355,19	heating
6,44	43,8888	15,1758	401,36	cooling	6,44	38,485	12,0382	369,69	heating	6,44	127,5433	40,9444	1210,52	cooling	6,44	33,7436	13,2305	286,74	heating
6,442	42,5136	8,2129	479,47	cooling	6,442	38,9828	15,2598	331,61	heating	6,442	126,0899	37,688	1235,73	cooling	6,442	33,312	9,7406	329,49	heating
6,444	43,3752	10,1154	464,92	cooling	6,444	37,6848	16,2606	299,48	heating	6,444	128,7571	58,8441	977,28	cooling	6,444	34,5452	11,9598	315,71	heating
6,446	41,8347	12,072	416,04	cooling	6,446	38,179	18,3224	277,57	heating	6,446	138,5975	53,625	1187,79	cooling	6,446	33,9123	5,5531	396,42	heating
6,448	42,0458	11,7684	423,23	cooling	6,448	37,861	17,0307	291,18	heating	6,448	132,269	81,974	703,05	cooling	6,448	34,7572	9,6525	350,93	
6,45	41,8943	15,012	375,78	cooling	6,45	38,2279	13,6875	343,04	heating	6,45	128,5241	81,1051	662,85	cooling	6,45	33,9667	6,41	385,2	
6,452	41,3187	12,0282	409,44	cooling	6,452	38,369	17,8302	287,1		6,452	128,639	54,3553	1038,38	cooling	6,452	34,8254	10,1502	344,92	
6,454	41,9083	6,4384	495,82	cooling	6,454	38,8933	18,5117	284,9		6,454	127,4381	52,5677	1046,58	cooling	6,454	33,0566	8,4813	343,53	
6,456	42,2095	12,242	418,9	cooling	6,456	38,6654	20,6101	252,39		6,456	123,5078	47,9123	1056,71	cooling	6,456	34,4545	11,2618	324,2	
6,458	41,2434	15,7741	356,02	cooling	6,458	38,6245	20,5923	252,06	heating	6,458	136,63	40,5849	1342,57	cooling	6,458	33,2404	10,3006	320,67	
6,46	40,9167	12,4016	398,6	cooling	6,46	38,8204	20,7055	253,22	heating	6,46	134,5552	71,7529	877,88	cooling	6,46	33,7895	10,9686	319	
6,462	42,328	15,5848	373,83	cooling	6,462	38,6544	12,3206	368,11	heating	6,462	135,5862	73,3249	870,32	cooling	6,462	33,0138	10,5371	314,19	
6,464	41,1916	4,4261	513,93	cooling	6,464	38,3738	13,3031	350,45	heating	6,464	139,0133	56,3501	1155,51	cooling	6,464	34,294	10,3091	335,27	
6,466	42,0493	10,0775	446,92	cooling	6,466	37,7084	12,1635	357,08		6,466	142,872	74,0096	962,59	cooling	6,466	33,4016	11,078	312,05	
6,468	41,0485	14,0076	377,99	cooling	6,468	38,3327	17,7054	288,34		6,468	134,446	58,758	1058,01	cooling	6,468	33,8324	8,596	352,77	
6,47	40,9211	11,6662	408,94	cooling	6,47	37,3211	15,9357	298,94		6,47	144,0057	53,8896	1259,69	cooling	6,47	34,5834	14,0864	286,52	cooling
6,472	40,7163	6,5919	477,01	cooling	6,472	37,8452	14,0268	332,95		6,472	140,6286	72,3732	954,11	cooling	6,472	34,3989	10,794	329,96	
6,474	40,7681	7,8908	459,58	cooling	6,474	38,1672	15,3957	318,31		6,474	138,7121	62,8894	1059,89	cooling	6,474	34,4292	15,4016	265,98	cooling
6,476	41,0492	5,1409	501,95	cooling	6,476	38,8127	19,9359	263,87		6,476	137,173	45,181	1285,91	cooling	6,476	34,124	12,2276	306,08	
6,478	41,0066	15,7123	353,58	cooling	6,478	38,2385	22,1869	224,38		6,478	137,1593	67,63	971,92	cooling	6,478	35,1597	13,3747	304,52	
6,48	39,5355	12,8057	373,64	cooling	6,48	39,0952	23,0538	224,23		6,48	127,0612	56,2624	989,66	cooling	6,48	34,0465	7,6489	369	
6,482	39,5438	4,583	488,7	cooling	6,482	37,955	20,665	241,69	heating	6,482	130,8499	60,4296	984,37	cooling	6,482	34,8194	11,3258	328,41	cooling
6,484	40,8734	6,2847	483,5	cooling	6,484	38,1014	17,1593	292,74	heating	6,484	129,9532	64,8682	909,79	cooling	6,484	34,0099	8,1296	361,77	cooling
6,486	39,305	2,13	519,65	cooling	6,486	37,914	19,9396	251,26	heating	6,486	129,7737	51,4151	1095,34	cooling	6,486	34,457	12,027	313,54	cooling
6,488	40,3811	6,05	479,9	cooling	6,488	38,0436	14,6504	327	heating	6,488	132,9296	46,2465	1211,7	cooling	6,488	32,9656	10,1023	319,59	cooling
6,49	40,3686	10,859	412,5	cooling	6,49	37,6332	13,8825	332	heating	6,49	137,01	69,7559	940,11	cooling	6,49	34,6298	9,2873	354,25	cooling
6,492	39,6752	6,0549	469,96	cooling	6,492	37,8237	12,8818	348,65	heating	6,492	132,9694	59,4407	1027,82	cooling	6,492	33,7076	11,1256	315,66	cooling
6,494	40,0044	1,4679	538,68	cooling	6,494	38,3654	14,7812	329,67		6,494	131,5037	70,2245	856,59	cooling	6,494	33,7538	10,3956	326,51	cooling
6,496	41,4547	13,6211	389,07	cooling	6,496	38,2128	12,9547	353,07		6,496	131,4854	65,5738	921,35	cooling	6,496	34,2424	14,2112	280,01	cooling
6,498	41,1672	6,2964	487,44	cooling	6,498	38,2571	16,7869	300,12		6,498	128,4341	54,7757	1029,64	cooling	6,498	34,8331	12,015	318,96	cooling
6,5	40,4415	11,4531	405,22	cooling	6,5	37,9564	15,4998	313,91		6,5	126,8276	54,2742	1014,19	cooling	6,5	33,933	12,1989	303,81	cooling
6,502	40,0151	12,1778	389,12	cooling	6,502	39,019	20,0159	265,64		6,502	126,5487	62,3316	897,66	cooling	6,502	34,3535	10,4255	334,48	cooling
6,504	39,6712	7,4227	450,79	cooling	6,504	37,6554	16,7928	291,63		6,504	125,2904	47,7886	1083,36	cooling	6,504	35,2392	16,0868	267,72	cooling
6,506	40,0291	6,0231	475,35	cooling	6,506	38,6956	16,3844	311,88		6,506	121,5961	67,7672	752,45	cooling	6,506	34,523	12,4318	308,8	cooling
6,508	39,3763	10,1798	408,12	cooling	6,508	38,0091	17,3121	289,31		6,508	120,9673	69,0723	725,42	cooling	6,508	34,6376	19,2195	215,52	cooling
6,51	40,5344	0,774	555,79	cooling	6,51	38,451	19,3291	267,3		6,51	120,1081	51,2045	963,17	cooling	6,51	33,818	13,9838	277,25	
6,512	40,7524	10,6993	420,1	cooling	6,512	37,4089	18,1153	269,7		6,512	121,6686	46,2917	1053,66	cooling	6,512	34,7619	14,9499	276,94	
6,514	40,0322	16,4923	329,05	cooling	6,514	38,5358	18,3376	282,34		6,514	125,8643	56,5057	969,53	cooling	6,514	33,0383	10,5058	314,97	
6,516	39,6609	9,01	428,45	cooling	6,516	37,2464	14,7465	314,51		6,516	132,3228	49,0867	1163,52	cooling	6,516	34,261	10,2843	335,16	
6,518	42,0075	12,9211	406,58	cooling	6,518	37,8677	15,0807	318,53		6,518	131,3942	67,8467	888,3	cooling	6,518	33,7235	6,4319	381,5	
6,52	39,4461	4,9466	482,25	cooling	6,52	37,8676	20,1105	248,22		6,52	131,1029	69,5424	860,52	cooling	6,52	33,96	7,213	373,88	cooling
6,522	40,9623	6,4047	483,06	cooling	6,522	38,4914	18,0636	285,55		6,522	131,0219	49,8802	1134,24	cooling	6,522	32,8948	5,5822	381,79	cooling
6,524	40,9553	16,3496	343,95	cooling	6,524	38,0615	17,3478	289,55		6,524	131,1025	42,7033	1235,69	cooling	6,524	34,4679	9,54	348,45	cooling
6,526	39,7948	12,9027	375,91	cooling	6,526	38,1442	16,5366	302,04		6,526	128,7342	39,7607	1243,72	cooling	6,526	33,2308	10,2129	321,75	cooling

6,528	39,5731	1,1866	536,59	cooling	6,528	38,8209	18,2764	287,18		6,528	131,8812	43,9312	1229,41	cooling	6,528	33,6987	9,4096	339,53	cooling
6,53	41,5628	10,8278	429,63	cooling	6,53	38,5301	16,9222	302,05		6,53	130,9986	68,369	875,47	cooling	6,53	33,8767	14,9286	264,87	cooling
6,532	39,7264	2,2504	523,86	cooling	6,532	38,3393	19,5435	262,74		6,532	129,058	63,443	917,2	cooling	6,532	34,2641	12,0765	310,15	cooling
6,534	39,6075	10,489	407,03	cooling	6,534	37,4045	15,6821	303,65		6,534	129,1332	44,3026	1185,81	cooling	6,534	33,3866	11,6132	304,36	cooling
6,536	38,7879	11,5897	380,19	cooling	6,536	38,2545	18,0604	282,28		6,536	133,0674	55,336	1086,57	cooling	6,536	33,6475	7,0189	372,23	cooling
6,538	38,3334	0,5053	528,78	cooling	6,538	36,1995	10,5144	359,04		6,538	127,1675	45,5545	1140,83	cooling	6,538	34,2767	7,1335	379,42	cooling
6,54	39,3226	1,1978	532,93	cooling	6,54	37,2736	11,5609	359,42		6,54	131,6989	49,6077	1147,51	cooling	6,54	33,7945	4,9324	403,45	cooling
6,542	39,0198	9,9895	405,8	cooling	6,542	36,3581	13,98	312,81		6,542	130,4177	62,0665	955,45	cooling	6,542	33,8126	13,1084	289,41	cooling
6,544	40,9428	6,4241	482,52	cooling	6,544	36,7535	15,2183	301,03		6,544	130,195	51,2593	1103,4	cooling	6,544	33,3301	11,5548	304,39	cooling
6,546	40,4349	15,7238	345,43	cooling	6,546	36,0875	14,4344	302,68		6,546	129,2686	38,9511	1262,5	cooling	6,546	34,1126	15,11	265,63	cooling
6,548	39,7461	12,8315	376,23	cooling	6,548	37,3257	15,5223	304,78		6,548	135,656	63,2828	1011,67	cooling	6,548	32,3724	10,1975	309,97	cooling
6,55	39,5675	1,0703	538,13	cooling	6,55	36,738	11,3983	354,21		6,55	130,2375	48,6169	1140,94	cooling	6,55	33,8376	10,8404	321,47	cooling
6,552	41,6255	10,4456	435,85	cooling	6,552	37,5351	10,9979	370,95		6,552	132,2916	52,1336	1120,49	cooling	6,552	33,059	8,9062	337,62	cooling
6,554	38,6228	6,2216	452,92	cooling	6,554	38,1678	15,0756	322,79		6,554	130,0592	60,2155	976,31	cooling	6,554	33,3327	10,4878	319,34	cooling
6,556	40,648	9,4535	436,05	cooling	6,556	38,3549	12,8846	356,04		6,556	131,1827	57,309	1032,64	cooling	6,556	32,3958	9,6961	317,31	cooling
6,558	40,0162	12,8937	379,13	cooling	6,558	38,592	17,5352	294,34		6,558	129,5702	47,122	1152,5	cooling	6,558	33,7898	10,813	321,18	cooling
6,56	40,0494	9,3664	428,9	cooling	6,56	37,8631	16,9251	292,68		6,56	137,4474	67,9777	971,08	cooling	6,56	32,524	10,3513	309,94	cooling
6,562	40,2038	4,463	499,6	cooling	6,562	38,7689	19,7804	265,43		6,562	138,7926	52,4208	1207,35	cooling	6,562	32,7788	10,0186	318,15	cooling
6,564	42,6833	16,0616	372,13	cooling	6,564	37,6442	18,409	268,88		6,564	136,2826	63,9138	1011,61	cooling	6,564	32,9405	12,3556	287,75	cooling
6,566	42,114	9,5271	455,52	cooling	6,566	37,5519	19,7174	249,3		6,566	135,3427	63,5664	1003,33	cooling	6,566	33,2663	8,94	340,05	cooling
6,568	42,4713	16,1567	367,84	cooling	6,568	36,7775	14,4981	311,43		6,568	138,3713	55,8954	1152,89	cooling	6,568	32,6012	10,3485	311,06	cooling
6,57	41,7273	12,5406	407,99	cooling	6,57	37,5039	14,6208	319,87		6,57	138,5652	42,7581	1339,24	cooling	6,57	32,6791	8,0465	344,33	cooling
6,572	41,9611	5,1485	514,59	cooling	6,572	37,0272	14,1112	320,33		6,572	143,8679	60,7397	1162,01	cooling	6,572	33,6884	13,2606	285,55	cooling
6,574	42,7905	9,2848	468,36	cooling	6,574	37,465	16,4559	293,68		6,574	149,0342	64,351	1183,75	cooling	6,574	32,8875	10,7871	308,93	cooling
6,576	42,7012	16,4344	367,17	cooling	6,576	37,0768	15,3873	303,19		6,576	145,225	82,1174	882,15	cooling	6,576	33,0503	15,7841	241,36	cooling
6,578	44,288	7,2483	517,76	cooling	6,578	36,7859	14,8465	306,68		6,578	145,0815	80,0593	908,91	cooling	6,578	32,885	13,4614	271,51	cooling
6,58	43,7079	4,117	553,42	cooling	6,58	36,7761	15,7944	293,29		6,58	144,594	63,263	1136,89	cooling	6,58	33,4861	14,8935	259,9	cooling
6,582	43,5321	2,6281	571,78	cooling	6,582	36,8438	13,6	324,91		6,582	143,063	48,6178	1320,2	cooling	6,582	32,2448	11,6857	287,39	cooling
6,584	43,038	4,7382	535,37	cooling	6,584	36,633	16,8454	276,6		6,584	143,331	70,5088	1017,95	cooling	6,584	33,5281	13,5641	279,07	cooling
6,586	44,2194	20,6303	329,74	cooling	6,586	36,0124	16,036	279,24		6,586	145,4805	75,1024	983,78	cooling	6,586	32,505	9,0668	327,63	
6,588	41,328	15,5894	359,79	cooling	6,588	36,7688	18,9136	249,59		6,588	140,3128	87,7601	734,61	cooling	6,588	32,7599	9,0325	331,67	cooling
6,59	42,4734	10,3732	448,71	cooling	6,59	36,7162	18,0576	260,82		6,59	140,0168	83,0895	795,76	cooling	6,59	31,4602	7,2202	338,84	cooling
6,592	41,741	11,3732	424,5	cooling	6,592	36,7458	20,6051	225,62		6,592	139,1851	56,7411	1152,44	cooling	6,592	32,843	9,6279	324,51	cooling
6,594	41,0309	11,1979	417,02	cooling	6,594	36,9725	16,2187	290,11		6,594	144,1029	51,2605	1297,8	cooling	6,594	31,6326	10,9674	288,87	cooling
6,596	40,8239	7,2649	469,11	cooling	6,596	37,8264	19,7846	252,2		6,596	144,1881	68,693	1055,31	cooling	6,596	31,9054	10,8299	294,6	cooling
6,598	42,8209	17,073	359,92	cooling	6,598	36,915	16,3576	287,36		6,598	147,9375	65,1388	1157,4	cooling	6,598	32,4267	12,7466	275,1	cooling
6,6	41,5897	6,0593	496,66	cooling	6,6	37,4431	14,9137	314,93		6,6	146,3505	83,7149	875,55	cooling	6,6	33,2851	10,6879	315,88	
6,602	41,6054	12,0952	412,51	cooling	6,602	37,2979	14,4891	318,83		6,602	146,8507	90,5868	786,49	cooling	6,602	32,5746	13,0335	273,16	cooling
6,604	41,214	18,9992	310,53	cooling	6,604	37,0066	13,3291	330,98		6,604	144,2169	63,2712	1131,5	cooling	6,604	32,7533	10,9944	304,16	
6,606	40,5197	15,5565	348,95	cooling	6,606	36,8543	13,092	332,16		6,606	147,9177	62,7802	1190,09	cooling	6,606	33,4957	12,7681	289,74	
6,608	40,9931	12,0563	404,49	cooling	6,608	37,9045	16,4542	299,84		6,608	147,9816	77,9027	979,6	cooling	6,608	32,5312	7,3694	351,73	
6,61	40,186	12,2434	390,6	cooling	6,61	36,7075	13,1854	328,8		6,61	147,6896	67,6938	1118,22	cooling	6,61	32,6422	11,1204	300,84	
6,612	41,4926	7,0689	481,19	cooling	6,612	37,2392	9,6744	385,32		6,612	144,1171	78,6499	915,13	cooling	6,612	32,2322	10,3359	306,08	
6,614	40,5964	15,8179	346,37	cooling	6,614	37,787	16,0756	303,49		6,614	142,7064	74,8832	948,07	cooling	6,614	32,8416	15,7175	239,37	
6,616	39,7861	15,0914	345,2	cooling	6,616	37,9142	14,6749	324,85		6,616	140,3085	48,5976	1281,98	cooling	6,616	31,401	13,9524	243,91	
6,618	39,688	5,0933	483,58	cooling	6,618	37,5438	16,8115	289,81		6,618	143,3855	59,4005	1173,99	cooling	6,618	32,7674	14,7745	251,51	
6,62	41,1404	9,9314	436,25	cooling	6,62	37,6918	15,3049	312,94		6,62	143,8282	60,338	1167,07	cooling	6,62	32,2374	10,7756	300,01	
6,622	38,8551	4,6907	477,57	cooling	6,622	38,1314	13,3928	345,81		6,622	142,6195	50,1874	1292,06	cooling	6,622	32,5014	10,5871	306,33	
6,624	39,7022	6,7259	460,96	cooling	6,624	38,1745	13,5857	343,72		6,624	141,7906	60,7234	1133,2	cooling	6,624	32,2963	11,5608	289,85	
6,626	39,3037	9,4328	417,55	cooling	6,626	37,6864	17,1344	287,29		6,626	139,9222	50,9761	1243,33	cooling	6,626	33,6133	13,0541	287,39	
6,628	38,8928	6,8662	447,68	cooling	6,628	37,9474	14,0497	334,05		6,628	142,0513	46,9591	1329,25	cooling	6,628	32,5078	14,6127	250,15	
6,63	38,9389	5,9814	460,7	cooling	6,63	38,8351	19,3552	272,3		6,63	149,8851	78,1519	1002,72	cooling	6,63	32,3878	12,8439	273,19	
6,632	40,0701	17,1774	320,01	cooling	6,632	37,3623	16,4516	292,3		6,632	152,5298	65,7599	1212,92	cooling	6,632	33,156	14,1065	266,28	
6,634	38,9207	7,2506	442,7	cooling	6,634	38,1256	12,7972	354,05		6,634	153,2878	72,605	1127,82	cooling	6,634	33,2101	12,2732	292,67	
6,636	38,7142	7,8162	431,91	cooling	6,636	37,7091	14,1031	329,98		6,636	153,6428	73,7009	1117,47	cooling	6,636	32,6504	15,6232	238,01	
6,638	38,3275	8,3366	419,23	cooling	6,638	37,3107	13,9656	326,33		6,638	153,3912	51,3112	1426,93	cooling	6,638	32,734	14,2846	257,9	

6,64	38,0751	4,7721	465,53	cooling	6,64	36,5278	16,0394	286,4		6,64	153,9131	49,6229	1457,82	cooling	6,64	33,5205	17,2795	227,03	
6,642	38,4851	9,2035	409,31	cooling	6,642	37,5468	21,3997	225,71		6,642	161,4053	78,5676	1157,95	cooling	6,642	32,2456	10,6505	301,87	heating
6,644	37,9247	15,3491	315,57	cooling	6,644	36,0909	18,5693	244,93		6,644	165,3321	61,2714	1454,61	cooling	6,644	32,7004	12,28	285,45	heating
6,646	38,6676	8,9986	414,73	cooling	6,646	36,9997	16,188	290,92		6,646	160,0813	77,9951	1147,44	cooling	6,646	32,3508	10,5464	304,79	heating
6,648	38,5069	11,1791	382	cooling	6,648	37,1736	18,9962	254,09		6,648	159,0228	73,0529	1201,73	cooling	6,648	32,6145	12,1137	286,57	heating
6,65	37,6416	11,6763	362,96	cooling	6,65	38,3064	17,0784	296,74		6,65	159,6335	55,8816	1450,3	cooling	6,65	31,647	12,2809	270,71	heating
6,652	36,9661	5,0788	445,74	cooling	6,652	37,655	19,309	256,45		6,652	163,4017	64,4351	1383,41	cooling	6,652	33,0012	15,7708	240,86	heating
6,654	38,0017	10,9994	377,45	cooling	6,654	37,9045	17,9718	278,63		6,654	162,3955	72,695	1253,88	cooling	6,654	32,1663	12,899	269,33	heating
6,656	35,9612	7,2396	401,49	cooling	6,656	38,0084	15,3152	317,22		6,656	170,7521	78,1832	1293,98	cooling	6,656	32,604	11,7447	291,58	heating
6,658	37,0558	8,2808	402,23	cooling	6,658	37,852	13,4664	340,87		6,658	170,2943	92,7971	1083,3	cooling	6,658	31,9989	10,4204	301,64	heating
6,66	36,5855	11,9591	344,24	cooling	6,66	36,9809	12,6035	340,76		6,66	170,5316	85,3262	1191,05	cooling	6,66	33,5915	10,9878	315,97	heating
6,662	35,9783	8,13	389,28	cooling	6,662	36,7505	12,0975	344,61		6,662	171,6853	71,6987	1397,67	cooling	6,662	32,6807	11,2263	299,9	heating
6,664	35,6662	2,6154	462	cooling	6,664	36,7848	13,7376	322,17		6,664	172,4389	68,5331	1452,45	cooling	6,664	32,8977	9,5594	326,24	heating
6,666	37,7332	11,4942	366,78	cooling	6,666	35,8996	14,1101	304,58		6,666	168,277	71,1867	1357,18	cooling	6,666	33,667	12,1996	300,08	heating
6,668	36,7944	2,9364	473,28	cooling	6,668	36,609	14,696	306,31		6,668	170,0198	83,7821	1205,48	cooling	6,668	34,1735	11,2453	320,5	
6,67	37,3759	5,8235	441,06	cooling	6,67	36,2591	12,3893	333,66		6,67	168,0906	88,1719	1117,14	cooling	6,67	33,2608	14,232	265,99	
6,672	36,7169	7,9919	401,53	cooling	6,672	37,0977	12,1103	349,29		6,672	167,7666	66,0786	1421,45	cooling	6,672	33,4732	14,1498	270,11	
6,674	36,3529	4,5161	445,03	cooling	6,674	36,543	16,4969	280,21		6,674	169,7526	49,2097	1685,01	cooling	6,674	34,1946	18,7169	216,36	heating
6,676	36,9395	8,1952	401,8	cooling	6,676	37,0649	17,4405	274,32		6,676	168,8209	63,0503	1478,52	cooling	6,676	33,0855	14,7636	256,11	heating
6,678	36,5856	9,7116	375,66	cooling	6,678	36,2511	17,2956	264,97		6,678	170,6896	86,1245	1182,1	cooling	6,678	33,3259	15,9728	242,57	heating
6,68	38,0052	2,52	496,03	cooling	6,68	36,2497	15,2503	293,54		6,68	170,7452	105,5503	911,33	cooling	6,68	32,778	11,2955	300,29	heating
6,682	37,4616	10,6468	374,83	cooling	6,682	36,1991	13,6508	315,19		6,682	169,3946	95,3126	1035,56	cooling	6,682	33,257	13,096	281,82	heating
6,684	36,9539	13,5426	327,25	cooling	6,684	36,6751	14,1487	314,89		6,684	167,0943	57,752	1528,44	cooling	6,684	31,9001	13,8748	251,97	heating
6,686	36,573	6,7282	417,19	cooling	6,686	35,6895	15,6708	279,83		6,686	170,2048	50,977	1666,63	cooling	6,686	33,1858	17,3713	221,06	heating
6,688	38,968	14,9192	336,17	cooling	6,688	35,3932	12,9066	314,33		6,688	164,2574	51,4306	1577,15	cooling	6,688	32,2076	15,9303	227,53	heating
6,69	36,1687	3,8107	452,32	cooling	6,69	36,5866	18,8472	247,97		6,69	161,6352	68,9581	1295,49	cooling	6,69	32,3971	15,1075	241,68	heating
6,692	38,5712	5,7476	458,83	cooling	6,692	35,6856	18,0023	247,19		6,692	160,5277	90,8133	974,5	cooling	6,692	32,4589	14,8365	246,34	heating
6,694	36,6812	12,7748	334,18	cooling	6,694	36,2455	18,3024	250,82		6,694	160,3196	93,0315	940,59	cooling	6,694	33,8958	14,5075	271,02	heating
6,696	36,2232	7,836	396,81	cooling	6,696	35,5923	16,2894	269,83		6,696	158,0921	66,7468	1276,87	cooling	6,696	33,0259	15,6531	242,85	heating
6,698	36,5707	4,0277	454,9	cooling	6,698	35,8602	14,039	305,03		6,698	168,0723	85,0003	1161,22	cooling	6,698	32,9671	13,2155	276,1	heating
6,7	39,3383	14,4701	347,62	cooling	6,7	35,0737	13,7517	298,05		6,7	170,3005	65,4851	1465,16	cooling	6,7	34,0672	15,2702	262,75	heating
6,702	38,5086	4,5821	474,24	cooling	6,702	36,0075	19,3237	233,22		6,702	165,3864	76,6543	1240,34	cooling	6,702	33,6062	12,7048	292,17	heating
6,704	38,6801	15,2693	327,25	cooling	6,704	34,9628	18,375	231,87		6,704	163,357	82,9563	1123,88	cooling	6,704	33,2373	14,8126	257,55	heating
6,706	37,3623	13,1179	338,9	cooling	6,706	35,5228	20,4872	210,17		6,706	162,7302	67,7181	1328,13	cooling	6,706	33,2875	14,6616	260,36	heating
6,708	37,2513	0,1094	519,19	cooling	6,708	34,7271	18,6687	224,47	heating	6,708	159,4948	59,5569	1396,98	cooling	6,708	34,1984	19,1157	210,83	heating
6,71	38,6131	4,9524	470,53	cooling	6,71	35,6521	14,9588	289,26	heating	6,71	164,9997	84,4186	1126,4	cooling	6,71	33,0921	16,9565	225,55	heating
6,712	37,2184	9,5739	386,43	cooling	6,712	35,5956	17,5366	252,44	heating	6,712	179,6614	65,2344	1599,52	cooling	6,712	33,6893	20,0174	191,11	heating
6,714	39,5054	10,283	408,49	cooling	6,714	35,0888	14,6312	285,97	heating	6,714	170,2862	91,1317	1106,46	cooling	6,714	33,2615	15,7739	244,45	heating
6,716	37,8823	16,546	298,25	cooling	6,716	36,6828	16,3254	284,57	heating	6,716	169,0005	91,5844	1082,16	cooling	6,716	33,7105	16,0718	246,56	heating
6,718	37,7426	9,6095	393,26	cooling	6,718	36,5846	17,4205	267,89	heating	6,718	169,7476	63,6463	1483,14	cooling	6,718	32,6115	14,817	248,74	heating
6,72	38,3109	4,2991	475,44	cooling	6,72	36,2143	16,9709	269	heating	6,72	171,6922	78,3454	1304,85	cooling	6,72	33,8037	16,0715	247,87	heating
6,722	40,6228	18,1069	314,74	cooling	6,722	36,3057	14,3615	306,75	heating	6,722	160,5798	73,1197	1222,56	cooling	6,722	33,2728	14,9649	255,92	heating
6,724	38,2597	9,2828	405,05	cooling	6,724	37,5423	18,4349	267,09	heating	6,724	173,7828	73,1752	1406,34	cooling	6,724	33,3574	14,7355	260,31	heating
6,726	40,3061	13,9119	368,95	cooling	6,726	36,4623	12,9829	328,21	heating	6,726	170,1358	99,4584	987,96	cooling	6,726	33,6043	15,5335	252,6	heating
6,728	38,7656	14,1656	343,87	cooling	6,728	37,3933	16,147	296,99	heating	6,728	169,045	86,421	1154,96	cooling	6,728	34,4927	15,5809	264,36	heating
6,73	38,948	7,4371	440,48	cooling	6,73	36,8719	13,843	321,91	heating	6,73	166,6989	48,0577	1658,43	cooling	6,73	33,5421	15,4715	252,6	heating
6,732	39,0378	6,682	452,29	cooling	6,732	37,2404	13,9483	325,59	heating	6,732	174,4295	77,9344	1348,86	cooling	6,732	33,56	11,5875	307,14	heating
6,734	39,6038	11,1742	397,4	cooling	6,734	36,7261	16,4619	283,26	heating	6,734	161,3957	52,8506	1517,3	cooling	6,734	34,5021	12,5653	306,64	heating
6,736	39,836	5,4364	480,86	cooling	6,736	36,6155	15,2027	299,32	heating	6,736	160,5934	64,6237	1341,51	cooling	6,736	34,1301	9,3913	345,81	heating
6,738	39,5555	16,5464	321,63	cooling	6,738	37,2556	15,8819	298,77	heating	6,738	158,9435	79,4203	1111,62	cooling	6,738	33,9614	12,2055	304,11	heating
6,74	38,982	14,0233	348,89	cooling	6,74	35,8379	13,8746	307,02	heating	6,74	159,1418	69,1785	1257,55	cooling	6,74	33,9464	12,467	300,25	heating
6,742	39,0412	4,2216	486,73	cooling	6,742	36,2383	10,7518	356,26	heating	6,742	161,3864	64,6813	1351,79	cooling	6,742	34,4052	15,1742	268,82	heating
6,744	40,4517	9,3476	434,79	cooling	6,744	36,517	10,4434	364,47		6,744	169,1048	99,8716	967,78	cooling	6,744	33,4637	14,1728	269,66	heating
6,746	37,9079	5,0706	459,02	cooling	6,746	36,3888	15,3958	293,45		6,746	171,8068	71,8064	1397,86	cooling	6,746	34,198	15,6699	259	heating
6,748	40,0854	10,4631	414,08	cooling	6,748	35,7451	12,7995	320,75		6,748	166,5261	86,8607	1113,6	cooling	6,748	33,8705	10,9929	319,8	heating
6,75	38,0636	14,0675	335,43	cooling	6,75	37,7706	20,1797	245,89		6,75	165,4717	93,4078	1007,35	cooling	6,75	33,8706	9,6134	339,08	heating

6,752	38,4381	8,452	419,16	cooling	6,752	37,2739	16,5833	289,22		6,752	163,6354	69,9541	1309,53	cooling	6,752	33,4619	9,2269	338,77	heating
6,754	38,2117	2,5817	498,05	cooling	6,754	37,2033	19,6622	245,2		6,754	165,2366	74,8023	1264,14	cooling	6,754	34,6667	11,0136	330,64	heating
6,756	40,0649	14,8884	351,93	cooling	6,756	36,9043	19,6889	240,64		6,756	165,0139	98,3125	932,39	cooling	6,756	34,0524	13,7135	284,31	heating
6,758	38,6575	9,1145	412,97	cooling	6,758	37,159	17,1866	279,18	heating	6,758	177,0848	80,3867	1351,7	cooling	6,758	33,7235	13,8684	277,54	heating
6,76	39,9109	11,4493	397,85	cooling	6,76	36,9685	15,3961	301,55	heating	6,76	169,4104	83,9488	1194,63	cooling	6,76	34,2974	15,7812	258,83	heating
6,762	38,9916	7,3687	442,04	cooling	6,762	36,0131	18,2105	248,85	heating	6,762	170,1248	75,5861	1321,51	cooling	6,762	35,1878	14,2558	292,6	heating
6,764	39,187	7,629	441,14	cooling	6,764	36,2954	14,6102	303,13	heating	6,764	170,6081	53,5774	1635,92	cooling	6,764	34,135	13,3692	290,28	heating
6,766	38,8228	10,982	389,17	cooling	6,766	35,7235	14,9852	289,89	heating	6,766	172,616	81,4143	1274,86	cooling	6,766	34,2632	10,1501	337,07	heating
6,768	38,7097	13,3022	355,16	cooling	6,768	35,8043	16,3212	272,34	heating	6,768	167,4325	85,512	1145,13	cooling	6,768	35,2689	13,0343	310,81	heating
6,77	39,4166	9,0929	423,88	cooling	6,77	35,9481	12,3316	330,12	heating	6,77	179,7682	78,1204	1420,89	cooling	6,77	34,8347	10,9555	333,8	heating
6,772	38,9345	14,3422	343,76	cooling	6,772	37,3732	16,9211	285,89	heating	6,772	174,0668	92,1024	1145,74	cooling	6,772	34,8047	16,2611	259,21	heating
6,774	38,6169	13,5554	350,32	cooling	6,774	36,1854	13,6613	314,85	heating	6,774	172,5823	89,6974	1158,61	cooling	6,774	34,7595	15,0664	275,28	heating
6,776	38,3216	7,4557	431,46	cooling	6,776	37,8544	15,6348	310,6	heating	6,776	172,7833	75,3149	1362,46	cooling	6,776	35,4876	16,1629	270,13	heating
6,778	38,9163	5,3358	469,41	cooling	6,778	37,9766	16,7499	296,72		6,778	173,9466	98,9268	1048,67	cooling	6,778	34,3295	13,0598	297,32	heating
6,78	38,1418	1,3942	513,68	cooling	6,78	38,0528	19,913	253,57		6,78	162,8418	73,425	1249,91	cooling	6,78	34,8112	14,5119	283,75	heating
6,782	39,0529	6,9216	449,15	cooling	6,782	37,7772	15,7791	307,5	heating	6,782	168,3756	63,6889	1463,36	cooling	6,782	34,6564	12,675	307,27	heating
6,784	38,4881	13,0909	355,01	cooling	6,784	38,6839	19,2421	271,77		6,784	168,1545	86,1012	1146,98	cooling	6,784	34,5794	13,0218	301,34	heating
6,786	38,1995	13,5137	345,07	cooling	6,786	37,6538	14,3711	325,46	heating	6,786	164,488	79,3797	1189,69	cooling	6,786	33,8968	12,1491	304	heating
6,788	37,8766	4,1509	471,44	cooling	6,788	37,3185	15,4666	305,46		6,788	164,3598	61,6382	1435,9	cooling	6,788	35,1636	13,2802	305,9	heating
6,79	39,374	6,1898	463,87	cooling	6,79	37,0199	15,2813	303,87		6,79	170,6194	76,9233	1309,73	cooling	6,79	34,3035	14,369	278,65	heating
6,792	38,7372	2,3514	508,62	cooling	6,792	36,8781	14,6666	310,48		6,792	164,2583	42,1213	1707,29	cooling	6,792	34,19	11,8501	312,28	heating
6,794	38,8731	9,1987	414,8	cooling	6,794	36,7087	15,9608	290,03		6,794	161,8714	55,8228	1482,4	cooling	6,794	34,7001	13,6133	294,76	heating
6,796	38,7273	12,1144	372,01	cooling	6,796	36,9923	19,0529	250,77		6,796	161,7467	86,6029	1050,4	cooling	6,796	35,4481	13,3235	309,27	heating
6,798	37,8649	7,4967	424,5	cooling	6,798	36,9835	17,6175	270,71		6,798	159,5446	83,2048	1067,12	cooling	6,798	34,5888	14,9858	274,02	heating
6,8	38,174	0,8053	522,36	cooling	6,8	36,9135	12,9479	335	heating	6,8	158,7324	88,116	987,11	cooling	6,8	34,788	13,7136	294,59	heating
6,802	38,717	6,498	450,37	cooling	6,802	37,597	15,5415	308,3		6,802	163,5958	109,7068	753,29	cooling	6,802	35,4419	13,2815	309,77	heating
6,804	39,7736	10,2028	413,36	cooling	6,804	38,1704	15,6156	315,28		6,804	162,0238	69,1054	1298,86	cooling	6,804	35,2353	9,2439	363,32	heating
6,806	38,7128	9,353	410,41	cooling	6,806	37,7745	20,6119	239,91		6,806	158,7627	68,7479	1258,27	cooling	6,806	34,6786	12,4705	310,44	
6,808	38,7036	6,5406	449,59	cooling	6,808	37,2897	19,3749	250,42		6,808	158,4034	73,1344	1191,93	cooling	6,808	34,447	10,9951	327,82	
6,81	38,4712	2,9345	496,75	cooling	6,81	38,044	18,935	267,12	heating	6,81	158,4075	63,8313	1322,03	cooling	6,81	35,0735	14,8195	283,12	
6,812	39,0189	6,9543	448,21	cooling	6,812	38,1984	14,8798	325,96	heating	6,812	157,1179	72,2837	1185,86	cooling	6,812	33,9875	13,3692	288,21	
6,814	38,0512	10,3659	387	cooling	6,814	37,674	17,118	287,34	heating	6,814	162,8035	90,2017	1014,87	cooling	6,814	34,6114	12,4272	310,1	
6,816	38,3049	14,022	339,44	cooling	6,816	37,3693	15,8758	300,45	heating	6,816	173,5819	61,5543	1565,98	cooling	6,816	34,6998	12,0952	315,98	
6,818	37,7398	10,3708	382,58	cooling	6,818	37,8136	16,553	297,19		6,818	165,7515	64,1847	1419,75	cooling	6,818	34,6327	12,5325	308,93	
6,82	37,8383	10,1767	386,67	cooling	6,82	37,1691	14,5979	315,51		6,82	164,5322	64,5063	1398,21	cooling	6,82	34,0198	10,7919	324,69	
6,822	38,3074	8,5711	415,67	cooling	6,822	37,1095	13,601	328,61		6,822	166,0357	63,2036	1437,44	cooling	6,822	35,4956	13,1319	312,61	
6,824	39,3019	13,24	364,31	cooling	6,824	37,4222	15,8405	301,68		6,824	163,2488	75,0002	1233,58	cooling	6,824	34,4097	11,4594	320,81	
6,826	39,4432	9,352	420,63	cooling	6,826	37,5058	17,0242	286,3		6,826	163,3636	77,6495	1198,16	cooling	6,826	34,6172	9,6661	348,78	
6,828	39,9077	14,9882	348,34	cooling	6,828	36,9234	16,8709	280,3		6,828	181,0007	59,3616	1700,33	cooling	6,828	34,4809	12,1962	311,51	
6,83	40,3042	11,1329	407,77	cooling	6,83	37,6991	15,4698	310,73		6,83	174,4013	90,5126	1172,64	cooling	6,83	34,8745	11,0358	333,23	
6,832	39,425	9,905	412,65	cooling	6,832	37,3902	16,0023	298,97		6,832	173,1464	100,723	1012,37	cooling	6,832	34,1157	12,5657	301,24	
6,834	39,5759	9,4701	420,83	cooling	6,834	37,0984	11,0833	363,65		6,834	171,4998	81,866	1252,95	cooling	6,834	34,3354	12,3533	307,28	
6,836	38,9347	10,7093	394,55	cooling	6,836	37,5884	15,3837	310,39		6,836	173,6691	85,8288	1227,88	cooling	6,836	35,0059	12,6655	312,29	
6,838	40,0827	14,0663	363,67	cooling	6,838	37,6681	17,084	287,74		6,838	158,6578	45,245	1585,34	cooling	6,838	34,7361	12,3819	312,48	
6,84	38,6598	12,2609	369,02	cooling	6,84	37,1247	17,6339	272,45		6,84	163,9198	49,755	1595,85	cooling	6,84	34,4202	12,0619	312,54	
6,842	39,8229	8,6201	436,17	cooling	6,842	37,0046	13,4975	328,59		6,842	161,5683	83,6768	1088,81	cooling	6,842	34,6641	10,2231	341,65	
6,844	40,027	9,6684	424,37	cooling	6,844	37,7715	12,2603	356,61		6,844	162,9992	91,7207	996,37	cooling	6,844	35,0264	11,7771	324,99	
6,846	40,9973	14,437	371,27	cooling	6,846	37,602	7,7763	416,92		6,846	162,3587	75,9477	1207,9	cooling	6,846	33,9385	8,5966	354,24	
6,848	41,3814	12,5771	402,64	cooling	6,848	38,3812	14,3841	335,44		6,848	169,6757	89,5049	1120,67	cooling	6,848	35,1888	11,1413	336,15	
6,85	42,5509	17,87	345	cooling	6,85	37,8646	16,4633	299,16		6,85	162,8268	60,6211	1428,68	cooling	6,85	34,5818	12,2117	312,7	cooling
6,852	41,945	7,531	481,06	cooling	6,852	38,5385	14,8745	330,79		6,852	167,0028	66,7686	1401,13	cooling	6,852	34,642	10,5789	336,37	cooling
6,854	42,0664	7,901	477,58	cooling	6,854	38,2018	15,4165	318,5		6,854	166,183	82,3885	1171,32	cooling	6,854	34,5072	11,1423	326,61	cooling
6,856	41,913	11,638	423,2	cooling	6,856	38,2123	14,9448	325,24		6,856	167,6726	84,5596	1161,8	cooling	6,856	35,2837	11,159	337,23	cooling
6,858	41,1019	16,0693	349,92	cooling	6,858	37,7934	13,8428	334,79		6,858	167,4051	81,7606	1197,18	cooling	6,858	34,6425	9,4668	351,92	cooling
6,86	41,4913	12,4239	406,32	cooling	6,86	37,5413	17,5246	279,8		6,86	172,5985	97,4247	1050,82	cooling	6,86	34,6247	9,7257	348,05	cooling
6,862	40,6418	9,7663	431,59	cooling	6,862	36,8098	18,9106	250,2		6,862	169,4301	78,1142	1276,46	cooling	6,862	34,147	7,2743	375,64	cooling

6,864	42,0881	6,1803	501,94	cooling	6,864	37,097	15,9112	296,15		6,864	171,015	93,2731	1086,72	cooling	6,864	35,8848	6,8123	406,39	cooling
6,866	41,3312	15,9941	354,17	cooling	6,866	37,5668	21,8841	219,22		6,866	168,9819	89,0839	1116,86	cooling	6,866	34,7101	8,448	367,11	cooling
6,868	41,3241	19,4511	305,75	cooling	6,868	36,534	14,5784	306,91		6,868	170,1633	76,4873	1309,45	cooling	6,868	34,4286	6,4581	390,99	cooling
6,87	40,4774	14,9565	356,74	cooling	6,87	38,7416	17,9254	290,98		6,87	169,9082	77,9344	1285,66	cooling	6,87	35,8025	13,0925	317,45	cooling
6,872	41,2908	13,7585	384,86	cooling	6,872	38,0493	18,0174	280,02		6,872	168,1408	74,2579	1312,34	cooling	6,872	35,5587	11,159	341,07	cooling
6,874	38,6329	5,8642	458,06	cooling	6,874	38,002	15,8873	309,13		6,874	173,1151	65,7599	1500,67	cooling	6,874	34,7336	12,2092	314,86	cooling
6,876	39,8696	7,9949	445,56	cooling	6,876	38,0571	12,2927	360,15		6,876	171,0343	84,211	1213,66	cooling	6,876	34,7194	9,9413	346,36	cooling
6,878	38,6019	14,8928	331,42	cooling	6,878	38,9521	14,7945	337,69		6,878	168,4858	68,0315	1404,2	cooling	6,878	35,5356	11,1734	340,55	cooling
6,88	38,5979	16,7601	305,26	cooling	6,88	37,6626	8,8817	402,31		6,88	166,9301	58,1165	1521,05	cooling	6,88	34,4189	6,4921	390,38	cooling
6,882	38,3694	7,9748	424,87	cooling	6,882	38,4863	17,5835	292,19		6,882	164,5462	59,9194	1462,53	cooling	6,882	34,9234	12,0531	319,69	cooling
6,884	40,3553	12,4637	389,88	cooling	6,884	37,2161	19,5921	246,36		6,884	161,7276	56,1568	1475,72	cooling	6,884	34,4864	10,4161	336,47	cooling
6,886	39,7574	9,0637	429,05	cooling	6,886	37,1788	13,6853	328,4		6,886	169,831	82,1266	1225,98	cooling	6,886	35,0357	13,0558	307,25	cooling
6,888	38,9668	17,4385	300,93	cooling	6,888	37,7747	16,4358	298,29		6,888	166,9072	103,6045	884,88	cooling	6,888	33,6989	11,8708	305,13	cooling
6,89	38,6787	15,8048	319,74	cooling	6,89	37,6785	14,2622	327,33		6,89	170,0094	89,3577	1127,39	cooling	6,89	34,8964	12,7638	309,38	cooling
6,892	38,3389	13,0324	353,75	cooling	6,892	38,4954	13,9575	343		6,892	171,2914	73,0953	1372,64	cooling	6,892	34,2925	14,3511	278,75	cooling
6,894	37,5745	5,0428	454,74	cooling	6,894	38,0832	18,4007	275,13		6,894	173,623	74,6956	1382,86	cooling	6,894	33,8651	13,6795	282,16	cooling
6,896	38,3982	11,6539	373,85	cooling	6,896	38,1688	19,1466	265,9		6,896	169,4103	72,6098	1353,13	cooling	6,896	34,4838	13,7986	289,15	cooling
6,898	39,2507	10,411	403,14	cooling	6,898	38,4184	13,9913	341,45		6,898	177,3597	88,1929	1246,42	cooling	6,898	35,7069	12,0404	330,82	cooling
6,9	37,4575	11,4671	363,31	cooling	6,9	39,3623	20,9404	257,51		6,9	177,0734	89,0198	1230,86	cooling	6,9	34,4486	10,5781	333,67	cooling
6,902	37,7014	7,1744	426,72	cooling	6,902	37,7252	12,6814	350,08		6,902	177,4616	74,5957	1437,91	cooling	6,902	34,4075	7,5534	375,38	cooling
6,904	37,6058	2,4077	492,02	cooling	6,904	39,7404	17,5349	310,4		6,904	171,6443	66,5056	1469,68	cooling	6,904	35,415	12,1811	324,78	cooling
6,906	38,9799	6,0853	459,82	cooling	6,906	38,4088	17,2912	295,19		6,906	175,9013	89,9775	1201,09	cooling	6,906	34,4331	9,763	344,85	cooling
6,908	37,0366	9,9303	378,9	cooling	6,908	38,1024	16,2959	304,82		6,908	171,5531	89,2963	1149,83	cooling	6,908	34,1638	14,1175	280,22	cooling
6,91	37,5492	11,7927	360,04	cooling	6,91	38,05	15,2883	318,17		6,91	172,2513	98,9255	1024,99	cooling	6,91	33,9611	12,9777	293,32	cooling
6,912	36,6294	8,0329	399,74	cooling	6,912	39,1565	20,5104	260,64		6,912	171,838	94,6944	1078,35	cooling	6,912	34,5908	13,9414	288,65	cooling
6,914	36,5944	7,8666	401,57	cooling	6,914	38,1473	14,4109	331,8		6,914	174,3778	86,323	1230,88	cooling	6,914	33,3774	11,1933	310,1	cooling
6,916	35,4056	0,5583	487,11	cooling	6,916	39,0819	19,858	268,72		6,916	175,8597	74,2515	1420,33	cooling	6,916	33,9539	12,1513	304,77	cooling
6,918	36,8667	10,8305	363,95	cooling	6,918	38,043	20,2395	248,87		6,918	182,1636	99,5922	1154,23	cooling	6,918	33,857	9,8121	336,11	cooling
6,92	35,5441	9,9405	357,9	cooling	6,92	38,0774	16,8006	297,42		6,92	171,7987	90,7909	1132,37	cooling	6,92	33,8176	8,8695	348,74	cooling
6,922	35,5149	11,9467	329,45	cooling	6,922	38,2744	21,3327	236,82		6,922	174,0891	93,4228	1127,6	cooling	6,922	33,0734	7,7972	353,32	cooling
6,924	36,1098	8,8388	381,21	cooling	6,924	38,0818	18,458	274,31		6,924	176,6621	88,2208	1236,28	cooling	6,924	34,2478	11,7771	314,11	cooling
6,926	35,8256	6,5244	409,59	cooling	6,926	38,5133	16,1588	312,48		6,926	173,1133	58,7603	1598,49	cooling	6,926	33,5513	14,2639	269,61	cooling
6,928	36,545	3,9798	455,21	cooling	6,928	37,8814	15,3979	314,29		6,928	170,3851	40,8672	1810,47	cooling	6,928	33,1422	13,3669	276,43	cooling
6,93	36,2278	10,1602	364,39	cooling	6,93	38,0586	13,7949	339,17		6,93	174,0177	64,667	1528,56	cooling	6,93	33,7383	14,5726	267,91	cooling
6,932	36,2329	6,7147	412,62	cooling	6,932	38,4288	8,634	416,49		6,932	168,3514	60,5986	1506,22	cooling	6,932	34,5813	11,0339	329,16	cooling
6,934	35,2659	9,1053	365,69	cooling	6,934	38,6904	15,6463	322,12		6,934	168,2543	71,738	1349,15	cooling	6,934	33,5617	11,5761	307,33	cooling
6,936	34,8178	8,5079	367,77	cooling	6,936	37,5258	13,0215	342,53		6,936	168,0876	86,9822	1133,73	cooling	6,936	33,394	8,2434	351,57	cooling
6,938	33,7347	3,79	418,58	cooling	6,938	39,3456	17,9274	299,39		6,938	167,6812	80,9897	1211,82	cooling	6,938	34,4742	9,4759	349,44	cooling
6,94	34,464	9,5495	348,27	cooling	6,94	38,2079	15,9555	311,06		6,94	169,5298	70,3834	1385,92	cooling	6,94	33,9436	7,6043	368,18	cooling
6,942	33,5978	8,6334	348,97	cooling	6,942	38,4118	15,8846	314,9		6,942	173,3288	84,3239	1244,16	cooling	6,942	33,1218	9,8897	324,75	cooling
6,944	33,4931	5,8194	386,84	cooling	6,944	38,0948	14,3902	331,36		6,944	173,4272	89,6576	1170,98	cooling	6,944	33,1873	10,2661	320,4	cooling
6,946	33,8927	5,4772	397,21	cooling	6,946	38,9247	17,8627	294,42		6,946	182,2982	99,3151	1159,98	cooling	6,946	33,7866	14,1446	274,57	cooling
6,948	33,4531	3,6414	416,72	cooling	6,948	37,6844	16,8885	290,7		6,948	182,2319	118,4761	891,21	cooling	6,948	32,2871	9,6905	315,87	cooling
6,95	34,578	4,8713	415,26	cooling	6,95	38,5075	19,1727	270,27		6,95	178,786	106,6195	1008,78	cooling	6,95	33,3857	10,0118	326,73	cooling
6,952	33,5884	10,2185	326,68	cooling	6,952	37,5398	17,1836	284,55		6,952	174,7146	74,5652	1399,94	cooling	6,952	32,717	7,0471	358,83	cooling
6,954	33,7319	4,2758	411,75	cooling	6,954	37,6664	14,1166	329,19		6,954	172,6254	62,0706	1545,39	cooling	6,954	33,1946	4,847	396,26	cooling
6,956	34,2214	7,754	369,98	cooling	6,956	37,6532	16,454	296,33		6,956	169,9186	71,2145	1379,74	cooling	6,956	32,3697	6,7066	358,73	cooling
6,958	34,1457	7,2987	375,28	cooling	6,958	38,08	14,8433	324,81		6,958	169,4932	64,1657	1472,32	cooling	6,958	33,6305	9,7942	333,2	cooling
6,96	33,5628	4,1678	410,9	cooling	6,96	37,5939	15,7719	305,04		6,96	169,6121	68,2207	1417,3	cooling	6,96	32,9055	9,6816	324,64	cooling
6,962	34,5907	9,5111	350,58	cooling	6,962	37,1109	12,5909	342,75		6,962	171,2168	76,3756	1325,74	cooling	6,962	33,2966	12,2631	294,02	cooling
6,964	33,885	9,3479	342,99	cooling	6,964	38,1031	15,3515	318,03		6,964	172,2513	84,6477	1224,57	cooling	6,964	33,2332	13,4836	276,07	cooling
6,966	33,6624	4,2193	411,57	cooling	6,966	37,9289	14,2751	330,64		6,966	175,1968	86,2972	1242,69	cooling	6,966	35,0593	12,9068	309,66	cooling
6,968	34,0551	10,0047	336,19	cooling	6,968	37,9223	18,0197	278,21	cooling	6,968	177,2227	91,5698	1197,3	cooling	6,968	33,5011	15,8842	246,26	cooling
6,97	32,8419	5,3648	384,09	cooling	6,97	37,5519	17,0131	287,1		6,97	181,2003	102,3174	1102,67	cooling	6,97	32,9674	10,7485	310,59	cooling
6,972	33,995	4,0373	418,76	cooling	6,972	38,7038	21,4331	241,42		6,972	183,043	85,5034	1363,46	cooling	6,972	34,4115	12,0148	313,07	cooling
6,974	32,9757	8,6701	339,76	cooling	6,974	36,9304	15,921	293,68		6,974	189,0961	81,0051	1510,95	cooling	6,974	33,8663	9,4287	341,6	cooling

6,976	32,8996	6,076	374,95	cooling	6,976	37,7827	15,4845	311,7		6,976	190,5132	84,1523	1486,77	cooling	6,976	32,9612	10,8162	309,55	cooling
6,978	32,5479	8,894	330,65	cooling	6,978	37,0686	14,6097	313,94		6,978	191,9245	91,7653	1400,08	cooling	6,978	32,6764	12,3838	283,66	cooling
6,98	32,1622	10,2725	305,98	cooling	6,98	37,4591	16,2748	296,13		6,98	192,796	87,0778	1477,78	cooling	6,98	33,4309	17,7979	218,53	cooling
6,982	31,2856	3,4096	389,66	cooling	6,982	36,5651	18,4728	252,9		6,982	199,7625	90,4322	1528,28	cooling	6,982	31,6592	11,6178	280,15	cooling
6,984	32,5693	6,152	369,28	cooling	6,984	37,507	18,0345	272,2		6,984	202,7875	86,7874	1621,51	cooling	6,984	32,7963	14,0503	262,04	cooling
6,986	31,472	9,0596	313,29	cooling	6,986	36,8399	14,1629	316,99		6,986	203,708	63,9711	1953,31	cooling	6,986	31,994	9,5401	313,87	cooling
6,988	31,273	4,091	379,96	cooling	6,988	37,1648	10,127	377,95		6,988	201,7512	79,7779	1705,01	cooling	6,988	33,0624	10,2529	318,84	cooling
6,99	32,4327	10,7535	303,04	cooling	6,99	37,6474	12,0081	358,4		6,99	201,342	97,3391	1453,81	cooling	6,99	31,9734	12,3822	273,86	cooling
6,992	31,952	7,0962	347,45	cooling	6,992	38,4127	13,1239	353,5		6,992	200,0906	88,4422	1560,68	cooling	6,992	33,1769	14,4869	261,26	cooling
6,994	32,5071	3,971	398,89	cooling	6,994	38,0476	20,4229	246,37		6,994	200,248	80,8143	1669,51	cooling	6,994	32,6983	13,4234	269,43	
6,996	32,2186	7,7754	341,68	cooling	6,996	37,2915	17,2044	280,79		6,996	205,682	114,1051	1280,11	cooling	6,996	32,6027	15,3743	240,83	
6,998	31,8809	8,3459	328,98	cooling	6,998	38,7147	18,4724	282,96		6,998	210,0489	117,2087	1297,77	cooling	6,998	32,453	14,1945	255,23	
7	31,1767	3,0781	392,78	cooling	7	37,7579	13,1214	344,38		7	219,3668	106,7985	1573,54	cooling	7	33,4314	13,7028	275,78	
7,002	31,946	5,7749	365,83	cooling	7,002	37,8874	16,5121	298,8		7,002	219,0853	117,4773	1420,33	cooling	7,002	32,1353	16,8209	214,07	
7,004	31,1735	-1,7785	460,62	cooling	7,004	37,3105	18,7191	259,88		7,004	219,0695	115,0555	1453,96	cooling	7,004	31,5161	12,6879	263,19	
7,006	32,9514	3,8539	406,74	cooling	7,006	38,0899	20,4033	247,23		7,006	220,0884	104,16	1620,51	cooling	7,006	33,033	15,8069	240,79	
7,008	31,7861	8,9719	318,91	cooling	7,008	36,7834	13,7298	322,26		7,008	218,7368	109,3458	1529,12	cooling	7,008	32,1046	14,5934	244,78	
7,01	32,1124	6,2562	361,43	cooling	7,01	38,0047	16,4857	300,8		7,01	225,3262	137,8373	1222,97	cooling	7,01	32,0934	13,2485	263,42	
7,012	32,5232	8,1171	341,16	cooling	7,012	37,119	12,8751	338,89		7,012	228,9151	105,0502	1731,45	cooling	7,012	32,4506	14,242	254,53	heating
7,014	32,5281	7,4144	351,05	cooling	7,014	38,315	11,6695	372,46		7,014	228,2983	127,284	1412,03	cooling	7,014	32,8293	15,7938	238,13	heating
7,016	32,6024	2,7606	417,14	cooling	7,016	37,354	12,9722	340,82		7,016	225,9502	125,3874	1405,72	cooling	7,016	31,8352	12,3376	272,55	heating
7,018	33,1907	7,6878	356,49	cooling	7,018	38,0356	11,4537	371,58		7,018	223,7881	102,1355	1700,52	cooling	7,018	33,3243	16,735	231,89	heating
7,02	31,9516	9,0409	320,26	cooling	7,02	37,8127	12,0147	360,62	cooling	7,02	223,4293	99,4129	1733,57	cooling	7,02	32,2189	14,8567	242,7	heating
7,022	32,0142	7,0862	348,46	cooling	7,022	37,9661	13,779	338,1	cooling	7,022	221,6626	113,4849	1512,16	cooling	7,022	32,4116	11,4253	293,36	heating
7,024	32,9224	14,1827	261,95	cooling	7,024	37,743	13,8515	333,97	cooling	7,024	222,8393	120,1414	1435,57	cooling	7,024	32,1821	13,5952	259,82	heating
7,026	32,0321	5,4865	371,07	cooling	7,026	38,5725	13,0599	356,63	cooling	7,026	219,6454	105,5786	1594,49	cooling	7,026	32,9601	10,9748	307,32	heating
7,028	33,3483	3,4713	417,64	cooling	7,028	37,6801	16,3227	298,54	cooling	7,028	220,3762	90,3111	1818,12	cooling	7,028	32,6267	10,1983	313,52	heating
7,03	32,6512	7,7622	347,91	cooling	7,03	37,1848	11,5375	358,51	cooling	7,03	218,8318	89,0808	1813,73	cooling	7,03	32,5103	11,4986	293,71	heating
7,032	32,6175	6,9155	359,28	cooling	7,032	38,6131	16,7048	306,24	cooling	7,032	218,0077	96,4363	1699,39	cooling	7,032	33,5989	12,2605	298,28	heating
7,034	32,55	6,1598	368,9	cooling	7,034	38,6562	17,0493	302,03	cooling	7,034	219,728	121,1974	1377,31	cooling	7,034	34,2625	12,5322	303,76	heating
7,036	33,4913	8,1498	354,24	cooling	7,036	38,344	16,7744	301,51	cooling	7,036	218,5534	125,7845	1296,77	cooling	7,036	33,5847	16,006	245,72	heating
7,038	31,9613	-1,9845	474,51	cooling	7,038	37,9393	15,4205	314,78	cooling	7,038	219,6665	110,7051	1523,12	cooling	7,038	33,0451	11,9353	295,08	heating
7,04	33,6207	6,6848	376,52	cooling	7,04	38,3558	16,0075	312,4	cooling	7,04	217,3824	87,7035	1812,72	cooling	7,04	34,3766	14,987	271,04	heating
7,042	31,7636	10,4763	297,57	cooling	7,042	37,1922	11,6206	357,45	cooling	7,042	218,4799	87,7884	1826,87	cooling	7,042	32,8149	14,2492	259,52	heating
7,044	32,3244	1,5443	430,26	cooling	7,044	37,7774	13,0537	345,6	cooling	7,044	216,1245	94,0809	1705,99	cooling	7,044	32,8427	14,709	253,48	heating
7,046	32,4113	0,5909	444,8	cooling	7,046	37,5564	15,2406	311,94	cooling	7,046	214,0412	94,977	1664,34	cooling	7,046	32,4925	17,0006	216,55	heating
7,048	32,8938	2,0446	431,23	cooling	7,048	37,9189	13,5386	340,8	cooling	7,048	214,2202	101,4859	1575,86	cooling	7,048	32,0488	14,8446	240,49	heating
7,05	34,124	0,5728	469	cooling	7,05	37,1551	16,2749	291,88	cooling	7,05	207,9773	100,6241	1500,64	cooling	7,05	31,4347	11,9944	271,75	heating
7,052	33,7734	12,535	296,88	cooling	7,052	38,0296	16,1858	305,34	cooling	7,052	210,41	107,0983	1444,14	cooling	7,052	32,8636	15,5432	242,11	heating
7,054	32,4355	11,7342	289,37	cooling	7,054	37,4698	15,904	301,46	cooling	7,054	214,9897	98,775	1624,51	cooling	7,054	32,0329	13,8614	254,01	heating
7,056	32,6313	3,1204	412,52	cooling	7,056	37,9516	13,8279	337,21	cooling	7,056	212,2774	106,9494	1472,33	cooling	7,056	32,3545	11,4276	292,53	heating
7,058	34,4496	8,3272	365,15	cooling	7,058	38,0469	17,6406	285,25	cooling	7,058	212,6794	103,9498	1519,88	cooling	7,058	32,8101	15,1714	246,56	heating
7,06	31,6149	0,0438	441,32	cooling	7,06	38,6108	12,774	361,16	cooling	7,06	213,231	88,0276	1750,16	cooling	7,06	33,6352	12,4899	295,58	heating
7,062	34,0358	4,658	410,66	cooling	7,062	37,9087	16,4719	299,65	cooling	7,062	217,2666	85,5992	1840,51	cooling	7,062	33,4583	13,9907	272,13	heating
7,064	32,1462	12,3203	277,14	cooling	7,064	37,6525	14,8422	318,85	cooling	7,064	222,1934	87,1699	1887,43	cooling	7,064	33,0713	12,4668	288,02	heating
7,066	32,4193	8,3409	336,58	cooling	7,066	38,2439	16,0906	309,67	cooling	7,066	226,8374	91,4464	1892,57	cooling	7,066	34,669	10,7605	334,21	heating
7,068	31,9471	4,1184	389	cooling	7,068	38,1264	16,2962	305,15	cooling	7,068	223,7257	124,4421	1387,84	cooling	7,068	35,3594	7,5246	389,09	heating
7,07	34,6055	13,4278	296,03	cooling	7,07	37,3269	17,543	276,55	cooling	7,07	222,3104	123,8892	1375,78	cooling	7,07	34,1743	10,4944	331,01	heating
7,072	32,976	0,6207	452,28	cooling	7,072	37,2856	10,6554	372,25	cooling	7,072	221,1573	91,9451	1806,2	cooling	7,072	33,4852	8,7187	346,2	heating
7,074	33,8515	10,2146	330,41	cooling	7,074	37,8877	10,9371	376,73	cooling	7,074	221,1293	88,7758	1850,11	cooling	7,074	34,8274	14,7479	280,68	heating
7,076	31,7666	10,0226	303,95	cooling	7,076	36,6402	10,0939	371,08	cooling	7,076	216,4478	95,1014	1696,24	cooling	7,076	33,4771	14,2503	268,76	heating
7,078	32,2417	2,124	421	cooling	7,078	37,3561	12,3246	349,9	cooling	7,078	223,6451	98,3108	1751,99	cooling	7,078	33,1304	14,6436	258,42	heating
7,08	32,4539	6,3464	364,95	cooling	7,08	37,1776	17,9003	269,47	cooling	7,08	219,0403	126,354	1295,62	cooling	7,08	32,7882	13,6786	267,12	heating
7,082	30,9938	5,5927	355,07	cooling	7,082	36,6457	15,0689	301,61	cooling	7,082	217,0495	128,2927	1240,69	cooling	7,082	33,0676	13,0917	279,23	heating
7,084	33,2696	1,394	445,57	cooling	7,084	36,6745	14,0272	316,58	cooling	7,084	218,1229	117,3086	1409,23	cooling	7,084	31,7671	10,3928	298,78	heating
7,086	32,2942	11,0404	297,1	cooling	7,086	37,4841	14,7336	318,02	cooling	7,086	213,5108	106,6053	1494,38	cooling	7,086	32,7659	14,189	259,68	heating

7,088	31,2904	8,9494	312,29	cooling	7,088	36,5781	13,4142	323,8	cooling	7,088	216,4371	99,9129	1628,84	cooling	7,088	32,2538	15,9528	227,86	heating
7,09	31,5813	3,1839	396,95	cooling	7,09	36,9349	10,8997	363,93	cooling	7,09	233,0198	76,9485	2181,65	cooling	7,09	32,4493	17,0004	215,95	heating
7,092	33,3478	12,316	293,99	cooling	7,092	37,4455	15,8073	302,47	cooling	7,092	225,8853	112,3839	1586,58	cooling	7,092	32,503	19,0639	187,86	heating
7,094	30,5483	3,4831	378,33	cooling	7,094	37,2317	12,2473	349,24	cooling	7,094	223,6733	120,2001	1446,4	cooling	7,094	33,8887	17,1762	233,62	heating
7,096	32,5167	7,1037	355,24	cooling	7,096	37,1585	13,4183	331,85	cooling	7,096	224,7105	89,3843	1891,66	cooling	7,096	33,3825	16,6121	234,43	heating
7,098	30,6588	9,5789	294,67	cooling	7,098	36,7702	13,0772	331,19	cooling	7,098	228,6454	90,3839	1932,69	cooling	7,098	32,9081	13,519	271,03	heating
7,1	30,561	4,9214	358,4	cooling	7,1	37,5322	14,8022	317,73	cooling	7,1	213,2054	65,6588	2062,48	cooling	7,1	34,251	14,9346	270,02	heating
7,102	30,0821	2,6979	382,79	cooling	7,102	36,5829	14,0863	314,47	cooling	7,102	213,7494	86,8798	1773,45	cooling	7,102	34,1652	11,978	310,14	heating
7,104	31,4317	13,2942	253,54	cooling	7,104	36,6058	18,2266	256,91	cooling	7,104	211,6825	112,2251	1390,27	cooling	7,104	33,1282	13,9756	267,72	heating
7,106	31,7785	3,7404	391,93	cooling	7,106	35,6949	14,2513	299,75	cooling	7,106	212,0405	116,4297	1336,5	cooling	7,106	32,4672	13,2437	268,72	heating
7,108	31,729	12,1918	273,1	cooling	7,108	37,0798	14,9482	309,37	cooling	7,108	210,7325	94,886	1619,36	cooling	7,108	33,5288	16,1314	243,19	heating
7,11	30,8482	8,5618	311,53	cooling	7,11	35,8545	13,9558	306,11	cooling	7,11	214,5129	90,6592	1731,29	cooling	7,11	32,2819	14,0098	255,42	heating
7,112	31,642	3,5138	393,19	cooling	7,112	37,0509	17,0402	279,72	cooling	7,112	211,3947	101,5607	1535,32	cooling	7,112	32,9507	16,5231	229,63	heating
7,114	32,4295	9,099	326,13	cooling	7,114	36,525	19,4134	239,19	cooling	7,114	213,4051	100,5251	1577,9	cooling	7,114	33,0642	15,4588	246,1	heating
7,116	31,5619	10,314	297,01	cooling	7,116	36,6037	18,119	258,39	cooling	7,116	213,1834	86,4897	1770,99	cooling	7,116	33,8391	15,7926	252,26	heating
7,118	33,8229	4,8439	405,08	cooling	7,118	36,3141	15,9119	285,19	cooling	7,118	215,7875	83,337	1851,46	cooling	7,118	33,1766	15,9866	240,29	heating
7,12	32,6931	10,0641	316,32	cooling	7,12	37,0448	14,9224	309,24	cooling	7,12	215,0018	77,0322	1928,61	cooling	7,12	33,8725	15,1984	261,04	heating
7,122	31,7008	6,1652	356,95	cooling	7,122	36,4301	15,7505	289,07	cooling	7,122	216,583	82,4547	1874,92	cooling	7,122	33,5489	16,9328	232,27	heating
7,124	31,5968	0,3702	436,5	cooling	7,124	35,9373	14,0557	305,87	cooling	7,124	215,6305	94,697	1690,47	cooling	7,124	32,4073	14,7791	246,42	heating
7,126	33,4882	8,8328	344,65	cooling	7,126	36,4541	15,6658	290,59	cooling	7,126	217,1168	103,7482	1584,72	cooling	7,126	33,0451	17,0319	223,84	heating
7,128	30,4584	-2,9265	466,67	cooling	7,128	35,9563	11,7052	338,99		7,128	218,8117	103,665	1609,58	cooling	7,128	33,3673	18,0779	213,72	heating
7,13	31,2488	-0,9722	450,4	cooling	7,13	36,4546	14,2138	310,89	cooling	7,13	220,7813	101,5548	1666,61	cooling	7,13	33,0903	19,0134	196,78	heating
7,132	30,2163	1,8405	396,65	cooling	7,132	35,7028	13,7623	306,7	cooling	7,132	221,6409	98,1192	1726,65	cooling	7,132	32,5164	15,0354	244,36	heating
7,134	30,2734	-0,0492	423,87	cooling	7,134	36,7877	17,8731	264,4	cooling	7,134	221,7694	100,0228	1701,84	cooling	7,134	34,4172	16,811	246,11	heating
7,136	30,4663	-1,163	442,13	cooling	7,136	35,6217	15,5086	281,15		7,136	223,9015	122,6902	1414,78	cooling	7,136	33,7916	9,8793	334,26	heating
7,138	32,4188	7,9253	342,38	cooling	7,138	36,3981	16,6474	276,09		7,138	220,7763	125,8661	1326,7	cooling	7,138	34,0365	12,67	298,67	heating
7,14	32,5746	-2,9855	497,08	cooling	7,14	35,8944	16,0189	277,83		7,14	222,2461	115,999	1485,18	cooling	7,14	33,3117	13,2312	280,7	heating
7,142	32,1689	4,3064	389,48	cooling	7,142	36,6531	18,9586	247,34		7,142	220,4517	111,3203	1525,5	cooling	7,142	34,0667	14,1544	278,35	heating
7,144	30,8574	1,9226	404,47	cooling	7,144	35,698	17,5778	253,29		7,144	221,1536	101,2359	1676,27	cooling	7,144	33,262	15,7822	244,34	heating
7,146	31,2503	-0,4499	443,12	cooling	7,146	36,4496	17,3299	267,27		7,146	220,1986	87,0381	1861,39	cooling	7,146	33,4325	15,8633	245,59	heating
7,148	31,5172	4,5133	377,47	cooling	7,148	35,8506	14,2649	301,74		7,148	220,9423	108,3734	1573,55	cooling	7,148	33,5984	13,7474	277,49	heating
7,15	30,8781	6,9402	334,62	cooling	7,15	36,1466	12,6893	327,9		7,15	220,2527	109,6122	1546,59	cooling	7,15	33,4642	13,5774	277,99	heating
7,152	32,4296	6,5831	361,3	cooling	7,152	36,2117	15,9379	283,4		7,152	222,5952	85,1426	1921,38	cooling	7,152	33,2892	12,6923	287,91	heating
7,154	31,6897	10,1561	301,01	cooling	7,154	36,5061	16,1486	284,57		7,154	221,6264	84,4259	1917,86	cooling	7,154	34,0671	9,6794	340,9	heating
7,156	30,9768	6,4502	342,85	cooling	7,156	36,3351	17,6318	261,44		7,156	225,2609	75,3707	2095,24	cooling	7,156	34,2714	13,4006	291,74	heating
7,158	31,4537	5,2791	365,88	cooling	7,158	35,9671	13,6519	311,93		7,158	228,776	85,9911	1995,92	cooling	7,158	33,1285	9,2254	334,13	heating
7,16	31,9242	11,282	288,55	cooling	7,16	36,6302	13,8744	318,09		7,16	225,3887	115,17	1540,69	cooling	7,16	34,804	16,1075	261,35	heating
7,162	30,9576	9,7532	296,41	cooling	7,162	36,4096	13,5995	318,85		7,162	221,9842	106,6147	1612,7	cooling	7,162	34,8954	15,6383	269,19	heating
7,164	30,8925	13,0416	249,53	cooling	7,164	36,5559	18,3223	254,88		7,164	222,933	89,5955	1863,86	cooling	7,164	34,1659	14,7223	271,79	heating
7,166	30,6726	6,5139	337,7	cooling	7,166	36,1953	17,145	266,29		7,166	221,754	98,7925	1718,82	cooling	7,166	33,6529	9,1745	342,17	heating
7,168	31,1479	4,1762	377,02	cooling	7,168	36,8587	20,1789	233,16		7,168	221,9534	81,1136	1968,73	cooling	7,168	35,2823	10,1097	351,88	heating
7,17	31,1036	1,4307	414,78	cooling	7,17	35,7931	17,219	259,64		7,17	222,6539	98,8024	1731,26	cooling	7,17	35,6918	6,3856	409,66	heating
7,172	31,6033	5,7786	360,99	cooling	7,172	36,4074	17,4555	264,92		7,172	220,2409	119,0196	1414,92	cooling	7,172	34,9461	13,7879	295,76	heating
7,174	31,6091	9,5406	308,48	cooling	7,174	35,9981	16,2951	275,42		7,174	218,2575	105,2406	1579,81	cooling	7,174	34,1425	12,432	303,48	heating
7,176	31,0238	8,0386	321,3	cooling	7,176	36,3792	17,5137	263,71	heating	7,176	221,6887	99,3203	1710,53	cooling	7,176	35,6398	13,352	311,55	heating
7,178	30,765	-0,1916	432,73	cooling	7,178	35,6326	17,6037	252,02	heating	7,178	223,5307	111,0338	1572,54	cooling	7,178	34,7197	10,5117	338,39	heating
7,18	31,5877	1,7429	417,19	cooling	7,18	35,9435	16,7201	268,72	heating	7,18	224,9562	87,9435	1915,23	cooling	7,18	34,0698	8,947	351,18	heating
7,182	30,2541	1,5189	401,68	cooling	7,182	35,5186	15,9779	273,15	heating	7,182	223,0357	102,2064	1689,02	cooling	7,182	34,4437	10,0833	340,52	heating
7,184	30,7515	6,0603	345,15	cooling	7,184	35,7031	15,2404	286,04	heating	7,184	221,8532	106,6306	1610,64	cooling	7,184	34,5135	12,5852	306,53	
7,186	30,6639	9,8819	290,5	cooling	7,186	36,1499	17,4396	261,54	heating	7,186	219,8676	90,0528	1814,62	cooling	7,186	33,5363	11,3421	310,24	
7,188	31,2814	1,5891	415,05	cooling	7,188	36,1549	17,0875	266,53	heating	7,188	223,1914	101,8711	1695,88	cooling	7,188	34,514	12,4724	308,11	
7,19	30,8603	1,1668	415,07	cooling	7,19	36,1532	18,6484	244,69	heating	7,19	220,0291	113,4143	1490,32	cooling	7,19	34,3538	15,6301	261,73	
7,192	31,1154	4,8079	367,74	cooling	7,192	35,9841	16,7064	269,47	heating	7,192	222,3769	105,028	1640,36	cooling	7,192	33,7645	10,0391	331,65	
7,194	30,996	5,8494	351,51	cooling	7,194	36,8167	19,7659	238,34	heating	7,194	221,0968	100,2693	1688,99	cooling	7,194	34,5903	13,8115	290,46	
7,196	31,4699	10,3284	295,53	cooling	7,196	36,0817	17,8064	255,46	heating	7,196	220,1455	95,9143	1736,57	cooling	7,196	35,1685	11,0808	336,71	
7,198	30,3257	9,5617	290,25	cooling	7,198	36,7138	19,5025	240,59	heating	7,198	219,2314	80,2061	1943,37	cooling	7,198	34,4253	10,7578	330,84	

7,2	30,9361	1,1589	416,24	cooling	7,2	36,2313	18,026	254,48	heating	7,2	224,7948	93,5777	1834,22	cooling	7,2	33,883	9,6745	338,4	
7,202	31,5422	5,1352	369,13	cooling	7,202	36,496	17,3217	268,03	heating	7,202	223,8754	91,8609	1845,37	cooling	7,202	35,4256	16,4901	264,69	
7,204	30,091	2,8664	380,56	cooling	7,204	35,9861	16,0665	278,45	heating	7,204	225,9443	125,4576	1404,65	cooling	7,204	34,9192	12,9164	307,57	
7,206	30,5463	4,4419	364,9	cooling	7,206	36,9121	17,1847	275,76	heating	7,206	224,444	135,5232	1242,98	cooling	7,206	34,3231	17,3602	237,12	
7,208	30,7817	5,6954	350,67	cooling	7,208	36,586	15,4464	295,5	heating	7,208	226,3443	120,2105	1483,59	cooling	7,208	33,536	14,4549	266,72	
7,21	31,3163	3,6716	386,43	cooling	7,21	36,9566	17,3391	274,22	heating	7,21	224,9476	101,8515	1720,7	cooling	7,21	34,6327	13,0882	301,16	
7,212	30,6427	-2,7241	466,42	cooling	7,212	36,6279	19,5044	239,36	heating	7,212	226,2416	93,8166	1851,11	cooling	7,212	33,619	12,3382	297,47	
7,214	32,1704	0,0813	448,56	cooling	7,214	37,247	19,5859	246,88	heating	7,214	225,6019	65,4495	2238,69	cooling	7,214	33,3957	12,9235	286,17	
7,216	31,5523	-0,5521	448,77	cooling	7,216	36,9843	23,8602	183,46	heating	7,216	222,2824	85,8835	1906,65	cooling	7,216	33,4806	13,7811	275,37	
7,218	32,1365	2,0632	420,38	cooling	7,218	36,5326	20,7401	220,76	heating	7,218	224,8304	123,8989	1410,87	cooling	7,218	33,5684	15,8728	247,36	
7,22	31,3864	2,7228	400,67	cooling	7,22	37,4624	19,8335	246,43	heating	7,22	221,7319	103,4894	1652,85	cooling	7,22	32,7893	16,1881	232,06	
7,222	32,6517	4,5229	393,2	cooling	7,222	37,3148	17,3754	278,72	heating	7,222	224,6538	110,7009	1592,89	cooling	7,222	33,7036	14,486	268,63	heating
7,224	32,4035	4,2202	393,96	cooling	7,224	36,788	16,2951	286,46	heating	7,224	220,0876	107,0689	1579,83	cooling	7,224	33,7542	16,6714	238,79	heating
7,226	31,9364	1,9616	419	cooling	7,226	36,8078	16,6436	281,87	heating	7,226	225,3347	89,4215	1899,86	cooling	7,226	33,192	10,7919	313,12	heating
7,228	32,2521	1,462	430,4	cooling	7,228	37,4336	20,8508	231,8	heating	7,228	222,3993	87,995	1878,77	cooling	7,228	34,2461	13,8838	284,64	heating
7,23	32,0251	2,0693	418,74	cooling	7,23	36,5729	13,2917	325,44	heating	7,23	224,7224	95,9423	1800,15	cooling	7,23	34,5222	11,0559	328,02	heating
7,232	31,9523	7,8305	337,19	cooling	7,232	37,4081	14,4076	321,51	heating	7,232	219,8441	94,1261	1757,35	cooling	7,232	34,0063	11,2684	317,84	heating
7,234	32,0566	13,3338	261,72	cooling	7,234	36,658	11,617	350,04	heating	7,234	229,1325	134,3393	1325,07	cooling	7,234	33,786	10,8952	319,98	heating
7,236	33,4336	12,3202	295,13	cooling	7,236	37,5049	13,0456	341,91	heating	7,236	221,3315	129,2983	1286,49	cooling	7,236	34,9027	14,2715	288,39	
7,238	33,3413	5,6436	387,17	cooling	7,238	37,1742	18,5664	260,11	heating	7,238	224,261	117,2014	1496,53	cooling	7,238	34,3376	10,3914	334,73	heating
7,24	33,7904	6,6434	379,47	cooling	7,24	36,983	17,8345	267,67	heating	7,24	221,4688	96,0494	1753,18	cooling	7,24	34,1264	13,6001	286,93	
7,242	33,1896	3,9252	409,07	cooling	7,242	37,2535	15,9625	297,62	heating	7,242	223,7999	73,4552	2101,6	cooling	7,242	33,4447	11,0932	312,44	
7,244	34,5497	11,4699	322,62	cooling	7,244	37,3826	18,8241	259,42	heating	7,244	220,9411	55,6251	2310,87	cooling	7,244	34,218	9,0072	352,41	
7,246	33,0663	12,3961	288,94	cooling	7,246	36,667	16,4059	283,22	heating	7,246	229,2478	101,5057	1785,64	cooling	7,246	33,5519	10,6594	320	
7,248	33,9673	7,4955	370,04	cooling	7,248	37,4977	17,0495	285,84	heating	7,248	224,6856	106,9729	1645,45	cooling	7,248	33,6328	11,636	307,48	
7,25	34,1776	3,5308	428,4	cooling	7,25	37,1157	21,2258	222,12	heating	7,25	224,0041	127,5034	1348,94	cooling	7,25	33,4616	11,9379	300,87	
7,252	33,792	2,5285	437,02	cooling	7,252	36,7395	16,3174	285,47	heating	7,252	222,0971	127,9334	1316,27	cooling	7,252	33,2929	11,8291	300,03	
7,254	33,4001	4,707	401,09	cooling	7,254	37,8317	21,2643	231,59	heating	7,254	222,5353	107,5742	1606,99	cooling	7,254	33,0468	12,7482	283,74	
7,256	34,566	11,7641	318,74	cooling	7,256	37,2071	18,0446	267,86	heating	7,256	221,0742	86,3801	1882,82	cooling	7,256	33,8708	11,3624	314,63	
7,258	34,2178	7,2595	376,84	cooling	7,258	37,4579	14,518	320,67	heating	7,258	227,4817	113,3066	1596	cooling	7,258	33,7289	15,6144	253,21	
7,26	34,2499	1,1351	462,9	cooling	7,26	37,9744	17,3645	288,1	heating	7,26	223,8757	95,6302	1792,68	cooling	7,26	33,0488	9,797	325,02	
7,262	34,4614	3,3154	435,38	cooling	7,262	37,7501	18,2753	272,23	heating	7,262	224,667	108,5995	1622,45	cooling	7,262	34,6563	11,5529	322,95	
7,264	34,4642	1,288	463,75	cooling	7,264	37,7412	16,9331	290,87	heating	7,264	227,3107	137,1669	1260,08	cooling	7,264	34,4207	7,6358	374,41	
7,266	34,5094	7,9037	371,91	cooling	7,266	38,1433	21,906	226,97	heating	7,266	225,8706	119,6359	1485	cooling	7,266	34,0671	6,7984	381,18	
7,268	33,7111	7,831	361,77	cooling	7,268	37,1084	13,895	324,49	heating	7,268	229,3687	97,2068	1847,43	cooling	7,268	33,5481	4,87	400,88	
7,27	34,9483	4,7392	422,28	cooling	7,27	38,254	15,7567	314,48	heating	7,27	234,6092	109,3959	1750,3	cooling	7,27	35,0807	9,847	352,73	
7,272	35,033	2,5436	454,15	cooling	7,272	37,035	17,553	272,33	heating	7,272	239,0957	94,23	2025,01	cooling	7,272	34,7962	6,4332	396,47	cooling
7,274	34,0577	4,4434	413,96	cooling	7,274	37,4857	13,0628	341,4	heating	7,274	235,3527	99,2358	1902,71	cooling	7,274	34,2347	11,2546	321,23	cooling
7,276	33,7435	6,3909	382,35	cooling	7,276	37,6001	15,3684	310,77	heating	7,276	235,5578	98,8766	1910,6	cooling	7,276	33,3308	7,9976	354,12	cooling
7,278	34,6053	11,751	319,47	cooling	7,278	37,0087	13,5214	328,32	heating	7,278	232,1136	80,405	2120,66	cooling	7,278	34,877	8,4666	369,18	cooling
7,28	33,921	8,0014	362,32	cooling	7,28	37,4208	11,6859	359,73	heating	7,28	233,1844	89,7346	2005,22	cooling	7,28	33,6574	7,6404	363,68	cooling
7,282	34,1199	8,0207	364,83	cooling	7,282	38,5529	19,1251	271,57	heating	7,282	231,5703	116,7028	1605,68	cooling	7,282	33,4978	10,5611	320,62	cooling
7,284	33,4766	7,2336	366,84	cooling	7,284	37,3216	15,7214	301,94	heating	7,284	233,3791	98,4274	1886,42	cooling	7,284	33,3364	11,1696	309,86	cooling
7,286	33,7484	3,8386	418,09	cooling	7,286	38,1196	13,8761	338,89	heating	7,286	231,1001	101,5453	1810,98	cooling	7,286	33,6154	11,6168	307,51	cooling
7,288	33,3391	10,1423	324,26	cooling	7,288	38,6228	19,5674	266,37	heating	7,288	229,7686	98,8078	1830,64	cooling	7,288	32,621	9,5505	322,49	cooling
7,29	32,7653	10,3867	312,82	cooling	7,29	38,1644	13,5457	344,13	heating	7,29	229,7211	85,576	2014,94	cooling	7,29	33,8592	10,8609	321,48	cooling
7,292	33,3963	9,3619	335,96	cooling	7,292	38,8142	18,9475	277,71	heating	7,292	231,2471	112,9814	1653,18	cooling	7,292	33,5857	13,393	282,26	cooling
7,294	33,8235	6,8403	377,18	cooling	7,294	37,4569	18,398	266,41	heating	7,294	235,3094	136,9177	1375,37	cooling	7,294	33,0334	11,1925	305,3	cooling
7,296	33,2698	1,3377	446,36	cooling	7,296	37,8307	14,5953	324,8	heating	7,296	241,9715	97,2217	2023,39	cooling	7,296	34,1643	16,4976	246,96	cooling
7,298	33,4174	2,7825	428,23	cooling	7,298	37,5148	19,3168	254,38	heating	7,298	238,1498	100,8725	1918,93	cooling	7,298	34,621	12,8323	304,57	cooling
7,3	33,7283	11,8221	306,22	cooling	7,3	36,5038	20,2796	226,79	heating	7,3	235,8702	116,6685	1666,26	cooling	7,3	34,0001	12,3392	302,79	cooling
7,302	33,2345	8,743	342,35	cooling	7,302	36,7557	15,4393	297,97	heating	7,302	236,7038	129,2204	1502,46	cooling	7,302	33,485	9,6328	333,42	cooling
7,304	33,9631	11,693	311,3	cooling	7,304	37,389	18,6231	262,32	heating	7,304	232,428	139,2346	1302,71	cooling	7,304	34,9076	11,5675	326,26	cooling
7,306	32,8394	5,394	383,65	cooling	7,306	36,6736	11,3366	354,17	heating	7,306	238,7468	152,9989	1198,63	cooling	7,306	34,4074	8,4984	362,17	cooling
7,308	33,4613	2,9238	426,87	cooling	7,308	38,0579	12,0223	363,94	heating	7,308	249,1976	130,8842	1653,85	cooling	7,308	34,0506	12,6232	299,52	cooling
7,31	32,7415	5,3855	382,4	cooling	7,31	38,1019	16,9932	295,07		7,31	238,2023	133,0038	1470,52	cooling	7,31	33,3279	9,6303	331,26	cooling

7,312	32,1101	5,4566	372,58	cooling	7,312	37,8634	12,8672	349,41		7,312	240,0496	121,5796	1656,04	cooling	7,312	34,591	11,824	318,25	cooling
7,314	32,9459	8,697	338,96	cooling	7,314	39,2745	20,2602	265,79		7,314	240,7315	109,1614	1839,15	cooling	7,314	32,871	12,1245	290	cooling
7,316	33,5996	12,922	289,04	cooling	7,316	38,3896	19,8443	259,24		7,316	238,3928	114,8666	1726,71	cooling	7,316	33,6281	12,8849	289,96	cooling
7,318	33,0049	6,8464	365,66	cooling	7,318	38,4123	16,2388	309,95		7,318	233,2752	108,7103	1741,23	cooling	7,318	33,3049	14,7575	259,27	cooling
7,32	32,8837	8,0553	347,06	cooling	7,32	38,4029	18,8583	273,2		7,32	244,4639	110,0719	1878,6	cooling	7,32	33,0237	13,4614	273,45	cooling
7,322	32,6692	9,1586	328,64	cooling	7,322	37,7634	18,6393	267,33		7,322	240,1632	131,4118	1520,18	cooling	7,322	32,6167	13,1587	271,99	cooling
7,324	33,3982	6,3406	378,23	cooling	7,324	38,0664	17,0344	294	heating	7,324	246,6309	109,9463	1910,65	cooling	7,324	33,8494	13,3839	286,08	cooling
7,326	33,4933	12,8419	288,68	cooling	7,326	37,9944	23,6237	200,88	heating	7,326	245,837	104,2015	1979,85	cooling	7,326	33,1369	14,2154	264,49	
7,328	32,6944	7,5241	351,84	cooling	7,328	37,6454	15,6534	307,42	heating	7,328	244,0074	105,4056	1937,45	cooling	7,328	32,6261	10,6196	307,62	
7,33	33,9133	9,1213	346,56	cooling	7,33	38,7872	16,2487	315,05	heating	7,33	239,2614	83,2754	2180,45	cooling	7,33	34,0839	16,1601	250,55	
7,332	33,302	10,7291	315,54	cooling	7,332	38,3096	17,8227	286,38	heating	7,332	246,7601	93,3012	2145,13	cooling	7,332	34,5122	12,0189	314,42	
7,334	32,0424	4,8098	380,67	cooling	7,334	38,4872	16,368	309,19	heating	7,334	249,789	136,4764	1583,94	cooling	7,334	34,1836	15,0949	266,83	
7,336	33,1949	6,4194	374,28	cooling	7,336	39,2916	20,5033	262,63	heating	7,336	250,47	117,1217	1864,01	cooling	7,336	33,305	11,7258	301,65	
7,338	33,6577	11,0264	316,35	cooling	7,338	38,4662	16,5059	306,97	heating	7,338	250,5614	128,2008	1710,42	cooling	7,338	35,0172	13,137	305,85	
7,34	33,2828	1,2355	447,97	cooling	7,34	38,4491	8,0363	425,13	heating	7,34	246,3179	133,1244	1582,28	cooling	7,34	33,881	9,4662	341,28	
7,342	33,2305	6,7747	369,81	cooling	7,342	38,8294	11,3559	384,04		7,342	238,0658	95,9315	1986,83	cooling	7,342	33,8116	10,3567	327,87	
7,344	32,33	4,1577	393,81	cooling	7,344	37,612	9,4297	393,95		7,344	242,3972	78,9733	2284,42	cooling	7,344	33,3891	7,6727	359,48	
7,346	32,8079	-0,6421	467,58	cooling	7,346	38,1505	12,5953	357,22		7,346	242,706	102,8585	1954,86	cooling	7,346	33,9504	9,0138	348,58	
7,348	32,9747	5,0648	390,14	cooling	7,348	37,6718	20,0177	246,78		7,348	238,3499	101,3792	1914,65	cooling	7,348	32,5154	9,0079	328,6	cooling
7,35	32,4296	3,0325	410,93	cooling	7,35	37,7688	15,8156	306,87		7,35	240,8219	132,4043	1515,52	cooling	7,35	33,4479	8,9388	342,6	cooling
7,352	33,0346	0,5888	453,54	cooling	7,352	37,77	17,4641	283,85		7,352	238,6564	137,055	1420,24	cooling	7,352	33,0964	12,0002	294,89	cooling
7,354	33,0359	2,1826	431,28	cooling	7,354	37,405	17,0151	285,02		7,354	230,2451	126,3886	1451,76	cooling	7,354	32,55	8,6688	333,82	cooling
7,356	31,694	-3,9687	498,51	cooling	7,356	37,2956	13,965	326,13		7,356	234,7766	115,8188	1662,85	cooling	7,356	33,8542	13,6391	282,58	cooling
7,358	33,276	0,6835	455,59	cooling	7,358	38,6384	17,1809	299,94		7,358	232,1183	114,484	1644,35	cooling	7,358	34,5028	11,718	318,5	cooling
7,36	32,6875	10,4665	310,62	cooling	7,36	37,6528	16,7487	292,21		7,36	233,1736	110,6357	1712,9	cooling	7,36	33,885	11,9658	306,4	cooling
7,362	32,4955	1,6053	431,8	cooling	7,362	38,5608	15,7118	319,39		7,362	236,0018	114,8952	1692,89	cooling	7,362	33,2578	6,2117	378,06	cooling
7,364	32,8884	6,2867	371,85	cooling	7,364	38,9169	20,7353	254,15		7,364	240,1194	101,8661	1932,58	cooling	7,364	35,2205	9,7096	356,61	cooling
7,366	32,2791	4,2287	392,1	cooling	7,366	38,4026	17,3004	294,98		7,366	231,6551	106,9633	1743,01	cooling	7,366	34,6844	6,9439	387,77	cooling
7,368	32,3834	-1,7057	476,51	cooling	7,368	38,8634	18,2866	287,63		7,368	238,6519	125,5705	1580,71	cooling	7,368	34,4763	14,9304	273,22	cooling
7,37	33,2208	5,4278	388,5	cooling	7,37	38,505	18,5682	278,69		7,37	232,4374	130,0347	1431,44	cooling	7,37	33,3133	12,7685	287,19	cooling
7,372	32,9528	7,2114	359,83	cooling	7,372	38,867	15,3906	328,17		7,372	234,9646	113,8877	1692,48	cooling	7,372	35,1228	13,9635	295,78	cooling
7,374	33,536	5,2002	396,09	cooling	7,374	38,6294	16,635	307,45		7,374	233,8489	106,5311	1779,71	cooling	7,374	33,1773	9,4525	331,64	cooling
7,376	33,8674	15,4366	257,64	cooling	7,376	38,5373	14,8454	331,18		7,376	232,1171	97,4335	1882,68	cooling	7,376	33,4489	8,8567	343,76	cooling
7,378	32,4303	8,536	334,01	cooling	7,378	38,3696	11,4491	376,31		7,378	228,2249	77,3271	2109,33	cooling	7,378	32,8283	9,4353	327	cooling
7,38	33,5276	5,0331	398,31	cooling	7,38	39,323	16,3584	321,01		7,38	233,6107	93,6738	1956,11	cooling	7,38	33,1389	9,5819	329,29	cooling
7,382	33,0192	7,2219	360,61	cooling	7,382	38,2407	15,4063	319,19		7,382	227,5277	116,7042	1549,15	cooling	7,382	32,193	9,0002	324,2	cooling
7,384	32,9202	3,6615	408,99	cooling	7,384	39,405	17,7995	302,01		7,384	229,1964	123,9803	1470,76	cooling	7,384	33,323	11,765	301,35	cooling
7,386	32,9316	11,4394	300,43	cooling	7,386	38,7342	18,9594	276,42		7,386	227,833	116,0039	1563,2	cooling	7,386	32,4474	13,1914	269,17	cooling
7,388	32,2337	12,2064	279,95	cooling	7,388	38,8898	14,4902	341,07		7,388	228,7775	116,5664	1568,54	cooling	7,388	32,3866	12,1972	282,22	cooling
7,39	31,9539	1,8517	420,78	cooling	7,39	39,2366	15,2399	335,44		7,39	225,1301	85,441	1952,65	cooling	7,39	33,0308	18,4631	203,64	cooling
7,392	33,9042	2,909	433,27	cooling	7,392	38,7131	13,3783	354,14		7,392	237,8913	79,3787	2215,77	cooling	7,392	33,414	14,0881	270,15	cooling
7,394	32,3344	0,7143	442	cooling	7,394	38,7783	11,6702	378,93		7,394	231,735	107,5163	1736,39	cooling	7,394	33,3878	15,178	254,55	
7,396	33,7522	2,8945	431,34	cooling	7,396	38,5663	15,4019	323,8	cooling	7,396	233,6581	119,6086	1594,24	cooling	7,396	32,392	10,1081	311,5	
7,398	33,6275	11,4002	310,7	cooling	7,398	38,5251	16,8433	303,08	cooling	7,398	233,4617	114,5748	1661,86	cooling	7,398	33,987	10,9631	321,84	
7,4	32,5988	1,6924	432,02	cooling	7,4	38,1774	16,0767	308,94	cooling	7,4	235,9879	133,001	1439,6	cooling	7,4	33,6845	11,3663	311,98	
7,402	32,7992	0,1271	456,71	cooling	7,402	38,7799	20,3258	257,96	cooling	7,402	225,2787	99,6553	1756,03	cooling	7,402	33,5243	17,1152	229,37	
7,404	32,3902	5,4538	376,53	cooling	7,404	38,0053	17,0385	293,08	cooling	7,404	241,5776	91,2631	2101,18	cooling	7,404	32,6245	12,6144	279,71	
7,406	32,1327	2,829	409,62	cooling	7,406	38,9832	19,6084	270,83		7,406	233,5568	112,7571	1688,6	cooling	7,406	34,8398	17,1886	246,74	
7,408	31,8987	5,7061	366,13	cooling	7,408	38,0323	19,3747	260,81		7,408	231,3536	122,7709	1517,83	cooling	7,408	33,0005	12,0069	293,46	
7,41	31,6076	7,2403	340,62	cooling	7,41	38,6598	16,5734	308,73		7,41	230,1218	100,4054	1813,24	cooling	7,41	33,645	11,5537	308,8	
7,412	31,2973	-0,323	442,01	cooling	7,412	38,5285	16,2528	311,38		7,412	229,6852	89,3127	1962,2	cooling	7,412	32,9318	12,4082	286,89	
7,414	33,394	10,0655	326,1	cooling	7,414	38,6389	14,6897	334,77		7,414	229,2589	101,7127	1782,91	cooling	7,414	33,0821	11,5075	301,58	
7,416	31,3236	10,0728	297,05	cooling	7,416	38,6766	11,9724	373,29		7,416	236,6748	103,8316	1856,95	cooling	7,416	32,0557	11,7066	284,45	
7,418	32,4878	8,1681	339,95	cooling	7,418	38,8875	15,2481	330,44	cooling	7,418	234,2787	119,5398	1603,88	cooling	7,418	32,7797	14,643	253,52	
7,42	32,1882	9,4492	317,86	cooling	7,42	38,3169	17,4676	291,44	cooling	7,42	231,8368	130,5576	1415,73	cooling	7,42	31,7151	14,5381	240,11	
7,422	31,5475	4,3287	380,48	cooling	7,422	38,2277	16,9517	297,41	cooling	7,422	233,9858	125,2757	1519,61	cooling	7,422	31,6421	12,637	265,66	

7,424	31,1019	4,2321	375,6	cooling	7,424	38,8886	18,7348	281,72	cooling	7,424	233,0264	103,8288	1805,99	cooling	7,424	32,4432	16,9856	216,07	
7,426	31,3697	10,1369	296,8	cooling	7,426	38,4037	13,2411	351,74		7,426	228,5536	89,4661	1944,24	cooling	7,426	32,7425	11,1416	301,95	heating
7,428	30,1697	6,6711	328,48	cooling	7,428	38,8654	15,0295	333,19	cooling	7,428	239,6694	121,7835	1647,87	cooling	7,428	32,7054	15,2695	243,73	
7,43	30,1202	8,1015	307,79	cooling	7,43	38,4479	16,638	304,87	cooling	7,43	238,6987	114,1953	1740,37	cooling	7,43	31,7452	10,6279	295,19	heating
7,432	29,1119	9,4997	274,15	cooling	7,432	38,8442	18,3409	286,6	cooling	7,432	235,8677	121,4826	1598,93	cooling	7,432	33,9254	12,5336	299,03	heating
7,434	28,7453	2,2413	370,49	cooling	7,434	38,4189	17,4347	293,33	cooling	7,434	235,1923	112,3525	1717,12	cooling	7,434	33,3204	10,3236	321,46	heating
7,436	28,9223	2,9983	362,38	cooling	7,436	38,7672	19,5123	269,16		7,436	238,1339	105,1042	1859,56	cooling	7,436	33,0632	11,9766	294,76	
7,438	27,9879	1,4989	370,28	cooling	7,438	38,5078	14,9258	329,64		7,438	229,2175	87,8809	1975,68	cooling	7,438	32,5424	8,7001	333,28	
7,44	29,4473	3,4197	363,83	cooling	7,44	38,7502	16,0792	316,91		7,44	230,0918	99,2697	1828,7	cooling	7,44	33,9259	13,4149	286,71	
7,442	29,5035	8,2794	296,68	cooling	7,442	38,0165	15,6801	312,23		7,442	229,9066	110,1228	1674,4	cooling	7,442	31,9931	10,9965	293,5	
7,444	29,2769	7,5378	303,88	cooling	7,444	38,5287	16,8279	303,35		7,444	228,6521	110,4011	1652,97	cooling	7,444	33,3632	12,0502	297,92	
7,446	29,1587	2,2931	375,54	cooling	7,446	38,6273	20,012	260,22		7,446	228,7469	117,472	1555,46	cooling	7,446	32,4926	11,7855	289,45	
7,448	30,7713	8,7941	307,21	cooling	7,448	38,2645	18,0043	283,21		7,448	234,697	139,6422	1328,73	cooling	7,448	32,3686	7,6881	345	
7,45	29,3735	5,2578	337,1	cooling	7,45	38,1759	12,3656	360,79		7,45	232,8656	96,9462	1899,95	cooling	7,45	32,5042	12,264	282,93	
7,452	29,8565	4,0835	360,27	cooling	7,452	38,5173	11,0877	383,43		7,452	228,9859	78,5607	2102,72	cooling	7,452	33,07	12,1932	291,83	
7,454	29,3606	3,0239	368,15	cooling	7,454	38,1529	9,8646	395,43	cooling	7,454	230,8412	86,7339	2014,41	cooling	7,454	32,7347	13,9895	262,03	
7,456	29,4089	2,0902	381,87	cooling	7,456	38,2203	11,4815	373,77	cooling	7,456	228,9697	89,314	1952,18	cooling	7,456	32,1422	11,2122	292,57	
7,458	29,465	5,7273	331,82	cooling	7,458	38,5046	19,0107	272,5	cooling	7,458	228,8918	99,2988	1811,52	cooling	7,458	33,854	15,2916	259,48	
7,46	29,5667	8,9256	288,53	cooling	7,46	38,3973	16,9205	300,21	cooling	7,46	236,362	134,4218	1424,97	cooling	7,46	33,803	9,6093	338,19	
7,462	29,6981	4,8465	347,39	cooling	7,462	38,2371	18,2559	279,31	cooling	7,462	239,851	118,5026	1696,27	cooling	7,462	33,6207	15,8228	248,79	
7,464	29,6036	2,601	377,46	cooling	7,464	38,0991	17,2533	291,39	cooling	7,464	234,699	127,7638	1494,8	cooling	7,464	32,5377	11,513	293,89	
7,466	29,6712	3,2708	369,04	cooling	7,466	37,9357	13,0594	347,73	cooling	7,466	233,9445	106,796	1777,35	cooling	7,466	34,7128	15,2513	272,04	
7,468	29,5981	0,707	403,85	cooling	7,468	38,4016	12,0873	367,84	cooling	7,468	232,1391	91,3064	1968,63	cooling	7,468	32,9108	11,4674	299,75	
7,47	30,5464	6,7133	333,15	cooling	7,47	37,8102	15,3083	314,54	cooling	7,47	230,1031	80,8709	2086,04	cooling	7,47	33,1676	12,3588	290,88	
7,472	29,6399	5,4347	338,35	cooling	7,472	37,9653	14,7744	324,17	cooling	7,472	232,1563	105,8207	1765,98	cooling	7,472	32,5002	8,219	339,41	
7,474	30,2897	6,0017	339,51	cooling	7,474	38,4102	19,3461	266,49	cooling	7,474	233,5428	134,5918	1383,19	cooling	7,474	33,6773	9,5342	337,49	
7,476	29,7404	3,9937	359,9	cooling	7,476	37,5728	15,5051	308,47	cooling	7,476	232,9461	147,024	1201,06	cooling	7,476	31,7959	8,4645	326,14	
7,478	29,6821	5,0119	344,85	cooling	7,478	38,0329	11,5192	370,62	cooling	7,478	230,7916	112,5444	1652,92	cooling	7,478	33,0068	13,5707	271,69	
7,48	29,2717	1,3741	389,97	cooling	7,48	38,446	14,7455	331,3	cooling	7,48	230,4161	83,4729	2054,05	cooling	7,48	31,9702	13,8583	253,18	
7,482	29,9849	5,5475	341,6	cooling	7,482	37,9484	13,7078	338,85	cooling	7,482	227,7503	73,3829	2157,83	cooling	7,482	32,3801	8,6	332,41	
7,484	30,0221	5,9297	336,78	cooling	7,484	37,8133	14,6813	323,35	cooling	7,484	230,5836	74,8461	2176,98	cooling	7,484	32,8371	11,2862	301,25	
7,486	29,9995	7,5796	313,4	cooling	7,486	37,9967	17,4327	287,45	cooling	7,486	230,0633	111,5402	1656,78	cooling	7,486	33,567	9,3786	338,12	
7,488	30,2577	3,1901	378,36	cooling	7,488	37,3391	14,5937	317,95	cooling	7,488	231,3339	136,6506	1323,53	cooling	7,488	33,1405	13,5748	273,5	
7,49	30,2455	1,0837	407,64	cooling	7,49	38,1668	16,9977	295,91	cooling	7,49	233,3493	139,7053	1309	cooling	7,49	32,3638	11,7084	288,73	
7,492	31,0008	6,668	340,14	cooling	7,492	37,69	19,9395	248,13	cooling	7,492	241,2494	130,9385	1541,98	cooling	7,492	33,9234	15,0425	263,93	
7,494	30,4496	5,7638	345,07	cooling	7,494	38,2221	14,8811	326,27	cooling	7,494	238,1851	107,863	1821,71	cooling	7,494	33,6302	10,9664	316,81	
7,496	30,5423	4,9158	358,22	cooling	7,496	38,1953	17,6734	286,87	cooling	7,496	240,0707	103,4318	1910,01	cooling	7,496	33,3125	20,1694	183,72	
7,498	30,3513	4,0059	368,27	cooling	7,498	37,7519	14,6616	322,77	cooling	7,498	234,7669	117,6422	1637,23	cooling	7,498	32,1313	16,8733	213,28	
7,5	29,8166	3,3983	369,29	cooling	7,5	38,0365	10,6002	383,52	cooling	7,5	231,2951	126,9621	1458,42	cooling	7,5	34,2474	20,6918	189,49	heating
7,502	30,6401	6,9386	331,31	cooling	7,502	38,5642	12,3797	366,02	cooling	7,502	225,3613	116,7793	1517,82	cooling	7,502	33,0098	14,0898	264,47	heating
7,504	29,9572	7,8252	309,37	cooling	7,504	37,4834	11,2976	366,04	cooling	7,504	232,7894	124,1555	1518,54	cooling	7,504	33,0063	13,0455	279,02	heating
7,506	30,2172	-3,6141	472,91	cooling	7,506	37,9201	12,4742	355,7	cooling	7,506	236,0379	94,7175	1975,45	cooling	7,506	32,8261	12,1268	289,35	heating
7,508	31,3338	2,5094	402,92	cooling	7,508	37,8209	17,9541	277,71	cooling	7,508	231,2316	118,2726	1579	cooling	7,508	33,4423	15,7139	247,82	heating
7,51	30,6495	5,0451	357,91	cooling	7,51	36,7655	13,1035	330,76	cooling	7,51	233,1251	137,4103	1337,95	cooling	7,51	31,985	15,0221	237,12	heating
7,512	30,7733	9,7249	294,23	cooling	7,512	37,5692	13,9693	329,89	cooling	7,512	230,1309	129,6178	1405,02	cooling	7,512	33,2244	17,5063	219,72	heating
7,514	31,0854	13,2789	248,91	cooling	7,514	37,035	17,1569	277,87	cooling	7,514	228,374	92,469	1899,75	cooling	7,514	32,2917	16,282	223,79	heating
7,516	30,3781	6,8422	329	cooling	7,516	37,3134	14,7969	314,75	cooling	7,516	236,107	96,9599	1945,07	cooling	7,516	32,3952	11,6831	289,52	heating
7,518	31,1398	0,1215	433,59	cooling	7,518	36,9168	18,2242	261,29	cooling	7,518	249,4515	91,6146	2206,33	cooling	7,518	32,5884	16,0794	230,77	heating
7,52	30,332	2,6611	386,8	cooling	7,52	36,6815	16,9769	275,44	cooling	7,52	241,7606	139,2147	1433,44	cooling	7,52	33,4727	11,1264	312,37	heating
7,522	30,1995	5,6254	343,51	cooling	7,522	36,1398	10,9081	352,7	cooling	7,522	241,4761	144,4416	1356,4	cooling	7,522	33,1994	14,9266	255,43	heating
7,524	30,5314	9,6425	292	cooling	7,524	36,8723	13,0873	332,48	cooling	7,524	244,4145	119,3826	1747,76	cooling	7,524	32,6203	12,2568	284,65	heating
7,526	30,0791	8,3047	304,37	cooling	7,526	35,8579	11,9004	334,89	cooling	7,526	244,9135	101,4708	2005,12	cooling	7,526	34,2217	15,1125	267,12	heating
7,528	30,7283	4,7518	363,11	cooling	7,528	36,5949	11,3807	352,46	cooling	7,528	240,4399	91,3436	2084,15	cooling	7,528	34,2953	13,3599	292,65	heating
7,53	29,649	5,2341	341,29	cooling	7,53	36,311	17,1477	267,87	cooling	7,53	251,4287	96,0579	2171,85	cooling	7,53	33,2128	16,3835	235,25	heating
7,532	30,023	3,4866	370,94	cooling	7,532	36,0644	15,1877	291,83	cooling	7,532	248,2796	142,9262	1472,68	cooling	7,532	32,9285	10,9506	307,22	heating
7,534	29,4491	3,1267	367,95	cooling	7,534	37,1614	16,9566	282,43	cooling	7,534	244,9962	152,4432	1293,75	cooling	7,534	34,8312	17,2536	245,71	heating

7,536	30,3705	4,9652	355,13	cooling	7,536	36,9846	16,0074	293,23	cooling	7,536	244,2224	121,565	1714,57	cooling	7,536	32,6764	11,0698	302,03	heating
7,538	29,1974	1,0187	393,9	cooling	7,538	36,9513	9,6521	381,6	cooling	7,538	244,6527	102,3748	1988,83	cooling	7,538	33,154	12,2918	291,62	heating
7,54	30,303	1,1425	407,62	cooling	7,54	37,0532	12,1444	348,19	cooling	7,54	237,4945	90,2338	2058,49	cooling	7,54	32,6205	13,5027	267,24	heating
7,542	29,6672	5,3618	339,75	cooling	7,542	36,7304	17,1777	273,32	cooling	7,542	245,4594	99,8652	2035,19	cooling	7,542	33,5723	10,0484	328,83	heating
7,544	29,4557	2,0887	382,55	cooling	7,544	35,5633	15,2744	283,61	cooling	7,544	242,419	132,7148	1533,5	cooling	7,544	32,4458	10,1547	311,6	heating
7,546	29,2412	1,8722	382,58	cooling	7,546	36,6055	18,3269	255,51	cooling	7,546	242,2174	129,4256	1576,66	cooling	7,546	33,8139	12,9714	291,35	heating
7,548	29,6907	5,0175	344,89	cooling	7,548	35,026	11,3539	330,9	cooling	7,548	242,1292	93,5209	2077,32	cooling	7,548	32,6122	10,3074	311,79	heating
7,55	29,6212	4,4707	351,57	cooling	7,55	36,4011	9,1924	380,34	cooling	7,55	252,3637	114,7538	1923,58	cooling	7,55	33,5806	12,379	296,37	heating
7,552	29,0524	1,8698	379,97	cooling	7,552	35,9391	12,305	330,37	cooling	7,552	247,5761	126,4925	1692,57	cooling	7,552	33,2325	17,4808	220,18	heating
7,554	29,3565	5,2184	337,42	cooling	7,554	35,9343	10,7102	352,6	cooling	7,554	254,0077	125,4219	1797,44	cooling	7,554	34,8764	13,1275	304,02	heating
7,556	28,97	6,0211	320,79	cooling	7,556	36,519	10,4045	365,04	cooling	7,556	249,9318	118,1482	1842,14	cooling	7,556	33,9621	19,7602	198,52	heating
7,558	29,4491	9,1811	283,32	cooling	7,558	37,4807	17,7074	276,4	cooling	7,558	249,9105	112,309	1923,47	cooling	7,558	32,5787	11,9254	288,7	heating
7,56	28,34	4,3716	335,04	cooling	7,56	36,5403	14,0626	314,2	cooling	7,56	244,0522	96,4491	2063,27	cooling	7,56	34,5966	12,2839	311,9	heating
7,562	29,6841	1,3655	395,85	cooling	7,562	37,3801	16,8664	286,75	cooling	7,562	250,7567	121,347	1808,96	cooling	7,562	34,3578	11,6355	317,62	heating
7,564	29,1682	6,8893	311,43	cooling	7,564	36,152	16,008	281,58	cooling	7,564	248,742	124,3521	1738,79	cooling	7,564	33,7956	9,9607	333,18	heating
7,566	28,9621	9,0913	277,76	cooling	7,566	35,9749	12,568	327,19	cooling	7,566	245,2264	129,5119	1617,52	cooling	7,566	33,5819	10,6178	321	heating
7,568	28,4264	6,7303	303,28	cooling	7,568	36,0705	18,3678	247,46	cooling	7,568	246,0301	126,7714	1667,06	cooling	7,568	34,5266	17,9627	231,54	heating
7,57	29,5304	5,9868	329,11	cooling	7,57	35,1808	17,0083	254,02	cooling	7,57	249,1853	117,9936	1833,87	cooling	7,57	31,666	10,1891	300,22	heating
7,572	28,4671	3,6381	347,07	cooling	7,572	35,6607	13,949	303,5	cooling	7,572	248,4008	97,7531	2105,83	cooling	7,572	33,9459	15,4353	258,75	heating
7,574	29,5196	8,3831	295,46	cooling	7,574	35,8761	17,4769	257,19	cooling	7,574	257,8728	106,5732	2114,94	cooling	7,574	31,7773	11,4647	283,94	heating
7,576	28,9557	10,9412	251,82	cooling	7,576	35,6657	16,9829	261,16		7,576	265,1241	111,9619	2140,98	cooling	7,576	33,5152	6,2531	381,08	heating
7,578	29,3631	1,6871	386,87	cooling	7,578	36,0989	16,6548	271,8		7,578	260,313	152,4513	1507,75	cooling	7,578	32,4001	12,2657	281,45	heating
7,58	29,0397	0,5577	398,14	cooling	7,58	36,5869	20,6031	223,43		7,58	258,8277	162,223	1350,39	cooling	7,58	33,3529	11,1093	310,93	heating
7,582	29,3533	4,6281	345,62	cooling	7,582	36,1095	14,0181	308,81		7,582	259,1704	134,2465	1746,25	cooling	7,582	32,9417	10,5082	313,59	heating
7,584	29,7581	6,4971	325,15	cooling	7,584	37,1651	15,7829	298,89		7,584	259,1553	127,5848	1839,16	cooling	7,584	33,7831	12,7703	293,73	heating
7,586	29,1927	6,3926	318,71	cooling	7,586	35,8591	11,9828	333,76		7,586	257,9141	129,1119	1800,46	cooling	7,586	34,5839	12,2556	312,12	heating
7,588	29,2842	4,3254	348,89	cooling	7,588	36,3147	9,209	378,9		7,588	266,2291	99,9794	2323,92	cooling	7,588	35,5631	11,5635	335,48	heating
7,59	29,0725	2,7494	367,96	cooling	7,59	36,0817	13,0478	321,98		7,59	263,7739	113,8335	2095,94	cooling	7,59	33,9801	19,2845	205,42	heating
7,592	29,7297	6,7623	321,05	cooling	7,592	35,4992	13,3885	309,07		7,592	265,3849	129,4182	1900,61	cooling	7,592	32,7693	9,2067	329,37	heating
7,594	28,2101	2,472	359,78	cooling	7,594	35,5277	12,3464	324,04		7,594	266,7749	128,5971	1931,52	cooling	7,594	35,952	17,2385	261,59	heating
7,596	29,8787	-0,1745	420,1	cooling	7,596	35,8832	17,1414	261,98		7,596	271,1339	146,9546	1735,84	cooling	7,596	33,9643	12,9003	294,44	heating
7,598	28,986	1,4645	384,71	cooling	7,598	34,9926	13,0067	307,33		7,598	269,8976	154,9527	1606,76	cooling	7,598	34,7636	14,4221	284,34	heating
7,6	29,5447	4,5175	349,84	cooling	7,6	35,4377	12,6535	318,49		7,6	281,4556	118,775	2274,03	cooling	7,6	33,6037	15,6397	251,11	heating
7,602	29,0205	5,7232	325,66	cooling	7,602	35,5594	16,24	270,06		7,602	278,6504	140,5961	1929,79	cooling	7,602	34,9958	15,8223	268,02	heating
7,604	30,0827	6,0574	335,84	cooling	7,604	35,5081	11,8259	331,04		7,604	277,291	145,3073	1844,94	cooling	7,604	32,8671	8,291	343,54	heating
7,606	28,9926	3,6328	354,49	cooling	7,606	36,3156	14,0879	310,71		7,606	275,0314	123,2166	2122,15	cooling	7,606	35,0899	17,6836	243,31	heating
7,608	29,2555	3,8728	354,81	cooling	7,608	35,6345	11,9396	331,22		7,608	273,1739	107,5947	2314,55	cooling	7,608	32,9114	10,4194	314,4	heating
7,61	29,5681	7,8315	303,84	cooling	7,61	36,3858	9,9149	370,02		7,61	272,6032	132,3919	1959,95	cooling	7,61	34,6925	13,8431	291,44	heating
7,612	29,1168	7,6298	300,36	cooling	7,612	36,1374	12,7611	326,77	cooling	7,612	275,1374	133,6173	1978,24	cooling	7,612	32,3967	14,5111	250,01	heating
7,614	28,6672	6,0708	315,86	cooling	7,614	35,6628	15,5724	280,83	cooling	7,614	271,1948	142,1242	1804,22	cooling	7,614	33,954	10,13	333,02	heating
7,616	28,6887	6,5448	309,54	cooling	7,616	35,3587	14,0925	297,27	cooling	7,616	270,183	132,1619	1929,33	cooling	7,616	33,6991	13,3592	284,32	heating
7,618	29,4861	9,1959	283,63	cooling	7,618	35,8769	21,0679	207,01		7,618	271,4717	118,2527	2141,77	cooling	7,618	32,7366	10,6543	308,68	heating
7,62	28,2835	7,768	286,78	cooling	7,62	34,32	15,6202	261,4		7,62	269,5688	102,3075	2338,06	cooling	7,62	34,4459	12,0822	312,61	heating
7,622	28,4235	5,6936	317,73	cooling	7,622	34,6252	14,4135	282,53		7,622	266,6953	96,0997	2384,67	cooling	7,622	35,0063	12,3222	317,09	heating
7,624	28,1018	3,4443	344,68	cooling	7,624	34,3596	16,6704	247,27		7,624	270,2917	114,3731	2179,51	cooling	7,624	33,8489	13,1202	289,76	heating
7,626	28,8581	8,5665	283,65	cooling	7,626	33,9125	15,1072	262,87		7,626	266,9402	119,0674	2067,04	cooling	7,626	33,3713	7,7257	358,49	heating
7,628	27,7329	8,9006	263,25	cooling	7,628	34,2331	16,3434	250,07		7,628	267,2601	108,6315	2217,39	cooling	7,628	35,9528	17,2291	261,73	
7,63	28,5567	3,5306	349,83	cooling	7,63	34,3882	18,7606	218,45		7,63	265,8848	105,5046	2241,88	cooling	7,63	33,7946	8,9124	347,82	
7,632	28,4451	1,5492	375,96	cooling	7,632	34,4946	15,2868	268,5		7,632	265,9478	125,1809	1967,71	cooling	7,632	34,9124	16,0153	264,15	
7,634	28,0956	4,2563	333,24	cooling	7,634	34,5588	12,6053	306,88		7,634	259,029	119,9973	1943,46	cooling	7,634	33,2165	12,0692	295,61	
7,636	27,505	4,5433	320,97	cooling	7,636	34,9886	13,8874	294,96		7,636	262,4525	123,3053	1945,07	cooling	7,636	34,5148	10,2707	338,9	
7,638	28,0749	4,9511	323,24	cooling	7,638	34,7137	9,2797	355,53		7,638	259,9669	123,1232	1912,87	cooling	7,638	32,7457	9,2354	328,64	
7,64	27,7479	5,2875	313,96	cooling	7,64	34,747	12,5425	310,39		7,64	261,653	116,8513	2024,11	cooling	7,64	33,6223	11,8843	303,86	
7,642	27,0098	0,3272	372,98	cooling	7,642	33,8944	13,3193	287,61		7,642	259,7531	114,0436	2036,8	cooling	7,642	33,3214	10,7532	315,47	
7,644	28,1062	4,9204	324,1	cooling	7,644	34,0746	13,2201	291,51		7,644	266,3651	138,1698	1791,98	cooling	7,644	33,4002	11,6088	304,61	
7,646	27,5329	5,127	313,2	cooling	7,646	34,3028	12,1515	309,64		7,646	261,0645	132,4969	1797,18	cooling	7,646	32,911	10,0038	320,21	

7,648	27,3195	1,2653	364,2	cooling	7,648	34,2943	13,0992	296,28		7,648	260,4798	138,4907	1705,23	cooling	7,648	34,4671	9,1741	353,56	
7,65	27,4029	-0,4199	388,92	cooling	7,65	34,8165	12,6687	309,59		7,65	259,5729	127,8659	1841,07	cooling	7,65	33,7087	12,1699	301,08	
7,652	27,6982	3,5285	337,86	cooling	7,652	35,3991	18,3256	238,66		7,652	260,9162	112,2502	2078,13	cooling	7,652	32,6946	4,4258	395,16	
7,654	27,6967	5,7473	306,82	cooling	7,654	35,2223	20,6722	203,39		7,654	262,7977	118,9257	2011,12	cooling	7,654	34,9237	12,1065	318,95	
7,656	27,3562	9,488	249,77	cooling	7,656	34,9996	18,5389	230,1	heating	7,656	273,5052	125,9025	2063,27	cooling	7,656	34,1094	7,1625	376,68	
7,658	26,6327	1,8813	345,99	cooling	7,658	35,1601	16,9793	254,14	heating	7,658	273,1257	134,4272	1938,8	cooling	7,658	34,0179	10,8414	323,97	
7,66	27,238	-1,2941	398,84	cooling	7,66	34,4848	13,6934	290,63	heating	7,66	273,7774	147,1833	1769,6	cooling	7,66	33,2309	10,1685	322,38	
7,662	26,9642	1,0613	362,08	cooling	7,662	33,866	12,681	296,14	heating	7,662	274,4183	135,7623	1938,21	cooling	7,662	34,5294	13,9186	288,11	cooling
7,664	26,8936	1,6471	352,91	cooling	7,664	33,7924	13,5485	282,98	heating	7,664	274,6832	134,8242	1955,02	cooling	7,664	33,2364	9,1193	337,12	
7,666	26,53	6,0483	286,3	cooling	7,666	33,6206	14,9544	260,93	heating	7,666	275,7968	144,1846	1839,74	cooling	7,666	33,6437	14,3502	269,69	cooling
7,668	25,6886	1,9066	332,44	cooling	7,668	34,0495	14,282	276,32	heating	7,668	275,6504	134,792	1968,99	cooling	7,668	32,9692	7,6653	353,71	
7,67	25,897	-1,9919	389,85	cooling	7,67	34,3192	15,8863	257,66	heating	7,67	277,7997	127,3905	2102,5	cooling	7,67	34,0775	9,685	340,97	cooling
7,672	26,5046	1,2894	352,47	cooling	7,672	35,3599	14,4716	291,99	heating	7,672	279,5047	145,321	1875,69	cooling	7,672	32,3359	9,0671	325,26	cooling
7,674	25,9277	0,7506	351,94	cooling	7,674	35,5478	16,2958	269,11	heating	7,674	278,5686	132,7776	2037,94	cooling	7,674	33,4943	9,3038	338,15	cooling
7,676	26,4381	-3,3898	416,95	cooling	7,676	36,166	18,1309	252,1	heating	7,676	276,6147	131,4295	2029,47	cooling	7,676	33,3639	13,4851	277,88	cooling
7,678	26,7086	2,0319	344,94	cooling	7,678	35,9546	17,526	257,6	heating	7,678	282,3583	159,338	1719,64	cooling	7,678	32,806	10,7777	307,92	cooling
7,68	25,9132	-0,5527	369,95	cooling	7,68	35,9655	14,5469	299,4	heating	7,68	285,2593	142,1944	1999,84	cooling	7,68	33,2161	12,8731	284,37	cooling
7,682	26,3641	0,1872	365,91	cooling	7,682	35,1242	12,4049	317,58	heating	7,682	281,9134	137,8083	2014,38	cooling	7,682	34,0742	12,6315	299,74	cooling
7,684	26,4102	-0,8443	380,98	cooling	7,684	34,3198	9,4799	347,22	heating	7,684	280,6097	156,9534	1728,53	cooling	7,684	33,0301	13,2953	275,86	cooling
7,686	25,9805	-5,6758	442,51	cooling	7,686	34,3672	12,2911	308,59	heating	7,686	280,2333	140,4961	1953,32	cooling	7,686	32,8838	9,4859	327,07	
7,688	26,2683	-0,2835	371,16	cooling	7,688	33,6449	13,9889	274,76	heating	7,688	278,3748	126,2223	2126,87	cooling	7,688	34,1701	16,7096	244,07	cooling
7,69	25,5807	3,8325	304,01	cooling	7,69	34,0266	13,2822	289,98	heating	7,69	283,642	145,9475	1924,76	cooling	7,69	33,5258	12,275	297,05	
7,692	25,1774	-2,0847	381,08	cooling	7,692	34,0021	11,4967	314,59	heating	7,692	288,4589	127,4722	2250,36	cooling	7,692	33,2599	18,2584	209,7	
7,694	25,901	-2,482	396,75	cooling	7,694	34,9885	10,5316	341,87	heating	7,694	281,8859	141,619	1960,72	cooling	7,694	32,384	12,6828	275,39	
7,696	26,3618	0,2117	365,54	cooling	7,696	35,0883	11,3614	331,67	heating	7,696	280,5897	129,4366	2112,9	cooling	7,696	34,0188	12,4889	300,96	
7,698	26,9758	1,8736	350,89	cooling	7,698	36,2161	12,1806	335,98		7,698	281,2582	108,0822	2420,74	cooling	7,698	32,9504	9,8078	323,5	
7,7	26,6326	7,2743	270,6	cooling	7,7	36,5371	14,1157	313,42		7,7	281,0992	115,5833	2313,67	cooling	7,7	33,0223	14,0713	264,91	
7,702	26,0365	1,5622	342,11	cooling	7,702	37,2497	16,4259	291,09		7,702	290,6821	148,893	1982	cooling	7,702	32,6189	14,5638	252,38	
7,704	26,8508	-0,3048	379,6	cooling	7,704	37,0938	18,7131	256,93		7,704	323,5203	125,5326	2767,57	cooling	7,704	33,1307	15,4895	246,6	
7,706	27,1604	2,7225	341,61	cooling	7,706	36,5127	16,7786	275,85		7,706	309,692	160,0104	2092,33	cooling	7,706	32,0938	10,9959	294,92	
7,708	26,7397	4,8529	305,94	cooling	7,708	35,7399	14,9515	290,59		7,708	304,8409	127,3122	2481,59	cooling	7,708	33,8579	10,5867	325,3	
7,71	26,9621	4,0765	319,91	cooling	7,71	35,1372	13,7386	299,12		7,71	306,0099	97,024	2921,31	cooling	7,71	32,555	11,6404	292,36	
7,712	26,9567	4,296	316,76	cooling	7,712	35,517	17,5156	251,63		7,712	318,9784	157,4683	2257,67	cooling	7,712	32,769	10,4156	312,47	
7,714	26,6757	0,4993	365,91	cooling	7,714	35,0343	17,9149	239,3	heating	7,714	327,5933	116,2445	2954,34	cooling	7,714	32,8227	13,8322	265,46	
7,716	27,1386	4,066	322,52	cooling	7,716	35,9381	16,8345	267,04	heating	7,716	307,9382	153,5831	2157,66	cooling	7,716	33,8482	10,4734	326,74	
7,718	27,0402	3,1688	333,69	cooling	7,718	35,5273	13,0149	314,69	heating	7,718	307,0276	170,4094	1909,72	cooling	7,718	33,0776	14,0894	265,43	
7,72	27,2934	5,2891	307,59	cooling	7,72	36,3547	11,8051	343,17	heating	7,72	312,6529	129,3322	2562,55	cooling	7,72	32,8356	12,3149	286,85	
7,722	26,8421	6,0499	290,64	cooling	7,722	36,1433	14,0868	308,32		7,722	314,4645	168,7976	2036,21	cooling	7,722	34,2343	15,9156	256,07	
7,724	26,567	5,9403	288,33	cooling	7,724	36,9639	18,9598	251,67		7,724	292,7156	154,132	1937,19	cooling	7,724	33,9547	14,0274	278,55	
7,726	26,7957	5,239	301,33	cooling	7,726	36,2612	18,1519	253,14	heating	7,726	289,3976	157,871	1838,55	cooling	7,726	33,1991	15,0691	253,43	
7,728	26,383	1,5401	347,27	cooling	7,728	36,581	18,7642	249,05	heating	7,728	290,4932	158,2624	1848,39	cooling	7,728	32,9632	9,0868	333,76	
7,73	26,584	-0,3579	376,61	cooling	7,73	35,862	17,139	261,72	heating	7,73	285,949	127,3988	2216,3	cooling	7,73	34,1149	11,9476	309,87	
7,732	26,5726	4,8079	304,24	cooling	7,732	35,9545	12,1359	332,95	heating	7,732	282,6885	106,6011	2461,44	cooling	7,732	32,1857	6,2724	362,23	
7,734	26,1093	5,2084	292,16	cooling	7,734	35,7074	10,0494	358,66	heating	7,734	289,2744	137,6707	2119,19	cooling	7,734	33,722	11,0102	317,48	
7,736	25,9649	2,1247	333,25	cooling	7,736	35,7882	10,7488	350,01	heating	7,736	289,1526	110,8607	2492,26	cooling	7,736	32,5184	11,8883	288,38	
7,738	26,0614	0,7615	353,65	cooling	7,738	36,5167	15,305	296,51		7,738	282,221	130,1647	2125,52	cooling	7,738	32,861	8,9314	334,5	
7,74	26,0117	-3,719	415,59	cooling	7,74	36,1629	17,4378	261,75		7,74	284,1904	148,1871	1901,12	cooling	7,74	32,7097	10,865	305,36	
7,742	26,0717	1,5951	342,15	cooling	7,742	36,2077	16,0276	282,09		7,742	280,254	114,2759	2320,13	cooling	7,742	33,8108	7,2952	370,65	
7,744	25,2375	2,8521	312,91	cooling	7,744	36,3053	12,8238	328,24		7,744	283,7614	98,1933	2593,97	cooling	7,744	32,9799	6,1876	374,52	
7,746	25,1182	-0,5062	358,19	cooling	7,746	37,4506	17,2579	282,26		7,746	287,1223	121,3262	2317,58	cooling	7,746	33,0984	6,9979	364,85	
7,748	25,6504	-1,7987	383,7	cooling	7,748	36,6236	15,9562	288,9		7,748	294,9122	118,3923	2467,49	cooling	7,748	33,6971	8,7422	348,83	
7,75	25,1617	0,1707	349,34	cooling	7,75	37,397	17,4914	278,25		7,75	295,0374	152,274	1995,62	cooling	7,75	34,1838	5,8751	395,71	cooling
7,752	25,3603	-1,9675	382	cooling	7,752	36,9739	16,2511	289,68		7,752	291,0897	148,5425	1992,6	cooling	7,752	33,4377	12,246	296,23	cooling
7,754	25,8002	5,1845	288,18	cooling	7,754	36,9625	13,3696	329,79		7,754	290,9941	127,1688	2290,03	cooling	7,754	32,6072	5,6523	376,79	cooling
7,756	24,7045	2,284	313,41	cooling	7,756	36,4481	13,6378	318,85		7,756	289,5495	123,9464	2314,89	cooling	7,756	34,1528	11,8613	311,6	cooling
7,758	24,8138	0,1632	344,58	cooling	7,758	36,8626	17,3678	272,51		7,758	281,5761	105,2766	2464,41	cooling	7,758	32,0263	10,5433	300,3	cooling

7,76	25,3219	4,9775	284,38	cooling	7,76	36,0742	14,8339	296,91		7,76	284,8859	135,5707	2087,2	cooling	7,76	32,3576	11,8911	286,09	cooling
7,762	24,9703	3,9867	293,32	cooling	7,762	36,2968	16,3254	279,17		7,762	283,6307	175,2373	1515,18	cooling	7,762	32,344	13,1531	268,26	cooling
7,764	24,942	3,4343	300,65	cooling	7,764	35,9034	18,6929	240,58		7,764	280,5886	141,2393	1947,9	cooling	7,764	32,3641	11,0038	298,59	cooling
7,766	24,6581	5,5775	266,72	cooling	7,766	36,1868	14,9124	297,38	heating	7,766	284,259	105,4012	2500,17	cooling	7,766	32,0739	8,1537	334,37	cooling
7,768	23,9786	1,1761	318,75	cooling	7,768	36,5815	13,557	321,85		7,768	289,9194	112,1343	2485,17	cooling	7,768	33,1692	11,7181	299,85	cooling
7,77	24,5424	2,7132	305,14	cooling	7,77	36,4811	10,9991	356,2		7,77	292,7845	118,2698	2439,46	cooling	7,77	31,781	8,6998	322,64	cooling
7,772	23,9259	5,1424	262,57	cooling	7,772	37,44	13,3114	337,28		7,772	294,8454	161,4312	1864,93	cooling	7,772	32,4826	9,4872	321,44	cooling
7,774	24,2017	-0,3255	342,85	cooling	7,774	37,7694	16,5918	296,03		7,774	291,5533	160,0498	1838,22	cooling	7,774	33,0902	15,4205	247	cooling
7,776	24,1989	3,074	295,3	cooling	7,776	37,4362	17,4151	279,86		7,776	289,431	125,6034	2290,07	cooling	7,776	33,4583	9,5142	334,7	cooling
7,778	23,9655	3,8056	281,81	cooling	7,778	37,2512	13,5759	330,95		7,778	288,1001	136,4638	2119,65	cooling	7,778	33,0679	14,3126	262,17	cooling
7,78	24,1932	0,0275	337,8	cooling	7,78	37,7378	14,7844	320,85		7,78	288,5325	152,396	1902,99	cooling	7,78	32,0407	9,8688	309,93	
7,782	25,6357	1,0425	343,78	cooling	7,782	36,811	11,81	349,48		7,782	291,2367	139,3867	2122,64	cooling	7,782	33,3727	10,2135	323,73	
7,784	25,6701	0,9323	345,8	cooling	7,784	36,9067	14,6998	310,42		7,784	285,5441	136,804	2079,17	cooling	7,784	32,6032	11,1044	300,52	cooling
7,786	26,709	0,6861	363,76	cooling	7,786	36,3478	16,536	276,94		7,786	282,7811	107,6599	2447,93	cooling	7,786	31,6337	12,3849	269,07	
7,788	27,3717	4,8038	315,47	cooling	7,788	36,4733	16,1619	283,92		7,788	285,7419	91,3578	2717,2	cooling	7,788	31,5571	9,2083	312,4	
7,79	26,935	2,3683	343,41	cooling	7,79	36,3478	16,2644	280,74		7,79	286,4791	123,258	2281,59	cooling	7,79	32,2784	15,5721	233,53	
7,792	27,3957	1,4461	362,74	cooling	7,792	36,5254	15,3627	295,82		7,792	284,7245	136,1909	2076,28	cooling	7,792	30,5468	11,3229	268,72	
7,794	27,5012	3,453	336,16	cooling	7,794	36,6998	12,479	338,57		7,794	297,2971	129,9786	2338,87	cooling	7,794	31,8424	16,1677	219,11	
7,796	27,8657	3,7224	337,49	cooling	7,796	37,1273	14,7728	312,48		7,796	291,8393	150,0098	1982,57	cooling	7,796	30,9842	15,8266	211,88	
7,798	27,4749	5,9096	301,45	cooling	7,798	36,9899	18,3039	261,2		7,798	287,3549	148,6098	1939,45	cooling	7,798	31,5554	11,2252	284,19	
7,8	27,5559	5,0694	314,33	cooling	7,8	37,2173	16,8014	285,38		7,8	284,6559	128,6174	2181,19	cooling	7,8	31,6659	14,1834	244,38	
7,802	27,0136	-0,4247	383,55	cooling	7,802	37,34	16,3981	292,74		7,802	283,1726	130,0362	2140,62	cooling	7,802	32,2107	14,2998	250,37	
7,804	27,9549	1,2877	372,77	cooling	7,804	36,6949	10,874	360,94		7,804	281,4548	130,6657	2107,81	cooling	7,804	31,4582	12,0039	271,94	
7,806	26,6878	2,2154	342,09	cooling	7,806	37,3656	12,7198	344,51		7,806	293,3579	116,6286	2470,41	cooling	7,806	32,0908	12,4628	274,37	
7,808	27,5895	3,2601	340,09	cooling	7,808	37,1071	16,0178	294,8		7,808	288,2442	134,5423	2148,52	cooling	7,808	32,0003	9,7397	311,17	heating
7,81	26,8603	4,8678	307,42	cooling	7,81	36,8004	15,1041	303,28		7,81	286,5039	121,177	2311,02	cooling	7,81	32,9667	8,7628	338,33	
7,812	26,5807	1,7395	347,24	cooling	7,812	37,014	13,3337	331,02		7,812	293,8404	121,4435	2409,85	cooling	7,812	31,9765	14,9905	237,44	
7,814	26,4763	1,9241	343,2	cooling	7,814	37,3556	14,7016	316,67		7,814	301,808	144,4604	2199,49	cooling	7,814	31,5277	8,1142	327,29	heating
7,816	26,9905	5,0752	306,34	cooling	7,816	37,0333	11,1581	361,7		7,816	309,0386	180,1546	1801,61	cooling	7,816	33,0426	9,9646	322,6	
7,818	25,959	2,8468	323,07	cooling	7,818	37,5361	14,3028	324,77		7,818	318,734	154,5336	2295,28	cooling	7,818	31,7884	7,9614	333,07	
7,82	25,8781	2,8026	322,56	cooling	7,82	37,09	16,213	291,83		7,82	321,4375	150,7962	2385,31	cooling	7,82	31,7726	7,7104	336,35	
7,822	25,7216	3,5709	309,63	cooling	7,822	37,2609	16,0454	296,56		7,822	315,5162	137,1332	2493,53	cooling	7,822	31,817	12,1942	274,3	
7,824	25,7597	0,9611	346,65	cooling	7,824	37,2804	18,7604	258,88		7,824	317,3542	145,6533	2400,12	cooling	7,824	31,5708	13,6447	250,58	
7,826	25,8991	2,9223	321,18	cooling	7,826	36,9635	16,313	288,66		7,826	314,9693	152,4065	2272,39	cooling	7,826	30,9925	9,2303	304,2	
7,828	25,3613	2,0281	326,16	cooling	7,828	36,82	11,4444	354,71		7,828	308,1253	140,0122	2349,97	cooling	7,828	32,3923	12,9706	271,49	
7,83	26,451	2,7759	330,94	cooling	7,83	36,9797	12,3285	344,59		7,83	306,72	131,0376	2455,78	cooling	7,83	31,0062	10,5413	286,07	
7,832	25,5142	4,139	298,79	cooling	7,832	36,6262	14,2256	313,13		7,832	312,2196	143,4821	2358,7	cooling	7,832	31,5964	9,1458	313,83	
7,834	25,3139	1,9094	327,16	cooling	7,834	37,1663	17,976	268,25		7,834	313,7397	161,677	2125,61	cooling	7,834	31,6868	12,1832	272,63	
7,836	25,4736	1,7101	332,18	cooling	7,836	37,1182	22,818	199,9		7,836	313,4638	165,1221	2073,6	cooling	7,836	32,2012	8,2045	335,44	
7,838	25,8429	-0,2602	364,88	cooling	7,838	36,9071	16,8792	279,96		7,838	319,2204	158,675	2244,19	cooling	7,838	31,8872	8,4596	327,48	
7,84	24,8927	-3,9754	403,53	cooling	7,84	37,7962	18,2106	273,78		7,84	323,8767	122,1021	2820,51	cooling	7,84	31,8904	7,1564	345,75	
7,842	25,7363	0,8114	348,41	cooling	7,842	37,3566	19,471	250,01		7,842	324,745	129,8108	2724,89	cooling	7,842	32,9116	7,1107	360,66	
7,844	24,7275	2,9285	304,72	cooling	7,844	37,0644	17,8017	269,26		7,844	318,1109	131,9039	2602,9	cooling	7,844	32,8075	9,2721	328,99	
7,846	25,0011	2,3624	316,46	cooling	7,846	37,2585	16,9793	283,47		7,846	322,047	149,1736	2416,51	cooling	7,846	31,9025	12,5043	271,16	
7,848	24,8434	5,2617	273,72	cooling	7,848	37,3527	18,1803	268		7,848	312,1095	147,1636	2305,7	cooling	7,848	31,6938	8,5202	323,93	
7,85	25,2448	2,3287	320,33	cooling	7,85	37,0333	16,3313	289,38		7,85	312,3363	150,8352	2257,55	cooling	7,85	32,6223	10,1612	313,97	
7,852	25,2383	4,3124	292,51	cooling	7,852	37,4474	19,4537	251,53		7,852	304,1728	129,4419	2442,48	cooling	7,852	30,834	5,4406	354,96	
7,854	24,3257	5,8202	258,68	cooling	7,854	36,7359	16,0295	289,44		7,854	304,5729	122,8518	2540,19	cooling	7,854	31,7856	7,4981	339,5	
7,856	24,1142	2,3772	303,85	cooling	7,856	37,0853	13,0788	335,57		7,856	307,6822	114,5013	2700,38	cooling	7,856	31,1247	10,929	282,31	
7,858	24,7298	3,6135	295,18	cooling	7,858	36,9283	15,0601	305,68		7,858	312,2295	121,7293	2662,91	cooling	7,858	30,9169	10,136	290,49	
7,86	24,5926	6,8787	247,61	cooling	7,86	37,1441	14,7431	313,13		7,86	311,8543	144,1818	2343,81	cooling	7,86	30,7746	11,4355	270,33	
7,862	23,9692	2,9751	293,47	cooling	7,862	37,1972	15,6628	301,02		7,862	311,7352	131,5143	2519,22	cooling	7,862	31,7182	14,6177	239,04	
7,864	24,9656	6,1782	262,62	cooling	7,864	37,0389	14,4304	316,03		7,864	318,0444	117,2227	2807,19	cooling	7,864	30,8417	14,6389	226,49	
7,866	23,5933	3,5001	280,87	cooling	7,866	37,3133	14,7147	315,9		7,866	320,8291	114,5516	2883,45	cooling	7,866	30,9535	15,0317	222,56	
7,868	24,1417	2,4772	302,84	cooling	7,868	38,1145	18,5217	273,88		7,868	314,931	163,8201	2112,31	cooling	7,868	31,7084	16,5579	211,78	
7,87	23,7864	3,3778	285,28	cooling	7,87	37,7808	19,9541	249,19		7,87	310,797	150,761	2237,07	cooling	7,87	32,1551	13,1719	265,36	heating

7,872	24,0813	-0,0471	337,28	cooling	7,872	38,0828	17,8385	282,98		7,872	312,2672	139,3473	2417,16	cooling	7,872	31,9566	16,0829	221,89	heating
7,874	24,3221	-1,4417	360,14	cooling	7,874	38,3408	20,7122	246,42		7,874	303,21	97,0821	2881,36	cooling	7,874	31,6525	13,3331	256,08	heating
7,876	24,1291	1,9021	310,7	cooling	7,876	38,1379	18,6431	272,51		7,876	301,5975	103,3317	2771,46	cooling	7,876	33,327	13,8132	272,77	heating
7,878	23,7557	0,0825	330,92	cooling	7,878	37,8624	18,0997	276,25		7,878	303,0795	157,2196	2038,91	cooling	7,878	32,2097	11,1498	294,39	heating
7,88	24,2611	-0,0887	340,37	cooling	7,88	37,9346	18,0835	277,49	heating	7,88	301,5525	164,3771	1917,51	cooling	7,88	32,0752	11,4048	288,94	heating
7,882	24,7982	0,8808	334,33	cooling	7,882	38,0493	16,6529	299,09	heating	7,882	304,8554	143,9489	2249,23	cooling	7,882	31,8596	11,6873	281,98	heating
7,884	24,6127	-2,7614	382,65	cooling	7,884	37,7778	18,4425	270,28	heating	7,884	311,4316	159,3962	2125,23	cooling	7,884	31,948	13,9517	251,56	heating
7,886	25,5405	1,6706	333,67	cooling	7,886	37,6719	20,5377	239,51	heating	7,886	307,1026	177,4702	1812,07	cooling	7,886	30,435	11,2498	268,18	heating
7,888	24,5461	1,0554	328,36	cooling	7,888	37,5363	16,2159	298,03	heating	7,888	305,8602	135,1253	2386,62	cooling	7,888	31,5536	12,1907	270,66	heating
7,89	25,1963	1,0301	337,81	cooling	7,89	38,4573	16,4708	307,34	heating	7,89	312,8724	146,6956	2322,91	cooling	7,89	31,2363	11,4788	276,18	heating
7,892	24,5583	-0,554	351,03	cooling	7,892	37,7896	16,8362	292,9	heating	7,892	325,417	132,7049	2693,83	cooling	7,892	31,3287	9,1103	310,58	heating
7,894	25	-0,8075	360,75	cooling	7,894	38,6899	17,929	290,21	heating	7,894	316,1934	127,8031	2633,42	cooling	7,894	31,96	13,2616	261,38	heating
7,896	24,7188	-0,4642	352,02	cooling	7,896	38,5805	22,2875	227,75	heating	7,896	313,4391	140,9526	2411,1	cooling	7,896	32,5546	13,1367	271,43	heating
7,898	24,7972	3,9876	290,89	cooling	7,898	38,4071	21,7402	232,98	heating	7,898	309,4422	145,8102	2287,33	cooling	7,898	32,104	14,9901	239,23	heating
7,9	24,0353	1,5988	313,63	cooling	7,9	37,902	17,9555	278,82	heating	7,9	307,0296	105,9645	2810,59	cooling	7,9	31,8182	16,8075	209,83	heating
7,902	24,9049	2,4106	314,44	cooling	7,902	38,8079	17,7662	294,13	heating	7,902	315,7762	91,512	3134,88	cooling	7,902	33,0109	20,2721	178,07	heating
7,904	24,8258	1,9967	319,12	cooling	7,904	37,7983	16,8338	293,05	heating	7,904	324,4491	131,6711	2694,75	cooling	7,904	32,6228	16,7657	221,66	heating
7,906	24,9845	1,3834	329,91	cooling	7,906	37,8683	16,9275	292,72	heating	7,906	342,1233	95,7229	3444,31	cooling	7,906	32,1253	18,2243	194,32	heating
7,908	25,6524	3,6045	308,2	cooling	7,908	37,8916	21,3045	231,86	heating	7,908	341,1864	175,9341	2309,98	cooling	7,908	31,7457	12,2456	272,58	heating
7,91	25,5608	2,8807	317,03	cooling	7,91	37,658	19,555	253,05	heating	7,91	333,8403	157,3886	2466,53	cooling	7,91	33,6619	12,3771	297,53	heating
7,912	25,8835	4,4338	299,83	cooling	7,912	37,7321	18,955	262,48	heating	7,912	330,8833	108,6499	3106,49	cooling	7,912	32,0816	10,9855	294,89	heating
7,914	25,5042	6,8517	260,73	cooling	7,914	37,6756	15,3952	311,45	heating	7,914	334,7074	126,1948	2914,7	cooling	7,914	32,7039	12,4819	282,67	heating
7,916	26,4044	8,9454	244,05	cooling	7,916	38,7208	12,4722	366,92	heating	7,916	323,7	91,9235	3239,89	cooling	7,916	33,0036	14,4522	259,32	heating
7,918	25,786	3,9074	305,83	cooling	7,918	38,666	15,2559	327,24	heating	7,918	310,31	132,898	2479,96	cooling	7,918	33,1376	14,6345	258,65	heating
7,92	25,9346	4,1772	304,14	cooling	7,92	38,8906	21,1516	247,97	heating	7,92	326,7421	171,4692	2170,49	cooling	7,92	32,0931	11,5765	286,79	heating
7,922	25,3105	2,95	312,57	cooling	7,922	38,4638	20,6209	249,42	heating	7,922	319,7079	169,4472	2100,42	cooling	7,922	33,0556	13,237	277,03	heating
7,924	25,8945	7,7438	253,72	cooling	7,924	39,3131	20,8469	258,13	heating	7,924	310,064	123,6574	2605,69	cooling	7,924	32,2623	12,6991	273,46	heating
7,926	25,1043	6,1392	265,1	cooling	7,926	37,9739	14,6799	325,62	heating	7,926	330,6822	168,8691	2261,91	cooling	7,926	31,7365	8,0348	331,31	heating
7,928	25,3391	2,9152	313,45	cooling	7,928	38,7869	11,8526	376,5	heating	7,928	401,8751	152,8078	3481,59	cooling	7,928	32,8137	12,2055	288,07	heating
7,93	25,6829	-1,3265	377,55	cooling	7,93	38,1693	16,8914	297,43	heating	7,93	377,2981	259,6626	1644,37	cooling	7,93	33,3701	10,9225	313,78	heating
7,932	25,5295	0,6664	347,55	cooling	7,932	37,7366	18,0721	274,88	heating	7,932	369,6442	164,0519	2873,88	cooling	7,932	33,1574	14,1806	265,27	heating
7,934	25,2043	3,8565	298,41	cooling	7,934	37,4637	16,5221	292,73	heating	7,934	359,2437	161,4183	2765,31	cooling	7,934	32,4134	12,8314	273,73	heating
7,936	25,1818	5,4783	275,43	cooling	7,936	38,4789	16,0613	313,36	heating	7,936	433,3614	201,5494	3240,39	cooling	7,936	33,9747	13,9268	280,24	heating
7,938	25,4816	3,6687	304,91	cooling	7,938	38,1264	10,995	379,26	heating	7,938	556,0522	242,3987	4384,41	cooling	7,938	33,3971	12,5015	292,09	heating
7,94	24,8596	-1,1124	363,05	cooling	7,94	38,5849	11,5658	377,69		7,94	752,4059	488,8008	3684,81	cooling	7,94	33,1061	17,4197	219,27	heating
7,942	25,2383	-1,8659	378,88	cooling	7,942	38,793	18,1442	288,64							7,942	32,5887	16,3581	226,88	heating
7,944	25,0222	-1,0595	364,58	cooling	7,944	38,3192	16,947	298,75							7,944	33,5218	19,1724	200,58	heating
7,946	24,8373	1,1913	330,54	cooling	7,946	38,9438	21,0991	249,44							7,946	31,6402	15,6121	224,05	heating
7,948	24,4471	0,2835	337,77	cooling	7,948	38,0329	19,526	258,7							7,948	32,2394	11,9351	283,82	heating
7,95	25,3079	4,1912	295,18	cooling	7,95	38,6873	15,9227	318,22							7,95	32,7074	12,6333	280,61	heating
7,952	24,8864	0,7238	337,76	cooling	7,952	38,0437	18,8521	268,27							7,952	32,263	9,4847	318,41	heating
7,954	24,6359	0,5467	336,73	cooling	7,954	37,7526	20,3985	242,58							7,954	32,5833	11,7021	291,89	heating
7,956	24,5264	-0,2051	345,71	cooling	7,956	37,3972	18,5524	263,42							7,956	33,45	13,7383	275,54	heating
7,958	24,7827	1,3856	327,06	cooling	7,958	38,4067	22,3865	223,94	heating						7,958	33,0729	14,9956	252,69	heating
7,96	24,8489	2,7575	308,8	cooling	7,96	37,2731	16,6509	288,27	heating						7,96	32,6086	11,7403	291,71	heating
7,962	24,4655	6,0867	256,91	cooling	7,962	38,6652	15,0587	329,98	heating						7,962	33,8868	15,5035	256,97	heating
7,964	24,486	2,1004	312,92	cooling	7,964	38,1775	17,981	282,32	heating						7,964	33,973	10,3566	330,12	heating
7,966	24,539	0,2329	339,76	cooling	7,966	37,939	12,5387	355,06	heating						7,966	33,725	14,8219	264,24	heating
7,968	24,021	-0,4273	341,75	cooling	7,968	39,0081	14,5992	341,2							7,968	32,9516	13,4667	272,37	heating
7,97	23,8741	1,9066	307,07	cooling	7,97	39,0971	17,2502	305,39							7,97	34,8205	15,8425	265,28	heating
7,972	24,2974	7,7492	231,32	cooling	7,972	39,1929	15,6707	328,81							7,972	33,8312	13,6114	282,64	heating
7,974	23,6029	4,5855	265,83	cooling	7,974	39,1312	17,2397	306,01							7,974	33,3402	13,8087	273,02	heating
7,976	23,8207	0,8659	320,87	cooling	7,976	39,1549	16,698	313,91							7,976	33,3611	12,7048	288,74	heating
7,978	24,6779	0,3106	340,62	cooling	7,978	38,4604	11,7119	373,9							7,978	33,9022	13,2657	288,47	heating
7,98	24,6638	-1,0696	359,71	cooling	7,98	39,2912	17,482	304,86							7,98	32,3748	11,2872	294,77	heating
7,982	25,3386	-1,157	370,37	cooling	7,982	37,9827	14,3151	330,84							7,982	33,2738	13,7812	272,48	heating

7,984	25,9943	4,5327	300	cooling	7,984	39,1241	17,2586	305,65		7,984	33,0021	15,8055	240,38	heating
7,986	26,2684	0,3116	362,84	cooling	7,986	38,1492	17,6168	287,01		7,986	32,5439	11,4711	294,57	heating
7,988	26,4032	3,8868	314,75	cooling	7,988	38,3314	14,5093	333		7,988	32,8363	14,1433	261,3	heating
7,99	26,0171	6,0329	279,35	cooling	7,99	38,4971	13,6245	347,68		7,99	33,7808	10,0385	331,88	heating
7,992	26,1705	5,2099	293	cooling	7,992	38,5217	14,7399	332,43		7,992	33,7412	13,4766	283,27	heating
7,994	27,1694	7,3847	276,56	cooling	7,994	38,487	12,7859	359,26		7,994	33,0366	13,2321	276,84	heating
7,996	26,1172	5,1285	293,39	cooling	7,996	39,0114	18,5686	285,76		7,996	34,9161	18,3598	231,43	heating
7,998	26,4332	2,2628	337,87	cooling	7,998	38,1887	16,4553	303,8		7,998	34,8461	14,2842	287,42	heating
8	26,601	6,7388	277,64	cooling	8	38,0757	10,9868	378,66		8	34,2431	16,2713	251,22	heating
8,002	25,5518	4,7012	291,46	cooling	8,002	38,8702	16,3149	315,29	cooling	8,002	33,4434	9,8301	330,08	heating
8,004	25,8303	3,8347	307,47	cooling	8,004	38,0794	13,6181	341,93	cooling	8,004	35,4277	12,4962	320,55	heating
8,006	25,9244	7,4371	258,43	cooling	8,006	38,4962	17,0749	299,44	cooling	8,006	33,2574	9,4151	333,28	heating
8,008	25,7448	4,0978	302,59	cooling	8,008	37,8326	19,8641	251,17	cooling	8,008	33,6096	10,7773	319,16	heating
8,01	25,5491	7,0728	258,27	cooling	8,01	38,0522	17,0998	292,88		8,01	33,455	12,2309	296,68	heating
8,012	25,6806	9,3158	228,76		8,012	38,2639	13,7699	342,39		8,012	33,8842	12,0819	304,76	heating
8,014	25,092	2,0699	321,81		8,014	38,5746	19,0381	273,09		8,014	32,7936	13,5644	268,8	heating
8,016	26,2103	-0,1034	367,83		8,016	38,4243	17,8522	287,57		8,016	34,0063	12,1671	305,28	heating
8,018	25,3328	0,0527	353,38		8,018	39,28	25,4743	192,98		8,018	33,6457	9,3787	339,22	heating
8,02	26,3837	2,1623	338,58	cooling	8,02	37,9472	21,594	228,59		8,02	33,6747	4,9392	401,68	heating
8,022	26,0894	8,3491	247,98	cooling	8,022	37,9695	13,0087	348,91		8,022	34,3445	9,9045	341,64	heating
8,024	25,4704	1,9531	328,74		8,024	38,5988	14,5196	336,59		8,024	35,2132	7,6142	385,79	
8,026	25,8325	-3,0422	403,63	cooling	8,026	37,6708	14,229	327,68		8,026	34,8911	15,166	275,73	
8,028	26,4153	1,572	347,27	cooling	8,028	38,1146	17,5966	286,81		8,028	33,7283	12,2436	300,33	
8,03	25,2949	-2,1192	383,21	cooling	8,03	38,2754	23,5463	205,89		8,03	36,0187	15,453	287,48	
8,032	25,7213	-1,0694	374,49	cooling	8,032	37,8097	22,7975	209,85		8,032	34,8333	10,4271	341,16	
8,034	25,9908	2,8519	323,45	cooling	8,034	37,799	16,1279	302,93		8,034	34,5111	11,4422	322,47	
8,036	25,2003	-4,0172	408,42	cooling	8,036	38,4618	18,987	272,23		8,036	33,7387	9,826	334,26	
8,038	26,1884	-1,408	385,76	cooling	8,038	38,3984	15,1517	324,96		8,038	35,2106	14,9791	282,81	
8,04	25,2568	1,8991	326,51	cooling	8,04	38,3979	20,5138	249,99		8,04	32,868	12,5273	284,33	
8,042	25,9099	0,362	357,12	cooling	8,042	37,7346	20,4763	241,25		8,042	34,0865	12,4978	301,78	
8,044	25,4747	5,0267	285,83	cooling	8,044	38,0215	19,24	262,54		8,044	33,1696	11,5581	302,1	
8,046	25,032	4,0917	292,71	cooling	8,046	38,2598	16,5498	303,47	heating	8,046	33,3602	7,6104	359,94	
8,048	24,3506	-2,9187	381,18	cooling	8,048	37,5137	14,507	321,6		8,048	33,0724	9,9475	323,25	
8,05	25,1518	2,023	323,31	cooling	8,05	38,0806	13,9491	337,32		8,05	34,4631	8,2937	365,81	
8,052	23,3679	-1,2389	343,97	cooling	8,052	38,0297	19,6144	257,42		8,052	34,0669	11,83	310,84	
8,054	24,9115	-2,0348	376,67	cooling	8,054	37,2409	19,9139	242,21		8,054	33,5555	9,9876	329,44	
8,056	24,9161	6,1636	262,13	cooling	8,056	37,4506	19,2212	254,82	heating	8,056	35,1601	15,8605	269,78	
8,058	24,2539	0,9158	326,23	cooling	8,058	37,7068	20,0329	247,05	heating	8,058	34,9741	9,6104	354,55	
8,06	25,2406	0,5889	344,59	cooling	8,06	37,4727	17,019	285,91	heating	8,06	34,6317	11,5485	322,67	
8,062	25,8595	7,2439	260,22	cooling	8,062	37,6275	22,6854	208,87	heating	8,062	33,6059	6,9343	372,83	
8,064	24,9489	-0,1232	350,47	cooling	8,064	37,4217	21,1445	227,53	heating	8,064	35,8727	13,0525	318,99	cooling
8,066	25,4952	1,2025	339,58	cooling	8,066	37,1665	17,6016	273,49	heating	8,066	33,7877	11,2639	314,85	cooling
8,068	25,1591	5,9467	268,56	cooling	8,068	37,8497	18,9454	264,25	heating	8,068	34,014	16,2533	248,27	cooling
8,07	24,2542	0,7655	328,34	cooling	8,07	37,4863	17,2534	282,83	heating	8,07	33,0445	14,042	265,63	
8,072	25,2593	7,4923	248,36	cooling	8,072	38,6985	13,7139	349,25	heating	8,072	34,8067	14,4387	284,71	
8,074	24,0847	4,1673	278,42	cooling	8,074	38,1944	18,8553	270,33	heating	8,074	32,7524	10,267	314,31	
8,076	25,649	-4,116	416,07	cooling	8,076	37,3467	14,1167	324,72	heating	8,076	33,9351	9,9153	335,76	
8,078	25,7767	3,5888	310,15	cooling	8,078	38,2825	15,1025	324,02	heating	8,078	33,1367	10,7249	313,28	
8,08	25,5096	4,1797	298,16	cooling	8,08	38,1724	17,9338	282,91	heating	8,08	33,1989	9,4899	331,42	
8,082	25,8722	2,2406	330,34	cooling	8,082	37,7247	14,3173	327,2	heating	8,082	33,4558	14,5868	263,76	
8,084	27,3227	9,4316	250,09	cooling	8,084	38,1941	17,9203	283,4	heating	8,084	34,4108	12,8641	301,19	
8,086	25,7041	1,0316	344,89	cooling	8,086	38,1978	21,0855	239,2	heating	8,086	34,161	14,0302	281,4	
8,088	27,7557	0,6839	378,42	cooling	8,088	37,3235	16,2798	294,16	heating	8,088	33,4198	7,6335	360,45	
8,09	27,0704	7,6314	271,73	cooling	8,09	39,0026	21,8276	240,08	heating	8,09	35,2965	12,5394	318,11	
8,092	26,9692	1,7376	352,7	cooling	8,092	37,7261	21,6141	225,22	heating	8,092	34,3756	6,1272	394,87	
8,094	27,9846	3,8854	336,87	cooling	8,094	38,9108	17,3415	301,51	heating	8,094	34,6964	9,8784	346,92	cooling

8,096	27,9738	6,6102	298,63	cooling	8,096	38,0236	20,3231	247,43	heating	8,096	33,3987	6,8329	371,35	cooling
8,098	27,2738	0,0736	380,22	cooling	8,098	37,7839	17,5374	283,02	heating	8,098	35,3359	11,1043	338,72	cooling
8,1	27,5462	5,0771	314,08	cooling	8,1	37,3537	14,6708	317,07	heating	8,1	33,181	10,9084	311,34	cooling
8,102	26,8469	5,0538	304,64	cooling	8,102	37,7453	18,2366	272,7	heating	8,102	33,7457	16,0932	246,76	cooling
8,104	26,5602	-1,7011	395,05	cooling	8,104	37,1534	18,0553	266,96	heating	8,104	32,6905	14,4958	254,33	cooling
8,106	27,6732	4,9759	317,27	cooling	8,106	38,0579	18,8676	268,25	heating	8,106	33,611	14,3232	269,62	cooling
8,108	26,3102	5,2451	294,46	cooling	8,108	37,5693	20,3254	241,04	heating	8,108	32,3885	12,005	284,93	cooling
8,11	27,7463	3,5062	338,84	cooling	8,11	37,3192	14,0505	325,26	heating	8,11	33,9564	9,9191	336,01	
8,112	26,5826	3,8327	318,01	cooling	8,112	38,6002	16,7907	304,86	heating	8,112	33,2814	12,0715	296,48	cooling
8,114	26,8349	-3,0366	417,56	cooling	8,114	38,0387	17,8839	281,73	heating	8,114	32,9165	7,5136	355,09	
8,116	26,847	-3,6387	426,14	cooling	8,116	38,6176	19,5737	266,21	heating	8,116	34,251	13,9241	284,14	cooling
8,118	27,1807	4,7384	313,71	cooling	8,118	38,0495	20,9498	239,03	heating	8,118	34,2472	9,7262	342,77	cooling
8,12	25,9888	3,3323	316,7	cooling	8,12	37,9263	17,1989	289,74	heating	8,12	34,0961	12,3968	303,32	cooling
8,122	27,0716	3,2308	333,26	cooling	8,122	37,2452	12,6064	344,41	heating	8,122	33,3349	9,9493	326,9	cooling
8,124	26,136	1,3576	346,36	cooling	8,124	38,0026	16,566	299,65	heating	8,124	34,701	14,978	275,7	cooling
8,126	25,8628	-3,8006	414,65	cooling	8,126	37,1212	16,6964	285,51	heating	8,126	33,0959	8,7368	340,5	cooling
8,128	26,2909	-1,5553	389,25	cooling	8,128	37,8908	18,7404	267,69	heating	8,128	33,9618	13,7985	281,85	cooling
8,13	26,5553	1,3757	351,97	cooling	8,13	37,5645	21,2095	228,62	heating	8,13	32,4962	7,5579	348,6	cooling
8,132	27,4359	3,3631	336,5	cooling	8,132	38,0153	16,7779	296,87	heating	8,132	34,1452	9,7725	340,69	cooling
8,134	27,527	7,5514	279,23	cooling	8,134	38,3728	17,4614	292,31	heating	8,134	32,0936	8,7745	325,97	cooling
8,136	27,7682	6,3368	299,58	cooling	8,136	38,6445	19,2084	271,69	heating	8,136	33,239	9,0464	338,18	cooling
8,138	27,3962	2,2835	351,04	cooling	8,138	39,707	23,0477	232,87	heating	8,138	32,4145	10,1775	310,84	cooling
8,14	27,9923	5,625	312,66	cooling	8,14	39,4969	25,1741	200,21	heating	8,14	32,6249	10,0125	316,09	cooling
8,142	27,7408	5,8898	305,44	cooling	8,142	39,0507	21,9397	239,19	heating	8,142	32,4195	11,004	299,36	cooling
8,144	28,4056	9,2667	267,53	cooling	8,144	38,6105	14,2851	340,03	heating	8,144	33,914	12,6635	297,05	cooling
8,146	27,2932	9,2239	252,58	cooling	8,146	39,5139	17,3594	309,69	heating	8,146	32,7664	14,4481	256,06	cooling
8,148	27,1857	5,4891	303,29	cooling	8,148	38,1733	17,3993	290,39	heating	8,148	32,7943	8,8967	334,05	cooling
8,15	27,1028	3,6902	327,27	cooling	8,15	38,7378	19,5822	267,77	heating	8,15	34,1834	14,2998	277,94	cooling
8,152	26,6968	1,3554	354,23	cooling	8,152	37,883	18,7958	266,81	heating	8,152	33,2706	6,8091	369,89	cooling
8,154	27,4191	2,9206	342,45	cooling	8,154	38,098	15,0107	322,73	heating	8,154	33,7474	11,6819	308,44	cooling
8,156	27,4531	4,3915	322,37	cooling	8,156	37,8844	13,7409	337,49	heating	8,156	32,4898	8,8518	330,42	cooling
8,158	27,4777	6,8625	288,17	cooling	8,158	38,9745	16,7114	311,2	heating	8,158	34,0976	12,798	297,74	cooling
8,16	27,0918	3,556	329	cooling	8,16	38,5976	15,4168	324,03	heating	8,16	32,5884	8,939	330,58	cooling
8,162	27,6419	-0,0174	386,64	cooling	8,162	39,2071	18,1383	294,51	heating	8,162	32,7596	12,0489	289,5	cooling
8,164	27,5883	-3,8496	439,46	cooling	8,164	38,8785	20,212	260,93	heating	8,164	31,9476	7,4681	342,19	cooling
8,166	27,4699	1,9939	356,12	cooling	8,166	39,0795	16,19	319,96	heating	8,166	32,8718	10,7191	309,66	cooling
8,168	27,2834	5,0801	310,37	cooling	8,168	39,166	17,8357	298,17	heating	8,168	31,0936	8,5795	314,71	cooling
8,17	27,475	7,2379	282,89	cooling	8,17	38,3422	15,1149	324,68	heating	8,17	32,7305	10,973	304,14	cooling
8,172	27,1494	1,3173	361,09	cooling	8,172	39,1503	16,9641	310,13	heating	8,172	31,9512	13,3894	259,47	cooling
8,174	27,2936	-3,4421	429,64	cooling	8,174	38,718	19,1776	273,15	heating	8,174	32,0529	10,5909	300,01	cooling
8,176	27,831	-2,7164	427,01	cooling	8,176	38,2665	18,8146	271,91	heating	8,176	32,6933	14,3595	256,28	cooling
8,178	27,1392	2,8247	339,88	cooling	8,178	38,2973	14,8603	327,62	heating	8,178	33,3778	11,091	311,54	
8,18	27,0814	8,9105	254	cooling	8,18	39,0907	17,2273	305,62	heating	8,18	32,7689	12,5003	283,32	
8,182	27,0283	8,8646	253,9	cooling	8,182	38,3493	13,4822	347,6	heating	8,182	32,2474	7,1127	351,35	
8,184	27,5383	5,3267	310,49	cooling	8,184	38,996	17,0657	306,55		8,184	33,8073	12,4001	299,24	cooling
8,186	27,5486	0,198	382,32	cooling	8,186	38,5468	16,7691	304,42		8,186	32,7041	6,8897	360,85	
8,188	27,9716	2,316	358,63	cooling	8,188	39,1159	16,0172	322,89		8,188	32,751	13,8896	263,65	cooling
8,19	28,2168	3,5866	344,29	cooling	8,19	38,6972	18,0085	289,2		8,19	32,0248	10,5915	299,61	
8,192	27,7903	6,568	296,66	cooling	8,192	38,7459	17,713	294,01		8,192	33,4153	13,4104	279,64	
8,194	27,3823	2,8656	342,71	cooling	8,194	38,646	15,3448	325,72		8,194	31,7979	8,3295	328,05	
8,196	28,0045	4,7814	324,62	cooling	8,196	38,6688	15,7448	320,44		8,196	32,6481	10,4049	310,93	
8,198	28,1278	5,3454	318,46	cooling	8,198	38,0318	14,8871	323,53		8,198	31,9301	8,5463	326,87	
8,2	27,6164	2,9779	344,41	cooling	8,2	38,9589	15,796	323,78		8,2	32,542	10,8637	303,03	cooling
8,202	27,9577	-0,4332	396,86	cooling	8,202	38,6374	17,6642	293,17		8,202	31,3943	12,8433	259,32	
8,204	28,0725	0,9968	378,48	cooling	8,204	38,2678	10,451	388,84		8,204	32,8878	13,6461	268,97	
8,206	28,1496	3,0007	351,54	cooling	8,206	38,8933	12,6667	366,61		8,206	32,132	14,9933	239,57	

8,208	28,4298	7,1216	297,86	cooling	8,208	38,7757	14,4807	339,61		8,208	32,3151	10,898	299,38	
8,21	28,8226	8,7187	281,02	cooling	8,21	38,3961	15,3547	322,09		8,21	32,686	12,2909	285,09	
8,212	28,9262	4,9423	335,26	cooling	8,212	38,6212	17,1099	300,7		8,212	33,6199	8,3089	353,81	
8,214	28,5598	4,2807	339,39	cooling	8,214	38,797	16,3657	313,56		8,214	32,6071	10,4155	310,21	
8,216	28,9266	5,8049	323,21	cooling	8,216	38,5524	11,7575	374,55		8,216	32,4796	7,7029	346,34	
8,218	28,573	5,3454	324,69	cooling	8,218	38,4833	15,5175	321,03	cooling	8,218	33,6043	15,4128	254,29	
8,22	29,3482	2,0448	381,66	cooling	8,22	37,443	13,5021	334,66	cooling	8,22	32,5624	10,3688	310,23	
8,222	28,764	1,7676	377,37	cooling	8,222	38,0863	16,2031	305,89	cooling	8,222	32,9478	14,8057	253,6	
8,224	29,3412	4,2862	350,23	cooling	8,224	37,4552	22,2504	212,54	cooling	8,224	32,1016	10,5111	301,8	
8,226	28,5234	4,7544	332,26	cooling	8,226	37,4885	20,5188	237,21		8,226	33,0004	11,4498	301,25	
8,228	29,168	4,0417	351,23	cooling	8,228	37,6559	18,8935	262,27		8,228	31,6751	10,0932	301,68	
8,23	28,6869	3,5566	351,28	cooling	8,23	37,7315	17,0339	289,32		8,23	32,982	11,2421	303,89	
8,232	29,0588	3,6724	354,87	cooling	8,232	37,8243	13,7422	336,63		8,232	32,0611	10,7691	297,63	
8,234	28,4068	3,6922	345,47	cooling	8,234	39,0436	15,8308	324,48		8,234	32,3153	11,5242	290,63	
8,236	27,7179	0,4028	381,82	cooling	8,236	38,7431	19,1856	273,38		8,236	31,9819	11,4687	286,74	
8,238	27,622	-2,3211	418,56	cooling	8,238	38,7372	16,5164	310,61		8,238	33,242	11,3884	305,48	
8,24	28,0783	0,4536	386,15	cooling	8,24	39,3411	20,9089	257,65		8,24	32,118	14,3192	248,8	
8,242	28,2416	4,4814	332,13	cooling	8,242	38,7874	16,37	313,36		8,242	32,1649	7,8148	340,38	
8,244	27,8199	8,1852	274,46	cooling	8,244	38,9793	14,4114	343,42		8,244	33,8321	13,5813	283,08	
8,246	28,1894	9,6027	259,81	cooling	8,246	38,9107	15,0735	333,21		8,246	33,519	8,4178	350,88	
8,248	27,3885	1,7774	358,01	cooling	8,248	39,2603	15,4832	332,37		8,248	32,94	11,6799	297,18	
8,25	28,2694	-0,4353	401,25	cooling	8,25	39,2482	15,0434	338,35		8,25	33,0267	10,4369	315,77	
8,252	27,438	-1,2715	401,32	cooling	8,252	39,067	17,6008	300,07		8,252	34,0702	11,636	313,6	
8,254	28,5493	1,4702	378,52	cooling	8,254	38,5982	12,3368	367,1		8,254	32,6625	7,7717	347,94	
8,256	27,8212	7,1046	289,59	cooling	8,256	39,6764	15,3004	340,74	cooling	8,256	33,4436	14,2551	268,23	
8,258	27,6026	6,2984	297,8	cooling	8,258	38,5437	16,8136	303,75	cooling	8,258	32,4239	11,1168	297,84	
8,26	27,0647	1,6054	355,88	cooling	8,26	38,8344	16,8221	307,7	cooling	8,26	33,7381	16,1967	245,2	
8,262	27,825	4,2972	328,88	cooling	8,262	38,4162	19,1005	270,01	cooling	8,262	31,2945	13,5994	247,35	
8,264	26,8625	3,1345	331,68	cooling	8,264	38,0431	17,392	288,67		8,264	32,884	13,5788	269,86	
8,266	27,2422	3,8261	327,32	cooling	8,266	37,6907	16,3065	298,92		8,266	31,6507	14,3083	242,42	
8,268	27,0826	4,9083	309,96	cooling	8,268	38,1186	18,2197	278,16		8,268	31,6223	14,6795	236,84	
8,27	26,9103	0,7697	365,41	cooling	8,27	37,449	16,511	292,68		8,27	31,3639	16,4352	208,68	
8,272	27,5397	3,2429	339,63	cooling	8,272	37,7787	15,7406	308,06		8,272	33,0595	16,9154	225,67	heating
8,274	27,5117	5,9117	301,94	cooling	8,274	37,8788	19,5259	256,55		8,274	31,6333	15,9157	219,71	heating
8,276	28,0211	3,2475	346,3	cooling	8,276	37,9648	16,1162	305,41		8,276	32,1228	13,1026	265,87	heating
8,278	27,9445	2,3358	357,97	cooling	8,278	38,3318	16,8386	300,44		8,278	33,4867	18,4593	210,06	heating
8,28	28,3525	5,3704	321,26	cooling	8,28	38,0751	14,0562	335,75		8,28	33,6343	12,8157	291,01	heating
8,282	27,6596	4,5064	323,65	cooling	8,282	38,7828	13,5843	352,24		8,282	33,2096	17,3329	221,93	heating
8,284	28,5568	8,9525	274,04	cooling	8,284	38,4735	15,822	316,63		8,284	32,9394	14,2306	261,52	heating
8,286	27,4314	4,2977	323,37	cooling	8,286	38,2115	17,5119	289,35		8,286	34,826	16,3111	258,81	heating
8,288	28,0079	0,4208	385,63	cooling	8,288	37,9357	14,9362	321,5		8,288	33,2981	11,0741	310,66	heating
8,29	27,6418	1,7879	361,4	cooling	8,29	38,4713	15,7687	317,35		8,29	33,4514	13,1273	284,1	heating
8,292	27,4261	3,851	329,54	cooling	8,292	37,4727	12,3931	350,58		8,292	32,6312	9,8138	318,95	heating
8,294	27,1741	6,6272	287,21	cooling	8,294	38,2631	14,9859	325,38	cooling	8,294	33,8485	15,9661	249,97	heating
8,296	27,3358	9,4124	250,54	cooling	8,296	37,5679	18,0885	272,29	cooling	8,296	31,5206	12,9981	258,92	heating
8,298	27,1967	4,8641	312,18	cooling	8,298	37,5986	14,8299	318,27		8,298	33,6293	15,1595	258,18	heating
8,3	27,3559	2,7354	344,16	cooling	8,3	37,7969	15,0556	317,89		8,3	32,3445	15,5618	234,6	heating
8,302	27,2078	5,1083	308,92	cooling	8,302	38,3107	16,1126	310,3	cooling	8,302	32,408	12,3187	280,82	heating
8,304	26,5572	0,3603	366,19	cooling	8,304	38,3197	17,6118	289,47	cooling	8,304	32,7429	13,2903	271,92	heating
8,306	27,3967	2,611	346,47	cooling	8,306	38,8368	23,0728	220,36		8,306	34,2796	13,0675	296,51	heating
8,308	26,4892	1,1808	353,77	cooling	8,308	38,6862	25,2448	187,89		8,308	33,3348	16,1323	240,46	heating
8,31	26,87	-1,5852	397,76	cooling	8,31	38,8441	19,5311	269,97		8,31	33,3967	18,4305	209,2	heating
8,312	26,7696	1,323	355,71	cooling	8,312	38,9014	18,9682	278,64		8,312	34,7592	22,7488	167,89	heating
8,314	26,4009	2,7777	330,22	cooling	8,314	38,0851	13,9984	336,7		8,314	34,2826	13,2763	293,64	heating
8,316	25,8482	-1,432	381,34	cooling	8,316	38,8651	17,211	302,69		8,316	34,2576	17,7992	230,06	heating
8,318	26,8894	4,3913	314,49	cooling	8,318	38,2125	20,7826	243,64		8,318	33,0814	11,4309	302,64	heating

8,32	25,8452	2,8733	321,11	cooling	8,32	37,9748	22,1836	220,74		8,32	35,3207	15,9497	270,78	heating
8,322	26,2897	0,5805	359,38	cooling	8,322	37,9026	19,4956	257,3		8,322	32,9857	13,3089	275,05	heating
8,324	26,3524	1,4895	347,55	cooling	8,324	38,3862	16,9695	299,37		8,324	33,5643	12,9693	287,89	heating
8,326	26,4162	-2,873	409,42	cooling	8,326	37,8926	11,5995	367,54		8,326	33,0666	9,9654	322,92	heating
8,328	26,7083	-2,406	406,98	cooling	8,328	38,6916	16,6469	308,15		8,328	33,6714	13,8304	277,35	heating
8,33	26,7714	4,4323	312,27	cooling	8,33	37,8831	18,1759	275,48		8,33	32,0204	10,4212	301,92	heating
8,332	26,5768	4,1404	313,63	cooling	8,332	37,9981	16,7611	296,86		8,332	34,1524	17,0969	238,41	heating
8,334	27,1017	4,5337	315,47	cooling	8,334	38,0909	20,1171	251,25		8,334	32,3029	15,4148	236,07	heating
8,336	26,8558	4,6038	311,05	cooling	8,336	38,0406	17,9071	281,44		8,336	32,7587	10,9178	305,3	heating
8,338	26,6382	-0,5234	379,68	cooling	8,338	38,319	17,717	287,99		8,338	33,7393	16,7186	237,93	heating
8,34	28,1245	8,8303	269,7	cooling	8,34	37,9579	17,1234	291,23		8,34	33,9317	11,6689	311,2	heating
8,342	26,6706	10,8627	220,97	cooling	8,342	38,0827	15,2741	318,83		8,342	33,5377	12,3071	296,77	heating
8,344	27,2885	8,3016	265,41	cooling	8,344	38,3555	12,7959	357,29		8,344	33,2478	13,3382	278,31	heating
8,346	26,9433	8,2103	261,86	cooling	8,346	38,294	16,9327	298,6		8,346	34,2589	15,0345	268,73	heating
8,348	26,6639	4,2548	313,25	cooling	8,348	37,7221	14,1496	329,51		8,348	33,7915	12,0712	303,62	heating
8,35	26,055	0,7388	353,88	cooling	8,35	38,8509	19,4789	270,79		8,35	33,3991	15,7161	247,18	heating
8,352	27,2917	10,1269	239,94	cooling	8,352	38,1492	18,5397	274,11		8,352	33,0008	8,1364	347,57	heating
8,354	26,124	6,1118	279,74	cooling	8,354	38,3183	15,9605	312,53		8,354	34,6436	15,7646	263,9	heating
8,356	27,2852	7,2081	280,65	cooling	8,356	38,6832	15,8386	319,33		8,356	32,0106	11,5468	286,05	heating
8,358	26,5584	7,7996	262,22	cooling	8,358	39,0383	18,0535	293,34		8,358	33,3558	13,3391	279,8	heating
8,36	26,6855	-0,506	380,1	cooling	8,36	38,8426	17,8221	293,84		8,36	32,9481	14,672	255,47	heating
8,362	26,7927	-3,0597	417,29	cooling	8,362	39,305	22,9157	229,1		8,362	32,9395	12,4671	286,17	heating
8,364	26,486	0,1023	368,8	cooling	8,364	38,6288	21,1181	244,77		8,364	33,0307	13,1145	278,4	heating
8,366	26,3709	-1,2868	386,61	cooling	8,366	38,7366	17,9742	290,23		8,366	34,6543	19,2443	215,41	heating
8,368	26,9911	5,4042	301,75	cooling	8,368	38,5518	19,4679	266,77		8,368	33,022	16,3503	233,05	heating
8,37	26,3051	4,2572	308,2	cooling	8,37	38,4879	18,3152	281,98		8,37	33,3299	14,2368	266,89	heating
8,372	25,951	-5,9187	445,49	cooling	8,372	38,3537	18,3633	279,44		8,372	33,6489	15,7567	250,11	heating
8,374	27,0204	-4,4094	439,34	cooling	8,374	37,9639	13,093	347,66	heating	8,374	33,735	9,9677	332,23	heating
8,376	25,6401	-4,5019	421,34	cooling	8,376	38,5288	12,2786	366,94		8,376	33,6176	16,2559	242,69	heating
8,378	26,463	-0,7829	380,86	cooling	8,378	38,8608	15,1212	331,84		8,378	32,8747	13,6847	268,25	heating
8,38	26,0651	3,7697	311,66	cooling	8,38	38,8602	21,2251	246,51		8,38	34,1842	15,2484	264,7	heating
8,382	26,2767	2,474	332,73	cooling	8,382	38,655	18,9756	275,09		8,382	33,3904	13,592	276,75	heating
8,384	25,4416	0,9114	342,9	cooling	8,384	39,5349	20,9457	259,85		8,384	33,3786	13,7622	274,21	heating
8,386	25,6146	4,6916	292,47	cooling	8,386	38,8261	17,7457	294,67		8,386	33,8215	11,16	316,78	heating
8,388	24,9013	0,5859	339,89	cooling	8,388	38,9039	18,512	285,05		8,388	34,1896	14,889	269,79	heating
8,39	25,9046	4,6972	296,45	cooling	8,39	38,3626	17,4	293,03		8,39	32,4906	9,1377	326,44	heating
8,392	25,1	4,7658	284,24	cooling	8,392	38,8219	19,7462	266,65		8,392	33,7836	12,4081	298,8	heating
8,394	25,832	2,7226	323,04	cooling	8,394	38,0746	20,3226	248,15		8,394	33,1087	12,317	290,64	heating
8,396	26,2047	6,4829	275,68	cooling	8,396	38,217	20,0786	253,55	heating	8,396	32,8697	11,1022	304,28	heating
8,398	25,2581	5,6953	273,46	cooling	8,398	38,105	16,7261	298,84	heating	8,398	32,8465	13,7119	267,47	heating
8,4	25,5485	2,3941	323,66	cooling	8,4	38,4552	15,2524	324,34	heating	8,4	33,9546	13,1277	291,13	heating
8,402	25,6875	2,6707	321,74	cooling	8,402	38,1914	16,2988	306,03	heating	8,402	33,2353	12,4186	290,99	heating
8,404	25,6179	0,0899	356,84	cooling	8,404	38,8271	18,7906	280,08		8,404	33,3589	11,4612	306,1	heating
8,406	25,1065	0,173	348,53	cooling	8,406	38,2905	20,3766	250,41	heating	8,406	34,2891	14,5661	275,7	heating
8,408	25,1809	3,9171	297,24	cooling	8,408	37,7559	13,5147	338,86	heating	8,408	34,8624	12,074	318,55	heating
8,41	23,6767	-3,482	379,64	cooling	8,41	38,017	12,5775	355,61		8,41	34,0241	15,4652	259,43	heating
8,412	24,2072	-1,7619	363,01	cooling	8,412	37,9524	13,6658	339,49		8,412	33,8163	11,0304	318,51	heating
8,414	22,9287	-1,3216	338,98	cooling	8,414	37,8014	17,4958	283,84		8,414	34,6648	10,6829	335,23	heating
8,416	23,7271	1,5573	309,9	cooling	8,416	37,9016	17,8678	280,04		8,416	34,1721	7,3679	374,68	heating
8,418	23,1218	4,4963	260,36	cooling	8,418	38,919	18,0113	292,26		8,418	33,9171	10,8377	322,62	heating
8,42	22,8689	1,3263	301,13	cooling	8,42	38,9117	12,5028	369,16		8,42	34,0674	8,7972	353,24	heating
8,422	22,8415	-6,4969	410,11	cooling	8,422	39,4528	15,2559	338,24		8,422	34,5946	12,1102	314,3	
8,424	23,2469	-3,912	379,64	cooling	8,424	38,8797	15,6381	324,88		8,424	33,1034	7,8008	353,69	
8,426	22,4911	-4,2024	373,14	cooling	8,426	39,2488	17,0342	310,53		8,426	34,3222	10,0131	339,81	
8,428	23,0306	0,501	314,93	cooling	8,428	37,9753	16,0433	306,58		8,428	33,9313	12,3847	301,19	
8,43	23,0198	5,5369	244,38	cooling	8,43	37,947	17,5534	285,07		8,43	33,7051	10,7458	320,94	

8,432	22,3695	0,7618	302,04	cooling	8,432	37,3706	16,1297	296,92		8,432	33,3522	10,3828	321,08	
8,434	22,8288	-0,4677	325,65	cooling	8,434	37,3414	13,0754	339,2		8,434	34,6366	10,8597	332,36	
8,436	22,3909	-0,088	314,22	cooling	8,436	37,1865	12,8923	339,6		8,436	33,416	8,9408	342,13	
8,438	22,4024	0,3441	308,34	cooling	8,438	37,7358	15,7135	307,84		8,438	33,9259	10,0138	334,26	
8,44	22,2451	2,6784	273,51	cooling	8,44	37,7318	18,8432	264,03		8,44	34,2307	13,4831	290,02	
8,442	22,2025	4,5987	246,07	cooling	8,442	37,5165	16,6245	292,04		8,442	34,3913	10,1221	339,25	
8,444	22,0799	1,1838	292,1	cooling	8,444	38,0161	17,5167	286,55		8,444	34,0456	14,13	278,39	
8,446	22,4772	4,1813	255,75	cooling	8,446	38,2946	16,7842	300,68		8,446	33,5614	11,242	311,99	
8,448	21,8815	1,296	287,75	cooling	8,448	38,4551	19,5747	263,92		8,448	34,5871	12,687	306,13	
8,45	22,568	0,5995	307,09	cooling	8,45	38,0281	14,5998	327,49		8,45	33,6075	11,0312	315,58	
8,452	22,8849	3,5493	270,28	cooling	8,452	39,0703	15,6184	327,82		8,452	33,5188	9,7217	332,65	
8,454	22,7011	2,4817	282,64	cooling	8,454	38,4438	13,2174	352,63		8,454	33,6393	8,3858	353,01	
8,456	23,1798	-0,1046	325,48	cooling	8,456	38,7152	15,4039	325,86		8,456	33,5654	9,5802	335,28	
8,458	23,2003	0,1202	322,63	cooling	8,458	38,5355	18,5804	278,94		8,458	32,5474	6,2169	368,06	
8,46	22,5972	-3,4373	363,92	cooling	8,46	38,5811	19,1848	271,13		8,46	34,0671	10,7259	326,28	cooling
8,462	22,9827	-1,555	343	cooling	8,462	37,8443	14,9241	320,39		8,462	33,4047	12,7456	288,78	cooling
8,464	22,7313	2,0419	289,21	cooling	8,464	38,2603	16,7771	300,3		8,464	33,3868	10,5066	319,83	cooling
8,466	22,913	1,5183	299,07	cooling	8,466	37,4261	13,8408	329,69		8,466	33,7157	12,7919	292,48	cooling
8,468	23,0883	2,3232	290,27	cooling	8,468	37,9956	13,4682	342,86		8,468	34,4398	10,6291	332,84	cooling
8,47	23,0837	-0,5896	330,92	cooling	8,47	37,7935	16,4469	298,39		8,47	33,5501	11,8803	302,91	cooling
8,472	23,1972	-4,0367	380,69	cooling	8,472	38,5441	14,8302	331,48		8,472	33,4383	13,4029	280,06	cooling
8,474	23,6153	-2,671	367,44	cooling	8,474	39,1466	18,4183	289,75		8,474	33,9021	15,6446	255,21	cooling
8,476	23,2309	-2,6223	361,39	cooling	8,476	39,2056	19,1205	280,76		8,476	34,0564	14,0669	279,42	
8,478	23,8437	0,1495	331,21	cooling	8,478	38,9733	17,4043	301,5		8,478	33,1716	16,4071	234,34	
8,48	23,6976	5,6836	251,81	cooling	8,48	39,7597	19,955	276,84		8,48	33,1444	11,6021	301,13	
8,482	23,1873	1,943	296,96	cooling	8,482	38,2652	18,832	271,65		8,482	34,341	12,5101	305,16	
8,484	23,6134	1,0971	314,74	cooling	8,484	37,8822	12,3046	357,54		8,484	33,9136	9,7083	338,35	
8,486	23,3899	-0,507	334,04	cooling	8,486	38,1723	15,325	319,37		8,486	33,6219	12,2801	298,33	
8,488	23,5208	-4,2703	388,48	cooling	8,488	37,544	14,4986	322,14		8,488	33,4787	10,5778	320,12	
8,49	23,9746	0,6999	325,35	cooling	8,49	37,9211	22,6241	213,83		8,49	34,2564	12,6307	302,3	
8,492	23,8359	5,8778	251,03	cooling	8,492	37,5473	26,694	151,71		8,492	32,2761	7,4364	347,22	
8,494	23,3855	2,4581	292,53	cooling	8,494	38,3745	23,5295	207,51		8,494	33,7266	11,2157	314,67	
8,496	24,1249	3,7455	284,87	cooling	8,496	38,4383	18,0414	285,12		8,496	32,6972	10,6344	308,41	
8,498	23,2813	-0,5843	333,61	cooling	8,498	38,7204	16,5383	310,07		8,498	33,1359	10,3626	318,34	
8,5	24,1397	-2,8388	377,12	cooling	8,5	38,5952	15,2474	326,37		8,5	32,1575	8,9542	324,35	
8,502	24,0147	4,0313	279,34	cooling	8,502	38,9902	20,722	255,36		8,502	33,6712	9,1092	343,34	
8,504	23,6658	6,1861	244,34	cooling	8,504	37,4231	19,4395	251,38		8,504	32,6999	7,5513	351,54	cooling
8,506	23,5835	4,319	269,29	cooling	8,506	38,186	17,2996	291,96	heating	8,506	33,0285	11,073	306,91	cooling
8,508	24,0271	3,0162	293,7	cooling	8,508	37,7785	16,8538	292,5	heating	8,508	33,3775	14,224	267,74	cooling
8,51	23,463	-3,8134	381,28	cooling	8,51	37,8966	12,835	350,32	heating	8,51	33,8388	11,8155	307,85	cooling
8,512	24,1583	-2,514	372,84	cooling	8,512	38,519	16,1269	313,01		8,512	33,1629	15,3672	248,76	cooling
8,514	23,7634	4,6368	267,36	cooling	8,514	38,7888	16,1904	315,89		8,514	32,4753	11,8186	288,75	
8,516	23,3655	5,1567	254,53	cooling	8,516	38,558	14,7374	332,98		8,516	33,9528	14,4593	272,49	
8,518	23,8429	5,9765	249,74	cooling	8,518	38,6792	13,3183	354,51		8,518	32,4234	11,4431	293,27	
8,52	23,5553	1,9575	301,91	cooling	8,52	39,0288	13,6023	355,42		8,52	32,9502	11,2825	302,88	
8,522	24,2937	-2,9313	380,57	cooling	8,522	38,681	12,8488	361,1		8,522	33,0547	12,819	282,87	
8,524	24,3907	0,5284	333,56	cooling	8,524	38,7062	20,3473	256,63		8,524	32,8545	13,2885	273,5	
8,526	24,2941	4,3701	278,51	cooling	8,526	37,7252	18,1213	274,03		8,526	32,1805	10,9428	296,87	
8,528	23,8787	5,6419	254,92	cooling	8,528	38,893	19,4106	272,34		8,528	33,5047	14,593	264,36	
8,53	24,2949	8,4212	221,89	cooling	8,53	38,0668	17,9415	281,32		8,53	32,8066	11,5177	297,59	
8,532	23,4581	1,2878	309,91		8,532	38,5433	15,9885	315,28		8,532	32,6896	8,7284	334,94	
8,534	24,51	-1,3156	361		8,534	38,5388	18,9903	273,26		8,534	33,5012	13,0296	286,16	
8,536	24,1192	0,9025	324,53		8,536	38,4418	20,6855	248,21		8,536	34,0537	10,0243	335,89	
8,538	23,8928	1,8572	308,02	cooling	8,538	37,7508	18,0742	275,05		8,538	33,6815	11,7468	306,61	
8,54	23,9101	4,7572	267,73	cooling	8,54	38,5125	19,6058	264,29		8,54	32,9858	10,2326	318,06	
8,542	23,6262	2,6571	293,12		8,542	37,9089	18,6269	269,53		8,542	34,2025	8,9003	353,69	

8,544	24,0061	-2,9751	377,16		8,544	37,8569	15,5575	311,71		8,544	33,7753	7,5101	367,15	
8,546	24,2837	-0,4862	346,25	cooling	8,546	37,9812	19,241	261,96		8,546	32,9567	12,0342	292,47	
8,548	24,0186	1,6798	312,26	cooling	8,548	37,7152	14,9342	318,44		8,548	32,46	10,6849	304,38	
8,55	23,9306	1,0196	320,26	cooling	8,55	38,1205	16,7819	298,28		8,55	33,6086	15,4911	253,26	
8,552	24,4675	4,8591	274,1	cooling	8,552	37,9176	17,5728	284,39		8,552	31,7443	10,539	296,42	
8,554	23,4745	-0,3189	332,6	cooling	8,554	38,0049	18,0996	278,25		8,554	32,6718	9,2868	326,89	
8,556	24,2815	-1,0805	354,52	cooling	8,556	37,6553	16,5961	294,38		8,556	32,7857	11,3441	299,72	
8,558	23,7443	2,3033	299,71	cooling	8,558	36,963	15,7558	296,44		8,558	32,4394	10,822	302,18	
8,56	23,479	2,7523	289,73	cooling	8,56	36,293	10,9577	354,15		8,56	32,786	13,0202	276,3	
8,562	23,3056	3,0556	283,07	cooling	8,562	36,7302	14,1187	316,07		8,562	33,7148	14,1539	273,43	
8,564	23,7442	5,1286	260,22	cooling	8,564	36,03	16,0691	279,02		8,564	33,3133	11,6059	303,44	
8,566	23,1953	-0,8505	336,13		8,566	36,2972	18,5787	247,68		8,566	32,9765	10,8309	309,56	
8,568	23,5962	0,1342	327,96	cooling	8,568	35,841	18,6772	239,92		8,568	33,9586	15,4898	258,17	
8,57	22,9635	0,3913	315,53	cooling	8,57	36,1806	16,8491	270,23		8,57	33,8942	11,1388	318,09	
8,572	23,0954	-0,7187	332,89	cooling	8,572	35,9944	15,7736	282,66	heating	8,572	33,8692	15,7601	253,14	
8,574	23,2007	1,9447	297,13	cooling	8,574	36,8704	17,0098	277,62	heating	8,574	32,783	14,1696	260,19	
8,576	22,8937	1,6057	297,58	cooling	8,576	36,5615	19,2219	242,38	heating	8,576	34,3583	13,5551	290,8	
8,578	23,3474	0,8701	314,2	cooling	8,578	36,8214	18,5102	255,96	heating	8,578	33,4581	11,7353	303,65	
8,58	22,9957	1,9287	294,49	cooling	8,58	36,961	19,6315	242,24	heating	8,58	33,1313	10,7524	312,82	
8,582	22,5594	0,5278	307,97	cooling	8,582	37,2328	16,7754	285,96	heating	8,582	33,0584	7,6547	355,11	
8,584	22,5803	0,4258	309,69	cooling	8,584	37,2309	17,4202	276,92	heating	8,584	33,9805	11,8454	309,42	
8,586	22,612	2,4312	282,1	cooling	8,586	37,7402	18,5217	268,65	heating	8,586	32,5122	10,4821	307,95	
8,588	22,051	1,2623	290,6		8,588	38,1383	20,1833	250,98	heating	8,588	33,1853	15,0794	253,09	
8,59	22,546	5,1446	243,25		8,59	37,7071	14,6561	322,22	heating	8,59	32,836	17,9151	208,57	
8,592	21,7551	4,8631	236,12		8,592	37,7775	11,8019	363,1	heating	8,592	32,28	11,6142	288,88	
8,594	21,7489	2,2136	273,08		8,594	37,5731	9,286	395,41	heating	8,594	32,475	9,831	316,53	
8,596	21,5142	1,3216	282,26		8,596	38,2675	12,7438	356,78		8,596	32,9948	9,0286	335,01	
8,598	21,705	-0,36	308,44		8,598	37,1998	16,6644	287,05		8,598	32,9495	9,0865	333,57	
8,6	21,9522	-2,0538	335,57		8,6	37,7692	18,3144	271,95		8,6	32,6203	11,3966	296,68	
8,602	21,8748	0,1202	304,1		8,602	37,3724	16,6858	289,17		8,602	33,5568	15,0516	258,68	
8,604	21,3252	-1,931	325,09		8,604	37,853	13,8612	335,37		8,604	34,2657	11,319	320,76	
8,606	21,8181	-0,253	308,52		8,606	37,4208	11,6049	360,87		8,606	33,6897	14,7399	264,89	
8,608	21,2479	0,9596	283,6		8,608	38,4916	14,0362	341,85		8,608	32,6747	9,3474	326,08	
8,61	21,1871	-0,43	302,18		8,61	37,6315	16,886	289,99		8,61	34,147	11,445	317,34	
8,612	21,5473	1,1346	285,34		8,612	37,4951	16,724	290,35		8,612	33,3114	9,2233	336,72	
8,614	21,1501	0,2049	292,78		8,614	37,296	18,2424	266,34		8,614	33,0307	7,713	353,9	
8,616	21,0361	-1,9543	321,37		8,616	37,1308	14,6052	314,87		8,616	33,4772	9,927	329,2	
8,618	21,1983	-0,9406	309,47		8,618	37,4343	16,0144	299,42		8,618	33,6414	11,4039	310,85	
8,62	21,3252	0,1399	296,14		8,62	37,234	17,7238	272,72		8,62	33,0344	8,9771	336,29	
8,622	21,13	0,1149	293,76		8,622	38,1568	19,3614	262,73		8,622	33,5611	9,7251	333,19	
8,624	21,3697	4,1631	240,52		8,624	38,0611	19,248	262,98		8,624	32,9806	6,159	374,93	
8,626	20,9487	2,0075	264,77		8,626	38,5636	20,194	256,78		8,626	32,8981	3,9528	404,61	cooling
8,628	21,4011	-0,4179	305		8,628	37,9062	16,2121	303,25		8,628	33,1699	11,2873	305,89	cooling
8,63	21,3819	-1,7426	323,25		8,63	38,4113	18,3635	280,24		8,63	33,2076	11,6886	300,8	cooling
8,632	21,4463	-3,31	346,06		8,632	36,5734	16,4117	281,83		8,632	33,2279	16,2173	237,78	cooling
8,634	21,7977	-0,7197	314,76		8,634	36,9888	16,8099	282,07		8,634	32,3235	13,9168	257,3	cooling
8,636	21,5004	2,9324	259,55		8,636	36,232	17,5471	261,19		8,636	33,7071	12,0944	302,11	
8,638	21,0053	-0,4144	299,42		8,638	36,2986	14,3085	307,39	heating	8,638	33,9346	9,84	336,81	
8,64	21,3548	-1,8214	323,97		8,64	37,1327	16,0246	295,06		8,64	33,5323	11,6048	306,51	
8,642	21,3348	-0,1957	300,96		8,642	37,7717	17,2254	287,21		8,642	33,3144	12,2313	294,71	
8,644	21,0168	-2,4822	328,48		8,644	37,978	16,9826	293,48		8,644	34,2426	17,1486	238,95	
8,646	21,2973	0,7901	286,66		8,646	38,2317	18,6657	273,5		8,646	32,7875	13,2479	273,13	
8,648	21,0833	0,8535	282,78		8,648	38,5194	19,9571	259,47	heating	8,648	33,2653	12,6555	288,09	
8,65	21,0504	-2,5769	330,27		8,65	37,9518	14,9627	321,35	heating	8,65	32,6853	13,8474	263,33	
8,652	21,6695	-1,809	328,19		8,652	38,0949	17,3741	289,65	heating	8,652	32,3712	10,9901	298,88	
8,654	21,4855	0,258	296,73		8,654	36,5002	10,748	359,98	heating	8,654	32,4463	12,1632	283,53	

8,656	21,963	-0,2927	311,1		8,656	37,3787	12,0047	354,69		8,656	32,8494	12,1614	289,19	
8,658	21,9408	3,4181	258,92		8,658	36,4131	11,7974	344,09		8,658	32,9908	13,7248	269,31	
8,66	21,8993	0,9618	292,68		8,66	36,3562	12,3222	335,96		8,66	32,5276	12,1891	284,3	
8,662	22,4342	-0,689	323,23		8,662	36,9551	13,7441	324,46		8,662	34,1526	16,7013	243,94	
8,664	23,2197	3,4124	276,88		8,664	37,4632	15,7037	304,17		8,664	34,1481	12,1159	307,98	
8,666	22,5893	1,5787	293,7		8,666	37,0922	11,5086	357,62		8,666	33,9826	13,783	282,36	
8,668	23,3358	3,8624	272,21		8,668	37,8325	15,4032	313,53		8,668	32,7872	8,5657	338,58	
8,67	23,4028	7,7506	218,79		8,67	36,9717	15,9841	293,37		8,67	34,1619	11,4778	317,09	
8,672	23,3728	4,9613	257,37		8,672	37,3264	16,4454	291,89		8,672	32,7391	12,447	283,65	
8,674	24,2305	3,6505	287,68		8,674	37,1088	21,0388	224,64		8,674	32,7651	16,2471	230,9	
8,676	24,4025	5,131	269,39		8,676	37,0998	15,5951	300,6		8,676	32,3242	15,9805	228,46	
8,678	24,5435	0,5503	335,39		8,678	37,2527	14,3173	320,6		8,678	32,8423	15,2491	245,93	
8,68	24,3885	4,329	280,4		8,68	37,1353	13,6157	328,77		8,68	31,9991	13,6865	255,98	heating
8,682	24,0183	4,4578	273,43		8,682	37,3524	13,5621	332,55		8,682	32,8182	13,9904	263,18	heating
8,684	23,8274	-0,9121	345,82		8,684	38,2474	16,2976	306,83		8,684	32,744	15,9492	234,77	heating
8,686	25,0905	1,0669	335,81		8,686	37,575	18,6581	264,43		8,686	32,4863	12,044	285,75	heating
8,688	24,2958	2,7502	301,17		8,688	37,6453	12,0782	357,39		8,688	33,2593	14,7884	258,2	heating
8,69	24,5879	4,3468	282,94		8,69	38,4372	15,3359	322,92		8,69	33,6208	13,0136	288,06	heating
8,692	24,1213	7,6621	230,07		8,692	37,6236	11,848	360,3		8,692	33,2447	15,9833	241,29	heating
8,694	23,5463	1,5149	307,97		8,694	38,2381	14,1625	336,54		8,694	32,7063	13,2838	271,5	heating
8,696	23,7295	-4,435	393,7		8,696	38,3553	17,6722	289,12		8,696	34,1536	16,3359	249,07	heating
8,698	24,1255	-0,2648	340,94		8,698	39,1795	17,1598	307,8		8,698	33,2742	10,8598	313,32	heating
8,7	24,0748	3,3289	290		8,7	39,01	16,0342	321,17		8,7	33,3456	13,8272	272,84	heating
8,702	24,1075	6,2129	250,14		8,702	39,2554	16,1307	323,25		8,702	32,5204	10,8285	303,22	heating
8,704	24,0724	4,4103	274,85		8,704	38,3871	12,6272	360,09		8,704	34,019	13,312	289,45	heating
8,706	23,5831	-4,0328	386,03		8,706	38,7595	17,8392	292,44	cooling	8,706	32,8216	13,4809	270,36	heating
8,708	24,5798	-3,8953	398,04		8,708	36,928	19,8765	238,35	cooling	8,708	33,3003	12,2715	293,95	heating
8,71	23,5653	-1,4996	350,37		8,71	37,6252	19,8519	248,44		8,71	32,6597	11,7731	291,96	heating
8,712	23,6677	1,4699	310,29		8,712	36,916	20,2213	233,37		8,712	32,5966	10,7094	305,95	heating
8,714	23,3236	5,0321	255,69		8,714	36,9421	13,9033	322,05		8,714	32,1579	11,6982	286	heating
8,716	22,9843	2,6714	283,94		8,716	37,9092	14,7923	323,14		8,716	33,207	10,7697	313,64	heating
8,718	22,7055	-1,946	344,59		8,718	38,2345	16,6063	302,33		8,718	32,5682	11,5296	294,09	heating
8,72	22,8819	-0,4799	326,56		8,72	38,2889	18,3172	279,17		8,72	32,3619	7,3699	349,35	heating
8,722	22,4564	-2,6257	350,61		8,722	38,5639	21,2515	242		8,722	33,6142	14,0129	274	heating
8,724	22,3285	-1,0978	327,47		8,724	38,8841	23,0529	221,3		8,724	33,437	13,5071	278,59	heating
8,726	22,3179	2,9196	271,16		8,726	38,0342	15,5434	314,39		8,726	33,3331	18,1123	212,76	heating
8,728	21,8396	1,1375	289,38		8,728	38,1985	13,8848	339,87		8,728	32,804	14,627	254,09	heating
8,73	22,8217	3,4065	271,4		8,73	36,7508	7,4524	409,55		8,73	34,2387	13,5433	289,29	heating
8,732	21,9471	2,5228	271,52		8,732	37,4663	8,9007	399,3		8,732	33,0893	8,0319	350,27	heating
8,734	22,4248	0,7736	302,65		8,734	36,3654	12,3298	335,98		8,734	33,7151	12,6976	293,79	heating
8,736	22,2758	3,1381	267,52		8,736	37,0401	13,073	335,03		8,736	33,0814	14,5906	258,47	heating
8,738	22,3605	3,7651	259,94		8,738	37,4706	13,8716	329,88	cooling	8,738	33,9653	17,7819	226,22	heating
8,74	22,2698	-0,8175	322,73		8,74	38,0673	13,9955	336,49	cooling	8,74	32,6744	17,1856	216,51	heating
8,742	23,4568	0,3064	323,61		8,742	38,5439	12,1628	368,77	cooling	8,742	34,1458	13,1831	293,03	heating
8,744	23,1178	-3,998	379,04		8,744	39,7375	14,8451	347,96	cooling	8,744	33,366	12,4652	292,16	heating
8,746	23,6362	-3,5879	380,55		8,746	39,0835	17,1475	306,63	cooling	8,746	33,33	10,8948	313,61	heating
8,748	23,687	1,5581	309,33		8,748	38,6047	14,9801	330,24	cooling	8,748	33,5067	14,5542	264,93	heating
8,75	23,3003	1,3297	307,12		8,75	38,9098	18,9576	278,9	cooling	8,75	34,6333	14,5591	280,61	heating
8,752	24,0593	3,7058	284,51		8,752	37,7383	14,1261	330,06	cooling	8,752	33,8168	17,7644	224,39	heating
8,754	23,5355	3,8995	274,48		8,754	37,9069	15,4986	313,24	cooling	8,754	33,2398	13,0215	282,62	heating
8,756	23,7652	-3,1301	375,96		8,756	37,3527	17,4638	278,02	cooling	8,756	35,0126	17,1753	249,34	heating
8,758	23,8524	-6,0514	418,01		8,758	37,8818	20,943	236,78	cooling	8,758	34,2555	10,9627	325,6	heating
8,76	24,0588	-4,2338	395,49	cooling	8,76	37,5474	19,9756	245,63	cooling	8,76	34,2365	12,2179	307,79	heating
8,762	23,965	-3,8489	388,8	cooling	8,762	37,4384	18,1438	269,71		8,762	33,5529	8,9251	344,26	heating
8,764	24,7785	5,0667	275,54	cooling	8,764	37,3603	9,3287	391,84		8,764	35,1146	12,1985	320,33	heating
8,766	23,7916	3,866	278,53	cooling	8,766	38,4675	11,5874	375,74	cooling	8,766	33,4841	12,7123	290,36	heating

8,768	23,8989	-1,1141	349,64	cooling	8,768	37,6912	13,0568	344,35	cooling	8,768	34,5262	18,7246	220,88	heating
8,77	24,0589	-1,7053	360,15	cooling	8,77	38,5107	15,9566	315,27	cooling	8,77	33,4821	17,7284	220,21	heating
8,772	23,7382	-0,1652	334,13	cooling	8,772	38,5415	18,6985	277,38	cooling	8,772	34,1135	15,8952	254,66	heating
8,774	24,0137	3,5202	286,47	cooling	8,774	38,1976	14,9209	325,37	cooling	8,774	33,181	13,6027	273,67	heating
8,776	23,83	9,0531	206,56	cooling	8,776	38,1937	13,4169	346,34	cooling	8,776	34,631	10,2961	340,17	heating
8,778	23,5113	4,6585	263,53	cooling	8,778	38,3406	13,7563	343,65	cooling	8,778	34,133	14,2461	277,99	heating
8,78	23,6414	0,5958	322,14	cooling	8,78	37,3931	13,5149	333,78	cooling	8,78	33,474	12,4285	294,18	heating
8,782	23,6091	-0,2572	333,62	cooling	8,782	37,2556	14,7383	314,76	cooling	8,782	34,8286	16,1857	260,6	heating
8,784	23,4469	-0,2987	331,93	cooling	8,784	37,1034	19,6927	243,38	cooling	8,784	35,0247	11,952	322,52	heating
8,786	23,9355	7,2609	233,09	cooling	8,786	36,9395	16,0509	291,99	cooling	8,786	34,4794	13,6858	290,66	heating
8,788	23,1779	7,3761	220,89	cooling	8,788	37,1544	17,6213	273,04	cooling	8,788	33,9191	9,4425	342,15	heating
8,79	23,0616	0,5962	314,03		8,79	37,0122	13,712	325,7	cooling	8,79	36,0727	16,9659	267,08	heating
8,792	23,3598	-4,3284	387,04		8,792	38,2427	14,5822	330,74	cooling	8,792	33,9736	12,7273	296,99	heating
8,794	22,9779	-5,8915	403,55	cooling	8,794	38,3362	16,846	300,4	cooling	8,794	34,9298	16,3188	260,15	heating
8,796	23,353	-1,8137	351,79	cooling	8,796	38,4356	18,1284	283,87	cooling	8,796	33,8454	13,5291	283,99	heating
8,798	23,0997	6,2832	235,07	cooling	8,798	38,545	17,593	292,88	cooling	8,798	35,123	13,3838	303,88	heating
8,8	22,7013	2,4696	282,81	cooling	8,8	38,5986	18,6951	278,22	cooling	8,8	33,3574	12,0749	297,5	heating
8,802	22,6294	-2,7998	355,46	cooling	8,802	37,4248	15,4173	307,63		8,802	34,689	12,8388	305,43	heating
8,804	22,6641	-6,1361	402,58	cooling	8,804	37,9144	16,3248	301,79		8,804	33,8222	11,0924	317,73	heating
8,806	22,3547	-8,2019	427,14	cooling	8,806	36,7991	17,6341	267,9		8,806	34,1253	10,0099	337,1	heating
8,808	22,9261	-1,0197	334,73	cooling	8,808	36,7038	15,4586	296,98		8,808	33,9946	12,4886	300,62	heating
8,81	22,5527	3,9216	260,44	cooling	8,81	37,0124	18,8982	253,21		8,81	35,3049	12,3258	321,21	heating
8,812	22,9541	3,3891	273,49	cooling	8,812	37,3455	15,7277	302,18		8,812	34,1922	16,4902	247,45	
8,814	23,4685	3,0951	284,79	cooling	8,814	37,4348	13,0177	341,31		8,814	33,3309	9,8839	327,75	heating
8,816	23,2577	0,4038	319,46	cooling	8,816	38,0797	12,8579	352,56		8,816	35,3282	14,2055	295,26	
8,818	23,7093	-1,13	347,22	cooling	8,818	38,5046	14,9557	329,18		8,818	34,5947	8,7885	360,73	
8,82	23,8264	3,9734	277,52	cooling	8,82	38,697	15,371	326,06		8,82	34,6164	11,3516	325,21	
8,822	23,9065	5,0708	263,29	cooling	8,822	38,2699	18,2344	280,07		8,822	33,9551	10,2612	331,21	
8,824	23,9813	6,0926	250,06	cooling	8,824	37,4084	11,9991	355,18		8,824	35,6232	15,4415	282,11	
8,826	24,3622	6,6019	248,26	cooling	8,826	38,0478	11,5155	370,88	cooling	8,826	33,6638	8,8565	346,77	
8,828	24,0416	4,6917	270,48		8,828	36,8672	11,8735	349,37	cooling	8,828	34,5944	14,5915	279,61	
8,83	24,5106	6,4067	253,07		8,83	37,2499	14,0158	324,78	cooling	8,83	33,2673	11,4037	305,62	
8,832	24,0337	6,7317	241,86		8,832	36,8984	16,8	280,95	cooling	8,832	34,5952	12,6089	307,34	
8,834	24,3386	4,6438	275,3		8,834	37,3613	18,2617	266,98	cooling	8,834	32,8984	12,4418	285,95	
8,836	24,4126	5,049	270,67		8,836	36,786	15,6081	296,04	cooling	8,836	34,1958	11,699	314,47	
8,838	24,098	2,9051	296,24		8,838	38,2765	16,8057	300,13	cooling	8,838	33,7756	12,4356	298,3	
8,84	24,3263	0,9156	327,25		8,84	37,6711	15,0772	315,83	cooling	8,84	33,6928	12,3278	298,65	
8,842	24,3816	1,6398	317,9		8,842	38,1949	13,36	347,16	cooling	8,842	33,9931	14,9634	266,01	
8,844	24,4165	1,5069	320,24		8,844	38,4244	17,7309	289,26	cooling	8,844	35,3323	14,4312	292,17	
8,846	24,5202	2,8828	302,46		8,846	38,5019	17,3596	295,54	cooling	8,846	34,4336	15,3221	267,15	
8,848	24,8046	4,2147	287,82		8,848	38,281	18,3021	279,27	cooling	8,848	33,9294	7,414	370,65	
8,85	24,4525	0,5713	333,82		8,85	37,8284	17,3283	286,56		8,85	36,069	13,8593	310,46	
8,852	24,9739	-1,248	366,54		8,852	38,4106	15,6252	318,51		8,852	34,6881	9,0801	357,96	
8,854	24,5895	-2,871	383,86		8,854	38,0802	13,7851	339,61		8,854	34,8231	15,1132	275,51	
8,856	25,0318	-1,6425	372,87		8,856	38,1786	16,7907	298,97	cooling	8,856	33,8589	12,1184	303,9	
8,858	25,1006	3,81	297,61	cooling	8,858	37,2362	13,4307	332,77	cooling	8,858	35,1965	11,1205	336,55	
8,86	24,8559	4,4566	285,15	cooling	8,86	38,3737	17,4128	293	cooling	8,86	33,6041	6,989	372,04	
8,862	25,0323	4,4913	287,13	cooling	8,862	37,0386	16,5253	286,75	cooling	8,862	34,2879	12,0002	311,55	
8,864	25,2256	5,9239	269,81		8,864	38,0091	16,9747	294,03		8,864	33,4933	11,5715	306,43	
8,866	25,2392	3,6244	302,14		8,866	37,9405	16,3192	302,23		8,866	34,1245	14,9498	268,03	
8,868	25,346	0,9747	340,67		8,868	38,5908	14,4315	337,71		8,868	32,6712	14,1494	258,91	
8,87	25,4536	0,7572	345,22		8,87	38,6792	12,7126	362,98		8,87	34,2751	11,9464	312,12	
8,872	25,1152	-1,0685	366,01	cooling	8,872	39,2355	16,0852	323,61	cooling	8,872	33,9081	14,4126	272,52	
8,874	25,2547	2,0576	324,26	cooling	8,874	38,7052	14,6753	335,9	cooling	8,874	33,5291	9,8498	331	
8,876	24,7418	3,7433	293,53	cooling	8,876	38,3158	13,3726	348,67	cooling	8,876	34,6718	14,1509	286,85	
8,878	25,2404	3,3683	305,74	cooling	8,878	38,0709	16,3507	303,62	cooling	8,878	34,9	12,1547	317,95	

8,88	25,3655	1,702	330,78	cooling	8,88	37,5972	15,6102	307,35	cooling	8,88	34,1665	12,6226	301,15	
8,882	25,186	-0,0725	353,08	cooling	8,882	37,5357	19,9854	245,33	cooling	8,882	34,049	8,3978	358,57	
8,884	25,0963	-2,0238	379,1	cooling	8,884	36,6044	16,5117	280,87	cooling	8,884	35,4026	12,9398	314	
8,886	24,9639	-2,4205	382,79	cooling	8,886	37,4471	16,4668	293,27	cooling	8,886	34,5192	10,107	341,25	
8,888	24,9152	-1,9949	376,16	cooling	8,888	37,4827	16,0406	299,73		8,888	34,74	14,4679	283,37	
8,89	24,9934	1,1854	332,8	cooling	8,89	37,5407	17,7041	277,29		8,89	33,8222	9,8546	335,03	
8,892	25,2809	1,5181	332,17	cooling	8,892	38,0776	17,0742	293,6		8,892	34,9046	8,6345	367,22	
8,894	25,0217	-0,635	358,64	cooling	8,894	38,8577	22,1164	234,02		8,894	33,48	7,3653	365,04	cooling
8,896	24,9658	-1,0368	363,48	cooling	8,896	38,4464	20,1684	255,5		8,896	34,4006	9,8364	343,37	cooling
8,898	24,3804	-3,2257	385,89	cooling	8,898	38,8337	20,7685	252,53		8,898	33,5387	10,4569	322,65	cooling
8,9	25,1627	-0,4361	357,83	cooling	8,9	38,2225	16,6355	301,75		8,9	33,8829	11,3831	314,51	cooling
8,902	24,5763	3,2379	298,28	cooling	8,902	37,9747	12,4256	357,14		8,902	33,0635	11,3336	303,75	cooling
8,904	24,6385	3,8185	291,03	cooling	8,904	37,4612	14,3457	323,12		8,904	34,6378	10,8194	332,95	cooling
8,906	24,4596	4,1691	283,63	cooling	8,906	37,2838	14,054	324,72		8,906	33,8245	13,9929	277,22	cooling
8,908	24,3084	4,9396	270,75	cooling	8,908	37,0161	13,6939	326,01		8,908	33,7215	11,198	314,85	cooling
8,91	23,2576	-1,6286	347,87	cooling	8,91	37,0904	12,5518	343,01		8,91	34,5864	14,5368	280,26	cooling
8,912	24,034	1,0105	321,83	cooling	8,912	37,1258	11,5695	357,24		8,912	34,566	11,6458	320,39	cooling
8,914	23,3893	2,1546	296,83	cooling	8,914	38,4138	13,219	352,19		8,914	34,2199	14,0246	282,3	cooling
8,916	23,0569	1,8455	296,5	cooling	8,916	37,8883	18,0398	277,45	cooling	8,916	33,6851	11,0874	315,88	cooling
8,918	22,8147	2,8221	279,47	cooling	8,918	38,1105	14,3265	332,46		8,918	35,3406	16,3723	265,15	cooling
8,92	22,7857	1,5492	296,86	cooling	8,92	39,0685	15,9839	322,69	cooling	8,92	33,9479	10,5741	326,73	
8,922	23,1497	-1,2814	341,51	cooling	8,922	38,6696	13,2403	355,46	cooling	8,922	34,1021	13,7905	283,93	cooling
8,924	23,386	2,5634	291,07	cooling	8,924	38,5287	13,7814	345,93	cooling	8,924	33,1306	8,946	338,06	
8,926	23,4181	2,7814	288,47	cooling	8,926	38,4782	17,5785	292,15	cooling	8,926	34,4485	9,9667	342,22	cooling
8,928	23,4969	2,9462	287,27	cooling	8,928	38,4561	19,4843	265,2	cooling	8,928	32,8198	9,1177	331,32	cooling
8,93	23,3441	2,7338	288,1	cooling	8,93	37,7686	18,1436	274,33	cooling	8,93	33,7847	12,5523	296,8	cooling
8,932	22,7486	-3,2904	363,99	cooling	8,932	38,0332	18,2736	276,21	cooling	8,932	33,0697	11,5062	301,43	cooling
8,934	23,9119	-1,7847	359,2	cooling	8,934	37,1092	12,8061	339,72		8,934	33,6919	12,0721	302,21	cooling
8,936	23,5742	3,0172	287,36	cooling	8,936	38,0494	14,1788	333,68	cooling	8,936	32,6754	11,1761	300,53	cooling
8,938	23,7889	2,7601	293,95	cooling	8,938	36,9628	17,6671	269,73	cooling	8,938	34,0715	11,9511	309,21	cooling
8,94	23,6436	3,9733	274,96	cooling	8,94	37,6937	18,0767	274,22		8,94	33,1168	15,9771	239,59	cooling
8,942	23,2902	2,6679	288,27	cooling	8,942	37,2653	18,7211	259,22		8,942	32,7202	11,2369	300,31	
8,944	22,6461	-3,0433	359,1	cooling	8,944	36,9829	14,0954	319,93		8,944	33,4982	13,0736	285,51	
8,946	23,3465	2,0093	298,26	cooling	8,946	37,1721	11,2185	362,79		8,946	33,7394	10,8151	320,45	
8,948	22,3664	2,6625	275,43	cooling	8,948	37,7516	13,1857	343,39		8,948	32,9953	12,3899	288,03	
8,95	22,6377	1,2596	298,83	cooling	8,95	37,0248	15,6852	298,3	cooling	8,95	32,8809	11,8442	294,06	
8,952	22,3499	3,9104	257,76	cooling	8,952	37,1074	16,2688	291,29	cooling	8,952	33,7573	17,6144	225,65	
8,954	21,7674	1,4104	284,56	cooling	8,954	37,7497	20,6538	238,98		8,954	32,7579	12,2723	286,36	
8,956	21,974	-2,1026	336,56	cooling	8,956	37,0254	15,5803	299,77		8,956	33,112	15,3571	248,19	
8,958	22,0598	1,0593	293,56	cooling	8,958	37,4558	14,5799	319,77		8,958	32,3407	9,891	313,81	
8,96	21,4577	-0,239	303,29	cooling	8,96	36,9751	12,8502	337,23		8,96	33,6985	9,7085	335,34	
8,962	21,7927	3,5333	255,24	cooling	8,962	37,7197	13,657	336,36		8,962	32,5475	9,7616	318,51	
8,964	21,6711	6,9447	205,85		8,964	37,1546	15,9859	295,91		8,964	33,5556	9,4849	336,47	
8,966	21,2662	2,3554	264,35		8,966	37,7069	18,2558	271,9		8,966	32,9466	8,4796	342,01	
8,968	21,76	1,0003	290,19		8,968	37,2578	15,3096	306,8		8,968	33,1369	8,1821	348,83	
8,97	21,5723	2,5685	265,64		8,97	38,0496	16,5048	301,16		8,97	32,7407	8,9873	332,04	
8,972	21,7127	1,2494	286,05		8,972	37,4798	16,6255	291,51		8,972	33,9422	11,5328	313,25	
8,974	22,0378	3,1219	264,42		8,974	38,3307	16,5332	304,7		8,974	32,8725	13,5343	270,32	
8,976	22,0228	1,8943	281,37		8,976	37,7549	17,1724	287,71		8,976	32,823	7,7125	351,01	
8,978	22,1452	-2,1933	340,22		8,978	37,3449	13,2525	336,78		8,978	33,799	9,7053	336,8	
8,98	22,6566	1,0476	302,06		8,98	38,0397	15,0617	321,2		8,98	33,397	6,6764	373,51	cooling
8,982	21,7952	0,0279	304,28		8,982	37,8743	13,193	345,01		8,982	33,1016	11,7935	297,85	cooling
8,984	22,1775	-0,4235	315,93		8,984	37,6424	10,8093	375,09		8,984	32,7434	12,6578	280,77	cooling
8,986	22,1384	1,7068	285,6		8,986	37,4571	9,7102	387,86	cooling	8,986	33,7087	13,7677	278,75	cooling
8,988	21,887	-0,5751	313,99		8,988	38,0612	14,2216	333,24	cooling	8,988	32,6573	8,9559	331,31	
8,99	22,101	-2,3803	342,21		8,99	37,156	14,2878	319,66	cooling	8,99	33,0883	10,1075	321,24	cooling

8,992	22,2632	-0,894	323,7		8,992	37,758	19,859	250,2	cooling	8,992	32,2415	6,1328	364,96	cooling
8,994	21,8239	-3,534	354,47		8,994	36,9075	14,8047	308,96	cooling	8,994	33,0646	9,1569	334,19	cooling
8,996	22,1739	-1,7208	334,01		8,996	37,7683	11,822	362,69	cooling	8,996	32,1272	11,6796	285,83	cooling
8,998	22,0878	2,0581	279,99		8,998	36,9899	12,1524	347,19	cooling	8,998	33,2976	12,1531	295,57	cooling
9	22,0256	1,2816	289,97		9	37,3483	13,6746	330,92	cooling	9	32,5824	13,5481	266,07	cooling
9,002	22,2104	2,5773	274,44		9,002	37,0901	16,1227	293,09	cooling	9,002	32,5747	10,0777	314,47	cooling
9,004	21,8781	2,263	274,19		9,004	37,2709	19,4499	249,11	cooling	9,004	33,1935	10,826	312,66	cooling
9,006	21,7717	-1,6898	327,96		9,006	36,8038	15,3092	300,46	cooling	9,006	33,5923	9,6351	334,89	cooling
9,008	22,3419	-0,4637	318,79		9,008	37,7245	12,8542	347,65	cooling	9,008	32,7563	8,9353	332,98	cooling
9,01	22,0121	-0,6955	317,42		9,01	37,2657	13,0046	339,13	cooling	9,01	32,8423	7,4	355,65	cooling
9,012	22,3223	-2,0676	340,94		9,012	36,7567	8,6567	392,8	cooling	9,012	33,5505	12,2033	298,4	cooling
9,014	22,7189	1,758	293		9,014	37,9928	14,9916	321,52	cooling	9,014	32,8743	10,6996	309,97	cooling
9,016	22,3909	1,1926	296,32		9,016	37,4954	17,0407	285,93	cooling	9,016	32,9308	15,1612	248,39	cooling
9,018	22,5455	-1,8358	340,81		9,018	37,4876	17,5737	278,37	cooling	9,018	32,1779	11,9241	283,12	cooling
9,02	22,9615	0,3163	316,55		9,02	37,1623	14,4187	317,92	cooling	9,02	33,2111	10,3118	320,1	cooling
9,022	22,689	-0,3541	322,11		9,022	37,7169	13,9241	332,59	cooling	9,022	32,094	6,8931	352,27	cooling
9,024	22,7419	0,3152	313,49		9,024	37,1045	12,786	339,94	cooling	9,024	32,8125	9,4705	326,29	cooling
9,026	22,5218	2,6242	278,14		9,026	37,2737	21,1561	225,3	cooling	9,026	32,0503	10,3416	303,46	cooling
9,028	21,9591	-2,0851	336,1		9,028	36,5158	19,591	236,58	cooling	9,028	32,7528	14,6533	253	cooling
9,03	22,3778	-3,196	357,48		9,03	37,3489	18,7192	260,42	cooling	9,03	31,7109	13,2136	258,57	cooling
9,032	21,8397	0,1679	302,94	cooling	9,032	36,5288	16,4998	279,98		9,032	32,948	12,5229	285,51	
9,034	21,628	-0,2274	305,51	cooling	9,034	36,8798	14,3627	314,76		9,034	32,047	12,3727	275,02	
9,036	21,7613	2,8696	264,08	cooling	9,036	36,9305	15,5742	298,53		9,036	32,1753	10,8148	298,59	
9,038	21,4702	2,7053	262,31		9,038	36,7139	16,7083	279,65		9,038	32,6709	13,4481	268,71	
9,04	21,0852	-2,6027	331,12		9,04	36,7159	18,0684	260,66		9,04	32,8498	10,7142	309,42	
9,042	21,8988	-1,3564	325,07		9,042	37,2759	18,7329	259,2		9,042	32,4423	10,0478	313,04	
9,044	21,2971	-2,5225	332,96		9,044	36,7322	17,1576	273,62		9,044	32,1132	7,4577	344,65	
9,046	21,7211	-1,9716	331,19		9,046	36,5781	11,0968	356,19		9,046	33,0078	12,01	293,52	
9,048	21,677	2,8996	262,48	cooling	9,048	37,2525	12,9985	339,04		9,048	32,4436	12,0075	285,67	
9,05	21,683	1,9625	275,66		9,05	37,0428	13,062	335,22		9,05	32,4912	18,2979	198,4	
9,052	21,8911	-1,0049	320,05		9,052	37,1155	19,3861	247,83		9,052	31,7161	13,8114	250,28	
9,054	21,8207	-1,3452	323,82		9,054	36,6825	19,6991	237,4		9,054	32,8156	13,0356	276,49	
9,056	21,6725	-3,8798	357,18		9,056	37,2778	18,0658	268,56		9,056	31,8512	8,7669	322,68	
9,058	21,987	0,5016	300,33	cooling	9,058	36,8013	13,6866	323,11		9,058	32,4371	9,6353	318,74	
9,06	21,6775	2,5995	266,68		9,06	36,9804	14,3134	316,85		9,06	32,035	9,1316	320,16	
9,062	21,1125	-2,7252	333,22		9,062	36,8067	14,8566	306,83		9,062	32,4914	10,588	306,18	
9,064	22,2221	-1,4791	331,31		9,064	37,2139	19,8113	243,26		9,064	32,1623	10,1484	307,72	
9,066	21,2624	-2,5896	333,42	cooling	9,066	36,2125	20,1353	224,74		9,066	33,035	12,8191	282,59	
9,068	21,584	-2,2253	332,82	cooling	9,068	36,7221	18,0976	260,34		9,068	32,0992	13,8099	255,66	
9,07	21,2577	0,8138	285,77	cooling	9,07	36,4325	15,4225	293,69		9,07	32,1697	11,0604	295,08	
9,072	21,3453	-0,0875	299,6	cooling	9,072	36,5038	12,1964	339,78		9,072	32,9509	12,8819	280,53	
9,074	21,2175	-2,2397	327,9	cooling	9,074	36,7778	14,6185	309,75		9,074	32,8422	8,9273	334,29	
9,076	20,9044	0,8758	279,97	cooling	9,076	37,0648	16,3886	289,02		9,076	32,4122	12,4109	279,59	
9,078	20,5582	-0,206	290,25	cooling	9,078	36,8178	20,213	232,11		9,078	31,8914	12,867	265,93	
9,08	20,5065	1,8082	261,37		9,08	36,7634	20,6937	224,63		9,08	32,659	15,7138	236,87	
9,082	20,2573	3,3314	236,6		9,082	37,2755	21,5542	219,76		9,082	32,0806	12,8291	269,11	
9,084	19,8454	0,948	264,16		9,084	37,2022	16,8847	284,01	heating	9,084	31,7467	14,2351	244,79	
9,086	20,6238	0,2575	284,69		9,086	37,3448	17,8184	272,95	heating	9,086	31,2286	9,0844	309,54	
9,088	19,8677	0,7168	267,7		9,088	36,884	15,4763	299,25	heating	9,088	32,0899	13,5616	259	
9,09	20,2135	0,8941	270,06		9,09	37,5547	16,7447	290,89	heating	9,09	30,9387	14,7578	226,18	heating
9,092	20,1254	2,5894	245,13		9,092	36,8875	16,0396	291,42	heating	9,092	31,7714	16,3259	215,9	heating
9,094	20,699	4,4497	227,14		9,094	37,0138	16,2702	289,96	heating	9,094	31,6658	15,9322	219,93	heating
9,096	20,3126	4,0155	227,81		9,096	36,7371	12,7064	335,91	heating	9,096	31,725	12,2303	272,51	heating
9,098	21,1317	5,5873	217,29		9,098	37,1236	12,9754	337,56	heating	9,098	31,8656	9,7275	309,46	heating
9,1	20,8544	3,4612	243,13	heating	9,1	36,3671	13,6725	317,24		9,1	32,6643	12,9933	274,97	heating
9,102	20,8986	-0,9645	305,61	heating	9,102	36,9247	17,2347	275,24		9,102	31,7942	13,1763	260,25	heating

9,104	20,7132	-4,7625	356,11	heating
9,106	21,3056	-3,0197	340,03	heating
9,108	21,3587	-1,2322	315,79	
9,11	21,2798	2,0225	269,19	
9,112	21,4618	0,5267	292,64	
9,114	21,2894	-4,7398	363,85	
9,116	21,6625	-6,5954	395	
9,118	21,3266	-4,8734	366,24	
9,12	22,0792	-2,0837	337,76	
9,122	21,79	-0,4647	311,09	
9,124	22,0497	0,8401	296,48	
9,126	21,611	-1,405	321,73	
9,128	22,505	0,2804	310,67	
9,13	22,084	-0,1268	310,47	
9,132	22,2144	1,2351	293,26	
9,134	22,0457	3,7016	256,42	
9,136	21,4985	5,3164	226,2	
9,138	21,4958	3,475	251,9	
9,14	21,6902	2,0876	274,02	
9,142	22,5101	-1,4828	335,39	
9,144	22,3697	-0,9248	325,62	
9,146	22,8167	2,8249	279,46	
9,148	22,3254	1,9234	285,19	
9,15	23,1747	1,9962	296,04	
9,152	22,7234	-0,0125	317,81	
9,154	23,075	-2,8942	363,01	
9,156	22,9607	-1,2088	337,85	
9,158	22,642	0,0336	316,03	
9,16	22,7018	-1,8747	343,54	
9,162	23,5536	1,4016	309,65	
9,164	23,3295	-2,1484	356,14	
9,166	23,5954	-5,1538	401,87	
9,168	23,7123	-2,4533	365,76	cooling
9,17	23,1501	-1,1227	339,3	cooling
9,172	23,4747	3,336	281,51	cooling
9,174	23,0945	6,2645	235,26	cooling
9,176	23,1078	0,4172	317,18	cooling
9,178	23,2984	-2,0075	353,74	cooling
9,18	23,0678	0,1673	320,11	cooling
9,182	22,6741	1,0209	302,68	cooling
9,184	23,0408	6,3993	232,62	cooling
9,186	22,5027	6,6649	221,39	
9,188	22,5889	0,8544	303,82	
9,19	22,8513	0,5813	311,3	
9,192	22,6471	0,4404	310,42	
9,194	22,9152	2,2571	288,77	
9,196	23,2498	6,6721	231,73	
9,198	22,8231	3,6141	268,51	
9,2	23,2259	-0,4121	330,42	
9,202	23,0195	-0,6323	330,62	
9,204	22,7923	-0,869	330,75	
9,206	22,9548	2,8479	281,06	
9,208	22,9527	5,9404	237,81	
9,21	22,8987	1,3289	301,51	
9,212	23,065	1,6553	299,28	
9,214	23,0226	0,9298	308,82	

9,104	36,6817	20,8341	221,53	
9,106	36,5269	18,7857	248	heating
9,108	36,6796	18,3647	256,02	heating
9,11	37,2043	18,2287	265,25	heating
9,112	36,7745	19,1044	247	heating
9,114	36,9872	17,5347	271,92	heating
9,116	37,5279	20,2756	241,16	heating
9,118	37,6069	17,6329	279,21	heating
9,12	37,4463	19,0186	257,59	heating
9,122	37,1044	17,0109	280,88	heating
9,124	37,7217	17,4447	283,44	heating
9,126	37,2086	15,6211	301,76	heating
9,128	37,2569	17,0299	282,74	heating
9,13	37,0094	16,2721	289,88	heating
9,132	37,3712	17,9124	272	heating
9,134	36,6403	17,52	267,27	heating
9,136	37,286	18,0856	268,39	heating
9,138	37,0898	18,0364	266,34	heating
9,14	37,2729	17,6276	274,61	heating
9,142	37,269	19,9102	242,65	heating
9,144	37,5406	18,9656	259,65	heating
9,146	37,4185	20,1604	241,24	heating
9,148	37,0666	17,0172	280,26	heating
9,15	37,7885	17,3529	285,66	heating
9,152	37,4393	15,7716	302,88	heating
9,154	37,1495	18,5068	260,6	heating
9,156	36,5985	19,007	245,9	heating
9,158	37,2704	22,6107	204,92	heating
9,16	36,7535	20,387	228,78	heating
9,162	37,3461	19,6561	247,28	heating
9,164	36,9774	16,9295	280,24	heating
9,166	37,0409	14,8075	310,79	heating
9,168	36,6304	14,0127	316,16	heating
9,17	37,0376	12,9901	336,15	heating
9,172	36,9173	13,7102	324,4	heating
9,174	36,8271	12,6635	337,77	heating
9,176	37,1825	14,9394	310,92	heating
9,178	37,3476	15,9053	299,73	heating
9,18	37,1028	17,8627	268,95	heating
9,182	37,039	14,807	310,77	heating
9,184	37,8536	15,3197	314,99	heating
9,186	37,7585	11,887	361,65	heating
9,188	37,7497	14,1802	329,47	heating
9,19	37,3491	14,844	314,59	heating
9,192	37,7399	18,5857	267,75	heating
9,194	36,8554	17,7358	267,26	heating
9,196	37,0123	17,9024	267,13	heating
9,198	36,9601	14,6346	312,08	heating
9,2	37,7658	15,205	315,37	heating
9,202	36,9744	14,0775	320,06	heating
9,204	37,8154	14,2527	329,37	heating
9,206	37,3194	15,1174	310,35	
9,208	37,4519	14,9284	314,85	
9,21	37,3445	16,9849	284,6	
9,212	37,9032	15,6611	310,91	
9,214	37,2177	12,7995	341,33	

9,104	32,3145	12,2385	280,63	heating
9,106	33,0366	15,935	239,06	heating
9,108	32,9777	12,516	286,02	heating
9,11	32,7465	16,3527	229,16	heating
9,112	32,3726	14,6404	247,87	heating
9,114	32,9034	13,5759	270,17	heating
9,116	32,2419	11,9515	283,63	heating
9,118	31,7592	12,7606	265,57	heating
9,12	31,5853	12,6613	264,53	heating
9,122	31,8902	16,4659	215,61	heating
9,124	31,3631	13,6771	247,22	heating
9,126	32,6424	13,642	265,6	heating
9,128	32,0946	12,2386	277,56	heating
9,13	32,3173	8,4059	334,25	heating
9,132	33,0274	14,4916	259,1	heating
9,134	33,4944	18,6061	208,12	heating
9,136	32,7882	18,1033	205,27	heating
9,138	33,2643	18,0267	213	heating
9,14	33,2482	16,3343	236,43	heating
9,142	33,6816	12,187	300,46	heating
9,144	33,2548	18,278	209,35	heating
9,146	32,7747	13,6986	266,66	heating
9,148	33,8591	12,1478	303,49	heating
9,15	33,593	11,0205	315,53	heating
9,152	33,0687	9,4427	330,26	heating
9,154	33,2256	11,9016	298,08	heating
9,156	33,3323	17,4574	221,91	heating
9,158	32,1994	12,4076	276,66	heating
9,16	33,4197	15,4632	251,01	heating
9,162	32,153	12,8575	269,72	heating
9,164	33,2282	9,1004	337,27	heating
9,166	33,0793	13,271	276,89	heating
9,168	33,4931	12,1958	297,71	heating
9,17	32,847	9,2584	329,73	heating
9,172	33,8949	11,7832	309,09	heating
9,174	33,6541	10,057	329,85	heating
9,176	34,3714	12,9268	299,76	heating
9,178	33,2263	18,3715	207,65	heating
9,18	32,9184	13,7135	268,46	heating
9,182	33,7099	13,3256	284,94	heating
9,184	32,8887	12,4425	285,81	heating
9,186	33,6567	12,0302	302,31	heating
9,188	33,3588	14,6368	261,71	heating
9,19	33,28	14,5168	262,28	heating
9,192	32,8009	10,547	311,08	heating
9,194	34,4839	13,0783	299,22	heating
9,196	32,9272	12,8004	281,34	heating
9,198	33,4617	14,3284	267,45	heating
9,2	33,996	17,3586	232,57	heating
9,202	34,1822	12,3599	305,04	heating
9,204	33,6645	9,8782	332,5	heating
9,206	33,8702	8,4902	354,77	heating
9,208	35,2669	9,4776	360,5	heating
9,21	34,5883	11,7413	319,37	heating
9,212	34,2776	14,9646	269,97	heating
9,214	34,0652	13,6407	285,5	heating

9,216	22,8156	-1,2112	335,86		9,216	37,282	9,0685	394,38		9,216	35,03	16,5439	258,41	heating
9,218	23,6948	2,28	299,35		9,218	37,3482	9,6295	387,47		9,218	33,3643	13,3267	280,1	heating
9,22	23,5809	5,3047	255,47		9,22	37,5118	10,6905	374,92		9,22	34,2989	14,5278	276,37	heating
9,222	24,1839	4,6091	273,63		9,222	37,2704	15,8781	299,03		9,222	33,3702	12,4829	291,97	heating
9,224	23,8563	5,8183	252,14		9,224	36,798	12,6698	337,28		9,224	33,2654	9,334	334,53	heating
9,226	23,7051	2,45	297,12		9,226	37,9442	11,7696	365,88		9,226	33,1146	9,9246	324,16	heating
9,228	23,4637	-3,2294	373,13		9,228	37,3204	9,444	389,67	cooling	9,228	33,9773	13,5764	285,17	heating
9,23	24,4543	1,1687	325,5		9,23	37,7924	12,6491	351,47	cooling	9,23	33,4001	15,2614	253,55	heating
9,232	23,677	1,335	312,31		9,232	37,6274	17,05	287,64	cooling	9,232	33,399	17,3176	224,79	heating
9,234	24,1552	2,2607	306,05		9,234	38,1341	21,0451	238,88	cooling	9,234	34,1263	17,6289	230,61	heating
9,236	23,8562	3,6447	282,53		9,236	36,9032	16,7649	281,5	cooling	9,236	34,2653	12,7439	300,84	heating
9,238	23,3848	0,244	323,47		9,238	37,8479	15,6286	310,59	cooling	9,238	34,0744	12,6407	299,61	heating
9,24	23,8374	-1,7486	357,65		9,24	37,0137	14,0885	320,46	cooling	9,24	33,8621	11,3037	315,33	heating
9,242	23,8851	2,4244	299,99		9,242	37,1737	13,2263	334,75	cooling	9,242	35,2403	14,592	288,63	heating
9,244	23,7954	0,003	332,58		9,244	36,9063	14,8098	308,88	cooling	9,244	33,9779	14,4948	272,34	heating
9,246	23,5305	2,1395	299,01		9,246	37,2193	12,8046	341,28	cooling	9,246	33,9845	14,0948	278,03	heating
9,248	23,4388	3,3598	280,67		9,248	36,8925	13,1708	331,59	cooling	9,248	33,9535	11,9822	307,13	heating
9,25	22,6014	-2,4056	349,56		9,25	36,7783	14,2082	315,5	cooling	9,25	34,6406	14,5472	280,88	heating
9,252	23,6798	0,3658	325,89		9,252	37,2625	15,9526	297,88	cooling	9,252	33,5291	14,3462	268,15	heating
9,254	22,8711	3,7408	267,41		9,254	37,4498	15,7962	302,69	cooling	9,254	34,2472	17,1676	238,75	heating
9,256	23,1767	4,3164	263,64		9,256	37,4052	17,9555	271,88	cooling	9,256	33,7694	15,1615	260,11	heating
9,258	23,1132	8,9185	198,42		9,258	36,8645	11,6134	352,97	cooling	9,258	33,4994	9,913	329,7	heating
9,26	22,5393	6,2585	227,58		9,26	37,9787	11,3391	372,38	cooling	9,26	33,7563	9,092	344,77	heating
9,262	22,7069	-0,9374	330,51		9,262	36,7786	9,022	388	cooling	9,262	34,1681	9,0011	351,8	heating
9,264	23,152	0,6979	313,88		9,264	37,074	11,1185	362,82	cooling	9,264	33,9675	10,5094	327,91	heating
9,266	22,8238	-1,8962	345,55		9,266	36,6764	14,2356	313,69	cooling	9,266	33,5345	10,5787	320,89	heating
9,268	23,0945	0,7696	312,07		9,268	36,6782	14,6411	308,05	cooling	9,268	34,4749	12,1492	312,08	
9,27	22,9762	5,1837	248,71		9,27	36,1218	12,4613	330,74	cooling	9,27	34,2742	11,6725	315,94	
9,272	21,8131	-2,3102	337,21		9,272	37,1236	16,5143	288,09	cooling	9,272	33,9908	15,0302	265,04	
9,274	23,1888	-1,4945	345,04		9,274	36,5745	16,9389	274,48	cooling	9,274	33,5751	13,4048	281,95	
9,276	21,9981	-0,1873	310,12		9,276	36,982	15,4957	300,35	cooling	9,276	34,678	13,724	292,91	
9,278	22,2593	-3,5746	361,12		9,278	37,0241	15,802	296,65	cooling	9,278	33,7131	10,0796	330,36	
9,28	22,4572	0,2664	310,19		9,28	37,1046	11,0203	364,62	cooling	9,28	34,0136	9,6621	340,4	
9,282	22,0252	-0,7175	317,91		9,282	37,0545	13,2685	332,49	cooling	9,282	33,9642	8,1516	360,82	
9,284	22,1356	-6,3039	397,54		9,284	36,5052	14,0986	313,21	cooling	9,284	34,2242	8,7678	355,84	
9,286	23,3724	0,4637	320,23	cooling	9,286	36,7983	16,0312	290,29	cooling	9,286	33,4791	10,4772	321,53	
9,288	22,7114	-1,3597	336,48	cooling	9,288	36,5888	17,0426	273,23	cooling	9,288	34,2085	13,0336	295,99	
9,29	23,199	-0,3391	329,03	cooling	9,29	36,1118	17,3945	261,64	cooling	9,29	34,0478	13,187	291,6	
9,292	23,3108	7,2679	224,26	cooling	9,292	35,819	11,7756	336,09	cooling	9,292	33,6836	10,2728	327,25	
9,294	22,2585	2,2742	279,35		9,294	36,6164	13,6751	320,68	cooling	9,294	34,6073	13,8473	290,19	
9,296	23,5339	2,0143	300,81		9,296	35,5481	11,3547	338,19	cooling	9,296	34,5253	13,0633	300,01	
9,298	22,7922	3,1818	274,12		9,298	35,9995	14,5725	299,52	cooling	9,298	34,2052	14,0286	282,04	
9,3	23,1698	-2,9467	365,07		9,3	35,6651	14,977	289,19	cooling	9,3	33,4662	10,4187	322,17	
9,302	23,4692	2,2755	296,26		9,302	36,0603	10,5555	356,52	cooling	9,302	34,7974	9,6483	351,55	
9,304	22,6102	5,0106	246,02		9,304	36,0657	7,7317	396,07	cooling	9,304	34,102	7,2911	374,78	
9,306	22,2718	1,7432	286,96		9,306	36,6957	9,9666	373,63	cooling	9,306	34,2309	12,0774	309,67	
9,308	23,4345	8,9403	202,61		9,308	36,4779	12,5502	334,47	cooling	9,308	33,9351	12,3044	302,37	
9,31	22,2732	4,7909	244,38		9,31	36,4084	15,3972	293,7	cooling	9,31	34,6641	13,2427	299,44	
9,312	22,727	-1,5489	339,34		9,312	35,6694	13,6114	308,34	cooling	9,312	33,2815	10,6471	316,4	
9,314	22,9595	2,223	289,86		9,314	36,0611	10,1296	362,48	cooling	9,314	33,6996	10,5356	323,8	
9,316	22,1122	-0,8157	320,5		9,316	35,5025	14,8269	289,01	cooling	9,316	33,8034	12,6136	296,2	
9,318	22,7586	1,9612	290,72		9,318	34,8766	16,495	256,95	cooling	9,318	33,3726	11,2403	309,38	
9,32	22,2497	6,1332	225,28		9,32	35,3018	20,0362	213,39	cooling	9,32	33,363	10,2717	322,78	
9,322	22,0762	-0,8894	321,03		9,322	35,0639	17,0616	251,64	cooling	9,322	33,776	9,1383	344,4	
9,324	22,3253	-0,032	312,52		9,324	34,9838	14,151	291,21	cooling	9,324	33,6003	11,4374	309,8	
9,326	21,501	1,5298	279,17		9,326	35,2729	13,786	300,36	cooling	9,326	32,9075	10,0853	319,02	

9,328	20,9982	-3,4732	342,07		9,328	35,924	17,3386	259,8	cooling	9,328	34,6894	15,0076	275,12	cooling
9,33	22,0417	1,4358	288,04		9,33	35,6578	17,3991	255,23		9,33	34,5972	11,4726	323,25	
9,332	21,0404	0,3941	288,61		9,332	36,3924	21,3314	210,53		9,332	34,5195	12,6949	305,08	
9,334	21,4581	-1,6152	322,53		9,334	35,8994	19,5799	228,12		9,334	33,7097	9,6608	336,17	
9,336	21,3479	3,0161	256,25		9,336	36,125	15,1944	292,58		9,336	35,0545	10,7643	339,54	cooling
9,338	20,3762	-0,734	295,09		9,338	35,5289	13,3826	309,57		9,338	34,3313	9,3232	349,58	cooling
9,34	20,8075	-0,9573	304,24		9,34	35,9214	12,6745	324,96		9,34	33,8515	12,7631	294,78	cooling
9,342	21,3467	6,3195	210,06		9,342	35,518	13,3469	309,92		9,342	33,934	13,0949	291,3	cooling
9,344	21,1227	3,0121	253,16		9,344	35,5554	13,1598	313,06		9,344	34,7052	14,6821	279,89	cooling
9,346	21,6797	1,8658	276,97		9,346	35,3779	12,4513	320,48		9,346	33,3717	11,1076	311,22	cooling
9,348	21,3957	2,4195	265,26		9,348	36,2029	13,4397	318,2		9,348	33,4928	10,6323	319,56	cooling
9,35	21,1998	-3,7513	348,78	heating	9,35	36,0457	17,2158	263,21		9,35	33,7709	12,1415	302,35	cooling
9,352	22,0769	-0,2409	311,97		9,352	36,0831	14,484	301,92		9,352	33,4092	9,4099	335,47	cooling
9,354	21,4835	1,8238	274,81		9,354	37,0565	14,9818	308,57		9,354	33,0088	8,5097	342,46	cooling
9,356	21,8834	0,5082	298,79		9,356	36,6313	12,6118	335,76		9,356	33,5551	6,8732	372,97	cooling
9,358	22,1413	4,4215	247,7		9,358	36,7059	13,573	323,36		9,358	33,2133	7,199	363,64	cooling
9,36	21,5616	1,1876	284,8		9,36	36,4422	16,7868	274,75		9,36	32,694	7,2568	355,57	cooling
9,362	21,6624	-4,862	370,77		9,362	36,5291	18,1894	256,36		9,362	33,0621	11,9667	294,88	cooling
9,364	22,6289	-0,7526	326,84		9,364	35,7088	15,6458	280,45		9,364	33,6532	8,0347	358,11	cooling
9,366	21,7917	-2,3543	337,53		9,366	36,3578	17,9922	256,72		9,366	33,3002	8,7128	343,7	cooling
9,368	22,6633	2,572	280,85		9,368	35,6184	17,2319	257,02		9,368	32,7568	5,2339	384,73	cooling
9,37	22,1777	6,387	220,73		9,37	36,2999	16,4951	276,84		9,37	34,1599	7,4424	373,47	cooling
9,372	21,7654	-2,5555	339,97		9,372	35,9357	17,1602	262,45		9,372	33,9041	7,6769	366,62	cooling
9,374	22,3968	-5,0068	383,06		9,374	36,5458	15,0402	300,62		9,374	33,2009	9,5491	330,62	cooling
9,376	22,1741	-2,2057	340,79		9,376	36,3593	15,9053	285,92		9,376	33,2084	6,9437	367,14	cooling
9,378	22,3122	-1,5763	333,92		9,378	36,3049	16,5361	276,34		9,378	34,2999	8,1066	366,14	cooling
9,38	22,4064	4,3689	252,14		9,38	36,4905	17,2762	268,59		9,38	32,916	8,0587	347,47	cooling
9,382	21,8251	4,0312	248,73		9,382	37,1449	20,538	232,14		9,382	33,3291	11,5402	304,58	cooling
9,384	21,6367	-1,3793	321,73		9,384	36,9114	22,5264	201,08	heating	9,384	33,2782	14,2325	266,23	cooling
9,386	22,257	1,632	288,31		9,386	36,9066	18,0468	263,63	heating	9,386	33,2179	10,0483	323,88	cooling
9,388	21,1715	-1,2573	313,52		9,388	37,9873	17,9822	279,64	heating	9,388	32,7092	9,354	326,47	cooling
9,39	22,0076	0,1889	304,99		9,39	37,3393	12,5593	346,39	heating	9,39	33,4194	8,5388	347,79	cooling
9,392	21,6777	3,121	259,4		9,392	37,9678	15,5162	313,84	heating	9,392	33,0716	10,0865	321,3	cooling
9,394	21,5015	1,7245	276,45		9,394	37,0177	17,7038	269,98	heating	9,394	32,7595	11,0412	303,59	cooling
9,396	21,857	1,0336	291,08		9,396	37,2675	17,5641	275,42	heating	9,396	33,4743	14,2963	268,08	cooling
9,398	21,7902	0,1232	302,87		9,398	36,4214	14,8093	302,1	heating	9,398	33,6732	10,1626	328,64	cooling
9,4	22,1051	-2,2304	340,17		9,4	36,8475	15,822	293,9	heating	9,4	33,34	13,1894	281,68	cooling
9,402	22,2485	2,2022	280,22		9,402	36,4776	13,6402	319,23	heating	9,402	32,6808	12,8012	277,89	cooling
9,404	21,9656	3,75	254,63		9,404	37,1296	17,8328	269,74	heating	9,404	34,2838	15,6929	259,87	cooling
9,406	21,7543	0,4653	297,59		9,406	36,407	18,5289	249,91	heating	9,406	33,419	13,9374	272,32	cooling
9,408	22,1008	-0,7439	319,34		9,408	36,9145	18,6744	254,97	heating	9,408	33,3065	11,2479	308,35	cooling
9,41	21,147	-6,9978	393,42		9,41	36,7981	19,9327	235,75	heating	9,41	33,1408	8,6992	341,66	cooling
9,412	21,9739	-5,5838	385,22		9,412	36,1921	15,0149	296,03	heating	9,412	33,771	8,9874	346,44	cooling
9,414	21,7175	-1,3727	322,77		9,414	36,9841	14,3206	316,8	heating	9,414	32,3883	12,6212	276,32	cooling
9,416	21,6819	0,5554	295,32		9,416	36,571	13,5686	321,54	heating	9,416	33,0814	15,0814	251,61	cooling
9,418	21,7609	1,3514	285,29		9,418	36,4613	14,6784	304,49	heating	9,418	32,8798	17,0921	220,69	cooling
9,42	21,8255	0,7207	295,01		9,42	36,434	15,6666	290,3	heating	9,42	32,5328	10,7909	303,92	cooling
9,422	22,0803	-5,811	389,88		9,422	37,077	17,7874	269,64	heating	9,422	32,5844	11,2195	298,65	cooling
9,424	22,3558	-4,276	372,27		9,424	37,1346	15,9998	295,43	heating	9,424	33,2716	9,2653	335,57	cooling
9,426	22,5016	-1,8419	340,29	cooling	9,426	37,6469	20,6047	238,22	heating	9,426	32,9523	12,221	289,79	cooling
9,428	22,4231	-0,8397	325,18	cooling	9,428	37,1661	15,8789	297,56	heating	9,428	32,35	10,829	300,83	cooling
9,43	23,0766	2,8437	282,83	cooling	9,43	37,6106	13,9627	330,56	heating	9,43	33,5452	14,5118	266,06	cooling
9,432	22,2016	1,1778	293,88	cooling	9,432	36,7959	12,376	341,35	heating	9,432	33,076	11,5913	300,32	
9,434	23,4231	-0,7833	338,37	cooling	9,434	36,4917	11,2053	353,47	heating	9,434	33,1091	16,0634	238,27	
9,436	23,3808	-1,4803	347,52	cooling	9,436	36,604	17,2791	270,13		9,436	32,2483	14,8537	243,15	
9,438	24,1855	-0,0648	338,98	cooling	9,438	35,7578	18,5562	240,45		9,438	34,1426	15,9928	253,71	

9,44	24,342	3,0837	297,16	cooling	9,44	35,771	16,0743	275,33	heating	9,44	32,7617	11,8698	292,04	
9,442	25,1991	8,0136	240,23	cooling	9,442	36,5316	15,5511	293,28	heating	9,442	33,1575	10,9222	310,82	
9,444	24,6768	2,456	310,61	cooling	9,444	35,9371	14,0178	306,4	heating	9,444	32,7068	9,4333	325,33	
9,446	25,1828	2,6859	314,47	cooling	9,446	36,2378	12,7489	328,34	heating	9,446	34,0599	12,6085	299,86	
9,448	24,8896	0,2107	344,97	cooling	9,448	36,3958	17,2029	268,29		9,448	32,3421	14,098	255,03	
9,45	24,8484	1,7255	323,22	cooling	9,45	36,1498	12,1377	335,65	heating	9,45	33,1205	14,7151	257,28	
9,452	25,0403	5,99	266,29	cooling	9,452	36,5691	14,0228	315,16		9,452	32,802	13,9943	262,9	
9,454	24,3518	6,4681	249,99	cooling	9,454	36,5493	15,2663	297,5		9,454	32,6134	9,9233	317,17	
9,456	25,4966	5,1711	284,12		9,456	37,3147	15,572	303,93		9,456	32,4344	11,8937	287,13	
9,458	24,4949	4,7686	275,74		9,458	37,536	18,1714	270,69		9,458	32,9088	11,1287	304,45	
9,46	24,5455	-0,6408	352,07		9,46	37,4927	20,1005	243,12		9,46	32,7082	12,6974	279,72	
9,462	24,2815	-1,2391	356,74		9,462	37,4478	17,751	275,33	heating	9,462	32,0061	11,1018	292,21	
9,464	25,0432	3,3014	303,92	cooling	9,464	37,8862	18,5754	269,94	heating	9,464	33,376	14,36	265,81	
9,466	24,1528	1,3765	318,38	cooling	9,466	36,7425	15,4974	296,98	heating	9,466	33,1212	9,5649	329,28	
9,468	25,2759	7,1738	253,04	cooling	9,468	37,3306	13,3513	335,2	heating	9,468	33,4673	11,1937	311,35	
9,47	24,2923	4,8061	272,39		9,47	36,5238	15,1651	298,56		9,47	32,5468	7,0307	356,68	
9,472	24,6519	0,2677	340,86		9,472	36,3746	12,6044	332,27		9,472	34,5359	9,528	349,57	
9,474	24,6765	1,1976	328,2		9,474	36,5632	14,248	311,93		9,474	33,467	10,4229	322,12	
9,476	23,9361	0,7823	323,66		9,476	37,1633	14,3711	318,6		9,476	33,5966	13,7812	276,99	
9,478	24,4321	-0,7927	352,61		9,478	36,9569	13,6636	325,61		9,478	33,2741	13,7261	273,25	
9,48	24,0246	4,6934	270,22	cooling	9,48	37,7098	16,5659	295,56		9,48	34,2933	15,305	265,43	
9,482	23,6478	-1,5874	352,75		9,482	37,999	18,5229	272,25		9,482	32,5362	12,8905	274,62	
9,484	23,837	-2,3298	365,77	cooling	9,484	38,3843	15,6898	317,23		9,484	33,1324	11,8232	297,87	
9,486	24,1043	1,6085	314,46	cooling	9,486	38,5902	18,8408	276,07		9,486	33,1306	11,9716	295,77	
9,488	22,9647	-2,4875	355,78	cooling	9,488	37,682	15,1995	314,27		9,488	32,8904	9,4676	327,42	
9,49	24,478	1,3232	323,67	cooling	9,49	38,2836	15,3218	320,97		9,49	32,8757	13,9522	264,52	
9,492	23,3489	1,9683	298,87	cooling	9,492	36,9163	15,6012	297,95		9,492	33,1964	14,1048	266,87	
9,494	23,8412	-3,7637	385,88	cooling	9,494	37,0117	14,3191	317,21		9,494	33,2915	17,5365	220,23	
9,496	23,8492	-2,828	372,91	cooling	9,496	36,7775	15,628	295,64		9,496	32,1591	13,519	260,56	
9,498	23,5347	-2,8956	369,46	cooling	9,498	36,9874	18,1239	263,68		9,498	33,6864	16,8981	234,68	
9,5	23,5243	-4,4419	390,93	cooling	9,5	36,446	16,9623	272,35		9,5	33,4887	14,4112	266,68	heating
9,502	24,0019	4,9769	265,94	cooling	9,502	37,6404	18,5318	267,11		9,502	33,6064	17,7792	221,24	heating
9,504	23,0273	1,6478	298,85	cooling	9,504	36,9815	15,4199	301,4		9,504	32,6102	13,2475	270,66	heating
9,506	23,4465	0,3499	322,86	cooling	9,506	37,59	13,3807	338,41		9,506	34,7595	16,1987	259,45	heating
9,508	23,6849	4,3287	270,57	cooling	9,508	37,5207	17,1049	285,38		9,508	33,634	13,3192	283,97	heating
9,51	22,6862	0,0573	316,32	cooling	9,51	37,0589	13,7695	325,55		9,51	33,8904	17,5287	228,71	heating
9,512	23,6484	-1,2086	347,47	cooling	9,512	37,0437	13,1693	333,73		9,512	33,4856	18,1213	214,77	heating
9,514	22,974	0,5256	313,79	cooling	9,514	36,5175	11,941	343,54		9,514	34,4721	18,8569	218,28	heating
9,516	23,2813	-2,4211	359,28	cooling	9,516	36,9753	11,2193	360,03		9,516	33,1754	17,6556	216,94	heating
9,518	23,4874	4,8686	260,26	cooling	9,518	36,6357	14,5955	308,09		9,518	33,6426	15,9783	246,92	heating
9,52	22,9054	6,6114	227,77	cooling	9,52	36,4418	19,5662	235,9		9,52	33,9459	15,0064	264,75	heating
9,522	22,2419	-3,0947	354,17	cooling	9,522	35,8993	14,6111	297,58		9,522	33,7971	11,8109	307,33	heating
9,524	23,2098	-0,8878	336,85	cooling	9,524	37,3612	14,8531	314,63		9,524	33,8141	13,8443	279,15	heating
9,526	21,9939	-2,0233	335,73	cooling	9,526	36,1802	9,3089	375,62		9,526	34,3806	12,6477	303,79	heating
9,528	22,564	-1,2044	332,25	cooling	9,528	37,2582	9,5552	387,25		9,528	34,4055	17,5888	235,07	heating
9,53	23,143	5,0281	253,22	cooling	9,53	37,04	14,0909	320,79		9,53	33,2315	13,9614	269,37	heating
9,532	22,5841	-0,0498	316,39	cooling	9,532	37,1032	15,4602	302,54		9,532	34,7488	20,0232	205,84	heating
9,534	24,0539	-3,2154	381,18	cooling	9,534	37,0577	17,9382	267,26		9,534	34,1891	16,3044	250	heating
9,536	24,251	2,9121	298,29	cooling	9,536	37,2693	21,5533	219,69		9,536	34,1379	15,6514	258,41	heating
9,538	24,0341	-2,5751	371,96	cooling	9,538	36,3784	18,4008	251,3		9,538	33,4144	10,713	317,33	heating
9,54	24,5637	0,0789	342,26	cooling	9,54	35,9149	15,7811	281,44		9,54	35,0284	12,4247	315,97	heating
9,542	24,7908	6,0511	261,95	cooling	9,542	35,7368	15,9546	276,53		9,542	33,7794	10,586	324,21	heating
9,544	24,0149	0,4048	330,03	cooling	9,544	35,3975	11,4538	334,7		9,544	34,3225	17,7385	231,82	heating
9,546	25,3712	4,8384	287,02	cooling	9,546	36,0511	15,4515	287,95		9,546	33,6675	19,0309	204,6	heating
9,548	24,4419	4,1878	283,12	cooling	9,548	35,4488	13,866	301,7		9,548	34,8335	17,385	243,9	heating
9,55	25,1333	-2,3081	383,59	cooling	9,55	36,7222	13,9598	318,18		9,55	33,5856	15,5693	251,84	heating

9,552	25,5628	4,5236	294,1	cooling	9,552	36,7604	15,5813	296,05		9,552	33,9374	11,4647	314,14	heating
9,554	24,3482	4,7623	273,78	cooling	9,554	36,882	16,1608	289,65		9,554	34,1046	11,9146	310,18	heating
9,556	24,8869	1,9	321,32	cooling	9,556	36,6196	11,7584	347,52		9,556	33,9462	11,8037	309,52	heating
9,558	25,2221	7,4465	248,48	cooling	9,558	37,0895	12,8883	338,3		9,558	33,5236	14,7756	262,07	heating
9,56	24,1436	0,8944	324,99	cooling	9,56	35,5992	8,7763	374,94		9,56	34,2936	14,3585	278,66	heating
9,562	25,1076	0,7394	340,63	cooling	9,562	35,7469	9,5323	366,44		9,562	34,2671	19,4015	207,8	heating
9,564	25,1273	8,6894	229,78	cooling	9,564	34,8523	14,5401	283,93		9,564	33,209	13,9755	268,86	heating
9,566	24,0156	1,5294	314,32	cooling	9,566	34,2689	12,9881	297,47		9,566	34,8207	16,8895	250,65	heating
9,568	24,6993	1,7442	320,88	cooling	9,568	34,5427	12,6205	306,44		9,568	34,597	10,9517	330,53	heating
9,57	24,403	2,4839	306,4	cooling	9,57	35,3375	13,2015	309,43		9,57	34,7069	11,6481	322,33	heating
9,572	24,0007	-6,8051	430,62	cooling	9,572	35,8772	14,2511	302,3		9,572	33,9524	10,0388	334,28	heating
9,574	24,9168	-0,1586	350,52	cooling	9,574	36,7023	16,6956	279,67		9,574	35,6779	14,2313	299,79	heating
9,576	23,8607	4,3756	272,37	cooling	9,576	36,9791	17,3409	274,51		9,576	34,4872	10,6569	333,11	heating
9,578	24,6067	1,7796	319,09	cooling	9,578	37,0918	12,2607	347,1		9,578	34,7874	15,0169	276,36	heating
9,58	25,13	5,0841	280,21	cooling	9,58	37,1262	15,0161	309,07		9,58	33,6936	11,0114	317,06	heating
9,582	24,579	-0,1462	345,62	cooling	9,582	36,0431	12,7262	325,94		9,582	35,4157	13,0205	313,05	heating
9,584	25,3051	-5,8831	435,96	cooling	9,584	36,4726	16,5797	278,07		9,584	33,8245	14,7172	267,09	heating
9,586	25,5492	2,5508	321,48	cooling	9,586	35,401	18,7924	232,16		9,586	34,1822	11,7869	313,05	heating
9,588	24,6594	2,3705	311,57	cooling	9,588	35,3471	18,3095	238,16		9,588	34,5778	12,421	309,72	heating
9,59	25,222	2,9385	311,49	cooling	9,59	35,3314	16,8442	258,42		9,59	34,6305	12,1854	313,75	heating
9,592	25,5255	8,0518	244,26	cooling	9,592	35,8603	16,4274	271,64		9,592	33,861	12,1729	303,17	heating
9,594	24,3534	4,4803	277,8	cooling	9,594	35,2926	12,3081	321,29		9,594	34,8264	15,2364	273,84	heating
9,596	25,2116	4,6673	287,18	cooling	9,596	36,6483	18,4956	253,75		9,596	34,3561	18,3489	223,76	heating
9,598	24,4007	4,8907	272,72	cooling	9,598	35,8943	20,2132	219,2		9,598	33,6003	11,9077	303,23	heating
9,6	24,5821	-1,721	367,68	cooling	9,6	36,2944	18,2449	252,31		9,6	34,9937	16,8296	253,91	heating
9,602	24,7057	-0,278	349,24	cooling	9,602	36,37	19,136	240,91		9,602	34,1404	11,6187	314,82	heating
9,604	25,0853	5,7501	270,28	cooling	9,604	36,2595	13,5414	317,57	heating	9,604	34,6448	13,7192	292,51	heating
9,606	24,5853	4,2166	284,72	cooling	9,606	36,6693	12,182	342,3		9,606	33,6318	14,5297	267,02	heating
9,608	25,5679	6,0111	273,38	cooling	9,608	36,3237	15,8638	286		9,608	35,0326	16,5575	258,25	heating
9,61	24,8804	1,859	321,81	cooling	9,61	36,71	17,9846	261,75		9,61	34,1666	14,7094	271,98	heating
9,612	25,565	-1,9922	385,21	cooling	9,612	37,1169	19,2125	250,28		9,612	34,7923	16,7316	252,46	heating
9,614	25,3972	-1,3386	373,73	cooling	9,614	37,6585	21,3059	228,58	heating	9,614	34,2157	12,6472	301,5	heating
9,616	25,1231	-0,0713	352,18	cooling	9,616	37,2182	13,8524	326,62	heating	9,616	35,9041	15,0828	291,05	heating
9,618	25,213	0,1614	350,18	cooling	9,618	38,8105	16,9772	305,2	heating	9,618	34,2735	17,1147	239,85	heating
9,62	25,1622	2,9158	310,97	cooling	9,62	37,2745	12,1632	351,02	heating	9,62	34,9889	15,2775	275,54	heating
9,622	24,959	1,4632	328,44	cooling	9,622	37,5958	11,6271	363		9,622	35,2537	18,8116	229,84	heating
9,624	24,7901	1,7447	322,14	cooling	9,624	37,0477	14,1646	319,87		9,624	34,7248	14,011	289,55	heating
9,626	24,938	1,4396	328,47	cooling	9,626	36,5106	13,3893	323,2		9,626	35,1657	14,5691	287,91	heating
9,628	24,7727	0,109	344,76	cooling	9,628	36,2374	13,5251	317,48		9,628	35,5406	13,8454	303,27	heating
9,63	25,744	1,5095	338,76	cooling	9,63	36,3142	19,358	237,02		9,63	35,2948	15,5477	276,04	heating
9,632	25,1928	-2,7483	390,57	cooling	9,632	35,9825	15,5713	285,32		9,632	34,3519	10,1445	338,38	heating
9,634	26,1634	-2,8179	405,11	cooling	9,634	36,3057	15,5224	290,52		9,634	35,684	14,7448	292,7	heating
9,636	25,6983	-0,5888	367,45	cooling	9,636	36,478	17,2207	269,19		9,636	34,718	10,6104	336,99	heating
9,638	25,7629	2,7817	321,24	cooling	9,638	36,5633	11,9864	343,55		9,638	35,4308	17,6763	248,18	heating
9,64	25,9118	8,0262	250,01	cooling	9,64	38,0837	18,5497	273,06		9,64	34,0662	17,8811	226,24	heating
9,642	25,7857	7,4435	256,4	cooling	9,642	37,3402	17,4918	277,45		9,642	35,7754	18,9641	235	heating
9,644	26,1973	-0,1602	368,44	cooling	9,644	38,6753	19,1269	273,26		9,644	34,4583	13,531	292,53	heating
9,646	26,4635	-0,2106	372,87	cooling	9,646	38,8266	21,2696	245,42		9,646	35,1818	10,4686	345,45	heating
9,648	26,2683	-0,9182	380,03	cooling	9,648	38,3415	20,3209	251,9		9,648	34,493	5,3168	407,84	heating
9,65	25,9144	-1,0927	377,52	cooling	9,65	38,3886	17,6787	289,49	heating	9,65	35,9767	8,3592	386,05	heating
9,652	26,4854	5,8932	287,85	cooling	9,652	38,5347	22,2053	228,26	heating	9,652	34,4124	10,6357	332,36	
9,654	25,4157	5,6431	276,39	cooling	9,654	37,7264	16,7761	292,85	heating	9,654	35,3804	11,9475	327,56	
9,656	26,454	5,983	286,15	cooling	9,656	38,2686	18,9711	269,75	heating	9,656	35,3425	17,4451	250,18	
9,658	25,7839	4,2315	301,27	cooling	9,658	37,8754	18,8175	266,4	heating	9,658	35,0843	14,5044	287,68	
9,66	25,7538	-2,7918	399,03	cooling	9,66	38,2355	15,3726	319,59	heating	9,66	35,2191	13,5703	302,62	
9,662	26,1534	-2,4804	400,26	cooling	9,662	38,8454	19,4477	271,15	heating	9,662	35,712	11,3716	340,24	

9,664	26,3092	4,3336	307,19	cooling	9,664	38,7299	19,1141	274,2	heating	9,664	35,4335	12,6189	318,91	
9,666	25,7703	0,824	348,71	cooling	9,666	38,7387	19,1132	274,33	heating	9,666	34,4296	7,5858	375,24	
9,668	26,32	-0,2191	370,98	cooling	9,668	38,436	19,2583	268,08	heating	9,668	35,9789	14,7945	296,13	
9,67	26,1136	-0,3581	370,03	cooling	9,67	37,6669	16,4713	296,28	heating	9,67	34,7253	11,231	328,42	
9,672	25,9632	2,3806	329,65	cooling	9,672	38,2	16,5187	303,07	heating	9,672	35,306	13,9688	298,26	
9,674	26,2735	8,9091	242,73	cooling	9,674	37,3464	19,966	242,95	heating	9,674	34,1192	11,4073	317,48	
9,676	25,1399	4,4675	288,97	cooling	9,676	37,2079	16,777	285,59	heating	9,676	35,7082	12,2141	328,41	
9,678	25,923	1,5324	340,94	cooling	9,678	37,9177	18,6012	270,02	heating	9,678	34,8133	8,8229	363,31	
9,68	25,2561	2,986	311,3	cooling	9,68	37,6763	13,3519	340,02	heating	9,68	34,9624	10,1787	346,44	
9,682	25,1627	1,8265	326,21	cooling	9,682	38,3809	11,6075	374,25	heating	9,682	34,213	7,8048	369,15	
9,684	25,6122	3,3262	311,52	cooling	9,684	38,6001	13,2254	354,7	heating	9,684	35,7887	12,0194	332,26	
9,686	26,2989	7,7073	259,88	cooling	9,686	39,3403	15,4433	334,04		9,686	34,1177	10,6761	327,68	cooling
9,688	26,2512	5,102	295,63	cooling	9,688	38,8539	15,5183	326,2		9,688	34,6471	8,5916	364,22	cooling
9,69	27,181	9,3496	249,26	cooling	9,69	38,9519	17,6823	297,32		9,69	35,0438	11,3097	331,77	cooling
9,692	26,8753	9,3748	244,63	cooling	9,692	37,8766	14,0722	332,75		9,692	34,7561	8,7103	364,08	cooling
9,694	26,8613	5,0978	304,22	cooling	9,694	38,4553	17,3219	295,41		9,694	34,7139	11,593	323,2	cooling
9,696	27,3322	2,9881	340,29	cooling	9,696	37,0813	19,963	239,29		9,696	34,9142	12,7824	309,37	cooling
9,698	26,9474	-0,3965	382,23	cooling	9,698	37,751	20,9582	234,74		9,698	34,9195	13,7341	296,14	cooling
9,7	27,8836	1,2089	372,87	cooling	9,7	37,3371	21,0482	227,69		9,7	34,2633	10,1101	337,63	cooling
9,702	27,5289	8,0372	272,47	cooling	9,702	37,2436	15,1808	308,41	heating	9,702	35,2519	16,6035	260,68	cooling
9,704	27,2197	6,5902	288,37	cooling	9,704	37,5781	14,4455	323,36	heating	9,704	34,5204	12,906	302,14	cooling
9,706	27,2047	3,9573	324,96	cooling	9,706	37,8862	17,76	281,33		9,706	35,0087	16,2581	262,11	cooling
9,708	27,2361	4,1445	322,79	cooling	9,708	37,3209	18,5706	262,1		9,708	33,7438	12,483	297,19	cooling
9,71	26,5846	-1,2614	389,25	cooling	9,71	37,3644	18,5855	262,5	heating	9,71	35,1878	13,1767	307,68	cooling
9,712	27,1037	-0,0628	379,75	cooling	9,712	37,903	21,1808	233,75	heating	9,712	34,3365	11,5663	318,29	cooling
9,714	26,7255	1,8082	348,31	cooling	9,714	37,9765	17,7534	282,69	heating	9,714	34,4431	13,0585	298,92	cooling
9,716	26,6604	2,1032	343,27	cooling	9,716	38,9581	20,0559	264,22	heating	9,716	34,1379	12,6735	300,04	cooling
9,718	27,0843	5,6996	298,93	cooling	9,718	38,7396	17,7169	293,87	heating	9,718	34,9467	16,1586	262,63	cooling
9,72	26,7682	4,3036	314,02	cooling	9,72	39,9413	17,6828	311,14	heating	9,72	33,5307	14,9251	260,08	
9,722	26,8901	-2,1085	405,36	cooling	9,722	39,0425	17,7723	297,32	heating	9,722	34,1667	12,1789	307,36	
9,724	26,8421	-0,862	387,26	cooling	9,724	39,0487	19,3382	275,52	heating	9,724	34,0586	13,0862	293,16	
9,726	26,4565	-0,2926	373,91	cooling	9,726	38,2051	17,3943	290,9	heating	9,726	33,6977	6,578	379,09	
9,728	26,3795	-0,2026	371,58	cooling	9,728	38,6411	18,657	279,35	heating	9,728	34,0153	10,2132	332,72	
9,73	26,6254	3,4638	323,76	cooling	9,73	37,8573	13,9863	333,68	heating	9,73	34,0766	8,9203	351,65	cooling
9,732	26,1905	0,721	356,03	cooling	9,732	38,8472	12,3174	370,85	heating	9,732	34,279	9,7292	343,17	cooling
9,734	26,7339	-0,0905	374,96	cooling	9,734	39,3306	15,2868	336,1		9,734	34,0376	7,5412	370,38	cooling
9,736	26,92	3,1015	332,95	cooling	9,736	39,1814	15,2685	334,27		9,736	35,0314	14,1235	292,26	cooling
9,738	26,4628	2,1803	339,43	cooling	9,738	40,0012	18,887	295,14		9,738	34,334	11,6665	316,86	cooling
9,74	26,798	2,6873	337,03	cooling	9,74	39,8001	19,3931	285,26		9,74	34,8883	16,9969	250,1	cooling
9,742	26,5728	2,8118	332,14	cooling	9,742	39,831	18,5942	296,86		9,742	33,5909	11,9343	302,73	cooling
9,744	26,3653	-1,1873	385,14	cooling	9,744	39,2787	17,2189	308,36		9,744	34,8099	10,8775	334,54	cooling
9,746	26,229	1,7156	342,66	cooling	9,746	39,4628	20,0518	271,34		9,746	33,8867	10,5713	325,91	cooling
9,748	25,6198	4,4343	296,14	cooling	9,748	38,7145	16,1816	314,98		9,748	34,0582	12,866	296,23	cooling
9,75	25,2064	3,0363	309,91	cooling	9,75	39,1156	19,3141	276,8		9,75	33,7186	14,2322	272,39	cooling
9,752	25,387	5,7685	274,24	cooling	9,752	38,7551	15,8236	320,55		9,752	34,2808	13,8496	285,6	cooling
9,754	24,5342	2,3726	309,79	cooling	9,754	39,6013	14,2391	354,53		9,754	33,1825	9,9786	324,36	cooling
9,756	24,5576	-1,5522	364,98	cooling	9,756	39,9273	15,5139	341,26		9,756	34,0357	8,2094	361,01	cooling
9,758	24,4067	0,7551	330,61	cooling	9,758	39,9287	17,5964	312,17		9,758	33,7363	9,4064	340,1	cooling
9,76	23,6848	-1,2667	348,79	cooling	9,76	40,1967	18,6738	300,86		9,76	34,0213	6,2947	387,58	cooling
9,762	24,4185	-0,3366	346,04	cooling	9,762	40,3577	18,7023	302,71		9,762	34,0224	11,4077	316,12	cooling
9,764	24,9408	2,9159	307,88	cooling	9,764	40,2182	15,0829	351,35		9,764	33,8905	7,7091	365,98	cooling
9,766	25,0144	-0,955	363,01	cooling	9,766	40,8521	13,0125	389,16		9,766	34,2033	10,257	334,73	cooling
9,768	25,6414	0,9181	345,59	cooling	9,768	40,3627	15,1026	353,1		9,768	33,7073	9,437	339,26	cooling
9,77	25,2993	2,8873	313,29	cooling	9,77	40,4559	14,5726	361,81		9,77	34,0362	11,2213	318,92	cooling
9,772	25,1343	-2,8525	391,21	cooling	9,772	40,6883	19,6269	294,41	cooling	9,772	33,9363	8,0121	362,38	cooling
9,774	25,5814	-2,5578	393,34	cooling	9,774	40,8006	20,9268	277,81	cooling	9,774	33,8123	12,4102	299,17	cooling

9,776	25,2502	-0,7677	363,69	cooling	9,776	40,5145	22,1505	256,7		9,776	32,7529	7,0422	359,4	cooling
9,778	25,609	3,3239	311,51	cooling	9,778	40,6262	21,2035	271,5		9,778	34,1839	10,2506	334,55	cooling
9,78	25,7215	9,5466	226,1	cooling	9,78	40,8276	19,5912	296,85		9,78	33,1916	8,8803	339,84	cooling
9,782	25,3492	4,8538	286,5	cooling	9,782	40,4807	14,6776	360,69		9,782	33,7387	9,4821	339,07	cooling
9,784	26,0146	-1,7517	388,13	cooling	9,784	40,8109	17,3169	328,41		9,784	33,163	10,86	311,76	cooling
9,786	26,4996	-0,98	384,12	cooling	9,786	40,2165	16,8168	327,09		9,786	33,7293	10,3563	326,72	cooling
9,788	26,1191	0,4143	359,31	cooling	9,788	40,8199	19,2992	300,83		9,788	32,6585	7,5258	351,32	cooling
9,79	26,2087	3,4157	318,61	cooling	9,79	39,9867	20,4737	272,76		9,79	33,5378	8,4335	350,92	cooling
9,792	25,9697	5,2805	289,21	cooling	9,792	40,5666	19,251	297,96		9,792	32,8484	9,4533	327,03	cooling
9,794	26,0779	3,7649	311,9	cooling	9,794	40,2132	15,1029	351,01		9,794	33,651	10,0343	330,13	cooling
9,796	26,3362	5,9714	284,67	cooling	9,796	40,4357	13,3826	378,16		9,796	32,8634	13,3178	273,22	cooling
9,798	26,7398	5,5328	296,44	cooling	9,798	40,075	14,9795	350,8	cooling	9,798	33,3258	10,7387	315,73	cooling
9,8	27,0495	2,3204	345,68	cooling	9,8	40,5867	20,2742	283,94	cooling	9,8	33,4138	16,5314	235,99	cooling
9,802	27,2716	1,7886	356,21	cooling	9,802	39,9323	22,5941	242,36	cooling	9,802	32,7407	13,5503	268,25	cooling
9,804	26,6739	0,0858	371,66	cooling	9,804	39,474	17,2461	310,71		9,804	33,6961	14,8333	263,67	cooling
9,806	27,5636	4,1142	327,79	cooling	9,806	39,7267	14,2603	355,98		9,806	33,3849	12,7544	288,38	cooling
9,808	27,3519	4,8912	313,97	cooling	9,808	39,5679	11,4969	392,39	cooling	9,808	33,7056	14,0824	274,3	cooling
9,81	27,4388	2,9744	341,98	cooling	9,81	39,5039	13,5556	362,72	cooling	9,81	33,0565	11,4355	302,23	cooling
9,812	27,7908	6,1534	302,46	cooling	9,812	39,08	12,1297	376,72	cooling	9,812	34,0644	15,2069	263,6	cooling
9,814	27,3474	8,0713	269,45	cooling	9,814	39,6996	13,5224	365,92	cooling	9,814	32,6129	12,3637	283,05	cooling
9,816	27,6917	9,0598	260,45	cooling	9,816	39,0381	12,1352	376,06	cooling	9,816	33,8652	14,9079	264,99	cooling
9,818	27,4202	8,1015	270,05	cooling	9,818	39,0883	14,5499	343,01	cooling	9,818	32,4921	12,6926	276,77	
9,82	27,3531	0,1283	380,56	cooling	9,82	38,9239	15,2803	330,5	cooling	9,82	33,3582	9,7912	329,43	
9,822	27,5928	-0,1159	387,33	cooling	9,822	39,3452	17,9257	299,41	cooling	9,822	32,5115	11,0759	299,64	
9,824	27,4103	6,5587	291,47	cooling	9,824	38,7104	16,5817	309,33	cooling	9,824	33,1969	10,7484	313,8	
9,826	26,6957	8,2126	258,37	cooling	9,826	39,3468	18,1725	295,99	cooling	9,826	32,9682	12,3132	288,73	
9,828	27,557	9,4931	252,51	cooling	9,828	39,238	19,2941	278,79	cooling	9,828	33,3882	12,2115	296,02	
9,83	27,1468	3,711	327,6	cooling	9,83	39,3946	17,6042	304,6	cooling	9,83	33,4772	14,1918	269,58	
9,832	27,6291	0,6858	376,63	cooling	9,832	39,4846	17,7435	303,91	cooling	9,832	33,9951	13,0595	292,65	
9,834	27,5676	5,5792	307,37	cooling	9,834	39,7129	15,619	336,8	cooling	9,834	34,0793	17,2047	235,88	
9,836	27,1022	3,888	324,5	cooling	9,836	39,5864	15,4966	336,74	cooling	9,836	33,2838	11,8318	299,87	
9,838	27,4334	1,0439	368,89	cooling	9,838	39,4239	15,137	339,49	cooling	9,838	34,4278	15,3037	267,33	
9,84	27,8241	4,637	324,12	cooling	9,84	39,3699	16,3179	322,23	cooling	9,84	32,7927	11,1702	302,25	
9,842	27,7138	2,3941	353,93	cooling	9,842	39,3726	16,1971	323,96	cooling	9,842	33,7302	14,0234	275,47	
9,844	27,6797	-1,607	409,38	cooling	9,844	39,1169	18,3782	289,9	cooling	9,844	32,3761	13,5145	263,66	
9,846	27,3428	-0,7108	392,15	cooling	9,846	38,6182	14,3889	338,69	cooling	9,846	33,4657	17,7903	219,12	
9,848	26,8463	-2,1339	405,1	cooling	9,848	39,2714	15,0849	338,09	cooling	9,848	31,8408	14,3178	244,95	
9,85	27,5447	-0,2197	388,11	cooling	9,85	38,6473	15,9027	317,94	cooling	9,85	32,9397	14,5853	256,57	
9,852	27,2317	-0,2518	384,18	cooling	9,852	39,213	20,2	265,77	cooling	9,852	31,9871	9,2752	317,48	
9,854	27,4486	1,1757	367,26	cooling	9,854	38,9339	21,5735	242,67	cooling	9,854	33,035	6,6799	368,41	
9,856	27,2515	5,4151	305,24	cooling	9,856	39,2161	19,5446	274,98	cooling	9,856	32,5038	7,1042	355,05	
9,858	26,5458	6,1123	285,63	cooling	9,858	38,8258	15,7908	322	cooling	9,858	33,2421	8,5005	345,85	
9,86	26,1792	0,9794	352,26	cooling	9,86	39,4067	17,5251	305,87	cooling	9,86	33,1508	12,8202	284,19	
9,862	26,2516	-2,4097	400,64	cooling	9,862	38,8352	18,5855	283,06	cooling	9,862	32,9153	10,1918	317,64	
9,864	26,6503	-2,421	406,37	cooling	9,864	38,8402	17,9241	292,38	cooling	9,864	33,59	12,2471	298,34	
9,866	26,18	2,1892	335,36	cooling	9,866	38,5731	16,7472	305,09	cooling	9,866	33,2264	8,6399	343,68	
9,868	26,0399	7,8952	253,64	cooling	9,868	38,7504	13,2918	355,87	cooling	9,868	33,7086	13,1258	287,72	
9,87	25,4811	5,2964	282,15	cooling	9,87	38,6694	13,3991	353,24	cooling	9,87	32,8373	9,1108	331,66	
9,872	25,8984	0,9283	349,05	cooling	9,872	38,678	13,4956	352,01	cooling	9,872	33,964	13,7939	281,95	
9,874	25,9239	-0,1545	364,54	cooling	9,874	39,0075	15,8304	323,98	cooling	9,874	32,9223	11,306	302,16	
9,876	26,0968	1,2259	347,66	cooling	9,876	38,753	14,3494	341,13	cooling	9,876	33,6336	14,5982	266,09	
9,878	25,8816	0,3725	356,58	cooling	9,878	38,7717	16,1139	316,72	cooling	9,878	32,8538	11,9991	291,52	
9,88	25,5702	-0,6769	366,9	cooling	9,88	38,5017	15,0819	327,37	cooling	9,88	33,6852	15,7659	250,49	
9,882	25,2886	-2,0807	382,58	cooling	9,882	39,1658	17,4182	304	cooling	9,882	32,5485	12,9813	273,52	
9,884	25,345	0,7114	344,34	cooling	9,884	38,5661	17,7354	291,18	cooling	9,884	33,428	14,9725	257,98	
9,886	25,5559	2,9835	315,53	cooling	9,886	38,9971	19,6627	270,27	cooling	9,886	32,3455	13,592	262,15	

9,888	25,4402	2,4559	321,29	cooling	9,888	38,7385	19,1055	274,44	cooling	9,888	32,7597	8,8539	334,17	
9,89	25,5216	3,0457	314,18	cooling	9,89	38,9083	17,7598	295,62	cooling	9,89	32,9006	9,9289	321,11	
9,892	25,3456	5,3454	279,57	cooling	9,892	38,5432	16,578	307,04	cooling	9,892	33,16	7,0007	365,67	
9,894	25,2014	4,7107	286,43	cooling	9,894	39,0816	18,8938	282,2	cooling	9,894	33,1543	10,8787	311,38	
9,896	25,2221	2,385	319,23	cooling	9,896	38,4503	19,8981	259,33	cooling	9,896	33,2604	13,3096	278,88	
9,898	24,9676	-0,4299	355,02	cooling	9,898	38,2896	18,4091	277,9		9,898	33,0962	16,5354	231,5	
9,9	25,2271	0,6213	343,95	cooling	9,9	38,2301	20,3581	249,82		9,9	32,9018	12,3931	286,68	
9,902	25,1123	8,6694	229,85	cooling	9,902	38,0271	20,1068	250,5		9,902	32,8627	16,7221	225,62	
9,904	24,8296	10,6122	198,74	cooling	9,904	37,8556	22,6393	212,7		9,904	32,1401	10,3553	304,52	
9,906	24,5749	4,269	283,85	cooling	9,906	37,5212	21,4052	225,28		9,906	32,9399	15,9792	237,09	
9,908	25,7118	0,9309	346,4	cooling	9,908	37,988	19,3084	261,11		9,908	31,7178	15,4614	227,24	
9,91	25,0062	0,4711	342,96	cooling	9,91	38,1954	17,7289	286,09		9,91	32,6292	16,5128	225,28	heating
9,912	25,3775	3,9188	299,96	cooling	9,912	38,5151	20,6785	249,33		9,912	32,558	16,9342	218,4	heating
9,914	24,8979	5,6433	269,15	cooling	9,914	38,5111	19,9248	259,81		9,914	32,7246	16,9893	219,96	heating
9,916	24,8308	1,7671	322,4	cooling	9,916	39,2276	22,4601	234,39		9,916	32,4135	13,914	258,6	heating
9,918	24,8186	0,8866	334,53	cooling	9,918	38,2165	17,2138	293,59	heating	9,918	34,1569	16,994	239,91	heating
9,92	25,3915	5,645	276,03	cooling	9,92	38,6306	15,35	325,43	heating	9,92	32,4305	13,1983	268,84	heating
9,922	25,1199	3,7721	298,41	cooling	9,922	38,1494	15,8029	312,37	heating	9,922	33,3032	10,2058	322,87	heating
9,924	25,1987	3,3273	305,73	cooling	9,924	37,7828	14,0086	332,33		9,924	33,0756	13,7406	270,27	heating
9,926	25,1395	0,7618	340,76	cooling	9,926	37,844	16,4291	299,35		9,926	33,2016	9,2876	334,28	heating
9,928	25,2033	-0,8844	364,67	cooling	9,928	37,9875	19,5535	257,68		9,928	32,9667	13,6544	269,96	heating
9,93	26,0079	4,4349	301,56	cooling	9,93	37,5766	18,94	260,51		9,93	33,1354	15,7497	243,03	heating
9,932	24,7142	3,9682	290	cooling	9,932	37,8642	18,0395	277,12	heating	9,932	33,4303	16,8115	232,31	heating
9,934	25,1888	-1,7735	376,89	cooling	9,934	37,9694	17,8178	281,69	heating	9,934	33,6656	15,3557	255,94	heating
9,936	24,7544	-0,1457	348,07	cooling	9,936	38,3773	15,4043	321,13	heating	9,936	33,2837	16,1567	239,41	heating
9,938	24,8015	3,7346	294,48	cooling	9,938	38,4893	19,0344	271,95		9,938	33,3345	9,8304	328,55	heating
9,94	24,2205	4,1124	281,08	cooling	9,94	38,0777	15,6549	313,44	heating	9,94	34,3429	16,3496	251,52	heating
9,942	25,0482	5,5266	272,88	cooling	9,942	38,7244	16,2763	313,79		9,942	32,3952	11,2196	296	heating
9,944	24,3609	-0,4998	347,51	cooling	9,944	38,3635	16,9432	299,42		9,944	33,7673	13,0943	288,98	heating
9,946	24,8417	1,2203	330,19	cooling	9,946	38,177	17,9719	282,44		9,946	33,0613	15,0335	252	heating
9,948	24,5361	5,1627	270,81	cooling	9,948	38,3575	19,5516	262,88		9,948	33,4283	14,1716	269,18	heating
9,95	24,0364	1,8544	310,07	cooling	9,95	38,2202	18,9922	268,78		9,95	32,7772	12,5318	283	heating
9,952	24,9138	2,8908	307,85	cooling	9,952	37,8103	14,1272	331,05	heating	9,952	34,1674	15,3588	262,92	heating
9,954	24,4513	4,435	279,8	cooling	9,954	38,4035	15,7496	316,67		9,954	32,7555	10,7618	307,44	heating
9,956	25,1656	2,8979	311,27	cooling	9,956	37,8351	16,3088	300,9		9,956	33,6935	12,4408	297,08	heating
9,958	24,9734	4,5055	286,11	cooling	9,958	38,1765	17,3603	290,98		9,958	33,1853	16,2874	236,21	heating
9,96	25,0252	1,1168	334,2	cooling	9,96	38,0043	19,4875	258,84		9,96	33,8911	11,5648	312,09	heating
9,962	25,0963	-3,2734	396,57	cooling	9,962	38,142	15,947	310,25		9,962	33,7166	16,2396	244,3	heating
9,964	26,1467	1,8265	339,96	cooling	9,964	38,0596	16,2683	304,61		9,964	33,43	12,7177	289,53	heating
9,966	25,5915	1,1556	341,58	cooling	9,966	37,7867	15,4788	311,83		9,966	34,2837	12,9642	298,01	heating
9,968	26,0053	2,1865	332,95	cooling	9,968	37,9859	15,1168	319,67		9,968	33,6456	11,6971	306,81	heating
9,97	25,3755	1,4833	333,98	cooling	9,97	38,3155	16,4878	305,12		9,97	33,8333	12,2112	302,24	heating
9,972	25,3586	-1,8296	380,05	cooling	9,972	37,8562	18,4444	271,35		9,972	33,9346	10,2956	330,44	heating
9,974	26,0812	1,8013	339,4	cooling	9,974	37,7046	16,6544	294,25		9,974	34,8065	17,2271	245,73	heating
9,976	25,4031	3,6914	303,5	cooling	9,976	38,2596	18,7379	272,88		9,976	33,2015	12,4715	289,78	heating
9,978	25,5087	1,8404	330,85	cooling	9,978	37,6107	14,9657	316,54		9,978	35,1922	17,777	243,44	heating
9,98	25,3992	3,7993	301,93	cooling	9,98	38,2533	14,5532	331,29		9,98	33,7476	15,6984	252,3	heating
9,982	25,1273	1,3021	333,04	cooling	9,982	37,8956	13,5162	340,79		9,982	34,4282	12,524	306,19	heating
9,984	24,9343	-3,2535	394,02	cooling	9,984	38,342	12,2274	365,04		9,984	33,8212	12,7563	294,46	heating
9,986	25,7395	1,2399	342,47	cooling	9,986	37,8177	11,2275	371,69		9,986	34,8282	13,4846	298,35	heating
9,988	25,0785	0,4256	344,61	cooling	9,988	38,4151	14,1065	339,8		9,988	33,6426	11,4076	310,81	heating
9,99	25,7119	3,9644	304	cooling	9,99	37,8985	15,2852	316,1		9,99	34,3983	14,0046	285,07	heating
9,992	25,1412	4,271	291,73	cooling	9,992	38,2397	16,7137	300,9		9,992	33,77	12,8022	293,1	heating
9,994	24,5811	-3,5977	393,9	cooling	9,994	37,9662	17,2783	289,19		9,994	34,4191	10,0667	340,41	heating
9,996	24,913	-4,5039	411,2	cooling	9,996	38,2391	16,703	301,04		9,996	33,7843	12,6969	294,77	heating
9,998	24,3758	0,8173	329,31	cooling	9,998	38,0432	18,5897	271,93		9,998	33,6232	6,23	382,92	heating

10	24,1029	0,8472	325,08	cooling	10	37,6791	17,2674	285,33		10	34,7302	9,8286	348,09	heating
10,002	24,1609	4,3031	277,58	cooling	10,002	38,1078	16,245	305,61		10,002	33,3	10,4277	319,72	
10,004	23,3961	1,5012	306,06	cooling	10,004	38,0468	15,165	319,85		10,004	33,8793	15,1727	261,49	
10,006	23,5044	-1,461	348,98	cooling	10,006	38,2549	18,5719	275,14		10,006	33,4	16,7034	233,39	
10,008	24,0476	5,4474	260	cooling	10,008	37,998	18,9875	265,74		10,008	33,7648	18,9642	206,89	heating
10,01	23,0987	3,4735	274,33	cooling	10,01	38,5447	21,2849	241,27		10,01	32,7978	13,7121	266,79	heating
10,012	23,5526	0,1507	327,12	cooling	10,012	37,9224	16,9978	292,5		10,012	34,9076	18,7709	225,57	heating
10,014	23,471	2,8825	287,8	cooling	10,014	38,4054	15,9946	313,27		10,014	33,4884	14,5789	264,33	heating
10,016	22,9341	1,8689	294,46	cooling	10,016	38,212	15,2955	320,34		10,016	34,1204	13,3249	290,69	heating
10,018	23,2649	2,1121	295,69	cooling	10,018	38,21	16,4959	303,53		10,018	33,5637	13,8154	276,05	heating
10,02	22,942	0,8242	309,17	cooling	10,02	37,8767	18,829	266,26		10,02	34,4101	11,7011	317,44	heating
10,022	22,8079	-4,3319	379,37	cooling	10,022	37,9298	20,0824	249,48		10,022	33,786	12,0175	304,29	heating
10,024	23,2706	2,1603	295,09	cooling	10,024	37,7612	17,1242	288,48		10,024	33,9546	13,1898	290,26	heating
10,026	22,5765	6,5368	224,21	cooling	10,026	37,8115	14,4557	326,48		10,026	34,3421	13,5708	290,35	heating
10,028	22,6	4,7933	248,91	cooling	10,028	37,9846	15,6603	312,06		10,028	34,6847	12,5151	309,9	heating
10,03	22,8666	2,3329	287,03	cooling	10,03	37,931	16,2211	303,47		10,03	34,1561	13,1378	293,8	heating
10,032	22,3497	-5,0979	383,68	cooling	10,032	37,7258	20,0608	246,93		10,032	34,3637	7,5296	375,1	heating
10,034	23,1675	-3,234	369,05	cooling	10,034	37,338	17,8793	272		10,034	35,3506	12,9675	312,88	heating
10,036	23,1505	4,3013	263,48	cooling	10,036	38,1473	16,0634	308,7		10,036	33,5301	10,7617	318,27	heating
10,038	22,7789	0,4475	312,16	cooling	10,038	38,354	13,0604	353,57		10,038	34,4233	16,2854	253,54	
10,04	23,7683	-1,8732	358,43	cooling	10,04	38,3408	15,0028	326,23		10,04	33,7615	16,4372	242,17	heating
10,042	23,9684	1,0377	320,54	cooling	10,042	38,0219	14,7003	326		10,042	33,8885	16,0273	249,67	heating
10,044	24,1216	1,4998	316,22	cooling	10,044	38,1659	15,7461	313,4		10,044	32,8978	11,4376	299,98	heating
10,046	24,1582	5,1861	265,2	cooling	10,046	37,5959	12,7374	347,49		10,046	34,3841	14,7	275,15	heating
10,048	23,585	1,7659	305	cooling	10,048	37,8517	11,622	366,65		10,048	33,6378	10,7642	319,74	heating
10,05	23,4498	-2,9875	369,55	cooling	10,05	37,7985	11,095	373,28		10,05	34,2639	12,5053	304,15	heating
10,052	23,826	3,6029	282,69	cooling	10,052	37,9603	12,8829	350,55		10,052	33,8804	12,7273	295,69	heating
10,054	22,7945	6,0685	233,8	cooling	10,054	37,7091	15,6418	308,47	cooling	10,054	35,0234	11,1461	333,77	heating
10,056	23,1398	2,3648	290,4	cooling	10,056	38,1114	17,7386	284,78	cooling	10,056	34,4676	12,0821	312,92	heating
10,058	23,3221	0,5116	318,86	cooling	10,058	38,0223	18,5364	272,38		10,058	34,1997	8,7968	355,09	
10,06	23,2268	-2,3708	357,82	cooling	10,06	37,9924	16,9593	294,01		10,06	34,962	9,377	357,64	
10,062	23,7269	1,4722	311,09	cooling	10,062	38,0312	17,5364	286,49		10,062	34,7592	9,5265	352,72	
10,064	23,5084	5,3269	254,15	cooling	10,064	37,9577	16,0988	305,56		10,064	34,3164	12,6895	302,31	
10,066	23,4455	1,4041	308,11	cooling	10,066	37,6309	16,783	291,42		10,066	34,6679	13,7486	292,42	
10,068	23,4452	-0,119	329,39	cooling	10,068	37,4019	15,1448	311,12		10,068	35,2164	16,5282	261,23	
10,07	23,1488	0,4351	317,5	cooling	10,07	37,694	15,2862	313,23		10,07	34,0856	8,4475	358,38	
10,072	22,7336	-0,9924	331,65	cooling	10,072	37,6831	14,8379	319,34		10,072	35,2374	10,6206	344,11	
10,074	22,927	0,7623	309,83	cooling	10,074	37,7125	18,1157	273,93		10,074	34,7544	9,746	349,58	
10,076	22,4105	-0,5955	321,59	cooling	10,076	37,3924	16,5542	291,29		10,076	34,865	11,4913	326,73	
10,078	22,9132	-0,9367	333,39	cooling	10,078	37,9111	18,2175	275,29		10,078	34,3754	13,5541	291,05	
10,08	23,0105	1,7534	297,14	cooling	10,08	37,4234	17,9687	271,95		10,08	35,1255	14,4129	289,53	
10,082	22,7315	1,9072	291,09	cooling	10,082	37,7534	18,806	264,86		10,082	34,6249	11,2365	326,94	
10,084	22,9068	1,1669	303,89	cooling	10,084	37,7382	19,8255	250,39		10,084	34,5823	11,6575	320,45	
10,086	22,8626	0,3184	315,13	cooling	10,086	37,6629	17,7188	278,79		10,086	34,4003	9,0396	354,5	
10,088	22,9124	-2,2881	352,26	cooling	10,088	37,4207	14,5459	319,76		10,088	35,4929	8,065	383,4	
10,09	22,5087	-3,4965	363,51	cooling	10,09	37,9698	15,7158	311,08		10,09	34,8605	14,429	285,6	cooling
10,092	22,3634	-1,3636	331,67	cooling	10,092	37,3672	17,2366	281,4		10,092	34,6076	13,5099	294,92	
10,094	21,815	-1,6213	327,6	cooling	10,094	37,3822	17,5716	276,92		10,094	35,4516	15,6345	277,01	
10,096	21,7359	-2,8516	343,7	cooling	10,096	37,3846	19,3022	252,77		10,096	34,8623	12,0563	318,79	
10,098	21,6645	-2,7328	341,04	cooling	10,098	37,2669	15,2098	308,33		10,098	34,4979	10,604	334	
10,1	21,6965	-0,3366	307,99	cooling	10,1	37,246	15,2999	306,77		10,1	34,9247	10,1519	346,29	
10,102	21,3845	-0,8216	310,41	cooling	10,102	37,1966	15,2722	306,47		10,102	34,7725	12,4455	312,1	cooling
10,104	21,318	-0,4537	304,34	cooling	10,104	37,8406	17,7254	281,18		10,104	33,8871	8,3846	356,49	cooling
10,106	21,5012	-1,9924	328,41	cooling	10,106	37,8635	20,3691	244,55		10,106	34,6633	14,2198	285,77	cooling
10,108	22,379	-2,9117	353,53	cooling	10,108	37,7404	22,5416	212,46		10,108	34,117	12,4497	302,88	cooling
10,11	23,1844	-3,1433	368,02	cooling	10,11	37,6913	19,4253	255,33		10,11	34,225	13,7234	286,58	cooling

10,112	23,7217	0,6261	322,84	cooling	10,112	38,4526	19,3554	266,95	heating	10,112	33,9581	16,1685	248,67	cooling
10,114	24,2299	4,8677	270,66	cooling	10,114	37,8841	17,3216	287,43	heating	10,114	34,9104	13,2424	302,89	
10,116	24,0334	3,9718	280,43	cooling	10,116	38,7596	20,2142	259,24	heating	10,116	34,7692	11,5782	324,18	
10,118	24,1009	0,8346	325,23	cooling	10,118	38,3191	21,8929	229,61	heating	10,118	34,7597	11,9891	318,3	
10,12	24,2282	1,2808	320,77	cooling	10,12	38,485	20,6071	249,91	heating	10,12	34,8731	11,1804	331,19	
10,122	24,2936	0,9393	326,46	cooling	10,122	38,118	19,8259	255,7	heating	10,122	36,1271	14,7336	299,05	cooling
10,124	24,5283	1,3445	324,08	cooling	10,124	38,4938	19,6776	263,02	heating	10,124	34,6439	15,8193	263,14	
10,126	24,4269	1,9331	314,43	cooling	10,126	37,9251	18,3816	273,19	heating	10,126	34,9542	9,5516	355,09	
10,128	24,2203	-0,5758	346,61	cooling	10,128	37,6962	18,0065	275,23	heating	10,128	35,5226	11,1211	341,1	
10,13	24,8982	4,0806	291	cooling	10,13	37,3555	17,1178	282,89	heating	10,13	34,3732	6,2755	392,76	cooling
10,132	24,6221	8,3699	227,18	cooling	10,132	37,4938	14,0567	327,62	heating	10,132	34,4264	8,771	358,62	cooling
10,134	24,5021	4,8309	274,97	cooling	10,134	37,5126	16,0749	299,67	heating	10,134	34,6781	14,9459	275,83	cooling
10,136	24,6609	0,2817	340,79	cooling	10,136	37,2872	16,1086	296,05	heating	10,136	34,3675	17,3222	238,27	cooling
10,138	24,3334	-2,6815	377,63	cooling	10,138	38,0654	17,3188	290,01	heating	10,138	33,8519	15,7665	252,81	cooling
10,14	24,4577	0,7051	332,03	cooling	10,14	37,8084	15,9833	305,08	heating	10,14	34,2737	17,051	240,75	
10,142	24,3141	7,9054	229,37	cooling	10,142	38,4107	17,4045	293,64	heating	10,142	34,02	9,6184	341,1	
10,144	23,8908	6,1356	248,19	cooling	10,144	38,459	17,4811	293,24	heating	10,144	34,4352	9,6656	346,24	
10,146	24,1379	3,8662	283,37	cooling	10,146	39,2654	20,5164	262,08	heating	10,146	33,7151	9,8768	333,22	
10,148	23,8811	2,8523	293,95	cooling	10,148	38,44	20,267	254,03	heating	10,148	34,9983	10,389	344	cooling
10,15	23,7018	2,0103	303,22	cooling	10,15	38,2973	20,9367	242,68	heating	10,15	34,6361	14,4783	281,78	cooling
10,152	23,9715	5,0442	264,58	cooling	10,152	37,5102	19,4444	252,53	heating	10,152	33,8308	11,7282	308,96	cooling
10,154	23,2705	1,1294	309,5		10,154	37,3943	17,86	273,06	heating	10,154	34,6199	8,084	370,93	cooling
10,156	23,5992	0,1585	327,67		10,156	37,0716	16,4824	287,81	heating	10,156	35,3769	8,3151	378,28	cooling
10,158	23,7584	5,6911	252,55		10,158	37,598	17,8312	276,31	heating	10,158	33,9575	6,4112	385,06	cooling
10,16	23,2159	3,8688	270,44		10,16	37,3958	19,4113	251,4	heating	10,16	34,4385	6,8921	385,06	cooling
10,162	23,4138	0,133	325,43		10,162	37,3843	18,7041	261,12	heating	10,162	34,9374	13,4294	300,65	cooling
10,164	23,1821	-1,2341	341,3		10,164	37,868	19,8434	251,96	heating	10,164	34,0633	10,6911	326,71	cooling
10,166	23,0355	-4,2974	382,07		10,166	38,039	18,4727	273,51	heating	10,166	34,0664	13,8826	282,14	cooling
10,168	23,2598	2,1473	295,12	cooling	10,168	38,4615	21,473	237,47	heating	10,168	34,3761	15,6191	262,19	cooling
10,17	22,6027	5,2237	242,93	cooling	10,17	38,3253	21,1384	240,25	heating	10,17	34,3053	13,6439	288,82	cooling
10,172	22,36	1,049	297,9		10,172	38,9081	20,4392	258,17	heating	10,172	33,7989	13,2631	287,06	cooling
10,174	22,8784	1,0248	305,48		10,174	38,6045	17,3186	297,55	heating	10,174	34,1988	15,8856	255,99	cooling
10,176	22,0013	-1,5553	329,29		10,176	38,7221	17,8361	291,95	heating	10,176	33,997	10,6966	325,71	cooling
10,178	21,7784	-2,7694	343,14		10,178	38,5509	18,8112	275,93	heating	10,178	33,9344	7,2645	372,81	cooling
10,18	21,458	-0,4503	306,25		10,18	38,6221	22,5199	225,08	heating	10,18	33,4689	5,3316	393,32	cooling
10,182	20,7761	-4,3912	351,8		10,182	37,8558	20,3804	244,28	heating	10,182	34,7242	6,954	388,19	cooling
10,184	20,8639	-4,465	354,06	cooling	10,184	38,1591	17,0749	294,73	heating	10,184	34,2995	12,7947	300,6	cooling
10,186	20,6943	-0,4643	295,77	cooling	10,186	37,9221	14,4413	328,23	heating	10,186	33,7895	15,0636	261,76	cooling
10,188	20,6232	-2,6516	325,35	cooling	10,188	37,7849	12,5738	352,41	heating	10,188	34,3245	14,6589	274,9	cooling
10,19	20,9956	-0,7304	303,7	cooling	10,19	37,9287	16,1227	304,82	heating	10,19	35,1973	12,2585	320,65	cooling
10,192	20,6401	-1,1576	304,7	cooling	10,192	37,7585	17,2662	286,45	heating	10,192	33,8973	9,3808	342,7	cooling
10,194	20,6197	-3,8841	342,53	cooling	10,194	37,4778	18,3848	266,89	heating	10,194	34,0364	8,8211	352,47	cooling
10,196	20,9818	0,3773	288,02	cooling	10,196	37,242	15,3394	306,17	heating	10,196	34,4313	12,6934	303,86	cooling
10,198	20,0669	0,1338	278,63	cooling	10,198	37,9877	13,9836	335,54	heating	10,198	33,8273	12,6955	295,39	cooling
10,2	20,5314	-3,1434	330,94	cooling	10,2	38,41	13,0065	355,1	heating	10,2	33,5412	15,6171	250,55	cooling
10,202	21,0622	0,9106	281,69	cooling	10,202	38,7942	17,8382	292,93	heating	10,202	33,5358	14,8155	261,68	cooling
10,204	20,7237	1,7759	264,86	cooling	10,204	38,862	18,5553	283,86	heating	10,204	33,7854	13,0821	289,4	cooling
10,206	20,7838	1,9552	263,2		10,206	39,6983	20,4187	269,5	heating	10,206	33,0291	11,8517	296,03	cooling
10,208	20,7923	3,3216	244,21		10,208	39,0499	18,6138	285,67	heating	10,208	33,463	12,1385	298,08	cooling
10,21	20,4909	-3,0801	329,49		10,21	39,4649	20,5132	264,92	heating	10,21	33,7491	12,741	293,66	cooling
10,212	20,9951	-3,9859	349,2		10,212	39,1291	21,6645	244,13	heating	10,212	33,6064	14,6281	265,29	cooling
10,214	21,128	0,3894	289,89		10,214	38,987	19,6298	270,58	heating	10,214	33,2818	13,5007	276,51	
10,216	20,7249	-0,5037	296,74		10,216	38,3826	15,432	320,82	heating	10,216	34,4207	14,1066	283,96	
10,218	21,276	3,1336	253,6		10,218	38,7256	11,9088	374,86	heating	10,218	33,7708	13,3103	286,01	
10,22	20,7704	4,4702	227,85		10,22	38,4755	12,4515	363,78	heating	10,22	33,1437	9,8896	325,06	
10,222	20,8601	-1,2725	309,38		10,222	38,4057	14,8173	329,73	heating	10,222	34,0103	12,5117	300,52	

10,224	21,0247	-2,8718	334,04		10,224	38,3473	17,5645	290,51		10,224	33,7749	11,0613	317,5	
10,226	20,5723	-4,1537	345,63		10,226	38,7427	15,6461	322,86		10,226	33,4346	9,0064	341,47	
10,228	20,5211	-5,3229	361,26		10,228	38,4583	14,8359	330,21		10,228	33,4295	11,4883	306,71	
10,23	21,0262	0,1345	292,03		10,23	38,2418	11,4041	375,15		10,23	33,5196	12,6209	292,13	
10,232	20,2972	-4,296	343,78		10,232	38,9268	14,42	342,57		10,232	32,9962	10,0642	320,56	
10,234	20,8484	-7,4498	395,57		10,234	38,7702	16,6142	309,71		10,234	33,3705	13,654	275,61	
10,236	21,1548	-1,6746	319,12	cooling	10,236	38,587	18,7771	276,91		10,236	32,8895	10,37	314,79	
10,238	20,3589	-2,495	319,46	cooling	10,238	38,3256	15,9932	312,17		10,238	33,5082	9,2333	339,33	
10,24	21,005	-1,2258	310,75	cooling	10,24	39,0527	16,1144	320,64		10,24	33,0554	11,7309	298,08	
10,242	20,506	-0,4483	292,91	cooling	10,242	38,5376	15,5119	321,86		10,242	33,5238	11,8737	302,64	
10,244	20,4624	-4,5756	349,99	cooling	10,244	38,8578	17,9262	292,59		10,244	33,8017	13,4895	283,94	
10,246	20,695	-1,1936	305,97	cooling	10,246	38,4645	16,9995	300,05		10,246	33,5437	15,3723	254,01	
10,248	20,4999	2,4231	252,69	cooling	10,248	38,9928	14,6196	340,7		10,248	33,3904	11,2805	309,06	
10,25	20,7717	-1,0609	305,19	cooling	10,25	38,2827	10,502	388,33		10,25	34,4385	11,4718	321,04	
10,252	21,8162	2,4016	271,39	cooling	10,252	39,1203	11,0045	393,02		10,252	33,656	11,4478	310,44	
10,254	21,4458	1,4148	280		10,254	38,6355	14,4549	338,01	cooling	10,254	33,1961	8,7404	341,85	
10,256	21,7945	0,5776	296,58		10,256	38,5488	15,3607	324,14	cooling	10,256	34,3903	13,4709	292,42	
10,258	22,268	2,9875	269,51		10,258	38,4199	17,1103	297,88	cooling	10,258	33,5546	10,6826	319,72	
10,26	22,2219	1,1594	294,42		10,26	38,5728	15,8428	317,73	cooling	10,26	33,4836	8,4713	349,63	
10,262	23,1296	1,5512	301,63		10,262	38,535	15,2761	325,13	cooling	10,262	33,0972	7,5876	356,59	
10,264	22,9723	4,3146	260,81		10,264	38,6421	15,5255	323,14	cooling	10,264	33,3777	6,4712	376,11	cooling
10,266	23,1416	3,4248	275,61		10,266	39,2481	18,5509	289,32	cooling	10,266	33,1621	8,8425	339,95	cooling
10,268	23,304	5,179	253,36		10,268	38,7947	16,3935	313,14	cooling	10,268	32,9272	15,5214	243,31	cooling
10,27	23,2998	6,0653	240,91		10,27	39,0576	18,6605	285,12	cooling	10,27	32,802	12,5082	283,68	cooling
10,272	23,2341	1,8509	298,9		10,272	38,384	18,1831	282,38	cooling	10,272	33,1381	13,5985	273,13	cooling
10,274	24,008	2,0256	307,28		10,274	39,1109	20,5984	258,78		10,274	32,2853	12,4843	276,79	
10,276	23,5798	-0,5999	338		10,276	38,4348	19,69	262,02		10,276	32,776	12,0359	289,92	
10,278	23,5424	-0,7542	339,63		10,278	38,9925	17,3155	303,01		10,278	33,2395	16,8632	228,92	
10,28	23,4826	2,4732	293,68		10,28	38,7352	12,7061	363,85		10,28	32,1594	15,5882	231,64	
10,282	23,0701	2,6044	286,08		10,282	38,8394	11,8399	377,41		10,282	33,1308	15,2994	249,26	
10,284	23,367	2,4327	292,63		10,284	38,583	14,7061	333,76	cooling	10,284	33,7877	15,7807	251,71	
10,286	23,2525	2,7634	286,41		10,286	38,8297	17,8672	293,02	cooling	10,286	33,4484	12,9733	286,21	
10,288	23,2228	-1,2846	342,58		10,288	38,5819	19,5667	265,8	cooling	10,288	33,3589	10,4951	319,6	
10,29	23,4885	0,5403	320,78		10,29	38,2378	14,9136	326,04		10,29	34,1829	11,723	313,96	
10,292	23,4672	3,4209	280,22		10,292	39,2683	16,127	323,48	cooling	10,292	34,1994	8,1752	363,78	
10,294	23,5242	2,8169	289,46		10,294	39,008	15,0979	334,23	cooling	10,294	33,903	13,2162	289,17	
10,296	24,2184	4,3741	277,39		10,296	38,9756	16,835	309,49	cooling	10,296	33,1063	13,5333	273,6	
10,298	23,9806	1,7131	311,27		10,298	38,7296	17,5565	295,97	cooling	10,298	33,6014	15,3079	255,72	
10,3	24,5011	0,0397	341,93		10,3	39,3987	20,5319	263,73	cooling	10,3	33,0512	17,6392	215,44	
10,302	24,8707	4,4942	284,83		10,302	38,7246	16,2949	313,53		10,302	32,2989	16,7043	217,99	
10,304	24,5043	3,9306	287,59		10,304	38,8032	16,3367	314,05		10,304	32,9886	15,2257	248,3	heating
10,306	24,72	1,8105	320,24		10,306	38,3862	15,1928	324,21		10,306	32,7985	13,7653	266,06	heating
10,308	24,958	2,0708	319,93		10,308	38,6195	16,9928	302,31	cooling	10,308	32,4479	9,2438	324,36	heating
10,31	24,4669	-3,6334	392,8		10,31	37,8452	17,8052	280,13	cooling	10,31	33,2871	11,9806	297,83	heating
10,312	25,0402	-2,8173	389,41		10,312	37,9106	17,4264	286,34		10,312	33,1506	15,3328	249,07	heating
10,314	24,6449	2,3902	311,09		10,314	37,6726	15,8316	305,31		10,314	32,848	13,8491	265,58	heating
10,316	24,2404	1,89	312,42	cooling	10,316	37,556	14,614	320,7		10,316	33,4211	17,9588	216,14	heating
10,318	24,4368	1,2254	324,46	cooling	10,318	37,2887	14,9278	312,57		10,318	33,559	15,3517	254,51	heating
10,32	24,216	0,7159	328,5	cooling	10,32	37,7778	13,6267	337,6		10,32	33,4103	12,5515	291,58	heating
10,322	24,1302	-1,7559	361,85	cooling	10,322	37,9374	16,1975	303,89		10,322	33,2074	13,082	281,32	heating
10,324	24,4906	3,6518	291,3	cooling	10,324	38,0126	17,9	281,14		10,324	33,0531	10,5639	314,37	heating
10,326	23,9102	2,5542	298,53	cooling	10,326	38,6249	22,0963	231,05		10,326	33,893	10,8434	322,2	heating
10,328	23,9234	-1,9087	361,09	cooling	10,328	38,6376	19,9948	260,6		10,328	33,1183	13,6419	272,25	heating
10,33	24,4698	-0,2523	345,58	cooling	10,33	38,3853	17,8307	287,32		10,33	33,0354	13,5025	273,04	heating
10,332	23,8344	-1,2968	351,3	cooling	10,332	38,0202	13,413	343,97		10,332	33,5418	13,9975	273,2	heating
10,334	24,2907	0,8364	327,86	cooling	10,334	38,7859	16,7607	307,88		10,334	33,0407	11,6717	298,71	heating

10,336	24,0227	5,1029	264,47	cooling	10,336	38,069	19,1418	264,57		10,336	32,8755	9,5718	325,75	heating
10,338	23,8904	3,4119	286,26	cooling	10,338	38,4052	21,981	229,59		10,338	33,3981	12,5294	291,71	heating
10,34	24,3336	2,964	298,72	cooling	10,34	38,1879	22,1079	224,78		10,34	33,1975	10,9344	311,21	heating
10,342	24,2773	3,4098	291,7	cooling	10,342	38,4564	19,0505	271,27		10,342	32,9261	10,6761	311,02	heating
10,344	24,3041	-0,4824	346,48	cooling	10,344	38,1188	17,1036	293,76		10,344	33,8403	13,2802	287,4	heating
10,346	24,7962	2,2884	314,63	cooling	10,346	38,7555	16,8653	305,99		10,346	33,6012	12,3259	297,4	heating
10,348	24,3756	1,4912	319,89	cooling	10,348	38,298	17,1078	296,21		10,348	34,2241	12,4963	303,72	heating
10,35	24,3548	1,49	319,62	cooling	10,35	38,5552	18,9897	273,5		10,35	33,6591	12,5059	295,69	heating
10,352	24,823	6,6041	254,67	cooling	10,352	38,1525	20,3411	248,98		10,352	34,3165	10,9273	326,95	heating
10,354	24,1545	4,4421	275,55	cooling	10,354	38,4302	16,7795	302,64		10,354	33,9101	11,9317	307,23	
10,356	24,6793	2,4171	311,19	cooling	10,356	38,1365	18,5416	273,91		10,356	33,1913	12,8902	283,78	
10,358	24,3321	2,4828	305,42	cooling	10,358	37,6237	16,0988	300,89		10,358	33,4605	11,7989	302,8	
10,36	24,2973	-1,0459	354,26	cooling	10,36	38,536	19,4371	266,97		10,36	34,2246	12,952	297,36	
10,362	24,5931	-0,0323	344,23	cooling	10,362	38,2255	19,6244	260,02		10,362	33,6344	11,574	308,37	
10,364	24,1897	1,1981	321,39	cooling	10,364	38,0114	17,7426	283,33	heating	10,364	33,5215	11,0449	314,19	
10,366	24,3844	-1,8604	366,86	cooling	10,366	38,084	17,0915	293,45	heating	10,366	34,2223	14,9438	269,48	
10,368	24,6983	-0,2196	348,32	cooling	10,368	38,6759	21,0719	246,08	heating	10,368	33,1168	11,9156	296,36	
10,37	24,3282	-0,2203	343,15	cooling	10,37	37,958	20,9557	237,67	heating	10,37	33,3443	10,3198	321,85	
10,372	24,0936	-0,229	339,99	cooling	10,372	38,6522	25,8918	178,37	heating	10,372	33,1855	13,1972	279,41	
10,374	24,7008	4,7457	278,94	cooling	10,374	37,8506	23,4671	201,06	heating	10,374	32,7137	13,8461	263,74	
10,376	24,1045	4,8125	269,67	cooling	10,376	38,5614	19,9314	260,42	heating	10,376	32,1548	15,0642	238,9	
10,378	24,2866	2,6814	302,01	cooling	10,378	38,211	19,7961	257,41	heating	10,378	32,4914	18,3564	197,59	heating
10,38	24,1868	1,3212	319,63	cooling	10,38	38,6199	16,8401	304,45	heating	10,38	32,2051	14,3971	248,93	heating
10,382	24,0267	-1,9066	362,51	cooling	10,382	38,7588	18,4916	283,31	heating	10,382	32,7189	12,6321	280,78	heating
10,384	23,9107	-0,3777	339,52	cooling	10,384	38,4663	19,4068	266,42	heating	10,384	33,3333	13,2908	280,16	heating
10,386	23,9125	4,1907	275,68	cooling	10,386	38,5609	18,1623	285,14	heating	10,386	34,1823	11,7022	314,24	heating
10,388	23,9015	4,9565	264,82	cooling	10,388	39,0068	16,6236	312,88	heating	10,388	34,8672	15,6782	268,23	heating
10,39	24,2637	7,2888	237,28	cooling	10,39	38,7224	16,6204	308,95	heating	10,39	34,0626	18,989	210,71	heating
10,392	23,8829	8,1773	219,54	cooling	10,392	38,5919	14,5658	335,85	heating	10,392	34,315	17,0926	240,74	heating
10,394	23,4782	1,4932	307,32		10,394	39,4358	18,3635	294,56	heating	10,394	34,0126	15,6689	256,42	heating
10,396	23,9514	-0,2271	337,98		10,396	38,552	14,5995	334,82	heating	10,396	33,1579	12,2656	292,04	heating
10,398	23,3901	0,957	313,58		10,398	39,2584	15,9614	325,66	heating	10,398	32,7617	8,2834	342,17	heating
10,4	23,0321	2,9262	281,05		10,4	38,7391	16,6199	309,19	heating	10,4	33,3272	10,84	314,34	heating
10,402	23,1944	9,2876	194,4		10,402	39,404	17,4884	306,35	heating	10,402	32,7905	11,2554	301,03	heating
10,404	22,8566	7,7509	211,15		10,404	38,9202	19,2559	274,88	heating	10,404	33,2123	11,7359	300,21	heating
10,406	23,0255	3,0415	279,35		10,406	39,2485	18,3255	292,47	heating	10,406	33,8771	16,1861	247,29	heating
10,408	23,7379	4,6786	266,42		10,408	38,9779	14,771	338,38	heating	10,408	33,3804	13,6326	276,04	heating
10,41	23,0601	0,7286	312,16		10,41	39,334	13,8669	355,99	heating	10,41	34,1034	14,8465	269,18	heating
10,412	23,9812	1,7036	311,41		10,412	38,9628	14,294	344,83		10,412	34,002	16,2939	247,53	heating
10,414	23,917	5,9028	251,81		10,414	39,4114	15,36	336,2		10,414	34,1435	14,1322	279,73	heating
10,416	23,5074	1,1323	312,77		10,416	39,0245	19,5157	272,7		10,416	33,7419	14,038	275,43	heating
10,418	24,3073	2,9177	298,99		10,418	38,5103	17,4991	293,71		10,418	34,1649	16,1879	251,29	heating
10,42	24,0491	5,1281	264,49		10,42	39,2158	19,9251	269,66		10,42	34,0976	11,2701	319,09	heating
10,422	24,0942	-1,5083	357,88		10,422	38,6314	16,2392	313,01		10,422	34,6763	14,6706	279,65	heating
10,424	24,7496	0,9265	333,01		10,424	38,7537	17,6665	294,77		10,424	33,6042	14,335	269,35	heating
10,426	24,4672	0,0629	341,14		10,426	38,1412	17,308	291,22		10,426	34,5111	13,1929	298	heating
10,428	24,3766	-3,6514	391,79		10,428	38,7733	19,3915	270,93		10,428	34,7053	14,9028	276,81	heating
10,43	25,0719	1,4104	330,75		10,43	38,1225	17,6619	286,01		10,43	34,037	12,2308	304,82	heating
10,432	24,0107	-0,6233	344,35		10,432	38,6328	18,8647	276,33		10,432	34,4482	10,9073	329,07	heating
10,434	24,8595	-0,6231	356,21		10,434	38,5778	19,6455	264,64		10,434	34,6776	13,8484	291,16	heating
10,436	24,6261	4,3987	282,75	cooling	10,436	39,4173	22,7279	233,29		10,436	34,2328	13,3419	292,02	heating
10,438	24,2027	1,9287	311,36		10,438	38,7771	19,6558	267,29	heating	10,438	34,3692	12,9456	299,47	heating
10,44	24,5956	0,3374	339,09		10,44	39,8099	16,1878	330,2	heating	10,44	34,6273	13,6067	293,84	heating
10,442	24,4892	2,5772	306,3	cooling	10,442	39,4216	15,9577	327,99	heating	10,442	34,1044	7,7758	368,04	heating
10,444	24,5275	1,7481	318,42	cooling	10,444	39,5441	17,8771	302,87		10,444	34,7659	11,8891	319,78	heating
10,446	24,7273	6,5178	254,54	cooling	10,446	39,2437	23,409	221,35		10,446	34,272	11,9209	312,44	heating

10,448	24,6399	7,0848	245,39		10,448	39,2285	22,3255	236,28	heating	10,448	34,5093	11,6744	319,2	
10,45	24,5421	1,9694	315,53		10,45	38,6846	19,843	263,38	heating	10,45	34,2836	13,8308	285,9	
10,452	25,3505	2,5324	318,96		10,452	38,5873	15,1753	327,26	heating	10,452	34,3821	13,127	297,11	
10,454	24,722	0,075	344,53		10,454	39,3344	16,1621	323,91	heating	10,454	35,1164	9,815	353,68	
10,456	25,0514	0,3421	345,4		10,456	39,3263	14,6705	344,65	heating	10,456	35,2344	13,3415	306,03	
10,458	24,961	2,6356	312,08	cooling	10,458	39,4528	16,4865	321,03		10,458	34,4836	12,0395	313,73	
10,46	24,5558	2,9603	301,87	cooling	10,46	39,2411	15,0312	338,42		10,46	35,2718	13,9204	298,46	
10,462	24,5716	0,6398	334,53	cooling	10,462	40,0652	15,298	346,21		10,462	35,2624	17,5084	248,17	
10,464	24,5244	1,5539	321,09	cooling	10,464	39,1621	11,5786	385,58		10,464	34,7255	13,9402	290,55	
10,466	24,6464	0,0116	344,36	cooling	10,466	39,961	14,4647	356,4		10,466	34,8732	13,3578	300,75	
10,468	24,395	-0,5397	348,55	cooling	10,468	39,1722	15,2127	334,92		10,468	34,4362	10,6541	332,44	
10,47	24,8603	0,3791	342,21	cooling	10,47	39,2979	14,938	340,52		10,47	34,4858	9,9395	343,12	
10,472	24,7045	0,2732	341,51	cooling	10,472	38,4191	13,8818	343		10,472	34,5348	10,6387	334,03	
10,474	25,155	1,6412	328,69	cooling	10,474	38,7528	13,0099	359,85		10,474	34,3714	13,7318	288,51	
10,476	24,907	3,3357	301,54	cooling	10,476	38,3031	16,2411	308,39	cooling	10,476	34,1965	11,6885	314,63	
10,478	25,0664	4,978	280,81	cooling	10,478	37,8581	17,7845	280,6		10,478	34,9111	15,2854	274,34	
10,48	25,0871	4,4583	288,36	cooling	10,48	38,0105	17,9628	280,24		10,48	34,8092	12,4063	313,16	
10,482	24,8913	3,1901	303,35	cooling	10,482	38,2835	14,0082	339,33		10,482	35,1526	12,606	315,17	
10,484	24,6991	0,1108	343,71	cooling	10,484	38,2326	13,288	348,69		10,484	34,6369	11,2603	326,77	
10,486	24,6625	0,6556	335,58	cooling	10,486	38,1798	13,472	345,38	cooling	10,486	35,2847	12,1726	323,07	
10,488	24,6564	3,1296	300,91	cooling	10,488	39,0828	19,63	271,92	cooling	10,488	34,9991	12,4293	315,49	
10,49	24,0026	1,7242	311,42	cooling	10,49	38,5582	16,7214	305,25	cooling	10,49	35,1116	11,888	324,63	
10,492	24,2709	1,1038	323,84	cooling	10,492	39,1423	16,8454	311,68	cooling	10,492	34,5736	11,9532	316,2	
10,494	24,366	3,8271	287,1	cooling	10,494	38,7032	13,3142	354,9	cooling	10,494	34,8519	13,8049	294,21	
10,496	24,5616	6,4283	253,48	cooling	10,496	39,4967	14,4804	349,69	cooling	10,496	34,4206	15,0406	270,9	
10,498	24,6005	7,8926	233,55	cooling	10,498	38,6034	15,6637	320,66	cooling	10,498	34,8322	14,1328	289,35	
10,5	24,5973	3,9381	288,78		10,5	38,899	17,4062	300,44	cooling	10,5	34,3684	15,6088	262,23	
10,502	24,7439	-1,7143	369,85		10,502	38,4346	16,5648	305,71	cooling	10,502	34,2228	11,4576	318,22	
10,504	25,1308	-1,6459	374,3	cooling	10,504	38,4865	15,1331	326,45	cooling	10,504	34,8143	13,6119	296,38	
10,506	25,3079	2,058	325	cooling	10,506	38,0271	14,4199	329,99	cooling	10,506	34,6267	12,1927	313,59	
10,508	25,5437	3,8976	302,58	cooling	10,508	38,747	16,5601	310,14	cooling	10,508	34,6536	14,4259	282,75	
10,51	25,4776	5,9568	272,87	cooling	10,51	38,3167	20,4161	250,22	cooling	10,51	34,3278	14,4285	278,16	
10,512	25,3781	4,9813	285,12	cooling	10,512	38,2052	17,5481	288,76	cooling	10,512	35,0876	15,4448	274,58	
10,514	25,1703	2,793	312,8	cooling	10,514	38,7262	17,5752	295,66	cooling	10,514	34,5605	10,9546	329,97	
10,516	24,9056	0,4189	342,29	cooling	10,516	38,1997	13,0248	351,91	cooling	10,516	35,055	13,3606	303,26	
10,518	24,2684	-3,0835	382,34	cooling	10,518	38,3053	15,9213	312,89	cooling	10,518	34,2515	12,6071	302,56	
10,52	24,2449	-2,3676	372	cooling	10,52	37,8577	17,5957	283,23	cooling	10,52	35,3258	15,0387	283,58	
10,522	23,4927	2,6071	291,95	cooling	10,522	38,5009	17,6327	291,71	cooling	10,522	34,5486	16,1341	257,41	
10,524	23,0669	4,9829	252,79	cooling	10,524	38,2211	13,7379	342,24	cooling	10,524	35,1042	15,174	278,59	
10,526	23,0268	4,2083	263,06	cooling	10,526	38,9016	15,0742	333,07	cooling	10,526	34,4105	12,4912	306,4	
10,528	23,0337	4,5572	258,27	cooling	10,528	38,6339	16,4869	309,58	cooling	10,528	34,6224	7,7717	375,33	
10,53	22,909	3,0381	277,77		10,53	39,0713	20,6335	257,73	cooling	10,53	34,4812	7,6575	374,96	
10,532	23,146	4,549	259,96		10,532	38,0163	19,712	255,87	cooling	10,532	35,0624	10,8404	338,59	
10,534	22,8635	4,929	250,7		10,534	38,2858	17,3408	292,78		10,534	34,3744	14,6258	276,06	
10,536	22,3701	2,1617	282,48		10,536	37,2731	16,9927	283,49		10,536	34,1202	12,33	304,59	
10,538	22,5096	2,9888	272,87		10,538	36,5238	16,3278	282,31		10,538	34,7864	13,7531	294,01	
10,54	22,1176	3,5568	259,45		10,54	36,4206	19,3781	238,23		10,54	34,3657	6,715	386,52	
10,542	21,7707	2,8795	264,07		10,542	36,5666	18,4941	252,63		10,542	34,7203	8,3363	368,81	
10,544	21,858	6,3804	216,35		10,544	36,5612	17,7567	262,86		10,544	33,9112	8,3721	357	cooling
10,546	21,3467	4,3143	238,09		10,546	36,8868	13,7741	323,08		10,546	35,082	12,434	316,58	cooling
10,548	22,0869	1,5271	287,4		10,548	38,1433	15,4432	317,31		10,548	33,9796	11,1923	318,53	cooling
10,55	22,259	1,5555	289,4		10,55	38,3033	15,104	324,29		10,55	34,4272	13,1083	298,01	cooling
10,552	22,1078	-1,678	332,49		10,552	38,7602	19,461	269,77		10,552	33,576	9,966	330,03	cooling
10,554	22,2637	-3,2353	356,44		10,554	38,5769	18,5505	279,94		10,554	34,5075	10,6855	333	cooling
10,556	22,1205	0,2205	306,13		10,556	38,991	17,3541	302,45		10,556	33,7362	11,3304	313,2	cooling
10,558	21,748	-0,3759	309,26		10,558	37,9121	12,6814	352,69		10,558	34,6394	12,0313	316,03	cooling

10,56	22,0622	-0,4365	314,5	
10,562	22,1944	-2,0199	338,48	
10,564	21,7997	-3,8695	358,82	
10,566	22,1241	0,4219	303,36	
10,568	21,4812	0,7089	290,37	
10,57	22,3428	0,878	300,05	
10,572	22,3478	0,8152	300,99	
10,574	23,004	2,0471	292,95	
10,576	23,5172	1,1753	312,31	
10,578	23,9429	1,0617	319,84	
10,58	24,473	-0,8495	353,97	
10,582	24,4953	1,7512	317,93	
10,584	24,9949	2,0672	320,49	
10,586	24,6643	0,4401	338,62	
10,588	25,3573	0,5969	346,11	
10,59	24,6499	-0,0639	345,46	
10,592	25,3284	3,9614	298,68	cooling
10,594	24,5549	2,925	302,35	cooling
10,596	24,7413	1,7795	320,97	cooling
10,598	24,6839	-1,1831	361,58	cooling
10,6	24,4184	0,4455	335,11	cooling
10,602	24,6885	3,7451	292,76	cooling
10,604	24,1196	4,7706	270,47	cooling
10,606	25,0645	2,4131	316,63	cooling
10,608	24,5371	1,1361	327,11	cooling
10,61	24,9041	-2,8096	387,4	cooling
10,612	24,7909	-2,7538	385,04	cooling
10,614	25,6154	4,1635	299,87	cooling
10,616	24,6387	2,8611	304,42	cooling
10,618	25,4125	6,9002	258,77	cooling
10,62	24,8404	7,2078	246,48	cooling
10,622	24,2705	2,393	305,82	cooling
10,624	24,5974	2,7043	306,03	cooling
10,626	23,6694	2,056	302,12	cooling
10,628	24,4966	1,1785	325,95	cooling
10,63	24,0406	6,9814	238,46	cooling
10,632	24,0709	7,7152	228,63	
10,634	23,7819	5,63	253,74	
10,636	24,3631	5,8699	258,51	
10,638	23,3502	-1,329	344,98	
10,64	24,3282	-0,8637	352,15	
10,642	23,8045	0,691	323,09	
10,644	23,6233	-1,0016	344,22	
10,646	23,9559	0,2056	331,99	
10,648	23,4778	1,4409	308,04	
10,65	24,1325	2,2314	306,15	
10,652	23,5958	4,0083	273,81	
10,654	23,7225	0,6033	323,17	
10,656	23,3875	-3,6811	378,38	
10,658	24,0768	0,4041	330,91	cooling
10,66	23,2675	0,3754	320	cooling
10,662	23,83	2,5917	296,88	cooling
10,664	23,4373	1,6814	304,12	cooling
10,666	23,0182	-1,0933	337,04	cooling
10,668	23,2487	1,0473	310,34	cooling
10,67	22,9098	4,7198	254,27	cooling

10,56	38,0033	13,8792	337,22	
10,562	37,3366	16,1858	295,66	
10,564	37,7505	19,1097	260,57	
10,566	37,3455	17,474	277,77	
10,568	38,4325	15,1024	326,12	
10,57	38,1861	15,5412	316,54	
10,572	38,3143	14,9394	326,75	
10,574	38,6789	17,8797	290,74	
10,576	38,8248	17,7207	295	
10,578	38,366	17,616	290,05	
10,58	38,0376	16,8039	296,82	
10,582	38,305	19,426	263,9	
10,584	37,4538	17,7123	275,96	
10,586	37,8218	21,2413	231,77	
10,588	37,1765	19,7206	244,01	
10,59	37,9122	18,1836	275,78	
10,592	37,12	15,0417	308,62	
10,594	38,0091	16,1831	305,1	
10,596	37,9091	17,8971	279,74	
10,598	38,4341	17,7734	288,81	
10,6	38,2616	15,5564	317,38	
10,602	38,9294	15,1154	332,88	
10,604	38,2109	17,3682	291,35	
10,606	37,6515	17,4934	281,78	
10,608	37,5288	19,3114	254,65	
10,61	37,1665	17,5384	274,37	
10,612	37,1623	20,5466	232,26	
10,614	36,9482	20,5719	228,92	heating
10,616	37,9367	22,9943	208,87	heating
10,618	37,4181	19,8024	246,24	heating
10,62	38,2885	20,7298	245,44	heating
10,622	38,3806	21,8143	231,57	heating
10,624	38,9924	24,1016	208,15	heating
10,626	38,3544	23,1066	213,14	heating
10,628	38,8585	20,8116	252,27	heating
10,63	38,1258	16,1705	306,9	heating
10,632	38,0878	13,5292	343,29	heating
10,634	37,9422	15,3055	316,43	heating
10,636	38,2766	16,007	311,3	heating
10,638	38,1193	19,3242	262,73	heating
10,64	38,1399	17,0969	294,15	heating
10,642	39,1525	17,8062	298,39	heating
10,644	38,9701	16,4298	315,08	heating
10,646	39,2214	20,6158	260,08	heating
10,648	38,945	22,944	223,67	heating
10,65	39,1768	23,1192	224,46	heating
10,652	38,2749	17,7558	286,83	heating
10,654	38,8295	18,602	282,75	heating
10,656	38,3188	19,7885	259,03	heating
10,658	38,7139	22,9522	220,32	heating
10,66	38,1789	21,8422	228,36	heating
10,662	39,1625	17,676	300,35	heating
10,664	39,097	16,8618	310,82	heating
10,666	39,0858	14,502	343,64	heating
10,668	39,3573	16,24	323,15	heating
10,67	39,2925	16,916	312,79	heating

10,56	33,8794	11,2895	315,77	cooling
10,562	33,8275	5,0121	402,8	cooling
10,564	34,2702	6,853	383,25	cooling
10,566	34,7558	10,0346	345,57	cooling
10,568	34,0774	15,0087	266,55	cooling
10,57	33,6415	12,652	293,4	cooling
10,572	34,5977	14,4615	281,47	cooling
10,574	33,8707	7,3719	370,41	cooling
10,576	34,2943	9,5077	346,48	cooling
10,578	33,5571	8,8788	344,97	cooling
10,58	34,8572	13,2727	301,72	cooling
10,582	33,7725	11,953	305	cooling
10,584	34,2302	11,9864	310,94	cooling
10,586	33,4309	8,3666	350,36	cooling
10,588	34,2791	10,5215	332,1	cooling
10,59	33,2648	10,9838	311,46	cooling
10,592	34,1287	13,5198	288,08	cooling
10,594	33,5664	17,4122	225,81	cooling
10,596	33,3383	13,913	271,54	cooling
10,598	33,718	11,7831	306,62	cooling
10,6	34,2767	9,1711	350,94	cooling
10,602	33,8914	10,9757	320,33	cooling
10,604	33,3669	9,268	336,87	cooling
10,606	34,4684	14,9004	273,53	cooling
10,608	33,7371	11,9953	303,92	cooling
10,61	34,2142	16,264	250,92	cooling
10,612	33,1861	12,2677	292,41	cooling
10,614	34,0804	10,8927	324,13	cooling
10,616	33,034	8,2551	346,37	cooling
10,618	33,5904	10,2925	325,67	cooling
10,62	33,056	10,0845	321,11	cooling
10,622	33,4401	12,5074	292,61	cooling
10,624	32,8712	12,8358	280,07	cooling
10,626	33,6129	9,8471	332,21	cooling
10,628	33,5811	12,0389	301,13	cooling
10,63	32,9555	7,8992	350,25	cooling
10,632	33,948	10,6154	326,16	cooling
10,634	33,6255	7,7619	361,54	cooling
10,636	33,3011	9,9751	326,06	cooling
10,638	32,8081	9,2523	329,27	cooling
10,64	34,0634	16,0144	252,3	cooling
10,642	32,8315	10,8583	307,15	cooling
10,644	33,4245	14,0758	270,47	cooling
10,646	32,5028	10,3836	309,19	cooling
10,648	33,5493	8,691	347,48	cooling
10,65	32,5576	7,2249	354,11	cooling
10,652	33,472	11,0088	314	cooling
10,654	32,493	10,8253	302,88	cooling
10,656	33,0407	11,7161	298,09	cooling
10,658	32,1747	11,8285	284,41	cooling
10,66	33,0001	8,0854	348,27	cooling
10,662	32,4828	9,4854	321,47	cooling
10,664	32,4661	9,4544	321,67	cooling
10,666	32,9723	12,4662	286,65	cooling
10,668	33,3694	11,9097	299,97	cooling
10,67	32,7255	15,0206	247,49	cooling

10,672	23,1353	2,8311	283,82	cooling	10,672	38,7256	18,03	289,29	heating	10,672	32,3434	10,1355	310,43	cooling
10,674	22,7436	0,1703	315,54		10,674	38,5305	16,6125	306,38	heating	10,674	34,0344	14,9422	266,88	cooling
10,676	22,9219	-1,1296	336,2		10,676	39,0056	17,5656	299,7	heating	10,676	33,0392	11,3907	302,61	
10,678	22,5122	-2,1965	345,39	cooling	10,678	38,6062	14,4617	337,5	heating	10,678	33,5247	12,86	288,86	
10,68	22,9292	2,0783	291,46	cooling	10,68	38,815	16,4324	312,88	heating	10,68	32,5998	14,489	253,16	
10,682	22,2543	0,9922	297,21	cooling	10,682	38,4507	14,1541	339,63	heating	10,682	33,056	16,369	233,26	
10,684	22,6925	-1,2464	334,63	cooling	10,684	39,3591	17,3067	308,26	heating	10,684	31,5507	12,001	273,28	
10,686	22,5681	-1,4233	335,36	cooling	10,686	38,646	18,7972	277,46	heating	10,686	33,2293	16,7641	230,16	
10,688	22,1791	-0,3648	315,13	cooling	10,688	39,1399	21,9461	240,34	heating	10,688	31,6409	13,645	251,56	
10,69	22,0159	-1,5303	329,14	cooling	10,69	38,7783	22,9994	220,57	heating	10,69	32,571	12,28	283,64	
10,692	22,009	-1,093	322,93	cooling	10,692	38,39	19,2354	267,75	heating	10,692	31,6937	13,4632	254,84	
10,694	21,894	-1,7989	331,19	cooling	10,694	38,0716	15,2167	319,48	heating	10,694	32,475	8,2618	338,46	
10,696	21,4839	0,7578	289,72	cooling	10,696	38,5563	15,0596	328,45	heating	10,696	32,1847	8,5933	329,77	
10,698	21,1865	1,294	278,07	cooling	10,698	38,1949	18,688	272,68	heating	10,698	32,2658	10,5811	303,12	
10,7	20,6225	-2,902	328,84	cooling	10,7	38,1167	22,3023	221,06	heating	10,7	33,0304	13,9435	266,81	
10,702	21,0087	-3,5541	343,35	cooling	10,702	38,3985	25,183	184,73	heating	10,702	33,199	14,8036	257,14	
10,704	20,6235	-3,9741	343,84	cooling	10,704	38,0552	18,5229	273,03	heating	10,704	32,6908	19,7456	180,96	
10,706	20,9287	-3,3409	339,25	cooling	10,706	38,3421	16,176	309,85	heating	10,706	31,8177	10,676	295,53	heating
10,708	20,8276	-1,9197	317,97	cooling	10,708	38,3717	12,5364	361,14	heating	10,708	33,9793	12,4361	301,14	heating
10,71	20,5296	-3,4795	335,61	cooling	10,71	39,6003	15,0114	343,72	heating	10,71	32,2251	8,3538	333,69	heating
10,712	20,7525	-4,0584	346,82	cooling	10,712	39,6736	16,648	321,86	heating	10,712	33,2366	11,2395	307,49	
10,714	21,1494	1,4628	275,19	cooling	10,714	40,261	20,1703	280,84	heating	10,714	32,4245	14,3263	252,98	
10,716	20,7203	1,1115	274,1	cooling	10,716	40,3546	21,5461	262,91	heating	10,716	33,4434	18,3562	210,9	
10,718	21,1109	3,366	248,05	cooling	10,718	40,231	19,8346	285,11	heating	10,718	31,6389	12,644	265,52	heating
10,72	21,1715	3,8234	242,5	cooling	10,72	39,5353	15,3973	337,41	heating	10,72	33,6035	14,5464	266,39	heating
10,722	21,1648	-0,3557	300,82		10,722	39,6554	15,0816	343,5	heating	10,722	32,0767	9,6816	313,05	heating
10,724	21,806	0,5977	296,46		10,724	38,5846	15,9644	316,2	heating	10,724	32,7899	8,9188	333,68	heating
10,726	21,4613	1,5708	278,04		10,726	38,1546	17,5209	288,43		10,726	32,1434	13,9993	253,63	heating
10,728	21,6969	-1,8866	329,66		10,728	38,2102	20,3445	249,74		10,728	32,9881	13,1217	277,7	heating
10,73	21,886	0,5033	298,9		10,73	39,005	17,9963	293,67	heating	10,73	32,4932	14,9493	245,24	heating
10,732	21,4771	-1,1917	316,88		10,732	39,419	20,6847	261,88	heating	10,732	32,3533	14,1041	255,1	heating
10,734	21,6371	-3,4809	351,11		10,734	39,5329	17,7628	304,31	heating	10,734	33,4334	14,3002	267,45	heating
10,736	21,7799	0,4286	298,46	cooling	10,736	40,5121	17,1103	327,12	heating	10,736	33,5879	11,1074	314,25	heating
10,738	20,9897	-1,83	318,99	cooling	10,738	40,4915	15,3166	351,91	heating	10,738	33,4784	15,8501	246,42	heating
10,74	21,4163	-1,754	323,89	cooling	10,74	39,9698	15,7189	338,99		10,74	32,616	9,9923	316,25	heating
10,742	21,432	0,2887	295,55	cooling	10,742	39,6924	15,5505	337,47		10,742	34,7528	13,9987	290,11	heating
10,744	20,8149	-2,2158	321,93	cooling	10,744	39,499	16,6808	318,96		10,744	32,8989	10,6813	310,57	heating
10,746	21,2244	0,097	295,33	cooling	10,746	38,2591	11,6404	372,09		10,746	33,6378	13,1291	286,68	heating
10,748	20,4764	2,3774	253	cooling	10,748	38,7253	11,5061	380,48		10,748	32,72	13,29	271,6	heating
10,75	20,6769	0,5775	280,96		10,75	38,3929	11,1075	381,41		10,75	33,7012	16,4654	240,93	heating
10,752	20,9484	3,3282	246,3		10,752	39,1211	12,0938	377,8		10,752	32,054	13,5599	258,52	heating
10,754	20,8265	2,4006	257,57		10,754	39,0317	15,9986	321,97	cooling	10,754	33,838	14,7125	267,35	heating
10,756	21,0527	1,1658	277,99		10,756	39,7301	17,0583	316,92	cooling	10,756	32,5524	11,7608	290,64	heating
10,758	21,4115	4,9185	230,55		10,758	39,3869	18,314	294,57	cooling	10,758	33,2707	11,0435	310,7	heating
10,76	20,8438	1,5999	269		10,76	39,0904	16,5936	314,47	cooling	10,76	32,9261	15,9566	237,21	heating
10,762	21,1557	-0,3522	300,65		10,762	39,1433	16,1418	321,53	cooling	10,762	33,8706	14,5893	269,52	heating
10,764	21,0363	0,4855	287,27		10,764	39,3191	15,7127	329,98	cooling	10,764	33,3377	15,9167	243,52	heating
10,766	20,4154	-1,357	304,34		10,766	38,736	16,8094	306,5	cooling	10,766	33,0783	12,9511	281,35	heating
10,768	20,8475	0,8994	278,85		10,768	38,4717	13,6578	346,86	cooling	10,768	34,2787	15,4661	262,97	heating
10,77	20,275	0,7172	273,39		10,77	38,9992	13,2393	360,09	cooling	10,77	34,1929	12,9232	297,32	heating
10,772	20,9074	-1,549	313,91		10,772	38,6249	11,2709	382,37	cooling	10,772	33,7916	16,0744	247,66	heating
10,774	21,0258	-0,8446	305,72		10,774	39,0528	14,3907	344,74	cooling	10,774	33,0495	11,8715	296,04	heating
10,776	21,1101	-3,2562	340,61		10,776	38,8245	13,9461	347,76	cooling	10,776	35,0799	19,1338	222,9	heating
10,778	21,3209	-6,3732	387,12		10,778	39,3503	12,3763	377,06	cooling	10,778	33,1522	14,5138	260,54	heating
10,78	22,016	-1,6102	330,26		10,78	38,7292	10,7548	391,04	cooling	10,78	33,9565	15,5578	257,19	heating
10,782	21,6227	-0,2879	306,28		10,782	39,0412	13,0077	363,91	cooling	10,782	33,0487	13,4638	273,77	heating

10,784	22,141	3,5083	260,46		10,784	38,8603	17,174	303,14	cooling	10,784	33,9522	14,8295	267,31	heating
10,786	22,0147	6,5694	215,9		10,786	38,9259	19,0481	277,86	cooling	10,786	32,3747	13,5142	263,64	heating
10,788	21,534	2,5511	265,35		10,788	38,3233	16,8591	300,04	cooling	10,788	33,9608	16,4413	244,9	heating
10,79	21,866	0,9695	292,1		10,79	38,8496	16,7995	308,23	cooling	10,79	33,0709	14,7898	255,54	heating
10,792	21,2333	1,4393	276,69		10,792	38,2938	19,0502	269	cooling	10,792	33,6329	13,445	282,2	heating
10,794	21,3006	-0,0231	298,07		10,794	38,2056	17,3649	291,32	cooling	10,794	33,5979	14,3497	269,06	heating
10,796	21,0674	0,1626	292,22		10,796	38,8834	18,8548	279,97	cooling	10,796	34,6506	9,6	350,17	heating
10,798	21,4076	1,1659	282,95		10,798	38,9725	17,1355	305,25	cooling	10,798	34,4299	13,5471	291,91	heating
10,8	21,3188	-0,6921	307,68		10,8	38,7808	17,6018	296,05	cooling	10,8	33,6668	11,8568	304,87	heating
10,802	22,4202	4,0038	257,43		10,802	38,5174	15,9853	314,97	cooling	10,802	34,9439	15,4827	272,04	heating
10,804	22,1002	4,1226	251,3		10,804	39,0226	15,2711	332,01	cooling	10,804	34,3643	11,2503	323,1	heating
10,806	22,6126	3,5494	266,48		10,806	38,7649	13,501	353,15	cooling	10,806	33,8177	12,9898	291,14	heating
10,808	22,9787	3,5526	271,55		10,808	38,923	18,1456	290,44	cooling	10,808	33,0501	8,7671	339,44	heating
10,81	22,8748	0,195	317,03	heating	10,81	38,1326	15,9344	310,3	cooling	10,81	34,5859	16,2573	256,21	heating
10,812	23,4075	-2,4213	361,05		10,812	38,755	15,4248	326,12	cooling	10,812	32,6521	14,9386	247,61	heating
10,814	23,2335	-0,1606	327,01		10,814	37,8561	15,6556	310,33	cooling	10,814	33,6249	19,3902	198,98	heating
10,816	23,3955	0,024	326,7		10,816	38,2485	17,0576	296,22	cooling	10,816	32,9566	16,7119	227,08	heating
10,818	23,4625	0,8792	315,68		10,818	38,0108	18,7438	269,32	cooling	10,818	33,9608	14,507	271,94	heating
10,82	23,6248	2,9743	288,66		10,82	37,9841	17,976	279,68	cooling	10,82	33,0544	13,4711	273,75	heating
10,822	23,191	0,7876	313,17		10,822	37,6776	15,6624	307,74	cooling	10,822	34,295	16,2641	252,05	heating
10,824	23,7027	2,3128	299		10,824	38,4766	17,8694	288,06	cooling	10,824	33,6951	17,1366	231,46	heating
10,826	23,128	0,3409	318,53		10,826	37,9774	21,8111	225,98	cooling	10,826	33,7773	15,4735	255,86	heating
10,828	23,1462	-2,6525	360,63		10,828	37,916	21,0984	235,09		10,828	33,5896	14,7953	262,72	heating
10,83	23,4857	-2,7644	366,94		10,83	38,6106	22,5043	225,14		10,83	34,5955	10,4259	337,86	heating
10,832	23,3104	-2,5445	361,41		10,832	38,4004	17,8928	286,67		10,832	33,8691	14,9265	264,79	heating
10,834	23,7651	-0,8359	343,88		10,834	38,5414	19,3188	268,7		10,834	33,2667	11,8497	299,38	heating
10,836	23,1731	1,8339	298,29		10,836	38,1741	21,734	229,81		10,836	34,6975	15,8384	263,62	heating
10,838	23,1017	-0,0779	324,02		10,838	38,6368	23,1312	216,75		10,838	34,3332	10,646	331,11	heating
10,84	23,2071	1,7254	300,28		10,84	38,1455	20,3658	248,53		10,84	34,165	11,0679	322,86	heating
10,842	23,1328	4,4994	260,47		10,842	38,3079	20,8524	244		10,842	33,8801	8,8048	350,51	heating
10,844	22,7402	2,7841	278,96		10,844	38,0003	16,25	304,04	heating	10,844	35,3193	14,9951	284,1	heating
10,846	23,0525	3,9744	266,68		10,846	38,7191	16,5991	309,2	heating	10,846	33,719	12,8301	292	heating
10,848	22,569	0,1694	313,11		10,848	37,7243	14,9475	318,39		10,848	34,5605	16,061	258,6	heating
10,85	22,6463	-2,0395	345,07		10,85	38,4829	17,4152	294,5		10,85	33,6662	12,6612	293,62	heating
10,852	23,0812	0,4887	315,81		10,852	38,4318	21,4971	236,72		10,852	34,5365	10,7492	332,51	heating
10,854	22,7233	0,2237	314,51		10,854	38,2382	19,2312	265,69	heating	10,854	33,1482	10,0414	323	heating
10,856	23,6102	1,2598	312,43		10,856	38,1729	17,7016	286,16	heating	10,856	34,5637	11,2912	325,32	heating
10,858	23,1905	2,2387	292,88		10,858	38,6203	18,585	280,06	heating	10,858	33,6895	12,6812	293,67	heating
10,86	23,4026	-1,7439	351,51		10,86	38,0477	18,7388	269,91	heating	10,86	33,796	9,2079	343,71	heating
10,862	23,4384	-2,4483	361,86		10,862	38,1525	20,4667	247,22	heating	10,862	34,365	11,0241	326,27	heating
10,864	23,4447	-1,4993	348,68	cooling	10,864	38,3061	23,1814	211,42	heating	10,864	35,4441	9,4036	364,01	heating
10,866	23,1953	-2,6648	361,49	cooling	10,866	38,5789	19,034	273,21	heating	10,866	34,8265	13,0675	304,16	
10,868	23,6469	0,7035	320,71	cooling	10,868	38,7303	22,8436	222,07	heating	10,868	34,6037	11,4992	322,97	
10,87	23,0272	0,3508	316,98	cooling	10,87	38,0062	20,7654	241	heating	10,87	36,1051	16,3605	276	
10,872	23,1069	-0,0154	323,22	cooling	10,872	39,0131	19,9733	266,15	heating	10,872	35,2631	9,144	365,11	
10,874	23,4478	2,9361	286,72	cooling	10,874	38,9949	18,2508	289,97	heating	10,874	35,4028	11,9964	327,19	
10,876	23,0492	2,092	292,95	cooling	10,876	38,6879	17,5369	295,66	heating	10,876	34,4221	7,5457	375,69	
10,878	23,7746	5,4768	255,78	cooling	10,878	38,7053	17,4707	296,83	heating	10,878	36,2156	12,208	335,59	
10,88	23,3241	7,9407	215,04	cooling	10,88	38,9797	21,1182	249,68	heating	10,88	34,4593	11,6976	318,17	
10,882	23,5806	3,7207	277,61		10,882	37,894	17,3554	287,1	heating	10,882	34,8859	13,8962	293,4	
10,884	23,5906	1,6716	306,4		10,884	38,4648	18,7039	276,23	heating	10,884	34,2245	9,2024	349,77	
10,886	23,5145	0,7432	318,31		10,886	38,0097	18,9659	266,2	heating	10,886	35,4942	10,2653	352,66	
10,888	23,6896	1,572	309,17		10,888	38,1455	17,2128	292,61	heating	10,888	34,0944	9,7061	340,91	
10,89	24,2545	8,4307	221,19		10,89	37,9041	19,6125	255,69	heating	10,89	35,7698	15,7209	280,25	
10,892	23,2867	5,4781	248,94		10,892	38,0754	20,3085	248,35	heating	10,892	34,8016	17,8759	236,6	
10,894	23,4969	-1,5002	349,42		10,894	38,0114	20,4058	246,1	heating	10,894	35,0826	15,1971	277,97	

10,896	23,4666	-4,7117	393,89		10,896	38,2555	19,4265	263,2	heating		10,896	35,1678	16,1918	265,26	
10,898	23,2759	-5,838	406,97		10,898	38,3792	16,8258	301,29	heating		10,898	35,6778	10,9712	345,36	
10,9	23,4251	-3,1768	371,86	cooling	10,9	39,314	14,139	351,91	heating		10,9	35,0635	11,2713	332,58	
10,902	23,5826	1,1116	314,11	cooling	10,902	39,0774	17,5759	300,56	heating		10,902	34,338	10,0481	339,54	
10,904	23,652	0,7382	320,3	cooling	10,904	38,7883	16,8231	307,04	heating		10,904	35,2853	14,6588	288,33	
10,906	23,6013	1,6748	306,5	cooling	10,906	39,2857	17,3217	307,02	heating		10,906	34,7562	11,425	326,14	
10,908	23,8102	4,2026	274,09	cooling	10,908	38,9848	13,4186	357,38	heating		10,908	34,8668	15,1699	275,33	
10,91	23,5726	3,1983	284,8	cooling	10,91	38,9157	13,4749	355,62	heating		10,91	34,0939	9,4218	344,88	
10,912	24,0745	3,2583	290,98	cooling	10,912	38,6633	14,3336	340,09	heating		10,912	35,7987	13,2468	315,24	
10,914	23,5237	3,6496	277,81	cooling	10,914	39,1084	17,1598	306,81	heating		10,914	34,0808	10,4477	330,36	
10,916	23,7464	2,8946	291,48		10,916	38,0465	15,5134	314,98	heating		10,916	34,7039	12,0098	317,23	
10,918	23,4993	1,0443	313,89		10,918	38,6206	16,7522	305,69			10,918	34,1966	11,0882	323,02	
10,92	23,678	-1,0718	345,97		10,92	38,0007	17,262	289,9			10,92	34,838	12,8622	307,19	
10,922	23,4079	-3,2765	373,01	cooling	10,922	38,3947	17,6011	290,66			10,922	33,8129	12,7826	293,97	
10,924	24,1814	1,5295	316,64	cooling	10,924	38,0821	18,2151	277,71			10,924	34,9246	14,6448	283,48	
10,926	23,5463	2,7086	291,28	cooling	10,926	38,9313	16,2473	317,09			10,926	34,2023	13,9222	283,49	
10,928	23,6056	2,1679	299,67	cooling	10,928	38,8201	16,7244	308,86			10,928	34,1493	9,9538	338,22	
10,93	23,5692	2,3367	296,8	cooling	10,93	39,1241	17,8293	297,67			10,93	34,4589	11,3337	323,26	
10,932	23,4344	0,1502	325,48	cooling	10,932	39,7002	20,8984	262,82			10,932	34,9346	8,4348	370,43	
10,934	23,9501	0,4538	328,44	cooling	10,934	39,7046	19,9112	276,68			10,934	34,5345	12,0669	314,06	cooling
10,936	23,7276	5,2101	258,85	cooling	10,936	39,4349	20,2142	268,68			10,936	33,8243	8,4615	354,53	cooling
10,938	23,7344	7,427	227,95	cooling	10,938	38,965	15,2657	331,28	heating		10,938	35,2663	11,4332	333,15	cooling
10,94	23,6954	8,0839	218,23		10,94	40,0437	15,1972	347,32			10,94	34,5956	7,6609	376,51	cooling
10,942	23,7579	5,5973	253,86		10,942	38,7354	12,9132	360,96			10,942	34,5259	10,4179	336,99	cooling
10,944	23,4433	-1,5074	348,77		10,944	39,2009	17,1206	308,65			10,944	34,028	8,5329	356,38	cooling
10,946	24,0535	-1,0545	350,97		10,946	38,5169	20,6693	249,48			10,946	35,2293	11,6503	329,6	cooling
10,948	23,5723	-0,2757	333,36		10,948	38,9056	21,1557	248,12			10,948	33,6209	5,5156	392,87	cooling
10,95	23,7744	2,464	297,89		10,95	38,1539	16,6275	300,91			10,95	34,653	7,7126	376,59	cooling
10,952	23,542	2,928	288,15		10,952	39,3119	16,3332	321,21			10,952	33,8577	9,094	346,16	cooling
10,954	23,364	-0,3351	331,28		10,954	39,0543	16,3202	317,79			10,954	34,5222	11,6404	319,85	cooling
10,956	23,8258	-1,4718	353,62		10,956	39,277	19,4771	276,77			10,956	33,355	12,7318	288,28	cooling
10,958	23,5535	0,9182	316,41		10,958	39,177	22,1981	237,34			10,958	34,393	10,9946	327,07	cooling
10,96	23,8219	2,1553	302,87		10,96	39,4094	19,0105	285,15			10,96	33,7465	8,8315	348,27	cooling
10,962	23,6386	3,5704	280,52		10,962	39,5605	21,4926	252,56			10,962	33,8898	6,8328	378,22	cooling
10,964	23,8903	4,4949	271,12		10,964	38,4334	19,2647	267,95			10,964	33,9138	10,9667	320,77	cooling
10,966	23,644	4,5332	267,14		10,966	39,3314	20,3573	265,23	heating		10,966	34,5772	13,4501	295,33	cooling
10,968	23,9268	4,61	270,02		10,968	38,7769	18,4059	284,76	heating		10,968	33,7701	16,8438	236,6	cooling
10,97	23,6169	1,9585	302,75		10,97	38,5201	17,7793	289,93	heating		10,97	33,2264	10,0175	324,43	cooling
10,972	23,7467	2,5926	295,7		10,972	38,3079	15,2766	321,94	heating		10,972	34,535	9,901	344,35	cooling
10,974	23,4279	2,5813	291,4		10,974	39,3207	19,0975	282,69	heating		10,974	33,4993	5,1016	396,96	cooling
10,976	23,3342	3,0299	283,82		10,976	38,2166	14,5227	331,2	heating		10,976	33,9671	12,5738	299,05	cooling
10,978	23,1808	0,5453	316,41		10,978	38,9	16,0185	319,85			10,978	33,0618	14,9823	252,72	cooling
10,98	23,076	-1,8434	348,34		10,98	38,4007	16,069	312,16			10,98	34,1544	16,4951	246,85	cooling
10,982	23,4857	-1,0569	343,07		10,982	38,5806	14,4679	337,06			10,982	33,088	12,6343	285,91	cooling
10,984	23,1278	1,0675	308,37		10,984	38,7209	17,0843	302,45			10,984	34,2923	12,9246	298,69	cooling
10,986	23,0171	1,6898	298,12		10,986	38,9901	16,0796	320,25			10,986	33,4947	8,0837	355,21	cooling
10,988	22,5518	2,6841	277,72		10,988	39,0553	17,9866	294,51			10,988	34,5012	11,3648	323,41	cooling
10,99	22,2545	1,9856	283,33		10,99	38,7024	19,1829	272,85			10,99	33,3808	13,4623	278,43	cooling
10,992	22,2279	2,3384	278,02		10,992	38,9183	18,2471	288,95			10,992	34,2417	12,4933	304,01	cooling
10,994	22,292	4,9436	242,51		10,994	39,2079	12,7195	370,27			10,994	33,8281	15,3022	258,96	cooling
10,996	21,9317	2,7644	267,93		10,996	39,2744	18,3812	292,06			10,996	33,1639	11,1773	307,34	cooling
10,998	21,7967	1,2323	287,46		10,998	38,4333	16,6254	304,84			10,998	33,96	13,1435	290,98	cooling
11	21,8156	2,612	268,44		11	39,3063	18,895	285,32			11	34,0662	13,0181	294,22	cooling
11,002	21,566	3,0418	258,94		11,002	38,0634	15,068	321,44			11,002	33,4077	12,6431	290,26	cooling
11,004	21,685	2,8848	262,8		11,004	38,1268	14,9344	324,2			11,004	33,1948	7,3185	361,71	cooling
11,006	21,2567	1,9381	270,05		11,006	38,072	16,769	297,78			11,006	34,7279	12,344	312,89	cooling

11,008	21,4119	0,7045	289,46		11,008	38,5661	18,9586	274,08		11,008	33,5758	7,3614	366,44	cooling
11,01	21,6714	2,5705	267		11,01	37,9111	13,7865	337,23		11,01	34,3048	13,4165	291,99	cooling
11,012	21,7055	3,6793	251,98		11,012	38,8318	11,5805	380,93		11,012	33,3006	12,4171	291,92	cooling
11,014	21,9217	1,9017	279,85	heating	11,014	38,6142	10,637	391,08		11,014	34,354	11,6093	317,94	cooling
11,016	22,3725	-0,9991	326,7	heating	11,016	38,9217	12,3526	371,4		11,016	33,168	7,993	351,91	cooling
11,018	22,5662	-3,479	364,07		11,018	38,7443	18,5863	281,78	cooling	11,018	34,1336	7,8592	367,28	cooling
11,02	22,604	-3,6004	366,3		11,02	38,7339	17,9511	290,51		11,02	33,456	3,189	423,09	cooling
11,022	22,7468	-0,7865	328,96		11,022	38,3805	17,5047	291,81		11,022	33,817	6,8669	376,72	cooling
11,024	22,6467	0,6735	307,15		11,024	37,912	14,0902	332,99		11,024	32,7733	11,9276	291,39	cooling
11,026	22,5988	2,143	285,94		11,026	38,2216	12,0981	365,17		11,026	33,6005	14,5182	266,74	cooling
11,028	22,3759	1,5237	291,48		11,028	38,1941	10,1108	392,56	cooling	11,028	33,2839	17,7895	216,59	cooling
11,03	22,5537	0,731	305,05		11,03	37,9573	16,3572	301,94	cooling	11,03	32,7169	11,8409	291,82	cooling
11,032	22,5695	2,1515	285,41		11,032	37,4295	16,7933	288,46	cooling	11,032	33,7199	10,7547	321,02	cooling
11,034	22,5485	3,9855	259,48		11,034	38,0602	18,0911	279,14	cooling	11,034	33,8891	9,9964	333,98	cooling
11,036	22,5876	2,6921	278,11		11,036	36,8243	11,9405	347,84	cooling	11,036	33,5405	13,1238	285,4	cooling
11,038	23,0036	-0,6085	330,06		11,038	37,405	10,9398	369,94	cooling	11,038	33,0541	11,6967	298,55	cooling
11,04	23,4389	-1,6114	350,17		11,04	37,1387	14,5454	315,82	cooling	11,04	34,7327	19,262	216,26	cooling
11,042	23,5427	-2,1629	359,33		11,042	37,4657	16,8282	288,48	cooling	11,042	32,8628	13,1775	275,17	cooling
11,044	23,6723	-1,1069	346,38		11,044	37,0922	16,6225	286,14	cooling	11,044	33,5188	14,0678	271,9	
11,046	24,0513	1,141	320,25		11,046	38,1814	15,6656	314,74	cooling	11,046	32,3985	11,354	294,17	
11,048	23,8545	-1,0018	347,45		11,048	38,0084	14,3437	330,8	cooling	11,048	33,0379	9,834	324,36	
11,05	24,4678	-2,7896	381,02		11,05	38,8339	16,8235	307,67	cooling	11,05	31,9825	8,9311	322,22	
11,052	24,5986	-1,9709	371,4		11,052	38,9224	21,3837	245,16	cooling	11,052	33,2254	14,0404	268,18	
11,054	24,7425	-1,0331	360,3	cooling	11,054	39,4689	18,4129	294,33	cooling	11,054	32,4366	12,3985	280,1	
11,056	24,8013	2,2005	315,92	cooling	11,056	39,3051	19,7945	272,73	cooling	11,056	32,9111	11,5635	298,41	
11,058	25,1722	7,2653	250,31	cooling	11,058	38,3262	15,1223	324,36		11,058	33,0335	12,4624	287,55	
11,06	25,0272	5,1682	277,6	cooling	11,06	39,2003	17,3733	305,11		11,06	33,4868	7,5204	362,97	
11,062	25,3021	1,9904	325,86	cooling	11,062	38,4199	17,7056	289,56		11,062	33,8305	10,7284	322,93	
11,064	25,331	0,576	346,04	cooling	11,064	38,0482	18,2422	276,86		11,064	33,1302	12,6085	286,86	
11,066	25,0428	-0,0817	351,2	cooling	11,066	37,7772	15,7532	307,86		11,066	33,9012	13,7191	282,12	
11,068	24,9632	2,3034	316,75	cooling	11,068	38,4421	15,6793	318,19		11,068	33,1918	8,8061	340,88	
11,07	24,6521	4,055	287,92	cooling	11,07	37,1241	10,5408	371,59		11,07	33,0134	11,0942	306,4	
11,072	24,4944	-1,9131	369,14	cooling	11,072	38,2775	15,335	320,7	cooling	11,072	31,8805	8,6753	324,38	
11,074	24,263	-6,0888	424,27	cooling	11,074	37,4924	17,2935	282,35	cooling	11,074	33,2413	16,6958	231,28	
11,076	24,2359	-5,5852	416,85	cooling	11,076	37,9962	16,1592	305,25	cooling	11,076	31,5656	15,2359	228,27	
11,078	24,0151	-2,4161	369,47	cooling	11,078	38,0177	18,955	266,47	cooling	11,078	32,4153	14,717	247,4	
11,08	24,4317	2,421	307,68	cooling	11,08	38,5521	16,4838	308,48		11,08	32,0929	11,4876	288,03	
11,082	23,8331	1,6213	310,49	cooling	11,082	38,3014	15,0913	324,44		11,082	33,0376	12,5583	286,27	
11,084	24,1356	-0,6533	346,51	cooling	11,084	38,1256	17,4228	289,39		11,084	32,3395	14,2211	253,27	
11,086	23,945	-0,7133	344,69	cooling	11,086	38,154	19,6087	259,24		11,086	33,2462	19,903	186,52	
11,088	23,9235	0,588	326,2	cooling	11,088	38,0272	15,8388	310,16		11,088	32,4074	19,825	175,88	
11,09	24,2625	4,6585	274,03	cooling	11,09	38,2665	20,0529	254,6		11,09	32,5668	14,7009	249,74	heating
11,092	24,7114	7,6649	238,29	cooling	11,092	37,3981	17,2244	282		11,092	32,5306	11,7665	290,25	heating
11,094	25,0588	2,916	309,52	cooling	11,094	39,0924	21,3575	247,91		11,094	32,7198	7,8579	347,53	heating
11,096	25,6814	-1,5887	381,2	cooling	11,096	37,9147	20,0505	249,72		11,096	32,7096	13,4434	269,31	heating
11,098	25,9206	-3,8694	416,42	cooling	11,098	38,5318	20,3863	253,65		11,098	32,1943	14,8361	242,64	heating
11,1	25,7209	-1,2716	377,32	cooling	11,1	38,2891	19,1062	268,15		11,1	33,2209	18,4969	205,82	heating
11,102	25,5803	6,2089	270,78	cooling	11,102	38,9411	20,7705	254		11,102	33,0734	13,8889	268,17	heating
11,104	24,619	4,5105	281,09	cooling	11,104	37,9551	18,3214	274,45		11,104	33,1976	13,4475	276,08	heating
11,106	25,4061	-0,8597	367,16	cooling	11,106	38,9725	23,0318	222,83		11,106	32,9091	9,0126	334,04	heating
11,108	25,3873	-2,6869	392,44	cooling	11,108	38,0566	22,6627	215,18	heating	11,108	33,8732	13,7893	280,74	heating
11,11	25,4745	-1,6622	379,33	cooling	11,11	37,7253	16,9566	290,31	heating	11,11	32,9195	13,9154	265,65	heating
11,112	25,4403	2,0552	326,89	cooling	11,112	38,489	19,7906	261,38	heating	11,112	33,4297	15,3837	252,26	heating
11,114	25,6899	6,0823	274,09	cooling	11,114	37,8407	15,4097	313,55	heating	11,114	32,8194	12,1582	288,81	heating
11,116	25,5917	4,7489	291,35	cooling	11,116	38,406	19,9269	258,31	heating	11,116	33,2406	10,6139	316,29	heating
11,118	26,0827	7,0182	266,49	cooling	11,118	37,4555	20,2613	240,35	heating	11,118	32,4961	9,5498	320,76	heating

11,12	25,6627	5,0052	288,76	cooling	11,12	38,5306	19,6822	263,47	heating	11,12	33,5513	12,2886	297,22	heating
11,122	25,6336	0,5287	350,93	cooling	11,122	38,3082	14,8664	327,68	heating	11,122	32,8909	13,198	275,28	heating
11,124	26,0232	2,8744	323,59	cooling	11,124	38,6979	19,2713	271,56	heating	11,124	33,2571	12,3127	292,77	heating
11,126	25,4519	3,3703	308,67	cooling	11,126	38,5165	13,946	343,46	heating	11,126	33,1976	14,2805	264,43	heating
11,128	26,0544	2,0484	335,57	cooling	11,128	39,6761	21,9227	248,17	heating	11,128	33,7566	12,0199	303,85	heating
11,13	26,2053	4,2549	306,83	cooling	11,13	37,9617	21,2311	233,87	heating	11,13	33,1733	12,1822	293,42	heating
11,132	26,3707	4,3054	308,44	cooling	11,132	37,9486	20,378	245,61	heating	11,132	32,9232	8,211	345,44	heating
11,134	26,3311	2,3721	334,91	cooling	11,134	38,1217	23,5919	203,11	heating	11,134	33,8571	12,8381	293,81	heating
11,136	26,8275	4,4083	313,39	cooling	11,136	36,8315	19,6637	239,98	heating	11,136	33,7132	14,0173	275,32	heating
11,138	25,7675	-0,3006	364,39	cooling	11,138	37,3123	20,7032	232,17	heating	11,138	33,461	16,7647	233,39	heating
11,14	26,1029	-2,7686	403,58	cooling	11,14	37,7435	23,7633	195,42	heating	11,14	33,1043	12,24	291,65	heating
11,142	26,0317	-1,4018	383,48	cooling	11,142	37,2248	22,5874	204,61	heating	11,142	34,3997	15,0494	270,49	heating
11,144	26,0006	-0,4565	369,83	cooling	11,144	37,2485	18,9562	255,7	heating	11,144	33,1166	12,5256	287,83	heating
11,146	26,0711	1,4198	344,59	cooling	11,146	38,3932	23,9229	202,27	heating	11,146	33,7023	15,1791	258,93	heating
11,148	25,8907	4,5836	297,84	cooling	11,148	38,4458	17,4897	292,94	heating	11,148	32,9853	14,3388	260,65	heating
11,15	25,6654	3,0843	315,65	cooling	11,15	38,182	22,274	222,37	heating	11,15	33,5923	13,0773	286,77	heating
11,152	25,4297	3,0863	312,33	cooling	11,152	37,5028	18,1369	270,71	heating	11,152	33,2038	13,6608	273,18	heating
11,154	25,5105	4,8841	288,33	cooling	11,154	38,6247	17,3254	297,73	heating	11,154	34,2353	14,3322	278,22	heating
11,156	25,042	1,7613	325,43	cooling	11,156	38,4575	14,764	331,2	heating	11,156	33,9487	14,3198	274,38	heating
11,158	25,6382	1,1843	341,83	cooling	11,158	38,3234	16,6127	303,48	heating	11,158	34,502	15,1772	270,13	heating
11,16	25,4157	2,6102	318,79	cooling	11,16	38,8784	14,994	333,87	heating	11,16	34,4377	17,3419	238,97	heating
11,162	25,7397	4,7558	293,32	cooling	11,162	39,5994	16,9116	317,14	heating	11,162	34,7369	13,1602	301,61	heating
11,164	25,8788	5,7225	281,76	cooling	11,164	38,2717	12,0909	365,97	heating	11,164	34,3881	15,6896	261,38	heating
11,166	26,0945	6,6043	272,44	cooling	11,166	38,8608	14,8137	336,14	heating	11,166	33,394	9,8573	329,01	heating
11,168	26,1507	3,2821	319,67	cooling	11,168	37,9218	17,8899	280,02	heating	11,168	34,1219	12,4005	303,63	heating
11,17	26,3715	1,0325	354,2	cooling	11,17	37,5826	16,3013	297,48	heating	11,17	33,5286	16,2227	241,91	heating
11,172	26,6152	-0,0729	373,06	cooling	11,172	37,0718	18,1295	264,79	heating	11,172	33,3578	18,6765	205,22	heating
11,174	26,2319	0,0373	366,16	cooling	11,174	38,0223	16,6224	299,14	heating	11,174	33,0964	13,6606	271,68	heating
11,176	26,1301	0,7534	354,73	cooling	11,176	37,6875	15,3152	312,73	heating	11,176	33,9421	11,2281	317,51	heating
11,178	25,5162	2,9538	315,39	cooling	11,178	38,0083	13,6174	340,95	heating	11,178	33,6645	7,0101	372,59	heating
11,18	25,2925	5,0498	282,96	cooling	11,18	38,9284	17,7363	296,23	heating	11,18	34,8576	13,5957	297,21	heating
11,182	24,8546	4,7406	281,16	cooling	11,182	39,7503	18,3882	298,61	heating	11,182	34,2164	16,9104	241,91	heating
11,184	25,3364	3,5488	304,56	cooling	11,184	39,5827	24,5298	210,42	heating	11,184	34,854	15,8153	266,13	heating
11,186	25,7793	3,0752	317,37	cooling	11,186	38,9516	19,4342	272,82	heating	11,186	34,5948	15,7388	263,58	heating
11,188	25,9145	7,1729	261,98	cooling	11,188	40,2031	20,3882	276,98	heating	11,188	34,6066	10,8772	331,7	heating
11,19	25,6034	8,2374	242,75	cooling	11,19	38,7326	15,9904	317,9	heating	11,19	34,0652	10,6273	327,63	heating
11,192	25,0921	3,9486	295,55	cooling	11,192	38,5109	17,5101	293,56	heating	11,192	33,9695	11,2284	317,89	heating
11,194	25,5137	-0,1154	358,26	cooling	11,194	38,0044	14,0394	335	heating	11,194	33,6837	12,1192	301,44	heating
11,196	24,643	-2,2601	376,07	cooling	11,196	38,7726	14,6718	336,89	heating	11,196	34,3649	13,8735	286,44	heating
11,198	24,7796	-0,1181	348,03	cooling	11,198	37,7081	13,5675	337,45	heating	11,198	33,9295	17,8006	225,46	heating
11,2	24,4187	5,0955	270,11	cooling	11,2	38,8748	16,8663	307,65	heating	11,2	33,7215	10,0941	330,28	heating
11,202	24,406	5,8228	259,77	cooling	11,202	38,5207	15,6651	319,49		11,202	34,8011	13,3134	300,37	heating
11,204	24,1331	1,4743	316,74	cooling	11,204	38,9088	12,4668	369,62		11,204	33,9217	12,7934	295,34	heating
11,206	24,5641	1,2201	326,31	cooling	11,206	39,0827	12,9299	365,58		11,206	34,0035	11,8383	309,84	heating
11,208	24,3633	2,3411	307,84	cooling	11,208	39,9554	12,0419	390,19		11,208	34,1667	11,7098	313,91	heating
11,21	24,2692	6,0699	254,4	cooling	11,21	39,1733	16,6156	315,32		11,21	34,492	11,5744	320,35	heating
11,212	24,2714	9,9939	199,58	cooling	11,212	38,471	16,0825	312,96		11,212	34,0619	9,1864	347,72	heating
11,214	23,8671	7,8581	223,78	cooling	11,214	39,9605	22,0827	249,91		11,214	35,2117	18,0089	240,47	
11,216	24,1728	1,3864	318,52		11,216	39,5565	19,179	284,85		11,216	33,8704	17,1661	233,5	heating
11,218	23,7109	-2,1034	360,85		11,218	39,515	19,5356	279,28		11,218	34,7728	14,0835	289,21	heating
11,22	23,965	-3,2332	380,19	cooling	11,22	39,1005	15,1716	334,49		11,22	34,7383	15,6094	267,39	heating
11,222	23,9462	1,0053	320,68	cooling	11,222	40,3276	17,7347	315,82		11,222	35,0685	9,381	359,07	heating
11,224	24,42	6,3106	253,14	cooling	11,224	38,752	15,6004	323,63		11,224	34,7294	8,8514	361,74	heating
11,226	23,8898	2,268	302,24	cooling	11,226	39,5199	22,5277	237,53		11,226	34,6666	10,1989	342,02	
11,228	24,9813	-0,1671	351,54	cooling	11,228	38,2921	19,1532	267,53		11,228	34,4629	10,6583	332,75	
11,23	24,6044	-1,3969	363,46	cooling	11,23	38,8688	15,7413	323,29		11,23	35,2427	13,3497	306,03	

11,232	24,5386	-0,4478	349,27	cooling	11,232	37,4878	12,3671	351,15		11,232	34,5143	17,5974	236,47	
11,234	24,5926	3,6239	293,11	cooling	11,234	38,5677	11,9732	371,75		11,234	34,4111	10,6605	332	
11,236	24,2702	3,9878	283,52	cooling	11,236	38,4248	11,4824	376,61		11,236	35,2941	12,785	314,64	
11,238	24,4412	0,6066	333,17	cooling	11,238	38,7089	10,7836	390,35		11,238	34,2472	7,2498	377,38	
11,24	24,1132	1,5015	316,08	cooling	11,24	38,8172	11,9153	376,05	cooling	11,24	34,533	6,89	386,41	
11,242	24,0882	1,132	320,89	cooling	11,242	39,7181	13,8217	361,99	cooling	11,242	34,5579	10,9763	329,64	
11,244	23,9037	0,7178	324,1	cooling	11,244	39,0528	18,4053	288,62	cooling	11,244	34,6883	13,3792	297,87	
11,246	24,4538	3,2526	296,36	cooling	11,246	38,2039	12,2788	362,39	cooling	11,246	34,2629	13,9479	283,97	
11,248	23,8604	0,8257	321,99	cooling	11,248	39,8685	14,47	355,03	cooling	11,248	34,8817	18,6423	227	
11,25	24,7085	2,4332	311,38	cooling	11,25	39,0326	11,0323	391,4	cooling	11,25	33,6733	11,6874	307,33	
11,252	24,1123	3,907	282,44	cooling	11,252	38,7883	15,7063	322,65	cooling	11,252	34,8936	9,6206	353,28	
11,254	23,9301	3,8088	281,27	cooling	11,254	38,2337	14,742	328,38	cooling	11,254	34,4739	10,4181	336,26	
11,256	24,0465	5,7769	255,38	cooling	11,256	39,2045	16,7833	313,41	cooling	11,256	35,0765	8,8432	366,7	
11,258	24,0651	7,987	224,75	cooling	11,258	37,8884	13,8996	335,33	cooling	11,258	34,7547	15,4967	269,2	
11,26	24,0125	3,38	288,41		11,26	38,5544	19,254	269,79	cooling	11,26	34,3704	14,5682	276,8	
11,262	24,1464	1,1841	320,98		11,262	37,8416	16,6331	296,46	cooling	11,262	34,3116	10,1512	337,73	
11,264	24,2916	-0,0894	340,81		11,264	38,1485	15,1231	321,86	cooling	11,264	34,9123	10,2857	344,24	
11,266	23,918	-2,3134	366,68		11,266	37,505	16,6759	291,16	cooling	11,266	33,6773	8,0486	358,25	
11,268	24,3827	-0,2797	344,74	cooling	11,268	38,2674	16,5966	302,93	cooling	11,268	34,1514	6,6959	383,79	
11,27	23,8481	-2,8354	372,99	cooling	11,27	38,3578	18,9655	271,08	cooling	11,27	34,1673	12,8702	297,7	cooling
11,272	24,4533	-4,2157	400,75	cooling	11,272	38,1871	17,0524	295,43	cooling	11,272	33,6325	10,796	319,22	cooling
11,274	24,6476	-0,1786	347,03	cooling	11,274	38,5544	17,4986	294,33	cooling	11,274	34,4179	13,4844	292,62	cooling
11,276	24,3068	1,7666	315,08	cooling	11,276	39,5029	15,6276	333,74	cooling	11,276	33,8175	13,319	286,54	cooling
11,278	24,5575	0,6423	334,3	cooling	11,278	38,7392	20,6002	253,56	cooling	11,278	34,6554	10,9434	331,46	cooling
11,28	24,5717	2,1704	313,14	cooling	11,28	38,2117	16,0578	309,68	cooling	11,28	34,5179	13,4929	293,9	cooling
11,282	24,4232	-0,184	343,97	cooling	11,282	39,6073	18,6421	293,06	cooling	11,282	34,4918	13,355	295,46	cooling
11,284	24,4973	0,646	333,41	cooling	11,284	39,3226	15,5176	332,76	cooling	11,284	34,0487	11,002	322,16	cooling
11,286	24,8666	4,0841	290,51	cooling	11,286	38,8676	16,7194	309,6	cooling	11,286	34,7499	13,3308	299,41	cooling
11,288	24,1065	1,3262	318,44	cooling	11,288	38,7213	17,4408	297,47	cooling	11,288	33,777	13,566	282,52	cooling
11,29	24,5663	1,672	320,03	cooling	11,29	38,8321	18,3685	286,05	cooling	11,29	34,9119	16,9755	250,72	
11,292	23,8587	0,9469	320,27	cooling	11,292	37,5044	14,2583	324,95	cooling	11,292	33,7384	18,3474	215,14	
11,294	23,7348	-2,0048	359,8	cooling	11,294	38,6491	19,6837	265,11	cooling	11,294	33,8643	11,3733	314,39	
11,296	23,7634	-1,426	352,11	cooling	11,296	37,4324	20,394	238,17		11,296	34,3138	11,6585	316,69	
11,298	23,2869	0,7701	314,75	cooling	11,298	37,8934	18,6955	268,36		11,298	33,8998	8,8924	349,57	
11,3	23,4129	0,6123	318,72	cooling	11,3	37,3264	21,8621	216,17		11,3	34,1869	10,3209	333,61	
11,302	23,3298	4,6493	261,13	cooling	11,302	38,1437	19,7906	256,55		11,302	33,7867	13,5735	282,55	
11,304	23,276	3,0429	282,83	cooling	11,304	37,6083	19,9121	247,37		11,304	34,2156	13,8273	285	
11,306	22,9478	0,2782	316,89	cooling	11,306	37,5284	18,8327	261,34		11,306	33,8487	8,8363	349,64	
11,308	23,6328	3,7064	278,54	cooling	11,308	38,0503	20,2281	249,13		11,308	34,2952	10,6328	330,77	
11,31	22,2596	1,8254	285,64	cooling	11,31	38,7074	19,8313	263,86		11,31	33,1733	4,0005	407,79	
11,312	22,798	1,0876	303,48	cooling	11,312	37,8038	21,3283	230,3		11,312	34,7518	9,3031	355,73	cooling
11,314	22,1719	0,9236	297,02	cooling	11,314	37,5351	12,5853	348,76	heating	11,314	33,2049	10,1121	322,8	cooling
11,316	22,4718	-0,5555	321,89	cooling	11,316	39,4871	15,7746	331,47		11,316	34,0158	11,196	318,99	cooling
11,318	22,1031	0,0137	308,78	cooling	11,318	38,3293	15,1838	323,54		11,318	33,5726	12,7145	291,57	cooling
11,32	22,1008	2,9716	267,4	cooling	11,32	38,847	22,4157	229,69		11,32	33,5509	9,7127	333,22	cooling
11,322	21,8195	-0,2719	308,8	cooling	11,322	38,2233	24,3502	193,92		11,322	33,3742	7,9449	355,46	cooling
11,324	21,9054	-0,0966	307,56	cooling	11,324	38,5154	20,5984	250,45	heating	11,324	34,0337	10,3058	331,68	cooling
11,326	22,0213	0,2024	305	cooling	11,326	37,7145	15,5261	310,16	heating	11,326	33,2993	10,9161	312,88	cooling
11,328	21,5955	-1,0429	316,45	cooling	11,328	38,429	14,6355	332,6	heating	11,328	33,4831	10,2186	325,2	cooling
11,33	22,0356	0,7191	297,97	cooling	11,33	37,7642	13,0976	344,8	heating	11,33	33,8132	14,482	270,22	cooling
11,332	21,0668	-0,0553	295,26	cooling	11,332	37,9429	15,173	318,29		11,332	33,7485	11,1593	315,76	cooling
11,334	21,5585	-2,0429	329,91	cooling	11,334	37,9243	19,2051	261,67		11,334	33,7485	15,5853	253,89	cooling
11,336	21,2047	-1,6196	319,05	cooling	11,336	38,4878	16,624	305,62		11,336	33,0036	10,5121	314,4	cooling
11,338	21,7445	-1,1353	319,83	cooling	11,338	38,5936	20,1162	258,29		11,338	33,6812	9,7524	334,49	cooling
11,34	21,0602	-3,5081	343,43	cooling	11,34	38,0257	15,981	308,15		11,34	33,4867	9,3575	337,29	cooling
11,342	21,4636	-2,1202	329,67	cooling	11,342	39,0935	16,9892	308,99		11,342	33,25	10,2467	321,55	cooling

11,344	20,9258	-1,073	307,51	cooling	11,344	38,6375	14,9082	331,7		11,344	33,0735	7,9347	351,4	cooling
11,346	20,6348	0,174	286,01	cooling	11,346	38,3835	16,417	307,06		11,346	33,6469	12,0915	301,31	cooling
11,348	20,6448	1,9645	261,12	cooling	11,348	38,1809	14,1659	335,69		11,348	32,9332	11,4244	300,66	cooling
11,35	20,3204	3,2187	239,06	cooling	11,35	39,0723	18,2152	291,55		11,35	33,5817	13,2995	283,52	cooling
11,352	20,7796	3,2516	245,02		11,352	37,8473	17,5921	283,14		11,352	32,7179	12,6235	280,89	cooling
11,354	20,5493	4,3086	227,02		11,354	38,8416	21,4977	242,44		11,354	33,0688	9,439	330,31	cooling
11,356	20,9525	3,9141	238,17		11,356	38,1191	19,9046	254,61		11,356	32,7839	9,6172	323,84	cooling
11,358	20,7895	1,5881	268,41		11,358	38,1941	13,8981	339,62		11,358	33,3452	9,4816	333,58	cooling
11,36	21,5599	3,4076	253,74		11,36	38,2368	14,4913	331,93		11,36	32,6486	11,1476	300,55	cooling
11,362	20,8595	2,8693	251,48		11,362	39,0979	17,9391	295,77		11,362	32,6946	10,8136	305,86	cooling
11,364	21,3824	3,1355	255,07		11,364	38,058	20,2291	249,22		11,364	32,8752	14,0203	263,56	cooling
11,366	21,1344	2,2419	264,09		11,366	37,909	20,9321	237,31		11,366	33,0524	13,5155	273,1	cooling
11,368	21,0371	-0,6415	303,04		11,368	38,3072	23,0701	212,99		11,368	32,7029	14,9494	248,17	cooling
11,37	21,0848	-1,571	316,7		11,37	38,4135	16,3686	308,16	heating	11,37	32,2804	8,8621	327,35	
11,372	21,1575	1,4613	275,32		11,372	38,3103	16,8665	299,75	heating	11,372	33,1067	8,0598	350,12	
11,374	20,7116	-0,3743	294,75		11,374	37,9761	13,5317	341,7	heating	11,374	32,6547	7,5808	350,5	cooling
11,376	20,7099	1,1829	272,96		11,376	39,7204	18,4179	297,78		11,376	33,1514	13,8132	270,32	cooling
11,378	20,5697	3,0737	244,57		11,378	38,6335	20,6958	250,74		11,378	32,3377	14,7015	246,53	cooling
11,38	20,4038	0,65	276,13	heating	11,38	38,4914	22,5544	222,78	heating	11,38	33,159	15,5484	246,17	
11,382	21,3227	2,2619	266,44	heating	11,382	38,0203	19,8654	253,78	heating	11,382	32,4201	12,2917	281,36	
11,384	20,2631	0,125	281,5	heating	11,384	38,7712	21,7358	238,13	heating	11,384	33,0688	11,8708	296,32	
11,386	20,6117	-3,2896	334,11	heating	11,386	37,0513	16,2552	290,7	heating	11,386	32,2993	11,7783	286,85	
11,388	20,4297	-2,1767	316	heating	11,388	38,2955	19,0269	269,35	heating	11,388	32,5804	11,4637	295,18	
11,39	20,205	-2,1797	312,91		11,39	37,4126	21,2408	226,06	heating	11,39	32,3127	12,1653	281,63	
11,392	20,1555	-2,7225	319,8		11,392	37,4502	18,2098	268,95	heating	11,392	32,8183	12,8278	279,44	
11,394	20,7771	1,976	262,81		11,394	38,3235	20,8225	244,64	heating	11,394	32,3249	13,057	269,34	
11,396	20,1009	-0,5673	288,91		11,396	39,3768	19,47	278,27	heating	11,396	32,436	10,5272	306,25	
11,398	20,6546	1,4216	268,85		11,398	39,4135	18,2118	296,37	heating	11,398	32,9385	13,4817	271,98	
11,4	20,3951	3,4942	236,25	heating	11,4	38,9927	19,1047	278,01	heating	11,4	32,7992	11,4637	298,24	
11,402	20,2879	1,041	269,04	heating	11,402	39,7129	19,8951	277,02	heating	11,402	32,9751	12,4605	286,76	
11,404	20,7135	3,1338	245,74	heating	11,404	39,4474	13,8037	358,46	heating	11,404	32,3124	8,0846	338,67	
11,406	20,0843	6,9376	183,77	heating	11,406	39,727	18,1111	302,16	heating	11,406	33,2341	9,3094	334,43	
11,408	20,2708	4,3959	221,91	heating	11,408	38,4202	13,0022	355,31	heating	11,408	32,6698	10,1714	314,49	
11,41	20,5887	6,9699	190,37	heating	11,41	40,0813	16,9598	323,2	heating	11,41	32,9407	17,6963	213,09	
11,412	20,2797	4,7714	216,78	heating	11,412	38,0535	16,403	302,64	heating	11,412	32,1588	14,8179	242,4	
11,414	20,3706	0,0369	284,23	heating	11,414	38,4261	15,83	315,86	heating	11,414	33,4151	12,3627	294,28	
11,416	21,2886	2,6182	260,98	heating	11,416	38,5006	15,8357	316,82	heating	11,416	32,6195	6,6718	362,71	
11,418	20,2532	-1,1749	299,53	heating	11,418	39,1271	14,4658	344,73	heating	11,418	33,5187	9,1992	339,95	
11,42	20,8876	-2,2268	323,1	heating	11,42	38,4847	12,0955	368,88		11,42	32,8122	11,8878	292,49	
11,422	20,5526	-0,1014	288,71	heating	11,422	39,5012	16,2894	324,47		11,422	33,2151	14,4791	261,9	
11,424	20,3943	-1,51	306,19	heating	11,424	38,4394	15,4513	321,34		11,424	32,5484	15,2174	242,26	
11,426	20,3419	-1,5667	306,25	heating	11,426	38,8814	14,0958	346,47		11,426	33,0855	15,0351	252,32	
11,428	20,1155	0,454	274,84	heating	11,428	39,3604	17,6818	303,03		11,428	32,4126	14,4739	250,76	
11,43	19,9769	-4,4618	341,62	heating	11,43	39,8131	15,6904	337,2		11,43	32,1544	10,1934	306,98	
11,432	20,4769	-2,2172	317,23	heating	11,432	39,7197	21,9483	248,42		11,432	32,5512	11,8153	289,86	
11,434	20,3149	-1,8613	309,99	heating	11,434	38,7527	19,1605	273,87		11,434	32,6155	10,8449	304,32	
11,436	20,3621	-3,727	336,73	heating	11,436	39,9715	17,0113	320,95		11,436	32,6379	16,4247	226,64	
11,438	21,2712	1,0437	282,75	heating	11,438	39,5211	15,3727	337,56		11,438	32,0768	13,77	255,9	heating
11,44	20,5059	1,145	270,64	heating	11,44	39,1951	13,7608	355,53		11,44	33,5243	15,6896	249,3	heating
11,442	21,2152	0,192	293,87	heating	11,442	38,6125	12,7132	362,03		11,442	33,0753	12,7857	283,62	heating
11,444	20,9599	2,1776	262,55	heating	11,444	39,0829	17,093	307,39		11,444	33,4988	15,0494	257,9	heating
11,446	20,7692	-0,7376	300,63	heating	11,446	37,5922	15,2154	312,79		11,446	33,033	12,6264	285,25	heating
11,448	21,0836	-1,1589	310,92	heating	11,448	39,0648	17,772	297,64		11,448	34,0234	13,2373	290,56	heating
11,45	21,3719	3,7913	245,75	heating	11,45	37,9605	17,5494	285,32		11,45	33,2126	9,0966	337,11	heating
11,452	20,966	0,8082	281,78	heating	11,452	38,3236	11,801	370,75		11,452	33,6421	10,2006	327,68	heating
11,454	21,5539	3,1473	257,3	heating	11,454	38,5747	16,1815	313,02		11,454	32,7317	10,0356	317,26	heating

11,456	21,2868	3,4972	248,67	heating	11,456	38,8752	14,0572	346,92		11,456	32,9125	9,9357	321,18	
11,458	21,07	0,117	292,89	heating	11,458	38,3959	13,401	349,39	cooling	11,458	32,4145	12,0919	284,08	
11,46	21,6251	1,5629	280,44	heating	11,46	38,8032	15,9279	319,76	cooling	11,46	32,7595	13,4069	270,52	
11,462	20,8649	-1,2731	309,46	heating	11,462	38,8613	17,151	303,48	cooling	11,462	32,2625	14,7171	245,26	
11,464	21,5334	-4,2172	359,95	heating	11,464	38,5918	15,9495	316,51	cooling	11,464	32,1606	10,374	304,54	heating
11,466	21,4064	0,078	298,14	heating	11,466	38,254	22,1755	224,75	cooling	11,466	32,6724	10,3506	312,03	heating
11,468	21,0921	0,3965	289,29	heating	11,468	37,5151	16,1518	298,63		11,468	32,9431	8,575	340,63	heating
11,47	21,1802	-0,3361	300,77	heating	11,47	38,9403	18,0035	292,67		11,47	32,9625	14,4557	258,7	
11,472	21,3706	1,5903	276,5	heating	11,472	37,8847	21,0051	235,95		11,472	32,5711	14,3839	254,23	
11,474	20,9448	-1,6805	316,27	heating	11,474	38,3105	19,7344	259,67		11,474	33,7141	17,2663	229,91	heating
11,476	21,5818	1,3271	283,13	heating	11,476	38,0029	22,5767	215,64		11,476	33,2861	13,1644	281,27	heating
11,478	21,2884	2,6401	260,68	heating	11,478	37,7512	19,9873	248,31		11,478	33,4584	13,4225	280,07	heating
11,48	21,2775	-0,9261	310,37	heating	11,48	37,1928	13,4096	332,45		11,48	32,7112	12,0414	288,93	heating
11,482	22,0311	-0,3042	312,21	heating	11,482	38,9267	17,1588	304,28		11,482	33,4865	16,1887	241,8	heating
11,484	21,5469	-3,3121	347,49	heating	11,484	37,5589	17,9007	274,79		11,484	32,781	16,2742	230,74	heating
11,486	22,5802	-5,1391	387,47		11,486	38,0798	13,3231	346,06		11,486	33,1324	16,4294	233,48	heating
11,488	22,6322	-2,168	346,67		11,488	38,8474	20,0544	262,7		11,488	32,6393	15,3637	241,49	heating
11,49	22,6347	-2,1358	346,25		11,49	38,8288	16,9737	305,5		11,49	32,9984	11,7962	296,38	heating
11,492	22,6936	-1,3543	336,15		11,492	38,6443	18,3269	284,01		11,492	32,76	10,8873	305,75	heating
11,494	22,8833	1,0946	304,57		11,494	38,7361	18,6275	281,09		11,494	33,2934	11,3897	306,18	heating
11,496	22,3908	-4,2938	373,01		11,496	39,1427	15,0167	337,25		11,496	32,9351	13,5102	271,53	heating
11,498	23,1098	-2,1161	352,62		11,498	38,6297	12,2439	368,83		11,498	33,1774	12,7735	285,22	heating
11,5	22,9608	2,0293	292,59		11,5	38,0828	16,9971	294,75		11,5	33,1976	13,3141	277,94	heating
11,502	22,6806	1,8066	291,79		11,502	37,3257	13,2944	335,92		11,502	33,2634	9,991	325,31	heating
11,504	23,3213	4,5548	262,33		11,504	38,3477	16,6871	302,78		11,504	33,2058	13,6782	272,97	heating
11,506	22,6072	2,0663	287,13		11,506	37,1152	18,6044	258,75		11,506	32,5514	11,2256	298,1	heating
11,508	23,7651	-1,292	350,26		11,508	37,774	16,2188	301,31		11,508	33,3235	11,023	311,73	heating
11,51	23,4664	1,5037	307,01		11,51	37,6331	19,0363	259,96		11,51	32,962	8,9968	335	heating
11,512	23,5046	1,5614	306,73		11,512	37,3576	17,3436	279,77		11,512	33,5937	10,0633	328,92	heating
11,514	23,3041	0,4927	318,87		11,514	36,9192	13,3	330,16		11,514	33,1225	11,5336	301,78	heating
11,516	24,2032	4,5795	274,31		11,516	38,395	15,9086	314,33		11,516	34,1273	16,6431	244,4	heating
11,518	22,9865	-1,129	337,1		11,518	37,3571	18,1207	268,9		11,518	33,3918	16,7727	232,31	heating
11,52	23,791	-0,7511	343,06		11,52	37,3005	13,1611	337,43		11,52	34,0846	16,3329	248,14	heating
11,522	23,019	0,565	313,87		11,522	38,1651	17,826	284,31		11,522	33,2646	13,3887	277,84	heating
11,524	22,4849	-0,1624	316,58		11,524	37,7156	13,171	343,1		11,524	33,8052	9,0077	346,63	heating
11,526	22,3279	1,1737	295,7		11,526	37,6932	14,6558	322,03		11,526	33,4557	9,3826	336,51	heating
11,528	22,4585	3,3555	267,03		11,528	37,6821	16,014	302,89		11,528	33,7334	11,1662	315,46	heating
11,53	22,3478	-2,874	352,56		11,53	38,2196	14,579	330,46		11,53	33,2919	14,378	264,39	heating
11,532	21,8994	-2,7262	344,23		11,532	37,6403	12,4392	352,28		11,532	33,2278	12,7263	286,58	heating
11,534	21,8389	-1,2125	322,22		11,534	37,8821	16,5664	297,96		11,534	33,7654	13,232	287,03	heating
11,536	21,2619	-1,2358	314,48	cooling	11,536	37,5687	14,8512	317,56		11,536	33,7681	9,7031	336,39	heating
11,538	21,5622	0,2354	298,12	cooling	11,538	38,5677	16,6497	306,38		11,538	33,9519	12,1971	304,1	heating
11,54	20,6407	-3,0982	331,83		11,54	37,6565	17,1793	286,24		11,54	33,4992	13,6298	277,74	heating
11,542	22,307	-1,0083	325,91	cooling	11,542	38,633	14,252	340,81		11,542	34,4772	19,1489	214,27	heating
11,544	21,5476	2,1983	270,47	cooling	11,544	38,2637	17,9161	284,43		11,544	33,8409	15,8537	251,44	heating
11,546	21,6462	3,8342	248,99		11,546	37,9169	18,3569	273,42		11,546	34,6188	15,4731	267,63	heating
11,548	21,2489	3,5858	246,9		11,548	38,0155	20,6993	242,05		11,548	33,5246	10,3959	323,3	heating
11,55	21,5884	4,3651	240,76		11,55	38,734	21,3741	242,67		11,55	34,446	8,9424	356,5	heating
11,552	20,9016	-0,1821	294,72		11,552	37,7533	21,8633	222,12		11,552	33,8856	11,6314	311,08	heating
11,554	20,9811	1,5803	271,19		11,554	37,3963	16,95	285,81		11,554	34,0111	14,1641	277,43	heating
11,556	20,6458	0,8571	276,62		11,556	38,2807	19,6212	260,83		11,556	33,5913	14,7787	262,97	heating
11,558	20,3992	2,2129	254,22		11,558	37,3111	14,9591	312,45		11,558	34,0767	15,5622	258,81	heating
11,56	20,4846	3,8635	232,34		11,56	37,3778	18,6011	262,47		11,56	33,7592	13,4679	283,64	heating
11,562	20,0788	-0,0266	281,04		11,562	36,9272	19,9785	236,92		11,562	34,3355	11,1103	324,65	heating
11,564	21,6615	-2,5356	338,24		11,564	37,5732	19,4432	253,43		11,564	33,9614	12,0292	306,58	heating
11,566	20,8478	-1,7557	315,96		11,566	36,6276	15,9389	289,2	heating	11,566	33,4268	6,4177	377,55	heating

11,568	21,3972	-1,053	313,82		11,568	37,3495	17,9685	270,92	heating	11,568	34,2111	10,9241	325,52	
11,57	21,1235	0,173	292,86		11,57	36,8628	17,8089	266,34	heating	11,57	33,5196	14,6431	263,87	
11,572	21,7353	1,7729	279,04		11,572	37,2648	17,3783	277,98	heating	11,572	33,6244	18,0867	217,19	
11,574	20,979	-3,937	348,29		11,574	36,7586	18,1901	259,56	heating	11,574	33,0462	16,8909	225,83	heating
11,576	21,8503	-1,1091	320,94		11,576	37,5129	16,9887	286,9	heating	11,576	33,7322	15,2629	258,17	heating
11,578	21,1227	-2,6609	332,46		11,578	36,9828	18,7682	254,61	heating	11,578	33,4064	9,3826	335,82	heating
11,58	21,0344	-0,7147	304,02		11,58	36,6112	17,6259	265,39	heating	11,58	34,2685	12,939	298,15	heating
11,582	20,7687	1,0912	275,06		11,582	37,0585	18,9197	253,55	heating	11,582	33,3319	13,4694	277,65	heating
11,584	20,4631	-0,895	298,55		11,584	37,6526	16,2107	299,73	heating	11,584	34,3299	14,7963	273,05	heating
11,586	21,6527	-1,3976	322,21		11,586	37,4477	20,4135	238,11	heating	11,586	34,0227	16,3448	247,11	heating
11,588	20,6271	0,5827	280,19		11,588	36,68	16,066	288,15	heating	11,588	34,3661	13,2848	294,69	heating
11,59	20,8112	-0,9944	304,81		11,59	38,3539	19,8977	257,99	heating	11,59	34,067	11,7223	312,35	heating
11,592	20,3889	1,3374	266,31		11,592	37,0728	18,3473	261,76	heating	11,592	34,3475	12,8208	300,91	heating
11,594	20,4917	4,3763	225,27		11,594	37,2568	18,9978	255,23	heating	11,594	34,2103	14,3368	277,8	heating
11,596	19,4021	0,1121	269,65		11,596	36,9408	18,1365	262,86	heating	11,596	34,1612	11,8922	311,29	heating
11,598	20,6457	3,5749	238,63		11,598	37,8708	21,1063	234,34	heating	11,598	34,174	12,4902	303,11	heating
11,6	19,6297	2,3765	241,17		11,6	36,7777	19,1248	246,76	heating	11,6	33,5117	6,3431	379,78	heating
11,602	19,6403	1,5679	252,63	heating	11,602	38,0069	22,1818	221,21	heating	11,602	34,2399	7,1139	379,18	
11,604	19,3799	2,038	242,41	heating	11,604	37,3864	21,3233	224,54	heating	11,604	34,0244	8,8808	351,47	
11,606	19,141	-0,4948	274,48	heating	11,606	37,6077	16,5489	294,37	heating	11,606	34,1805	11,9539	310,7	
11,608	19,7709	-4,3155	336,69	heating	11,608	37,5773	17,8016	276,43	heating	11,608	33,5613	10,4758	322,7	
11,61	19,1178	-0,8121	278,59	heating	11,61	37,7425	17,5264	282,59	heating	11,61	34,5434	13,1717	298,75	
11,612	19,1385	-2,3557	300,46	heating	11,612	37,6968	19,5943	253,05	heating	11,612	33,9097	10,9748	320,6	
11,614	18,6752	-3,97	316,55	heating	11,614	37,1429	18,5075	260,49	heating	11,614	34,5159	13,8777	288,49	
11,616	19,1543	-2,4645	302,2	heating	11,616	37,7736	16,7473	293,92	heating	11,616	33,7021	14,8993	262,84	
11,618	18,2252	-6,1372	340,55	heating	11,618	38,0781	11,016	378,29	heating	11,618	34,2038	12,1363	308,47	
11,62	19,7912	-3,5017	325,6		11,62	38,8468	18,1426	289,41	heating	11,62	33,973	8,7899	352,02	
11,622	19,2632	-0,9751	282,9		11,622	38,1908	15,3025	319,94	heating	11,622	34,5353	8,3967	365,38	
11,624	19,1585	-2,192	298,45		11,624	40,1854	19,5453	288,52	heating	11,624	34,1117	11,0107	322,92	
11,626	19,1983	-4,1811	326,81		11,626	39,1101	17,5601	301,24	heating	11,626	34,185	13,1495	294,04	
11,628	19,7563	-1,5103	297,28		11,628	39,2083	17,2349	307,16	heating	11,628	34,2248	15,6697	259,37	
11,63	19,5485	-3,3891	320,63		11,63	38,6436	16,6026	308,1	heating	11,63	34,3786	13,3	294,65	
11,632	19,2857	0,4831	262,83		11,632	38,8345	19,9817	263,53	heating	11,632	34,3829	14,3969	279,38	
11,634	19,9962	5,4289	203,63		11,634	37,6423	17,7078	278,65	heating	11,634	34,1183	10,7112	327,2	
11,636	19,3314	3,4693	221,73	heating	11,636	38,3195	21,0988	240,72	heating	11,636	34,8123	12,1861	316,28	
11,638	20,1818	3,1472	238,12	heating	11,638	37,729	21,8852	221,47	heating	11,638	34,5101	11,5447	321,02	
11,64	19,6363	1,4291	254,51	heating	11,64	37,7649	17,0944	288,94	heating	11,64	34,5916	13,5573	294,03	
11,642	20,9442	-1,0817	307,89	heating	11,642	38,9295	20,6004	256,21	heating	11,642	33,8945	11,8483	308,17	
11,644	20,5175	2,0098	258,71	heating	11,644	39,0807	16,9756	309	heating	11,644	34,4471	11,8188	316,31	
11,646	20,4975	4,4656	224,1	heating	11,646	39,5173	17,7799	303,86	heating	11,646	34,0168	12,3575	302,77	
11,648	20,5407	-0,2819	291,07	heating	11,648	39,2927	16,8787	313,31	heating	11,648	34,6495	14,9956	274,73	
11,65	21,3624	0,7577	288,02	heating	11,65	40,3064	17,9162	312,98	heating	11,65	33,8219	15,7976	251,95	
11,652	20,3488	-2,8574	324,39	heating	11,652	40,1828	12,3937	388,45	heating	11,652	34,3724	15,0525	270,06	
11,654	20,091	-3,6378	331,69	heating	11,654	40,7187	20,7767	278,76	heating	11,654	34,0704	13,7961	283,41	
11,656	20,1461	1,11	266,1	heating	11,656	39,8243	19,4417	284,92	heating	11,656	35,0392	10,618	341,37	
11,658	18,8001	0,7318	252,57	heating	11,658	41,0886	22,6885	257,21	heating	11,658	34,3167	9,8744	341,67	
11,66	18,8752	-2,8923	304,28	heating	11,66	39,8583	19,7332	281,32	heating	11,66	34,3536	6,767	385,62	
11,662	19,2312	0,3713	263,63	heating	11,662	39,7419	13,811	362,48	heating	11,662	34,535	9,9615	343,5	
11,664	19,6852	-3,2169	320,14	heating	11,664	39,8363	11,4974	396,14	heating	11,664	34,5373	11,2831	325,06	
11,666	19,1476	-3,9758	323,23	heating	11,666	39,6494	15,8254	333,02		11,666	34,4927	14,3592	281,44	
11,668	20,0673	1,5647	258,64	heating	11,668	38,5404	14,4295	337,04		11,668	34,2422	15,1914	266,3	
11,67	19,4111	-2,03	299,71	heating	11,67	39,2231	16,4766	317,96		11,67	34,6897	13,3214	298,7	
11,672	20,5811	-0,8385	299,41	heating	11,672	38,3909	18,9166	272,22		11,672	34,604	9,4041	352,26	
11,674	19,839	0,8303	265,71	heating	11,674	37,8962	11,4097	370,24		11,674	34,9086	11,6805	324,69	
11,676	20,6472	-2,3683	321,72	heating	11,676	39,1487	18,5234	288,31		11,676	33,7949	9,3247	342,06	
11,678	20,9048	0,5535	284,48	heating	11,678	38,9312	17,8292	294,97		11,678	35,3018	12,8151	314,33	

11,68	20,9066	3,845	238,5	heating	11,68	39,1076	19,1377	279,15		11,68	34,5708	16,0723	258,58	
11,682	20,8537	-1,2221	308,59	heating	11,682	39,2358	21,0899	253,65		11,682	34,9115	16,1701	261,98	
11,684	22,5181	4,77	248,09	heating	11,684	40,1714	23,5093	232,91		11,684	34,3269	17,8508	230,31	
11,686	21,5224	2,0389	272,35	heating	11,686	40,0426	18,2508	304,62		11,686	34,732	16,3628	256,77	
11,688	21,74	-1,3175	322,31	heating	11,688	40,6916	25,0081	219,23		11,688	34,1929	10,5608	330,34	
11,69	22,543	3,7541	262,64	heating	11,69	39,4184	17,8123	302,02	heating	11,69	35,0266	12,862	309,83	
11,692	21,4913	-0,5581	308,22	heating	11,692	40,7845	17,2658	328,76	heating	11,692	33,8107	12,4732	298,27	
11,694	22,064	-5,4907	385,17	heating	11,694	39,0445	15,857	324,13		11,694	34,1834	9,3082	347,72	
11,696	22,7786	2,416	284,64	heating	11,696	39,3044	14,0476	353,05		11,696	34,2822	10,6559	330,26	
11,698	22,9416	0,7995	309,51	heating	11,698	39,0868	17,067	307,8		11,698	33,8455	6,3982	383,67	
11,7	22,7092	2,1565	287,3	heating	11,7	39,4774	21,0581	257,48		11,7	34,6961	8,4365	367,07	
11,702	22,6645	5,2892	242,88	heating	11,702	38,8327	16,9954	305,25		11,702	33,9157	13,1801	289,85	cooling
11,704	21,5507	-3,3037	347,43	heating	11,704	40,4623	17,1794	325,46		11,704	34,1832	12,5428	302,5	cooling
11,706	22,7767	-2,5216	353,63	heating	11,706	39,3396	15,2647	336,53		11,706	34,577	11,0477	328,91	cooling
11,708	21,4212	-1,2409	316,78		11,708	39,8261	12,7576	378,38		11,708	34,7675	12,0743	317,22	cooling
11,71	21,892	-6,1975	392,65		11,71	40,3753	21,774	260,02		11,71	33,7924	6,7599	377,87	cooling
11,712	22,0475	-0,4269	314,16		11,712	40,1103	20,1473	279,05		11,712	35,0469	10,9895	336,29	cooling
11,714	21,4408	2,4526	265,43		11,714	40,0028	21,2636	261,95		11,714	34,4873	12,2492	310,86	cooling
11,716	21,1265	-2,6884	332,9		11,716	39,4082	20,1582	269,09		11,716	34,4013	9,7953	343,96	cooling
11,718	22,7725	3,7736	265,58		11,718	39,9852	18,8432	295,53		11,718	34,2926	12,7825	300,68	cooling
11,72	21,8332	-0,2304	308,42		11,72	39,6552	15,6929	334,96		11,72	34,099	12,504	301,87	cooling
11,722	22,3428	-0,5488	319,99		11,722	39,662	20,3062	270,57		11,722	34,241	10,8392	327,12	cooling
11,724	22,5089	5,7134	234,78		11,724	38,8372	14,013	347,01		11,724	34,0548	12,2865	304,29	cooling
11,726	21,8578	3,26	259,97		11,726	40,1902	16,0762	337,08		11,726	33,9673	13,5835	284,94	cooling
11,728	22,15	0,7351	299,35		11,728	38,4545	13,4651	349,31		11,728	33,9675	12,7783	296,19	cooling
11,73	22,4312	5,2598	240,03		11,73	39,3018	13,7651	356,97		11,73	34,1254	15,6222	258,65	cooling
11,732	22,7541	-0,2401	321,43		11,732	38,7294	14,7745	334,85		11,732	33,5968	11,9389	302,74	cooling
11,734	22,5063	2,2496	283,16		11,734	38,918	14,2745	344,48		11,734	34,1952	12,314	305,87	cooling
11,736	22,1155	1,76	284,54		11,736	38,5641	13,1732	354,93		11,736	33,6457	12,3626	297,51	cooling
11,738	21,7268	-5,3023	377,83		11,738	39,7203	14,6593	350,31	cooling	11,738	33,9949	14,3551	274,54	cooling
11,74	23,5253	0,3143	324,45		11,74	39,1553	17,0816	308,56	cooling	11,74	34,2256	14,2249	279,58	
11,742	22,0751	1,0778	293,51		11,742	39,2177	15,225	335,38	cooling	11,742	34,2918	14,9455	270,43	
11,744	23,3871	-0,6989	336,69		11,744	40,1419	17,3448	318,67	cooling	11,744	33,7746	13,9846	276,63	
11,746	23,2518	2,245	293,64		11,746	39,9875	12,4789	384,53	cooling	11,746	34,334	16,7306	246,07	
11,748	23,1436	-0,5037	330,55		11,748	39,4647	12,9144	371,13	cooling	11,748	34,2898	17,3457	236,85	
11,75	23,212	-2,4307	358,45		11,75	38,6398	9,5	407,33	cooling	11,75	34,1353	15,2567	263,9	
11,752	24,9043	5,9825	264,5		11,752	39,6139	14,9885	344,23	cooling	11,752	33,8277	13,1688	288,78	
11,754	23,9133	-0,105	335,74		11,754	38,1873	12,5746	358,03	cooling	11,754	34,0852	9,2992	346,47	
11,756	24,4107	1,2615	323,59		11,756	38,5786	15,8969	317,06	cooling	11,756	33,9669	7,9798	363,26	
11,758	23,7118	3,7362	279,23		11,758	38,0692	14,7939	325,35	cooling	11,758	33,7014	9,8819	332,96	
11,76	23,1787	-0,3042	328,26		11,76	38,6338	14,6703	334,97	cooling	11,76	33,5499	13,9233	274,35	
11,762	23,4345	2,0686	298,66		11,762	37,7737	14,8646	320,23	cooling	11,762	33,4855	14,7229	262,27	
11,764	22,8167	5,6593	239,83		11,764	38,6169	18,1328	286,34	cooling	11,764	33,2135	13,7215	272,47	
11,766	23,5403	1,2987	310,91		11,766	38,3133	16,7433	301,52	cooling	11,766	33,4608	9,0543	341,17	
11,768	23,0772	4,0665	265,74		11,768	39,1158	18,5161	287,95	cooling	11,768	33,5889	7,7712	360,89	
11,77	22,6529	1,7443	292,27		11,77	38,5812	18,8037	276,46	cooling	11,77	33,1781	7,0075	365,83	
11,772	22,4654	-2,7064	351,86		11,772	39,6959	16,0298	330,82	cooling	11,772	33,6577	11,4976	309,77	
11,774	23,6192	2,4579	295,8		11,774	38,9411	18,2919	288,64	cooling	11,774	34,1124	15,4233	261,25	
11,776	22,2392	2,6528	273,79		11,776	38,1281	12,8627	353,17	cooling	11,776	34,0275	15,1264	264,21	
11,778	23,0296	4,3084	261,69		11,778	39,0476	16,3595	317,15	cooling	11,778	33,8796	13,1821	289,32	
11,78	22,2059	4,53	247,08		11,78	39,0585	17,5098	301,22	cooling	11,78	34,6569	14,9039	276,12	
11,782	22,0508	0,5841	300,07		11,782	38,0209	17,6519	284,73	cooling	11,782	34,942	14,9276	279,77	
11,784	21,8576	0,8464	293,7		11,784	38,0704	18,8361	268,87	cooling	11,784	34,5859	13,9267	288,79	
11,786	22,5337	6,1701	228,74		11,786	38,5956	23,9756	204,37	cooling	11,786	34,2301	12,205	307,88	
11,788	21,7795	3,0092	262,38		11,788	37,6204	18,9536	260,93	cooling	11,788	34,5919	10,2475	340,3	
11,79	21,9898	6,6477	214,46		11,79	38,4488	22,7466	219,49		11,79	34,091	10,4029	331,12	

11,792	21,6298	7,7545	193,96	heating	11,792	37,895	19,4698	257,56		11,792	33,6703	11,6273	308,13
11,794	21,9278	4,7051	240,75	heating	11,794	39,56	19,1707	285,01		11,794	33,5011	10,4122	322,75
11,796	22,2812	2,5777	275,43	heating	11,796	38,6743	22,6246	224,35		11,796	33,7306	10,8058	320,45
11,798	22,096	-0,3376	313,59	heating	11,798	39,3274	23,1736	225,81		11,798	33,0557	10,7913	311,22
11,8	23,3647	-0,5615	334,45	heating	11,8	38,7904	21,7949	237,57		11,8	33,5573	11,0796	314,2
11,802	22,8025	3,8679	264,68	heating	11,802	38,9978	21,1905	248,92		11,802	33,7611	10,2853	328,16
11,804	22,6029	1,9968	288,04	heating	11,804	38,1924	18,1729	279,84		11,804	33,3935	9,5326	333,54
11,806	22,2395	-1,2248	328	heating	11,806	39,1951	18,004	296,22		11,806	33,6527	10,5069	323,54
11,808	22,9965	0,5834	313,3	heating	11,808	37,9379	19,9399	251,58		11,808	34,1949	13,0306	295,85
11,81	22,0811	-1,0492	323,33	heating	11,81	37,7352	15,6704	308,43	heating	11,81	33,9286	13,3178	288,11
11,812	22,9235	2,6153	283,88	heating	11,812	38,7601	20,3321	257,6	heating	11,812	33,5995	14,9711	260,4
11,814	22,7438	2,8028	278,75	heating	11,814	38,3782	18,8999	272,28	heating	11,814	34,1153	14,0629	280,3
11,816	23,5211	0,8583	316,79	heating	11,816	38,5206	19,122	271,16	heating	11,816	34,2864	10,4561	333,11
11,818	23,8954	2,0493	305,38	heating	11,818	38,2451	18,2285	279,8	heating	11,818	34,1563	9,9075	338,96
11,82	24,4668	4,5416	278,52	heating	11,82	38,9696	18,6115	284,58	heating	11,82	33,7248	12,0395	303,13
11,822	25,3537	0,7859	343,42	heating	11,822	38,1329	15,7469	312,92	heating	11,822	34,1009	15,0391	266,46
11,824	25,2586	2,5933	316,83		11,824	39,1182	19,2535	277,68	heating	11,824	33,6333	15,0756	259,41
11,826	25,1841	3,6263	301,35		11,826	38,4318	16,4243	307,63	heating	11,826	33,1399	10,7316	313,24
11,828	24,9681	5,0881	277,89		11,828	39,4645	16,5016	320,99	heating	11,828	33,3779	7,8512	356,82
11,83	25,8581	10,0278	221,28		11,83	38,3445	16,7509	301,85	heating	11,83	33,0583	9,1066	334,81
11,832	24,3114	2,9741	298,26	heating	11,832	39,3614	15,4308	334,51	heating	11,832	32,7526	11,0162	303,84
11,834	25,0946	2,5594	315,01	heating	11,834	38,57	14,9712	329,88		11,834	32,9573	13,3153	274,57
11,836	24,4518	5,9336	258,86		11,836	38,5657	14,2518	339,87		11,836	33,3855	14,2787	267,09
11,838	24,1432	4,6078	273,08	heating	11,838	38,6114	17,7865	291,1		11,838	33,0342	14,3122	261,71
11,84	24,2315	3,858	284,79	heating	11,84	38,916	19,4281	272,41		11,84	33,6085	16,5878	237,92
11,842	24,4402	4,5115	278,57	heating	11,842	37,9758	19,907	252,58		11,842	33,9385	17,8651	224,68
11,844	24,7339	-2,8228	385,2	heating	11,844	37,9822	16,3177	302,84		11,844	34,0051	15,8873	253,26
11,846	24,7167	1,2996	327,34		11,846	38,8975	17,9977	292,15		11,846	33,4913	13,8311	274,82
11,848	24,3072	2,3823	306,48		11,848	38,3863	15,7209	316,83		11,848	33,7529	11,2113	315,1
11,85	23,958	-1,704	358,72		11,85	38,7534	19,6588	266,92		11,85	33,8526	10,2117	330,46
11,852	25,1469	4,074	294,57		11,852	38,1666	18,6398	272,96		11,852	33,5989	11,5122	308,74
11,854	23,605	2,1507	299,9		11,854	38,8577	18,7354	281,28		11,854	33,4522	13,6508	276,8
11,856	24,9274	-0,5207	355,73		11,856	37,7708	16,6171	295,7		11,856	33,5587	15,0216	259,12
11,858	24,3673	1,3448	321,82		11,858	38,5752	15,3174	325,11		11,858	33,5087	13,6874	277,07
11,86	23,9113	-3,8858	388,56		11,86	38,1213	13,6718	341,77		11,86	33,0991	7,0555	364,05
11,862	24,0638	-6,0515	420,97		11,862	38,501	15,4065	322,83		11,862	33,8321	6,3189	384,59
11,864	25,3648	4,8718	286,46		11,864	38,0788	16,3073	304,33		11,864	33,4017	7,7896	358,02
11,866	23,8469	-2,4348	367,38		11,866	38,9495	18,112	291,28		11,866	33,419	11,338	308,66
11,868	24,5585	-2,825	382,78	cooling	11,868	38,3281	21,52	234,95		11,868	33,4473	14,786	260,86
11,87	24,2201	0,7555	328	cooling	11,87	38,1598	19,8877	255,42		11,87	33,6692	13,9711	275,35
11,872	23,9442	0,0589	333,88	cooling	11,872	39,3656	24,3276	210,21		11,872	33,2682	9,8958	326,71
11,874	24,7479	3,8283	292,42	cooling	11,874	39,0166	21,5896	243,6	heating	11,874	33,7125	11,3775	312,21
11,876	24,2701	5,3808	264,04	cooling	11,876	39,0122	19,3378	275,02	heating	11,876	33,8724	12,3213	301,25
11,878	25,5454	1,7554	332,55	cooling	11,878	38,7162	16,4117	311,78	heating	11,878	33,7628	11,2955	314,06
11,88	25,7113	9,5126	226,43	cooling	11,88	39,3247	17,7795	301,17	heating	11,88	33,5022	11,925	301,62
11,882	25,2871	9,6631	218,4	cooling	11,882	38,4266	18,0258	285,17	heating	11,882	33,7425	12,746	293,5
11,884	25,1541	3,2185	306,63		11,884	38,847	23,776	210,67	heating	11,884	34,092	14,9027	268,24
11,886	26,4996	6,3252	282,01		11,886	38,0126	20,0842	250,61	heating	11,886	33,2653	14,2839	265,33
11,888	24,799	0,2235	343,53		11,888	38,5192	19,3706	267,67	heating	11,888	33,1619	12,0812	294,68
11,89	25,6898	2,088	329,92		11,89	37,6083	17,2325	284,82	heating	11,89	33,1624	11,5928	301,51
11,892	24,9643	6,0907	263,83		11,892	38,5103	15,1249	326,89	heating	11,892	32,8771	12,2263	288,67
11,894	24,1406	0,7799	326,55		11,894	38,5416	19,2532	269,62	heating	11,894	32,6934	10,5492	309,54
11,896	24,5034	0,9262	329,57		11,896	38,3344	21,375	237,07	heating	11,896	33,3868	12,0611	298,1
11,898	24,3625	5,3232	266,14		11,898	39,1714	22,9362	226,94	heating	11,898	33,1135	10,903	310,47
11,9	24,736	-4,0974	403,05		11,9	39,7961	22,8083	237,46	heating	11,9	33,1667	10,2411	320,47
11,902	24,6486	-3,3715	391,68	cooling	11,902	39,0461	20,2263	263,07	heating	11,902	33,8682	12,1474	303,63

11,904	24,4543	-0,8196	353,29	cooling	11,904	39,0239	14,9729	336,2	heating	11,904	33,4628	9,5325	334,51	
11,906	24,1533	0,5742	329,6	cooling	11,906	40,1254	18,7065	299,4	heating	11,906	33,4593	9,0127	341,73	
11,908	25,1209	7,4635	246,82	cooling	11,908	39,2038	14,3382	347,58	heating	11,908	33,3487	10,9297	313,38	
11,91	23,3875	1,588	304,72	cooling	11,91	39,4056	15,5737	333,14	heating	11,91	34,0889	12,3501	303,88	
11,912	24,698	-3,4321	393,22	cooling	11,912	39,2442	14,8001	341,69	heating	11,912	33,6934	12,7389	292,91	
11,914	24,1818	2,913	297,31	cooling	11,914	39,8659	14,8913	349,11	heating	11,914	33,5761	14,262	269,98	
11,916	23,6167	4,8538	262,28	cooling	11,916	39,0715	13,4654	357,94	heating	11,916	33,8196	13,1357	289,13	
11,918	23,3167	3,8413	272,24	cooling	11,918	39,5773	16,7229	319,47		11,918	34,036	13,8799	281,75	
11,92	24,0964	7,3926	233,49	cooling	11,92	39,1956	14,7357	341,91		11,92	33,1262	10,5821	315,13	
11,922	23,0134	-0,7452	332,11		11,922	39,2706	13,5883	359		11,922	33,3729	8,6098	346,15	
11,924	23,8174	2,8277	293,41		11,924	38,9421	14,7246	338,53		11,924	33,5722	11,0595	314,69	
11,926	23,2962	6,6166	233,16		11,926	39,7123	13,1303	371,58		11,926	33,1449	14,6503	258,53	
11,928	22,8892	0,8526	308,04		11,928	39,1676	16,5163	316,63		11,928	33,274	15,9621	242	
11,93	23,784	1,9698	304,93		11,93	38,7307	16,8662	305,63		11,93	33,4489	15,5849	249,71	
11,932	23,4534	5,471	251,37		11,932	39,5006	17,8462	302,7		11,932	33,4616	11,507	306,89	
11,934	23,8646	-0,1581	335,8		11,934	40,1014	14,9162	352,05		11,934	33,5964	11,9458	302,64	
11,936	23,7959	3,7467	280,26		11,936	39,6635	16,7499	320,3		11,936	33,9546	15,3589	259,94	
11,938	23,2841	4,2804	265,64		11,938	39,5043	12,3669	379,34		11,938	33,7006	15,6125	252,84	
11,94	22,9092	0,8155	308,84		11,94	40,5022	14,5172	363,23	cooling	11,94	33,4969	16,8068	233,3	
11,942	23,9816	6,2383	248,03		11,942	39,5353	12,8947	372,4	cooling	11,942	33,4505	16,1071	242,44	
11,944	22,7119	3,9434	262,36		11,944	39,4242	14,2657	351,68	cooling	11,944	34,1776	16,2129	251,12	heating
11,946	23,7084	0,1896	328,76		11,946	39,3981	15,2918	336,97	cooling	11,946	34,1468	17,0158	239,47	heating
11,948	23,5042	2,0411	300,02		11,948	39,6597	16,5595	322,91	cooling	11,948	33,4783	16,5003	237,33	heating
11,95	23,2775	0,7929	314,3		11,95	38,7573	15,0094	331,96	cooling	11,95	34,211	16,5986	246,2	heating
11,952	23,7708	2,6804	294,81		11,952	39,4349	18,7532	289,1	cooling	11,952	34,4271	17,3773	238,33	heating
11,954	24,0551	5,7232	256,25		11,954	38,6505	17,5308	295,22	cooling	11,954	34,1882	13,7521	285,67	heating
11,956	23,807	-0,8777	345,06		11,956	38,7686	14,3211	341,74	cooling	11,956	34,1712	11,7283	313,72	heating
11,958	24,011	1,5261	314,31		11,958	38,7562	18,2126	287,17	cooling	11,958	34,1514	10,3566	332,62	heating
11,96	23,5757	4,6365	264,74		11,96	38,8658	16,88	307,33	cooling	11,96	33,7181	10,223	328,43	heating
11,962	23,0964	0,6293	314,06		11,962	38,5783	16,3208	311,13	cooling	11,962	33,7339	12,6997	294,03	heating
11,964	23,7383	0,6949	322,11		11,964	38,2629	14,2323	335,91	cooling	11,964	33,7033	11,064	316,46	heating
11,966	23,299	0,1956	322,95		11,966	38,8009	13,9421	347,49	cooling	11,966	33,8146	7,8086	363,52	heating
11,968	24,0269	0,2793	331,96		11,968	38,6162	11,1338	384,16	cooling	11,968	33,9549	9,9968	334,9	
11,97	23,5436	5,5546	251,46		11,97	38,5471	16,1559	313	cooling	11,97	33,8144	11,7943	307,81	
11,972	22,8548	1,3207	301,02		11,972	37,9534	15,914	308,08	cooling	11,972	34,3277	14,245	280,73	
11,974	23,1359	-3,4157	371,15		11,974	38,7455	17,3267	299,4	cooling	11,974	33,6437	14,5819	266,46	
11,976	23,9103	1,1973	317,49		11,976	37,6438	14,7602	319,88	cooling	11,976	33,9486	12,0426	306,21	
11,978	23,2486	-0,4935	331,88		11,978	37,6596	16,3495	297,88	cooling	11,978	34,6989	14,1253	287,59	
11,98	23,7179	-1,4043	351,17		11,98	36,8957	14,7308	309,83	cooling	11,98	34,147	12,8089	298,28	
11,982	23,419	0,2311	324,13		11,982	37,177	15,5836	301,84	cooling	11,982	33,5414	9,922	330,16	
11,984	23,0591	-0,9503	335,62		11,984	36,3126	16,3082	279,63	cooling	11,984	34,1249	12,9444	296,07	
11,986	23,2587	0,289	321,08		11,986	36,8335	15,8759	292,96	cooling	11,986	33,8997	14,0782	277,07	
11,988	23,5289	1,7114	304,98	cooling	11,988	36,9309	17,7963	267,47	cooling	11,988	33,7511	13,1893	287,42	
11,99	23,3553	-3,2302	371,63	cooling	11,99	36,9187	16,7257	282,27	cooling	11,99	33,9944	14,7119	269,54	
11,992	23,286	-2,1098	355	cooling	11,992	37,3695	13,6697	331,29	cooling	11,992	33,8388	10,3482	328,36	
11,994	23,0146	0,2283	318,52	cooling	11,994	38,1508	14,1237	335,86	cooling	11,994	34,1179	10,4095	331,41	
11,996	22,9452	-0,0246	321,08	cooling	11,996	37,6573	18,8195	263,32	cooling	11,996	33,774	14,2474	272,95	
11,998	23,3098	1,691	302,2	cooling	11,998	36,951	14,0986	319,44	cooling	11,998	33,9045	13,8689	280,07	
12	22,9358	0,4311	314,58	cooling	12	38,2608	17,7192	287,14	cooling	12	33,9227	13,8416	280,7	
12,002	23,6938	1,4879	310,41	cooling	12,002	36,9409	15,6405	297,75	cooling	12,002	33,4251	13,089	284,27	
12,004	23,7926	7,6622	225,48	cooling	12,004	37,04	16,0303	293,68	cooling	12,004	34,1128	13,9862	281,34	
12,006	23,5695	5,1685	257,22	cooling	12,006	36,8141	18,4735	256,37		12,006	34,2663	16,6004	246,94	
12,008	23,6874	-1,8875	357,5		12,008	36,8042	17,821	265,36		12,008	33,5878	14,0239	273,47	heating
12,01	24,8757	0,089	346,48	cooling	12,01	35,9263	12,3151	330,05		12,01	33,9041	10,8496	322,27	heating
12,012	23,9792	1,0564	320,43	cooling	12,012	37,2719	16,6821	287,81		12,012	34,669	12,3555	311,91	
12,014	24,166	3,9869	282,07	cooling	12,014	36,3798	16,1927	282,19		12,014	34,2327	11,4423	318,58	

12,016	23,8197	4,8057	265,79	cooling	12,016	36,7052	15,8756	291,17	
12,018	23,8704	-1,8173	359,08		12,018	36,6915	19,4171	241,47	
12,02	24,409	-4,1124	398,69	cooling	12,02	37,1644	16,3807	290,52	
12,022	24,6409	0,2723	340,64	cooling	12,022	37,5982	18,0696	272,98	
12,024	25,0634	1,0329	335,91	cooling	12,024	37,3879	16,9925	285,1	
12,026	24,9208	3,381	301,09	cooling	12,026	39,075	16,2756	318,7	
12,028	24,8254	3,2611	301,44	cooling	12,028	39,8661	18,3826	300,31	
12,03	24,8044	0,5327	339,28	cooling	12,03	40,0069	23,5918	229,46	
12,032	25,693	1,3006	340,97	cooling	12,032	39,4116	18,4951	292,38	
12,034	25,0697	2,4071	316,79	cooling	12,034	40,8109	22,5704	254,97	
12,036	25,4721	2,8727	315,91	cooling	12,036	38,1421	17,8383	283,82	
12,038	25,2711	5,3379	278,64	cooling	12,038	38,5658	17,4245	295,52	
12,04	25,2991	4,6373	288,82	cooling	12,04	37,2142	17,9372	269,46	
12,042	24,8646	-1,0941	362,86	cooling	12,042	37,1109	13,9482	323,78	
12,044	25,9142	-0,3881	367,67	cooling	12,044	36,286	10,6359	358,55	
12,046	25,6528	4,507	295,59	cooling	12,046	37,9923	14,6082	326,88	
12,048	25,7252	10,8136	208,44	cooling	12,048	37,2609	13,7434	328,74	
12,05	25,2927	10,453	207,44	cooling	12,05	38,3013	16,0226	311,42	
12,052	25,5241	3,6178	306,22		12,052	38,6076	21,2372	242,81	
12,054	25,6536	-3,3013	404,75		12,054	39,0855	17,9185	295,88	
12,056	25,4493	-3,144	399,69	cooling	12,056	39,0288	19,4518	273,66	
12,058	25,3958	2,6624	317,78	cooling	12,058	38,1117	17,7846	284,14	
12,06	25,0993	7,0127	252,82	cooling	12,06	39,0094	17,6767	298,2	
12,062	25,0827	6,82	255,29	cooling	12,062	38,0877	16,7986	297,59	
12,064	24,8588	2,4187	313,68	cooling	12,064	37,9181	20,5237	243,15	
12,066	25,2218	-0,4639	359,05	cooling	12,066	37,3303	18,6034	261,77	
12,068	25,0084	0,0325	349,13	cooling	12,068	38,7185	19,8176	264,21	
12,07	24,6016	1,807	318,64	cooling	12,07	37,5739	17,0753	286,54	
12,072	24,2755	2,302	307,16	cooling	12,072	38,7657	19,586	268,1	
12,074	24,4963	4,1685	284,15	cooling	12,074	38,1417	19,2776	263,69	heating
12,076	24,6428	5,0923	273,29	cooling	12,076	38,4341	17,4227	293,71	heating
12,078	23,9625	2,0173	306,76	cooling	12,078	38,0197	18,9775	266,18	heating
12,08	24,0531	-0,6348	345,1	cooling	12,08	38,9465	20,6482	255,78	heating
12,082	23,8336	-1,7382	357,46	cooling	12,082	38,5456	22,4745	224,65	heating
12,084	23,9421	-1,0962	350	cooling	12,084	38,515	20,502	251,8	heating
12,086	23,7951	-0,65	341,71	cooling	12,086	39,5537	21,7224	249,26	heating
12,088	24,3598	0,8849	328,15	cooling	12,088	39,7664	18,9909	290,41	heating
12,09	23,8534	0,0991	332,05	cooling	12,09	39,4704	21,8752	245,95	heating
12,092	23,0707	1,1622	306,25	cooling	12,092	38,8699	19,9426	264,58	heating
12,094	22,3652	0,5879	304,41	cooling	12,094	40,2762	22,9434	242,29	heating
12,096	22,1052	-1,3421	327,76	cooling	12,096	38,8528	20,0723	262,52	heating
12,098	21,7787	-2,9519	345,7	cooling	12,098	39,3077	22,6325	233,09	heating
12,1	21,6119	-0,3036	306,35	cooling	12,1	38,694	22,1423	231,37	heating
12,102	21,4982	-0,2461	303,95	cooling	12,102	39,6836	22,2294	243,98	heating
12,104	21,2801	-1,3486	316,32	cooling	12,104	38,7533	20,506	255,07	heating
12,106	21,0946	-4,456	357,16	cooling	12,106	39,9018	21,0694	263,25	heating
12,108	21,4942	-4,4147	362,17	cooling	12,108	39,4448	20,5688	263,86	heating
12,11	22,3092	-0,2305	315,07	cooling	12,11	39,5695	21,0511	258,86	heating
12,112	22,3395	1,3061	294,02	cooling	12,112	39,6189	22,2256	243,13	heating
12,114	22,2851	1,8244	286,01	cooling	12,114	40,208	19,562	288,6	heating
12,116	22,4626	0,0681	313,04	cooling	12,116	40,2943	24,7114	217,83	heating
12,118	22,2919	-1,9718	339,17	cooling	12,118	39,0362	20,9707	252,53	heating
12,12	22,4755	0,6027	305,75	cooling	12,12	40,9142	22,5306	256,98	heating
12,122	22,1754	2,403	276,39	cooling	12,122	40,2368	19,5342	289,39	heating
12,124	22,5369	3,2872	269,08	cooling	12,124	40,2019	20,9948	268,49	heating
12,126	22,044	6,6097	215,75	cooling	12,126	39,2503	18,5018	290,03	heating

12,016	33,8508	13,9259	278,52	
12,018	33,9138	14,4166	272,54	
12,02	33,9054	10,5269	326,8	
12,022	33,9248	11,1136	318,87	
12,024	33,6384	11,3664	311,33	
12,026	34,2242	11,5957	316,31	
12,028	34,0518	13,4172	288,44	
12,03	33,6979	14,9653	261,85	
12,032	33,9613	15,3069	260,76	
12,034	33,7006	14,6863	265,79	
12,036	33,5476	11,1503	313,08	
12,038	34,1473	12,3692	304,42	
12,04	34,0917	12,6461	299,78	
12,042	33,546	12,8018	289,97	
12,044	34,0246	14,9755	266,28	
12,046	34,5566	15,8234	261,86	
12,048	34,5258	16,4913	252,1	heating
12,05	34,164	15,6903	258,24	heating
12,052	34,5481	14,2114	284,28	heating
12,054	34,8998	12,8331	308,46	heating
12,056	34,1017	11,6813	313,4	heating
12,058	34,5711	11,9865	315,7	heating
12,06	34,3751	13,0697	297,82	heating
12,062	34,0827	11,9723	309,07	
12,064	33,7886	12,3477	299,71	
12,066	34,0743	9,7748	339,67	
12,068	34,0927	7,5246	371,38	
12,07	33,9071	9,1741	345,73	
12,072	34,5044	12,1242	312,84	
12,074	34,562	14,9444	274,23	
12,076	34,0771	16,0139	252,5	
12,078	34,0478	13,8766	281,96	
12,08	35,2834	14,8034	286,28	
12,082	34,3805	12,3344	308,17	
12,084	34,3961	11,7106	317,11	
12,086	34,5307	13,165	298,66	
12,088	34,5415	13,2565	297,53	
12,09	33,8487	14,1462	275,41	
12,092	33,8712	12,364	300,64	
12,094	34,2996	9,0024	353,62	
12,096	33,9931	7,8619	365,27	
12,098	33,9396	10,6373	325,73	
12,1	34,0912	10,0403	336,2	
12,102	34,0308	11,5532	314,2	
12,104	33,1275	9,5019	330,25	
12,106	34,6266	11,9382	317,15	
12,108	34,2029	11,4411	318,18	
12,11	33,8047	9,443	340,54	
12,112	33,951	9,8575	336,79	
12,114	34,905	12,6786	310,69	
12,116	34,0211	12,4318	301,79	
12,118	34,1511	13,2365	292,36	
12,12	34,4273	12,8965	300,97	
12,122	34,6101	10,2071	341,12	
12,124	34,1171	11,5235	315,83	
12,126	34,1318	9,7172	341,28	cooling

12,128	21,765	4,1274	246,55	cooling
12,13	22,1103	-1,0539	323,8	
12,132	22,7698	-1,2429	335,66	
12,134	22,2582	-1,1493	327,2	cooling
12,136	22,743	2,5029	282,93	cooling
12,138	22,6387	5,2256	243,41	cooling
12,14	22,2288	0,2859	306,73	
12,142	22,7227	-0,7448	328,04	
12,144	23,2697	2,4957	290,39	
12,146	22,9651	0,5472	313,37	
12,148	22,6024	1,2608	298,32	
12,15	22,3405	0,7705	301,52	
12,152	22,5094	-0,3226	319,16	
12,154	22,8106	2,0884	289,67	
12,156	22,5186	1,5027	293,77	
12,158	23,7689	-1,2723	350,04	
12,16	24,2178	1,2267	321,38	
12,162	24,4389	1,4581	321,24	
12,164	24,6956	1,9078	318,54	
12,166	25,6576	6,1047	273,32	cooling
12,168	24,5635	5,941	260,32	
12,17	25,0325	7,8208	240,59	
12,172	24,9603	7,656	241,89	
12,174	25,315	3,1733	309,51	
12,176	25,08	1,8895	324,17	
12,178	25,5032	5,5628	278,74	
12,18	25,6148	3,6349	307,25	
12,182	25,5969	4,7452	291,48	
12,184	25,47	1,6184	333,41	
12,186	26,041	0,2373	360,7	
12,188	26,672	4,0869	315,71	
12,19	25,7506	5,5868	281,86	
12,192	26,7603	3,0373	331,61	
12,194	26,456	3,4113	322,13	
12,196	27,0301	0,8575	365,85	
12,198	26,7463	0,7678	363,14	
12,2	27,6136	6,9577	288,74	cooling
12,202	26,2931	7,5023	262,67	cooling
12,204	27,0321	6,1121	292,43	
12,206	26,1465	2,3828	332,18	
12,208	26,6289	0,5502	364,54	
12,21	26,13	-0,712	375,21	cooling
12,212	26,085	3,2957	318,56	cooling
12,214	25,9363	8,1629	248,45	cooling
12,216	25,9876	11,1114	207,95	cooling
12,218	26,2948	8,6911	246,07	
12,22	25,9648	5,0987	291,68	
12,222	26,3004	3,6369	316,8	
12,224	25,6832	4,9799	289,4	
12,226	25,8692	7,1197	262,09	
12,228	25,0515	3,2597	304,62	
12,23	25,4195	1,1042	339,89	
12,232	24,9831	0,7531	338,7	
12,234	24,9981	3,44	301,35	
12,236	25,0341	3,5429	300,42	
12,238	25,0949	0,2692	347,03	

12,128	40,3436	22,9604	242,99	heating
12,13	38,4139	17,1443	297,32	heating
12,132	38,7982	20,9155	249,97	heating
12,134	37,6147	18,7959	263,06	heating
12,136	37,7147	15,7955	306,4	heating
12,138	37,4511	16,3358	295,16	heating
12,14	38,0341	15,8249	310,45	heating
12,142	38,8987	19,4807	271,44	heating
12,144	38,8316	20,3807	257,92	heating
12,146	39,973	21,2354	261,92	heating
12,148	40,467	15,4771	349,32	heating
12,15	40,5076	19,6077	292,15	heating
12,152	39,5185	14,5226	349,41	heating
12,154	40,8624	19,466	299,09	heating
12,156	39,5472	14,5446	349,5	heating
12,158	39,6549	15,6013	336,23	heating
12,16	39,8961	16,3894	328,59	heating
12,162	40,1761	17,4118	318,21	heating
12,164	39,5855	14,5814	349,52	heating
12,166	40,0798	17,0535	321,87	heating
12,168	39,3078	16,5033	318,77	
12,17	39,2435	14,6645	343,58	
12,172	38,9122	18,6803	282,81	
12,174	39,5349	15,842	331,19	
12,176	38,9045	18,5271	284,84	
12,178	38,6427	16,5361	309,02	
12,18	39,3819	16,7936	315,75	
12,182	40,1725	14,2743	362,02	
12,184	39,5411	20,2235	270,03	
12,186	39,2274	18,4426	290,54	
12,188	40,7007	21,3569	270,4	
12,19	39,5353	14,174	354,51	
12,192	39,6728	13,6327	364	
12,194	38,8627	14,1149	345,94	
12,196	39,4542	15,8888	329,41	
12,198	38,0226	14,4608	329,36	
12,2	39,2478	17,4719	304,39	
12,202	38,0073	16,1278	305,84	
12,204	38,7372	16,6176	309,2	
12,206	38,4339	19,6248	262,92	
12,208	39,3546	16,3451	321,64	
12,21	39,1428	19,43	275,56	
12,212	38,7529	17,2176	301,03	
12,214	40,3171	18,728	301,78	
12,216	40,244	16,5354	331,41	
12,218	39,9995	19,1381	291,61	
12,22	39,8731	17,0966	318,38	
12,222	41,6461	22,0214	274,33	
12,224	39,4044	13,1784	366,6	
12,226	40,6104	14,7859	360,99	
12,228	39,3127	15,7806	328,94	
12,23	39,6463	15,6386	335,59	
12,232	38,5168	17,0662	299,85	cooling
12,234	39,398	18,7395	288,78	cooling
12,236	38,879	20,3283	259,31	
12,238	38,7803	20,2156	259,51	

12,128	34,4857	9,69	346,61	cooling
12,13	33,6195	8,8928	345,64	cooling
12,132	33,7372	14,3655	270,79	cooling
12,134	33,5327	14,3925	267,55	cooling
12,136	33,4948	14,9139	259,73	cooling
12,138	32,6479	11,034	302,13	
12,14	34,1206	13,2811	291,3	
12,142	33,8899	12,762	295,34	
12,144	33,7427	14,2222	272,87	
12,146	33,8082	14,451	270,58	
12,148	34,9105	14,7933	281,21	
12,15	34,2877	14,0691	282,63	
12,152	34,1301	14,1731	278,97	
12,154	34,2895	12,7749	300,74	
12,156	34,5885	10,081	342,58	
12,158	33,7946	10,0829	331,46	
12,16	33,984	7,073	376,18	
12,162	34,5679	9,1241	355,67	
12,164	34,0104	8,3148	359,19	
12,166	33,8392	9,6427	338,23	cooling
12,168	34,319	9,6391	344,99	cooling
12,17	34,1029	10,5672	328,99	cooling
12,172	33,3954	8,1883	352,36	cooling
12,174	34,506	11,4583	322,17	cooling
12,176	34,0726	9,1527	348,34	cooling
12,178	33,9015	9,0533	347,34	cooling
12,18	33,2344	9,0456	338,12	cooling
12,182	34,561	12,6632	306,1	cooling
12,184	33,633	14,5386	266,91	cooling
12,186	33,5782	12,7877	290,62	cooling
12,188	33,8503	10,436	327,3	cooling
12,19	34,4948	9,2965	352,23	cooling
12,192	33,3602	10,9169	313,72	cooling
12,194	33,7174	13,4563	283,22	cooling
12,196	33,6113	15,4665	253,64	cooling
12,198	32,8145	10,8199	307,45	cooling
12,2	32,9913	12,0338	292,96	cooling
12,202	32,9625	9,3227	330,45	cooling
12,204	33,3934	10,7587	316,4	cooling
12,206	32,7744	12,2793	286,49	cooling
12,208	33,9733	17,1351	235,37	cooling
12,21	33,4693	15,7077	248,28	
12,212	33,4985	14,8765	260,31	
12,214	32,9863	10,2324	318,07	
12,216	34,4984	11,7072	318,59	
12,218	33,1362	13,8599	269,45	
12,22	33,0843	13,303	276,51	
12,222	33,4659	11,7624	303,38	
12,224	33,8912	7,5745	367,87	
12,226	33,3811	9,6895	331,17	
12,228	33,6074	10,1625	327,73	
12,23	34,0064	13,1853	291,05	
12,232	32,8423	9,7789	322,39	
12,234	32,968	11,9302	294,08	
12,236	32,8725	10,5867	311,52	
12,238	32,9831	15,2228	248,26	

12,24	25,8231	-1,6894	384,58		12,24	39,2216	18,1784	294,15		12,24	31,9493	11,506	285,77	
12,242	25,4788	-0,5733	364,17	cooling	12,242	40,3256	13,6078	373,47		12,242	33,6201	15,1727	257,87	
12,244	25,8763	1,88	335,43	cooling	12,244	40,1711	18,9198	297,06		12,244	33,4095	13,5349	277,82	
12,246	25,1837	3,16	307,86	cooling	12,246	39,4385	15,4136	335,83		12,246	33,5748	14,0465	272,98	
12,248	25,4275	3,4277	307,52	cooling	12,248	41,6146	21,4614	281,71		12,248	32,9436	12,2243	289,62	
12,25	24,7452	0,4686	339,35	cooling	12,25	40,3872	16,1233	339,17		12,25	34,3546	15,3569	265,56	
12,252	25,4688	2,9597	314,64	cooling	12,252	40,6087	16,0364	343,48		12,252	33,1233	14,9736	253,71	
12,254	24,6581	-0,3364	349,39	cooling	12,254	40,0393	14,1923	361,3	cooling	12,254	32,8054	13,5153	269,65	
12,256	25,2373	-0,2198	355,85	cooling	12,256	40,7771	16,5195	339,08	cooling	12,256	32,6912	10,3532	312,25	
12,258	25,3208	4,0703	297,05	cooling	12,258	39,1339	15,3118	333	cooling	12,258	33,2424	7,0313	366,39	
12,26	24,9443	6,5245	257,48	cooling	12,26	39,7465	19,6466	280,97	cooling	12,26	32,2784	8,1323	337,53	
12,262	25,1909	4,6428	287,23	cooling	12,262	38,9791	17,2759	303,38	cooling	12,262	32,6727	10,4095	311,21	
12,264	25,0311	4,9564	280,62	cooling	12,264	39,4382	16,8043	316,39	cooling	12,264	33,0252	12,8665	281,79	
12,266	25,0599	2,072	321,34	cooling	12,266	38,7019	15,4889	324,48	cooling	12,266	32,7852	8,659	337,25	
12,268	24,9068	2,1831	317,64	cooling	12,268	39,9357	12,5439	382,9	cooling	12,268	32,8859	11,3055	301,66	
12,27	25,4066	7,3698	252,13	cooling	12,27	39,7278	17,0828	316,54	cooling	12,27	32,9035	9,1071	332,64	
12,272	24,2482	3,1109	295,47	cooling	12,272	38,7576	12,4493	367,75	cooling	12,272	33,284	10,7787	314,59	
12,274	24,9836	0,0922	347,95		12,274	39,9884	14,0706	362,29	cooling	12,274	32,315	10,2183	308,88	
12,276	24,3325	-1,3321	358,75	cooling	12,276	40,0673	12,377	387,07	cooling	12,276	33,2324	15,2226	251,75	
12,278	24,4708	-0,9979	356,02	cooling	12,278	39,3544	15,7231	330,33	cooling	12,278	33,0507	15,0841	251,15	
12,28	24,1302	1,6835	313,77	cooling	12,28	38,9863	15,2704	331,51	cooling	12,28	33,1544	16,5668	231,87	
12,282	24,215	4,6218	273,88	cooling	12,282	40,3412	20,9383	271,22	cooling	12,282	32,3027	10,7884	300,74	
12,284	23,9204	-1,4443	354,56	cooling	12,284	38,7583	12,8576	362,05	cooling	12,284	34,1604	13,084	294,62	
12,286	24,061	-0,8664	348,45	cooling	12,286	39,4124	15,6285	332,46	cooling	12,286	33,2208	13,1693	280,29	
12,288	23,7908	-1,3792	351,84	cooling	12,288	38,6755	16,4805	310,25	cooling	12,288	33,5183	12,8828	288,45	
12,29	23,3043	-2,9497	366,99	cooling	12,29	39,5599	20,5763	265,36	cooling	12,29	33,8335	13,7643	280,54	
12,292	23,8367	1,1509	317,11	cooling	12,292	38,3689	22,793	217,73	cooling	12,292	34,3611	12,4404	306,42	
12,294	23,0888	4,1828	264,28	cooling	12,294	39,3055	21,7676	245,15	cooling	12,294	33,2671	13,1178	281,66	
12,296	23,7326	2,9936	289,9	cooling	12,296	38,8848	18,7744	281,11	cooling	12,296	33,3426	13,3755	279,11	
12,298	23,3514	1,2043	309,58	cooling	12,298	39,0128	16,6223	312,99	cooling	12,298	33,448	13,0205	285,55	
12,3	23,0174	-3,6258	372,43	cooling	12,3	38,7081	14,9713	331,81	cooling	12,3	33,1923	10,7974	313,05	
12,302	23,0574	-4,5102	385,35	cooling	12,302	39,9699	12,2965	386,83	cooling	12,302	33,0199	17,0931	222,63	
12,304	23,4305	4,9926	257,73	cooling	12,304	39,2652	17,624	302,51	cooling	12,304	32,9989	13,9815	265,83	
12,306	22,5493	4,301	255,08	cooling	12,306	38,6243	14,997	330,27	cooling	12,306	33,7924	15,6645	253,4	heating
12,308	22,6901	4,0202	260,98	cooling	12,308	40,1941	20,0647	281,38	cooling	12,308	32,9468	9,1469	332,69	heating
12,31	22,5669	3,2525	269,99	cooling	12,31	39,8733	15,9986	333,73	cooling	12,31	34,3871	10,6621	331,64	
12,312	21,6924	-0,1972	305,98	cooling	12,312	39,425	18,4353	293,4	cooling	12,312	34,1303	12,6881	299,73	
12,314	22,2797	3,1681	267,15	cooling	12,314	39,0007	17,6057	299,07	cooling	12,314	33,9801	17,3095	233,03	
12,316	21,2776	4,5003	234,52	cooling	12,316	40,6449	23,3968	241,1	cooling	12,316	33,1969	15,7284	244,19	heating
12,318	21,867	0,6805	296,16		12,318	39,2374	18,0847	295,68	cooling	12,318	34,5601	17,3347	240,79	heating
12,32	21,9234	3,9305	251,51		12,32	39,4814	20,7103	262,39	cooling	12,32	33,081	13,0353	280,21	heating
12,322	21,8284	4,9876	235,41		12,322	38,6467	17,9996	288,62		12,322	33,5255	11,6614	305,63	heating
12,324	21,7925	1,279	286,75		12,324	39,676	19,3846	283,64		12,324	33,6555	13,7714	277,95	heating
12,326	22,4246	2,0955	284,17		12,326	37,9858	17,076	292,29		12,326	33,8795	9,4782	341,09	heating
12,328	21,5511	-1,8753	327,47		12,328	39,1421	17,4331	303,46		12,328	33,5275	12,2306	297,7	heating
12,33	22,0136	0,2641	304,03		12,33	38,3151	15,1366	324		12,33	33,8262	14,1719	274,74	heating
12,332	21,6315	3,8316	248,82		12,332	38,85	14,903	334,74		12,332	34,2572	16,6077	246,71	heating
12,334	20,8758	-0,2089	294,73		12,334	38,4146	17,9272	286,38		12,334	34,0281	13,5041	286,9	heating
12,336	21,2863	-1,5372	319,04		12,336	39,6265	17,4687	309,73		12,336	33,9546	15,1407	262,99	heating
12,338	21,2434	2,452	262,68		12,338	39,0401	20,9564	252,78		12,338	34,3218	11,6713	316,62	heating
12,34	21,7754	2,9449	263,22		12,34	39,102	21,0794	251,93		12,34	34,5917	17,6837	236,35	heating
12,342	21,4387	4,4978	236,81		12,342	39,9249	24,2787	218,71		12,342	33,1223	12,5621	287,4	heating
12,344	21,6641	1,7444	278,45		12,344	40,4272	21,2841	267,59		12,344	34,7545	15,3142	271,75	heating
12,346	21,5	-0,8108	311,87		12,346	39,5738	23,7395	221,34		12,346	34,1635	17,0064	239,83	heating
12,348	22,4897	5,943	231,3		12,348	38,8169	18,7306	280,78		12,348	33,9917	17,4966	230,58	heating
12,35	21,4646	6,815	204,78		12,35	40,7553	24,2319	230,97		12,35	33,5733	16,5363	238,15	heating

12,352	22,0248	3,6557	256,77	heating	12,352	39,0098	19,8329	268,07		12,352	34,5737	14,6026	279,17	heating
12,354	21,7437	0,9172	291,12	heating	12,354	39,5193	22,6746	235,46	heating	12,354	33,4049	9,0975	339,78	heating
12,356	21,5566	-0,6615	310,58	heating	12,356	38,7304	21,0379	247,32	heating	12,356	33,991	14,0693	278,48	heating
12,358	21,6115	0,903	289,47	heating	12,358	39,639	18,996	288,56	heating	12,358	33,3558	17,3459	223,8	heating
12,36	21,5578	2,7969	262,25	heating	12,36	38,3282	14,1795	337,56	heating	12,36	34,1862	14,3257	277,62	heating
12,362	21,8186	-0,2732	308,81	heating	12,362	40,2139	18,4425	304,33	heating	12,362	33,5266	14,9505	259,67	heating
12,364	22,0148	0,2423	304,35	heating	12,364	38,8939	18,5171	284,84	heating	12,364	33,7271	7,5175	366,37	heating
12,366	22,4388	0,15	311,56	heating	12,366	39,5398	19,601	278,71	heating	12,366	34,7798	9,4078	354,66	heating
12,368	22,5096	-2,5691	350,56	heating	12,368	38,9951	22,8701	225,4	heating	12,368	34,0277	9,4679	343,31	heating
12,37	23,3654	-1,3713	345,78		12,37	40,0999	19,2753	291,1	heating	12,37	34,2544	13,5855	288,92	heating
12,372	22,9751	-1,0371	335,66		12,372	39,3384	20,452	264	heating	12,372	34,7522	15,1384	274,17	heating
12,374	23,7524	1,6327	309,2		12,374	39,1225	17,6266	300,48	heating	12,374	34,5605	17,0251	245,12	heating
12,376	23,4257	1,9769	299,82		12,376	40,3686	17,6922	316,98	heating	12,376	33,6647	9,9556	331,42	heating
12,378	23,4021	-3,5167	376,28		12,378	40,6173	16,0419	343,53	heating	12,378	34,9672	14,8692	280,94	heating
12,38	23,4423	-7,4722	432,14		12,38	39,8391	19,8572	279,32	heating	12,38	33,7516	12,1123	302,49	heating
12,382	23,8409	-3,2241	378,33		12,382	39,2495	14,1562	350,77	heating	12,382	34,4711	14,6607	276,92	heating
12,384	23,7152	0,4716	324,91		12,384	41,8569	21,4679	285,01		12,384	33,6404	15,0983	259,19	heating
12,386	23,5073	2,9219	287,75		12,386	39,6451	16,8834	318,17		12,386	34,7041	12,2147	314,37	heating
12,388	23,5203	0,6311	319,96		12,388	40,5086	21,6725	263,3		12,388	34,0126	13,3868	288,32	heating
12,39	23,5907	-3,6253	380,44		12,39	39,2688	20,8127	257,99		12,39	34,0429	14,1261	278,41	heating
12,392	24,3066	-0,8688	351,91	cooling	12,392	40,0761	19,102	293,19	heating	12,392	33,8839	13,8059	280,66	heating
12,394	23,6086	-0,1761	332,47	cooling	12,394	38,2544	14,8117	327,69	heating	12,394	34,9427	15,3629	273,7	heating
12,396	23,7955	0,0222	332,32	cooling	12,396	39,6244	20,5351	266,84		12,396	33,5381	14,4933	266,22	heating
12,398	23,6746	2,2116	300,02	cooling	12,398	38,5038	18,8129	275,25	heating	12,398	34,6357	11,307	326,1	heating
12,4	23,5368	4,6222	264,4	cooling	12,4	39,2985	23,2407	224,46	heating	12,4	34,8543	16,2555	259,98	heating
12,402	23,1347	3,0655	280,54	cooling	12,402	38,4284	23,0607	214,82	heating	12,402	34,2762	11,974	311,75	heating
12,404	22,8627	1,9907	291,76	cooling	12,404	39,7008	16,7064	321,43	heating	12,404	34,9803	15,7407	268,94	heating
12,406	22,3129	-1,6614	335,12	cooling	12,406	39,7483	20,6584	266,85	heating	12,406	34,7459	15,1164	274,39	heating
12,408	22,246	1,1092	295,46	cooling	12,408	38,6114	15,5312	322,63	heating	12,408	35,0371	15,2046	277,23	heating
12,41	21,8789	5,4797	229,24	cooling	12,41	40,6807	15,359	353,96	heating	12,41	34,2349	14,0199	282,58	heating
12,412	21,6955	4,497	240,41		12,412	40,7606	13,934	375	heating	12,412	35,2879	17,6277	246,86	heating
12,414	22,2749	3,6876	259,82		12,414	40,0164	16,1078	334,21		12,414	34,1676	14,8873	269,51	heating
12,416	21,8343	0,4227	299,3		12,416	39,2338	13,7369	356,41		12,416	34,9346	17,0486	250,02	heating
12,418	22,4191	0,9898	299,55		12,418	41,3798	22,1295	269,09		12,418	33,8076	12,9416	291,68	heating
12,42	22,517	4,0249	258,49		12,42	39,019	12,5858	369,5		12,42	35,5214	11,8176	331,34	heating
12,422	22,2787	2,5018	276,45		12,422	40,5015	17,9536	315,19		12,422	34,2027	11,5156	317,13	heating
12,424	22,4372	1,9961	285,74		12,424	39,392	17,1588	310,79		12,424	35,0193	11,5438	328,15	heating
12,426	22,5334	6,0802	229,99		12,426	40,0027	15,6801	339,99		12,426	35,0196	13,8088	296,49	heating
12,428	21,9903	1,17	291,04		12,428	38,9444	16,0444	320,11		12,428	35,3592	10,0399	353,93	heating
12,43	22,1987	-0,2217	313,4		12,43	39,6852	17,143	315,11		12,43	35,1016	9,5288	357,47	heating
12,432	21,8696	-0,4656	312,21		12,432	39,6625	16,5543	323,02		12,432	35,4756	8,9106	371,34	
12,434	21,8251	-0,0944	306,4		12,434	39,3481	18,6721	289,02		12,434	35,7909	12,795	321,45	
12,436	22,6312	7,5364	211		12,436	39,9057	18,3683	301,06		12,436	34,9849	8,275	373,36	
12,438	21,8896	5,7888	225,06		12,438	40,5198	13,2198	381,61		12,438	35,7974	12,8911	320,2	
12,44	22,4386	0,2412	310,29		12,44	40,4474	18,188	311,15		12,44	35,101	12,4798	316,21	
12,442	23,0815	2,2193	291,62		12,442	39,1156	10,2073	404,09		12,442	35,744	17,7037	252,18	
12,444	22,5363	-0,2091	317,95		12,444	41,598	17,0645	342,94	cooling	12,444	34,3838	13,5237	291,59	
12,446	23,3436	2,0105	298,21		12,446	39,7891	12,5615	380,6	cooling	12,446	35,6014	14,5064	294,88	
12,448	23,1634	6,1549	237,75		12,448	39,7433	15,3601	340,84	cooling	12,448	34,4059	10,1776	338,67	
12,45	22,8691	-0,3779	324,96		12,45	39,3183	17,4227	306,07	cooling	12,45	34,9708	12,9367	308	
12,452	23,5329	2,1226	299,28		12,452	40,0144	20,6256	271,03	cooling	12,452	34,3442	14,7495	273,91	
12,454	22,8979	3,6748	268,71		12,454	38,7461	14,0505	345,21	cooling	12,454	35,1154	13,5882	300,92	
12,456	22,549	-3,2865	361,14		12,456	40,007	17,5339	314,14	cooling	12,456	34,4278	11,5826	319,34	
12,458	23,1103	-0,303	327,28		12,458	39,4597	13,6727	360,46	cooling	12,458	34,9295	12,0932	319,22	
12,46	22,2503	0,9041	298,39		12,46	39,7234	13,1871	370,94	cooling	12,46	34,811	13,4508	298,58	
12,462	22,3534	-1,1194	328,11		12,462	39,5972	16,924	316,94	cooling	12,462	35,0268	10,9707	336,27	

12,464	22,6886	1,8011	291,98		12,464	40,4174	14,1607	367,03	cooling	12,464	34,8071	12,5259	311,46	
12,466	21,6571	-3,1432	346,67		12,466	40,3876	18,5282	305,56	cooling	12,466	35,0393	6,9047	393,28	
12,468	22,4323	-1,7305	337,76		12,468	39,3552	17,0883	311,26	cooling	12,468	35,5613	10,809	346	
12,47	21,9427	1,0714	291,75		12,47	40,6123	18,296	311,95	cooling	12,47	34,7952	8,7993	363,38	
12,472	21,9923	-3,3057	353,63		12,472	41,0707	13,1621	390,12	cooling	12,472	35,4284	12,1032	326,05	cooling
12,474	22,5333	-1,7912	340,02		12,474	40,3371	15,1667	351,85	cooling	12,474	34,8873	9,6411	352,91	cooling
12,476	22,2487	0,5447	303,39		12,476	40,3363	13,0152	381,91	cooling	12,476	35,22	14,5975	288,27	cooling
12,478	22,4122	-1,1632	329,55		12,478	41,6908	22,1945	272,53	cooling	12,478	34,0069	11,7109	311,66	cooling
12,48	23,0572	3,6719	270,98		12,48	40,2902	17,529	318,17	cooling	12,48	35,4336	13,5078	306,49	cooling
12,482	22,0637	-2,3998	341,96		12,482	40,2324	20,9317	269,8	cooling	12,482	34,2702	9,7551	342,68	cooling
12,484	22,8179	-6,8504	414,72		12,484	39,6328	20,3114	270,08	cooling	12,484	34,7461	10,9143	333,13	cooling
12,486	23,3188	-1,1237	341,67	cooling	12,486	39,8023	18,3896	299,32	cooling	12,486	33,9224	12,4772	299,77	cooling
12,488	22,6708	-1,606	339,35	cooling	12,488	38,9749	16,6925	311,47	cooling	12,488	34,9228	13,6773	296,98	cooling
12,49	23,2441	0,4282	318,93	cooling	12,49	39,1902	14,5638	344,24	cooling	12,49	33,6876	10,8574	319,13	cooling
12,492	23,4687	3,7416	275,76	cooling	12,492	38,7169	13,3965	353,94	cooling	12,492	34,5644	9,9213	344,47	cooling
12,494	23,2345	-1,5693	346,72	cooling	12,494	38,5957	16,6657	306,55	cooling	12,494	34,425	12,0933	312,16	cooling
12,496	23,8859	1,46	313,48	cooling	12,496	38,8425	22,4747	228,8	cooling	12,496	34,4491	9,1072	354,24	cooling
12,498	23,4195	3,449	279,16	cooling	12,498	39,8726	20,736	267,5	cooling	12,498	34,2829	12,8806	299,17	cooling
12,5	23,5891	0,1591	327,52	cooling	12,5	39,1059	24,5096	204,03	cooling	12,5	34,2245	10,1335	336,76	cooling
12,502	24,1375	7,2352	236,27	cooling	12,502	38,644	17,4439	296,35	cooling	12,502	34,9983	13,221	304,41	cooling
12,504	23,1723	8,3636	207		12,504	40,0416	19,6031	285,7	cooling	12,504	34,2763	11,5031	318,34	cooling
12,506	23,5205	3,4406	280,69		12,506	39,4555	14,8742	343,61		12,506	34,6016	13,5646	294,07	cooling
12,508	23,4518	3,0209	285,59		12,508	38,7831	17,5558	296,73	cooling	12,508	34,2413	10,3454	334,03	cooling
12,51	22,645	-1,344	335,33		12,51	38,5692	19,0793	272,44	cooling	12,51	34,8185	14,6705	281,64	cooling
12,512	22,8658	1,8533	293,72		12,512	39,5388	25,1428	201,24		12,512	33,4144	8,8455	343,44	cooling
12,514	22,9221	10,4579	174,23		12,514	38,0002	18,6294	270,78		12,514	35,047	13,2494	304,7	cooling
12,516	21,7473	4,2714	244,29		12,516	39,0209	18,3967	288,3		12,516	33,5631	11,9404	302,25	cooling
12,518	22,5197	1,3483	295,94		12,518	38,6322	16,0104	316,22		12,518	34,0218	13,0585	293,04	cooling
12,52	22,0511	-0,0428	308,84		12,52	39,2424	16,2232	321,77		12,52	33,6199	15,2241	257,15	cooling
12,522	22,2446	-3,0801	354		12,522	38,8011	18,5989	282,4		12,522	34,5389	14,2765	283,24	cooling
12,524	22,5864	2,6178	279,13		12,524	39,8629	18,6089	297,1		12,524	33,6464	8,5181	351,26	cooling
12,526	22,0932	4,7964	241,78		12,526	39,1688	19,6729	272,52		12,526	34,3998	8,0231	368,71	cooling
12,528	22,5068	2,3573	281,66		12,528	38,7967	20,4457	256,52		12,528	34,0128	9,3399	344,89	cooling
12,53	22,7959	5,5579	240,96		12,53	39,5257	23,2547	227,44		12,53	34,0876	6,3221	388,12	cooling
12,532	22,0677	1,1821	291,95		12,532	40,5226	18,2446	311,41		12,532	34,2144	12,9767	296,87	cooling
12,534	22,3817	-2,9992	354,79		12,534	39,9357	21,4921	257,81		12,534	33,7469	10,6849	322,37	cooling
12,536	22,8376	3,2556	273,73		12,536	39,1853	15,154	335,92		12,536	34,4911	15,1173	270,82	cooling
12,538	21,8007	2,2027	273,95		12,538	41,3742	19,7787	301,87		12,538	33,4626	11,4539	307,65	cooling
12,54	22,4322	2,3675	280,47		12,54	40,2297	17,4324	318,67		12,54	34,3311	11,3727	320,92	cooling
12,542	22,112	1,1478	293,05		12,542	39,6992	19,8736	277,13		12,542	33,943	6,738	380,29	cooling
12,544	22,1319	-3,2383	354,64		12,544	39,3609	18,7715	287,81		12,544	34,5392	12,5736	307,05	cooling
12,546	22,5643	-0,9675	328,94		12,546	40,2461	23,4932	234,18		12,546	33,407	12,3127	294,87	cooling
12,548	22,4554	2,5484	278,27		12,548	38,2102	15,7741	313,62		12,548	34,8545	18,6747	226,17	cooling
12,55	22,8151	-0,2201	322		12,55	39,24	15,4766	332,18		12,55	33,3449	14,5951	262,09	cooling
12,552	23,1302	2,4588	288,96		12,552	38,6274	14,7115	334,31		12,552	33,8907	12,1291	304,19	cooling
12,554	22,8574	2,2755	287,71		12,554	38,7676	15,241	328,87		12,554	33,1577	10,7272	313,55	cooling
12,556	23,0302	1,7573	297,36		12,556	38,6197	20,6426	251,29		12,556	34,0066	9,9472	336,31	cooling
12,558	23,377	4,538	263,34		12,558	39,3188	20,3657	264,94		12,558	33,0457	8,7279	339,93	cooling
12,56	22,5488	0,7715	304,41		12,56	38,686	20,1911	258,53		12,56	33,6228	8,8431	346,38	cooling
12,562	23,079	-0,5815	330,74		12,562	38,1805	18,3435	277,29		12,562	33,5142	11,2414	311,34	cooling
12,564	22,7738	3,166	274,09		12,564	39,1602	20,7473	257,39		12,564	33,7538	10,5903	323,79	cooling
12,566	22,5019	5,3781	239,37		12,566	39,1547	15,5196	330,38		12,566	33,4633	13,6634	276,77	cooling
12,568	22,3917	6,0057	229,05		12,568	38,1726	18,7909	270,93		12,568	33,4907	8,8623	344,27	cooling
12,57	22,2334	2,8477	270,98		12,57	37,4767	14,6275	319,4		12,57	34,0098	11,2909	317,58	cooling
12,572	22,4375	-4,455	375,92	heating	12,572	39,4405	21,7958	246,65		12,572	33,3319	7,8138	356,7	cooling
12,574	22,4523	-1,6697	337,19		12,574	38,1493	18,7822	270,72		12,574	33,4912	9,2078	339,45	cooling

12,576	22,2056	0,3336	305,74		12,576	38,3597	18,4076	278,9		12,576	33,241	7,6267	358,05	cooling
12,578	22,2455	1,476	290,33		12,578	38,6012	17,0795	300,84	heating	12,578	33,9114	14,3648	273,23	cooling
12,58	22,6422	2,7231	278,44		12,58	39,3028	19,3624	278,74		12,58	33,0332	14,4371	259,94	cooling
12,582	22,1336	-0,1612	311,65		12,582	38,7563	18,3233	285,62	heating	12,582	34,3251	20,1228	198,53	cooling
12,584	22,9845	-1,1032	336,71		12,584	40,2412	20,8114	271,6	heating	12,584	32,9044	15,5826	242,13	cooling
12,586	22,6506	0,3956	311,09		12,586	39,7149	19,8046	278,32	heating	12,586	33,5019	11,9034	301,92	
12,588	22,8413	1,3776	300,03		12,588	39,8089	18,8748	292,63	heating	12,588	32,9566	9,2118	331,92	
12,59	22,56	1,9623	287,92		12,59	40,3186	23,4726	235,48	heating	12,59	33,9892	10,518	328,09	
12,592	22,5824	1,508	294,59		12,592	40,8872	20,5381	284,45	heating	12,592	32,9883	13,2018	276,59	
12,594	22,1347	-1,8778	335,66		12,594	40,0639	23,9387	225,41	heating	12,594	33,4692	14,2382	268,82	
12,596	21,8251	0,7877	294,07		12,596	38,875	19,4549	271,46	heating	12,596	33,1867	14,1623	265,93	
12,598	21,7031	3,6937	251,74		12,598	40,2097	19,1482	294,41	heating	12,598	33,8521	11,4979	312,48	
12,6	21,8189	6,438	215		12,6	39,7085	14,7923	348,29	heating	12,6	33,5965	13,2937	283,8	
12,602	22,2497	5,4598	234,7		12,602	38,6703	19,2316	271,72	heating	12,602	33,8422	11,1646	317	
12,604	22,5358	1,7661	290,33		12,604	38,4268	19,4741	264,93	heating	12,604	34,1934	13,6018	287,84	
12,606	23,7437	1,014	317,73		12,606	39,5308	25,5961	194,79	heating	12,606	33,5765	9,0839	342,37	
12,608	23,5023	3,4645	280,1		12,608	37,612	20,1818	243,65	heating	12,608	34,4029	13,5394	291,64	
12,61	24,0041	5,1908	262,98		12,61	38,818	18,5196	283,74	heating	12,61	33,7995	13,8016	279,54	
12,612	23,9327	5,2776	260,77	heating	12,612	38,5705	16,8184	304,06	heating	12,612	34,4122	17,4289	237,4	
12,614	24,2822	2,5397	303,93	heating	12,614	38,8969	16,5142	312,88	heating	12,614	33,2188	12,4947	289,69	
12,616	23,805	-2,2455	364,15	heating	12,616	38,8055	23,0609	220,09	heating	12,616	34,3241	12,7624	301,4	
12,618	24,2024	0,4196	332,45		12,618	39,7667	24,635	211,52	heating	12,618	32,9472	8,4119	342,97	
12,62	23,7137	2,6484	294,46		12,62	38,7299	21,854	235,9	heating	12,62	33,6722	10,0308	330,47	
12,622	23,7973	3,2564	287,13		12,622	38,5445	17,8994	288,59	heating	12,622	32,7153	11,074	302,51	
12,624	23,9304	3,131	290,75		12,624	39,4544	20,1607	269,7	heating	12,624	33,6731	14,0462	274,36	
12,626	23,8631	-0,5514	341,28		12,626	40,2179	15,6507	343,41	heating	12,626	32,666	14,5499	253,24	
12,628	24,483	-2,9763	383,84		12,628	39,5128	20,5101	265,63	heating	12,628	33,1368	10,2963	319,28	
12,63	24,5513	0,9715	329,61		12,63	38,8812	15,0897	332,57	heating	12,63	32,9416	7,0993	361,24	
12,632	24,7643	6,4109	256,55		12,632	41,1354	16,672	341,96	heating	12,632	33,2642	3,6057	414,58	
12,634	24,3865	7,1426	241,04		12,634	40,2133	15,1857	349,85	heating	12,634	33,3413	8,2974	350,08	
12,636	24,5167	3,8454	288,95		12,636	39,7885	18,433	298,52	heating	12,636	33,2954	9,8737	327,4	cooling
12,638	23,952	-1,4334	354,85		12,638	39,4673	17,5988	305,69	heating	12,638	33,5684	14,8727	261,34	cooling
12,64	24,199	0,6078	329,77		12,64	40,4622	19,9068	287,33	heating	12,64	33,3215	12,4486	291,77	
12,642	23,5734	4,5983	265,24		12,642	38,4995	14,0234	342,14	heating	12,642	33,9251	14,5328	271,08	
12,644	23,3885	4,1777	268,54		12,644	39,4373	15,0547	340,83	heating	12,644	33,3022	10,8895	313,3	
12,646	23,5377	1,8679	302,91		12,646	38,8498	14,6046	338,91		12,646	33,9663	15,1334	263,26	
12,648	23,6573	0,3068	326,41		12,648	39,2765	15,7807	328,44		12,648	32,7017	10,8237	305,82	
12,65	24,0841	-0,6826	346,2		12,65	38,4852	20,3494	253,51		12,65	33,8296	11,4503	312,83	
12,652	24,0744	1,2223	319,44		12,652	39,4341	21,3648	252,58		12,652	32,6735	10,5318	309,51	
12,654	24,2869	2,8185	300,1		12,654	38,5468	19,3218	268,74		12,654	33,4047	14,129	269,45	
12,656	23,9563	1,0675	319,95		12,656	38,2909	15,6645	316,28	heating	12,656	32,354	16,0747	227,56	
12,658	24,8205	3,211	302,07		12,658	39,0685	18,4587	288,09		12,658	33,0041	16,146	235,65	
12,66	23,8722	2,309	301,42		12,66	39,9118	16,0681	333,3		12,66	32,5262	13,7188	262,9	
12,662	24,2924	2,7447	301,2		12,662	39,1854	17,6567	300,94		12,662	33,6863	13,0307	288,74	
12,664	23,7339	2,9377	290,7		12,664	38,9606	12,5479	369,21		12,664	33,2087	13,3313	277,86	
12,666	23,8234	2,4279	299,08		12,666	40,9641	17,8256	323,44		12,666	33,6464	10,9983	316,59	
12,668	23,746	0,2213	328,84		12,668	40,3675	18,0454	312,03		12,668	33,6621	14,0786	273,75	
12,67	24,6676	2,4169	311,03		12,67	39,719	20,5845	267,47		12,67	33,499	10,2189	325,42	
12,672	25,1433	1,9485	324,23		12,672	39,2542	16,4713	318,47		12,672	34,0429	13,9824	280,42	
12,674	25,2145	5,1493	280,48		12,674	39,9287	17,1741	318,08		12,674	33,3882	12,9773	285,31	
12,676	25,3137	7,2201	252,92		12,676	38,1272	14,9009	324,67		12,676	33,8774	14,461	271,41	
12,678	24,9359	3,6469	297,59		12,678	38,9602	15,915	322,14		12,678	33,2837	12,6423	288,54	
12,68	25,5989	1,39	338,4		12,68	38,416	17,1574	297,16		12,68	33,8553	15,6046	255,12	
12,682	24,9486	-0,4377	354,86		12,682	38,6746	18,3898	283,55		12,682	32,5882	9,8925	317,25	
12,684	25,7779	0,812	348,99		12,684	38,7592	22,0896	233,02		12,684	33,8155	13,6508	281,87	
12,686	25,4381	4,5545	291,92		12,686	40,1876	20,2971	278,04		12,686	32,2481	11,2179	293,97	

12,688	25,1689	4,0798	294,79		12,688	39,2286	17,9562	297,36		12,688	33,5128	13,158	284,53	
12,69	24,7065	-0,813	356,72		12,69	39,4613	13,3233	365,37		12,69	32,4824	14,5357	250,87	
12,692	25,7004	1,9351	332,2		12,692	40,9458	18,9205	307,88		12,692	32,9827	13,0552	278,56	
12,694	25,2593	-0,3307	357,71		12,694	40,7566	16,9501	332,78		12,694	32,7208	12,8663	277,54	heating
12,696	25,2411	2,7178	314,84	cooling	12,696	40,0048	18,7595	296,98		12,696	33,4064	13,7136	275,28	heating
12,698	24,8854	4,5748	283,91	cooling	12,698	39,8448	17,8268	307,78		12,698	33,1991	12,8271	284,77	heating
12,7	24,418	1,4433	321,15	cooling	12,7	41,1283	20,1939	292,63		12,7	33,7739	12,5943	296,06	heating
12,702	24,8861	1,0395	333,34	cooling	12,702	39,8623	16,916	320,76		12,702	33,5806	16,3351	241,07	heating
12,704	24,5856	2,8649	303,62	cooling	12,704	40,3626	19,0887	297,38		12,704	33,4672	14,4485	265,85	heating
12,706	25,3423	0,8028	343,03	cooling	12,706	39,8559	16,28	329,56		12,706	34,1588	19,4076	206,2	heating
12,708	25,2087	5,1859	279,89	cooling	12,708	40,7785	16,5145	339,18		12,708	33,2132	13,0853	281,36	heating
12,71	24,9228	6,1171	262,87	cooling	12,71	39,3416	17,8229	300,8		12,71	34,1918	14,2626	278,58	heating
12,712	24,6555	2,8062	305,42		12,712	40,5939	20,7412	277,51		12,712	33,1578	11,1951	307,01	heating
12,714	25,7496	5,9026	277,43		12,714	39,2047	21,4696	247,91		12,714	33,7968	13,7185	280,67	heating
12,716	24,598	0,3928	338,35		12,716	38,9787	20,2229	262,18		12,716	33,0016	12,5296	286,17	heating
12,718	25,2706	0,315	348,84		12,718	38,4191	20,2117	254,51		12,718	33,5514	15,1227	257,61	heating
12,72	24,809	5,3912	271,43	cooling	12,72	40,1629	17,1958	321,05		12,72	33,029	13,8312	268,36	heating
12,722	24,092	5,0773	265,8		12,722	39,3555	13,7667	357,69		12,722	33,9386	17,4539	230,43	heating
12,724	24,2263	3,9829	282,97		12,724	39,7135	10,5686	407,4		12,724	32,9833	15,0793	250,27	heating
12,726	24,2211	4,8827	270,32		12,726	41,5796	18,9027	316,99		12,726	33,784	12,2904	300,45	heating
12,728	24,6671	-0,1083	346,32		12,728	41,3034	19,742	301,4		12,728	33,6495	11,0969	315,25	heating
12,73	24,5328	2,605	306,52		12,73	40,3615	21,0149	270,44		12,73	34,0553	8,2046	361,35	heating
12,732	24,2544	3,1333	295,24		12,732	39,5545	15,4112	337,49		12,732	34,1543	13,0525	294,97	heating
12,734	24,0343	1,0115	321,82		12,734	41,3902	16,4538	348,57		12,734	34,0139	10,8665	323,57	heating
12,736	25,4475	7,3442	253,06		12,736	39,5719	14,0357	356,96		12,736	34,3308	14,9286	271,21	heating
12,738	24,4451	5,5356	264,33		12,738	39,8429	19,2806	287,43		12,738	34,1346	14,4751	274,81	heating
12,74	25,0897	3,6594	299,56		12,74	39,0066	15,4082	329,87		12,74	34,4092	16,6981	247,58	heating
12,742	24,6452	4,7909	277,53		12,742	40,7961	17,0173	332,39		12,742	34,3903	13,5493	291,33	heating
12,744	24,0713	1,6025	314,08		12,744	39,264	17,8154	299,82	cooling	12,744	34,698	15,621	266,67	heating
12,746	24,0852	-0,6648	345,97		12,746	41,1	20,3421	290,16	cooling	12,746	34,1884	9,0609	351,25	heating
12,748	24,4971	3,2255	297,35		12,748	40,5102	17,0399	328,08		12,748	34,9773	15,454	272,91	heating
12,75	24,4921	-0,236	345,66		12,75	40,0386	15,8967	337,47	cooling	12,75	33,5415	16,4011	239,6	heating
12,752	24,1358	-0,2792	341,29		12,752	39,8946	17,7565	309,46	cooling	12,752	34,2647	16,8437	243,52	heating
12,754	24,1393	0,8272	325,87		12,754	40,8932	17,7516	323,48	cooling	12,754	33,7874	18,863	208,62	heating
12,756	23,624	-2,631	367,01		12,756	40,215	20,0676	281,63	cooling	12,756	33,9829	15,6095	256,83	heating
12,758	24,3607	-1,9977	368,45	cooling	12,758	39,1833	16,286	320,07	cooling	12,758	33,2505	15,3147	250,72	heating
12,76	23,6506	-1,2649	348,28	cooling	12,76	41,9049	24,1874	247,66	cooling	12,76	33,7376	14,2267	272,73	heating
12,762	24,0711	-2,2758	368,29	cooling	12,762	41,0912	23,5931	244,6		12,762	33,0844	8,4187	344,79	heating
12,764	23,9362	-0,209	337,51	cooling	12,764	40,6574	24,1338	230,97		12,764	34,8208	9,3083	356,63	heating
12,766	23,3773	-0,6599	336	cooling	12,766	40,0402	19,1269	292,34		12,766	33,7917	15,1351	260,79	heating
12,768	23,0825	-4,7606	389,21	cooling	12,768	42,1951	21,0561	295,49		12,768	34,3102	14,2548	280,35	heating
12,77	23,9423	-0,1712	337,07	cooling	12,77	40,1573	15,8252	340,13		12,77	34,1234	20,6448	188,41	heating
12,772	23,6715	2,6582	293,73	cooling	12,772	40,1501	21,9124	254,94		12,772	33,6619	13,2969	284,67	heating
12,774	23,2347	0,6725	315,39	cooling	12,774	39,2645	18,7154	287,25		12,774	34,4731	10,4297	336,09	heating
12,776	23,5545	2,5338	293,84	cooling	12,776	40,1925	16,7767	327,32		12,776	34,8082	11,6734	323,39	heating
12,778	22,7847	-0,6145	327,09	cooling	12,778	39,2198	17,2139	307,61		12,778	34,7197	11,3154	327,16	heating
12,78	23,0875	-2,9293	363,68	cooling	12,78	40,6712	17,7811	319,97		12,78	34,2354	10,6521	329,66	heating
12,782	23,4426	2,0352	299,24	cooling	12,782	40,8496	18,6739	309,98		12,782	34,5688	18,1097	230,07	heating
12,784	23,7772	1,4256	312,44	cooling	12,784	40,097	16,1961	334,1		12,784	33,1162	14,4179	261,37	heating
12,786	24,0021	1,3359	316,84	cooling	12,786	40,7006	18,4133	311,54		12,786	35,0218	16,8899	253,46	heating
12,788	23,8314	5,518	255,99	cooling	12,788	41,2038	17,7539	327,79		12,788	33,4544	13,0237	285,59	heating
12,79	23,3922	2,991	285,18	cooling	12,79	40,4021	20,8636	273,12		12,79	34,9559	6,7082	394,86	heating
12,792	24,1987	4,7283	272,17	cooling	12,792	39,0527	15,1761	333,76		12,792	34,1276	9,8528	339,33	heating
12,794	23,8588	4,2327	274,34	cooling	12,794	40,818	22,3716	257,85		12,794	34,5092	11,2121	325,66	heating
12,796	23,6152	-0,9821	343,83	cooling	12,796	39,3947	18,7578	288,47		12,796	33,7375	10,1136	330,23	heating
12,798	24,07	2,4303	302,49	cooling	12,798	39,4573	17,9799	300,22		12,798	35,3655	14,6127	290,09	

12,8	22,9655	2,8088	281,76	cooling	12,8	39,1068	13,4993	357,96		12,8	34,0969	14,7666	270,21	
12,802	22,7473	1,9589	290,59	cooling	12,802	40,8445	14,9223	362,35		12,802	35,1073	13,465	302,53	heating
12,804	23,4648	6,581	236,01	cooling	12,804	39,9387	15,5886	340,38		12,804	34,7544	18,5419	226,63	
12,806	23,4348	2,5117	292,47		12,806	40,3493	21,9538	257,14		12,806	34,1216	10,722	327,09	heating
12,808	23,7708	0,509	325,16		12,808	39,7411	19,0483	289,25		12,808	35,2067	13,8791	298,13	heating
12,81	23,415	2,7383	289,03		12,81	40,2906	16,1067	338,06		12,81	34,6102	15,136	272,22	heating
12,812	23,2888	0,5097	318,42		12,812	39,6379	17,6284	307,66		12,812	35,3251	15,0829	282,96	heating
12,814	23,9026	4,2836	274,24		12,814	40,9052	17,5163	326,94		12,814	34,5967	16,4854	253,17	heating
12,816	23,3257	5,4706	249,59		12,816	40,3594	20,5799	276,49		12,816	34,4887	17,9456	231,25	heating
12,818	23,5114	1,8489	302,81		12,818	39,4964	16,2372	325,13		12,818	33,4493	10,5541	320,04	heating
12,82	24,0525	3,7622	283,63		12,82	40,9268	20,0944	291,2		12,82	35,1162	14,4183	289,33	heating
12,822	23,2837	2,4019	291,9		12,822	41,4639	21,1693	283,69		12,822	33,3913	10,7805	316,07	heating
12,824	23,402	0,5057	320,06		12,824	40,6593	24,3585	227,86		12,824	35,0121	8,6877	367,98	heating
12,826	24,3398	4,1876	281,7		12,826	40,0472	20,3269	275,66		12,826	34,3498	13,1082	296,93	
12,828	24,2698	0,7385	328,93		12,828	41,5528	20,6991	291,5		12,828	34,6923	13,3913	297,76	
12,83	24,7156	0,0611	344,63		12,83	40,0314	15,7013	340,1		12,83	34,377	13,399	293,24	
12,832	24,6972	1,7477	320,8		12,832	40,1676	19,0378	295,36		12,832	35,1426	12,4439	317,29	
12,834	24,951	-1,4251	368,7		12,834	39,1278	16,1722	320,88		12,834	34,3422	8,0214	367,93	
12,836	25,4791	0,1402	354,2		12,836	40,9078	18,1996	317,43		12,836	35,0307	6,0951	404,48	
12,838	24,8961	-0,5052	355,07	cooling	12,838	39,1736	21,2728	250,23		12,838	34,9462	10,1928	346,02	
12,84	25,543	-0,6184	365,7	cooling	12,84	40,3685	24,5393	221,27		12,84	34,5888	7,3884	380,22	
12,842	25,7311	1,5559	337,93	cooling	12,842	39,9358	21,7438	254,3		12,842	35,333	14,2308	294,98	
12,844	25,0813	-2,8761	390,8	cooling	12,844	40,2675	16,2345	335,95		12,844	34,1931	12,7932	299,14	
12,846	25,167	-4,3669	412,84	cooling	12,846	39,8882	15,0735	346,87		12,846	35,5111	13,7403	304,32	
12,848	25,7334	0,2905	355,65	cooling	12,848	41,1132	17,2214	333,97		12,848	34,4953	12,178	311,96	
12,85	24,8648	-2,2656	379,24	cooling	12,85	40,1686	21,2914	263,88		12,85	35,012	10,6017	341,22	
12,852	25,2828	2,2382	322,13	cooling	12,852	39,1529	18,9514	282,39		12,852	34,2956	7,9401	368,41	
12,854	24,4652	2,6005	305,64	cooling	12,854	40,34	23,4389	236,25		12,854	35,2379	10,1025	351,36	
12,856	24,8472	-2,2047	378,15	cooling	12,856	39,7894	18,6716	295,19		12,856	33,8195	6,3104	384,54	cooling
12,858	25,0414	0,0326	349,59	cooling	12,858	40,0875	19,8156	283,37		12,858	35,0152	10,013	349,49	cooling
12,86	24,5281	0,7826	331,93	cooling	12,86	39,406	16,5102	320,05		12,86	33,94	15,111	263,2	cooling
12,862	25,248	-1,3159	371,32	cooling	12,862	41,21	18,2032	321,6		12,862	34,1853	14,0269	281,78	cooling
12,864	25,4299	5,7047	275,73	cooling	12,864	39,9373	17,2607	316,99		12,864	34,186	15,0207	267,9	cooling
12,866	24,6662	3,9973	288,92	cooling	12,866	40,6108	23,0224	245,86		12,866	34,2721	9,3285	348,67	cooling
12,868	24,6977	2,9901	303,44	cooling	12,868	39,8766	22,2499	246,39		12,868	34,5648	8,231	368,11	cooling
12,87	25,2299	5,685	273,21	cooling	12,87	40,6014	22,8808	247,71		12,87	34,5133	9,07	355,66	cooling
12,872	23,8087	-1,2077	349,69	cooling	12,872	39,3161	23,227	224,9		12,872	34,577	14,4417	281,46	cooling
12,874	24,4196	0,9842	327,59	cooling	12,874	40,2651	22,9669	241,8	heating	12,874	33,9932	11,9108	308,68	cooling
12,876	23,6657	6,1498	244,85	cooling	12,876	39,7521	20,6033	267,67	heating	12,876	34,5989	14,7824	277,01	cooling
12,878	23,5496	4,2755	269,42	cooling	12,878	39,3664	13,5038	361,52	heating	12,878	33,6185	11,0661	315,25	cooling
12,88	23,3075	3,8123	272,51	cooling	12,88	40,5372	18,5127	307,87	heating	12,88	34,5318	14,8631	274,94	cooling
12,882	23,8153	4,7656	266,29	cooling	12,882	41,0163	19,8816	295,43		12,882	33,3353	12,5472	290,59	cooling
12,884	23,9776	-1,4781	355,83	cooling	12,884	40,5008	26,9141	189,92		12,884	34,2986	12,4651	305,2	cooling
12,886	24,3355	2,9251	299,29	cooling	12,886	39,3893	20,8797	258,74	heating	12,886	33,7589	11,0602	317,29	cooling
12,888	23,7032	3,3543	284,45	cooling	12,888	41,1411	19,3303	304,88	heating	12,888	34,5364	13,5658	293,14	cooling
12,89	23,9794	1,0965	319,87	cooling	12,89	40,4788	13,5877	375,9	heating	12,89	33,691	14,0482	274,58	cooling
12,892	24,3969	1,9565	313,68	cooling	12,892	40,2926	19,5532	289,91	heating	12,892	34,3514	14,4534	278,14	
12,894	23,6023	-0,6967	339,66	cooling	12,894	39,8157	18,7745	294,13	heating	12,894	33,4822	10,2138	325,26	
12,896	24,6184	-3,1278	387,85	cooling	12,896	40,9295	20,6747	283,13	heating	12,896	34,1242	7,2844	375,18	
12,898	24,1734	2,2505	306,45	cooling	12,898	39,548	18,3293	296,61	heating	12,898	33,5153	10,3946	323,19	cooling
12,9	23,8997	2,4278	300,15	cooling	12,9	40,2951	18,36	306,62	heating	12,9	33,1974	9,1529	336,11	cooling
12,902	23,5737	-2,5299	364,89	cooling	12,902	39,9599	18,9923	293,1		12,902	33,3799	14,6331	262,05	cooling
12,904	24,8762	-1,0252	362,06	cooling	12,904	39,7787	14,8505	348,46		12,904	33,0116	13,9923	265,86	cooling
12,906	23,5039	-5,5375	405,96	cooling	12,906	39,5884	18,1644	299,48		12,906	33,7231	14,6544	266,55	
12,908	24,1384	-3,8838	391,71	cooling	12,908	40,3447	17,6702	316,96		12,908	33,9044	12,8988	293,63	
12,91	24,052	3,6932	284,58	cooling	12,91	39,7321	17,2574	314,16		12,91	34,3915	14,4357	278,95	

12,912	23,6817	3,6852	279,52	cooling	12,912	39,6887	13,5476	365,41		12,912	33,902	10,8975	321,57	
12,914	23,3623	-2,6031	362,96	cooling	12,914	40,8105	21,4781	270,24		12,914	34,3967	15,2383	267,81	
12,916	24,9402	4,1573	290,51	cooling	12,916	41,1345	19,8645	297,32		12,916	33,0343	11,3296	303,4	
12,918	24,1464	2,4373	303,46	cooling	12,918	40,6654	24,3375	228,24		12,918	33,8125	13,8763	278,68	
12,92	24,2118	6,2671	250,84	cooling	12,92	39,9159	19,852	280,46		12,92	32,5989	14,1595	257,76	
12,922	24,0582	8,9839	210,72	cooling	12,922	41,4271	21,4322	279,5		12,922	32,9783	12,5271	285,88	
12,924	23,3716	0,9606	313,27	cooling	12,924	40,3355	19,0587	297,42		12,924	32,8412	13,2939	273,24	
12,926	24,1244	-2,6506	374,27	cooling	12,926	40,6185	20,4532	281,88		12,926	33,2341	10,909	312,07	
12,928	23,2325	-2,0163	352,94	cooling	12,928	40,1407	19,4394	289,37		12,928	33,1135	6,1574	376,81	
12,93	23,9121	-3,4362	382,29	cooling	12,93	40,9156	19,5122	299,19		12,93	33,9594	6,0202	390,55	
12,932	23,9008	3,4237	286,24	cooling	12,932	39,4701	17,5489	306,43		12,932	33,6399	9,6026	336,01	
12,934	23,304	3,9307	270,81	cooling	12,934	41,0252	21,0047	279,86		12,934	33,8237	10,3333	328,36	
12,936	23,1789	-2,7165	361,98	cooling	12,936	40,1437	23,2381	236,32		12,936	33,9657	16,1881	248,5	
12,938	24,3247	-1,9154	366,8	cooling	12,938	40,6014	18,7721	305,14	heating	12,938	33,3355	9,7025	330,35	
12,94	23,0047	-7,8449	431,23	cooling	12,94	40,0872	18,9713	295,17		12,94	34,118	9,8391	339,38	
12,942	23,5204	-4,017	384,93	cooling	12,942	40,889	15,1571	359,69		12,942	33,2243	8,6511	343,5	
12,944	23,2052	5,0731	253,46	cooling	12,944	40,3167	16,0793	338,8		12,944	33,9089	11,9799	306,53	cooling
12,946	22,5883	4,0786	258,74	cooling	12,946	40,048	14,8178	352,68		12,946	33,4655	12,9769	286,4	cooling
12,948	22,801	1,5331	297,29	cooling	12,948	40,9764	19,8624	295,14		12,948	34,111	17,2532	235,65	
12,95	22,9099	1,0675	305,32	cooling	12,95	41,1522	17,8388	325,89		12,95	33,1244	13,9153	268,51	
12,952	23,3585	-3,5356	375,94	cooling	12,952	40,6302	22,1313	258,59		12,952	34,0542	14,2805	276,41	
12,954	23,3532	3,7949	273,4	cooling	12,954	39,6488	14,956	345,17		12,954	32,8387	9,4373	327,12	
12,956	22,6806	4,7325	250,89	cooling	12,956	41,5345	15,3872	365,5		12,956	33,6616	7,2881	368,66	
12,958	22,7509	-0,82	329,49	cooling	12,958	40,0599	11,2032	403,37		12,958	33,1515	8,6866	341,98	
12,96	23,5411	1,5482	307,43	cooling	12,96	40,6454	13,6041	378		12,96	33,7716	11,5571	310,53	
12,962	22,3657	0,559	304,83	cooling	12,962	39,8957	14,055	361,22	cooling	12,962	33,358	14,0423	270	
12,964	23,3369	0,6934	316,52	cooling	12,964	40,7852	16,7812	335,54	cooling	12,964	33,5365	11,7079	305,13	
12,966	22,898	4,8983	251,61	cooling	12,966	39,1686	16,6963	314,13	cooling	12,966	33,8524	12,9918	291,6	
12,968	22,4159	1,6628	290,1	cooling	12,968	40,4638	19,8197	288,57	cooling	12,968	33,6717	10,841	319,14	
12,97	22,6934	0,9327	304,18	cooling	12,97	39,4507	19,0038	285,82	cooling	12,97	33,8286	13,0025	291,12	
12,972	23,9474	5,499	257,88	cooling	12,972	39,6464	12,5554	378,69	cooling	12,972	33,4209	12,5866	291,23	
12,974	22,9785	0,2181	318,16	cooling	12,974	39,52	13,4766	364,05	cooling	12,974	33,7121	15,1104	260,02	
12,976	23,2977	3,2017	280,91	cooling	12,976	40,0377	12,0217	391,62	cooling	12,976	33,0176	11,683	298,23	
12,978	22,5692	5,5751	237,55	cooling	12,978	39,7107	15,0644	344,52	cooling	12,978	33,8604	14,7152	267,62	
12,98	22,7609	5,2422	244,88		12,98	39,4965	13,314	365,99	cooling	12,98	33,0177	12,2948	289,67	
12,982	22,7748	6,2574	230,89		12,982	41,03	17,9864	322,11	cooling	12,982	33,7591	16,1751	245,8	
12,984	22,0425	3,228	263		12,984	40,945	15,241	359,3	cooling	12,984	33,0323	13,8582	268,03	
12,986	23,2618	0,5616	317,32		12,986	40,4076	19,9228	286,35	cooling	12,986	33,9759	12,2761	303,33	
12,988	22,7953	5,7706	237,98		12,988	38,9692	12,5807	368,87	cooling	12,988	33,601	11,5346	308,46	
12,99	22,4958	3,5478	264,86		12,99	40,9855	13,6489	382,12	cooling	12,99	33,687	10,2419	327,73	
12,992	22,6152	1,3055	297,88		12,992	39,1819	11,5951	385,62	cooling	12,992	33,7738	12,4953	297,44	
12,994	23,7714	4,562	268,52		12,994	39,495	17,4063	308,77	cooling	12,994	33,8359	13,9503	277,97	
12,996	22,5835	-0,0021	315,71		12,996	38,7777	19,6394	267,52	cooling	12,996	33,151	12,8679	283,53	
12,998	23,23	3,1146	281,18		12,998	39,8085	20,2909	272,83	cooling	12,998	33,536	11,0225	314,71	
13	22,3859	2,2324	281,72		13	38,4677	14,9647	328,54	cooling	13	33,3552	11,4006	306,89	
13,002	22,3501	-3,9237	367,27		13,002	40,3201	16,5608	332,12	cooling	13,002	33,5724	7,2179	368,4	
13,004	22,4099	-4,7268	379,33		13,004	39,5153	15,35	337,8	cooling	13,004	34,134	12,9862	295,62	
13,006	22,3689	-1,9822	340,39		13,006	39,8821	13,0896	374,52	cooling	13,006	33,6875	10,9934	317,23	
13,008	22,983	-3,3423	367,99		13,008	39,256	16,475	318,44	cooling	13,008	34,1568	13,351	290,83	
13,01	22,1929	-0,7253	320,36		13,01	39,633	14,9423	345,14	cooling	13,01	33,8941	14,4948	271,17	
13,012	21,8527	-4,2033	364,22		13,012	39,2352	17,6348	301,94	cooling	13,012	34,0001	12,8859	295,15	
13,014	21,8921	-5,2985	380,08		13,014	38,3656	14,8617	328,55	cooling	13,014	34,0771	10,3152	332,16	
13,016	22,8237	0,476	312,39	cooling	13,016	39,7792	17,6535	309,28	cooling	13,016	34,1689	12,4702	303,32	
13,018	21,6622	-2,8644	342,85	cooling	13,018	39,9925	14,5151	356,14	cooling	13,018	33,3262	9,6538	330,91	
13,02	22,8469	-2,0699	348,3	cooling	13,02	39,5876	19,4214	281,89	cooling	13,02	34,4749	14,131	284,38	
13,022	22,4018	0,9523	299,83	cooling	13,022	38,4302	14,2946	337,38	cooling	13,022	33,0858	13,8144	269,39	

13,024	22,5192	0,379	309,49	cooling	13,024	40,7353	20,6545	280,7	cooling	13,024	33,9798	9,9603	335,76
13,026	22,6486	2,0215	288,34	cooling	13,026	39,5435	19,6133	278,59	cooling	13,026	33,7206	14,5775	267,59
13,028	23,4307	6,0348	243,17	cooling	13,028	39,3179	23,1887	225,46	cooling	13,028	33,8467	13,0188	291,14
13,03	23,4417	2,8143	288,34	cooling	13,03	38,685	21,1892	244,57	cooling	13,03	33,2655	9,6843	329,63
13,032	23,2984	7,3186	223,37	cooling	13,032	39,7897	19,9285	277,63	cooling	13,032	34,6538	11,578	322,57
13,034	22,6494	4,9282	247,72		13,034	38,1233	12,2213	362,07	cooling	13,034	33,5635	9,4572	336,97
13,036	22,3383	-2,0051	340,28		13,036	39,1423	14,8645	339,37	cooling	13,036	34,292	10,0772	338,49
13,038	23,1067	2,3937	289,54		13,038	38,4224	17,5649	291,56	cooling	13,038	33,8452	17,1138	233,88
13,04	21,8207	3,7122	253,13		13,04	39,0257	17,8851	295,51	cooling	13,04	33,5196	10,9695	315,22
13,042	22,9631	2,5369	285,53		13,042	38,2381	20,7152	244,94	cooling	13,042	34,3162	13,058	297,16
13,044	22,4684	3,7226	262,04		13,044	39,216	19,7337	272,33	cooling	13,044	33,4289	13,3242	281,03
13,046	22,2879	-0,4276	317,53		13,046	38,9757	19,5228	271,92	cooling	13,046	34,261	11,5577	317,36
13,048	22,6663	-1,0529	331,56		13,048	38,8943	15,5859	325,82		13,048	33,7397	13,957	276,53
13,05	23,5877	6,1426	243,86		13,05	39,4525	15,7994	330,63	cooling	13,05	33,618	12,5974	293,84
13,052	22,5246	-1,0635	329,73		13,052	40,0341	12,0096	391,74	cooling	13,052	33,4974	8,396	350,88
13,054	22,9692	-2,6675	358,36		13,054	39,6372	17,6963	306,7	cooling	13,054	35,162	14,9638	282,34
13,056	22,4959	-3,2131	359,37		13,056	38,6349	14,456	337,98	cooling	13,056	33,2372	13,1764	280,42
13,058	22,3874	-3,9692	368,43		13,058	40,3789	20,0389	284,32	cooling	13,058	34,4647	12,9074	301,34
13,06	22,6331	2,0959	287,08		13,06	38,5436	18,0501	286,47	cooling	13,06	33,8623	14,5768	269,58
13,062	22,1035	2,685	271,44		13,062	38,4177	17,4637	292,91	cooling	13,062	33,8379	7,1667	372,82
13,064	23,086	-1,9303	349,69		13,064	37,7388	14,3799	326,52	cooling	13,064	33,933	9,041	347,95
13,066	22,7426	3,1306	274,15		13,066	38,4991	14,1324	340,61	cooling	13,066	33,7119	11,2043	314,62
13,068	22,3146	0,3064	307,64		13,068	37,1778	13,0445	337,35	cooling	13,068	34,0016	11,7152	311,53
13,07	22,5765	-4,1479	373,57		13,07	38,6352	18,4796	281,75	cooling	13,07	33,8875	15,7974	252,87
13,072	23,6829	1,9575	303,69		13,072	37,814	21,6832	225,48	cooling	13,072	33,6468	15,9949	246,75
13,074	22,1294	0,2244	306,2		13,074	38,321	21,0018	242,1		13,074	33,8677	11,4614	313,21
13,076	22,991	5,6952	241,77		13,076	38,0714	22,391	219,19		13,076	34,3769	14,4767	278,18
13,078	22,0578	8,6059	188,04		13,078	39,0474	16,4274	316,19		13,078	33,2727	8,8352	341,6
13,08	21,7237	2,6042	267,26		13,08	39,0014	18,6782	284,09		13,08	34,7024	13,732	293,14
13,082	22,18	2,0867	280,87		13,082	38,4837	17,7583	289,71		13,082	33,6951	16,4332	241,3
13,084	22,612	5,5345	238,72		13,084	39,5698	21,4137	253,79		13,084	33,6942	14,2643	271,6
13,086	22,7062	0,3357	312,71		13,086	39,2579	17,2516	307,62		13,086	33,9668	15,3957	259,6
13,088	22,5633	3,8597	261,45		13,088	39,0812	22,398	233,21		13,088	34,097	13,3909	289,44
13,09	22,09	2,6377	271,91		13,09	38,2164	18,9491	269,33		13,09	33,7141	11,6639	308,23
13,092	22,2996	0,1152	310,11		13,092	40,0979	23,018	238,75		13,092	34,0972	14,5171	273,7
13,094	23,3515	6,5047	235,49		13,094	38,3797	19,5683	262,95		13,094	33,4381	11,6689	304,3
13,096	22,1188	2,1199	279,55		13,096	38,9567	18,4225	287,04		13,096	34,2139	10,959	325,07
13,098	23,4678	-1,8252	353,56		13,098	38,2311	15,9295	311,74		13,098	33,6595	13,4139	283
13,1	22,9424	0,6664	311,39		13,1	39,141	18,6371	286,61		13,1	34,2943	7,5496	373,85
13,102	22,6236	-0,2325	319,49		13,102	37,7782	20,3495	243,63		13,102	35,0097	14,0617	292,82
13,104	22,7318	-0,9816	331,48		13,104	39,2348	23,5892	218,7		13,104	33,9639	12,0571	306,23
13,106	23,3383	1,1708	309,87		13,106	38,0985	23,6852	201,48	heating	13,106	34,2727	11,6288	316,53
13,108	22,9438	-4,3897	382,08		13,108	38,3171	17,462	291,52	heating	13,108	35,005	14,2896	289,57
13,11	22,9787	0,7149	311,21		13,11	38,6642	17,5579	295,03	heating	13,11	33,8501	12,4071	299,74
13,112	22,5156	4,2991	254,64		13,112	39,3747	14,2344	351,42	heating	13,112	34,0061	12,0714	306,61
13,114	22,1403	0,6897	299,85		13,114	39,0624	19,2145	277,45	heating	13,114	34,0652	17,4174	232,71
13,116	22,7154	-0,7746	328,36		13,116	38,549	18,7838	276,29	heating	13,116	33,3129	10,6755	316,44
13,118	22,574	-1,3715	334,72		13,118	40,1509	24,5031	218,73	heating	13,118	33,7373	11,5735	309,82
13,12	23,68	0,0209	330,72		13,12	39,2307	21,9599	241,42	heating	13,12	33,102	12,3995	289,39
13,122	23,2209	5,167	252,37		13,122	39,2418	25,5029	192,05	heating	13,122	34,2996	9,7448	343,24
13,124	22,8201	4,706	253,21		13,124	38,2545	20,6267	246,41	heating	13,124	33,8354	14,8451	265,46
13,126	22,7191	1,4046	297,94		13,126	39,4849	20,8663	260,26	heating	13,126	33,303	12,9514	284,48
13,128	23,4385	3,5356	278,21		13,128	37,3691	15,0046	312,62	heating	13,128	33,2498	11,6704	301,65
13,13	22,6298	-0,6991	326,1		13,13	38,6356	16,8916	303,95	heating	13,13	34,2943	15,3492	264,82
13,132	22,4769	-1,9411	341,33		13,132	37,6615	20,5794	238,78	heating	13,132	32,5606	10,4611	308,92
13,134	22,1525	1,4699	289,11		13,134	38,0873	23,027	210,52	heating	13,134	34,0343	9,0837	348,77

13,136	21,5459	1,9953	273,29		13,136	37,9136	25,5751	172,47	heating	13,136	33,5801	12,5883	293,43	
13,138	21,7065	2,1901	272,81		13,138	39,5181	21,9762	245,21	heating	13,138	33,4226	7,9498	356,07	
13,14	21,8842	1,9248	279		13,14	38,9756	18,4205	287,33	heating	13,14	33,7235	13,9109	276,95	
13,142	22,3284	-3,1865	356,66		13,142	39,0731	12,1364	376,54	heating	13,142	33,5994	14,2874	269,95	
13,144	22,5522	-0,8036	326,48		13,144	40,5115	17,5743	320,63	heating	13,144	33,9344	9,8024	337,33	
13,146	22,1326	3,5593	259,63		13,146	40,3428	16,4863	333,48	heating	13,146	33,8198	12,3212	300,52	
13,148	22,0458	1,0154	293,97		13,148	40,2518	23,3082	236,85	heating	13,148	33,7804	14,5189	269,25	
13,15	22,7145	0,5092	310,4		13,15	39,2803	20,4635	263,03	heating	13,15	33,3648	10,4264	320,65	
13,152	22,5913	0,5238	308,47		13,152	40,8309	23,2432	245,85	heating	13,152	33,9023	14,9121	265,45	
13,154	22,7536	-0,0479	318,73		13,154	38,6163	15,7702	319,36	heating	13,154	32,6281	12,1142	286,75	
13,156	22,6389	5,1973	243,81		13,156	39,6565	17,757	306,12	heating	13,156	33,9615	12,5791	298,89	
13,158	21,8108	1,7632	280,24		13,158	38,6223	15,7496	319,73	heating	13,158	33,53	14,7779	262,13	
13,16	21,5879	-5,1047	373,12		13,16	39,2767	15,3798	334,04	heating	13,16	33,3061	10,834	314,13	
13,162	22,5001	-0,4635	321		13,162	38,2267	14,8458	326,83	heating	13,162	34,0383	10,5273	328,65	
13,164	21,9286	0,3295	301,92		13,164	39,6344	16,6283	321,59	heating	13,164	34,495	14,5636	278,61	
13,166	22,2697	-0,1804	313,82		13,166	38,6982	15,3993	325,68	heating	13,166	33,7509	15,7516	251,6	
13,168	21,779	3,1036	261,05		13,168	38,9451	14,4118	342,94	heating	13,168	33,6654	15,2402	257,56	
13,17	21,5189	0,2566	297,22		13,17	39,4744	17,6365	305,26	heating	13,17	33,9098	16,7986	239,19	
13,172	21,9115	-1,2027	323,1		13,172	40,0218	13,6301	368,92	heating	13,172	33,0419	11,3669	302,98	
13,174	22,4944	1,8913	288		13,174	39,9554	19,429	286,93		13,174	33,6206	15,0498	259,59	
13,176	21,9323	-4,7731	373,3		13,176	39,1916	15,479	331,47		13,176	33,094	14,5834	258,75	
13,178	22,5191	-2,1811	345,27		13,178	40,845	20,9537	278,05		13,178	33,2533	13,1747	280,67	heating
13,18	21,7632	0,3057	299,94		13,18	39,6387	19,391	283,03		13,18	32,9147	11,3304	301,72	heating
13,182	21,4322	-3,5531	349,26		13,182	39,5655	23,9004	218,97		13,182	33,6361	16,589	238,29	heating
13,184	22,3456	-1,841	338,09		13,184	39,1404	22,2938	235,49	heating	13,184	33,2836	14,029	269,15	heating
13,186	22,1324	-2,7743	348,16		13,186	40,0982	25,3077	206,75	heating	13,186	33,427	15,0509	256,87	heating
13,188	22,7259	-2,6017	354,04	cooling	13,188	38,5596	18,6318	278,56	heating	13,188	32,9408	13,7299	268,54	heating
13,19	22,7565	3,761	265,53	cooling	13,19	39,8946	20,8939	265,6	heating	13,19	33,4041	12,7331	288,95	heating
13,192	22,6731	1,2814	299,02	cooling	13,192	38,7882	17,363	299,49	heating	13,192	33,3641	14,1333	268,82	heating
13,194	23,3386	-2,3371	358,91	cooling	13,194	39,2018	13,9302	353,26	heating	13,194	33,0737	12,1317	292,74	heating
13,196	24,4683	1,7161	318,04	cooling	13,196	39,5867	17,7	305,94	heating	13,196	33,488	9,3957	336,77	heating
13,198	24,4522	-0,4856	348,59	cooling	13,198	40,5542	16,3393	338,49	heating	13,198	33,9503	10,0998	333,39	heating
13,2	24,9959	4,3646	288,4	cooling	13,2	39,7422	19,2302	286,73	heating	13,2	33,5493	12,843	289,44	heating
13,202	24,4166	5,5394	263,87	cooling	13,202	39,2038	17,0363	309,87	heating	13,202	33,7668	11,6771	308,78	heating
13,204	24,1825	0,0106	337,89	cooling	13,204	39,9021	16,4948	327,2	heating	13,204	34,1982	12,9178	297,47	heating
13,206	25,1426	1,9656	323,98	cooling	13,206	40,5035	12,7664	387,72		13,206	33,5801	7,9582	358,16	heating
13,208	24,3099	1,1263	324,07	cooling	13,208	39,4386	18,1998	296,89		13,208	34,0303	11,3221	317,43	
13,21	24,8931	0,9601	334,55	cooling	13,21	39,5735	14,2721	353,68		13,21	33,7014	12,9831	289,61	
13,212	24,5309	5,4321	266,97	cooling	13,212	40,8685	22,059	262,93		13,212	33,5665	13,7816	276,56	
13,214	23,9374	3,0335	292,21	cooling	13,214	39,2158	15,9051	325,85		13,214	33,0986	11,698	299,15	
13,216	24,3343	2,1654	309,89	cooling	13,216	39,5503	14,2878	353,13		13,216	33,8284	14,0168	276,94	
13,218	24,4552	4,2201	282,86	cooling	13,218	39,7151	12,72	377,35		13,218	33,4087	11,3722	308,04	
13,22	24,4549	1,1903	325,21	cooling	13,22	39,8118	11,6661	393,44		13,22	33,8944	12,1702	303,67	
13,222	24,5411	8,4211	225,33	cooling	13,222	38,8371	11,7998	377,94		13,222	33,3258	11,4311	306,06	
13,224	24,0836	10,2881	192,84		13,224	39,9404	16,3474	329,8	cooling	13,224	34,1204	12,163	306,93	
13,226	23,8059	2,8505	292,93		13,226	38,8111	13,7036	350,97	cooling	13,226	33,6903	14,2306	272,02	
13,228	24,851	1,3625	328,33		13,228	39,3103	13,5717	359,79	cooling	13,228	33,5441	13,8702	275,01	
13,23	23,6213	-4,2104	389,05		13,23	38,9746	16,2264	317,99	cooling	13,23	33,1994	11,0132	310,13	
13,232	24,6908	-1,7308	369,33		13,232	40,5576	13,7776	374,34	cooling	13,232	33,8189	13,4881	284,19	
13,234	24,0096	5,0542	264,97		13,234	39,4333	19,8849	273,26	cooling	13,234	32,6692	13,9896	261,11	
13,236	23,3816	0,4968	319,9		13,236	38,9217	17,1045	304,97	cooling	13,236	33,1105	12,1281	293,3	
13,238	23,7909	-3,3069	378,79		13,238	39,9526	17,0475	320,18	cooling	13,238	33,2485	11,4987	304,03	
13,24	23,9995	-2,1821	365,98	cooling	13,24	39,687	13,8105	361,71	cooling	13,24	33,0051	6,5074	370,4	
13,242	23,8531	-6,6386	426,23	cooling	13,242	38,7325	15,7742	320,92	cooling	13,242	33,3818	7,9797	355,08	
13,244	23,8868	0,3561	328,92	cooling	13,244	38,8043	13,6962	350,97	cooling	13,244	33,3239	12,8209	286,6	
13,246	23,1857	1,0775	309,04	cooling	13,246	39,7196	18,171	301,22	cooling	13,246	33,242	13,0321	282,51	

13,248	23,0738	-3,162	366,74	cooling	13,248	38,0209	14,7497	325,3	cooling	13,248	32,9325	10,2599	316,93	
13,25	23,9893	2,521	300,09	cooling	13,25	39,2025	17,6659	301,05	cooling	13,25	33,714	11,776	306,66	
13,252	22,9487	1,7802	295,9	cooling	13,252	38,3757	15,6052	318,3	cooling	13,252	32,8772	9,6488	324,7	
13,254	24,2006	2,318	305,89	cooling	13,254	38,8938	12,6741	366,51	cooling	13,254	33,2209	11,9143	297,83	
13,256	23,3778	5,9082	244,2	cooling	13,256	37,8498	12,3687	356,19	cooling	13,256	32,4478	12,2224	282,72	
13,258	22,8988	1,0895	304,86	cooling	13,258	39,3018	13,7184	357,62	cooling	13,258	33,316	13,2366	280,68	
13,26	23,1562	0,1997	320,9	cooling	13,26	38,051	14,8765	323,95	cooling	13,26	32,8681	13,2455	274,3	
13,262	23,4701	4,8126	260,8	cooling	13,262	38,2113	15,3499	319,57	cooling	13,262	33,2036	13,3677	277,28	
13,264	22,8928	2,9225	279,16	cooling	13,264	39,1433	18,7427	285,17	cooling	13,264	33,6478	12,1455	300,57	
13,266	23,294	7,0635	226,88	cooling	13,266	40,0545	16,1615	333,99	cooling	13,266	33,6941	14,7955	264,17	
13,268	22,6826	5,0584	246,36		13,268	39,3589	18,4	292,97	cooling	13,268	33,0758	16,112	237,13	
13,27	22,9897	-0,069	322,33		13,27	38,9829	15,3061	330,97	cooling	13,27	33,1893	15,7013	244,46	
13,272	23,4425	2,1689	297,37		13,272	40,073	17,156	320,35	cooling	13,272	33,5122	15,0778	257,69	heating
13,274	23,5933	3,6203	279,19		13,274	38,6978	13,0168	358,98	cooling	13,274	32,7282	12,1853	287,16	heating
13,276	24,523	3,0082	300,75		13,276	38,7367	18,0462	289,22	cooling	13,276	33,3805	14,0076	270,81	heating
13,278	23,7824	4,9143	263,75		13,278	38,0088	17,4687	287,12	cooling	13,278	32,9273	15,4476	244,34	heating
13,28	23,5491	1,7566	304,63		13,28	38,7092	20,9248	248,6	cooling	13,28	33,3963	15,9842	243,39	heating
13,282	23,5757	0,6307	320,74		13,282	37,0099	17,1307	277,88	cooling	13,282	32,5396	12,3104	282,78	heating
13,284	24,3193	5,1963	267,31		13,284	38,2678	16,1081	309,76	cooling	13,284	34,4651	15,61	263,57	heating
13,286	23,2115	1,5895	302,24		13,286	37,7434	16,1915	301,26	cooling	13,286	33,4575	13,0086	285,85	heating
13,288	23,7966	0,8293	321,05		13,288	37,5738	16,8222	290,08	cooling	13,288	33,892	13,3441	287,23	heating
13,29	23,0445	-0,1624	324,4		13,29	37,8218	19,6208	254,42	cooling	13,29	33,1593	12,081	294,64	heating
13,292	23,0108	-2,3422	354,4		13,292	38,9444	20,6655	255,51	cooling	13,292	34,149	13,0817	294,49	heating
13,294	23,0498	-1,0576	336,99		13,294	37,6686	19,557	253,17		13,294	33,3473	10,3346	321,68	heating
13,296	23,719	2,8753	291,36		13,296	37,4045	15,5123	306,02		13,296	33,8892	12,4729	299,37	heating
13,298	24,0341	0,2597	332,33		13,298	38,9294	20,1418	262,62		13,298	33,9917	8,8927	350,85	heating
13,3	23,7766	4,2918	272,37		13,3	38,9082	16,1015	318,8		13,3	34,0126	11,0165	321,45	heating
13,302	23,3673	3,1416	282,73		13,302	38,6208	19,8877	261,86		13,302	33,7005	15,1941	258,69	heating
13,304	23,3495	0,3545	321,44		13,304	38,3367	16,4616	305,78		13,304	33,0492	12,7166	284,22	heating
13,306	24,0931	5,5306	259,48		13,306	39,3257	16,6691	316,71		13,306	34,439	13,6625	290,42	heating
13,308	22,9745	4,4395	259,09		13,308	37,827	14,7843	322,1		13,308	32,9097	12,5843	284,12	heating
13,31	24,1702	6,1548	251,83		13,31	38,3821	19,8351	259,26		13,31	34,0608	13,178	291,91	heating
13,312	23,4523	6,7984	232,8		13,312	37,9972	17,9626	280,05		13,312	33,5788	15,4636	253,22	heating
13,314	23,2532	0,2558	321,47		13,314	37,8649	16,4166	299,82		13,314	34,1251	14,6743	271,89	heating
13,316	23,8586	0,3871	328,1		13,316	36,9581	13,2037	332,05		13,316	33,3086	7,2235	364,63	heating
13,318	24,4679	5,7201	262,07		13,318	38,2956	12,3002	363,38		13,318	35,3545	13,5449	304,87	heating
13,32	23,8669	1,2758	315,79		13,32	37,7799	15,5744	310,4		13,32	34,3174	11,3457	321,11	heating
13,322	24,3161	2,6576	302,75		13,322	37,2793	17,7653	272,78		13,322	34,3901	12,8984	300,42	
13,324	23,6851	-0,7341	341,34		13,324	38,1108	21,4481	232,92		13,324	34,072	13,1033	293,11	
13,326	23,9091	-4,3697	395,3		13,326	38,9344	16,6065	312,11		13,326	34,2762	11,1411	323,4	
13,328	24,3299	1,4112	320,37		13,328	38,1663	14,5337	330,35		13,328	34,3639	10,1706	338,19	
13,33	23,9063	3,5456	284,61		13,33	37,8501	11,1574	373,12		13,33	34,0876	13,3606	289,73	
13,332	24,8872	2,9376	306,82		13,332	39,3529	17,724	302,34		13,332	34,6003	10,1564	341,69	
13,334	24,3168	4,4595	277,58		13,334	38,1069	17,7456	284,62		13,334	34,4665	8,5495	362,28	
13,336	24,0479	-0,178	338,64		13,336	38,0003	22,6214	214,97		13,336	34,4002	10,0741	340,04	
13,338	24,3409	-1,8768	366,48		13,338	37,7481	20,8346	236,43		13,338	33,9589	8,7289	352,68	
13,34	24,9419	3,0556	305,94		13,34	38,2522	19,8629	257,05		13,34	35,0474	12,1363	320,26	
13,342	23,9525	1,8288	309,26		13,342	37,0174	17,8007	268,62		13,342	33,8813	9,9457	334,58	
13,344	24,6444	5,7561	264,03		13,344	37,6812	19,8024	249,92		13,344	34,3955	10,4896	334,17	
13,346	23,9554	7,5646	229,12		13,346	37,3426	17,9395	271,23		13,346	34,128	12,7436	298,92	
13,348	23,9061	4,6576	269,07		13,348	37,2501	15,9899	297,19		13,348	34,0641	14,3218	275,97	
13,35	23,7694	2,7595	293,69		13,35	37,1466	17,8168	270,2		13,35	33,4716	12,3887	294,71	
13,352	24,3515	3,2568	294,87		13,352	38,3409	18,1991	281,55		13,352	34,4484	16,9923	244,01	
13,354	24,3852	-0,9554	354,22		13,354	37,9249	22,6738	213,19		13,354	34,0626	16,634	243,63	
13,356	24,1321	4,1969	278,66		13,356	37,5137	18,2314	269,54	heating	13,356	34,1744	17,1667	237,74	
13,358	23,4394	5,8911	245,3		13,358	39,0774	18,5028	287,6	heating	13,358	34,0183	15,9384	252,73	

13,36	23,4136	4,1397	269,42		13,36	39,4502	15,6427	332,79	heating	13,36	34,1126	11,0386	322,54	
13,362	24,0781	5,2969	262,53		13,362	38,5956	18,0248	287,55	heating	13,362	34,4095	9,8918	342,72	
13,364	23,1652	1,7405	299,49		13,364	38,5417	19,3873	267,75	heating	13,364	34,3811	14,1913	282,22	
13,366	24,4558	0,4115	336,1		13,366	39,7422	23,6052	225,57	heating	13,366	34,6385	13,771	291,7	
13,368	24,0978	4,2539	277,39		13,368	38,6142	17,5797	294,03	heating	13,368	34,5795	11,9283	316,63	
13,37	24,0728	3,0549	293,8		13,37	38,3185	19,7693	259,29	heating	13,37	34,37	12,9103	299,97	
13,372	24,1726	1,7773	313,05		13,372	37,5396	17,2653	283,4	heating	13,372	33,9902	11,5377	313,85	
13,374	25,6393	5,3396	283,76		13,374	38,5501	18,3306	282,64	heating	13,374	34,979	15,2294	276,07	
13,376	24,7455	-4,0829	402,98		13,376	37,4815	20,4684	237,82	heating	13,376	34,1645	13,6102	287,32	
13,378	25,1626	-1,1472	367,77		13,378	38,1408	21,2362	236,3	heating	13,378	34,1689	11,2564	320,28	
13,38	24,4433	0,937	328,58		13,38	37,8609	19,6927	253,96	heating	13,38	34,1908	11,4752	317,53	
13,382	24,4045	-0,3777	346,42		13,382	37,9712	20,5388	243,68	heating	13,382	34,1104	12,7033	299,24	
13,384	24,9554	3,9407	293,75		13,384	37,0264	18,4097	260,23	heating	13,384	33,6179	9,1671	341,79	
13,386	24,1668	3,0027	295,84		13,386	38,494	19,4196	266,63	heating	13,386	34,6411	11,912	317,72	
13,388	25,7236	-3,1521	403,64		13,388	37,5526	22,7289	207,21	heating	13,388	34,3233	10,3847	334,63	
13,39	25,1911	1,4882	331,33	cooling	13,39	37,0371	19,7338	241,87	heating	13,39	34,1555	10,3575	332,66	
13,392	24,769	0,7062	336,36	cooling	13,392	38,1648	21,8592	227,93	heating	13,392	34,0997	11,9737	309,29	
13,394	24,7468	1,17	329,57	cooling	13,394	38,8646	18,5425	284,07	heating	13,394	34,0654	11,344	317,61	
13,396	26,3021	10,0732	226,86	cooling	13,396	38,4605	19,4424	265,85	heating	13,396	34,4043	12,0064	313,09	
13,398	24,393	2,1343	311,14	cooling	13,398	38,5145	20,4859	252,01	heating	13,398	33,8146	13,4204	285,08	
13,4	25,6164	3,6346	307,27	cooling	13,4	39,8364	25,1201	205,71	heating	13,4	34,1176	10,5552	329,37	
13,402	24,6372	2,9819	302,71	cooling	13,402	39,0012	21,8208	240,16	heating	13,402	34,131	8,8191	353,82	
13,404	24,6202	-1,788	369,15		13,404	38,7459	23,4214	214,21	heating	13,404	34,0163	8,9343	350,61	
13,406	24,6513	-0,9026	357,21	cooling	13,406	38,2081	17,4507	290,16	heating	13,406	33,6956	6,4296	381,14	cooling
13,408	25,0558	2,5795	314,19	cooling	13,408	38,9239	16,0617	319,58	heating	13,408	34,7151	9,9315	346,44	cooling
13,41	25,6929	-2,4623	393,57	cooling	13,41	37,3065	14,2622	322,12	heating	13,41	34,1529	9,3922	346,12	cooling
13,412	24,8733	2,0015	319,71	cooling	13,412	37,7442	15,4832	311,18	heating	13,412	33,8352	5,9338	390,02	cooling
13,414	24,3504	0,4018	334,77	cooling	13,414	37,7059	16,8928	290,94	heating	13,414	34,2707	8,9564	353,86	cooling
13,416	23,9395	-1,8135	359,99	cooling	13,416	38,0951	19,8164	255,51	heating	13,416	33,6677	11,4767	310,2	cooling
13,418	24,3831	1,447	320,61	cooling	13,418	37,6765	19,7772	250,21	heating	13,418	33,5219	10,1943	326,09	cooling
13,42	22,7779	-1,6353	341,26	cooling	13,42	39,2944	19,8045	272,44	heating	13,42	34,009	14,1992	276,91	cooling
13,422	23,9951	-1,1074	350,89	cooling	13,422	38,5578	18,8382	275,65	heating	13,422	33,7913	12,5042	297,56	cooling
13,424	22,9964	3,4959	272,59	cooling	13,424	38,2744	13,6071	344,81	heating	13,424	33,775	8,5415	352,73	cooling
13,426	22,8227	5,2228	246,02	cooling	13,426	39,0507	16,8562	310,25	heating	13,426	33,7959	11,391	313,19	cooling
13,428	22,5932	4,0799	258,79	cooling	13,428	39,5981	19,1441	285,92	heating	13,428	33,3716	8,2851	350,67	cooling
13,43	23,5516	4,1389	271,36	cooling	13,43	38,3421	20,4254	250,45	heating	13,43	33,8667	8,3535	356,64	cooling
13,432	23,0471	-0,9008	334,76	cooling	13,432	38,086	18,7717	269,99	heating	13,432	33,8478	12,7423	295,02	cooling
13,434	23,0053	1,3172	303,17	cooling	13,434	39,3328	21,0239	255,93	heating	13,434	33,659	11,0699	315,76	cooling
13,436	22,8142	2,9675	277,43	cooling	13,436	38,7406	15,0688	330,9	heating	13,436	34,5701	12,6376	306,58	cooling
13,438	22,6929	-0,3312	321,84	cooling	13,438	38,6012	17,8205	290,48	heating	13,438	33,6651	15,2809	256,98	cooling
13,44	23,1289	-1,6629	346,55	cooling	13,44	38,1453	15,3905	318,08	heating	13,44	33,4549	11,9948	299,98	cooling
13,442	23,4425	1,5488	306,04	cooling	13,442	39,1822	14,237	348,7	heating	13,442	33,901	14,4132	272,41	cooling
13,444	24,4162	1,5664	319,41	cooling	13,444	37,8888	15,313	315,58	heating	13,444	33,4625	13,696	276,31	cooling
13,446	24,0124	4,9239	266,83	cooling	13,446	38,4181	17,6973	289,65	heating	13,446	33,142	8,8952	338,93	cooling
13,448	23,6754	0,7787	320,06	cooling	13,448	38,2713	18,3468	278,51	heating	13,448	33,6926	14,3283	270,68	cooling
13,45	23,4929	-5,2289	401,49	cooling	13,45	38,4696	20,1874	255,56	heating	13,45	32,8913	14,3733	258,85	cooling
13,452	24,9789	0,4526	342,84	cooling	13,452	37,9735	18,9515	265,9	heating	13,452	33,0888	12,4113	289,04	cooling
13,454	23,7607	0,7521	321,63	cooling	13,454	39,2171	16,855	312,59	heating	13,454	33,4181	13,5833	277,26	
13,456	24,1001	3,5847	286,77	cooling	13,456	38,5967	16,5229	308,56	heating	13,456	33,4663	10,5994	319,65	
13,458	23,7351	7,1004	232,53	cooling	13,458	37,9358	9,1761	402,02	heating	13,458	34,0688	10,6836	326,89	
13,46	23,3428	4,0578	269,58	cooling	13,46	39,4691	13,8194	358,54	heating	13,46	33,4326	14,4695	265,08	
13,462	23,1248	0,0756	322,19	cooling	13,462	39,4507	16,3235	323,28		13,462	33,4069	12,4516	292,93	
13,464	23,6085	1,0758	314,97	cooling	13,464	38,6614	18,1641	286,52		13,464	33,5277	13,3037	282,7	
13,466	23,7755	-4,3399	393,01	cooling	13,466	38,4482	16,6953	304,07		13,466	33,1054	14,3265	262,5	
13,468	23,6101	1,0621	315,19	cooling	13,468	39,3797	17,3273	308,26		13,468	33,085	11,5887	300,49	
13,47	23,0666	4,1992	263,74	cooling	13,47	38,5601	12,0041	371,21		13,47	33,9949	12,1124	305,88	

13,472	22,1423	-1,7353	333,77	cooling	13,472	38,6972	15,5925	322,97		13,472	32,9849	10,9739	307,68	
13,474	23,0242	-1,2725	339,63	cooling	13,474	38,1806	14,9885	324,19		13,474	33,1614	10,8169	312,34	
13,476	21,1672	-3,11	339,36	cooling	13,476	38,974	16,9485	307,88		13,476	33,2775	12,3225	292,92	
13,478	21,9912	0,154	305,25	cooling	13,478	38,0651	20,8091	241,21		13,478	33,8646	13,2458	288,22	
13,48	21,3609	5,6405	219,75	cooling	13,48	38,5681	21,2133	242,59		13,48	33,5251	12,9596	287,48	
13,482	20,999	2,0914	264,3	cooling	13,482	38,5945	20,8294	248,33		13,482	33,3812	13,9828	271,16	
13,484	20,8716	-2,2716	323,51	cooling	13,484	38,2271	15,9449	311,47		13,484	33,0157	12,25	290,27	
13,486	21,9552	3,9734	251,36	cooling	13,486	38,8744	14,8048	336,46		13,486	32,8229	11,7354	294,77	
13,488	21,5601	1,6747	277,97		13,488	39,9786	16,6193	326,53		13,488	32,439	8,6342	332,76	
13,49	21,3884	3,4611	250,6		13,49	39,3963	21,0064	257,06		13,49	33,5421	10,5854	320,9	
13,492	21,0088	2,5561	257,94		13,492	39,1291	18,0101	295,21		13,492	33,932	12,335	301,89	
13,494	20,6851	-1,683	312,67		13,494	40,5285	21,8255	261,44		13,494	33,549	13,4082	281,54	
13,496	21,5629	1,9816	273,72		13,496	39,6583	16,0413	330,13		13,496	33,9555	13,0177	292,68	
13,498	20,6465	2,5738	252,63		13,498	39,2005	16,6848	314,74		13,498	33,7206	9,3263	341	
13,5	21,9584	-2,7845	345,87		13,5	38,2421	15,9848	311,12		13,5	34,1602	6,9814	379,92	
13,502	21,8209	-2,1012	334,4		13,502	38,5838	14,9114	330,9		13,502	33,6663	6,6907	377,08	
13,504	22,1512	-0,3034	313,88		13,504	37,3346	13,2562	336,58		13,504	33,8578	7,5798	367,33	
13,506	22,0005	-1,0607	322,36		13,506	37,5513	15,2539	311,68		13,506	33,1254	9,3972	331,69	cooling
13,508	23,5527	3,2561	283,72		13,508	37,146	14,8599	311,53		13,508	33,1304	13,3187	276,94	cooling
13,51	22,7083	-2,2612	349,04		13,51	37,4075	15,4221	307,32		13,51	32,596	11,5864	293,68	cooling
13,512	23,1282	0,4014	317,69		13,512	36,9771	16,2448	289,81		13,512	33,9558	16,6447	241,98	cooling
13,514	22,8692	5,6884	240,16		13,514	38,1994	14,8691	326,12		13,514	33,2634	16,7271	231,15	
13,516	22,4665	2,5776	278,02		13,516	37,9055	16,1541	304,05		13,516	33,351	14,4626	264,03	
13,518	22,842	-1,1191	334,94		13,518	37,6434	11,3764	367,17		13,518	33,3353	13,907	271,58	
13,52	23,0039	1,6741	298,16		13,52	38,8836	13,2576	358,21		13,52	32,9374	12,2035	289,83	
13,522	23,4871	-0,1804	330,84		13,522	39,2255	12,6246	371,84		13,522	33,0463	10,2393	318,81	
13,524	22,9374	2,917	279,86		13,524	38,9745	15,75	324,64		13,524	33,1991	14,7952	257,26	
13,526	22,5705	1,5288	294,13		13,526	38,8263	15,5185	325,81	cooling	13,526	32,8209	13,257	273,47	
13,528	21,978	-5,6309	385,93		13,528	39,9943	18,646	298,42	cooling	13,528	32,793	12,6218	281,96	
13,53	23,7001	-0,1342	333,17		13,53	39,1247	16,3835	317,89	cooling	13,53	33,3185	13,945	270,81	
13,532	22,1519	-1,3263	328,19		13,532	39,4601	19,0719	285	cooling	13,532	32,9839	9,472	328,66	
13,534	22,4919	-1,9245	341,31		13,534	38,7461	17,3464	299,13		13,534	33,7722	12,9982	290,39	
13,536	22,0706	-1,6389	331,42	cooling	13,536	38,8835	14,524	340,51		13,536	33,1454	17,3861	220,29	
13,538	21,8093	-2,5627	340,68	cooling	13,538	37,8257	15,6608	309,83	cooling	13,538	33,3685	15,538	249,24	
13,54	21,9376	-0,4111	312,4	cooling	13,54	38,3058	16,4077	306,1	cooling	13,54	33,4219	15,8879	245,1	
13,542	21,8497	5,9445	222,33	cooling	13,542	37,9491	19,0902	263,62	cooling	13,542	33,1716	15,4291	248,01	
13,544	22,0274	2,6478	270,9		13,544	37,6419	17,7278	278,37		13,544	33,1012	9,4211	331,01	heating
13,546	21,4315	3,8798	245,35		13,546	38,5973	20,3393	255,22		13,546	33,6662	12,0345	302,38	
13,548	21,3676	3,782	245,82		13,548	39,1052	16,9161	310,17		13,548	32,8652	11,4878	298,82	
13,55	20,6649	-1,6465	311,88		13,55	38,902	19,9515	264,9		13,55	33,1311	11,6415	300,39	
13,552	22,0834	1,5115	287,56		13,552	38,1997	14,6456	329,25		13,552	33,1476	17,4606	219,28	
13,554	20,6758	-0,0398	289,57		13,554	40,12	15,8205	339,67		13,554	32,6987	18,2488	201,99	heating
13,556	22,051	2,9185	267,44		13,556	39,5974	12,9508	372,48		13,556	32,9254	16,5751	228,55	heating
13,558	21,8895	4,4788	243,38		13,558	39,4806	17,2282	311,06		13,558	33,1893	17,7474	215,86	heating
13,56	21,8383	-0,7388	315,59		13,56	38,7391	16,0453	317,23		13,56	32,3648	12,615	276,07	heating
13,562	22,1652	-6,1259	395,47		13,562	39,6328	18,3597	297,37		13,562	33,0137	11,2414	304,34	heating
13,564	22,6963	-4,1247	374,92		13,564	37,8242	16,6762	295,62		13,564	33,0075	11,5496	299,95	heating
13,566	23,1513	-6,9183	420,33		13,566	38,0593	17,7514	283,87		13,566	33,0564	8,3451	345,43	heating
13,568	22,5855	-1,6837	339,25	cooling	13,568	37,2446	15,8781	298,67		13,568	33,4677	12,0209	299,79	heating
13,57	22,4613	0,6079	305,48	cooling	13,57	37,5904	13,3956	338,21		13,57	33,5214	16,6233	236,21	heating
13,572	22,0528	-0,2352	311,55	cooling	13,572	37,1003	14,3687	317,75		13,572	33,4887	15,0741	257,41	heating
13,574	22,7653	2,6106	281,73	cooling	13,574	38,2337	14,8201	327,29		13,574	33,8237	15,1579	260,92	heating
13,576	21,5838	0,9791	288,02	cooling	13,576	37,8715	18,2218	274,67		13,576	33,2324	12,6152	288,2	heating
13,578	22,6491	-0,9398	329,74		13,578	37,5866	16,2479	298,28		13,578	33,1693	9,9965	323,92	heating
13,58	22,2012	0,8861	297,95	cooling	13,58	38,649	18,5802	280,53		13,58	33,0962	14,3962	261,4	heating
13,582	22,0773	1,6251	285,89		13,582	39,069	14,1321	348,58		13,582	32,0896	13,0056	266,77	heating

13,584	21,9169	0,6678	297,03		13,584	38,555	18,1561	285,15		13,584	32,7587	12,8412	278,42	heating
13,586	22,9781	3,1697	276,89		13,586	37,8286	16,1025	303,7		13,586	32,6515	13,9747	261,07	heating
13,588	22,5395	-1,3537	333,99		13,588	39,5551	20,4893	266,51		13,588	32,7878	11,9015	291,96	heating
13,59	22,4942	1,822	288,97		13,59	38,6863	18,5082	282,06		13,59	33,0345	10,6456	312,96	heating
13,592	22,3745	4,4251	250,91		13,592	38,7706	22,9392	221,3		13,592	33,5375	12,9862	287,28	heating
13,594	22,1032	-0,0196	309,24		13,594	38,1884	20,9574	240,86		13,594	33,1589	10,4592	317,31	heating
13,596	22,4305	-2,9157	354,3		13,596	39,3097	21,7677	245,21		13,596	33,8957	13,2365	288,79	heating
13,598	22,2724	-1,5298	332,72		13,598	37,8427	18,6068	268,89		13,598	33,7366	12,6736	294,43	heating
13,6	22,8743	-1,0825	334,88		13,6	38,9312	18,3345	287,91		13,6	33,6146	8,6632	348,78	heating
13,602	22,3884	3,8538	259,09		13,602	38,2921	19,401	264,07		13,602	33,6088	11,0958	314,7	heating
13,604	22,0684	3,929	253,56		13,604	38,0439	17,0389	293,62		13,604	33,3264	11,9486	298,83	heating
13,606	21,953	-0,0495	307,56		13,606	37,9003	18,4307	272,16		13,606	33,8667	10,5905	325,37	heating
13,608	22,9863	1,9815	293,62		13,608	39,0103	17,7094	297,76		13,608	34,0472	13,0165	293,98	heating
13,61	21,8472	0,6408	296,43		13,61	38,1781	18,4533	275,72		13,61	33,7959	11,6247	309,92	heating
13,612	22,32	0,48	305,29		13,612	37,7068	12,7515	348,84		13,612	34,1787	11,1154	322,39	
13,614	22,089	1,9321	281,76		13,614	39,3012	16,7355	315,43		13,614	34,2403	15,8884	256,53	
13,616	22,0053	1,0824	292,47		13,616	38,8864	11,4948	382,89		13,616	33,0952	13,1852	278,31	
13,618	22,1174	1,6124	286,63		13,618	38,771	16,8531	306,38		13,618	33,623	14,3754	269,05	heating
13,62	22,2334	2,969	269,29		13,62	37,9283	15,696	310,77		13,62	32,7183	14,3223	257,15	heating
13,622	22,53	-1,652	338,03		13,622	39,2752	18,1905	294,73		13,622	32,6866	11,8291	291,56	heating
13,624	22,3805	1,3855	293,48		13,624	37,2745	16,2646	293,69		13,624	32,9303	13,1381	276,66	heating
13,626	22,2929	3,7426	259,3		13,626	37,9222	19,4476	258,25		13,626	33,0385	14,2964	261,99	heating
13,628	22,0497	3,8293	254,69		13,628	37,142	17,2544	278		13,628	33,1155	14,3166	262,78	heating
13,63	23,0616	7,8091	213,21		13,63	37,8403	17,5408	283,76		13,63	33,7764	18,9391	207,4	heating
13,632	21,5556	3,4783	252,69		13,632	36,6879	16,5013	282,18		13,632	33,8697	15,9537	250,44	heating
13,634	22,8447	0,6605	310,1		13,634	38,2653	14,8961	326,67		13,634	34,3397	12,3915	306,8	heating
13,636	22,2574	3,0948	267,86		13,636	37,9381	18,0096	278,57		13,636	34,3428	14,6012	275,96	heating
13,638	22,2569	2,6122	274,6		13,638	37,8376	14,9065	320,54		13,638	34,1246	12,9388	296,15	heating
13,64	21,9465	1,471	286,22		13,64	39,4776	20,2007	269,46		13,64	34,5302	12,079	313,83	heating
13,642	23,267	6,5725	233,36		13,642	40,2393	19,338	292,17		13,642	33,8764	11,4376	313,66	heating
13,644	22,3385	-1,007	326,34	heating	13,644	39,7172	22,1706	245,27		13,644	33,6415	9,804	333,21	heating
13,646	22,6638	-0,9137	329,58		13,646	39,2296	17,2936	306,63		13,646	34,1491	14,341	276,89	heating
13,648	22,4037	-0,1919	315,85		13,648	41,1841	21,1289	280,34		13,648	34,0586	18,3353	219,79	heating
13,65	22,0624	-2,7744	347,18		13,65	39,5351	15,5357	335,48		13,65	33,7416	13,9999	275,96	heating
13,652	22,7423	0,7039	308,06		13,652	39,8826	22,8019	238,76		13,652	34,6633	13,4465	296,58	heating
13,654	21,826	1,5584	283,31		13,654	38,6349	23,2508	215,05		13,654	34,0777	10,1489	334,49	heating
13,656	23,0502	-3,0086	364,26		13,656	39,5331	25,5394	195,61	heating	13,656	34,6928	11,2341	327,92	heating
13,658	22,4294	0,4974	306,58		13,658	37,5778	23,303	199,54	heating	13,658	34,3976	14,1304	283,31	heating
13,66	22,4456	2,0179	285,55		13,66	38,6946	21,1894	244,7	heating	13,66	34,4843	14,0969	284,99	heating
13,662	22,0744	-1,242	325,93		13,662	37,984	18,034	278,87	heating	13,662	34,2931	12,8111	300,29	heating
13,664	23,7341	5,407	256,18		13,664	38,144	15,7079	313,62	heating	13,664	34,5069	14,2164	283,63	heating
13,666	21,9082	1,8867	279,87		13,666	38,1813	17,4713	289,49	heating	13,666	34,4333	11,3955	322,04	heating
13,668	22,8611	2,7026	281,79		13,668	39,5334	16,364	323,87	heating	13,668	34,8972	10,9417	334,86	heating
13,67	22,5879	6,3609	226,83		13,67	38,9549	20,525	257,62	heating	13,67	34,4889	11,6498	319,26	
13,672	21,9636	1,1043	291,58		13,672	38,6362	16,0044	316,36	heating	13,672	34,5938	12,1327	313,97	
13,674	22,1003	-2,0763	337,95		13,674	40,6042	18,9647	302,49	heating	13,674	35,0138	16,3938	260,28	
13,676	22,6306	2,7715	277,6		13,676	40,3161	13,0981	380,47	heating	13,676	34,0838	15,6419	257,79	
13,678	22,7608	-3,5106	367,23		13,678	40,2438	18,5434	303,34	heating	13,678	34,3255	13,1667	295,77	heating
13,68	22,4985	-2,0168	342,69		13,68	39,4856	18,0912	299,06	heating	13,68	34,4519	12,6513	304,74	heating
13,682	22,2026	-1,0815	325,48		13,682	41,0139	24,2918	233,75	heating	13,682	34,5193	13,058	300	
13,684	21,8968	-4,6615	371,25		13,684	38,6831	19,8115	263,8	heating	13,684	34,1628	11,9796	310,09	
13,686	23,0306	2,6762	284,52		13,686	39,0102	18,5464	286,05	heating	13,686	34,6749	13,8655	290,89	
13,688	21,4391	2,8822	259,4		13,688	37,9144	14,4244	328,35	heating	13,688	34,2874	10,5858	331,31	
13,69	22,5741	-1,3643	334,62		13,69	38,122	12,9026	352,53	heating	13,69	34,6023	10,281	339,98	
13,692	22,3396	1,8228	286,79		13,692	37,082	13,2452	333,2		13,692	34,4324	11,5906	319,3	
13,694	21,8373	1,1934	288,57		13,694	38,7425	15,9474	318,64		13,694	34,3515	11,349	321,54	

13,696	21,6626	-0,114	304,4
13,698	22,9066	5,7037	240,47
13,7	21,8868	-1,0998	321,32
13,702	22,4341	3,5505	263,96
13,704	21,8905	7,1154	206,53
13,706	21,4863	-1,1367	316,24
13,708	22,2835	-2,7937	350,54
13,71	21,3925	-1,8181	324,45
13,712	22,3366	-1,5799	334,32
13,714	21,9447	4,6225	242,14
13,716	21,4995	2,8641	260,49
13,718	21,4565	-1,4348	319,99
13,72	22,7377	3,4994	268,92
13,722	21,3208	-2,426	331,94
13,724	21,9178	-1,1055	321,83
13,726	20,9819	1,0715	278,32
13,728	20,5533	-1,6945	310,99
13,73	20,7004	0,4411	283,19
13,732	20,6126	3,1963	243,45
13,734	20,9877	-5,073	364,29
13,736	21,1101	-4,0539	351,76
13,738	20,8341	-3,3794	338,47
13,74	20,8468	-4,1192	348,99
13,742	21,8406	1,6975	281,57
13,744	20,5042	-1,1352	302,49
13,746	21,5684	-0,052	302,22
13,748	21,274	5,4718	220,89
13,75	20,8257	2,286	259,16
13,752	20,8819	-0,8039	303,14
13,754	21,7253	3,9309	248,74
13,756	21,5649	0,7011	291,64
13,758	21,7812	4,7124	238,6
13,76	21,0762	2,9959	252,74
13,762	21,0323	-1,4841	314,75
13,764	21,7793	3,0792	261,4
13,766	20,4498	0,6376	276,94
13,768	21,3409	-4,247	357,68
13,77	21,0962	-1,6001	317,26
13,772	20,6276	-3,0829	331,44
13,774	20,6122	-2,9092	328,79
13,776	21,2262	0,6589	287,5
13,778	20,2287	-5,6338	361,52
13,78	20,9798	-0,6515	302,37
13,782	20,1198	2,221	250,2
13,784	20,1844	-2,1146	311,71
13,786	20,876	-1,1937	308,5
13,788	20,5614	0,7506	276,93
13,79	21,6546	2,1761	272,28
13,792	21,2655	7,7972	188,27
13,794	20,7462	1,1355	274,13
13,796	20,9687	-2,5457	328,7
13,798	21,7452	2,6506	266,91
13,8	20,6424	-1,2274	305,71
13,802	21,5142	1,0012	286,74
13,804	20,7057	1,8889	263,03
13,806	20,8919	-1,2797	309,93

13,696	37,7529	18,3739	270,89	
13,698	37,6959	14,7131	321,27	
13,7	38,5225	19,4945	265,98	
13,702	39,1236	17,6985	299,49	
13,704	38,4817	21,8768	232,11	
13,706	38,0537	16,3549	303,32	heating
13,708	40,1016	18,1787	306,45	
13,71	39,4219	11,8878	384,89	
13,712	39,201	16,4949	317,4	
13,714	38,7978	16,246	315,24	
13,716	40,2539	21,7329	258,9	
13,718	38,1771	19,0291	267,66	
13,72	39,5962	19,1861	285,3	
13,722	38,5946	14,6779	334,32	
13,724	38,9769	10,4009	399,45	
13,726	38,1367	13,1279	349,59	
13,728	39,7591	16,0792	331,01	
13,73	38,4596	20,3252	253,49	
13,732	38,5468	19,2053	270,37	
13,734	39,4271	21,3511	252,68	
13,736	40,0877	15,0018	350,66	
13,738	39,0788	16,5612	314,76	
13,74	38,5751	12,8874	359,08	
13,742	40,5442	19,0764	300,09	
13,744	38,9819	15,7388	324,9	
13,746	39,4113	19,907	272,64	
13,748	38,4996	17,4901	293,68	
13,75	39,9892	19,2958	289,26	
13,752	38,1045	17,4146	289,21	
13,754	39,5775	20,083	272,5	
13,756	38,5469	17,4748	294,56	
13,758	39,3244	17,904	299,43	
13,76	38,6801	19,0825	273,95	
13,762	40,5017	19,4948	293,65	
13,764	39,3376	22,3122	237,99	
13,766	38,7889	16,8615	306,51	
13,768	40,2986	19,6261	288,97	
13,77	40,7603	15,9518	346,79	
13,772	39,9793	18,0392	306,69	
13,774	39,4061	15,615	332,56	
13,776	41,4723	21,7266	276,02	
13,778	40,0861	15,328	346,08	
13,78	40,7717	21,7329	266,13	
13,782	39,6568	15,869	332,52	
13,784	41,4442	16,8588	343,67	
13,786	39,2345	14,6425	343,76	
13,788	40,339	16,695	330,51	
13,79	39,671	18,8104	291,6	
13,792	39,4939	21,0523	257,79	
13,794	38,706	20,114	259,89	
13,796	40,3011	18,481	305,01	
13,798	38,8727	18,9139	279	
13,8	38,3618	12,6256	359,75	
13,802	40,3133	20,1794	281,44	
13,804	40,0366	16,1868	333,38	
13,806	39,8302	20,7697	266,44	

13,696	34,3547	10,7006	330,65	
13,698	34,4579	11,9332	314,86	
13,7	34,9208	13,5118	299,27	
13,702	34,708	12,3959	311,89	
13,704	34,4401	10,2845	337,66	
13,706	34,4415	7,6544	374,44	
13,708	34,9017	10,2443	344,67	
13,71	34,6329	10,5639	336,45	
13,712	35,1068	12,3552	318,03	
13,714	35,1802	13,1078	308,54	
13,716	34,851	14,3098	287,14	
13,718	34,5769	14,1366	285,72	
13,72	34,7952	13,4484	298,4	
13,722	34,5318	11,2214	325,85	
13,724	34,5012	11,2387	325,17	
13,726	34,6081	13,7828	291,11	
13,728	34,4096	13,7646	288,59	
13,73	34,2043	12,3475	305,53	
13,732	34,3994	10,1394	339,12	
13,734	34,8386	10,351	342,3	
13,736	34,8886	11,3547	328,97	
13,738	34,6056	15,0261	273,69	cooling
13,74	34,6962	16,5676	253,41	
13,742	34,6415	16,1684	258,23	
13,744	34,3026	11,9656	312,24	
13,746	34,0926	8,6116	356,19	
13,748	34,1927	8,9219	353,25	
13,75	33,6162	11,3383	311,41	
13,752	33,4319	12,0997	298,19	
13,754	34,1122	11,1341	321,2	
13,756	34,06	10,8971	323,78	
13,758	33,9822	10,4163	329,42	cooling
13,76	34,0181	12,1906	305,12	cooling
13,762	34,1195	11,5029	316,15	cooling
13,764	33,811	9,6654	337,52	cooling
13,766	34,2383	7,8837	368,4	cooling
13,768	34,6238	8,8064	360,89	cooling
13,77	34,4988	10,3018	338,24	cooling
13,772	33,9841	12,6426	298,32	cooling
13,774	34,1944	12,5176	303,01	cooling
13,776	34,5157	13,4346	294,68	cooling
13,778	33,8983	10,1111	332,51	cooling
13,78	33,9977	7,4641	370,9	cooling
13,782	33,9998	8,6455	354,41	cooling
13,784	33,4711	10,2709	324,3	cooling
13,786	33,3838	11,686	303,3	cooling
13,788	33,801	11,2775	314,85	cooling
13,79	33,622	8,8464	346,33	cooling
13,792	34,0494	10,4839	329,41	cooling
13,794	33,8272	13,4252	285,19	cooling
13,796	33,8484	11,6086	310,88	cooling
13,798	33,5544	9,2879	339,21	cooling
13,8	33,7402	5,6728	392,34	cooling
13,802	33,9576	5,0452	404,15	cooling
13,804	33,9972	9,7795	338,53	cooling
13,806	33,5101	10,7954	317,52	cooling

13,808	21,2376	0,1673	294,53	
13,81	21,4099	0,3644	294,18	
13,812	22,0637	-4,7672	375,06	
13,814	22,0223	0,4417	301,66	
13,816	21,8578	1,9455	278,34	
13,818	21,7558	2,0358	275,66	
13,82	22,5517	4,2243	256,19	
13,822	21,3933	-1,8238	324,54	
13,824	22,5944	-0,0858	317,04	
13,826	21,6242	3,6492	251,26	
13,828	21,4107	2,1209	269,64	
13,83	21,1921	0,8492	284,36	
13,832	21,5079	2,3847	267,31	
13,834	21,2637	-0,4387	303,37	heating
13,836	21,1301	3,1346	251,55	
13,838	20,8006	1,9269	263,83	heating
13,84	20,7933	-2,3209	323,1	heating
13,842	21,514	-1,9433	327,9	heating
13,844	20,5717	-3,426	335,45	
13,846	21,7248	-1,0692	318,63	
13,848	21,0316	1,1116	278,45	
13,85	21,1213	-0,5724	303,25	
13,852	20,9859	-1,8219	318,82	
13,854	21,4578	-2,2576	331,51	
13,856	21,2995	-7,8586	407,59	
13,858	21,6852	-1,1644	319,4	
13,86	21,3533	2,5312	263,11	
13,862	21,1233	1,8381	269,58	
13,864	21,6194	2,8939	261,75	
13,866	20,7508	-0,5445	297,68	
13,868	22,3925	-0,6073	321,5	
13,87	21,4939	4,0615	243,68	
13,872	21,3968	2,3594	266,12	
13,874	21,1236	-0,1819	297,82	
13,876	22,4451	5,1913	241,18	
13,878	20,9302	1,0572	277,8	
13,88	21,6447	3,0843	259,45	
13,882	20,735	1,3654	270,76	
13,884	20,7614	-2,8771	330,43	heating
13,886	20,7798	-2,4565	324,81	
13,888	21,3808	2,1315	269,08	
13,89	21,796	-2,2026	335,47	
13,892	21,1509	0,2809	291,73	
13,894	20,9204	1,5887	270,23	
13,896	20,4903	0,9408	273,27	
13,898	21,2597	1,9519	269,89	
13,9	19,934	-3,9271	333,54	
13,902	21,4365	-4,4426	361,75	
13,904	20,4939	-0,0489	287,16	
13,906	20,3891	1,1235	269,31	
13,908	20,0459	-0,2712	284	
13,91	21,2482	3,3235	250,56	
13,912	20,8455	-2,5689	327,3	
13,914	20,7962	-1,3915	310,15	
13,916	20,6738	-1,6811	312,49	
13,918	20,519	-1,4143	306,6	

13,808	39,2294	18,0943	295,44
13,81	41,0115	21,0858	278,53
13,812	39,7038	15,2195	342,25
13,814	39,6964	18,9678	289,76
13,816	39,0082	13,7964	352,42
13,818	40,0205	17,3196	317,33
13,82	38,0782	16,3963	303,08
13,822	39,5597	18,0195	301,1
13,824	39,3898	20,2696	267,27
13,826	39,5327	17,3981	309,41
13,828	39,5309	17,8448	303,14
13,83	40,9366	18,7128	310,66
13,832	39,5574	18,9044	288,7
13,834	39,3394	15,3187	335,77
13,836	40,8175	22,8765	250,79
13,838	41,4527	18,2178	324,79
13,84	40,3853	24,8889	216,62
13,842	39,5428	19,5745	279,13
13,844	41,3312	19,6589	302,95
13,846	39,9095	15,129	346,39
13,848	39,7327	19,9492	276,54
13,85	39,0518	17,5676	300,32
13,852	40,5977	22,6361	251,08
13,854	38,2565	17,023	296,81
13,856	39,3436	17,7859	301,35
13,858	38,5364	16,1828	312,47
13,86	39,3047	16,041	325,19
13,862	38,5415	18,7938	276,04
13,864	40,2111	20,0801	281,4
13,866	39,2977	20,6529	260,63
13,868	39,5834	19,7213	277,64
13,87	40,3128	22,4521	249,67
13,872	40,5173	16,4934	335,82
13,874	39,6368	19,2503	284,97
13,876	38,7843	14,5146	339,25
13,878	40,2992	18,0214	311,41
13,88	39,0784	16,8589	310,6
13,882	39,3017	18,9529	284,45
13,884	38,8946	16,8006	308,84
13,886	39,6619	19,8524	276,91
13,888	38,1946	16,9946	296,35
13,89	39,7827	16,8431	320,66
13,892	38,9924	17,4278	301,44
13,894	39,0614	15,1247	334,6
13,896	39,7144	18,982	289,81
13,898	40,2353	16,3448	333,95
13,9	39,4999	17,0188	314,25
13,902	39,6434	15,051	343,77
13,904	41,0375	20,6847	284,5
13,906	40,0891	17,9515	309,45
13,908	39,9311	21,6817	255,1
13,91	38,9358	14,9985	334,61
13,912	40,2447	15,8816	340,56
13,914	38,7271	13,865	347,54
13,916	39,3751	14,2962	350,57
13,918	38,9281	14,5376	340,94

13,808	33,7647	10,9595	318,78	cooling
13,81	33,8934	13,0502	291,36	cooling
13,812	33,2071	9,6206	329,7	cooling
13,814	33,7221	10,3911	326,13	cooling
13,816	33,2178	13,5292	275,22	cooling
13,818	33,1463	14,2252	264,49	cooling
13,82	33,2305	14,6817	259,28	cooling
13,822	33,2755	10,5417	317,78	cooling
13,824	33,1309	6,7343	368,98	cooling
13,826	33,8117	10,1727	330,44	cooling
13,828	33,6048	10,9878	316,15	cooling
13,83	33,737	12,3699	298,68	cooling
13,832	33,4706	16,2213	241,12	cooling
13,834	33,0096	13,8866	267,31	cooling
13,836	33,6948	16,6388	238,42	cooling
13,838	32,7499	15,3857	242,73	cooling
13,84	32,673	9,849	319,05	
13,842	32,9026	10,5336	312,69	
13,844	32,5306	11,3574	295,97	
13,846	32,5191	10,7699	304,02	
13,848	33,4775	15,4553	251,92	
13,85	32,7535	13,1491	274,04	
13,852	33,2735	12,232	294,13	
13,854	33,2422	15,7689	244,25	
13,856	32,9617	13,6918	269,36	
13,858	33,0415	13,2581	276,54	
13,86	33,1572	14,2086	264,87	
13,862	33,3587	11,3687	307,39	
13,864	33,4441	11,7251	303,6	
13,866	32,8851	11,7749	295,09	
13,868	33,1094	9,8381	325,3	
13,87	33,852	13,2881	287,45	
13,872	32,914	10,7194	310,25	
13,874	33,4634	11,0674	313,06	
13,876	32,9334	14,3058	260,39	
13,878	33,0178	15,7483	241,4	
13,88	32,8316	15,2507	245,75	
13,882	33,3452	14,9573	257,04	
13,884	32,7474	8,8664	333,82	
13,886	33,0871	11,0373	308,22	
13,888	32,2955	11,9917	283,82	
13,89	32,6859	12,3552	284,19	
13,892	32,4436	13,6984	262,03	
13,894	32,1546	12,8481	269,88	
13,896	32,3201	11,605	289,57	
13,898	32,1245	13,4588	260,92	
13,9	32,0722	13,9156	253,8	heating
13,902	32,423	11,354	294,51	heating
13,904	33,2595	10,5747	317,1	heating
13,906	33,3555	10,6129	317,91	heating
13,908	33,6633	13,4675	282,31	
13,91	33,2938	12,1108	296,11	
13,912	33,5531	13,9417	274,14	
13,914	33,3008	13,8295	272,18	heating
13,916	33,2711	12,9317	284,31	heating
13,918	33,2845	11,4026	305,88	heating

13,92	20,8546	3,3093	245,26		13,92	39,4174	16,8024	316,12		13,92	33,1984	12,3428	291,53	heating
13,922	20,3356	6,6209	191,71		13,922	39,1003	17,3808	303,61		13,922	33,4469	13,2619	282,16	heating
13,924	21,3599	4,2346	239,39	heating	13,924	40,6598	18,9235	303,84		13,924	33,4851	18,0889	215,22	heating
13,926	20,478	3,1028	242,88	heating	13,926	39,3193	18,4813	291,28		13,926	33,0697	17,7027	214,81	heating
13,928	20,6823	-0,1345	290,99	heating	13,928	39,2524	11,6284	386,14		13,928	33,2949	14,0599	268,88	heating
13,93	20,5991	-3,1724	332,29	heating	13,93	40,1349	15,6041	342,9	cooling	13,93	33,5247	12,9338	287,83	heating
13,932	21,6167	0,9434	288,98	heating	13,932	39,6401	13,6799	362,89	cooling	13,932	33,4255	11,6102	304,95	heating
13,934	20,4954	-0,7538	297,03	heating	13,934	38,8153	18,7324	280,73	cooling	13,934	33,5568	14,0892	272,13	heating
13,936	21,6467	0,2942	298,48	heating	13,936	38,7982	20,3681	257,63	cooling	13,936	33,6197	13,5212	280,95	heating
13,938	21,071	2,5512	258,88	heating	13,938	39,8162	22,1894	246,4	cooling	13,938	33,6137	11,9964	302,18	heating
13,94	20,7599	1,3158	271,8	heating	13,94	39,7153	16,9681	317,97		13,94	34,1172	13,7945	284,08	heating
13,942	20,7756	-1,6207	313,07	heating	13,942	40,3711	18,8486	300,85		13,942	33,6191	16,4318	240,25	heating
13,944	21,6918	0,2849	299,24	heating	13,944	39,9378	13,6858	366,97		13,944	33,6374	16,6563	237,37	heating
13,946	22,0397	-3,1424	352,01	heating	13,946	40,631	15,342	353,5	cooling	13,946	32,9367	16,1755	234,3	heating
13,948	21,6776	0,8056	291,76	heating	13,948	39,4784	17,0527	313,48	cooling	13,948	32,6041	9,3521	325,03	heating
13,95	21,739	2,682	266,39	heating	13,95	39,9584	17,7144	310,94	cooling	13,95	32,9344	6,739	366,17	heating
13,952	21,3197	-0,9039	310,65	heating	13,952	38,9705	19,3369	274,45	cooling	13,952	32,6138	7,8551	346,09	heating
13,954	21,7252	-1,5458	325,29	heating	13,954	38,3389	15,0538	325,49	cooling	13,954	32,8097	10,5575	311,05	heating
13,956	21,0451	-1,5363	315,65		13,956	38,9388	13,2339	359,32	cooling	13,956	33,194	16,8232	228,84	heating
13,958	21,9624	-2,3148	339,36		13,958	40,0558	14,6706	354,85	cooling	13,958	33,2075	17,9553	213,2	heating
13,96	21,3429	1,5955	276,04		13,96	38,7748	16,7409	308	cooling	13,96	33,0138	12,3787	288,45	heating
13,962	20,926	3,538	243,06		13,962	38,9848	15,7793	324,38	cooling	13,962	34,0486	10,9451	322,95	heating
13,964	20,6157	1,1942	271,48		13,964	40,3488	22,5649	248,59	cooling	13,964	33,3833	8,1327	352,97	heating
13,966	21,6274	3,1003	258,98		13,966	39,529	17,6363	306,03	cooling	13,966	33,8644	8,2233	358,42	heating
13,968	20,6375	-3,1694	332,78	heating	13,968	39,2473	18,947	283,77	cooling	13,968	33,4179	12,1829	296,83	heating
13,97	20,6916	-2,6457	326,22		13,97	38,7224	16,9036	305	cooling	13,97	33,595	11,9598	302,43	heating
13,972	20,9261	2,8106	253,23		13,972	39,0565	14,4394	344,11	cooling	13,972	33,7777	12,6444	295,41	heating
13,974	20,5906	3,0318	245,45		13,974	37,9796	12,2847	359,18	cooling	13,974	33,9509	14,0748	277,84	heating
13,976	20,7828	1,5667	268,61	heating	13,976	38,6514	15,8119	319,26	cooling	13,976	33,9347	12,2537	303,07	heating
13,978	21,503	3,1288	256,84	heating	13,978	38,618	15,4714	323,55	cooling	13,978	34,3282	13,4909	291,27	heating
13,98	22,0141	-2,6797	345,18	heating	13,98	39,4248	21,1119	255,99	cooling	13,98	34,0688	12,1553	306,32	heating
13,982	21,963	-0,2833	310,97	heating	13,982	38,7914	22,7356	224,44	cooling	13,982	33,9074	8,2978	357,98	
13,984	21,5475	1,4957	280,29	heating	13,984	40,1003	19,3879	289,53		13,984	34,4518	11,5548	320,07	
13,986	21,398	-1,8104	324,42	heating	13,986	39,247	17,8144	299,6		13,986	33,4321	13,3778	280,33	
13,988	22,5641	2,4794	280,75		13,988	38,2707	11,1667	378,87		13,988	33,9385	14,6762	269,26	
13,99	20,9468	1,1344	276,95		13,99	39,0906	14,6926	341,05	cooling	13,99	33,4434	14,6444	262,78	
13,992	21,9072	-0,8283	317,81	heating	13,992	38,7969	17,5495	297,01	cooling	13,992	33,8431	11,2385	315,98	
13,994	21,681	3,8178	249,7		13,994	37,5486	20,262	241,64	cooling	13,994	33,4547	9,5084	334,73	
13,996	21,2199	2,2198	265,59	heating	13,996	37,632	19,2953	256,32		13,996	34,0108	12,5039	300,63	
13,998	21,0995	0,7477	284,49	heating	13,998	39,1479	21,0241	253,34		13,998	33,5435	12,9633	287,68	
14	22,777	7,8911	208,08	heating	14	38,3557	13,6806	344,92		14	34,1405	17,2367	236,29	
14,002	21,8622	0,4227	299,69	heating	14,002	39,1504	15,198	334,82		14,002	33,3634	18,8347	203,09	
14,004	22,4298	1,871	287,38	heating	14,004	38,7301	13,6643	350,38		14,004	33,758	16,0433	247,63	heating
14,006	21,8628	2,964	264,18	heating	14,006	39,4439	14,8037	344,43		14,006	33,9855	12,7603	296,7	heating
14,008	21,993	-1,7485	331,87	heating	14,008	38,8118	18,6194	282,26	cooling	14,008	33,6037	7,7451	361,47	heating
14,01	22,1704	-3,0823	353	heating	14,01	39,9658	21,0689	264,15		14,01	33,9972	9,2032	346,58	heating
14,012	22,2686	0,5854	303,1	heating	14,012	39,5428	22,0992	243,84		14,012	33,5703	10,9335	316,43	
14,014	23,1351	-3,5544	373,08	heating	14,014	39,14	21,0574	252,77		14,014	33,9525	14,2504	275,41	
14,016	22,6959	2,1209	287,61		14,016	39,5599	21,1798	256,93		14,016	33,9349	18,2815	218,81	
14,018	22,3199	3,1559	267,88		14,018	40,1954	17,4115	318,49		14,018	34,1783	19,2176	209,13	heating
14,02	21,8964	0,3803	300,76		14,02	39,2854	18,7071	287,65		14,02	34,0615	14,0192	280,16	heating
14,022	23,1511	4,9094	254,99		14,022	38,6108	15,0017	330,02		14,022	34,3352	13,1426	296,24	heating
14,024	21,5087	4,2059	241,87		14,024	40,1195	19,3163	290,8		14,024	33,9302	10,8944	322,01	heating
14,026	22,5123	5,1386	242,86	heating	14,026	38,8492	15,0601	332,54		14,026	34,207	11,5951	316,08	heating
14,028	21,7245	5,2046	230,92	heating	14,028	38,5644	15,2227	326,28		14,028	34,0388	12,0877	306,84	heating
14,03	21,647	0,4113	296,84	heating	14,03	38,4063	13,6435	346,15		14,03	34,0289	10,1938	333,18	heating

14,032	21,1786	-3,3509	342,89	heating	14,032	39,5351	16,5329	321,54		14,032	34,2581	11,3691	319,95	
14,034	21,7343	1,2165	286,81	heating	14,034	38,363	17,4037	292,98		14,034	33,9683	12,5537	299,34	
14,036	21,3442	0,941	285,21	heating	14,036	39,377	20,9451	257,65		14,036	34,0728	14,7133	270,62	
14,038	21,212	5,4038	220,98	heating	14,038	38,9444	18,7862	281,78		14,038	34,2625	15,7952	258,15	
14,04	21,1061	4,8228	227,62	heating	14,04	39,1167	16,9954	309,22		14,04	34,1974	13,9319	283,28	
14,042	21,0331	-1,7807	318,9	heating	14,042	38,8771	19,6757	268,41		14,042	34,5762	12,0183	315,33	heating
14,044	22,14	-4,7137	375,38	heating	14,044	39,9611	20,8261	267,48		14,044	34,2211	13,8564	284,67	
14,046	21,3998	-4,0553	355,82	heating	14,046	39,1335	23,6699	216,16		14,046	33,88	10,9571	320,43	
14,048	22,3704	0,5693	304,75	heating	14,048	38,1722	19,7756	257,16		14,048	34,2042	11,9988	310,4	
14,05	21,9638	5,3981	231,56		14,05	39,6072	21,0082	259,99		14,05	33,941	10,7537	324,12	
14,052	22,2295	3,5348	261,32	heating	14,052	39,9216	17,2203	317,33		14,052	34,1982	9,0885	351	
14,054	22,0161	-1,4936	328,63	heating	14,054	39,5298	19,5781	278,9		14,054	34,1685	11,2478	320,4	
14,056	22,3534	-1,4671	332,98	heating	14,056	38,9964	16,9523	308,14		14,056	34,2409	14,1774	280,46	
14,058	22,2922	-2,1415	341,55		14,058	41,0722	23,1546	250,46		14,058	33,9837	13,5629	285,45	
14,06	22,1539	0,7295	299,48		14,06	39,7132	19,9894	275,71		14,06	34,6838	15,8565	263,18	
14,062	21,9448	-0,2992	310,94		14,062	39,2915	23,3791	222,43		14,062	33,9806	13,7951	282,16	
14,064	21,7413	-2,5508	339,57		14,064	38,504	19,6138	264,06	heating	14,064	34,586	11,8275	318,13	
14,066	22,3428	-0,1813	314,85		14,066	39,6473	19,4645	282,13	heating	14,066	34,1969	11,8366	312,56	
14,068	21,5919	1,6727	278,44		14,068	38,0551	17,2753	290,47	heating	14,068	34,0476	10,0774	335,07	
14,07	22,516	3,2745	268,97		14,07	38,3505	18,3874	279,05	heating	14,07	34,042	10,9136	323,3	
14,072	22,4527	2,2995	281,71		14,072	38,3431	18,0863	283,16	heating	14,072	34,2457	12,1274	309,18	
14,074	22,9756	0,3194	316,7		14,074	38,3522	18,0639	283,6	heating	14,074	33,9762	10,4155	329,34	
14,076	22,7889	-0,06	319,39		14,076	38,427	19,814	260,18	heating	14,076	33,9726	10,1844	332,52	
14,078	23,3764	4,0982	269,48		14,078	39,8422	19,4889	284,51	heating	14,078	33,8645	12,3672	300,5	
14,08	23,0961	2,2999	290,7		14,08	39,7899	22,0712	247,68	heating	14,08	33,5818	9,9442	330,42	
14,082	23,27	2,4131	291,55		14,082	39,2825	16,867	313,34	heating	14,082	34,086	10,1782	334,2	
14,084	23,1941	0,3857	318,83		14,084	41,1143	19,2624	305,46	heating	14,084	33,7445	7,3858	368,46	
14,086	23,2891	-2,5854	361,69		14,086	40,85	14,0073	375,22	heating	14,086	34,0211	8,4258	357,78	
14,088	23,7364	-2,1638	362,05		14,088	40,3418	17,0551	325,51	heating	14,088	33,6404	12,6753	293,06	
14,09	23,1281	-0,5801	331,41		14,09	39,5885	17,1553	313,58		14,09	33,4624	14,6428	263,07	
14,092	23,9684	2,0236	306,76		14,092	40,8855	23,5695	242,05		14,092	33,6343	12,3174	297,98	
14,094	23,9058	3,4176	286,39		14,094	39,4485	22,9111	231,17	heating	14,094	34,0017	11,021	321,24	
14,096	24,4188	1,7812	316,44		14,096	39,2805	24,7828	202,66	heating	14,096	33,741	7,634	364,94	
14,098	24,1154	-1,6743	360,5		14,098	39,2625	20,5426	261,68	heating	14,098	34,1088	9,0491	350,3	
14,1	24,6151	-0,4003	349,68		14,1	39,8832	18,4969	298,95	heating	14,1	33,8309	10,7265	322,96	cooling
14,102	24,2335	0,4666	332,23		14,102	39,0233	18,9782	280,2	heating	14,102	33,4459	8,2882	351,67	cooling
14,104	24,0615	3,3544	289,45		14,104	40,3906	18,9093	300,28	heating	14,104	33,8743	10,2246	330,59	cooling
14,106	23,7484	1,8514	306,09		14,106	40,4206	23,0703	242,53	heating	14,106	33,4912	10,0101	328,23	cooling
14,108	24,1041	1,4853	316,18		14,108	39,6374	19,7416	278,11	heating	14,108	33,836	11,3305	314,59	cooling
14,11	24,294	4,1753	281,23		14,11	40,8264	24,4016	229,59	heating	14,11	33,4093	12,531	291,85	cooling
14,112	23,7714	6,3427	243,63		14,112	41,1787	20,1686	293,69	heating	14,112	33,1526	12,814	284,3	cooling
14,114	24,0317	5,4998	259,05		14,114	40,1883	20,0369	281,69	heating	14,114	33,1741	10,0961	322,6	cooling
14,116	23,7461	3,8268	278,44		14,116	39,5303	16,1602	326,68	heating	14,116	33,4056	11,0241	312,86	cooling
14,118	23,9443	1,5764	312,67		14,118	41,4821	21,575	278,27	heating	14,118	33,289	9,3153	335,12	cooling
14,12	23,8245	0,4812	326,3		14,12	40,5675	16,9513	330,12	heating	14,12	33,3841	12,6835	289,36	cooling
14,122	24,6277	3,2412	298,95		14,122	40,4945	21,7417	262,14	heating	14,122	33,2255	14,6157	260,14	cooling
14,124	24,0118	2,7235	297,58		14,124	39,9999	19,5543	285,8	heating	14,124	33,1645	15,0275	253,53	cooling
14,126	24,4918	1,3609	323,33		14,126	41,6147	23,0313	259,77	heating	14,126	33,3928	14,1205	269,4	
14,128	24,7078	-1,4063	365,04		14,128	39,9891	23,3191	233,02	heating	14,128	33,239	12,5293	289,49	
14,13	25,2984	-1,8897	380,05		14,13	40,6712	21,023	274,65	heating	14,13	33,4848	13,5212	279,06	
14,132	25,0478	-0,1364	352,04		14,132	40,5293	19,0948	299,62	heating	14,132	33,4072	15,8737	245,09	
14,134	24,8514	4,1674	289,13	cooling	14,134	40,1926	14,044	365,52	heating	14,134	33,1803	16,3489	235,28	
14,136	24,2548	1,3784	319,78	cooling	14,136	39,7362	14,3694	354,59	heating	14,136	33,3446	13,9394	271,26	
14,138	23,9147	-0,5492	341,97	cooling	14,138	41,1696	16,0365	351,32	heating	14,138	33,3223	13,0364	283,57	
14,14	23,5604	-0,8048	340,59	cooling	14,14	40,2587	21,3188	264,75	heating	14,14	33,5891	9,6892	334,09	
14,142	22,9484	0,7893	309,75	cooling	14,142	39,9727	19,4747	286,53	heating	14,142	33,7166	12,3534	298,63	

14,144	23,1463	6,5392	232,14	cooling	14,144	41,5555	25,9593	218,01	heating	14,144	33,3587	11,432	306,5	
14,146	22,3675	7,095	213,49		14,146	42,0961	17,7082	340,91	heating	14,146	33,2732	11,0599	310,51	
14,148	22,7009	1,8398	291,61		14,148	41,849	18,7325	323,13	heating	14,148	33,469	11,3505	309,18	
14,15	23,0812	-0,6408	331,6		14,15	41,2219	15,0145	366,34	heating	14,15	33,3259	11,593	303,79	
14,152	23,6219	1,7905	305,17		14,152	43,5988	20,8609	317,84		14,152	33,6032	11,4339	309,89	
14,154	23,8747	5,0436	263,23		14,154	42,7757	21,4951	297,47		14,154	33,5409	13,329	282,53	
14,156	24,3176	7,2858	238,08		14,156	41,7845	25,6749	225,19		14,156	33,2217	10,3463	319,76	
14,158	24,2554	0,5819	330,92		14,158	40,9394	19,3128	302,31		14,158	33,734	11,6968	308,05	
14,16	24,5902	-3,9736	399,28		14,16	42,4458	19,0943	326,42		14,16	33,4807	13,5707	278,31	
14,162	24,9153	-2,0971	377,59		14,162	39,8551	11,992	389,49		14,162	33,5127	11,6831	305,15	
14,164	24,7472	2,8288	306,39	cooling	14,164	41,0497	13,857	380,11		14,164	33,5974	13,6155	279,32	
14,166	25,2219	9,3988	221,18	cooling	14,166	40,3509	16,4024	334,76		14,166	33,578	13,3842	282,28	
14,168	24,5064	8,1718	228,33		14,168	41,1012	16,863	338,81		14,168	33,5139	12,1138	299,14	
14,17	24,8194	0,8546	334,99		14,17	40,1857	20,1493	280,08		14,17	33,836	13,723	281,15	
14,172	24,9193	-0,7262	358,49		14,172	41,5358	19,2524	311,49		14,172	33,0736	10,9851	308,76	
14,174	24,8721	1,5474	326,04		14,174	40,7671	19,2891	300,23		14,174	33,8308	8,2037	358,23	
14,176	24,7917	4,1791	288,13		14,176	40,3551	16,5485	332,78		14,176	33,7234	9,7278	335,42	
14,178	24,9937	7,6309	242,71		14,178	41,3357	19,0688	311,26		14,178	33,5767	8,437	351,42	
14,18	24,6546	3,7644	292,01		14,18	42,1254	15,2711	375,38		14,18	33,5709	10,052	328,76	
14,182	24,6072	0,8582	331,97		14,182	41,5026	19,2663	310,83		14,182	33,4281	10,8541	315,55	
14,184	24,6213	1,8025	318,97		14,184	40,5982	14,1313	369,97		14,184	33,3037	9,8058	328,47	
14,186	24,2327	2,7688	300,03		14,186	43,0702	21,3738	303,28	cooling	14,186	33,5974	11,2678	312,14	
14,188	24,7914	5,9951	262,74		14,188	41,1009	16,883	338,53	cooling	14,188	33,383	10,7321	316,63	
14,19	24,3855	7,3203	238,55		14,19	41,5137	17,4685	336,12	cooling	14,19	33,1471	8,4678	344,98	
14,192	24,6252	4,7858	277,33		14,192	40,5984	14,3607	366,76	cooling	14,192	33,4838	9,8125	330,89	
14,194	24,373	3,6966	289,03		14,194	41,79	12,3795	411,11	cooling	14,194	32,9474	7,6488	353,64	cooling
14,196	24,3295	3,6828	288,61		14,196	39,9793	9,3286	428,45	cooling	14,196	33,8052	9,1069	345,24	cooling
14,198	24,24	3,6311	288,08		14,198	41,0762	14,4025	372,86	cooling	14,198	33,4975	13,4975	279,57	cooling
14,2	24,8446	6,7028	253,6		14,2	40,4593	15,553	348,15	cooling	14,2	33,8113	16,449	242,7	cooling
14,202	24,9649	0,9471	335,73		14,202	41,0999	16,2545	347,3	cooling	14,202	33,933	19,3191	204,28	
14,204	25,963	-4,2383	422,17		14,204	40,7512	18,4113	312,28	cooling	14,204	33,9547	20,3042	190,81	
14,206	26,5502	-2,4138	404,87		14,206	41,7775	14,3731	383,07	cooling	14,206	33,4975	15,6229	249,86	
14,208	25,9226	0,5545	354,61		14,208	41,436	17,6331	332,73	cooling	14,208	33,7439	12,4259	297,99	
14,21	26,1563	5,9184	282,9		14,21	40,2483	12,4896	388,02	cooling	14,21	33,4987	10,4779	321,8	
14,212	25,5849	6,5172	266,54		14,212	42,2927	17,0766	352,48	cooling	14,212	33,168	8,6741	342,39	
14,214	25,8238	0,0689	360,01		14,214	41,2735	13,0228	394,9	cooling	14,214	33,5116	11,6183	306,04	
14,216	26,0149	-2,0795	392,72		14,216	40,7746	14,4523	367,95	cooling	14,216	33,1651	12,5126	288,69	
14,218	25,7295	0,2138	356,67	cooling	14,218	39,763	12,596	379,75	cooling	14,218	33,5935	14,1863	271,28	
14,22	25,1176	3,1748	306,73	cooling	14,22	41,0402	18,2122	319,1	cooling	14,22	33,3254	15,9934	242,28	
14,222	25,2818	10,2222	210,51	cooling	14,222	38,7516	14,6962	336,26	cooling	14,222	33,2957	14,6705	260,35	
14,224	24,5221	7,3764	239,67	cooling	14,224	40,2983	19,6858	288,13	cooling	14,224	32,9968	12,0593	292,67	
14,226	25,0797	3,219	305,58		14,226	39,1458	19,9322	268,58	cooling	14,226	33,298	13,0872	282,52	
14,228	25,2952	2,3569	320,64		14,228	39,9861	17,1832	318,75	cooling	14,228	32,994	11,0544	306,68	
14,23	25,0837	0,3855	345,24		14,23	39,1897	17,1946	307,46	cooling	14,23	33,6088	12,1491	299,97	
14,232	25,6464	4,1361	300,68		14,232	40,2843	13,6146	372,8	cooling	14,232	33,2372	13,0388	282,34	
14,234	25,3671	7,0672	255,81		14,234	40,153	13,5527	371,83	cooling	14,234	33,3542	10,9931	312,58	
14,236	25,5054	3,5392	307,05		14,236	40,2213	14,5431	358,94	cooling	14,236	33,696	13,3365	284,6	
14,238	26,0334	3,7728	311,17		14,238	41,0577	20,708	284,46	cooling	14,238	33,2946	13,391	278,22	
14,24	25,7078	2,1343	329,52		14,24	41,3007	17,1671	337,35	cooling	14,24	33,2639	11,372	306,02	
14,242	25,7987	0,6656	351,32		14,242	41,0531	20,7562	283,72	cooling	14,242	33,4956	12,7073	290,59	
14,244	26,2432	7,5812	260,87		14,244	39,9477	11,8977	392,1	cooling	14,244	33,0216	12,435	287,77	
14,246	25,5363	5,6731	277,66		14,246	42,1134	16,5033	357,99	cooling	14,246	33,1315	12,6675	286,06	
14,248	25,9775	0,2025	360,3		14,248	40,2418	14,1033	365,38	cooling	14,248	33,2177	16,6609	231,44	
14,25	26,1964	1,0758	351,15		14,25	40,4603	17,7445	317,53	cooling	14,25	32,699	11,6274	294,55	
14,252	25,6602	0,3483	353,82	cooling	14,252	39,6487	17,8977	304,05	cooling	14,252	33,7123	11,6812	307,96	
14,254	25,9046	4,4975	299,24	cooling	14,254	40,2086	20,0387	281,95	cooling	14,254	33,4943	11,2279	311,25	

14,256	25,52	7,5137	251,7	cooling	14,256	38,2171	15,4122	318,78	cooling	14,256	33,5268	6,6719	375,39	
14,258	25,4597	1,8955	329,39		14,258	39,6962	16,2478	327,77	cooling	14,258	33,854	9,3801	342,11	
14,26	25,9287	0,4017	356,83	cooling	14,26	38,2034	15,1764	321,88	cooling	14,26	33,6079	13,3459	283,23	
14,262	25,4142	-0,0878	356,48	cooling	14,262	38,7792	14,1945	343,66	cooling	14,262	33,5956	13,0453	287,26	
14,264	25,5618	-2,0508	385,98	cooling	14,264	39,0074	19,9176	266,85	cooling	14,264	34,1474	16,2452	250,25	
14,266	26,2135	3,1243	322,75	cooling	14,266	40,2006	17,4108	318,57	cooling	14,266	32,8843	11,5597	298,09	
14,268	25,6573	2,7452	320,28	cooling	14,268	39,6413	17,6358	307,6	cooling	14,268	33,8947	9,3073	343,69	
14,27	26,2851	3,7884	314,47	cooling	14,27	39,577	15,0097	343,41	cooling	14,27	33,7536	12,4252	298,14	
14,272	26,4394	5,2864	295,69	cooling	14,272	40,875	18,6565	310,58	cooling	14,272	33,1356	8,9856	337,58	
14,274	25,9099	0,4621	355,72	cooling	14,274	41,0821	16,2537	347,06	cooling	14,274	33,6643	10,3384	326,06	
14,276	26,3261	0,9701	354,44	cooling	14,276	40,742	23,6748	238,57	cooling	14,276	33,6681	13,7124	278,95	
14,278	26,1168	4,314	304,77	cooling	14,278	39,8329	20,5103	270,1	cooling	14,278	33,7196	11,8649	305,5	
14,28	26,3984	-0,5977	377,37	cooling	14,28	41,4702	23,7776	247,32	cooling	14,28	33,9222	17,5911	228,28	
14,282	27,0711	-0,9789	392,1	cooling	14,282	39,2166	18,5399	289,03	cooling	14,282	33,2039	17,3277	221,93	
14,284	26,83	-0,0465	375,69	cooling	14,284	40,0678	18,6994	298,7	cooling	14,284	32,9761	10,0865	319,96	
14,286	26,284	1,5787	345,34	cooling	14,286	39,0568	17,7017	298,51	cooling	14,286	33,6234	12,656	293,09	
14,288	26,1392	8,423	247,65	cooling	14,288	39,6713	19,8476	277,11	cooling	14,288	32,3768	8,3093	336,43	
14,29	24,8049	5,6308	268,03	cooling	14,29	38,1039	17,394	289,49	cooling	14,29	33,3793	9,4875	333,97	
14,292	25,245	2,8732	312,72	cooling	14,292	40,1283	17,6807	313,78	cooling	14,292	33,1946	14,4242	262,38	
14,294	25,0899	2,5738	314,74	cooling	14,294	38,7011	19,0632	274,51	cooling	14,294	33,0256	11,7387	297,56	
14,296	24,7362	-0,5608	353,61	cooling	14,296	39,0132	18,9242	280,81	cooling	14,296	34,2845	13,5916	289,26	
14,298	25,1267	2,282	319,33	cooling	14,298	39,7119	24,3135	215,25	cooling	14,298	34,0144	14,2083	276,86	
14,3	24,9312	6,2064	261,75	cooling	14,3	40,774	21,2773	272,54		14,3	34,3989	8,7531	358,49	
14,302	24,739	1,4185	325,99	cooling	14,302	40,1478	21,4818	260,92		14,302	34,8009	15,7264	266,63	
14,304	24,8234	2,6323	310,2	cooling	14,304	39,6986	17,6795	307,79		14,304	34,1009	15,3755	261,75	
14,306	24,6816	4,4586	282,69	cooling	14,306	41,3369	21,4427	278,09		14,306	33,9245	10,8388	322,7	
14,308	24,3572	3,7494	288,07	cooling	14,308	40,2127	17,4679	317,94		14,308	34,7838	15,9471	263,31	
14,31	24,4968	5,9816	258,82	cooling	14,31	40,2162	23,912	227,91		14,31	33,3731	11,0382	312,21	
14,312	23,6517	3,091	287,41	cooling	14,312	38,9557	20,391	259,51		14,312	34,4751	13,0832	299,03	
14,314	24,3535	-0,072	341,43	cooling	14,314	40,4662	23,3085	239,84		14,314	33,6928	18,2029	216,53	
14,316	24,0615	4,7382	270,11	cooling	14,316	38,1066	20,6978	243,35		14,316	33,3033	12,984	284,03	
14,318	23,5172	5,337	254,13	cooling	14,318	39,7598	21,5043	255,18		14,318	33,9334	11,7186	310,53	
14,32	23,5075	3,3081	282,36	cooling	14,32	39,1191	19,2197	278,16		14,32	33,6639	14,5195	267,61	
14,322	24,1135	4,8842	268,8	cooling	14,322	40,0448	19,6796	284,67		14,322	33,8875	12,421	300,07	
14,324	23,5534	1,9336	302,21		14,324	39,8075	21,1999	260,11		14,324	34,0145	17,9473	224,6	
14,326	24,1574	1,0253	323,35		14,326	41,3862	22,4369	264,88	heating	14,326	33,2677	16,3711	236,19	heating
14,328	24,1934	2,5282	302,85		14,328	39,742	23,3182	229,58	heating	14,328	33,3148	11,0895	310,68	heating
14,33	24,1668	0,8246	326,29		14,33	39,3622	17,9023	299,98	heating	14,33	34,3898	13,4703	292,42	heating
14,332	24,2048	0,0128	338,17		14,332	40,8758	21,885	265,46	heating	14,332	33,6027	9,4806	337,19	heating
14,334	24,4341	0,5714	333,57	cooling	14,334	39,9991	17,0493	320,8	heating	14,334	34,5966	9,5815	349,67	heating
14,336	24,3819	-1,8326	366,44	cooling	14,336	39,8645	22,1429	247,72	heating	14,336	34,0914	10,7541	326,22	
14,338	24,61	1,6279	321,25	cooling	14,338	39,0685	19,3869	275,12	heating	14,338	34,4511	10,5178	334,55	
14,34	24,1957	5,5214	261,04	cooling	14,34	41,0587	22,0905	265,15	heating	14,34	34,4643	13,9486	286,78	
14,342	23,6807	2,4473	296,81	cooling	14,342	39,4111	17,8608	301,24	heating	14,342	33,9882	14,9506	266,12	
14,344	23,834	-1,4216	353,04	cooling	14,344	40,0995	22,3305	248,38	heating	14,344	34,2103	11,3356	319,75	
14,346	23,1692	-4,7162	389,8	cooling	14,346	39,4453	20,2002	269,02	heating	14,346	34,629	15,8692	262,24	
14,348	23,4504	-4,3628	388,79	cooling	14,348	40,3909	23,0911	241,83	heating	14,348	33,8529	11,4098	313,72	
14,35	23,537	3,1444	285,06	cooling	14,35	38,584	18,6812	278,21	heating	14,35	34,0434	11,1747	319,67	
14,352	23,3595	5,9292	243,65	cooling	14,352	40,102	17,1765	320,46	heating	14,352	34,169	13,2972	291,76	
14,354	23,3573	3,4312	278,54	cooling	14,354	39,3062	16,4011	320,18	heating	14,354	33,5907	10,7127	319,8	
14,356	23,9946	2,5453	299,83	cooling	14,356	38,954	15,0626	333,97	heating	14,356	34,3013	14,6748	274,35	
14,358	23,8256	-0,9225	345,94	cooling	14,358	39,7985	21,6026	254,35	heating	14,358	33,5876	15,8254	248,29	
14,36	23,8811	1,1602	317,6	cooling	14,36	40,5549	22,781	248,45	heating	14,36	33,847	12,5468	297,74	
14,362	23,3611	4,4176	264,8	cooling	14,362	39,7003	24,174	217,04	heating	14,362	34,1093	14,4761	274,44	
14,364	23,0116	2,3979	288,15	cooling	14,364	39,5911	19,1667	285,5	heating	14,364	33,6992	13,2731	285,53	
14,366	23,3645	3,3019	280,45	cooling	14,366	40,588	18,0662	314,82	heating	14,366	33,9488	11,9498	307,51	

14,368	23,0078	4,2855	261,71	cooling	14,368	39,531	12,0427	384,24	heating	14,368	34,1421	14,8974	269,01	
14,37	23,1189	0,9162	310,36		14,37	39,9675	18,4341	301,01	heating	14,37	33,8247	9,0669	346,08	
14,372	23,2645	2,4531	290,91		14,372	39,4963	19,6089	278	heating	14,372	34,2021	9,816	340,88	
14,374	22,9267	2,8643	280,44		14,374	40,4834	23,3798	239,08	heating	14,374	34,0692	11,2666	318,75	
14,376	22,6762	0,107	315,48		14,376	38,7427	21,4127	242,25	heating	14,376	33,6203	7,9323	359,08	
14,378	23,1311	-0,1916	326,02		14,378	39,4331	18,7573	289,02	heating	14,378	34,2301	8,9434	353,47	
14,38	22,4023	-5,5807	391,16		14,38	38,8843	13,991	347,97	heating	14,38	33,353	8,2723	350,59	
14,382	22,9183	-4,9481	389,53	cooling	14,382	38,9865	12,2659	373,51	heating	14,382	34,0704	8,2244	361,29	
14,384	22,8999	2,0628	291,27	cooling	14,384	38,7238	13,2183	356,53	heating	14,384	33,892	14,5978	269,7	
14,386	22,6884	5,3113	242,91	cooling	14,386	40,0038	16,1199	333,86		14,386	33,5882	15,0392	259,29	
14,388	23,0305	4,5049	258,96	cooling	14,388	38,8357	20,0521	262,57		14,388	33,8311	15,3141	258,84	
14,39	22,8331	-1,769	343,9		14,39	38,3569	18,1234	282,83		14,39	34,2662	16,5207	248,06	
14,392	23,3115	-7,3103	428,05	cooling	14,392	39,343	20,3769	265,12		14,392	33,8257	11,3574	314,07	
14,394	23,4419	-1,0243	342	cooling	14,394	39,4103	17,9341	300,21		14,394	33,8473	11,3449	314,55	
14,396	22,8566	2,7324	281,31	cooling	14,396	38,7471	19,6104	267,5		14,396	33,4213	11,5379	305,9	
14,398	22,8536	1,5367	297,98	cooling	14,398	38,5514	17,4065	295,57	heating	14,398	33,6112	12,0876	300,87	
14,4	23,5889	4,6198	265,16	cooling	14,4	39,5048	18,504	293,56	heating	14,4	33,5757	15,3833	254,3	
14,402	22,9941	0,7078	311,53	cooling	14,402	38,2081	11,8923	367,86		14,402	32,9567	15,0687	250,05	
14,404	23,1416	0,8545	311,54	cooling	14,404	39,0214	13,6137	355,16		14,404	33,7361	14,6	267,49	
14,406	22,7594	-0,2245	321,28	cooling	14,406	38,9158	15,2716	330,51		14,406	33,2441	16,241	237,68	
14,408	22,6413	-5,6911	396,05	cooling	14,408	39,3163	16,938	312,82		14,408	33,1966	13,891	269,86	
14,41	23,4053	-2,8386	366,85	cooling	14,41	38,6458	17,3637	297,49		14,41	33,2653	13,397	277,73	
14,412	23,1222	2,7931	284,17	cooling	14,412	39,4675	14,1421	354,01		14,412	33,8298	13,9459	277,95	
14,414	23,774	4,4249	270,47	cooling	14,414	39,1663	10,2407	404,34		14,414	33,3166	11,7314	301,73	
14,416	24,2787	8,0621	226,68	cooling	14,416	39,2363	9,2825	418,71		14,416	33,7337	13,6049	281,37	
14,418	24,5624	5,2857	269,46	cooling	14,418	39,9855	14,2845	359,26	cooling	14,418	33,3594	12,6743	289,15	
14,42	25,2633	1,1734	336,74		14,42	41,242	16,9958	338,93	cooling	14,42	33,7845	11,3302	313,88	
14,422	26,1491	3,1259	321,83	cooling	14,422	40,4081	22,1543	255,16	cooling	14,422	33,92	13,3443	287,62	
14,424	25,193	-0,8006	363,35	cooling	14,424	39,9388	17,7007	310,86	cooling	14,424	33,6548	14,0453	274,11	
14,426	25,7142	2,0356	330,99	cooling	14,426	41,4426	17,6661	332,36	cooling	14,426	34,3073	16,5351	248,43	
14,428	25,2797	5,6466	274,44	cooling	14,428	40,6059	11,754	403,31	cooling	14,428	33,448	15,1655	255,56	heating
14,43	25,249	4,1771	294,55	cooling	14,43	40,0905	11,7077	396,75	cooling	14,43	33,8751	14,3199	273,35	heating
14,432	25,6429	4,5884	294,31	cooling	14,432	39,7502	12,9878	374,1	cooling	14,432	33,689	16,8839	234,91	heating
14,434	25,6134	3,7984	304,94	cooling	14,434	40,1802	17,0065	323,93	cooling	14,434	33,6065	17,493	225,24	heating
14,436	26,1651	-0,3175	370,19	cooling	14,436	38,8741	16,8505	307,86	cooling	14,436	33,3677	14,2572	267,14	heating
14,438	26,4866	2,6789	332,8	cooling	14,438	39,7689	20,0216	276,04	cooling	14,438	33,7579	11,6752	308,68	heating
14,44	26,1828	1,1724	349,61	cooling	14,44	39,1384	18,9685	281,95	cooling	14,44	33,7035	7,8408	361,52	heating
14,442	26,6287	-1,5397	393,75	cooling	14,442	39,4728	16,3094	323,79	cooling	14,442	33,8266	11,1716	316,68	heating
14,444	27,2982	0,2527	378,06	cooling	14,444	39,2116	15,5167	331,22	cooling	14,444	33,3539	13,9715	270,94	heating
14,446	26,7969	-0,6342	383,45	cooling	14,446	40,0634	12,7418	381,92	cooling	14,446	33,3072	12,4778	291,16	heating
14,448	27,6317	4,5815	322,21	cooling	14,448	39,5581	14,0843	356,09	cooling	14,448	33,8425	14,0133	277,18	heating
14,45	27,2873	5,9649	298,06	cooling	14,45	38,7455	13,4408	353,72	cooling	14,45	33,3662	11,9753	299,01	heating
14,452	27,1563	-0,2844	383,58	cooling	14,452	39,5301	17,6659	305,63	cooling	14,452	33,6892	10,7357	320,86	heating
14,454	27,7024	-1,6416	410,18	cooling	14,454	39,4613	16,4039	322,31	cooling	14,454	33,55	10,1566	327,01	heating
14,456	26,9952	-3,4982	426,25	cooling	14,456	38,757	18,1154	288,54	cooling	14,456	33,4329	8,7009	345,72	heating
14,458	27,8286	-1,7707	413,75	cooling	14,458	38,3842	14,5624	332,99	cooling	14,458	33,4886	7,7172	360,25	
14,46	27,711	6,5066	296,41	cooling	14,46	40,0078	15,9656	336,08	cooling	14,46	33,7376	12,3399	299,11	
14,462	27,1923	6,9811	282,52	cooling	14,462	39,0031	10,3655	400,31	cooling	14,462	33,4033	11,2932	309,07	
14,464	27,431	7,5394	278,06	cooling	14,464	39,6472	12,1955	383,73	cooling	14,464	33,906	10,4451	327,95	
14,466	27,793	8,2336	273,41	cooling	14,466	39,3521	14,1573	352,19	cooling	14,466	33,7428	9,5075	338,77	
14,468	27,0965	-1,0445	393,37	cooling	14,468	39,7968	16,6933	322,95	cooling	14,468	33,7842	9,3944	340,93	
14,47	28,0847	1,4521	372,28	cooling	14,47	38,5581	18,0115	287,21	cooling	14,47	33,7394	10,6834	322,29	
14,472	27,7617	4,723	322,05	cooling	14,472	39,0646	18,4611	288,01	cooling	14,472	33,6753	10,9461	317,72	
14,474	28,1016	0,7962	381,69	cooling	14,474	38,1456	15,2752	319,69	cooling	14,474	33,5575	9,064	342,38	
14,476	28,4011	3,0162	354,84	cooling	14,476	37,6954	9,4433	394,92	cooling	14,476	33,9398	12,2081	303,78	
14,478	27,8472	4,7678	322,62	cooling	14,478	37,9503	9,5668	396,76	cooling	14,478	33,6317	14,365	269,32	

14,48	28,2613	1,3631	376	cooling	14,48	38,5315	10,4478	392,57	cooling	14,48	33,7496	13,329	285,45	
14,482	27,9087	7,9433	279,09	cooling	14,482	38,1965	19,2991	264,16	cooling	14,482	33,6541	12,6541	293,55	
14,484	26,9908	3,6118	326,8	cooling	14,484	37,2142	19,396	249,07	cooling	14,484	33,4318	10,0354	327,05	
14,486	27,0187	-3,4939	426,52	cooling	14,486	38,7922	20,835	251,01	cooling	14,486	33,5645	11,7768	304,56	
14,488	27,5562	-1,2032	402,01	cooling	14,488	37,8523	13,58	339,29	cooling	14,488	33,436	13,5252	278,32	
14,49	26,2242	-0,4651	373,08	cooling	14,49	37,688	14,4595	324,7	cooling	14,49	33,3214	13,5214	276,77	
14,492	27,2713	6,5009	290,34	cooling	14,492	37,2354	13,5628	330,91	cooling	14,492	33,6702	14,9368	261,87	
14,494	26,3711	11,8219	203,38	cooling	14,494	38,648	17,106	301,13	cooling	14,494	33,1861	14,1422	266,2	
14,496	26,04	3,3439	317,26	cooling	14,496	37,3637	14,032	326,14	cooling	14,496	33,1158	9,2601	333,47	
14,498	26,0886	-1,738	388,97	cooling	14,498	38,0161	15,1762	319,27	cooling	14,498	33,4988	9,8803	330,15	
14,5	27,0397	2,8081	338,72	cooling	14,5	37,4021	15,0444	312,53	cooling	14,5	33,3044	9,6228	331,03	
14,502	26,1437	1,2388	348,13	cooling	14,502	37,3667	15,7646	301,97	cooling	14,502	33,5363	11,5517	307,31	
14,504	26,4988	5,9651	287,03	cooling	14,504	36,7026	18,6526	252,31	cooling	14,504	33,8296	16,1209	247,54	
14,506	25,3831	3,9546	299,54	cooling	14,506	37,0878	17,5987	272,43	cooling	14,506	33,513	14,1976	270	
14,508	25,4304	-0,5888	363,71	cooling	14,508	36,807	17,0236	276,54	cooling	14,508	33,5355	11,8955	302,5	
14,51	25,3545	3,5614	304,63	cooling	14,51	36,2887	12,9155	326,72	cooling	14,51	33,6486	11,3883	311,17	
14,512	24,5608	6,0299	259,03	cooling	14,512	37,4069	14,5613	319,35	cooling	14,512	33,4098	8,841	343,43	
14,514	25,4871	2,8271	316,75	cooling	14,514	37,8007	13,179	344,17	cooling	14,514	34,1343	11,5005	316,39	
14,516	25,2664	3,0848	310,07	cooling	14,516	37,5883	19,0537	259,09	cooling	14,516	33,6004	15,2572	256,41	
14,518	25,2496	-0,8798	365,25	cooling	14,518	36,6591	16,9143	276	cooling	14,518	33,6718	12,4369	296,83	
14,52	25,1257	-1,6348	374,07	cooling	14,52	37,9881	19,0587	264,6	cooling	14,52	33,8712	13,1384	289,81	
14,522	25,9612	4,2582	303,38	cooling	14,522	36,2281	12,2919	334,59	cooling	14,522	33,6399	12,2461	299,05	
14,524	24,4836	2,182	311,74	cooling	14,524	36,096	11,624	342,08	cooling	14,524	33,4693	10,055	327,3	
14,526	25,3684	6,3153	266,33	cooling	14,526	35,3877	11,1539	338,75	cooling	14,526	34,0651	12,0152	308,22	
14,528	24,1741	6,4053	248,38	cooling	14,528	36,0446	14,7399	297,81	cooling	14,528	33,4849	10,5227	320,98	
14,53	24,1256	2,0694	308,31	cooling	14,53	35,251	16,3836	263,74	cooling	14,53	33,9006	11,2522	316,59	
14,532	23,6819	-0,7056	340,9	cooling	14,532	36,6163	17,9732	260,6	cooling	14,532	34,1454	15,466	261,11	
14,534	24,5366	1,7001	319,22	cooling	14,534	36,8989	17,5009	271,16	cooling	14,534	33,5164	13,3173	282,35	
14,536	24,7003	-1,5718	367,24	cooling	14,536	37,5816	15,9563	302,29		14,536	33,817	11,6231	310,24	
14,538	24,87	3,6985	295,95	cooling	14,538	37,7082	17,1921	286,79		14,538	33,4012	10,3191	322,65	
14,54	24,5795	3,9145	288,86	cooling	14,54	37,7965	14,1699	330,26		14,54	33,9257	4,9358	405,24	
14,542	24,4964	3,2217	297,39	cooling	14,542	36,9536	18,0299	264,53		14,542	34,1786	9,3462	347,12	
14,544	25,1862	6,9143	255,41	cooling	14,544	35,3326	14,9798	284,5		14,544	34,0796	14,1701	278,31	
14,546	23,67	2,9862	289,13	cooling	14,546	36,4939	18,2373	255,2		14,546	33,5021	12,0203	300,28	
14,548	24,7293	-0,3728	350,89	cooling	14,548	36,1738	15,6005	287,58		14,548	34,2276	12,5688	302,76	
14,55	23,9558	0,759	324,26	cooling	14,55	36,197	16,5056	275,26		14,55	33,6484	10,5206	323,29	
14,552	24,3474	1,2691	322,6	cooling	14,552	36,3186	12,8586	327,94		14,552	33,8705	7,3375	370,89	
14,554	23,9871	1,1262	319,56	cooling	14,554	37,9853	16,1151	305,71		14,554	34,4283	13,3002	295,34	
14,556	24,6947	5,5055	268,24	cooling	14,556	36,3777	11,9668	341,23		14,556	33,7148	12,4343	297,47	
14,558	23,6363	0,8092	319,09	cooling	14,558	37,4222	14,7628	316,74		14,558	34,1076	9,9506	337,68	
14,56	23,8541	4,0753	276,48	cooling	14,56	36,6413	17,0525	273,82		14,56	34,186	12,4631	303,65	
14,562	23,507	7,4766	224,08	cooling	14,562	36,4571	14,5254	306,57		14,562	34,1091	11,9355	309,95	
14,564	23,2754	4,06	268,6	cooling	14,564	35,7325	16	275,83		14,564	34,2265	11,2123	321,71	
14,566	23,4776	-2,0566	356,93	cooling	14,566	36,6289	17,6824	264,84		14,566	34,5101	16,5903	250,49	cooling
14,568	23,7392	0,2486	328,36	cooling	14,568	35,9806	16,4513	272,99		14,568	33,9522	14,1724	276,49	
14,57	24,153	2,0959	308,33	cooling	14,57	36,4473	13,4331	321,7		14,57	34,6671	13,3553	297,91	
14,572	23,5677	5,835	247,88	cooling	14,572	37,7632	17,1689	287,88		14,572	34,3705	12,2148	309,7	
14,574	23,7314	5,5652	253,94	cooling	14,574	38,2413	12,8196	355,36		14,574	34,2261	8,7825	355,66	
14,576	23,0634	-0,8169	333,81		14,576	38,3169	19,6829	260,48		14,576	34,6755	9,1322	357,06	
14,578	24,1323	-1,7762	362,16	cooling	14,578	36,7262	17,4178	269,9		14,578	34,2025	10,5437	330,72	cooling
14,58	23,1286	-0,5596	331,13	cooling	14,58	38,158	19,3272	263,23		14,58	33,9692	9,1771	346,56	cooling
14,582	23,7444	-0,1372	333,83	cooling	14,582	37,1706	14,6875	314,28		14,582	33,886	8,8934	349,36	cooling
14,584	23,5372	-0,116	330,64	cooling	14,584	36,8601	15,0869	304,36		14,584	34,5285	12,8376	303,21	cooling
14,586	24,2194	3,7922	285,54	cooling	14,586	36,9234	11,7425	351,99		14,586	33,8435	14,7619	266,73	cooling
14,588	23,8681	5,6336	254,89	cooling	14,588	38,8749	17,8032	294,55		14,588	34,1679	17,5559	232,21	cooling
14,59	24,5334	10,2567	199,57	cooling	14,59	37,3674	15,0627	311,79		14,59	33,4494	14,1773	269,4	

14,592	23,758	2,6863	294,55		14,592	38,535	17,4866	294,23		14,592	33,9187	12,0709	305,4	
14,594	23,6574	-3,5388	380,16		14,594	37,8924	19,1396	262,14		14,594	33,4611	9,9167	329,12	
14,596	23,7731	-2,3937	365,77		14,596	37,3619	14,372	321,36		14,596	34,0197	13,5204	286,55	
14,598	23,0628	0,3114	318,03	cooling	14,598	36,9176	16,8354	280,72		14,598	33,4361	14,4968	264,74	
14,6	23,3618	2,8867	286,21	cooling	14,6	37,3075	19,2571	252,32		14,6	33,4421	14,3602	266,74	
14,602	22,8578	5,3322	244,98	cooling	14,602	36,3546	18,8678	244,44		14,602	33,3378	12,5841	290,11	
14,604	23,1176	0,4905	316,29		14,604	36,3767	15,5145	291,62		14,604	33,4274	11,4677	306,96	
14,606	22,877	-0,3489	324,66		14,606	37,5239	15,1431	312,85		14,606	33,6295	10,4676	323,77	
14,608	22,8573	1,5201	298,26		14,608	38,7141	6,8186	445,85		14,608	33,5152	11,4624	308,27	
14,61	21,9397	-3,2979	352,78		14,61	39,0173	15,9441	322,53		14,61	33,5815	12,1712	299,28	
14,612	23,2182	-0,7565	335,13	cooling	14,612	37,9623	15,2851	316,99		14,612	33,5103	13,9215	273,82	
14,614	22,4896	-1,2202	331,43	cooling	14,614	39,7704	19,6875	280,73		14,614	33,6327	16,1566	244,29	
14,616	23,0814	-0,9475	335,89	cooling	14,616	38,5454	16,7616	304,51		14,616	33,1576	12,6827	286,21	
14,618	23,3006	3,2538	280,23	cooling	14,618	37,4195	14,3107	323,03		14,618	33,898	12,7777	295,23	
14,62	22,8325	2,7648	280,52	cooling	14,62	37,1968	10,3162	375,75		14,62	33,1551	10,6127	315,11	
14,622	23,0425	-1,7761	346,93	cooling	14,622	37,718	14,6745	322,11		14,622	33,252	9,3487	334,13	
14,624	23,6217	1,218	313,17	cooling	14,624	36,1151	12,2101	334,16		14,624	33,248	9,8422	327,18	
14,626	23,6116	-4,3003	390,17	cooling	14,626	37,7027	14,881	319,01		14,626	33,1404	9,503	330,42	
14,628	23,6788	-6,3438	419,67	cooling	14,628	38,0311	20,3664	246,93		14,628	33,1862	8,7224	341,97	
14,63	23,7651	-3,6498	383,22	cooling	14,63	37,3677	14,0602	325,8		14,63	33,4401	15,7608	247,13	
14,632	23,0721	-3,9262	377,4	cooling	14,632	37,9596	17,7319	282,75		14,632	32,9632	14,3956	259,55	
14,634	24,2659	2,8555	299,29	cooling	14,634	38,4056	14,529	333,76		14,634	33,2108	13,5022	275,5	
14,636	23,3537	4,7902	259,49	cooling	14,636	37,4732	13,5913	333,83		14,636	33,1274	12,3554	290,36	
14,638	24,1709	3,727	285,78	cooling	14,638	36,8698	10,7973	364,46		14,638	33,036	8,6162	341,35	
14,64	24,3662	4,733	274,44	cooling	14,64	37,9811	15,7376	310,93	cooling	14,64	33,6252	10,6508	321,15	
14,642	24,1656	1,0074	323,72	cooling	14,642	37,8821	10,0285	389,35	cooling	14,642	33,135	12,5956	287,11	
14,644	24,6634	-2,0301	373,13	cooling	14,644	37,6153	17,3419	283,39	cooling	14,644	33,2451	11,4852	304,17	
14,646	25,1841	2,9321	311,05	cooling	14,646	37,0361	12,7566	339,39	cooling	14,646	33,4937	11,1333	312,56	
14,648	24,6672	1,712	320,88	cooling	14,648	39,2868	15,2296	336,28	cooling	14,648	33,3026	11,0178	311,51	
14,65	24,2455	4,9826	269,27	cooling	14,65	38,2453	13,5668	344,97	cooling	14,65	33,2531	8,8897	340,56	
14,652	23,8393	5,6969	253,6	cooling	14,652	38,3623	13,3546	349,57	cooling	14,652	33,3381	10,3749	320,99	
14,654	23,5994	1,3609	310,86	cooling	14,654	38,2405	13,3086	348,51	cooling	14,654	33,1589	9,4397	331,56	
14,656	24,3337	4,2558	280,66	cooling	14,656	38,434	15,6121	319,02	cooling	14,656	33,5035	12,5247	293,25	
14,658	23,3325	4,3077	265,94	cooling	14,658	36,8081	13,862	320,75	cooling	14,658	32,9236	8,4135	342,61	
14,66	23,633	0,3636	325,27	cooling	14,66	37,9709	14,488	328,26	cooling	14,66	33,1966	6,4183	374,32	
14,662	23,3194	0,2226	322,86	cooling	14,662	37,3216	14,19	323,34	cooling	14,662	33,1907	7,6875	356,5	cooling
14,664	22,9345	-0,9963	334,52	cooling	14,664	37,606	8,8651	401,75	cooling	14,664	32,9881	10,0123	321,17	cooling
14,666	23,0392	0,5203	314,78	cooling	14,666	38,4324	14,9927	327,65	cooling	14,666	33,0826	12,2515	291,19	cooling
14,668	23,2476	4,5507	261,35	cooling	14,668	39,1467	13,1818	362,95	cooling	14,668	33,3664	16,0641	241,86	cooling
14,67	22,7931	-0,3797	323,92	cooling	14,67	38,4077	16,4421	307,05	cooling	14,67	33,1807	12,4571	289,68	
14,672	22,8464	-1,0111	333,49	cooling	14,672	37,9564	14,165	332,57	cooling	14,672	33,6354	12,0556	301,65	
14,674	22,4844	-0,3889	319,73	cooling	14,674	39,3149	17,4737	305,31	cooling	14,674	33,2175	10,1972	321,79	
14,676	22,2622	-1,0806	326,3	cooling	14,676	38,7524	12,1688	371,6	cooling	14,676	33,2608	8,8969	340,57	
14,678	22,549	1,1347	299,34	cooling	14,678	38,2267	15,2044	321,82	cooling	14,678	33,3062	13,0639	282,96	cooling
14,68	22,2021	0,6749	300,92	cooling	14,68	37,3939	8,8594	398,87	cooling	14,68	33,0106	14,9054	253,08	
14,682	22,7099	0,811	306,11	cooling	14,682	39,0377	10,1412	403,93	cooling	14,682	33,4662	14,0055	272,03	
14,684	22,4489	5,6204	235,24	cooling	14,684	37,1911	7,9961	408,1	cooling	14,684	33,5215	12,5233	293,52	
14,686	22,0829	5,5268	231,43	cooling	14,686	38,2893	12,8055	356,23	cooling	14,686	33,7723	11,9246	305,4	
14,688	21,9669	0,6452	298,05		14,688	37,3906	12,8641	342,84	cooling	14,688	33,7186	13,0831	288,45	
14,69	22,1004	-3,3537	355,81		14,69	38,1478	13,3848	346,15	cooling	14,69	33,7674	17,0531	233,64	
14,692	21,5825	-7,1688	401,9		14,692	37,0009	14,7596	310,9	cooling	14,692	33,1085	14,191	264,44	
14,694	22,0004	-2,5279	342,87	cooling	14,694	38,2967	14,5274	332,26	cooling	14,694	33,5438	15,5457	251,59	
14,696	21,6773	2,3948	269,54	cooling	14,696	37,2585	15,4816	304,41	cooling	14,696	32,6963	12,707	279,42	
14,698	21,5884	1,5484	280,13	cooling	14,698	36,795	10,4673	368,02	cooling	14,698	32,7712	11,9075	291,64	
14,7	21,8933	2,7578	267,49	cooling	14,7	37,9707	13,1814	346,52	cooling	14,7	32,6836	12,4037	283,48	
14,702	21,7611	3,7536	251,72		14,702	38,5064	12,0825	369,37	cooling	14,702	32,7368	11,5084	296,74	

14,704	22,0836	1,227	291,54	
14,706	21,9539	1,5605	285,07	
14,708	21,6553	-0,8142	314,09	
14,71	21,593	-2,6102	338,32	
14,712	22,1124	2,9782	267,47	
14,714	21,4988	4,6695	235,25	
14,716	21,892	2,4543	271,71	
14,718	21,9075	1,9934	278,37	
14,72	21,7734	0,1785	301,86	
14,722	21,7737	-0,8453	316,18	
14,724	22,2023	0,2982	306,19	
14,726	21,8219	-4,6979	370,71	
14,728	21,9829	-4,541	370,76	
14,73	21,7401	-1,5509	325,57	
14,732	21,5446	-0,2089	304,08	
14,734	22,0125	3,7097	255,85	
14,736	21,5076	2,4551	266,33	
14,738	22,1724	-0,6636	319,21	
14,74	22,1221	2,0002	281,27	
14,742	21,6252	1,2269	285,14	
14,744	21,5053	-0,7845	311,58	
14,746	22,2005	3,1906	265,73	
14,748	21,5325	0,2887	296,96	
14,75	21,7892	1,8305	278,99	
14,752	21,1667	2,8661	255,82	
14,754	20,6672	-2,363	321,93	
14,756	21,1833	-1,0469	310,74	
14,758	20,8689	3,905	237,13	
14,76	20,8534	2,5592	255,72	
14,762	20,7584	5,7947	209,17	
14,764	20,3371	4,4707	221,79	
14,766	20,187	-3,3383	328,85	heating
14,768	21,4648	-2,8473	339,85	heating
14,77	20,7605	-3,8368	343,83	
14,772	21,5794	-2,6517	338,71	
14,774	21,5179	1,2673	283,07	
14,776	21,3591	0,6948	288,86	
14,778	21,8132	2,6843	267,39	
14,78	22,0625	6,9246	211,61	
14,782	22,0279	0,2068	305,03	
14,784	22,2206	-3,2494	356,03	
14,786	21,8351	-6,1958	391,83	
14,788	21,6251	-6,9167	398,97	
14,79	22,5319	3,0869	271,81	
14,792	21,4448	5,4375	223,76	
14,794	21,8687	2,9844	263,97	
14,796	21,6104	3,5933	251,85	
14,798	21,1543	0,9696	282,15	
14,8	21,3424	-0,0246	298,68	
14,802	21,6653	1,1717	286,47	
14,804	21,4056	-6,6399	392,03	
14,806	22,0122	-3,3155	354,04	
14,808	21,6798	2,7403	264,75	
14,81	21,3184	1,4355	277,93	
14,812	22,24	2,8672	270,8	
14,814	21,4437	0,7418	289,38	

14,704	37,5524	15,649	306,18	cooling
14,706	37,2625	10,8725	368,89	cooling
14,708	39,5211	16,7903	317,74	cooling
14,71	38,1	10,4572	386,41	cooling
14,712	38,3133	14,8436	328,07	cooling
14,714	37,2647	12,0817	352,02	cooling
14,716	38,4471	12,226	366,53	cooling
14,718	36,5347	10,6679	361,58	cooling
14,72	37,3144	16,6201	289,28	cooling
14,722	36,4882	17,3786	267,12	cooling
14,724	36,859	17,4329	271,55	cooling
14,726	36,0728	14,8033	297,32	cooling
14,728	37,9035	10,5452	382,43	cooling
14,73	37,2174	14,0745	323,5	cooling
14,732	36,4871	8,6779	388,73	cooling
14,734	38,357	14,7818	329,55	cooling
14,736	37,7689	14,6357	323,37	cooling
14,738	37,0396	17,2684	276,37	cooling
14,74	36,5144	13,3746	323,46	cooling
14,742	37,9562	15,7566	310,32	cooling
14,744	36,2018	9,6639	370,96	cooling
14,746	36,9446	17,8801	266,49	cooling
14,748	35,6996	15,7518	278,84	cooling
14,75	36,7636	14,7219	308,11	cooling
14,752	35,5018	16,65	263,52	cooling
14,754	36,3299	18,3897	250,78	cooling
14,756	36,0155	18,3871	246,42	cooling
14,758	36,0588	17,2764	262,55	cooling
14,76	36,0356	14,9403	294,88	cooling
14,762	37,7706	12,4231	354,32	cooling
14,764	36,758	19,0095	248,1	cooling
14,766	36,4369	17,5013	264,69	cooling
14,768	37,8753	23,2971	203,78	cooling
14,77	37,4397	16,876	287,45	
14,772	36,8413	16,6616	282,08	
14,774	36,2357	13,1683	322,45	
14,776	37,2533	16,354	292,14	
14,778	35,6495	15,2441	285,24	
14,78	36,1182	20,7055	215,45	
14,782	35,3063	16,832	258,24	
14,784	36,5204	16,4928	279,96	
14,786	35,0516	14,2281	291,08	
14,788	36,8767	15,3194	301,34	
14,79	36,2069	18,7605	243,88	
14,792	36,0471	16,9355	267,15	
14,794	36,4383	19,3396	239,01	
14,796	37,6859	18,9089	262,48	
14,798	36,1847	19,3645	235,12	
14,8	36,2785	16,3544	278,51	heating
14,802	37,7247	20,2477	244,3	heating
14,804	37,1314	15,3907	303,9	heating
14,806	37,4908	21,658	221,32	heating
14,808	36,5263	18,029	258,57	heating
14,81	38,5705	20,5282	252,21	heating
14,812	36,2559	15,1183	295,47	heating
14,814	36,769	15,7989	293,13	heating

14,704	33,1531	10,6425	314,66	
14,706	33,7894	14,6706	267,25	
14,708	33,4009	11,406	307,46	
14,71	33,9831	12,408	301,59	
14,712	33,5413	15,1491	257,1	
14,714	33,1638	12,7257	285,69	
14,716	33,0363	13,3758	274,82	
14,718	33,0186	15,2198	248,8	
14,72	32,8286	13,8719	264,99	
14,722	33,4292	15,3857	252,22	heating
14,724	33,1064	13,8362	269,37	heating
14,726	33,6041	9,1779	341,44	heating
14,728	33,9953	11,1075	319,94	heating
14,73	33,6134	12,7674	291,4	
14,732	33,6938	15,0917	260,03	
14,734	33,4648	15,8555	246,15	heating
14,736	33,5224	13,0461	286,23	heating
14,738	33,321	10,332	321,35	heating
14,74	33,8251	12,1457	303,05	heating
14,742	33,4168	11,1683	311	heating
14,744	33,8135	9,6263	338,1	
14,746	33,3346	10,4509	319,88	
14,748	33,8895	14,8873	265,62	
14,75	33,3777	17,4145	223,14	
14,752	33,4998	16,1645	242,32	heating
14,754	33,614	15,7582	249,6	heating
14,756	33,5836	12,8045	290,46	heating
14,758	33,6691	14,3611	269,9	heating
14,76	33,4649	15,0804	256,99	heating
14,762	33,7511	13,9655	276,57	heating
14,764	33,7349	12,4685	297,27	heating
14,766	33,5784	14,4467	267,43	heating
14,768	33,087	12,303	290,53	heating
14,77	33,474	14,407	266,53	heating
14,772	32,7849	11,3579	299,52	heating
14,774	33,3015	10,7913	314,66	heating
14,776	33,7057	14,7456	265,03	heating
14,778	33,6375	15,0431	259,92	heating
14,78	34,0245	14,485	273,13	heating
14,782	34,5002	16,049	257,92	heating
14,784	34,7494	15,4563	269,69	heating
14,786	34,8159	13,919	292,11	heating
14,788	34,8672	13,8308	294,06	heating
14,79	34,4071	10,8867	328,78	heating
14,792	34,6774	15,1455	273,03	heating
14,794	34,1307	17,9135	226,69	heating
14,796	34,2651	19,176	210,92	heating
14,798	34,2189	16,0446	254,05	heating
14,8	34,6185	15,1562	272,05	heating
14,802	34,8283	13,4326	299,08	heating
14,804	35,5818	16,1066	272,23	heating
14,806	35,4543	15,0236	285,59	heating
14,808	35,6586	15,3855	283,39	heating
14,81	35,261	15,2221	280,11	heating
14,812	35,0468	15,0028	280,19	heating
14,814	34,5305	14,4188	281,13	heating

14,816	22,1156	-1,4368	329,23
14,818	22,0168	3,975	252,2
14,82	21,4844	0,9673	286,8
14,822	21,8311	-4,3401	365,84
14,824	22,6257	-0,9862	330,06
14,826	22,0434	-3,5029	357,1
14,828	22,3177	0,5253	304,63
14,83	21,8343	2,962	263,81
14,832	21,3594	-1,4599	318,98
14,834	22,411	2,6622	276,06
14,836	21,0764	1,9616	267,2
14,838	21,8793	-2,1517	335,92
14,84	21,974	3,0052	265,15
14,842	21,5521	1,8618	275,24
14,844	21,6192	-0,9275	315,17
14,846	22,468	3,3144	267,74
14,848	21,4978	-4,81	367,74
14,85	22,4614	-2,5238	349,26
14,852	22,0334	2,7646	269,35
14,854	21,4711	-0,364	305,22
14,856	22,4406	3,6811	262,23
14,858	21,404	4,4534	236,94
14,86	22,4234	-1,4491	333,7
14,862	22,2474	3,4305	263,03
14,864	21,5166	0,5961	292,44
14,866	21,2966	-3,8056	350,89
14,868	22,4915	3,567	264,54
14,87	21,0469	-1,6577	317,38
14,872	22,1369	1,3063	291,18
14,874	21,3725	5,2555	225,29
14,876	20,8779	0,3419	287,06
14,878	21,4994	2,1533	270,43
14,88	21,0702	2,5439	258,97
14,882	21,5494	-6,8328	396,74
14,884	22,0205	-2,1348	337,66
14,886	21,6504	0,159	300,42
14,888	21,6131	0,6421	293,14
14,89	22,7012	7,0775	218,4
14,892	20,97	-1,5047	314,16
14,894	22,5823	-1,6416	338,61
14,896	21,6268	2,4836	267,59
14,898	21,3327	-3,1882	342,76
14,9	22,1804	-4,1813	368,5
14,902	22,2474	-1,9579	338,35
14,904	22,7815	-3,8667	372,5
14,906	23,0456	6,9336	225,22
14,908	22,3786	7,3995	209,38
14,91	22,3381	0,2227	309,14
14,912	23,6459	2,1713	300,18
14,914	22,1337	-4,5708	373,29
14,916	23,4672	-3,8037	381,21
14,918	22,4158	2,5077	278,29
14,92	21,8412	-0,5341	312,77
14,922	22,1978	-0,6177	318,93
14,924	22,7338	5,4836	241,13
14,926	22,6812	1,4397	296,92

14,816	35,9208	14,8612	294,38	heating
14,818	36,6678	16,693	279,22	heating
14,82	35,2783	14,1601	295,2	heating
14,822	37,3427	18,4037	264,74	heating
14,824	36,1639	18,8154	242,51	heating
14,826	36,2316	16,8823	270,48	heating
14,828	36,9925	22,5351	202,09	heating
14,83	37,5052	18,8467	260,82	heating
14,832	37,1752	22,3959	206,59	heating
14,834	36,2127	18,6054	246,12	heating
14,836	37,8204	17,3096	286,71	heating
14,838	37,3041	11,8201	356,23	heating
14,84	36,8627	15,933	292,57	heating
14,842	36,5141	14,1277	312,93	heating
14,844	38,2699	22,7801	216,52	heating
14,846	36,2484	18,6172	246,46	heating
14,848	37,4742	19,1193	256,57	heating
14,85	36,5462	17,5132	266,05	heating
14,852	36,5552	13,5465	321,63	heating
14,854	36,365	15,6543	289,51	heating
14,856	37,3192	17,9735	270,42	heating
14,858	36,7036	18,096	260,11	heating
14,86	36,6062	17,2748	270,22	heating
14,862	37,5535	20,6456	236,35	heating
14,864	37,913	16,5574	298,52	heating
14,866	37,7648	22,6782	210,89	heating
14,868	36,7373	16,3412	285,11	heating
14,87	38,4787	18,9598	272,85	heating
14,872	36,6153	14,9435	302,94	heating
14,874	37,0588	18,8453	254,6	heating
14,876	36,7006	20,4311	227,42	heating
14,878	37,1913	21,4082	220,62	heating
14,88	36,4187	16,2675	281,68	heating
14,882	38,1708	15,9319	310,87	heating
14,884	37,1334	13,5949	329,03	heating
14,886	37,2768	13,4611	332,91	heating
14,888	37,8101	19,525	255,6	heating
14,89	38,3112	16,2708	308,09	heating
14,892	37,9534	19,6253	256,2	heating
14,894	36,8852	13,3001	329,68	heating
14,896	39,1925	14,953	338,83	heating
14,898	38,7083	11,0691	386,35	heating
14,9	38,5951	15,5888	321,59	heating
14,902	38,3987	13,6918	345,37	heating
14,904	40,3212	21,2495	266,59	heating
14,906	39,0638	15,4533	330,04	heating
14,908	38,6791	19,1642	272,79	heating
14,91	37,6236	14,3045	325,97	heating
14,912	38,4017	13,6345	346,21	heating
14,914	36,92	10,9628	362,84	
14,916	37,6412	13,3766	339,18	
14,918	37,448	14,3513	322,86	
14,92	37,5862	17,294	283,66	
14,922	37,1159	17,2065	278,3	
14,924	38,4726	14,4034	336,45	
14,926	37,913	15,1964	317,54	

14,816	34,1767	14,1841	279,47	heating
14,818	33,8282	15,6799	253,69	heating
14,82	33,6787	15,176	258,64	heating
14,822	33,8987	12,6809	296,59	heating
14,824	34,3704	8,8352	356,94	heating
14,826	34,9685	12,0778	319,98	heating
14,828	35,0448	14,5138	286,99	heating
14,83	35,3576	18,6829	233,09	heating
14,832	35,0928	15,4611	274,42	heating
14,834	35,1499	10,153	349,42	heating
14,836	35,193	6,1563	405,89	heating
14,838	35,1765	7,8406	382,12	heating
14,84	34,7785	8,9152	361,53	
14,842	35,0611	13,4452	302,16	
14,844	34,7485	14,2564	286,45	
14,846	35,0222	12,9142	309,04	
14,848	35,0275	14,3056	289,66	
14,85	34,9976	12,6109	312,93	
14,852	35,0461	13,5037	301,13	
14,854	35,2001	16,3281	263,8	
14,856	34,8871	16,0159	263,79	
14,858	35,2629	12,7181	315,14	
14,86	35,085	12,3637	317,61	
14,862	34,8812	7,3421	384,96	
14,864	35,3533	9,5475	360,73	
14,866	35,0234	10,6364	340,89	
14,868	34,8514	9,0061	361,28	
14,87	35,0245	8,5396	370,22	
14,872	35,0766	9,2726	360,7	
14,874	34,6745	9,2565	355,31	cooling
14,876	35,0497	15,6453	271,24	cooling
14,878	34,1166	15,6829	257,68	cooling
14,88	34,6051	11,3	325,77	
14,882	34,4075	9,0321	354,71	
14,884	34,6448	4,9003	415,78	cooling
14,886	34,6647	8,1818	370,19	cooling
14,888	34,8128	16,672	253,58	cooling
14,89	34,1355	19,1856	208,98	cooling
14,892	34,659	17,2624	243,18	cooling
14,894	34,3327	14,4452	278	
14,896	34,3163	8,3018	363,64	
14,898	34,8184	11,3129	328,57	
14,9	34,4685	13,2596	296,47	cooling
14,902	34,9118	14,8605	280,29	
14,904	35,1219	17,7959	242,19	
14,906	35,1972	16,2699	264,58	
14,908	34,8811	10,7273	337,63	
14,91	35,3156	10,035	353,38	
14,912	34,587	8,841	359,89	
14,914	35,3229	14,3935	292,56	cooling
14,916	34,4665	16,9247	245,21	
14,918	34,859	12,0761	318,47	
14,92	35,0985	11,6395	327,92	
14,922	35,2664	14,0724	296,26	
14,924	35,2139	15,5145	275,37	
14,926	35,4554	16,7788	261,07	

14,928	23,3243	7,2523	224,66		14,928	37,4119	9,6302	388,35	14,928	35,1073	14,2684	291,3	
14,93	22,9784	5,5435	243,71		14,93	38,9523	15,7089	324,91	14,93	35,1386	9,5578	357,58	
14,932	23,2755	0,6959	315,63		14,932	38,8552	14,4344	341,37	14,932	35,5107	11,1771	340,15	
14,934	24,5528	7,5652	237,46		14,934	38,1566	18,843	269,97	14,934	34,5354	10,1664	340,64	
14,936	23,3055	7,4385	221,8		14,936	37,8371	17,2176	288,23	14,936	34,7028	14,4195	283,53	
14,938	24,3987	3,8194	287,67		14,938	38,9573	19,1455	276,94	14,938	33,9374	15,4093	259	
14,94	23,7983	2,7301	294,5		14,94	37,7184	12,1224	357,8	14,94	34,4548	12,2924	309,8	
14,942	23,2902	-2,4377	359,64		14,942	38,1333	15,3056	319,1	14,942	34,3833	6,6198	388,09	
14,944	23,1618	-3,5825	373,85		14,944	37,6121	13,502	337,02	14,944	34,8683	5,4453	411,29	
14,946	23,2431	1,6286	302,14		14,946	38,0479	14,3777	330,87	14,946	34,5973	5,963	400,27	cooling
14,948	22,4458	-1,4297	333,74		14,948	37,3119	15,9012	299,29	14,948	34,9712	12,7796	310,21	cooling
14,95	22,7991	3,3315	272,13		14,95	38,4812	17,6185	291,63	14,95	34,374	15,1147	269,22	cooling
14,952	22,171	5,4721	233,43		14,952	37,7296	19,4901	254,96	14,952	34,8657	13,803	294,43	cooling
14,954	21,6571	1,8639	276,68		14,954	37,3882	17,2491	281,51	14,954	34,8836	12,5275	312,51	cooling
14,956	22,3153	1,613	289,39		14,956	38,0447	18,6149	271,6	14,956	34,5552	8,5258	363,85	cooling
14,958	21,6779	-2,9255	343,92		14,958	39,0606	16,0596	321,52	14,958	34,8692	9,5287	354,22	cooling
14,96	22,8106	-7,1312	418,54		14,96	38,2054	18,2564	278,86	14,96	34,9034	12,1279	318,37	cooling
14,962	22,9457	-2,1627	350,98		14,962	37,8742	14,7641	323,04	14,962	34,8107	14,312	286,54	cooling
14,964	22,7366	-0,3001	322,02		14,964	39,6175	20,6536	265,09	14,964	34,824	13,7743	294,24	cooling
14,966	22,8675	0,9976	305,71		14,966	38,5471	16,5255	307,83	14,966	35,0445	15,1865	277,59	cooling
14,968	23,5857	6,9353	232,75		14,968	38,2437	19,112	267,43	14,968	34,6603	10,4928	337,83	cooling
14,97	22,598	2,0598	287,09		14,97	37,6803	15,4886	310,21	14,97	34,9194	9,8239	350,8	cooling
14,972	23,2222	2,0622	295,79		14,972	38,7601	17,6687	294,83	14,972	34,6062	9,2121	354,97	cooling
14,974	22,7576	3,6617	266,93		14,974	37,2931	12,4187	347,71	14,974	34,7902	12,1849	315,99	cooling
14,976	22,3203	-0,7838	322,96		14,976	37,463	16,5134	292,84	14,976	34,4452	13,8437	287,98	cooling
14,978	22,8049	-1,7153	342,76		14,978	37,0142	15,7382	297,41	14,978	34,3846	12,6322	304,07	cooling
14,98	22,4793	-0,8029	325,45		14,98	37,8041	18,1398	274,88	14,98	34,3486	9,4468	348,09	cooling
14,982	23,0328	-1,288	339,97		14,982	36,5451	16,7011	277,39	14,982	34,4749	8,6185	361,43	cooling
14,984	23,0823	8,0451	210,2		14,984	37,5954	17,9221	275	14,984	34,1847	10,4639	331,58	cooling
14,986	22,6825	9,8743	179,04		14,986	36,9577	16,0627	292,08	14,986	34,4978	14,014	286,33	cooling
14,988	22,6262	4,1361	258,46		14,988	37,165	14,0831	322,65	14,988	34,1277	14,8406	269,61	cooling
14,99	23,6906	4,72	265,18		14,99	37,4613	14,9826	314,22	14,99	34,1102	9,064	350,11	cooling
14,992	22,6346	0,4508	310,1		14,992	38,4484	13,7289	345,54	14,992	34,2407	7,8329	369,14	cooling
14,994	23,4907	1,7894	303,35		14,994	37,5451	18,9102	260,49	14,994	34,0011	7,3217	372,94	cooling
14,996	23,226	6,4247	234,86		14,996	37,2265	19,7208	244,7	14,996	33,6739	10,7347	320,65	cooling
14,998	22,8202	2,5864	282,84		14,998	38,1733	23,21	209,16	14,998	33,9082	14,0381	277,75	cooling
15	23,3041	0,7525	315,24		15	38,6816	16,9742	303,44	15	33,8679	14,163	275,45	cooling
15,002	23,153	2,2172	292,65		15,002	37,6947	16,1497	301,17	15,002	34,0199	9,5888	341,51	cooling
15,004	23,439	0,3507	322,74		15,004	37,3923	11,0882	367,69	15,004	34,0367	8,9412	350,8	cooling
15,006	23,6531	6,973	233,16		15,006	38,8454	15,999	319,36	15,006	33,6026	7,7043	362,02	cooling
15,008	23,1849	5,8366	242,5		15,008	37,9748	13,2607	345,47	15,008	33,7496	12,2887	299,99	cooling
15,01	23,1022	-1,3911	342,38	heating	15,01	37,899	17,5387	284,61	15,01	33,0899	15,1395	250,92	cooling
15,012	23,9328	1,4595	314,14		15,012	37,4938	14,3891	322,97	15,012	33,1087	14,0718	266,11	cooling
15,014	22,5786	0,4004	310,02		15,014	38,4168	13,679	345,8	15,014	33,1917	12,759	285,62	cooling
15,016	23,5041	1,9523	301,26		15,016	37,0431	11,0431	363,44	15,016	33,3036	11,6376	302,86	cooling
15,018	23,2291	3,7219	272,68		15,018	38,4548	14,98	328,14	15,018	33,2805	9,9997	325,43	cooling
15,02	23,1533	-2,0458	352,25		15,02	37,4339	16,2935	295,51	15,02	34,2474	12,9413	297,83	cooling
15,022	23,7291	-3,0059	373,72		15,022	37,8904	16,5719	298	15,022	33,891	13,4516	285,71	cooling
15,024	23,7103	0,8737	319,22		15,024	37,7637	18,2358	272,97	15,024	34,2502	12,2854	307,03	cooling
15,026	23,9637	0,9665	321,47		15,026	39,1506	15,7749	326,76	15,026	34,2555	13,0809	295,99	cooling
15,028	24,158	6,1396	251,87		15,028	38,1271	17,7133	285,35	15,028	34,4283	9,5566	347,67	cooling
15,03	23,8372	3,8904	278,83		15,03	37,979	16,5621	299,38	15,03	34,1699	8,4065	360,13	cooling
15,032	23,8745	-1,8952	360,22		15,032	39,0203	19,1832	277,29	15,032	34,2069	10,4645	331,88	cooling
15,034	24,6643	1,8892	318,36		15,034	38,9663	15,123	333,29	15,034	34,0505	13,2713	290,46	cooling
15,036	23,747	1,5214	310,68		15,036	38,2762	18,3711	278,24	15,036	33,882	12,0726	304,86	cooling
15,038	24,6943	1,7773	320,35		15,038	37,9501	16,1927	304,14	15,038	33,8553	9,4834	340,68	cooling

15,04	24,3098	3,1911	295,21		15,04	39,1316	20,9281	254,46		15,04	34,035	5,7919	394,8	cooling
15,042	24,0449	-1,0345	350,57		15,042	37,1231	16,5175	288,04		15,042	34,4497	7,175	381,26	cooling
15,044	24,2808	-1,052	354,11		15,044	38,0528	16,8759	296,02		15,044	33,7913	10,5404	325,01	cooling
15,046	24,4023	3,3357	294,48		15,046	37,4843	16,5161	293,11		15,046	33,7226	14,2419	272,31	cooling
15,048	24,2294	2,7016	300,93		15,048	37,8973	16,3625	301,02		15,048	33,4696	13,9537	272,8	cooling
15,05	24,4799	5,9756	258,66		15,05	36,8683	15,6443	296,68		15,05	33,9794	12,5999	298,85	cooling
15,052	23,9715	2,7424	296,75		15,052	38,6759	18,6428	280,03		15,052	33,1714	11,6753	300,48	cooling
15,054	23,8766	-4,5499	397,36		15,054	37,3491	18,9594	257,06		15,054	33,4068	11,8987	300,65	cooling
15,056	24,4533	-2,5197	377,04		15,056	37,4905	17,561	278,59		15,056	32,665	13,4479	268,63	cooling
15,058	23,7241	0,6277	322,85	cooling	15,058	38,3753	20,2158	253,84		15,058	32,697	13,558	267,53	cooling
15,06	24,1877	4,4212	276,31	cooling	15,06	39,2678	15,6846	329,66		15,06	32,5706	13,254	270,02	cooling
15,062	23,8294	8,6607	212,04		15,062	38,4952	16,4346	308,37		15,062	33,2208	13,0662	281,73	cooling
15,064	23,605	6,8861	233,71		15,064	37,9521	14,3627	329,74		15,064	33,1685	11,6766	300,42	cooling
15,066	23,4775	2,4872	293,41		15,066	39,3161	17,2628	308,27		15,066	33,2306	11,0882	309,52	
15,068	24,2364	2,6626	301,57		15,068	37,727	12,5987	351,26		15,068	33,3202	11,304	307,75	
15,07	24,0745	-0,2917	340,6		15,07	38,2244	17,2666	292,96		15,07	33,0244	8,8979	337,25	
15,072	24,6672	1,6564	321,66		15,072	37,2991	16,2417	294,35		15,072	33,5158	10,2628	325,04	cooling
15,074	24,3128	0,1138	338,27		15,074	39,1104	20,3143	262,74		15,074	32,594	8,9594	330,38	cooling
15,076	24,35	-3,3803	387,63		15,076	37,2169	17,3157	278,19		15,076	33,2952	10,4464	319,39	cooling
15,078	24,9684	1,1854	332,45		15,078	38,2856	16,4119	305,76		15,078	32,7027	12,3912	283,92	cooling
15,08	24,6887	7,1863	244,66	cooling	15,08	37,3032	13,2328	336,47		15,08	32,9088	13,7084	268,39	cooling
15,082	25,4129	8,1629	241,13		15,082	37,6828	15,9853	303,3		15,082	32,5154	14,3046	254,56	
15,084	25,2484	8,1934	238,4		15,084	36,6651	16,0718	287,86		15,084	33,2062	18,0896	211,31	
15,086	25,0808	-0,2609	354,24		15,086	37,6964	16,645	294,27		15,086	32,3393	12,0839	283,14	
15,088	25,182	-6,3698	441,05		15,088	36,8169	18,6177	254,4		15,088	32,841	11,4135	299,52	
15,09	25,8793	0,406	356,08	cooling	15,09	36,3845	15,0576	298,12		15,09	32,4718	14,4269	252,24	
15,092	24,8834	1,3493	328,97	cooling	15,092	38,424	18,4304	279,48		15,092	32,1652	15,3639	234,86	
15,094	25,3893	3,5745	304,94	cooling	15,094	38,3299	13,5502	346,38		15,094	32,6412	20,9426	163,53	
15,096	25,1194	3,3589	304,18	cooling	15,096	38,4183	13,8732	343,1		15,096	32,3106	21,7364	147,81	
15,098	24,6225	-1,5491	365,84	cooling	15,098	37,8332	11,8163	363,68		15,098	32,7399	15,8632	235,91	heating
15,1	25,2859	0,8004	342,27	cooling	15,1	39,36	16,9183	313,7		15,1	32,6702	14,1016	259,56	heating
15,102	24,4499	3,0328	299,38	cooling	15,102	37,566	12,4399	351,23		15,102	32,4706	13,7872	261,17	heating
15,104	24,8985	0,3219	343,54	cooling	15,104	37,9441	16,8676	294,62		15,104	32,0966	10,1369	306,97	heating
15,106	24,8815	3,7523	295,36	cooling	15,106	36,5609	12,0198	343,05		15,106	33,4128	15,9118	244,64	heating
15,108	24,5799	2,7331	305,39	cooling	15,108	37,8162	15,7647	308,25		15,108	32,6119	13,833	262,5	heating
15,11	24,5324	0,1941	340,21	cooling	15,11	36,2978	12,3651	334,54		15,11	33,2304	14,2992	264,63	heating
15,112	25,5619	7,383	254,11	cooling	15,112	36,9925	11,3251	358,79		15,112	32,7939	15,2534	245,19	heating
15,114	24,6317	5,6202	265,75	cooling	15,114	37,0202	10,5904	369,45	cooling	15,114	32,5245	11,7338	290,62	heating
15,116	25,3804	3,3906	307,38	cooling	15,116	36,9713	12,1128	347,48	cooling	15,116	32,8449	8,8793	335	heating
15,118	25,1547	3,0797	308,58	cooling	15,118	36,5354	11,5352	349,47	cooling	15,118	32,957	11,0231	306,6	heating
15,12	24,6165	-2,9178	384,89	cooling	15,12	37,9493	14,2774	330,9	cooling	15,12	33,1226	10,7748	312,39	heating
15,122	25,3449	-1,3682	373,41	cooling	15,122	37,0369	14,9876	308,22	cooling	15,122	33,3273	14,8769	257,91	heating
15,124	25,384	7,7421	246,61	cooling	15,124	37,1057	11,8466	353,09	cooling	15,124	32,785	15,8723	236,41	heating
15,126	24,9636	7,8045	239,86	cooling	15,126	38,4119	17,9496	286,03	cooling	15,126	32,4649	9,3479	323,14	heating
15,128	25,0934	9,0895	223,71	cooling	15,128	38,1251	13,0836	350,04	cooling	15,128	33,4695	10,7999	316,89	heating
15,13	24,854	5,7441	267,13		15,13	37,6523	13,888	332,19	cooling	15,13	32,6261	9,8981	317,7	heating
15,132	24,6098	-4,2026	402,76		15,132	37,4431	14,7913	316,64	cooling	15,132	32,8168	12,8247	279,46	heating
15,134	25,1254	-5,5615	428,96	cooling	15,134	38,5309	18,9707	273,42	cooling	15,134	32,5898	16,8167	220,48	heating
15,136	24,7817	-3,9449	401,56	cooling	15,136	37,484	16,4161	294,5	cooling	15,136	32,3862	14,7005	247,22	heating
15,138	25,1031	-0,8409	362,66	cooling	15,138	37,7747	18,5464	268,78	cooling	15,138	32,6093	8,8481	332,15	heating
15,14	24,7782	4,1597	288,22	cooling	15,14	36,8532	11,4869	354,58	cooling	15,14	33,4056	10,9358	314,09	heating
15,142	24,0167	3,3221	289,28	cooling	15,142	37,8606	11,108	373,96	cooling	15,142	33,4894	8,2669	352,57	heating
15,144	24,1418	2,5899	301,26	cooling	15,144	36,3107	10,3156	363,37	cooling	15,144	33,6814	12,2444	299,66	heating
15,146	23,9296	2,9732	292,94	cooling	15,146	37,3447	12,2853	350,29	cooling	15,146	33,2038	15,7784	243,58	heating
15,148	23,2234	-1,5966	346,95	cooling	15,148	36,9125	15,7771	295,44	cooling	15,148	33,019	13,2699	276,06	heating
15,15	23,446	-2,8009	366,89	cooling	15,15	36,6629	16,0989	287,45	cooling	15,15	33,4123	12,4217	293,42	heating

15,152	23,4082	-4,1499	385,22	cooling	15,152	37,0997	16,976	281,3	cooling	15,152	33,1726	12,8619	283,91	heating
15,154	23,7193	-5,6793	410,95	cooling	15,154	37,9732	15,0604	320,29	cooling	15,154	33,0581	9,6465	327,26	heating
15,156	23,7371	-0,1498	333,9	cooling	15,156	36,865	12,724	337,46	cooling	15,156	33,4752	10,8192	316,7	heating
15,158	23,8019	6,1869	246,23	cooling	15,158	36,5495	11,0746	356,1	cooling	15,158	33,0621	10,9123	309,62	heating
15,16	23,795	8,0953	219,46	cooling	15,16	38,032	19,8279	254,47	cooling	15,16	33,0491	8,5531	342,42	heating
15,162	23,7013	7,696	223,73	cooling	15,162	36,7455	14,7239	307,83	cooling	15,162	33,6278	14,4779	267,69	
15,164	23,434	2,2148	296,61	cooling	15,164	36,8798	17,4805	271,17	cooling	15,164	33,3167	15,2276	252,86	heating
15,166	24,0212	0,3014	331,57	cooling	15,166	35,905	15,7677	281,49	cooling	15,166	33,4208	12,5228	292,12	heating
15,168	23,9562	0,9528	321,55	cooling	15,168	37,2534	16,0758	296,03	cooling	15,168	33,5601	12,8451	289,57	heating
15,17	23,3875	1,5178	305,71	cooling	15,17	35,9204	14,7851	295,44	cooling	15,17	33,267	12,6628	288,02	heating
15,172	23,5726	3,1171	285,94	cooling	15,172	36,2966	15,4166	291,87	cooling	15,172	32,9441	9,0742	333,67	heating
15,174	23,1762	2,1041	294,56	cooling	15,174	35,6401	9,156	370,21	cooling	15,174	33,5416	12,6201	292,45	heating
15,176	23,5394	0,6664	319,73	cooling	15,176	36,6468	13,9544	317,21	cooling	15,176	32,8983	9,8311	322,45	heating
15,178	23,4477	2,5061	292,73	cooling	15,178	35,2901	15,8507	271,73	cooling	15,178	33,7685	13,0844	289,13	
15,18	23,8495	2,5861	297,23	cooling	15,18	36,8083	15,2617	301,19	cooling	15,18	33,0784	16,9381	225,62	
15,182	23,9377	0,2152	331,61	cooling	15,182	36,5047	17,3464	267,8	cooling	15,182	33,0246	15,1348	250,07	heating
15,184	23,9215	-0,5427	341,97	cooling	15,184	35,8888	11,615	339,31	cooling	15,184	33,0286	11,4077	302,23	heating
15,186	23,728	-1,9357	358,74	cooling	15,186	37,8107	15,1564	316,67	cooling	15,186	33,7765	13,0155	290,21	heating
15,188	24,0284	0,0757	334,82	cooling	15,188	38,1425	16,3093	305,2	cooling	15,188	33,0212	8,0744	348,72	heating
15,19	24,0527	2,0625	307,39	cooling	15,19	37,375	16,8629	286,73	cooling	15,19	33,7948	12,2795	300,75	heating
15,192	23,7068	2,1775	300,95	cooling	15,192	37,0526	15,7085	298,36	cooling	15,192	33,0283	16,8157	226,63	
15,194	24,2081	2,0537	309,69	cooling	15,194	38,2895	20,5558	247,89	cooling	15,194	32,8127	13,9932	263,07	heating
15,196	23,616	0,7076	320,22	cooling	15,196	36,9696	13,0413	334,48		15,196	33,3245	14,7656	259,43	heating
15,198	23,8519	-0,6761	342,87	cooling	15,198	36,881	17,5997	269,52		15,198	33,4548	13,9807	272,22	heating
15,2	23,4754	-1,7333	352,38	cooling	15,2	35,7421	14,1304	302,1		15,2	33,6736	8,2579	355,27	heating
15,202	24,116	0,4962	330,17	cooling	15,202	36,7773	16,4882	283,61		15,202	33,6674	8,9106	346,06	heating
15,204	23,7114	3,8056	278,25	cooling	15,204	35,4548	19,1122	228,45		15,204	33,4782	10,8874	315,79	
15,206	23,4607	5,4803	251,34	cooling	15,206	35,4852	17,9384	245,28		15,206	33,1332	8,2916	347,25	
15,208	23,1424	1,6171	300,89	cooling	15,208	35,8966	15,8986	279,54		15,208	34,02	13,8052	282,57	
15,21	23,2939	-1,2206	342,68	cooling	15,21	35,8866	16,6524	268,87		15,21	32,9345	12,5201	285,36	
15,212	22,992	-3,911	376,06	cooling	15,212	35,7137	16,3067	271,28		15,212	33,6456	10,4148	324,73	
15,214	23,1136	-0,9692	336,64	cooling	15,214	37,2552	16,5904	288,86		15,214	33,1084	11,2751	305,2	
15,216	23,2427	3,9754	269,33	cooling	15,216	36,5433	16,6518	278,05		15,216	33,0529	9,9326	323,19	
15,218	22,905	5,7062	240,41	cooling	15,218	36,1956	9,6449	371,14		15,218	32,9789	7,3447	358,33	
15,22	23,2796	6,2499	238,05	cooling	15,22	37,6743	15,2381	313,62		15,22	34,2296	15,5873	260,59	
15,222	23,1472	4,3258	263,09	cooling	15,222	37,3259	12,822	342,53		15,222	33,3988	17,4312	223,2	
15,224	23,6481	2,2166	299,58		15,224	36,5568	15,103	299,89		15,224	34,0919	16,0028	252,86	
15,226	23,3298	1,1618	309,88		15,226	35,9029	13,7975	309		15,226	33,7848	14,6407	267,61	
15,228	23,2556	0,1173	323,44		15,228	36,8845	16,3267	287,37		15,228	33,544	9,5845	334,92	
15,23	23,3442	-1,2807	344,22		15,23	35,8447	13,3553	314,37		15,23	33,794	10,6027	324,18	
15,232	23,421	-0,6707	336,77	cooling	15,232	35,2629	14,3444	292,41		15,232	33,785	15,053	261,85	
15,234	23,193	-0,3391	328,94	cooling	15,234	35,0388	11,3874	330,61		15,234	33,5074	14,4917	265,81	
15,236	23,4572	3,7048	276,11	cooling	15,236	35,7745	14,8672	292,25		15,236	33,7484	12,4038	298,37	
15,238	23,5119	5,0884	257,53	cooling	15,238	34,2934	14,534	276,21		15,238	33,8312	12,7615	294,52	
15,24	23,2631	4,8902	256,83		15,24	35,1363	14,2725	291,65		15,24	33,2792	8,1864	350,76	
15,242	23,4719	2,576	292,09		15,242	34,7959	13,6531	295,54		15,242	33,8093	10,3383	328,09	
15,244	22,9784	-4,9156	389,92		15,244	35,0404	11,2652	332,34		15,244	33,561	11,6561	306,2	
15,246	23,9543	-5,3517	409,65		15,246	35,2308	16,2042	265,96		15,246	33,7331	11,0047	317,71	
15,248	23,3428	-1,7812	351,2	cooling	15,248	36,567	18,5583	251,73		15,248	33,132	9,2098	334,4	
15,25	22,8904	0,4218	314,08	cooling	15,25	35,5556	18,1164	243,77		15,25	33,0236	9,4029	330,18	
15,252	22,5089	1,4437	294,46	cooling	15,252	35,7206	14,941	290,47		15,252	32,75	9,3766	326,73	
15,254	22,4244	3,009	271,4	cooling	15,254	36,5713	18,1501	257,5		15,254	33,0234	11,1851	305,27	
15,256	22,0261	2,1316	278,09	cooling	15,256	36,8546	16,549	283,84		15,256	32,9629	12,4484	286,76	
15,258	22,554	4,2722	255,55	cooling	15,258	36,1659	20,5261	218,62		15,258	33,5703	15,3582	254,58	
15,26	22,3276	-0,2046	314,97		15,26	35,6439	18,3558	241,66	heating	15,26	33,2216	16,0251	240,38	
15,262	22,3281	-3,2033	356,89		15,262	36,8861	19,8524	238,11	heating	15,262	33,2291	14,8608	256,76	

15,264	22,405	-3,2336	358,39	cooling	15,264	35,8116	17,516	255,75	heating	15,264	33,1059	13,8224	269,55	
15,266	21,7869	-4,8506	372,35	cooling	15,266	35,6906	19,0622	232,44	heating	15,266	33,2818	9,4917	332,55	
15,268	22,7074	0,1566	315,23	cooling	15,268	35,2733	16,3703	264,24	heating	15,268	33,4924	9,1125	340,79	
15,27	22,1586	5,3106	235,51	cooling	15,27	35,9867	17,285	261,42	heating	15,27	33,4527	12,6652	290,58	
15,272	22,7853	6,3113	230,28	cooling	15,272	34,6068	15,0813	272,94	heating	15,272	33,3143	13,9365	270,87	
15,274	22,7262	6,5258	226,46		15,274	35,9455	14,8929	294,28	heating	15,274	33,2262	9,4409	332,48	
15,276	22,5082	1,1653	298,34		15,276	35,399	14,5002	292,13	heating	15,276	33,664	9,5502	337,07	
15,278	22,3896	-4,539	376,42		15,278	35,4813	12,3228	323,72	heating	15,278	33,4338	12,5461	291,98	
15,28	22,9368	0,5168	313,4		15,28	36,053	17,3462	261,49	heating	15,28	33,7794	16,6985	238,77	
15,282	22,6227	1,5546	294,5		15,282	37,2264	20,0592	239,97	heating	15,282	33,1991	15,2947	250,28	
15,284	22,6385	5,0365	246,05		15,284	36,2541	18,4928	248,28	heating	15,284	33,6515	12,7813	291,73	
15,286	22,7176	7,667	210,38		15,286	36,2774	14,5131	304,23	heating	15,286	33,1426	8,06	350,62	
15,288	22,124	1,2696	291,51		15,288	37,6017	18,4454	267,78	heating	15,288	33,6733	6,3862	381,43	
15,29	23,2633	-1,3525	344,09		15,29	37,1185	14,9523	309,85	heating	15,29	32,8941	8,3336	343,32	
15,292	22,6561	-0,688	326,32		15,292	36,6232	20,0003	232,36	heating	15,292	33,307	13,843	272,08	
15,294	23,0508	-1,3275	340,77		15,294	35,6613	16,1147	273,23	heating	15,294	32,9547	16,9083	224,31	
15,296	23,0885	1,3665	303,64		15,296	37,0956	16,2863	290,88	heating	15,296	33,5645	17,1655	229,23	
15,298	23,0527	3,0513	279,59		15,298	35,9171	17,3674	259,3	heating	15,298	33,246	14,3525	264,1	
15,3	22,9971	2,5422	285,93		15,3	35,7398	18,0275	247,59	heating	15,3	33,7454	11,5398	310,4	
15,302	23,8062	6,6693	239,55		15,302	35,6825	14,635	294,21	heating	15,302	33,566	10,7326	319,18	
15,304	23,5018	2,3041	296,31		15,304	36,3963	16,0782	284,02	heating	15,304	33,5527	9,1515	341,09	
15,306	23,6141	-0,2584	333,7		15,306	34,8155	12,2907	314,86	heating	15,306	33,5407	9,4136	337,26	
15,308	23,7131	-0,6936	341,17		15,308	36,5874	16,5514	280,07	heating	15,308	33,7174	12,0391	303,03	
15,31	23,0133	-4,5056	384,67		15,31	35,7075	20,2298	216,36	heating	15,31	33,3186	12,5239	290,68	
15,312	23,874	-0,1038	335,17		15,312	35,5631	13,6622	306,14	heating	15,312	33,2907	11,9919	297,73	
15,314	23,0067	3,4923	272,78		15,314	36,5182	19,165	242,57	heating	15,314	33,7291	13,3996	284,18	
15,316	23,4475	2,0475	299,14		15,316	36,6789	18,4202	255,23	heating	15,316	33,2348	9,2498	335,28	
15,318	23,4223	2,531	292,03		15,318	35,9796	14,635	298,37	heating	15,318	33,6619	6,862	374,62	
15,32	23,53	0,5738	320,89		15,32	36,0578	13,6761	312,86	heating	15,32	33,588	9,5964	335,37	
15,322	23,3306	-7,287	427,99		15,322	36,8618	14,9827	305,84	heating	15,322	33,4002	9,3228	336,57	
15,324	24,4905	-3,4672	390,81	cooling	15,324	36,8072	15,3539	299,88	heating	15,324	33,2294	11,5713	302,75	
15,326	23,8274	-1,4495	353,33	cooling	15,326	36,3705	22,0341	200,4	heating	15,326	33,6048	13,7855	277,05	
15,328	23,5894	2,3732	296,57	cooling	15,328	35,4359	14,1106	298,1	heating	15,328	32,9369	9,3925	329,12	
15,33	23,6115	7,3012	227,99	cooling	15,33	37,6579	16,4311	296,72	heating	15,33	33,7332	9,0263	345,37	
15,332	23,1319	5,9498	240,18	cooling	15,332	35,5916	16,4933	266,97	heating	15,332	33,4595	13,2457	282,56	
15,334	23,9821	2,6743	297,85		15,334	36,6705	17,0935	273,66	heating	15,334	33,6506	10,4529	324,27	
15,336	23,7089	2,3047	299,2		15,336	36,368	19,7971	231,64	heating	15,336	33,544	10,4895	322,27	
15,338	24,3048	-1,0502	354,43		15,338	35,9349	16,1634	276,38	heating	15,338	33,4085	10,3161	322,8	
15,34	24,3206	-1,9049	366,59	cooling	15,34	35,5923	14,3263	297,27	heating	15,34	33,5414	10,6544	319,93	
15,342	24,5654	-0,7573	353,97	cooling	15,342	36,7957	19,0924	247,47	heating	15,342	33,5895	11,5786	307,68	
15,344	23,6705	-3,8852	385,19	cooling	15,344	35,5159	16,0443	272,18	heating	15,344	33,5249	12,447	294,64	
15,346	24,6687	0,1258	343,07	cooling	15,346	35,2981	10,7443	343,23	heating	15,346	33,437	7,7091	359,64	
15,348	24,0047	2,3967	302,05	cooling	15,348	36,2288	15,3632	291,67	heating	15,348	33,8597	8,0458	360,84	cooling
15,35	24,0973	4,1975	278,17	cooling	15,35	36,1381	10,9675	351,85	heating	15,35	33,2023	7,6196	357,61	cooling
15,352	23,9646	5,5967	256,76	cooling	15,352	36,6171	14,6356	307,27	heating	15,352	33,6224	10,4378	324,09	cooling
15,354	23,999	4,2118	276,59	cooling	15,354	35,3449	11,0357	339,81	heating	15,354	33,3516	12,7258	288,32	cooling
15,356	23,9319	-0,9716	348,12	cooling	15,356	37,332	13,256	336,55	heating	15,356	33,6657	14,726	264,75	cooling
15,358	23,7762	0,9891	318,53	cooling	15,358	36,4707	14,7579	303,51	heating	15,358	33,2155	12,2828	292,61	cooling
15,36	23,7786	2,791	293,37	cooling	15,36	35,5435	17,9385	246,09	heating	15,36	33,4058	10,9815	313,46	cooling
15,362	23,5502	4,1801	270,77	cooling	15,362	35,0135	12,1451	319,67	heating	15,362	33,1212	10,3476	318,34	cooling
15,364	23,7189	4,0338	275,17	cooling	15,364	36,9271	16,5594	284,71	heating	15,364	32,9909	10,4831	314,63	cooling
15,366	23,3642	0,7826	315,66	cooling	15,366	35,1536	12,9542	310,31	heating	15,366	32,8771	12,9801	278,13	cooling
15,368	24,2784	1,6011	317	cooling	15,368	36,2827	17,2077	266,64	heating	15,368	32,5109	13,0104	272,59	cooling
15,37	23,6806	1,8132	305,67	cooling	15,37	35,9915	18,7215	241,41	heating	15,37	32,4071	12,2903	281,2	
15,372	23,7092	2,4999	296,47	cooling	15,372	36,064	12,9434	323,19	heating	15,372	32,2377	10,8489	298,98	
15,374	23,4541	0,8916	315,39	cooling	15,374	36,4335	15,0828	298,45	heating	15,374	32,6105	12,0873	286,88	

15,376	23,7326	-0,5917	340,02	cooling	15,376	37,1339	20,0462	238,86	heating	15,376	32,3654	10,2416	309,26	
15,378	23,4911	-1,0573	343,15	cooling	15,378	36,3818	20,0757	227,94	heating	15,378	33,0822	13,2883	276,69	
15,38	23,9876	3,7235	283,26	cooling	15,38	35,8707	16,6074	269,27	heating	15,38	32,6587	12,0725	287,76	
15,382	23,681	1,8316	305,42	cooling	15,382	36,7981	18,8557	250,81	heating	15,382	33,0211	11,5771	299,76	
15,384	23,6062	0,4937	323,08	cooling	15,384	37,1301	13,5853	329,12	heating	15,384	32,8973	11,591	297,83	
15,386	23,4519	-0,2152	330,83	cooling	15,386	37,041	19,1182	250,53	heating	15,386	32,9458	10,7095	310,83	
15,388	23,5894	0,0096	329,61	cooling	15,388	35,6627	14,8353	291,14	heating	15,388	32,9533	8,3125	344,44	
15,39	23,9741	2,4396	301,02	cooling	15,39	37,6192	18,9424	261,07	heating	15,39	33,251	11,8892	298,61	
15,392	23,5524	3,0364	286,78	cooling	15,392	36,2653	19,6463	232,31	heating	15,392	32,9592	14,6309	256,2	
15,394	23,9149	3,0433	291,75	cooling	15,394	35,6628	19,2507	229,42	heating	15,394	33,1421	15,2302	250,38	
15,396	23,9023	4,1492	276,12	cooling	15,396	35,297	13,3666	306,55	heating	15,396	33,187	16,8648	228,16	
15,398	23,7555	2,4398	297,96	cooling	15,398	35,9418	13,1619	318,43	heating	15,398	32,8775	14,3968	258,33	
15,4	23,9212	0,8702	322,22		15,4	34,3379	10,986	326,42	heating	15,4	32,8739	13,8572	265,83	
15,402	23,726	-0,7223	341,75	cooling	15,402	35,7831	16,534	269,07	heating	15,402	32,9175	13,567	270,49	
15,404	23,9032	-0,896	346,66	cooling	15,404	35,5712	18,337	240,91	heating	15,404	32,4923	12,6959	276,72	
15,406	23,7988	4,3421	271,97	cooling	15,406	36,0333	13,0752	320,92	heating	15,406	32,7641	10,4735	311,59	
15,408	23,3126	5,0293	255,57	cooling	15,408	37,0131	19,1723	249,39	heating	15,408	32,809	12,7894	279,84	
15,41	23,4287	3,7011	275,76		15,41	37,4325	18,5989	263,27	heating	15,41	32,6741	12,6019	280,58	
15,412	23,9457	5,6724	255,43		15,412	36,9471	19,6628	241,61	heating	15,412	32,6132	14,1895	257,54	
15,414	23,4612	3,0152	285,8		15,414	35,6561	15,058	287,93	heating	15,414	32,5719	12,3938	282,06	
15,416	24,0472	1,0591	321,34		15,416	36,2112	14,9032	297,85	heating	15,416	32,6721	10,235	313,64	
15,418	23,932	0,0909	333,26		15,418	35,8469	12,6189	324,69	heating	15,418	33,003	11,4597	301,14	
15,42	23,6679	-6,4137	420,5		15,42	35,2093	19,8322	214,95	heating	15,42	32,8689	12,6366	282,82	
15,422	24,2962	-5,302	413,74	cooling	15,422	34,01	14,9415	266,55	heating	15,422	32,8902	11,618	297,35	
15,424	24,167	0,7185	327,77	cooling	15,424	35,9962	16,0721	278,51	heating	15,424	32,9596	11,8192	295,51	
15,426	23,8529	1,5064	312,37	cooling	15,426	34,6972	12,1078	315,77	heating	15,426	32,7683	12,0443	289,69	
15,428	24,0694	5,5877	258,35	cooling	15,428	35,5169	14,5557	293,01	heating	15,428	33,4013	12,6141	290,57	
15,43	23,5413	3,2412	283,76	cooling	15,43	35,4588	16,6439	263	heating	15,43	32,9583	14,3471	260,16	
15,432	23,5832	-2,5635	365,49	cooling	15,432	35,9112	14,347	301,44	heating	15,432	33,14	11,5582	301,68	
15,434	24,3457	1,4919	319,46	cooling	15,434	35,5295	13,8384	303,21	heating	15,434	32,948	11,0907	305,53	
15,436	23,4009	0,2349	323,83	cooling	15,436	37,2897	17,9092	270,91	heating	15,436	33,7696	13,4225	284,42	
15,438	23,9247	-0,2015	337,25	cooling	15,438	37,2626	20,0694	240,33	heating	15,438	32,7937	11,3687	299,49	
15,44	23,6582	5,0776	259,73	cooling	15,44	37,312	15,5044	304,84	heating	15,44	33,5988	11,6059	307,43	
15,442	23,1847	4,1481	266,1	cooling	15,442	38,18	19,3012	263,9	heating	15,442	33,1649	13,3136	277,49	
15,444	23,1897	4,1361	266,34	cooling	15,444	38,405	15,2214	324,07	heating	15,444	33,2245	11,1776	308,18	
15,446	23,3501	7,5753	220,51	cooling	15,446	37,798	19,1863	260,16	heating	15,446	33,2003	11,5256	302,98	
15,448	22,7833	-0,0677	319,42		15,448	36,4718	15,0392	299,6	heating	15,448	33,408	12,3043	295	
15,45	23,4499	-0,3016	332,01		15,45	37,8074	15,9588	305,41	heating	15,45	33,9085	10,2541	330,65	
15,452	22,9245	-0,2153	323,46		15,452	37,1771	13,544	330,36	heating	15,452	33,3604	14,5886	262,4	
15,454	22,9023	-1,6321	342,95		15,454	36,9879	20,0974	236,1	heating	15,454	33,1869	14,9422	255,03	
15,456	23,7165	5,467	255,1	cooling	15,456	36,427	15,2194	296,45	heating	15,456	32,6305	9,6516	321,21	
15,458	23,0844	7,0521	224,11		15,458	38,5325	16,7415	304,61	heating	15,458	33,5663	11,8336	303,79	
15,46	23,4223	1,1929	310,73		15,46	36,7778	13,5351	324,9	heating	15,46	32,1426	11,3244	291,01	
15,462	23,4932	2,3682	295,3		15,462	37,4134	15,3139	308,92	heating	15,462	33,7697	13,9107	277,6	
15,464	23,0437	-1,3074	340,39		15,464	36,972	19,1666	248,89	heating	15,464	32,7989	17,124	219,11	
15,466	23,2651	-2,5636	361,05		15,466	36,5954	16,7034	278,06	heating	15,466	32,9097	12,82	280,82	heating
15,468	24,0113	3,1854	291,12		15,468	36,2202	17,9492	255,4	heating	15,468	32,9333	10,1309	318,74	heating
15,47	23,0689	-0,0461	323,11		15,47	37,4313	20,5501	235,97	heating	15,47	34,0927	14,2704	277,09	heating
15,472	24,1358	2,4264	303,46		15,472	36,5784	19,4985	238,75	heating	15,472	33,3109	12,3075	293,6	heating
15,474	23,5077	6,8076	233,44		15,474	36,6327	13,7576	319,76	heating	15,474	33,5359	12,8353	289,36	heating
15,476	22,9774	-0,611	329,73		15,476	38,053	18,5833	272,16	heating	15,476	33,1236	11,306	304,98	heating
15,478	23,5769	-3,2195	374,57		15,478	38,1955	11,1452	378,12	heating	15,478	33,0856	8,1703	348,28	heating
15,48	23,1031	-2,1978	353,67		15,48	38,7748	20,6304	253,63	heating	15,48	33,3178	13,1485	281,94	
15,482	22,951	-6,1813	407,23	cooling	15,482	37,5182	18,2941	268,72	heating	15,482	32,6966	13,9597	261,91	
15,484	22,6196	0,7505	305,7	cooling	15,484	39,4606	18,1449	297,96	heating	15,484	33,8697	13,4092	286,01	
15,486	21,9451	3,7361	254,54	cooling	15,486	38,7073	17,1648	301,13	heating	15,486	33,1577	12,4595	289,33	

15,488	21,2375	-0,298	301,03	cooling	15,488	38,1981	20,0366	253,87	heating	15,488	33,4333	10,0231	327,24	
15,49	22,1901	4,3303	249,65	cooling	15,49	37,8797	15,7685	309,08	heating	15,49	33,2842	8,9834	339,69	
15,492	20,884	2,226	260,81		15,492	38,947	21,4516	244,56	heating	15,492	34,0784	13,8792	282,35	
15,494	21,9734	-1,492	328,01		15,494	36,6761	16,9365	275,93	heating	15,494	33,0792	13,1678	278,33	
15,496	21,5798	-0,6967	311,39		15,496	38,2646	19,6309	260,47	heating	15,496	34,1802	16,4544	247,78	
15,498	21,448	-3,064	342,64		15,498	37,5751	22,9383	204,6	heating	15,498	33,3462	15,3127	252,08	
15,5	21,6031	-1,8798	328,26		15,5	37,4379	15,7148	303,66	heating	15,5	33,498	13,1357	284,63	heating
15,502	22,3761	7,5198	207,67	cooling	15,502	38,3419	19,6101	261,84	heating	15,502	33,1378	14,0975	266,16	heating
15,504	21,5124	3,5474	251,12		15,504	38,5127	15,8273	317,11	heating	15,504	33,3322	14,9978	256,29	heating
15,506	22,2316	2,1179	281,16		15,506	38,3281	17,1192	296,47	heating	15,506	33,4844	14,6228	263,66	heating
15,508	21,9441	0,9167	293,93		15,508	37,7878	18,9052	263,95	heating	15,508	32,9746	12,9271	280,23	heating
15,51	21,5602	-4,0251	357,64		15,51	38,5778	22,8724	219,54	heating	15,51	33,455	11,249	310,41	heating
15,512	22,3316	-0,72	322,23		15,512	38,3839	16,4147	307,1	heating	15,512	33,5643	11,9259	302,47	heating
15,514	21,678	1,9343	275,99		15,514	37,6595	21,3435	228,07	heating	15,514	33,7642	14,8897	263,84	heating
15,516	22,1345	-1,8187	334,83		15,516	36,2173	11,8231	340,99	heating	15,516	33,3484	11,3287	307,8	heating
15,518	22,0375	1,3197	289,6		15,518	37,9571	16,2786	303,03	heating	15,518	34,5528	11,1981	326,46	heating
15,52	21,6275	1,4412	282,17		15,52	35,9369	13,6616	311,38	heating	15,52	33,7212	5,9661	387,98	heating
15,522	21,4562	-2,5904	336,14		15,522	36,6659	11,7852	347,8	heating	15,522	34,3213	11,9511	312,7	
15,524	22,387	0,1462	310,89		15,524	37,4094	13,8542	329,27	heating	15,524	33,5817	15,2857	255,75	
15,526	21,6653	-2,2229	333,92		15,526	38,13	13,6731	341,87	heating	15,526	33,9129	15,4172	258,54	
15,528	22,2848	0,2615	307,85		15,528	38,2869	13,7916	342,41	heating	15,528	33,7601	14,5477	268,56	
15,53	21,958	3,7469	254,56		15,53	39,9211	18,5546	298,67	heating	15,53	33,6608	12,4968	295,84	heating
15,532	21,4919	-1,1358	316,3		15,532	38,484	16,2398	310,94	heating	15,532	34,272	8,3754	362	heating
15,534	22,0795	-3,2598	354,21		15,534	38,0913	10,2179	389,63		15,534	34,2126	11,3705	319,3	
15,536	21,9792	-0,4639	313,72		15,536	37,6835	14,0637	330,17		15,536	33,6099	10,3071	325,74	
15,538	22,0398	-1,8185	333,5		15,538	38,0067	9,13	403,65		15,538	34,0913	9,7821	339,81	
15,54	22,0259	0,0169	307,65		15,54	37,3456	17,1427	282,41		15,54	34,7413	17,0975	246,63	
15,542	21,6818	-1,3348	321,74		15,542	36,4793	15,0558	299,47		15,542	33,7335	15,455	255,51	
15,544	21,5996	-4,6969	367,59		15,544	38,3312	17,7997	287		15,544	34,2345	15,3951	263,35	
15,546	22,4758	2,3151	281,82	cooling	15,546	38,5558	15,7948	318,16		15,546	33,9468	12,4973	299,83	
15,548	21,4072	2,834	259,63	cooling	15,548	38,1771	16,6758	300,56		15,548	33,7602	8,7484	349,63	
15,55	21,5542	1,3723	282,11		15,55	38,1493	13,3933	346,05		15,55	34,0684	10,2501	332,94	
15,552	21,2133	2,0309	268,14		15,552	39,7262	21,3033	257,52		15,552	34,0773	13,9908	280,78	
15,554	20,4649	-1,2186	303,1		15,554	37,491	16,0409	299,84		15,554	33,8728	9,5629	339,82	
15,556	20,6021	-1,0602	302,81		15,556	38,6209	18,8611	276,21		15,556	34,1232	12,8716	297,07	
15,558	20,2728	1,2754	265,55		15,558	37,4901	16,5529	292,67		15,558	33,4962	12,0175	300,24	
15,56	20,1604	-2,554	317,51		15,56	37,9846	12,2828	359,27		15,56	34,1365	12,2128	306,46	
15,562	20,4038	0,7278	275,04		15,562	37,0168	15,1563	305,58		15,562	33,8813	12,8452	294,05	
15,564	20,2254	3,1753	238,33		15,564	38,1279	17,278	291,45		15,564	33,8094	11,0608	317,99	
15,566	20,1171	-0,8003	292,4		15,566	37,4216	17,3603	280,43		15,566	34,2188	13,9126	283,85	
15,568	21,1803	2,6355	259,23		15,568	38,2503	16,7091	301,11		15,568	33,9383	17,5308	229,35	
15,57	20,4058	1,3365	266,56		15,57	38,3272	17,5301	290,71		15,57	33,4013	13,0586	284,36	
15,572	21,1914	-0,4497	302,51		15,572	39,7677	14,891	347,74		15,572	34,0756	10,8612	324,5	
15,574	21,2346	0,7747	286		15,574	39,1268	21,4321	247,35		15,574	34,119	13,039	294,67	
15,576	20,8631	-3,3267	338,14		15,576	38,0016	16,1093	306,02		15,576	33,993	11,604	312,96	
15,578	21,1827	-5,6901	375,64		15,578	39,4503	19,2668	282,14		15,578	34,1358	14,952	268,16	
15,58	21,5379	-1,1054	316,52		15,58	38,7387	15,9359	318,75		15,58	33,7885	14,234	273,34	
15,582	21,1367	-3,993	351,28		15,582	37,6533	18,8274	263,16		15,582	33,9126	14,4036	272,71	
15,584	21,6286	-0,6763	311,79		15,584	36,8902	15,3095	301,67		15,584	33,7668	15,6861	252,74	
15,586	21,3105	0,3887	292,46		15,586	38,0568	18,652	271,25		15,586	33,8092	16,1629	246,67	
15,588	21,116	-3,3259	341,66		15,588	36,4229	11,2102	352,44		15,588	33,768	12,3246	299,75	heating
15,59	21,9275	-1,0093	320,62		15,59	37,2222	14,5358	317,12		15,59	34,0852	12,5244	301,39	heating
15,592	20,9729	-3,4315	341,14		15,592	36,7108	16,5805	281,39		15,592	33,7915	11,0598	317,75	
15,594	21,7645	-4,0875	361,37		15,594	37,3228	17,4887	277,25		15,594	34,1167	12,888	296,74	
15,596	21,8343	3,2277	260,09	cooling	15,596	36,3112	16,5664	276		15,596	33,672	12,8468	291,11	
15,598	21,5063	3,5338	251,23		15,598	37,5288	16,3818	295,6		15,598	33,8259	12,3561	300,12	

15,6	21,7981	3,3809	257,45	
15,602	22,2155	5,9967	226,71	
15,604	21,6685	-0,2089	305,81	
15,606	22,3486	1,246	294,98	
15,608	21,6849	1,2388	285,81	
15,61	21,4063	-5,2835	373,08	
15,612	21,826	-4,7331	371,26	
15,614	21,3365	-2,112	327,78	
15,616	21,7573	-0,4693	310,7	
15,618	21,9757	5,7962	226,16	
15,62	21,1923	-0,1699	298,61	
15,622	21,7398	-4,9304	372,81	
15,624	21,9804	-1,4017	326,85	
15,626	21,4226	-2,7438	337,81	
15,628	21,8168	-0,6645	314,26	
15,63	21,5654	1,0265	287,1	
15,632	21,4384	-0,521	306,96	
15,634	21,8745	4,1377	247,94	
15,636	21,1115	1,3102	276,79	
15,638	21,4649	-5,388	375,36	
15,64	21,4529	-3,3891	347,25	
15,642	20,7304	-2,9736	331,35	
15,644	20,8523	0,2043	288,63	cooling
15,646	20,8923	5,4726	215,54	
15,648	20,1613	1,9043	255,21	
15,65	20,7423	2,3998	256,4	
15,652	20,4451	2,4243	251,9	
15,654	20,4377	-2,6156	322,25	
15,656	20,8613	-1,5457	313,22	
15,658	20,6078	-1,283	306	
15,66	21,075	-1,1378	310,5	
15,662	21,0677	4,1712	236,19	
15,664	20,4428	2,9669	244,29	
15,666	20,7282	0,3883	284,32	
15,668	20,9227	-0,3009	296,67	
15,67	20,0998	-6,2138	367,83	
15,672	20,5702	-4,137	345,37	
15,674	20,0999	1,552	259,27	
15,676	19,913	2,8989	237,83	
15,678	20,1609	6,4085	192,24	
15,68	20,1901	6,1935	195,65	
15,682	20,2931	-0,6016	292,08	heating
15,684	20,4897	-0,0061	286,5	heating
15,686	20,2315	-1,5128	303,95	heating
15,688	20,8748	-3,245	337,16	heating
15,69	21,5081	-0,6867	310,25	
15,692	20,8414	-2,8528	331,21	
15,694	21,4419	-2,4604	334,12	
15,696	21,1307	0,3637	290,29	
15,698	20,9342	-1,8416	318,37	
15,7	21,4272	-0,8105	310,85	
15,702	21,3027	1,3105	279,46	
15,704	21,0089	-1,9914	321,51	
15,706	21,1695	0,3105	291,58	
15,708	20,6816	-1,8019	314,29	
15,71	20,982	-2,729	331,44	

15,6	37,0197	15,9439	294,61	
15,602	37,0263	12,6356	340,95	
15,604	37,2431	16,195	294,22	
15,606	38,1732	15,3848	318,55	
15,608	37,4249	19,235	254,27	
15,61	36,9649	12,1837	346,4	
15,612	38,8545	15,5817	325,32	
15,614	37,8239	12,2236	357,85	
15,616	37,9901	18,6892	269,8	
15,618	37,2304	18,4327	262,76	
15,62	38,5543	19,9321	260,31	
15,622	37,0249	15,312	303,51	
15,624	38,2438	20,1667	252,69	
15,626	37,1465	18,186	265,04	
15,628	37,9943	17,4967	286,53	
15,63	36,972	18,5935	256,9	
15,632	38,2678	16,0272	310,89	
15,634	37,6092	17,239	284,75	
15,636	37,4367	15,0245	313,29	
15,638	37,8756	16,6371	296,88	
15,64	39,1037	17,0571	308,18	
15,642	38,1535	18,1113	280,16	
15,644	37,8636	12,1468	359,48	
15,646	40,042	21,3083	261,87	
15,648	38,7384	16,683	308,3	
15,65	38,7016	22,1843	230,89	
15,652	37,3943	20,7233	233,04	
15,654	38,6766	19,8717	262,86	
15,656	37,0238	15,5298	300,45	heating
15,658	37,9564	20,6733	241,59	heating
15,66	37,2881	16,5438	289,97	heating
15,662	38,9615	19,9139	266,26	heating
15,664	37,5559	18,1146	271,76	heating
15,666	39,7907	16,4455	326,33	heating
15,668	39,3778	21,5058	249,82	heating
15,67	38,792	17,8914	292,16	heating
15,672	39,5232	19,9248	273,96	heating
15,674	40,2391	20,4203	277,04	heating
15,676	38,8408	20,4272	257,39	heating
15,678	38,3804	15,9208	313,95	heating
15,68	40,3708	22,5817	248,67	heating
15,682	39,1752	14,4646	345,42	heating
15,684	40,5121	23,6738	235,37	heating
15,686	39,6153	22,5446	238,62	heating
15,688	41,5951	21,169	285,53	heating
15,69	39,8121	16,8447	321,05	heating
15,692	39,8449	17,9071	306,66	heating
15,694	38,7753	14,4261	340,37	heating
15,696	39,1362	20,0036	267,45	heating
15,698	36,9762	19,0501	250,58	heating
15,7	38,6746	18,0803	287,88	heating
15,702	38,0243	22,7475	213,55	heating
15,704	37,4265	15,4867	306,68	heating
15,706	39,147	18,1616	293,34	heating
15,708	40,0302	19,5825	285,83	heating
15,71	39,2244	21,8408	243	heating

15,6	33,9409	12,817	295,28	
15,602	33,969	14,1391	277,19	
15,604	33,7687	14,1015	274,92	
15,606	33,8216	11,8936	306,52	
15,608	33,8181	9,9627	333,46	
15,61	34,1607	10,5805	329,62	
15,612	34,2995	15,2265	266,61	
15,614	34,0357	14,4494	273,79	
15,616	34,1608	14,1927	279,12	
15,618	34,0547	11,6933	312,58	
15,62	34,3495	12,5373	304,9	
15,622	34,0472	13,8625	282,15	
15,624	33,6418	11,2977	312,34	
15,626	33,6378	9,8447	332,59	
15,628	33,7465	11,2045	315,1	
15,63	33,3966	8,9487	341,74	
15,632	34,2368	10,6249	330,06	
15,634	33,7516	10,6941	322,31	
15,636	34,1441	10,1689	335,14	
15,638	34,0627	13,374	289,2	
15,64	33,8547	11,1169	317,84	
15,642	33,9352	9,7129	338,59	
15,644	34,0851	12,4598	302,29	
15,646	33,6234	10,3386	325,49	
15,648	34,1227	11,4654	316,72	
15,65	33,9199	12,5048	299,35	
15,652	33,8654	7,9083	362,84	
15,654	34,9915	12,3192	316,93	
15,656	34,2859	14,219	280,51	
15,658	34,9167	16,0653	263,51	
15,66	34,747	19,5033	213,08	
15,662	34,3577	15,2218	267,49	
15,664	34,3095	11,341	321,07	
15,666	34,385	13,8141	287,55	
15,668	33,7027	11,966	303,85	
15,67	34,3813	13,9775	285,21	
15,672	34,0589	15,5005	259,42	
15,674	34,1051	10,8676	324,82	
15,676	34,42	12,333	308,74	
15,678	33,9993	14,3757	274,31	
15,68	34,0192	14,9906	265,99	
15,682	34,1472	16,6436	244,67	
15,684	33,9969	16,3239	247,04	
15,686	33,7115	9,9611	331,99	
15,688	34,2139	9,5368	344,95	
15,69	33,6774	10,1922	328,29	
15,692	34,1617	14,7639	271,15	
15,694	33,8588	17,6582	226,46	
15,696	34,1828	17,7724	229,39	
15,698	33,883	13,7904	280,86	
15,7	34,0689	9,4018	344,81	heating
15,702	33,9721	7,2057	374,15	
15,704	34,0373	8,3294	359,36	
15,706	33,7986	10,2122	329,7	
15,708	33,9603	12,8496	295,1	
15,71	34,0188	14,7864	268,84	

15,712	21,5914	3,9874	246,08		15,712	39,4045	18,9566	285,83	heating	15,712	33,8913	12,3368	301,3	
15,714	20,686	2,8377	249,49		15,714	41,1711	23,8622	241,95	heating	15,714	34,1193	10,1549	334,99	
15,716	21,2804	0,9631	284,01		15,716	39,6155	15,6427	335,1	heating	15,716	33,8284	5,8524	391,06	
15,718	20,8984	-0,0059	292,21		15,718	40,0625	24,0756	223,47	heating	15,718	34,1504	6,3013	389,29	
15,72	20,6837	-4,3984	350,61		15,72	38,6493	23,1286	216,96	heating	15,72	34,175	10,5418	330,36	
15,722	21,0614	-2,662	331,62		15,722	40,0433	22,1612	249,97	heating	15,722	33,9785	10,74	324,84	
15,724	20,8979	2,1121	262,6		15,724	38,5441	20,0315	258,78	heating	15,724	33,8758	8,7544	351,16	
15,726	20,7739	1,1977	273,65		15,726	39,5126	20,4769	266,09	heating	15,726	33,8598	9,0045	347,44	cooling
15,728	21,1328	5,8889	213,09		15,728	38,8231	18,0296	290,66	heating	15,728	33,3868	5,6141	388,22	cooling
15,73	20,9146	5,6691	213,11		15,73	39,5085	20,3439	267,89	heating	15,73	33,8743	8,5478	354,03	cooling
15,732	20,998	2,463	259,09	heating	15,732	38,0085	17,6305	284,85	heating	15,732	33,4234	12,9917	285,61	cooling
15,734	21,7064	5,1674	231,19	heating	15,734	40,0921	18,5306	301,4	heating	15,734	33,5244	12,6947	291,17	cooling
15,736	20,7148	2,0992	260,22	heating	15,736	39,1843	22,983	226,47	heating	15,736	33,6203	14,3192	269,8	cooling
15,738	21,5129	0,2114	297,76	heating	15,738	38,8464	15,9291	320,35	heating	15,738	33,2896	12,8283	286,02	cooling
15,74	20,9077	0,3173	287,82	heating	15,74	40,696	20,2991	285,12	heating	15,74	33,0258	7,809	352,49	cooling
15,742	20,4168	-3,4562	333,71	heating	15,742	41,4383	18,911	314,9	heating	15,742	33,5171	8,1661	354,37	cooling
15,744	20,5198	-0,9587	300,24	heating	15,744	40,7929	21,6721	267,28	heating	15,744	32,801	8,5834	338,53	cooling
15,746	20,5121	3,7321	234,56	heating	15,746	40,5773	20,1225	285,93	heating	15,746	33,1735	10,5118	316,78	cooling
15,748	20,594	-0,8939	300,37	heating	15,748	41,9679	23,1812	262,61	heating	15,748	33,228	17,8301	215,24	cooling
15,75	21,3579	-1,9106	325,26	heating	15,75	40,3045	15,6671	344,39	heating	15,75	32,7347	15,7456	237,48	cooling
15,752	21,3953	-6,4633	389,42	heating	15,752	40,5678	21,4714	266,94	heating	15,752	33,0583	12,5399	286,82	
15,754	21,7227	-9,1832	432,02		15,754	39,0878	16,1095	321,2	heating	15,754	33,1227	11,9371	296,14	
15,756	22,5402	-0,704	324,92		15,756	40,9604	18,0563	320,17	heating	15,756	32,9221	8,838	336,66	
15,758	21,8665	3,6853	254,15		15,758	39,2719	18,1919	294,67	heating	15,758	33,1473	11,3392	304,85	
15,76	22,4865	3,053	271,65		15,76	40,0054	20,8491	267,78	heating	15,76	33,0964	14,8438	255,15	
15,762	22,398	3,6335	262,3		15,762	39,083	18,0374	294,19	heating	15,762	32,8188	11,4972	298,04	
15,764	22,1845	-0,0581	310,92		15,764	39,4885	16,8983	315,78	heating	15,764	33,548	12,9762	287,56	
15,766	22,1465	-2,272	341,33		15,766	38,7749	13,8215	348,81	heating	15,766	32,8736	13,1192	276,14	
15,768	22,4891	0,9425	301,19		15,768	40,1849	13,62	371,34	heating	15,768	33,1339	9,9043	324,72	
15,77	21,9274	-1,8747	332,72		15,77	39,1028	21,3891	247,61		15,77	33,2706	12,8103	286,01	
15,772	22,3569	-2,2791	344,37		15,772	38,5184	19,6895	263,2	heating	15,772	33,0052	13,3146	275,25	
15,774	22,0946	-0,304	313,1		15,774	39,8894	20,3956	272,49	heating	15,774	32,9223	12,6265	283,71	
15,776	21,8322	1,0816	290,06		15,776	40,4258	13,6283	374,59	heating	15,776	33,2538	15,3484	250,29	
15,778	22,1037	3,7331	256,79		15,778	39,9214	15,8412	336,61		15,778	32,7758	14,6681	253,12	
15,78	21,8398	4,8377	237,67		15,78	39,6427	12,4171	380,57		15,78	32,8493	11,7653	294,72	
15,782	22,3116	3,1025	268,52		15,782	41,4585	19,8496	302,06		15,782	32,8816	13,7723	267,12	
15,784	22,0777	1,8381	282,92		15,784	39,7973	15,22	343,55		15,784	32,5971	12,7201	277,85	
15,786	22,0731	-1,2131	325,51		15,786	40,1459	19,7202	285,52		15,786	32,6654	12,2321	285,63	
15,788	21,8552	-1,9142	332,26		15,788	39,5868	17,7717	304,94		15,788	32,8145	13,1703	274,6	
15,79	22,4084	2,4594	278,86		15,79	39,8945	16,4377	327,89		15,79	32,6554	12,5559	280,96	
15,792	21,9095	3,3965	258,78		15,792	38,3511	15,666	317,1		15,792	32,8857	9,8685	321,75	
15,794	22,4793	4,7893	247,28		15,794	39,8754	20,6867	268,23		15,794	33,05	11,2332	304,97	
15,796	21,9899	2,0845	278,25		15,796	38,7594	20,5765	254,17		15,796	32,9066	9,9573	320,8	
15,798	22,0667	-1,9465	335,67		15,798	39,2414	18,9876	283,12		15,798	32,9849	9,0855	334,08	
15,8	22,184	-1,4218	329,97		15,8	39,8433	21,8884	250,98		15,8	33,2597	13,2287	280	
15,802	22,2888	2,4029	277,98		15,802	41,7143	17,5201	338,2		15,802	32,8567	14,3165	259,16	
15,804	22,4707	3,6573	262,98		15,804	40,3247	22,4671	249,62		15,804	33,0519	13,295	276,17	
15,806	22,3055	4,2019	253,06		15,806	39,5296	18,218	297,9		15,806	33,0442	12,7556	283,61	
15,808	22,4628	2,0735	285,01		15,808	40,9887	18,3818	316,01		15,808	32,7449	9,6309	323,1	
15,81	22,1643	1,0672	294,91		15,81	41,2234	13,7492	384,05		15,81	33,0332	6,7464	367,45	
15,812	22,4577	1,6755	290,5		15,812	39,6908	19,2018	286,41		15,812	33,1144	9,971	323,51	
15,814	22,0109	1,6849	284,13		15,814	39,6652	15,9307	331,77		15,814	32,7388	8,8144	334,43	
15,816	22,6138	4,0272	259,81		15,816	40,9501	21,5835	270,72		15,816	33,124	11,0575	308,46	
15,818	22,0146	2,2246	276,64	heating	15,818	38,5602	14,5707	335,34		15,818	32,8193	11,1741	302,57	
15,82	22,43	-0,0846	314,72	heating	15,82	39,3002	16,8905	313,25		15,82	32,6742	9,5208	323,65	
15,822	22,3077	-0,9815	325,55	heating	15,822	39,2211	18,9716	283,06		15,822	32,9521	11,5631	298,99	

15,824	22,4673	-0,0166	314,29		15,824	39,3402	17,9965	298,35		15,824	33,1099	16,5845	231	
15,826	22,5816	1,0546	300,92		15,826	38,1615	14,4172	331,91		15,826	32,5413	13,389	267,72	
15,828	22,3914	1,1385	297,08		15,828	39,8963	19,8034	280,87		15,828	33,2197	14,916	255,86	
15,83	22,4045	-4,0521	369,82		15,83	39,1838	20,5551	260,4		15,83	32,7521	13,4571	269,72	
15,832	22,5644	-3,5801	365,46		15,832	39,6476	19,1095	287,09		15,832	32,7872	5,8528	376,5	
15,834	22,7998	1,7158	294,72		15,834	40,5517	22,4269	253,36		15,834	32,8728	3,7664	406,86	
15,836	22,321	3,5745	262,05		15,836	41,1032	15,0431	364,28		15,836	33,0587	7,8379	352,55	
15,838	22,8176	3,8402	265,28		15,838	40,1975	17,2963	320,13		15,838	33,0186	11,4389	301,65	
15,84	22,1205	-0,4161	315,03		15,84	39,1196	15,5877	328,94		15,84	33,0906	15,1318	251,04	
15,842	22,5545	-2,6218	351,93		15,842	40,3892	17,1914	324,27		15,842	32,5327	12,7681	276,28	
15,844	22,5001	-0,2755	318,37		15,844	39,8761	11,595	395,33		15,844	32,9022	11,6205	297,49	
15,846	22,7294	2,4599	283,34		15,846	39,3954	19,7263	274,95		15,846	32,9897	16,5014	230,48	
15,848	22,5894	-0,4028	321,4		15,848	38,8048	16,0325	318,32		15,848	32,4423	15,922	230,93	
15,85	22,892	0,4064	314,32		15,85	40,6746	18,9353	303,88		15,85	32,9568	14,9191	252,14	
15,852	22,9069	1,8776	293,96		15,852	38,8654	11,5611	381,67		15,852	32,6373	12,3757	283,23	
15,854	22,8189	4,8643	250,98		15,854	40,0462	13,2399	374,71		15,854	32,7513	9,1753	329,56	
15,856	22,8478	5,0597	248,65		15,856	39,7561	14,5441	352,43	cooling	15,856	32,486	9,0726	327,28	
15,858	22,3199	1,32	293,55		15,858	40,053	18,4401	302,12	cooling	15,858	32,8077	13,7009	267,08	
15,86	22,9969	1,8469	295,65		15,86	38,4678	16,7451	303,65	cooling	15,86	32,1699	15,8248	228,48	
15,862	22,3898	3,7724	260,24		15,862	39,8166	17,6661	309,63	cooling	15,862	32,4011	15,5016	236,23	
15,864	22,7419	4,6932	252,29		15,864	38,1739	17,2668	292,25	cooling	15,864	31,7708	13,5955	254,06	heating
15,866	22,3691	3,8322	259,12		15,866	37,948	11,3857	371,3	cooling	15,866	32,0953	13,0036	266,87	heating
15,868	22,6365	1,8957	289,93		15,868	39,137	12,9493	366,07	cooling	15,868	31,7283	10,8489	291,86	heating
15,87	22,5691	-0,1953	318,21		15,87	40,1427	10,9116	408,61	cooling	15,87	32,324	12,2918	280,02	heating
15,872	23,0256	3,6436	270,93		15,872	39,2704	16,1228	323,57	cooling	15,872	32,0413	14,45	245,9	heating
15,874	22,8929	3,3158	273,66		15,874	38,7705	15,849	320,41	cooling	15,874	32,0075	12,6702	270,31	heating
15,876	23,3723	4,174	268,36		15,876	40,2843	21,1181	267,91	cooling	15,876	32,114	12,7667	270,45	heating
15,878	23,7654	4,4757	269,64	heating	15,878	38,8625	12,7845	364,53	cooling	15,878	32,0112	11,402	288,09	heating
15,88	23,5814	0,6532	320,5	heating	15,88	38,5295	18,2304	283,75	cooling	15,88	32,3428	9,1135	324,71	heating
15,882	24,4297	-3,2863	387,43	heating	15,882	37,48	16,0482	299,58	cooling	15,882	32,9004	10,949	306,85	heating
15,884	23,9534	-2,0474	363,45		15,884	38,6043	17,7353	291,72	cooling	15,884	32,9856	12,3356	288,66	heating
15,886	24,3844	1,5441	319,27		15,886	36,4805	11,7017	346,37	cooling	15,886	32,6858	11,1812	300,6	heating
15,888	24,0141	5,1051	264,32		15,888	38,1553	14,3446	332,84	cooling	15,888	33,0647	15,9075	239,83	heating
15,89	24,2069	4,8926	269,99		15,89	37,8366	15,426	313,27	cooling	15,89	32,2534	13,7988	257,97	heating
15,892	23,7933	0,3668	327,47		15,892	38,3733	16,783	301,8	cooling	15,892	32,8174	11,6542	295,83	heating
15,894	24,1626	0,989	323,93		15,894	37,7883	16,135	302,68	cooling	15,894	32,5188	9,9344	315,7	heating
15,896	23,6907	1,0393	316,63		15,896	39,7992	14,7039	350,8	cooling	15,896	32,3822	6,7261	358,64	heating
15,898	23,5702	2,1257	299,76		15,898	38,4233	16,0293	313,04	cooling	15,898	32,636	8,163	342,1	heating
15,9	23,2209	1,4026	304,99		15,9	38,2514	14,5373	331,49	cooling	15,9	32,3729	11,832	287,13	
15,902	22,7621	-0,1347	320,06		15,902	39,7011	19,4707	282,79	cooling	15,902	32,4939	11,8482	288,6	
15,904	22,9944	-0,2242	324,56		15,904	40,5608	14,7154	361,28	cooling	15,904	32,9635	12,5799	284,93	
15,906	22,5767	1,5722	293,61		15,906	39,6443	18,8626	290,5	cooling	15,906	32,7542	10,4741	311,44	
15,908	22,8486	0,555	311,63		15,908	39,5228	17,97	301,28	cooling	15,908	32,6161	6,3884	366,62	
15,91	22,6045	0,6031	307,55		15,91	41,4408	22,1803	269,23	cooling	15,91	33,0038	9,025	335,19	
15,912	23,1459	4,0279	267,24		15,912	39,9518	15,1986	346,01	cooling	15,912	32,6083	9,3321	325,37	
15,914	22,4933	1,8398	288,71		15,914	39,9008	19,4861	285,37	cooling	15,914	33,0345	10,9014	309,39	
15,916	23,085	-0,9907	336,54		15,916	39,0044	17,7236	297,47	cooling	15,916	32,8296	11,2786	301,25	
15,918	22,8777	-1,1846	336,35		15,918	39,4714	17,573	306,11	cooling	15,918	32,8825	9,8016	322,64	
15,92	23,0654	2,6002	286,07		15,92	37,2569	11,704	357,19	cooling	15,92	32,7139	8,5369	337,96	
15,922	22,9784	7,2177	220,31		15,922	38,7278	14,3848	340,28	cooling	15,922	32,934	10,5535	312,85	
15,924	22,6381	5,4306	240,54		15,924	37,9287	16,276	302,67	cooling	15,924	32,8585	10,8493	307,66	
15,926	22,7168	-2,9411	358,66		15,926	37,8491	16,6096	296,9	cooling	15,926	33,1562	11,8292	298,12	
15,928	22,8404	-3,9901	375,05		15,928	37,8408	17,7261	281,17	cooling	15,928	32,8984	11,5943	297,8	
15,93	22,9533	-1,5188	342,08		15,93	39,6566	16,6023	322,27	cooling	15,93	32,8604	10,0438	318,94	
15,932	22,6497	1,7595	292,02		15,932	38,1802	19,4813	261,38	cooling	15,932	32,6306	9,7803	319,41	
15,934	22,9582	3,6603	269,76		15,934	37,5081	16,0481	299,98	cooling	15,934	32,6877	10,2301	313,92	

15,936	22,2929	-1,925	338,53		15,936	39,4511	20,9034	259,27	cooling	15,936	32,8106	11,0149	304,67	
15,938	23,1574	-3,6254	374,38		15,938	39,4667	15,5796	333,91	cooling	15,938	33,108	13,0236	280,75	
15,94	23,2462	-1,4483	345,19		15,94	38,295	16,7839	300,69	cooling	15,94	32,7617	13,5541	268,49	
15,942	23,8517	-1,5335	354,85		15,942	38,2928	12,7052	357,68	cooling	15,942	32,9346	13,5653	270,75	
15,944	24,2794	-0,5706	347,37	cooling	15,944	40,0838	16,7484	326,19	cooling	15,944	32,5995	12,8053	276,69	
15,946	24,4268	2,2563	309,91	cooling	15,946	38,272	12,7713	356,46	cooling	15,946	32,7579	11,0055	304,07	
15,948	24,2666	1,3688	320,08	cooling	15,948	39,1978	19,3059	278,06	cooling	15,948	32,7869	10,8035	307,3	
15,95	24,3686	4,6162	276,11	cooling	15,95	38,6303	18,845	276,57	cooling	15,95	32,7746	9,8061	321,07	
15,952	24,1299	4,8795	269,09	cooling	15,952	39,2356	18,4273	290,87	cooling	15,952	32,6234	9,814	318,84	
15,954	24,333	2,63	303,38		15,954	37,583	14,9632	316,19	cooling	15,954	32,8933	14,8076	252,81	
15,956	25,0667	3,1822	305,91		15,956	39,3756	16,1073	325,26	cooling	15,956	32,3423	16,4938	221,54	
15,958	24,8342	-0,921	360,02		15,958	38,376	20,1867	254,26	cooling	15,958	32,4181	13,6562	262,26	
15,96	25,572	-3,3893	404,84	cooling	15,96	37,8523	19,7351	253,25	cooling	15,96	32,7531	13,0447	275,49	
15,962	25,4004	-0,6064	363,54	cooling	15,962	39,5906	23,8883	219,5		15,962	32,3217	10,3204	307,55	
15,964	25,2589	2,524	317,8	cooling	15,964	41,0128	19,3321	303,06		15,964	32,643	11,0442	301,92	
15,966	25,1993	4,1232	294,61	cooling	15,966	39,958	21,3824	259,66		15,966	32,5568	17,2148	214,46	
15,968	25,1252	4,4245	289,37	cooling	15,968	39,5311	18,799	289,8		15,968	32,4313	18,351	196,82	
15,97	24,9394	0,2776	344,74	cooling	15,97	41,6544	24,2849	242,8		15,97	32,6912	16,7144	223,33	heating
15,972	24,9174	1,1232	332,61	cooling	15,972	40,2172	18,3725	305,36		15,972	33,1427	13,0628	280,69	heating
15,974	24,7556	1,2146	329,07	cooling	15,974	39,2363	23,3605	221,92		15,974	32,9726	6,263	373,36	heating
15,976	24,3961	-0,9733	354,63	cooling	15,976	38,4621	20,2821	254,13		15,976	33,6653	8,6498	349,68	heating
15,978	24,7686	-0,842	358	cooling	15,978	39,5416	20,8842	260,8		15,978	33,1093	13,9302	268,09	heating
15,98	24,1779	-2,5074	373,02	cooling	15,98	37,4184	15,3024	309,15		15,98	33,152	15,4459	247,5	heating
15,982	24,9449	0,3386	343,96	cooling	15,982	38,6642	16,3671	311,68		15,982	33,4499	19,3041	197,74	heating
15,984	24,6707	2,88	304,6	cooling	15,984	38,3683	16,246	309,24		15,984	33,2225	18,6722	203,39	heating
15,986	24,6715	0,4725	338,27	cooling	15,986	39,0601	18,1779	291,9		15,986	33,2491	13,5334	275,6	heating
15,988	24,9784	-0,2963	353,3	cooling	15,988	38,9536	20,6029	256,52		15,988	33,5414	15,0893	257,93	heating
15,99	25,2124	2,3862	319,08	cooling	15,99	40,9561	21,4447	272,74		15,99	33,0341	13,4937	273,15	heating
15,992	25,0645	-1,4537	370,69	cooling	15,992	39,7128	22,5417	240,03		15,992	33,0188	9,3173	331,31	heating
15,994	25,1506	-0,3536	356,51	cooling	15,994	38,4512	17,8853	287,48	heating	15,994	33,5093	9,6399	333,66	heating
15,996	25,177	0,7264	341,78	cooling	15,996	39,8835	21,3351	259,28	heating	15,996	32,9252	7,0791	361,29	heating
15,998	24,6931	-0,5522	352,89	cooling	15,998	40,544	16,0358	342,59	heating	15,998	33,6468	9,9079	331,84	heating
16	25,1798	2,8492	312,15	cooling	16	39,1144	17,9182	296,29		16	33,2272	14,9418	255,6	heating
16,002	24,3306	2,6672	302,82	cooling	16,002	38,5879	15,2925	325,64		16,002	33,0618	12,7195	284,36	heating
16,004	25,083	1,4132	330,87	cooling	16,004	40,6548	21,3836	269,38		16,004	33,1659	11,5147	302,65	heating
16,006	24,8316	3,3339	300,51	cooling	16,006	38,8438	15,6886	323,68		16,006	33,4971	15,5513	250,86	heating
16,008	25,0648	4,6206	285,78	cooling	16,008	38,2215	19,422	262,79		16,008	33,0471	14,6931	256,56	heating
16,01	25,1536	1,1592	335,41	cooling	16,01	38,1008	18,7685	270,24		16,01	33,6411	16,7402	236,25	heating
16,012	25,3429	-1,5687	376,18	cooling	16,012	38,9015	19,1344	276,31		16,012	33,1841	15,199	251,4	heating
16,014	25,1756	-5,4265	427,77	cooling	16,014	37,0926	16,7315	284,62	heating	16,014	33,2722	9,1172	337,65	heating
16,016	25,3032	-1,5173	374,91	cooling	16,016	38,2794	20,1002	254,12	heating	16,016	33,6613	7,1208	371	heating
16,018	25,5436	5,2782	283,28	cooling	16,018	37,6031	19,8656	247,94	heating	16,018	33,5109	5,6592	389,32	heating
16,02	24,7858	5,0592	275,75	cooling	16,02	38,0954	19,4039	261,28	heating	16,02	33,7304	7,3162	369,23	
16,022	25,3725	2,1198	325,04	cooling	16,022	37,5437	20,0705	244,25	heating	16,022	33,6629	14,5907	266,6	
16,024	24,805	1,2496	329,27	cooling	16,024	39,7888	17,2148	315,55	heating	16,024	33,3181	17,5704	220,13	
16,026	25,4616	2,7219	317,87	cooling	16,026	38,4377	20,895	245,22	heating	16,026	33,4976	17,4095	224,89	heating
16,028	24,9622	3,4476	300,74	cooling	16,028	37,6066	17,3719	282,85	heating	16,028	33,6847	16,6968	237,46	heating
16,03	25,5381	2,5	322,04	cooling	16,03	39,5811	19,7823	276,76	heating	16,03	33,6036	12,7331	291,74	heating
16,032	25,4187	-1,9657	382,79	cooling	16,032	40,9534	15,6034	354,36	heating	16,032	33,8895	11,95	306,68	heating
16,034	26,4616	-0,3404	374,65	cooling	16,034	39,4089	17,8089	301,94	heating	16,034	33,6738	12,7989	291,8	heating
16,036	26,0996	1,2323	347,61	cooling	16,036	39,2092	16,9645	310,95	heating	16,036	33,7448	12,7148	293,97	heating
16,038	26,3592	5,548	290,91	cooling	16,038	40,8689	23,0853	248,59	heating	16,038	33,7167	12,7429	293,18	heating
16,04	25,942	7,142	262,8	cooling	16,04	38,8299	16,8148	307,74	heating	16,04	33,8846	13,7059	282,07	heating
16,042	25,3504	3,0685	311,47	cooling	16,042	38,9469	18,9918	278,94	heating	16,042	33,6153	12,6935	292,46	heating
16,044	25,463	-0,9922	369,81	cooling	16,044	38,3409	19,0552	269,59	heating	16,044	33,7654	13,1514	288,15	heating
16,046	25,2416	-1,1027	368,25	cooling	16,046	38,7535	19,382	270,78	heating	16,046	33,6187	13,6056	279,75	heating

16,048	25,2988	-1,6905	377,27	cooling	16,048	37,0318	20,9344	225,02	heating
16,05	25,3499	1,8887	327,95	cooling	16,05	38,52	22,0775	229,84	heating
16,052	25,7148	5,7681	278,83	cooling	16,052	37,5493	19,8338	247,64	heating
16,054	25,3683	3,0066	312,58	cooling	16,054	38,5988	17,9901	288,08	heating
16,056	25,8454	1,6856	337,72	cooling	16,056	39,5046	19,6379	277,71	heating
16,058	25,1719	-1,4949	372,76	cooling	16,058	41,351	15,6154	359,75	heating
16,06	25,3723	-1,4181	374,49	cooling	16,06	40,3982	20,3598	280,11	heating
16,062	25,32	3,052	311,27	cooling	16,062	39,6732	17,9227	304,04	heating
16,064	24,761	3,8369	292,49	cooling	16,064	41,7896	20,5195	297,32	heating
16,066	24,9573	1,4277	328,91	cooling	16,066	41,454	17,6961	332,1	heating
16,068	24,8474	0,0984	345,95	cooling	16,068	40,5142	18,8563	302,75	heating
16,07	25,0663	-1,1626	366,64	cooling	16,07	39,6694	16,9265	317,91	heating
16,072	25,1094	2,6818	313,5	cooling	16,072	40,7765	21,3257	271,89	heating
16,074	25,2099	6,6587	259,32	cooling	16,074	38,1814	15,9797	310,35	heating
16,076	24,6448	4,336	283,89	cooling	16,076	39,8365	17,5196	311,96	heating
16,078	24,9547	4,0243	292,57	cooling	16,078	38,9607	19,286	275,02	heating
16,08	24,0139	0,1327	333,82	cooling	16,08	39,0774	15,218	333,52	heating
16,082	24,1946	-0,8345	349,87	cooling	16,082	38,8817	15,8718	321,64	heating
16,084	24,0051	0,559	327,74	cooling	16,084	40,5181	16,3769	337,46	
16,086	23,5077	-1,6856	352,16	cooling	16,086	39,2704	16,4948	318,37	
16,088	23,8374	-2,0842	362,34	cooling	16,088	39,4303	15,8745	329,27	
16,09	23,7163	2,0265	303,19	cooling	16,09	40,9811	21,434	273,24	
16,092	23,5824	1,6408	306,71	cooling	16,092	41,0108	12,7398	395,19	
16,094	23,5133	3,3091	282,42	cooling	16,094	40,5497	17,0973	327,83	
16,096	23,7181	4,2227	272,52	cooling	16,096	39,5317	14,659	347,68	
16,098	23,3233	-1,7515	350,51	cooling	16,098	41,7914	18,366	327,45	
16,1	23,9479	-3,7682	387,43	cooling	16,1	40,1739	17,5636	316,06	
16,102	23,4609	-4,9394	396,99	cooling	16,102	40,0568	19,4449	288,12	
16,104	23,8679	-2,3202	366,07	cooling	16,104	39,7705	18,2283	301,13	
16,106	23,8833	4,1199	276,26	cooling	16,106	40,612	22,7785	249,29	
16,108	23,6066	6,4648	239,62	cooling	16,108	38,377	18,2935	280,74	
16,11	23,7671	5,1886	259,7	cooling	16,11	40,0763	17,234	319,3	
16,112	24,0485	6,5316	244,86	cooling	16,112	39,1805	19,1342	280,22	
16,114	23,7167	0,8004	320,34	cooling	16,114	38,6286	14,1333	342,41	
16,116	23,8875	-2,0629	362,75	cooling	16,116	39,8714	17,2169	316,68	
16,118	24,1289	-1,6789	360,75	cooling	16,118	40,9152	15,4161	356,44	
16,12	24,0606	-1,1151	351,92	cooling	16,12	40,2617	16,9852	325,37	
16,122	24,6967	5,6598	266,11	cooling	16,122	39,8583	16,1742	331,07	
16,124	24,1366	9,8998	199,01	cooling	16,124	41,9999	22,9689	266,02	
16,126	24,0979	6,7133	243,01	cooling	16,126	40,564	13,0436	384,69	
16,128	24,227	3,3333	292,06	cooling	16,128	40,4034	16,7998	329,94	
16,13	23,8475	-0,678	342,83	cooling	16,13	39,1039	15,0035	336,89	
16,132	23,9455	-2,9727	376,28	cooling	16,132	40,898	18,3848	314,7	cooling
16,134	23,8545	-0,0287	333,85	cooling	16,134	39,1523	17,4386	303,53	cooling
16,136	23,5389	0,8905	316,59	cooling	16,136	40,3807	19,5864	290,67	cooling
16,138	23,5623	3,2265	284,26	cooling	16,138	40,1055	17,2676	319,24	cooling
16,14	24,0027	5,1973	262,87	cooling	16,14	40,2943	20,6702	274,32	cooling
16,142	23,6861	0,4847	324,32	cooling	16,142	38,9204	20,3924	258,99	
16,144	24,5084	0,4636	336,11	cooling	16,144	39,8989	15,8346	336,38	
16,146	24,2756	3,099	296,02	cooling	16,146	38,9806	17,8981	294,7	
16,148	24,3732	2,8045	301,5	cooling	16,148	37,473	12,623	347,37	
16,15	24,4847	1,1947	325,56	cooling	16,15	39,5955	17,323	311,34	
16,152	24,0439	-2,8819	376,38	cooling	16,152	39,8296	13,7744	364,21	
16,154	24,1274	-4,0871	394,4	cooling	16,154	39,857	16,2041	330,63	cooling
16,156	23,7078	4,2979	271,32	cooling	16,156	39,5562	12,3997	379,61	cooling
16,158	23,2791	7,0589	226,73	cooling	16,158	43,0094	23,3581	274,7	cooling

16,048	33,8459	14,2101	274,48	heating
16,05	33,6956	14,3956	269,79	heating
16,052	34,3157	17,3806	236,73	heating
16,054	33,869	17,0064	235,71	heating
16,056	33,9029	15,7032	254,4	heating
16,058	33,8984	15,3774	258,9	heating
16,06	33,8921	12,4201	300,15	heating
16,062	33,9641	9,5521	341,24	heating
16,064	34,2928	14,1749	281,22	heating
16,066	33,8243	15,3656	258,02	heating
16,068	33,8544	16,0642	248,68	heating
16,07	34,0499	16,6635	243,04	heating
16,072	33,7765	10,6601	323,13	heating
16,074	34,7577	9,292	355,97	heating
16,076	34,4186	12,3041	309,13	heating
16,078	34,4572	11,2366	324,59	heating
16,08	34,5468	13,4614	294,74	heating
16,082	34,6126	14,9634	274,67	heating
16,084	34,1064	9,881	338,64	heating
16,086	34,7351	11,5986	323,41	heating
16,088	33,9229	11,5606	312,59	heating
16,09	34,1225	8,4561	358,78	
16,092	34,442	11,1815	325,15	
16,094	33,9231	10,6186	325,76	
16,096	34,2141	10,8909	326,02	
16,098	34,0131	15,4579	259,37	
16,1	33,7105	14,7765	264,67	
16,102	33,8097	13,1418	288,91	
16,104	34,0991	12,6272	300,14	
16,106	33,7573	8,0665	359,12	
16,108	34,3161	9,2808	349,96	
16,11	33,7374	9,7359	335,51	
16,112	33,923	8,313	357,99	
16,114	34,0916	9,9249	337,81	
16,116	33,8985	9,5165	340,82	
16,118	34,2762	10,3811	334,02	
16,12	34,3416	13,34	293,57	
16,122	34,0713	10,8184	325,04	
16,124	34,2795	9,3252	348,82	
16,126	34,4449	9,8196	344,23	
16,128	34,2584	7,2609	377,38	cooling
16,13	34,8381	10,4485	340,93	cooling
16,132	34,2379	12,1945	308,13	cooling
16,134	34,3011	11,3009	321,51	cooling
16,136	34,2916	12,9492	298,34	cooling
16,138	34,0168	12,1889	305,12	cooling
16,14	34,0768	12,9285	295,62	cooling
16,142	34,0077	17,1203	236,06	cooling
16,144	33,5167	15,037	258,32	cooling
16,146	33,9249	14,2217	275,42	
16,148	33,7986	12,9952	290,8	
16,15	33,7213	10,7785	320,71	
16,152	34,0202	13,0744	292,79	
16,154	33,5646	13,1585	285,25	
16,156	33,8609	11,4987	312,59	
16,158	33,9821	12,2349	303,99	

16,16	23,1368	3,6056	273,02	cooling	16,16	40,7304	15,4484	353,4	cooling	16,16	33,8041	9,6585	337,52	
16,162	24,1154	0,586	328,91	cooling	16,162	41,1107	17,3854	331,64	cooling	16,162	33,9624	8,5752	354,88	
16,164	23,6764	-5,8706	413,02	cooling	16,164	39,8922	13,8812	363,6	cooling	16,164	34,002	10,3049	331,25	
16,166	24,9153	-2,5013	383,24	cooling	16,166	40,7674	14,1409	372,2	cooling	16,166	33,6096	8,2538	354,44	cooling
16,168	24,5238	4,8728	274,69	cooling	16,168	38,6058	11,9105	373,16	cooling	16,168	33,7968	9,4996	339,64	cooling
16,17	24,4494	7,0253	243,56	cooling	16,17	39,8952	16,5198	326,75	cooling	16,17	33,8614	11,9734	305,96	cooling
16,172	24,3893	5,0696	270,06	cooling	16,172	38,9528	12,8972	364,22	cooling	16,172	33,836	12,57	297,27	cooling
16,174	24,3197	1,2671	322,24	cooling	16,174	39,2828	14,8813	341,1	cooling	16,174	33,9404	13,9006	280,13	cooling
16,176	24,2641	-3,1476	383,17	cooling	16,176	39,4036	19,1501	283,11	cooling	16,176	33,5172	12,326	296,22	cooling
16,178	24,0134	1,6962	311,96	cooling	16,178	40,6521	14,0155	372,34	cooling	16,178	33,8474	9,7092	337,42	cooling
16,18	23,6817	5,1193	259,48	cooling	16,18	40,6237	18,332	311,61	cooling	16,18	33,7121	9,4157	339,63	cooling
16,182	23,4659	4,0712	271,11	cooling	16,182	38,8744	13,5437	354,09	cooling	16,182	33,5171	9,6069	334,23	cooling
16,184	24,317	3,0784	296,88	cooling	16,184	41,6176	18,4214	324,25	cooling	16,184	33,4377	10,7317	317,4	cooling
16,186	24,1607	-1,1663	354,03	cooling	16,186	40,8793	17,0721	332,79	cooling	16,186	33,2361	12,9458	283,63	cooling
16,188	25,2727	-0,2418	356,65	cooling	16,188	39,7556	19,9788	276,45	cooling	16,188	32,8672	13,676	268,26	cooling
16,19	24,8725	4,2531	288,23	cooling	16,19	38,7137	13,9284	346,46	cooling	16,19	32,8796	15,5239	242,61	cooling
16,192	24,9268	6,3202	260,09	cooling	16,192	41,2529	23,4589	248,73	cooling	16,192	33,0802	15,5332	245,28	
16,194	24,7566	4,797	279,01	cooling	16,194	38,503	14,0456	341,88	cooling	16,194	33,0175	14,9119	253,09	
16,196	24,9472	3,7915	295,73	cooling	16,196	39,0161	15,447	329,46	cooling	16,196	33,1846	14,1372	266,25	
16,198	24,759	1,2476	328,65	cooling	16,198	38,4611	14,8405	330,18	cooling	16,198	32,9847	11,1192	305,65	
16,2	24,6462	0,9649	331,03	cooling	16,2	39,0236	14,6228	341,09	cooling	16,2	33,4521	9,7976	330,65	
16,202	24,3011	-0,3933	345,19	cooling	16,202	37,6476	15,6353	307,7	cooling	16,202	32,9728	7,9762	349,42	
16,204	24,3291	0,5151	332,88	cooling	16,204	38,4476	18,0494	285,14	cooling	16,204	33,326	6,0907	380,71	
16,206	24,3537	1,2835	322,49	cooling	16,206	38,2833	13,2123	350,46	cooling	16,206	33,4495	8,2224	352,64	
16,208	24,2631	0,3339	334,49	cooling	16,208	38,1545	12,0318	365,16	cooling	16,208	33,237	10,395	319,3	cooling
16,21	24,4895	1,7473	317,9	cooling	16,21	38,4432	17,5891	291,51	cooling	16,21	32,8882	10,9025	307,33	cooling
16,212	24,2886	2,6446	302,55	cooling	16,212	39,542	15,1609	340,81	cooling	16,212	33,5726	15,4825	252,87	cooling
16,214	24,3852	2,0207	312,62	cooling	16,214	39,3276	21,0007	256,18	cooling	16,214	33,0347	13,0492	279,37	
16,216	24,6438	3,1545	300,39	cooling	16,216	37,8365	15,5262	311,86	cooling	16,216	33,1868	11,5291	302,74	
16,218	24,593	2,8614	303,78	cooling	16,218	40,2829	21,2221	266,44	cooling	16,218	33,1356	13,0345	280,98	
16,22	24,6714	1,5003	323,9	cooling	16,22	39,9105	18,3272	301,7	cooling	16,22	32,6169	10,6824	306,61	
16,222	24,4894	1,2198	325,27	cooling	16,222	38,9073	19,3874	272,86	cooling	16,222	32,9961	11,937	294,38	
16,224	24,319	1,4576	319,57	cooling	16,224	38,1443	11,4652	372,94	cooling	16,224	32,6234	14,8453	248,51	
16,226	24,4544	3,3393	295,16	cooling	16,226	40,6774	16,0735	343,93	cooling	16,226	32,9902	11,9893	293,56	
16,228	24,5936	4,89	275,43	cooling	16,228	39,1896	11,2097	391,12	cooling	16,228	32,9896	14,7949	254,34	
16,23	24,6822	4,0041	289,05	cooling	16,23	39,351	17,3349	307,75	cooling	16,23	32,7037	16,6063	225,02	
16,232	24,4508	2,4708	307,25	cooling	16,232	38,5349	18,0929	285,75	cooling	16,232	32,2146	12,889	270,14	
16,234	24,1869	-0,0996	339,49	cooling	16,234	39,5147	19,0764	285,7	cooling	16,234	33,2705	16,3642	236,33	
16,236	23,8748	-0,1886	336,37	cooling	16,236	37,6742	15,9367	303,86	cooling	16,236	32,232	14,0302	254,43	
16,238	23,7648	1,6967	308,48	cooling	16,238	38,6135	15,4688	323,53	cooling	16,238	32,9976	11,8064	296,22	
16,24	23,283	2,7722	286,71	cooling	16,24	38,2053	13,9375	339,23	cooling	16,24	33,2106	14,35	263,64	
16,242	23,4059	4,6755	261,82	cooling	16,242	38,4155	11,4026	377,6	cooling	16,242	32,8587	10,9062	306,86	
16,244	22,7251	2,9282	276,73	cooling	16,244	38,5535	17,4577	294,89	cooling	16,244	33,0812	8,59	342,35	
16,246	22,6086	-0,9529	329,35		16,246	39,4283	16,0529	326,75	cooling	16,246	33,6254	13,1198	286,64	
16,248	22,5433	-1,695	338,82	cooling	16,248	39,2405	18,4465	290,67	cooling	16,248	33,4218	10,38	322,09	
16,25	22,585	-0,6726	325,11	cooling	16,25	38,5642	14,1215	341,67	cooling	16,25	33,6048	13,1254	286,27	
16,252	23,048	2,3662	289,1	cooling	16,252	40,0919	17,1344	320,91	cooling	16,252	33,4547	14,6977	262,2	
16,254	23,1542	6,0268	239,42	cooling	16,254	39,7455	12,5318	380,41	cooling	16,254	32,8775	7,3354	357,04	
16,256	23,0979	2,6783	285,44		16,256	39,17	17,3612	304,85	cooling	16,256	33,8279	7,8559	363,05	
16,258	23,0357	-1,7352	346,26		16,258	37,8433	10,1689	386,85	cooling	16,258	32,7477	7,9778	346,25	
16,26	23,1833	-3,8787	378,29		16,26	39,79	15,0714	345,53	cooling	16,26	33,4258	8,4766	348,75	
16,262	22,6602	-3,1711	361,08	cooling	16,262	38,2123	16,92	297,64	cooling	16,262	33,4386	14,9325	258,69	
16,264	23,2314	3,9975	268,86	cooling	16,264	38,3673	19,5222	263,43	cooling	16,264	33,1061	15,6573	243,91	
16,266	22,4829	7,0269	216,05	cooling	16,266	37,8683	18,2713	273,94	cooling	16,266	33,0273	11,8221	296,42	
16,268	22,691	3,7902	264,21		16,268	38,5725	17,3057	297,28	cooling	16,268	33,908	15,3796	259	
16,27	22,6911	2,6108	280,69		16,27	37,4004	14,2549	323,54	cooling	16,27	33,0358	11,4619	301,57	

16,272	22,511	0,3793	309,37	
16,274	22,706	-1,4718	337,97	
16,276	23,0357	0,9035	309,38	
16,278	22,8576	-2,7641	358,15	
16,28	23,5276	-2,9356	369,92	
16,282	23,8851	0,991	320,03	cooling
16,284	23,0204	-1,1954	338,5	cooling
16,286	23,6593	0,8457	318,9	cooling
16,288	22,9821	2,6316	284,47	cooling
16,29	23,2543	-1,6461	348,07	cooling
16,292	23,2685	-3,3735	372,42	cooling
16,294	23,3959	-3,8608	381,01	cooling
16,296	23,3343	-4,2972	386,25	cooling
16,298	23,9206	4,6991	268,69	cooling
16,3	23,3976	7,8473	217,37	cooling
16,302	23,9058	5,0504	263,57	cooling
16,304	24,3237	4,1104	282,55	cooling
16,306	23,4667	-0,8819	340,36	cooling
16,308	23,6179	-2,1106	359,65	cooling
16,31	23,0312	0,2116	318,98	cooling
16,312	22,8306	-0,2391	322,48	cooling
16,314	22,8915	0,5487	312,32	cooling
16,316	23,201	1,8518	298,43	cooling
16,318	23,1116	-1,9963	350,97	cooling
16,32	23,939	1,2719	316,85	cooling
16,322	23,2974	1,605	303,23	cooling
16,324	23,4253	-0,7058	337,32	cooling
16,326	23,4027	-0,0952	328,47	cooling
16,328	22,7971	0,7163	308,66	cooling
16,33	22,9887	2,431	287,37	cooling
16,332	23,0846	5,1939	250,08	cooling
16,334	23,2594	3,3064	278,91	cooling
16,336	23,6261	1,5378	308,76	cooling
16,338	23,9666	4,231	275,87	cooling
16,34	23,9341	5,8244	253,15	cooling
16,342	24,2196	7,4438	234,5	
16,344	23,69	1,233	313,91	
16,346	23,9552	-3,6314	385,62	
16,348	23,7714	-1,2285	349,46	
16,35	23,1785	2,8673	283,92	cooling
16,352	22,9237	4,8522	252,61	
16,354	22,9305	4,9805	250,91	
16,356	22,7843	0,9816	304,77	
16,358	23,202	-0,799	335,5	
16,36	23,1896	0,0148	323,95	
16,362	23,1343	-0,4138	329,17	
16,364	22,9018	1,1772	303,68	
16,366	22,7218	3,2949	271,56	
16,368	22,4116	4,0504	256,66	
16,37	22,3527	3,2518	267	
16,372	21,6416	0,5893	294,28	
16,374	21,3893	-0,3385	303,72	
16,376	20,9336	0,2673	288,88	
16,378	20,6829	-2,3039	321,32	
16,38	20,7295	-5,4892	366,5	
16,382	21,2302	-4,6814	362,21	

16,272	38,9449	18,6371	283,87	cooling
16,274	37,8072	21,1567	232,75	cooling
16,276	38,1406	17,0308	295,08	cooling
16,278	38,4029	22,3362	224,59	cooling
16,28	38,9446	17,5923	298,47	cooling
16,282	38,3027	16,6438	302,76	cooling
16,284	38,502	14,4608	336,06	cooling
16,286	39,5729	16,9022	316,9	cooling
16,288	39,7463	16,1394	329,99	cooling
16,29	39,299	23,095	226,51	cooling
16,292	38,3286	15,9534	312,77	
16,294	40,0457	17,4947	315,23	
16,296	38,2369	16,3077	306,54	
16,298	38,6584	15,8619	318,66	
16,3	37,9257	15,9022	307,86	
16,302	38,0779	14,9999	322,6	
16,304	36,6616	12,2412	341,36	cooling
16,306	38,2793	13,9821	339,64	cooling
16,308	36,9872	14,6209	312,65	cooling
16,31	37,3067	11,2934	363,63	cooling
16,312	37,7122	15,1242	315,75	cooling
16,314	38,3922	11,2696	379,13	cooling
16,316	37,9161	15,7408	309,98	cooling
16,318	37,4893	16,3211	295,9	cooling
16,32	39,1222	19,0293	280,87	cooling
16,322	38,5732	15,5847	321,35	cooling
16,324	38,1881	19,5952	259,9	cooling
16,326	37,1977	13,5275	330,87	cooling
16,328	38,9768	17,3581	302,2	cooling
16,33	36,6947	16,2914	285,21	cooling
16,332	37,7662	18,5618	268,45	cooling
16,334	36,8925	17,1651	275,76	cooling
16,336	37,1019	11,9472	351,62	
16,338	36,4401	10,8572	357,61	cooling
16,34	38,0263	15,9598	308,46	cooling
16,342	36,8357	20,461	228,89	cooling
16,344	36,8505	17,9823	263,75	
16,346	38,0528	21,1924	235,68	
16,348	38,5713	15,1375	327,57	
16,35	38,1582	18,7668	271,06	
16,352	37,4035	15,9695	299,62	
16,354	39,2534	18,8824	284,76	
16,356	38,1785	14,1598	335,75	
16,358	38,2886	18,2169	280,57	
16,36	37,4822	15,6412	305,31	
16,362	39,2027	20,0696	267,45	
16,364	36,9706	15,2028	304,28	
16,366	38,0709	16,5892	300,28	
16,368	37,1525	16,16	293,44	
16,37	37,412	14,8232	315,76	
16,372	36,3285	15,4739	291,52	
16,374	37,4714	15,4718	307,52	
16,376	36,6158	14,7229	306,03	
16,378	36,6649	11,7608	348,12	
16,38	37,751	16,0141	303,85	
16,382	38,569	14,0561	342,65	

16,272	33,6403	11,821	305	
16,274	33,4429	13,9584	272,36	
16,276	32,9484	7,7059	352,85	
16,278	33,7433	8,1917	357,17	
16,28	33,3276	11,1147	310,5	
16,282	33,6753	10,7244	320,82	
16,284	33,6865	15,1989	258,43	
16,286	33,4064	15,8219	245,8	
16,288	33,1798	10,0816	322,88	
16,29	34,3568	13,1801	296,02	
16,292	33,4594	9,2329	338,65	
16,294	34,1003	8,6244	356,11	
16,296	33,8681	12,2489	302,2	
16,298	33,4545	9,8342	330,18	
16,3	33,7218	6,8427	375,73	
16,302	33,9962	9,894	336,91	
16,304	33,3802	9,9531	327,48	
16,306	33,8213	13,6657	281,74	
16,308	33,3038	17,3752	222,66	
16,31	32,8088	13,2217	273,8	
16,312	33,3175	12,7682	287,25	
16,314	33,1576	10,574	315,68	
16,316	33,2967	7,6717	358,2	
16,318	33,4069	10,5648	319,3	
16,32	33,0895	11,0823	307,63	
16,322	33,1628	9,8266	326,21	
16,324	33,5963	14,7036	264,09	
16,326	33,0516	14,8898	253,88	
16,328	33,6016	15,7585	249,42	
16,33	33,0528	17,2442	220,98	
16,332	32,8629	14,7138	253,7	
16,334	33,1522	14,2545	264,16	
16,336	33,4936	14,6147	263,9	
16,338	32,9189	11,2249	303,25	
16,34	33,4687	11,8048	302,83	
16,342	33,0808	13,9045	268,06	
16,344	33,1489	12,6856	286,05	
16,346	33,4094	13,3495	280,41	
16,348	33,612	14,9807	260,44	
16,35	33,4104	14,1968	268,58	heating
16,352	33,2919	14,9421	256,5	heating
16,354	33,2892	15,0248	255,31	heating
16,356	33,253	11,8398	299,32	heating
16,358	34,0721	12,5369	301,03	heating
16,36	33,5646	13,4585	281,05	heating
16,362	33,9111	13,7001	282,52	heating
16,364	33,4446	13,3317	281,15	heating
16,366	33,6757	12,2199	299,92	heating
16,368	33,3858	10,7276	316,73	heating
16,37	33,7149	13,2354	286,27	heating
16,372	33,0607	13,953	267,1	heating
16,374	33,1645	13,8572	269,89	heating
16,376	33,0792	13,0148	280,47	heating
16,378	33,2517	11,1538	308,9	heating
16,38	33,6389	10,9621	316,99	heating
16,382	33,6276	11,6476	307,25	heating

16,384	21,5063	-2,4012	334,19		16,384	38,0671	21,3836	233,21		16,384	33,7992	11,6516	309,59	heating
16,386	21,5483	1,9231	274,33		16,386	37,0171	16,8627	281,73		16,386	33,6609	10,3164	326,32	heating
16,388	21,8276	5,1255	233,47		16,388	39,033	17,1542	305,83		16,388	34,0429	10,995	322,17	
16,39	21,3597	0,6828	289,03		16,39	37,7976	11,4356	368,5		16,39	33,6192	9,5865	335,94	
16,392	22,0982	-3,1966	353,58		16,392	37,7397	15,1916	315,19		16,392	34,1202	11,9228	310,29	
16,394	21,4163	-3,7848	352,27		16,394	36,9369	12,8092	337,27		16,394	33,6702	12,7779	292,04	
16,396	21,36	-3,8582	352,51		16,396	38,1957	15,269	320,48		16,396	33,9882	14,2458	275,97	
16,398	21,0236	-0,5822	302,02		16,398	36,0775	10,9595	351,11		16,398	33,6648	14,1253	273,13	
16,4	21,2455	4,3176	236,63		16,4	37,4849	14,6368	319,38		16,4	33,9318	13,919	279,75	
16,402	20,8621	2,5774	255,59		16,402	36,8192	16,0404	290,46		16,402	33,9957	13,3515	288,58	
16,404	21,4894	0,4732	293,77		16,404	37,5258	15,8325	303,24		16,404	34,0511	14,1283	278,49	
16,406	21,3424	-3,2109	343,22		16,406	37,1713	16,8593	283,93		16,406	33,8319	14,2846	273,24	
16,408	21,5804	-4,093	358,88		16,408	38,5923	15,4554	323,42		16,408	33,9309	13,0752	291,53	
16,41	22,0312	0,2153	304,95		16,41	37,8299	17,319	286,71		16,41	34,0983	12,8332	297,25	
16,412	21,9416	2,9773	265,09		16,412	37,1986	15,0025	310,27		16,412	33,7984	10,8417	320,9	
16,414	22,3108	2,208	281,01		16,414	38,3551	19,7095	260,64		16,414	34,3096	12,7769	301	
16,416	21,8763	1,3325	287,17		16,416	38,2945	14,8883	327,18		16,416	34,2904	15,7247	259,52	
16,418	21,9573	-0,4289	312,92		16,418	37,7707	16,6155	295,72		16,418	34,1966	17,6102	231,85	
16,42	21,4079	-0,963	312,71		16,42	36,9876	11,1827	360,72		16,42	33,8986	15,2663	260,45	heating
16,422	21,7967	0,7137	294,71		16,422	38,901	15,7097	324,18		16,422	34,2352	13,6525	287,71	heating
16,424	21,0015	-1,1837	310,12		16,424	37,3088	13,2376	336,48		16,424	33,8811	9,5524	340,08	heating
16,426	22,0197	2,3108	275,5		16,426	37,4916	20,1206	242,82	cooling	16,426	33,8381	9,4983	340,23	heating
16,428	21,137	3,0575	252,72		16,428	36,5529	16,855	275,35		16,428	33,3615	11,0183	312,33	
16,43	21,2264	-0,2087	299,63		16,43	37,8606	15,869	307,41		16,43	33,3349	11,9176	299,38	
16,432	21,0937	-1,5693	316,79		16,432	36,2211	12,0398	338,02		16,432	33,3448	13,283	280,43	
16,434	21,1245	-0,4533	301,62		16,434	37,4944	12,4	350,78		16,434	33,3158	12,2363	294,66	
16,436	20,9967	-1,4843	314,25		16,436	36,9697	15,531	299,68		16,436	33,497	7,8813	358,07	
16,438	20,9646	0,9986	279,1		16,438	36,9301	15,7677	295,82		16,438	34,0844	7,0661	377,68	
16,44	20,8736	0,0406	291,21		16,44	36,6135	16,1167	286,51		16,44	33,743	8,4517	353,53	
16,442	20,6423	-1,8256	314,07		16,442	38,6265	16,9544	302,94		16,442	34,0151	10,67	326,33	
16,444	21,0042	1,01	279,49		16,444	37,4562	19,5644	250,1		16,444	34,0309	14,4916	273,13	
16,446	20,2813	1,0173	269,28		16,446	37,4219	14,936	314,32		16,446	34,0034	13,2757	289,74	
16,448	20,8945	-0,1477	294,14		16,448	39,1122	20,8501	255,28		16,448	34,0736	12,0061	308,47	
16,45	20,4965	-2,1081	315,98		16,45	38,8656	12,8069	364,26		16,45	34,1333	10,7011	327,55	
16,452	20,8424	-1,9964	319,25		16,452	38,5831	15,5889	321,42		16,452	34,023	10,87	323,65	
16,454	20,6237	-0,8689	300,44		16,454	38,0141	17,2878	289,72		16,454	34,1464	13,9856	281,82	
16,456	20,8323	1,0377	276,7		16,456	39,462	21,4239	252,15		16,456	34,0157	14,9494	266,52	
16,458	20,5016	-0,3888	292,02		16,458	37,6399	18,359	269,52		16,458	33,9449	11,652	311,62	
16,46	20,6112	1,2507	270,63		16,46	37,9602	21,2551	233,51		16,46	34,4231	12,1098	311,91	
16,462	20,5946	1,9574	260,52		16,462	36,7613	14,3926	312,68		16,462	33,9268	11,2418	317,1	
16,464	20,3948	1,9833	257,37		16,464	38,4451	16,9794	300,06		16,464	34,5183	13,9819	287,07	
16,466	20,4648	0,1445	284,05		16,466	36,0693	15,7282	284,34		16,466	34,2279	17,7917	229,75	
16,468	20,2078	-3,8924	336,88		16,468	37,6808	16,1243	301,33		16,468	34,0754	14,369	275,47	
16,47	20,6719	-4,6198	353,54		16,47	36,8643	17,7716	266,89		16,47	34,0846	11,2387	319,35	
16,472	20,4214	0,321	280,97		16,472	36,5735	13,4041	323,87		16,472	34,2248	11,2107	321,7	
16,474	20,4514	4,2489	226,49		16,474	36,8338	14,3538	314,24		16,474	33,7159	8,5263	352,11	
16,476	20,0052	1,4962	258,73		16,476	38,1086	16,4341	302,98		16,476	34,1759	9,6374	343,01	
16,478	20,5017	-1,2391	303,9		16,478	37,0574	18,2765	262,53		16,478	33,7979	10,9607	319,23	
16,48	20,3077	-3,1126	327,38		16,48	36,8292	16,3944	285,65		16,48	33,5546	7,9722	357,6	
16,482	20,6488	-0,0886	289,88		16,482	38,1901	20,9304	241,26		16,482	33,9879	9,0101	349,15	
16,484	20,4701	2,6939	248,49		16,484	37,4339	11,5916	361,24		16,484	33,7915	9,0481	345,88	
16,486	20,4347	4,1553	227,56		16,486	37,7684	16,0418	303,7		16,486	34,0601	8,4013	358,67	cooling
16,488	20,4888	3,4927	237,58	heating	16,488	36,4621	13,7071	318,08		16,488	34,0337	13,0211	293,72	cooling
16,49	20,5037	1,9173	259,81	heating	16,49	38,4194	17,5998	291,03		16,49	33,9077	15,0767	263,23	cooling
16,492	20,8248	-0,3682	296,25	heating	16,492	36,6814	15,6829	293,53		16,492	33,4692	11,9482	300,83	
16,494	20,7853	-2,5174	325,74	heating	16,494	37,2216	15,9198	297,77		16,494	34,0314	12,5884	299,74	

16,496	21,169	0,0966	294,56	heating	16,496	36,6361	10,7034	362,5		16,496	33,4494	10,5393	320,25	
16,498	21,0065	3,0961	250,36	heating	16,498	37,8224	15,2939	314,92		16,498	34,1376	11,417	317,6	
16,5	21,0354	3,2377	248,79	heating	16,5	35,9799	13,3487	316,35		16,5	33,5773	13,5182	280,4	
16,502	20,9637	0,2803	289,12	heating	16,502	37,4436	13,9512	328,39		16,502	33,396	14,0168	270,89	
16,504	20,9415	-1,2687	310,47	heating	16,504	36,6517	15,472	296,06		16,504	33,3078	11,6766	302,37	
16,506	21,0397	-2,1329	323,92	heating	16,506	36,2743	11,7061	343,43		16,506	33,6348	12,6571	293,24	
16,508	21,1136	1,3604	276,12	heating	16,508	37,4715	18,7601	261,56		16,508	33,4679	11,5965	305,73	
16,51	20,9315	1,2897	274,56	heating	16,51	37,8635	19,4203	257,81		16,51	33,6798	12,1574	300,85	
16,512	20,7999	-1,4091	310,45	heating	16,512	37,2922	20,5094	234,6		16,512	33,8929	13,3296	287,44	
16,514	21,2864	-1,0537	312,28	heating	16,514	36,7026	15,9137	290,6		16,514	33,4705	12,5708	292,15	
16,516	20,8664	-0,3435	296,48	heating	16,516	38,2948	18,6595	274,47		16,516	33,9077	13,1703	289,88	
16,518	21,1015	0,9436	281,78	heating	16,518	37,7309	12,4963	352,74		16,518	33,8089	12,9237	291,94	
16,52	20,8105	2,0053	262,87	heating	16,52	37,7985	19,6707	253,4		16,52	34,0851	9,8058	339,39	
16,522	20,9031	-0,8812	304,51	heating	16,522	36,7982	15,6948	294,99		16,522	33,6897	6,8885	374,64	
16,524	21,0506	-4,6458	359,2	heating	16,524	38,4912	18,8293	274,85		16,524	33,8519	8,9909	347,52	
16,526	21,4497	-3,2406	345,13		16,526	36,5584	16,3838	282,01		16,526	33,4051	9,6738	331,73	
16,528	21,2483	-5,9142	379,69		16,528	37,1248	18,2608	263,69		16,528	33,7631	14,4661	269,74	cooling
16,53	21,3758	-4,0025	354,75		16,53	36,7887	17,282	272,67		16,53	33,2974	14,9515	256,45	
16,532	21,4659	-0,2503	303,56		16,532	37,2355	19,2867	250,9		16,532	33,5027	11,9434	301,37	
16,534	21,1827	-0,6088	304,61		16,534	36,2488	17,9542	255,73		16,534	33,6203	10,0013	330,16	
16,536	21,8656	-0,7687	316,4		16,536	37,5936	19,0016	259,89	heating	16,536	33,4711	11,2944	310	
16,538	21,1202	-1,2847	313,19		16,538	37,0957	21,9705	211,43	heating	16,538	33,3822	11,6169	304,25	
16,54	21,4166	-3,0753	342,36		16,54	36,8057	19,3782	243,61	heating	16,54	33,527	13,2808	283,01	
16,542	21,2125	-0,8824	308,85		16,542	37,7887	22,5743	212,67	heating	16,542	33,3851	12,3335	294,27	
16,544	21,3134	0,3586	292,92		16,544	38,2118	16,5519	302,77	heating	16,544	33,3226	9,5007	332,99	
16,546	21,1231	0,2483	291,8		16,546	37,5947	15,5927	307,55	heating	16,546	33,4269	11,0397	312,94	
16,548	21,6979	4,6917	237,72		16,548	36,9951	12,1417	347,41	heating	16,548	32,9186	12,7783	281,53	
16,55	21,1749	2,7615	257,39		16,55	38,5476	19,3228	268,73	heating	16,55	32,7831	15,3326	243,93	
16,552	21,5912	1,4915	280,96		16,552	37,4562	16,5712	291,94	heating	16,552	32,6875	17,5328	211,84	
16,554	21,8743	2,4788	271,12		16,554	37,6273	21,5092	225,31	heating	16,554	32,6541	17,1402	216,86	
16,556	21,2975	-0,713	307,67		16,556	36,9048	17,8301	266,64	heating	16,556	32,6554	13,04	274,19	
16,558	22,1069	0,6787	299,53		16,558	37,8786	19,0521	263,17	heating	16,558	33,0881	12,6164	286,16	
16,56	21,5263	3,8533	247,04		16,56	36,4819	17,939	259,2	heating	16,56	32,9344	13,2727	274,84	
16,562	21,8877	1,9598	278,56		16,562	37,4468	18,7796	260,94	heating	16,562	32,898	14,1817	261,63	
16,564	21,7431	-1,7574	328,5		16,564	37,1817	17,228	278,92	heating	16,564	32,8052	13,6569	267,67	
16,566	22,0811	-4,759	375,18		16,566	37,3499	14,9636	312,93	heating	16,566	33,1429	13,0709	280,58	
16,568	21,5568	-6,287	389,22		16,568	37,163	14,3071	319,49	heating	16,568	32,9165	10,7598	309,72	
16,57	22,0191	2,633	270,99		16,57	38,4854	13,626	347,5	heating	16,57	33,2537	11,4292	305,07	
16,572	21,3081	5,8993	215,39		16,572	37,7053	18,9236	262,54	heating	16,572	33,0934	13,4417	274,7	
16,574	21,6511	3,6914	251,05		16,574	37,2657	15,7202	301,17	heating	16,574	33,0956	12,3609	289,84	
16,576	22,1504	1,7399	285,31		16,576	38,8826	20,356	258,97	heating	16,576	32,8554	9,9846	319,7	
16,578	22,2804	-0,8777	323,72		16,578	38,7977	14,7228	336,53	heating	16,578	33,307	9,1582	337,56	
16,58	23,0815	-1,1641	338,92		16,58	37,8698	15,495	312,77	heating	16,58	33,2645	9,0004	339,18	
16,582	22,7773	0,689	308,76		16,582	37,5656	16,4434	295,26	heating	16,582	33,2039	10,8406	312,61	
16,584	23,1665	1,8666	297,74		16,584	38,4563	23,9299	203,06	heating	16,584	32,9713	12,2687	289,39	
16,586	23,0902	3,5882	272,61		16,586	36,9378	18,524	257,4	heating	16,586	33,0053	9,6594	326,34	
16,588	23,3869	5,2185	253,97		16,588	37,1509	19,9702	240,16	heating	16,588	33,1975	7,9723	352,61	
16,59	22,9741	-0,0597	321,98		16,59	36,5609	15,8345	289,73	heating	16,59	32,7497	6,5827	365,78	
16,592	23,4769	-1,2211	345,24		16,592	37,8181	17,5696	283,04	heating	16,592	33,045	9,7858	325,13	
16,594	23,2574	-1,9729	352,68		16,594	36,2189	21,0003	212,73	heating	16,594	32,6933	14,6964	251,57	
16,596	23,3627	0,3577	321,58		16,596	37,4444	21,131	228,04	heating	16,596	32,5304	17,3807	211,77	
16,598	23,5818	3,1864	285,1		16,598	37,024	18,2894	261,88	heating	16,598	32,2761	14,8313	243,85	
16,6	23,5028	2,4229	294,67		16,6	38,1312	17,0341	294,91	heating	16,6	32,9058	12,6256	283,49	
16,602	24,2538	-0,4438	345,23		16,602	37,2611	15,7987	300,01	heating	16,602	32,7413	8,3526	340,92	
16,604	24,4454	1,8219	316,24		16,604	39,0368	17,1103	306,5	heating	16,604	32,7457	8,7156	335,9	
16,606	25,0593	5,5355	272,91		16,606	37,8477	18,1034	276	heating	16,606	32,4836	9,8707	316,09	

16,608	24,9038	6,3701	259,07		16,608	37,856	14,305	329,21	heating	16,608	32,6634	12,0493	288,15	
16,61	24,9978	3,5961	299,16		16,61	38,6636	19,7701	264,1	heating	16,61	32,6312	13,6136	265,84	
16,612	24,7132	-1,8737	371,64		16,612	39,0144	17,5331	300,28	heating	16,612	32,6593	14,3474	255,97	
16,614	25,0707	-2,8707	390,58		16,614	38,5473	19,093	271,94	heating	16,614	32,9717	12,9961	279,23	
16,616	25,0856	1,9692	323,13		16,616	38,041	16,1997	305,31	heating	16,616	32,7836	11,4805	297,79	
16,618	24,9166	7,7508	239,95		16,618	39,3131	17,0294	311,49	heating	16,618	32,6691	10,1625	314,61	
16,62	24,5857	5,6822	264,24		16,62	38,7062	12,4932	366,42	heating	16,62	32,4496	8,3306	337,15	
16,622	24,7889	1,4834	325,78		16,622	39,2717	16,6404	316,35	heating	16,622	32,9357	10,6541	311,46	
16,624	24,805	-3,7409	399,03		16,624	38,7165	14,795	334,39	heating	16,624	32,4584	11,6871	290,35	
16,626	24,7002	-4,6335	410,04		16,626	39,9741	20,5263	271,85	heating	16,626	32,641	14,0772	259,49	
16,628	24,7129	-1,3533	364,37	cooling	16,628	38,1212	20,1927	250,61	heating	16,628	32,4889	13,7605	261,8	
16,63	24,3285	1,6593	316,88	cooling	16,63	39,0018	18,9157	280,77	heating	16,63	32,6631	11,9236	289,91	
16,632	24,7079	4,7111	279,53	cooling	16,632	37,9146	15,8422	308,54	heating	16,632	32,7922	10,8033	307,37	
16,634	24,4339	4,9607	272,21	cooling	16,634	37,9168	14,1294	332,51	heating	16,634	33,0112	13,4971	272,78	
16,636	24,4176	-0,531	348,74	cooling	16,636	37,6377	18,5725	266,5	heating	16,636	32,7867	13,8901	264,15	
16,638	24,4913	-2,0698	371,29	cooling	16,638	38,3724	20,5035	249,78	heating	16,638	32,6627	14,2519	257,36	
16,64	24,2386	-1,5648	360,69	cooling	16,64	37,5288	20,4793	238,33	heating	16,64	32,4387	12,1343	283,82	
16,642	23,9675	-3,5176	384,2	cooling	16,642	37,5088	15,8112	303,3	heating	16,642	32,204	7,9314	339,3	
16,644	24,5453	-0,6078	351,6	cooling	16,644	38,8973	19,8181	266,7	heating	16,644	32,5357	9,959	315,59	
16,646	24,2768	1,5262	318,02	cooling	16,646	38,5708	16,7801	304,6	heating	16,646	32,1688	13,7081	258,05	
16,648	24,2985	4,3643	278,65	cooling	16,648	38,4874	19,4857	265,62	heating	16,648	32,2089	15,4524	234,23	
16,65	24,0588	6,6035	244	cooling	16,65	38,0158	13,1232	347,96	heating	16,65	32,2742	14,5389	247,91	heating
16,652	23,3248	2,4043	292,44	cooling	16,652	39,6918	14,2504	355,63	heating	16,652	32,2878	9,8618	313,48	heating
16,654	23,3545	-3,1218	370,1	cooling	16,654	38,4394	14,6379	332,71	heating	16,654	32,6781	6,3465	368,08	heating
16,656	23,3659	-3,0042	368,61	cooling	16,656	38,691	18,4348	283,15		16,656	32,6783	10,5039	309,96	
16,658	23,6614	-0,8846	343,12	cooling	16,658	38,0707	15,455	316,13		16,658	32,2487	11,7818	286,1	
16,66	23,4955	4,2162	269,5	cooling	16,66	38,7973	14,3774	341,35		16,66	32,2792	12,9371	270,37	
16,662	23,1091	4,4212	261,23	cooling	16,662	37,6867	11,141	371,07		16,662	32,2622	13,246	265,82	
16,664	22,8101	0,6476	309,8	cooling	16,664	39,251	13,8756	354,71		16,664	31,8942	10,0149	305,84	
16,666	23,3949	2,4905	292,21	cooling	16,666	38,4097	16,9597	299,84		16,666	32,4279	12,4101	279,82	
16,668	22,7121	2,8695	277,37	cooling	16,668	38,5925	16,3687	310,66		16,668	32,1433	15,2357	236,34	
16,67	23,2193	2,6545	287,46	cooling	16,67	38,6697	18,3388	284,2		16,67	31,945	12,4966	271,86	heating
16,672	23,171	3,3103	277,62	cooling	16,672	39,2858	15,236	336,18		16,672	32,3219	12,059	283,25	heating
16,674	22,9384	1,0288	306,26	cooling	16,674	38,7232	14,3688	340,44		16,674	32,1935	10,2066	307,34	heating
16,676	22,9472	-2,222	351,83	cooling	16,676	38,4986	11,7796	373,49		16,676	32,5772	9,0686	328,61	heating
16,678	23,1357	-3,1085	366,85	cooling	16,678	39,4838	16,3658	323,15		16,678	32,4889	13,5082	265,32	heating
16,68	23,3357	-2,8616	366,2	cooling	16,68	38,9024	15,6984	324,36		16,68	32,0361	12,6959	270,35	heating
16,682	23,5043	4,4054	266,97	cooling	16,682	38,5227	19,3329	268,24		16,682	32,1428	10,0041	309,47	heating
16,684	23,3138	9,4501	193,79	cooling	16,684	37,6644	13,1955	342,04		16,684	32,333	8,7158	330,13	heating
16,686	22,9706	6,4448	231,01	cooling	16,686	38,9288	14,0635	347,58		16,686	32,0708	6,4091	358,71	
16,688	23,5285	2,0196	300,66		16,688	37,5568	12,786	346,26		16,688	32,6441	10,6049	308,08	
16,69	22,802	-4,0359	375,15		16,69	37,9974	15,6679	312,13	cooling	16,69	32,293	14,8379	244	
16,692	23,2966	-1,8791	351,92	cooling	16,692	37,2736	12,5956	344,96	cooling	16,692	31,9272	11,2117	289,57	
16,694	22,9204	4,1245	262,74	cooling	16,694	37,8877	10,718	379,79	cooling	16,694	32,3792	9,4812	320,08	
16,696	22,4737	3,6067	263,73		16,696	37,0659	8,579	398,2	cooling	16,696	32,2105	6,5224	359,08	
16,698	22,3772	0,2622	309,14		16,698	38,357	13,1638	352,16	cooling	16,698	32,4624	4,8683	385,72	
16,7	22,882	1,5616	298,03		16,7	37,472	16,1657	297,83	cooling	16,7	32,4136	8,8416	329,5	
16,702	22,8634	0,3106	315,25		16,702	37,4769	13,8324	330,52	cooling	16,702	32,1801	11,0662	295,14	
16,704	22,612	3,1518	272,03		16,704	38,0806	16,6486	299,59	cooling	16,704	32,0892	9,9787	309,07	
16,706	22,3905	3,6639	261,77		16,706	38,6426	15,9298	317,49	cooling	16,706	32,5739	11,2754	297,72	
16,708	22,055	-0,8981	320,85		16,708	38,3283	17,1175	296,5	cooling	16,708	32,3132	9,2616	322,23	
16,71	22,3699	-4,6983	378,37		16,71	38,1369	15,4477	317,16	cooling	16,71	32,9267	13,4953	271,62	
16,712	21,8549	-6,9039	402,01		16,712	38,7241	16,874	305,43	cooling	16,712	32,1656	16,6251	217,23	
16,714	22,7141	-2,7809	356,38	cooling	16,714	38,099	15,0064	322,8	cooling	16,714	32,0407	15,1652	235,89	
16,716	22,5109	5,5842	236,61	cooling	16,716	38,0533	19,3342	261,67	cooling	16,716	31,9579	14,6969	241,28	
16,718	22,5076	8,6681	193,46	cooling	16,718	37,1579	14,6386	314,79	cooling	16,718	31,765	13,9819	248,58	

16,72	22,4964	5,9522	231,26	
16,722	23,0417	2,8325	282,49	
16,724	22,6244	-4,2476	375,63	
16,726	23,089	-2,6323	359,55	
16,728	23,1984	0,9431	311,1	
16,73	23,2881	1,4868	304,75	
16,732	23,6205	3,2688	284,49	
16,734	23,627	6,3837	241,04	
16,736	24,0102	4,2224	276,6	
16,738	23,799	1,1595	316,47	
16,74	23,9218	-2,2688	366,11	
16,742	23,9945	-2,1106	364,91	
16,744	24,7079	3,6603	294,21	
16,746	23,918	3,2046	289,54	
16,748	24,4575	4,1011	284,55	
16,75	24,2125	3,8995	283,95	
16,752	24,2213	1,4954	317,67	
16,754	24,2789	0,8768	327,13	
16,756	24,3228	1,935	312,95	
16,758	24,6159	-0,1363	346	
16,76	24,2708	1,9091	312,58	
16,762	24,0018	0,7425	325,13	
16,764	23,4519	-4,1827	386,29	
16,766	24,0294	-2,4683	370,4	cooling
16,768	23,1979	-0,3142	328,66	cooling
16,77	23,7675	1,3758	313	cooling
16,772	23,3295	4,0261	269,83	cooling
16,774	22,942	0,7011	310,89	cooling
16,776	22,9086	-3,3578	367,17	cooling
16,778	23,4309	0,8891	315,1	cooling
16,78	23,0409	0,0678	321,13	cooling
16,782	23,153	1,1014	308,25	cooling
16,784	23,4855	3,038	285,82	cooling
16,786	23,0426	-0,7763	332,95	cooling
16,788	24,1343	0,3791	332,06	cooling
16,79	23,5653	3,1772	285	cooling
16,792	24,1663	1,6075	315,34	cooling
16,794	23,9178	2,5537	298,64	cooling
16,796	23,7217	-0,074	332,63	cooling
16,798	23,3287	-7,0377	424,48	cooling
16,8	24,2763	-1,891	365,78	cooling
16,802	23,1773	0,0306	323,56	cooling
16,804	23,3878	3,7935	273,9	cooling
16,806	23,4433	8,0645	214,97	cooling
16,808	22,5864	3,8784	261,51	cooling
16,81	23,3815	2,3905	293,42	cooling
16,812	23,0228	4,6872	256,3	cooling
16,814	23,3428	-0,0173	326,54	cooling
16,816	23,074	1,4568	302,18	cooling
16,818	23,1491	4,692	258	cooling
16,82	22,4827	1,7918	289,23	
16,822	23,3707	5,7137	246,82	
16,824	22,1609	2,2133	278,84	
16,826	22,6726	-3,6307	367,68	
16,828	22,8345	-1,8941	345,67	
16,83	22,2704	-1,1414	327,26	

16,72	37,9457	14,9268	321,77	cooling
16,722	36,4918	14,9937	300,51	cooling
16,724	36,7727	15,8183	292,91	cooling
16,726	36,1063	14,0803	307,89	cooling
16,728	36,4657	11,4188	350,12	cooling
16,73	36,4181	7,6103	402,69	cooling
16,732	38,0589	11,4089	372,53	cooling
16,734	37,3546	13,6222	331,74	cooling
16,736	37,3509	9,8495	384,43	cooling
16,738	38,1936	13,6927	342,49	cooling
16,74	37,7936	11,1698	372,16	cooling
16,742	37,8006	13,8647	334,59	cooling
16,744	37,3948	13,5888	332,77	cooling
16,746	37,9002	12,5381	354,52	cooling
16,748	37,2244	10,6274	371,79	cooling
16,75	37,535	16,5586	293,22	cooling
16,752	36,9159	11,5415	354,7	cooling
16,754	38,0842	15,5842	314,52	cooling
16,756	36,782	16,0832	289,34	cooling
16,758	37,4278	15,3738	308,28	cooling
16,76	37,2982	16,8853	285,34	cooling
16,762	36,9238	15,0212	306,17	cooling
16,764	37,3758	13,6022	332,32	cooling
16,766	38,1016	17,8258	283,43	cooling
16,768	36,8392	17,301	273,11	cooling
16,77	36,9463	13,0878	333,51	cooling
16,772	38,057	16,7766	297,47	cooling
16,774	37,5251	11,9955	356,87	cooling
16,776	37,7346	15,7451	307,38	cooling
16,778	37,0566	14,6789	312,81	cooling
16,78	37,7738	13,3356	341,61	cooling
16,782	37,4255	13,922	328,54	cooling
16,784	37,2755	18,0949	268,12	cooling
16,786	36,8972	15,7772	295,23	cooling
16,788	37,0418	18,3795	260,87	cooling
16,79	35,7273	14,5699	295,75	cooling
16,792	36,7198	13,2678	327,82	cooling
16,794	36,1755	15,3615	290,95	cooling
16,796	35,5879	11,0622	342,83	cooling
16,798	36,1315	11,8745	339,08	cooling
16,8	36,3043	12,6182	331,1	cooling
16,802	35,7886	13,6383	309,63	cooling
16,804	35,967	14,5601	299,24	cooling
16,806	36,9894	18,3105	261,1	cooling
16,808	36,5992	12,268	340,11	cooling
16,81	36,4965	13,7765	317,59	cooling
16,812	35,7618	10,8994	347,54	cooling
16,814	36,402	14,0126	312,97	cooling
16,816	35,3532	15,6163	275,89	cooling
16,818	35,4304	18,3431	238,85	cooling
16,82	35,2521	14,4876	290,26	cooling
16,822	35,8279	13,2155	316,09	cooling
16,824	35,4773	12,2059	325,3	cooling
16,826	36,5284	16,5087	279,85	cooling
16,828	36,2012	19,1331	238,59	cooling
16,83	36,0709	17,7894	255,55	

16,72	31,7066	12,3782	270,18	heating
16,722	31,7271	12,7394	265,42	heating
16,724	31,533	11,3467	282,18	heating
16,726	31,7276	11,2503	286,24	heating
16,728	32,3148	14,4649	249,52	heating
16,73	32,4008	15,2694	239,47	heating
16,732	32,9595	17,0574	222,29	heating
16,734	32,5765	15,1891	243,05	heating
16,736	32,7955	11,9913	290,81	heating
16,738	32,415	10,6886	303,7	heating
16,74	32,2355	10,6645	301,53	heating
16,742	32,0862	10,054	307,98	heating
16,744	32,3278	11,2901	294,08	heating
16,746	32,1689	9,1824	321,32	heating
16,748	32,6573	8,641	335,71	heating
16,75	32,8926	10,8932	307,52	heating
16,752	32,742	13,3133	271,58	heating
16,754	32,8952	15,7706	239,38	heating
16,756	32,6259	15,2064	243,5	heating
16,758	32,7237	13,68	266,2	heating
16,76	32,3902	12,324	280,5	heating
16,762	32,6933	13,6535	266,15	heating
16,764	32,6004	12,7611	277,32	heating
16,766	32,7159	12,541	282,02	heating
16,768	32,5127	9,5639	320,79	heating
16,77	32,9129	8,9195	335,39	heating
16,772	32,934	10,0449	319,96	heating
16,774	32,946	13,3961	273,28	heating
16,776	32,9938	13,34	274,73	heating
16,778	33,0415	11,0146	307,9	heating
16,78	32,9745	8,2341	345,83	heating
16,782	32,9339	7,707	352,64	heating
16,784	33,1247	10,4777	316,57	
16,786	33,0641	12,3447	289,63	
16,788	32,9555	13,5533	271,21	
16,79	33,0315	13,3091	275,69	
16,792	33,0815	13,6735	271,3	
16,794	32,9749	12,53	285,79	heating
16,796	33,0031	14,5184	258,39	heating
16,798	32,9528	14,5311	257,51	heating
16,8	32,7906	13,694	266,94	heating
16,802	32,7598	10,408	312,44	heating
16,804	33,217	9,877	326,26	heating
16,806	33,398	10,3794	321,76	heating
16,808	33,6278	12,9143	289,54	heating
16,81	33,5708	14,2836	269,61	heating
16,812	33,715	13,5223	282,26	heating
16,814	33,4974	11,4963	307,54	heating
16,816	33,5602	10,3359	324,64	heating
16,818	33,3704	11,838	300,99	
16,82	33,0844	14,0057	266,69	
16,822	32,715	14,937	248,51	
16,824	32,9321	15,0068	250,57	heating
16,826	32,9376	15,2972	246,59	heating
16,828	33,043	14,1575	263,99	heating
16,83	33,2588	13,9047	270,54	heating

16,832	22,4482	-2,8621	353,8		16,832	36,0459	18,4576	245,86		16,832	33,4008	13,1359	283,27	heating
16,834	23,0014	2,0494	292,88	cooling	16,834	36,2425	17,4427	262,79		16,834	33,5089	13,6899	277,04	heating
16,836	22,6323	0,5672	308,44	cooling	16,836	35,1673	19,1001	224,6		16,836	33,3987	13,9207	272,27	heating
16,838	22,7619	0,2985	314,01	cooling	16,838	34,8437	18,9485	222,19		16,838	33,7263	15,3757	256,51	heating
16,84	23,0331	2,7287	283,83	cooling	16,84	35,3029	19,9955	213,97		16,84	33,5738	13,3635	282,51	heating
16,842	22,3128	-1,6502	334,97		16,842	35,57	14,8619	289,47		16,842	33,5813	8,8768	345,33	heating
16,844	23,1735	-0,2066	326,82	cooling	16,844	35,1715	16,2309	264,76		16,844	33,4785	6,2191	381,05	heating
16,846	22,3564	2,83	272,95	cooling	16,846	35,4334	15,4195	279,76		16,846	33,7342	7,4005	368,11	heating
16,848	22,7002	4,0813	260,26	cooling	16,848	36,7528	19,9642	234,68	heating	16,848	33,5196	10,2653	325,06	
16,85	22,6209	5,6523	237,2		16,85	36,2516	16,8605	271,06	heating	16,85	33,7191	12,3332	298,94	
16,852	22,4657	2,9398	272,94		16,852	36,2213	15,0577	295,84	heating	16,852	33,4222	10,7708	316,63	
16,854	22,4614	-3,7298	366,11		16,854	35,7899	11,3467	341,68	heating	16,854	33,7353	9,588	337,54	
16,856	23,4409	-0,6468	336,71		16,856	35,7985	13,0686	317,73	heating	16,856	33,3788	9,2231	337,66	
16,858	23,1485	-0,1334	325,45		16,858	34,5833	15,0567	272,95		16,858	33,4272	7,9157	356,61	
16,86	23,0331	1,9801	294,29		16,86	35,0541	18,4426	232,21	heating	16,86	33,2307	8,0723	351,68	
16,862	23,1263	4,857	255,38		16,862	34,5463	17,1244	243,53	heating	16,862	33,1651	7,9365	352,66	
16,864	22,4915	2,0091	286,31		16,864	35,1328	13,6504	300,29	heating	16,864	33,1074	9,4898	330,14	
16,866	23,0596	0,9965	308,41		16,866	35,5643	12,6954	319,67	heating	16,866	33,0048	10,9734	307,97	
16,868	22,4442	2,6036	277,34		16,868	37,1896	13,0445	337,51	heating	16,868	32,9416	13,3884	273,32	
16,87	22,7751	1,068	303,43		16,87	36,8144	17,1737	274,55	heating	16,87	32,8553	13,9276	264,58	
16,872	22,5694	0,5929	307,2		16,872	36,7513	14,917	305,21	heating	16,872	33,0156	12,7771	282,9	
16,874	22,4348	-1,0341	328,06		16,874	37,5824	15,8665	303,56	heating	16,874	32,8862	10,5836	311,76	
16,876	22,1331	-3,6634	360,6		16,876	37,3692	14,2332	323,41	heating	16,876	32,9384	10,3134	316,26	
16,878	22,9881	0,235	318,05		16,878	36,4141	17,0121	271,21		16,878	32,7692	11,4708	297,72	
16,88	22,286	-0,3336	316,19		16,88	36,0575	17,6515	257,29		16,88	33,0142	15,1722	249,4	
16,882	22,3707	-0,1407	314,68		16,882	36,4499	19,4967	236,98	heating	16,882	32,4486	14,5141	250,7	
16,884	22,254	0,1345	309,2		16,884	35,4924	14,2838	296,46	heating	16,884	33,0092	12,6004	285,28	
16,886	21,998	-2,8405	347,21		16,886	36,6101	16,7542	277,56	heating	16,886	33,2818	12,0674	296,55	
16,888	22,2371	-2,9231	351,7	cooling	16,888	36,2087	16,422	276,59	heating	16,888	33,4082	10,3782	321,93	
16,89	21,9235	-0,0578	307,27	cooling	16,89	36,9527	18,1409	262,96	heating	16,89	33,5966	12,02	301,61	
16,892	22,1087	-0,8363	320,74	cooling	16,892	36,2735	20,2854	223,49	heating	16,892	33,6418	13,431	282,52	
16,894	21,9588	0,3679	301,81	cooling	16,894	36,8515	19,5988	241,17	heating	16,894	33,3895	11,0282	312,58	
16,896	21,9466	1,263	289,13	cooling	16,896	36,4048	15,9713	285,63	heating	16,896	33,5391	12,1713	298,69	
16,898	21,6362	-1,2084	319,33	cooling	16,898	36,9666	14,0044	320,98	heating	16,898	33,3836	13,1905	282,27	
16,9	22,5055	0,4021	308,97	cooling	16,9	37,5552	14,3922	323,78	heating	16,9	33,3397	11,8885	299,86	
16,902	21,5971	-1,2678	319,62	cooling	16,902	38,1678	15,874	311,63	heating	16,902	33,6198	13,6127	279,67	
16,904	21,7838	0,7109	294,57	cooling	16,904	37,2643	19,1362	253,4	heating	16,904	32,8802	9,9488	320,55	
16,906	21,5325	2,7885	262,01	cooling	16,906	37,0047	16,0213	293,32	heating	16,906	33,8746	9,3875	342,29	
16,908	21,2928	-0,5927	305,93		16,908	38,1126	17,5178	287,88	heating	16,908	33,5595	10,9001	316,75	
16,91	21,3441	-4,4186	360,12		16,91	37,3549	15,987	298,69	heating	16,91	33,4814	9,916	329,41	
16,912	21,2998	-3,4772	346,35	cooling	16,912	36,9136	20,3538	231,48	heating	16,912	33,4231	11,3813	308,11	
16,914	21,5579	-4,163	359,54	cooling	16,914	36,0784	19,9352	225,66	heating	16,914	33,4925	14,1984	269,7	
16,916	21,4212	2,1693	269,11	cooling	16,916	36,2326	19,0307	240,46	heating	16,916	32,7681	10,4916	311,39	
16,918	21,1318	4,192	236,79	cooling	16,918	35,4178	14,4379	293,27	heating	16,918	33,3988	13,9465	271,91	
16,92	21,0353	0,9314	281,02		16,92	36,4889	17,1359	270,53	heating	16,92	32,9322	14,8869	252,25	
16,922	22,2609	3,658	260,04		16,922	36,4979	19,7367	234,3	heating	16,922	33,1974	14,0152	268,14	
16,924	21,3706	-1,5024	319,73		16,924	36,5344	19,4242	239,18	heating	16,924	33,5259	16,8088	233,68	
16,926	22,0712	-2,293	340,57		16,926	36,1673	16,6121	273,35	heating	16,926	32,8591	13,6195	268,94	
16,928	21,9586	-0,0989	308,33		16,928	37,0676	14,085	321,26	heating	16,928	33,8649	12,7058	295,77	
16,93	21,6921	-0,8903	315,67		16,93	36,2893	12,9158	326,73	heating	16,93	33,4734	14,9003	259,62	
16,932	22,336	4,1636	254,02		16,932	36,5234	14,428	308,86	heating	16,932	33,5415	13,7485	276,68	
16,934	21,9639	7,7023	199,36		16,934	37,001	20,143	235,65	heating	16,934	33,3435	13,4542	278,02	
16,936	22,3308	1	298,17		16,936	37,5609	17,1022	285,98	heating	16,936	33,7169	16,8055	236,4	
16,938	22,3035	-0,2024	314,6		16,938	37,0495	17,2894	276,22	heating	16,938	32,7684	11,1436	302,28	heating
16,94	22,0035	-3,2572	353,11		16,94	37,0446	13,3034	331,87	heating	16,94	33,6609	12,5485	295,12	heating
16,942	21,9489	-6,4932	397,58		16,942	37,7566	13,3411	341,29	heating	16,942	33,0989	11,6243	300,18	heating

16,944	23,0108	0,8465	309,82	16,944	37,0221	15,7952	296,72	heating	16,944	33,4028	10,4591	320,72	heating
16,946	21,7559	-1,4959	325,03	16,946	35,6754	17,5582	253,25	heating	16,946	33,4954	11,3823	309,11	
16,948	22,7568	0,5561	310,33	16,948	35,1864	14,1195	294,48	heating	16,948	33,5677	11,824	303,94	
16,95	22,6727	5,695	237,32	16,95	35,5483	14,347	296,36	heating	16,95	33,8229	10,1206	331,32	
16,952	22,3611	2,394	279,11	16,952	34,265	10,4515	332,88	heating	16,952	33,7426	11,6566	308,73	
16,954	23,3985	3,4135	279,36	16,954	35,4626	13,4173	308,16	heating	16,954	33,7722	13,9615	276,92	
16,956	23,0841	4,5278	259,39	16,956	34,8833	17,4826	243,24	heating	16,956	33,4979	14,0164	272,32	
16,958	23,5685	-3,1027	372,82	16,958	35,3627	13,263	308,92	heating	16,958	33,6674	13,9426	275,72	
16,96	23,6055	-0,5377	337,49	16,96	35,9697	13,7219	310,99	heating	16,96	33,3926	10,9608	313,56	
16,962	23,2721	0,1991	322,53	16,962	37,3552	16,5261	291,16	heating	16,962	33,6274	11,1988	313,52	
16,964	23,0536	-1,3933	341,73	16,964	36,6429	17,7884	263,56	heating	16,964	33,3932	11,5121	305,87	
16,966	24,3741	9,0447	214,28	16,966	36,975	21,977	209,65	heating	16,966	33,3062	13,6266	275,09	
16,968	22,4874	4,9328	245,39	16,968	36,4765	21,4854	209,55	heating	16,968	33,257	11,724	301	
16,97	23,733	1,2841	313,8	16,97	36,4148	13,9851	313,53	heating	16,97	33,3634	10,2841	322,61	
16,972	23,4609	3,8456	274,19	16,972	35,8267	14,3664	299,98	heating	16,972	33,65	10,3027	326,36	
16,974	22,7502	-2,7022	355,79	16,974	35,2405	10,6473	343,78	heating	16,974	33,3261	11,9028	299,47	
16,976	23,2537	-5,485	401,72	16,976	36,2816	14,994	297,57	heating	16,976	33,4204	14,7592	260,86	
16,978	23,7689	2,1791	301,79	16,978	35,138	17,1153	251,93	heating	16,978	33,0525	15,5636	244,47	
16,98	23,2004	-1,4824	345,03	16,98	35,2597	13,3694	305,99	heating	16,98	33,0641	14,1619	264,22	
16,982	23,6377	4,5901	266,26	16,982	35,3379	9,5327	360,72	heating	16,982	33,1586	14,3204	263,33	
16,984	23,0031	6,9689	224,13	16,984	35,8704	10,1458	359,59	heating	16,984	33,1069	15,5641	245,22	heating
16,986	22,4062	-0,7609	323,84	16,986	35,1404	8,7828	368,44	heating	16,986	33,2823	16,2802	237,67	heating
16,988	23,5306	3,194	284,27	16,988	36,6022	14,6629	306,68		16,988	33,3386	17,4331	222,33	heating
16,99	21,9563	1,5806	284,82	16,99	35,6038	16,5102	266,9		16,99	33,4176	15,517	250,22	heating
16,992	22,8439	-2,5192	354,54	16,992	36,0496	12,3215	331,68		16,992	33,5225	16,0765	243,87	heating
16,994	22,7387	2,2501	286,4	16,994	37,416	16,6732	289,95		16,994	33,5129	16,5571	237,02	heating
16,996	21,9249	0,4087	300,76	16,996	37,8161	17,1823	288,43		16,996	33,2022	15,9241	241,52	heating
16,998	22,0518	-2,188	338,84	16,998	37,613	18,8561	262,19		16,998	33,3167	13,8967	271,46	heating
17	22,7223	4,7092	251,8	17	37,1276	18,2291	264,17		17	33,2402	12,258	293,3	heating
17,002	21,8827	0,4025	300,26	17,002	37,9057	16,4094	300,49		17,002	33,2093	10,0685	323,47	heating
17,004	22,4141	1,8376	287,63	17,004	37,3075	13,0008	339,77		17,004	33,2523	10,7068	315,15	heating
17,006	22,0468	4,5893	244,03	17,006	36,8305	18,8235	251,71		17,006	33,2572	10,3381	320,37	heating
17,008	21,096	-2,0736	323,88	17,008	35,9897	17,0253	265,09		17,008	33,4555	9,2803	337,93	heating
17,01	21,5979	-0,8262	313,46	17,01	36,9935	20,287	233,53		17,01	33,2676	8,3109	348,86	heating
17,012	20,257	-1,5191	304,4	17,012	35,8657	18,5478	242,08	heating	17,012	33,347	8,5018	347,3	heating
17,014	20,6534	-5,8307	370,21	17,014	36,6074	16,6819	278,53	heating	17,014	33,2593	9,4824	332,37	heating
17,016	20,7844	1,3477	271,7	17,016	36,955	15,123	305,18	heating	17,016	33,1375	10,0186	323,17	
17,018	20,0656	2,885	240,16	17,018	36,9837	15,8144	295,91	heating	17,018	32,9986	11,731	297,29	
17,02	20,1592	-0,7902	292,84	17,02	36,3128	13,7576	315,29	heating	17,02	33,1239	11,2574	305,66	
17,022	20,8725	1,6421	268,81	17,022	36,8376	15,6267	296,5	heating	17,022	33,1665	9,6787	328,32	
17,024	20,2017	-5,3787	357,58	17,024	36,0645	15,8683	282,31	heating	17,024	33,2691	8,5529	345,5	
17,026	20,8726	-4,9294	360,67	17,026	35,419	10,8724	343,13	heating	17,026	33,3268	9,734	329,79	
17,028	20,7332	-0,8206	301,29	17,028	36,4601	13,6819	318,41		17,028	33,6548	10,9099	317,94	
17,03	20,2418	-2,778	321,78	17,03	36,3506	10,8204	356,87		17,03	33,7921	13,6522	281,53	
17,032	21,1419	1,3602	276,52	17,032	35,9266	11,072	347,43		17,032	33,4854	12,6452	291,32	
17,034	20,2161	2,4663	248,12	17,034	35,8168	11,2665	343,18		17,034	33,7658	13,6793	280,78	
17,036	20,5565	-1,9396	314,46	17,036	36,7535	13,8597	320,02		17,036	33,6249	14,1737	271,9	
17,038	20,5274	0,3822	281,6	17,038	36,6184	11,6658	348,8		17,038	33,5489	14,5703	265,29	
17,04	19,8779	-1,5911	300,1	17,04	36,0838	17,9042	254,12		17,04	33,4176	13,5362	277,91	
17,042	20,0566	-3,6466	331,34	17,042	35,4706	16,0556	271,39		17,042	33,7699	13,7488	279,87	
17,044	20,7448	0,9149	277,19	17,044	35,9754	18,0087	251,15		17,044	33,4681	10,982	314,32	
17,046	20,0259	-4,477	342,51	17,046	35,2057	17,6337	245,63		17,046	33,6891	10,3444	326,32	
17,048	20,8494	-1,7642	316,1	17,048	35,6107	16,969	260,58		17,048	33,6768	10,775	320,13	
17,05	20,6063	3,7428	235,73	17,05	36,2718	19,0747	240,39		17,05	33,8229	12,2249	301,91	
17,052	20,0615	1,6815	256,92	17,052	36,1185	19,1625	237,02		17,052	33,505	13,3406	281,87	
17,054	20,841	4,3491	230,53	17,054	36,1252	18,2441	249,95	heating	17,054	33,4042	13,5148	278,02	

17,056	19,7334	1,0936	260,56	
17,058	20,3768	-5,4334	360,79	
17,06	20,1782	-0,6049	290,52	
17,062	19,4628	0,5739	264,04	
17,064	19,4062	-0,0328	271,73	
17,066	20,2156	6,5837	190,55	
17,068	19,3272	2,6337	233,35	heating
17,07	20,1226	2,5592	245,51	heating
17,072	20,0064	2,2397	248,35	heating
17,074	20,218	-2,099	311,96	heating
17,076	21,4006	1,9641	271,69	heating
17,078	21,1099	3,8749	240,92	heating
17,08	22,1402	0,8178	298,06	heating
17,082	22,2802	3,9671	255,99	heating
17,084	22,1769	2,5048	274,99	heating
17,086	22,3426	-0,8289	323,9	heating
17,088	22,9553	0,8173	309,46	heating
17,09	22,2249	-3,0532	353,35	heating
17,092	22,6412	-0,1092	318,02	heating
17,094	22,1576	2,9086	269,07	heating
17,096	21,9194	0,026	306,04	heating
17,098	22,6625	-0,2943	320,9	heating
17,1	22,4126	-2,4577	347,65	heating
17,102	23,4825	-3,083	371,35	
17,104	23,6451	3,089	287,34	
17,106	23,4373	3,9755	272,05	
17,108	23,4795	1,1777	311,75	
17,11	24,3723	2,5675	304,8	
17,112	23,7297	-2,0351	360,15	
17,114	24,3363	-0,2512	343,7	
17,116	23,835	4,382	271,92	
17,118	23,4344	3,0547	284,88	
17,12	23,8358	2,8087	293,93	
17,122	23,8296	2,3953	299,62	
17,124	24,4709	-1,7464	366,48	
17,126	24,6064	1,9917	316,12	
17,128	24,0394	3,3053	289,83	
17,13	23,806	3,0313	290,4	
17,132	24,0584	4,7194	270,33	
17,134	23,2837	-1,5521	347,17	
17,136	24,2186	0,3719	333,34	
17,138	23,8288	4,6895	267,54	
17,14	23,3925	0,033	326,53	
17,142	24,0964	0,2888	332,79	
17,144	23,7137	1,4663	310,99	
17,146	24,1616	-0,7418	348,11	
17,148	24,1706	4,1813	279,42	
17,15	23,4479	1,6143	305,2	
17,152	23,5071	-0,5573	336,38	
17,154	24,0533	4,9177	267,49	
17,156	22,8895	-1,498	340,9	
17,158	23,9033	-0,0675	335,08	
17,16	23,2536	3,2762	279,25	
17,162	23,0148	0,0124	321,54	
17,164	23,8128	3,3881	285,51	
17,166	23,3417	3,7114	274,4	

17,056	37,1591	20,3974	234,3	heating
17,058	36,2846	17,722	259,48	heating
17,06	36,8605	15,3236	301,05	heating
17,062	36,9712	16,9185	280,31	heating
17,064	37,0036	12,0832	348,35	heating
17,066	36,3791	14,1012	311,41	heating
17,068	35,9849	13,8058	310,03	heating
17,07	36,7068	13,6119	322,83	heating
17,072	36,8244	15,6748	295,64	heating
17,074	35,5905	15,8929	275,34	heating
17,076	35,7071	13,0668	316,48	heating
17,078	36,1355	16,1221	279,76	
17,08	35,0035	12,1078	320,05	heating
17,082	36,7009	15,5772	295,28	
17,084	37,0625	20,0277	238,12	
17,086	37,3012	18,6027	261,38	heating
17,088	37,3679	22,6453	205,8	heating
17,09	37,3266	21,0489	227,54	heating
17,092	36,3567	16,6302	275,75	heating
17,094	35,9466	16,3828	273,47	heating
17,096	35,8257	17,3738	257,93	heating
17,098	36,3863	15,1492	296,86	heating
17,1	35,6617	18,6123	238,33	heating
17,102	35,3606	12,7756	315,7	heating
17,104	37,4973	15,6985	304,71	heating
17,106	36,7798	14,5733	310,41	heating
17,108	36,9117	11,6302	353,4	heating
17,11	37,2187	13,7167	328,52	heating
17,112	37,1245	15,524	301,94	heating
17,114	36,04	13,2273	318,89	heating
17,116	37,3615	18,7527	260,12	heating
17,118	36,3757	17,1148	269,24	heating
17,12	36,9847	15,5218	300,02	heating
17,122	36,6916	19,0444	246,68	heating
17,124	37,3264	16,4246	292,18	heating
17,126	37,5428	20,4503	238,93	heating
17,128	36,9323	21,2633	219,03	heating
17,13	37,8593	19,5696	255,66	heating
17,132	37,3251	18,9662	256,63	heating
17,134	36,3773	19,7175	232,88	heating
17,136	35,9411	17,0862	263,56	heating
17,138	37,389	21,7604	218,47	heating
17,14	35,9261	19,1007	235,19	heating
17,142	36,6729	20,9693	219,51	heating
17,144	35,9757	20,9591	209,91	heating
17,146	36,1097	18,7095	243,23	heating
17,148	35,5797	17,7728	248,91	heating
17,15	36,5641	16,871	275,28	heating
17,152	36,5709	14,5071	308,42	heating
17,154	36,2861	12,8548	327,53	heating
17,156	37,0045	15,5111	300,45	heating
17,158	37,4421	14,0819	326,54	heating
17,16	37,1649	15,4439	303,63	heating
17,162	36,8453	13,9298	320,32	heating
17,164	37,8248	16,7649	294,39	heating
17,166	36,918	15,6385	297,46	heating

17,056	33,4098	12,1649	296,97	
17,058	33,3629	10,0196	326,3	
17,06	33,252	9,1632	336,73	
17,062	33,2384	10,1731	322,42	
17,064	33,2962	12,9373	284,59	
17,066	33,0635	12,9266	281,48	
17,068	33,3029	11,7712	300,98	
17,07	33,1787	7,9401	352,8	
17,072	33,2782	6,619	372,66	
17,074	32,8188	7,1914	358,23	
17,076	32,7664	10,7507	307,75	
17,078	32,5165	14,0133	258,65	
17,08	32,6518	15,6622	237,49	
17,082	32,6903	14,5546	253,51	
17,084	32,9376	13,375	273,46	
17,086	32,877	11,4344	299,74	
17,088	33,3243	10,6823	316,5	
17,09	33,4506	12,1235	298,12	
17,092	33,4064	14,3672	266,14	
17,094	33,2846	18,4953	206,73	
17,096	33,0328	18,7645	199,45	
17,098	32,8289	16,9201	222,38	heating
17,1	32,8149	13,7884	265,96	heating
17,102	32,8912	10,7649	309,29	heating
17,104	32,9996	9,2352	332,19	heating
17,106	33,2114	11,8842	298,12	heating
17,108	33,1157	11,7944	298,04	heating
17,11	33,7105	13,2087	286,58	heating
17,112	33,2643	13,6612	274,02	heating
17,114	33,2843	12,8952	285,01	heating
17,116	33,1878	12,1244	294,44	heating
17,118	33,3834	10,8681	314,73	heating
17,12	33,152	8,8278	340,02	heating
17,122	33,4033	9,6235	332,41	
17,124	33,2178	11,907	297,89	
17,126	33,0652	12,3779	289,18	
17,128	33,1152	12,3627	290,09	
17,13	33,4135	10,4072	321,59	
17,132	33,6063	9,5442	336,35	
17,134	33,1659	8,8185	340,34	
17,136	33,2537	10,7736	314,24	
17,138	32,971	12,4906	286,28	
17,14	33,1374	13,905	268,84	
17,142	33,0192	13,8009	268,64	
17,144	33,4012	13,5733	277,16	
17,146	33,2542	13,1111	281,57	
17,148	33,4729	15,6772	248,76	
17,15	33,179	15,7986	242,95	
17,152	33,5457	15,3506	254,34	heating
17,154	33,2938	14,4816	262,97	heating
17,156	33,2104	12,899	283,92	heating
17,158	33,3297	14,2047	267,34	heating
17,16	33,1534	13,2205	278,63	heating
17,162	33,3652	10,7314	316,39	heating
17,164	33,7181	10,5705	323,57	heating
17,166	33,9095	11,7184	310,2	heating

17,168	23,8315	-0,4051	338,79		17,168	36,4385	18,5975	249,39	heating	17,168	33,7165	11,3314	312,91	heating
17,17	23,8676	4,9957	263,8		17,17	36,1714	15,1867	293,33	heating	17,17	33,7498	13,841	278,3	
17,172	23,094	3,4799	274,18		17,172	37,4075	16,1299	297,43	heating	17,172	33,1416	11,5102	302,37	heating
17,174	23,0848	-0,9207	335,56		17,174	36,6136	18,4	254,6	heating	17,174	33,3988	11,1155	311,49	
17,176	23,5228	0,5673	320,88		17,176	37,316	20,8238	230,54	heating	17,176	32,9826	11,3354	302,6	
17,178	22,7443	-2,6335	354,74		17,178	37,6023	23,3186	199,67	heating	17,178	33,0163	11,0374	307,23	
17,18	23,5448	3,2713	283,39		17,18	37,4776	22,1661	214,03	heating	17,18	32,9881	11,8638	295,29	
17,182	22,8152	7,4535	214,73		17,182	36,9473	17,9573	265,45	heating	17,182	33,1998	12,4747	289,71	
17,184	22,364	1,7964	287,51		17,184	37,9689	15,761	310,43	heating	17,184	33,2012	10,0714	323,32	
17,186	22,8095	0,2082	315,93		17,186	36,8357	17,0017	277,25	heating	17,186	33,6113	10,6524	320,93	
17,188	22,2465	0,2437	307,57		17,188	36,1251	16,0075	281,21	heating	17,188	33,1265	11,0989	307,91	
17,19	22,6607	1,3106	298,44		17,19	36,7409	21,7135	210,06	heating	17,19	33,3705	12,419	292,87	
17,192	22,4496	7,2695	212,2		17,192	36,511	17,7994	261,56	heating	17,192	33,3002	15,9126	243,05	
17,194	21,9796	3,2895	261,26		17,194	36,4258	17,2928	267,45	heating	17,194	32,8727	14,4816	257,08	
17,196	22,2187	-1,1717	326,96		17,196	36,0456	13,1745	319,7	heating	17,196	32,8675	13,8587	265,72	
17,198	22,7791	1,6644	295,15		17,198	37,7266	13,5619	337,79	heating	17,198	32,6662	14,2678	257,18	
17,2	21,8393	-2,337	337,95		17,2	37,08	14,0448	322	heating	17,2	32,6174	12,3556	283,23	heating
17,202	22,8153	0,0761	317,86		17,202	37,3313	17,2096	281,27	heating	17,202	32,9882	12,2568	289,79	heating
17,204	22,2583	3,6806	259,69		17,204	37,0012	14,8629	309,46	heating	17,204	33,3056	11,5054	304,73	heating
17,206	22,221	1,2708	292,85		17,206	37,7382	15,9485	304,59	heating	17,206	33,1237	7,0816	364,03	heating
17,208	22,6878	0,752	306,63		17,208	35,5307	14,1665	298,64	heating	17,208	33,6253	10,8078	318,95	
17,21	22,8089	-0,4531	325,17		17,21	36,3698	14,5633	304,82	heating	17,21	32,809	13,6165	268,28	
17,212	23,1095	-3,748	375,43		17,212	36,1543	17,1578	265,54	heating	17,212	33,2071	15,7705	243,74	
17,214	23,5125	3,3898	281,28		17,214	35,9719	13,3952	315,59	heating	17,214	32,8231	15,7326	238,9	
17,216	22,924	4,6345	255,66		17,216	36,5469	15,4112	295,45	heating	17,216	32,911	10,6669	310,94	heating
17,218	22,9469	1,8628	294,72		17,218	37,4597	17,9456	272,78	heating	17,218	33,4057	6,3535	378,15	heating
17,22	23,0101	0,0728	320,63		17,22	36,9656	19,6584	241,93	heating	17,22	33,7192	7,8191	362,04	
17,222	22,1563	-5,8169	391,02		17,222	36,3665	19,6262	234,01	heating	17,222	33,1997	9,656	329,11	
17,224	23,0151	-3,1632	365,93		17,224	37,5384	22,7024	207,39	heating	17,224	33,4779	14,3082	267,96	
17,226	22,2757	1,6576	288,21		17,226	36,9877	15,6468	298,31	heating	17,226	33,3118	16,4606	235,55	
17,228	22,3624	-0,207	315,49		17,228	36,8875	17,1211	276,31	heating	17,228	33,2474	12,0458	296,37	
17,23	23,0041	1,2513	304,07		17,23	35,7996	16,0447	276,14	heating	17,23	33,7397	12,7416	293,52	
17,232	23,1539	-0,2515	327,17		17,232	37,4005	18,138	269,26	heating	17,232	33,1216	12,255	291,68	
17,234	23,3949	-5,2542	400,47		17,234	36,2971	18,2567	252,18	heating	17,234	33,0379	11,5552	300,3	
17,236	24,1124	-0,7688	347,8	cooling	17,236	36,6879	18,7872	250,23	heating	17,236	32,825	11,5117	297,93	
17,238	23,4277	-1,7289	351,65	cooling	17,238	36,763	16,4171	284,4	heating	17,238	33,0409	10,4493	315,8	
17,24	23,9857	-0,6258	344,03	cooling	17,24	37,8715	17,5579	283,95	heating	17,24	33,2523	9,3521	334,09	
17,242	23,8676	1,6032	311,22	cooling	17,242	36,3186	15,2433	294,6	heating	17,242	33,4689	14,3927	266,66	
17,244	23,1901	-5,3533	398,99	cooling	17,244	37,3809	15,6661	303,54	heating	17,244	32,689	12,5115	282,05	
17,246	23,9478	-5,022	404,95	cooling	17,246	36,9248	17,4412	272,35	heating	17,246	33,2152	12,1035	295,11	
17,248	23,4111	-0,7859	338,24	cooling	17,248	36,1948	10,925	353,23	heating	17,248	32,9936	10,9255	308,48	
17,25	23,5365	-1,4437	349,19	cooling	17,25	36,8678	13,2041	330,78	heating	17,25	33,0446	7,6096	355,54	
17,252	24,3808	5,6147	262,32	cooling	17,252	37,3024	13,9796	326,02	heating	17,252	33,5114	9,7424	332,26	
17,254	23,9817	5,1326	263,48	cooling	17,254	37,2398	15,6601	301,65	heating	17,254	33,2143	14,1507	266,48	
17,256	24,1899	-2,2666	369,82	cooling	17,256	36,7567	13,8017	320,88	heating	17,256	33,0918	16,0238	238,59	
17,258	24,5752	0,9907	329,68	cooling	17,258	38,3064	15,1127	324,21	heating	17,258	33,2918	18,0597	212,92	
17,26	23,7696	-2,1581	362,43	cooling	17,26	37,9819	8,5979	410,75	heating	17,26	33,2449	16,4173	235,23	
17,262	24,379	-1,334	359,43	cooling	17,262	38,8214	15,4984	326,02		17,262	33,4003	14,1853	268,6	heating
17,264	24,6323	6,9956	246,53	cooling	17,264	37,8751	15,8347	308,09		17,264	33,4832	17,9828	216,67	heating
17,266	23,8093	5,6047	254,47	cooling	17,266	40,062	18,9831	294,65		17,266	33,086	15,651	243,71	heating
17,268	24,678	5,2452	271,64	cooling	17,268	38,8221	17,8554	293,08		17,268	33,4646	13,9925	272,19	heating
17,27	23,7424	4,6413	267	cooling	17,27	39,2719	17,1298	309,51		17,27	33,5141	12,5316	293,3	heating
17,272	23,8828	-2,3268	366,37	cooling	17,272	38,9501	14,8022	337,55		17,272	33,5858	11,9404	302,57	heating
17,274	24,6626	-1,2029	361,56	cooling	17,274	39,7744	19,4011	284,79		17,274	33,7644	14,576	268,23	heating
17,276	24,6264	-1,1338	360,09	cooling	17,276	38,1317	19,4342	261,36		17,276	33,3209	14,9661	256,57	heating
17,278	24,2615	-3,5403	388,63	cooling	17,278	38,8362	18,7262	281,11		17,278	33,4146	10,2224	324,19	heating

17,28	24,5662	1,933	316,38	cooling	17,28	38,1665	18,5145	274,71		17,28	34,1638	10,9221	324,88	heating
17,282	23,7112	1,18	314,95	cooling	17,282	37,3248	11,7576	357,39		17,282	33,9009	9,9579	334,69	heating
17,284	24,0231	1,317	317,4	cooling	17,284	38,5685	15,5385	321,92		17,284	34,412	12,1704	310,91	heating
17,286	23,9605	3,3016	288,78	cooling	17,286	38,6816	14,389	339,58		17,286	34,3617	15,8266	259,09	heating
17,288	23,4825	-2,736	366,49	cooling	17,288	38,9935	15,8994	322,82		17,288	34,226	13,3634	291,63	heating
17,29	23,7436	-4,5689	395,77	cooling	17,29	38,3106	12,2907	363,72		17,29	34,3085	12,9437	298,65	heating
17,292	22,59	-0,8161	327,18	cooling	17,292	40,1279	15,8809	338,94		17,292	34,3768	13,0244	298,48	heating
17,294	21,8017	-0,0293	305,17	cooling	17,294	38,7624	10,4336	395,99		17,294	34,1649	11,0604	322,97	heating
17,296	21,9137	3,7541	253,84	cooling	17,296	39,0293	18,4231	288,04		17,296	34,4516	13,3962	294,32	heating
17,298	21,2991	2,9942	255,87	cooling	17,298	37,4069	17,2474	281,8		17,298	33,8815	12,4065	300,19	heating
17,3	21,2591	-0,6509	306,27	cooling	17,3	38,717	19,4072	269,92		17,3	34,3408	8,9914	354,35	
17,302	21,661	1,1288	287,01	cooling	17,302	37,0125	17,6465	270,71		17,302	34,419	11,5041	320,32	
17,304	21,6752	1,2258	285,85	cooling	17,304	37,4991	17,2703	282,77		17,304	33,9082	10,493	327,31	
17,306	21,977	0,7082	297,31	cooling	17,306	37,6465	17,7959	277,48		17,306	34,4104	13,6146	290,69	
17,308	22,5231	2,5114	279,73	cooling	17,308	37,9883	20,4021	245,83		17,308	34,3552	16,4389	250,44	
17,31	22,7418	-0,2083	320,81	cooling	17,31	37,3739	21,1481	226,81		17,31	34,0743	13,1414	292,61	
17,312	22,9205	-0,0714	321,39	cooling	17,312	38,2939	21,256	238,17		17,312	34,4177	13,9798	285,69	
17,314	22,5261	1,9737	287,29	cooling	17,314	38,0842	21,2571	235,22		17,314	34,0681	13,9546	281,16	
17,316	22,1356	1,1463	293,4	cooling	17,316	37,3289	13,8699	327,92		17,316	33,9375	12,1247	304,91	
17,318	22,7569	3,4952	269,25	cooling	17,318	38,7684	20,1206	260,67		17,318	34,3539	16,6963	246,83	
17,32	22,1605	2,6093	273,3	cooling	17,32	38,202	16,6451	301,33		17,32	33,6733	14,5332	267,55	
17,322	22,2708	-1,303	329,53	cooling	17,322	38,3884	17,714	289		17,322	34,3799	12,3898	307,39	heating
17,324	22,225	-1,8477	336,5	cooling	17,324	37,4396	13,6432	332,64		17,324	34,1734	14,8278	270,42	
17,326	21,9742	-0,9933	321,05	cooling	17,326	39,1144	15,537	329,58		17,326	33,7993	11,7797	307,8	heating
17,328	21,9563	-0,2081	309,83	cooling	17,328	38,3426	12,61	359,7		17,328	34,1471	13,979	281,92	
17,33	22,1058	-0,2589	312,63	cooling	17,33	38,8278	18,2613	287,49		17,33	34,2405	15,9964	255,02	heating
17,332	22,6969	-2,8065	356,5	cooling	17,332	38,1414	13,5899	343,19		17,332	34,0265	10,7781	324,98	heating
17,334	22,7506	0,1855	315,43	cooling	17,334	39,4541	12,3814	378,44		17,334	34,8592	12,3198	315,07	heating
17,336	22,77	4,5932	254,09	cooling	17,336	38,2417	9,7327	398,51		17,336	34,1523	13,2722	291,87	
17,338	22,2725	4,592	247,15	cooling	17,338	39,1013	10,3615	401,74		17,338	34,2907	11,1462	323,53	
17,34	22,7029	2,0637	288,51		17,34	39,4797	16,6466	319,17	cooling	17,34	34,7292	17,2105	244,89	
17,342	21,9255	-5,4077	382,08		17,342	39,0686	15,973	322,84	cooling	17,342	33,9853	15,0034	265,34	
17,344	22,6218	-7,5526	421,79	cooling	17,344	39,2053	17,7623	299,74	cooling	17,344	34,4502	11,0945	326,48	heating
17,346	22,3833	-2,986	354,62	cooling	17,346	39,5161	14,4054	351,01	cooling	17,346	34,4541	11,3482	322,99	
17,348	22,1102	-0,779	319,96	cooling	17,348	39,0765	15,6231	327,84	cooling	17,348	34,1811	8,4693	359,41	
17,35	22,0385	0,4708	301,48	cooling	17,35	38,3403	13,4967	347,28	cooling	17,35	34,5203	9,7066	346,86	
17,352	22,5466	3,4979	266,27	cooling	17,352	39,9535	23,6954	227,26	cooling	17,352	34,5541	13,2691	297,53	
17,354	22,6815	-1,4973	337,98	cooling	17,354	39,5044	19,9243	273,7	cooling	17,354	34,3032	10,7172	329,7	
17,356	22,7073	-2,6214	354,06	cooling	17,356	39,7481	24,3283	215,55		17,356	35,0142	12,7507	311,21	
17,358	22,5856	-1,3151	334,1	cooling	17,358	38,8709	19,1591	275,54		17,358	34,499	12,3673	309,37	
17,36	22,1772	-2,5852	346,14	cooling	17,36	40,8089	21,5384	269,37		17,36	34,7908	11,7493	322,09	
17,362	22,5845	-0,1499	317,79	cooling	17,362	39,9425	19,1293	290,94		17,362	34,8526	15,3443	272,7	
17,364	21,8569	0,1796	303,02	cooling	17,364	40,1202	20,4938	274,35		17,364	34,185	12,7275	299,94	
17,366	22,4906	0,896	301,86	cooling	17,366	39,4141	15,6437	332,28		17,366	34,4116	10,8776	328,97	
17,368	22,5001	3,3641	267,49	cooling	17,368	40,1182	17,7458	312,73		17,368	34,1776	13,361	290,99	
17,37	22,097	3,2614	263,29	cooling	17,37	38,271	17,928	284,36		17,37	33,9109	13,7452	281,89	
17,372	21,9979	0,1728	305,08	cooling	17,372	38,8487	19,5478	269,8		17,372	33,8722	14,8935	265,29	
17,374	22,2902	-2,0579	340,35	cooling	17,374	38,0612	19,2139	263,46		17,374	34,0015	17,0398	237,1	
17,376	22,098	-6,6179	401,41	cooling	17,376	37,9968	12,5799	355,29		17,376	33,8049	11,9025	306,16	
17,378	22,1496	-3,4767	358,22	cooling	17,378	37,7442	12,5975	351,51		17,378	34,2856	10,1889	336,84	
17,38	22,0961	0,8638	296,8	cooling	17,38	38,5297	11,4111	379,08		17,38	34,0436	8,1129	362,47	
17,382	21,7648	1,311	285,91	cooling	17,382	38,4223	17,915	286,66		17,382	34,4195	10,1478	339,28	
17,384	22,269	3,6262	260,6	cooling	17,384	38,2645	18,3364	278,57		17,384	34,4832	12,3673	309,15	
17,386	21,7093	1,1011	288,07	cooling	17,386	39,4347	23,436	223,64		17,386	34,1533	10,6411	328,67	
17,388	22,6488	-2,6606	353,79	cooling	17,388	39,6397	17,461	310,03		17,388	34,4829	9,1476	354,15	
17,39	22,6524	-0,4326	322,69	cooling	17,39	39,7297	19,7968	278,63		17,39	34,257	10,0723	338,07	

17,392	22,1927	1,048	295,57	cooling	17,392	38,75	16,411	312,27		17,392	34,1193	9,3353	346,44	
17,394	21,9546	-2,0794	335,96	cooling	17,394	40,0058	21,2574	262,07		17,394	34,2565	10,6371	330,16	
17,396	22,5767	-0,2006	318,39	cooling	17,396	38,4292	20,3731	252,4		17,396	34,366	11,8494	314,75	cooling
17,398	22,0137	-0,5539	315,46	cooling	17,398	38,4334	21,1668	241,36		17,398	34,4108	9,959	341,8	cooling
17,4	22,1355	2,4261	275,51	cooling	17,4	37,5321	18,7899	261,99		17,4	34,6564	11,4198	324,81	cooling
17,402	22,0422	6,4055	218,58	cooling	17,402	38,4022	20,2302	254,02		17,402	34,5622	10,8553	331,39	cooling
17,404	21,6109	4,4297	240,17	cooling	17,404	36,9597	18,9415	251,87		17,404	34,6176	12,3993	310,58	cooling
17,406	21,2836	-2,5181	332,71		17,406	38,4173	18,5455	277,78	heating	17,406	34,5613	11,9938	315,46	cooling
17,408	21,6488	-4,9589	371,94		17,408	38,0579	19,2338	263,13	heating	17,408	34,2845	8,6763	357,96	cooling
17,41	21,7798	-5,1515	376,46	cooling	17,41	38,4396	13,3994	350,03	heating	17,41	34,6574	5,8082	403,27	cooling
17,412	21,5117	-1,4696	321,24	cooling	17,412	38,9467	17,7193	296,73		17,412	34,1073	7,2103	375,98	cooling
17,414	21,2113	3,2276	251,38	cooling	17,414	39,4149	16,9218	314,42		17,414	34,1291	11,4033	317,67	cooling
17,416	21,3293	5,0214	227,96	cooling	17,416	38,9983	17,8638	295,43		17,416	34,0669	16,9403	239,41	cooling
17,418	21,2608	1,4948	276,3		17,418	38,8609	13,7992	350,32		17,418	33,9788	16,6174	242,69	cooling
17,42	21,1671	-1,6026	318,29		17,42	39,8723	16,1484	331,62		17,42	34,1378	13,7542	284,93	cooling
17,422	21,3253	-2,8104	337,38		17,422	39,3456	10,8395	398,47		17,422	34,264	14,1228	281,54	cooling
17,424	21,3117	-2,7114	335,81		17,424	39,7929	16,9908	318,74		17,424	34,2005	14,1905	279,71	cooling
17,426	21,1402	-0,3458	300,34	cooling	17,426	38,578	15,4687	323,03		17,426	33,908	15,0706	263,32	cooling
17,428	21,1245	2,8891	254,9	cooling	17,428	40,4893	18,2077	311,46		17,428	33,9078	14,9114	265,54	
17,43	21,5822	2,8134	262,36		17,43	39,0327	18,243	290,61		17,43	33,209	11,154	308,3	
17,432	21,6522	-0,134	304,54		17,432	39,6633	19,0063	288,75		17,432	33,5107	10,5849	320,47	
17,434	21,261	-1,1773	313,65		17,434	38,6257	20,7751	249,53		17,434	32,7811	10,3028	314,21	
17,436	21,2637	0,1597	295		17,436	38,8581	22,1271	233,87		17,436	33,2313	9,0319	338,27	
17,438	21,4572	3,669	248,65		17,438	37,2244	18,762	258,08		17,438	33,411	12,2257	296,14	
17,44	21,3247	5,4233	222,28		17,44	38,8339	17,6651	295,91		17,44	33,555	13,7776	276,46	
17,442	21,6165	5,3312	227,64		17,442	37,7414	18,6307	267,14		17,442	33,5976	11,9872	302,08	
17,444	21,9221	1,9623	279,01		17,444	38,2401	15,1942	322,15		17,444	34,0968	12,5746	300,85	
17,446	21,8514	0,1152	303,84		17,446	38,9889	22,4275	231,5		17,446	33,8523	10,6608	324,18	
17,448	21,5229	1,1259	285,12		17,448	39,3901	19,3012	280,81		17,448	34,0773	10,7754	325,73	
17,45	21,0693	0,3366	289,81		17,45	39,3978	19,453	278,8		17,45	34,224	15,3349	264,04	
17,452	21,905	2,7083	268,34		17,452	39,2019	15,8834	325,96		17,452	33,4197	12,3184	294,96	
17,454	21,8852	3,9632	250,52		17,454	40,5659	17,3291	324,82		17,454	33,8801	9,6127	339,22	
17,456	21,2708	2,2163	266,35		17,456	40,0832	13,9338	365,53		17,456	33,5984	8,6226	349,12	
17,458	21,313	1,5178	276,71		17,458	40,3477	20,3483	279,56		17,458	33,9789	8,3318	358,51	
17,46	20,9177	-1,0231	306,7		17,46	38,7665	13,5604	352,34		17,46	33,895	11,5415	312,47	cooling
17,462	20,6557	-5,4499	364,92		17,462	40,5486	15,1062	355,65		17,462	33,8091	13,1694	288,51	cooling
17,464	21,1541	-2,7996	334,84		17,464	38,1606	13,4288	345,71		17,464	33,4298	8,8642	343,39	cooling
17,466	21,3565	-1,9053	325,17		17,466	39,233	16,2594	321,14		17,466	33,9412	8,869	350,47	cooling
17,468	21,4476	-0,5567	307,59		17,468	38,1737	19,1319	266,18		17,468	33,4339	9,2317	338,31	cooling
17,47	21,2993	3,7135	245,82		17,47	38,4016	19,1598	268,97		17,47	33,6768	11,402	311,37	cooling
17,472	20,4667	-1,1249	301,82		17,472	37,6919	18,5271	267,9		17,472	33,7183	16,6646	238,39	cooling
17,474	21,4012	-3,3471	345,94		17,474	39,2119	20,2604	264,91		17,474	33,0322	14,2927	261,95	cooling
17,476	21,2625	-0,153	299,36		17,476	38,6966	22,9665	219,88		17,476	33,6675	13,9336	275,85	
17,478	20,6515	-4,2036	347,44		17,478	39,1841	20,5378	260,65		17,478	33,5243	14,9968	258,99	
17,48	21,127	-0,0703	296,31		17,48	40,8048	26,3929	201,46		17,48	33,4243	12,4868	292,68	
17,482	20,7374	3,1828	245,39		17,482	40,8	20,4458	284,52	heating	17,482	33,4818	12,0746	299,24	
17,484	20,0057	-4,5923	343,84		17,484	41,2067	21,488	275,64	heating	17,484	33,3614	14,3724	265,44	
17,486	21,1059	-1,8435	320,8		17,486	40,1647	16,8234	326,28	heating	17,486	32,856	11,6086	297,01	
17,488	20,8706	-0,3281	296,33		17,488	41,0041	18,1345	319,68	heating	17,488	33,5683	15,1066	258,07	
17,49	21,2169	-2,6791	334,03		17,49	39,1	12,6633	369,55		17,49	33,3148	13,9467	270,74	
17,492	21,0649	4,1369	236,63		17,492	39,3982	15,4404	334,89		17,492	33,7956	11,6227	309,94	
17,494	20,4405	3,4667	237,27		17,494	38,2037	11,0426	379,67		17,494	33,9432	11,6817	311,18	
17,496	20,8937	-1,8954	318,56		17,496	39,8784	13,6122	367,16		17,496	33,4787	9,3065	337,89	
17,498	21,2461	1,2065	280,12		17,498	38,911	13,4965	355,26		17,498	33,9937	11,5023	314,4	
17,5	20,2347	-4,003	338,81		17,5	40,5846	13,6243	376,86		17,5	33,7309	14,953	262,49	
17,502	21,1781	-2,5653	331,9		17,502	40,3374	16,5644	332,31		17,502	33,4502	11,5862	305,63	

17,504	20,8108	3,051	248,26		17,504	40,4256	15,1952	352,68		17,504	34,0174	10,3447	330,91	
17,506	19,8122	-2,1592	307,13		17,506	40,2527	15,967	339,48	cooling	17,506	33,3293	10,1182	324,46	
17,508	21,249	1,2652	279,34		17,508	40,842	17,2218	330,18	cooling	17,508	33,2622	7,4648	360,61	
17,51	21,0734	3,5608	244,8		17,51	39,6308	21,7315	250,21	cooling	17,51	33,5752	12,6635	292,32	
17,512	20,8955	-1,8522	317,98		17,512	38,8948	17,2197	302,99	cooling	17,512	33,5532	13,4777	280,63	
17,514	20,9121	1,6766	268,88		17,514	40,1459	22,128	251,86	cooling	17,514	33,6082	12,1912	299,38	
17,516	20,2933	-0,5102	290,8		17,516	39,4264	14,306	351,15		17,516	33,9348	14,8649	266,57	
17,518	20,4492	-7,0284	384,1		17,518	40,378	16,2481	337,3		17,518	33,4298	14,0767	270,53	
17,52	21,1274	0,5592	287,51		17,52	40,1129	15,5395	343,5	cooling	17,52	33,7788	15,0198	262,22	
17,522	20,4946	-0,2069	289,38		17,522	41,3581	17,92	327,63	cooling	17,522	33,5459	17,6084	222,78	
17,524	21,0464	0,0136	294,01		17,524	40,0281	15,2812	345,93	cooling	17,524	33,2704	14,3672	264,24	
17,526	20,8463	4,7973	224,34		17,526	40,6809	19,5933	294,77	cooling	17,526	34,172	14,3771	276,7	
17,528	19,8529	-0,8899	289,95		17,528	39,4288	16,1893	324,85	cooling	17,528	33,3007	15,4376	249,7	
17,53	21,1269	-1,2532	312,84		17,53	40,5012	18,9113	301,79	cooling	17,53	33,5561	14,9978	259,42	
17,532	20,705	1,4042	269,8		17,532	39,1899	19,5348	274,75	cooling	17,532	33,6232	17,8791	220,08	
17,534	20,4971	-1,7943	311,6		17,534	40,25	16,5293	331,58	cooling	17,534	34,0055	17,0019	237,68	heating
17,536	20,8347	1,839	265,53		17,536	39,4721	18,4039	294,5	cooling	17,536	33,2755	10,6213	316,67	heating
17,538	20,6951	5,4179	213,55		17,538	38,9732	14,7833	338,14	cooling	17,538	34,2554	11,6609	315,84	heating
17,54	20,4946	1,3661	267,39	heating	17,54	39,6981	15,2018	342,42	cooling	17,54	33,5261	9,0522	342,11	heating
17,542	21,4573	4,7377	233,71	heating	17,542	40,8009	15,1014	359,24	cooling	17,542	33,9182	7,7603	365,65	heating
17,544	21,4265	1,8932	273,05	heating	17,544	40,1708	18,8674	297,79	cooling	17,544	33,6364	11,2012	313,61	
17,546	21,6017	-0,2443	305,37	heating	17,546	39,8929	16,1797	331,47	cooling	17,546	33,5995	10,5761	321,83	
17,548	21,5199	1,2387	283,5	heating	17,548	40,8259	22,1018	261,74	cooling	17,548	34,0631	9,9314	337,33	
17,55	20,6108	-4,2933	348,12	heating	17,55	39,4139	14,6995	345,47	cooling	17,55	33,4266	11,2557	309,92	
17,552	21,9171	-3,1714	350,7	heating	17,552	39,9688	18,4458	300,86	cooling	17,552	33,5039	10,0286	328,15	
17,554	21,1185	0,7947	284,1		17,554	39,155	17,6507	300,6	cooling	17,554	33,5214	10,6726	319,39	
17,556	21,3727	-0,6811	308,28		17,556	39,9767	20,5931	270,95	cooling	17,556	33,8208	13,2467	287,59	
17,558	21,6195	1,6511	279,13		17,558	38,4866	21,9436	231,25	cooling	17,558	33,1026	12,5206	287,71	
17,56	21,7841	1,6736	281,11		17,56	39,1189	23,6113	216,77		17,56	33,9133	13,6537	283,2	
17,562	21,5434	-4,2014	359,87		17,562	38,2192	17,3148	292,21		17,562	33,2872	13,1549	281,42	
17,564	22,9865	2,3372	288,65		17,564	39,0493	14,8062	338,88		17,564	33,796	13,3912	285,23	
17,566	22,6834	2,1425	287,13		17,566	38,1735	12,5466	358,23		17,566	33,5584	13,3554	282,41	
17,568	22,7995	1,953	291,4		17,568	39,6992	12,8425	375,42		17,568	33,9615	10,9914	321,09	
17,57	22,6713	4,2652	257,29		17,57	38,9585	17,3422	302,16	cooling	17,57	33,9319	9,5553	340,75	
17,572	21,9096	0,558	298,46		17,572	39,1487	13,6375	356,61	cooling	17,572	33,8976	11,2896	316,03	
17,574	22,8038	0,5488	311,09		17,574	40,5412	16,1943	340,33	cooling	17,574	33,7098	12,1929	300,77	
17,576	21,9502	2,9792	265,19		17,576	40,4404	11,7177	401,5	cooling	17,576	34,02	15,1001	264,47	
17,578	22,1374	-2,4814	344,13		17,578	39,7655	13,1648	371,84	cooling	17,578	33,8868	16,2206	246,95	
17,58	21,7874	-1,317	322,97		17,58	38,8972	11,7728	379,16	cooling	17,58	33,7292	15,0505	261,1	
17,582	21,4267	0,5114	292,37		17,582	39,8873	16,0976	332,54	cooling	17,582	34,2293	16,9852	241,05	
17,584	20,377	-4,1796	343,27		17,584	38,7888	11,6136	379,87	cooling	17,584	33,6393	16,1312	244,74	
17,586	21,7232	0,5701	295,69		17,586	39,787	15,9132	333,72	cooling	17,586	34,0754	16,3034	248,43	
17,588	20,8779	-0,9416	305		17,588	38,8643	12,7689	364,77	cooling	17,588	33,555	15,8644	247,29	heating
17,59	21,3104	-0,5433	305,48		17,59	40,1351	14,5185	358,08	cooling	17,59	34,1229	16,5064	246,25	heating
17,592	21,3972	2,1136	269,56		17,592	38,7604	14,1006	344,71	cooling	17,592	33,6301	14,5195	267,14	heating
17,594	20,8196	-1,5109	312,15		17,594	40,0633	15,4261	344,39	cooling	17,594	34,1534	15,7702	256,97	heating
17,596	21,5792	-3,7408	353,94		17,596	39,1378	14,9384	338,27	cooling	17,596	33,4793	13,559	278,46	heating
17,598	21,2768	-0,6412	306,38		17,598	39,4296	14,391	350	cooling	17,598	34,061	14,4701	273,85	heating
17,6	22,074	0,9759	294,92		17,6	38,8882	15,2324	330,67	cooling	17,6	33,5219	13,4111	281,12	heating
17,602	21,8168	5,3204	230,6		17,602	39,8009	15,3586	341,67	cooling	17,602	33,7291	13,0516	289,04	heating
17,604	21,9271	5,9943	222,72		17,604	38,8653	18,8331	280,02	cooling	17,604	33,7556	13,7778	279,26	heating
17,606	21,2338	-1,9529	324,12		17,606	38,8409	17,0661	304,38	cooling	17,606	33,9015	14,6857	268,61	heating
17,608	22,6441	-2,2906	348,55		17,608	40,057	20,3863	274,97	cooling	17,608	33,9795	14,4075	273,59	heating
17,61	21,5491	-6,142	387,08		17,61	39,872	13,7562	365,06	cooling	17,61	33,7548	14,6561	266,97	heating
17,612	22,0456	-4,4664	370,6		17,612	40,0953	16,6334	327,96	cooling	17,612	33,9187	13,7595	281,8	heating
17,614	22,0642	0,8811	296,11		17,614	39,4599	13,9099	357,15	cooling	17,614	33,6773	10,5149	323,78	heating

17,616	21,5775	1,8494	275,77		17,616	40,7329	16,5763	337,67	cooling	17,616	34,3347	11,2399	322,83	heating
17,618	21,8297	0,1604	302,91		17,618	39,2561	12,9335	367,95	cooling	17,618	33,8444	10,513	326,14	heating
17,62	22,2572	5,2227	238,12		17,62	39,765	16,1962	329,46	cooling	17,62	34,4038	14,3532	280,28	heating
17,622	22,6109	0,8803	303,76		17,622	38,325	13,4496	347,72	cooling	17,622	33,8446	13,8867	278,98	heating
17,624	22,4986	-0,1701	316,88		17,624	38,8081	16,985	305,05	cooling	17,624	34,0783	14,2159	277,65	heating
17,626	22,8933	1,2959	301,9		17,626	37,1851	16,6458	287,11	cooling	17,626	34,0953	13,1364	292,97	heating
17,628	22,1995	-3,5373	359,76		17,628	38,6755	17,0741	301,95	cooling	17,628	34,3018	14,0942	282,47	heating
17,63	23,3926	-0,2989	331,17		17,63	37,9608	18,4553	272,66	cooling	17,63	33,9482	12,3799	301,49	heating
17,632	22,399	2,1264	283,38		17,632	38,2296	17,0581	295,95	cooling	17,632	33,7981	14,214	273,76	heating
17,634	22,9897	2,2948	289,29		17,634	38,8419	19,0704	276,38	cooling	17,634	33,8122	12,2014	302,09	heating
17,636	23,0017	3,3188	275,14		17,636	39,593	17,8471	303,98	cooling	17,636	33,7655	10,681	322,69	heating
17,638	22,6708	2,0305	288,52		17,638	38,7323	18,0602	288,96	cooling	17,638	34,2612	14,6254	274,48	heating
17,64	22,7974	-1,73	342,86		17,64	38,5784	16,0772	314,53	cooling	17,64	33,8572	15,1714	261,2	heating
17,642	23,3318	1,9809	298,45		17,642	39,2873	20,0858	268,41	cooling	17,642	34,3919	16,4094	251,37	heating
17,644	22,8769	-1,8769	346,02		17,644	38,2324	13,6585	343,51	cooling	17,644	33,967	14,0248	278,76	heating
17,646	22,7409	-1,2145	334,86		17,646	38,711	17,4782	296,8	cooling	17,646	34,0151	10,4355	329,61	heating
17,648	22,7334	1,7636	293,13		17,648	38,0991	14,7682	326,13	cooling	17,648	34,3432	9,0874	353,04	heating
17,65	21,8144	-2,0691	333,86		17,65	39,5189	15,6399	333,79	cooling	17,65	34,4557	13,7593	289,3	heating
17,652	22,3491	1,6097	289,91		17,652	38,4838	16,4136	308,51	cooling	17,652	34,0925	14,0295	280,45	heating
17,654	21,6728	5,7308	222,85		17,654	39,4173	20,0493	270,74	cooling	17,654	34,3956	16,0311	256,71	heating
17,656	21,9467	2,4984	271,86		17,656	38,5803	18,5763	279,63	cooling	17,656	34,4634	13,8171	288,6	heating
17,658	21,7818	4,6425	239,58		17,658	38,9313	20,287	260,62	cooling	17,658	34,3432	12,1256	310,57	heating
17,66	21,1095	0,3141	290,69		17,66	37,7982	17,5623	282,87	cooling	17,66	34,356	11,7894	315,45	heating
17,662	21,3992	-7,0235	397,31		17,662	39,1182	16,4658	316,65	cooling	17,662	34,0794	11,2404	319,26	heating
17,664	22,1795	-2,3263	342,55		17,664	37,8647	18,6163	269,06	cooling	17,664	34,21	10,9708	324,85	heating
17,666	21,3348	-1,4259	318,16		17,666	37,7559	14,9208	319,2	cooling	17,666	34,0237	11,2319	318,59	
17,668	22,2356	3,0734	267,86		17,668	39,1266	19,512	274,18	cooling	17,668	34,0174	10,7804	324,82	
17,67	22,1026	7,2375	207,79		17,67	39,1075	16,4905	316,15		17,67	34,3131	11,1226	324,17	
17,672	21,9977	0,4847	300,72		17,672	38,8249	16,5864	310,86	cooling	17,672	34,1427	11,7999	312,32	
17,674	22,8434	-0,552	327,03		17,674	38,6378	15,3933	324,92	cooling	17,674	34,0441	10,0665	335,17	
17,676	23,0581	-0,212	325,28		17,676	39,3402	16,6884	316,64	cooling	17,676	34,0954	10,3608	331,77	
17,678	22,8957	-4,7091	385,87		17,678	38,3109	11,5562	373,99	cooling	17,678	34,1119	9,6102	342,5	
17,68	23,5887	0,8641	317,66		17,68	38,4824	19,057	271,54	cooling	17,68	34,1696	12,6169	301,28	
17,682	22,6022	-0,6517	325,05		17,682	37,3602	17,1883	281,97	cooling	17,682	34,1287	15,7133	257,42	
17,684	23,2674	-1,0161	339,45		17,684	38,3452	21,1042	241	cooling	17,684	33,8439	18,579	213,38	
17,686	23,4666	7,9525	216,86		17,686	36,6777	19,61	238,58		17,686	33,7339	17,7252	223,78	
17,688	22,4552	5,6642	234,71		17,688	37,6288	18,4427	268,19		17,688	33,7707	15,0576	261,58	
17,69	23,5418	3,5898	278,9		17,69	37,2521	16,57	289,1		17,69	33,9028	12,0827	305,01	
17,692	23,364	2,7215	288,55		17,692	37,5162	15,7095	304,83		17,692	34,0384	14,9104	267,38	
17,694	23,4989	-6,4942	419,26		17,694	37,4299	14,0778	326,43		17,694	33,6234	16,7461	235,92	
17,696	24,165	-2,8974	378,29		17,696	39,04	16,1292	320,26		17,696	33,7851	17,4616	228,18	heating
17,698	24,3397	3,7433	287,91		17,698	38,1761	19,3307	263,43		17,698	33,4604	13,2108	283,06	heating
17,7	23,5992	1,5413	308,34		17,7	38,2146	16,6915	300,86		17,7	33,7635	8,8006	348,94	heating
17,702	24,4693	7,2648	240,49		17,702	39,1546	19,4783	275,05		17,702	33,9872	8,8762	351,02	heating
17,704	23,0022	1,1312	305,72		17,704	39,1316	14,6799	341,8		17,704	33,9843	8,8961	350,7	
17,706	23,9419	-4,0881	391,82		17,706	38,3721	15,1675	324,37		17,706	33,7262	8,3595	354,59	
17,708	23,4481	1,4965	306,85		17,708	37,9405	12,5327	355,16		17,708	34,0121	9,3986	344,06	
17,71	22,3767	-0,2433	316,19		17,71	38,8475	15,9568	319,98		17,71	33,6354	8,2672	354,61	
17,712	22,761	-1,2996	336,33		17,712	37,5594	13,5106	336,17		17,712	34,0933	7,4493	372,44	
17,714	22,7893	3,7458	266,2	cooling	17,714	38,1799	18,6257	273,34		17,714	34,0051	9,5372	342,03	
17,716	22,1978	-4,8761	378,45		17,716	37,586	16,0395	301,19		17,716	34,0676	9,0618	349,54	
17,718	23,1683	-1,758	348,43	cooling	17,718	38,453	15,4459	321,61		17,718	34,0052	11,5156	314,37	
17,72	23,2506	1,6899	301,39	cooling	17,72	37,3669	13,494	333,71		17,72	33,7407	11,6434	308,89	
17,722	22,3453	-4,3641	373,36	cooling	17,722	38,7627	14,9201	333,28		17,722	34,0721	11,4995	315,53	
17,724	23,8075	1,433	312,76	cooling	17,724	37,9004	16,2117	303,18		17,724	33,9837	12,0214	307	
17,726	22,1166	1,6231	286,47	cooling	17,726	38,1662	17,6394	286,93		17,726	33,7242	13,2545	286,14	

17,728	23,2691	-2,423	359,14	cooling	17,728	38,2473	18,9835	269,28		17,728	33,45	11,8804	301,51	
17,73	22,6932	-0,4773	323,89	cooling	17,73	39,3689	19,1913	282,05		17,73	33,7936	12,0199	304,36	
17,732	22,4406	-2,2133	344,62	cooling	17,732	38,2469	21,0571	240,29		17,732	33,2643	7,8058	355,87	
17,734	22,4169	-4,1443	371,29	cooling	17,734	37,6824	15,1294	315,26		17,734	33,8291	8,0182	360,8	
17,736	23,232	4,9032	256,21	cooling	17,736	39,1157	18,6099	286,64		17,736	33,706	9,3724	340,15	cooling
17,738	22,1847	0,7019	300,3	cooling	17,738	38,0141	15,3104	317,36		17,738	33,9803	11,5329	313,78	cooling
17,74	22,9372	3,1454	276,66	cooling	17,74	38,1265	18,3591	276,32		17,74	33,4677	12,9515	286,79	cooling
17,742	22,7065	5,3966	241,97	cooling	17,742	37,7547	20,1482	246,11		17,742	33,4593	12,7608	289,33	cooling
17,744	21,9122	2,0912	277,07	cooling	17,744	38,501	21,1465	242,59		17,744	33,3355	12,153	296,1	cooling
17,746	22,5848	3,7577	263,17	cooling	17,746	37,3026	14,64	316,79		17,746	33,2782	12,7085	287,53	cooling
17,748	21,881	2,7099	267,98		17,748	38,6159	17,3751	296,91		17,748	32,9372	12,7055	282,81	
17,75	22,7167	-2,7951	356,62		17,75	37,689	12,6575	349,9		17,75	33,2226	11,8673	298,52	
17,752	22,5435	1,6946	291,44		17,752	38,3003	14,4207	333,8		17,752	33,2577	12,6961	287,42	
17,754	22,1018	2,4616	274,54		17,754	37,1804	16,4114	290,32		17,754	33,0505	10,4021	316,59	
17,756	21,8916	-0,1288	307,81		17,756	37,9101	15,2263	317,09		17,756	33,6621	12,8957	290,28	
17,758	22,5347	5,1765	242,64		17,758	37,373	18,4491	264,53		17,758	33,4989	13,6978	276,79	
17,76	21,4857	1,3231	281,84		17,76	36,467	14,7135	304,08		17,76	33,6917	13,5482	281,58	
17,762	22,3761	0,7469	302,34		17,762	37,7639	13,8515	334,26		17,762	33,3904	13,0222	284,72	
17,764	22,5819	2,6233	278,99		17,764	38,3937	14,1433	338,99		17,764	33,6077	13,111	286,51	
17,766	21,7464	-1,8172	329,38		17,766	37,4324	16,3682	294,45		17,766	33,3397	10,4067	320,57	
17,768	22,2863	-1,7601	336,13		17,768	37,5855	14,1307	327,86		17,768	33,7087	11,4045	311,78	
17,77	21,9971	1,3144	289,11		17,77	39,2932	21,6889	246,08		17,77	33,0853	7,7925	353,56	
17,772	22,2465	1,451	290,69		17,772	38,1075	15,6472	313,96		17,772	33,6235	6,3211	381,65	
17,774	22,1454	4,7662	242,94		17,774	38,6031	21,0472	245,41		17,774	33,4156	9,0062	341,21	
17,776	21,7748	4,9951	234,56		17,776	37,799	19,5709	254,8		17,776	33,2622	10,886	312,79	cooling
17,778	21,6941	0,7302	293,04		17,778	38,546	19,1357	271,33		17,778	33,2869	13,3535	278,64	cooling
17,78	21,8527	1,4149	285,69		17,78	37,5565	19,739	249,06		17,78	33,2502	13,6397	274,12	
17,782	21,0745	-2,7262	332,7		17,782	37,9699	23,3354	204,57		17,782	32,9601	7,901	350,29	
17,784	21,4884	-4,3984	361,86		17,784	37,7366	21,6756	224,51		17,784	33,2068	4,3714	403,08	cooling
17,786	21,8048	1,4091	285,1		17,786	37,7796	23,1097	205,06	heating	17,786	33,2332	7,3088	362,38	cooling
17,788	21,0081	3,1265	249,96		17,788	37,06	19,5991	244,08	heating	17,788	32,8525	9,7066	323,55	cooling
17,79	21,4462	2,6584	262,63		17,79	38,5532	17,4862	294,49	heating	17,79	33,4219	17,6062	221,08	cooling
17,792	21,5466	2,7204	263,16		17,792	37,8688	18,494	270,83	heating	17,792	32,6788	16,5272	225,78	cooling
17,794	21,6438	-0,6102	311,08		17,794	37,3876	11,8001	357,67	heating	17,794	32,9705	11,9158	294,31	
17,796	21,8147	-1,2502	322,41		17,796	38,612	14,2854	340,05	heating	17,796	32,8355	9,2518	329,66	
17,798	21,9404	3,5284	257,37		17,798	38,6914	13,8553	347,17	heating	17,798	32,8394	10,6361	310,37	
17,8	21,6394	0,8367	290,79		17,8	37,8897	16,878	293,71		17,8	32,8006	12,4596	284,34	
17,802	21,7924	-0,0021	304,66		17,802	37,6828	16,5181	295,85		17,802	32,9096	17,1988	219,61	
17,804	21,9939	1,0219	293,16		17,804	38,743	20,4635	255,52		17,804	32,3929	11,9187	286,2	
17,806	21,7859	0,1837	301,97		17,806	37,9236	16,6889	296,83	heating	17,806	32,8005	8,6892	337,04	
17,808	22,4573	2,7697	275,2		17,808	38,1621	21,8728	227,7	heating	17,808	32,7039	9,2464	327,9	
17,81	21,4643	2,6119	263,53		17,81	37,9191	20,684	240,92	heating	17,81	32,5997	8,1203	342,19	
17,812	21,7951	-0,1406	306,63		17,812	38,8667	22,9313	222,75	heating	17,812	33,0364	10,0762	320,95	
17,814	21,4754	-1,6536	323,31		17,814	37,7577	21,2544	230,69	heating	17,814	32,6484	7,87	346,37	
17,816	21,6047	-1,6945	325,69		17,816	38,8227	21,0941	247,82	heating	17,816	32,8341	6,0503	374,4	cooling
17,818	21,018	-4,0644	350,61		17,818	38,4692	19,754	261,61	heating	17,818	33,1687	10,1213	322,17	cooling
17,82	21,5309	-0,4285	306,96		17,82	38,3013	18,0992	282,39	heating	17,82	32,6068	13,9112	261,34	cooling
17,822	21,0687	-0,3533	299,45		17,822	38,1329	19,205	264,58	heating	17,822	32,5746	13,5584	265,82	cooling
17,824	20,9088	-1,5395	313,79		17,824	39,2948	19,9157	270,89	heating	17,824	32,5769	15,4774	239,02	
17,826	21,0873	-2,1517	324,85		17,826	38,4918	21,1392	242,56	heating	17,826	32,4529	10,641	304,9	
17,828	21,1195	-1,6846	318,77		17,828	38,2346	16,956	297,44	heating	17,828	32,7476	9,9059	319,29	
17,83	21,8278	-0,2387	308,46		17,83	39,2947	18,1844	295,09	heating	17,83	32,8798	11,3481	300,98	
17,832	21,2318	1,36	277,78		17,832	39,4382	15,2855	337,62	heating	17,832	32,6868	13,3031	270,96	
17,834	21,4888	0,0261	300,02		17,834	38,9534	18,6387	283,97	heating	17,834	32,8838	16,4096	230,28	
17,836	21,1311	-1,7295	319,56		17,836	38,6604	15,8603	318,71	heating	17,836	32,5113	15,8007	233,59	
17,838	21,4581	0,409	294,24		17,838	40,0233	17,6472	312,79	heating	17,838	32,6983	10,9218	304,4	

17,84	20,7619	-0,6132	298,79		17,84	38,7674	12,4409	368,01	heating	17,84	33,166	11,8651	297,76	
17,842	21,4397	2,59	263,49		17,842	38,9313	13,6737	353,06	heating	17,842	32,4458	12,182	283,26	
17,844	21,1209	2,2128	264,31		17,844	38,3145	12,3569	362,85	heating	17,844	32,6923	12,823	277,74	
17,846	21,1524	-0,2567	299,27		17,846	38,6785	13,5066	351,87		17,846	32,5406	15,3181	240,75	
17,848	21,4977	-1,7462	324,92		17,848	37,6853	13,9991	331,1		17,848	32,5632	11,6547	292,27	
17,85	21,5449	-3,1369	345,01		17,85	38,7645	17,3168	299,81		17,85	32,6231	11,029	301,85	
17,852	22,272	-2,705	349,14		17,852	38,1466	18,4764	274,96		17,852	33,0369	13,1752	277,64	
17,854	21,8531	1,3456	286,67		17,854	38,0469	18,4382	274,1		17,854	32,3107	13,2297	266,72	
17,856	21,7731	-1,1401	320,29		17,856	38,1709	19,2319	264,74		17,856	32,7447	12,9337	276,93	
17,858	21,8263	-1,8386	330,8		17,858	39,1032	17,7132	299		17,858	32,244	10,8255	299,4	
17,86	22,0722	3,4964	259,66		17,86	38,6113	20,0365	259,65		17,86	32,7815	8,434	340,34	
17,862	21,1972	3,1546	252,21		17,862	38,2178	16,5262	303,22		17,862	32,9214	13,2502	274,97	
17,864	21,9407	5,1973	234,05		17,864	39,9646	18,8203	295,57		17,864	32,3987	13,4887	264,33	
17,866	21,2824	1,7782	272,64		17,866	39,6015	15,7552	333,34		17,866	32,6066	11,4167	296,2	
17,868	21,3523	-3,2036	343,25		17,868	39,1572	17,0367	309,21		17,868	32,4044	9,6464	318,12	
17,87	21,8812	-0,6826	315,41		17,87	38,836	14,3886	341,74		17,87	32,7653	7,9472	346,92	
17,872	21,7545	-0,0945	305,42		17,872	39,9032	16,2069	331,24		17,872	32,7976	8,9704	333,07	
17,874	22,2742	0,9977	297,41		17,874	38,6696	13,5927	350,54		17,874	33,064	12,3695	289,28	
17,876	21,8335	4,316	244,87		17,876	39,1756	17,4548	303,63		17,876	32,2446	10,9049	298,3	
17,878	21,548	-0,9609	314,64		17,878	38,5061	15,4956	321,65		17,878	32,9792	12,5684	285,31	
17,88	22,0132	-1,1187	323,35		17,88	38,8391	13,8053	349,94		17,88	32,4212	11,03	299,02	
17,882	21,4606	0,981	286,27		17,882	37,9889	12,5611	355,44		17,882	32,7922	9,3818	327,24	
17,884	20,8362	-1,5134	312,41		17,884	39,1423	14,3881	346,03		17,884	32,583	14,4717	253,17	
17,886	21,5423	3,0205	258,91		17,886	38,7432	17,9168	291,12		17,886	32,0043	15,7394	227,36	
17,888	20,2955	3,9007	229,17		17,888	38,2377	16,5867	302,65		17,888	32,0635	13,5853	258,3	
17,89	20,1247	-0,1849	283,9		17,89	38,7807	16,9758	304,8		17,89	32,1435	12,2263	278,41	
17,892	20,2926	1,0688	268,72		17,892	39,6641	15,8902	332,32		17,892	32,153	8,3973	332,07	
17,894	20,2501	0,1087	281,55		17,894	38,9031	16,9918	306,29		17,894	32,5929	7,754	347,21	
17,896	20,4883	0,3596	281,37		17,896	38,6027	13,8731	345,68		17,896	32,8202	14,2291	259,88	
17,898	20,4332	3,8217	232,21		17,898	40,0419	18,5515	300,4		17,898	32,2209	15,5998	232,34	
17,9	20,2855	-1,8138	308,92	heating	17,9	39,6412	16,4784	323,78		17,9	32,9706	18,5495	201,59	
17,902	21,1784	-2,3259	328,56	heating	17,902	39,1709	21,4723	247,4	cooling	17,902	32,3832	15,2989	238,81	heating
17,904	20,681	-1,491	309,93		17,904	38,5477	19,8479	261,39		17,904	33,0108	11,1164	306,05	heating
17,906	20,9757	-0,4349	299,29		17,906	39,6792	19,4221	283,16		17,906	32,7425	12,0502	289,25	heating
17,908	21,766	3,1294	260,51		17,908	38,5085	15,5154	321,41		17,908	32,8326	12,9679	277,68	heating
17,91	21,1926	1,5564	274,48		17,91	38,762	16,5213	310,89		17,91	32,3936	11,6662	289,74	heating
17,912	21,5535	-3,6952	352,94		17,912	38,4909	14,4365	336,25		17,912	33,2219	15,3979	249,15	heating
17,914	22,1196	-1,5627	331,04		17,914	38,9861	14,9022	336,66		17,914	32,2085	12,9828	268,75	heating
17,916	22,2438	-1,4815	331,64		17,916	38,2825	14,0304	339,01		17,916	33,0936	14,203	264,06	heating
17,918	21,8464	0,2778	301,5		17,918	39,5807	16,0542	328,87		17,918	32,3805	15,7103	233,02	heating
17,92	21,8079	3,8738	250,69		17,92	38,8683	16,4256	313,72	cooling	17,92	32,1966	12,8412	270,56	heating
17,922	21,1998	-1,333	314,97		17,922	38,3107	11,5074	374,67	cooling	17,922	32,4572	12,7201	275,9	heating
17,924	22,1444	-1,2761	327,38		17,924	39,0173	13,4096	357,96	cooling	17,924	32,4105	12,9352	272,24	heating
17,926	21,418	-0,1111	300,94		17,926	39,2217	13,8412	354,78	cooling	17,926	32,847	12,6989	281,64	heating
17,928	21,7867	-0,7605	315,18		17,928	38,8638	20,4906	256,83	cooling	17,928	32,7019	16,3441	228,66	heating
17,93	22,2376	1,5461	289,24		17,93	38,3284	22,7865	217,25	cooling	17,93	33,0519	18,1971	207,65	heating
17,932	21,4211	1,8463	273,63		17,932	39,2859	23,8219	216,16	cooling	17,932	32,7464	16,5809	225,97	heating
17,934	21,1748	-1,6549	319,12		17,934	38,5385	16,8451	303,24		17,934	33,3889	16,9203	230,21	heating
17,936	21,6746	1,142	287,02		17,936	38,58	18,2767	283,81		17,936	32,4824	11,7902	289,25	heating
17,938	21,4026	0,6741	289,75		17,938	38,1829	15,5751	316,02		17,938	33,6569	11,9919	302,84	heating
17,94	21,1674	-1,7769	320,73		17,94	39,1412	17,7148	299,51		17,94	32,6918	11,1096	301,69	heating
17,942	21,2831	0,5688	289,56		17,942	38,051	16,4183	302,39		17,942	33,138	14,1738	265,09	heating
17,944	20,7839	-1,1806	307,03		17,944	38,5584	17,1351	299,47		17,944	32,6366	16,3132	228,18	heating
17,946	21,1566	-0,9384	308,86		17,946	38,3494	15,9811	312,68		17,946	32,726	15,9693	234,23	heating
17,948	20,6682	1,1296	273,12		17,948	38,1709	13,1044	350,39		17,948	32,5014	12,0681	285,63	heating
17,95	20,9289	0,3609	287,51		17,95	38,2434	14,3629	333,82		17,95	33,0816	12,5876	286,48	heating

17,952	21,1325	-0,1903	298,06		17,952	38,7676	15,4944	325,32		17,952	32,9083	9,6425	325,22	heating
17,954	20,4247	-1,4233	305,4		17,954	38,3876	18,3105	280,65		17,954	33,1616	11,6542	300,64	heating
17,956	20,2017	-4,0568	339,1		17,956	37,9729	18,5833	271,04		17,956	32,9822	14,7642	254,66	heating
17,958	20,5048	-1,4297	306,61		17,958	39,159	22,6513	230,75		17,958	32,683	12,4359	283,02	heating
17,96	20,7058	0,0976	288,07		17,96	39,3681	18,6714	289,31		17,96	33,2495	13,9692	269,51	heating
17,962	20,4799	-0,8353	297,96		17,962	39,4287	19,9198	272,71		17,962	32,7345	14,6977	252,13	heating
17,964	20,7949	-0,8335	302,33		17,964	38,6147	12,4531	365,7		17,964	32,9045	12,6339	283,35	heating
17,966	20,2526	-5,6942	362,7		17,966	40,1306	11,7798	396,3		17,966	32,6303	11,575	294,32	heating
17,968	20,9362	-5,2816	366,49		17,968	39,3454	9,9675	410,66		17,968	32,6873	11,6963	293,42	heating
17,97	20,6598	-2,3095	321,08		17,97	38,8513	12,7734	364,53	cooling	17,97	32,4487	9,4315	321,75	heating
17,972	21,4666	-0,3578	305,07		17,972	38,6126	14,8465	332,21	cooling	17,972	33,3531	13,3821	279,17	heating
17,974	22,3957	1,3162	294,66		17,974	39,0924	19,0606	280,02	cooling	17,974	32,6393	10,4736	309,84	heating
17,976	22,3612	1,0578	297,79		17,976	37,5343	14,1824	326,43	cooling	17,976	33,5168	10,35	323,84	heating
17,978	22,4943	-1,9394	341,55		17,978	38,225	15,4044	319	cooling	17,978	33,0952	11,5816	300,73	heating
17,98	22,886	0,279	316,01		17,98	37,4995	16,3433	295,73	cooling	17,98	33,3806	11,9327	299,81	heating
17,982	23,1276	2,1834	292,77		17,982	37,5209	16,4485	294,56	cooling	17,982	33,2243	11,5742	302,64	heating
17,984	22,7842	2,5043	283,48		17,984	37,7371	22,0685	219,02	cooling	17,984	33,9426	14,0864	277,56	heating
17,986	23,1848	3,1594	279,93		17,986	38,4564	22,6707	220,66		17,986	33,287	11,5543	303,79	heating
17,988	22,9168	0,4985	313,37		17,988	38,4514	20,6204	249,25		17,988	33,8171	12,6284	296,19	heating
17,99	23,5977	-1,012	344,01		17,99	38,1618	15,346	318,93		17,99	33,4857	14,0191	272,11	heating
17,992	23,1897	-0,0222	324,47		17,992	38,9851	13,7491	352,76		17,992	33,4798	11,8138	302,86	heating
17,994	23,7621	3,2491	286,74		17,994	39,4628	11,4731	391,25		17,994	33,8259	12,9439	291,9	heating
17,996	23,7122	2,2029	300,67		17,996	39,0276	17,9406	294,77		17,996	33,6881	12,8481	291,31	heating
17,998	23,4665	0,4965	321,09		17,998	38,0575	16,6093	299,81		17,998	34,1263	13,3295	290,71	heating
18	23,4349	-1,6286	350,35		18	39,5662	20,482	266,77		18	33,6032	13,442	281,82	heating
18,002	23,423	-0,7925	338,5		18,002	38,2168	15,8899	312,1		18,002	33,8836	14,2956	273,81	heating
18,004	23,7782	2,5063	297,35		18,004	38,3301	16,3307	307,52		18,004	33,429	12,7347	289,28	heating
18,006	23,0548	3,9128	267,58		18,006	37,9083	15,6952	310,51		18,006	33,9177	15,5221	257,14	heating
18,008	23,6032	3,7089	278,09		18,008	38,2359	16,4953	303,9		18,008	33,3259	13,8404	272,38	heating
18,01	23,0053	1,309	303,28		18,01	37,0795	15,9021	296,03		18,01	33,9498	13,9544	279,51	heating
18,012	23,4484	0,8239	316,26		18,012	37,6556	18,6904	265,11		18,012	33,5152	11,7316	304,5	heating
18,014	22,752	-0,486	324,83		18,014	36,9224	18,156	262,33		18,014	33,7232	11,9314	304,62	heating
18,016	23,8218	2,9399	291,9		18,016	36,7643	16,421	284,37		18,016	33,5127	12,826	289,17	heating
18,018	23,1428	2,7887	284,52		18,018	36,671	16,0198	288,67		18,018	33,7971	15,9	250,18	heating
18,02	23,5869	3,1616	285,52		18,02	37,4205	11,6033	360,89		18,02	33,6016	16,0828	244,89	heating
18,022	23,5952	1,3937	310,34		18,022	37,3453	13,4179	334,47		18,022	33,7531	15,8472	250,3	heating
18,024	23,5627	0,2571	325,78		18,024	36,8008	11,6118	352,1		18,024	33,8538	15,6494	254,47	heating
18,026	23,5456	-2,4806	363,81		18,026	38,441	17,0995	298,32		18,026	33,7471	12,8805	291,69	heating
18,028	23,3794	-2,5817	362,9		18,028	38,5025	18,2147	283,59		18,028	33,8683	11,5923	311,39	heating
18,03	24,3	-0,7565	350,25	cooling	18,03	38,2044	20,888	242,06		18,03	33,9327	11,6031	312,13	heating
18,032	23,5978	1,9594	302,47	cooling	18,032	37,816	16,0776	303,87		18,032	33,8488	13,0088	291,31	heating
18,034	24,2049	4,2831	278,48	cooling	18,034	39,0419	15,7451	325,66		18,034	33,8295	12,402	299,53	heating
18,036	23,6376	3,7293	278,29	cooling	18,036	37,4547	9,9301	384,75		18,036	34,0174	13,2494	290,31	heating
18,038	24,4661	2,0287	313,64		18,038	37,3344	14,1254	324,43		18,038	33,6003	9,4556	337,51	heating
18,04	23,6879	-3,2952	377,18		18,04	36,3636	16,3566	279,67		18,04	33,9152	10,1994	331,51	heating
18,042	24,0737	-2,2011	367,28	cooling	18,042	36,5878	16,4213	281,9		18,042	33,478	10,2415	324,81	heating
18,044	23,774	-0,4933	339,22	cooling	18,044	35,9503	15,5566	285,07		18,044	33,8111	12,108	303,38	heating
18,046	23,6475	2,4121	296,84	cooling	18,046	36,6483	13,9632	317,1		18,046	33,6203	10,9619	316,73	heating
18,048	23,3796	3,5819	276,74	cooling	18,048	36,5235	12,6578	333,61		18,048	33,8158	8,7218	350,78	
18,05	23,3907	3,293	280,94	cooling	18,05	36,3918	11,7883	343,92		18,05	33,9613	7,3223	372,37	
18,052	23,8609	-0,3854	338,93	cooling	18,052	36,8596	15,399	299,99		18,052	34,2486	10,1147	337,36	
18,054	23,5385	-0,7513	339,53	cooling	18,054	37,4875	15,3224	309,84		18,054	33,8333	11,8494	307,3	
18,056	23,768	-0,309	336,56	cooling	18,056	37,3474	20,1697	240,12		18,056	34,0631	13,4897	287,59	
18,058	23,6296	-0,2239	333,44	cooling	18,058	36,6661	16,5017	281,87		18,058	33,882	12,8482	294,02	
18,06	24,3279	2,2111	309,16	cooling	18,06	38,221	18,6163	274,05		18,06	33,8336	7,9452	361,88	
18,062	23,4677	-0,2961	332,18	cooling	18,062	37,5553	13,9523	329,94		18,062	34,2745	8,9111	354,54	

18,064	23,9474	-1,1469	350,78	cooling	18,064	37,1198	13,7753	326,32		18,064	34,0029	9,7093	339,59	
18,066	23,8341	-0,0496	333,86	cooling	18,066	36,7704	12,6261	337,5		18,066	33,9889	12,7866	296,38	
18,068	23,7591	1,5781	310,06	cooling	18,068	37,6228	16,643	293,27		18,068	33,849	15,1233	261,76	
18,07	23,5372	2,7716	290,27	cooling	18,07	36,2584	15,0272	296,78		18,07	33,8521	14,2494	274,02	
18,072	24,0378	6,7459	241,72	cooling	18,072	36,4857	17,1733	269,96		18,072	34,1166	10,4649	330,62	
18,074	24,212	3,6441	287,51	cooling	18,074	35,8552	14,9784	291,83		18,074	34,1626	11,5282	316,4	
18,076	24,0931	0,4983	329,82	cooling	18,076	36,2459	11,5602	345,07		18,076	33,9785	10,3771	329,91	
18,078	23,9506	-0,9644	348,27	cooling	18,078	36,2911	12,2444	336,14		18,078	34,4658	13,7372	289,75	
18,08	23,6804	-1,9118	357,74	cooling	18,08	37,4126	11,4424	363,02		18,08	34,0124	14,3643	274,65	
18,082	24,1192	-0,7151	347,15	cooling	18,082	37,3781	15,1754	310,36		18,082	33,8182	12,0792	303,88	
18,084	23,9628	1,2177	317,94	cooling	18,084	36,9668	15,7316	296,84		18,084	34,0587	13,1291	292,56	
18,086	24,3357	2,0281	311,83	cooling	18,086	37,4573	18,0417	271,4		18,086	34,0277	13,5914	285,67	
18,088	24,4161	0,3935	335,8	cooling	18,088	37,7425	14,3476	327,03		18,088	33,6503	12,9485	289,38	
18,09	24,5358	0,1861	340,37	cooling	18,09	37,0103	15,3892	302,23		18,09	33,877	14,625	269,11	
18,092	24,315	-0,6564	349,06	cooling	18,092	36,4429	10,5899	361,39		18,092	33,4596	13,418	280,15	
18,094	24,5595	-0,7245	353,43	cooling	18,094	37,8501	14,1121	331,82	cooling	18,094	33,9314	12,1638	304,28	
18,096	25,0472	-0,3335	354,79	cooling	18,096	36,9361	10,358	371,52	cooling	18,096	33,7782	13,309	286,13	
18,098	24,5606	1,9221	316,45	cooling	18,098	37,1244	13,0943	335,9	cooling	18,098	34,1776	10,2145	334,97	
18,1	24,6127	-0,6689	353,4	cooling	18,1	36,8822	13,7865	322,84	cooling	18,1	34,2304	11,341	319,96	
18,102	24,5344	-0,8085	354,26	cooling	18,102	37,6933	15,3538	312,27	cooling	18,102	33,9955	10,0617	334,56	
18,104	24,7059	1,2332	328,11	cooling	18,104	36,4009	14,1993	310,35	cooling	18,104	33,8294	7,3899	369,59	
18,106	24,2673	2,6374	302,35	cooling	18,106	36,7747	15,8	293,2	cooling	18,106	34,6344	10,1037	342,9	
18,108	25,1616	5,7622	271,17	cooling	18,108	35,8591	13,5837	311,38	cooling	18,108	33,9882	10,971	321,75	
18,11	24,565	4,3688	282,31	cooling	18,11	36,1485	12,9608	324,13	cooling	18,11	34,1218	9,698	341,41	
18,112	24,8344	4,4178	285,39	cooling	18,112	35,4632	14,9132	287,26	cooling	18,112	34,3969	12,7755	302,24	
18,114	24,4036	4,4025	279,59	cooling	18,114	36,4106	14,4319	307,23	cooling	18,114	33,968	10,4047	329,38	
18,116	24,4067	2,341	308,45	cooling	18,116	36,1148	16,5597	273,35	cooling	18,116	34,3399	9,6851	344,64	
18,118	24,3959	0,8903	328,57	cooling	18,118	35,8526	11,9743	333,78	cooling	18,118	34,4106	13,7802	288,38	cooling
18,12	24,2015	1,76	313,7	cooling	18,12	37,1533	12,8642	339,53	cooling	18,12	34,7068	14,3545	284,5	cooling
18,122	24,2173	0,563	330,65	cooling	18,122	37,8769	12,8252	350,19	cooling	18,122	34,5168	15,4499	266,53	
18,124	24,0058	2,9933	293,72	cooling	18,124	37,3239	15,1338	310,18	cooling	18,124	34,6237	14,4442	282,08	
18,126	23,4593	1,6231	305,24	cooling	18,126	36,783	13,2459	329,01	cooling	18,126	34,0783	10,3928	331,09	
18,128	23,8766	2,5046	298,75	cooling	18,128	37,6684	19,213	257,98	cooling	18,128	35,0255	13,4569	301,5	
18,13	24,2897	4,5629	275,75	cooling	18,13	35,8044	14,4968	297,85	cooling	18,13	33,8899	15,7102	254,12	
18,132	23,2881	1,1618	309,29	cooling	18,132	35,7639	15,0889	289,01	cooling	18,132	34,531	14,7448	276,58	
18,134	24,0102	-0,2513	339,14	cooling	18,134	34,9189	13,4359	300,3	cooling	18,134	34,4819	15,0738	271,3	
18,136	23,7077	-0,8963	343,93	cooling	18,136	35,8779	12,9484	320,52	cooling	18,136	34,3016	8,7138	357,68	
18,138	23,3306	-5,4093	401,74	cooling	18,138	34,8842	12,8645	307,8	cooling	18,138	34,3149	4,2875	419,74	
18,14	24,2994	1,8089	314,38	cooling	18,14	35,8387	15,2436	287,89	cooling	18,14	35,0858	9,1344	362,76	
18,142	23,7573	5,84	250,46	cooling	18,142	35,344	13,8054	301,08	cooling	18,142	34,3329	9,1616	351,86	cooling
18,144	23,7863	3,1507	288,46	cooling	18,144	35,9965	13,5308	314,04	cooling	18,144	34,4226	10,5903	333,14	cooling
18,146	23,9677	4,812	267,77	cooling	18,146	35,5317	14,899	288,41	cooling	18,146	34,2758	13,5645	289,51	cooling
18,148	23,4018	0,3594	322,1	cooling	18,148	36,5082	13,515	321,41		18,148	33,3544	9,6559	331,27	cooling
18,15	23,6956	-3,5091	380,28	cooling	18,15	35,5888	16,2149	270,82	cooling	18,15	34,2537	11,5166	317,83	cooling
18,152	23,9125	2,1684	303,95	cooling	18,152	34,7465	12,7574	307,37		18,152	33,3452	12,8255	286,83	cooling
18,154	23,1852	1,4468	303,87	cooling	18,154	36,0209	16,166	277,54		18,154	34,3315	10,5417	332,55	cooling
18,156	24,0169	4,217	276,77	cooling	18,156	36,26	16,1669	280,87		18,156	33,8525	12,5922	297,19	cooling
18,158	23,4704	6,3736	238,99	cooling	18,158	36,1434	19,6058	231,17		18,158	33,7599	12,6766	294,71	cooling
18,16	23,0085	0,5394	314,08	cooling	18,16	35,653	18,0861	245,56		18,16	33,1464	11,2091	306,65	cooling
18,162	23,6936	2,1983	300,47	cooling	18,162	36,8994	20,5313	228,8		18,162	34,1484	16,9276	240,72	cooling
18,164	23,5815	4,0742	272,68	cooling	18,164	35,1861	14,2643	292,46		18,164	33,188	16,833	228,62	
18,166	23,5677	2,6502	292,4	cooling	18,166	35,8125	16,1972	274,19		18,166	34,0493	13,4965	287,3	
18,168	23,6488	4,4721	268,06		18,168	34,6694	14,8074	277,64		18,168	33,8417	12,739	294,99	
18,17	22,812	-1,4779	339,54		18,17	35,4433	13,4024	308,1		18,17	33,408	8,9977	341,22	
18,172	23,1105	-5,3313	397,58		18,172	34,3863	12,2436	309,52		18,172	33,587	10,5637	321,83	
18,174	23,129	-0,064	324,2	cooling	18,174	35,6563	11,9016	332,06		18,174	33,6893	15,8267	249,69	

18,176	22,6061	1,0776	300,94	cooling	18,176	35,5535	10,6992	347,43		18,176	33,6003	16,9467	232,79	
18,178	22,9898	4,6009	257,05	cooling	18,178	36,4688	11,2832	352,06		18,178	33,5892	15,6006	251,45	
18,18	22,3239	5,7901	231,12	cooling	18,18	36,9712	14,2058	318,23		18,18	33,7067	14,4449	269,25	
18,182	21,9868	-0,9679	320,87		18,182	37,8065	13,2195	343,69		18,182	33,1659	9,6332	328,95	
18,184	22,7346	-0,9591	331,2		18,184	37,2254	18,5925	260,46		18,184	33,6998	10,2726	327,48	
18,186	22,5577	-0,3654	320,43		18,186	35,8524	15,2415	288,11		18,186	33,2008	12,4047	290,7	
18,188	22,4126	-1,6118	335,83		18,188	37,0627	18,3503	261,57		18,188	33,5615	14,3179	269	
18,19	22,7337	3,2619	272,19	cooling	18,19	36,1629	16,9738	268,24		18,19	33,2058	15,1366	252,58	
18,192	21,9361	1,9586	279,26		18,192	35,9474	17,4461	258,62		18,192	33,4236	15,3791	252,24	
18,194	22,1417	-0,0356	310,01		18,194	35,8193	13,1704	316,6		18,194	33,2554	13,2138	280,15	
18,196	22,2212	4,3458	249,87		18,196	37,4818	16,8446	288,48		18,196	33,5201	10,0934	327,47	
18,198	21,7887	1,9347	277,53		18,198	36,0963	12,8242	325,31		18,198	33,4115	8,3979	349,65	
18,2	22,0988	2,4962	274,02		18,2	36,9657	17,0083	278,97		18,2	33,4339	8,0476	354,86	
18,202	21,6388	4,0514	245,85		18,202	35,9179	16,2166	275,4		18,202	33,2594	7,543	359,48	
18,204	21,2075	-2,8341	336,07		18,204	36,214	13,8456	312,68		18,204	33,171	7,3971	360,28	
18,206	22,1892	-3,337	356,82		18,206	35,1166	14,9468	281,94		18,206	33,5588	13,1967	284,63	
18,208	22,2565	-0,4204	316,99		18,208	35,3683	14,5881	290,48		18,208	33,0663	15,6431	243,55	
18,21	22,2278	-0,4587	317,12		18,21	34,9024	13,8794	293,87		18,21	33,3498	16,8822	230,19	
18,212	22,7051	3,9545	262,11		18,212	35,0992	14,7076	285,05		18,212	32,848	13,0091	277,32	
18,214	22,1087	1,6296	286,27		18,214	35,3039	16,9103	257,11		18,214	33,1954	8,8321	340,56	
18,216	22,3356	-4,1611	370,38		18,216	36,358	16,7129	274,61		18,216	33,144	7,3064	361,17	
18,218	22,564	-2,9997	357,34		18,218	35,9773	22,314	190,99		18,218	33,49	10,9605	314,93	
18,22	22,5746	-0,4378	321,68		18,22	35,1527	16,0281	267,33	heating	18,22	33,1775	13,469	275,5	
18,222	22,9566	2,4273	286,97		18,222	36,6902	18,3204	256,78	heating	18,222	33,3214	16,0316	241,69	
18,224	22,5134	4,5285	251,4		18,224	35,7673	15,1916	287,62	heating	18,224	32,7326	14,4479	255,59	
18,226	22,0435	-0,0879	309,36		18,226	35,1863	15,7788	271,29	heating	18,226	32,9482	13,1781	276,36	
18,228	23,0336	1,6876	298,38		18,228	34,654	15,297	270,58	heating	18,228	33,0445	14,7514	255,71	
18,23	22,6847	3,5807	267,05		18,23	35,6125	18,471	239,61	heating	18,23	33,0503	14,836	254,61	
18,232	22,6726	4,3771	255,74		18,232	34,6849	14,6093	280,63	heating	18,232	33,1201	13,6011	272,85	
18,234	22,9412	5,6782	241,31		18,234	36,0044	20,7232	213,61	heating	18,234	33,0798	10,0417	322,04	
18,236	22,3814	0,8189	301,41		18,236	35,3565	19,7612	218	heating	18,236	33,024	8,3979	344,24	
18,238	22,3542	-3,9893	368,24		18,238	36,4541	18,0814	256,82	heating	18,238	33,1383	11,4169	303,63	
18,24	22,3145	-0,2456	315,36		18,24	35,736	18,2017	245,1	heating	18,24	33,2673	15,567	247,42	
18,242	22,1703	1,4604	289,49		18,242	36,3271	15,5334	290,67	heating	18,242	33,0189	12,6769	284,35	
18,244	21,8211	4,2407	245,75		18,244	35,7916	16,1381	274,73	heating	18,244	33,2426	9,6366	329,98	
18,246	21,4579	7,3659	196,99		18,246	35,1687	16,6324	259,11	heating	18,246	32,8607	5,3776	384,17	
18,248	20,523	0,0883	285,65		18,248	35,0021	18,4796	230,96	heating	18,248	33,3018	7,5874	359,45	
18,25	21,697	-0,5593	311,11		18,25	35,7586	16,9189	263,35	heating	18,25	33,0301	14,457	259,62	
18,252	21,0186	-0,2422	297,19		18,252	35,1981	18,9523	227,09	heating	18,252	32,9078	18,6353	199,51	
18,254	21,3041	-2,7141	335,74		18,254	35,1071	12,7063	313,13	heating	18,254	32,7837	16,3262	230,05	
18,256	21,8215	2,7159	267,07		18,256	37,1485	17,0128	281,47	heating	18,256	33,1912	13,6902	272,6	
18,258	21,2566	1,9914	269,3		18,258	36,5587	14,3518	310,42	heating	18,258	32,7748	9,3518	327,42	
18,26	21,3306	-4,613	362,65		18,26	36,4616	17,0115	271,88	heating	18,26	33,3727	10,6272	317,95	
18,262	22,617	2,2246	285,06		18,262	35,8844	15,9694	278,38	heating	18,262	33,2591	14,8597	257,2	
18,264	22,0887	1,0444	294,17		18,264	36,4266	16,5246	278,2	heating	18,264	33,1396	14,4389	261,41	
18,266	22,5722	2,0079	287,46		18,266	34,984	13,8708	295,13	heating	18,266	33,1276	13,4208	275,47	
18,268	22,7547	9,3265	187,71		18,268	35,6379	16,4316	268,48	heating	18,268	33,1029	12,1316	293,15	
18,27	21,3409	-0,0634	299,2		18,27	34,9628	14,9991	279,06	heating	18,27	33,1025	13,2581	277,4	
18,272	22,6345	-2,9362	357,44		18,272	35,586	13,3284	311,13	heating	18,272	33,0748	15,4382	246,53	heating
18,274	21,9029	0,6858	296,58		18,274	35,3434	15,499	277,4	heating	18,274	33,0841	15,0034	252,74	heating
18,276	21,7204	-2,9057	344,24		18,276	36,5733	15,5721	293,57	heating	18,276	33,1315	9,5852	329,14	heating
18,278	22,4668	4,9631	244,68		18,278	35,972	18,7324	240,98	heating	18,278	33,473	9,5286	334,71	heating
18,28	21,8327	9,1304	177,56		18,28	35,6387	16,9472	261,28	heating	18,28	33,0352	8,4357	343,86	heating
18,282	21,74	-0,6399	312,84	heating	18,282	36,0556	17,9189	253,52	heating	18,282	33,7005	12,741	292,98	
18,284	23,2551	5,0936	253,87		18,284	36,3732	13,0564	325,93	heating	18,284	33,3959	15,5772	249,08	
18,286	21,899	-0,9115	318,86	heating	18,286	35,8155	15,795	279,86	heating	18,286	33,203	13,8218	270,92	

18,288	22,6175	-2,0603	344,96		18,288	35,4795	12,6405	319,26	heating	18,288	33,0103	10,6904	312	heating
18,29	23,2778	9,2938	195,48		18,29	37,3851	16,3676	293,79	heating	18,29	32,9427	10,4206	314,83	
18,292	21,559	2,8673	261,28	heating	18,292	36,9238	15,161	304,21	heating	18,292	32,4776	9,1452	326,15	
18,294	22,9688	1,889	294,66	heating	18,294	37,5547	19,9877	245,56	heating	18,294	32,8736	12,9384	278,67	
18,296	22,6596	8,4705	198,34	heating	18,296	37,3022	18,8948	257,31	heating	18,296	32,5125	15,0544	244,04	
18,298	22,3786	-4,0409	369,31	heating	18,298	38,4718	19,1538	270,04	heating	18,298	32,6449	12,8148	277,2	
18,3	23,1265	-0,2957	327,41	heating	18,3	36,8602	14,721	309,47	heating	18,3	32,7104	13,0538	274,77	heating
18,302	22,6261	4,2636	256,68	heating	18,302	37,6146	14,4944	323,18	heating	18,302	32,8576	11,9786	291,86	heating
18,304	22,3223	-4,8554	379,9	heating	18,304	36,126	14,9124	296,54	heating	18,304	32,9028	10,0493	319,46	heating
18,306	23,885	5,3524	259,06		18,306	36,1005	16,758	270,38	heating	18,306	33,0494	9,8022	324,96	
18,308	21,4899	0,1332	298,53	heating	18,308	35,3705	19,6013	220,43	heating	18,308	32,8024	10,4657	312,23	
18,31	22,5453	-6,256	402,6		18,31	36,6114	17,8264	262,59	heating	18,31	32,9696	10,2849	317,1	
18,312	23,1298	2,835	283,69		18,312	36,6619	19,346	242,05	heating	18,312	33,1411	14,092	266,28	
18,314	21,4393	-2,5146	334,84		18,314	36,9165	17,0316	277,96	heating	18,314	32,8944	14,932	251,09	
18,316	22,8335	-0,8028	330,4		18,316	38,3812	20,9485	243,68	heating	18,316	33,1448	15,0359	253,14	
18,318	22,7152	8,1609	203,45		18,318	38,3247	19,5816	262	heating	18,318	33,1302	14,5888	259,18	heating
18,32	22,5209	-2,9716	356,35		18,32	37,6719	22,1559	216,89	heating	18,32	33,0799	13,4625	274,22	heating
18,322	23,36	1,7088	302,65		18,322	36,5565	18,231	256,16	heating	18,322	32,8594	11,0106	305,41	heating
18,324	22,9263	6,2228	233,49		18,324	37,871	22,2314	218,62	heating	18,324	33,5165	13,7007	277	heating
18,326	22,7514	-2,215	348,99		18,326	35,947	16,6696	269,47	heating	18,326	32,9849	14,139	263,44	heating
18,328	24,5116	7,8983	232,23		18,328	36,8038	18,4838	256,09	heating	18,328	33,4095	14,0083	271,2	heating
18,33	22,2106	3,8922	256,06		18,33	35,9715	15,273	289,33	heating	18,33	32,8894	13,1332	276,16	heating
18,332	23,3505	-2,5373	361,87		18,332	37,2155	14,9312	311,5	heating	18,332	33,0168	11,0507	307,05	heating
18,334	23,8202	4,4843	270,29		18,334	36,2261	14,8271	299,13	heating	18,334	33,0287	12,8246	282,42	heating
18,336	22,5389	-1,7555	339,6		18,336	37,8177	19,6202	254,37	heating	18,336	33,4763	16,8229	232,79	heating
18,338	23,8312	-0,89	345,57		18,338	37,1669	22,6318	203,18	heating	18,338	32,9573	14,3023	260,77	heating
18,34	23,7966	8,6543	211,67		18,34	37,1817	21,3203	221,72	heating	18,34	33,2481	11,1616	308,74	heating
18,342	23,4485	-0,6489	336,85		18,342	37,108	19,3878	247,7	heating	18,342	33,0772	9,7304	326,35	heating
18,344	24,1736	1,8698	311,77		18,344	37,9268	14,2672	330,73	heating	18,344	33,1077	8,2257	347,81	heating
18,346	23,5186	2,503	293,77		18,346	37,6587	15,4204	310,86	heating	18,346	33,4212	12,2634	295,75	heating
18,348	23,3024	-5,7414	405,99		18,348	37,4475	12,5617	347,87	heating	18,348	33,0972	14,9893	253,12	heating
18,35	24,6843	4,5943	280,83		18,35	39,3165	19,7667	273,28	heating	18,35	33,3695	14,2529	267,22	heating
18,352	22,7269	3,6443	266,75		18,352	38,9133	18,1182	290,68	heating	18,352	33,019	12,7217	283,73	heating
18,354	23,9117	-2,1188	363,87		18,354	38,6833	20,3802	255,85	heating	18,354	33,1266	11,2699	305,52	heating
18,356	24,0162	4,6118	271,25		18,356	37,7945	15,264	314,94	heating	18,356	32,9162	8,3044	344,04	heating
18,358	22,8833	-1,2339	337,12		18,358	38,6563	15,9134	317,91	heating	18,358	33,9053	11,4968	313,24	heating
18,36	23,7671	-2,5777	368,26		18,36	36,6756	11,5868	350,7	heating	18,36	33,1052	10,1797	320,46	heating
18,362	24,3523	4,2416	281,12		18,362	36,9868	17,0711	278,39	heating	18,362	33,9251	11,0997	319,07	
18,364	23,4299	-3,5338	376,91		18,364	36,0895	20,0295	224,49	heating	18,364	33,306	11,2353	308,52	
18,366	24,6219	1,0931	328,9		18,366	36,3669	21,419	208,95	heating	18,366	33,4256	10,9142	314,68	
18,368	23,6943	2,8219	291,77		18,368	35,5858	18,7593	235,21	heating	18,368	33,4186	14,1412	269,47	
18,37	23,8085	-2,7204	370,83		18,37	37,207	15,6928	300,74	heating	18,37	33,4258	16,1385	241,65	
18,372	24,4765	4,8055	274,97	cooling	18,372	37,1049	14,5404	315,42	heating	18,372	33,2772	13,7111	273,5	
18,374	23,7064	7,0588	232,71		18,374	37,5659	12,311	353,03	heating	18,374	33,2794	10,7704	314,64	heating
18,376	24,4783	3,5188	292,98		18,376	38,6911	16,9547	303,84	heating	18,376	33,2337	7,9591	353,3	
18,378	24,4211	8,1052	228,07		18,378	39,2283	14,3461	347,82	heating	18,378	33,4802	7,7534	359,62	
18,38	23,5554	1,5358	307,8		18,38	38,3646	15,2699	322,83	heating	18,38	34,0248	14,2701	276,14	
18,382	24,1346	0,8325	325,73		18,382	37,7171	13,1199	343,83	heating	18,382	33,4607	14,1995	269,24	
18,384	24,6683	7,2896	242,93		18,384	38,3989	17,2403	295,77	heating	18,384	34,127	12,704	299,46	
18,386	23,3013	1,4832	304,98		18,386	37,393	12,9866	341,17	heating	18,386	33,8273	9,7036	337,21	
18,388	24,5614	4,4905	280,56		18,388	37,1774	16,1332	294,17	heating	18,388	34,0091	10,8171	324,19	
18,39	23,5592	4,8284	261,83		18,39	36,3682	11,2364	351,31	heating	18,39	33,7031	13,9858	275,62	
18,392	23,743	-1,5579	353,67		18,392	37,4622	12,7567	345,35		18,392	33,7816	17,9472	221,34	
18,394	24,2425	1,113	323,32		18,394	35,7895	13,8646	306,48		18,394	33,4074	16,6421	234,35	
18,396	24,1635	2,0889	308,57		18,396	36,1383	17,2616	263,87		18,396	33,881	15,6433	254,94	
18,398	24,7396	-0,4942	352,73		18,398	35,3055	18,7542	231,36		18,398	33,7208	11,995	303,69	heating

18,4	24,305	4,3532	278,9	18,4	35,4214	19,1454	227,51	heating	18,4	34,0912	10,408	331,06	heating
18,402	23,7202	-0,7638	342,25	18,402	34,3364	14,7135	274,3	heating	18,402	33,8876	11,5851	311,75	
18,404	24,0706	-2,9727	378,03	18,404	36,2641	13,6735	315,78	heating	18,404	33,8531	9,7863	336,42	
18,406	24,7516	3,41	298,32	18,406	35,4166	13,7878	302,34	heating	18,406	33,9233	10,1311	332,58	
18,408	23,3991	2,202	296,3	18,408	36,0639	9,8785	366,03	heating	18,408	33,4866	10,5778	320,23	
18,41	24,6396	5,1326	272,68	18,41	37,427	15,6546	304,35		18,41	32,952	12,3597	287,85	
18,412	23,6314	5,3767	255,17	18,412	38,3688	14,2293	337,44		18,412	32,6768	13,4284	269,06	
18,414	23,7411	0,805	320,61	18,414	38,3416	15,8179	314,85		18,414	32,5973	12,2496	284,43	
18,416	23,6969	0,6766	321,79	18,416	38,1674	14,8717	325,64		18,416	32,8658	8,0924	346,3	
18,418	24,2543	4,6271	274,36	18,418	39,3334	16,8665	314,05		18,418	33,4992	9,9037	329,83	
18,42	23,9418	3,9099	280,02	18,42	38,9645	13,7263	352,79		18,42	33,3102	11,3986	306,29	
18,422	23,9715	5,5282	257,81	18,422	39,0749	19,8501	268,73		18,422	33,4863	15,0146	258,21	
18,424	23,6666	2,7071	292,98	18,424	38,0677	14,9489	323,17		18,424	33,2503	16,3042	236,88	
18,426	23,7148	0,5183	324,25	18,426	40,2298	20,4337	276,72		18,426	33,3989	14,5808	263,05	
18,428	23,977	2,2148	304,2	18,428	38,3377	17,9067	285,59		18,428	33,1493	11,6831	300,07	
18,43	23,1435	0,7191	313,46	18,43	39,4269	18,0088	299,39		18,43	33,1922	12,2345	292,96	
18,432	23,8083	1,756	308,26	18,432	38,7911	16,5152	311,38		18,432	32,762	13,1083	274,73	
18,434	23,2837	1,7115	301,55	18,434	38,8093	13,808	349,48		18,434	32,8573	13,67	268,21	
18,436	23,3133	-0,173	328,3	18,436	37,7706	10,3569	383,2		18,436	32,7007	12,9485	276,11	
18,438	23,3926	1,4234	307,1	18,438	38,6109	13,1941	355,29		18,438	32,9804	10,9545	307,89	
18,44	23,355	2,7015	288,7	18,44	37,397	14,3036	322,81		18,44	32,9838	10,4782	314,6	
18,442	23,6835	0,7745	320,23	18,442	37,5302	15,3344	310,26		18,442	32,9233	9,9668	320,9	
18,444	23,6126	4,5094	267,04	18,444	38,2549	21,4695	234,64		18,444	32,8551	10,9346	306,42	
18,446	23,3204	4,3	265,88	18,446	38,7542	18,1118	288,55		18,446	32,5791	8,6777	334,11	
18,448	22,9427	-0,0101	320,85	18,448	39,0211	20,9199	253,03		18,448	32,822	8,0136	346,78	
18,45	23,5302	2,2764	297,1	18,45	38,5084	17,329	296,06		18,45	32,4437	6,8746	357,42	
18,452	22,4956	1,3334	295,82	18,452	40,327	20,3882	278,71		18,452	32,7387	9,0438	331,22	
18,454	23,0854	1,3026	304,49	18,454	39,5629	17,5887	307,17		18,454	32,4121	11,7025	289,49	
18,456	22,8608	1,0004	305,58	18,456	39,3774	20,0515	270,15		18,456	32,612	14,9221	247,28	
18,458	22,8398	-3,238	364,53	18,458	38,6351	16,7732	305,6		18,458	32,3077	12,5968	275,53	
18,46	23,1412	-3,02	365,69	18,46	39,2968	19,2333	280,46		18,46	33,0358	11,6115	299,48	
18,462	23,3717	4,6418	261,82	18,462	37,564	14,2119	326,43		18,462	32,653	10,2945	312,54	
18,464	23,0818	4,5537	259	18,464	38,724	16,3047	313,39		18,464	32,6726	11,959	289,55	
18,466	22,8896	3,6274	269,26	18,466	38,1714	14,8202	326,42		18,466	32,3844	13,8278	259,39	
18,468	22,7629	0,942	305,02	18,468	38,9844	15,2005	332,46		18,468	32,326	13,3757	264,9	
18,47	22,6334	-2,0482	345,01	18,47	38,488	18,5184	279,15		18,47	32,3654	13,2779	266,82	
18,472	23,0545	1,686	298,7	18,472	39,2599	18,4808	290,46		18,472	32,6672	15,2976	242,8	
18,474	22,1298	3,0825	266,25	18,474	38,4752	19,4236	266,31		18,474	32,7276	15,5205	240,53	
18,476	22,327	2,617	275,52	18,476	38,0297	17,6694	284,61		18,476	32,7667	13,8318	264,68	heating
18,478	22,0536	2,156	278,14	18,478	38,2594	18,2154	280,18		18,478	32,7122	13,6665	266,23	heating
18,48	21,6265	-2,95	343,54	18,48	38,9111	15,9836	320,49		18,48	32,4988	8,7789	331,57	heating
18,482	22,0888	-4,4354	370,77	18,482	38,3056	18,7628	273,18		18,482	32,9922	9,4486	329,1	heating
18,484	22,3487	-0,3243	316,94	18,484	38,2759	15,5571	317,58		18,484	32,8312	12,27	287,41	
18,486	22,0009	-1,3154	325,93	18,486	40,1525	21,6852	258,15		18,486	32,8741	16,3314	231,24	
18,488	22,257	0,9574	297,74	18,488	39,4275	18,2041	296,67		18,488	32,8267	16,3473	230,36	heating
18,49	21,6487	-0,8749	314,85	18,49	39,7674	19,5362	282,8		18,49	32,7225	15,3375	243,02	heating
18,492	21,4541	-5,5899	378,03	18,492	38,9894	16,6297	312,56		18,492	32,5854	11,0444	301,11	heating
18,494	21,5864	-0,6254	310,49	18,494	39,3111	15,9629	326,37		18,494	33,0056	11,3475	302,75	heating
18,496	20,8381	2,3733	258,11	18,496	37,752	14,5823	323,88		18,496	32,9545	11,3952	301,37	heating
18,498	21,2178	2,7603	258,01	18,498	38,2372	18,5696	274,92		18,498	33,2237	11,5643	302,77	heating
18,5	21,1736	4,7568	229,48	18,5	37,1759	15,2849	306		18,5	33,3151	13,5908	275,72	heating
18,502	20,3872	-2,4226	318,85	18,502	38,0573	14,4767	329,62		18,502	33,3743	15,3485	251,98	heating
18,504	20,8069	-1,8024	316,05	18,504	37,4107	13,3676	336,09		18,504	33,3748	16,1938	240,17	heating
18,506	20,6379	1,9951	260,6	18,506	38,8369	10,315	398,69		18,506	33,5298	15,0402	258,46	heating
18,508	20,2114	-1,3865	301,91	18,508	38,3117	14,9006	327,25		18,508	33,4609	12,331	295,36	heating
18,51	20,9619	2,9066	252,39	18,51	37,8122	11,7824	363,86		18,51	33,8617	10,1718	331,15	heating

18,512	20,4073	3,3011	239,12	
18,514	21,3978	0,1369	297,2	
18,516	22,0784	3,1634	264,4	
18,518	21,5404	-1,1694	317,45	
18,52	22,2131	-5,692	390,07	
18,522	22,6268	0,1514	314,17	
18,524	21,6992	-3,9945	359,16	
18,526	22,3589	-4,101	369,87	
18,528	22,4249	0,2752	309,62	
18,53	21,5766	-2,8371	341,27	
18,532	22,0585	0,2985	304,17	
18,534	21,2143	0,0868	295,33	
18,536	21,6142	-4,3145	362,44	
18,538	22,1146	1,983	281,41	
18,54	21,487	2,5055	265,33	
18,542	21,9493	-1,8144	332,18	
18,544	22,3593	1,3738	293,35	
18,546	21,5911	-1,9164	328,6	
18,548	22,5016	1,6128	292	
18,55	22,4468	8,1716	199,55	
18,552	21,5522	2,2869	269,3	
18,554	22,0119	0,3535	302,75	
18,556	21,6067	1,1574	285,85	
18,558	21,5011	-3,3554	347,46	
18,56	21,8084	0,1	303,45	
18,562	21,6475	1,6153	280,02	
18,564	21,6882	-2,3015	335,34	
18,566	21,8924	0,3842	300,65	
18,568	21,094	-2,5538	330,56	
18,57	21,6422	-5,0807	373,55	
18,572	21,9153	0,8182	294,91	
18,574	20,9957	-2,0028	321,49	
18,576	21,361	-1,7755	323,41	
18,578	21,1558	0,1395	293,78	
18,58	20,6628	-4,5755	352,79	
18,582	21,0641	-2,2607	326,05	
18,584	20,6375	1,1727	272,09	cooling
18,586	20,3109	-2,167	314,21	
18,588	20,1552	-0,2157	284,75	
18,59	19,3747	-0,2177	273,87	
18,592	19,5223	-1,4397	293,02	
18,594	19,8526	2,0924	248,26	
18,596	19,3451	0,0423	269,83	
18,598	19,8393	-1,3599	296,33	
18,6	19,7031	-2,2409	306,74	
18,602	19,6518	-5,3367	349,3	
18,604	19,8743	-5,1272	349,48	
18,606	20,1857	-2,5636	318	
18,608	20,3882	-3,0545	327,69	
18,61	20,7354	0,1362	287,95	
18,612	20,5614	-0,8447	299,23	
18,614	20,9134	-2,4394	326,44	
18,616	21,2375	0,0955	295,53	
18,618	20,7668	0,0327	289,83	
18,62	21,2436	0,3088	292,64	
18,622	20,968	1,5001	272,13	

18,512	38,5171	11,894	372,15	
18,514	39,2499	13,665	357,64	cooling
18,516	37,8866	15,409	314,2	cooling
18,518	38,0438	14,2944	331,98	cooling
18,52	39,1121	22,3205	234,72	cooling
18,522	37,9045	16,1509	304,08	cooling
18,524	38,67	18,5793	280,84	cooling
18,526	37,7351	18,3124	271,5	cooling
18,528	38,3758	16,8318	301,15	
18,53	37,0386	18,3478	261,27	
18,532	37,3653	21,9198	215,9	
18,534	36,2762	17,1621	267,19	
18,536	37,5586	18,8831	261,06	
18,538	36,1919	15,6977	286,48	
18,54	38,3261	12,6003	359,61	
18,542	37,9834	17,4087	287,6	
18,544	38,1404	14,5552	329,69	
18,546	39,3923	17,9237	300,1	
18,548	40,4354	22,2706	253,92	
18,55	39,2016	20,5645	260,52	
18,552	39,1761	18,8531	284,09	
18,554	39,4409	21,9355	244,7	
18,556	37,7818	10,5681	380,41	
18,558	38,5066	13,6693	347,19	
18,56	36,7851	11,7891	349,41	
18,562	38,0058	11,991	363,65	
18,564	36,6413	16,3015	284,32	
18,566	36,9893	18,4629	258,97	
18,568	36,4204	14,4394	307,26	
18,57	37,596	17,7063	278,03	
18,572	36,5491	15,6483	292,16	
18,574	38,4854	18,0725	285,34	
18,576	37,6672	25,564	169,19	
18,578	37,4345	21,0028	229,69	
18,58	39,3143	24,4605	207,63	
18,582	39,2593	22,2472	237,8	heating
18,584	38,9782	17,9461	294	heating
18,586	38,811	16,7652	308,17	heating
18,588	39,2361	18,2636	293,16	heating
18,59	37,8134	10,4336	382,73	heating
18,592	39,1128	17,7661	298,4	
18,594	37,3098	15,2471	308,4	
18,596	38,9638	16,8348	309,33	
18,598	37,8108	19,8747	250,72	
18,6	38,7688	16,7401	307,93	
18,602	38,8202	14,1292	345,14	
18,604	39,7686	18,0877	303,07	
18,606	39,5838	17,4449	309,47	
18,608	40,9976	20,3891	288,08	
18,61	39,6311	24,3867	213,09	
18,612	38,9424	17,86	294,7	
18,614	40,3185	21,4774	263,37	
18,616	39,3802	16,7859	315,83	
18,618	39,5848	16,5724	321,68	
18,62	39,0178	17,1759	305,32	
18,622	39,6756	20,0735	274,01	

18,512	33,7011	11,2846	313,35	heating
18,514	33,6676	10,2201	327,76	heating
18,516	33,6465	11,4593	310,14	heating
18,518	33,5808	11,6195	306,99	heating
18,52	33,5097	12,1483	298,6	heating
18,522	33,4852	11,8136	302,94	heating
18,524	33,337	10,797	315,07	heating
18,526	33,2437	8,9106	340,14	
18,528	33,5412	12,5426	293,53	
18,53	33,1911	16,3869	234,9	
18,532	33,4049	17,1233	227,59	
18,534	33,2051	13,5529	274,71	heating
18,536	33,5875	8,9983	343,72	heating
18,538	33,4828	7,2466	366,74	heating
18,54	33,8382	9,3698	342,03	
18,542	33,5131	12,2852	296,73	
18,544	33,6773	13,815	277,64	
18,546	33,5756	13,4975	280,66	
18,548	33,7277	11,575	309,66	
18,55	33,8863	13,4796	285,25	
18,552	33,7128	15,9566	248,21	
18,554	33,796	17,5203	227,51	
18,556	33,4799	13,6813	276,75	heating
18,558	33,73	10,9642	318,23	heating
18,56	33,5507	9,1719	340,78	heating
18,562	33,855	11,9863	305,69	
18,564	33,4696	13,6588	276,93	
18,566	33,4887	14,4775	265,75	
18,568	33,1016	11,5018	301,93	
18,57	33,2882	9,5166	332,29	
18,572	33,3188	10,0166	325,73	
18,574	33,4005	10,1259	325,34	
18,576	33,3933	11,2968	308,88	
18,578	33,2633	10,6836	315,63	
18,58	33,3592	11,0865	311,34	
18,582	33,7312	13,5931	281,5	
18,584	33,7254	15,0616	260,89	
18,586	33,8605	12,6976	295,83	
18,588	33,9472	12,0505	306,08	
18,59	33,5456	8,4956	350,16	
18,592	33,7226	11,1386	315,69	
18,594	33,4834	15,3299	253,76	
18,596	33,3357	15,2434	252,9	
18,598	33,3674	13,5657	276,8	
18,6	33,5466	10,9037	316,51	
18,602	33,4695	7,0988	368,62	
18,604	34,1042	10,5109	329,8	
18,606	33,5849	13,3206	283,26	
18,608	33,9263	12,2734	302,68	
18,61	33,5357	11,5444	307,41	
18,612	33,297	9,791	328,58	
18,614	33,3667	11,7982	301,5	
18,616	33,3727	12,3361	294,06	
18,618	33,1124	8,2738	347,21	
18,62	33,4684	7,3629	364,92	
18,622	33,3228	9,1458	337,96	

18,624	20,9827	1,7343	269,06
18,626	20,647	1,3158	270,22
18,628	21,299	5,9277	214,87
18,63	20,9174	4,5024	229,46
18,632	20,855	2,8488	251,7
18,634	20,7093	-0,7961	300,61
18,636	20,5569	-4,0438	343,88
18,638	21,5108	-0,7894	311,72
18,64	20,9276	1,6228	269,85
18,642	21,4609	-1,0354	314,47
18,644	21,378	-2,0999	328,19
18,646	21,5146	-2,0267	329,07
18,648	21,0635	-3,4452	342,59
18,65	21,7778	2,5531	268,73
18,652	21,4159	2,4292	265,41
18,654	21,4482	2,1179	270,21
18,656	21,675	1,4901	282,15
18,658	20,9846	-2,2415	324,67
18,66	22,2182	-1,2071	327,45
18,662	21,4829	2,0853	271,15
18,664	21,9909	0,6365	298,5
18,666	21,5717	1,061	286,71
18,668	21,8304	2,3048	272,94
18,67	21,0505	-0,4512	300,56
18,672	21,8984	3,7484	253,71
18,674	21,3043	2,0938	268,54
18,676	21,6108	-0,7685	312,83
18,678	21,9571	-2,5693	342,84
18,68	21,596	-4,1178	359,44
18,682	22,6457	-0,3001	320,75
18,684	21,8121	2,9702	263,38
18,686	22,3727	1,0404	298,19
18,688	22,0537	1,1672	291,96
18,69	22,4567	1,9017	287,33
18,692	21,8336	-0,7389	315,53
18,694	22,4868	1,6115	291,81
18,696	21,5377	-3,5259	350,35
18,698	22,2989	-2,7558	350,23
18,7	22,1177	0,4717	302,58
18,702	21,5339	-1,0413	315,57
18,704	22,1883	2,31	277,87
18,706	21,535	2,2441	269,66
18,708	21,8153	-1,5946	327,24
18,71	21,9211	2,7703	267,7
18,712	21,4996	2,661	263,34
18,714	21,6536	0,6111	294,14
18,716	22,2789	5,2317	238,3
18,718	21,3962	-0,0385	299,63
18,72	22,4378	-1,0446	328,25
18,722	22,1414	2,7411	271,19
18,724	21,8189	1,4967	284,07
18,726	22,1366	3,8605	255,47
18,728	21,9535	1,6911	283,24
18,73	21,9519	-6,9886	404,55
18,732	22,4233	-2,9815	355,12
18,734	21,0228	-0,1392	295,81

18,624	38,5034	16,8196	303,11	
18,626	39,0977	22,5031	231,97	
18,628	37,762	17,7855	279,24	heating
18,63	38,4433	19,3272	267,21	heating
18,632	36,9008	18,1663	261,88	heating
18,634	37,5543	15,5677	307,34	heating
18,636	37,8282	15,4919	312,23	heating
18,638	38,0228	13,1856	347,19	heating
18,64	39,3475	13,8453	356,48	
18,642	40,6288	14,6346	363,36	
18,644	40,0999	15,3016	346,64	
18,646	39,971	12,622	382,3	
18,648	40,958	18,0713	319,92	
18,65	40,3787	14,794	357,64	
18,652	39,688	20,0264	274,84	
18,654	38,9954	18,107	291,99	
18,656	39,8784	19,7117	281,9	
18,658	39,0224	16,4087	316,11	
18,66	39,1281	19,2888	277,32	
18,662	39,2219	21,5575	246,92	
18,664	39,5341	23,1494	229,03	
18,666	38,4065	18,553	277,52	
18,668	39,4803	17,6769	304,78	
18,67	38,8923	16,9683	306,46	
18,672	39,4589	17,5033	306,91	
18,674	39,8407	22,7147	239,4	
18,676	40,7132	19,9098	290,8	
18,678	40,0201	21,6728	256,47	
18,68	39,7046	20,0902	274,18	
18,682	40,1089	18,4488	302,78	
18,684	40,0298	15,4618	343,42	
18,686	39,1644	18,0671	294,91	
18,688	38,4066	13,7082	345,25	
18,69	39,0676	14,396	344,87	
18,692	37,803	10,8116	377,3	
18,694	38,8673	13,8729	349,38	
18,696	38,8826	17,6004	297,49	
18,698	39,5014	19,1548	284,42	
18,7	39,2231	18,4929	289,78	
18,702	40,1944	17,6313	315,4	
18,704	39,459	15,8754	329,66	
18,706	39,5389	15,3824	337,67	
18,708	39,9659	19,8581	281,08	
18,71	40,2935	16,8587	327,58	
18,712	39,8355	18,3077	300,93	
18,714	39,5697	15,2813	339,52	
18,716	40,6294	15,537	350,75	
18,718	40,1899	13,9377	366,97	
18,72	39,8836	17,5075	312,79	cooling
18,722	39,4472	15,9941	327,84	cooling
18,724	39,9499	18,2309	303,6	cooling
18,726	38,3881	13,8716	342,7	cooling
18,728	39,0411	13,9497	350,74	cooling
18,73	38,4949	13,72	346,32	cooling
18,732	38,7229	10,1519	399,38	cooling
18,734	38,8451	10,8318	391,59	cooling

18,624	33,2202	10,5285	317,2
18,626	33,7598	16,1531	246,12
18,628	32,9941	12,0451	292,84
18,63	33,5511	8,2665	353,44
18,632	33,1079	8,7434	340,58
18,634	32,9846	8,2775	345,37
18,636	33,3517	12,3947	292,95
18,638	33,438	17,4087	224,07
18,64	33,0543	12,6038	285,87
18,642	33,8396	13,5753	283,27
18,644	33,2413	14,2088	266,05
18,646	33,1102	11,0728	308,05
18,648	33,5735	15,097	258,27
18,65	32,7383	12,1071	288,39
18,652	33,2716	11,0512	310,61
18,654	32,9552	15,6592	241,77
18,656	32,571	15,7922	234,54
18,658	32,9775	15,0318	250,85
18,66	33,3688	16,069	241,83
18,662	32,9861	8,0795	348,16
18,664	33,6196	9,7691	333,4
18,666	32,6763	11,3336	298,34
18,668	32,6202	9,6311	321,35
18,67	32,8407	13,057	276,55
18,672	32,1848	8,8708	325,89
18,674	32,8026	3,8228	405,09
18,676	33,2504	8,4851	346,18
18,678	32,5674	9,5588	321,63
18,68	32,9762	10,7656	310,47
18,682	33,132	13,1573	279,22
18,684	32,6908	5,5928	378,79
18,686	33,5905	9,9407	330,59
18,688	32,9779	15,7301	241,1
18,69	32,902	15,539	242,71
18,692	33,297	16,4637	235,3
18,694	32,8084	12,737	280,57
18,696	33,3013	8,2743	349,84
18,698	33,4753	15,2757	254,4
18,7	32,5963	14,5975	251,6
18,702	33,0824	12,8027	283,48
18,704	33,2008	13,8515	270,48
18,706	32,6306	7,4495	351,99
18,708	33,1646	10,3274	319,23
18,71	32,8271	12,696	281,4
18,712	32,6765	10,144	314,97
18,714	33,546	13,4335	281,14
18,716	32,9213	15,1479	248,44
18,718	33,4271	12,0201	299,24
18,72	33,3514	14,9726	256,91
18,722	32,915	9,5974	325,95
18,724	33,1543	6,6563	370,4
18,726	33,3234	10,0755	324,97
18,728	32,5068	9,6616	319,34
18,73	33,158	15,1879	251,2
18,732	32,7926	19,4214	186,91
18,734	32,5443	14,2726	255,41

18,736	20,5398	-0,0803	288,24		18,736	39,9358	13,309	372,2	cooling	18,736	33,6077	12,7863	291,05	
18,738	19,9922	2,7442	241,1		18,738	39,3075	14,8679	341,63	cooling	18,738	32,8105	7,8441	348,99	
18,74	18,7118	-3,2355	306,79		18,74	39,5183	17,1361	312,87	cooling	18,74	33,8544	5,9861	389,56	
18,742	19,3533	-5,4334	346,48		18,742	39,3764	18,0232	298,49	cooling	18,742	33,6945	13,3765	284,02	
18,744	19,1565	-0,5356	275,27		18,744	39,6753	11,088	399,61	cooling	18,744	33,3738	14,1338	268,95	
18,746	19,2087	-0,6977	278,26		18,746	38,7539	11,703	378,13	cooling	18,746	33,0999	12,7475	284,5	
18,748	19,6704	2,4075	241,31		18,748	37,9187	9,6604	395,01	cooling	18,748	33,5978	14,2092	271,02	
18,75	20,0155	3,9678	224,32		18,75	39,1611	16,2391	320,42	cooling	18,75	32,9803	9,9112	322,47	
18,752	19,7045	-3,1577	319,58		18,752	38,4299	18,7718	274,79	cooling	18,752	33,6242	10,9417	317,07	
18,754	20,8055	-1,1649	307,11		18,754	38,3408	21,5992	234,02	cooling	18,754	33,1826	13,0513	281,4	
18,756	19,9822	-1,1204	294,98		18,756	38,5366	19,59	264,85	cooling	18,756	32,997	9,5348	327,97	
18,758	20,5387	0,6575	277,91		18,758	39,6842	19,4875	282,32	cooling	18,758	33,549	11,4691	308,64	
18,76	20,3841	3,5505	235,31		18,76	38,5128	12,4136	364,83	cooling	18,76	32,8132	12,0587	290,12	
18,762	19,9238	-2,3241	310,99		18,762	39,4716	16,1055	326,62	cooling	18,762	33,2154	12,6024	288,14	
18,764	20,6991	-7,018	387,44		18,764	38,2969	15,9685	312,12	cooling	18,764	33,0936	15,6239	244,2	
18,766	20,4423	-5,0944	356,96		18,766	38,5861	15,0819	328,55	cooling	18,766	33,1092	14,1209	265,43	
18,768	20,4647	-5,5844	364,13		18,768	38,0631	14,8203	324,9	cooling	18,768	33,0328	10,585	313,79	
18,77	20,506	-1,1748	303,07		18,77	38,822	11,0856	387,71	cooling	18,77	33,8379	13,5953	282,96	
18,772	20,5779	0,8534	275,72		18,772	38,7845	11,996	374,46	cooling	18,772	33,0421	11,1816	305,58	
18,774	20,1542	-5,4651	358,12		18,774	38,7006	15,1529	329,16	cooling	18,774	33,5812	12,4899	294,83	
18,776	21,1995	-2,4268	330,26		18,776	39,3596	15,9728	326,91	cooling	18,776	32,9823	14,0469	264,69	
18,778	20,3963	-3,1467	329,1		18,778	39,6697	12,4163	380,96	cooling	18,778	32,6642	9,3009	326,58	
18,78	20,9654	-1,381	312,37		18,78	39,0051	16,1165	319,95	cooling	18,78	32,7376	8,2931	341,7	
18,782	20,9052	2,3364	259,56		18,782	38,3955	16,7306	302,84	cooling	18,782	32,4245	9,0291	327,03	
18,784	20,8856	1,9036	265,34		18,784	39,5375	23,3642	226,08	cooling	18,784	32,6393	9,5463	322,81	
18,786	21,4861	6,00E-04	300,34		18,786	37,8285	19,3461	258,36	cooling	18,786	32,8572	13,1985	274,8	
18,788	21,1386	0,5823	287,35		18,788	38,1881	18,0588	281,38	cooling	18,788	32,5708	11,7568	290,95	
18,79	21,0697	-1,8413	320,26		18,79	37,9207	14,942	321,21	cooling	18,79	32,7229	7,3614	354,52	
18,792	21,1388	0,4163	289,67		18,792	38,5555	13,0016	357,21	cooling	18,792	33,6933	13,2354	285,97	
18,794	21,1062	1,3285	276,46		18,794	37,9388	10,6782	381,06	cooling	18,794	32,9783	14,299	261,11	
18,796	20,8594	-1,9129	318,32		18,796	38,966	14,3792	343,69	cooling	18,796	33,8463	17,7832	224,54	
18,798	21,2847	-2,4406	331,64		18,798	38,6001	15,4415	323,72	cooling	18,798	33,3716	18,5727	206,87	
18,8	21,1056	-3,2389	340,3		18,8	38,7319	16,0105	317,61	cooling	18,8	33,1192	13,5436	273,64	
18,802	21,1971	-0,1005	297,71		18,802	38,8967	16,6184	311,42	cooling	18,802	33,156	11,1604	307,47	heating
18,804	21,1531	4,0841	238,6		18,804	39,2177	12,1917	377,78	cooling	18,804	33,05	12,4786	287,56	
18,806	21,0588	3,981	238,72		18,806	39,1656	14,8479	339,92	cooling	18,806	32,9805	11,1438	305,24	
18,808	21,717	0,9453	290,36		18,808	38,5067	14,7859	331,58	cooling	18,808	33,6059	18,04	217,59	
18,81	21,287	-0,6972	307,31		18,81	39,563	16,8483	317,52	cooling	18,81	33,2246	20,0752	183,81	heating
18,812	21,3469	-1,5887	320,61		18,812	39,3805	14,1489	352,7	cooling	18,812	33,4483	16,7479	233,45	heating
18,814	21,1704	-0,4065	301,61		18,814	38,6308	15,882	318	cooling	18,814	33,8248	16,1214	247,47	heating
18,816	21,2253	0,1169	295,06		18,816	38,3851	13,4247	348,91	cooling	18,816	33,1927	9,8346	326,51	heating
18,818	20,8202	-1,4041	310,66		18,818	39,1411	17,5773	301,43	cooling	18,818	33,9106	9,7413	337,85	heating
18,82	20,7546	-1,8743	316,32		18,82	37,7356	13,5397	338,22	cooling	18,82	33,5974	13,215	284,91	heating
18,822	20,5045	-2,123	316,3		18,822	38,0162	11,4229	371,73	cooling	18,822	33,4044	12,179	296,7	heating
18,824	20,1328	-3,547	331,01		18,824	38,1908	12,066	365,19	cooling	18,824	33,7472	11,364	312,88	heating
18,826	20,2457	-3,7253	335,08		18,826	37,9962	14,8629	323,37	cooling	18,826	34,1867	14,6397	273,24	heating
18,828	20,4287	-3,3642	332,59		18,828	37,3035	18,2325	266,58	cooling	18,828	33,8468	11,7783	308,49	heating
18,83	21,046	-3,7185	346,17		18,83	37,6733	19,0273	260,64	cooling	18,83	34,3017	13,6341	288,9	heating
18,832	20,9024	-3,1842	336,7		18,832	37,4117	17,1333	283,46	cooling	18,832	33,62	8,9423	344,96	heating
18,834	21,1349	-2,8381	335,11		18,834	37,3943	14,4579	320,62	cooling	18,834	33,8625	4,911	404,7	heating
18,836	20,9999	-2,8796	333,8		18,836	37,9471	19,3577	259,85	cooling	18,836	33,8369	7,1083	373,63	
18,838	21,2755	-2,1557	327,53	cooling	18,838	38,4511	16,7966	302,7	cooling	18,838	33,8193	10,755	322,41	
18,84	20,8348	-4,1415	349,13	cooling	18,84	38,07	18,5512	272,84	cooling	18,84	33,7501	12,9182	291,2	
18,842	21,18	-3,6121	346,56	cooling	18,842	37,8401	17,522	284,02	cooling	18,842	33,6783	15,2557	257,52	
18,844	20,8147	-1,8386	316,66	cooling	18,844	38,7148	19,4099	269,85	cooling	18,844	33,4189	12,3731	294,19	
18,846	20,4801	-1,5721	308,26	cooling	18,846	39,0612	17,2707	304,6	cooling	18,846	34,0181	12,8366	296,09	

18,848	20,5704	0,8574	275,56	cooling	18,848	38,3779	19,6528	261,75	cooling	18,848	33,7293	13,5836	281,61	
18,85	20,4882	1,994	258,52	cooling	18,85	38,094	15,5627	314,95	cooling	18,85	33,7612	12,5819	296,06	
18,852	20,8003	-0,4605	297,19	cooling	18,852	38,9546	19,6581	269,74	cooling	18,852	34,0834	16,2733	248,96	
18,854	20,8217	-1,1666	307,36	cooling	18,854	37,5304	19,0183	258,77	cooling	18,854	33,8093	15,9327	249,89	
18,856	20,9994	0,3637	288,46	cooling	18,856	37,3472	17,745	274,01		18,856	33,7794	15,3404	257,75	
18,858	20,8	0,8568	278,78		18,858	37,4418	19,3255	253,24		18,858	33,9743	16,9098	238,54	heating
18,86	21,2234	3,9802	241,03		18,86	37,0472	18,5992	257,88		18,86	34,109	16,4311	247,11	heating
18,862	20,5877	2,8333	248,18		18,862	36,4811	19,3759	239,11		18,862	34,0963	12,6993	299,1	heating
18,864	20,9047	2,068	263,31		18,864	36,9405	21,1657	220,51		18,864	33,9657	13,0533	292,32	heating
18,866	20,3597	0,9988	270,64		18,866	35,9847	19,1493	235,33		18,866	33,6772	10,2794	327,07	heating
18,868	20,0442	-1,8102	305,49		18,868	36,3457	17,09	269,17		18,868	34,1117	12,0418	308,5	heating
18,87	19,8995	-2,0244	306,46		18,87	36,9851	22,7964	198,34		18,87	33,7973	14,7877	265,73	heating
18,872	19,9764	0,728	269,06		18,872	37,5875	17,46	281,35	heating	18,872	34,0565	15,5927	258,1	heating
18,874	19,7714	-2,3828	309,68		18,874	37,7195	19,9194	248,82	heating	18,874	34,269	13,289	293,27	heating
18,876	20,071	-3,1841	325,07		18,876	37,3228	18,4724	263,5	heating	18,876	33,9013	10,5705	326,13	heating
18,878	20,101	-3,567	330,84		18,878	38,2515	18,1929	280,39	heating	18,878	33,8202	8,4342	354,86	heating
18,88	20,2522	-5,1042	354,45		18,88	37,942	16,8096	295,4	heating	18,88	34,2283	10,9971	324,74	
18,882	21,1191	0,2551	291,65		18,882	36,9635	17,0409	278,49	heating	18,882	34,4629	13,8271	288,46	
18,884	20,5836	1,3493	268,87		18,884	37,0865	14,8163	311,3	heating	18,884	33,8718	13,5287	284,37	
18,886	20,906	-1,773	317,02		18,886	37,6409	20,764	235,91	heating	18,886	34,058	13,0778	293,27	
18,888	20,9405	-2,0624	321,55		18,888	36,0046	17,3986	260,08	heating	18,888	33,6522	10,7425	320,24	
18,89	20,7832	-1,8992	317,07		18,89	37,3801	15,5352	305,36	heating	18,89	33,9013	11,9063	307,46	
18,892	20,5032	-2,0934	315,87		18,892	37,6848	18,0146	274,96	heating	18,892	33,265	11,1269	309,46	
18,894	21,1871	4,3677	235,11		18,894	37,6963	13,2475	341,76	heating	18,894	33,7577	14,1002	274,78	
18,896	20,5535	5,1566	215,23		18,896	38,2532	17,1955	294,36	heating	18,896	33,4509	14,0532	271,15	
18,898	20,6854	4,3517	228,32		18,898	38,7624	16,7455	307,76	heating	18,898	33,4384	14,9297	258,72	
18,9	20,1318	2,3918	247,98		18,9	38,0343	13,2767	346,07	heating	18,9	33,409	13,2079	282,38	
18,902	19,7761	-2,4784	311,08		18,902	37,9819	12,1764	360,72	heating	18,902	33,8242	13,8874	278,69	
18,904	19,7967	-3,6004	327,06		18,904	38,3081	15,4573	319,42		18,904	33,9498	12,1519	304,7	
18,906	19,8491	0,3628	272,39		18,906	38,4571	11,7495	373,33		18,906	33,7331	11,604	309,33	
18,908	19,8691	1,5503	256,07		18,908	38,0195	18,7939	268,75		18,908	34,0577	10,5919	328,02	
18,91	20,1182	0,7861	270,23		18,91	36,7831	15,8492	292,62		18,91	33,8974	11,5297	312,67	
18,912	19,9061	-1,0568	293,03		18,912	38,4441	20,212	254,86		18,912	34,1538	13,7184	285,66	
18,914	19,5918	-5,2309	346,98		18,914	37,2088	20,4188	234,7		18,914	33,5848	11,2895	311,65	
18,916	19,784	-4,8963	344,99		18,916	37,0933	18,7641	256,22		18,916	34,289	11,7023	315,73	
18,918	19,7489	-2,2012	306,83		18,918	37,2012	15,3896	304,89	heating	18,918	33,8838	9,6933	338,15	
18,92	19,5901	0,5262	266,49		18,92	38,2178	16,887	298,17		18,92	33,7808	10,212	329,46	
18,922	19,4872	0,5724	264,4		18,922	37,131	13,1111	335,76		18,922	33,9056	12,1372	304,29	
18,924	19,15	-0,9739	281,3		18,924	38,7089	16,8089	306,13		18,924	33,4696	13,0927	284,84	
18,926	18,9412	-2,8532	304,65		18,926	38,2774	19,6422	260,49		18,926	33,8054	9,731	336,52	
18,928	18,792	-2,3552	295,61		18,928	38,3236	17,7993	286,9		18,928	33,5024	9,6239	333,79	
18,93	19,0584	-1,029	280,79		18,93	38,4182	21,1048	242,01		18,93	33,7904	8,8346	348,84	
18,932	18,9723	0,3751	259,96		18,932	38,7654	18,5267	282,91	heating	18,932	33,4689	11,6123	305,52	
18,934	18,7324	1,7259	237,73		18,934	38,4633	19,3593	267,05	heating	18,934	33,8158	15,5067	255,93	
18,936	18,7104	2,193	230,89	heating	18,936	37,8186	18,773	266,23	heating	18,936	33,0984	13,5278	273,57	
18,938	18,6418	0,4652	254,08	heating	18,938	38,9589	23,9008	210,49	heating	18,938	33,5731	10,8949	317,01	
18,94	18,5004	-3,804	311,78	heating	18,94	38,7691	20,0952	261,03	heating	18,94	33,1611	7,6541	356,55	
18,942	18,1492	-4,1378	311,54	heating	18,942	38,2026	19,448	262,16	heating	18,942	33,0966	7,314	360,4	
18,944	18,2482	-2,8906	295,49	heating	18,944	37,3833	11,0556	368,02	heating	18,944	33,2498	9,2754	335,13	
18,946	17,9134	-0,2922	254,49	heating	18,946	38,9652	12,8502	365,05	heating	18,946	33,1324	10,6248	314,62	
18,948	18,0807	0,2277	249,56	heating	18,948	37,8586	11,6306	366,63	heating	18,948	33,5222	8,0667	355,83	
18,95	18,0446	-1,0017	266,24	heating	18,95	38,2509	16,7455	300,61		18,95	33,4504	10,16	325,56	
18,952	18,8194	-2,2452	294,45	heating	18,952	38,0241	17,6811	284,36		18,952	33,7482	9,7925	334,87	cooling
18,954	18,6062	-2,097	289,4	heating	18,954	38,7464	19,6558	266,86		18,954	33,6345	13,1049	286,97	cooling
18,956	18,9852	-1,8891	291,79	heating	18,956	37,7806	19,8553	250,57		18,956	34,3048	19,0473	213,28	cooling
18,958	19,2295	-0,1076	270,3	heating	18,958	38,41	18,9696	271,75	heating	18,958	33,4278	18,2389	212,32	

18,96	19,6497	1,8627	248,64	heating	18,96	38,2043	17,8215	284,92	heating	18,96	34,2384	17,2517	237,45	
18,962	19,449	0,721	261,79	heating	18,962	37,6877	15,0217	316,84	heating	18,962	33,2276	13,7814	271,83	
18,964	19,8454	1,2617	259,77	heating	18,964	37,7806	16,6149	295,86	heating	18,964	33,1527	11,3478	304,8	
18,966	19,8465	0,1649	275,12	heating	18,966	38,4754	15,5325	320,71	heating	18,966	33,0794	13,9737	267,07	
18,968	19,446	-0,4282	277,81	heating	18,968	38,2171	19,3492	263,75		18,968	32,763	14,0961	260,94	
18,97	19,6645	2,7908	235,87	heating	18,97	37,6464	15,2353	313,27	heating	18,97	33,0164	10,6148	313,14	
18,972	19,3287	3,7851	217,28	heating	18,972	39,6055	20,1652	271,75		18,972	32,8676	12,2848	287,72	
18,974	19,7869	1,8618	250,57	heating	18,974	39,2832	16,1892	322,82	heating	18,974	33,0472	12,1808	291,68	
18,976	19,4985	1,6601	249,36	heating	18,976	39,2795	15,1129	337,81		18,976	32,7435	12,6009	281,56	
18,978	19,8667	-0,4484	283,97	heating	18,978	38,7133	11,4121	381,63		18,978	33,2488	13,6595	273,83	
18,98	19,935	-0,9625	292,12	heating	18,98	39,6694	15,5093	337,72		18,98	32,3953	7,9383	341,87	
18,982	20,3174	-0,2765	287,87	heating	18,982	37,9423	14,523	327,37		18,982	33,5838	7,4627	365,13	
18,984	20,0832	-2,6164	317,31	heating	18,984	38,656	21,1244	245,07		18,984	32,5431	8,5602	335,25	
18,986	20,7558	-1,1141	305,71	heating	18,986	37,2483	18,2853	265,07		18,986	33,0019	9,973	321,91	
18,988	20,4838	0,1077	284,83	heating	18,988	38,0462	17,2139	291,21		18,988	32,8332	11,7364	294,9	
18,99	20,5615	-0,9506	300,71	heating	18,99	37,1388	21,3979	220,03		18,99	32,9685	10,1075	319,56	
18,992	20,9406	0,8415	280,96	heating	18,992	37,3597	17,6787	275,11		18,992	32,9692	7,5365	355,51	
18,994	20,5945	-0,7601	298,51	heating	18,994	37,2419	16,214	293,94		18,994	33,1764	10,553	316,24	
18,996	21,0451	-1,9959	322,08	heating	18,996	37,2783	16,6055	288,98		18,996	33,1419	10,6879	313,87	
18,998	21,1495	1,0851	280,47	heating	18,998	37,5995	15,4186	310,06		18,998	32,8563	12,3815	286,21	
19	21,4447	2,2941	267,7	heating	19	38,1393	11,5256	372,02		19	33,2292	14,7378	258,48	
19,002	21,7371	3,9407	248,77	heating	19,002	37,7673	15,0673	317,31		19,002	32,4411	9,0407	327,1	
19,004	22,8828	8,4315	202,01	heating	19,004	36,7678	8,1099	400,6		19,004	33,3118	9,789	328,81	
19,006	22,81	4,7018	253,13	heating	19,006	39,1533	14,7676	340,88		19,006	32,3037	10,3217	307,28	
19,008	23,8937	3,7756	281,22	heating	19,008	37,6751	12,2954	354,77		19,008	33,2171	12,3308	291,96	
19,01	23,6738	3,5681	281,05	heating	19,01	38,1767	13,1277	350,15		19,01	33,0286	15,125	250,27	
19,012	24,0152	3,0804	292,64	heating	19,012	37,8914	13,1084	346,43		19,012	33,211	14,0032	268,5	
19,014	24,3572	6,6365	247,71	heating	19,014	38,8526	15,1492	331,34		19,014	33,2207	8,8223	341,05	
19,016	24,4633	8,4009	224,53	heating	19,016	37,6593	12,0842	357,5	cooling	19,016	33,6852	9,7129	335,1	
19,018	24,7202	5,4757	269,01	heating	19,018	38,6396	17,5791	294,39	cooling	19,018	33,251	9,616	330,38	
19,02	25,0852	6,0252	266,43	heating	19,02	37,7425	16,6845	294,36	cooling	19,02	33,2171	10,6681	315,2	
19,022	24,6553	4,5	281,74	heating	19,022	38,2867	16,2349	308,25	cooling	19,022	33,3457	11,6001	303,97	
19,024	24,7083	2,2739	313,6	heating	19,024	37,1105	14,5029	316,02	cooling	19,024	32,9219	8,0906	347,11	
19,026	25,3084	4,2215	294,76	heating	19,026	37,8135	10,7334	378,54	cooling	19,026	33,4721	9,5174	334,85	
19,028	25,077	1,1844	333,98	heating	19,028	37,7725	13,5403	338,73	cooling	19,028	33,0122	9,7747	324,83	
19,03	26,1633	2,7584	327,17	heating	19,03	36,9982	12,2672	345,7	cooling	19,03	33,51	10,4067	322,95	
19,032	26,2867	5,7962	286,43	heating	19,032	37,7285	16,2492	300,25	cooling	19,032	33,2202	11,9832	296,86	cooling
19,034	26,4736	3,1886	325,49	heating	19,034	38,1166	16,1499	307,06	cooling	19,034	33,0179	12,1476	291,74	
19,036	26,6127	1,6932	348,34	heating	19,036	37,3092	17,2969	279,74	cooling	19,036	33,1209	9,9128	324,41	
19,038	26,4861	0,1068	368,74	heating	19,038	36,8283	13,9746	319,46	cooling	19,038	33,5552	13,1234	285,61	
19,04	26,5242	-1,5836	392,91	heating	19,04	38,0023	20,7009	241,85	cooling	19,04	32,9789	11,9055	294,57	
19,042	26,7578	3,7999	320,92		19,042	37,1696	19,409	248,27	cooling	19,042	33,2709	14,0001	269,38	
19,044	25,8115	5,0864	289,71		19,044	37,3469	22,6555	205,36		19,044	33,168	14,9736	254,33	
19,046	25,9157	2,7677	323,57		19,046	36,6476	17,5803	266,53		19,046	32,7769	10,1724	315,98	
19,048	26,0895	7,0874	265,62		19,048	38,049	16,1261	306,45		19,048	33,1973	10,4748	317,63	
19,05	25,4479	4,8017	288,6		19,05	37,085	15,8283	297,14		19,05	33,1377	13,3552	276,53	
19,052	25,7333	4,3309	299,17		19,052	37,6867	19,1305	259,39		19,052	33,186	14,4685	261,64	
19,054	25,9744	6,9808	265,5		19,054	37,006	16,8086	282,33		19,054	32,8547	15,9867	235,79	
19,056	25,4015	4,2627	295,49		19,056	37,7192	15,9874	303,78		19,056	32,7406	14,2334	258,7	
19,058	26,087	5,0431	294,16		19,058	36,9842	15,5602	299,48		19,058	33,022	8,7939	338,67	
19,06	25,8528	7,1896	260,88		19,06	38,0391	12,9286	351,01		19,06	33,5738	13,4922	280,71	
19,062	25,7405	2,4385	325,73		19,062	37,909	14,835	322,54		19,062	33,119	13,9842	267,48	
19,064	26,1508	4,4813	302,91		19,064	37,6281	14,7117	320,34		19,064	33,3843	15,8917	244,52	
19,066	25,6053	7,2672	256,34		19,066	38,3538	18,8995	271,94		19,066	33,2329	15,3236	250,35	
19,068	25,418	4,3982	293,83		19,068	38,7136	15,3862	326,08		19,068	33,0176	12,8748	281,57	
19,07	25,6041	4,788	290,98		19,07	38,3484	14,5994	331,98		19,07	33,3608	13,3037	280,37	

19,072	25,2507	2,3288	320,41		19,072	37,5788	9,7789	388,6		19,072	33,3315	15,3907	250,79	
19,074	25,4244	0,3861	350		19,074	38,8881	15,3385	329,19		19,074	33,1433	12,1265	293,78	heating
19,076	25,847	4,5468	297,74		19,076	37,4104	12,4232	349,28		19,076	33,1777	11,6626	300,75	heating
19,078	25,2224	4,1002	295,26		19,078	37,4567	15,6646	304,62	cooling	19,078	32,8423	8,5377	339,74	
19,08	26,0184	5,1608	291,56		19,08	36,4162	14,6393	304,41	cooling	19,08	33,2863	5,9765	381,75	
19,082	25,8433	5,6517	282,25		19,082	37,3451	16,3689	293,22	cooling	19,082	33,409	10,2179	324,18	
19,084	25,9563	-0,1293	364,64		19,084	36,0758	15,7745	283,78	cooling	19,084	33,0159	11,7758	296,91	
19,086	26,417	0,1082	367,76		19,086	36,9684	17,2474	275,67		19,086	32,9974	11,7779	296,62	
19,088	25,9993	1,3548	344,49		19,088	36,6552	16,0199	288,45		19,088	33,152	11,0061	309,57	
19,09	25,9038	1,7316	337,89		19,09	37,5345	16,4923	294,14		19,09	32,6333	6,2192	369,23	
19,092	26,2772	6,4446	277,23		19,092	36,8998	16,3798	286,84		19,092	33,3461	6,2551	378,69	
19,094	26,0378	5,0935	292,77		19,094	38,0231	14,9914	321,95		19,094	33,3224	12,1208	296,37	
19,096	26,2147	-0,654	375,58		19,096	37,4977	18,6237	263,83		19,096	33,1821	13,1635	279,83	
19,098	26,8058	1,0505	360,02		19,098	36,7897	14,5296	311,16		19,098	33,259	14,7796	258,31	
19,1	26,2505	-0,1333	368,81		19,1	37,5717	17,5563	279,79		19,1	33,0828	12,2001	291,91	
19,102	26,8083	2,4338	340,72	cooling	19,102	37,5789	14,8434	317,81		19,102	33,5083	10,7592	318	
19,104	26,659	5,0631	301,88	cooling	19,104	37,0839	13,5938	328,36		19,104	33,3883	14,0402	270,46	
19,106	26,7813	1,3935	354,88	cooling	19,106	36,8605	9,8435	377,66		19,106	33,0881	15,1438	250,83	
19,108	27,3294	2,9679	340,54	cooling	19,108	38,2522	16,8004	299,87		19,108	32,7669	12,5537	282,55	
19,11	26,638	3,8935	317,93	cooling	19,11	37,3401	17,5923	276,05		19,11	33,1831	13,3249	277,59	
19,112	26,5969	2,7494	333,35	cooling	19,112	37,8497	23,3716	202,38		19,112	32,3946	11,7527	288,54	
19,114	26,7585	3,9997	318,13	cooling	19,114	36,9344	19,6785	241,21		19,114	33,2558	15,8017	243,98	
19,116	26,037	-0,3117	368,32	cooling	19,116	38,3454	20,2965	252,3		19,116	32,9596	19,278	191,25	
19,118	26,1886	-1,7621	390,71	cooling	19,118	36,6516	16,747	278,24		19,118	33,1367	15,9037	240,89	heating
19,12	26,3147	4,0909	310,66	cooling	19,12	37,2791	17,4062	277,79		19,12	33,041	14,7609	255,53	heating
19,122	25,574	3,5225	308,25	cooling	19,122	36,7122	17,6381	266,63		19,122	33,0191	12,9067	281,14	heating
19,124	25,8151	6,842	265,22	cooling	19,124	37,2111	18,0936	267,23		19,124	33,5619	13,4532	281,09	heating
19,126	25,4744	8,2249	241,12	cooling	19,126	36,7479	18,6321	253,23		19,126	33,5123	17,5006	223,82	heating
19,128	25,1179	0,5482	343,45	cooling	19,128	37,5748	18,0206	273,34		19,128	33,2601	17,7503	216,8	heating
19,13	25,2922	-0,3742	358,78	cooling	19,13	37,4046	15,6719	303,79		19,13	33,3351	11,7977	301,06	heating
19,132	24,5648	2,1376	313,5	cooling	19,132	37,4854	13,4348	336,19		19,132	33,6206	8,6525	349,02	heating
19,134	24,5402	2,3511	310,17	cooling	19,134	37,8959	17,6139	283,51		19,134	32,8988	3,8303	406,33	heating
19,136	25,2991	7,6585	246,59	cooling	19,136	37,5837	12,4732	351,01		19,136	33,5903	8,8046	346,47	heating
19,138	24,707	3,8422	291,66	cooling	19,138	38,0186	17,7848	282,84		19,138	33,3458	13,0242	284,07	
19,14	25,4652	-1,0822	371,09	cooling	19,14	36,445	15,2229	296,65		19,14	33,1832	12,6133	287,54	
19,142	25,9109	1,2594	344,59	cooling	19,142	38,1455	20,1636	251,36		19,142	32,9725	10,4991	314,14	
19,144	25,5921	1,1887	341,12	cooling	19,144	37,3208	20,6489	233,05		19,144	33,3183	10,6167	317,33	
19,146	25,7486	2,5655	324,07	cooling	19,146	37,2655	19,6503	246,23	heating	19,146	33,4837	9,0927	340,95	
19,148	25,6925	3,4774	310,53	cooling	19,148	36,8689	12,1775	345,15	heating	19,148	33,3826	12,7605	288,27	
19,15	25,4386	-2,7311	393,77	cooling	19,15	38,2214	15,3217	320,1		19,15	32,999	11,6566	298,33	
19,152	25,791	-3,5014	409,47	cooling	19,152	36,7208	11,8314	347,92		19,152	33,5084	10,2945	324,5	
19,154	25,3102	1,1555	337,65	cooling	19,154	37,7654	15,3722	313,02		19,154	33,3183	12,1989	295,22	
19,156	25,132	3,4183	303,53	cooling	19,156	37,1824	19,3969	248,62		19,156	33,0191	12,614	285,23	
19,158	25,6336	7,7099	250,55	cooling	19,158	36,9344	15,9265	293,66		19,158	33,5238	16,2024	242,13	
19,16	24,988	3,7864	296,37	cooling	19,16	37,1121	19,2571	249,59		19,16	33,3787	16,5675	235	
19,162	25,3021	-3,0135	395,81	cooling	19,162	37,0136	17,703	269,93		19,162	33,0781	15,2965	248,56	
19,164	25,7048	-1,9434	386,48	cooling	19,164	36,8912	16,892	279,56	heating	19,164	33,1174	13,6357	272,33	heating
19,166	25,6224	2,4156	324,4	cooling	19,166	36,8868	18,3733	258,79	heating	19,166	33,5144	13,9319	273,73	heating
19,168	25,8223	5,6674	281,74	cooling	19,168	37,2158	22,3491	207,81	heating	19,168	33,3848	13,0946	283,63	heating
19,17	26,1867	8,8007	243,03	cooling	19,17	37,3436	16,3959	292,82	heating	19,17	33,4892	16,1515	242,36	heating
19,172	25,814	2,4088	327,17	cooling	19,172	37,4592	20,5	237,06	heating	19,172	33,2686	14,4099	263,62	heating
19,174	25,764	-1,7146	384,11	cooling	19,174	36,525	12,3187	338,37	heating	19,174	33,6318	12,875	290,15	heating
19,176	25,3628	-0,9377	367,64	cooling	19,176	38,2564	13,791	341,99	heating	19,176	33,4288	11,5875	305,31	heating
19,178	24,9792	0,084	348	cooling	19,178	37,5459	12,8652	345	heating	19,178	33,597	11,3248	311,33	heating
19,18	25,4691	3,5864	305,89	cooling	19,18	37,94	12,9282	349,63	heating	19,18	34,0716	11,8586	310,5	heating
19,182	25,1407	3,9499	296,22	cooling	19,182	37,916	12,1553	360,1		19,182	33,9552	11,7376	310,57	heating

19,184	25,202	-1,5656	374,17	cooling	19,184	38,6941	16,2583	313,62	
19,186	25,413	-2,4469	389,44	cooling	19,186	37,1508	13,1403	335,63	
19,188	25,1378	-0,1448	353,41	cooling	19,188	38,0707	16,6304	299,7	
19,19	25,0644	-0,4175	356,2	cooling	19,19	36,8294	17,6129	268,62	
19,192	25,5502	3,0074	315,12	cooling	19,192	36,9314	15,771	295,79	
19,194	25,1072	-0,2652	354,67	cooling	19,194	36,4618	19,886	231,7	
19,196	25,3204	-2,252	385,42	cooling	19,196	36,9331	19,1703	248,3	
19,198	25,399	0,1829	352,48	cooling	19,198	36,7956	19,2549	245,19	
19,2	24,9005	-1,0723	363,06	cooling	19,2	37,02	16,1137	292,24	heating
19,202	25,4774	-0,7491	366,61	cooling	19,202	38,1322	15,7655	312,65	heating
19,204	25,4724	3,2729	310,32	cooling	19,204	38,8641	13,7493	351,07	
19,206	25,5119	1,4417	336,47	cooling	19,206	38,9604	21,047	250,4	
19,208	25,8378	2,1418	331,23	cooling	19,208	37,8372	16,5561	297,48	
19,21	25,7003	2,8033	320,07	cooling	19,21	39,3536	19,7292	274,32	
19,212	25,303	-1,1778	370,16	cooling	19,212	37,813	16,5433	297,32	
19,214	25,735	0,2882	355,71	cooling	19,214	37,5814	16,5041	294,63	
19,216	25,8334	2,6149	324,56	cooling	19,216	36,7034	14,4253	311,41	
19,218	25,7796	4,224	301,32	cooling	19,218	37,66	18,2544	271,26	
19,22	26,0884	5,9305	281,78	cooling	19,22	36,4936	17,1616	270,23	
19,222	25,8636	2,2858	329,58	cooling	19,222	37,483	18,7387	262,02	
19,224	26,5861	-0,7694	382,39	cooling	19,224	37,241	16,0662	295,99	heating
19,226	26,1499	0,7417	355,17	cooling	19,226	38,1779	15,7452	313,58	heating
19,228	25,9864	0,7472	352,81	cooling	19,228	37,7764	19,5739	254,44	
19,23	25,9728	3,351	316,22	cooling	19,23	38,16	16,6803	300,25	heating
19,232	25,8928	5,132	290,21	cooling	19,232	37,9368	14,6656	325,3	heating
19,234	25,5755	1,2169	340,5	cooling	19,234	37,5284	12,5406	349,29	
19,236	26,1555	-1,2163	382,62	cooling	19,236	37,4764	15,7389	303,86	
19,238	26,1539	-3,2159	410,55	cooling	19,238	37,6584	13,7574	334,1	
19,24	25,8395	-3,2693	406,9	cooling	19,24	37,332	18,3844	264,86	
19,242	26,0321	2,2447	332,51	cooling	19,242	36,9975	17,25	276,04	
19,244	25,7418	4,7932	292,83	cooling	19,244	38,358	20,6346	247,75	
19,246	26,2244	3,8536	312,71	cooling	19,246	37,7843	15,7853	307,51	
19,248	26,3659	2,1534	338,45	cooling	19,248	38,1409	15,6522	314,36	
19,25	26,6485	0,5146	365,31	cooling	19,25	37,3906	13,2939	336,84	
19,252	26,9299	2,2451	345,06	cooling	19,252	38,3203	16,9747	298,38	
19,254	26,5737	3,7477	319,07	cooling	19,254	36,8372	16,3043	287,02	
19,256	26,358	2,4099	334,76	cooling	19,256	37,6088	15,318	311,59	
19,258	26,6395	1,4184	352,55	cooling	19,258	37,3064	14,1057	324,31	
19,26	26,5971	0,2877	367,77	cooling	19,26	37,9708	12,2358	359,74	
19,262	26,6876	0,3644	367,96	cooling	19,262	37,5588	13,3387	338,56	
19,264	26,566	2,5213	336,11	cooling	19,264	38,4083	12,4801	362,44	
19,266	26,4015	1,4352	348,99	cooling	19,266	37,7496	12,8909	347,49	
19,268	26,9001	1,4188	356,19	cooling	19,268	37,856	12,7456	351,01	
19,27	26,6796	-2,2688	404,66	cooling	19,27	38,3425	18,2592	280,73	
19,272	27,0775	-4,0949	435,74	cooling	19,272	38,2132	15,8895	312,05	
19,274	27,3035	-0,324	386,19	cooling	19,274	38,0727	20,0767	251,56	
19,276	26,8562	2,9187	334,61	cooling	19,276	36,9775	15,1542	305,06	
19,278	26,8773	4,7769	308,93	cooling	19,278	38,181	14,6271	329,25	
19,28	27,0112	6,7379	283,39	cooling	19,28	37,2051	10,6899	370,64	
19,282	26,8432	2,6912	337,61	cooling	19,282	37,7349	13,3492	340,88	
19,284	27,0926	3,005	336,71	cooling	19,284	36,9884	14,9894	307,51	
19,286	26,4876	5,6057	291,9	cooling	19,286	37,7291	20,376	242,57	
19,288	26,0504	3,3026	317,98	cooling	19,288	36,2146	15,816	285,14	
19,29	26,6646	5,5427	295,25	cooling	19,29	37,5165	12,5371	349,18	
19,292	26,1983	4,0584	309,48	cooling	19,292	36,8367	12,4319	341,14	
19,294	26,4405	1,0693	354,65	cooling	19,294	37,1927	15,0566	309,43	

19,184	33,5444	10,3359	324,42	heating
19,186	33,9103	11,2333	316,99	heating
19,188	33,8552	13,4786	284,84	
19,19	33,649	12,9927	288,74	
19,192	33,2949	14,5123	262,55	
19,194	33,4086	13,4436	279,08	heating
19,196	33,1459	12,0275	295,2	heating
19,198	33,2784	12,805	286,19	heating
19,2	33,4372	15,9413	244,57	heating
19,202	34,0159	15,3497	260,93	heating
19,204	33,9607	14,8373	267,32	heating
19,206	33,9567	11,73	310,7	heating
19,208	34,3237	10,3693	334,85	heating
19,21	34,1698	12,1734	307,48	heating
19,212	34,0346	14,1771	277,58	heating
19,214	33,8157	13,5865	282,77	heating
19,216	34,6422	12,6714	307,12	heating
19,218	34,1435	10,0829	336,33	heating
19,22	34,7722	13,293	300,25	
19,222	33,895	15,9149	251,33	
19,224	34,2806	16,5209	248,25	heating
19,226	33,8003	14,7338	266,52	heating
19,228	34,087	12,877	296,48	heating
19,23	33,8792	13,0702	290,88	heating
19,232	33,8938	13,4527	285,74	heating
19,234	34,0406	12,821	296,62	heating
19,236	34,1074	11,8671	310,89	heating
19,238	34,4931	13,3084	296,13	heating
19,24	34,0493	12,0984	306,84	heating
19,242	34,6449	13,6217	293,87	heating
19,244	33,7701	10,6409	323,31	heating
19,246	34,2918	10,068	338,61	
19,248	33,8467	10,0133	333,15	
19,25	34,2279	11,8215	313,21	
19,252	33,9412	11,7058	310,82	
19,254	34,3568	13,0564	297,75	
19,256	34,0502	11,7251	312,07	
19,258	33,9565	10,5718	326,88	
19,26	33,9784	9,8408	337,41	
19,262	33,951	11,5776	312,75	
19,264	33,9705	16,1712	248,81	
19,266	33,489	16,0245	244,13	
19,268	33,9261	14,1618	276,28	
19,27	33,6569	8,1404	356,68	
19,272	34,0634	7,9868	364,51	
19,274	34,0002	11,0219	321,2	
19,276	34,1617	15,0069	267,76	
19,278	33,8095	12,1677	302,52	
19,28	34,2013	10,4135	332,52	
19,282	34,1343	8,0212	365,02	
19,284	34,1979	11,06	323,43	
19,286	33,6993	12,8488	291,46	
19,288	33,7991	10,8883	320,26	
19,29	33,9305	9,7048	338,64	
19,292	33,7708	7,9649	360,73	
19,294	33,85	8,8013	350,14	

19,296	26,8087	5,1385	302,92	cooling	19,296	36,89	20,4142	230,31	
19,298	26,0553	5,3714	289,13	cooling	19,298	37,2355	18,6365	259,99	
19,3	26,0302	2,0166	335,67	cooling	19,3	37,0066	18,9068	253,01	
19,302	26,2539	2,8161	327,63	cooling	19,302	36,6562	15,2933	298,62	
19,304	25,9268	-1,8752	388,63	cooling	19,304	37,5267	16,0376	300,39	
19,306	26,0816	-9,00E-04	364,59	cooling	19,306	36,9438	10,9747	363,01	
19,308	25,4471	5,8085	274,52	cooling	19,308	36,6944	14,8784	304,96	
19,31	24,7857	4,6967	280,81	cooling	19,31	35,7465	11,8571	333,94	
19,312	25,463	5,6308	277,23	cooling	19,312	37,1121	15,3521	304,17	
19,314	25,31	4,8887	285,46	cooling	19,314	36,0919	13,5036	315,75	
19,316	25,4955	-0,45	362,68	cooling	19,316	37,032	17,475	273,38	
19,318	26,2982	1,7873	342,63	cooling	19,318	36,8549	17,2217	274,44	
19,32	25,8213	2,9034	320,36	cooling	19,32	37,9174	19,4859	257,65	
19,322	25,7193	0,9564	346,15	cooling	19,322	36,9391	16,062	291,83	
19,324	26,1913	6,7305	272,03	cooling	19,324	37,7822	15,2372	315,15	
19,326	25,6532	5,7831	277,76	cooling	19,326	36,9578	16,5106	285,82	
19,328	25,6971	0,2143	356,21	cooling	19,328	36,3977	17,491	264,29	
19,33	25,63	0,7037	348,43	cooling	19,33	35,8066	20,3012	216,74	
19,332	25,0162	-2,2272	380,82	cooling	19,332	35,8887	15,9892	278,17	
19,334	25,4465	-2,4904	390,52	cooling	19,334	35,4752	13,2087	311,25	
19,336	25,3858	1,963	327,42	cooling	19,336	35,4294	8,5183	376,18	
19,338	25,3221	0,9828	340,23	cooling	19,338	36,6988	13,7849	320,3	
19,34	26,1274	4,1816	306,77	cooling	19,34	36,939	13,1333	332,77	
19,342	25,8459	5,8186	279,95	cooling	19,342	37,383	17,5843	276,76	
19,344	25,8414	1,1354	345,35	cooling	19,344	37,2811	15,3011	307,25	
19,346	26,7089	2,0922	344,1	cooling	19,346	38,394	17,3228	294,54	
19,348	26,747	3,0004	331,94	cooling	19,348	36,9747	12,5294	341,71	
19,35	26,6633	3,7572	320,19	cooling	19,35	37,0469	13,6136	327,56	
19,352	26,7337	8,7182	251,83	cooling	19,352	35,8911	11,6346	339,07	
19,354	25,7413	4,6033	295,48	cooling	19,354	36,351	13,5764	318,36	
19,356	25,934	-0,552	370,23	cooling	19,356	35,6067	13,4773	309,34	
19,358	25,6066	-0,1388	359,88	cooling	19,358	36,8404	11,9636	347,74	
19,36	25,6097	-0,4395	364,13	cooling	19,36	36,6717	12,6077	336,38	
19,362	25,5843	4,1497	299,62	cooling	19,362	37,0835	12,0049	350,56	
19,364	25,0618	7,4775	245,8	cooling	19,364	37,2724	14,3457	320,48	cooling
19,366	24,5754	2,4408	309,41	cooling	19,366	37,696	14,0407	330,67	cooling
19,368	25,3577	1,027	340,11	cooling	19,368	36,8539	16,3286	286,91	cooling
19,37	24,9524	-0,8336	360,45	cooling	19,37	36,4143	14,9871	299,52	cooling
19,372	25,3753	-1,5673	376,62	cooling	19,372	37,0084	21,4549	217,41	cooling
19,374	25,5422	2,7526	318,57	cooling	19,374	36,4933	17,9991	258,52	
19,376	25,1067	4,0335	294,57	cooling	19,376	36,7464	17,2538	272,48	
19,378	25,4005	4,7736	288,33	cooling	19,378	36,8478	12,2404	343,98	
19,38	25,3527	4,7751	287,64	cooling	19,38	38,062	15,3209	317,89	
19,382	25,8834	1,5996	339,45	cooling	19,382	37,1676	17,5231	274,6	
19,384	25,908	3,0142	320,02	cooling	19,384	37,6132	21,213	229,25	
19,386	25,839	5,3154	286,89	cooling	19,386	36,7579	18,4084	256,5	
19,388	25,2691	2,8004	314,08	cooling	19,388	37,5155	19,8331	247,17	
19,39	25,934	4,4903	299,75	cooling	19,39	36,0697	17,549	258,89	
19,392	25,0133	2,0558	320,91	cooling	19,392	37,1118	17,1866	278,52	
19,394	25,7896	1,6694	337,16	cooling	19,394	36,0768	18,2276	249,51	
19,396	25,3419	2,8976	313,74	cooling	19,396	36,3022	19,1064	240,37	
19,398	25,0026	1,0667	334,59	cooling	19,398	36,4622	23,2402	184,82	heating
19,4	25,0928	-0,0262	351,13	cooling	19,4	37,3371	22,6959	204,66	heating
19,402	25,2991	1,103	338,23	cooling	19,402	36,786	20,943	221,46	heating
19,404	25,5304	0,5008	349,88	cooling	19,404	36,9395	17,1925	276,03	heating
19,406	25,6555	3,4994	309,71	cooling	19,406	37,5954	20,2244	242,82	heating

19,296	33,9328	10,3605	329,51	cooling
19,298	33,5199	10,5481	321,11	cooling
19,3	33,625	9,7275	334,05	cooling
19,302	33,6805	12,8292	291,47	cooling
19,304	33,2786	13,024	283,13	cooling
19,306	33,2598	16,5031	234,23	cooling
19,308	33,0605	15,618	243,82	
19,31	33,316	12,1754	295,51	
19,312	33,5772	10,1771	327,1	
19,314	33,631	10,8681	318,19	
19,316	33,6944	12,0776	302,17	
19,318	33,8802	14,9762	264,25	
19,32	33,3281	11,8838	299,76	
19,322	33,8192	9,0647	346,03	
19,324	33,5644	10,4863	322,6	
19,326	33,5424	11,844	303,31	
19,328	33,4276	13,9254	272,61	
19,33	33,7333	12,703	293,97	
19,332	33,5071	8,4589	350,14	
19,334	33,8028	6,1608	386,39	
19,336	33,685	7,7381	362,7	cooling
19,338	33,717	9,1982	342,74	cooling
19,34	33,6227	12,0981	300,88	cooling
19,342	33,3575	9,8381	328,77	cooling
19,344	33,6827	8,0383	358,47	cooling
19,346	33,3387	6,5878	373,94	cooling
19,348	33,6606	8,8607	346,67	cooling
19,35	33,5656	11,907	302,75	cooling
19,352	33,7637	14,0485	275,59	cooling
19,354	33,3135	12,1836	295,37	cooling
19,356	33,8835	13,4193	286,06	cooling
19,358	33,5986	14,7965	262,83	cooling
19,36	33,8468	16,4091	243,75	cooling
19,362	33,5686	16,3088	241,27	cooling
19,364	33,5564	13,3595	282,32	
19,366	33,3564	12,0975	297,17	
19,368	33,463	12,3511	295,11	
19,37	33,6341	13,2524	284,91	
19,372	33,4775	11,8207	302,73	
19,374	33,3708	12,6442	289,73	
19,376	33,0244	11,2885	303,83	
19,378	33,2281	12,5872	288,53	
19,38	32,908	12,5122	285,1	
19,382	33,0711	11,6134	299,95	
19,384	32,8214	10,3518	314,09	
19,386	32,8555	11,8231	294	
19,388	32,4968	12,3365	281,81	
19,39	33,246	13,8632	270,94	
19,392	33,2436	12,8646	284,87	
19,394	33,5571	10,6119	320,74	
19,396	33,6369	12,0207	302,16	
19,398	33,6611	11,7096	306,85	
19,4	33,4442	11,6326	304,89	
19,402	33,6457	11,4894	309,71	
19,404	33,7789	11,9868	304,62	
19,406	33,6875	10,5415	323,55	

19,408	25,7106	5,1557	287,33	cooling	19,408	36,9369	16,3145	288,27	heating	19,408	33,5091	10,7098	318,7	
19,41	25,2878	0,8381	341,77	cooling	19,41	36,9762	18,7574	254,67	heating	19,41	33,3468	8,5241	346,98	
19,412	26,0996	0,8678	352,7	cooling	19,412	36,3	15,4867	290,94	heating	19,412	33,6983	11,2565	313,7	
19,414	25,5175	0,0736	355,67	cooling	19,414	37,424	17,1429	283,5	heating	19,414	33,0815	13,9993	266,74	
19,416	26,3087	3,2933	321,72	cooling	19,416	36,2697	14,2136	308,31	heating	19,416	33,254	15,647	246,12	
19,418	25,6246	5,4972	281,35	cooling	19,418	37,3422	14,975	312,66	heating	19,418	33,0927	13,6092	272,35	
19,42	25,3463	3,016	312,15	cooling	19,42	36,8296	12,2842	343,11	heating	19,42	33,3935	12,278	295,16	
19,422	25,3129	0,5471	346,19	cooling	19,422	37,6565	14,7495	320,21	heating	19,422	33,082	12,2246	291,56	
19,424	25,1897	-0,3172	356,55	cooling	19,424	36,6812	15,4056	297,4	heating	19,424	33,6302	14,7149	264,41	
19,426	25,1694	-0,2556	355,4	cooling	19,426	37,7008	17,2431	285,97	heating	19,426	33,0775	14,4274	260,7	
19,428	25,3213	4,9842	284,28	cooling	19,428	36,7274	18,8505	249,89	heating	19,428	33,3407	13,11	282,8	
19,43	24,8123	3,1646	302,6	cooling	19,43	36,8027	16,683	281,24	heating	19,43	32,9268	11,7088	296,6	
19,432	24,8355	0,5013	340,16	cooling	19,432	37,1098	17,1195	279,43	heating	19,432	33,1828	12,4948	289,19	
19,434	24,8193	1,4119	327,2	cooling	19,434	37,4451	12,9394	342,55	heating	19,434	32,7394	15,7336	237,72	
19,436	23,7962	-3,7815	385,5	cooling	19,436	37,3362	13,2158	337,17	heating	19,436	32,6339	14,6709	251,1	
19,438	23,6823	-1,0042	345,08	cooling	19,438	37,2596	11,8934	354,58	heating	19,438	33,134	12,453	289,09	
19,44	22,4752	2,8613	274,17	cooling	19,44	38,0301	18,0342	279,51		19,44	33,2116	10,0274	324,08	
19,442	21,9973	1,1632	291,23	cooling	19,442	37,1145	17,0044	281,11	heating	19,442	33,2028	10,1479	322,27	
19,444	22,7737	4,1432	260,43	cooling	19,444	36,9867	18,6435	256,41	heating	19,444	33,0327	10,9485	308,7	
19,446	22,0597	1,0514	293,67	cooling	19,446	36,2484	12,4454	332,73	heating	19,446	33,4493	12,4244	293,9	
19,448	23,1185	-6,1481	409,1	cooling	19,448	37,0357	15,046	307,38	heating	19,448	33,0075	10,3612	316,56	
19,45	23,8665	-0,0916	334,9	cooling	19,45	35,7629	14,3149	299,81		19,45	33,4954	11,3287	309,86	
19,452	23,1562	-0,2891	327,73	cooling	19,452	36,4544	17,2073	269,05		19,452	33,0281	9,8351	324,2	
19,454	23,1904	-2,929	365,11	cooling	19,454	36,135	18,0853	252,31	heating	19,454	33,6234	11,7187	306,19	
19,456	24,3239	3,6315	289,25	cooling	19,456	36,6236	19,7558	235,79	heating	19,456	32,9021	10,7913	309,08	
19,458	23,3019	-2,4524	360,01	cooling	19,458	36,3382	18,3296	251,73	heating	19,458	33,0953	10,5957	314,51	
19,46	24,032	-0,7733	346,74	cooling	19,46	37,4405	17,0594	284,9	heating	19,46	32,9636	11,793	295,93	
19,462	23,5137	4,7998	261,59	cooling	19,462	36,689	15,8584	291,18	heating	19,462	32,7657	12,844	278,48	
19,464	22,8475	0,2949	315,25	cooling	19,464	36,5077	13,2594	324,98	heating	19,464	32,8148	12,4121	285,2	
19,466	23,787	2,1089	303,03	cooling	19,466	36,4809	15,2123	297,3	heating	19,466	32,8061	13,5268	269,5	
19,468	23,3053	5,6058	247,41	cooling	19,468	36,5481	14,2668	311,46	heating	19,468	32,5925	15,1164	244,29	
19,47	23,9611	-0,4309	340,96	cooling	19,47	35,7949	16,8752	264,47	heating	19,47	32,4558	16,9043	217,39	
19,472	24,171	6,2633	250,32	cooling	19,472	36,1118	15,0782	294,02	heating	19,472	32,8057	17,6278	212,16	
19,474	23,234	6,2409	237,54	cooling	19,474	37,1546	18,3136	263,37	heating	19,474	32,8122	12,7807	280,01	heating
19,476	22,8455	-0,2024	322,18	cooling	19,476	37,207	14,4747	317,76	heating	19,476	33,2981	13,6485	274,67	heating
19,478	24,3651	5,558	262,89	cooling	19,478	37,797	14,8839	320,29	heating	19,478	32,6408	11,3961	296,97	heating
19,48	22,7095	1,3447	298,65	cooling	19,48	38,3685	14,5788	332,54	heating	19,48	33,0719	13,2698	276,8	heating
19,482	23,7849	0,3144	328,08	cooling	19,482	38,567	17,9526	288,16		19,482	32,6808	12,826	277,54	heating
19,484	23,2278	4,7569	258,2	cooling	19,484	37,034	16,7943	282,92		19,484	32,5366	9,552	321,29	heating
19,486	22,3553	-2,104	341,91	cooling	19,486	37,3217	17,88	271,77	heating	19,486	33,0953	7,7413	354,41	heating
19,488	22,7481	-4,4144	379,69	cooling	19,488	36,2065	15,2306	293,21	heating	19,488	32,9893	10,4441	315,15	heating
19,49	22,6106	-0,8955	328,58	cooling	19,49	36,6786	14,7478	306,56	heating	19,49	32,8213	10,608	310,51	
19,492	22,4807	-6,2004	400,92	cooling	19,492	35,9347	15,3128	288,26	heating	19,492	33,0966	13,1807	278,4	
19,494	22,687	-0,1237	318,86	cooling	19,494	37,6335	17,8136	277,05	heating	19,494	32,858	12,1355	289,67	
19,496	21,9542	2,7962	267,8	cooling	19,496	36,8535	19,1132	247,98	heating	19,496	32,9753	9,1899	332,48	
19,498	21,4926	-4,1685	358,7	cooling	19,498	37,4738	17,3418	281,42	heating	19,498	33,469	12,2625	296,44	
19,5	22,9264	-1,552	342,17	cooling	19,5	38,2426	20,721	244,93	heating	19,5	33,0894	9,2625	333,07	
19,502	21,7115	-3,5587	353,24	cooling	19,502	38,5399	18,6278	278,34	heating	19,502	33,33	10,5763	318,06	
19,504	22,8451	-1,439	339,46	cooling	19,504	38,3876	19,0386	270,47	heating	19,504	33,1667	11,8838	297,5	
19,506	22,344	7,4281	208,5	cooling	19,506	38,1612	16,7747	298,95	heating	19,506	33,1246	10,2215	320,15	
19,508	21,4275	3,663	248,32	cooling	19,508	38,7125	17,9371	290,41	heating	19,508	33,7938	12,0644	303,75	
19,51	21,6232	-1,7726	327,04	cooling	19,51	37,941	14,6909	325	heating	19,51	33,1441	14,7515	257,1	
19,512	21,7648	-0,6742	313,67	cooling	19,512	38,5817	18,6566	278,52	heating	19,512	33,4687	12,481	293,38	
19,514	21,4885	-3,7115	352,26	cooling	19,514	37,7062	15,1281	315,61	heating	19,514	33,7138	16,408	241,91	
19,516	21,6606	2,1152	273,22	cooling	19,516	38,8152	17,9564	291,57	heating	19,516	33,0684	15,1776	250,09	
19,518	21,0941	6,2298	207,78	cooling	19,518	37,3563	14,8703	314,32	heating	19,518	32,9433	13,6535	269,64	

19,52	20,7906	2,3553	257,7	cooling	19,52	38,4054	14,3927	335,66	heating	19,52	33,2086	14,8478	256,66	heating
19,522	21,7993	2,9449	263,56		19,522	37,7314	14,2692	327,97		19,522	32,8482	11,0438	304,79	heating
19,524	21,3884	1,5463	277,36		19,524	38,081	16,5995	300,28		19,524	32,9384	10,4857	313,86	heating
19,526	21,9358	0,532	299,19		19,526	38,1784	17,7017	286,23		19,526	33,1674	14,6741	258,51	
19,528	21,5206	3,0377	258,36		19,528	38,8518	20,7849	252,55		19,528	32,7303	14,3128	257,45	heating
19,53	21,2137	1,0396	282		19,53	37,8917	19,9196	251,22		19,53	33,3982	16,0256	242,84	heating
19,532	21,4929	-1,9137	327,19		19,532	37,9382	15,9765	306,99	heating	19,532	32,9763	15,2281	248,09	heating
19,534	21,7401	-2,0346	332,33		19,534	38,6921	17,1533	301,08	heating	19,534	33,6862	8,8182	347,62	heating
19,536	21,5979	-4,9424	370,99		19,536	38,4025	13,5678	347,15	heating	19,536	33,4022	8,2399	351,73	heating
19,538	21,82	-1,6117	327,54		19,538	38,0801	15,1677	320,28		19,538	33,238	8,7521	342,28	heating
19,54	21,3347	2,2658	266,55		19,54	37,8191	11,6577	365,7		19,54	33,2419	10,9837	311,14	
19,542	21,011	1,5063	272,65		19,542	38,7607	15,2805	328,22		19,542	34,068	16,0128	252,38	
19,544	21,7962	2,8844	264,36		19,544	37,6931	13,8211	333,7		19,544	33,2604	15,9411	242,1	
19,546	21,3908	-0,6804	308,52		19,546	37,9927	14,1941	332,67		19,546	33,7674	14,3902	270,87	heating
19,548	21,8323	-2,4382	339,27		19,548	38,2216	11,6013	372,11		19,548	33,9449	14,968	265,27	heating
19,55	21,5988	-1,007	316		19,55	38,1893	14,2238	335		19,55	33,4131	10,0315	326,84	heating
19,552	21,718	-0,3779	308,87		19,552	37,2591	12,0712	352,09		19,552	33,4892	10,3696	323,18	heating
19,554	21,7156	-0,8702	315,72		19,554	38,4091	14,6752	331,77		19,554	33,0766	13,0752	279,59	heating
19,556	22,0294	0,9657	294,44		19,556	37,6577	14,3273	326,13		19,556	33,1379	12,5596	287,65	heating
19,558	21,9151	-0,1201	308,02		19,558	37,7127	15,4053	311,82		19,558	32,6263	13,546	266,71	heating
19,56	21,8154	-0,1118	306,51		19,56	37,7591	19,3221	257,72		19,56	32,7253	14,1976	258,99	heating
19,562	21,4549	-0,8008	311,1		19,562	38,3473	16,9472	299,14		19,562	32,4677	9,5446	320,43	heating
19,564	21,2887	-2,5788	333,63		19,564	37,466	14,4225	322,11		19,564	33,6704	11,736	306,61	heating
19,566	21,7524	-4,0887	361,22		19,566	37,3031	11,8774	355,41		19,566	32,8743	9,559	325,91	heating
19,568	21,4467	-4,2316	358,95	cooling	19,568	37,7522	15,2203	314,96	cooling	19,568	33,8019	8,878	348,4	
19,57	21,6504	-2,4727	337,2	cooling	19,57	37,5519	15,8597	303,23	cooling	19,57	33,6214	12,3993	296,65	
19,572	21,1885	-2,3371	328,85	cooling	19,572	37,1941	19,5746	246,29	cooling	19,572	33,3265	11,472	305,49	
19,574	21,592	-1,1956	318,54	cooling	19,574	37,3622	17,0622	283,76		19,574	33,6256	11,311	311,92	
19,576	21,5783	0,5614	293,79	cooling	19,576	38,5445	20,045	258,6		19,576	33,9566	13,0988	291,56	
19,578	21,9151	2,1019	276,96	cooling	19,578	37,5682	14,5946	321,14		19,578	33,4855	11,0721	313,31	
[truncated: 637,411 more chars]
